# Detailed transcriptome atlas of the pancreatic beta cell

**DOI:** 10.1186/1755-8794-2-3

**Published:** 2009-01-15

**Authors:** Burak Kutlu, David Burdick, David Baxter, Joanne Rasschaert, Daisy Flamez, Decio L Eizirik, Nils Welsh, Nathan Goodman, Leroy Hood

**Affiliations:** 1 Institute for Systems Biology, Seattle, WA, USA; 2 Laboratory of Experimental Medicine, Universite Libre de Bruxelles, Brussels, Belgium; 3 Department of Medical Cell Biology, Uppsala University, Uppsala, Sweden

## Abstract

**Background:**

Gene expression patterns provide a detailed view of cellular functions. Comparison of profiles in disease vs normal conditions provides insights into the processes underlying disease progression. However, availability and integration of public gene expression datasets remains a major challenge. The aim of the present study was to explore the transcriptome of pancreatic islets and, based on this information, to prepare a comprehensive and open access inventory of insulin-producing beta cell gene expression, the Beta Cell Gene Atlas (BCGA).

**Methods:**

We performed Massively Parallel Signature Sequencing (MPSS) analysis of human pancreatic islet samples and microarray analyses of purified rat beta cells, alpha cells and INS-1 cells, and compared the information with available array data in the literature.

**Results:**

MPSS analysis detected around 7600 mRNA transcripts, of which around a third were of low abundance. We identified 2000 and 1400 transcripts that are enriched/depleted in beta cells compared to alpha cells and INS-1 cells, respectively. Microarray analysis identified around 200 transcription factors that are differentially expressed in either beta or alpha cells. We reanalyzed publicly available gene expression data and integrated these results with the new data from this study to build the BCGA. The BCGA contains basal (untreated conditions) gene expression level estimates in beta cells as well as in different cell types in human, rat and mouse pancreas. Hierarchical clustering of expression profile estimates classify cell types based on species while beta cells were clustered together.

**Conclusion:**

Our gene atlas is a valuable source for detailed information on the gene expression distribution in beta cells and pancreatic islets along with insulin producing cell lines. The BCGA tool, as well as the data and code used to generate the Atlas are available at the T1Dbase website (T1DBase.org).

## Background

The pancreas is composed of two types of tissue: exocrine and endocrine. The exocrine pancreas is made of acinar cells and secretes digestive enzymes into a network of ducts, while the endocrine pancreas consists of the islets of Langerhans and secretes hormones into the bloodstream. Pancreatic β cells are highly specialized cells within the islets of Langerhans responsible for producing vast amounts of insulin in response to changing glucose levels in blood. β cells are affected during Type-1 Diabetes (T1D) and Type-2 Diabetes (T2D) and are a major focal point of researchers in both fields. Availability of a complete list of transcripts expressed in human β cells, along with the transcriptomes of other cell types in endocrine and exocrine pancreas will aid T1D and T2D research.

Microarray technology is presently the preferred method for global (comprehensive) gene expression measurement and has been applied successfully to pancreatic islet and β cell-focused studies from human, mouse and rat [1-4]. MPSS is an alternative technology that estimates gene expression by counting short sequence signatures generated from up to one million expressed sequences per run. MPSS analyses provide very deep transcriptome analyses of individual tissues or cell types [5]. Unlike microarrays, MPSS eliminates the need to predefine genes that can be detected and samples the transcriptome deeply enough to detect transcripts expressed at levels as low as three copies per cell [6].

Systems biology is a multi-disciplinary science that seeks to quantify the molecular elements of a biological system, determine their interactions, integrate these data into molecular network models and then correlate network dynamics (changes in the components and architecture of the network) with developmental, physiological and pathological behaviors [7]. Such dynamic models serve to generate predictive hypotheses that can be experimentally verified. A first step toward constructing a systems biology network model is to build a comprehensive quantitative expressed-mRNA database reflecting dynamically changing transcriptomes of the cell types of interest (at different stages of their development, functional operation or disease progression). There are two types of dynamic molecular networks that in practice are closely integrated: protein and gene regulatory networks. Protein networks (protein/protein/small molecule interactions), for example, transmit information from the cell surface to the nucleus, mediate metabolism and provide the cell with structural integrity. On the other hand, gene regulatory networks integrate/modulate information and control behavior of protein networks or complex molecular machines through the action of transcription factors. Hence, delineation of the expression patterns of transcription factors of a particular cell type provides the components of its gene regulatory networks and initial insights into the networks that mediate its functional regulation. Specific changes observed in these networks under diseased states might serve as biomarkers of disease progression. Moreover, specific expression patterns, static or temporal, of a gene can provide important clues to its physiological function.

Current efforts to catalog gene expression in tissues lack detailed information about pancreas and pancreatic islets. Symatlas, the most widely used database of tissue expression, contains human and mouse expression data from up to 79 tissues under basal conditions [8]. In Symatlas, pancreas is represented as only one column without disclosing differences at the cellular level. The same is true for other complex tissues, such as liver and lung. Nor is an effort made to compare differences and commonalities between the same tissue in human and mouse. Having more detailed atlases that attempt to represent information from multicellular organs across different species and different data types is warranted.

There are several technical challenges that preclude obtaining of pure human β cells. These include limited availability of human material, no established protocol to isolate β cells from human pancreatic islets and the lack of human beta cell lines. In order to obtain a relatively complete β cell gene expression profile, we performed MPSS analysis of two independent human islet samples. Human islets contain α and β cells in a ratio between 0.3–0.5 to 1 and make up approximately 80% of the whole cell population, that are difficult to separate in human samples. Accordingly, we also characterized by microarray analysis rat β and α cells that are readily separated by FACS. The assumption is that the islet-cell transcriptomes will be similar in human and rat (and mouse). We also performed microarray analyses on the rat INS-1E cells, a glucose-responsive insulinoma cell line commonly used as a model to study β cell biology. We combined these four new types of data with expression data from the literature and public databases on pancreas and insulin-producing cells. The resulting 'β Cell Gene Atlas' (BCGA) incorporates basal gene expression data from experiments performed with pancreatic β and other cells in human, rat and mouse. The data assembled in the BCGA provide a major resource contribution to our understanding of β cells and the expression landscapes of cells in the pancreas that will benefit efforts to understand and ultimately prevent diabetes.

## Methods

### Cell culture

Human pancreatic islets from two healthy donors were cultured for 48 h in complete RPMI medium [9]. (Donor 1: 45 years old female, BMI 25.7, Blood Group A; Donor 2: 69 year old male, BMI 24.7, Blood Group O). Human islets used were of excellent quality: β cell percentages of the two donors were 53 and 59%. Insulin release stimulation indices (16.7 vs 1.67 mM glucose) were 8.9 and 10.4. β cell percentage analysis and insulin release determination were performed as described [10]. INS-1 cells were cultured in complete medium and collected for analysis after 48 h [11]. Primary rat β cells (>87% β cells) and non-β cells (<3% beta cells, mostly α) were isolated by FACS mediated purification of two different rat islet preparations [12]. Total RNA was isolated using either column based (Qiagen RNEasy) or a Trizol-based method (UltraSpec). Insulin and glucagon-positive cell percentages were estimated by immuno-histochemical staining. Human islet study was approved by the Institutional Review Board (exempt no #4). Rat samples were prepared following the guidelines of the Belgian Regulations for Animal Care.

### Massively Parallel Signature Sequencing Analysis

mRNA was processed according to the MPSS (Solexa, CA) protocol as described [5]. MPSS procedure creates 17 bp signatures of the transcripts. The abundance of each signature was converted to transcripts per million (tpm) for purpose of comparison between samples. Each signature was aligned to the current human genome (UCSC hg18) and coding sequences (RefSeq, mRNA, and EST) by modified BLAT analysis to detect short sequence alignments [13]. In case a gene was represented by more than one signature, their tpm values were added to obtain a final tpm count for that gene.

### Microarray analysis

Low-level processing, normalization and statistical analysis of microarrays were performed using R/Bioconductor packages (Release 2.1) [14]. MAS5 algorithm was selected as the method of normalization with target intensity set to 1500. Differential expression analysis was assessed by linear models and empirical Bayes moderated F statistics within the Limma R package [15]. Biological Process GO terms for each gene were obtained from Bioconductor Project metadata packages and 'GOstats' package was used to calculate the enrichment of GO terms [16]. All datasets can be accessed at NCBI-GEO repository (GSE13381).

### Downloaded Datasets

Microarray expression datasets were downloaded from public databases [see Additional File 1] in raw form (if available), and were reanalyzed, reannotated, and integrated with the new data generated here. The datasets we downloaded were generated from the following sources: 1. Human: laser capture microdissected β cells, pancreatic islets, exocrine pancreas, ductal cells, 2. Mouse: islets, whole pancreas, MIN6 cell lines, 3. Rat: FACS-purified β cells, α cells (non-β cells from FACS isolation), islets and whole pancreas.

### Homology Analysis and Expression Data Integration for BCGA

Homologs of human, rat, and mouse genes were obtained from several sources: HomoloGene [17], Mouse Genome Database [18], Rat Genome Database [19], Ensembl [20], Inparanoid [21], OrthoMCL [22] and KEGG [23]. The data sources were used in the order listed here and all consistent orthology information was added to the homology database. No attempt was made to weight information based on the number of sources reporting the information because many of the sources are not independent.

Expression signal intensity values in each array were converted to ranks within the experiments; the highest value was used for genes represented by more than one probe. The rank values of genes in a given cell type were averaged with other calculated values from experiments performed with the same cell type. We observed that probe signal intensities are bimodal distributed and genes with Affymetrix absence and presence calls belong to the population with lower and higher means, respectively. Therefore, we performed model based clustering and calculated for each gene the probability of belonging to the group of present genes [24]. A final probability score of expression for each gene was calculated by combining probabilities using Fisher's method [25].

## Results

### MPSS expression data analysis

**Figure 1:**
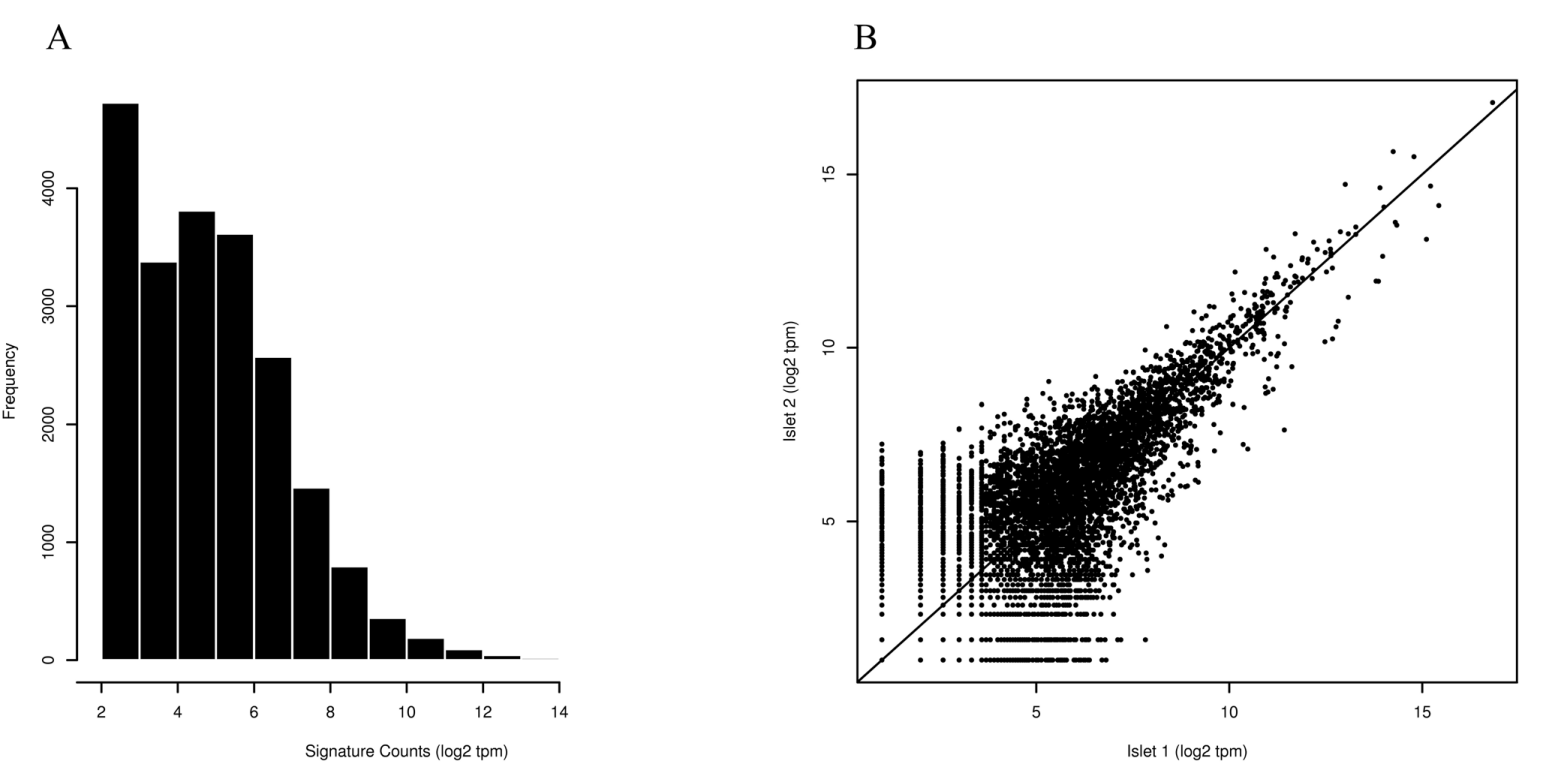
**A) Histogram of the signature counts in the raw MPSS dataset (before annotation)**. The frequencies of signature logarithm 2 scale counts in both MPSS samples are plotted. B) Scatter plot of signature counts first islet sample (on x axis) vs second islet sample (on y axis). Tpm = transcripts per million.

1,051,000 and 1,313,239 signatures were sequenced, respectively, in the first and second MPSS islet libraries. The distribution of the raw signatures, i.e. before they are mapped to specific genes, displays a skewed structure towards low-copy transcripts, suggesting that the majority of signatures fall in this category (Figure 1a). There were 11,946 unique signatures in islet sample 1 and 14,356 in islet sample 2. When the transcriptomes were combined there were 20,719 discrete sequences. The higher number of signatures in islet sample 2 probably reflects, in part, the greater depth of sequencing. The mean signature counts were 121.7 and 109.4 tpm while the median counts were 20 and 13 tpm. There was good correlation between the two samples for complete observations (Spearman 0.70) (Figure 1b). The existence of signatures unique to each library is probably an indication of donor variability that has been previously observed in large scale studies of human islet transplantation [26].

In order to annotate raw signatures, we aligned each 17 bp sequence to human genome or to ESTs and we determined Entrez Gene IDs that overlap with the genomic regions [see Additional File 1]. This signature-to-gene mapping was used to generate a gene-centric expression set [see Additional File 2].

There were 5,080 different genes detected in sample 1 and 5,420 genes in sample 2 at a level of at least 5 tpm. The two data sets generate 6,941 different genes, of which 3,552 are shared. We found 1196 unique signatures that could be mapped to genomic regions that overlap with coding sequences, but did not have any Entrez Gene IDs. Table 1 lists the most abundant transcripts in the MPSS result set. Insulin is at the top of the list with an average level of 126,753 tpm, corresponding roughly to 13% of the entire mRNA population in these human islet samples. Since our islet samples were composed of approximately 55–60% insulin-producing β cells, this data is in line with previous estimates of insulin mRNA copy numbers in β cells, namely insulin constitutes about 30% of the total mRNA [27]. Glucagon and somatostatin are also in the list of highly expressed genes (30,845 and 17,395 tpm respectively, Table 1) which correspond with their levels of 30% and 15% in the pancreatic islets.

**Table 1:** Expression of most enriched genes in the MPSS dataset

Gene id	Symbols	Description	Islet1	Islet2	Average
3630	INS	Insulin	115806	137701	126753
5068	REG3A	Regenerating islet-derived 3 α	28372	46821	37596
57521	KIAA1303	Raptor	19447	51634	35540
5967	REG1A	Regenerating islet-derived 1 α	44348	17588	30968
2641	GCG	Glucagon	16163	37866	27014
5645	PRSS2	Protease, Serine, 2	35590	8946	22268
2778	GNAS	GNAS complex locus	16287	27305	21796
6750	SST	Somatostatin	22199	12592	17395
9568	GABBR2	Gamma-aminobutyric acid (GABA) B receptor, 2	20203	12561	16382
440387	CTRB2	Chymotrypsinogen B2	21726	5667	13696
653	BMP5	Bone morphogenetic protein 5	5759	8451	7105
23521	RPL13A	Ribosomal protein L13a	6447	6452	6449
5968	REG1B	Regenerating islet derived 1 β	9519	3176	6347

'Gene id' = Entrez Gene ids, 'Symbols' = HUGO names for each gene, 'Description' = detailed gene name, 'Islet1' = tpm counts in MPSS sample from islet 1, 'Islet2' = tpm counts in MPSS sample from islet 2, 'Average' = average tpm counts in MPSS sample in both samples

In order to assess the functional processes associated with the most abundant genes, we performed GO term enrichment analysis. The top 200 transcripts are related to various processes including protein biosynthesis, protein metabolism, cellular biosynthesis and some stress response related categories [see Additional File 3]. The stress response processes probably originate from the stress induced by islet isolation. On the other hand, biological processes related to regulation, transcription and transcriptional activity are enriched among the 3591 transcripts that are detected at levels lower than 25 tpm. Indeed, most pancreatic islet specific transcription factors are expressed at levels of 150 tpm or lower with the exception of PAX6 which is expressed at average 565 tpm (Table 2). This is consistent with a recent study of transcription factor expression in adult human tissues indicating that most are expressed below 20 copies per cell [28]. Domain-based classification of transcription factors in the MPSS dataset was performed using the list of 790 transcription factors studied by Kong et al. [28] [see Additional File 4]. In the MPSS dataset, there are 243 transcription factors expressed at a minimum average level of 5 tpm and 223 of these could be classified into one of the 25 transcription factor classes.

**Table 2:** Expression levels of select transcription factors in the MPSS dataset

Gene id	Symbols	Description	Islet1	Islet2	Average
3110	HLXB9	Homeobox HB9	49	66	57
6927	HNF1	Transcription factor 1, hepatic	46	0	23
3170	HNF3	Forkhead box A2	63	232	147
6925	HNF4	Transcription factor 4	14	23	18
3175	HNF6	One cut domain, family member 1	6	3	4.5
3651	IPF1	Insulin promoter factor 1	38	48	43
3670	ISL1	ISL1 transcription factor, LIM/homeodomain	114	82	98
389692	MAFA	v-maf oncogene homolog A	169	80	124
4760	NEUROD1	Neurogenic differentiation 1	12	66	39
4821	NKX2-2	NK2 transcription factor related, locus 2	205	36	120
4825	NKX6-1	NK6 transcription factor related, locus 1	67	90	78
5080	PAX6	Paired box gene 6	418	713	565

'Gene id' = Entrez Gene ids, 'Symbols' = HUGO names for each gene, 'Description' = detailed gene name, 'Islet1' = tpm counts in MPSS sample from islet 1, 'Islet2' = tpm counts in MPSS sample from islet 2, 'Average' = average tpm counts in MPSS sample in both samples

With the aim to assess whether transcripts reliably detected by MPSS but not by microarrays were real, we performed RT-PCR analysis of MAFA, SAA1, INCA1, KCNIP3 and ZFXH2 genes in untreated control islets. We also included as a positive control INS and NEUROD1 in this analysis. Expression of all of the genes in the human pancreatic islets was validated by RT-PCR assays [see Additional File 1].

### Genes expressed in β cells, α cells and INS-1 cells

Currently, no established protocol exists for isolating large amounts of viable, purified human β cells with minimum impact on gene expression. On the other hand, methods are well established for isolation of β cells from rat and mouse which does not interfere with normal transcription patterns [29]. In order to estimate expression levels of transcripts in human β cells, we took advantage of the fact that 88% of the genes detected in these analyses had direct human and rat orthologs. We compared our MPSS data from human islets with microarray data from 2 independent samples of rat FACS-purified β cells, non-β cells after β cell selection (mostly glucagon-positive α cells), and 2 samples of insulin-producing INS-1 cell lines. Purity of primary rat β cells was 87% and 89%, and the rest of the cells were mainly α cells. The non-β cell samples consisted of 74.2% and 83% of glucagon-positive cells, with less than 5% β cells (data not shown). The results [see Additional File 5] indicate that there are 8783–9696 genes expressed in rat β, α, and INS-1 cells (threshold average signal intensity = 250, target intensity = 1500).

Differential expression analysis of genes expressed in rat β vs rat non-β(mostly α) cells identified 960 genes that are enriched in β cells (fold enrichment ≥ 2, FDR 2%). On the other hand, there were 699 genes that were more highly expressed in α cells compared to β cells (fold enrichment ≥ 2, FDR 2%). 4294 genes are shared between the two cell types (fold change <2 and FDR >10%). Consistent with this data, GO terms related to glycolysis pathway and carbohydrate metabolism are significantly enriched within the group of genes highly expressed in rat beta cells (p < 0.01, hypergeometric test) [see Additional File 6]. There were 346 and 312 transcription factors expressed in rat β and α cells, respectively [see Additional File 4]. Some transcription factors are expressed in only one cell type (qualitative differences), while some are expressed at different levels (quantitative differences). There are 58 and 24 transcription factors expressed only in β and α cells, respectively. In β cells, 41 transcription factors were expressed higher than in α cells and 71 transcriptional regulators were enriched in α cells.

With the aim of identifying α and β cell specific genes in human, we compared the list of α and β cell-enriched transcripts with a list of genes that contain human pancreatic islet-specific transcripts. The latter list was obtained by comparing pancreatic islet MPSS data with a published MPSS dataset of 32 human tissues [30]. A gene was called 80% or more specific to pancreatic islets if the signature count in islets were equal or greater than 80% of the total count of signatures across all tissues at a minimum level of 20 tpm. There are 940 genes that are specific to pancreatic islets [see Additional File 7]. 324 out of these 940 genes are probably expressed mainly in β cells, as they are expressed at higher levels (>= 1.5 fold) in rat β cells compared to α cells [see Additional File 8]. Meanwhile, 119 genes are expressed only or higher than 1.5-fold in α cells compared to β cells [see Additional File 8]. The overlap of the pancreatic islet specific genes with genes expressed in β cells includes known transcription factors, such as IPF1, TCF1, NKX6-1 and NEUROD1, and genes such as ALDOA, IGF2 and GLP1R.

### Construction of β cell Gene Atlas

The task of finding out whether a gene is expressed primarily in pancreatic β cells, islets or cell lines in different species is very difficult and requires time consuming analysis of diverse publications and/or re-analysis of available microarray data. We have collected almost all available public microarray data related to pancreas cells and from that constructed the BCGA. The expression data comes from 131 array analyses (mainly Affymetrix platform) derived from 28 experiments, each performed with β cells, α cells (current study), cell lines, islets, exocrine pancreas, ductal cells or whole pancreas [see Additional File 1]. Expression estimates were obtained as outlined in the Materials and Methods section.

**Figure 2:**
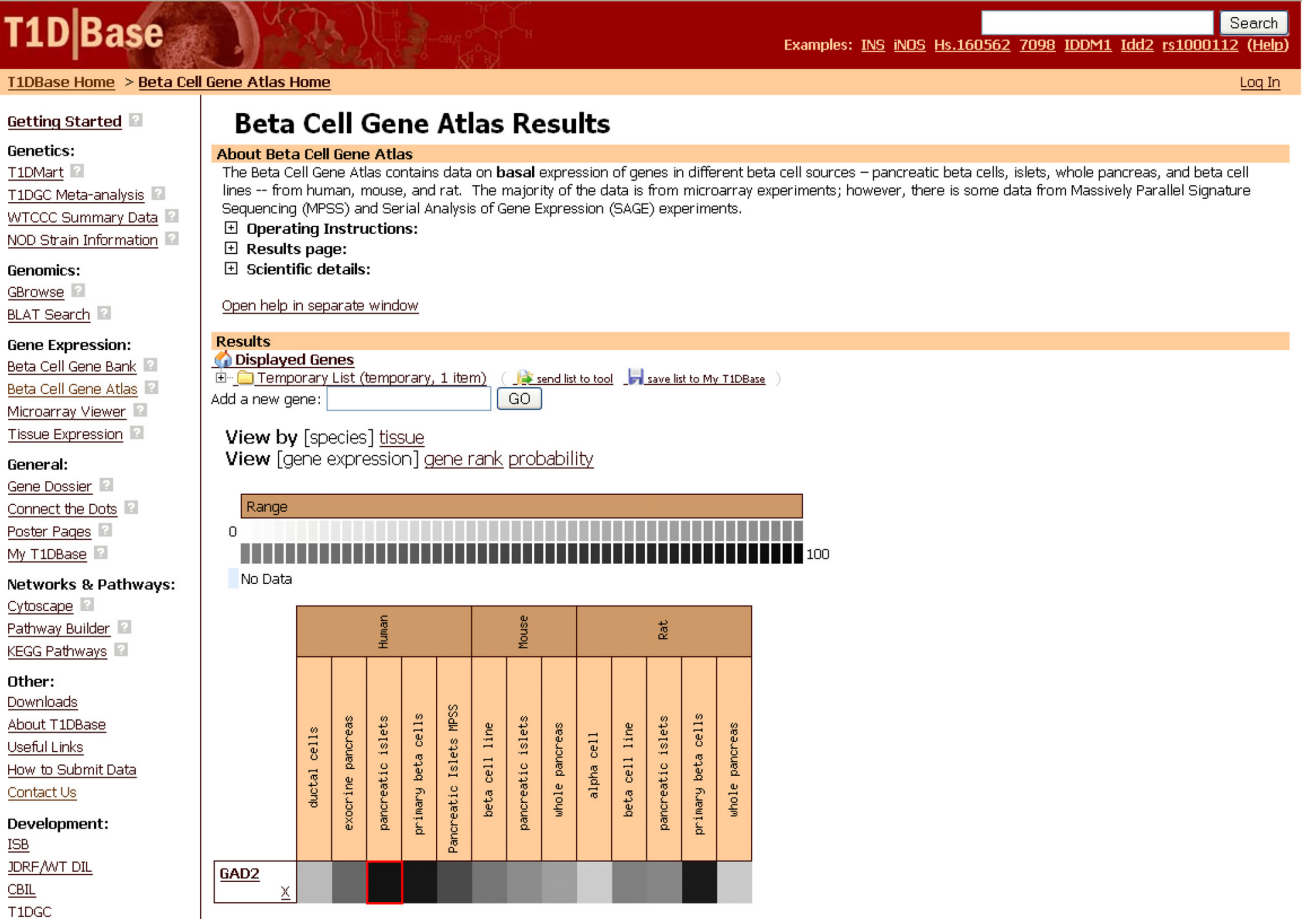
**'β Cell Gene Atlas' results page**. If a gene is estimated to be present at a high level, it is denoted with a darker cell color. The intermediate abundances are indicated with lighter cell colors. Genes expressed with a probability score of 0.95 or higher are designated with a red border color. The results can be viewed either with by "species" or "tissue" groupings. The actual values used to draw the heatmap or the probability scores can be viewed with "gene rank" or "probability" view, respectively.

The Atlas accepts any type of public transcript identifier which may be entered directly or uploaded from a file. The relative expression levels under basal conditions are indicated by a grey-scale heatmap (Figure 2). The darker cell colors indicate higher basal gene expression in the cells. The view can be switched to two different views to visualize the exact rank estimates with 95% confidence intervals or a probability score of presence.

**Figure 3:**
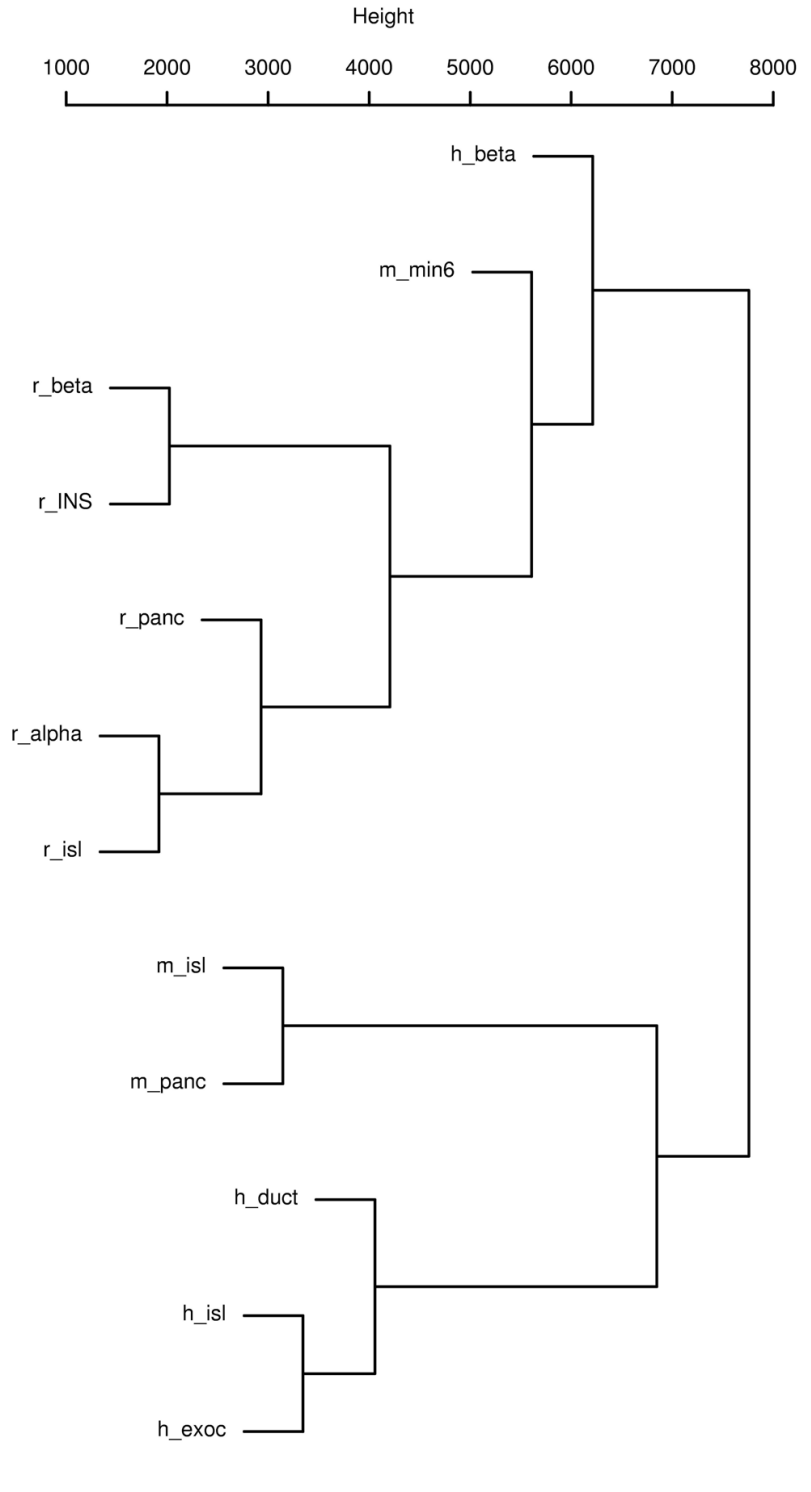
**Hierarchical clustering of tissue expression profiles: The two main branches of the group are β cells/cell lines and islets/pancreas**. h_beta: Human beta cells, m_min6: MIN6 cells, r_beta: rat beta cells, r_INS: INS-1 cells, r_panc: Rat pancreas, r_alpha: Rat alpha cells, r_isl: Rat islets, m_isl: Mouse islets, m_panc: mouse pancreas, h_duct: human ductal cells, h_isl: Human islets, h_exoc: Human exocrine cells.

To obtain a high level comparison between different cell types across three species, we performed hierarchical clustering using all the gene expression values of different cell types present in our Atlas. The integrated expression profiles were used to calculate a similarity matrix using Euclidian distance metric. The dendogram was built by average linkage clustering. This analysis roughly separates the cells based on species (Figure 3). Human β cells and mouse MIN6 cells (Table 2) are in the same branch as other insulin producing cells. This suggests that insulin-producing cells have similar profiles across three species. There are approximately 120 distinct transcripts ubiquitously expressed within the top 15% across all cell types and species. This list includes basal cellular functions or structure – such as 20 different ribosome subunits, several actin gene isoforms, genes involved in protein translation and structural proteins. These genes are likely candidates to serve as internal controls in RT-PCR expression studies.

We then looked at the genes that are enriched in the pancreatic islets compared to the rest of the cells in the pancreas, including duct and exocrine pancreas cells. There are 851 genes that fulfill such criteria. In order to assess whether this list is supported by independent experiments, we analyzed microarray data generated with two different genetic mice models. First, we obtained the list of genes that are significantly altered in TCF1/HNF1 knockout pancreas [31]. This perturbation leads to a progressive reduction in β cell number, proliferation rate, and pancreatic insulin content. We compared the list of genes that are decreased in TCF1 -/- pancreas compared to wild-type pancreas with the list of islet-enriched genes. As expected, this list has a significant overlap with the islet-enriched genes (112 out of 851 genes, p = 4.0E-5, hypergeometric test). As a negative control, we also inspected genes that are increased in response to TCF1 perturbation; there was no significant overlap with this group (p = 0.929). The overlapping list includes NKX6-1, ARNT2, GAD1, GCK, PCSK1 (PC1/PC3), and TMEM27. These genes have previously been determined to be either specific to pancreatic β cells or to play a crucial in the functioning of β cells. We performed a similar test with microarray data of AKR/J mouse pancreas . AKR/J mouse are resistant to obesity when fed a high fat diet and the islet area increases by 1.4 fold [32]. We performed analysis for significance of overlap with the list of genes increased or decreased in AKR/J mouse pancreas compared to B6 mice. There was a significant overlap (p = 0.0166) of the islet-enriched genes with the increased genes. We failed, however, to a find a significance of the overlap of decreased genes (p = 0.1136). All together, these results suggest that BCGA is reasonably successful in identifying genes expressed in different parts of pancreas.

## Discussion

We have used both MPSS and microarray data generated in the current study, together with publicly available expression data, to build a repository of β cell and pancreas gene expression, the β cell Gene Atlas (BCGA). The BCGA contains expression information for genes expressed particularly in β cells and other cell types in pancreas of humans, rats and mice. Obtaining a complete list of genes expressed in a certain cell type is the first step in generating a model of the biological networks that are active under normal conditions but perturbed under disease. Therefore, the Atlas is an essential tool that will allow application of systems biology to the field of pancreatic islet research and T1D research in particular.

BCGA can be queried with virtually any public identifier (Gene symbols, Entrez Gene ids, ENSEMBL ids) from all three species. Results are returned for all species that is orthologous to the queried gene. BCGA is a resource that allows side by side comparison of microarray data related to pancreas with a special emphasis on pancreatic β cells. BCGA will be useful in several research areas: β cell-specific biomarker discovery (as potential candidates for early diagnosis, determining the stages of disease progression and response to therapy), β cell regeneration (as a benchmark to compare regenerated islets) and pancreas development projects (as a list of genes (or transcripts) expressed in functional β (and α) cells encoding protein molecular machines, protein interaction networks and transcription factors encoding gene regulatory networks).

Microarrays are presently the standard method for global gene expression studies. However, the list of genes represented on microarrays is based on *a priori *knowledge and represents a subset of the whole genome. Compared to microarrays, MPSS data has the advantage of being quantitative and permits comparison of transcript copies between different datasets. MPSS analysis of two human islet samples resulted in detection of around 7,600 transcripts. These genes include many highly characterized genes as well as genes whose functions in islets are unknown. Results from microarray and MPSS platforms agree to a certain extent, while there are some genes that are only detected by one method. Similar moderate agreement between the two platforms has been observed in other studies [33,34]. MPSS technology is unable to detect approximately 7% of known genes lacking a DpnII restriction enzyme site [30]. The present MPSS data do not include 630 genes that are detected in the upper 90^th ^percentile levels in the human islet microarray data, which contains around 130 genes that lack a DpnII site. Therefore, this is not enough to explain the observed discrepancies between the two technologies. Microarray probes may have undesired properties, such as cross-hybridization, that could lead to false positives that are not detected by MPSS. The two methods have differential bias, including those related to G+C content of gene sequences [35]. The effect of G+C content on the stability of hybridized sequences is well known, with higher G+C content corresponding to more stable DNA duplexes in microarray platform. On the other hand, the G+C content might interfere with the sequencing properties in MPSS data. Another possible source of error is the annotation of the MPSS signatures. Around 20% of the signatures in our islet dataset are mapped to more than one gene (i.e. the signatures align to more than one location along the human genome) and these were discarded from the analysis (Supplementary Information). There are also many signatures not converted to genes. The results of Encyclopedia of DNA Elements (ENCODE) project that focuses on functional elements of 1% of the human genome, has recently been published [36]. The majority (>60%) of interrogated loci presented potential new exons mapping in their introns, while two-thirds (68%) of the investigated loci showed potential new putative TSS upstream of their annotated first exon, often reaching into neighboring genes [36]. Current human genome annotations probably fail to determine these yet to be identified exons and transcripts. Furthermore, some signatures (2209) could not be aligned to the genome during our annotation process. In subsequent analysis, approximately 1000 signatures could be mapped to mitochondrial genome. We have been able to align a subset of the unannotated signatures to transcripts in NCBI Trace Archive database . These signatures are probably from genes residing in regions that could not be assembled to the rest of human genome. This might indicate widespread existence of unknown exons and/or genes that remain to be identified. Finally, polymorphisms probably contribute to the high number of transcripts detected as present in the BCGA. The human islet microarray data in the atlas originate from 20 different individual samples and 9 experiments while the MPSS data are derived from only two individuals.

The variability in the MPSS results (see Results) might be explained by the differences in the depth of sequencing (1.05 million vs 1.31 million signatures) and is in line with previous observations of the variability between individual donors [37]. It also could arise from cells being at different stages of physiological response. One also must note that there may be heterogeneity in the cell populations in the pancreas that the ratios of these different cell types may differ in different human islet preparations. Limited availability and prohibitively expensive cost of MPSS precluded us from performing more MPSS experiments. However, the cost of signature sequencing based experiments (such as Solexa, ABI) has dropped dramatically. We plan to carry out more experiments of this type of next-gen sequencing and add to BCGA.

Transcription factors are essential for maintaining the gene regulatory networks that regulate all cellular functions: knowledge of regulatory behavior is key to predictive models of biological systems [38]. In the present study we have identified 346 and 312 transcription factors that are expressed in rat β and α cells respectively. Transcription factors with HLH, HD, bZip, NR and LIM domains make up around 70% of the basally and differentially expressed factors in rat β and α cells [see Additional File 4]. Complexity of bZip transcription factors functioning is achieved through dimerization and high throughput analysis of bZip-bZip interactions detected 200 preferential binding sites that regulate distinct genes [39]. Atf-3 and binding partners Fos and Jun are enriched in α cells while interacting partners Atf-6 and Xbp-1 are enriched in β cells [see Additional File 4]. Atf-6 and Xbp-1 are involved in initial stages of the unfolded protein response and higher expression of these genes might be indicative of a stronger unfolded protein response in β cells compared to α cells, as suggested by previous studies [40].

The quantitative nature of MPSS data and its digital counting of individual mRNA transcripts allow direct comparison of these datasets without further normalization. Comparison of MPSS expression in human islets to other tissues indicates that 940 genes are expressed at >80% specificity in the pancreatic islet cells. 324 out of 940 islet specific genes are enriched in β cells compared to α cells. Among these genes are well-known β cell-specific genes such as PDX1, NEUROD1, IAPP, TCF1, NKX6-1 and ICA-512 [see Additional File 7]. Massive β cell death takes place during T1D and to a certain extent in T2D, raising the possibility to search for β cell specific blood markers secreted or released by β cell-specific genes. It is expected that the levels of these β cell-specific blood proteins will reflect the operation of the networks present in these cells, which should allow us to distinguish between normal and diseased β cells. Moreover, new blood proteins may be released from β cells undergoing apoptosis thus providing an indication of the extent of β cell destruction. Accordingly, blood from healthy and recently diagnosed people could be monitored to follow β cell mass and network perturbation during disease progression. The results of the present study, and the newly constructed BCGA, provide a list of potential candidates for this novel approach.

It is worth mentioning that the information contained in the BCGA is affected by the limitations inherent to microarray platforms and the algorithms utilized for homologue detection. For example, there are currently no tools in the BCGA to match genes belonging to the same family across species. We expect to improve these aspects in the future releases.

## Conclusion

The BCGA is a repository that integrates high-throughput gene expression datasets obtained in untreated β cells and other cells of the pancreas. The available datasets include the rat β and α cells microarray data performed in this study, MPSS data from human pancreatic islets as well as other publicly available dataset. BCGA contains gene abundance estimates for each gene in different cell types across three species. It is well known that gene networks are dynamic and change during cell cycle and physiological/pathophysiological responses. Our BCGA data provides an initial picture of the status of these networks and levels of gene expression under basal conditions. These data enable interesting comparisons between different cells and cell lines in human, rat and mouse pancreas. Our analysis using different models of β cell expansion or reduction suggests that BCGA contains cell-specific data. This information will be valuable for future studies involving expression changes and dynamic processes. The presently developed data pipeline is set up to incorporate any new microarray data or other platforms, such as proteomics, and to make it available to the research community promptly on T1DBase. The atlas is available at T1DBase , a bioinformatics resource for T1D researchers [41]. This cumulative information, coupled to pathway analysis, will be of great value for the ultimate understanding of gene/protein networks regulating β cell development, function and death.

## Abbreviations

BCGA: β cell Gene Atlas; MPSS: Massively Parallel Signature Sequencing; Tpm: transcripts per million; FDR: False Discovery Rate.

## Competing interests

The authors declare that they have no competing interests.

## Authors' contributions

BK, D. Baxter, JR, DF and NW performed experiments, BK analyzed data, BK, D. Burdick and NG wrote software, BK, NG, DE and LH conceived of the study, and participated in its design and coordination. DE and NG helped to draft the manuscript. All authors read and approved the final manuscript.

## Pre-publication history

The pre-publication history for this paper can be accessed here:



## Supplementary Material

Additional file 1 **Supplementary methods**. Supplementary information about Methods and additional findings. Click here for file

Additional file 2 **MPSS gene expression data. **Tpm values for each gene in MPSS data. Click here for file

Additional file 3 G**O term enrichment analysis of MPSS data.** GO term enrichment analysis of 1: top 200 genes, 2: genes expressed lower than 25 transcripts per million (tpm) in the human islet MPSS data Click here for file

Additional file 4 **Transcription factor analysis**. Analysis of transcription factors in rat INS cell data based on domain classification. Click here for file

Additional file 5 **INS-1 cell microarray data analysis results**. This file contains 1. MAS5-normalized expression data of rat β, α and INS cell. 2. Differential expression analysis of genes expressed in β vs α cells. 3. Differential expression analysis of genes expressed in β vs INS cells. Click here for file

Additional file 6 **GO term enrichment analysis of INS cell data.** GO term enrichment analysis genes expressed in β, α and INS cells. Click here for file

Additional file 7 **Pancreatic islet-specific genes**. Genes that are enriched in pancreatic islets compared to other tissues in the body. Click here for file

Additional file 8 T**he overlap of these pancreatic islet-specific genes with β and α cell enriched genes**. The intersection of genes in Additional File 7 with either genes expressed strongly in either β or α cells. Click here for file
