# Supplementary material for: Detailed transcriptome atlas of the pancreatic beta cell
Source: BMC Med Genomics. 2009 Jan 15;2:3. doi: 10.1186/1755-8794-2-3 (PMC2635377; doi:10.1186/1755-8794-2-3)
Supplement: Additional file 1 — Supplementary methods. Supplementary information about Methods and additional findings. [file 1755-8794-2-3-S1.pdf]

Additional data file-1

Table of contents:

A. Supplementary Table of studies used to construct the BCGA (β Cell Gene Atlas)
B. Annotation of MPSS signatures
C. Discrepancy between MPSS and microarray data
D. Figure S2
E. RT-PCR analysis of transcripts detected by MPSS
F. Legends for Additional data files 2-8
G. References

A. Supplementary Table of studies used to construct the BCGA (β Cell Gene Atlas)

Authors	GEO	RAD3	Array Express	Cell/tissue type	Species	Reference
Akpinar et al.	gse3544			Islets	Rat	[1]
Cardozo et al		2021	E-CBIL-8	β cells	Rat	[2]
Cardozo et al		2020	E-CBIL-9	β cells	Rat	[3]
Casas et al	gse2253			MIN6 Cell lines	Mouse	
Dohi et al			E_MEXP_536	Islets	Mouse	[4]
Ge et al	gse2361			Islets	Human	
Gomis et al	gse2470			Islets	Rat	
Gunton et al		2160		Islets	Human	[5]
Habener et al		870		Islets	Human	
Ishikava et al	gse1542			Ductal cells	Human	[6]
Kaestner et al		1390		Islets	Mouse	
Kaur et al	gse769			Whole pancreas	Mouse	[7]
Kutlu et al		2002	E-CBIL-10	INS-1 Cell lines	Rat	[8]
Logsdon et al	gse3311			Whole pancreas	Rat	[9]
Maffei et al		751		Islets Exocrine cells	Human	[10]
Mathis et al	gse2254			Islets	Mouse	[11]
Parton et al	gse2582			Islets	Rat	[12]
Rasschaert et al		2001	E-CBIL-11	β cells	Rat	[13]
Shumeli et al	gse803			Islets	Human	[14]
Su et al	gse1133			Islets	Human	[15]
Thomas et al	gse1591			INS-1 Cell lines	Rat	[16]
Vukkadapu et al	gse1623			Islets	Mouse	[17]
Weir et al		810		β cells	Human	
Yliipaasto et al				Islets	Human	[18]
Yoon et al	gse2719			Islets	Human	
Zhang et al	gse2060			Islets, MIN6 cell lines	Human, mouse	[19]

Supplementary Table 1. The list of publications that were used for the Gene Atlas Construction

Legend. GEO: NCBI Gene Expression Omnibus database identifier [20], RAD3: RNA Abundance Database identifier [21], Array Express: EBI Array Express Database identifier [22].

B. Annotation of MPSS signatures

After the exploratory analysis of the MPSS results, we attempted to annotate the signatures and identify the gene sequences from which they originate (Figure S1). We performed Blat analysis with the most current version of the Human Genome Assembly (UCSC hg18). Blat is an alignment tool like BLAST, but it is structured differently. On DNA, Blat works by keeping an index of the entire genome in memory. Thus, the target database of BLAT is not a set of GenBank sequences, but instead an index derived from the assembly of the entire genome [23]. There were 16873 out of an initial number of 20719 signatures with exact hits to the genome, for which we could align the whole 17 bp (Figure S1). Of these 16873, 9906 signatures had a unique location on the human genome. Moreover there were 5161 signatures that were aligned to more than one location on the human genome, with more than one genomic location containing the signature sequence. The majority of these repetitive sequences (66%) had two or three different locations on the genome. In this group, we identified signatures that could be mapped to a unique coding sequence at a unique location while the rest of its coordinates did not contain any coding sequence. On the other hand, there were 3876 signatures that could not be mapped to any region on the human genome. A subset of these signatures, 801, could be mapped to EST sequence databases at NCBI. 2418 of the 3876 signatures could be aligned to the genome with one base off. These signatures are probably products of alternative splicing or individual polymorphisms.

Around 10% of all signatures, 2209, could neither be aligned to human genome nor aligned with an expressed sequence and were thus left unannotated. The correlation among these signatures was higher than the overall correlation (Spearman rank correlation, 0.75). The mean signature count for these unannotated transcripts was 507 and 424 tpm for islet sample 1 and 2, respectively. A proportion (5%) of the unannotated signatures had hits in the NCBI Trace Archive database and these might represent transcripts found within regions that are not assembled to the current human genome build.

C. **Figure S1**

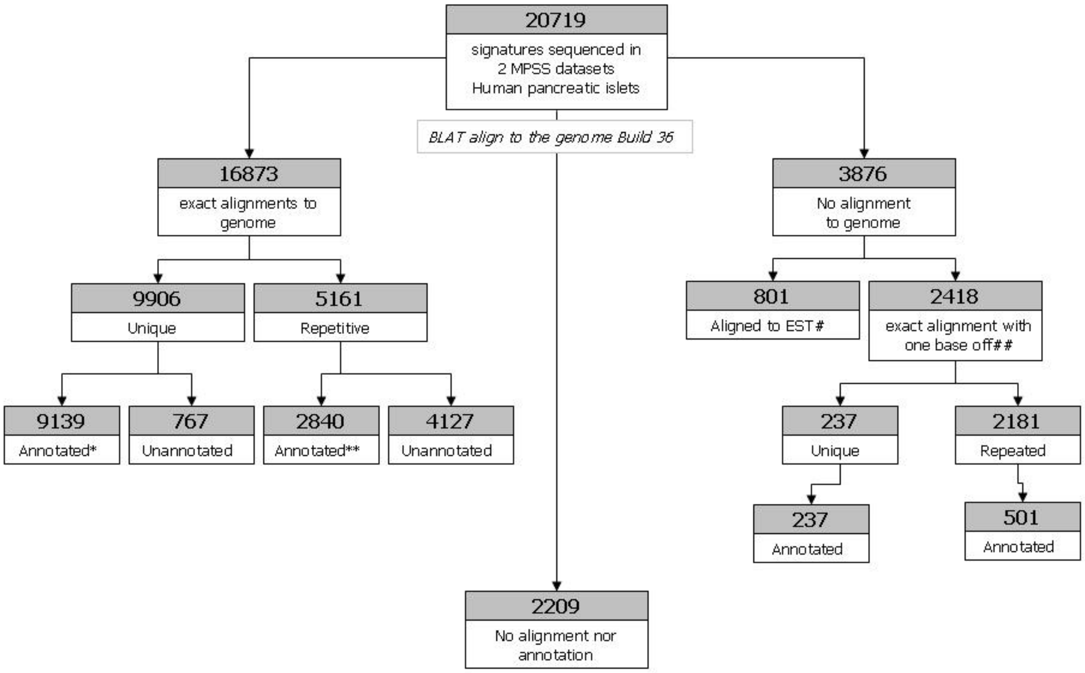

Figure S1: Summary of the annotation of the MPSS signatures

* Overlap with a coding sequence that has a Entrez Gene id (Refseq, mRNA, EST)
** Overlap with only 1 coding sequence that has a Entrez Gene id while the other signatures do not
# Blast hit in human EST database
Alignment to the genome with only one base difference

D. **Discrepancy between MPSS and microarray data**

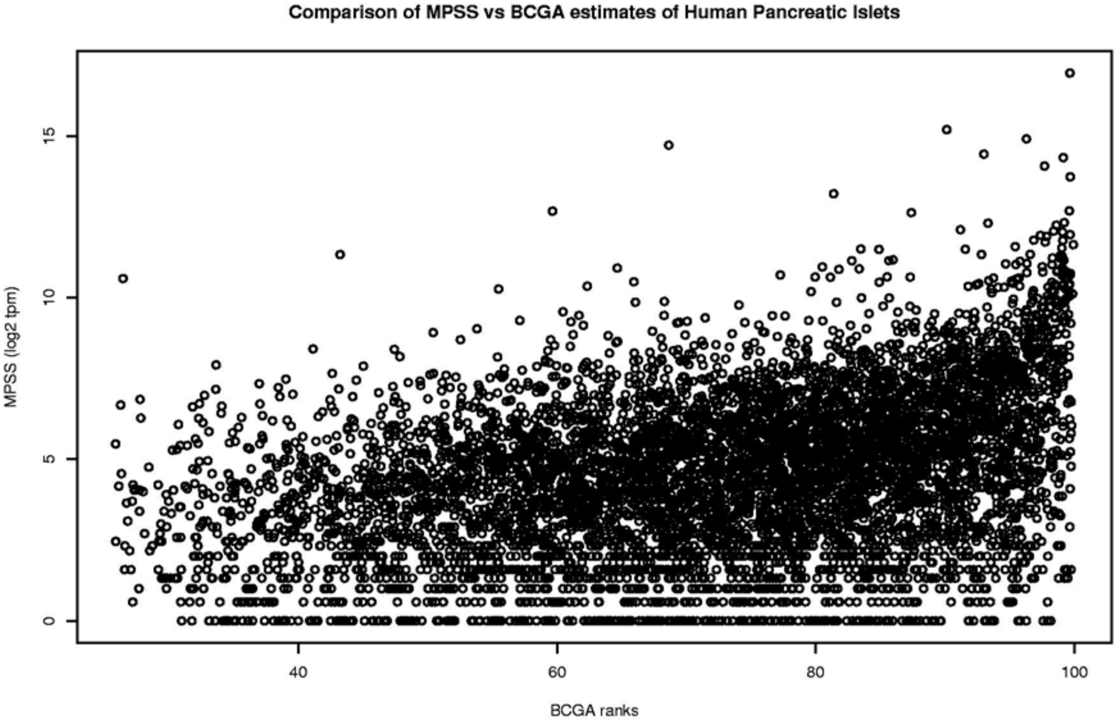

Figure S2: Comparison of MPSS data and BCGA (β Cell Gene Atlas) estimates for human pancreatic islets. Each dot corresponds to the level of expression of a gene detected by MPSS (Log 2 scale) and the rank of the gene in BCGA in human pancreatic islets. Most values agree and the higher expression of a gene in MPSS corresponds to the higher BCGA rank.

E. RT-PCR analysis of transcripts detected by MPSS

With the aim to assess whether transcripts detected by MPSS, but not by microarray, were really expressed, we performed RT-PCR analysis of MAFA, SAA1, INCA1, KCNIP3 and ZFXH2 genes in untreated control islets. We also included as a positive control in this analysis INS and NEUROD1. Although, MAFA is a well-described beta cell transcription factor, microarrays do not contain probes for this gene. MAFA is among the genes detected in human pancreatic islets by MPSS.

We used ABI Taqman probes to detect expression of these transcripts corrected by a control gene (RPLPO) in untreated human pancreatic islets cultured for different time points. These samples are part of a time series study of human islets exposed to cytokines for different time points (Kutlu et al., manuscript in preparation). We calculated the difference between average Ct values (Cycle of amplification that pass the threshold of detection) of RPLPO and tested genes. The Ct values were above 38, and the threshold was reliably detected in the ABI Real-Time PCR system. The results are expressed both

in terms of the cycle numbers to reach the levels of Insulin gene expression and the absolute copy numbers. The absolute copy values were obtained by preparing a standard curve with serial dilutions of insulin gene, corresponding to different copy numbers. The copy numbers of other genes were estimated using the relative abundance compared to the insulin gene.

Gene Name	Average difference in Ct (Tested Gene – RPLPO)	Number of cycle to reach Insulin levels	Copy number in sample
Insulin	-8.28	1	1.6E+7
Neurod1	2.87	11.15	7311.2
MAFA	5.45	13.73	1222.6
SAA1	7.63	15.91	269.8
INCA1	8.10	16.39	193.9
KCNIP3	7.83	16.11	234.0
ZFXH2	4.87	13.15	1828.5

F. Legends for Additional Files:

Additional File 2: MPSS gene expression data.
'Gene id' = Entrez Gene ids, 'Symbols' = HUGO names for each gene, 'description' = detailed gene name, 'islet1' = tpm counts in MPSS sample from islet 1, 'islet2' = tpm counts in MPSS sample from islet 2, 'average' = average tpm counts in MPSS sample in both samples

Additional File 3: GO term enrichment analysis of 1: top 200 genes, 2: genes expressed lower than 25 transcripts per million (tpm) in the human islet MPSS data P.value = Fisher's Exact test

Additional File 4: Transcription factor analysis

Additional File 5: MAS5-normalized expression data of rat beta, alpha and INS cells. (target intensity value set to 1500)
Gene id = Entrez Gene id, gene_names , Beta = gene signal intensity in rat beta cells, Alpha = gene signal intensity in rat alpha cells, INS = gene signal intensity in rat INS cells

Differential expression analysis of genes expressed in Beta vs Alpha.
Gene_id = Entrez Gene id, M= log2 sclae fold change (1 = 2 fold change), P.value = p value obtained using the moderated t-statistic obtained with shrinked variances using a Bayes Model , adj.P.Val= Adjusted (Benjamini and Hochberg procedure) p-values to correct for multiple testing.

Differential expression analysis of genes expressed in Beta vs INS.
Gene_id = Entrez Gene id, M= log2 scale fold change (1 = 2 fold change), P.value = p value obtained using the moderated t-statistic obtained with shrinked variances using a Bayes Model , adj.P.Val= Adjusted (Benjamini and Hochberg procedure) p-values to correct for multiple testing.

Additional File 6: GO term enrichment analysis genes expressed in Beta, Alpha and INS cells. P.value = Fisher's Exact test

Additional File 7: Pancreatic islet-specific genes
The file contains expression levels of genes in various tissues expressed in transcripts per million. Tissues include pancreatic islets (current data), adrenal gland, bladder, bone marrow, brain-amygdala, brain-caudate nucleus, brain-cerebellum, brain-corpus callosum, brain-fetal, brain-hypothalamus, brain-thalamus, heart, kidney, lung, mammary gland, pancreas, pituitary gland, placenta, prostate, retina, salivary gland, small intestine, spinal cord, spleen, stomach, testis, thymus, thyroid, trachea, uterus, colon anf monocytes. The column "Enrichment score in islets" indicates the percentage of average signature counts in islets to the overall count of signatures in islets and other tissues combined. A "yes" denotes if the signature is seen in islets only.

Additional File 8: Overlap of the islet-specific genes with β and α-cell enriched genes
There are two lists which contain the overlap of the "islet-enriched" genes with those that are expressed highly in rat beta or alpha cells.

G. References

1. **Akpinar P, Kuwajima S, Krutzfeldt J, Stoffel M: Tmem27: a cleaved and shed plasma membrane protein that stimulates pancreatic beta cell proliferation. *Cell Metab* 2005, 2(6):385-397.**
2. **Cardozo AK, Kruhoffer M, Leeman R, Orntoft T, Eizirik DL: Identification of novel cytokine-induced genes in pancreatic beta-cells by high-density oligonucleotide arrays. *Diabetes* 2001, 50(5):909-920.**
3. **Cardozo AK, Heimberg H, Heremans Y, Leeman R, Kutlu B, Kruhoffer M, Orntoft T, Eizirik DL: A comprehensive analysis of cytokine-induced and nuclear factor-kappa B-dependent genes in primary rat pancreatic beta-cells. *J Biol Chem* 2001, 276(52):48879-48886.**
4. **Dohi T, Salz W, Costa M, Ariyan C, Basadonna GP, Altieri DC: Inhibition of apoptosis by survivin improves transplantation of pancreatic islets for treatment of diabetes in mice. *EMBO Rep* 2006, 7(4):438-443.**
5. **Gunton JE, Kulkarni RN, Yim S, Okada T, Hawthorne WJ, Tseng YH, Roberson RS, Ricordi C, O'Connell PJ, Gonzalez FJ *et al*: Loss of ARNT/HIF1beta mediates altered gene expression and pancreatic-islet dysfunction in human type 2 diabetes. *Cell* 2005, 122(3):337-349.**

6. **Ishikawa M, Yoshida K, Yamashita Y, Ota J, Takada S, Kisanuki H, Koinuma K, Choi YL, Kaneda R, Iwao T *et al*: Experimental trial for diagnosis of pancreatic ductal carcinoma based on gene expression profiles of pancreatic ductal cells. *Cancer Sci* 2005, 96(7):387-393.**
7. **Kaur S, Norkina O, Ziemer D, Samuelson LC, De Lisle RC: Acidic duodenal pH alters gene expression in the cystic fibrosis mouse pancreas. *Am J Physiol Gastrointest Liver Physiol* 2004, 287(2):G480-490.**
8. **Kutlu B, Cardozo AK, Darville MI, Kruhoffer M, Magnusson N, Orntoft T, Eizirik DL: Discovery of gene networks regulating cytokine-induced dysfunction and apoptosis in insulin-producing INS-1 cells. *Diabetes* 2003, 52(11):2701-2719.**
9. **Kubisch CH, Gukovsky I, Lugea A, Pandol SJ, Kuick R, Misek DE, Hanash SM, Logsdon CD: Long-term ethanol consumption alters pancreatic gene expression in rats: a possible connection to pancreatic injury. *Pancreas* 2006, 33(1):68-76.**
10. **Maffei A, Liu Z, Witkowski P, Moschella F, Del Pozzo G, Liu E, Herold K, Winchester RJ, Hardy MA, Harris PE: Identification of tissue-restricted transcripts in human islets. *Endocrinology* 2004, 145(10):4513-4521.**
11. **Matos M, Park R, Mathis D, Benoist C: Progression to islet destruction in a cyclophosphamide-induced transgenic model: a microarray overview. *Diabetes* 2004, 53(9):2310-2321.**
12. **Parton LE, Diraison F, Neill SE, Ghosh SK, Rubino MA, Bisi JE, Briscoe CP, Rutter GA: Impact of PPARgamma overexpression and activation on pancreatic islet gene expression profile analyzed with oligonucleotide microarrays. *Am J Physiol Endocrinol Metab* 2004, 287(3):E390-404.**
13. **Rasschaert J, Liu D, Kutlu B, Cardozo AK, Kruhoffer M, TF OR, Eizirik DL: Global profiling of double stranded RNA- and IFN-gamma-induced genes in rat pancreatic beta cells. *Diabetologia* 2003, 46(12):1641-1657.**
14. **Yanai I, Benjamin H, Shmoish M, Chalifa-Caspi V, Shklar M, Ophir R, Bar-Even A, Horn-Saban S, Safran M, Domany E *et al*: Genome-wide midrange transcription profiles reveal expression level relationships in human tissue specification. *Bioinformatics* 2005, 21(5):650-659.**
15. **Su AI, Wiltshire T, Batalov S, Lapp H, Ching KA, Block D, Zhang J, Soden R, Hayakawa M, Kreiman G *et al*: A gene atlas of the mouse and human protein-encoding transcriptomes. *Proc Natl Acad Sci U S A* 2004, 101(16):6062-6067.**
16. **Thomas H, Senkel S, Erdmann S, Arndt T, Turan G, Klein-Hitpass L, Ryffel GU: Pattern of genes influenced by conditional expression of the transcription factors HNF6, HNF4alpha and HNF1beta in a pancreatic beta-cell line. *Nucleic Acids Res* 2004, 32(19):e150.**
17. **Vukkadapu SS, Belli JM, Ishii K, Jegga AG, Hutton JJ, Aronow BJ, Katz JD: Dynamic interaction between T cell-mediated beta-cell damage and beta-cell repair in the run up to autoimmune diabetes of the NOD mouse. *Physiol Genomics* 2005, 21(2):201-211.**
18. **Ylipaasto P, Kutlu B, Rasilainen S, Rasschaert J, Salmela K, Teerijoki H, Korsgren O, Lahesmaa R, Hovi T, Eizirik DL *et al*: Global profiling of**

coxsackievirus- and cytokine-induced gene expression in human pancreatic islets. ***Diabetologia*** **2005, 48(8):1510-1522.**

19. Zhang X, Odom DT, Koo SH, Conkright MD, Canettieri G, Best J, Chen H, Jenner R, Herbolsheimer E, Jacobsen E ***et al*****: Genome-wide analysis of cAMP-response element binding protein occupancy, phosphorylation, and target gene activation in human tissues.** ***Proc Natl Acad Sci U S A*** **2005, 102(12):4459-4464.**

20. Barrett T, Troup DB, Wilhite SE, Ledoux P, Rudnev D, Evangelista C, Kim IF, Soboleva A, Tomashevsky M, Edgar R: NCBI GEO: mining tens of millions of expression profiles--database and tools update. ***Nucleic Acids Res*** **2007, 35(Database issue):D760-765.**

21. Mazzarelli JM, Brestelli J, Gorski RK, Liu J, Manduchi E, Pinney DF, Schug J, White P, Kaestner KH, Stoeckert CJ, Jr.: EPConDB: a web resource for gene expression related to pancreatic development, beta-cell function and diabetes. ***Nucleic Acids Res*** **2007, 35(Database issue):D751-755.**

22. Parkinson H, Kapushesky M, Shojatalab M, Abeygunawardena N, Coulson R, Farne A, Holloway E, Kolesnykov N, Lilja P, Lukk M ***et al*****: ArrayExpress--a public database of microarray experiments and gene expression profiles.** ***Nucleic Acids Res*** **2007, 35(Database issue):D747-750.**

23. Hinrichs AS, Karolchik D, Baertsch R, Barber GP, Bejerano G, Clawson H, Diekhans M, Furey TS, Harte RA, Hsu F ***et al*****: The UCSC Genome Browser Database: update 2006.** ***Nucleic Acids Res*** **2006, 34(Database issue):D590-598.**

24. Denoeud F, Kapranov P, Ucla C, Frankish A, Castelo R, Drenkow J, Lagarde J, Alioto T, Manzano C, Chrast J ***et al*****: Prominent use of distal 5' transcription start sites and discovery of a large number of additional exons in ENCODE regions.** ***Genome Res*** **2007, 17(6):746-759.**

25. Jongeneel CV, Delorenzi M, Iseli C, Zhou D, Haudenschild CD, Khrebtukova I, Kuznetsov D, Stevenson BJ, Strausberg RL, Simpson AJ ***et al*****: An atlas of human gene expression from massively parallel signature sequencing (MPSS).** ***Genome Res*** **2005, 15(7):1007-1014.**

26. Siddiqui AS, Delaney AD, Schnerch A, Griffith OL, Jones SJ, Marra MA: Sequence biases in large scale gene expression profiling data. ***Nucleic Acids Res*** **2006, 34(12):e83.**
