# Supplementary material for: Detailed transcriptome atlas of the pancreatic beta cell
Source: BMC Med Genomics. 2009 Jan 15;2:3. doi: 10.1186/1755-8794-2-3 (PMC2635377; doi:10.1186/1755-8794-2-3)
Supplement: Additional file 2 — MPSS gene expression data. Tpm values for each gene in MPSS data. [file 1755-8794-2-3-S2.pdf]

gene_id	symbol	description	islet1	islet2	mean
3630	INS	insulin	115806	137701	126754
5068	REG3A	regenerating islet-derived 3 alpha	28372	46821	37597
57521	KIAA1303	raptor	19447	51634	35541
5967	REG1A	regenerating islet-derived 1 alpha (pancreatic stone protein,	44348	17588	30968
2641	GCG	glucagon	16163	37866	27015
5645	PRSS2	protease, serine, 2 (trypsin 2)	35590	8946	22268
2778	GNAS	GNAS complex locus	16287	27305	21796
6750	SST	somatostatin	22199	12592	17396
9568	GABBR2	gamma-aminobutyric acid (GABA) B receptor, 2	20203	12561	16382
440387	CTRB2	chymotrypsinogen B2	21726	5667	13697
653	BMP5	bone morphogenetic protein 5	5759	8451	7105
23521	RPL13A	ribosomal protein L13a	6447	6452	6449.5
5968	REG1B	regenerating islet-derived 1 beta (pancreatic stone protein, p	9519	3176	6347.5
3934	LCN2	lipocalin 2 (oncogene 24p3)	3855	6226	5040.5
4267	CD99	CD99 molecule	3789	5970	4879.5
1113	CHGA	chromogranin A (parathyroid secretory protein 1)	2371	6431	4401
5644	PRSS1	protease, serine, 1 (trypsin 1)	6982	1555	4268.5
6154	RPL26	ribosomal protein L26	3330	4292	3811
4946	OAZ1	ornithine decarboxylase antizyme 1	2419	4631	3525
1645	AKR1C1	aldo-keto reductase family 1, member C1 (dihydrodiol dehyd	5767	1204	3485.5
2895	GRID2	glutamate receptor, ionotropic, delta 2	2805	3955	3380
6168	RPL37A	ribosomal protein L37a	2822	3897	3359.5
728941	LOC728941	hypothetical protein LOC728941	2482	4237	3359.5
3326	HSP90AB1	heat shock protein 90kDa alpha (cytosolic), class B member	2966	3313	3139.5
2023	ENO1	enolase 1, (alpha)	1992	4249	3120.5
7425	VGF	VGF nerve growth factor inducible	2347	3489	2918
4282	MIF	macrophage migration inhibitory factor (glycosylation-inhibiti	1136	4683	2909.5
226	ALDOA	aldolase A, fructose-bisphosphate	1996	3772	2884
3481	IGF2	insulin-like growth factor 2 (somatomedin A)	1726	3600	2663
1938	EEF2	eukaryotic translation elongation factor 2	2888	2214	2551
375	ARF1	ADP-ribosylation factor 1	2286	2541	2413.5
1471	CST3	cystatin C (amyloid angiopathy and cerebral hemorrhage)	1869	2773	2321
9235	IL32	interleukin 32	2357	2269	2313
5034	P4HB	procollagen-proline, 2-oxoglutarate 4-dioxygenase (proline 4	2312	2231	2271.5
6277	S100A6	S100 calcium binding protein A6	1348	3088	2218
5265	SERPINA1	serpin peptidase inhibitor, clade A (alpha-1 antiproteinase, a	1196	3190	2193
9277	WDR46	WD repeat domain 46	4195	14	2104.5
1191	CLU	clusterin	1895	2306	2100.5
10169	SERF2	small EDRK-rich factor 2	1878	2291	2084.5
6208	RPS14	ribosomal protein S14	1625	2435	2030
6158	RPL28	ribosomal protein L28	1862	2124	1993
1277	COL1A1	collagen, type I, alpha 1	2843	1107	1975
6124	RPL4	ribosomal protein L4	1909	1998	1953.5
81532	HCCA2	HCCA2 protein	1634	2167	1900.5
6128	RPL6	ribosomal protein L6	1510	2192	1851
3107	HLA-C	major histocompatibility complex, class I, C	1440	2191	1815.5
10209	EIF1	eukaryotic translation initiation factor 1	1825	1770	1797.5
6152	RPL24	ribosomal protein L24	1895	1668	1781.5
7167	TPI1	triosephosphate isomerase 1	1446	2102	1774
486	FXYD2	FXYD domain containing ion transport regulator 2	1809	1711	1760

7314	UBB	ubiquitin B	1667	1668	1667.5
1364	CLDN4	claudin 4	1399	1934	1666.5
79144	C20orf149	chromosome 20 open reading frame 149	1925	1333	1629
3576	IL8	interleukin 8	1762	1433	1597.5
6833	ABCC8	ATP-binding cassette, sub-family C (CFTR/MRP), member 8	1687	1489	1588
7076	TIMP1	TIMP metallopeptidase inhibitor 1	1903	1214	1558.5
629	CFB	complement factor B	2196	904	1550
2899	GRIK3	glutamate receptor, ionotropic, kainate 3	779	2310	1544.5
1646	AKR1C2	aldo-keto reductase family 1, member C2 (dihydrodiol dehyd	2770	199	1484.5
10983	CCNI	cyclin I	1600	1317	1458.5
9518	GDF15	growth differentiation factor 15	1543	1352	1447.5
5315	PKM2	pyruvate kinase, muscle	1059	1831	1445
7104	TM4SF4	transmembrane 4 L six family member 4	1074	1810	1442
7168	TPM1	tropomyosin 1 (alpha)	1473	1378	1425.5
1358	CPA2	carboxypeptidase A2 (pancreatic)	2356	446	1401
6157	RPL27A	ribosomal protein L27a	1605	1143	1374
25824	PRDX5	peroxiredoxin 5	1216	1526	1371
9349	RPL23	ribosomal protein L23	1918	810	1364
7316	UBC	ubiquitin C	1014	1707	1360.5
1915	EEF1A1	eukaryotic translation elongation factor 1 alpha 1	1052	1637	1344.5
975	CD81	CD81 molecule	1337	1315	1326
1057	CELP	carboxyl ester lipase pseudogene	2072	550	1311
5284	PIGR	polymeric immunoglobulin receptor	676	1939	1307.5
7009	TEGT	testis enhanced gene transcript (BAX inhibitor 1)	1183	1385	1284
304	ANXA2P2	annexin A2 pseudogene 2	1184	1377	1280.5
972	CD74	CD74 molecule, major histocompatibility complex, class II inv	625	1851	1238
1719	DHFR	dihydrofolate reductase	1561	908	1234.5
92292	GLYATL1	glycine-N-acyltransferase-like 1	857	1611	1234
5878	RAB5C	RAB5C, member RAS oncogene family	920	1547	1233.5
292	SLC25A5	solute carrier family 25 (mitochondrial carrier; adenine nucle	1193	1192	1192.5
5319	PLA2G1B	phospholipase A2, group IB (pancreas)	1970	411	1190.5
2197	FAU	Finkel-Biskis-Reilly murine sarcoma virus (FBR-MuSV) ubiqu	810	1544	1177
378938	MALAT1	metastasis associated lung adenocarcinoma transcript 1 (no	1376	956	1166
166	AES	amino-terminal enhancer of split	811	1520	1165.5
55997	CFC1	cripto, FRL-1, cryptic family 1	692	1611	1151.5
6229	RPS24	ribosomal protein S24	1130	1171	1150.5
90701	SEC11C	SEC11 homolog C (S. cerevisiae)	1234	1063	1148.5
6132	RPL8	ribosomal protein L8	878	1413	1145.5
506	ATP5B	ATP synthase, H+ transporting, mitochondrial F1 complex, b	1109	1155	1132
4708	NDUFB2	NADH dehydrogenase (ubiquinone) 1 beta subcomplex, 2, 8	963	1270	1116.5
7178	TPT1	tumor protein, translationally-controlled 1	917	1311	1114
37	ACADVL	acyl-Coenzyme A dehydrogenase, very long chain	1017	1188	1102.5
6138	RPL15	ribosomal protein L15	1010	1183	1096.5
10158	PDZK1IP1	PDZK1 interacting protein 1	1065	1020	1042.5
10653	SPINT2	serine peptidase inhibitor, Kunitz type, 2	973	1046	1009.5
4316	MMP7	matrix metallopeptidase 7 (matrilysin, uterine)	766	1225	995.5
6185	RPN2	ribophorin II	926	1037	981.5
3094	HINT1	histidine triad nucleotide binding protein 1	704	1240	972
57142	RTN4	reticulon 4	826	1116	971
966	CD59	CD59 molecule, complement regulatory protein	1045	878	961.5
1329	COX5B	cytochrome c oxidase subunit Vb	938	981	959.5

2771	GNAI2	guanine nucleotide binding protein (G protein), alpha inhibitir	873	1017	945
4736	RPL10A	ribosomal protein L10a	1131	750	940.5
3688	ITGB1	integrin, beta 1 (fibronectin receptor, beta polypeptide, antige	727	1141	934
7077	TIMP2	TIMP metallopeptidase inhibitor 2	555	1304	929.5
9378	NRXN1	neurexin 1	967	884	925.5
8870	IER3	immediate early response 3	808	1036	922
5660	PSAP	prosaposin (variant Gaucher disease and variant metachrom	1124	703	913.5
11337	GABARAP	GABA(A) receptor-associated protein	674	1148	911
51330	TNFRSF12A	tumor necrosis factor receptor superfamily, member 12A	654	1123	888.5
6193	RPS5	ribosomal protein S5	902	821	861.5
10916	MAGED2	melanoma antigen family D, 2	659	1051	855
2934	GSN	gelsolin (amyloidosis, Finnish type)	692	1014	853
283120	H19	H19, imprinted maternally expressed untranslated mRNA	0	1686	843
5406	PNLIP	pancreatic lipase	1520	147	833.5
9045	RPL14	ribosomal protein L14	1336	312	824
27018	NGFRAP1	nerve growth factor receptor (TNFRSF16) associated proteir	568	1072	820
203068	TUBB	tubulin, beta	718	918	818
3916	LAMP1	lysosomal-associated membrane protein 1	1047	580	813.5
91368	MGC13017	similar to RIKEN cDNA A430101B06 gene	1035	591	813
387	RHOA	ras homolog gene family, member A	690	899	794.5
10109	ARPC2	actin related protein 2/3 complex, subunit 2, 34kDa	611	973	792
79002	C19orf43	chromosome 19 open reading frame 43	573	952	762.5
1401	CRP	C-reactive protein, pentraxin-related	1365	149	757
5479	PPIB	peptidylprolyl isomerase B (cyclophilin B)	697	811	754
2950	GSTP1	glutathione S-transferase pi	1017	478	747.5
5052	PRDX1	peroxiredoxin 1	948	547	747.5
10961	ERP29	endoplasmic reticulum protein 29	715	766	740.5
5694	PSMB6	proteasome (prosome, macropain) subunit, beta type, 6	623	841	732
10952	SEC61B	Sec61 beta subunit	666	784	725
6748	SSR4	signal sequence receptor, delta (translocon-associated prote	896	547	721.5
2877	GPX2	glutathione peroxidase 2 (gastrointestinal)	901	529	715
10975	UQCR	ubiquinol-cytochrome c reductase, 6.4kDa subunit	585	839	712
23770	FKBP8	FK506 binding protein 8, 38kDa	660	754	707
57706	DENND1A	DENN/MADD domain containing 1A	703	695	699
5636	PRPSAP2	phosphoribosyl pyrophosphate synthetase-associated protei	798	586	692
293	SLC25A6	solute carrier family 25 (mitochondrial carrier; adenine nucle	585	786	685.5
1495	CTNNA1	catenin (cadherin-associated protein), alpha 1, 102kDa	432	933	682.5
29106	SCG3	secretogranin III	379	980	679.5
10487	CAP1	CAP, adenylate cyclase-associated protein 1 (yeast)	175	1178	676.5
5037	PEBP1	phosphatidylethanolamine binding protein 1	675	673	674
390332	LOC390332	similar to retinol dehydrogenase 16	508	836	672
780	DDR1	discoidin domain receptor family, member 1	839	495	667
950	SCARB2	scavenger receptor class B, member 2	729	601	665
10590	SCGN	secretagogin, EF-hand calcium binding protein	792	536	664
54543	TOMM7	translocase of outer mitochondrial membrane 7 homolog (ye	365	961	663
6746	SSR2	signal sequence receptor, beta (translocon-associated protei	709	599	654
7529	YWHAB	tyrosine 3-monooxygenase/tryptophan 5-monooxygenase ac	475	820	647.5
5066	PAM	peptidylglycine alpha-amidating monooxygenase	706	588	647
51773	RSF1	remodeling and spacing factor 1	637	650	643.5
1192	CLIC1	chloride intracellular channel 1	487	796	641.5
29927	SEC61A1	Sec61 alpha 1 subunit (S. cerevisiae)	687	594	640.5

2919	CXCL1	chemokine (C-X-C motif) ligand 1 (melanoma growth stimula	858	417	637.5
56005	C19orf10	chromosome 19 open reading frame 10	763	508	635.5
7422	VEGFA	vascular endothelial growth factor A	536	731	633.5
4043	LRPAP1	low density lipoprotein receptor-related protein associated pr	541	722	631.5
10227	TETRAN	tetracycline transporter-like protein	719	539	629
7317	UBE1	ubiquitin-activating enzyme E1 (A1S9T and BN75 temperatu	673	574	623.5
723961	INS-IGF2	insulin- insulin-like growth factor 2	852	390	621
7534	YWHAZ	tyrosine 3-monooxygenase/tryptophan 5-monooxygenase ac	538	691	614.5
509	ATP5C1	ATP synthase, H+ transporting, mitochondrial F1 complex, g	453	773	613
8644	AKR1C3	aldo-keto reductase family 1, member C3 (3-alpha hydroxyst	764	459	611.5
4924	NUCB1	nucleobindin 1	355	863	609
50506	DUOX2	dual oxidase 2	605	604	604.5
7103	TSPAN8	tetraspanin 8	384	823	603.5
2495	FTH1	ferritin, heavy polypeptide 1	1204	0	602
51142	CHCHD2	coiled-coil-helix-coiled-coil-helix domain containing 2	570	632	601
10537	UBD	ubiquitin D	591	603	597
960	CD44	CD44 molecule (Indian blood group)	868	316	592
90861	HN1L	hematological and neurological expressed 1-like	1153	25	589
4582	MUC1	mucin 1, cell surface associated	612	564	588
81502	HM13	histocompatibility (minor) 13	698	460	579
6628	SNRPB	small nuclear ribonucleoprotein polypeptides B and B1	534	624	579
1460	CSNK2B	casein kinase 2, beta polypeptide	463	690	576.5
50854	C6orf48	chromosome 6 open reading frame 48	442	707	574.5
7873	ARMET	arginine-rich, mutated in early stage tumors	673	473	573
5080	PAX6	paired box gene 6 (aniridia, keratitis)	418	713	565.5
4357	MPST	mercaptopyruvate sulfurtransferase	274	857	565.5
7086	TKT	transketolase (Wernicke-Korsakoff syndrome)	735	385	560
7431	VIM	vimentin	298	821	559.5
283131	TncRNA	trophoblast-derived noncoding RNA	453	664	558.5
51604	PIGT	phosphatidylinositol glycan anchor biosynthesis, class T	568	544	556
1327	COX4I1	cytochrome c oxidase subunit IV isoform 1	769	329	549
710	SERPING1	serpin peptidase inhibitor, clade G (C1 inhibitor), member 1,	529	559	544
64787	EPS8L2	EPS8-like 2	491	597	544
8714	ABCC3	ATP-binding cassette, sub-family C (CFTR/MRP), member 3	647	438	542.5
23423	TMED3	transmembrane emp24 protein transport domain containing 3	625	460	542.5
7857	SCG2	secretogranin II (chromogranin C)	364	721	542.5
311	ANXA11	annexin A11	420	659	539.5
2821	GPI	glucose phosphate isomerase	318	760	539
2907	GRINA	glutamate receptor, ionotropic, N-methyl D-asparate-associa	509	568	538.5
5464	PPA1	pyrophosphatase (inorganic) 1	512	558	535
1153	CIRBP	cold inducible RNA binding protein	344	718	531
3315	HSPB1	heat shock 27kDa protein 1	561	494	527.5
351	APP	amyloid beta (A4) precursor protein (peptidase nexin-II, Alzh	261	786	523.5
4666	NACA	nascent-polypeptide-associated complex alpha polypeptide	483	559	521
1778	DYNC1H1	dynein, cytoplasmic 1, heavy chain 1	351	688	519.5
6881	TAF10	TAF10 RNA polymerase II, TATA box binding protein (TBP)-	532	491	511.5
3074	HEXB	hexosaminidase B (beta polypeptide)	522	496	509
53635	PTOV1	prostate tumor overexpressed gene 1	448	566	507
51060	TXNDC12	thioredoxin domain containing 12 (endoplasmic reticulum)	625	386	505.5
6659	SOX4	SRY (sex determining region Y)-box 4	376	631	503.5
1727	CYB5R3	cytochrome b5 reductase 3	504	499	501.5

5687	PSMA6	proteasome (prosome, macropain) subunit, alpha type, 6	463	537	500
7277	TUBA1	tubulin, alpha 1	591	407	499
5502	PPP1R1A	protein phosphatase 1, regulatory (inhibitor) subunit 1A	322	675	498.5
5539	PPY	pancreatic polypeptide	0	997	498.5
10549	PRDX4	peroxiredoxin 4	491	500	495.5
216	ALDH1A1	aldehyde dehydrogenase 1 family, member A1	324	665	494.5
3490	IGFBP7	insulin-like growth factor binding protein 7	480	506	493
9060	PAPSS2	3'-phosphoadenosine 5'-phosphosulfate synthase 2	419	565	492
10935	PRDX3	peroxiredoxin 3	473	506	489.5
29952	DPP7	dipeptidyl-peptidase 7	311	666	488.5
2896	GRN	granulin	613	356	484.5
5763	PTMS	parathymosin	410	558	484
1278	COL1A2	collagen, type I, alpha 2	725	242	483.5
5430	POLR2A	polymerase (RNA) II (DNA directed) polypeptide A, 220kDa	288	676	482
977	CD151	CD151 molecule (Raph blood group)	274	690	482
87	ACTN1	actinin, alpha 1	323	637	480
10525	HYOU1	hypoxia up-regulated 1	296	650	473
27230	SERP1	stress-associated endoplasmic reticulum protein 1	623	318	470.5
518	ATP5G3	ATP synthase, H+ transporting, mitochondrial F0 complex, s	481	454	467.5
3675	ITGA3	integrin, alpha 3 (antigen CD49C, alpha 3 subunit of VLA-3 r	236	696	466
388610	LOC388610	hypothetical LOC388610	406	517	461.5
5901	RAN	RAN, member RAS oncogene family	332	591	461.5
440400	MGC71993	similar to DNA segment, Chr 11, Brigham & Womens Geneti	370	550	460
2802	GOLGA3	golgi autoantigen, golgin subfamily a, 3	489	429	459
10397	NDRG1	N-myc downstream regulated gene 1	238	680	459
1363	CPE	carboxypeptidase E	615	302	458.5
285773	LOC285773	hypothetical protein LOC285773	382	535	458.5
50486	G0S2	G0/G1switch 2	784	131	457.5
4725	NDUFS5	NADH dehydrogenase (ubiquinone) Fe-S protein 5, 15kDa (I	428	481	454.5
727826	LOC727826	similar to ribosomal protein S11	217	687	452
91689	C22orf32	chromosome 22 open reading frame 32	365	537	451
2920	CXCL2	chemokine (C-X-C motif) ligand 2	625	271	448
6418	SET	SET translocation (myeloid leukemia-associated)	331	564	447.5
5879	RAC1	ras-related C3 botulinum toxin substrate 1 (rho family, small	443	449	446
2130	EWSR1	Ewing sarcoma breakpoint region 1	325	566	445.5
54361	WNT4	wingless-type MMTV integration site family, member 4	361	526	443.5
7052	TGM2	transglutaminase 2 (C polypeptide, protein-glutamine-gamma	428	453	440.5
801	CALM1	calmodulin 1 (phosphorylase kinase, delta)	551	329	440
9500	MAGED1	melanoma antigen family D, 1	357	523	440
308	ANXA5	annexin A5	370	506	438
4718	NDUFC2	NADH dehydrogenase (ubiquinone) 1, subcomplex unknowr	333	541	437
388	RHOB	ras homolog gene family, member B	331	543	437
2547	XRCC6	X-ray repair complementing defective repair in Chinese ham	522	350	436
10400	PEMT	phosphatidylethanolamine N-methyltransferase	297	573	435
481	ATP1B1	ATPase, Na+/K+ transporting, beta 1 polypeptide	367	496	431.5
10960	LMAN2	lectin, mannose-binding 2	534	328	431
513	ATP5D	ATP synthase, H+ transporting, mitochondrial F1 complex, d	402	460	431
3304	HSPA1B	heat shock 70kDa protein 1B	378	478	428
25844	YIPF3	Yip1 domain family, member 3	307	530	418.5
9948	WDR1	WD repeat domain 1	340	493	416.5
1345	COX6C	cytochrome c oxidase subunit VIc	323	510	416.5

55959	SULF2	sulfatase 2	556	274	415
4832	NME3	non-metastatic cells 3, protein expressed in	389	441	415
6210	RPS15A	ribosomal protein S15a	79	745	412
10972	TMED10	transmembrane emp24-like trafficking protein 10 (yeast)	250	573	411.5
1028	CDKN1C	cyclin-dependent kinase inhibitor 1C (p57, Kip2)	183	635	409
1650	DDOST	dolichyl-diphosphooligosaccharide-protein glycosyltransfera	528	289	408.5
55384	MEG3	maternally expressed 3	349	468	408.5
682	BSG	basigin (Ok blood group)	456	352	404
5641	LGMN	legumain	307	495	401
10103	TSPAN1	tetraspanin 1	162	630	396
7182	NR2C2	nuclear receptor subfamily 2, group C, member 2	327	456	391.5
6205	RPS11	ribosomal protein S11	0	780	390
1992	SERPINB1	serpin peptidase inhibitor, clade B (ovalbumin), member 1	288	490	389
128240	APOA1BP	apolipoprotein A-I binding protein	270	508	389
9446	GSTO1	glutathione S-transferase omega 1	352	422	387
388125	NLF2	nuclear localized factor 2	323	450	386.5
8766	RAB11A	RAB11A, member RAS oncogene family	300	471	385.5
9528	TMEM59	transmembrane protein 59	311	457	384
808	CALM3	calmodulin 3 (phosphorylase kinase, delta)	508	259	383.5
54940	OCIAD1	OCIA domain containing 1	398	367	382.5
4833	NME4	non-metastatic cells 4, protein expressed in	296	465	380.5
2286	FKBP2	FK506 binding protein 2, 13kDa	449	308	378.5
8655	DYNLL1	dynein, light chain, LC8-type 1	274	483	378.5
51160	VPS28	vacuolar protein sorting 28 homolog (S. cerevisiae)	351	404	377.5
10155	TRIM28	tripartite motif-containing 28	327	428	377.5
10992	SF3B2	splicing factor 3b, subunit 2, 145kDa	376	377	376.5
23353	UNC84A	unc-84 homolog A (C. elegans)	280	467	373.5
51631	LUC7L2	LUC7-like 2 (S. cerevisiae)	221	524	372.5
1366	CLDN7	claudin 7	376	367	371.5
11330	CTRC	chymotrypsin C (caldecrin)	620	122	371
10618	TGOLN2	trans-golgi network protein 2	242	499	370.5
7295	TXN	thioredoxin	353	387	370
3914	LAMB3	laminin, beta 3	591	143	367
3482	IGF2R	insulin-like growth factor 2 receptor	306	426	366
5686	PSMA5	proteasome (prosome, macropain) subunit, alpha type, 5	315	413	364
29100	HSPC171	HSPC171 protein	459	268	363.5
51371	POMP	proteasome maturation protein	353	374	363.5
55004	C11orf59	chromosome 11 open reading frame 59	244	481	362.5
284361	LOC284361	hematopoietic signal peptide-containing	295	426	360.5
4061	LY6E	lymphocyte antigen 6 complex, locus E	236	485	360.5
5798	PTPRN	protein tyrosine phosphatase, receptor type, N	503	215	359
2987	GUK1	guanylate kinase 1	422	295	358.5
64856	VWA1	von Willebrand factor A domain containing 1	414	300	357
23753	SDF2L1	stromal cell-derived factor 2-like 1	426	286	356
360	AQP3	aquaporin 3 (Gill blood group)	197	514	355.5
2720	GLB1	galactosidase, beta 1	325	384	354.5
6050	RNH1	ribonuclease/angiogenin inhibitor 1	414	294	354
3032	HADHB	hydroxyacyl-Coenzyme A dehydrogenase/3-ketoacyl-Coenz	443	258	350.5
23646	PLD3	phospholipase D family, member 3	465	233	349
79006	METRN	meteorin, glial cell differentiation regulator	203	495	349
26471	NUPR1	nuclear protein 1	404	289	346.5

6638	SNRPN	small nuclear ribonucleoprotein polypeptide N	374	319	346.5
126003	TRAPPC5	trafficking protein particle complex 5	330	362	346
537	ATP6AP1	ATPase, H+ transporting, lysosomal accessory protein 1	315	377	346
824	CAPN2	calpain 2, (m/II) large subunit	290	401	345.5
80700	UBXD1	UBX domain containing 1	288	398	343
222962	SLC29A4	solute carrier family 29 (nucleoside transporters), member 4	335	347	341
9512	PMPCB	peptidase (mitochondrial processing) beta	310	372	341
7335	UBE2V1	ubiquitin-conjugating enzyme E2 variant 1	258	424	341
5211	PFKL	phosphofructokinase, liver	213	468	340.5
5799	PTPRN2	protein tyrosine phosphatase, receptor type, N polypeptide 2	250	429	339.5
10897	YIF1A	Yip1 interacting factor homolog A (S. cerevisiae)	327	351	339
1889	ECE1	endothelin converting enzyme 1	382	291	336.5
8721	EDF1	endothelial differentiation-related factor 1	328	345	336.5
140809	SRXN1	sulfiredoxin 1 homolog (S. cerevisiae)	512	160	336
51065	RPS27L	ribosomal protein S27-like	378	292	335
1087	CEACAM7	carcinoembryonic antigen-related cell adhesion molecule 7	203	467	335
3872	KRT17	keratin 17	93	576	334.5
9741	LAPTM4A	lysosomal-associated protein transmembrane 4 alpha	189	479	334
51079	NDUFA13	NADH dehydrogenase (ubiquinone) 1 alpha subcomplex, 13	264	403	333.5
378	ARF4	ADP-ribosylation factor 4	311	355	333
1175	AP2S1	adaptor-related protein complex 2, sigma 1 subunit	349	314	331.5
4709	NDUFB3	NADH dehydrogenase (ubiquinone) 1 beta subcomplex, 3, 1	380	281	330.5
6430	SFRS5	splicing factor, arginine/serine-rich 5	242	419	330.5
8664	EIF3S7	eukaryotic translation initiation factor 3, subunit 7 zeta, 66/67	370	289	329.5
3303	HSPA1A	heat shock 70kDa protein 1A	250	407	328.5
539	ATP5O	ATP synthase, H+ transporting, mitochondrial F1 complex, C	138	519	328.5
4082	MARCKS	myristoylated alanine-rich protein kinase C substrate	191	464	327.5
204	AK2	adenylate kinase 2	227	426	326.5
10540	DCTN2	dynactin 2 (p50)	332	320	326
5518	PPP2R1A	protein phosphatase 2 (formerly 2A), regulatory subunit A (P	193	459	326
10519	CIB1	calcium and integrin binding 1 (calmyrin)	296	355	325.5
3313	HSPA9	heat shock 70kDa protein 9 (mortalin)	245	406	325.5
2664	GDI1	GDP dissociation inhibitor 1	323	325	324
4720	NDUFS2	NADH dehydrogenase (ubiquinone) Fe-S protein 2, 49kDa (I	292	356	324
3959	LGALS3BP	lectin, galactoside-binding, soluble, 3 binding protein	516	131	323.5
7384	UQCRC1	ubiquinol-cytochrome c reductase core protein I	250	396	323
6609	SMPD1	sphingomyelin phosphodiesterase 1, acid lysosomal (acid sp	435	209	322
5688	PSMA7	proteasome (prosome, macropain) subunit, alpha type, 7	273	371	322
3015	H2AFZ	H2A histone family, member Z	282	361	321.5
84707	BEX2	brain expressed X-linked 2	282	360	321
3336	HSPE1	heat shock 10kDa protein 1 (chaperonin 10)	276	366	321
170463	SSBP4	single stranded DNA binding protein 4	152	490	321
5714	PSMD8	proteasome (prosome, macropain) 26S subunit, non-ATPase	311	325	318
84171	LOXL4	lysyl oxidase-like 4	288	340	314
64834	ELOVL1	elongation of very long chain fatty acids (FEN1/Elo2, SUR4/E	262	366	314
11030	RBPMS	RNA binding protein with multiple splicing	213	415	314
26173	INTS1	integrator complex subunit 1	357	269	313
83795	KCNK16	potassium channel, subfamily K, member 16	185	441	313
10509	SEMA4B	sema domain, immunoglobulin domain (Ig), transmembrane	146	480	313
9246	UBE2L6	ubiquitin-conjugating enzyme E2L 6	207	418	312.5
51637	C14orf166	chromosome 14 open reading frame 166	250	374	312

4070	TACSTD2	tumor-associated calcium signal transducer 2	209	415	312
5266	PI3	peptidase inhibitor 3, skin-derived (SKALP)	497	126	311.5
3069	HDLBP	high density lipoprotein binding protein (vigilin)	457	166	311.5
1508	CTSB	cathepsin B	213	407	310
1973	EIF4A1	eukaryotic translation initiation factor 4A, isoform 1	404	214	309
51046	ST8SIA3	ST8 alpha-N-acetyl-neuraminide alpha-2,8-sialyltransferase	277	341	309
10882	C1QL1	complement component 1, q subcomponent-like 1	416	201	308.5
169026	SLC30A8	solute carrier family 30 (zinc transporter), member 8	185	431	308
22883	CLSTN1	calsyntenin 1	134	482	308
2752	GLUL	glutamate-ammonia ligase (glutamine synthetase)	321	294	307.5
64710	NUCKS1	nuclear casein kinase and cyclin-dependent kinase substrate	213	401	307
552900	BOLA2	bolA homolog 2 (E. coli)	337	272	304.5
3093	HIP2	huntingtin interacting protein 2	270	339	304.5
11258	DCTN3	dynactin 3 (p22)	250	359	304.5
9961	MVP	major vault protein	317	290	303.5
6125	RPL5	ribosomal protein L5	278	328	303
51310	SLC22A17	solute carrier family 22 (organic cation transporter), member	232	372	302
51150	SDF4	stromal cell derived factor 4	280	320	300
6319	SCD	stearoyl-CoA desaturase (delta-9-desaturase)	279	321	300
6281	S100A10	S100 calcium binding protein A10	272	328	300
54461	FBXW5	F-box and WD-40 domain protein 5	296	302	299
6342	SCP2	sterol carrier protein 2	277	321	299
126328	NDUFA11	NADH dehydrogenase (ubiquinone) 1 alpha subcomplex, 11	280	317	298.5
5885	RAD21	RAD21 homolog (S. pombe)	224	373	298.5
79095	C9orf16	chromosome 9 open reading frame 16	154	442	298
6804	STX1A	syntaxin 1A (brain)	246	349	297.5
7763	ZFAND5	zinc finger, AN1-type domain 5	213	382	297.5
1655	DDX5	DEAD (Asp-Glu-Ala-Asp) box polypeptide 5	162	433	297.5
30851	TAX1BP3	Tax1 (human T-cell leukemia virus type I) binding protein 3	148	444	296
57410	SCYL1	SCY1-like 1 (S. cerevisiae)	225	366	295.5
259	AMBP	alpha-1-microglobulin/bikunin precursor	305	284	294.5
29997	GLTSCR2	glioma tumor suppressor candidate region gene 2	379	209	294
4259	MGST3	microsomal glutathione S-transferase 3	218	370	294
1577	CYP3A5	cytochrome P450, family 3, subfamily A, polypeptide 5	412	170	291
10594	PRPF8	PRP8 pre-mRNA processing factor 8 homolog (S. cerevisiae	178	402	290
7132	TNFRSF1A	tumor necrosis factor receptor superfamily, member 1A	383	196	289.5
2665	GDI2	GDP dissociation inhibitor 2	164	414	289
65264	UBE2Z	ubiquitin-conjugating enzyme E2Z (putative)	336	240	288
641638	SNHG6	small nucleolar RNA host gene (non-protein coding) 6	209	365	287
10284	SAP18	Sin3A-associated protein, 18kDa	179	395	287
7263	TST	thiosulfate sulfurtransferase (rhodanese)	292	281	286.5
83857	TMTC1	transmembrane and tetratricopeptide repeat containing 1	144	429	286.5
81873	ARPC5L	actin related protein 2/3 complex, subunit 5-like	278	294	286
5792	PTPRF	protein tyrosine phosphatase, receptor type, F	215	355	285
3312	HSPA8	heat shock 70kDa protein 8	278	291	284.5
10237	SLC35B1	solute carrier family 35, member B1	164	405	284.5
10971	YWHAQ	tyrosine 3-monooxygenase/tryptophan 5-monooxygenase ac	242	325	283.5
2935	GSPT1	G1 to S phase transition 1	199	368	283.5
1615	DARS	aspartyl-tRNA synthetase	203	363	283
1649	DDIT3	DNA-damage-inducible transcript 3	150	416	283
51596	CUTA	cutA divalent cation tolerance homolog (E. coli)	256	308	282

23042	KIAA0251	KIAA0251 protein	341	220	280.5
284371	LOC284371	hypothetical protein LOC284371	40	521	280.5
23194	FBXL7	F-box and leucine-rich repeat protein 7	458	102	280
5209	PFKFB3	6-phosphofructo-2-kinase/fructose-2,6-biphosphatase 3	91	467	279
396	ARHGDIA	Rho GDP dissociation inhibitor (GDI) alpha	229	327	278
8788	DLK1	delta-like 1 homolog (Drosophila)	200	356	278
5445	PON2	paraoxonase 2	262	293	277.5
23261	CAMTA1	calmodulin binding transcription activator 1	233	322	277.5
4676	NAP1L4	nucleosome assembly protein 1-like 4	197	358	277.5
127687	C1orf122	chromosome 1 open reading frame 122	195	359	277
2316	FLNA	filamin A, alpha (actin binding protein 280)	217	336	276.5
514	ATP5E	ATP synthase, H+ transporting, mitochondrial F1 complex, e	336	216	276
4125	MAN2B1	mannosidase, alpha, class 2B, member 1	288	264	276
9071	CLDN10	claudin 10	455	96	275.5
23256	SCFD1	sec1 family domain containing 1	325	224	274.5
23451	SF3B1	splicing factor 3b, subunit 1, 155kDa	282	267	274.5
9380	GRHPR	glyoxylate reductase/hydroxypyruvate reductase	248	300	274
4312	MMP1	matrix metallopeptidase 1 (interstitial collagenase)	122	426	274
427	ASAH1	N-acylsphingosine amidohydrolase (acid ceramidase) 1	290	257	273.5
4478	MSN	moesin	262	284	273
56937	TMEPAI	transmembrane, prostate androgen induced RNA	282	263	272.5
10723	SLC12A7	solute carrier family 12 (potassium/chloride transporters), me	150	391	270.5
29099	COMMD9	COMM domain containing 9	231	309	270
23207	PLEKHM2	pleckstrin homology domain containing, family M (with RUN	116	422	269
7270	TTF1	transcription termination factor, RNA polymerase I	311	226	268.5
3727	JUND	jun D proto-oncogene	363	172	267.5
79586	CHPF	chondroitin polymerizing factor	301	233	267
1917	EEF1A2	eukaryotic translation elongation factor 1 alpha 2	268	265	266.5
6625	SNRP70	small nuclear ribonucleoprotein 70kDa polypeptide (RNP ant	370	162	266
7267	TTC3	tetratricopeptide repeat domain 3	204	328	266
83658	DYNLRB1	dynein, light chain, roadblock-type 1	270	260	265
51655	RASD1	RAS, dexamethasone-induced 1	465	64	264.5
149603	RNF187	ring finger protein 187	333	196	264.5
55111	PLEKHJ1	pleckstrin homology domain containing, family J member 1	268	260	264
4616	GADD45B	growth arrest and DNA-damage-inducible, beta	225	303	264
9538	EI24	etoposide induced 2.4 mRNA	177	351	264
1312	COMT	catechol-O-methyltransferase	221	306	263.5
1981	EIF4G1	eukaryotic translation initiation factor 4 gamma, 1	204	322	263
8636	SSNA1	Sjogren's syndrome nuclear autoantigen 1	121	404	262.5
2873	GPS1	G protein pathway suppressor 1	221	302	261.5
2548	GAA	glucosidase, alpha; acid (Pompe disease, glycogen storage	163	360	261.5
80347	COASY	Coenzyme A synthase	196	324	260
706	TSPO	translocator protein (18kDa)	266	250	258
10521	DDX17	DEAD (Asp-Glu-Ala-Asp) box polypeptide 17	182	332	257
51061	TXNDC11	thioredoxin domain containing 11	311	201	256
5859	QARS	glutaminyl-tRNA synthetase	311	201	256
738	C11orf2	chromosome 11 open reading frame2	297	215	256
55450	CAMK2N1	calcium/calmodulin-dependent protein kinase II inhibitor 1	178	334	256
2267	FGL1	fibrinogen-like 1	274	237	255.5
10273	STUB1	STIP1 homology and U-box containing protein 1	262	248	255
6947	TCN1	transcobalamin I (vitamin B12 binding protein, R binder famil	165	345	255

10016	PDCD6	programmed cell death 6	246	263	254.5
3957	LGALS2	lectin, galactoside-binding, soluble, 2 (galectin 2)	322	186	254
79081	C11orf48	chromosome 11 open reading frame 48	268	240	254
79042	TSEN34	tRNA splicing endonuclease 34 homolog (S. cerevisiae)	221	282	251.5
4691	NCL	nucleolin	198	305	251.5
51520	LARS	leucyl-tRNA synthetase	160	341	250.5
6455	SH3GL1	SH3-domain GRB2-like 1	270	230	250
374882	UNQ501	MBC3205	182	317	249.5
118	ADD1	adducin 1 (alpha)	245	252	248.5
51280	GOLPH2	golgi phosphoprotein 2	164	332	248
5970	RELA	v-rel reticuloendotheliosis viral oncogene homolog A, nuclea	293	202	247.5
2628	GATM	glycine amidinotransferase (L-arginine:glycine amidinotransf	229	266	247.5
4701	NDUFA7	NADH dehydrogenase (ubiquinone) 1 alpha subcomplex, 7,	173	322	247.5
51714	SELT	selenoprotein T	246	246	246
9516	LITAF	lipopolysaccharide-induced TNF factor	426	65	245.5
334	APLP2	amyloid beta (A4) precursor-like protein 2	274	217	245.5
7184	HSP90B1	heat shock protein 90kDa beta (Grp94), member 1	284	206	245
65991	FUNDC2	FUN14 domain containing 2	258	232	245
3309	HSPA5	heat shock 70kDa protein 5 (glucose-regulated protein, 78kD	424	62	243
7307	U2AF1	U2 small nuclear RNA auxiliary factor 1	201	285	243
1504	CTRB1	chymotrypsinogen B1	383	102	242.5
11316	COPE	coatomer protein complex, subunit epsilon	260	225	242.5
217	ALDH2	aldehyde dehydrogenase 2 family (mitochondrial)	144	341	242.5
2647	BLOC1S1	biogenesis of lysosome-related organelles complex-1, subur	129	356	242.5
3460	IFNGR2	interferon gamma receptor 2 (interferon gamma transducer 1	234	250	242
388564	LOC388564	hypothetical gene supported by BC052596	77	407	242
10575	CCT4	chaperonin containing TCP1, subunit 4 (delta)	175	308	241.5
192286	HIGD2A	HIG1 domain family, member 2A	337	145	241
64332	NFKBIZ	nuclear factor of kappa light polypeptide gene enhancer in B	334	147	240.5
5054	SERPINE1	serpin peptidase inhibitor, clade E (nexin, plasminogen activ	163	318	240.5
7111	TMOD1	tropomodulin 1	130	351	240.5
6203	RPS9	ribosomal protein S9	343	137	240
8140	SLC7A5	solute carrier family 7 (cationic amino acid transporter, y+ sy	215	265	240
57111	RAB25	RAB25, member RAS oncogene family	153	327	240
11047	ADRM1	adhesion regulating molecule 1	286	193	239.5
116985	CENTD2	centaurin, delta 2	162	317	239.5
5214	PFKP	phosphofructokinase, platelet	61	418	239.5
5713	PSMD7	proteasome (prosome, macropain) 26S subunit, non-ATPase	195	283	239
55845	C3orf10	chromosome 3 open reading frame 10	272	204	238
57134	MAN1C1	mannosidase, alpha, class 1C, member 1	127	349	238
7916	BAT2	HLA-B associated transcript 2	311	164	237.5
475	ATOX1	ATX1 antioxidant protein 1 homolog (yeast)	282	193	237.5
6774	STAT3	signal transducer and activator of transcription 3 (acute-phas	258	215	236.5
4071	TM4SF1	transmembrane 4 L six family member 1	255	218	236.5
55858	TMEM165	transmembrane protein 165	235	238	236.5
7812	CSDE1	cold shock domain containing E1, RNA-binding	225	247	236
572	BAD	BCL2-antagonist of cell death	177	295	236
9167	COX7A2L	cytochrome c oxidase subunit VIIa polypeptide 2 like	165	307	236
6678	SPARC	secreted protein, acidic, cysteine-rich (osteonectin)	317	154	235.5
4722	NDUFS3	NADH dehydrogenase (ubiquinone) Fe-S protein 3, 30kDa (I	183	288	235.5
6522	SLC4A2	solute carrier family 4, anion exchanger, member 2 (erythroc	205	264	234.5

51107	APH1A	anterior pharynx defective 1 homolog A (C. elegans)	273	195	234
23555	TSPAN15	tetraspanin 15	205	263	234
7262	PHLDA2	pleckstrin homology-like domain, family A, member 2	266	201	233.5
10923	SUB1	SUB1 homolog (S. cerevisiae)	209	258	233.5
2030	SLC29A1	solute carrier family 29 (nucleoside transporters), member 1	197	270	233.5
10847	SRCAP	Snf2-related CBP activator protein	172	295	233.5
4191	MDH2	malate dehydrogenase 2, NAD (mitochondrial)	104	363	233.5
50624	CUZD1	CUB and zona pellucida-like domains 1	393	73	233
633	BGN	biglycan	289	175	232
1717	DHCR7	7-dehydrocholesterol reductase	264	200	232
153830	FLJ31951	hypothetical protein FLJ31951	173	290	231.5
8824	CES2	carboxylesterase 2 (intestine, liver)	262	200	231
6829	SUPT5H	suppressor of Ty 5 homolog (S. cerevisiae)	118	344	231
10342	TFG	TRK-fused gene	180	281	230.5
55611	OTUB1	OTU domain, ubiquitin aldehyde binding 1	161	300	230.5
9274	BCL7C	B-cell CLL/lymphoma 7C	173	287	230
55201	MAP1S	microtubule-associated protein 1S	287	170	228.5
1891	ECH1	enoyl Coenzyme A hydratase 1, peroxisomal	261	196	228.5
55052	MRPL20	mitochondrial ribosomal protein L20	202	255	228.5
358	AQP1	aquaporin 1 (Colton blood group)	254	201	227.5
5702	PSMC3	proteasome (prosome, macropain) 26S subunit, ATPase, 3	234	220	227
8649	MAP2K1IP1	mitogen-activated protein kinase kinase 1 interacting protein	266	186	226
51673	CGI-38	brain specific protein	55	395	225
9555	H2AFY	H2A histone family, member Y	103	346	224.5
8793	TNFRSF10D	tumor necrosis factor receptor superfamily, member 10d, de	0	447	223.5
2029	ENSA	endosulfine alpha	211	235	223
8733	GPAA1	glycosylphosphatidylinositol anchor attachment protein 1 hor	173	273	223
132299	OCIAD2	OCIA domain containing 2	163	283	223
5126	PCSK2	proprotein convertase subtilisin/kexin type 2	89	355	222
8894	EIF2S2	eukaryotic translation initiation factor 2, subunit 2 beta, 38kD	235	208	221.5
51070	NOSIP	nitric oxide synthase interacting protein	169	273	221
55593	OTUD5	OTU domain containing 5	111	331	221
23392	KIAA0368	KIAA0368	116	325	220.5
4885	NPTX2	neuronal pentraxin II	441	0	220.5
57515	SERINC1	serine incorporator 1	248	192	220
10456	HAX1	HCLS1 associated protein X-1	146	294	220
1500	CTNND1	catenin (cadherin-associated protein), delta 1	186	252	219
1198	CLK3	CDC-like kinase 3	298	139	218.5
64374	SIL1	SIL1 homolog, endoplasmic reticulum chaperone (S. cerevis	238	199	218.5
6605	SMARCE1	SWI/SNF related, matrix associated, actin dependent regula	221	216	218.5
11014	KDELR2	KDEL (Lys-Asp-Glu-Leu) endoplasmic reticulum protein rete	209	227	218
51329	ARL6IP4	ADP-ribosylation-like factor 6 interacting protein 4	342	93	217.5
8106	PABPN1	poly(A) binding protein, nuclear 1	189	245	217
3615	IMPDH2	IMP (inosine monophosphate) dehydrogenase 2	320	113	216.5
23204	ARL6IP1	ADP-ribosylation factor-like 6 interacting protein 1	215	218	216.5
103	ADAR	adenosine deaminase, RNA-specific	209	224	216.5
1365	CLDN3	claudin 3	177	256	216.5
4723	NDUFV1	NADH dehydrogenase (ubiquinone) flavoprotein 1, 51kDa	242	190	216
9249	DHRS3	dehydrogenase/reductase (SDR family) member 3	209	222	215.5
3801	KIFC3	kinesin family member C3	162	269	215.5
140823	C20orf52	chromosome 20 open reading frame 52	0	431	215.5

8667	EIF3S3	eukaryotic translation initiation factor 3, subunit 3 gamma, 40	146	283	214.5
64112	MOAP1	modulator of apoptosis 1	73	356	214.5
54097	FAM3B	family with sequence similarity 3, member B	97	331	214
4328	NA	NA	426	0	213
821	CANX	calnexin	192	233	212.5
4245	MGAT1	mannosyl (alpha-1,3-)-glycoprotein beta-1,2-N-acetylglucosa	109	316	212.5
51693	TRAPPC2L	trafficking protein particle complex 2-like	310	113	211.5
8531	CSDA	cold shock domain protein A	363	59	211
5725	PTBP1	polypyrimidine tract binding protein 1	207	215	211
55700	RPRC1	arginine/proline rich coiled-coil 1	156	266	211
25915	C3orf60	chromosome 3 open reading frame 60	200	220	210
6597	SMARCA4	SWI/SNF related, matrix associated, actin dependent regula	152	268	210
6389	SDHA	succinate dehydrogenase complex, subunit A, flavoprotein (F	163	255	209
373156	GSTK1	glutathione S-transferase kappa 1	242	175	208.5
391	RHOG	ras homolog gene family, member G (rho G)	198	218	208
4710	NDUFB4	NADH dehydrogenase (ubiquinone) 1 beta subcomplex, 4, 1	186	230	208
347734	SLC35B2	solute carrier family 35, member B2	268	147	207.5
3134	HLA-F	major histocompatibility complex, class I, F	68	346	207
25804	LSM4	LSM4 homolog, U6 small nuclear RNA associated (S. cerevi	278	135	206.5
7531	YWHAE	tyrosine 3-monooxygenase/tryptophan 5-monooxygenase ac	183	230	206.5
51112	TTC15	tetratricopeptide repeat domain 15	258	154	206
51341	ZBTB7A	zinc finger and BTB domain containing 7A	240	172	206
84525	HOP	homeodomain-only protein	261	150	205.5
54460	MRPS21	mitochondrial ribosomal protein S21	179	231	205
1535	CYBA	cytochrome b-245, alpha polypeptide	43	367	205
55905	ZNF313	zinc finger protein 313	68	340	204
3281	HSBP1	heat shock factor binding protein 1	319	88	203.5
4735	SEP2	septin 2	213	194	203.5
80223	RAB11FIP1	RAB11 family interacting protein 1 (class I)	140	267	203.5
10755	GIPC1	GIPC PDZ domain containing family, member 1	102	305	203.5
9266	PSCD2	pleckstrin homology, Sec7 and coiled-coil domains 2 (cytohe	44	362	203
3654	IRAK1	interleukin-1 receptor-associated kinase 1	183	222	202.5
2026	ENO2	enolase 2 (gamma, neuronal)	88	317	202.5
22877	MLXIP	MLX interacting protein	261	143	202
137964	AGPAT6	1-acylglycerol-3-phosphate O-acyltransferase 6 (lysophosph	221	183	202
2063	NR2F6	nuclear receptor subfamily 2, group F, member 6	204	199	201.5
3123	HLA-DRB1	major histocompatibility complex, class II, DR beta 1	181	222	201.5
1487	CTBP1	C-terminal binding protein 1	158	245	201.5
170622	COMMD6	COMM domain containing 6	127	276	201.5
29101	SSU72	SSU72 RNA polymerase II CTD phosphatase homolog (S. c	230	172	201
5184	PEPD	peptidase D	127	275	201
85437	ZCRB1	zinc finger CCHC-type and RNA binding motif 1	213	188	200.5
56943	ENY2	enhancer of yellow 2 homolog (Drosophila)	200	201	200.5
3638	INSIG1	insulin induced gene 1	165	235	200
1942	EFNA1	ephrin-A1	117	283	200
3976	LIF	leukemia inhibitory factor (cholinergic differentiation factor)	75	325	200
5159	PDGFRB	platelet-derived growth factor receptor, beta polypeptide	292	106	199
10548	TM9SF1	transmembrane 9 superfamily member 1	91	307	199
84313	VPS25	vacuolar protein sorting 25 homolog (S. cerevisiae)	227	170	198.5
7791	ZYX	zyxin	113	284	198.5
10498	CARM1	coactivator-associated arginine methyltransferase 1	291	104	197.5

5699	PSMB10	proteasome (prosome, macropain) subunit, beta type, 10	140	254	197
51606	ATP6V1H	ATPase, H+ transporting, lysosomal 50/57kDa, V1 subunit H	339	54	196.5
5531	PPP4C	protein phosphatase 4 (formerly X), catalytic subunit	268	124	196
6717	SRI	sorcin	232	160	196
6809	STX3	syntaxin 3	201	191	196
9416	DDX23	DEAD (Asp-Glu-Ala-Asp) box polypeptide 23	170	222	196
10245	TIMM17B	translocase of inner mitochondrial membrane 17 homolog B	152	240	196
664	BNIP3	BCL2/adenovirus E1B 19kDa interacting protein 3	100	292	196
10079	ATP9A	ATPase, Class II, type 9A	150	241	195.5
9217	VAPB	VAMP (vesicle-associated membrane protein)-associated pr	69	322	195.5
6372	CXCL6	chemokine (C-X-C motif) ligand 6 (granulocyte chemotactic p	341	49	195
5063	PAK3	p21 (CDKN1A)-activated kinase 3	239	151	195
8566	PDXK	pyridoxal (pyridoxine, vitamin B6) kinase	248	141	194.5
22876	INPP5F	inositol polyphosphate-5-phosphatase F	164	225	194.5
26003	GORASP2	golgi reassembly stacking protein 2, 55kDa	253	135	194
8938	BAIAP3	BAI1-associated protein 3	164	224	194
22937	SCAP	SREBP cleavage-activating protein	156	232	194
60684	FLJ12716	FLJ12716 protein	128	260	194
4501	MT1X	metallothionein 1X	88	300	194
11331	PHB2	prohibitin 2	251	136	193.5
163	AP2B1	adaptor-related protein complex 2, beta 1 subunit	198	189	193.5
1718	DHCR24	24-dehydrocholesterol reductase	181	206	193.5
5496	PPM1G	protein phosphatase 1G (formerly 2C), magnesium-depende	150	237	193.5
3020	H3F3A	H3 histone, family 3A	132	255	193.5
5860	QDPR	quinoid dihydropteridine reductase	187	199	193
84681	HINT2	histidine triad nucleotide binding protein 2	284	101	192.5
9578	CDC42BPB	CDC42 binding protein kinase beta (DMPK-like)	159	226	192.5
80755	AARSD1	alanyl-tRNA synthetase domain containing 1	0	385	192.5
26232	FBXO2	F-box protein 2	108	276	192
4925	NUCB2	nucleobindin 2	308	75	191.5
10654	PMVK	phosphomevalonate kinase	173	210	191.5
515	ATP5F1	ATP synthase, H+ transporting, mitochondrial F0 complex, s	163	220	191.5
4728	NDUFS8	NADH dehydrogenase (ubiquinone) Fe-S protein 8, 23kDa (I	146	237	191.5
5976	UPF1	UPF1 regulator of nonsense transcripts homolog (yeast)	229	153	191
9188	DDX21	DEAD (Asp-Glu-Ala-Asp) box polypeptide 21	126	256	191
7089	TLE2	transducin-like enhancer of split 2 (E(sp1) homolog, Drosoph	107	275	191
127544	IBRDC3	IBR domain containing 3	201	180	190.5
6520	SLC3A2	solute carrier family 3 (activators of dibasic and neutral amin	378	2	190
84335	AKT1S1	AKT1 substrate 1 (proline-rich)	223	157	190
92949	ADAMTSL1	ADAMTS-like 1	280	99	189.5
57655	GRAMD1A	GRAM domain containing 1A	225	151	188
10272	FSTL3	follistatin-like 3 (secreted glycoprotein)	203	173	188
51293	CD320	CD320 molecule	174	202	188
10313	RTN3	reticulon 3	173	203	188
153572	IRX2	iroquois homeobox protein 2	261	114	187.5
10589	DRAP1	DR1-associated protein 1 (negative cofactor 2 alpha)	185	189	187
116372	LYPD1	LY6/PLAUR domain containing 1	162	212	187
6182	MRPL12	mitochondrial ribosomal protein L12	251	122	186.5
10480	PCID1	PCI domain containing 1 (herpesvirus entry mediator)	200	173	186.5
10574	CCT7	chaperonin containing TCP1, subunit 7 (eta)	177	196	186.5
1982	EIF4G2	eukaryotic translation initiation factor 4 gamma, 2	0	373	186.5

51433	ANAPC5	anaphase promoting complex subunit 5	195	176	185.5
3716	JAK1	Janus kinase 1 (a protein tyrosine kinase)	185	186	185.5
115353	LRRC42	leucine rich repeat containing 42	53	318	185.5
10652	YKT6	YKT6 v-SNARE homolog (S. cerevisiae)	241	129	185
4628	MYH10	myosin, heavy chain 10, non-muscle	166	202	184
8558	CDK10	cyclin-dependent kinase (CDC2-like) 10	142	224	183
6337	SCNN1A	sodium channel, nonvoltage-gated 1 alpha	190	175	182.5
4716	NDUFB10	NADH dehydrogenase (ubiquinone) 1 beta subcomplex, 10,	185	180	182.5
284106	LOC284106	hypothetical protein LOC284106	155	210	182.5
6421	SFPQ	splicing factor proline/glutamine-rich (polypyrimidine tract bin	168	196	182
113419	TEX261	testis expressed sequence 261	104	260	182
207	AKT1	v-akt murine thymoma viral oncogene homolog 1	82	282	182
1173	AP2M1	adaptor-related protein complex 2, mu 1 subunit	311	52	181.5
224	ALDH3A2	aldehyde dehydrogenase 3 family, member A2	221	142	181.5
9935	MAFB	v-maf musculoaponeurotic fibrosarcoma oncogene homolog	180	183	181.5
64979	MRPL36	mitochondrial ribosomal protein L36	138	225	181.5
4792	NFKBIA	nuclear factor of kappa light polypeptide gene enhancer in B	266	96	181
2885	GRB2	growth factor receptor-bound protein 2	174	188	181
23108	GARNL4	GTPase activating Rap/RanGAP domain-like 4	160	202	181
908	CCT6A	chaperonin containing TCP1, subunit 6A (zeta 1)	152	210	181
6878	TAF6	TAF6 RNA polymerase II, TATA box binding protein (TBP)-a	138	222	180
26090	ABHD12	abhydrolase domain containing 12	166	193	179.5
55577	NAGK	N-acetylglucosamine kinase	213	145	179
80781	COL18A1	collagen, type XVIII, alpha 1	193	165	179
23385	NCSTN	nicastrin	186	172	179
8904	CPNE1	copine I	140	218	179
6813	STXBP2	syntaxin binding protein 2	136	222	179
51602	NOP5/NOP5	nucleolar protein NOP5/NOP58	130	228	179
10095	ARPC1B	actin related protein 2/3 complex, subunit 1B, 41kDa	274	83	178.5
563	AZGP1	alpha-2-glycoprotein 1, zinc-binding	124	233	178.5
5300	PIN1	protein (peptidylprolyl cis/trans isomerase) NIMA-interacting	163	193	178
51186	WBP5	WW domain binding protein 5	92	263	177.5
10248	POP7	processing of precursor 7, ribonuclease P subunit (S. cerevis	170	184	177
5438	POLR2I	polymerase (RNA) II (DNA directed) polypeptide I, 14.5kDa	100	254	177
65993	MRPS34	mitochondrial ribosomal protein S34	211	142	176.5
133619	PRRC1	proline-rich coiled-coil 1	175	178	176.5
10771	ZMYND11	zinc finger, MYND domain containing 11	102	251	176.5
310	ANXA7	annexin A7	175	177	176
9130	FAM50A	family with sequence similarity 50, member A	171	181	176
192683	SCAMP5	secretory carrier membrane protein 5	169	183	176
8761	PABPC4	poly(A) binding protein, cytoplasmic 4 (inducible form)	249	102	175.5
57085	AGTRAP	angiotensin II receptor-associated protein	108	243	175.5
84530	KIAA1853	KIAA1853	0	351	175.5
115098	CCDC124	coiled-coil domain containing 124	188	161	174.5
51646	YPEL5	yippee-like 5 (Drosophila)	89	260	174.5
5524	PPP2R4	protein phosphatase 2A, regulatory subunit B' (PR 53)	120	228	174
9361	LONP1	lon peptidase 1, mitochondrial	97	251	174
64743	WDR13	WD repeat domain 13	132	215	173.5
1808	DPYSL2	dihydropyrimidinase-like 2	131	216	173.5
6192	RPS4Y1	ribosomal protein S4, Y-linked 1	0	347	173.5
28958	CCDC56	coiled-coil domain containing 56	294	52	173

54867	FLJ20254	hypothetical protein FLJ20254	144	202	173
81552	ECOP	EGFR-coamplified and overexpressed protein	140	206	173
3983	ABLIM1	actin binding LIM protein 1	134	212	173
811	CALR	calreticulin	328	17	172.5
124565	MGC15523	hypothetical protein MGC15523	134	211	172.5
27044	SND1	staphylococcal nuclease and tudor domain containing 1	127	217	172
3628	INPP1	inositol polyphosphate-1-phosphatase	221	122	171.5
7538	ZFP36	zinc finger protein 36, C3H type, homolog (mouse)	86	257	171.5
22985	ACIN1	apoptotic chromatin condensation inducer 1	227	115	171
11253	MAN1B1	mannosidase, alpha, class 1B, member 1	205	137	171
90993	CREB3L1	cAMP responsive element binding protein 3-like 1	176	166	171
4781	NFIB	nuclear factor I/B	132	210	171
1116	CHI3L1	chitinase 3-like 1 (cartilage glycoprotein-39)	12	330	171
55608	ANKRD10	ankyrin repeat domain 10	166	175	170.5
6647	SOD1	superoxide dismutase 1, soluble (amyotrophic lateral scleros	196	144	170
118487	CHCHD1	coiled-coil-helix-coiled-coil-helix domain containing 1	184	156	170
775	CACNA1C	calcium channel, voltage-dependent, L type, alpha 1C subur	130	210	170
2782	GNB1	guanine nucleotide binding protein (G protein), beta polypep	117	223	170
3694	ITGB6	integrin, beta 6	90	250	170
219402	MTIF3	mitochondrial translational initiation factor 3	77	263	170
5906	RAP1A	RAP1A, member of RAS oncogene family	231	108	169.5
8237	USP11	ubiquitin specific peptidase 11	79	260	169.5
8565	YARS	tyrosyl-tRNA synthetase	132	206	169
23277	KIAA0664	KIAA0664	221	116	168.5
3735	KARS	lysyl-tRNA synthetase	180	155	167.5
56997	CABC1	chaperone, ABC1 activity of bc1 complex homolog (S. pomb	274	60	167
4190	MDH1	malate dehydrogenase 1, NAD (soluble)	178	156	167
3028	HSD17B10	hydroxysteroid (17-beta) dehydrogenase 10	161	173	167
128866	CHMP4B	chromatin modifying protein 4B	81	253	167
10994	ILVBL	ilvB (bacterial acetolactate synthase)-like	138	195	166.5
745	C11orf9	chromosome 11 open reading frame 9	109	224	166.5
9094	UNC119	unc-119 homolog (C. elegans)	221	111	166
6364	CCL20	chemokine (C-C motif) ligand 20	201	131	166
9148	NEURL	neuralized homolog (Drosophila)	152	180	166
1725	DHPS	deoxyhypusine synthase	128	204	166
64215	DNAJC1	DnaJ (Hsp40) homolog, subfamily C, member 1	118	214	166
388344	LOC388344	similar to ribosomal protein L13	203	128	165.5
51495	PTPLAD1	protein tyrosine phosphatase-like A domain containing 1	191	140	165.5
89891	WDR34	WD repeat domain 34	132	199	165.5
11325	DDX42	DEAD (Asp-Glu-Ala-Asp) box polypeptide 42	85	246	165.5
9414	TJP2	tight junction protein 2 (zona occludens 2)	26	305	165.5
7503	XIST	X (inactive)-specific transcript	331	0	165.5
7037	TFRC	transferrin receptor (p90, CD71)	150	180	165
3880	KRT19	keratin 19	123	207	165
11214	AKAP13	A kinase (PRKA) anchor protein 13	63	267	165
221035	REEP3	receptor accessory protein 3	61	269	165
5886	RAD23A	RAD23 homolog A (S. cerevisiae)	45	285	165
7841	GCS1	glucosidase I	207	122	164.5
2011	MARK2	MAP/microtubule affinity-regulating kinase 2	166	163	164.5
10616	RBCK1	RanBP-type and C3HC4-type zinc finger containing 1	124	205	164.5
9230	RAB11B	RAB11B, member RAS oncogene family	178	150	164

222068	TMED4	transmembrane emp24 protein transport domain containing	169	159	164
6015	RING1	ring finger protein 1	148	180	164
80145	THOC7	THO complex 7 homolog (Drosophila)	195	132	163.5
374395	LOC374395	similar to RIKEN cDNA 1810059G22	238	88	163
9790	BMS1L	BMS1-like, ribosome assembly protein (yeast)	138	188	163
9425	CDYL	chromodomain protein, Y-like	317	8	162.5
58472	SQRDL	sulfide quinone reductase-like (yeast)	156	169	162.5
51075	TXNDC14	thioredoxin domain containing 14	150	175	162.5
5684	PSMA3	proteasome (prosome, macropain) subunit, alpha type, 3	135	189	162
7879	RAB7	RAB7, member RAS oncogene family	132	192	162
3927	LASP1	LIM and SH3 protein 1	131	193	162
153571	CEI	coordinated expression to IRXA2 homeobox gene	112	212	162
94103	ORMDL3	ORM1-like 3 (S. cerevisiae)	112	212	162
57805	KIAA1967	KIAA1967	223	100	161.5
3685	ITGAV	integrin, alpha V (vitronectin receptor, alpha polypeptide, ant	150	173	161.5
2039	EPB49	erythrocyte membrane protein band 4.9 (dematin)	102	221	161.5
9324	HMGN3	high mobility group nucleosomal binding domain 3	188	134	161
22921	MSRB2	methionine sulfoxide reductase B2	173	149	161
83988	NCALD	neurocalcin delta	175	145	160
54502	FLJ20273	RNA-binding protein	149	171	160
4121	MAN1A1	mannosidase, alpha, class 1A, member 1	85	235	160
6777	STAT5B	signal transducer and activator of transcription 5B	78	242	160
27338	UBE2S	ubiquitin-conjugating enzyme E2S	303	16	159.5
5955	RCN2	reticulocalbin 2, EF-hand calcium binding domain	215	104	159.5
6294	SAFB	scaffold attachment factor B	211	108	159.5
5434	POLR2E	polymerase (RNA) II (DNA directed) polypeptide E, 25kDa	201	118	159.5
54840	APTX	aprataxin	158	161	159.5
5408	PNLIPRP2	pancreatic lipase-related protein 2	130	189	159.5
90427	BMF	Bcl2 modifying factor	209	109	159
8804	CREG1	cellular repressor of E1A-stimulated genes 1	207	111	159
57104	PNPLA2	patatin-like phospholipase domain containing 2	163	155	159
3420	IDH3B	isocitrate dehydrogenase 3 (NAD+) beta	145	173	159
7032	TFF2	trefoil factor 2 (spasmolytic protein 1)	0	318	159
26528	DAZAP1	DAZ associated protein 1	273	43	158
58190	CTDSP1	CTD (carboxy-terminal domain, RNA polymerase II, polypep	116	200	158
51337	C8orf55	chromosome 8 open reading frame 55	97	219	158
2319	FLOT2	flotillin 2	187	128	157.5
23645	PPP1R15A	protein phosphatase 1, regulatory (inhibitor) subunit 15A	157	158	157.5
79004	CUEDC2	CUE domain containing 2	140	175	157.5
58505	DC2	DC2 protein	89	226	157.5
7466	WFS1	Wolfram syndrome 1 (wolframin)	240	74	157
5436	POLR2G	polymerase (RNA) II (DNA directed) polypeptide G	112	202	157
57153	SLC44A2	solute carrier family 44, member 2	71	243	157
57804	POLD4	polymerase (DNA-directed), delta 4	120	193	156.5
26118	WSB1	WD repeat and SOCS box-containing 1	110	203	156.5
7528	YY1	YY1 transcription factor	73	240	156.5
51078	THAP4	THAP domain containing 4	150	162	156
8525	DGKZ	diacylglycerol kinase, zeta 104kDa	114	198	156
3430	IFI35	interferon-induced protein 35	97	215	156
79143	LENG4	leukocyte receptor cluster (LRC) member 4	70	242	156
4809	NHP2L1	NHP2 non-histone chromosome protein 2-like 1 (S. cerevisia	195	116	155.5

2990	GUSB	glucuronidase, beta	189	122	155.5
4256	MGP	matrix Gla protein	118	193	155.5
23164	M-RIP	myosin phosphatase-Rho interacting protein	118	193	155.5
30000	TNPO2	transportin 2 (importin 3, karyopherin beta 2b)	104	207	155.5
9725	TMEM63A	transmembrane protein 63A	79	232	155.5
64114	TMBIM1	transmembrane BAX inhibitor motif containing 1	73	238	155.5
8668	EIF3S2	eukaryotic translation initiation factor 3, subunit 2 beta, 36kD	307	3	155
80790	CMIP	c-Maf-inducing protein	223	87	155
124446	LOC124446	hypothetical protein BC017488	178	132	155
871	SERPINH1	serpin peptidase inhibitor, clade H (heat shock protein 47), m	129	181	155
4711	NDUFB5	NADH dehydrogenase (ubiquinone) 1 beta subcomplex, 5, 1	116	194	155
2799	GNS	glucosamine (N-acetyl)-6-sulfatase (Sanfilippo disease IIID)	199	110	154.5
715	C1R	complement component 1, r subcomponent	233	75	154
5571	PRKAG1	protein kinase, AMP-activated, gamma 1 non-catalytic subur	199	109	154
928	CD9	CD9 molecule	190	118	154
26010	DNAPTP6	DNA polymerase-transactivated protein 6	138	170	154
8289	ARID1A	AT rich interactive domain 1A (SWI-like)	107	201	154
826	CAPNS1	calpain, small subunit 1	100	208	154
28978	TMEM14A	transmembrane protein 14A	53	255	154
26000	TBC1D10B	TBC1 domain family, member 10B	160	147	153.5
136319	MTPN	myotrophin	115	192	153.5
144348	ZNF664	zinc finger protein 664	75	232	153.5
90459	THEX1	three prime histone mRNA exonuclease 1	174	132	153
55690	PACS1	phosphofurin acidic cluster sorting protein 1	138	168	153
22992	FBXL11	F-box and leucine-rich repeat protein 11	102	204	153
51652	VPS24	vacuolar protein sorting 24 homolog (S. cerevisiae)	228	77	152.5
7358	UGDH	UDP-glucose dehydrogenase	142	163	152.5
4324	MMP15	matrix metallopeptidase 15 (membrane-inserted)	98	207	152.5
9213	XPR1	xenotropic and polytropic retrovirus receptor	93	211	152
126208	ZNF787	zinc finger protein 787	261	42	151.5
5999	RGS4	regulator of G-protein signalling 4	113	190	151.5
694	BTG1	B-cell translocation gene 1, anti-proliferative	204	98	151
79039	DDX54	DEAD (Asp-Glu-Ala-Asp) box polypeptide 54	158	144	151
2109	ETFB	electron-transfer-flavoprotein, beta polypeptide	115	187	151
6252	RTN1	reticulon 1	138	163	150.5
3550	IK	IK cytokine, down-regulator of HLA II	211	89	150
667	DST	dystonin	130	169	149.5
55832	CAND1	cullin-associated and neddylation-dissociated 1	121	178	149.5
3597	IL13RA1	interleukin 13 receptor, alpha 1	107	192	149.5
22861	NLRP1	NLR family, pyrin domain containing 1	184	114	149
10317	B3GALT5	UDP-Gal:betaGlcNAc beta 1,3-galactosyltransferase, polype	148	150	149
50632	DRD1IP	dopamine receptor D1 interacting protein	58	240	149
8694	DGAT1	diacylglycerol O-acyltransferase homolog 1 (mouse)	48	250	149
29803	REPIN1	replication initiator 1	232	65	148.5
56288	PARD3	par-3 partitioning defective 3 homolog (C. elegans)	188	109	148.5
8666	EIF3S4	eukaryotic translation initiation factor 3, subunit 4 delta, 44kD	165	132	148.5
3609	ILF3	interleukin enhancer binding factor 3, 90kDa	126	171	148.5
85014	TMEM141	transmembrane protein 141	104	193	148.5
1410	CRYAB	crystallin, alpha B	96	201	148.5
7033	TFF3	trefoil factor 3 (intestinal)	83	214	148.5
64951	MRPS24	mitochondrial ribosomal protein S24	186	110	148

9698	PUM1	pumilio homolog 1 (Drosophila)	177	119	148
89927	C16orf45	chromosome 16 open reading frame 45	162	134	148
3993	LLGL2	lethal giant larvae homolog 2 (Drosophila)	87	209	148
1755	DMBT1	deleted in malignant brain tumors 1	36	260	148
10450	PPIE	peptidylprolyl isomerase E (cyclophilin E)	166	129	147.5
14	AAMP	angio-associated, migratory cell protein	119	176	147.5
3170	FOXA2	forkhead box A2	63	232	147.5
9780	FAM38A	family with sequence similarity 38, member A	29	266	147.5
2266	FGG	fibrinogen gamma chain	173	121	147
1843	DUSP1	dual specificity phosphatase 1	114	180	147
64771	C6orf106	chromosome 6 open reading frame 106	103	191	147
1351	COX8A	cytochrome c oxidase subunit 8A (ubiquitous)	28	266	147
369	ARAF	v-raf murine sarcoma 3611 viral oncogene homolog	211	82	146.5
29058	C20orf30	chromosome 20 open reading frame 30	179	114	146.5
2521	FUS	fusion (involved in t(12;16) in malignant liposarcoma)	171	122	146.5
196740	C10orf72	chromosome 10 open reading frame 72	112	181	146.5
3249	HPN	hepsin (transmembrane protease, serine 1)	207	85	146
7392	USF2	upstream transcription factor 2, c-fos interacting	123	169	146
390502	SERPINA2	serpin peptidase inhibitor, clade A (alpha-1 antiproteinase, a	67	225	146
11345	GABARAPL2	GABA(A) receptor-associated protein-like 2	134	157	145.5
9114	ATP6V0D1	ATPase, H+ transporting, lysosomal 38kDa, V0 subunit d1	92	199	145.5
3421	IDH3G	isocitrate dehydrogenase 3 (NAD+) gamma	58	233	145.5
25930	PTPN23	protein tyrosine phosphatase, non-receptor type 23	18	273	145.5
29926	GMPPA	GDP-mannose pyrophosphorylase A	221	69	145
90864	SPSB3	splA/ryanodine receptor domain and SOCS box containing 3	217	73	145
4784	NFIX	nuclear factor I/X (CCAAT-binding transcription factor)	168	122	145
28962	OSTM1	osteopetrosis associated transmembrane protein 1	183	106	144.5
10111	RAD50	RAD50 homolog (S. cerevisiae)	162	127	144.5
6184	RPN1	ribophorin I	147	142	144.5
22906	TRAK1	trafficking protein, kinesin binding 1	149	139	144
6579	SLCO1A2	solute carrier organic anion transporter family, member 1A2	267	20	143.5
5905	RANGAP1	Ran GTPase activating protein 1	131	156	143.5
8263	F8A1	coagulation factor VIII-associated (intronic transcript) 1	91	196	143.5
51699	VPS29	vacuolar protein sorting 29 homolog (S. cerevisiae)	106	180	143
29086	HSPC142	HSPC142 protein	102	184	143
5226	PGD	phosphogluconate dehydrogenase	123	162	142.5
4087	SMAD2	SMAD family member 2	237	47	142
274	BIN1	bridging integrator 1	181	103	142
7035	TFPI	tissue factor pathway inhibitor (lipoprotein-associated coagul	159	125	142
9261	MAPKAPK2	mitogen-activated protein kinase-activated protein kinase 2	96	187	141.5
9448	MAP4K4	mitogen-activated protein kinase kinase kinase kinase 4	64	219	141.5
9066	SYT7	synaptotagmin VII	89	193	141
196515	FLJ30092	AF-1 specific protein phosphatase	8	274	141
6431	SFRS6	splicing factor, arginine/serine-rich 6	159	122	140.5
10553	HTATIP2	HIV-1 Tat interactive protein 2, 30kDa	157	124	140.5
2017	CTTN	cortactin	134	147	140.5
203	AK1	adenylate kinase 1	123	158	140.5
3300	DNAJB2	DnaJ (Hsp40) homolog, subfamily B, member 2	45	236	140.5
26073	POLDIP2	polymerase (DNA-directed), delta interacting protein 2	223	57	140
51042	ZNF593	zinc finger protein 593	223	57	140
6510	SLC1A5	solute carrier family 1 (neutral amino acid transporter), meml	171	109	140

10312	TCIRG1	T-cell, immune regulator 1, ATPase, H+ transporting, lysosoı	166	114	140
5018	OXA1L	oxidase (cytochrome c) assembly 1-like	154	126	140
3182	HNRPAB	heterogeneous nuclear ribonucleoprotein A/B	138	142	140
92856	IMP4	IMP4, U3 small nucleolar ribonucleoprotein, homolog (yeast)	118	162	140
51599	LSR	lipolysis stimulated lipoprotein receptor	53	227	140
11100	HNRPUL1	heterogeneous nuclear ribonucleoprotein U-like 1	180	99	139.5
79735	TBC1D17	TBC1 domain family, member 17	120	158	139
3005	H1F0	H1 histone family, member 0	109	169	139
54915	YTHDF1	YTH domain family, member 1	48	230	139
55585	UBE2Q1	ubiquitin-conjugating enzyme E2Q (putative) 1	146	131	138.5
27101	CACYBP	calcyclin binding protein	121	156	138.5
84269	CHCHD5	coiled-coil-helix-coiled-coil-helix domain containing 5	106	171	138.5
8878	SQSTM1	sequestosome 1	258	18	138
54472	TOLLIP	toll interacting protein	207	69	138
126321	C19orf28	chromosome 19 open reading frame 28	121	155	138
140465	MYL6B	myosin, light chain 6B, alkali, smooth muscle and non-muscl	69	207	138
23648	SSBP3	single stranded DNA binding protein 3	130	145	137.5
55748	CNDP2	CNDP dipeptidase 2 (metallopeptidase M20 family)	130	145	137.5
112770	C1orf85	chromosome 1 open reading frame 85	104	171	137.5
10611	PDLIM5	PDZ and LIM domain 5	96	179	137.5
140738	TMEM37	transmembrane protein 37	79	196	137.5
51510	CHMP5	chromatin modifying protein 5	142	132	137
8991	SELENBP1	selenium binding protein 1	121	153	137
123096	SLC25A29	solute carrier family 25, member 29	103	171	137
51368	TEX264	testis expressed sequence 264	57	217	137
51552	RAB14	RAB14, member RAS oncogene family	213	60	136.5
1908	EDN3	endothelin 3	160	113	136.5
7162	TPBG	trophoblast glycoprotein	134	139	136.5
26229	B3GAT3	beta-1,3-glucuronyltransferase 3 (glucuronosyltransferase I)	123	150	136.5
7494	XBP1	X-box binding protein 1	215	57	136
91	ACVR1B	activin A receptor, type IB	148	124	136
1212	CLTB	clathrin, light chain (Lcb)	146	126	136
55851	PSENEN	presenilin enhancer 2 homolog (C. elegans)	125	147	136
27183	VPS4A	vacuolar protein sorting 4 homolog A (S. cerevisiae)	86	186	136
23019	CNOT1	CCR4-NOT transcription complex, subunit 1	81	191	136
51031	C17orf25	chromosome 17 open reading frame 25	65	207	136
441381	LRRC24	leucine rich repeat containing 24	183	88	135.5
79139	DERL1	Der1-like domain family, member 1	178	93	135.5
22949	LTB4DH	leukotriene B4 12-hydroxydehydrogenase	175	96	135.5
54583	EGLN1	egl nine homolog 1 (C. elegans)	119	152	135.5
58986	TMEM8	transmembrane protein 8 (five membrane-spanning domains	116	155	135.5
9584	RBM39	RNA binding motif protein 39	93	177	135
5717	PSMD11	proteasome (prosome, macropain) 26S subunit, non-ATPase	93	177	135
9943	OXSR1	oxidative-stress responsive 1	189	80	134.5
51129	ANGPTL4	angiopoietin-like 4	67	202	134.5
7543	ZFX	zinc finger protein, X-linked	55	214	134.5
8933	FAM127A	family with sequence similarity 127, member A	158	110	134
10482	NXF1	nuclear RNA export factor 1	156	112	134
55749	CCAR1	cell division cycle and apoptosis regulator 1	71	197	134
64755	C16orf58	chromosome 16 open reading frame 58	91	176	133.5
5834	PYGB	phosphorylase, glycogen; brain	87	180	133.5

28996	HIPK2	homeodomain interacting protein kinase 2	71	196	133.5
6035	RNASE1	ribonuclease, RNase A family, 1 (pancreatic)	122	144	133
64960	MRPS15	mitochondrial ribosomal protein S15	187	78	132.5
6344	SCTR	secretin receptor	109	156	132.5
4048	LTA4H	leukotriene A4 hydrolase	98	167	132.5
3398	ID2	inhibitor of DNA binding 2, dominant negative helix-loop-heli	74	191	132.5
6836	SURF4	surfeit 4	200	64	132
23480	SEC61G	Sec61 gamma subunit	148	116	132
2794	GNL1	guanine nucleotide binding protein-like 1	132	132	132
985	CDC2L2	cell division cycle 2-like 2 (PITSLRE proteins)	223	39	131
25996	REXO2	REX2, RNA exonuclease 2 homolog (S. cerevisiae)	215	47	131
10430	TMEM147	transmembrane protein 147	205	57	131
5819	PVRL2	poliovirus receptor-related 2 (herpesvirus entry mediator B)	152	110	131
25828	TXN2	thioredoxin 2	102	160	131
9267	PSCD1	pleckstrin homology, Sec7 and coiled-coil domains 1(cytohe	171	90	130.5
30846	EHD2	EH-domain containing 2	158	103	130.5
54738	FEV	FEV (ETS oncogene family)	134	127	130.5
1831	TSC22D3	TSC22 domain family, member 3	120	141	130.5
283987	C17orf28	chromosome 17 open reading frame 28	160	100	130
10497	UNC13B	unc-13 homolog B (C. elegans)	138	122	130
2077	ERF	Ets2 repressor factor	183	76	129.5
26999	CYFIP2	cytoplasmic FMR1 interacting protein 2	179	80	129.5
840	CASP7	caspase 7, apoptosis-related cysteine peptidase	138	121	129.5
51447	IHPK2	inositol hexaphosphate kinase 2	92	167	129.5
4802	NFYC	nuclear transcription factor Y, gamma	37	222	129.5
28956	MAPBPIP	mitogen-activated protein-binding protein-interacting protein	190	68	129
9601	PDIA4	protein disulfide isomerase family A, member 4	144	114	129
8841	HDAC3	histone deacetylase 3	93	165	129
50485	SMARCAL1	SWI/SNF related, matrix associated, actin dependent regula	48	210	129
84275	SLC25A33	solute carrier family 25, member 33	138	119	128.5
7040	TGFB1	transforming growth factor, beta 1 (Camurati-Engelmann dis	125	132	128.5
9817	KEAP1	kelch-like ECH-associated protein 1	106	151	128.5
6461	SHB	Src homology 2 domain containing adaptor protein B	161	95	128
10695	TNRC5	trinucleotide repeat containing 5	90	166	128
56993	TOMM22	translocase of outer mitochondrial membrane 22 homolog (y	83	173	128
6272	SORT1	sortilin 1	66	190	128
7105	TSPAN6	tetraspanin 6	55	201	128
29979	UBQLN1	ubiquilin 1	45	211	128
57568	SIPA1L2	signal-induced proliferation-associated 1 like 2	194	61	127.5
54832	VPS13C	vacuolar protein sorting 13 homolog C (S. cerevisiae)	144	111	127.5
149466	C1orf210	chromosome 1 open reading frame 210	185	69	127
116541	MRPL54	mitochondrial ribosomal protein L54	129	125	127
3189	HNRPH3	heterogeneous nuclear ribonucleoprotein H3 (2H9)	121	133	127
5217	PFN2	profilin 2	109	145	127
10808	HSPH1	heat shock 105kDa/110kDa protein 1	101	153	127
10447	FAM3C	family with sequence similarity 3, member C	75	179	127
1822	ATN1	atrophin 1	160	93	126.5
10922	FASTK	Fas-activated serine/threonine kinase	140	113	126.5
5499	PPP1CA	protein phosphatase 1, catalytic subunit, alpha isoform	127	126	126.5
51690	LSM7	LSM7 homolog, U6 small nuclear RNA associated (S. cerevi	124	129	126.5
5691	PSMB3	proteasome (prosome, macropain) subunit, beta type, 3	123	130	126.5

55816	DOK5	docking protein 5	0	253	126.5
9223	MAGI1	membrane associated guanylate kinase, WW and PDZ doma	223	29	126
441308	OR4F21	olfactory receptor, family 4, subfamily F, member 21	136	116	126
83605	CCM2	cerebral cavernous malformation 2	102	150	126
7324	UBE2E1	ubiquitin-conjugating enzyme E2E 1 (UBC4/5 homolog, yeas	95	157	126
5685	PSMA4	proteasome (prosome, macropain) subunit, alpha type, 4	53	199	126
50813	COPS7A	COP9 constitutive photomorphogenic homolog subunit 7A (A	104	147	125.5
79137	C2orf17	chromosome 2 open reading frame 17	81	170	125.5
9646	CTR9	Ctr9, Paf1/RNA polymerase II complex component, homolog	106	144	125
200958	MUC20	mucin 20, cell surface associated	102	148	125
10488	CREB3	cAMP responsive element binding protein 3	93	157	125
5747	PTK2	PTK2 protein tyrosine kinase 2	77	173	125
163126	EID2	EP300 interacting inhibitor of differentiation 2	20	230	125
5441	POLR2L	polymerase (RNA) II (DNA directed) polypeptide L, 7.6kDa	198	51	124.5
374291	NDUFS7	NADH dehydrogenase (ubiquinone) Fe-S protein 7, 20kDa (I	186	63	124.5
389692	MAFA	v-maf musculoaponeurotic fibrosarcoma oncogene homolog	169	80	124.5
211	ALAS1	aminolevulinate, delta-, synthase 1	114	135	124.5
11070	TMEM115	transmembrane protein 115	89	160	124.5
23295	MGRN1	mahogunin, ring finger 1	233	15	124
3145	HMBS	hydroxymethylbilane synthase	116	132	124
91978	C19orf20	chromosome 19 open reading frame 20	85	163	124
1781	DYNC1I2	dynein, cytoplasmic 1, intermediate chain 2	49	199	124
4700	NDUFA6	NADH dehydrogenase (ubiquinone) 1 alpha subcomplex, 6,	22	225	123.5
54935	DUSP23	dual specificity phosphatase 23	154	92	123
55624	POMGNT1	protein O-linked mannose beta1,2-N-acetylglucosaminyltrans	113	133	123
60626	RIC8A	resistance to inhibitors of cholinesterase 8 homolog A (C. ele	112	134	123
4212	MEIS2	Meis1, myeloid ecotropic viral integration site 1 homolog 2 (r	91	155	123
23208	SYT11	synaptotagmin XI	73	173	123
4854	NOTCH3	Notch homolog 3 (Drosophila)	162	83	122.5
56900	C1orf119	chromosome 1 open reading frame 119	138	107	122.5
3589	IL11	interleukin 11	117	128	122.5
552889	LOC552889	hypothetical LOC552889	18	227	122.5
7251	TSG101	tumor susceptibility gene 101	171	73	122
858	CAV2	caveolin 2	120	124	122
7152	TOP1P2	topoisomerase (DNA) I pseudogene 2	119	125	122
10263	CDK2AP2	CDK2-associated protein 2	201	42	121.5
123016	TTC8	tetratricopeptide repeat domain 8	99	144	121.5
92154	LOC92154	hypothetical protein BC002770	81	162	121.5
3081	HGD	homogentisate 1,2-dioxygenase (homogentisate oxidase)	132	110	121
23365	ARHGEF12	Rho guanine nucleotide exchange factor (GEF) 12	123	119	121
23089	PEG10	paternally expressed 10	120	122	121
573	BAG1	BCL2-associated athanogene	112	130	121
23384	SPECC1L	SPECC1-like	86	156	121
90313	TP53I13	tumor protein p53 inducible protein 13	55	187	121
526	ATP6V1B2	ATPase, H+ transporting, lysosomal 56/58kDa, V1 subunit B	47	195	121
3375	IAPP	islet amyloid polypeptide	242	0	121
4821	NKX2-2	NK2 transcription factor related, locus 2 (Drosophila)	205	36	120.5
23113	PARC	p53-associated parkin-like cytoplasmic protein	92	149	120.5
10421	CD2BP2	CD2 (cytoplasmic tail) binding protein 2	53	188	120.5
10113	PREB	prolactin regulatory element binding	191	49	120
2644	GCHFR	GTP cyclohydrolase I feedback regulator	152	88	120

22818	COPZ1	coatomer protein complex, subunit zeta 1	150	90	120
1200	TPP1	tripeptidyl peptidase I	136	104	120
3913	LAMB2	laminin, beta 2 (laminin S)	130	110	120
66005	CHID1	chitinase domain containing 1	92	148	120
4642	MYO1D	myosin ID	65	175	120
29074	MRPL18	mitochondrial ribosomal protein L18	152	87	119.5
64969	MRPS5	mitochondrial ribosomal protein S5	102	137	119.5
10567	RABAC1	Rab acceptor 1 (prenylated)	86	153	119.5
9146	HGS	hepatocyte growth factor-regulated tyrosine kinase substrate	56	183	119.5
103910	MRLC2	myosin regulatory light chain MRLC2	23	216	119.5
7884	SLBP	stem-loop (histone) binding protein	124	114	119
136647	C7orf11	chromosome 7 open reading frame 11	120	118	119
6856	SYPL1	synaptophysin-like 1	67	171	119
55699	IARS2	isoleucine-tRNA synthetase 2, mitochondrial	129	108	118.5
22904	SBNO2	strawberry notch homolog 2 (Drosophila)	112	125	118.5
8775	NAPA	N-ethylmaleimide-sensitive factor attachment protein, alpha	67	170	118.5
112398	EGLN2	egl nine homolog 2 (C. elegans)	156	80	118
11230	PRAF2	PRA1 domain family, member 2	125	111	118
6604	SMARCD3	SWI/SNF related, matrix associated, actin dependent regula	81	155	118
23210	PTDSR	phosphatidylserine receptor	81	155	118
10431	TIMM23	translocase of inner mitochondrial membrane 23 homolog (y	22	214	118
8985	PLOD3	procollagen-lysine, 2-oxoglutarate 5-dioxygenase 3	192	43	117.5
140606	SELM	selenoprotein M	160	75	117.5
10075	HUWE1	HECT, UBA and WWE domain containing 1	136	99	117.5
2734	GLG1	golgi apparatus protein 1	94	141	117.5
4580	MTX1	metaxin 1	85	150	117.5
26001	RNF167	ring finger protein 167	82	153	117.5
23400	ATP13A2	ATPase type 13A2	223	11	117
26119	LDLRAP1	low density lipoprotein receptor adaptor protein 1	128	106	117
23029	RBM34	RNA binding motif protein 34	85	149	117
57106	NAT14	N-acetyltransferase 14	20	214	117
64866	CDCP1	CUB domain containing protein 1	88	145	116.5
92714	ARRDC1	arrestin domain containing 1	134	98	116
9168	TMSB10	thymosin, beta 10	82	150	116
376267	RAB15	RAB15, member RAS onocogene family	96	135	115.5
79171	MGC10433	hypothetical protein MGC10433	90	141	115.5
80218	NAT13	N-acetyltransferase 13	55	176	115.5
127262	FAM79A	family with sequence similarity 79, member A	99	131	115
345757	TMEM157	transmembrane protein 157	93	137	115
4681	NBL1	neuroblastoma, suppression of tumorigenicity 1	143	86	114.5
57405	SPBC25	spindle pole body component 25 homolog (S. cerevisiae)	121	108	114.5
4938	OAS1	2',5'-oligoadenylate synthetase 1, 40/46kDa	79	150	114.5
54916	C14orf101	chromosome 14 open reading frame 101	22	207	114.5
2926	GRSF1	G-rich RNA sequence binding factor 1	192	36	114
8760	CDS2	CDP-diacylglycerol synthase (phosphatidate cytidylyltransfer	134	94	114
1174	AP1S1	adaptor-related protein complex 1, sigma 1 subunit	104	124	114
10953	TOMM34	translocase of outer mitochondrial membrane 34	93	135	114
5049	PAFAH1B2	platelet-activating factor acetylhydrolase, isoform Ib, beta sul	59	169	114
7332	UBE2L3	ubiquitin-conjugating enzyme E2L 3	57	171	114
7327	UBE2G2	ubiquitin-conjugating enzyme E2G 2 (UBC7 homolog, yeast)	27	201	114
84153	RNASEH2C	ribonuclease H2, subunit C	134	93	113.5

219771	C10orf9	chromosome 10 open reading frame 9	101	126	113.5
55486	PARL	presenilin associated, rhomboid-like	144	82	113
3029	HAGH	hydroxyacylglutathione hydrolase	138	88	113
89941	RHOT2	ras homolog gene family, member T2	47	179	113
6240	RRM1	ribonucleotide reductase M1 polypeptide	116	109	112.5
5510	PPP1R7	protein phosphatase 1, regulatory subunit 7	104	121	112.5
148223	C19orf25	chromosome 19 open reading frame 25	95	130	112.5
22936	ELL2	elongation factor, RNA polymerase II, 2	73	152	112.5
10912	GADD45G	growth arrest and DNA-damage-inducible, gamma	18	207	112.5
51504	HSPC152	hypothetical protein HSPC152	160	63	111.5
229	ALDOB	aldolase B, fructose-bisphosphate	153	70	111.5
25805	BAMBI	BMP and activin membrane-bound inhibitor homolog (Xenop	97	126	111.5
2064	ERBB2	v-erb-b2 erythroblastic leukemia viral oncogene homolog 2, ı	89	134	111.5
8612	PPAP2C	phosphatidic acid phosphatase type 2C	79	144	111.5
10743	RAI1	retinoic acid induced 1	37	186	111.5
2648	GCN5L2	GCN5 general control of amino-acid synthesis 5-like 2 (yeas	191	31	111
10212	DDX39	DEAD (Asp-Glu-Ala-Asp) box polypeptide 39	126	96	111
55813	UTP6	UTP6, small subunit (SSU) processome component, homolo	93	129	111
2483	FRG1	FSHD region gene 1	83	139	111
3106	HLA-B	major histocompatibility complex, class I, B	0	222	111
91012	LASS5	LAG1 homolog, ceramide synthase 5 (S. cerevisiae)	182	39	110.5
113878	DTX2	deltex homolog 2 (Drosophila)	146	75	110.5
1647	GADD45A	growth arrest and DNA-damage-inducible, alpha	111	110	110.5
7360	UGP2	UDP-glucose pyrophosphorylase 2	69	152	110.5
65117	FLJ11021	similar to splicing factor, arginine/serine-rich 4	61	160	110.5
435	ASL	argininosuccinate lyase	22	199	110.5
3192	HNRPU	heterogeneous nuclear ribonucleoprotein U (scaffold attachn	154	66	110
79990	PLEKHH3	pleckstrin homology domain containing, family H (with MyTH	116	104	110
2021	ENDOG	endonuclease G	106	114	110
6388	SDF2	stromal cell-derived factor 2	106	114	110
23383	KIAA0892	KIAA0892	99	121	110
78992	YIPF2	Yip1 domain family, member 2	79	141	110
57617	VPS18	vacuolar protein sorting 18 homolog (S. cerevisiae)	67	153	110
51506	UFC1	ubiquitin-fold modifier conjugating enzyme 1	4	216	110
6533	SLC6A6	solute carrier family 6 (neurotransmitter transporter, taurine),	120	99	109.5
29015	SLC43A3	solute carrier family 43, member 3	97	122	109.5
29903	CCDC106	coiled-coil domain containing 106	58	161	109.5
23038	WDTC1	WD and tetratricopeptide repeats 1	201	17	109
51477	ISYNA1	myo-inositol 1-phosphate synthase A1	162	56	109
81628	TSC22D4	TSC22 domain family, member 4	114	104	109
11112	HIBADH	3-hydroxyisobutyrate dehydrogenase	104	114	109
81622	UNC93B1	unc-93 homolog B1 (C. elegans)	83	135	109
284424	C19orf30	chromosome 19 open reading frame 30	35	183	109
55207	ARL8B	ADP-ribosylation factor-like 8B	140	77	108.5
84154	BXDC1	brix domain containing 1	136	80	108
5339	PLEC1	plectin 1, intermediate filament binding protein 500kDa	136	80	108
10617	STAMBP	STAM binding protein	118	98	108
55147	RBM23	RNA binding motif protein 23	115	101	108
2065	ERBB3	v-erb-b2 erythroblastic leukemia viral oncogene homolog 3 (a	111	105	108
2288	FKBP4	FK506 binding protein 4, 59kDa	97	119	108
4814	NINJ1	ninjurin 1	97	119	108

587	BCAT2	branched chain aminotransferase 2, mitochondrial	144	71	107.5
11135	CDC42EP1	CDC42 effector protein (Rho GTPase binding) 1	119	96	107.5
7108	TM7SF2	transmembrane 7 superfamily member 2	81	134	107.5
6855	SYP	synaptophysin	29	186	107.5
8715	NOL4	nucleolar protein 4	215	0	107.5
51312	SLC25A37	solute carrier family 25, member 37	114	100	107
3988	LIPA	lipase A, lysosomal acid, cholesterol esterase (Wolman dise	109	105	107
80031	SEMA6D	sema domain, transmembrane domain (TM), and cytoplasmi	0	214	107
9823	ARMCX2	armadillo repeat containing, X-linked 2	136	77	106.5
11153	HYPE	Huntingtin interacting protein E	122	91	106.5
10555	AGPAT2	1-acylglycerol-3-phosphate O-acyltransferase 2 (lysophosph	119	94	106.5
3294	HSD17B2	hydroxysteroid (17-beta) dehydrogenase 2	97	116	106.5
4140	MARK3	MAP/microtubule affinity-regulating kinase 3	97	116	106.5
7045	TGFBI	transforming growth factor, beta-induced, 68kDa	91	122	106.5
7408	VASP	vasodilator-stimulated phosphoprotein	86	127	106.5
8446	DUSP11	dual specificity phosphatase 11 (RNA/RNP complex 1-intera	79	134	106.5
4218	RAB8A	RAB8A, member RAS oncogene family	111	101	106
140467	ZNF358	zinc finger protein 358	104	108	106
10577	NPC2	Niemann-Pick disease, type C2	103	109	106
80762	NDFIP1	Nedd4 family interacting protein 1	68	144	106
25837	RAB26	RAB26, member RAS oncogene family	65	147	106
7205	TRIP6	thyroid hormone receptor interactor 6	155	56	105.5
26519	TIMM10	translocase of inner mitochondrial membrane 10 homolog (y	128	83	105.5
84246	MED10	mediator of RNA polymerase II transcription, subunit 10 hom	56	155	105.5
57674	C17orf27	chromosome 17 open reading frame 27	43	168	105.5
154754	TRY6	trypsinogen C	211	0	105.5
4948	OCA2	oculocutaneous albinism II (pink-eye dilution homolog, mous	211	0	105.5
57662	KIAA1543	KIAA1543	148	62	105
5425	POLD2	polymerase (DNA directed), delta 2, regulatory subunit 50kD	110	100	105
5900	RALGDS	ral guanine nucleotide dissociation stimulator	106	104	105
1911	PHC1	polyhomeotic homolog 1 (Drosophila)	58	152	105
83460	TMEM93	transmembrane protein 93	0	210	105
9550	ATP6V1G1	ATPase, H+ transporting, lysosomal 13kDa, V1 subunit G1	177	32	104.5
10890	RAB10	RAB10, member RAS oncogene family	161	48	104.5
6150	MRPL23	mitochondrial ribosomal protein L23	161	48	104.5
30827	CXXC1	CXXC finger 1 (PHD domain)	124	85	104.5
55898	UNC45A	unc-45 homolog A (C. elegans)	116	93	104.5
5357	PLS1	plastin 1 (I isoform)	58	151	104.5
220717	RPLP0-like	similar to ribosomal protein P0	56	153	104.5
9049	AIP	aryl hydrocarbon receptor interacting protein	69	139	104
7343	UBTF	upstream binding transcription factor, RNA polymerase I	57	151	104
63891	RNF123	ring finger protein 123	51	157	104
51400	PPME1	protein phosphatase methylesterase 1	38	170	104
9926	LPGAT1	lysophosphatidylglycerol acyltransferase 1	116	91	103.5
29965	C16orf5	chromosome 16 open reading frame 5	91	116	103.5
3338	DNAJC4	DnaJ (Hsp40) homolog, subfamily C, member 4	85	122	103.5
57130	ATP13A1	ATPase type 13A1	83	124	103.5
9184	BUB3	BUB3 budding uninhibited by benzimidazoles 3 homolog (ye	77	130	103.5
2947	GSTM3	glutathione S-transferase M3 (brain)	65	142	103.5
2673	GFPT1	glutamine-fructose-6-phosphate transaminase 1	109	97	103
8661	EIF3S10	eukaryotic translation initiation factor 3, subunit 10 theta, 150	102	104	103

65982	ZNF447	zinc finger protein 447	96	110	103
4694	NDUFA1	NADH dehydrogenase (ubiquinone) 1 alpha subcomplex, 1,	79	127	103
51196	PLCE1	phospholipase C, epsilon 1	79	127	103
2314	FLII	flightless I homolog (Drosophila)	115	90	102.5
27173	SLC39A1	solute carrier family 39 (zinc transporter), member 1	115	90	102.5
128434	C20orf102	chromosome 20 open reading frame 102	61	144	102.5
405753	DUOXA2	dual oxidase maturation factor 2	173	31	102
57018	CCNL1	cyclin L1	130	74	102
8764	TNFRSF14	tumor necrosis factor receptor superfamily, member 14 (herp	106	98	102
353	APRT	adenine phosphoribosyltransferase	95	109	102
4154	MBNL1	muscleblind-like (Drosophila)	20	184	102
9844	ELMO1	engulfment and cell motility 1	92	111	101.5
10092	ARPC5	actin related protein 2/3 complex, subunit 5, 16kDa	63	140	101.5
5036	PA2G4	proliferation-associated 2G4, 38kDa	38	165	101.5
28959	TMEM176B	transmembrane protein 176B	194	8	101
64838	FNDC4	fibronectin type III domain containing 4	176	26	101
112942	CCDC104	coiled-coil domain containing 104	109	93	101
400949	FKSG49	FKSG49	100	102	101
1267	CNP	2',3'-cyclic nucleotide 3' phosphodiesterase	81	121	101
54972	TMEM132A	transmembrane protein 132A	76	126	101
3725	JUN	jun oncogene	33	169	101
8500	PPFIA1	protein tyrosine phosphatase, receptor type, f polypeptide (P	103	98	100.5
949	SCARB1	scavenger receptor class B, member 1	85	116	100.5
11336	EXOC3	exocyst complex component 3	83	118	100.5
9218	VAPA	VAMP (vesicle-associated membrane protein)-associated pr	39	162	100.5
3920	LAMP2	lysosomal-associated membrane protein 2	106	94	100
81031	SLC2A10	solute carrier family 2 (facilitated glucose transporter), memb	79	121	100
6730	SRP68	signal recognition particle 68kDa	73	127	100
1785	DNM2	dynamin 2	36	164	100
8974	P4HA2	procollagen-proline, 2-oxoglutarate 4-dioxygenase (proline 4	200	0	100
389831	LOC389831	hypothetical gene supported by AL713796	176	23	99.5
25776	PGEA1	PKD2 interactor, golgi and endoplasmic reticulum associatec	102	97	99.5
80011	NIP30	NEFA-interacting nuclear protein NIP30	92	107	99.5
1639	DCTN1	dynactin 1 (p150, glued homolog, Drosophila)	92	107	99.5
4041	LRP5	low density lipoprotein receptor-related protein 5	75	124	99.5
79897	RPP21	ribonuclease P 21kDa subunit	46	153	99.5
56946	C11orf30	chromosome 11 open reading frame 30	26	173	99.5
1622	DBI	diazepam binding inhibitor (GABA receptor modulator, acyl-C	178	20	99
51117	COQ4	coenzyme Q4 homolog (S. cerevisiae)	159	39	99
4088	SMAD3	SMAD family member 3	151	47	99
10534	SSSCA1	Sjogren's syndrome/scleroderma autoantigen 1	146	52	99
26097	C1orf77	chromosome 1 open reading frame 77	134	64	99
64943	NT5DC2	5'-nucleotidase domain containing 2	132	66	99
53916	RAB4B	RAB4B, member RAS oncogene family	79	119	99
55643	BTBD2	BTB (POZ) domain containing 2	184	13	98.5
8896	BUD31	BUD31 homolog (yeast)	156	41	98.5
80895	ILKAP	integrin-linked kinase-associated serine/threonine phosphata	146	51	98.5
84164	ASCC2	activating signal cointegrator 1 complex subunit 2	92	105	98.5
79142	PHF23	PHD finger protein 23	81	116	98.5
25849	DKFZP564O	DKFZP564O0823 protein	74	123	98.5
220988	HNRPA3	heterogeneous nuclear ribonucleoprotein A3	65	132	98.5

5087	PBX1	pre-B-cell leukemia transcription factor 1	123	73	98
3670	ISL1	ISL1 transcription factor, LIM/homeodomain, (islet-1)	114	82	98
79102	RNF26	ring finger protein 26	110	86	98
27111	SDCBP2	syndecan binding protein (syntenin) 2	106	90	98
84304	NUDT22	nudix (nucleoside diphosphate linked moiety X)-type motif 22	65	131	98
22887	FOXJ3	forkhead box J3	162	33	97.5
27154	BRPF3	bromodomain and PHD finger containing, 3	140	55	97.5
5320	PLA2G2A	phospholipase A2, group IIA (platelets, synovial fluid)	131	64	97.5
114971	PTPMT1	protein tyrosine phosphatase, mitochondrial 1	124	71	97.5
4830	NME1	non-metastatic cells 1, protein (NM23A) expressed in	124	71	97.5
64122	FN3K	fructosamine 3 kinase	91	104	97.5
84193	SETD3	SET domain containing 3	81	114	97.5
1523	CUTL1	cut-like 1, CCAAT displacement protein (Drosophila)	77	118	97.5
54531	MIER2	mesoderm induction early response 1, family member 2	61	134	97.5
51816	CECR1	cat eye syndrome chromosome region, candidate 1	45	150	97.5
7095	TLOC1	translocation protein 1	112	82	97
8976	WASL	Wiskott-Aldrich syndrome-like	109	85	97
5564	PRKAB1	protein kinase, AMP-activated, beta 1 non-catalytic subunit	106	88	97
55507	GPRC5D	G protein-coupled receptor, family C, group 5, member D	81	113	97
56647	BCCIP	BRCA2 and CDKN1A interacting protein	81	113	97
2952	GSTT1	glutathione S-transferase theta 1	0	194	97
3437	IFIT3	interferon-induced protein with tetratricopeptide repeats 3	194	0	97
112802	KRT71	keratin 71	154	39	96.5
10130	PDIA6	protein disulfide isomerase family A, member 6	152	41	96.5
156	ADRBK1	adrenergic, beta, receptor kinase 1	106	87	96.5
51177	PLEKHO1	pleckstrin homology domain containing, family O member 1	79	114	96.5
57019	CIAPIN1	cytokine induced apoptosis inhibitor 1	61	132	96.5
114131	UCN3	urocortin 3 (stresscopin)	38	155	96.5
114785	MBD6	methyl-CpG binding domain protein 6	18	175	96.5
10296	MAEA	macrophage erythroblast attacher	140	52	96
2806	GOT2	glutamic-oxaloacetic transaminase 2, mitochondrial (asparta	114	78	96
9927	MFN2	mitofusin 2	73	119	96
38	ACAT1	acetyl-Coenzyme A acetyltransferase 1 (acetoacetyl Coenzy	71	121	96
2995	GYPC	glycophorin C (Gerbich blood group)	110	81	95.5
9337	CNOT8	CCR4-NOT transcription complex, subunit 8	59	132	95.5
8543	LMO4	LIM domain only 4	149	41	95
1994	ELAVL1	ELAV (embryonic lethal, abnormal vision, Drosophila)-like 1	100	90	95
638	BIK	BCL2-interacting killer (apoptosis-inducing)	37	153	95
3998	LMAN1	lectin, mannose-binding, 1	36	154	95
2053	EPHX2	epoxide hydrolase 2, cytoplasmic	30	160	95
3181	HNRPA2B1	heterogeneous nuclear ribonucleoprotein A2/B1	158	31	94.5
4133	MAP2	microtubule-associated protein 2	75	114	94.5
9533	POLR1C	polymerase (RNA) I polypeptide C, 30kDa	39	150	94.5
5298	PIK4CB	phosphatidylinositol 4-kinase, catalytic, beta polypeptide	189	0	94.5
85365	ALG2	asparagine-linked glycosylation 2 homolog (S. cerevisiae, al	120	68	94
63931	MRPS14	mitochondrial ribosomal protein S14	118	70	94
8992	ATP6V0E1	ATPase, H+ transporting, lysosomal 9kDa, V0 subunit e1	95	93	94
5825	ABCD3	ATP-binding cassette, sub-family D (ALD), member 3	67	121	94
7520	XRCC5	X-ray repair complementing defective repair in Chinese ham	58	130	94
114804	RNF157	ring finger protein 157	44	144	94
80764	THAP7	THAP domain containing 7	38	150	94

23387	KIAA0999	KIAA0999 protein	104	83	93.5
54918	CMTM6	CKLF-like MARVEL transmembrane domain containing 6	102	85	93.5
51465	UBE2J1	ubiquitin-conjugating enzyme E2, J1 (UBC6 homolog, yeast)	56	131	93.5
1291	COL6A1	collagen, type VI, alpha 1	147	39	93
57761	TRIB3	tribbles homolog 3 (Drosophila)	138	48	93
283991	FAM100B	family with sequence similarity 100, member B	107	79	93
252969	NEIL2	nei like 2 (E. coli)	80	106	93
171023	ASXL1	additional sex combs like 1 (Drosophila)	64	122	93
22948	CCT5	chaperonin containing TCP1, subunit 5 (epsilon)	112	73	92.5
9552	SPAG7	sperm associated antigen 7	102	83	92.5
5652	PRSS8	protease, serine, 8 (prostasin)	87	98	92.5
221927	C7orf27	chromosome 7 open reading frame 27	73	112	92.5
8402	SLC25A11	solute carrier family 25 (mitochondrial carrier; oxoglutarate ca	51	134	92.5
60592	SCOC	short coiled-coil protein	14	171	92.5
7533	YWHAH	tyrosine 3-monooxygenase/tryptophan 5-monooxygenase ac	122	62	92
9898	UBAP2L	ubiquitin associated protein 2-like	104	80	92
253012	LOC253012	hypothetical protein LOC253012	84	100	92
56262	LRRC8A	leucine rich repeat containing 8 family, member A	54	130	92
440	ASNS	asparagine synthetase	42	142	92
81894	SLC25A28	solute carrier family 25, member 28	118	65	91.5
6288	SAA1	serum amyloid A1	106	77	91.5
6542	SLC7A2	solute carrier family 7 (cationic amino acid transporter, y+ sy	77	106	91.5
3021	H3F3B	H3 histone, family 3B (H3.3B)	67	116	91.5
93100	NAPRT1	nicotinate phosphoribosyltransferase domain containing 1	67	116	91.5
8470	SORBS2	sorbin and SH3 domain containing 2	32	151	91.5
10049	DNAJB6	DnaJ (Hsp40) homolog, subfamily B, member 6	18	165	91.5
10904	BLCAP	bladder cancer associated protein	110	72	91
1072	CFL1	cofilin 1 (non-muscle)	57	125	91
79961	DENND2D	DENN/MADD domain containing 2D	53	129	91
65110	UPF3A	UPF3 regulator of nonsense transcripts homolog A (yeast)	94	87	90.5
84064	HDHD2	haloacid dehalogenase-like hydrolase domain containing 2	85	96	90.5
1630	DCC	deleted in colorectal carcinoma	67	114	90.5
6386	SDCBP	syndecan binding protein (syntenin)	33	148	90.5
10955	SERINC3	serine incorporator 3	132	48	90
8874	ARHGEF7	Rho guanine nucleotide exchange factor (GEF) 7	93	87	90
23481	PES1	pescadillo homolog 1, containing BRCT domain (zebrafish)	77	103	90
55967	NDUFA12	NADH dehydrogenase (ubiquinone) 1 alpha subcomplex, 12	59	121	90
7936	RDBP	RD RNA binding protein	52	128	90
9719	ADAMTSL2	ADAMTS-like 2	41	139	90
51738	GHRL	ghrelin/obestatin preprohormone	180	0	90
800	CALD1	caldesmon 1	125	54	89.5
2634	GBP2	guanylate binding protein 2, interferon-inducible	109	70	89.5
79155	TNIP2	TNFAIP3 interacting protein 2	96	83	89.5
84310	MGC11257	hypothetical protein MGC11257	92	87	89.5
9409	PEX16	peroxisomal biogenesis factor 16	75	104	89.5
51124	IER3IP1	immediate early response 3 interacting protein 1	35	144	89.5
6434	SFRS10	splicing factor, arginine/serine-rich 10 (transformer 2 homolo	33	146	89.5
11334	TUSC2	tumor suppressor candidate 2	32	147	89.5
53826	FXYD6	FXYD domain containing ion transport regulator 6	24	155	89.5
10211	FLOT1	flotillin 1	0	179	89.5
116988	CENTG3	centaurin, gamma 3	126	52	89

10726	NUDC	nuclear distribution gene C homolog (A. nidulans)	118	60	89
10476	ATP5H	ATP synthase, H+ transporting, mitochondrial F0 complex, s	110	68	89
71	ACTG1	actin, gamma 1	79	99	89
1432	MAPK14	mitogen-activated protein kinase 14	69	109	89
79668	PARP8	poly (ADP-ribose) polymerase family, member 8	63	115	89
10142	AKAP9	A kinase (PRKA) anchor protein (yotiao) 9	49	129	89
80213	TM2D3	TM2 domain containing 3	47	131	89
4828	NMB	neuromedin B	18	160	89
150678	MYEOV2	myeloma overexpressed 2	178	0	89
3689	ITGB2	integrin, beta 2 (complement component 3 receptor 3 and 4 s	95	82	88.5
57583	TMEM181	transmembrane protein 181	90	87	88.5
1209	CLPTM1	cleft lip and palate associated transmembrane protein 1	69	108	88.5
2872	MKNK2	MAP kinase interacting serine/threonine kinase 2	69	108	88.5
4704	NDUFA9	NADH dehydrogenase (ubiquinone) 1 alpha subcomplex, 9,	67	110	88.5
3956	LGALS1	lectin, galactoside-binding, soluble, 1 (galectin 1)	63	114	88.5
347862	PDDC1	Parkinson disease 7 domain containing 1	0	177	88.5
90113	LOC90113	hypothetical protein BC009862	75	101	88
91942	NDUFA12L	NDUFA12-like	65	111	88
11148	HHLA2	HERV-H LTR-associating 2	65	111	88
54858	PGPEP1	pyroglutamyl-peptidase I	65	111	88
10856	RUVBL2	RuvB-like 2 (E. coli)	63	113	88
1208	CLPS	colipase, pancreatic	176	0	88
5969	REGL	regenerating islet-derived-like, pancreatic stone protein-like,	165	10	87.5
81618	ITM2C	integral membrane protein 2C	138	37	87.5
5689	PSMB1	proteasome (prosome, macropain) subunit, beta type, 1	65	110	87.5
84128	WDR75	WD repeat domain 75	61	114	87.5
11138	TBC1D8	TBC1 domain family, member 8 (with GRAM domain)	0	175	87.5
7417	VDAC2	voltage-dependent anion channel 2	110	64	87
6428	SFRS3	splicing factor, arginine/serine-rich 3	109	65	87
79073	TMEM109	transmembrane protein 109	99	75	87
8440	NCK2	NCK adaptor protein 2	87	87	87
283635	C14orf24	chromosome 14 open reading frame 24	68	106	87
170954	KIAA1949	KIAA1949	30	144	87
7866	IFRD2	interferon-related developmental regulator 2	119	54	86.5
23	ABCF1	ATP-binding cassette, sub-family F (GCN20), member 1	105	68	86.5
4927	NUP88	nucleoporin 88kDa	100	73	86.5
84305	WIBG	within bgcn homolog (Drosophila)	67	106	86.5
1112	CHES1	checkpoint suppressor 1	56	117	86.5
221749	C6orf145	chromosome 6 open reading frame 145	53	120	86.5
8710	SERPINB7	serpin peptidase inhibitor, clade B (ovalbumin), member 7	45	128	86.5
93964	FLJ14311	hypothetical gene FLJ14311	26	147	86.5
9812	KIAA0141	KIAA0141	108	64	86
79600	FLJ21127	tectonic	107	65	86
11031	RAB31	RAB31, member RAS oncogene family	106	66	86
6709	SPTAN1	spectrin, alpha, non-erythrocytic 1 (alpha-fodrin)	0	172	86
81533	ITFG1	integrin alpha FG-GAP repeat containing 1	168	3	85.5
4026	LPP	LIM domain containing preferred translocation partner in lipo	68	103	85.5
51574	LARP7	La ribonucleoprotein domain family, member 7	60	111	85.5
327	APEH	N-acylaminoacyl-peptide hydrolase	50	121	85.5
10658	CUGBP1	CUG triplet repeat, RNA binding protein 1	46	125	85.5
56654	NPDC1	neural proliferation, differentiation and control, 1	39	132	85.5

267	AMFR	autocrine motility factor receptor	33	138	85.5
9124	PDLIM1	PDZ and LIM domain 1 (elfin)	171	0	85.5
5711	PSMD5	proteasome (prosome, macropain) 26S subunit, non-ATPase	131	39	85
55332	DRAM	damage-regulated autophagy modulator	124	46	85
760	CA2	carbonic anhydrase II	124	46	85
6118	RPA2	replication protein A2, 32kDa	97	73	85
140733	C20orf133	chromosome 20 open reading frame 133	94	76	85
64223	GBL	G protein beta subunit-like	88	82	85
7342	UBP1	upstream binding protein 1 (LBP-1a)	67	103	85
5654	HTRA1	HtrA serine peptidase 1	51	119	85
55819	RNF130	ring finger protein 130	39	131	85
5270	SERPINE2	serpin peptidase inhibitor, clade E (nexin, plasminogen activa	0	170	85
4782	NFIC	nuclear factor I/C (CCAAT-binding transcription factor)	134	35	84.5
10330	TMEM4	transmembrane protein 4	96	73	84.5
7980	TFPI2	tissue factor pathway inhibitor 2	86	83	84.5
113386	LOC113386	similar to envelope protein	66	103	84.5
283229	EFCAB4A	EF-hand calcium binding domain 4A	61	108	84.5
79581	GPR172A	G protein-coupled receptor 172A	55	114	84.5
84693	MCEE	methylmalonyl CoA epimerase	51	118	84.5
5256	PHKA2	phosphorylase kinase, alpha 2 (liver)	38	131	84.5
16	AARS	alanyl-tRNA synthetase	148	20	84
5869	RAB5B	RAB5B, member RAS oncogene family	124	44	84
9328	GTF3C5	general transcription factor IIIC, polypeptide 5, 63kDa	122	46	84
10787	NCKAP1	NCK-associated protein 1	106	62	84
80271	ITPKC	inositol 1,4,5-trisphosphate 3-kinase C	106	62	84
27077	EPPB9	B9 protein	45	123	84
3275	PRMT2	protein arginine methyltransferase 2	21	147	84
2280	FKBP1A	FK506 binding protein 1A, 12kDa	0	168	84
253782	LASS6	LAG1 homolog, ceramide synthase 6 (S. cerevisiae)	75	92	83.5
4054	LTBP3	latent transforming growth factor beta binding protein 3	53	114	83.5
10576	CCT2	chaperonin containing TCP1, subunit 2 (beta)	43	124	83.5
395	ARHGAP6	Rho GTPase activating protein 6	42	125	83.5
997	CDC34	cell division cycle 34 homolog (S. cerevisiae)	0	167	83.5
57175	CORO1B	coronin, actin binding protein, 1B	153	13	83
9318	COPS2	COP9 constitutive photomorphogenic homolog subunit 2 (Ar	130	36	83
5695	PSMB7	proteasome (prosome, macropain) subunit, beta type, 7	81	85	83
9360	PPIG	peptidylprolyl isomerase G (cyclophilin G)	76	90	83
7057	THBS1	thrombospondin 1	73	93	83
51074	APIP	APAF1 interacting protein	58	108	83
90203	SNX21	sorting nexin family member 21	29	137	83
81539	SLC38A1	solute carrier family 38, member 1	111	54	82.5
7280	TUBB2A	tubulin, beta 2A	111	54	82.5
6195	RPS6KA1	ribosomal protein S6 kinase, 90kDa, polypeptide 1	91	74	82.5
2562	GABRB3	gamma-aminobutyric acid (GABA) A receptor, beta 3	87	78	82.5
11340	EXOSC8	exosome component 8	65	100	82.5
375743	PTAR1	protein prenyltransferase alpha subunit repeat containing 1	54	111	82.5
3925	STMN1	stathmin 1/oncoprotein 18	41	124	82.5
79784	MYH14	myosin, heavy chain 14	12	153	82.5
1809	DPYSL3	dihydropyrimidinase-like 3	6	159	82.5
64067	NPAS3	neuronal PAS domain protein 3	113	51	82
8881	CDC16	cell division cycle 16 homolog (S. cerevisiae)	88	76	82

1801	DPH1	DPH1 homolog (S. cerevisiae)	79	85	82
23039	XPO7	exportin 7	77	87	82
6275	S100A4	S100 calcium binding protein A4	53	111	82
9785	DHX38	DEAH (Asp-Glu-Ala-His) box polypeptide 38	37	127	82
6305	SBF1	SET binding factor 1	32	132	82
1593	CYP27A1	cytochrome P450, family 27, subfamily A, polypeptide 1	30	134	82
28970	C11orf54	chromosome 11 open reading frame 54	116	47	81.5
80023	NRSN2	neurensin 2	99	64	81.5
253982	ASPHD1	aspartate beta-hydroxylase domain containing 1	63	100	81.5
7169	TPM2	tropomyosin 2 (beta)	61	102	81.5
84557	MAP1LC3A	microtubule-associated protein 1 light chain 3 alpha	59	104	81.5
79169	C1orf35	chromosome 1 open reading frame 35	58	105	81.5
79034	C7orf26	chromosome 7 open reading frame 26	55	108	81.5
4738	NEDD8	neural precursor cell expressed, developmentally down-regu	55	108	81.5
57492	ARID1B	AT rich interactive domain 1B (SWI1-like)	50	113	81.5
58506	SR-A1	serine arginine-rich pre-mRNA splicing factor SR-A1	31	132	81.5
58517	RBM25	RNA binding motif protein 25	8	155	81.5
148413	LOC148413	hypothetical protein LOC148413	0	163	81.5
6575	SLC20A2	solute carrier family 20 (phosphate transporter), member 2	144	18	81
3487	IGFBP4	insulin-like growth factor binding protein 4	108	54	81
55568	GALNT10	UDP-N-acetyl-alpha-D-galactosamine:polypeptide N-acetylg	97	65	81
7204	TRIO	triple functional domain (PTPRF interacting)	92	70	81
285440	CYP4V2	cytochrome P450, family 4, subfamily V, polypeptide 2	86	76	81
64976	MRPL40	mitochondrial ribosomal protein L40	75	87	81
55837	C14orf11	chromosome 14 open reading frame 11	69	93	81
125988	P117	hypothetical protein P117	51	111	81
5986	RFNG	RFNG O-fucosylpeptide 3-beta-N-acetylglucosaminyltransfer	98	63	80.5
8924	HERC2	hect domain and RLD 2	97	64	80.5
6137	RPL13	ribosomal protein L13	69	92	80.5
3091	HIF1A	hypoxia-inducible factor 1, alpha subunit (basic helix-loop-he	59	102	80.5
22839	DLGAP4	discs, large (Drosophila) homolog-associated protein 4	56	105	80.5
23367	LARP1	La ribonucleoprotein domain family, member 1	35	126	80.5
56987	BBX	bobby sox homolog (Drosophila)	129	31	80
9260	PDLIM7	PDZ and LIM domain 7 (enigma)	81	79	80
50861	STMN3	stathmin-like 3	65	95	80
26207	PITPNC1	phosphatidylinositol transfer protein, cytoplasmic 1	43	117	80
64963	MRPS11	mitochondrial ribosomal protein S11	35	125	80
57584	ARHGAP21	Rho GTPase activating protein 21	30	130	80
117177	RAB3IP	RAB3A interacting protein (rabin3)	24	136	80
25897	RNF19	ring finger protein 19	18	142	80
64110	MAGEF1	melanoma antigen family F, 1	144	15	79.5
51474	LIMA1	LIM domain and actin binding 1	94	65	79.5
4696	NDUFA3	NADH dehydrogenase (ubiquinone) 1 alpha subcomplex, 3,	159	0	79.5
4791	NFKB2	nuclear factor of kappa light polypeptide gene enhancer in B	102	56	79
23131	GPATCH8	G patch domain containing 8	89	69	79
151636	DTX3L	deltex 3-like (Drosophila)	88	70	79
2355	FOSL2	FOS-like antigen 2	75	83	79
1356	CP	ceruloplasmin (ferroxidase)	71	87	79
5127	PCTK1	PCTAIRE protein kinase 1	71	87	79
55168	MRPS18A	mitochondrial ribosomal protein S18A	59	99	79
124995	MRPL10	mitochondrial ribosomal protein L10	24	134	79

113246	C12orf57	chromosome 12 open reading frame 57	108	49	78.5
171017	ZNF384	zinc finger protein 384	93	64	78.5
4825	NKX6-1	NK6 transcription factor related, locus 1 (Drosophila)	67	90	78.5
483	ATP1B3	ATPase, Na+/K+ transporting, beta 3 polypeptide	60	97	78.5
8763	CD164	CD164 molecule, sialomucin	36	121	78.5
51343	FZR1	fizzy/cell division cycle 20 related 1 (Drosophila)	18	139	78.5
9810	RNF40	ring finger protein 40	102	54	78
5154	PDGFA	platelet-derived growth factor alpha polypeptide	71	85	78
51035	LOC51035	unknown protein LOC51035	13	143	78
65003	MRPL11	mitochondrial ribosomal protein L11	113	42	77.5
53	ACP2	acid phosphatase 2, lysosomal	109	46	77.5
23105	FSTL4	follistatin-like 4	83	72	77.5
10573	MRPL28	mitochondrial ribosomal protein L28	75	80	77.5
114883	OSBPL9	oxysterol binding protein-like 9	65	90	77.5
79770	C5orf14	chromosome 5 open reading frame 14	48	107	77.5
27161	EIF2C2	eukaryotic translation initiation factor 2C, 2	146	8	77
55738	ARFGAP1	ADP-ribosylation factor GTPase activating protein 1	128	26	77
55379	LRRC59	leucine rich repeat containing 59	110	44	77
9100	USP10	ubiquitin specific peptidase 10	104	50	77
6721	SREBF2	sterol regulatory element binding transcription factor 2	81	73	77
222658	KCTD20	potassium channel tetramerisation domain containing 20	81	73	77
84326	MGC13114	hypothetical protein MGC13114	72	82	77
51319	RSRC1	arginine/serine-rich coiled-coil 1	69	85	77
55573	CDV3	CDV3 homolog (mouse)	55	99	77
25829	C22orf5	chromosome 22 open reading frame 5	53	101	77
56922	MCCC1	methylcrotonoyl-Coenzyme A carboxylase 1 (alpha)	46	108	77
554203	LOC554203	hypothetical LOC554203	32	122	77
119504	C10orf104	chromosome 10 open reading frame 104	15	139	77
1155	TBCB	tubulin folding cofactor B	154	0	77
23450	SF3B3	splicing factor 3b, subunit 3, 130kDa	154	0	77
54948	MRPL16	mitochondrial ribosomal protein L16	130	23	76.5
6772	STAT1	signal transducer and activator of transcription 1, 91kDa	122	31	76.5
81688	C6orf62	chromosome 6 open reading frame 62	110	43	76.5
79703	FLJ22531	hypothetical protein FLJ22531	104	49	76.5
55752	SEP11	septin 11	80	73	76.5
81577	GFOD2	glucose-fructose oxidoreductase domain containing 2	71	82	76.5
283232	TMEM80	transmembrane protein 80	63	90	76.5
51253	MRPL37	mitochondrial ribosomal protein L37	54	99	76.5
7326	UBE2G1	ubiquitin-conjugating enzyme E2G 1 (UBC7 homolog, yeast)	37	116	76.5
10413	YAP1	Yes-associated protein 1, 65kDa	35	118	76.5
5831	PYCR1	pyrroline-5-carboxylate reductase 1	127	25	76
11180	WDR6	WD repeat domain 6	127	25	76
3710	ITPR3	inositol 1,4,5-triphosphate receptor, type 3	118	34	76
84516	DCTN5	dynactin 5 (p25)	86	66	76
8943	AP3D1	adaptor-related protein complex 3, delta 1 subunit	84	68	76
79887	FLJ22662	hypothetical protein FLJ22662	83	69	76
23635	SSBP2	single-stranded DNA binding protein 2	83	69	76
221908	C7orf47	chromosome 7 open reading frame 47	79	73	76
79180	EFHD2	EF-hand domain family, member D2	79	73	76
377711	KIAA1833	hypothetical protein KIAA1833	67	85	76
11054	OGFR	opioid growth factor receptor	61	91	76

26137	ZBTB20	zinc finger and BTB domain containing 20	47	105	76
387856	LOC387856	similar to expressed sequence AI836003	41	111	76
2512	FTL	ferritin, light polypeptide	41	111	76
51170	HSD17B11	hydroxysteroid (17-beta) dehydrogenase 11	32	120	76
9784	SNX17	sorting nexin 17	28	124	76
161882	ZFPM1	zinc finger protein, multitype 1	27	125	76
10140	TOB1	transducer of ERBB2, 1	25	127	76
57038	RARSL	arginyl-tRNA synthetase-like	2	150	76
9526	MPDU1	mannose-P-dolichol utilization defect 1	0	152	76
5898	RALA	v-ral simian leukemia viral oncogene homolog A (ras related)	152	0	76
9984	THOC1	THO complex 1	148	3	75.5
5435	POLR2F	polymerase (RNA) II (DNA directed) polypeptide F	83	68	75.5
51143	DYNC1LI1	dynein, cytoplasmic 1, light intermediate chain 1	73	78	75.5
3786	KCNQ3	potassium voltage-gated channel, KQT-like subfamily, memb	47	104	75.5
84752	MGC4655	hypothetical protein MGC4655	47	104	75.5
50488	MINK1	misshapen-like kinase 1 (zebrafish)	151	0	75.5
65018	PINK1	PTEN induced putative kinase 1	151	0	75.5
64965	MRPS9	mitochondrial ribosomal protein S9	126	24	75
56927	GPR108	G protein-coupled receptor 108	112	38	75
79622	C16orf33	chromosome 16 open reading frame 33	109	41	75
64747	MFSD1	major facilitator superfamily domain containing 1	96	54	75
1969	EPHA2	EPH receptor A2	95	55	75
9588	PRDX6	peroxiredoxin 6	84	66	75
51747	CROP	cisplatin resistance-associated overexpressed protein	63	87	75
6251	RSU1	Ras suppressor protein 1	62	88	75
28976	ACAD9	acyl-Coenzyme A dehydrogenase family, member 9	50	100	75
23352	ZUBR1	zinc finger, UBR1 type 1	49	101	75
9275	BCL7B	B-cell CLL/lymphoma 7B	110	39	74.5
2921	CXCL3	chemokine (C-X-C motif) ligand 3	107	42	74.5
54963	UCKL1	uridine-cytidine kinase 1-like 1	102	47	74.5
8890	EIF2B4	eukaryotic translation initiation factor 2B, subunit 4 delta, 67k	102	47	74.5
64599	PERQ1	PERQ amino acid rich, with GYF domain 1	96	53	74.5
6640	SNTA1	syntrophin, alpha 1 (dystrophin-associated protein A1, 59kDa	77	72	74.5
11199	ANXA10	annexin A10	77	72	74.5
23199	KIAA0182	KIAA0182	69	80	74.5
25911	RP11-529I10	deleted in a mouse model of primary ciliary dyskinesia	67	82	74.5
10072	DPP3	dipeptidyl-peptidase 3	67	82	74.5
29990	PILRB	paired immunoglobin-like type 2 receptor beta	36	113	74.5
6016	RIT1	Ras-like without CAAX 1	26	123	74.5
30008	EFEMP2	EGF-containing fibulin-like extracellular matrix protein 2	20	129	74.5
220323	OAF	OAF homolog (Drosophila)	100	48	74
55081	IFT57	intraflagellar transport 57 homolog (Chlamydomonas)	89	59	74
55625	ZDHHC7	zinc finger, DHHC-type containing 7	89	59	74
3241	HPCAL1	hippocalcin-like 1	88	60	74
4853	NOTCH2	Notch homolog 2 (Drosophila)	83	65	74
7323	UBE2D3	ubiquitin-conjugating enzyme E2D 3 (UBC4/5 homolog, yeas	83	65	74
57140	RNPEPL1	arginyl aminopeptidase (aminopeptidase B)-like 1	82	66	74
79644	SRD5A2L	steroid 5 alpha-reductase 2-like	73	75	74
23303	KIF13B	kinesin family member 13B	61	87	74
6303	SAT1	spermidine/spermine N1-acetyltransferase 1	58	90	74
80150	ASRGL1	asparaginase like 1	55	93	74

652995	UCA1	urothelial cancer associated 1	32	116	74
2690	GHR	growth hormone receptor	26	122	74
64236	PDLIM2	PDZ and LIM domain 2 (mystique)	22	126	74
23386	NUDCD3	NudC domain containing 3	148	0	74
55740	ENAH	enabled homolog (Drosophila)	132	15	73.5
5529	PPP2R5E	protein phosphatase 2, regulatory subunit B (B56), epsilon is	121	26	73.5
9126	SMC3	structural maintenance of chromosomes 3	108	39	73.5
4047	LSS	lanosterol synthase (2,3-oxidosqualene-lanosterol cyclase)	93	54	73.5
9013	TAF1C	TATA box binding protein (TBP)-associated factor, RNA poly	83	64	73.5
3400	ID4	inhibitor of DNA binding 4, dominant negative helix-loop-heli	79	68	73.5
23102	TBC1D2B	TBC1 domain family, member 2B	71	76	73.5
23293	SMG6	Smg-6 homolog, nonsense mediated mRNA decay factor (C	69	78	73.5
81615	TMEM163	transmembrane protein 163	58	89	73.5
3669	ISG20	interferon stimulated exonuclease gene 20kDa	57	90	73.5
11188	NISCH	nischarin	51	96	73.5
10160	FARP1	FERM, RhoGEF (ARHGEF) and pleckstrin domain protein 1	49	98	73.5
8239	USP9X	ubiquitin specific peptidase 9, X-linked	17	130	73.5
10924	SMPDL3A	sphingomyelin phosphodiesterase, acid-like 3A	6	141	73.5
24148	PRPF6	PRP6 pre-mRNA processing factor 6 homolog (S. cerevisiae	0	147	73.5
2120	ETV6	ets variant gene 6 (TEL oncogene)	141	5	73
309	ANXA6	annexin A6	122	24	73
80176	SPSB1	splA/ryanodine receptor domain and SOCS box containing 1	102	44	73
51588	PIAS4	protein inhibitor of activated STAT, 4	61	85	73
6667	SP1	Sp1 transcription factor	59	87	73
10969	EBNA1BP2	EBNA1 binding protein 2	57	89	73
83593	RASSF5	Ras association (RalGDS/AF-6) domain family 5	45	101	73
6451	SH3BGRL	SH3 domain binding glutamic acid-rich protein like	35	111	73
409	ARRB2	arrestin, beta 2	35	111	73
219927	MRPL21	mitochondrial ribosomal protein L21	25	121	73
1326	MAP3K8	mitogen-activated protein kinase kinase kinase 8	21	125	73
83940	TATDN1	TatD DNase domain containing 1	106	39	72.5
1455	CSNK1G2	casein kinase 1, gamma 2	104	41	72.5
4255	MGMT	O-6-methylguanine-DNA methyltransferase	96	49	72.5
3035	HARS	histidyl-tRNA synthetase	86	59	72.5
56478	EIF4ENIF1	eukaryotic translation initiation factor 4E nuclear import facto	65	80	72.5
10455	PECI	peroxisomal D3,D2-enoyl-CoA isomerase	31	114	72.5
23363	OBSL1	obscurin-like 1	28	117	72.5
84148	MYST1	MYST histone acetyltransferase 1	10	135	72.5
55277	FLJ10986	hypothetical protein FLJ10986	130	14	72
497661	LOC497661	putative NFkB activating protein	97	47	72
27175	TUBG2	tubulin, gamma 2	97	47	72
6383	SDC2	syndecan 2 (heparan sulfate proteoglycan 1, cell surface-ass	95	49	72
3875	KRT18	keratin 18	90	54	72
9320	TRIP12	thyroid hormone receptor interactor 12	82	62	72
7593	MZF1	myeloid zinc finger 1	78	66	72
1604	CD55	CD55 molecule, decay accelerating factor for complement (C	77	67	72
9670	IPO13	importin 13	69	75	72
10043	TOM1	target of myb1 (chicken)	67	77	72
56172	ANKH	ankylosis, progressive homolog (mouse)	61	83	72
9139	CBFA2T2	core-binding factor, runt domain, alpha subunit 2; translocate	56	88	72
1490	CTGF	connective tissue growth factor	22	122	72

23517	SKIV2L2	superkiller viralicidic activity 2-like 2 (S. cerevisiae)	140	3	71.5
4817	NIT1	nitrilase 1	132	11	71.5
10569	SLU7	SLU7 splicing factor homolog (S. cerevisiae)	98	45	71.5
7050	TGIF	TGFB-induced factor (TALE family homeobox)	97	46	71.5
6764	ST5	suppression of tumorigenicity 5	69	74	71.5
4588	MUC6	mucin 6, oligomeric mucus/gel-forming	53	90	71.5
54788	DNAJB12	DnaJ (Hsp40) homolog, subfamily B, member 12	45	98	71.5
11346	SYNPO	synaptopodin	36	107	71.5
547	KIF1A	kinesin family member 1A	136	6	71
57597	BAHCC1	BAH domain and coiled-coil containing 1	132	10	71
805	CALM2	calmodulin 2 (phosphorylase kinase, delta)	121	21	71
10900	RPIP8	RaP2 interacting protein 8	119	23	71
5770	PTPN1	protein tyrosine phosphatase, non-receptor type 1	85	57	71
2621	GAS6	growth arrest-specific 6	65	77	71
4204	MECP2	methyl CpG binding protein 2 (Rett syndrome)	56	86	71
10099	TSPAN3	tetraspanin 3	120	21	70.5
23613	PRKCBP1	protein kinase C binding protein 1	92	49	70.5
1983	EIF5	eukaryotic translation initiation factor 5	88	53	70.5
53838	C11orf24	chromosome 11 open reading frame 24	79	62	70.5
25957	C6orf111	chromosome 6 open reading frame 111	56	85	70.5
56850	GRIPAP1	GRIP1 associated protein 1	51	90	70.5
23313	C22orf9	chromosome 22 open reading frame 9	50	91	70.5
6729	SRP54	signal recognition particle 54kDa	35	106	70.5
2274	FHL2	four and a half LIM domains 2	19	122	70.5
1514	CTSL	cathepsin L	0	141	70.5
81669	CCNL2	cyclin L2	104	36	70
116840	CNTROB	centrobin, centrosomal BRCA2 interacting protein	101	39	70
126789	PUSL1	pseudouridylate synthase-like 1	81	59	70
84617	TUBB6	tubulin, beta 6	77	63	70
51382	ATP6V1D	ATPase, H+ transporting, lysosomal 34kDa, V1 subunit D	71	69	70
9973	CCS	copper chaperone for superoxide dismutase	59	81	70
955	ENTPD6	ectonucleoside triphosphate diphosphohydrolase 6 (putative	50	90	70
5467	PPARD	peroxisome proliferator-activated receptor delta	38	102	70
5356	PLRG1	pleiotropic regulator 1 (PRL1 homolog, Arabidopsis)	36	104	70
57418	WDR18	WD repeat domain 18	29	111	70
1025	CDK9	cyclin-dependent kinase 9 (CDC2-related kinase)	140	0	70
26145	IRF2BP1	interferon regulatory factor 2 binding protein 1	111	28	69.5
55753	OGDHL	oxoglutarate dehydrogenase-like	102	37	69.5
92689	FAM114A1	family with sequence similarity 114, member A1	59	80	69.5
115416	C7orf30	chromosome 7 open reading frame 30	59	80	69.5
55000	TUG1	taurine upregulated gene 1	56	83	69.5
6494	SIPA1	signal-induced proliferation-associated gene 1	51	88	69.5
81855	SFXN3	sideroflexin 3	48	91	69.5
6814	STXBP3	syntaxin binding protein 3	46	93	69.5
5987	TRIM27	tripartite motif-containing 27	46	93	69.5
10076	PTPRU	protein tyrosine phosphatase, receptor type, U	40	99	69.5
50717	WDR42A	WD repeat domain 42A	31	108	69.5
80230	RUFY1	RUN and FYVE domain containing 1	25	114	69.5
6519	SLC3A1	solute carrier family 3 (cystine, dibasic and neutral amino aci	129	9	69
10025	THRAP5	thyroid hormone receptor associated protein 5	116	22	69
57619	SHROOM3	shroom family member 3	32	106	69

22848	AAK1	AP2 associated kinase 1	10	128	69
55262	C7orf43	chromosome 7 open reading frame 43	138	0	69
9099	USP2	ubiquitin specific peptidase 2	114	23	68.5
81858	SHARPIN	SHANK-associated RH domain interactor	89	48	68.5
26063	DECR2	2,4-dienoyl CoA reductase 2, peroxisomal	75	62	68.5
8554	PIAS1	protein inhibitor of activated STAT, 1	69	68	68.5
4905	NSF	N-ethylmaleimide-sensitive factor	69	68	68.5
817	CAMK2D	calcium/calmodulin-dependent protein kinase (CaM kinase) I	67	70	68.5
283899	CCDC95	coiled-coil domain containing 95	67	70	68.5
3339	HSPG2	heparan sulfate proteoglycan 2 (perlecan)	57	80	68.5
4837	NNMT	nicotinamide N-methyltransferase	52	85	68.5
388796	LOC388796	hypothetical LOC388796	120	16	68
9329	GTF3C4	general transcription factor IIIC, polypeptide 4, 90kDa	110	26	68
84152	PPP1R1B	protein phosphatase 1, regulatory (inhibitor) subunit 1B (dop	89	47	68
3155	HMGCL	3-hydroxymethyl-3-methylglutaryl-Coenzyme A lyase (hydro:	85	51	68
3816	KLK1	kallikrein 1	82	54	68
81	ACTN4	actinin, alpha 4	53	83	68
8603	C4orf8	chromosome 4 open reading frame 8	18	118	68
6902	TBCA	tubulin folding cofactor A	12	124	68
28998	MRPL13	mitochondrial ribosomal protein L13	47	88	67.5
5802	PTPRS	protein tyrosine phosphatase, receptor type, S	0	135	67.5
1213	CLTC	clathrin, heavy chain (Hc)	135	0	67.5
10791	VAMP5	vesicle-associated membrane protein 5 (myobrevin)	118	16	67
51776	ZAK	sterile alpha motif and leucine zipper containing kinase AZK	95	39	67
79594	C1orf166	chromosome 1 open reading frame 166	93	41	67
9093	DNAJA3	DnaJ (Hsp40) homolog, subfamily A, member 3	61	73	67
26065	LSM14A	LSM14A, SCD6 homolog A (S. cerevisiae)	57	77	67
56605	ERO1LB	ERO1-like beta (S. cerevisiae)	46	88	67
11243	PMF1	polyamine-modulated factor 1	39	95	67
7145	TNS1	tensin 1	4	130	67
1644	DDC	dopa decarboxylase (aromatic L-amino acid decarboxylase)	2	132	67
389203	LOC389203	hypothetical gene supported by BC032431	120	13	66.5
55657	ZNF692	zinc finger protein 692	71	62	66.5
549	AUH	AU RNA binding protein/enoyl-Coenzyme A hydratase	61	72	66.5
9322	TRIP10	thyroid hormone receptor interactor 10	48	85	66.5
1716	DGUOK	deoxyguanosine kinase	40	93	66.5
4037	LRP3	low density lipoprotein receptor-related protein 3	25	108	66.5
3098	HK1	hexokinase 1	17	116	66.5
9240	PNMA1	paraneoplastic antigen MA1	104	28	66
5329	PLAUR	plasminogen activator, urokinase receptor	87	45	66
147179	WIPF2	WAS/WASL interacting protein family, member 2	85	47	66
6690	SPINK1	serine peptidase inhibitor, Kazal type 1	85	47	66
64928	MRPL14	mitochondrial ribosomal protein L14	77	55	66
8819	SAP30	Sin3A-associated protein, 30kDa	67	65	66
286262	C9orf75	chromosome 9 open reading frame 75	63	69	66
10336	PCGF3	polycomb group ring finger 3	59	73	66
55110	FLJ10292	mago-nashi homolog 2	55	77	66
1672	DEFB1	defensin, beta 1	37	95	66
10269	ZMPSTE24	zinc metallopeptidase (STE24 homolog, yeast)	32	100	66
55651	NOLA2	nucleolar protein family A, member 2 (H/ACA small nucleolar	0	132	66
3749	KCNC4	potassium voltage-gated channel, Shaw-related subfamily, n	111	20	65.5

1294	COL7A1	collagen, type VII, alpha 1 (epidermolysis bullosa, dystrophic	102	29	65.5
6382	SDC1	syndecan 1	61	70	65.5
3691	ITGB4	integrin, beta 4	61	70	65.5
51114	ZDHHC9	zinc finger, DHHC-type containing 9	53	78	65.5
54968	TMEM70	transmembrane protein 70	46	85	65.5
10367	CBARA1	calcium binding atopy-related autoantigen 1	31	100	65.5
51108	METTL9	methyltransferase like 9	131	0	65.5
57634	EP400	E1A binding protein p400	95	35	65
7375	USP4	ubiquitin specific peptidase 4 (proto-oncogene)	73	57	65
10636	RGS14	regulator of G-protein signalling 14	47	83	65
139231	CXorf39	chromosome X open reading frame 39	44	86	65
2878	GPX3	glutathione peroxidase 3 (plasma)	20	110	65
10270	AKAP8	A kinase (PRKA) anchor protein 8	10	120	65
6711	SPTBN1	spectrin, beta, non-erythrocytic 1	130	0	65
26751	SH3YL1	SH3 domain containing, Ysc84-like 1 (S. cerevisiae)	94	35	64.5
498	ATP5A1	ATP synthase, H+ transporting, mitochondrial F1 complex, a	87	42	64.5
51015	ISOC1	isochorismatase domain containing 1	86	43	64.5
1108	CHD4	chromodomain helicase DNA binding protein 4	76	53	64.5
25833	POU2F3	POU domain, class 2, transcription factor 3	67	62	64.5
535	ATP6V0A1	ATPase, H+ transporting, lysosomal V0 subunit a1	65	64	64.5
117246	FTSJ3	FtsJ homolog 3 (E. coli)	61	68	64.5
284996	RNF149	ring finger protein 149	35	94	64.5
64689	GORASP1	golgi reassembly stacking protein 1, 65kDa	26	103	64.5
4741	NEFM	neurofilament, medium polypeptide 150kDa	8	121	64.5
6048	RNF5	ring finger protein 5	0	129	64.5
9491	PSMF1	proteasome (prosome, macropain) inhibitor subunit 1 (PI31)	115	13	64
114757	CYGB	cytoglobin	87	41	64
55062	WIPI1	WD repeat domain, phosphoinositide interacting 1	73	55	64
9510	ADAMTS1	ADAM metallopeptidase with thrombospondin type 1 motif, 1	73	55	64
10078	TSSC4	tumor suppressing subtransferable candidate 4	71	57	64
1832	DSP	desmoplakin	66	62	64
4794	NFKBIE	nuclear factor of kappa light polypeptide gene enhancer in B	59	69	64
4135	MAP6	microtubule-associated protein 6	55	73	64
6046	BRD2	bromodomain containing 2	54	74	64
54800	KLHL24	kelch-like 24 (Drosophila)	45	83	64
5157	PDGFRL	platelet-derived growth factor receptor-like	41	87	64
56915	EXOSC5	exosome component 5	120	7	63.5
161502	C15orf26	chromosome 15 open reading frame 26	114	13	63.5
23229	ARHGEF9	Cdc42 guanine nucleotide exchange factor (GEF) 9	95	32	63.5
23499	MACF1	microtubule-actin crosslinking factor 1	93	34	63.5
29079	MED4	mediator of RNA polymerase II transcription, subunit 4 homo	86	41	63.5
57215	THAP11	THAP domain containing 11	81	46	63.5
10724	MGEA5	meningioma expressed antigen 5 (hyaluronidase)	80	47	63.5
64949	MRPS26	mitochondrial ribosomal protein S26	71	56	63.5
27245	AHDC1	AT hook, DNA binding motif, containing 1	71	56	63.5
2145	EZH1	enhancer of zeste homolog 1 (Drosophila)	65	62	63.5
83752	LONP2	lon peptidase 2, peroxisomal	51	76	63.5
23025	UNC13A	unc-13 homolog A (C. elegans)	46	81	63.5
9577	BRE	brain and reproductive organ-expressed (TNFRSF1A modula	45	82	63.5
142	PARP1	poly (ADP-ribose) polymerase family, member 1	32	95	63.5
202915	MGC9712	hypothetical protein MGC9712	17	110	63.5

339229	LOC339229	hypothetical protein LOC339229	75	51	63
9905	RUTBC1	RUN and TBC1 domain containing 1	73	53	63
4864	NPC1	Niemann-Pick disease, type C1	71	55	63
846	CASR	calcium-sensing receptor (hypocalciuric hypercalcemia 1, se	69	57	63
55245	C20orf44	chromosome 20 open reading frame 44	61	65	63
51056	LAP3	leucine aminopeptidase 3	59	67	63
223	ALDH9A1	aldehyde dehydrogenase 9 family, member A1	59	67	63
404093	CUEDC1	CUE domain containing 1	51	75	63
11231	SEC63	SEC63 homolog (S. cerevisiae)	41	85	63
51123	ZNF706	zinc finger protein 706	0	126	63
10970	CKAP4	cytoskeleton-associated protein 4	73	52	62.5
79602	ADIPOR2	adiponectin receptor 2	45	80	62.5
23593	HEBP2	heme binding protein 2	40	85	62.5
23162	MAPK8IP3	mitogen-activated protein kinase 8 interacting protein 3	38	87	62.5
5007	OSBP	oxysterol binding protein	35	90	62.5
8405	SPOP	speckle-type POZ protein	33	92	62.5
6653	SORL1	sortilin-related receptor, L(DLR class) A repeats-containing	77	47	62
5720	PSME1	proteasome (prosome, macropain) activator subunit 1 (PA28	63	61	62
92579	G6PC3	glucose 6 phosphatase, catalytic, 3	61	63	62
3712	IVD	isovaleryl Coenzyme A dehydrogenase	39	85	62
7436	VLDLR	very low density lipoprotein receptor	25	99	62
84232	MAF1	MAF1 homolog (S. cerevisiae)	20	104	62
3624	INHBA	inhibin, beta A (activin A, activin AB alpha polypeptide)	19	105	62
1528	CYB5A	cytochrome b5 type A (microsomal)	17	107	62
8826	IQGAP1	IQ motif containing GTPase activating protein 1	10	114	62
201514	ZNF584	zinc finger protein 584	124	0	62
2261	FGFR3	fibroblast growth factor receptor 3 (achondroplasia, thanatop	124	0	62
3191	HNRPL	heterogeneous nuclear ribonucleoprotein L	87	36	61.5
8813	DPM1	dolichyl-phosphate mannosyltransferase polypeptide 1, catal	73	50	61.5
9774	BCLAF1	BCL2-associated transcription factor 1	54	69	61.5
10459	MAD2L2	MAD2 mitotic arrest deficient-like 2 (yeast)	36	87	61.5
7181	NR2C1	nuclear receptor subfamily 2, group C, member 1	30	93	61.5
80142	PTGES2	prostaglandin E synthase 2	99	23	61
4116	MAGOH	mago-nashi homolog, proliferation-associated (Drosophila)	93	29	61
10458	BAIAP2	BAI1-associated protein 2	86	36	61
23216	TBC1D1	TBC1 (tre-2/USP6, BUB2, cdc16) domain family, member 1	85	37	61
57026	PDXP	pyridoxal (pyridoxine, vitamin B6) phosphatase	81	41	61
90378	SAMD1	sterile alpha motif domain containing 1	81	41	61
5536	PPP5C	protein phosphatase 5, catalytic subunit	71	51	61
56999	ADAMTS9	ADAM metallopeptidase with thrombospondin type 1 motif, 9	61	61	61
139322	FAM121A	family with sequence similarity 121A	59	63	61
339290	LOC339290	hypothetical protein LOC339290	57	65	61
1912	PHC2	polyhomeotic homolog 2 (Drosophila)	53	69	61
2217	FCGRT	Fc fragment of IgG, receptor, transporter, alpha	45	77	61
80135	BXDC5	brix domain containing 5	45	77	61
5716	PSMD10	proteasome (prosome, macropain) 26S subunit, non-ATPase	42	80	61
55852	TEX2	testis expressed sequence 2	38	84	61
64170	CARD9	caspase recruitment domain family, member 9	35	87	61
196383	MGC7036	hypothetical protein MGC7036	34	88	61
10436	EMG1	EMG1 nucleolar protein homolog (S. cerevisiae)	34	88	61
6194	RPS6	ribosomal protein S6	0	122	61

4094	MAF	v-maf musculoaponeurotic fibrosarcoma oncogene homolog	122	0	61
64837	KLC2	kinesin light chain 2	122	0	61
5538	PPT1	palmitoyl-protein thioesterase 1 (ceroid-lipofuscinosis, neuro	122	0	61
9907	KIAA0415	KIAA0415 protein	113	8	60.5
55362	TMEM63B	transmembrane protein 63B	67	54	60.5
51734	SEPX1	selenoprotein X, 1	59	62	60.5
64748	LPPR2	lipid phosphate phosphatase-related protein type 2	59	62	60.5
22955	SCMH1	sex comb on midleg homolog 1 (Drosophila)	56	65	60.5
23524	SRRM2	serine/arginine repetitive matrix 2	55	66	60.5
88745	C6orf153	chromosome 6 open reading frame 153	50	71	60.5
124044	C16orf76	chromosome 16 open reading frame 76	43	78	60.5
55707	NECAP2	NECAP endocytosis associated 2	41	80	60.5
401647	C10orf132	chromosome 10 open reading frame 132	39	82	60.5
26123	C10orf61	chromosome 10 open reading frame 61	36	85	60.5
730125	LOC730125	hypothetical protein LOC730125	8	113	60.5
27258	LSM3	LSM3 homolog, U6 small nuclear RNA associated (S. cerevi	0	121	60.5
55544	RBM38	RNA binding motif protein 38	91	29	60
10562	OLFM4	olfactomedin 4	78	42	60
63905	MANBAL	mannosidase, beta A, lysosomal-like	63	57	60
27257	LSM1	LSM1 homolog, U6 small nuclear RNA associated (S. cerevi	33	87	60
54617	INOC1	INO80 complex homolog 1 (S. cerevisiae)	32	88	60
10898	CPSF4	cleavage and polyadenylation specific factor 4, 30kDa	22	98	60
6812	STXBP1	syntaxin binding protein 1	109	10	59.5
781	CACNA2D1	calcium channel, voltage-dependent, alpha 2/delta subunit 1	108	11	59.5
123169	LEO1	Leo1, Paf1/RNA polymerase II complex component, homolog	75	44	59.5
445328	FLJ43692	ARHGEF5-like	55	64	59.5
6892	TAPBP	TAP binding protein (tapasin)	45	74	59.5
283349	RASSF3	Ras association (RalGDS/AF-6) domain family 3	26	93	59.5
125061	AFMID	arylformamidase	0	119	59.5
116841	C1orf142	chromosome 1 open reading frame 142	0	119	59.5
51621	KLF13	Kruppel-like factor 13	119	0	59.5
55671	SMEK1	SMEK homolog 1, suppressor of mek1 (Dictyostelium)	90	28	59
686	BTD	biotinidase	85	33	59
51430	C1orf9	chromosome 1 open reading frame 9	84	34	59
135138	PACRG	PARK2 co-regulated	77	41	59
63874	ABHD4	abhydrolase domain containing 4	69	49	59
51131	PHF11	PHD finger protein 11	69	49	59
10625	IVNS1ABP	influenza virus NS1A binding protein	67	51	59
9052	GPRC5A	G protein-coupled receptor, family C, group 5, member A	59	59	59
252839	TMEM9	transmembrane protein 9	51	67	59
8517	IKBKG	inhibitor of kappa light polypeptide gene enhancer in B-cells,	50	68	59
4811	NID1	nidogen 1	49	69	59
816	CAMK2B	calcium/calmodulin-dependent protein kinase (CaM kinase) I	43	75	59
50855	PARD6A	par-6 partitioning defective 6 homolog alpha (C. elegans)	22	96	59
3268	HRBL	HIV-1 Rev binding protein-like	19	99	59
5818	PVRL1	poliovirus receptor-related 1 (herpesvirus entry mediator C; r	18	100	59
147093	LOC147093	hypothetical protein LOC147093	0	118	59
23215	BAT2D1	BAT2 domain containing 1	115	2	58.5
8086	AAAS	achalasia, adrenocortical insufficiency, alacrimia (Allgrove, tr	114	3	58.5
400569	MED11	mediator of RNA polymerase II transcription, subunit 11 hom	106	11	58.5
3960	LGALS4	lectin, galactoside-binding, soluble, 4 (galectin 4)	104	13	58.5

221477	C6orf89	chromosome 6 open reading frame 89	91	26	58.5
56889	TM9SF3	transmembrane 9 superfamily member 3	91	26	58.5
4134	MAP4	microtubule-associated protein 4	85	32	58.5
27344	PCSK1N	proprotein convertase subtilisin/kexin type 1 inhibitor	67	50	58.5
2022	ENG	endoglin (Osler-Rendu-Weber syndrome 1)	64	53	58.5
65123	INTS3	integrator complex subunit 3	57	60	58.5
51292	GMPR2	guanosine monophosphate reductase 2	52	65	58.5
823	CAPN1	calpain 1, (mu/I) large subunit	40	77	58.5
51704	GPRC5B	G protein-coupled receptor, family C, group 5, member B	35	82	58.5
9369	NRXN3	neurexin 3	8	109	58.5
5829	PXN	paxillin	117	0	58.5
50484	RRM2B	ribonucleotide reductase M2 B (TP53 inducible)	108	8	58
7991	TUSC3	tumor suppressor candidate 3	102	14	58
23181	DIP2A	DIP2 disco-interacting protein 2 homolog A (Drosophila)	98	18	58
1759	DNM1	dynamin 1	90	26	58
2593	GAMT	guanidinoacetate N-methyltransferase	84	32	58
197370	NSMCE1	non-SMC element 1 homolog (S. cerevisiae)	75	41	58
84976	DISP1	dispatched homolog 1 (Drosophila)	59	57	58
83855	KLF16	Kruppel-like factor 16	40	76	58
51399	TRAPPC4	trafficking protein particle complex 4	39	77	58
8073	PTP4A2	protein tyrosine phosphatase type IVA, member 2	28	88	58
714	C1QC	complement component 1, q subcomponent, C chain	6	110	58
25836	NIPBL	Nipped-B homolog (Drosophila)	6	110	58
23041	MON2	MON2 homolog (S. cerevisiae)	0	116	58
55973	BCAP29	B-cell receptor-associated protein 29	110	5	57.5
958	CD40	CD40 molecule, TNF receptor superfamily member 5	77	38	57.5
25953	PNKD	paroxysmal nonkinesiogenic dyskinesia	76	39	57.5
8876	VNN1	vanin 1	56	59	57.5
3110	HLXB9	homeobox HB9	49	66	57.5
92140	MTDH	metadherin	47	68	57.5
11035	RIPK3	receptor-interacting serine-threonine kinase 3	43	72	57.5
51218	GLRX5	glutaredoxin 5 homolog (S. cerevisiae)	32	83	57.5
2631	GBAS	glioblastoma amplified sequence	32	83	57.5
60412	EXOC4	exocyst complex component 4	27	88	57.5
9699	RIMS2	regulating synaptic membrane exocytosis 2	14	101	57.5
54480	CSGlcA-T	chondroitin sulfate glucuronyltransferase	115	0	57.5
10484	SEC23A	Sec23 homolog A (S. cerevisiae)	115	0	57.5
5495	PPM1B	protein phosphatase 1B (formerly 2C), magnesium-depende	86	28	57
11235	PDCD10	programmed cell death 10	75	39	57
3397	ID1	inhibitor of DNA binding 1, dominant negative helix-loop-heli	72	42	57
3837	KPNB1	karyopherin (importin) beta 1	67	47	57
1400	CRMP1	collapsin response mediator protein 1	57	57	57
54487	DGCR8	DiGeorge syndrome critical region gene 8	57	57	57
282991	BLOC1S2	biogenesis of lysosome-related organelles complex-1, subur	46	68	57
9673	SLC25A44	solute carrier family 25, member 44	43	71	57
6618	SNAPC2	small nuclear RNA activating complex, polypeptide 2, 45kDa	26	88	57
6778	STAT6	signal transducer and activator of transcription 6, interleukin-	23	91	57
6781	STC1	stanniocalcin 1	18	96	57
6541	SLC7A1	solute carrier family 7 (cationic amino acid transporter, y+ sy	12	102	57
27106	ARRDC2	arrestin domain containing 2	6	108	57
84306	PDCD2L	programmed cell death 2-like	0	114	57

9352	TXNL1	thioredoxin-like 1	114	0	57
79605	PGBD5	piggyBac transposable element derived 5	111	2	56.5
57167	SALL4	sal-like 4 (Drosophila)	110	3	56.5
4296	MAP3K11	mitogen-activated protein kinase kinase kinase 11	110	3	56.5
23587	C17orf81	chromosome 17 open reading frame 81	110	3	56.5
1434	CSE1L	CSE1 chromosome segregation 1-like (yeast)	93	20	56.5
586	BCAT1	branched chain aminotransferase 1, cytosolic	87	26	56.5
115939	C16orf42	chromosome 16 open reading frame 42	75	38	56.5
26993	AKAP8L	A kinase (PRKA) anchor protein 8-like	69	44	56.5
170506	DHX36	DEAH (Asp-Glu-Ala-His) box polypeptide 36	56	57	56.5
56996	SLC12A9	solute carrier family 12 (potassium/chloride transporters), me	56	57	56.5
55791	C1orf103	chromosome 1 open reading frame 103	53	60	56.5
64093	SMOC1	SPARC related modular calcium binding 1	49	64	56.5
7247	TSN	translin	41	72	56.5
1846	DUSP4	dual specificity phosphatase 4	36	77	56.5
25796	PGLS	6-phosphogluconolactonase	28	85	56.5
6236	RRAD	Ras-related associated with diabetes	19	94	56.5
29929	ALG6	asparagine-linked glycosylation 6 homolog (S. cerevisiae, al	18	95	56.5
54602	NDFIP2	Nedd4 family interacting protein 2	0	113	56.5
2739	GLO1	glyoxalase I	0	113	56.5
8662	EIF3S9	eukaryotic translation initiation factor 3, subunit 9 eta, 116kD	0	113	56.5
665	BNIP3L	BCL2/adenovirus E1B 19kDa interacting protein 3-like	0	113	56.5
6749	SSRP1	structure specific recognition protein 1	102	10	56
83667	SESN2	sestrin 2	102	10	56
55761	TTC17	tetratricopeptide repeat domain 17	95	17	56
55193	PB1	polybromo 1	83	29	56
10554	AGPAT1	1-acylglycerol-3-phosphate O-acyltransferase 1 (lysophosph	37	75	56
7764	ZNF217	zinc finger protein 217	14	98	56
8618	CADPS	Ca2+-dependent secretion activator	14	98	56
64426	SUDS3	suppressor of defective silencing 3 homolog (S. cerevisiae)	8	104	56
51375	SNX7	sorting nexin 7	112	0	56
9117	SEC22C	SEC22 vesicle trafficking protein homolog C (S. cerevisiae)	87	24	55.5
283375	SLC39A5	solute carrier family 39 (metal ion transporter), member 5	87	24	55.5
84798	C19orf48	chromosome 19 open reading frame 48	85	26	55.5
6601	SMARCC2	SWI/SNF related, matrix associated, actin dependent regula	71	40	55.5
79086	C19orf42	chromosome 19 open reading frame 42	68	43	55.5
55080	TAPBPL	TAP binding protein-like	67	44	55.5
2805	GOT1	glutamic-oxaloacetic transaminase 1, soluble (aspartate ami	60	51	55.5
10180	RBM6	RNA binding motif protein 6	59	52	55.5
51181	DCXR	dicarbonyl/L-xylulose reductase	49	62	55.5
51084	CRYL1	crystallin, lambda 1	38	73	55.5
8898	MTMR2	myotubularin related protein 2	29	82	55.5
9967	THRAP3	thyroid hormone receptor associated protein 3	19	92	55.5
6905	TBCE	tubulin folding cofactor E	0	111	55.5
595	CCND1	cyclin D1	0	111	55.5
80143	SIKE	suppressor of IKK epsilon	98	12	55
266722	HS6ST3	heparan sulfate 6-O-sulfotransferase 3	93	17	55
29950	SERTAD1	SERTA domain containing 1	85	25	55
7837	PXDN	peroxidasin homolog (Drosophila)	73	37	55
6697	SPR	sepiapterin reductase (7,8-dihydrobiopterin:NADP+ oxidored	65	45	55
9382	COG1	component of oligomeric golgi complex 1	50	60	55

79720	VPS37B	vacuolar protein sorting 37 homolog B (S. cerevisiae)	44	66	55
55140	ELP3	elongation protein 3 homolog (S. cerevisiae)	43	67	55
93185	IGSF8	immunoglobulin superfamily, member 8	37	73	55
56967	C14orf132	chromosome 14 open reading frame 132	36	74	55
22926	ATF6	activating transcription factor 6	36	74	55
54869	EPS8L1	EPS8-like 1	25	85	55
10040	TOM1L1	target of myb1-like 1 (chicken)	20	90	55
50626	CYHR1	cysteine/histidine-rich 1	12	98	55
11075	STMN2	stathmin-like 2	8	102	55
5460	POU5F1	POU domain, class 5, transcription factor 1	4	106	55
54020	SLC37A1	solute carrier family 37 (glycerol-3-phosphate transporter), m	2	108	55
4852	NPY	neuropeptide Y	0	110	55
51629	SLC25A39	solute carrier family 25, member 39	0	110	55
54913	RPP25	ribonuclease P 25kDa subunit	110	0	55
55213	RCBTB1	regulator of chromosome condensation (RCC1) and BTB (PO	86	23	54.5
51592	TRIM33	tripartite motif-containing 33	78	31	54.5
79447	C16orf53	chromosome 16 open reading frame 53	75	34	54.5
5002	SLC22A18	solute carrier family 22 (organic cation transporter), member	75	34	54.5
23560	GTPBP4	GTP binding protein 4	57	52	54.5
221496	LEMD2	LEM domain containing 2	44	65	54.5
129642	MBOAT2	membrane bound O-acyltransferase domain containing 2	6	103	54.5
7031	TFF1	trefoil factor 1 (breast cancer, estrogen-inducible sequence e	0	109	54.5
8460	TPST1	tyrosylprotein sulfotransferase 1	0	109	54.5
81544	GDPD5	glycerophosphodiester phosphodiesterase domain containin	99	9	54
7112	TMPO	thymopoietin	79	29	54
79726	WDR59	WD repeat domain 59	69	39	54
8508	NIPSNAP1	nipsnap homolog 1 (C. elegans)	63	45	54
7320	UBE2B	ubiquitin-conjugating enzyme E2B (RAD6 homolog)	59	49	54
113828	FAM83F	family with sequence similarity 83, member F	49	59	54
83549	UCK1	uridine-cytidine kinase 1	30	78	54
124935	SLC43A2	solute carrier family 43, member 2	28	80	54
6648	SOD2	superoxide dismutase 2, mitochondrial	26	82	54
23408	SIRT5	sirtuin (silent mating type information regulation 2 homolog) 5	4	104	54
1949	EFNB3	ephrin-B3	0	108	54
5407	PNLIPRP1	pancreatic lipase-related protein 1	108	0	54
421	ARVCF	armadillo repeat gene deletes in velocardiofacial syndrome	93	14	53.5
283149	BCL9L	B-cell CLL/lymphoma 9-like	81	26	53.5
5707	PSMD1	proteasome (prosome, macropain) 26S subunit, non-ATPase	67	40	53.5
10811	NOXA1	NADPH oxidase activator 1	61	46	53.5
1457	CSNK2A1	casein kinase 2, alpha 1 polypeptide	61	46	53.5
58476	TP53INP2	tumor protein p53 inducible nuclear protein 2	55	52	53.5
92305	TMEM129	transmembrane protein 129	54	53	53.5
53339	BTBD1	BTB (POZ) domain containing 1	50	57	53.5
283951	LOC283951	hypothetical protein LOC283951	47	60	53.5
23112	TNRC6B	trinucleotide repeat containing 6B	43	64	53.5
7920	BAT5	HLA-B associated transcript 5	40	67	53.5
283970	LOC283970	hypothetical protein LOC283970	38	69	53.5
30818	KCNIP3	Kv channel interacting protein 3, calsenilin	37	70	53.5
84287	ZDHHC16	zinc finger, DHHC-type containing 16	36	71	53.5
51282	SCAND1	SCAN domain containing 1	35	72	53.5
2107	ETF1	eukaryotic translation termination factor 1	32	75	53.5

7942	TFEB	transcription factor EB	32	75	53.5
828	CAPS	calcyphosine	30	77	53.5
114908	TMEM123	transmembrane protein 123	17	90	53.5
3320	HSP90AA1	heat shock protein 90kDa alpha (cytosolic), class A member	14	93	53.5
1793	DOCK1	dedicator of cytokinesis 1	8	99	53.5
90379	LOC90379	hypothetical protein BC002926	0	107	53.5
140597	TCEAL2	transcription elongation factor A (SII)-like 2	0	107	53.5
6723	SRM	spermidine synthase	107	0	53.5
9263	STK17A	serine/threonine kinase 17a (apoptosis-inducing)	104	2	53
79567	FAM65A	family with sequence similarity 65, member A	75	31	53
27348	TOR1B	torsin family 1, member B (torsin B)	69	37	53
5394	EXOSC10	exosome component 10	69	37	53
92609	TIMM50	translocase of inner mitochondrial membrane 50 homolog (S	59	47	53
57003	CCDC47	coiled-coil domain containing 47	56	50	53
23325	KIAA1033	KIAA1033	55	51	53
51493	C22orf28	chromosome 22 open reading frame 28	49	57	53
51188	SS18L2	synovial sarcoma translocation gene on chromosome 18-like	43	63	53
57530	CGN	cingulin	40	66	53
4919	ROR1	receptor tyrosine kinase-like orphan receptor 1	32	74	53
9064	MAP3K6	mitogen-activated protein kinase kinase kinase 6	26	80	53
7371	UCK2	uridine-cytidine kinase 2	26	80	53
27351	D15Wsu75e	DNA segment, Chr 15, Wayne State University 75, expresse	26	80	53
79683	ZDHHC14	zinc finger, DHHC-type containing 14	10	96	53
196403	DTX3	deltex 3 homolog (Drosophila)	8	98	53
5917	RARS	arginyl-tRNA synthetase	0	106	53
5162	PDHB	pyruvate dehydrogenase (lipoamide) beta	0	106	53
93487	C14orf32	chromosome 14 open reading frame 32	0	106	53
51507	C20orf43	chromosome 20 open reading frame 43	106	0	53
1723	DHODH	dihydroorotate dehydrogenase	106	0	53
10444	ZER1	zer-1 homolog (C. elegans)	106	0	53
3795	KHK	ketohexokinase (fructokinase)	102	3	52.5
25970	SH2B1	SH2B adaptor protein 1	82	23	52.5
3014	H2AFX	H2A histone family, member X	70	35	52.5
8202	NCOA3	nuclear receptor coactivator 3	63	42	52.5
9136	RRP9	RRP9, small subunit (SSU) processome component, homolo	59	46	52.5
50	ACO2	aconitase 2, mitochondrial	59	46	52.5
10758	TRAF3IP2	TRAF3 interacting protein 2	52	53	52.5
338657	CCDC84	coiled-coil domain containing 84	52	53	52.5
55234	SMU1	smu-1 suppressor of mec-8 and unc-52 homolog (C. elegans	51	54	52.5
84650	EBPL	emopamil binding protein-like	36	69	52.5
127829	ARL8A	ADP-ribosylation factor-like 8A	35	70	52.5
83482	SCRT1	scratch homolog 1, zinc finger protein (Drosophila)	35	70	52.5
5090	PBX3	pre-B-cell leukemia transcription factor 3	34	71	52.5
10949	HNRPA0	heterogeneous nuclear ribonucleoprotein A0	31	74	52.5
10614	HEXIM1	hexamethylene bis-acetamide inducible 1	26	79	52.5
2941	GSTA4	glutathione S-transferase A4	25	80	52.5
1266	CNN3	calponin 3, acidic	12	93	52.5
4350	MPG	N-methylpurine-DNA glycosylase	0	105	52.5
58485	TRAPPC1	trafficking protein particle complex 1	0	105	52.5
123606	NIPA1	non imprinted in Prader-Willi/Angelman syndrome 1	101	3	52
339287	MSL-1	male-specific lethal-1 homolog	93	11	52

24139	EML2	echinoderm microtubule associated protein like 2	89	15	52
23144	ZC3H3	zinc finger CCCH-type containing 3	62	42	52
155066	ATP6V0E2	ATPase, H+ transporting V0 subunit e2	53	51	52
64769	C1orf149	chromosome 1 open reading frame 149	51	53	52
23120	ATP10B	ATPase, Class V, type 10B	49	55	52
10120	ACTR1B	ARP1 actin-related protein 1 homolog B, centractin beta (yea	48	56	52
58526	MID1IP1	MID1 interacting protein 1 (gastrulation specific G12 homolo	47	57	52
57693	ZNF317	zinc finger protein 317	39	65	52
10474	TADA3L	transcriptional adaptor 3 (NGG1 homolog, yeast)-like	37	67	52
55072	RNF31	ring finger protein 31	35	69	52
25913	POT1	POT1 protection of telomeres 1 homolog (S. pombe)	8	96	52
23218	NBEAL2	neurobeachin-like 2	4	100	52
4298	MLLT1	myeloid/lymphoid or mixed-lineage leukemia (trithorax homo	104	0	52
9645	MICAL2	microtubule associated monoxygenase, calponin and LIM dc	104	0	52
163050	ZNF564	zinc finger protein 564	96	7	51.5
3843	RANBP5	RAN binding protein 5	87	16	51.5
55540	IL17RB	interleukin 17 receptor B	75	28	51.5
1783	DYNC1LI2	dynein, cytoplasmic 1, light intermediate chain 2	68	35	51.5
5361	PLXNA1	plexin A1	60	43	51.5
23463	ICMT	isoprenylcysteine carboxyl methyltransferase	59	44	51.5
64342	HS1BP3	HCLS1 binding protein 3	49	54	51.5
81556	C15orf44	chromosome 15 open reading frame 44	21	82	51.5
91869	RFT1	RFT1 homolog (S. cerevisiae)	2	101	51.5
26995	TRUB2	TruB pseudouridine (psi) synthase homolog 2 (E. coli)	79	23	51
11177	BAZ1A	bromodomain adjacent to zinc finger domain, 1A	73	29	51
84878	ZBTB45	zinc finger and BTB domain containing 45	71	31	51
147004	LOC147004	hypothetical protein LOC147004	63	39	51
112464	PRKCDBP	protein kinase C, delta binding protein	56	46	51
1186	CLCN7	chloride channel 7	40	62	51
8099	CDK2AP1	CDK2-associated protein 1	17	85	51
22836	RHOBTB3	Rho-related BTB domain containing 3	10	92	51
6811	STX5	syntaxin 5	102	0	51
57162	PELI1	pellino homolog 1 (Drosophila)	93	8	50.5
125950	RAVER1	ribonucleoprotein, PTB-binding 1	93	8	50.5
9091	PIGQ	phosphatidylinositol glycan anchor biosynthesis, class Q	91	10	50.5
10319	LAMC3	laminin, gamma 3	82	19	50.5
121274	ZNF641	zinc finger protein 641	81	20	50.5
5058	PAK1	p21/Cdc42/Rac1-activated kinase 1 (STE20 homolog, yeast)	75	26	50.5
4035	LRP1	low density lipoprotein-related protein 1 (alpha-2-macroglobu	73	28	50.5
85378	TUBGCP6	tubulin, gamma complex associated protein 6	69	32	50.5
8844	KSR1	kinase suppressor of ras 1	67	34	50.5
57107	PDSS2	prenyl (decaprenyl) diphosphate synthase, subunit 2	61	40	50.5
9221	NOLC1	nucleolar and coiled-body phosphoprotein 1	61	40	50.5
80346	REEP4	receptor accessory protein 4	56	45	50.5
6415	SEPW1	selenoprotein W, 1	52	49	50.5
80724	ACAD10	acyl-Coenzyme A dehydrogenase family, member 10	45	56	50.5
56061	UBPH	ubiquitin-binding protein homolog	39	62	50.5
6942	TCF20	transcription factor 20 (AR1)	25	76	50.5
162989	DEDD2	death effector domain containing 2	19	82	50.5
84726	KIAA0515	KIAA0515	16	85	50.5
8490	RGS5	regulator of G-protein signalling 5	0	101	50.5

59	ACTA2	actin, alpha 2, smooth muscle, aorta	0	101	50.5
57542	KLHDC5	kelch domain containing 5	92	8	50
55505	NOLA3	nucleolar protein family A, member 3 (H/ACA small nucleolar	88	12	50
8266	UBL4A	ubiquitin-like 4A	79	21	50
11158	RABL2B	RAB, member of RAS oncogene family-like 2B	71	29	50
285590	SH3PXD2B	SH3 and PX domains 2B	64	36	50
150465	TTL	tubulin tyrosine ligase	63	37	50
84058	WDR54	WD repeat domain 54	53	47	50
6398	SECTM1	secreted and transmembrane 1	43	57	50
55620	STAP2	signal-transducing adaptor protein-2	40	60	50
5863	RGL2	ral guanine nucleotide dissociation stimulator-like 2	40	60	50
11041	B3GNT1	UDP-GlcNAc:betaGal beta-1,3-N-acetylglucosaminyltransfer	38	62	50
27090	ST6GALNAC	ST6 (alpha-N-acetyl-neuraminyl-2,3-beta-galactosyl-1,3)-N-a	2	98	50
55666	NPLOC4	nuclear protein localization 4 homolog (S. cerevisiae)	0	100	50
91289	TMEM153	transmembrane protein 153	100	0	50
51081	MRPS7	mitochondrial ribosomal protein S7	100	0	50
64746	ACBD3	acyl-Coenzyme A binding domain containing 3	77	22	49.5
55153	SDAD1	SDA1 domain containing 1	71	28	49.5
64118	DUS1L	dihydrouridine synthase 1-like (S. cerevisiae)	61	38	49.5
115294	PCMTD1	protein-L-isoaspartate (D-aspartate) O-methyltransferase do	45	54	49.5
57222	ERGIC1	endoplasmic reticulum-golgi intermediate compartment (ERG	41	58	49.5
51439	FAM8A1	family with sequence similarity 8, member A1	39	60	49.5
2975	GTF3C1	general transcription factor IIIC, polypeptide 1, alpha 220kDa	33	66	49.5
126792	B3GALT6	UDP-Gal:betaGal beta 1,3-galactosyltransferase polypeptide	31	68	49.5
60682	SMAP1	stromal membrane-associated protein 1	30	69	49.5
64745	METT11D1	methyltransferase 11 domain containing 1	25	74	49.5
6687	SPG7	spastic paraplegia 7, paraplegin (pure and complicated autos	21	78	49.5
26292	MYCBP	c-myc binding protein	17	82	49.5
8837	CFLAR	CASP8 and FADD-like apoptosis regulator	14	85	49.5
50512	PODXL2	podocalyxin-like 2	12	87	49.5
5316	PKNOX1	PBX/knotted 1 homeobox 1	4	95	49.5
63894	C14orf133	chromosome 14 open reading frame 133	99	0	49.5
9701	SAPS2	SAPS domain family, member 2	99	0	49.5
1827	DSCR1	Down syndrome critical region gene 1	99	0	49.5
3601	IL15RA	interleukin 15 receptor, alpha	81	17	49
9394	HS6ST1	heparan sulfate 6-O-sulfotransferase 1	81	17	49
26589	MRPL46	mitochondrial ribosomal protein L46	75	23	49
147700	KLC3	kinesin light chain 3	67	31	49
26469	PTPN18	protein tyrosine phosphatase, non-receptor type 18 (brain-de	67	31	49
7311	UBA52	ubiquitin A-52 residue ribosomal protein fusion product 1	67	31	49
84248	FYTTD1	forty-two-three domain containing 1	64	34	49
23013	SPEN	spen homolog, transcriptional regulator (Drosophila)	53	45	49
140885	SIRPA	signal-regulatory protein alpha	51	47	49
5152	PDE9A	phosphodiesterase 9A	49	49	49
4217	MAP3K5	mitogen-activated protein kinase kinase kinase 5	49	49	49
91734	IDI2	isopentenyl-diphosphate delta isomerase 2	28	70	49
158584	FAAH2	fatty acid amide hydrolase 2	20	78	49
9522	SCAMP1	secretory carrier membrane protein 1	10	88	49
9061	PAPSS1	3'-phosphoadenosine 5'-phosphosulfate synthase 1	10	88	49
2954	GSTZ1	glutathione transferase zeta 1 (maleylacetoacetate isomeras	0	98	49
5573	PRKAR1A	protein kinase, cAMP-dependent, regulatory, type I, alpha (ti	98	0	49

6095	RORA	RAR-related orphan receptor A	98	0	49
83862	TMPIT	transmembrane protein induced by tumor necrosis factor alp	98	0	49
55870	ASH1L	ash1 (absent, small, or homeotic)-like (Drosophila)	87	10	48.5
57528	KCTD16	potassium channel tetramerisation domain containing 16	87	10	48.5
126917	LOC126917	hypothetical protein LOC126917	87	10	48.5
3918	LAMC2	laminin, gamma 2	85	12	48.5
93082	LINCR	likely ortholog of mouse lung-inducible Neutralized-related C	81	16	48.5
55101	FLJ10241	hypothetical protein FLJ10241	52	45	48.5
93099	DMKN	dermokine	46	51	48.5
131408	C3orf40	chromosome 3 open reading frame 40	45	52	48.5
7088	TLE1	transducin-like enhancer of split 1 (E(sp1) homolog, Drosoph	42	55	48.5
8476	CDC42BPA	CDC42 binding protein kinase alpha (DMPK-like)	40	57	48.5
285381	DPH3	DPH3, KTI11 homolog (S. cerevisiae)	38	59	48.5
201254	STRA13	stimulated by retinoic acid 13 homolog (mouse)	35	62	48.5
23433	RHOQ	ras homolog gene family, member Q	32	65	48.5
6827	SUPT4H1	suppressor of Ty 4 homolog 1 (S. cerevisiae)	28	69	48.5
55037	PTCD3	Pentatricopeptide repeat domain 3	97	0	48.5
1182	CLCN3	chloride channel 3	97	0	48.5
257218	SHPRH	SNF2 histone linker PHD RING helicase	97	0	48.5
79026	AHNAK	AHNAK nucleoprotein (desmoyokin)	89	7	48
1845	DUSP3	dual specificity phosphatase 3 (vaccinia virus phosphatase V	79	17	48
307	ANXA4	annexin A4	72	24	48
60487	TRMT11	tRNA methyltransferase 11 homolog (S. cerevisiae)	57	39	48
7351	UCP2	uncoupling protein 2 (mitochondrial, proton carrier)	56	40	48
58513	EPS15L1	epidermal growth factor receptor pathway substrate 15-like 1	55	41	48
79574	EPS8L3	EPS8-like 3	31	65	48
255458	LOC255458	hypothetical protein LOC255458	25	71	48
2971	GTF3A	general transcription factor IIIA	24	72	48
7818	DAP3	death associated protein 3	23	73	48
55041	PLEKHB2	pleckstrin homology domain containing, family B (evectins) m	18	78	48
8703	B4GALT3	UDP-Gal:betaGlcNAc beta 1,4- galactosyltransferase, polype	17	79	48
353376	TICAM2	toll-like receptor adaptor molecule 2	16	80	48
56853	BRUNOL4	bruno-like 4, RNA binding protein (Drosophila)	8	88	48
9252	RPS6KA5	ribosomal protein S6 kinase, 90kDa, polypeptide 5	0	96	48
374655	ZNF710	zinc finger protein 710	90	5	47.5
148156	ZNF558	zinc finger protein 558	51	44	47.5
26100	WIPI2	WD repeat domain, phosphoinositide interacting 2	49	46	47.5
11021	RAB35	RAB35, member RAS oncogene family	49	46	47.5
56834	GPR137	G protein-coupled receptor 137	43	52	47.5
6051	RNPEP	arginyl aminopeptidase (aminopeptidase B)	41	54	47.5
25825	BACE2	beta-site APP-cleaving enzyme 2	40	55	47.5
4129	MAOB	monoamine oxidase B	35	60	47.5
755	C21orf2	chromosome 21 open reading frame 2	28	67	47.5
23224	SYNE2	spectrin repeat containing, nuclear envelope 2	27	68	47.5
29005	PRO1073	PRO1073 protein	19	76	47.5
120071	GYLTL1B	glycosyltransferase-like 1B	12	83	47.5
22838	RNF44	ring finger protein 44	12	83	47.5
55352	C17orf79	chromosome 17 open reading frame 79	10	85	47.5
989	SEP7	septin 7	4	91	47.5
1362	CPD	carboxypeptidase D	89	5	47
2033	EP300	E1A binding protein p300	73	21	47

57189	KIAA1147	KIAA1147	71	23	47
10477	UBE2E3	ubiquitin-conjugating enzyme E2E 3 (UBC4/5 homolog, yeas	65	29	47
400879	LOC400879	hypothetical gene supported by AK096951	65	29	47
56265	CPXM1	carboxypeptidase X (M14 family), member 1	61	33	47
5527	PPP2R5C	protein phosphatase 2, regulatory subunit B (B56), gamma is	60	34	47
12	SERPINA3	serpin peptidase inhibitor, clade A (alpha-1 antiproteinase, a	56	38	47
10556	RPP30	ribonuclease P/MRP 30kDa subunit	44	50	47
51759	C9orf78	chromosome 9 open reading frame 78	38	56	47
57630	SH3RF1	SH3 domain containing ring finger 1	30	64	47
55621	TRMT1	TRM1 tRNA methyltransferase 1 homolog (S. cerevisiae)	30	64	47
9612	NCOR2	nuclear receptor co-repressor 2	27	67	47
3075	CFH	complement factor H	24	70	47
338699	ANKRD42	ankyrin repeat domain 42	22	72	47
5978	REST	RE1-silencing transcription factor	6	88	47
55718	POLR3E	polymerase (RNA) III (DNA directed) polypeptide E (80kD)	73	20	46.5
80255	SLC35F5	solute carrier family 35, member F5	69	24	46.5
283578	TMED8	transmembrane emp24 protein transport domain containing 8	67	26	46.5
79685	SAP30L	SAP30-like	65	28	46.5
7107	GPR137B	G protein-coupled receptor 137B	59	34	46.5
6311	ATXN2	ataxin 2	48	45	46.5
57109	REXO4	REX4, RNA exonuclease 4 homolog (S. cerevisiae)	44	49	46.5
124925	SEZ6	seizure related 6 homolog (mouse)	39	54	46.5
79176	FBXL15	F-box and leucine-rich repeat protein 15	36	57	46.5
84669	USP32	ubiquitin specific peptidase 32	26	67	46.5
2132	EXT2	exostoses (multiple) 2	26	67	46.5
6156	RPL30	ribosomal protein L30	20	73	46.5
624	BDKRB2	bradykinin receptor B2	14	79	46.5
65979	PHACTR4	phosphatase and actin regulator 4	10	83	46.5
5480	PPIC	peptidylprolyl isomerase C (cyclophilin C)	0	93	46.5
26286	ARFGAP3	ADP-ribosylation factor GTPase activating protein 3	0	93	46.5
11161	C14orf1	chromosome 14 open reading frame 1	93	0	46.5
55652	FLJ20489	hypothetical protein FLJ20489	90	2	46
404734	MASK-BP3	MASK-4E-BP3 alternate reading frame gene	85	7	46
49855	ZNF291	zinc finger protein 291	81	11	46
541471	LOC541471	hypothetical LOC541471	75	17	46
25839	COG4	component of oligomeric golgi complex 4	54	38	46
25879	WDSOF1	WD repeats and SOF1 domain containing	44	48	46
2230	FDX1	ferredoxin 1	44	48	46
124220	LOC124220	similar to common salivary protein 1	43	49	46
1360	CPB1	carboxypeptidase B1 (tissue)	40	52	46
9678	PHF14	PHD finger protein 14	39	53	46
80019	UBTD1	ubiquitin domain containing 1	28	64	46
11329	STK38	serine/threonine kinase 38	22	70	46
112479	EXOD1	exonuclease domain containing 1	19	73	46
8614	STC2	stanniocalcin 2	14	78	46
3177	SLC29A2	solute carrier family 29 (nucleoside transporters), member 2	12	80	46
168544	ZNF467	zinc finger protein 467	12	80	46
22874	PLEKHA6	pleckstrin homology domain containing, family A member 6	4	88	46
6093	ROCK1	Rho-associated, coiled-coil containing protein kinase 1	2	90	46
25923	DKFZP564J(	DKFZP564J0863 protein	92	0	46
3895	KTN1	kinectin 1 (kinesin receptor)	82	9	45.5

57409	MIF4GD	MIF4G domain containing	63	28	45.5
5111	PCNA	proliferating cell nuclear antigen	63	28	45.5
54985	HCFC1R1	host cell factor C1 regulator 1 (XPO1 dependent)	63	28	45.5
9957	HS3ST1	heparan sulfate (glucosamine) 3-O-sulfotransferase 1	40	51	45.5
9244	CRLF1	cytokine receptor-like factor 1	40	51	45.5
6092	ROBO2	roundabout, axon guidance receptor, homolog 2 (Drosophila	37	54	45.5
11156	PTP4A3	protein tyrosine phosphatase type IVA, member 3	29	62	45.5
54946	SLC41A3	solute carrier family 41, member 3	26	65	45.5
8930	MBD4	methyl-CpG binding domain protein 4	24	67	45.5
7444	VRK2	vaccinia related kinase 2	24	67	45.5
9121	SLC16A5	solute carrier family 16, member 5 (monocarboxylic acid tran	18	73	45.5
79629	OCEL1	occludin/ELL domain containing 1	15	76	45.5
7536	SF1	splicing factor 1	4	87	45.5
84992	PIGY	phosphatidylinositol glycan anchor biosynthesis, class Y	0	91	45.5
2804	GOLGB1	golgi autoantigen, golgin subfamily b, macrogolgin (with trans	0	91	45.5
164633	CABP7	calcium binding protein 7	0	91	45.5
197258	FUK	fucokinase	0	91	45.5
10445	MCRS1	microspherule protein 1	91	0	45.5
442117	GALNT17	polypeptide N-acetylgalactosaminyltransferase 17	91	0	45.5
10435	CDC42EP2	CDC42 effector protein (Rho GTPase binding) 2	69	21	45
4060	LUM	lumican	65	25	45
6199	RPS6KB2	ribosomal protein S6 kinase, 70kDa, polypeptide 2	62	28	45
3682	ITGAE	integrin, alpha E (antigen CD103, human mucosal lymphocy	54	36	45
6880	TAF9	TAF9 RNA polymerase II, TATA box binding protein (TBP)-a	31	59	45
41	ACCN2	amiloride-sensitive cation channel 2, neuronal	27	63	45
3832	KIF11	kinesin family member 11	0	90	45
57579	KIAA1411	KIAA1411	87	2	44.5
148646	C1orf188	chromosome 1 open reading frame 188	63	26	44.5
57037	ANKMY2	ankyrin repeat and MYND domain containing 2	63	26	44.5
5245	PHB	prohibitin	61	28	44.5
2051	EPHB6	EPH receptor B6	55	34	44.5
83985	SPIN1	spinster	53	36	44.5
7249	TSC2	tuberous sclerosis 2	51	38	44.5
7048	TGFBR2	transforming growth factor, beta receptor II (70/80kDa)	44	45	44.5
55129	TMEM16K	transmembrane protein 16K	43	46	44.5
64778	FNDC3B	fibronectin type III domain containing 3B	31	58	44.5
1997	ELF1	E74-like factor 1 (ets domain transcription factor)	26	63	44.5
81576	CCDC130	coiled-coil domain containing 130	16	73	44.5
8731	RNMT	RNA (guanine-7-) methyltransferase	12	77	44.5
6596	HLTF	helicase-like transcription factor	10	79	44.5
3486	IGFBP3	insulin-like growth factor binding protein 3	0	89	44.5
54856	GON4L	gon-4-like (C. elegans)	81	7	44
23294	ANKS1A	ankyrin repeat and sterile alpha motif domain containing 1A	81	7	44
94031	HTRA3	HtrA serine peptidase 3	77	11	44
5095	PCCA	propionyl Coenzyme A carboxylase, alpha polypeptide	73	15	44
348180	LOC348180	hypothetical protein LOC348180, isoform 1	67	21	44
54498	SMOX	spermine oxidase	61	27	44
55170	PRMT6	protein arginine methyltransferase 6	55	33	44
2014	EMP3	epithelial membrane protein 3	54	34	44
729597	LOC729597	similar to Williams Beuren syndrome chromosome region 19	48	40	44
91663	MYADM	myeloid-associated differentiation marker	46	42	44

3476	IGBP1	immunoglobulin (CD79A) binding protein 1	41	47	44
64073	C19orf33	chromosome 19 open reading frame 33	41	47	44
1666	DECR1	2,4-dienoyl CoA reductase 1, mitochondrial	31	57	44
6651	SON	SON DNA binding protein	16	72	44
9702	CEP57	centrosomal protein 57kDa	12	76	44
51389	RWDD1	RWD domain containing 1	10	78	44
1875	E2F5	E2F transcription factor 5, p130-binding	0	88	44
30836	DNTTIP2	deoxynucleotidyltransferase, terminal, interacting protein 2	0	88	44
3985	LIMK2	LIM domain kinase 2	0	88	44
3119	HLA-DQB1	major histocompatibility complex, class II, DQ beta 1	88	0	44
200844	FLJ42117	FLJ42117 protein	73	14	43.5
1652	DDT	D-dopachrome tautomerase	65	22	43.5
1066	CES1	carboxylesterase 1 (monocyte/macrophage serine esterase	63	24	43.5
10483	SEC23B	Sec23 homolog B (S. cerevisiae)	61	26	43.5
26205	GMEB2	glucocorticoid modulatory element binding protein 2	59	28	43.5
153561	GUSBP1	glucuronidase, beta pseudogene 1	53	34	43.5
831	CAST	calpastatin	52	35	43.5
9728	KIAA0256	KIAA0256 gene product	43	44	43.5
773	CACNA1A	calcium channel, voltage-dependent, P/Q type, alpha 1A sub	35	52	43.5
56940	DUSP22	dual specificity phosphatase 22	35	52	43.5
55227	LRRC1	leucine rich repeat containing 1	28	59	43.5
150223	LOC150223	hypothetical protein LOC150223	22	65	43.5
26523	EIF2C1	eukaryotic translation initiation factor 2C, 1	18	69	43.5
1965	EIF2S1	eukaryotic translation initiation factor 2, subunit 1 alpha, 35kI	16	71	43.5
55662	HIF1AN	hypoxia-inducible factor 1, alpha subunit inhibitor	14	73	43.5
374875	HSD11B1L	hydroxysteroid (11-beta) dehydrogenase 1-like	14	73	43.5
51523	CXXC5	CXXC finger 5	14	73	43.5
29995	LMCD1	LIM and cysteine-rich domains 1	4	83	43.5
51540	SCLY	selenocysteine lyase	4	83	43.5
57595	PDZD4	PDZ domain containing 4	2	85	43.5
390010	NKX1-2	NK1 transcription factor related, locus 2 (Drosophila)	0	87	43.5
8542	APOL1	apolipoprotein L, 1	0	87	43.5
9344	TAOK2	TAO kinase 2	0	87	43.5
54439	RBM27	RNA binding motif protein 27	87	0	43.5
5519	PPP2R1B	protein phosphatase 2 (formerly 2A), regulatory subunit A (P	83	3	43
83732	RIOK1	RIO kinase 1 (yeast)	79	7	43
4651	MYO10	myosin X	73	13	43
7366	UGT2B15	UDP glucuronosyltransferase 2 family, polypeptide B15	69	17	43
10417	SPON2	spondin 2, extracellular matrix protein	67	19	43
124402	FAM100A	family with sequence similarity 100, member A	58	28	43
4097	MAFG	v-maf musculoaponeurotic fibrosarcoma oncogene homolog	38	48	43
3651	PDX1	pancreatic and duodenal homeobox 1	38	48	43
27043	PELP1	proline, glutamic acid and leucine rich protein 1	38	48	43
5525	PPP2R5A	protein phosphatase 2, regulatory subunit B (B56), alpha iso	37	49	43
5210	PFKFB4	6-phosphofructo-2-kinase/fructose-2,6-biphosphatase 4	21	65	43
55182	C1orf164	chromosome 1 open reading frame 164	16	70	43
10061	ABCF2	ATP-binding cassette, sub-family F (GCN20), member 2	10	76	43
7073	TIAL1	TIA1 cytotoxic granule-associated RNA binding protein-like 1	86	0	43
51043	ZBTB7B	zinc finger and BTB domain containing 7B	86	0	43
64794	DDX31	DEAD (Asp-Glu-Ala-Asp) box polypeptide 31	86	0	43
81559	TRIM11	tripartite motif-containing 11	77	8	42.5

140680	C20orf96	chromosome 20 open reading frame 96	77	8	42.5
339122	RAB43	RAB43, member RAS oncogene family	71	14	42.5
191	AHCY	S-adenosylhomocysteine hydrolase	69	16	42.5
51026	GOLT1B	golgi transport 1 homolog B (S. cerevisiae)	59	26	42.5
60678	EEFSEC	eukaryotic elongation factor, selenocysteine-tRNA-specific	51	34	42.5
92912	UBE2Q2	ubiquitin-conjugating enzyme E2Q (putative) 2	49	36	42.5
80198	MUS81	MUS81 endonuclease homolog (S. cerevisiae)	49	36	42.5
84299	C17orf37	chromosome 17 open reading frame 37	48	37	42.5
59307	SIGIRR	single immunoglobulin and toll-interleukin 1 receptor (TIR) do	46	39	42.5
64087	MCCC2	methylcrotonoyl-Coenzyme A carboxylase 2 (beta)	45	40	42.5
3162	HMOX1	heme oxygenase (decycling) 1	42	43	42.5
402055	CTB-1048E9	similar to SRR1-like protein	28	57	42.5
9846	GAB2	GRB2-associated binding protein 2	26	59	42.5
8884	SLC5A6	solute carrier family 5 (sodium-dependent vitamin transporte	18	67	42.5
8553	BHLHB2	basic helix-loop-helix domain containing, class B, 2	18	67	42.5
55325	C4orf20	chromosome 4 open reading frame 20	14	71	42.5
26099	C1orf144	chromosome 1 open reading frame 144	8	77	42.5
84337	ELOF1	elongation factor 1 homolog (S. cerevisiae)	0	85	42.5
55191	NADSYN1	NAD synthetase 1	0	85	42.5
79719	FLJ11506	hypothetical protein FLJ11506	0	85	42.5
8519	IFITM1	interferon induced transmembrane protein 1 (9-27)	85	0	42.5
84300	C6orf125	chromosome 6 open reading frame 125	85	0	42.5
125150	SWS1	SWIM-domain containing Srs2 interacting protein 1	81	3	42
23598	PATZ1	POZ (BTB) and AT hook containing zinc finger 1	79	5	42
55661	DDX27	DEAD (Asp-Glu-Ala-Asp) box polypeptide 27	77	7	42
54836	BSPRY	B-box and SPRY domain containing	75	9	42
51076	CUTC	cutC copper transporter homolog (E. coli)	69	15	42
28566	TRBV21-1	T cell receptor beta variable 21-1	65	19	42
6523	SLC5A1	solute carrier family 5 (sodium/glucose cotransporter), memb	61	23	42
23504	RIMBP2	RIMS binding protein 2	58	26	42
5170	PDPK1	3-phosphoinositide dependent protein kinase-1	55	29	42
6907	TBL1X	transducin (beta)-like 1X-linked	45	39	42
9191	DEDD	death effector domain containing	42	42	42
64847	SPATA20	spermatogenesis associated 20	39	45	42
1522	CTSZ	cathepsin Z	39	45	42
9736	USP34	ubiquitin specific peptidase 34	38	46	42
84129	ACAD11	acyl-Coenzyme A dehydrogenase family, member 11	35	49	42
10105	PPIF	peptidylprolyl isomerase F (cyclophilin F)	20	64	42
64207	C14orf4	chromosome 14 open reading frame 4	20	64	42
162	AP1B1	adaptor-related protein complex 1, beta 1 subunit	10	74	42
7403	UTX	ubiquitously transcribed tetratricopeptide repeat, X chromoso	10	74	42
10298	PAK4	p21(CDKN1A)-activated kinase 4	4	80	42
283383	GPR133	G protein-coupled receptor 133	2	82	42
84265	POLR3GL	polymerase (RNA) III (DNA directed) polypeptide G (32kD)-li	0	84	42
5768	QSCN6	quiescin Q6	0	84	42
117178	SSX2IP	synovial sarcoma, X breakpoint 2 interacting protein	81	2	41.5
84818	IL17RC	interleukin 17 receptor C	78	5	41.5
6059	ABCE1	ATP-binding cassette, sub-family E (OABP), member 1	67	16	41.5
25939	SAMHD1	SAM domain and HD domain 1	65	18	41.5
55727	BTBD7	BTB (POZ) domain containing 7	64	19	41.5
27346	TMEM97	transmembrane protein 97	59	24	41.5

80233	C17orf70	chromosome 17 open reading frame 70	45	38	41.5
23557	SNAPAP	SNAP-associated protein	41	42	41.5
55677	IWS1	IWS1 homolog (S. cerevisiae)	40	43	41.5
1937	EEF1G	eukaryotic translation elongation factor 1 gamma	35	48	41.5
22879	MON1B	MON1 homolog B (yeast)	14	69	41.5
256691	MAMDC2	MAM domain containing 2	0	83	41.5
1616	DAXX	death-associated protein 6	0	83	41.5
80728	KIAA1688	KIAA1688 protein	83	0	41.5
56683	C21orf59	chromosome 21 open reading frame 59	83	0	41.5
3171	FOXA3	forkhead box A3	83	0	41.5
10307	APBB3	amyloid beta (A4) precursor protein-binding, family B, memb	83	0	41.5
128710	C20orf94	chromosome 20 open reading frame 94	77	5	41
6928	TCF2	transcription factor 2, hepatic; LF-B3; variant hepatic nuclear	76	6	41
203286	ANKS6	ankyrin repeat and sterile alpha motif domain containing 6	73	9	41
6257	RXRB	retinoid X receptor, beta	63	19	41
149473	CCDC24	coiled-coil domain containing 24	61	21	41
51527	C14orf129	chromosome 14 open reading frame 129	53	29	41
9918	NCAPD2	non-SMC condensin I complex, subunit D2	51	31	41
5413	SEP5	septin 5	51	31	41
84319	C3orf26	chromosome 3 open reading frame 26	50	32	41
4089	SMAD4	SMAD family member 4	46	36	41
7905	REEP5	receptor accessory protein 5	46	36	41
80344	WDR23	WD repeat domain 23	36	46	41
9703	KIAA0100	KIAA0100	32	50	41
25927	C2orf32	chromosome 2 open reading frame 32	31	51	41
84286	TMEM175	transmembrane protein 175	29	53	41
29966	STRN3	striatin, calmodulin binding protein 3	19	63	41
49856	WDR8	WD repeat domain 8	17	65	41
10713	USP39	ubiquitin specific peptidase 39	14	68	41
10083	USH1C	Usher syndrome 1C (autosomal recessive, severe)	10	72	41
55030	FBXO34	F-box protein 34	4	78	41
23767	FLRT3	fibronectin leucine rich transmembrane protein 3	2	80	41
9814	SFI1	Sfi1 homolog, spindle assembly associated (yeast)	2	80	41
8120	AP3B2	adaptor-related protein complex 3, beta 2 subunit	0	82	41
84056	KATNAL1	katanin p60 subunit A-like 1	0	82	41
145757	LOC145757	hypothetical protein LOC145757	0	82	41
1036	CDO1	cysteine dioxygenase, type I	0	82	41
400	ARL1	ADP-ribosylation factor-like 1	0	82	41
5606	MAP2K3	mitogen-activated protein kinase kinase 3	82	0	41
84451	KIAA1804	mixed lineage kinase 4	73	8	40.5
9605	C16orf7	chromosome 16 open reading frame 7	71	10	40.5
326625	MMAB	methylmalonic aciduria (cobalamin deficiency) cblB type	63	18	40.5
388403	YPEL2	yippee-like 2 (Drosophila)	52	29	40.5
152687	ZNF595	zinc finger protein 595	43	38	40.5
11046	SLC35D2	solute carrier family 35, member D2	29	52	40.5
142678	MIB2	mindbomb homolog 2 (Drosophila)	25	56	40.5
5306	PITPNA	phosphatidylinositol transfer protein, alpha	16	65	40.5
51228	GLTP	glycolipid transfer protein	12	69	40.5
2564	GABRE	gamma-aminobutyric acid (GABA) A receptor, epsilon	8	73	40.5
169792	GLIS3	GLIS family zinc finger 3	6	75	40.5
129684	CNTNAP5	contactin associated protein-like 5	0	81	40.5

6242	RTKN	rhotekin	81	0	40.5
79677	SMC6	structural maintenance of chromosomes 6	81	0	40.5
10592	SMC2	structural maintenance of chromosomes 2	77	3	40
54436	SH3TC1	SH3 domain and tetratricopeptide repeats 1	77	3	40
1346	COX7A1	cytochrome c oxidase subunit VIIa polypeptide 1 (muscle)	73	7	40
57533	TBC1D14	TBC1 domain family, member 14	72	8	40
6924	TCEB3	transcription elongation factor B (SIII), polypeptide 3 (110kDa	67	13	40
9325	TRIP4	thyroid hormone receptor interactor 4	63	17	40
55788	LMBRD1	LMBR1 domain containing 1	49	31	40
64219	PJA1	praja 1	49	31	40
57798	GATAD1	GATA zinc finger domain containing 1	40	40	40
55119	PRPF38B	PRP38 pre-mRNA processing factor 38 (yeast) domain conta	40	40	40
1999	ELF3	E74-like factor 3 (ets domain transcription factor, epithelial-s	39	41	40
9463	PICK1	protein interacting with PRKCA 1	35	45	40
55070	DET1	de-etiolated homolog 1 (Arabidopsis)	26	54	40
7705	ZNF146	zinc finger protein 146	17	63	40
55715	DOK4	docking protein 4	16	64	40
80772	MGC10334	hypothetical protein MGC10334	16	64	40
23413	FREQ	frequenin homolog (Drosophila)	10	70	40
339123	LOC339123	hypothetical LOC339123	8	72	40
55269	PSPC1	paraspeckle component 1	2	78	40
1857	DVL3	dishevelled, dsh homolog 3 (Drosophila)	0	80	40
51069	MRPL2	mitochondrial ribosomal protein L2	80	0	40
6867	TACC1	transforming, acidic coiled-coil containing protein 1	73	6	39.5
51490	C9orf114	chromosome 9 open reading frame 114	71	8	39.5
4756	NEO1	neogenin homolog 1 (chicken)	69	10	39.5
4848	CNOT2	CCR4-NOT transcription complex, subunit 2	69	10	39.5
5160	PDHA1	pyruvate dehydrogenase (lipoamide) alpha 1	69	10	39.5
64175	LEPRE1	leucine proline-enriched proteoglycan (leprecan) 1	67	12	39.5
7709	ZBTB17	zinc finger and BTB domain containing 17	59	20	39.5
54859	TMEM103	transmembrane protein 103	56	23	39.5
23592	LEMD3	LEM domain containing 3	54	25	39.5
1039	CDR2	cerebellar degeneration-related protein 2, 62kDa	48	31	39.5
55714	ODZ3	odz, odd Oz/ten-m homolog 3 (Drosophila)	48	31	39.5
9169	SFRS2IP	splicing factor, arginine/serine-rich 2, interacting protein	47	32	39.5
57507	ZNF608	zinc finger protein 608	42	37	39.5
57665	RDH14	retinol dehydrogenase 14 (all-trans/9-cis/11-cis)	40	39	39.5
662	BNIP1	BCL2/adenovirus E1B 19kDa interacting protein 1	36	43	39.5
65996	MGC2752	hypothetical protein MGC2752	34	45	39.5
9104	RGN	regucalcin (senescence marker protein-30)	32	47	39.5
7469	WHSC2	Wolf-Hirschhorn syndrome candidate 2	31	48	39.5
11097	NUPL2	nucleoporin like 2	30	49	39.5
131566	DCBLD2	discoidin, CUB and LCCL domain containing 2	30	49	39.5
54806	AHI1	Abelson helper integration site 1	26	53	39.5
10139	ARFRP1	ADP-ribosylation factor related protein 1	23	56	39.5
8507	ENC1	ectodermal-neural cortex (with BTB-like domain)	20	59	39.5
84950	PRPF38A	PRP38 pre-mRNA processing factor 38 (yeast) domain conta	4	75	39.5
5601	MAPK9	mitogen-activated protein kinase 9	2	77	39.5
51626	DYNC2LI1	dynein, cytoplasmic 2, light intermediate chain 1	0	79	39.5
9620	CELSR1	cadherin, EGF LAG seven-pass G-type receptor 1 (flamingo	0	79	39.5
2079	ERH	enhancer of rudimentary homolog (Drosophila)	0	79	39.5

23060	ZNF609	zinc finger protein 609	79	0	39.5
654029	LOC654029	similar to 40S ribosomal protein S10	79	0	39.5
8897	MTMR3	myotubularin related protein 3	72	6	39
10785	WDR4	WD repeat domain 4	71	7	39
8842	PROM1	prominin 1	67	11	39
261726	TIPRL	TIP41, TOR signalling pathway regulator-like (S. cerevisiae)	67	11	39
4695	NDUFA2	NADH dehydrogenase (ubiquinone) 1 alpha subcomplex, 2,	61	17	39
7389	UROD	uroporphyrinogen decarboxylase	58	20	39
8453	CUL2	cullin 2	58	20	39
4207	MEF2B	MADS box transcription enhancer factor 2, polypeptide B (m	57	21	39
55100	WDR70	WD repeat domain 70	55	23	39
27436	EML4	echinoderm microtubule associated protein like 4	53	25	39
490	ATP2B1	ATPase, Ca++ transporting, plasma membrane 1	41	37	39
4331	MNAT1	menage a trois homolog 1, cyclin H assembly factor (Xenopu	39	39	39
152006	RNF38	ring finger protein 38	37	41	39
64326	RFWD2	ring finger and WD repeat domain 2	37	41	39
3178	HNRPA1	heterogeneous nuclear ribonucleoprotein A1	37	41	39
22944	KIN	KIN, antigenic determinant of recA protein homolog (mouse)	29	49	39
80301	PLEKHQ1	pleckstrin homology domain containing, family Q member 1	26	52	39
26011	ODZ4	odz, odd Oz/ten-m homolog 4 (Drosophila)	24	54	39
130557	ZNF513	zinc finger protein 513	15	63	39
4760	NEUROD1	neurogenic differentiation 1	12	66	39
147965	FAM98C	family with sequence similarity 98, member C	4	74	39
1427	CRYGS	crystallin, gamma S	0	78	39
10605	PAIP1	poly(A) binding protein interacting protein 1	0	78	39
6129	RPL7	ribosomal protein L7	0	78	39
29881	NPC1L1	NPC1 (Niemann-Pick disease, type C1, gene)-like 1	0	78	39
9637	FEZ2	fasciculation and elongation protein zeta 2 (zygin II)	78	0	39
4170	MCL1	myeloid cell leukemia sequence 1 (BCL2-related)	72	5	38.5
8634	RTCD1	RNA terminal phosphate cyclase domain 1	67	10	38.5
2744	GLS	glutaminase	67	10	38.5
7072	TIA1	TIA1 cytotoxic granule-associated RNA binding protein	62	15	38.5
286343	C9orf150	chromosome 9 open reading frame 150	46	31	38.5
3693	ITGB5	integrin, beta 5	43	34	38.5
10283	SDCCAG10	serologically defined colon cancer antigen 10	40	37	38.5
1948	EFNB2	ephrin-B2	26	51	38.5
55701	FLJ10357	hypothetical protein FLJ10357	19	58	38.5
892	CCNC	cyclin C	17	60	38.5
4128	MAOA	monoamine oxidase A	6	71	38.5
79934	ADCK4	aarF domain containing kinase 4	0	77	38.5
1306	COL15A1	collagen, type XV, alpha 1	0	77	38.5
65992	C20orf116	chromosome 20 open reading frame 116	0	77	38.5
23350	SR140	U2-associated SR140 protein	0	77	38.5
5797	PTPRM	protein tyrosine phosphatase, receptor type, M	0	77	38.5
4050	LTB	lymphotoxin beta (TNF superfamily, member 3)	77	0	38.5
84991	RBM17	RNA binding motif protein 17	77	0	38.5
84364	ZNF289	zinc finger protein 289, ID1 regulated	65	11	38
158	ADSL	adenylosuccinate lyase	49	27	38
56924	PAK6	p21(CDKN1A)-activated kinase 6	47	29	38
9581	PREPL	prolyl endopeptidase-like	45	31	38
23187	PHLDB1	pleckstrin homology-like domain, family B, member 1	45	31	38

112	ADCY6	adenylate cyclase 6	43	33	38
57698	KIAA1598	KIAA1598	40	36	38
6385	SDC4	syndecan 4 (amphiglycan, ryudocan)	33	43	38
55076	TMEM45A	transmembrane protein 45A	30	46	38
23677	SH3BP4	SH3-domain binding protein 4	30	46	38
7260	TSSC1	tumor suppressing subtransferable candidate 1	25	51	38
55784	MCTP2	multiple C2 domains, transmembrane 2	14	62	38
4790	NFKB1	nuclear factor of kappa light polypeptide gene enhancer in B	8	68	38
2115	ETV1	ets variant gene 1	2	74	38
56941	C3orf37	chromosome 3 open reading frame 37	0	76	38
3337	DNAJB1	DnaJ (Hsp40) homolog, subfamily B, member 1	0	76	38
79684	C11orf61	chromosome 11 open reading frame 61	76	0	38
9520	NPEPPS	aminopeptidase puromycin sensitive	65	10	37.5
5971	RELB	v-rel reticuloendotheliosis viral oncogene homolog B, nuclea	57	18	37.5
9861	PSMD6	proteasome (prosome, macropain) 26S subunit, non-ATPase	52	23	37.5
55286	C4orf19	chromosome 4 open reading frame 19	40	35	37.5
8195	MKKS	McKusick-Kaufman syndrome	36	39	37.5
23379	KIAA0947	KIAA0947 protein	27	48	37.5
5193	PEX12	peroxisomal biogenesis factor 12	27	48	37.5
50999	TMED5	transmembrane emp24 protein transport domain containing 5	26	49	37.5
10946	SF3A3	splicing factor 3a, subunit 3, 60kDa	23	52	37.5
2776	GNAQ	guanine nucleotide binding protein (G protein), q polypeptide	22	53	37.5
25912	C1orf43	chromosome 1 open reading frame 43	21	54	37.5
2353	FOS	v-fos FBJ murine osteosarcoma viral oncogene homolog	18	57	37.5
6120	RPE	ribulose-5-phosphate-3-epimerase	15	60	37.5
6141	RPL18	ribosomal protein L18	10	65	37.5
4149	MAX	MYC associated factor X	6	69	37.5
659	BMPR2	bone morphogenetic protein receptor, type II (serine/threonir	0	75	37.5
5141	PDE4A	phosphodiesterase 4A, cAMP-specific (phosphodiesterase E	75	0	37.5
54707	ATPBD1B	ATP binding domain 1 family, member B	75	0	37.5
6879	TAF7	TAF7 RNA polymerase II, TATA box binding protein (TBP)-a	75	0	37.5
84909	C9orf3	chromosome 9 open reading frame 3	69	5	37
6675	UAP1	UDP-N-acteylglucosamine pyrophosphorylase 1	67	7	37
126119	JOSD2	Josephin domain containing 2	59	15	37
11068	CYB561D2	cytochrome b-561 domain containing 2	51	23	37
28977	MRPL42	mitochondrial ribosomal protein L42	50	24	37
254531	AGPAT7	1-acylglycerol-3-phosphate O-acyltransferase 7 (lysophosph	45	29	37
7127	TNFAIP2	tumor necrosis factor, alpha-induced protein 2	43	31	37
51569	UFM1	ubiquitin-fold modifier 1	42	32	37
23233	EXOC6B	exocyst complex component 6B	41	33	37
5912	RAP2B	RAP2B, member of RAS oncogene family	41	33	37
89801	PPP1R3F	protein phosphatase 1, regulatory (inhibitor) subunit 3F	40	34	37
54969	C4orf27	chromosome 4 open reading frame 27	38	36	37
10153	CEBPZ	CCAAT/enhancer binding protein zeta	36	38	37
78996	C7orf49	chromosome 7 open reading frame 49	35	39	37
1355	COX15	COX15 homolog, cytochrome c oxidase assembly protein (y	35	39	37
59274	MESDC1	mesoderm development candidate 1	33	41	37
1736	DKC1	dyskeratosis congenita 1, dyskerin	32	42	37
784	CACNB3	calcium channel, voltage-dependent, beta 3 subunit	30	44	37
2629	GBA	glucosidase, beta; acid (includes glucosylceramidase)	30	44	37
9354	UBE4A	ubiquitination factor E4A (UFD2 homolog, yeast)	27	47	37

57184	C15orf17	chromosome 15 open reading frame 17	27	47	37
29085	PHPT1	phosphohistidine phosphatase 1	20	54	37
3104	ZBTB48	zinc finger and BTB domain containing 48	20	54	37
7264	TSTA3	tissue specific transplantation antigen P35B	20	54	37
57459	GATAD2B	GATA zinc finger domain containing 2B	16	58	37
387496	RASL11A	RAS-like, family 11, member A	12	62	37
51668	C1orf41	chromosome 1 open reading frame 41	6	68	37
57610	RANBP10	RAN binding protein 10	4	70	37
2309	FOXO3A	forkhead box O3A	0	74	37
27248	C2orf30	chromosome 2 open reading frame 30	74	0	37
114599	SNORD15B	small nucleolar RNA, C/D box 15B	74	0	37
4240	MFGE8	milk fat globule-EGF factor 8 protein	71	2	36.5
6612	SUMO3	SMT3 suppressor of mif two 3 homolog 3 (S. cerevisiae)	62	11	36.5
149076	FLJ25476	FLJ25476 protein	53	20	36.5
80331	DNAJC5	DnaJ (Hsp40) homolog, subfamily C, member 5	51	22	36.5
5420	PODXL	podocalyxin-like	44	29	36.5
348174	LOC348174	secretory protein LOC348174	35	38	36.5
3133	HLA-E	major histocompatibility complex, class I, E	26	47	36.5
5252	PHF1	PHD finger protein 1	25	48	36.5
55504	TNFRSF19	tumor necrosis factor receptor superfamily, member 19	16	57	36.5
10241	CALCOCO2	calcium binding and coiled-coil domain 2	10	63	36.5
79188	TMEM43	transmembrane protein 43	6	67	36.5
125	ADH1B	alcohol dehydrogenase IB (class I), beta polypeptide	2	71	36.5
4319	MMP10	matrix metallopeptidase 10 (stromelysin 2)	0	73	36.5
896	CCND3	cyclin D3	0	73	36.5
84619	ZGPAT	zinc finger, CCCH-type with G patch domain	0	73	36.5
79885	HDAC11	histone deacetylase 11	0	73	36.5
9711	KIAA0226	KIAA0226	0	73	36.5
200185	KRTCAP2	keratinocyte associated protein 2	0	73	36.5
1635	DCTD	dCMP deaminase	0	73	36.5
2356	FPGS	folylpolyglutamate synthase	73	0	36.5
692312	PPAN-P2RY	PPAN-P2RY11	73	0	36.5
112752	C14orf179	chromosome 14 open reading frame 179	69	3	36
4820	NKTR	natural killer-tumor recognition sequence	69	3	36
5883	RAD9A	RAD9 homolog A (S. pombe)	67	5	36
9870	KIAA0317	KIAA0317	65	7	36
8233	ZRSR2	zinc finger (CCCH type), RNA-binding motif and serine/argin	65	7	36
84279	C2orf7	chromosome 2 open reading frame 7	58	14	36
284598	FLJ39609	hypothetical protein FLJ39609	55	17	36
2888	GRB14	growth factor receptor-bound protein 14	52	20	36
57468	SLC12A5	solute carrier family 12, (potassium-chloride transporter) mer	47	25	36
5106	PCK2	phosphoenolpyruvate carboxykinase 2 (mitochondrial)	46	26	36
9762	ProSAPiP1	ProSAPiP1 protein	40	32	36
6324	SCN1B	sodium channel, voltage-gated, type I, beta	35	37	36
207063	DHRSX	dehydrogenase/reductase (SDR family) X-linked	32	40	36
22820	COPG	coatomer protein complex, subunit gamma	27	45	36
6710	SPTB	spectrin, beta, erythrocytic (includes spherocytosis, clinical ty	26	46	36
54708	MAR5	membrane-associated ring finger (C3HC4) 5	23	49	36
11284	PNKP	polynucleotide kinase 3'-phosphatase	23	49	36
29072	SETD2	SET domain containing 2	10	62	36
9166	EBAG9	estrogen receptor binding site associated, antigen, 9	6	66	36

23034	SAMD4A	sterile alpha motif domain containing 4A	4	68	36
8540	AGPS	alkylglycerone phosphate synthase	2	70	36
79048	SECISBP2	SECIS binding protein 2	0	72	36
27072	VPS41	vacuolar protein sorting 41 homolog (S. cerevisiae)	0	72	36
863	CBFA2T3	core-binding factor, runt domain, alpha subunit 2; translocate	0	72	36
55722	CEP72	centrosomal protein 72kDa	69	2	35.5
642290	LOC642290	similar to Tricarboxylate transport protein, mitochondrial prec	58	13	35.5
51144	HSD17B12	hydroxysteroid (17-beta) dehydrogenase 12	56	15	35.5
54496	PRMT7	protein arginine methyltransferase 7	53	18	35.5
85456	TNKS1BP1	tankyrase 1 binding protein 1, 182kDa	51	20	35.5
23588	KLHDC2	kelch domain containing 2	46	25	35.5
79763	ISOC2	isochorismatase domain containing 2	45	26	35.5
128338	TMEM77	transmembrane protein 77	40	31	35.5
5954	RCN1	reticulocalbin 1, EF-hand calcium binding domain	38	33	35.5
56910	STARD7	START domain containing 7	35	36	35.5
133	ADM	adrenomedullin	35	36	35.5
57555	NLGN2	neuroligin 2	35	36	35.5
9993	DGCR2	DiGeorge syndrome critical region gene 2	30	41	35.5
605	BCL7A	B-cell CLL/lymphoma 7A	27	44	35.5
9334	B4GALT5	UDP-Gal:betaGlcNAc beta 1,4- galactosyltransferase, polype	24	47	35.5
9175	MAP3K13	mitogen-activated protein kinase kinase kinase 13	22	49	35.5
57580	PREX1	phosphatidylinositol 3,4,5-trisphosphate-dependent RAC exc	22	49	35.5
4205	MEF2A	MADS box transcription enhancer factor 2, polypeptide A (m	16	55	35.5
55093	C8orf32	chromosome 8 open reading frame 32	15	56	35.5
894	CCND2	cyclin D2	15	56	35.5
79666	PLEKHF2	pleckstrin homology domain containing, family F (with FYVE	8	63	35.5
10978	CLP1	CLP1, cleavage and polyadenylation factor I subunit, homolo	6	65	35.5
4953	ODC1	ornithine decarboxylase 1	6	65	35.5
5805	PTS	6-pyruvoyltetrahydropterin synthase	2	69	35.5
1806	DPYD	dihydropyrimidine dehydrogenase	2	69	35.5
22827	SIAHBP1	fuse-binding protein-interacting repressor	0	71	35.5
135892	TRIM50	tripartite motif-containing 50	0	71	35.5
10908	PNPLA6	patatin-like phospholipase domain containing 6	0	71	35.5
1601	DAB2	disabled homolog 2, mitogen-responsive phosphoprotein (Dr	0	71	35.5
440193	KIAA1509	KIAA1509	0	71	35.5
9118	INA	internexin neuronal intermediate filament protein, alpha	0	71	35.5
375790	AGRIN	agrin	0	71	35.5
51657	STYXL1	serine/threonine/tyrosine interacting-like 1	71	0	35.5
23589	CARHSP1	calcium regulated heat stable protein 1, 24kDa	71	0	35.5
51019	CCDC53	coiled-coil domain containing 53	71	0	35.5
1408	CRY2	cryptochrome 2 (photolyase-like)	71	0	35.5
6838	SURF6	surfeit 6	71	0	35.5
10725	NFAT5	nuclear factor of activated T-cells 5, tonicity-responsive	71	0	35.5
8148	TAF15	TAF15 RNA polymerase II, TATA box binding protein (TBP)-	71	0	35.5
93643	TJAP1	tight junction associated protein 1 (peripheral)	71	0	35.5
89797	NAV2	neuron navigator 2	67	3	35
23741	EID1	EP300 interacting inhibitor of differentiation 1	63	7	35
29116	MYLIP	myosin regulatory light chain interacting protein	59	11	35
29775	CARD10	caspase recruitment domain family, member 10	58	12	35
3373	HYAL1	hyaluronoglucosaminidase 1	48	22	35
84231	TRAF7	TNF receptor-associated factor 7	47	23	35

80148	PQLC1	PQ loop repeat containing 1	41	29	35
23513	SCRIB	scribbled homolog (Drosophila)	39	31	35
138716	C9orf23	chromosome 9 open reading frame 23	36	34	35
79590	MRPL24	mitochondrial ribosomal protein L24	27	43	35
84513	PPAPDC1B	phosphatidic acid phosphatase type 2 domain containing 1B	27	43	35
113189	D4ST1	dermatan 4 sulfotransferase 1	25	45	35
22919	MAPRE1	microtubule-associated protein, RP/EB family, member 1	17	53	35
5431	POLR2B	polymerase (RNA) II (DNA directed) polypeptide B, 140kDa	10	60	35
29946	SERTAD3	SERTA domain containing 3	10	60	35
8241	RBM10	RNA binding motif protein 10	4	66	35
10048	RANBP9	RAN binding protein 9	0	70	35
5738	PTGFRN	prostaglandin F2 receptor negative regulator	0	70	35
4355	MPP2	membrane protein, palmitoylated 2 (MAGUK p55 subfamily r	0	70	35
5600	MAPK11	mitogen-activated protein kinase 11	0	70	35
25878	MXRA5	matrix-remodelling associated 5	0	70	35
8774	NAPG	N-ethylmaleimide-sensitive factor attachment protein, gamm	70	0	35
10401	PIAS3	protein inhibitor of activated STAT, 3	59	10	34.5
10236	HNRPR	heterogeneous nuclear ribonucleoprotein R	52	17	34.5
10123	ARL4C	ADP-ribosylation factor-like 4C	50	19	34.5
6423	SFRP2	secreted frizzled-related protein 2	38	31	34.5
9570	GOSR2	golgi SNAP receptor complex member 2	38	31	34.5
23016	EXOSC7	exosome component 7	35	34	34.5
116983	CENTB5	centaurin, beta 5	30	39	34.5
902	CCNH	cyclin H	27	42	34.5
55930	MYO5C	myosin VC	22	47	34.5
148479	PHF13	PHD finger protein 13	22	47	34.5
23253	ANKRD12	ankyrin repeat domain 12	19	50	34.5
10058	ABCB6	ATP-binding cassette, sub-family B (MDR/TAP), member 6	18	51	34.5
51110	LACTB2	lactamase, beta 2	18	51	34.5
93	ACVR2B	activin A receptor, type IIB	14	55	34.5
9958	USP15	ubiquitin specific peptidase 15	14	55	34.5
127845	GOLT1A	golgi transport 1 homolog A (S. cerevisiae)	10	59	34.5
113026	PLCD3	phospholipase C, delta 3	0	69	34.5
83941	TM2D1	TM2 domain containing 1	0	69	34.5
10232	MSLN	mesothelin	0	69	34.5
4713	NDUFB7	NADH dehydrogenase (ubiquinone) 1 beta subcomplex, 7, 1	0	69	34.5
79786	C16orf44	chromosome 16 open reading frame 44	69	0	34.5
546	ATRX	alpha thalassemia/mental retardation syndrome X-linked (RA	69	0	34.5
124936	CYB5D2	cytochrome b5 domain containing 2	69	0	34.5
23196	FAM120A	family with sequence similarity 120A	69	0	34.5
9853	RUSC2	RUN and SH3 domain containing 2	69	0	34.5
1800	DPEP1	dipeptidase 1 (renal)	69	0	34.5
56681	SAR1A	SAR1 gene homolog A (S. cerevisiae)	66	2	34
10302	SNAPC5	small nuclear RNA activating complex, polypeptide 5, 19kDa	65	3	34
9489	PGS1	phosphatidylglycerophosphate synthase 1	59	9	34
23621	BACE1	beta-site APP-cleaving enzyme 1	56	12	34
221061	C10orf38	chromosome 10 open reading frame 38	46	22	34
5261	PHKG2	phosphorylase kinase, gamma 2 (testis)	46	22	34
7078	TIMP3	TIMP metallopeptidase inhibitor 3 (Sorsby fundus dystrophy,	46	22	34
80025	PANK2	pantothenate kinase 2 (Hallervorden-Spatz syndrome)	43	25	34
84441	MAML2	mastermind-like 2 (Drosophila)	40	28	34

134728	IRAK1BP1	interleukin-1 receptor-associated kinase 1 binding protein 1	39	29	34
493856	ZCD2	zinc finger, CDGSH-type domain 2	32	36	34
2775	GNAO1	guanine nucleotide binding protein (G protein), alpha activati	30	38	34
55294	FBXW7	F-box and WD-40 domain protein 7 (archipelago homolog, D	30	38	34
56667	MUC13	mucin 13, cell surface associated	29	39	34
23195	MDN1	MDN1, midasin homolog (yeast)	25	43	34
4345	CD200	CD200 molecule	23	45	34
2733	GLE1L	GLE1 RNA export mediator-like (yeast)	17	51	34
23705	IGSF4	immunoglobulin superfamily, member 4	16	52	34
63947	DMRTC1	DMRT-like family C1	15	53	34
8439	NSMAF	neutral sphingomyelinase (N-SMase) activation associated fa	15	53	34
6535	SLC6A8	solute carrier family 6 (neurotransmitter transporter, creatine	12	56	34
92291	CAPN13	calpain 13	6	62	34
55893	ZNF395	zinc finger protein 395	4	64	34
200186	CRTC2	CREB regulated transcription coactivator 2	4	64	34
146174	C16orf52	chromosome 16 open reading frame 52	68	0	34
57062	DDX24	DEAD (Asp-Glu-Ala-Asp) box polypeptide 24	65	2	33.5
7058	THBS2	thrombospondin 2	65	2	33.5
10138	YAF2	YY1 associated factor 2	59	8	33.5
160	AP2A1	adaptor-related protein complex 2, alpha 1 subunit	58	9	33.5
26499	PLEK2	pleckstrin 2	49	18	33.5
84661	LOC84661	dpy-30-like protein	49	18	33.5
56666	PANX2	pannexin 2	47	20	33.5
151516	SASP	skin aspartic protease	47	20	33.5
51603	KIAA0859	KIAA0859	44	23	33.5
1389	CREBL2	cAMP responsive element binding protein-like 2	42	25	33.5
23065	KIAA0090	KIAA0090	41	26	33.5
7064	THOP1	thimet oligopeptidase 1	26	41	33.5
406	ARNTL	aryl hydrocarbon receptor nuclear translocator-like	19	48	33.5
9445	ITM2B	integral membrane protein 2B	17	50	33.5
51290	ERGIC2	ERGIC and golgi 2	14	53	33.5
374969	CCDC23	coiled-coil domain containing 23	12	55	33.5
10628	TXNIP	thioredoxin interacting protein	8	59	33.5
1643	DDB2	damage-specific DNA binding protein 2, 48kDa	4	63	33.5
123803	NTAN1	N-terminal asparagine amidase	0	67	33.5
119391	GSTO2	glutathione S-transferase omega 2	0	67	33.5
79893	ZNF403	zinc finger protein 403	67	0	33.5
29089	UBE2T	ubiquitin-conjugating enzyme E2T (putative)	67	0	33.5
51307	FAM53C	family with sequence similarity 53, member C	67	0	33.5
55316	RSAD1	radical S-adenosyl methionine domain containing 1	67	0	33.5
2740	GLP1R	glucagon-like peptide 1 receptor	67	0	33.5
220002	CYBASC3	cytochrome b, ascorbate dependent 3	67	0	33.5
1284	COL4A2	collagen, type IV, alpha 2	64	2	33
9946	CRYZL1	crystallin, zeta (quinone reductase)-like 1	63	3	33
64975	MRPL41	mitochondrial ribosomal protein L41	57	9	33
57169	ZNFX1	zinc finger, NFX1-type containing 1	56	10	33
2939	GSTA2	glutathione S-transferase A2	55	11	33
23369	PUM2	pumilio homolog 2 (Drosophila)	51	15	33
6919	TCEA2	transcription elongation factor A (SII), 2	47	19	33
2180	ACSL1	acyl-CoA synthetase long-chain family member 1	45	21	33
550	AUP1	ancient ubiquitous protein 1	42	24	33

5462	POU5F1P1	POU domain, class 5, transcription factor 1 pseudogene 1	40	26	33
440275	EIF2AK4	eukaryotic translation initiation factor 2 alpha kinase 4	35	31	33
126299	ZNF428	zinc finger protein 428	28	38	33
89953	KLC4	kinesin light chain 4	28	38	33
134492	NUDCD2	NudC domain containing 2	24	42	33
5887	RAD23B	RAD23 homolog B (S. cerevisiae)	17	49	33
202	AIM1	absent in melanoma 1	15	51	33
1499	CTNNB1	catenin (cadherin-associated protein), beta 1, 88kDa	12	54	33
3866	KRT15	keratin 15	10	56	33
9075	CLDN2	claudin 2	6	60	33
57724	KIAA1632	KIAA1632	0	66	33
57348	TTYH1	tweety homolog 1 (Drosophila)	0	66	33
23264	ZC3H7B	zinc finger CCCH-type containing 7B	0	66	33
91875	TTC5	tetratricopeptide repeat domain 5	0	66	33
89849	ATG16L2	ATG16 autophagy related 16-like 2 (S. cerevisiae)	63	2	32.5
23173	METAP1	methionyl aminopeptidase 1	57	8	32.5
976	CD97	CD97 molecule	55	10	32.5
7226	TRPM2	transient receptor potential cation channel, subfamily M, mer	54	11	32.5
23172	KIAA0157	KIAA0157	49	16	32.5
2639	GCDH	glutaryl-Coenzyme A dehydrogenase	49	16	32.5
134510	UBLCP1	ubiquitin-like domain containing CTD phosphatase 1	45	20	32.5
55061	SUSD4	sushi domain containing 4	45	20	32.5
118987	PDZD8	PDZ domain containing 8	41	24	32.5
9179	AP4M1	adaptor-related protein complex 4, mu 1 subunit	40	25	32.5
7988	ZNF212	zinc finger protein 212	37	28	32.5
161424	C14orf21	chromosome 14 open reading frame 21	36	29	32.5
55854	ZC3H15	zinc finger CCCH-type containing 15	32	33	32.5
23077	MYCBP2	MYC binding protein 2	32	33	32.5
2002	ELK1	ELK1, member of ETS oncogene family	24	41	32.5
26020	LRP10	low density lipoprotein receptor-related protein 10	18	47	32.5
6202	RPS8	ribosomal protein S8	14	51	32.5
23476	BRD4	bromodomain containing 4	12	53	32.5
53981	CPSF2	cleavage and polyadenylation specific factor 2, 100kDa	6	59	32.5
725	C4BPB	complement component 4 binding protein, beta	6	59	32.5
23787	MTCH1	mitochondrial carrier homolog 1 (C. elegans)	6	59	32.5
9537	TP53I11	tumor protein p53 inducible protein 11	2	63	32.5
85446	ZFHX2	zinc finger homeobox 2	0	65	32.5
550112	LOC550112	hypothetical LOC550112	0	65	32.5
116461	C1orf19	chromosome 1 open reading frame 19	0	65	32.5
284677	C1orf204	chromosome 1 open reading frame 204	0	65	32.5
891	CCNB1	cyclin B1	0	65	32.5
10844	TUBGCP2	tubulin, gamma complex associated protein 2	65	0	32.5
9138	ARHGEF1	Rho guanine nucleotide exchange factor (GEF) 1	65	0	32.5
1385	CREB1	cAMP responsive element binding protein 1	65	0	32.5
114904	C1QTNF6	C1q and tumor necrosis factor related protein 6	65	0	32.5
54467	ANKIB1	ankyrin repeat and IBR domain containing 1	65	0	32.5
78986	DUSP26	dual specificity phosphatase 26 (putative)	65	0	32.5
26234	FBXL5	F-box and leucine-rich repeat protein 5	65	0	32.5
6478	SIAH2	seven in absentia homolog 2 (Drosophila)	65	0	32.5
604	BCL6	B-cell CLL/lymphoma 6 (zinc finger protein 51)	65	0	32.5
3376	IARS	isoleucine-tRNA synthetase	65	0	32.5

9158	FIBP	fibroblast growth factor (acidic) intracellular binding protein	65	0	32.5
79071	ELOVL6	ELOVL family member 6, elongation of long chain fatty acids	65	0	32.5
8260	ARD1A	ARD1 homolog A, N-acetyltransferase (S. cerevisiae)	65	0	32.5
23339	VPS39	vacuolar protein sorting 39 homolog (S. cerevisiae)	65	0	32.5
10279	PRSS16	protease, serine, 16 (thymus)	59	5	32
10951	CBX1	chromobox homolog 1 (HP1 beta homolog Drosophila)	59	5	32
1659	DHX8	DEAH (Asp-Glu-Ala-His) box polypeptide 8	58	6	32
54993	ZSCAN2	zinc finger and SCAN domain containing 2	57	7	32
51231	VRK3	vaccinia related kinase 3	51	13	32
10195	ALG3	asparagine-linked glycosylation 3 homolog (S. cerevisiae, al	50	14	32
5188	PET112L	PET112-like (yeast)	38	26	32
79676	OGFOD2	2-oxoglutarate and iron-dependent oxygenase domain conta	35	29	32
128439	C20orf198	chromosome 20 open reading frame 198	35	29	32
55300	PI4K2B	phosphatidylinositol 4-kinase type 2 beta	33	31	32
92181	UBTD2	ubiquitin domain containing 2	23	41	32
147807	ZNF524	zinc finger protein 524	18	46	32
5664	PSEN2	presenilin 2 (Alzheimer disease 4)	15	49	32
401474	SAMD12	sterile alpha motif domain containing 12	10	54	32
9295	SFRS11	splicing factor, arginine/serine-rich 11	6	58	32
201965	RWDD4A	RWD domain containing 4A	2	62	32
9798	KIAA0174	KIAA0174	0	64	32
22872	SEC31A	SEC31 homolog A (S. cerevisiae)	0	64	32
4502	MT2A	metallothionein 2A	0	64	32
64063	PRSS22	protease, serine, 22	0	64	32
6792	CDKL5	cyclin-dependent kinase-like 5	61	2	31.5
285419	LOC285419	hypothetical protein LOC285419	61	2	31.5
9053	MAP7	microtubule-associated protein 7	60	3	31.5
55151	TMEM38B	transmembrane protein 38B	50	13	31.5
22933	SIRT2	sirtuin (silent mating type information regulation 2 homolog) :	43	20	31.5
6310	ATXN1	ataxin 1	40	23	31.5
26268	FBXO9	F-box protein 9	39	24	31.5
6642	SNX1	sorting nexin 1	37	26	31.5
27102	EIF2AK1	eukaryotic translation initiation factor 2-alpha kinase 1	33	30	31.5
55246	CCDC25	coiled-coil domain containing 25	31	32	31.5
56954	NIT2	nitrilase family, member 2	29	34	31.5
84172	POLR1B	polymerase (RNA) I polypeptide B, 128kDa	27	36	31.5
8743	TNFSF10	tumor necrosis factor (ligand) superfamily, member 10	26	37	31.5
51147	ING4	inhibitor of growth family, member 4	22	41	31.5
79823	C2orf34	chromosome 2 open reading frame 34	21	42	31.5
51537	MTP18	mitochondrial protein 18 kDa	10	53	31.5
25977	NECAP1	NECAP endocytosis associated 1	6	57	31.5
9873	FCHSD2	FCH and double SH3 domains 2	4	59	31.5
55370	PPP4R1L	protein phosphatase 4, regulatory subunit 1-like	4	59	31.5
196743	PAOX	polyamine oxidase (exo-N4-amino)	4	59	31.5
56892	C8orf4	chromosome 8 open reading frame 4	4	59	31.5
115827	RAB3C	RAB3C, member RAS oncogene family	2	61	31.5
10190	TXNDC9	thioredoxin domain containing 9	0	63	31.5
7203	CCT3	chaperonin containing TCP1, subunit 3 (gamma)	0	63	31.5
84826	SFT2D3	SFT2 domain containing 3	0	63	31.5
54989	ZNF770	zinc finger protein 770	0	63	31.5
54619	CCNJ	cyclin J	0	63	31.5

5962	RDX	radixin	63	0	31.5
26747	NUFIP1	nuclear fragile X mental retardation protein interacting protei	63	0	31.5
10959	TMED2	transmembrane emp24 domain trafficking protein 2	63	0	31.5
27068	PPA2	pyrophosphatase (inorganic) 2	63	0	31.5
23067	SETD1B	SET domain containing 1B	63	0	31.5
57157	PHTF2	putative homeodomain transcription factor 2	63	0	31.5
4215	MAP3K3	mitogen-activated protein kinase kinase kinase 3	63	0	31.5
129563	MGC42174	hypothetical protein MGC42174	63	0	31.5
1337	COX6A1	cytochrome c oxidase subunit VIa polypeptide 1	63	0	31.5
10746	MAP3K2	mitogen-activated protein kinase kinase kinase 2	63	0	31.5
3611	ILK	integrin-linked kinase	60	2	31
8893	EIF2B5	eukaryotic translation initiation factor 2B, subunit 5 epsilon, 8	59	3	31
5884	RAD17	RAD17 homolog (S. pombe)	59	3	31
51099	ABHD5	abhydrolase domain containing 5	55	7	31
10128	LRPPRC	leucine-rich PPR-motif containing	54	8	31
488	ATP2A2	ATPase, Ca++ transporting, cardiac muscle, slow twitch 2	54	8	31
2983	GUCY1B3	guanylate cyclase 1, soluble, beta 3	52	10	31
340277	C7orf46	chromosome 7 open reading frame 46	52	10	31
54976	C20orf27	chromosome 20 open reading frame 27	49	13	31
60509	FLJ21839	hypothetical protein FLJ21839	47	15	31
220	ALDH1A3	aldehyde dehydrogenase 1 family, member A3	43	19	31
57532	NUFIP2	nuclear fragile X mental retardation protein interacting protei	41	21	31
399665	FAM102A	family with sequence similarity 102, member A	40	22	31
1804	DPP6	dipeptidyl-peptidase 6	34	28	31
2962	GTF2F1	general transcription factor IIF, polypeptide 1, 74kDa	29	33	31
55615	PRR5	proline rich 5 (renal)	28	34	31
54861	SNRK	SNF related kinase	28	34	31
847	CAT	catalase	27	35	31
6789	STK4	serine/threonine kinase 4	26	36	31
9686	VGLL4	vestigial like 4 (Drosophila)	24	38	31
64754	SMYD3	SET and MYND domain containing 3	20	42	31
64978	MRPL38	mitochondrial ribosomal protein L38	18	44	31
50848	F11R	F11 receptor	17	45	31
4688	NCF2	neutrophil cytosolic factor 2 (65kDa, chronic granulomatous	16	46	31
23080	KIAA0241	KIAA0241	6	56	31
10491	CRTAP	cartilage associated protein	4	58	31
7855	FZD5	frizzled homolog 5 (Drosophila)	2	60	31
9988	DMTF1	cyclin D binding myb-like transcription factor 1	2	60	31
55092	TMEM51	transmembrane protein 51	0	62	31
43	ACHE	acetylcholinesterase (Yt blood group)	0	62	31
51317	PHF21A	PHD finger protein 21A	0	62	31
463	ATBF1	AT-binding transcription factor 1	0	62	31
10479	SLC9A6	solute carrier family 9 (sodium/hydrogen exchanger), membe	0	62	31
84852	C1orf203	chromosome 1 open reading frame 203	0	62	31
10783	NEK6	NIMA (never in mitosis gene a)-related kinase 6	0	62	31
6745	SSR1	signal sequence receptor, alpha (translocon-associated prot	0	62	31
83892	KCTD10	potassium channel tetramerisation domain containing 10	62	0	31
2152	F3	coagulation factor III (thromboplastin, tissue factor)	62	0	31
10982	MAPRE2	microtubule-associated protein, RP/EB family, member 2	59	2	30.5
2767	GNA11	guanine nucleotide binding protein (G protein), alpha 11 (Gq	54	7	30.5
285550	LOC285550	hypothetical protein LOC285550	52	9	30.5

1029	CDKN2A	cyclin-dependent kinase inhibitor 2A (melanoma, p16, inhibit	51	10	30.5
5432	POLR2C	polymerase (RNA) II (DNA directed) polypeptide C, 33kDa	50	11	30.5
163732	CITED4	Cbp/p300-interacting transactivator, with Glu/Asp-rich carbox	44	17	30.5
34	ACADM	acyl-Coenzyme A dehydrogenase, C-4 to C-12 straight chair	43	18	30.5
8683	SFRS9	splicing factor, arginine/serine-rich 9	35	26	30.5
284459	HKR1	GLI-Kruppel family member HKR1	31	30	30.5
152330	CNTN4	contactin 4	30	31	30.5
35	ACADS	acyl-Coenzyme A dehydrogenase, C-2 to C-3 short chain	29	32	30.5
2669	GEM	GTP binding protein overexpressed in skeletal muscle	22	39	30.5
843	CASP10	caspase 10, apoptosis-related cysteine peptidase	20	41	30.5
6197	RPS6KA3	ribosomal protein S6 kinase, 90kDa, polypeptide 3	19	42	30.5
54331	GNG2	guanine nucleotide binding protein (G protein), gamma 2	18	43	30.5
84720	PIGO	phosphatidylinositol glycan anchor biosynthesis, class O	15	46	30.5
5709	PSMD3	proteasome (prosome, macropain) 26S subunit, non-ATPase	10	51	30.5
160897	GPR180	G protein-coupled receptor 180	10	51	30.5
54471	SMCR7L	Smith-Magenis syndrome chromosome region, candidate 7-l	8	53	30.5
57467	GUP1	GUP1 glycerol uptake/transporter homolog (S. cerevisiae)	4	57	30.5
8065	CUL5	cullin 5	2	59	30.5
8313	AXIN2	axin 2 (conductin, axil)	2	59	30.5
11122	PTPRT	protein tyrosine phosphatase, receptor type, T	0	61	30.5
8301	PICALM	phosphatidylinositol binding clathrin assembly protein	0	61	30.5
471	ATIC	5-aminoimidazole-4-carboxamide ribonucleotide formyltransf	61	0	30.5
140890	SFRS12	splicing factor, arginine/serine-rich 12	61	0	30.5
54557	SGTB	small glutamine-rich tetratricopeptide repeat (TPR)-containin	61	0	30.5
4013	LOH11CR2A	loss of heterozygosity, 11, chromosomal region 2, gene A	61	0	30.5
5786	PTPRA	protein tyrosine phosphatase, receptor type, A	61	0	30.5
51390	AIG1	androgen-induced 1	58	2	30
113791	MGC17330	HGFL gene	57	3	30
84260	TCHP	trichoplein, keratin filament binding	57	3	30
11015	KDELR3	KDEL (Lys-Asp-Glu-Leu) endoplasmic reticulum protein rete	46	14	30
9694	TTC35	tetratricopeptide repeat domain 35	35	25	30
9733	SART3	squamous cell carcinoma antigen recognized by T cells 3	32	28	30
10019	SH2B3	SH2B adaptor protein 3	26	34	30
53918	PELO	pelota homolog (Drosophila)	23	37	30
54093	SETD4	SET domain containing 4	22	38	30
113000	RPUSD1	RNA pseudouridylate synthase domain containing 1	18	42	30
285367	RPUSD3	RNA pseudouridylate synthase domain containing 3	17	43	30
2938	GSTA1	glutathione S-transferase A1	16	44	30
55750	MULK	multiple substrate lipid kinase	14	46	30
4915	NTRK2	neurotrophic tyrosine kinase, receptor, type 2	14	46	30
1181	CLCN2	chloride channel 2	6	54	30
10659	CUGBP2	CUG triplet repeat, RNA binding protein 2	4	56	30
23404	EXOSC2	exosome component 2	0	60	30
1397	CRIP2	cysteine-rich protein 2	0	60	30
26263	FBXO22	F-box protein 22	0	60	30
9427	ECEL1	endothelin converting enzyme-like 1	0	60	30
154215	TCBA1	T-cell lymphoma breakpoint associated target 1	0	60	30
6041	RNASEL	ribonuclease L (2',5'-oligoisoadenylate synthetase-depender	0	60	30
57223	SMEK2	SMEK homolog 2, suppressor of mek1 (Dictyostelium)	0	60	30
89822	KCNK17	potassium channel, subfamily K, member 17	56	3	29.5
9902	MRC2	mannose receptor, C type 2	51	8	29.5

9849	ZNF518	zinc finger protein 518	50	9	29.5
5122	PCSK1	proprotein convertase subtilisin/kexin type 1	49	10	29.5
115123	SEP3	membrane-associated ring finger (C3HC4) 3	48	11	29.5
5337	PLD1	phospholipase D1, phosphatidylcholine-specific	44	15	29.5
23012	STK38L	serine/threonine kinase 38 like	43	16	29.5
5439	POLR2J	polymerase (RNA) II (DNA directed) polypeptide J, 13.3kDa	39	20	29.5
124152	IQCK	IQ motif containing K	35	24	29.5
23170	TTLL12	tubulin tyrosine ligase-like family, member 12	35	24	29.5
29761	USP25	ubiquitin specific peptidase 25	32	27	29.5
54758	KLHDC4	kelch domain containing 4	31	28	29.5
348995	NUP43	nucleoporin 43kDa	27	32	29.5
55616	DDEFL1	development and differentiation enhancing factor-like 1	23	36	29.5
875	CBS	cystathionine-beta-synthase	23	36	29.5
55614	C20orf23	chromosome 20 open reading frame 23	13	46	29.5
8697	CDC23	cell division cycle 23 homolog (S. cerevisiae)	13	46	29.5
1769	DNAH8	dynein, axonemal, heavy chain 8	13	46	29.5
25979	DHRS7B	dehydrogenase/reductase (SDR family) member 7B	12	47	29.5
128077	LIX1L	Lix1 homolog (mouse)-like	10	49	29.5
50814	NSDHL	NAD(P) dependent steroid dehydrogenase-like	8	51	29.5
126353	C19orf21	chromosome 19 open reading frame 21	6	53	29.5
91746	YTHDC1	YTH domain containing 1	6	53	29.5
4436	MSH2	mutS homolog 2, colon cancer, nonpolyposis type 1 (E. coli)	2	57	29.5
57658	CALCOCO1	calcium binding and coiled-coil domain 1	0	59	29.5
54811	ZNF562	zinc finger protein 562	0	59	29.5
27019	DNAI1	dynein, axonemal, intermediate chain 1	0	59	29.5
11313	LYPLA2	lysophospholipase II	0	59	29.5
4641	MYO1C	myosin IC	0	59	29.5
220992	ZNF485	zinc finger protein 485	59	0	29.5
5950	RBP4	retinol binding protein 4, plasma	59	0	29.5
92170	MTG1	mitochondrial GTPase 1 homolog (S. cerevisiae)	59	0	29.5
81606	LBH	limb bud and heart development homolog (mouse)	59	0	29.5
55909	BIN3	bridging integrator 3	59	0	29.5
5372	PMM1	phosphomannomutase 1	59	0	29.5
9709	HERPUD1	homocysteine-inducible, endoplasmic reticulum stress-induc	59	0	29.5
10736	SIX2	sine oculis homeobox homolog 2 (Drosophila)	59	0	29.5
1293	COL6A3	collagen, type VI, alpha 3	55	3	29
51768	TM7SF3	transmembrane 7 superfamily member 3	53	5	29
55964	SEP3	septin 3	52	6	29
222865	TMEM130	transmembrane protein 130	51	7	29
22929	SEPHS1	selenophosphate synthetase 1	43	15	29
55914	ERBB2IP	erbb2 interacting protein	43	15	29
4739	NEDD9	neural precursor cell expressed, developmentally down-regu	41	17	29
4684	NCAM1	neural cell adhesion molecule 1	34	24	29
8220	DGCR14	DiGeorge syndrome critical region gene 14	30	28	29
473	RERE	arginine-glutamic acid dipeptide (RE) repeats	24	34	29
6464	SHC1	SHC (Src homology 2 domain containing) transforming prote	22	36	29
4717	NDUFC1	NADH dehydrogenase (ubiquinone) 1, subcomplex unknowr	21	37	29
93210	PERLD1	per1-like domain containing 1	20	38	29
7702	ZNF143	zinc finger protein 143	19	39	29
81890	QTRT1	queuine tRNA-ribosyltransferase 1 (tRNA-guanine transglyco	19	39	29
5833	PCYT2	phosphate cytidylyltransferase 2, ethanolamine	14	44	29

7458	EIF4H	eukaryotic translation initiation factor 4H	14	44	29
55848	C9orf46	chromosome 9 open reading frame 46	12	46	29
4086	SMAD1	SMAD family member 1	10	48	29
27134	TJP3	tight junction protein 3 (zona occludens 3)	0	58	29
64224	HERPUD2	HERPUD family member 2	55	2	28.5
91133	L3MBTL4	l(3)mbt-like 4 (Drosophila)	53	4	28.5
10290	SPEG	SPEG complex locus	52	5	28.5
9761	KIAA0152	KIAA0152	50	7	28.5
387647	LOC387647	hypothetical gene supported by BC014163	47	10	28.5
132158	GLYCTK	glycerate kinase	45	12	28.5
51260	CXorf26	chromosome X open reading frame 26	42	15	28.5
4524	MTHFR	5,10-methylenetetrahydrofolate reductase (NADPH)	42	15	28.5
203260	CCDC107	coiled-coil domain containing 107	40	17	28.5
55558	PLXNA3	plexin A3	39	18	28.5
585	BBS4	Bardet-Biedl syndrome 4	37	20	28.5
57187	THOC2	THO complex 2	35	22	28.5
10899	JTB	jumping translocation breakpoint	30	27	28.5
64420	SUSD1	sushi domain containing 1	26	31	28.5
10096	ACTR3	ARP3 actin-related protein 3 homolog (yeast)	25	32	28.5
2589	GALNT1	UDP-N-acetyl-alpha-D-galactosamine:polypeptide N-acetylg	23	34	28.5
583	BBS2	Bardet-Biedl syndrome 2	18	39	28.5
5780	PTPN9	protein tyrosine phosphatase, non-receptor type 9	12	45	28.5
221710	LOC221710	hypothetical protein LOC221710	8	49	28.5
147339	C18orf25	chromosome 18 open reading frame 25	6	51	28.5
142940	TRUB1	TruB pseudouridine (psi) synthase homolog 1 (E. coli)	6	51	28.5
10189	THOC4	THO complex 4	6	51	28.5
762	CA4	carbonic anhydrase IV	2	55	28.5
3488	IGFBP5	insulin-like growth factor binding protein 5	2	55	28.5
84910	TMEM87B	transmembrane protein 87B	2	55	28.5
9638	FEZ1	fasciculation and elongation protein zeta 1 (zygin I)	0	57	28.5
404037	HAPLN4	hyaluronan and proteoglycan link protein 4	0	57	28.5
23623	RUSC1	RUN and SH3 domain containing 1	0	57	28.5
6293	VPS52	vacuolar protein sorting 52 homolog (S. cerevisiae)	0	57	28.5
822	CAPG	capping protein (actin filament), gelsolin-like	0	57	28.5
757	TMEM50B	transmembrane protein 50B	0	57	28.5
7851	MALL	mal, T-cell differentiation protein-like	0	57	28.5
55773	TBC1D23	TBC1 domain family, member 23	0	57	28.5
2335	FN1	fibronectin 1	0	57	28.5
51027	BOLA1	bolA homolog 1 (E. coli)	0	57	28.5
1375	CPT1B	carnitine palmitoyltransferase 1B (muscle)	57	0	28.5
285749	LOC285749	hypothetical protein LOC285749	57	0	28.5
401539	FLJ42342	hypothetical gene supported by AK124333	53	3	28
51706	CYB5R1	cytochrome b5 reductase 1	53	3	28
389	RHOC	ras homolog gene family, member C	48	8	28
81693	AMN	amnionless homolog (mouse)	45	11	28
11164	NUDT5	nudix (nucleoside diphosphate linked moiety X)-type motif 5	43	13	28
3831	KNS2	kinesin 2	42	14	28
1613	DAPK3	death-associated protein kinase 3	41	15	28
55224	ETNK2	ethanolamine kinase 2	31	25	28
120	ADD3	adducin 3 (gamma)	25	31	28
55421	C17orf85	chromosome 17 open reading frame 85	19	37	28

84795	C10orf33	chromosome 10 open reading frame 33	17	39	28
26984	SEC22A	SEC22 vesicle trafficking protein homolog A (S. cerevisiae)	16	40	28
1739	DLG1	discs, large homolog 1 (Drosophila)	14	42	28
112609	C6orf117	chromosome 6 open reading frame 117	13	43	28
8209	C21orf33	chromosome 21 open reading frame 33	12	44	28
3777	KCNK3	potassium channel, subfamily K, member 3	10	46	28
55636	CHD7	chromodomain helicase DNA binding protein 7	6	50	28
57513	CASKIN2	CASK interacting protein 2	2	54	28
84623	KIRREL3	kin of IRRE like 3 (Drosophila)	0	56	28
401478	FLJ45872	FLJ45872 protein	0	56	28
55266	TMEM19	transmembrane protein 19	0	56	28
5336	PLCG2	phospholipase C, gamma 2 (phosphatidylinositol-specific)	0	56	28
26580	BSCL2	Bernardinelli-Seip congenital lipodystrophy 2 (seipin)	0	56	28
64651	AXUD1	AXIN1 up-regulated 1	0	56	28
2904	GRIN2B	glutamate receptor, ionotropic, N-methyl D-aspartate 2B	56	0	28
6591	SNAI2	snail homolog 2 (Drosophila)	56	0	28
84872	ZC3H10	zinc finger CCCH-type containing 10	56	0	28
51222	ZNF219	zinc finger protein 219	56	0	28
5192	PEX10	peroxisome biogenesis factor 10	56	0	28
2738	GLI4	GLI-Kruppel family member GLI4	56	0	28
57406	ABHD6	abhydrolase domain containing 6	53	2	27.5
81790	RNF170	ring finger protein 170	52	3	27.5
208	AKT2	v-akt murine thymoma viral oncogene homolog 2	42	13	27.5
8912	CACNA1H	calcium channel, voltage-dependent, alpha 1H subunit	39	16	27.5
131616	TMEM42	transmembrane protein 42	39	16	27.5
399909	PCNXL3	pecanex-like 3 (Drosophila)	38	17	27.5
8650	NUMB	numb homolog (Drosophila)	37	18	27.5
8444	DYRK3	dual-specificity tyrosine-(Y)-phosphorylation regulated kinase	35	20	27.5
114881	OSBPL7	oxysterol binding protein-like 7	35	20	27.5
873	CBR1	carbonyl reductase 1	34	21	27.5
9527	GOSR1	golgi SNAP receptor complex member 1	33	22	27.5
130612	MGC99813	similar to RIKEN cDNA A230078I05 gene	29	26	27.5
1612	DAPK1	death-associated protein kinase 1	29	26	27.5
152485	LOC152485	hypothetical protein LOC152485	25	30	27.5
10472	ZNF238	zinc finger protein 238	20	35	27.5
192668	CYS1	cystin 1	19	36	27.5
147184	TMEM99	transmembrane protein 99	19	36	27.5
23139	MAST2	microtubule associated serine/threonine kinase 2	16	39	27.5
51564	HDAC7A	histone deacetylase 7A	15	40	27.5
6426	SFRS1	splicing factor, arginine/serine-rich 1 (splicing factor 2, altern	15	40	27.5
56945	MRPS22	mitochondrial ribosomal protein S22	12	43	27.5
283537	LOC283537	hypothetical protein LOC283537	10	45	27.5
7106	TSPAN4	tetraspanin 4	8	47	27.5
54453	RIN2	Ras and Rab interactor 2	6	49	27.5
23135	JMJD3	jumonji domain containing 3	4	51	27.5
684	BST2	bone marrow stromal cell antigen 2	4	51	27.5
23140	ZZEF1	zinc finger, ZZ-type with EF-hand domain 1	0	55	27.5
8504	PEX3	peroxisomal biogenesis factor 3	0	55	27.5
6649	SOD3	superoxide dismutase 3, extracellular	55	0	27.5
5096	PCCB	propionyl Coenzyme A carboxylase, beta polypeptide	55	0	27.5
283238	MGC34821	hypothetical protein MGC34821	55	0	27.5

22901	ARSG	arylsulfatase G	55	0	27.5
29994	BAZ2B	bromodomain adjacent to zinc finger domain, 2B	49	5	27
9760	TOX	thymus high mobility group box protein TOX	47	7	27
84261	FBXW9	F-box and WD-40 domain protein 9	46	8	27
55787	CXorf15	chromosome X open reading frame 15	46	8	27
10550	ARL6IP5	ADP-ribosylation-like factor 6 interacting protein 5	43	11	27
25831	HECTD1	HECT domain containing 1	40	14	27
10054	SAE2	SUMO1 activating enzyme subunit 2	39	15	27
9885	OSBPL2	oxysterol binding protein-like 2	39	15	27
59271	C21orf63	chromosome 21 open reading frame 63	39	15	27
9487	PIGL	phosphatidylinositol glycan anchor biosynthesis, class L	34	20	27
28991	COMMD5	COMM domain containing 5	30	24	27
114928	GPRASP2	G protein-coupled receptor associated sorting protein 2	29	25	27
51742	ARID4B	AT rich interactive domain 4B (RBP1-like)	16	38	27
4059	BCAM	basal cell adhesion molecule (Lutheran blood group)	15	39	27
257407	LOC257407	hypothetical protein LOC257407	13	41	27
84134	TOMM40L	translocase of outer mitochondrial membrane 40 homolog-lik	12	42	27
767	CA8	carbonic anhydrase VIII	12	42	27
2191	FAP	fibroblast activation protein, alpha	8	46	27
728923	LOC728923	hypothetical protein LOC728923	8	46	27
440145	RP11-11C5.2	similar to RIKEN cDNA 2410129H14	8	46	27
284252	KCTD1	potassium channel tetramerisation domain containing 1	6	48	27
8153	RND2	Rho family GTPase 2	0	54	27
8701	DNAH11	dynein, axonemal, heavy chain 11	0	54	27
65975	STK33	serine/threonine kinase 33	0	54	27
5630	PRPH	peripherin	0	54	27
5514	PPP1R10	protein phosphatase 1, regulatory subunit 10	0	54	27
23512	SUZ12	suppressor of zeste 12 homolog (Drosophila)	0	54	27
79174	CRELD2	cysteine-rich with EGF-like domains 2	0	54	27
26775	SNORA72	small nucleolar RNA, H/ACA box 72	0	54	27
55884	WSB2	WD repeat and SOCS box-containing 2	0	54	27
9649	RALGPS1	Ral GEF with PH domain and SH3 binding motif 1	0	54	27
29105	C16orf80	chromosome 16 open reading frame 80	54	0	27
8078	USP5	ubiquitin specific peptidase 5 (isopeptidase T)	54	0	27
121506	C12orf46	chromosome 12 open reading frame 46	54	0	27
148304	C1orf74	chromosome 1 open reading frame 74	54	0	27
347735	SERINC2	serine incorporator 2	54	0	27
25984	KRT23	keratin 23 (histone deacetylase inducible)	48	5	26.5
60	ACTB	actin, beta	48	5	26.5
1465	CSRP1	cysteine and glycine-rich protein 1	45	8	26.5
175	AGA	aspartylglucosaminidase	43	10	26.5
51535	PPHLN1	periphilin 1	40	13	26.5
55230	USP40	ubiquitin specific peptidase 40	38	15	26.5
10207	INADL	InaD-like (Drosophila)	35	18	26.5
1119	CHKA	choline kinase alpha	33	20	26.5
11343	MGLL	monoglyceride lipase	30	23	26.5
64320	RNF25	ring finger protein 25	27	26	26.5
8682	PEA15	phosphoprotein enriched in astrocytes 15	20	33	26.5
23197	UBXD8	UBX domain containing 8	20	33	26.5
23053	KIAA0913	KIAA0913	20	33	26.5
55250	STATIP1	signal transducer and activator of transcription 3 interacting p	19	34	26.5

54852	PAQR5	progestin and adipoQ receptor family member V	17	36	26.5
57007	CXCR7	chemokine (C-X-C motif) receptor 7	17	36	26.5
60560	MAK10	MAK10 homolog, amino-acid N-acetyltransferase subunit, (S	17	36	26.5
51530	ZC3HC1	zinc finger, C3HC-type containing 1	13	40	26.5
8544	PIR	pirin (iron-binding nuclear protein)	13	40	26.5
9818	NUPL1	nucleoporin like 1	8	45	26.5
705	BYSL	bystin-like	0	53	26.5
57060	PCBP4	poly(rC) binding protein 4	0	53	26.5
57657	HCN3	hyperpolarization activated cyclic nucleotide-gated potassiur	0	53	26.5
130026	ICA1L	islet cell autoantigen 1,69kDa-like	0	53	26.5
7113	TMPRSS2	transmembrane protease, serine 2	0	53	26.5
818	CAMK2G	calcium/calmodulin-dependent protein kinase (CaM kinase) I	0	53	26.5
23468	CBX5	chromobox homolog 5 (HP1 alpha homolog, Drosophila)	0	53	26.5
140706	C20orf160	chromosome 20 open reading frame 160	0	53	26.5
83642	RP3-402G11	selenoprotein O	53	0	26.5
79866	C13orf34	chromosome 13 open reading frame 34	53	0	26.5
4773	NFATC2	nuclear factor of activated T-cells, cytoplasmic, calcineurin-d	53	0	26.5
64129	TINAGL1	tubulointerstitial nephritis antigen-like 1	53	0	26.5
51256	TBC1D7	TBC1 domain family, member 7	53	0	26.5
124401	ANKS3	ankyrin repeat and sterile alpha motif domain containing 3	53	0	26.5
2643	GCH1	GTP cyclohydrolase 1 (dopa-responsive dystonia)	53	0	26.5
7276	TTR	transthyretin (prealbumin, amyloidosis type I)	53	0	26.5
776	CACNA1D	calcium channel, voltage-dependent, L type, alpha 1D subur	49	3	26
65977	PLEKHA3	pleckstrin homology domain containing, family A (phosphoin	49	3	26
23556	PIGN	phosphatidylinositol glycan anchor biosynthesis, class N	47	5	26
79089	TMUB2	transmembrane and ubiquitin-like domain containing 2	46	6	26
7328	UBE2H	ubiquitin-conjugating enzyme E2H (UBC8 homolog, yeast)	45	7	26
6598	SMARCB1	SWI/SNF related, matrix associated, actin dependent regulat	44	8	26
54464	XRN1	5'-3' exoribonuclease 1	44	8	26
51115	FAM82B	family with sequence similarity 82, member B	41	11	26
27153	ZNF777	zinc finger protein 777	40	12	26
256987	SERINC5	serine incorporator 5	39	13	26
80325	ABTB1	ankyrin repeat and BTB (POZ) domain containing 1	38	14	26
79888	AYTL2	acyltransferase like 2	32	20	26
285971	ZNF775	zinc finger protein 775	28	24	26
4297	MLL	myeloid/lymphoid or mixed-lineage leukemia (trithorax homo	25	27	26
54942	C9orf6	chromosome 9 open reading frame 6	23	29	26
8623	ASMTL	acetylserotonin O-methyltransferase-like	10	42	26
6888	TALDO1	transaldolase 1	8	44	26
26260	FBXO25	F-box protein 25	8	44	26
22803	XRN2	5'-3' exoribonuclease 2	6	46	26
9553	MRPL33	mitochondrial ribosomal protein L33	6	46	26
650	BMP2	bone morphogenetic protein 2	6	46	26
155400	NSUN5B	NOL1/NOP2/Sun domain family, member 5B	6	46	26
25789	C19orf4	chromosome 19 open reading frame 4	4	48	26
339984	LOC339984	hypothetical protein LOC339984	0	52	26
51068	NMD3	NMD3 homolog (S. cerevisiae)	0	52	26
54107	POLE3	polymerase (DNA directed), epsilon 3 (p17 subunit)	0	52	26
54919	HEATR2	HEAT repeat containing 2	0	52	26
643210	FLJ40292	hypothetical protein LOC643210	0	52	26
5590	PRKCZ	protein kinase C, zeta	0	52	26

113178	SCAMP4	secretory carrier membrane protein 4	0	52	26
387119	C6orf204	chromosome 6 open reading frame 204	52	0	26
25903	OLFML2B	olfactomedin-like 2B	52	0	26
3491	CYR61	cysteine-rich, angiogenic inducer, 61	52	0	26
6742	SSBP1	single-stranded DNA binding protein 1	52	0	26
9572	NR1D1	nuclear receptor subfamily 1, group D, member 1	48	3	25.5
9242	MSC	musculin (activated B-cell factor-1)	46	5	25.5
3073	HEXA	hexosaminidase A (alpha polypeptide)	45	6	25.5
7084	TK2	thymidine kinase 2, mitochondrial	43	8	25.5
6713	SQLE	squalene epoxidase	42	9	25.5
23552	CCRK	cell cycle related kinase	28	23	25.5
9442	CRSP8	cofactor required for Sp1 transcriptional activation, subunit 8	20	31	25.5
23095	KIF1B	kinesin family member 1B	19	32	25.5
7474	WNT5A	wingless-type MMTV integration site family, member 5A	17	34	25.5
54890	ALKBH5	alkB, alkylation repair homolog 5 (E. coli)	10	41	25.5
11057	ABHD2	abhydrolase domain containing 2	8	43	25.5
4705	NDUFA10	NADH dehydrogenase (ubiquinone) 1 alpha subcomplex, 10	8	43	25.5
55341	LSG1	large subunit GTPase 1 homolog (S. cerevisiae)	4	47	25.5
9985	REC8L1	REC8-like 1 (yeast)	0	51	25.5
2615	LRRC32	leucine rich repeat containing 32	0	51	25.5
290	ANPEP	alanyl (membrane) aminopeptidase (aminopeptidase N, amii	0	51	25.5
55323	LARP6	La ribonucleoprotein domain family, member 6	0	51	25.5
55295	KLHL26	kelch-like 26 (Drosophila)	0	51	25.5
57553	MICAL3	microtubule associated monoxygenase, calponin and LIM dc	0	51	25.5
4318	MMP9	matrix metallopeptidase 9 (gelatinase B, 92kDa gelatinase, 9	0	51	25.5
54546	RNF186	ring finger protein 186	0	51	25.5
6341	SCO1	SCO cytochrome oxidase deficient homolog 1 (yeast)	0	51	25.5
6222	RPS18	ribosomal protein S18	0	51	25.5
57711	ZNF529	zinc finger protein 529	0	51	25.5
6786	STIM1	stromal interaction molecule 1	0	51	25.5
162282	ANKFN1	ankyrin-repeat and fibronectin type III domain containing 1	51	0	25.5
8370	HIST2H4A	histone cluster 2, H4a	51	0	25.5
3643	INSR	insulin receptor	51	0	25.5
65990	C16orf24	chromosome 16 open reading frame 24	51	0	25.5
259173	ALS2CL	ALS2 C-terminal like	51	0	25.5
8556	CDC14A	CDC14 cell division cycle 14 homolog A (S. cerevisiae)	51	0	25.5
7014	TERF2	telomeric repeat binding factor 2	51	0	25.5
80003	PCNXL2	pecanex-like 2 (Drosophila)	48	2	25
55146	ZDHHC4	zinc finger, DHHC-type containing 4	45	5	25
283901	LOC283901	hypothetical protein LOC283901	45	5	25
22837	COBLL1	COBL-like 1	45	5	25
83636	C19orf12	chromosome 19 open reading frame 12	38	12	25
26035	GLCE	UDP-glucuronic acid epimerase	38	12	25
26959	HBP1	HMG-box transcription factor 1	38	12	25
54442	KCTD5	potassium channel tetramerisation domain containing 5	35	15	25
6733	SRPK2	SFRS protein kinase 2	33	17	25
10300	KATNB1	katanin p80 (WD repeat containing) subunit B 1	30	20	25
866	SERPINA6	serpin peptidase inhibitor, clade A (alpha-1 antiproteinase, a	30	20	25
51116	MRPS2	mitochondrial ribosomal protein S2	27	23	25
214	ALCAM	activated leukocyte cell adhesion molecule	24	26	25
5289	PIK3C3	phosphoinositide-3-kinase, class 3	24	26	25

90411	MCFD2	multiple coagulation factor deficiency 2	24	26	25
57048	PLSCR3	phospholipid scramblase 3	24	26	25
3068	HDGF	hepatoma-derived growth factor (high-mobility group protein	21	29	25
567	B2M	beta-2-microglobulin	21	29	25
11005	SPINK5	serine peptidase inhibitor, Kazal type 5	21	29	25
64427	TTC31	tetratricopeptide repeat domain 31	19	31	25
10150	MBNL2	muscleblind-like 2 (Drosophila)	16	34	25
4131	MAP1B	microtubule-associated protein 1B	15	35	25
11222	MRPL3	mitochondrial ribosomal protein L3	13	37	25
25830	SULT4A1	sulfotransferase family 4A, member 1	13	37	25
23171	GPD1L	glycerol-3-phosphate dehydrogenase 1-like	8	42	25
79882	ZC3H14	zinc finger CCCH-type containing 14	8	42	25
4289	MKLN1	muskelin 1, intracellular mediator containing kelch motifs	6	44	25
51138	COPS4	COP9 constitutive photomorphogenic homolog subunit 4 (Ar	4	46	25
57403	RAB22A	RAB22A, member RAS oncogene family	2	48	25
2035	EPB41	erythrocyte membrane protein band 4.1 (elliptocytosis 1, RH	0	50	25
1861	TOR1A	torsin family 1, member A (torsin A)	0	50	25
115825	WDFY2	WD repeat and FYVE domain containing 2	50	0	25
8887	TAX1BP1	Tax1 (human T-cell leukemia virus type I) binding protein 1	50	0	25
5190	PEX6	peroxisomal biogenesis factor 6	50	0	25
961	CD47	CD47 molecule	47	2	24.5
11124	FAF1	Fas (TNFRSF6) associated factor 1	47	2	24.5
126432	FLJ45909	FLJ45909 protein	47	2	24.5
29123	ANKRD11	ankyrin repeat domain 11	47	2	24.5
56342	PPAN	peter pan homolog (Drosophila)	46	3	24.5
201895	C4orf34	chromosome 4 open reading frame 34	39	10	24.5
84919	PPP1R15B	protein phosphatase 1, regulatory (inhibitor) subunit 15B	38	11	24.5
688	KLF5	Kruppel-like factor 5 (intestinal)	31	18	24.5
7070	THY1	Thy-1 cell surface antigen	28	21	24.5
8451	CUL4A	cullin 4A	22	27	24.5
440712	C1orf186	chromosome 1 open reading frame 186	21	28	24.5
196441	CCDC131	coiled-coil domain containing 131	18	31	24.5
25962	KIAA1429	KIAA1429	18	31	24.5
642273	FAM110C	family with sequence similarity 110 member C	18	31	24.5
1106	CHD2	chromodomain helicase DNA binding protein 2	17	32	24.5
9644	SH3PXD2A	SH3 and PX domains 2A	15	34	24.5
5947	RBP1	retinol binding protein 1, cellular	10	39	24.5
54926	UBE2R2	ubiquitin-conjugating enzyme E2R 2	6	43	24.5
9968	MED12	mediator of RNA polymerase II transcription, subunit 12 hom	0	49	24.5
4733	DRG1	developmentally regulated GTP binding protein 1	0	49	24.5
9805	SCRN1	secernin 1	0	49	24.5
130916	MTERFD2	MTERF domain containing 2	0	49	24.5
56942	C16orf61	chromosome 16 open reading frame 61	0	49	24.5
55783	FLJ11171	hypothetical protein FLJ11171	0	49	24.5
5567	PRKACB	protein kinase, cAMP-dependent, catalytic, beta	0	49	24.5
23410	SIRT3	sirtuin (silent mating type information regulation 2 homolog) [illegible]	0	49	24.5
197322	LOC197322	hypothetical protein LOC197322	0	49	24.5
2124	EVI2B	ecotropic viral integration site 2B	0	49	24.5
6390	SDHB	succinate dehydrogenase complex, subunit B, iron sulfur (Ip)	0	49	24.5
23600	AMACR	alpha-methylacyl-CoA racemase	0	49	24.5
440258	LOC440258	similar to p40	0	49	24.5

1939	LGTN	ligatin	49	0	24.5
11212	PROSC	proline synthetase co-transcribed homolog (bacterial)	49	0	24.5
148022	TICAM1	toll-like receptor adaptor molecule 1	49	0	24.5
79038	ZFYVE21	zinc finger, FYVE domain containing 21	49	0	24.5
135932	TMEM139	transmembrane protein 139	45	3	24
489	ATP2A3	ATPase, Ca++ transporting, ubiquitous	45	3	24
7468	WHSC1	Wolf-Hirschhorn syndrome candidate 1	43	5	24
55118	CRTAC1	cartilage acidic protein 1	43	5	24
81887	LAS1L	LAS1-like (S. cerevisiae)	43	5	24
285282	RABL3	RAB, member of RAS oncogene family-like 3	42	6	24
632	BGLAP	bone gamma-carboxyglutamate (gla) protein (osteocalcin)	41	7	24
151230	KLHL23	kelch-like 23 (Drosophila)	40	8	24
91833	WDR20	WD repeat domain 20	39	9	24
197335	WDR90	WD repeat domain 90	35	13	24
150684	COMMD1	copper metabolism (Murr1) domain containing 1	35	13	24
23093	TTLL5	tubulin tyrosine ligase-like family, member 5	35	13	24
7786	MAP3K12	mitogen-activated protein kinase kinase kinase 12	35	13	24
84247	LDOC1L	leucine zipper, down-regulated in cancer 1-like	35	13	24
80164	FLJ22184	hypothetical protein FLJ22184	32	16	24
55654	TMEM127	transmembrane protein 127	31	17	24
84263	HSDL2	hydroxysteroid dehydrogenase like 2	29	19	24
9101	USP8	ubiquitin specific peptidase 8	29	19	24
22834	ZNF652	zinc finger protein 652	28	20	24
51454	GULP1	GULP, engulfment adaptor PTB domain containing 1	27	21	24
51547	SIRT7	sirtuin (silent mating type information regulation 2 homolog) 7	25	23	24
11311	VPS45	vacuolar protein sorting 45 homolog (S. cerevisiae)	23	25	24
83444	ZNHIT4	zinc finger, HIT type 4	22	26	24
25814	ATXN10	ataxin 10	20	28	24
10956	OS9	amplified in osteosarcoma	20	28	24
79661	NEIL1	nei endonuclease VIII-like 1 (E. coli)	20	28	24
10100	TSPAN2	tetraspanin 2	19	29	24
8611	PPAP2A	phosphatidic acid phosphatase type 2A	16	32	24
55503	TRPV6	transient receptor potential cation channel, subfamily V, men	10	38	24
79366	NSBP1	nucleosomal binding protein 1	8	40	24
6727	SRP14	signal recognition particle 14kDa (homologous Alu RNA bind	8	40	24
6302	TSPAN31	tetraspanin 31	4	44	24
1388	CREBL1	cAMP responsive element binding protein-like 1	2	46	24
25793	FBXO7	F-box protein 7	2	46	24
25937	WWTR1	WW domain containing transcription regulator 1	0	48	24
57616	TSHZ3	teashirt family zinc finger 3	0	48	24
29097	CNIH4	cornichon homolog 4 (Drosophila)	0	48	24
8533	COPS3	COP9 constitutive photomorphogenic homolog subunit 3 (Ar	0	48	24
388524	LOC388524	similar to Laminin receptor 1	0	48	24
22998	DKFZP686A	hypothetical protein	48	0	24
8934	RAB7L1	RAB7, member RAS oncogene family-like 1	48	0	24
54958	TMEM160	transmembrane protein 160	48	0	24
123264	OSTbeta	organic solute transporter beta	48	0	24
4942	OAT	ornithine aminotransferase (gyrate atrophy)	48	0	24
4891	SLC11A2	solute carrier family 11 (proton-coupled divalent metal ion tra	48	0	24
10226	M6PRBP1	mannose-6-phosphate receptor binding protein 1	48	0	24
81608	FIP1L1	FIP1 like 1 (S. cerevisiae)	48	0	24

10410	IFITM3	interferon induced transmembrane protein 3 (1-8U)	48	0	24
27237	ARHGEF16	Rho guanine exchange factor (GEF) 16	48	0	24
122830	NAT12	N-acetyltransferase 12	48	0	24
153527	ZMAT2	zinc finger, matrin type 2	45	2	23.5
23649	POLA2	polymerase (DNA directed), alpha 2 (70kD subunit)	44	3	23.5
3728	JUP	junction plakoglobin	39	8	23.5
51194	IPO11	importin 11	39	8	23.5
84640	USP38	ubiquitin specific peptidase 38	38	9	23.5
8831	SYNGAP1	synaptic Ras GTPase activating protein 1 homolog (rat)	38	9	23.5
55466	DNAJA4	DnaJ (Hsp40) homolog, subfamily A, member 4	33	14	23.5
1819	DRG2	developmentally regulated GTP binding protein 2	32	15	23.5
5586	PKN2	protein kinase N2	26	21	23.5
84162	KIAA1109	KIAA1109	24	23	23.5
136227	EMID2	EMI domain containing 2	21	26	23.5
55790	ChGn	chondroitin beta1,4 N-acetylgalactosaminyltransferase	19	28	23.5
1836	SLC26A2	solute carrier family 26 (sulfate transporter), member 2	18	29	23.5
2235	FECH	ferrochelatase (protoporphyria)	18	29	23.5
4209	MEF2D	MADS box transcription enhancer factor 2, polypeptide D (m	16	31	23.5
29057	TMEM29	transmembrane protein 29	16	31	23.5
51663	ZFR	zinc finger RNA binding protein	15	32	23.5
23768	FLRT2	fibronectin leucine rich transmembrane protein 2	14	33	23.5
2963	GTF2F2	general transcription factor IIF, polypeptide 2, 30kDa	13	34	23.5
1977	EIF4E	eukaryotic translation initiation factor 4E	12	35	23.5
3454	IFNAR1	interferon (alpha, beta and omega) receptor 1	10	37	23.5
83937	RASSF4	Ras association (RalGDS/AF-6) domain family 4	8	39	23.5
78997	GDAP1L1	ganglioside-induced differentiation-associated protein 1-like	6	41	23.5
84440	RAB11FIP4	RAB11 family interacting protein 4 (class II)	6	41	23.5
2892	GRIA3	glutamate receptor, ionotrophic, AMPA 3	0	47	23.5
80005	DOCK5	dedicator of cytokinesis 5	0	47	23.5
5355	PLP2	proteolipid protein 2 (colonic epithelium-enriched)	0	47	23.5
5903	RANBP2	RAN binding protein 2	0	47	23.5
84792	MGC12966	hypothetical protein LOC84792	47	0	23.5
203069	R3HCC1	R3H domain and coiled-coil containing 1	47	0	23.5
26507	CNNM1	cyclin M1	47	0	23.5
11129	SFRS16	splicing factor, arginine/serine-rich 16	47	0	23.5
54476	TRIAD3	TRIAD3 protein	47	0	23.5
10809	STARD10	START domain containing 10	47	0	23.5
10866	HCP5	HLA complex P5	47	0	23.5
1968	EIF2S3	eukaryotic translation initiation factor 2, subunit 3 gamma, 52	47	0	23.5
8459	TPST2	tyrosylprotein sulfotransferase 2	47	0	23.5
27316	RBMX	RNA binding motif protein, X-linked	47	0	23.5
8443	GNPAT	glyceronephosphate O-acyltransferase	47	0	23.5
829	CAPZA1	capping protein (actin filament) muscle Z-line, alpha 1	44	2	23
10162	MBOAT5	membrane bound O-acyltransferase domain containing 5	44	2	23
58508	MLL3	myeloid/lymphoid or mixed-lineage leukemia 3	43	3	23
10432	RBM14	RNA binding motif protein 14	43	3	23
84311	MRPL45	mitochondrial ribosomal protein L45	40	6	23
55132	LARP2	La ribonucleoprotein domain family, member 2	40	6	23
84886	C1orf198	chromosome 1 open reading frame 198	32	14	23
6574	SLC20A1	solute carrier family 20 (phosphate transporter), member 1	30	16	23
79001	VKORC1	vitamin K epoxide reductase complex, subunit 1	30	16	23

57176	VARSL	valyl-tRNA synthetase like	28	18	23
2923	PDIA3	protein disulfide isomerase family A, member 3	28	18	23
55178	RNMTL1	RNA methyltransferase like 1	21	25	23
26504	CNNM4	cyclin M4	20	26	23
4299	AFF1	AF4/FMR2 family, member 1	18	28	23
3930	LBR	lamin B receptor	18	28	23
27013	C2orf24	chromosome 2 open reading frame 24	18	28	23
64844	MAR7	membrane-associated ring finger (C3HC4) 7	17	29	23
285368	PRRT3	proline-rich transmembrane protein 3	15	31	23
23428	SLC7A8	solute carrier family 7 (cationic amino acid transporter, y+ sy	15	31	23
57707	KIAA1609	KIAA1609	14	32	23
813	CALU	calumenin	12	34	23
9921	RNF10	ring finger protein 10	12	34	23
4668	NAGA	N-acetylgalactosaminidase, alpha-	12	34	23
1203	CLN5	ceroid-lipofuscinosis, neuronal 5	10	36	23
391102	LOC391102	similar to 60S acidic ribosomal protein P0 (L10E)	10	36	23
4515	MTCP1	mature T-cell proliferation 1	10	36	23
29780	PARVB	parvin, beta	10	36	23
9236	CCPG1	cell cycle progression 1	8	38	23
80115	BAIAP2L2	BAI1-associated protein 2-like 2	6	40	23
221302	C6orf113	chromosome 6 open reading frame 113	4	42	23
79896	THNSL1	threonine synthase-like 1 (bacterial)	4	42	23
283587	LOC283587	hypothetical protein LOC283587	2	44	23
29107	NXT1	NTF2-like export factor 1	0	46	23
342979	LOC342979	hypothetical LOC342979	0	46	23
6955	TRA@	T cell receptor alpha locus	0	46	23
768	CA9	carbonic anhydrase IX	0	46	23
9410	WDR57	WD repeat domain 57 (U5 snRNP specific)	0	46	23
2624	GATA2	GATA binding protein 2	0	46	23
1045	CDX2	caudal type homeobox transcription factor 2	0	46	23
10276	NET1	neuroepithelial cell transforming gene 1	0	46	23
4678	NASP	nuclear autoantigenic sperm protein (histone-binding)	0	46	23
161823	ADAL	adenosine deaminase-like	0	46	23
11074	TRIM31	tripartite motif-containing 31	0	46	23
7223	TRPC4	transient receptor potential cation channel, subfamily C, mer	0	46	23
285116	AHCTF1P	AT hook containing transcription factor 1 pseudogene	0	46	23
91683	SYT12	synaptotagmin XII	0	46	23
6183	MRPS12	mitochondrial ribosomal protein S12	0	46	23
56951	C5orf15	chromosome 5 open reading frame 15	0	46	23
55084	FLJ10159	hypothetical protein FLJ10159	0	46	23
23246	BOP1	block of proliferation 1	0	46	23
23234	DNAJC9	DnaJ (Hsp40) homolog, subfamily C, member 9	0	46	23
94097	SFXN5	sideroflexin 5	0	46	23
28960	DCPS	decapping enzyme, scavenger	46	0	23
4976	OPA1	optic atrophy 1 (autosomal dominant)	46	0	23
5371	PML	promyelocytic leukemia	46	0	23
115106	CCDC5	coiled-coil domain containing 5 (spindle associated)	46	0	23
2966	GTF2H2	general transcription factor IIH, polypeptide 2, 44kDa	46	0	23
29956	LASS2	LAG1 homolog, ceramide synthase 2 (S. cerevisiae)	46	0	23
1892	ECHS1	enoyl Coenzyme A hydratase, short chain, 1, mitochondrial	46	0	23
7456	WIPF1	WAS/WASL interacting protein family, member 1	46	0	23

6927	TCF1	transcription factor 1, hepatic; LF-B1, hepatic nuclear factor (	46	0	23
7319	UBE2A	ubiquitin-conjugating enzyme E2A (RAD6 homolog)	46	0	23
91179	SCARF2	scavenger receptor class F, member 2	43	2	22.5
84450	ZNF512	zinc finger protein 512	42	3	22.5
4904	YBX1	Y box binding protein 1	40	5	22.5
403340	MGC70870	hypothetical LOC403340	32	13	22.5
84449	ZNF333	zinc finger protein 333	31	14	22.5
10057	ABCC5	ATP-binding cassette, sub-family C (CFTR/MRP), member 5	25	20	22.5
55197	P15RS	hypothetical protein FLJ10656	24	21	22.5
9076	CLDN1	claudin 1	22	23	22.5
8087	FXR1	fragile X mental retardation, autosomal homolog 1	20	25	22.5
55731	C17orf63	chromosome 17 open reading frame 63	19	26	22.5
501	ALDH7A1	aldehyde dehydrogenase 7 family, member A1	19	26	22.5
5271	SERPINB8	serpin peptidase inhibitor, clade B (ovalbumin), member 8	17	28	22.5
6416	MAP2K4	mitogen-activated protein kinase kinase 4	16	29	22.5
9821	RB1CC1	RB1-inducible coiled-coil 1	15	30	22.5
11167	FSTL1	follistatin-like 1	0	45	22.5
4659	PPP1R12A	protein phosphatase 1, regulatory (inhibitor) subunit 12A	0	45	22.5
56650	CLDND1	claudin domain containing 1	0	45	22.5
1741	DLG3	discs, large homolog 3 (neuroendocrine-dlg, Drosophila)	0	45	22.5
2710	GK	glycerol kinase	0	45	22.5
9731	KIAA0562	KIAA0562	0	45	22.5
23641	LDOC1	leucine zipper, down-regulated in cancer 1	0	45	22.5
678	ZFP36L2	zinc finger protein 36, C3H type-like 2	45	0	22.5
58489	LOC58489	hypothetical protein from EUROIMAGE 588495	45	0	22.5
90	ACVR1	activin A receptor, type I	45	0	22.5
79699	ZYG11B	zyg-11 homolog B (C. elegans)	45	0	22.5
7918	BAT4	HLA-B associated transcript 4	45	0	22.5
6117	RPA1	replication protein A1, 70kDa	45	0	22.5
29916	SNX11	sorting nexin 11	45	0	22.5
84168	ANTXR1	anthrax toxin receptor 1	45	0	22.5
51696	HECA	headcase homolog (Drosophila)	45	0	22.5
1290	COL5A2	collagen, type V, alpha 2	45	0	22.5
9501	RPH3AL	rabphilin 3A-like (without C2 domains)	45	0	22.5
10318	TNIP1	TNFAIP3 interacting protein 1	42	2	22
79892	C10orf119	chromosome 10 open reading frame 119	41	3	22
54880	BCOR	BCL6 co-repressor	41	3	22
8895	CPNE3	copine III	39	5	22
645460	FLJ44342	hypothetical LOC645460	39	5	22
9706	ULK2	unc-51-like kinase 2 (C. elegans)	38	6	22
56928	SPPL2B	signal peptide peptidase-like 2B	35	9	22
23011	RAB21	RAB21, member RAS oncogene family	33	11	22
127703	FLJ38984	hypothetical protein FLJ38984	32	12	22
51720	UIMC1	ubiquitin interaction motif containing 1	29	15	22
9559	VPS26A	vacuolar protein sorting 26 homolog A (yeast)	29	15	22
51155	HN1	hematological and neurological expressed 1	27	17	22
79571	GCC1	GRIP and coiled-coil domain containing 1	26	18	22
10287	RGS19	regulator of G-protein signalling 19	24	20	22
30844	EHD4	EH-domain containing 4	24	20	22
64864	RFXDC2	regulatory factor X domain containing 2	22	22	22
190	NR0B1	nuclear receptor subfamily 0, group B, member 1	20	24	22

10788	IQGAP2	IQ motif containing GTPase activating protein 2	18	26	22
5704	PSMC4	proteasome (prosome, macropain) 26S subunit, ATPase, 4	16	28	22
57609	DIP2B	DIP2 disco-interacting protein 2 homolog B (Drosophila)	6	38	22
128637	TBC1D20	TBC1 domain family, member 20	4	40	22
10144	FAM13A1	family with sequence similarity 13, member A1	4	40	22
345222	LOC345222	hypothetical gene supported by BC043530	0	44	22
23510	KCTD2	potassium channel tetramerisation domain containing 2	0	44	22
10295	BCKDK	branched chain ketoacid dehydrogenase kinase	0	44	22
10010	TANK	TRAF family member-associated NFKB activator	0	44	22
23508	TTC9	tetratricopeptide repeat domain 9	0	44	22
84871	AGBL4	ATP/GTP binding protein-like 4	0	44	22
6768	ST14	suppression of tumorigenicity 14 (colon carcinoma)	0	44	22
53904	MYO3A	myosin IIIA	0	44	22
80274	SCUBE1	signal peptide, CUB domain, EGF-like 1	0	44	22
8675	STX16	syntaxin 16	0	44	22
57180	ACTR3B	ARP3 actin-related protein 3 homolog B (yeast)	0	44	22
9869	SETDB1	SET domain, bifurcated 1	0	44	22
5585	PKN1	protein kinase N1	0	44	22
79870	BAALC	brain and acute leukemia, cytoplasmic	44	0	22
148327	CREB3L4	cAMP responsive element binding protein 3-like 4	44	0	22
7553	ZNF7	zinc finger protein 7	44	0	22
10328	COX4NB	COX4 neighbor	44	0	22
2330	FMO5	flavin containing monooxygenase 5	44	0	22
5331	PLCB3	phospholipase C, beta 3 (phosphatidylinositol-specific)	44	0	22
6780	STAU1	staufen, RNA binding protein, homolog 1 (Drosophila)	44	0	22
137392	FAM92A1	family with sequence similarity 92, member A1	44	0	22
54978	C2orf18	chromosome 2 open reading frame 18	44	0	22
11147	HHLA3	HERV-H LTR-associating 3	44	0	22
90990	KIFC2	kinesin family member C2	44	0	22
57035	C1orf63	chromosome 1 open reading frame 63	44	0	22
3936	LCP1	lymphocyte cytosolic protein 1 (L-plastin)	40	3	21.5
2913	GRM3	glutamate receptor, metabotropic 3	40	3	21.5
4645	MYO5B	myosin VB	38	5	21.5
80004	RBM35B	RNA binding motif protein 35B	37	6	21.5
9862	THRAP4	thyroid hormone receptor associated protein 4	35	8	21.5
10957	PNRC1	proline-rich nuclear receptor coactivator 1	32	11	21.5
2289	FKBP5	FK506 binding protein 5	26	17	21.5
284312	ZSCAN1	zinc finger and SCAN domain containing 1	25	18	21.5
83547	RILP	Rab interacting lysosomal protein	23	20	21.5
23580	CDC42EP4	CDC42 effector protein (Rho GTPase binding) 4	23	20	21.5
10228	STX6	syntaxin 6	22	21	21.5
55422	ZNF331	zinc finger protein 331	18	25	21.5
54987	C1orf123	chromosome 1 open reading frame 123	17	26	21.5
80324	PUS1	pseudouridylate synthase 1	15	28	21.5
90843	TCEAL8	transcription elongation factor A (SII)-like 8	12	31	21.5
8994	LIMD1	LIM domains containing 1	12	31	21.5
9896	KIAA0274	KIAA0274	10	33	21.5
254827	NAALADL2	N-acetylated alpha-linked acidic dipeptidase-like 2	8	35	21.5
6731	SRP72	signal recognition particle 72kDa	4	39	21.5
727897	MUC5B	mucin 5B, oligomeric mucus/gel-forming	2	41	21.5
7289	TULP3	tubby like protein 3	2	41	21.5

65987	KCTD14	potassium channel tetramerisation domain containing 14	2	41	21.5
7697	ZNF138	zinc finger protein 138	0	43	21.5
7629	ZNF76	zinc finger protein 76 (expressed in testis)	0	43	21.5
55035	NOL8	nucleolar protein 8	0	43	21.5
144871	LOC144871	hypothetical protein LOC144871	0	43	21.5
79832	QSER1	glutamine and serine rich 1	43	0	21.5
1600	DAB1	disabled homolog 1 (Drosophila)	43	0	21.5
84074	QRICH2	glutamine rich 2	43	0	21.5
150519	FLJ30428	similar to hypothetical protein A230046P18; cDNA sequence	43	0	21.5
55353	LAPTM4B	lysosomal associated protein transmembrane 4 beta	43	0	21.5
388789	LOC388789	hypothetical gene supported by AF147354	43	0	21.5
80028	FBXL18	F-box and leucine-rich repeat protein 18	43	0	21.5
8536	CAMK1	calcium/calmodulin-dependent protein kinase I	43	0	21.5
57572	DOCK6	dedicator of cytokinesis 6	43	0	21.5
1349	COX7B	cytochrome c oxidase subunit VIIb	43	0	21.5
7110	TMF1	TATA element modulatory factor 1	43	0	21.5
112869	CCDC101	coiled-coil domain containing 101	43	0	21.5
22916	NCBP2	nuclear cap binding protein subunit 2, 20kDa	43	0	21.5
84181	CHD6	chromodomain helicase DNA binding protein 6	43	0	21.5
55810	FOXJ2	forkhead box J2	43	0	21.5
10396	ATP8A1	ATPase, aminophospholipid transporter (APLT), Class I, typ	40	2	21
7919	BAT1	HLA-B associated transcript 1	39	3	21
124491	TMEM170	transmembrane protein 170	39	3	21
23178	PASK	PAS domain containing serine/threonine kinase	37	5	21
168620	BHLHB8	basic helix-loop-helix domain containing, class B, 8	37	5	21
400961	KIAA1155	KIAA1155 protein	37	5	21
81567	TXNDC5	thioredoxin domain containing 5	35	7	21
55603	FAM46A	family with sequence similarity 46, member A	35	7	21
171586	ABHD3	abhydrolase domain containing 3	31	11	21
221935	SDK1	sidekick homolog 1 (chicken)	30	12	21
5089	PBX2	pre-B-cell leukemia transcription factor 2	30	12	21
10559	SLC35A1	solute carrier family 35 (CMP-sialic acid transporter), membe	28	14	21
80308	FLAD1	FAD1 flavin adenine dinucleotide synthetase homolog (S. ce	24	18	21
9507	ADAMTS4	ADAM metallopeptidase with thrombospondin type 1 motif, 4	24	18	21
7268	TTC4	tetratricopeptide repeat domain 4	24	18	21
118788	PIK3AP1	phosphoinositide-3-kinase adaptor protein 1	23	19	21
5494	PPM1A	protein phosphatase 1A (formerly 2C), magnesium-depende	22	20	21
2302	FOXJ1	forkhead box J1	16	26	21
55425	KIAA1704	KIAA1704	16	26	21
55321	C20orf46	chromosome 20 open reading frame 46	14	28	21
10979	PLEKHC1	pleckstrin homology domain containing, family C (with FERM	12	30	21
23568	ARL2BP	ADP-ribosylation factor-like 2 binding protein	10	32	21
6732	SRPK1	SFRS protein kinase 1	8	34	21
84079	ANKRD27	ankyrin repeat domain 27 (VPS9 domain)	8	34	21
55236	UBE1L2	ubiquitin-activating enzyme E1-like 2	6	36	21
51374	C2orf28	chromosome 2 open reading frame 28	6	36	21
6666	SOX12	SRY (sex determining region Y)-box 12	4	38	21
22984	PDCD11	programmed cell death 11	2	40	21
23506	KIAA0240	KIAA0240	0	42	21
84034	EMILIN2	elastin microfibril interfacer 2	0	42	21
677814	SNORA31	small nucleolar RNA, H/ACA box 31	0	42	21

84915	C12orf34	chromosome 12 open reading frame 34	0	42	21
54205	CYCS	cytochrome c, somatic	0	42	21
342732	LOC342732	similar to keratin 8	0	42	21
9342	SNAP29	synaptosomal-associated protein, 29kDa	0	42	21
5914	RARA	retinoic acid receptor, alpha	0	42	21
170575	GIMAP1	GTPase, IMAP family member 1	0	42	21
6396	SEC13	SEC13 homolog (S. cerevisiae)	0	42	21
3382	ICA1	islet cell autoantigen 1, 69kDa	0	42	21
3954	LETM1	leucine zipper-EF-hand containing transmembrane protein 1	0	42	21
55799	CACNA2D3	calcium channel, voltage-dependent, alpha 2/delta 3 subunit	0	42	21
8925	HERC1	hect (homologous to the E6-AP (UBE3A) carboxyl terminus)	0	42	21
2131	EXT1	exostoses (multiple) 1	0	42	21
643749	LOC643749	hypothetical LOC643749	0	42	21
125170	SMCR7	Smith-Magenis syndrome chromosome region, candidate 7	0	42	21
25807	RHBDD3	rhomboid domain containing 3	42	0	21
55139	ANKZF1	ankyrin repeat and zinc finger domain containing 1	42	0	21
79734	KCTD17	potassium channel tetramerisation domain containing 17	42	0	21
84105	PCBD2	pterin-4 alpha-carbinolamine dehydratase/dimerization cofac	42	0	21
26136	TES	testis derived transcript (3 LIM domains)	42	0	21
10449	ACAA2	acetyl-Coenzyme A acyltransferase 2 (mitochondrial 3-oxoac	42	0	21
51710	ZNF44	zinc finger protein 44	42	0	21
1786	DNMT1	DNA (cytosine-5-)-methyltransferase 1	42	0	21
123920	CMTM3	CKLF-like MARVEL transmembrane domain containing 3	39	2	20.5
1756	DMD	dystrophin (muscular dystrophy, Duchenne and Becker types	39	2	20.5
9791	PTDSS1	phosphatidylserine synthase 1	38	3	20.5
54497	KIAA1414	KIAA1414 protein	38	3	20.5
55691	FRMD4A	FERM domain containing 4A	37	4	20.5
29092	HSPC157	HSPC157 protein	36	5	20.5
51119	SBDS	Shwachman-Bodian-Diamond syndrome	35	6	20.5
3714	JAG2	jagged 2	35	6	20.5
54906	C10orf18	chromosome 10 open reading frame 18	34	7	20.5
7337	UBE3A	ubiquitin protein ligase E3A (human papilloma virus E6-asso	33	8	20.5
1114	CHGB	chromogranin B (secretogranin 1)	33	8	20.5
3665	IRF7	interferon regulatory factor 7	30	11	20.5
83938	C10orf11	chromosome 10 open reading frame 11	29	12	20.5
130574	LYPD6	LY6/PLAUR domain containing 6	29	12	20.5
7852	CXCR4	chemokine (C-X-C motif) receptor 4	28	13	20.5
51191	HERC5	hect domain and RLD 5	26	15	20.5
11236	RNF139	ring finger protein 139	26	15	20.5
9125	RQCD1	RCD1 required for cell differentiation1 homolog (S. pombe)	23	18	20.5
4214	MAP3K1	mitogen-activated protein kinase kinase kinase 1	21	20	20.5
2803	GOLGA4	golgi autoantigen, golgin subfamily a, 4	21	20	20.5
3299	HSF4	heat shock transcription factor 4	20	21	20.5
6747	SSR3	signal sequence receptor, gamma (translocon-associated pr	19	22	20.5
4673	NAP1L1	nucleosome assembly protein 1-like 1	15	26	20.5
9863	MAGI2	membrane associated guanylate kinase, WW and PDZ doma	14	27	20.5
84993	UBL7	ubiquitin-like 7 (bone marrow stromal cell-derived)	13	28	20.5
23467	NPTXR	neuronal pentraxin receptor	12	29	20.5
128308	MRPL55	mitochondrial ribosomal protein L55	10	31	20.5
9852	EPM2AIP1	EPM2A (laforin) interacting protein 1	10	31	20.5
954	ENTPD2	ectonucleoside triphosphate diphosphohydrolase 2	10	31	20.5

9739	SETD1A	SET domain containing 1A	8	33	20.5
414	ARSD	arylsulfatase D	6	35	20.5
29110	TBK1	TANK-binding kinase 1	4	37	20.5
90324	CCDC97	coiled-coil domain containing 97	2	39	20.5
23550	PSD4	pleckstrin and Sec7 domain containing 4	2	39	20.5
10255	HCG9	HLA complex group 9	0	41	20.5
9586	CREB5	cAMP responsive element binding protein 5	0	41	20.5
729060	LOC729060	similar to High mobility group protein B1 (High mobility grouբ	0	41	20.5
348013	FAM70B	family with sequence similarity 70, member B	0	41	20.5
647215	LOC647215	hypothetical LOC647215	0	41	20.5
2110	ETFDH	electron-transferring-flavoprotein dehydrogenase	0	41	20.5
55591	VEZT	vezatin, adherens junctions transmembrane protein	0	41	20.5
25861	DFNB31	deafness, autosomal recessive 31	0	41	20.5
11142	PKIG	protein kinase (cAMP-dependent, catalytic) inhibitor gamma	0	41	20.5
650794	LOC650794	similar to FRAS1-related extracellular matrix protein 2 precu	0	41	20.5
23193	GANAB	glucosidase, alpha; neutral AB	41	0	20.5
92922	CCDC102A	coiled-coil domain containing 102A	41	0	20.5
6347	CCL2	chemokine (C-C motif) ligand 2	41	0	20.5
91782	CHMP7	CHMP family, member 7	41	0	20.5
4258	MGST2	microsomal glutathione S-transferase 2	41	0	20.5
25791	NGEF	neuronal guanine nucleotide exchange factor	41	0	20.5
84274	COQ5	coenzyme Q5 homolog, methyltransferase (S. cerevisiae)	41	0	20.5
7507	XPA	xeroderma pigmentosum, complementation group A	41	0	20.5
9674	KIAA0040	KIAA0040	41	0	20.5
4677	NARS	asparaginyl-tRNA synthetase	41	0	20.5
79838	TMC5	transmembrane channel-like 5	37	3	20
22882	ZHX2	zinc fingers and homeoboxes 2	37	3	20
2281	FKBP1B	FK506 binding protein 1B, 12.6 kDa	35	5	20
23098	SARM1	sterile alpha and TIR motif containing 1	32	8	20
9554	SEC22B	SEC22 vesicle trafficking protein homolog B (S. cerevisiae)	31	9	20
6674	SPAG1	sperm associated antigen 1	29	11	20
23265	EXOC7	exocyst complex component 7	29	11	20
324	APC	adenomatosis polyposis coli	25	15	20
26050	SLITRK5	SLIT and NTRK-like family, member 5	24	16	20
64857	PLEKHG2	pleckstrin homology domain containing, family G (with RhoG	22	18	20
666	BOK	BCL2-related ovarian killer	20	20	20
7071	KLF10	Kruppel-like factor 10	20	20	20
379025	FLJ31306	hypothetical protein FLJ31306	20	20	20
10152	ABI2	abl interactor 2	16	24	20
152815	THAP6	THAP domain containing 6	14	26	20
79058	ASPSCR1	alveolar soft part sarcoma chromosome region, candidate 1	12	28	20
6550	SLC9A3	solute carrier family 9 (sodium/hydrogen exchanger), membe	6	34	20
79145	CHCHD7	coiled-coil-helix-coiled-coil-helix domain containing 7	6	34	20
23247	KIAA0556	KIAA0556	4	36	20
255104	TMCO4	transmembrane and coiled-coil domains 4	4	36	20
10841	FTCD	formiminotransferase cyclodeaminase	4	36	20
2259	FGF14	fibroblast growth factor 14	2	38	20
6576	SLC25A1	solute carrier family 25 (mitochondrial carrier; citrate transpo	2	38	20
440600	LOC440600	hypothetical gene supported by BC053344	0	40	20
25875	LETMD1	LETM1 domain containing 1	0	40	20
57704	GBA2	glucosidase, beta (bile acid) 2	0	40	20

320	APBA1	amyloid beta (A4) precursor protein-binding, family A, memb	0	40	20
10615	SPAG5	sperm associated antigen 5	0	40	20
32	ACACB	acetyl-Coenzyme A carboxylase beta	0	40	20
6457	SH3GL3	SH3-domain GRB2-like 3	0	40	20
56256	SERTAD4	SERTA domain containing 4	0	40	20
57053	CHRNA10	cholinergic receptor, nicotinic, alpha 10	0	40	20
26508	HEYL	hairy/enhancer-of-split related with YRPW motif-like	40	0	20
200373	MGC33657	similar to hypothetical protein	40	0	20
199990	C1orf86	chromosome 1 open reading frame 86	40	0	20
30	ACAA1	acetyl-Coenzyme A acyltransferase 1 (peroxisomal 3-oxoacy	40	0	20
55176	SEC61A2	Sec61 alpha 2 subunit (S. cerevisiae)	40	0	20
55166	CENPQ	centromere protein Q	40	0	20
26262	TSPAN17	tetraspanin 17	40	0	20
79970	ZNF767	zinc finger family member 767	37	2	19.5
51086	TNNI3K	TNNI3 interacting kinase	37	2	19.5
51763	SKIP	skeletal muscle and kidney enriched inositol phosphatase	33	6	19.5
26985	AP3M1	adaptor-related protein complex 3, mu 1 subunit	32	7	19.5
5789	PTPRD	protein tyrosine phosphatase, receptor type, D	30	9	19.5
2184	FAH	fumarylacetoacetate hydrolase (fumarylacetoacetase)	28	11	19.5
10044	SH2D3C	SH2 domain containing 3C	24	15	19.5
65268	WNK2	WNK lysine deficient protein kinase 2	22	17	19.5
6934	TCF7L2	transcription factor 7-like 2 (T-cell specific, HMG-box)	20	19	19.5
157	ADRBK2	adrenergic, beta, receptor kinase 2	19	20	19.5
55075	UACA	uveal autoantigen with coiled-coil domains and ankyrin repea	18	21	19.5
10902	BRD8	bromodomain containing 8	17	22	19.5
25904	CNOT10	CCR4-NOT transcription complex, subunit 10	16	23	19.5
51499	TRIAP1	TP53 regulated inhibitor of apoptosis 1	10	29	19.5
5175	PECAM1	platelet/endothelial cell adhesion molecule (CD31 antigen)	6	33	19.5
51340	CRNKL1	Crn, crooked neck-like 1 (Drosophila)	2	37	19.5
5074	PAWR	PRKC, apoptosis, WT1, regulator	2	37	19.5
1292	COL6A2	collagen, type VI, alpha 2	0	39	19.5
27086	FOXP1	forkhead box P1	0	39	19.5
1396	CRIP1	cysteine-rich protein 1 (intestinal)	0	39	19.5
7544	ZFY	zinc finger protein, Y-linked	0	39	19.5
2161	F12	coagulation factor XII (Hageman factor)	0	39	19.5
643837	LOC643837	hypothetical protein LOC643837	0	39	19.5
26086	GPSM1	G-protein signalling modulator 1 (AGS3-like, C. elegans)	0	39	19.5
8786	RGS11	regulator of G-protein signalling 11	0	39	19.5
6672	SP100	SP100 nuclear antigen	0	39	19.5
3799	KIF5B	kinesin family member 5B	0	39	19.5
29766	TMOD3	tropomodulin 3 (ubiquitous)	0	39	19.5
6775	STAT4	signal transducer and activator of transcription 4	0	39	19.5
55012	C14orf10	chromosome 14 open reading frame 10	0	39	19.5
400451	LOC400451	hypothetical gene supported by AK075564; BC060873	0	39	19.5
6318	SERPINB4	serpin peptidase inhibitor, clade B (ovalbumin), member 4	0	39	19.5
282974	STK32C	serine/threonine kinase 32C	0	39	19.5
4664	NAB1	NGFI-A binding protein 1 (EGR1 binding protein 1)	39	0	19.5
55760	DHX32	DEAH (Asp-Glu-Ala-His) box polypeptide 32	39	0	19.5
55240	STEAP3	STEAP family member 3	39	0	19.5
51441	YTHDF2	YTH domain family, member 2	39	0	19.5
1576	CYP3A4	cytochrome P450, family 3, subfamily A, polypeptide 4	39	0	19.5

81557	MAGED4	melanoma antigen family D, 4	39	0	19.5
729467	LOC729467	hypothetical protein LOC729467	39	0	19.5
5118	PCOLCE	procollagen C-endopeptidase enhancer	39	0	19.5
340273	ABCB5	ATP-binding cassette, sub-family B (MDR/TAP), member 5	39	0	19.5
57505	AARSL	alanyl-tRNA synthetase like	39	0	19.5
266977	GPR110	G protein-coupled receptor 110	39	0	19.5
285176	LOC285176	similar to ribosomal protein L10	39	0	19.5
27352	RUTBC3	RUN and TBC1 domain containing 3	35	3	19
7043	TGFB3	transforming growth factor, beta 3	35	3	19
23236	PLCB1	phospholipase C, beta 1 (phosphoinositide-specific)	35	3	19
57095	C1orf128	chromosome 1 open reading frame 128	35	3	19
64968	MRPS6	mitochondrial ribosomal protein S6	33	5	19
5364	PLXNB1	plexin B1	33	5	19
8859	STK19	serine/threonine kinase 19	30	8	19
23673	STX12	syntaxin 12	28	10	19
126433	FBXO27	F-box protein 27	28	10	19
56243	KIAA1217	KIAA1217	27	11	19
5140	PDE3B	phosphodiesterase 3B, cGMP-inhibited	24	14	19
6470	SHMT1	serine hydroxymethyltransferase 1 (soluble)	22	16	19
89876	C3orf15	chromosome 3 open reading frame 15	20	18	19
4675	NAP1L3	nucleosome assembly protein 1-like 3	19	19	19
123036	MTAC2D1	membrane targeting (tandem) C2 domain containing 1	17	21	19
10240	MRPS31	mitochondrial ribosomal protein S31	10	28	19
55112	WDR60	WD repeat domain 60	10	28	19
2622	GAS8	growth arrest-specific 8	4	34	19
4139	MARK1	MAP/microtubule affinity-regulating kinase 1	2	36	19
163049	ZNF791	zinc finger protein 791	2	36	19
144402	CPNE8	copine VIII	0	38	19
9710	KIAA0355	KIAA0355	0	38	19
11062	DUS4L	dihydrouridine synthase 4-like (S. cerevisiae)	0	38	19
55023	PHIP	pleckstrin homology domain interacting protein	0	38	19
345557	PLCXD3	phosphatidylinositol-specific phospholipase C, X domain con	0	38	19
7738	ZNF184	zinc finger protein 184	0	38	19
57494	FAM80B	family with sequence similarity 80, member B	0	38	19
63926	ANKRD5	ankyrin repeat domain 5	0	38	19
347731	LRRTM3	leucine rich repeat transmembrane neuronal 3	38	0	19
26297	SERGEF	secretion regulating guanine nucleotide exchange factor	38	0	19
5813	PURA	purine-rich element binding protein A	38	0	19
4835	NQO2	NAD(P)H dehydrogenase, quinone 2	38	0	19
11201	POLI	polymerase (DNA directed) iota	38	0	19
646762	LOC646762	hypothetical protein LOC646762	38	0	19
29964	C6orf49	chromosome 6 open reading frame 49	38	0	19
10440	TIMM17A	translocase of inner mitochondrial membrane 17 homolog A	38	0	19
10945	KDELR1	KDEL (Lys-Asp-Glu-Leu) endoplasmic reticulum protein rete	38	0	19
7627	ZNF75A	zinc finger protein 75a	38	0	19
91694	LONRF1	LON peptidase N-terminal domain and ring finger 1	38	0	19
78987	CRELD1	cysteine-rich with EGF-like domains 1	38	0	19
10398	MYL9	myosin, light chain 9, regulatory	38	0	19
29934	SNX12	sorting nexin 12	38	0	19
2859	GPR35	G protein-coupled receptor 35	38	0	19
1027	CDKN1B	cyclin-dependent kinase inhibitor 1B (p27, Kip1)	35	2	18.5

64081	PBLD	phenazine biosynthesis-like protein domain containing	35	2	18.5
857	CAV1	caveolin 1, caveolae protein, 22kDa	32	5	18.5
79817	MOBKL2B	MOB1, Mps One Binder kinase activator-like 2B (yeast)	32	5	18.5
9887	SMG7	Smg-7 homolog, nonsense mediated mRNA decay factor (C	29	8	18.5
394	ARHGAP5	Rho GTPase activating protein 5	29	8	18.5
50809	HP1BP3	heterochromatin protein 1, binding protein 3	29	8	18.5
8704	B4GALT2	UDP-Gal:betaGlcNAc beta 1,4- galactosyltransferase, polype	23	14	18.5
57600	KIAA1450	KIAA1450 protein	23	14	18.5
112849	C14orf149	chromosome 14 open reading frame 149	23	14	18.5
29777	ABT1	activator of basal transcription 1	23	14	18.5
10322	SMYD5	SMYD family member 5	23	14	18.5
55622	TTC27	tetratricopeptide repeat domain 27	17	20	18.5
25940	FAM98A	family with sequence similarity 98, member A	15	22	18.5
56851	C15orf24	chromosome 15 open reading frame 24	15	22	18.5
6925	TCF4	transcription factor 4	14	23	18.5
54494	C11orf71	chromosome 11 open reading frame 71	8	29	18.5
10399	GNB2L1	guanine nucleotide binding protein (G protein), beta polypept	8	29	18.5
50804	MYEF2	myelin expression factor 2	8	29	18.5
8334	HIST1H2AC	histone cluster 1, H2ac	8	29	18.5
146542	ZNF688	zinc finger protein 688	8	29	18.5
5257	PHKB	phosphorylase kinase, beta	6	31	18.5
10082	GPC6	glypican 6	4	33	18.5
57862	ZNF410	zinc finger protein 410	0	37	18.5
84901	NFATC2IP	nuclear factor of activated T-cells, cytoplasmic, calcineurin-d	0	37	18.5
221120	ALKBH3	alkB, alkylation repair homolog 3 (E. coli)	0	37	18.5
51130	ASB3	ankyrin repeat and SOCS box-containing 3	0	37	18.5
4644	MYO5A	myosin VA (heavy chain 12, myoxin)	0	37	18.5
126526	C19orf47	chromosome 19 open reading frame 47	0	37	18.5
3858	KRT10	keratin 10 (epidermolytic hyperkeratosis; keratosis palmaris	0	37	18.5
283711	LOC283711	hypothetical protein LOC283711	0	37	18.5
80853	KIAA1718	KIAA1718 protein	0	37	18.5
55016	MAR1	membrane-associated ring finger (C3HC4) 1	0	37	18.5
54148	MRPL39	mitochondrial ribosomal protein L39	0	37	18.5
6573	SLC19A1	solute carrier family 19 (folate transporter), member 1	0	37	18.5
5744	PTHLH	parathyroid hormone-like hormone	0	37	18.5
89796	NAV1	neuron navigator 1	0	37	18.5
10801	SEP9	septin 9	0	37	18.5
27445	PCLO	piccolo (presynaptic cytomatrix protein)	0	37	18.5
54386	TERF2IP	telomeric repeat binding factor 2, interacting protein	0	37	18.5
730427	LOC730427	similar to neuron navigator 2 isoform 2	37	0	18.5
79901	CYBRD1	cytochrome b reductase 1	37	0	18.5
254427	C10orf47	chromosome 10 open reading frame 47	37	0	18.5
9567	GTPBP1	GTP binding protein 1	37	0	18.5
8555	CDC14B	CDC14 cell division cycle 14 homolog B (S. cerevisiae)	37	0	18.5
5631	PRPS1	phosphoribosyl pyrophosphate synthetase 1	37	0	18.5
7148	TNXB	tenascin XB	37	0	18.5
645090	LOC645090	hypothetical LOC645090	37	0	18.5
95	ACY1	aminoacylase 1	37	0	18.5
23616	SH3BP1	SH3-domain binding protein 1	37	0	18.5
1387	CREBBP	CREB binding protein (Rubinstein-Taybi syndrome)	33	3	18
56204	KIAA1370	KIAA1370	33	3	18

26509	FER1L3	fer-1-like 3, myoferlin (C. elegans)	33	3	18
64782	ISG20L1	interferon stimulated exonuclease gene 20kDa-like 1	31	5	18
165918	RNF168	ring finger protein 168	31	5	18
199731	IGSF4C	immunoglobulin superfamily, member 4C	28	8	18
387893	SETD8	SET domain containing (lysine methyltransferase) 8	28	8	18
23091	ZC3H13	zinc finger CCCH-type containing 13	27	9	18
196266	LOC196266	hypothetical protein	26	10	18
129285	CCDC128	coiled-coil domain containing 128	26	10	18
9619	ABCG1	ATP-binding cassette, sub-family G (WHITE), member 1	25	11	18
126075	LOC126075	hypothetical protein LOC126075	21	15	18
79651	RHBDF2	rhomboid 5 homolog 2 (Drosophila)	18	18	18
26130	GAPVD1	GTPase activating protein and VPS9 domains 1	16	20	18
81573	ANKRD13C	ankyrin repeat domain 13C	16	20	18
22990	PCNX	pecanex homolog (Drosophila)	14	22	18
23161	SNX13	sorting nexin 13	12	24	18
23324	MAN2B2	mannosidase, alpha, class 2B, member 2	12	24	18
6776	STAT5A	signal transducer and activator of transcription 5A	10	26	18
11034	DSTN	destrin (actin depolymerizing factor)	8	28	18
118424	UBE2J2	ubiquitin-conjugating enzyme E2, J2 (UBC6 homolog, yeast)	8	28	18
80006	FLJ13611	hypothetical protein FLJ13611	8	28	18
51752	ARTS-1	type 1 tumor necrosis factor receptor shedding aminopeptida	2	34	18
909	CD1A	CD1a molecule	2	34	18
1152	CKB	creatine kinase, brain	2	34	18
8609	KLF7	Kruppel-like factor 7 (ubiquitous)	2	34	18
91316	LOC91316	similar to bK246H3.1 (immunoglobulin lambda-like polypepti	0	36	18
2073	ERCC5	excision repair cross-complementing rodent repair deficiency	0	36	18
135	ADORA2A	adenosine A2a receptor	0	36	18
150962	FLJ32312	hypothetical protein FLJ32312	0	36	18
729578	LOC729578	hypothetical protein LOC729578	0	36	18
9317	PTER	phosphotriesterase related	0	36	18
10643	IGF2BP3	insulin-like growth factor 2 mRNA binding protein 3	0	36	18
727929	LOC727929	hypothetical protein LOC727929	0	36	18
53942	CNTN5	contactin 5	0	36	18
57578	KIAA1409	KIAA1409	0	36	18
2245	FGD1	FYVE, RhoGEF and PH domain containing 1 (faciogenital dy	0	36	18
9913	SUPT7L	suppressor of Ty 7 (S. cerevisiae)-like	0	36	18
2059	EPS8	epidermal growth factor receptor pathway substrate 8	0	36	18
6256	RXRA	retinoid X receptor, alpha	0	36	18
9123	SLC16A3	solute carrier family 16, member 3 (monocarboxylic acid tran	0	36	18
643494	LOC643494	similar to RNA binding motif protein 4B	0	36	18
55719	C10orf6	chromosome 10 open reading frame 6	36	0	18
23334	KIAA0467	KIAA0467	36	0	18
6351	CCL4	chemokine (C-C motif) ligand 4	36	0	18
7047	TGM4	transglutaminase 4 (prostate)	36	0	18
7979	SHFM1	split hand/foot malformation (ectrodactyly) type 1	36	0	18
84549	RBM13	RNA binding motif protein 13	36	0	18
9077	DIRAS3	DIRAS family, GTP-binding RAS-like 3	36	0	18
55971	BAIAP2L1	BAI1-associated protein 2-like 1	36	0	18
645580	FLJ37453	hypothetical protein LOC645580	36	0	18
84765	ZNF577	zinc finger protein 577	36	0	18
79024	MGC5590	hypothetical protein MGC5590	36	0	18

402483	FLJ45340	hypothetical gene supported by AK127273	36	0	18
23276	KLHL18	kelch-like 18 (Drosophila)	36	0	18
55367	LRDD	leucine-rich repeats and death domain containing	33	2	17.5
9464	HAND2	heart and neural crest derivatives expressed 2	33	2	17.5
5286	PIK3C2A	phosphoinositide-3-kinase, class 2, alpha polypeptide	33	2	17.5
51466	EVL	Enah/Vasp-like	33	2	17.5
80315	CPEB4	cytoplasmic polyadenylation element binding protein 4	32	3	17.5
643641	LOC643641	hypothetical protein LOC643641	30	5	17.5
6949	TCOF1	Treacher Collins-Franceschetti syndrome 1	29	6	17.5
2012	EMP1	epithelial membrane protein 1	29	6	17.5
10066	SCAMP2	secretory carrier membrane protein 2	29	6	17.5
54509	RHOF	ras homolog gene family, member F (in filopodia)	27	8	17.5
1080	CFTR	cystic fibrosis transmembrane conductance regulator (ATP-b	26	9	17.5
10520	ZNF211	zinc finger protein 211	25	10	17.5
8602	C4orf9	chromosome 4 open reading frame 9	24	11	17.5
80208	KIAA1840	KIAA1840	24	11	17.5
23227	MAST4	microtubule associated serine/threonine kinase family memb	22	13	17.5
124637	CYB5D1	cytochrome b5 domain containing 1	22	13	17.5
2010	EMD	emerin (Emery-Dreifuss muscular dystrophy)	20	15	17.5
8604	SLC25A12	solute carrier family 25 (mitochondrial carrier, Aralar), memb	15	20	17.5
1653	DDX1	DEAD (Asp-Glu-Ala-Asp) box polypeptide 1	12	23	17.5
54939	COMMD4	COMM domain containing 4	12	23	17.5
151194	FAM119A	family with sequence similarity 119, member A	12	23	17.5
9855	FARP2	FERM, RhoGEF and pleckstrin domain protein 2	12	23	17.5
55209	SETD5	SET domain containing 5	10	25	17.5
8019	BRD3	bromodomain containing 3	10	25	17.5
10154	PLXNC1	plexin C1	10	25	17.5
400120	LOC400120	hypothetical LOC400120	6	29	17.5
154007	C6orf151	chromosome 6 open reading frame 151	6	29	17.5
140459	ASB6	ankyrin repeat and SOCS box-containing 6	4	31	17.5
527	ATP6V0C	ATPase, H+ transporting, lysosomal 16kDa, V0 subunit c	2	33	17.5
23185	LARP5	La ribonucleoprotein domain family, member 5	0	35	17.5
4947	OAZ2	ornithine decarboxylase antizyme 2	0	35	17.5
152926	PPM1K	protein phosphatase 1K (PP2C domain containing)	35	0	17.5
9683	N4BP1	Nedd4 binding protein 1	35	0	17.5
23225	NUP210	nucleoporin 210kDa	35	0	17.5
7049	TGFBR3	transforming growth factor, beta receptor III (betaglycan, 300	35	0	17.5
5373	PMM2	phosphomannomutase 2	35	0	17.5
22897	CEP164	centrosomal protein 164kDa	35	0	17.5
116225	ZMYND19	zinc finger, MYND-type containing 19	35	0	17.5
56142	PCDHA6	protocadherin alpha 6	35	0	17.5
554313	HIST2H4B	histone cluster 2, H4b	35	0	17.5
8463	TEAD2	TEA domain family member 2	35	0	17.5
9877	ZC3H11A	zinc finger CCCH-type containing 11A	35	0	17.5
84067	C11orf56	chromosome 11 open reading frame 56	35	0	17.5
10267	RAMP1	receptor (G protein-coupled) activity modifying protein 1	35	0	17.5
28514	DLL1	delta-like 1 (Drosophila)	35	0	17.5
23676	SMPX	small muscle protein, X-linked	35	0	17.5
7128	TNFAIP3	tumor necrosis factor, alpha-induced protein 3	35	0	17.5
29082	CHMP4A	chromatin modifying protein 4A	35	0	17.5
329	BIRC2	baculoviral IAP repeat-containing 2	35	0	17.5

11007	CCDC85B	coiled-coil domain containing 85B	35	0	17.5
5414	SEP4	septin 4	35	0	17.5
30968	STOML2	stomatin (EPB72)-like 2	35	0	17.5
4430	MYO1B	myosin IB	35	0	17.5
797	CALCB	calcitonin-related polypeptide, beta	35	0	17.5
220136	CCDC11	coiled-coil domain containing 11	35	0	17.5
79724	ZNF768	zinc finger protein 768	35	0	17.5
8799	PEX11B	peroxisomal biogenesis factor 11B	35	0	17.5
1654	DDX3X	DEAD (Asp-Glu-Ala-Asp) box polypeptide 3, X-linked	35	0	17.5
27148	STK36	serine/threonine kinase 36, fused homolog (Drosophila)	35	0	17.5
84083	ZRANB3	zinc finger, RAN-binding domain containing 3	35	0	17.5
5936	RBM4	RNA binding motif protein 4	35	0	17.5
9897	KIAA0196	KIAA0196	35	0	17.5
91283	C9orf30	chromosome 9 open reading frame 30	35	0	17.5
10260	DENND4A	DENN/MADD domain containing 4A	35	0	17.5
9757	MLL4	myeloid/lymphoid or mixed-lineage leukemia 4	35	0	17.5
64786	TBC1D15	TBC1 domain family, member 15	32	2	17
93611	FBXO44	F-box protein 44	29	5	17
54581	SCAND2	SCAN domain containing 2	26	8	17
644075	LOC644075	hypothetical LOC644075	26	8	17
9715	KIAA0773	KIAA0773 gene product	26	8	17
2009	EML1	echinoderm microtubule associated protein like 1	25	9	17
144132	DNHD1	dynein heavy chain domain 1	25	9	17
2539	G6PD	glucose-6-phosphate dehydrogenase	24	10	17
8940	TOP3B	topoisomerase (DNA) III beta	23	11	17
78994	PRR14	proline rich 14	19	15	17
1947	EFNB1	ephrin-B1	18	16	17
28992	LRP16	LRP16 protein	17	17	17
26046	ZNF294	zinc finger protein 294	16	18	17
65094	JMJD4	jumonji domain containing 4	14	20	17
253039	LOC253039	hypothetical protein LOC253039	14	20	17
8648	NCOA1	nuclear receptor coactivator 1	12	22	17
9839	ZFHX1B	zinc finger homeobox 1b	12	22	17
55720	TSR1	TSR1, 20S rRNA accumulation, homolog (S. cerevisiae)	10	24	17
55051	C14orf102	chromosome 14 open reading frame 102	8	26	17
79960	PHF17	PHD finger protein 17	6	28	17
25885	POLR1A	polymerase (RNA) I polypeptide A, 194kDa	6	28	17
55501	CHST12	carbohydrate (chondroitin 4) sulfotransferase 12	6	28	17
24146	CLDN15	claudin 15	2	32	17
63929	XPNPEP3	X-prolyl aminopeptidase (aminopeptidase P) 3, putative	0	34	17
28965	SLC27A6	solute carrier family 27 (fatty acid transporter), member 6	0	34	17
130074	LOC130074	p20	0	34	17
118491	TTC18	tetratricopeptide repeat domain 18	0	34	17
10469	TIMM44	translocase of inner mitochondrial membrane 44 homolog (y	0	34	17
387927	LOC387927	similar to NIMA (never in mitosis gene a)-related expressed	0	34	17
9641	IKBKE	inhibitor of kappa light polypeptide gene enhancer in B-cells,	0	34	17
441951	C20orf199	chromosome 20 open reading frame 199	0	34	17
83450	LRRC48	leucine rich repeat containing 48	0	34	17
51287	CHCHD8	coiled-coil-helix-coiled-coil-helix domain containing 8	0	34	17
55684	C9orf86	chromosome 9 open reading frame 86	0	34	17
55764	IFT122	intraflagellar transport 122 homolog (Chlamydomonas)	0	34	17

115948	MGC20983	hypothetical protein MGC20983	0	34	17
8871	SYNJ2	synaptojanin 2	0	34	17
23008	KIAA0265	KIAA0265 protein	0	34	17
729625	LOC729625	hypothetical protein LOC729625	0	34	17
25759	SHC2	SHC (Src homology 2 domain containing) transforming prote	0	34	17
945	CD33	CD33 molecule	0	34	17
9474	ATG5	ATG5 autophagy related 5 homolog (S. cerevisiae)	0	34	17
92737	DNER	delta/notch-like EGF repeat containing	34	0	17
55589	BMP2K	BMP2 inducible kinase	34	0	17
23466	CBX6	chromobox homolog 6	34	0	17
116986	CENTG1	centaurin, gamma 1	31	2	16.5
79656	C1orf165	chromosome 1 open reading frame 165	30	3	16.5
503542	SPRN	shadow of prion protein homolog (zebrafish)	28	5	16.5
623	BDKRB1	bradykinin receptor B1	28	5	16.5
9878	KIAA0737	KIAA0737	22	11	16.5
39	ACAT2	acetyl-Coenzyme A acetyltransferase 2 (acetoacetyl Coenzy	21	12	16.5
6602	SMARCD1	SWI/SNF related, matrix associated, actin dependent regula	21	12	16.5
8131	C16orf35	chromosome 16 open reading frame 35	20	13	16.5
476	ATP1A1	ATPase, Na+/K+ transporting, alpha 1 polypeptide	20	13	16.5
27235	COQ2	coenzyme Q2 homolog, prenyltransferase (yeast)	20	13	16.5
23030	JMJD2B	jumonji domain containing 2B	16	17	16.5
51765	RP6-213H19	serine/threonine protein kinase MST4	15	18	16.5
6595	SMARCA2	SWI/SNF related, matrix associated, actin dependent regula	12	21	16.5
28988	DBNL	drebrin-like	12	21	16.5
23412	COMMD3	COMM domain containing 3	12	21	16.5
2494	NR5A2	nuclear receptor subfamily 5, group A, member 2	10	23	16.5
54059	C21orf57	chromosome 21 open reading frame 57	10	23	16.5
340481	ZDHHC21	zinc finger, DHHC-type containing 21	8	25	16.5
10865	ARID5A	AT rich interactive domain 5A (MRF1-like)	4	29	16.5
57732	ZFYVE28	zinc finger, FYVE domain containing 28	2	31	16.5
1161	ERCC8	excision repair cross-complementing rodent repair deficiency	2	31	16.5
3146	HMGB1	high-mobility group box 1	2	31	16.5
218	ALDH3A1	aldehyde dehydrogenase 3 family, memberA1	0	33	16.5
51473	DCDC2	doublecortin domain containing 2	0	33	16.5
57017	COQ9	coenzyme Q9 homolog (S. cerevisiae)	0	33	16.5
729165	LOC729165	hypothetical protein LOC729165	0	33	16.5
7465	WEE1	WEE1 homolog (S. pombe)	0	33	16.5
54	ACP5	acid phosphatase 5, tartrate resistant	0	33	16.5
9697	TRAM2	translocation associated membrane protein 2	33	0	16.5
3912	LAMB1	laminin, beta 1	33	0	16.5
84239	ATP13A4	ATPase type 13A4	33	0	16.5
23308	ICOSLG	inducible T-cell co-stimulator ligand	33	0	16.5
10768	AHCYL1	S-adenosylhomocysteine hydrolase-like 1	33	0	16.5
1196	CLK2	CDC-like kinase 2	33	0	16.5
344905	ATP13A5	ATPase type 13A5	33	0	16.5
28981	IFT81	intraflagellar transport 81 homolog (Chlamydomonas)	33	0	16.5
146456	TMED6	transmembrane emp24 protein transport domain containing	33	0	16.5
23310	NCAPD3	non-SMC condensin II complex, subunit D3	33	0	16.5
7752	ZNF200	zinc finger protein 200	33	0	16.5
22994	AZI1	5-azacytidine induced 1	33	0	16.5
90799	CCDC45	coiled-coil domain containing 45	33	0	16.5

11091	WDR5	WD repeat domain 5	33	0	16.5
60314	C12orf10	chromosome 12 open reading frame 10	33	0	16.5
57473	GM632	KIAA1196 protein	33	0	16.5
171024	SYNPO2	synaptopodin 2	33	0	16.5
729045	LOC729045	hypothetical protein LOC729045	30	2	16
2997	GYS1	glycogen synthase 1 (muscle)	30	2	16
5771	PTPN2	protein tyrosine phosphatase, non-receptor type 2	30	2	16
5522	PPP2R2C	protein phosphatase 2 (formerly 2A), regulatory subunit B (P	29	3	16
256364	EML3	echinoderm microtubule associated protein like 3	29	3	16
132204	SYNPR	synaptoporin	27	5	16
57509	MTUS1	mitochondrial tumor suppressor 1	27	5	16
5681	PSKH1	protein serine kinase H1	27	5	16
10988	METAP2	methionyl aminopeptidase 2	24	8	16
1797	DOM3Z	dom-3 homolog Z (C. elegans)	23	9	16
8941	CDK5R2	cyclin-dependent kinase 5, regulatory subunit 2 (p39)	19	13	16
3429	IFI27	interferon, alpha-inducible protein 27	17	15	16
111	ADCY5	adenylate cyclase 5	16	16	16
8879	SGPL1	sphingosine-1-phosphate lyase 1	15	17	16
9776	KIAA0652	KIAA0652	15	17	16
2137	EXTL3	exostoses (multiple)-like 3	13	19	16
23062	GGA2	golgi associated, gamma adaptin ear containing, ARF bindin	12	20	16
1316	KLF6	Kruppel-like factor 6	12	20	16
55220	KLHDC8A	kelch domain containing 8A	12	20	16
8295	TRRAP	transformation/transcription domain-associated protein	12	20	16
9181	ARHGEF2	rho/rac guanine nucleotide exchange factor (GEF) 2	12	20	16
79675	FASTKD1	FAST kinase domains 1	12	20	16
2879	GPX4	glutathione peroxidase 4 (phospholipid hydroperoxidase)	10	22	16
7813	EVI5	ecotropic viral integration site 5	10	22	16
51251	NT5C3	5'-nucleotidase, cytosolic III	8	24	16
55152	DALRD3	DALR anticodon binding domain containing 3	6	26	16
84750	FUT10	fucosyltransferase 10 (alpha (1,3) fucosyltransferase)	6	26	16
7185	TRAF1	TNF receptor-associated factor 1	6	26	16
80235	PIGZ	phosphatidylinositol glycan anchor biosynthesis, class Z	0	32	16
84942	WDR73	WD repeat domain 73	0	32	16
80114	BICC1	bicaudal C homolog 1 (Drosophila)	0	32	16
57198	ATP8B2	ATPase, Class I, type 8B, member 2	0	32	16
7443	VRK1	vaccinia related kinase 1	0	32	16
3416	IDE	insulin-degrading enzyme	0	32	16
149420	PDIK1L	PDLIM1 interacting kinase 1 like	0	32	16
347689	SOX2OT	SOX2 overlapping transcript (non-coding RNA)	0	32	16
160335	TMTC2	transmembrane and tetratricopeptide repeat containing 2	0	32	16
57731	SPTBN4	spectrin, beta, non-erythrocytic 4	0	32	16
388536	ZNF790	zinc finger protein 790	0	32	16
4775	NFATC3	nuclear factor of activated T-cells, cytoplasmic, calcineurin-d	0	32	16
122616	C14orf79	chromosome 14 open reading frame 79	0	32	16
148189	LOC148189	hypothetical protein LOC148189	0	32	16
94025	MUC16	mucin 16, cell surface associated	0	32	16
3297	HSF1	heat shock transcription factor 1	0	32	16
60343	FAM3A	family with sequence similarity 3, member A	0	32	16
51619	UBE2D4	ubiquitin-conjugating enzyme E2D 4 (putative)	32	0	16
440991	LOC440991	similar to 40S ribosomal protein S3	32	0	16

284040	CDRT4	CMT1A duplicated region transcript 4	32	0	16
151742	PPM1L	protein phosphatase 1 (formerly 2C)-like	32	0	16
56955	MEPE	matrix, extracellular phosphoglycoprotein with ASARM motif	32	0	16
124245	NHN1	conserved nuclear protein NHN1	32	0	16
27336	HTATSF1	HIV-1 Tat specific factor 1	32	0	16
81831	NETO2	neuropilin (NRP) and tolloid (TLL)-like 2	32	0	16
29850	TRPM5	transient receptor potential cation channel, subfamily M, mer	32	0	16
553103	LOC553103	hypothetical LOC553103	32	0	16
23380	SRGAP2	SLIT-ROBO Rho GTPase activating protein 2	32	0	16
6135	RPL11	ribosomal protein L11	29	2	15.5
440993	LOC440993	hypothetical gene supported by AK128346	28	3	15.5
81033	KCNH6	potassium voltage-gated channel, subfamily H (eag-related),	26	5	15.5
5411	PNN	pinin, desmosome associated protein	25	6	15.5
2034	EPAS1	endothelial PAS domain protein 1	24	7	15.5
5476	CTSA	cathepsin A	24	7	15.5
64121	RRAGC	Ras-related GTP binding C	24	7	15.5
79621	RNASEH2B	ribonuclease H2, subunit B	23	8	15.5
123355	LRRC28	leucine rich repeat containing 28	22	9	15.5
51322	WAC	WW domain containing adaptor with coiled-coil	20	11	15.5
8411	EEA1	early endosome antigen 1, 162kD	18	13	15.5
25782	RAB3GAP2	RAB3 GTPase activating protein subunit 2 (non-catalytic)	17	14	15.5
153443	SRFBP1	serum response factor binding protein 1	16	15	15.5
6683	SPAST	spastin	12	19	15.5
5638	PRRG1	proline rich Gla (G-carboxyglutamic acid) 1	12	19	15.5
284273	ZADH2	zinc binding alcohol dehydrogenase, domain containing 2	12	19	15.5
84895	FAM73B	family with sequence similarity 73, member B	10	21	15.5
91056	DKFZp761E1	DKFZp761E198 protein	10	21	15.5
9391	CIAO1	cytosolic iron-sulfur protein assembly 1 homolog (S. cerevisia	8	23	15.5
94239	H2AFV	H2A histone family, member V	6	25	15.5
116138	KLHDC3	kelch domain containing 3	6	25	15.5
10873	ME3	malic enzyme 3, NADP(+)-dependent, mitochondrial	2	29	15.5
9364	RAB28	RAB28, member RAS oncogene family	2	29	15.5
25934	NIPSNAP3A	nipsnap homolog 3A (C. elegans)	0	31	15.5
10291	SF3A1	splicing factor 3a, subunit 1, 120kDa	0	31	15.5
440456	LOC440456	similar to pleckstrin homology domain containing, family M (v	0	31	15.5
23332	CLASP1	cytoplasmic linker associated protein 1	0	31	15.5
220980	LOC220980	hypothetical protein LOC220980	0	31	15.5
10168	ZNF197	zinc finger protein 197	0	31	15.5
54552	GNL3L	guanine nucleotide binding protein-like 3 (nucleolar)-like	0	31	15.5
55298	RNF121	ring finger protein 121	0	31	15.5
81888	HYI	hydroxypyruvate isomerase homolog (E. coli)	0	31	15.5
6456	SH3GL2	SH3-domain GRB2-like 2	0	31	15.5
54443	ANLN	anillin, actin binding protein	0	31	15.5
55296	TBC1D19	TBC1 domain family, member 19	0	31	15.5
1824	DSC2	desmocollin 2	0	31	15.5
147968	CAPN12	calpain 12	0	31	15.5
4126	MANBA	mannosidase, beta A, lysosomal	0	31	15.5
390029	LOC390029	similar to cytoplasmic beta-actin	0	31	15.5
368	ABCC6	ATP-binding cassette, sub-family C (CFTR/MRP), member 6	0	31	15.5
11017	RY1	putative nucleic acid binding protein RY-1	0	31	15.5
728913	LOC728913	similar to Reticulocalbin-1 precursor	0	31	15.5

23544	SEZ6L	seizure related 6 homolog (mouse)-like	0	31	15.5
5288	PIK3C2G	phosphoinositide-3-kinase, class 2, gamma polypeptide	0	31	15.5
7412	VCAM1	vascular cell adhesion molecule 1	0	31	15.5
653688	NA	NA	0	31	15.5
25880	C16orf51	chromosome 16 open reading frame 51	0	31	15.5
321	APBA2	amyloid beta (A4) precursor protein-binding, family A, memb	0	31	15.5
130367	SGPP2	sphingosine-1-phosphate phosphotase 2	0	31	15.5
8541	PPFIA3	protein tyrosine phosphatase, receptor type, f polypeptide (P	0	31	15.5
9108	MTMR7	myotubularin related protein 7	0	31	15.5
29114	TAGLN3	transgelin 3	31	0	15.5
55125	CEP192	centrosomal protein 192kDa	31	0	15.5
22888	UBOX5	U-box domain containing 5	31	0	15.5
22870	SAPS1	SAPS domain family, member 1	31	0	15.5
3064	HD	huntingtin (Huntington disease)	31	0	15.5
51524	TMEM138	transmembrane protein 138	31	0	15.5
113251	LARP4	La ribonucleoprotein domain family, member 4	31	0	15.5
27089	UQCRQ	ubiquinol-cytochrome c reductase, complex III subunit VII, 9.	31	0	15.5
10656	KHDRBS3	KH domain containing, RNA binding, signal transduction ass	31	0	15.5
89978	ATPBD4	ATP binding domain 4	31	0	15.5
121642	ALKBH2	alkB, alkylation repair homolog 2 (E. coli)	31	0	15.5
79971	GPR177	G protein-coupled receptor 177	31	0	15.5
27067	STAU2	staufen, RNA binding protein, homolog 2 (Drosophila)	31	0	15.5
699	BUB1	BUB1 budding uninhibited by benzimidazoles 1 homolog (ye	31	0	15.5
81631	MAP1LC3B	microtubule-associated protein 1 light chain 3 beta	28	2	15
11282	MGAT4B	mannosyl (alpha-1,3-)-glycoprotein beta-1,4-N-acetylglucosa	27	3	15
145567	TTC7B	tetratricopeptide repeat domain 7B	27	3	15
5164	PDK2	pyruvate dehydrogenase kinase, isozyme 2	25	5	15
11118	BTN3A2	butyrophilin, subfamily 3, member A2	23	7	15
55285	RBM41	RNA binding motif protein 41	22	8	15
26505	CNNM3	cyclin M3	21	9	15
51422	PRKAG2	protein kinase, AMP-activated, gamma 2 non-catalytic subur	20	10	15
4175	MCM6	minichromosome maintenance deficient 6 homolog (S. cerev	20	10	15
65056	GPBP1	GC-rich promoter binding protein 1	19	11	15
58985	IL22RA1	interleukin 22 receptor, alpha 1	19	11	15
25820	ARIH1	ariadne homolog, ubiquitin-conjugating enzyme E2 binding p	15	15	15
23092	ARHGAP26	Rho GTPase activating protein 26	15	15	15
58473	PLEKHB1	pleckstrin homology domain containing, family B (evectins) n	14	16	15
150368	FAM109B	family with sequence similarity 109, member B	14	16	15
6891	TAP2	transporter 2, ATP-binding cassette, sub-family B (MDR/TAF	14	16	15
57679	ALS2	amyotrophic lateral sclerosis 2 (juvenile)	13	17	15
4216	MAP3K4	mitogen-activated protein kinase kinase kinase 4	13	17	15
7402	UTRN	utrophin	12	18	15
8720	MBTPS1	membrane-bound transcription factor peptidase, site 1	10	20	15
23429	RYBP	RING1 and YY1 binding protein	10	20	15
57680	CHD8	chromodomain helicase DNA binding protein 8	10	20	15
9688	NUP93	nucleoporin 93kDa	8	22	15
79811	SLTM	SAFB-like, transcription modulator	8	22	15
51692	CPSF3	cleavage and polyadenylation specific factor 3, 73kDa	6	24	15
60685	ZFAND3	zinc finger, AN1-type domain 3	6	24	15
51503	HSPC148	hypothetical protein HSPC148	6	24	15
4507	MTAP	methylthioadenosine phosphorylase	4	26	15

22907	DHX30	DEAH (Asp-Glu-Ala-His) box polypeptide 30	4	26	15
85442	KNDC1	kinase non-catalytic C-lobe domain (KIND) containing 1	4	26	15
57799	RAB40C	RAB40C, member RAS oncogene family	2	28	15
401237	FLJ22536	hypothetical locus LOC401237	2	28	15
29068	ZBTB44	zinc finger and BTB domain containing 44	2	28	15
51199	NIN	ninein (GSK3B interacting protein)	2	28	15
22982	DIP2C	DIP2 disco-interacting protein 2 homolog C (Drosophila)	0	30	15
375298	CERKL	ceramide kinase-like	30	0	15
28989	C9orf32	chromosome 9 open reading frame 32	30	0	15
10266	RAMP2	receptor (G protein-coupled) activity modifying protein 2	30	0	15
9592	IER2	immediate early response 2	30	0	15
55739	FLJ10769	hypothetical protein FLJ10769	30	0	15
57205	ATP10D	ATPase, Class V, type 10D	30	0	15
2052	EPHX1	epoxide hydrolase 1, microsomal (xenobiotic)	30	0	15
151613	TTC14	tetratricopeptide repeat domain 14	27	2	14.5
22993	KIAA0194	KIAA0194 protein	27	2	14.5
389332	LOC389332	hypothetical LOC389332	27	2	14.5
9092	SART1	squamous cell carcinoma antigen recognized by T cells	27	2	14.5
10046	CXorf6	chromosome X open reading frame 6	26	3	14.5
11012	KLK11	kallikrein-related peptidase 11	26	3	14.5
340348	TSPAN33	tetraspanin 33	22	7	14.5
716	C1S	complement component 1, s subcomponent	21	8	14.5
3653	IPW	imprinted in Prader-Willi syndrome	21	8	14.5
1540	CYLD	cylindromatosis (turban tumor syndrome)	19	10	14.5
59084	ENPP5	ectonucleotide pyrophosphatase/phosphodiesterase 5 (putat	18	11	14.5
283357	LOC283357	hypothetical protein LOC283357	17	12	14.5
55897	MESP1	mesoderm posterior 1 homolog (mouse)	16	13	14.5
5311	PKD2	polycystic kidney disease 2 (autosomal dominant)	16	13	14.5
23203	PMPCA	peptidase (mitochondrial processing) alpha	16	13	14.5
56970	ATXN7L3	ataxin 7-like 3	15	14	14.5
1357	CPA1	carboxypeptidase A1 (pancreatic)	14	15	14.5
22884	WDR37	WD repeat domain 37	12	17	14.5
10365	KLF2	Kruppel-like factor 2 (lung)	10	19	14.5
124989	C17orf57	chromosome 17 open reading frame 57	10	19	14.5
10390	CEPT1	choline/ethanolamine phosphotransferase 1	8	21	14.5
5980	REV3L	REV3-like, catalytic subunit of DNA polymerase zeta (yeast)	8	21	14.5
8908	GYG2	glycogenin 2	8	21	14.5
9911	TMCC2	transmembrane and coiled-coil domain family 2	8	21	14.5
330	BIRC3	baculoviral IAP repeat-containing 3	6	23	14.5
56952	PRTFDC1	phosphoribosyl transferase domain containing 1	4	25	14.5
9962	SLC23A2	solute carrier family 23 (nucleobase transporters), member 2	4	25	14.5
728640	LOC728640	hypothetical protein LOC728640	0	29	14.5
5929	RBBP5	retinoblastoma binding protein 5	0	29	14.5
51667	NUB1	negative regulator of ubiquitin-like proteins 1	0	29	14.5
9665	KIAA0430	KIAA0430	0	29	14.5
27069	GHITM	growth hormone inducible transmembrane protein	0	29	14.5
64499	TPSB2	tryptase beta 2	0	29	14.5
1021	CDK6	cyclin-dependent kinase 6	0	29	14.5
9229	DLGAP1	discs, large (Drosophila) homolog-associated protein 1	0	29	14.5
128414	C20orf58	chromosome 20 open reading frame 58	0	29	14.5
202018	FLJ90013	hypothetical protein FLJ90013	0	29	14.5

6122	RPL3	ribosomal protein L3	0	29	14.5
6700	SPRR2A	small proline-rich protein 2A	0	29	14.5
55660	PRPF40A	PRP40 pre-mRNA processing factor 40 homolog A (yeast)	0	29	14.5
80152	CENPT	centromere protein T	0	29	14.5
5293	PIK3CD	phosphoinositide-3-kinase, catalytic, delta polypeptide	0	29	14.5
51202	DDX47	DEAD (Asp-Glu-Ala-Asp) box polypeptide 47	0	29	14.5
79813	EHMT1	euchromatic histone-lysine N-methyltransferase 1	0	29	14.5
23326	USP22	ubiquitin specific peptidase 22	0	29	14.5
10329	TMEM5	transmembrane protein 5	0	29	14.5
6049	RNF6	ring finger protein (C3H2C3 type) 6	0	29	14.5
84572	GNPTG	N-acetylglucosamine-1-phosphate transferase, gamma subu	0	29	14.5
30061	SLC40A1	solute carrier family 40 (iron-regulated transporter), member	0	29	14.5
2048	EPHB2	EPH receptor B2	0	29	14.5
54704	PPM2C	protein phosphatase 2C, magnesium-dependent, catalytic su	0	29	14.5
6047	RNF4	ring finger protein 4	0	29	14.5
23281	KIAA0774	KIAA0774	29	0	14.5
30832	ZNF354C	zinc finger protein 354C	29	0	14.5
6990	DYNLT3	dynein, light chain, Tctex-type 3	29	0	14.5
79613	TMCO7	transmembrane and coiled-coil domains 7	29	0	14.5
8481	OFD1	oral-facial-digital syndrome 1	29	0	14.5
10171	RCL1	RNA terminal phosphate cyclase-like 1	29	0	14.5
10443	PFAAP5	phosphonoformate immuno-associated protein 5	29	0	14.5
4999	ORC2L	origin recognition complex, subunit 2-like (yeast)	29	0	14.5
283481	LOC283481	hypothetical protein LOC283481	29	0	14.5
84336	TMEM101	transmembrane protein 101	29	0	14.5
54982	CLN6	ceroid-lipofuscinosis, neuronal 6, late infantile, variant	29	0	14.5
26025	PCDHGA12	protocadherin gamma subfamily A, 12	29	0	14.5
90580	C19orf52	chromosome 19 open reading frame 52	29	0	14.5
57456	KIAA1143	KIAA1143	29	0	14.5
4034	LRCH4	leucine-rich repeats and calponin homology (CH) domain co	29	0	14.5
84236	RHBDD1	rhomboid domain containing 1	29	0	14.5
25909	AHCTF1	AT hook containing transcription factor 1	25	3	14
3964	LGALS8	lectin, galactoside-binding, soluble, 8 (galectin 8)	25	3	14
155435	RBM33	RNA binding motif protein 33	23	5	14
10564	ARFGEF2	ADP-ribosylation factor guanine nucleotide-exchange factor	21	7	14
6039	RNASE6	ribonuclease, RNase A family, k6	20	8	14
1001	CDH3	cadherin 3, type 1, P-cadherin (placental)	20	8	14
122961	HBLD1	HesB like domain containing 1	18	10	14
284454	LOC284454	hypothetical protein LOC284454	12	16	14
6447	SCG5	secretogranin V (7B2 protein)	12	16	14
1673	DEFB4	defensin, beta 4	10	18	14
23522	MYST4	MYST histone acetyltransferase (monocytic leukemia) 4	10	18	14
23023	TMCC1	transmembrane and coiled-coil domain family 1	8	20	14
533	ATP6V0B	ATPase, H+ transporting, lysosomal 21kDa, V0 subunit b	6	22	14
50862	RNF141	ring finger protein 141	4	24	14
114049	WBSCR22	Williams Beuren syndrome chromosome region 22	2	26	14
25998	IBTK	inhibitor of Bruton agammaglobulinemia tyrosine kinase	0	28	14
2597	GAPDH	glyceraldehyde-3-phosphate dehydrogenase	0	28	14
1687	DFNA5	deafness, autosomal dominant 5	0	28	14
56731	SLC2A4RG	SLC2A4 regulator	0	28	14
26586	CKAP2	cytoskeleton associated protein 2	0	28	14

83439	TCF7L1	transcription factor 7-like 1 (T-cell specific, HMG-box)	0	28	14
64924	SLC30A5	solute carrier family 30 (zinc transporter), member 5	0	28	14
26049	KIAA0888	KIAA0888 protein	0	28	14
57593	RP5-860F19	KIAA1442 protein	0	28	14
55766	H2AFJ	H2A histone family, member J	0	28	14
5047	PAEP	progestagen-associated endometrial protein (placental prote	0	28	14
94015	TTYH2	tweety homolog 2 (Drosophila)	0	28	14
23541	SEC14L2	SEC14-like 2 (S. cerevisiae)	0	28	14
64779	MTHFSD	methenyltetrahydrofolate synthetase domain containing	0	28	14
22954	TRIM32	tripartite motif-containing 32	0	28	14
7743	ZNF189	zinc finger protein 189	0	28	14
23471	TRAM1	translocation associated membrane protein 1	0	28	14
64094	SMOC2	SPARC related modular calcium binding 2	0	28	14
27325	TNRC8	trinucleotide repeat containing 8	0	28	14
201255	LRRC45	leucine rich repeat containing 45	0	28	14
51025	Magmas	mitochondria-associated protein involved in granulocyte-mac	28	0	14
148932	MOBKL2C	MOB1, Mps One Binder kinase activator-like 2C (yeast)	28	0	14
196463	LOC196463	hypothetical protein LOC196463	28	0	14
7153	TOP2A	topoisomerase (DNA) II alpha 170kDa	28	0	14
51	ACOX1	acyl-Coenzyme A oxidase 1, palmitoyl	28	0	14
78989	COLEC11	collectin sub-family member 11	28	0	14
337867	PHGDHL1	phosphoglycerate dehydrogenase like 1	28	0	14
81555	YIPF5	Yip1 domain family, member 5	28	0	14
24140	FTSJ1	FtsJ homolog 1 (E. coli)	28	0	14
10253	SPRY2	sprouty homolog 2 (Drosophila)	28	0	14
158056	MAMDC4	MAM domain containing 4	28	0	14
79874	RABEP2	rabaptin, RAB GTPase binding effector protein 2	28	0	14
54872	PIGG	phosphatidylinositol glycan anchor biosynthesis, class G	28	0	14
89848	FCHSD1	FCH and double SH3 domains 1	28	0	14
79953	C20orf39	chromosome 20 open reading frame 39	28	0	14
7923	HSD17B8	hydroxysteroid (17-beta) dehydrogenase 8	28	0	14
116113	FOXP4	forkhead box P4	28	0	14
90678	LRSAM1	leucine rich repeat and sterile alpha motif containing 1	28	0	14
3660	IRF2	interferon regulatory factor 2	28	0	14
440567	LOC440567	similar to Ubiquinol-cytochrome c reductase complex 11 kDa	28	0	14
9315	C5orf13	chromosome 5 open reading frame 13	28	0	14
9841	ZBTB24	zinc finger and BTB domain containing 24	24	3	13.5
783	CACNB2	calcium channel, voltage-dependent, beta 2 subunit	24	3	13.5
26160	IFT172	intraflagellar transport 172 homolog (Chlamydomonas)	23	4	13.5
9466	IL27RA	interleukin 27 receptor, alpha	22	5	13.5
51006	SLC35C2	solute carrier family 35, member C2	20	7	13.5
57498	KIDINS220	kinase D-interacting substance of 220 kDa	20	7	13.5
116844	LRG1	leucine-rich alpha-2-glycoprotein 1	19	8	13.5
11285	B4GALT7	xylosylprotein beta 1,4-galactosyltransferase, polypeptide 7 (	19	8	13.5
256536	TCERG1L	transcription elongation regulator 1-like	18	9	13.5
55735	DNAJC11	DnaJ (Hsp40) homolog, subfamily C, member 11	16	11	13.5
84680	PHACS	1-aminocyclopropane-1-carboxylate synthase	15	12	13.5
6560	SLC12A4	solute carrier family 12 (potassium/chloride transporters), me	15	12	13.5
30850	CDR2L	cerebellar degeneration-related protein 2-like	12	15	13.5
10587	TXNRD2	thioredoxin reductase 2	8	19	13.5
89122	TRIM4	tripartite motif-containing 4	6	21	13.5

9202	ZMYM4	zinc finger, MYM-type 4	4	23	13.5
653308	RP11-564C4	N-acylsphingosine amidohydrolase (non-lysosomal ceramida	4	23	13.5
55144	LRRC8D	leucine rich repeat containing 8 family, member D	2	25	13.5
11344	TWF2	twinfilin, actin-binding protein, homolog 2 (Drosophila)	2	25	13.5
10425	ARIH2	ariadne homolog 2 (Drosophila)	0	27	13.5
2186	BPTF	bromodomain PHD finger transcription factor	0	27	13.5
60490	PPCDC	phosphopantothenoylcysteine decarboxylase	0	27	13.5
51126	NAT5	N-acetyltransferase 5	0	27	13.5
51227	PIGP	phosphatidylinositol glycan anchor biosynthesis, class P	0	27	13.5
84967	LSM10	LSM10, U7 small nuclear RNA associated	0	27	13.5
56935	C11orf75	chromosome 11 open reading frame 75	0	27	13.5
29102	RNASEN	ribonuclease III, nuclear	0	27	13.5
79862	ZNF669	zinc finger protein 669	27	0	13.5
91947	ARRDC4	arrestin domain containing 4	27	0	13.5
80863	PRRT1	proline-rich transmembrane protein 1	27	0	13.5
3480	IGF1R	insulin-like growth factor 1 receptor	27	0	13.5
122481	AK7	adenylate kinase 7	27	0	13.5
56913	C1GALT1	core 1 synthase, glycoprotein-N-acetylgalactosamine 3-beta-	27	0	13.5
10855	HPSE	heparanase	27	0	13.5
5236	PGM1	phosphoglucomutase 1	27	0	13.5
91748	C14orf43	chromosome 14 open reading frame 43	27	0	13.5
11193	WBP4	WW domain binding protein 4 (formin binding protein 21)	27	0	13.5
55644	OSGEP	O-sialoglycoprotein endopeptidase	27	0	13.5
27109	ATP5S	ATP synthase, H+ transporting, mitochondrial F0 complex, s	27	0	13.5
56948	C14orf124	chromosome 14 open reading frame 124	27	0	13.5
27165	GLS2	glutaminase 2 (liver, mitochondrial)	27	0	13.5
54822	TRPM7	transient receptor potential cation channel, subfamily M, mer	27	0	13.5
1665	DHX15	DEAH (Asp-Glu-Ala-His) box polypeptide 15	24	2	13
51132	RNF12	ring finger protein 12	24	2	13
55015	PRPF39	PRP39 pre-mRNA processing factor 39 homolog (S. cerevisi	23	3	13
400566	LOC400566	hypothetical gene supported by AK128660	23	3	13
9019	MPZL1	myelin protein zero-like 1	23	3	13
50831	TAS2R3	taste receptor, type 2, member 3	21	5	13
93664	CADPS2	Ca2+-dependent activator protein for secretion 2	19	7	13
112616	CMTM7	CKLF-like MARVEL transmembrane domain containing 7	19	7	13
6038	RNASE4	ribonuclease, RNase A family, 4	18	8	13
25943	C20orf194	chromosome 20 open reading frame 194	18	8	13
83696	NIBP	NIK and IKK{beta} binding protein	18	8	13
10552	ARPC1A	actin related protein 2/3 complex, subunit 1A, 41kDa	18	8	13
11016	ATF7	activating transcription factor 7	17	9	13
3775	KCNK1	potassium channel, subfamily K, member 1	16	10	13
51106	TFB1M	transcription factor B1, mitochondrial	16	10	13
51230	PHF20	PHD finger protein 20	16	10	13
93974	ATPIF1	ATPase inhibitory factor 1	15	11	13
83699	SH3BGRL2	SH3 domain binding glutamic acid-rich protein like 2	13	13	13
6722	SRF	serum response factor (c-fos serum response element-bindir	8	18	13
403341	ZBTB34	zinc finger and BTB domain containing 34	6	20	13
54432	YIPF1	Yip1 domain family, member 1	6	20	13
215	ABCD1	ATP-binding cassette, sub-family D (ALD), member 1	4	22	13
84937	ZNRF1	zinc and ring finger 1	4	22	13
79710	MORC4	MORC family CW-type zinc finger 4	4	22	13

55526	DHTKD1	dehydrogenase E1 and transketolase domain containing 1	0	26	13
401778	LOC401778	similar to SERTA domain containing 4	0	26	13
64218	SEMA4A	sema domain, immunoglobulin domain (Ig), transmembrane	0	26	13
9915	ARNT2	aryl-hydrocarbon receptor nuclear translocator 2	0	26	13
28969	BZW2	basic leucine zipper and W2 domains 2	0	26	13
122769	PPIL5	peptidylprolyl isomerase (cyclophilin)-like 5	0	26	13
50807	DDEF1	development and differentiation enhancing factor 1	0	26	13
118672	C10orf89	chromosome 10 open reading frame 89	0	26	13
146330	FBXL16	F-box and leucine-rich repeat protein 16	0	26	13
5412	UBL3	ubiquitin-like 3	0	26	13
222183	FLJ37078	hypothetical protein FLJ37078	0	26	13
23452	ANGPTL2	angiopoietin-like 2	0	26	13
55187	VPS13D	vacuolar protein sorting 13 homolog D (S. cerevisiae)	0	26	13
221272	LOC221272	hypothetical protein LOC221272	0	26	13
7704	ZBTB16	zinc finger and BTB domain containing 16	0	26	13
347744	C6orf52	chromosome 6 open reading frame 52	0	26	13
6161	RPL32	ribosomal protein L32	0	26	13
57708	MIER1	mesoderm induction early response 1 homolog (Xenopus lae	0	26	13
119710	C11orf74	chromosome 11 open reading frame 74	0	26	13
56603	CYP26B1	cytochrome P450, family 26, subfamily B, polypeptide 1	0	26	13
6929	TCF3	transcription factor 3 (E2A immunoglobulin enhancer binding	0	26	13
116151	C20orf108	chromosome 20 open reading frame 108	26	0	13
64061	TSPYL2	TSPY-like 2	26	0	13
2067	ERCC1	excision repair cross-complementing rodent repair deficiency	26	0	13
400948	NA	NA	26	0	13
55006	FLJ20628	hypothetical protein FLJ20628	26	0	13
54883	CCDC49	coiled-coil domain containing 49	26	0	13
9748	SLK	STE20-like kinase (yeast)	26	0	13
401560	C9orf151	chromosome 9 open reading frame 151	26	0	13
10204	NUTF2	nuclear transport factor 2	26	0	13
23063	WAPAL	wings apart-like homolog (Drosophila)	26	0	13
554202	LOC554202	hypothetical LOC554202	26	0	13
440731	LOC440731	hypothetical LOC440731	26	0	13
338811	FAM19A2	family with sequence similarity 19 (chemokine (C-C motif)-lik	26	0	13
57539	WDR35	WD repeat domain 35	26	0	13
55064	C9orf68	chromosome 9 open reading frame 68	26	0	13
1496	CTNNA2	catenin (cadherin-associated protein), alpha 2	26	0	13
26098	C10orf137	chromosome 10 open reading frame 137	23	2	12.5
5208	PFKFB2	6-phosphofructo-2-kinase/fructose-2,6-biphosphatase 2	23	2	12.5
57818	G6PC2	glucose-6-phosphatase, catalytic, 2	23	2	12.5
8780	RIOK3	RIO kinase 3 (yeast)	23	2	12.5
9734	HDAC9	histone deacetylase 9	23	2	12.5
8417	STX7	syntaxin 7	22	3	12.5
5721	PSME2	proteasome (prosome, macropain) activator subunit 2 (PA28	22	3	12.5
3855	KRT7	keratin 7	22	3	12.5
128486	C20orf142	chromosome 20 open reading frame 142	22	3	12.5
149773	LOC149773	hypothetical protein LOC149773	22	3	12.5
26012	NELF	nasal embryonic LHRH factor	22	3	12.5
8621	CDC2L5	cell division cycle 2-like 5 (cholinesterase-related cell divisior	20	5	12.5
286334	LOC286334	hypothetical protein LOC286334	20	5	12.5
54732	TMED9	transmembrane emp24 protein transport domain containing !	18	7	12.5

7532	YWHAG	tyrosine 3-monooxygenase/tryptophan 5-monooxygenase ac	18	7	12.5
23411	SIRT1	sirtuin (silent mating type information regulation 2 homolog) :	14	11	12.5
25932	CLIC4	chloride intracellular channel 4	14	11	12.5
81846	SBF2	SET binding factor 2	14	11	12.5
4648	MYO7B	myosin VIIB	14	11	12.5
255520	ELMOD2	ELMO/CED-12 domain containing 2	12	13	12.5
4072	TACSTD1	tumor-associated calcium signal transducer 1	12	13	12.5
54815	GATAD2A	GATA zinc finger domain containing 2A	10	15	12.5
7334	UBE2N	ubiquitin-conjugating enzyme E2N (UBC13 homolog, yeast)	10	15	12.5
130576	LOC130576	hypothetical protein LOC130576	10	15	12.5
4595	MUTYH	mutY homolog (E. coli)	10	15	12.5
10127	ZNF263	zinc finger protein 263	8	17	12.5
79365	BHLHB3	basic helix-loop-helix domain containing, class B, 3	8	17	12.5
10533	ATG7	ATG7 autophagy related 7 homolog (S. cerevisiae)	8	17	12.5
747	C11orf11	chromosome 11 open reading frame 11	4	21	12.5
124808	CCDC43	coiled-coil domain containing 43	2	23	12.5
150166	LOC150166	hypothetical protein LOC150166	0	25	12.5
3030	HADHA	hydroxyacyl-Coenzyme A dehydrogenase/3-ketoacyl-Coenzy	0	25	12.5
4285	MIPEP	mitochondrial intermediate peptidase	0	25	12.5
57534	MIB1	mindbomb homolog 1 (Drosophila)	0	25	12.5
284600	LOC284600	hypothetical protein LOC284600	0	25	12.5
29923	HIG2	hypoxia-inducible protein 2	0	25	12.5
7170	TPM3	tropomyosin 3	0	25	12.5
203427	SLC25A43	solute carrier family 25, member 43	0	25	12.5
57556	SEMA6A	sema domain, transmembrane domain (TM), and cytoplasmi	0	25	12.5
10196	PRMT3	protein arginine methyltransferase 3	0	25	12.5
149448	LOC149448	hypothetical protein LOC149448	0	25	12.5
134957	STXBP5	syntaxin binding protein 5 (tomosyn)	0	25	12.5
5916	RARG	retinoic acid receptor, gamma	0	25	12.5
23405	DICER1	Dicer1, Dcr-1 homolog (Drosophila)	0	25	12.5
79739	TTLL7	tubulin tyrosine ligase-like family, member 7	0	25	12.5
286676	ILDR1	immunoglobulin-like domain containing receptor 1	0	25	12.5
54842	FLJ20160	FLJ20160 protein	0	25	12.5
64853	C1orf80	chromosome 1 open reading frame 80	0	25	12.5
84678	FBXL10	F-box and leucine-rich repeat protein 10	0	25	12.5
23198	PSME4	proteasome (prosome, macropain) activator subunit 4	0	25	12.5
7024	TFCP2	transcription factor CP2	0	25	12.5
201501	ZBTB7C	zinc finger and BTB domain containing 7C	0	25	12.5
55553	SOX6	SRY (sex determining region Y)-box 6	0	25	12.5
81037	CLPTM1L	CLPTM1-like	0	25	12.5
55770	EXOC2	exocyst complex component 2	25	0	12.5
7376	NR1H2	nuclear receptor subfamily 1, group H, member 2	25	0	12.5
55561	CDC42BPG	CDC42 binding protein kinase gamma (DMPK-like)	25	0	12.5
2140	EYA3	eyes absent homolog 3 (Drosophila)	25	0	12.5
84975	MFSD5	major facilitator superfamily domain containing 5	25	0	12.5
3109	HLA-DMB	major histocompatibility complex, class II, DM beta	25	0	12.5
2876	GPX1	glutathione peroxidase 1	25	0	12.5
152503	SH3D19	SH3 domain protein D19	25	0	12.5
387066	SNHG5	small nucleolar RNA host gene (non-protein coding) 5	25	0	12.5
11128	POLR3A	polymerase (RNA) III (DNA directed) polypeptide A, 155kDa	25	0	12.5
1282	COL4A1	collagen, type IV, alpha 1	25	0	12.5

221037	JMJD1C	jumonji domain containing 1C	25	0	12.5
195827	C9orf21	chromosome 9 open reading frame 21	25	0	12.5
85301	COL27A1	collagen, type XXVII, alpha 1	25	0	12.5
7415	VCP	valosin-containing protein	25	0	12.5
2193	FARSLA	phenylalanine-tRNA synthetase-like, alpha subunit	25	0	12.5
8568	D21S2056E	DNA segment on chromosome 21 (unique) 2056 expressed	25	0	12.5
6414	SEPP1	selenoprotein P, plasma, 1	22	2	12
9647	PPM1F	protein phosphatase 1F (PP2C domain containing)	21	3	12
200879	LIPH	lipase, member H	18	6	12
3836	KPNA1	karyopherin alpha 1 (importin alpha 5)	18	6	12
10238	WDR68	WD repeat domain 68	16	8	12
54825	PCLKC	protocadherin LKC	16	8	12
2260	FGFR1	fibroblast growth factor receptor 1 (fms-related tyrosine kinas	16	8	12
3183	HNRPC	heterogeneous nuclear ribonucleoprotein C (C1/C2)	16	8	12
9403	SEP15	15 kDa selenoprotein	15	9	12
23531	MMD	monocyte to macrophage differentiation-associated	13	11	12
10242	KCNMB2	potassium large conductance calcium-activated channel, sub	12	12	12
9135	RABEP1	rabaptin, RAB GTPase binding effector protein 1	12	12	12
57512	GPR158	G protein-coupled receptor 158	8	16	12
285966	FLJ40722	hypothetical protein FLJ40722	6	18	12
5218	PFTK1	PFTAIRE protein kinase 1	6	18	12
7248	TSC1	tuberous sclerosis 1	6	18	12
4534	MTM1	myotubularin 1	4	20	12
10749	KIF1C	kinesin family member 1C	4	20	12
11143	MYST2	MYST histone acetyltransferase 2	4	20	12
54896	PQLC2	PQ loop repeat containing 2	4	20	12
54477	PLEKHA5	pleckstrin homology domain containing, family A member 5	2	22	12
3422	IDI1	isopentenyl-diphosphate delta isomerase 1	2	22	12
440461	LOC440461	similar to Rho GTPase activating protein 15	0	24	12
7802	DNALI1	dynein, axonemal, light intermediate chain 1	0	24	12
28232	SLCO3A1	solute carrier organic anion transporter family, member 3A1	0	24	12
57470	LRRC47	leucine rich repeat containing 47	0	24	12
55103	RALGPS2	Ral GEF with PH domain and SH3 binding motif 2	0	24	12
286208	LOC286208	hypothetical protein LOC286208	0	24	12
222484	LNX2	ligand of numb-protein X 2	0	24	12
6134	RPL10	ribosomal protein L10	0	24	12
6331	SCN5A	sodium channel, voltage-gated, type V, alpha (long QT syndr	0	24	12
11020	RABL4	RAB, member of RAS oncogene family-like 4	0	24	12
10094	ARPC3	actin related protein 2/3 complex, subunit 3, 21kDa	0	24	12
4051	CYP4F3	cytochrome P450, family 4, subfamily F, polypeptide 3	0	24	12
84280	BTBD10	BTB (POZ) domain containing 10	24	0	12
89887	ZNF628	zinc finger protein 628	24	0	12
84330	ZNF414	zinc finger protein 414	24	0	12
8425	LTBP4	latent transforming growth factor beta binding protein 4	24	0	12
55656	INTS8	integrator complex subunit 8	24	0	12
55530	SVOP	SV2 related protein homolog (rat)	24	0	12
9312	KCNB2	potassium voltage-gated channel, Shab-related subfamily, m	24	0	12
2958	GTF2A2	general transcription factor IIA, 2, 12kDa	24	0	12
221895	JAZF1	JAZF zinc finger 1	24	0	12
79608	RIC3	resistance to inhibitors of cholinesterase 3 homolog (C. elega	24	0	12
23390	ZDHHC17	zinc finger, DHHC-type containing 17	24	0	12

5318	PKP2	plakophilin 2	24	0	12
51098	IFT52	intraflagellar transport 52 homolog (Chlamydomonas)	24	0	12
57190	SEPN1	selenoprotein N, 1	24	0	12
57168	ASPHD2	aspartate beta-hydroxylase domain containing 2	24	0	12
57623	ZFAT1	ZFAT zinc finger 1	24	0	12
6624	FSCN1	fascin homolog 1, actin-bundling protein (Strongylocentrotus	24	0	12
4359	MPZ	myelin protein zero (Charcot-Marie-Tooth neuropathy 1B)	24	0	12
728228	LOC728228	hypothetical protein LOC728228	24	0	12
26057	ANKRD17	ankyrin repeat domain 17	24	0	12
148430	LOC148430	hypothetical LOC148430	24	0	12
23243	ANKRD28	ankyrin repeat domain 28	24	0	12
112936	VPS26B	vacuolar protein sorting 26 homolog B (S. cerevisiae)	24	0	12
58491	ZNF71	zinc finger protein 71	24	0	12
83935	TMEM133	transmembrane protein 133	24	0	12
284338	MGC70924	hypothetical LOC284338	21	2	11.5
1946	EFNA5	ephrin-A5	20	3	11.5
253143	C22orf30	chromosome 22 open reading frame 30	20	3	11.5
4646	MYO6	myosin VI	19	4	11.5
284340	UNQ473	DMC	18	5	11.5
145781	Gcom1	GRINL1A combined protein	17	6	11.5
85369	FAM40A	family with sequence similarity 40, member A	16	7	11.5
55726	C12orf11	chromosome 12 open reading frame 11	16	7	11.5
3703	STT3A	STT3, subunit of the oligosaccharyltransferase complex, hor	16	7	11.5
23567	ZNF346	zinc finger protein 346	12	11	11.5
178	AGL	amylo-1, 6-glucosidase, 4-alpha-glucanotransferase (glycog	12	11	11.5
9615	GDA	guanine deaminase	12	11	11.5
55850	MDS032	uncharacterized hematopoietic stem/progenitor cells protein	12	11	11.5
91754	NEK9	NIMA (never in mitosis gene a)- related kinase 9	10	13	11.5
8792	TNFRSF11A	tumor necrosis factor receptor superfamily, member 11a, NF	10	13	11.5
2526	FUT4	fucosyltransferase 4 (alpha (1,3) fucosyltransferase, myeloid	8	15	11.5
54742	LY6K	lymphocyte antigen 6 complex, locus K	8	15	11.5
2595	GANC	glucosidase, alpha; neutral C	8	15	11.5
23177	CEP68	centrosomal protein 68kDa	8	15	11.5
5532	PPP3CB	protein phosphatase 3 (formerly 2B), catalytic subunit, beta i	6	17	11.5
5362	PLXNA2	plexin A2	2	21	11.5
150275	CCDC117	coiled-coil domain containing 117	0	23	11.5
54414	SIAE	sialic acid acetylesterase	0	23	11.5
440957	SNHG8	small nucleolar RNA host gene (non-protein coding) 8	0	23	11.5
9200	PTPLA	protein tyrosine phosphatase-like (proline instead of catalytic	0	23	11.5
338596	ST8SIA6	ST8 alpha-N-acetyl-neuraminide alpha-2,8-sialyltransferase	0	23	11.5
56901	NDUFA4L2	NADH dehydrogenase (ubiquinone) 1 alpha subcomplex, 4-l	0	23	11.5
83661	MS4A8B	membrane-spanning 4-domains, subfamily A, member 8B	0	23	11.5
27249	C2orf25	chromosome 2 open reading frame 25	0	23	11.5
641339	ZNF674	zinc finger protein 674	0	23	11.5
58487	CREBZF	CREB/ATF bZIP transcription factor	0	23	11.5
84912	SLC35B4	solute carrier family 35, member B4	0	23	11.5
9730	VPRBP	Vpr (HIV-1) binding protein	0	23	11.5
283848	FLJ37464	hypothetical protein FLJ37464	0	23	11.5
8379	MAD1L1	MAD1 mitotic arrest deficient-like 1 (yeast)	0	23	11.5
374907	B3GNT8	UDP-GlcNAc:betaGal beta-1,3-N-acetylglucosaminyltransfer	0	23	11.5
15	AANAT	arylalkylamine N-acetyltransferase	0	23	11.5

55095	SAMD4B	sterile alpha motif domain containing 4B	0	23	11.5
8916	HERC3	hect domain and RLD 3	0	23	11.5
9919	KIAA0310	KIAA0310	0	23	11.5
5187	PER1	period homolog 1 (Drosophila)	0	23	11.5
54681	PH-4	hypoxia-inducible factor prolyl 4-hydroxylase	0	23	11.5
770	CA11	carbonic anhydrase XI	0	23	11.5
55364	IMPACT	Impact homolog (mouse)	0	23	11.5
5167	ENPP1	ectonucleotide pyrophosphatase/phosphodiesterase 1	0	23	11.5
554226	LOC554226	hypothetical protein LOC554226	0	23	11.5
1877	E4F1	E4F transcription factor 1	0	23	11.5
23175	LPIN1	lipin 1	0	23	11.5
57185	NPAL3	NIPA-like domain containing 3	0	23	11.5
23052	ENDOD1	endonuclease domain containing 1	0	23	11.5
120103	SLC36A4	solute carrier family 36 (proton/amino acid symporter), memb	0	23	11.5
80830	APOL6	apolipoprotein L, 6	0	23	11.5
54933	RHBDL2	rhomboid, veinlet-like 2 (Drosophila)	0	23	11.5
79785	FLJ22655	hypothetical protein FLJ22655	0	23	11.5
440073	IQSEC3	IQ motif and Sec7 domain 3	0	23	11.5
6508	SLC4A3	solute carrier family 4, anion exchanger, member 3	0	23	11.5
94234	FOXQ1	forkhead box Q1	0	23	11.5
9112	MTA1	metastasis associated 1	0	23	11.5
3587	IL10RA	interleukin 10 receptor, alpha	0	23	11.5
91574	FLJ38663	hypothetical protein FLJ38663	0	23	11.5
54584	GNB1L	guanine nucleotide binding protein (G protein), beta polypept	23	0	11.5
60386	SLC25A19	solute carrier family 25 (mitochondrial deoxynucleotide carrie	23	0	11.5
6498	SKIL	SKI-like oncogene	23	0	11.5
23286	WWC1	WW and C2 domain containing 1	23	0	11.5
7059	THBS3	thrombospondin 3	23	0	11.5
115572	FAM46B	family with sequence similarity 46, member B	23	0	11.5
201164	LOC201164	similar to CG12314 gene product	23	0	11.5
55244	FLJ10847	hypothetical protein FLJ10847	23	0	11.5
8996	NOL3	nucleolar protein 3 (apoptosis repressor with CARD domain)	23	0	11.5
92558	CCDC64	coiled-coil domain containing 64	23	0	11.5
5393	EXOSC9	exosome component 9	23	0	11.5
29062	HSPC049	HSPC049 protein	23	0	11.5
6794	STK11	serine/threonine kinase 11	23	0	11.5
9696	CROCC	ciliary rootlet coiled-coil, rootletin	23	0	11.5
79411	GLB1L	galactosidase, beta 1-like	23	0	11.5
57136	C20orf3	chromosome 20 open reading frame 3	23	0	11.5
79184	BRCC3	BRCA1/BRCA2-containing complex, subunit 3	23	0	11.5
11179	ZNF277P	zinc finger protein 277 pseudogene	23	0	11.5
5291	PIK3CB	phosphoinositide-3-kinase, catalytic, beta polypeptide	23	0	11.5
10607	TBL3	transducin (beta)-like 3	23	0	11.5
2068	ERCC2	excision repair cross-complementing rodent repair deficiency	23	0	11.5
8833	GMPS	guanine monphosphate synthetase	23	0	11.5
27244	SESN1	sestrin 1	23	0	11.5
231	AKR1B1	aldo-keto reductase family 1, member B1 (aldose reductase)	23	0	11.5
9231	DLG5	discs, large homolog 5 (Drosophila)	19	3	11
84960	KIAA1984	KIAA1984	19	3	11
9712	USP6NL	USP6 N-terminal like	17	5	11
642797	FLJ45278	hypothetical protein LOC642797	17	5	11

649	BMP1	bone morphogenetic protein 1	14	8	11
23184	MESDC2	mesoderm development candidate 2	13	9	11
137835	TMEM71	transmembrane protein 71	13	9	11
55716	LMBR1L	limb region 1 homolog (mouse)-like	12	10	11
55692	LUC7L	LUC7-like (S. cerevisiae)	8	14	11
5927	JARID1A	jumonji, AT rich interactive domain 1A	8	14	11
91300	C19orf22	chromosome 19 open reading frame 22	6	16	11
8395	PIP5K1B	phosphatidylinositol-4-phosphate 5-kinase, type I, beta	6	16	11
2523	FUT1	fucosyltransferase 1 (galactoside 2-alpha-L-fucosyltransfera	6	16	11
135112	NCOA7	nuclear receptor coactivator 7	6	16	11
83737	ITCH	itchy homolog E3 ubiquitin protein ligase (mouse)	6	16	11
80820	KIAA1706	KIAA1706 protein	2	20	11
154467	C6orf129	chromosome 6 open reading frame 129	0	22	11
64216	TFB2M	transcription factor B2, mitochondrial	0	22	11
3340	NDST1	N-deacetylase/N-sulfotransferase (heparan glucosaminyl) 1	0	22	11
726	CAPN5	calpain 5	0	22	11
10285	SMNDC1	survival motor neuron domain containing 1	0	22	11
51136	LOC51136	PTD016 protein	0	22	11
10438	C1D	nuclear DNA-binding protein	0	22	11
25902	MTHFD1L	methylenetetrahydrofolate dehydrogenase (NADP+ depende	0	22	11
31	ACACA	acetyl-Coenzyme A carboxylase alpha	0	22	11
26054	SENP6	SUMO1/sentrin specific peptidase 6	0	22	11
10146	G3BP1	GTPase activating protein (SH3 domain) binding protein 1	0	22	11
57146	TMEM159	transmembrane protein 159	0	22	11
54463	FLJ20152	hypothetical protein FLJ20152	0	22	11
8408	ULK1	unc-51-like kinase 1 (C. elegans)	0	22	11
10528	NOL5A	nucleolar protein 5A (56kDa with KKE/D repeat)	0	22	11
23302	KIAA0523	KIAA0523 protein	0	22	11
134147	CMBL	carboxymethylenebutenolidase homolog (Pseudomonas)	0	22	11
92126	C18orf4	chromosome 18 open reading frame 4	0	22	11
83939	EIF2A	eukaryotic translation initiation factor 2A, 65kDa	0	22	11
23299	BICD2	bicaudal D homolog 2 (Drosophila)	0	22	11
9444	QKI	quaking homolog, KH domain RNA binding (mouse)	0	22	11
164045	HFM1	HFM1, ATP-dependent DNA helicase homolog (S. cerevisia	0	22	11
10914	PAPOLA	poly(A) polymerase alpha	0	22	11
5827	PXMP2	peroxisomal membrane protein 2, 22kDa	0	22	11
8642	DCHS1	dachsous 1 (Drosophila)	22	0	11
347527	ARSH	arylsulfatase family, member H	22	0	11
26273	FBXO3	F-box protein 3	22	0	11
2275	FHL3	four and a half LIM domains 3	22	0	11
5274	SERPINI1	serpin peptidase inhibitor, clade I (neuroserpin), member 1	22	0	11
253769	WDR27	WD repeat domain 27	22	0	11
1013	CDH15	cadherin 15, M-cadherin (myotubule)	22	0	11
7011	TEP1	telomerase-associated protein 1	22	0	11
147808	ZNF784	zinc finger protein 784	22	0	11
27349	MCAT	malonyl CoA:ACP acyltransferase (mitochondrial)	22	0	11
57628	DPP10	dipeptidyl-peptidase 10	22	0	11
3908	LAMA2	laminin, alpha 2 (merosin, congenital muscular dystrophy)	22	0	11
80222	TARSL1	threonyl-tRNA synthetase-like 1	22	0	11
91370	MGC20647	hypothetical protein MGC20647	22	0	11
10850	CCL27	chemokine (C-C motif) ligand 27	22	0	11

60312	AFAP	actin filament associated protein	22	0	11
4224	MEP1A	meprin A, alpha (PABA peptide hydrolase)	22	0	11
1728	NQO1	NAD(P)H dehydrogenase, quinone 1	22	0	11
8574	AKR7A2	aldo-keto reductase family 7, member A2 (aflatoxin aldehyde	22	0	11
90198	C19orf49	chromosome 19 open reading frame 49	22	0	11
1536	CYBB	cytochrome b-245, beta polypeptide (chronic granulomatous	22	0	11
51021	MRPS16	mitochondrial ribosomal protein S16	22	0	11
64983	MRPL32	mitochondrial ribosomal protein L32	22	0	11
7130	TNFAIP6	tumor necrosis factor, alpha-induced protein 6	22	0	11
80726	KIAA1683	KIAA1683	22	0	11
1594	CYP27B1	cytochrome P450, family 27, subfamily B, polypeptide 1	22	0	11
84166	NLRC5	NLR family, CARD domain containing 5	19	2	10.5
1272	CNTN1	contactin 1	19	2	10.5
57730	KIAA1641	KIAA1641	19	2	10.5
25980	C20orf4	chromosome 20 open reading frame 4	18	3	10.5
54462	KIAA1128	KIAA1128	18	3	10.5
56474	CTPS2	CTP synthase II	16	5	10.5
91010	FMNL3	formin-like 3	16	5	10.5
114294	LACTB	lactamase, beta	16	5	10.5
2646	GCKR	glucokinase (hexokinase 4) regulator	16	5	10.5
6391	SDHC	succinate dehydrogenase complex, subunit C, integral meml	14	7	10.5
65980	BRD9	bromodomain containing 9	13	8	10.5
643253	CCT6AP1	chaperonin containing TCP1, subunit 6A (zeta 1) pseudogen	13	8	10.5
9874	TLK1	tousled-like kinase 1	13	8	10.5
221443	C6orf130	chromosome 6 open reading frame 130	12	9	10.5
3551	IKBKB	inhibitor of kappa light polypeptide gene enhancer in B-cells,	12	9	10.5
23624	CBLC	Cas-Br-M (murine) ecotropic retroviral transforming sequenc	12	9	10.5
26007	DAK	dihydroxyacetone kinase 2 homolog (S. cerevisiae)	12	9	10.5
55036	CCDC40	coiled-coil domain containing 40	10	11	10.5
389389	FLJ90086	similar to AI661453 protein	10	11	10.5
238	ALK	anaplastic lymphoma kinase (Ki-1)	8	13	10.5
7163	TPD52	tumor protein D52	6	15	10.5
257364	SH3PX3	SH3 and PX domain containing 3	6	15	10.5
399959	LOC399959	hypothetical gene supported by BX647608	6	15	10.5
10230	NBR2	neighbor of BRCA1 gene 2	6	15	10.5
284702	LOC284702	hypothetical protein LOC284702	4	17	10.5
9454	HOMER3	homer homolog 3 (Drosophila)	4	17	10.5
59277	NTN4	netrin 4	2	19	10.5
653325	LOC653325	hypothetical LOC653325	0	21	10.5
27042	C1orf107	chromosome 1 open reading frame 107	0	21	10.5
11045	UPK1A	uroplakin 1A	0	21	10.5
60561	RINT1	RAD50 interactor 1	0	21	10.5
256355	MGC27348	ribosomal protein S2 pseudogene	0	21	10.5
8161	COIL	coilin	0	21	10.5
144874	LOC144874	hypothetical protein LOC144874	0	21	10.5
7275	TUB	tubby homolog (mouse)	0	21	10.5
7273	TTN	titin	0	21	10.5
55849	ALG13	asparagine-linked glycosylation 13 homolog (S. cerevisiae)	0	21	10.5
54902	TTC19	tetratricopeptide repeat domain 19	0	21	10.5
645638	LOC645638	similar to WDNM1-like protein	0	21	10.5
83698	CALN1	calneuron 1	0	21	10.5

7074	TIAM1	T-cell lymphoma invasion and metastasis 1	0	21	10.5
54475	NLE1	notchless homolog 1 (Drosophila)	0	21	10.5
55308	DDX19A	DEAD (Asp-Glu-Ala-As) box polypeptide 19A	0	21	10.5
5922	RASA2	RAS p21 protein activator 2	21	0	10.5
57497	LRFN2	leucine rich repeat and fibronectin type III domain containing	21	0	10.5
11001	SLC27A2	solute carrier family 27 (fatty acid transporter), member 2	21	0	10.5
285527	FRYL	FRY-like	21	0	10.5
57325	CSRP2BP	CSRP2 binding protein	21	0	10.5
23786	BCL2L13	BCL2-like 13 (apoptosis facilitator)	21	0	10.5
51255	LOC51255	hypothetical protein LOC51255	21	0	10.5
4884	NPTX1	neuronal pentraxin I	21	0	10.5
11276	AP1GBP1	AP1 gamma subunit binding protein 1	21	0	10.5
80196	RNF34	ring finger protein 34	21	0	10.5
29933	GPR132	G protein-coupled receptor 132	21	0	10.5
2674	GFRA1	GDNF family receptor alpha 1	21	0	10.5
121227	LRIG3	leucine-rich repeats and immunoglobulin-like domains 3	21	0	10.5
9829	DNAJC6	DnaJ (Hsp40) homolog, subfamily C, member 6	21	0	10.5
9413	C9orf61	chromosome 9 open reading frame 61	21	0	10.5
3547	IGSF1	immunoglobulin superfamily, member 1	21	0	10.5
5923	RASGRF1	Ras protein-specific guanine nucleotide-releasing factor 1	21	0	10.5
286053	NSMCE2	non-SMC element 2, MMS21 homolog (S. cerevisiae)	21	0	10.5
400892	FLJ42953	FLJ42953 protein	21	0	10.5
54495	TXNDC10	thioredoxin domain containing 10	21	0	10.5
284323	ZNF780A	zinc finger protein 780A	21	0	10.5
23527	CENTB2	centaurin, beta 2	21	0	10.5
64283	RGNEF	Rho-guanine nucleotide exchange factor	21	0	10.5
25851	DKFZP434B	DKFZP434B0335 protein	21	0	10.5
56171	DNAH7	dynein, axonemal, heavy chain 7	21	0	10.5
25874	BRP44	brain protein 44	21	0	10.5
3614	IMPDH1	IMP (inosine monophosphate) dehydrogenase 1	21	0	10.5
29928	TIMM22	translocase of inner mitochondrial membrane 22 homolog (y	21	0	10.5
2159	F10	coagulation factor X	21	0	10.5
10277	UBE4B	ubiquitination factor E4B (UFD2 homolog, yeast)	21	0	10.5
10915	TCERG1	transcription elongation regulator 1	18	2	10
54998	AURKAIP1	aurora kinase A interacting protein 1	18	2	10
6233	RPS27A	ribosomal protein S27a	17	3	10
54478	FAM64A	family with sequence similarity 64, member A	17	3	10
64428	NARFL	nuclear prelamin A recognition factor-like	17	3	10
54845	RBM35A	RNA binding motif protein 35A	15	5	10
10989	IMMT	inner membrane protein, mitochondrial (mitofilin)	15	5	10
153339	TMEM167	transmembrane protein 167	15	5	10
94121	SYTL4	synaptotagmin-like 4 (granuphilin-a)	15	5	10
23586	DDX58	DEAD (Asp-Glu-Ala-Asp) box polypeptide 58	15	5	10
55844	PPP2R2D	protein phosphatase 2, regulatory subunit B, delta isoform	12	8	10
23155	CLCC1	chloride channel CLIC-like 1	12	8	10
55049	FLJ20850	hypothetical protein FLJ20850	12	8	10
55686	MREG	melanoregulin	12	8	10
144717	FAM109A	family with sequence similarity 109, member A	12	8	10
7001	PRDX2	peroxiredoxin 2	10	10	10
79906	MORN1	MORN repeat containing 1	10	10	10
9923	ZBTB40	zinc finger and BTB domain containing 40	10	10	10

81566	C12orf22	chromosome 12 open reading frame 22	10	10	10
2066	ERBB4	v-erb-a erythroblastic leukemia viral oncogene homolog 4 (a	10	10	10
27037	HTF9C	HpaII tiny fragments locus 9C	8	12	10
50865	HEBP1	heme binding protein 1	8	12	10
9782	MATR3	matrin 3	6	14	10
6217	RPS16	ribosomal protein S16	6	14	10
84302	C9orf125	chromosome 9 open reading frame 125	6	14	10
9459	ARHGEF6	Rac/Cdc42 guanine nucleotide exchange factor (GEF) 6	4	16	10
622	BDH1	3-hydroxybutyrate dehydrogenase, type 1	4	16	10
153129	FLJ90709	hypothetical protein FLJ90709	4	16	10
8986	RPS6KA4	ribosomal protein S6 kinase, 90kDa, polypeptide 4	4	16	10
4091	SMAD6	SMAD family member 6	4	16	10
23219	FBXO28	F-box protein 28	2	18	10
3054	HCFC1	host cell factor C1 (VP16-accessory protein)	0	20	10
901	CCNG2	cyclin G2	0	20	10
84433	CARD11	caspase recruitment domain family, member 11	0	20	10
9781	RNF144	ring finger protein 144	0	20	10
3705	ITPK1	inositol 1,3,4-triphosphate 5/6 kinase	0	20	10
201626	2'-PDE	2'-phosphodiesterase	0	20	10
130355	LOC130355	hypothetical protein LOC130355	0	20	10
23301	EHBP1	EH domain binding protein 1	0	20	10
5481	PPID	peptidylprolyl isomerase D (cyclophilin D)	0	20	10
55729	ATF7IP	activating transcription factor 7 interacting protein	0	20	10
57158	JPH2	junctophilin 2	0	20	10
54956	PARP16	poly (ADP-ribose) polymerase family, member 16	0	20	10
29937	NENF	neuron derived neurotrophic factor	0	20	10
1347	COX7A2	cytochrome c oxidase subunit VIIa polypeptide 2 (liver)	0	20	10
55131	RBM28	RNA binding motif protein 28	0	20	10
55327	LIN7C	lin-7 homolog C (C. elegans)	0	20	10
23043	TNIK	TRAF2 and NCK interacting kinase	0	20	10
57829	ZP4	zona pellucida glycoprotein 4	0	20	10
51073	MRPL4	mitochondrial ribosomal protein L4	0	20	10
94059	LENG9	leukocyte receptor cluster (LRC) member 9	0	20	10
3748	KCNC3	potassium voltage-gated channel, Shaw-related subfamily, n	0	20	10
161582	DYX1C1	dyslexia susceptibility 1 candidate 1	0	20	10
3698	ITIH2	inter-alpha (globulin) inhibitor H2	0	20	10
3431	SP110	SP110 nuclear body protein	0	20	10
253558	LYCAT	lysocardiolipin acyltransferase	0	20	10
25948	KBTBD2	kelch repeat and BTB (POZ) domain containing 2	0	20	10
9021	SOCS3	suppressor of cytokine signaling 3	0	20	10
144100	PLEKHA7	pleckstrin homology domain containing, family A member 7	0	20	10
55122	C6orf166	chromosome 6 open reading frame 166	0	20	10
3706	ITPKA	inositol 1,4,5-trisphosphate 3-kinase A	0	20	10
4301	MLLT4	myeloid/lymphoid or mixed-lineage leukemia (trithorax homo	0	20	10
54212	SNTG1	syntrophin, gamma 1	0	20	10
102	ADAM10	ADAM metallopeptidase domain 10	0	20	10
6622	SNCA	synuclein, alpha (non A4 component of amyloid precursor)	0	20	10
84515	MCM8	MCM8 minichromosome maintenance deficient 8 (S. cerevis	0	20	10
54855	FAM46C	family with sequence similarity 46, member C	0	20	10
4239	MFAP4	microfibrillar-associated protein 4	0	20	10
55582	KIF27	kinesin family member 27	0	20	10

24138	IFIT5	interferon-induced protein with tetratricopeptide repeats 5	0	20	10
10156	RASA4	RAS p21 protein activator 4	0	20	10
2638	GC	group-specific component (vitamin D binding protein)	0	20	10
51386	EIF3S6IP	eukaryotic translation initiation factor 3, subunit 6 interacting	0	20	10
832	CAPZB	capping protein (actin filament) muscle Z-line, beta	0	20	10
54505	DHX29	DEAH (Asp-Glu-Ala-His) box polypeptide 29	0	20	10
146923	RUNDC1	RUN domain containing 1	0	20	10
8781	PSPHL	phosphoserine phosphatase-like	20	0	10
196394	AMN1	antagonist of mitotic exit network 1 homolog (S. cerevisiae)	20	0	10
29035	C16orf72	chromosome 16 open reading frame 72	20	0	10
114990	VASN	vasorin	20	0	10
9879	DDX46	DEAD (Asp-Glu-Ala-Asp) box polypeptide 46	20	0	10
123099	DEGS2	degenerative spermatocyte homolog 2, lipid desaturase (Drc	20	0	10
283869	NPW	neuropeptide W	20	0	10
79191	IRX3	iroquois homeobox protein 3	20	0	10
116285	ACSM1	acyl-CoA synthetase medium-chain family member 1	20	0	10
115817	DHRS1	dehydrogenase/reductase (SDR family) member 1	20	0	10
79794	C12orf49	chromosome 12 open reading frame 49	20	0	10
54887	C6orf107	chromosome 6 open reading frame 107	20	0	10
117247	SLC16A10	solute carrier family 16, member 10 (aromatic amino acid tra	20	0	10
3572	IL6ST	interleukin 6 signal transducer (gp130, oncostatin M receptor	20	0	10
84277	WBSCR18	Williams Beuren syndrome chromosome region 18	20	0	10
26122	EPC2	enhancer of polycomb homolog 2 (Drosophila)	20	0	10
6448	SGSH	N-sulfoglucosamine sulfohydrolase (sulfamidase)	20	0	10
4045	LSAMP	limbic system-associated membrane protein	20	0	10
441024	MTHFD2L	methylenetetrahydrofolate dehydrogenase (NADP+ depende	17	2	9.5
29925	GMPPB	GDP-mannose pyrophosphorylase B	17	2	9.5
8437	RASAL1	RAS protein activator like 1 (GAP1 like)	17	2	9.5
151534	LOC151534	hypothetical protein BC009264	16	3	9.5
55251	PCMTD2	protein-L-isoaspartate (D-aspartate) O-methyltransferase do	16	3	9.5
51385	ZNF589	zinc finger protein 589	16	3	9.5
84458	LCOR	ligand dependent nuclear receptor corepressor	16	3	9.5
56652	PEO1	progressive external ophthalmoplegia 1	14	5	9.5
285672	P18SRP	P18SRP protein	14	5	9.5
5255	PHKA1	phosphorylase kinase, alpha 1 (muscle)	14	5	9.5
375757	C9orf119	chromosome 9 open reading frame 119	14	5	9.5
6988	TCTA	T-cell leukemia translocation altered gene	14	5	9.5
79098	C1orf116	chromosome 1 open reading frame 116	13	6	9.5
85865	GTPBP10	GTP-binding protein 10 (putative)	12	7	9.5
1838	DTNB	dystrobrevin, beta	12	7	9.5
143187	VTI1A	vesicle transport through interaction with t-SNAREs homolog	12	7	9.5
51274	KLF3	Kruppel-like factor 3 (basic)	10	9	9.5
1545	CYP1B1	cytochrome P450, family 1, subfamily B, polypeptide 1	8	11	9.5
55219	TMEM57	transmembrane protein 57	8	11	9.5
155061	ZNF746	zinc finger protein 746	8	11	9.5
51085	MLXIPL	MLX interacting protein-like	8	11	9.5
283742	FAM98B	family with sequence similarity 98, member B	8	11	9.5
84439	KIAA1822	KIAA1822	8	11	9.5
8654	PDE5A	phosphodiesterase 5A, cGMP-specific	6	13	9.5
23176	SEP8	septin 8	6	13	9.5
254263	CNIH2	cornichon homolog 2 (Drosophila)	6	13	9.5

64422	ATG3	ATG3 autophagy related 3 homolog (S. cerevisiae)	4	15	9.5
317649	EIF4E3	eukaryotic translation initiation factor 4E family member 3	4	15	9.5
26149	ZNF658	zinc finger protein 658	4	15	9.5
83878	USHBP1	Usher syndrome 1C binding protein 1	2	17	9.5
2220	FCN2	ficolin (collagen/fibrinogen domain containing lectin) 2 (hucol	2	17	9.5
7430	VIL2	villin 2 (ezrin)	2	17	9.5
79053	ALG8	asparagine-linked glycosylation 8 homolog (S. cerevisiae, al	2	17	9.5
7760	ZNF213	zinc finger protein 213	2	17	9.5
143384	C10orf46	chromosome 10 open reading frame 46	0	19	9.5
282969	C10orf125	chromosome 10 open reading frame 125	0	19	9.5
54778	RNF111	ring finger protein 111	0	19	9.5
25873	RPL36	ribosomal protein L36	0	19	9.5
7525	YES1	v-yes-1 Yamaguchi sarcoma viral oncogene homolog 1	0	19	9.5
54805	CNNM2	cyclin M2	0	19	9.5
55841	WWC3	WWC family member 3	0	19	9.5
55343	SLC35C1	solute carrier family 35, member C1	0	19	9.5
4330	MN1	meningioma (disrupted in balanced translocation) 1	0	19	9.5
9054	NFS1	NFS1 nitrogen fixation 1 homolog (S. cerevisiae)	0	19	9.5
80201	HKDC1	hexokinase domain containing 1	0	19	9.5
79623	GALNT14	UDP-N-acetyl-alpha-D-galactosamine:polypeptide N-acetylg	0	19	9.5
51421	AMOTL2	angiomotin like 2	0	19	9.5
140685	ZBTB46	zinc finger and BTB domain containing 46	0	19	9.5
286256	LCN12	lipocalcin 12	0	19	9.5
29071	C1GALT1C1	C1GALT1-specific chaperone 1	0	19	9.5
3763	KCNJ6	potassium inwardly-rectifying channel, subfamily J, member	0	19	9.5
9025	RNF8	ring finger protein 8	0	19	9.5
85459	KIAA1731	KIAA1731	0	19	9.5
10262	SF3B4	splicing factor 3b, subunit 4, 49kDa	0	19	9.5
114112	TXNRD3	thioredoxin reductase 3	0	19	9.5
58493	C9orf80	chromosome 9 open reading frame 80	0	19	9.5
84941	HSH2D	hematopoietic SH2 domain containing	0	19	9.5
56905	C15orf39	chromosome 15 open reading frame 39	0	19	9.5
26220	DGCR5	DiGeorge syndrome critical region gene 5 (non-coding)	0	19	9.5
4040	LRP6	low density lipoprotein receptor-related protein 6	0	19	9.5
379013	RNF138P1	ring finger protein 138 pseudogene 1	0	19	9.5
79085	SLC25A23	solute carrier family 25 (mitochondrial carrier; phosphate car	19	0	9.5
54553	DKFZP434I0	hypothetical protein DKFZP434I0714	19	0	9.5
5581	PRKCE	protein kinase C, epsilon	19	0	9.5
55156	ARMC1	armadillo repeat containing 1	19	0	9.5
8729	GBF1	golgi-specific brefeldin A resistance factor 1	19	0	9.5
84837	C14orf128	chromosome 14 open reading frame 128	19	0	9.5
10634	GAS2L1	growth arrest-specific 2 like 1	19	0	9.5
221424	C6orf154	chromosome 6 open reading frame 154	19	0	9.5
9351	SLC9A3R2	solute carrier family 9 (sodium/hydrogen exchanger), membe	19	0	9.5
6992	PPP1R11	protein phosphatase 1, regulatory (inhibitor) subunit 11	19	0	9.5
1340	COX6B1	cytochrome c oxidase subunit Vib polypeptide 1 (ubiquitous)	19	0	9.5
53340	SPA17	sperm autoantigenic protein 17	19	0	9.5
51362	CDC40	cell division cycle 40 homolog (S. cerevisiae)	19	0	9.5
22913	RALY	RNA binding protein, autoantigenic (hnRNP-associated with	19	0	9.5
10465	PPIH	peptidylprolyl isomerase H (cyclophilin H)	19	0	9.5
1810	DR1	down-regulator of transcription 1, TBP-binding (negative cofa	19	0	9.5

150051	LOC150051	hypothetical LOC150051	19	0	9.5
7041	TGFB1I1	transforming growth factor beta 1 induced transcript 1	19	0	9.5
55556	ENOSF1	enolase superfamily member 1	19	0	9.5
3663	IRF5	interferon regulatory factor 5	19	0	9.5
55349	CHDH	choline dehydrogenase	19	0	9.5
170679	PSORS1C1	psoriasis susceptibility 1 candidate 1	19	0	9.5
5931	RBBP7	retinoblastoma binding protein 7	19	0	9.5
338707	B4GALNT4	beta-1,4-N-acetyl-galactosaminyl transferase 4	19	0	9.5
1634	DCN	decorin	19	0	9.5
6599	SMARCC1	SWI/SNF related, matrix associated, actin dependent regula	19	0	9.5
220965	FAM13C1	family with sequence similarity 13, member C1	19	0	9.5
22905	EPN2	epsin 2	16	2	9
30001	ERO1L	ERO1-like (S. cerevisiae)	13	5	9
140462	ASB9	ankyrin repeat and SOCS box-containing 9	13	5	9
221545	C6orf136	chromosome 6 open reading frame 136	12	6	9
5424	POLD1	polymerase (DNA directed), delta 1, catalytic subunit 125kDa	10	8	9
90673	PPP1R3E	protein phosphatase 1, regulatory (inhibitor) subunit 3E	10	8	9
54934	C12orf41	chromosome 12 open reading frame 41	10	8	9
10197	PSME3	proteasome (prosome, macropain) activator subunit 3 (PA28	10	8	9
27342	RABGEF1	RAB guanine nucleotide exchange factor (GEF) 1	10	8	9
3800	KIF5C	kinesin family member 5C	10	8	9
90809	TMEM55B	transmembrane protein 55B	10	8	9
6820	SULT2B1	sulfotransferase family, cytosolic, 2B, member 1	10	8	9
89782	LMLN	leishmanolysin-like (metallopeptidase M8 family)	10	8	9
9827	KIAA0258	KIAA0258	8	10	9
10327	AKR1A1	aldo-keto reductase family 1, member A1 (aldehyde reducta	6	12	9
134549	SHROOM1	shroom family member 1	4	14	9
9666	DZIP3	zinc finger DAZ interacting protein 3	4	14	9
60313	GPBP1L1	GC-rich promoter binding protein 1-like 1	4	14	9
2317	FLNB	filamin B, beta (actin binding protein 278)	2	16	9
51100	SH3GLB1	SH3-domain GRB2-like endophilin B1	0	18	9
6650	SOLH	small optic lobes homolog (Drosophila)	0	18	9
5325	PLAGL1	pleiomorphic adenoma gene-like 1	0	18	9
3910	LAMA4	laminin, alpha 4	0	18	9
9721	GPRIN2	G protein regulated inducer of neurite outgrowth 2	0	18	9
5198	PFAS	phosphoribosylformylglycinamidine synthase (FGAR amidotr	0	18	9
81796	SLCO5A1	solute carrier organic anion transporter family, member 5A1	0	18	9
85329	LGALS12	lectin, galactoside-binding, soluble, 12 (galectin 12)	0	18	9
5116	PCNT	pericentrin (kendrin)	0	18	9
157680	VPS13B	vacuolar protein sorting 13 homolog B (yeast)	0	18	9
160622	GRASP	GRP1 (general receptor for phosphoinositides 1)-associated	0	18	9
54839	LRRC49	leucine rich repeat containing 49	0	18	9
114796	KIAA1908	KIAA1908 protein	0	18	9
8800	PEX11A	peroxisomal biogenesis factor 11A	0	18	9
55755	CDK5RAP2	CDK5 regulatory subunit associated protein 2	0	18	9
4052	LTBP1	latent transforming growth factor beta binding protein 1	0	18	9
5082	PDCL	phosducin-like	0	18	9
27030	MLH3	mutL homolog 3 (E. coli)	0	18	9
3915	LAMC1	laminin, gamma 1 (formerly LAMB2)	0	18	9
6499	SKIV2L	superkiller viralicidic activity 2-like (S. cerevisiae)	0	18	9
10490	VTI1B	vesicle transport through interaction with t-SNAREs homolog	0	18	9

51109	RDH11	retinol dehydrogenase 11 (all-trans/9-cis/11-cis)	18	0	9
9842	PLEKHM1	pleckstrin homology domain containing, family M (with RUN	18	0	9
116987	CENTG2	centaurin, gamma 2	18	0	9
285676	ZNF454	zinc finger protein 454	18	0	9
2817	GPC1	glypican 1	18	0	9
79639	TMEM53	transmembrane protein 53	18	0	9
219972	MPEG1	macrophage expressed gene 1	18	0	9
11099	PTPN21	protein tyrosine phosphatase, non-receptor type 21	18	0	9
9245	GCNT3	glucosaminyl (N-acetyl) transferase 3, mucin type	18	0	9
7555	CNBP	CCHC-type zinc finger, nucleic acid binding protein	18	0	9
54764	ZRANB1	zinc finger, RAN-binding domain containing 1	18	0	9
718	C3	complement component 3	18	0	9
10581	IFITM2	interferon induced transmembrane protein 2 (1-8D)	18	0	9
80821	DDHD1	DDHD domain containing 1	18	0	9
1676	DFFA	DNA fragmentation factor, 45kDa, alpha polypeptide	18	0	9
9476	NAPSA	napsin A aspartic peptidase	18	0	9
151162	LOC151162	hypothetical protein LOC151162	18	0	9
126133	ALDH16A1	aldehyde dehydrogenase 16 family, member A1	18	0	9
23035	PHLPPL	PH domain and leucine rich repeat protein phosphatase-like	18	0	9
57016	AKR1B10	aldo-keto reductase family 1, member B10 (aldose reductase	18	0	9
8622	PDE8B	phosphodiesterase 8B	18	0	9
349565	NMNAT3	nicotinamide nucleotide adenylyltransferase 3	18	0	9
84736	MGC10850	hypothetical protein MGC10850	18	0	9
84916	CIRH1A	cirrhosis, autosomal recessive 1A (cirhin)	18	0	9
6901	TAZ	tafazzin (cardiomyopathy, dilated 3A (X-linked); endocardial	18	0	9
128989	C22orf25	chromosome 22 open reading frame 25	18	0	9
8372	HYAL3	hyaluronoglucosaminidase 3	18	0	9
2257	FGF12	fibroblast growth factor 12	15	2	8.5
3608	ILF2	interleukin enhancer binding factor 2, 45kDa	15	2	8.5
374378	GALNTL4	UDP-N-acetyl-alpha-D-galactosamine:polypeptide N-acetylg	14	3	8.5
6899	TBX1	T-box 1	14	3	8.5
26058	TNRC15	trinucleotide repeat containing 15	14	3	8.5
22938	SNW1	SNW domain containing 1	14	3	8.5
23731	C9orf5	chromosome 9 open reading frame 5	14	3	8.5
79679	VTCN1	V-set domain containing T cell activation inhibitor 1	12	5	8.5
55638	FLJ20366	hypothetical protein FLJ20366	12	5	8.5
1314	COPA	coatomer protein complex, subunit alpha	12	5	8.5
3185	HNRPF	heterogeneous nuclear ribonucleoprotein F	12	5	8.5
2909	GRLF1	glucocorticoid receptor DNA binding factor 1	12	5	8.5
124975	GGT6	gamma-glutamyltransferase 6 homolog (rat)	12	5	8.5
29887	SNX10	sorting nexin 10	10	7	8.5
83860	TAF3	TAF3 RNA polymerase II, TATA box binding protein (TBP)-a	10	7	8.5
753	C18orf1	chromosome 18 open reading frame 1	10	7	8.5
8028	MLLT10	myeloid/lymphoid or mixed-lineage leukemia (trithorax homo	6	11	8.5
81550	TDRD3	tudor domain containing 3	4	13	8.5
6536	SLC6A9	solute carrier family 6 (neurotransmitter transporter, glycine),	4	13	8.5
118426	LOH12CR1	loss of heterozygosity, 12, chromosomal region 1	4	13	8.5
57827	C6orf47	chromosome 6 open reading frame 47	4	13	8.5
10174	SORBS3	sorbin and SH3 domain containing 3	4	13	8.5
84315	MON1A	MON1 homolog A (yeast)	2	15	8.5
352954	GATS	opposite strand transcription unit to STAG3	2	15	8.5

83871	RAB34	RAB34, member RAS oncogene family	2	15	8.5
80212	CCDC92	coiled-coil domain containing 92	2	15	8.5
283788	LOC283788	hypothetical protein LOC283788	2	15	8.5
84333	PCGF5	polycomb group ring finger 5	2	15	8.5
929	CD14	CD14 molecule	2	15	8.5
9659	PDE4DIP	phosphodiesterase 4D interacting protein (myomegalin)	2	15	8.5
27343	POLL	polymerase (DNA directed), lambda	2	15	8.5
10806	SDCCAG8	serologically defined colon cancer antigen 8	2	15	8.5
79982	DNAJB14	DnaJ (Hsp40) homolog, subfamily B, member 14	0	17	8.5
26030	PLEKHG3	pleckstrin homology domain containing, family G (with RhoG	0	17	8.5
18	ABAT	4-aminobutyrate aminotransferase	0	17	8.5
84662	GLIS2	GLIS family zinc finger 2	0	17	8.5
51306	C5orf5	chromosome 5 open reading frame 5	0	17	8.5
7703	PCGF2	polycomb group ring finger 2	0	17	8.5
7291	TWIST1	twist homolog 1 (acrocephalosyndactyly 3; Saethre-Chotzen	0	17	8.5
30845	EHD3	EH-domain containing 3	0	17	8.5
3455	IFNAR2	interferon (alpha, beta and omega) receptor 2	0	17	8.5
84660	CCDC62	coiled-coil domain containing 62	0	17	8.5
196264	LOC196264	hypothetical protein LOC196264	0	17	8.5
123775	C16orf46	chromosome 16 open reading frame 46	0	17	8.5
7068	THRB	thyroid hormone receptor, beta (erythroblastic leukemia viral	0	17	8.5
401164	LOC401164	hypothetical gene supported by AY494056	0	17	8.5
140707	BRI3BP	BRI3 binding protein	0	17	8.5
55565	LOC55565	hypothetical protein LOC55565	0	17	8.5
4680	CEACAM6	carcinoembryonic antigen-related cell adhesion molecule 6 (	0	17	8.5
158326	FREM1	FRAS1 related extracellular matrix 1	0	17	8.5
7019	TFAM	transcription factor A, mitochondrial	0	17	8.5
400410	LOC400410	similar to cervical cancer suppressor-1	0	17	8.5
947	CD34	CD34 molecule	0	17	8.5
9668	ZNF432	zinc finger protein 432	0	17	8.5
23129	PLXND1	plexin D1	0	17	8.5
8803	SUCLA2	succinate-CoA ligase, ADP-forming, beta subunit	0	17	8.5
1609	DGKQ	diacylglycerol kinase, theta 110kDa	0	17	8.5
57510	XPO5	exportin 5	0	17	8.5
8805	TRIM24	tripartite motif-containing 24	0	17	8.5
5097	PCDH1	protocadherin 1 (cadherin-like 1)	17	0	8.5
283209	PGM2L1	phosphoglucomutase 2-like 1	17	0	8.5
9656	MDC1	mediator of DNA damage checkpoint 1	17	0	8.5
58525	WIZ	WIZ zinc finger	17	0	8.5
60485	SAV1	salvador homolog 1 (Drosophila)	17	0	8.5
4323	MMP14	matrix metallopeptidase 14 (membrane-inserted)	17	0	8.5
10178	ODZ1	odz, odd Oz/ten-m homolog 1(Drosophila)	17	0	8.5
27434	POLM	polymerase (DNA directed), mu	17	0	8.5
10333	TLR6	toll-like receptor 6	17	0	8.5
2189	FANCG	Fanconi anemia, complementation group G	17	0	8.5
25961	NUDT13	nudix (nucleoside diphosphate linked moiety X)-type motif 1[illegible]	17	0	8.5
64429	ZDHHC6	zinc finger, DHHC-type containing 6	17	0	8.5
729115	LOC729115	hypothetical protein LOC729115	17	0	8.5
23269	MGA	MAX gene associated	17	0	8.5
130340	AP1S3	adaptor-related protein complex 1, sigma 3 subunit	17	0	8.5
375704	C9orf165	chromosome 9 open reading frame 165	17	0	8.5

56912	C11orf60	chromosome 11 open reading frame 60	17	0	8.5
84255	SLC37A3	solute carrier family 37 (glycerol-3-phosphate transporter), m	17	0	8.5
81844	TRIM56	tripartite motif-containing 56	17	0	8.5
84953	MICALCL	MICAL C-terminal like	17	0	8.5
5000	ORC4L	origin recognition complex, subunit 4-like (yeast)	17	0	8.5
114823	LENG8	leukocyte receptor cluster (LRC) member 8	17	0	8.5
1462	CSPG2	chondroitin sulfate proteoglycan 2 (versican)	17	0	8.5
55174	INTS10	integrator complex subunit 10	17	0	8.5
23037	PDZD2	PDZ domain containing 2	17	0	8.5
10973	ASCC3	activating signal cointegrator 1 complex subunit 3	17	0	8.5
80117	ARL14	ADP-ribosylation factor-like 14	17	0	8.5
51104	C9orf77	chromosome 9 open reading frame 77	17	0	8.5
79607	FAM118B	family with sequence similarity 118, member B	14	2	8
80097	FAM128B	family with sequence similarity 128, member B	14	2	8
1760	DMPK	dystrophia myotonica-protein kinase	14	2	8
23300	ASCIZ	ATM/ATR-Substrate Chk2-Interacting Zn2+-finger protein	14	2	8
63910	C20orf59	chromosome 20 open reading frame 59	14	2	8
79575	ABHD8	abhydrolase domain containing 8	14	2	8
339201	C17orf65	chromosome 17 open reading frame 65	13	3	8
114799	ESCO1	establishment of cohesion 1 homolog 1 (S. cerevisiae)	13	3	8
406991	MIRN21	microRNA 21	13	3	8
64759	TNS3	tensin 3	12	4	8
81579	PLA2G12A	phospholipase A2, group XIIA	10	6	8
80306	MED28	mediator of RNA polymerase II transcription, subunit 28 hom	10	6	8
80179	MYOHD1	myosin head domain containing 1	10	6	8
728215	LOC728215	similar to transmembrane protein 28	8	8	8
5108	PCM1	pericentriolar material 1	8	8	8
91750	LIN52	lin-52 homolog (C. elegans)	8	8	8
9657	IQCB1	IQ motif containing B1	8	8	8
160518	MGC24039	hypothetical protein MGC24039	8	8	8
7517	XRCC3	X-ray repair complementing defective repair in Chinese ham	8	8	8
27314	RAB30	RAB30, member RAS oncogene family	8	8	8
400950	LOC400950	FLJ42903 protein	6	10	8
6223	RPS19	ribosomal protein S19	6	10	8
3423	IDS	iduronate 2-sulfatase (Hunter syndrome)	6	10	8
10347	ABCA7	ATP-binding cassette, sub-family A (ABC1), member 7	6	10	8
55968	NSFL1C	NSFL1 (p97) cofactor (p47)	4	12	8
23231	KIAA0746	KIAA0746 protein	4	12	8
22890	ZBTB1	zinc finger and BTB domain containing 1	4	12	8
2800	GOLGA1	golgi autoantigen, golgin subfamily a, 1	4	12	8
11332	ACOT7	acyl-CoA thioesterase 7	2	14	8
5588	PRKCQ	protein kinase C, theta	2	14	8
134429	STARD4	START domain containing 4, sterol regulated	2	14	8
5530	PPP3CA	protein phosphatase 3 (formerly 2B), catalytic subunit, alpha	0	16	8
10346	TRIM22	tripartite motif-containing 22	0	16	8
25907	TMEM158	transmembrane protein 158	0	16	8
55957	F25965	protein F25965	0	16	8
84514	GHDC	GH3 domain containing	0	16	8
6726	SRP9	signal recognition particle 9kDa	0	16	8
7485	WRB	tryptophan rich basic protein	0	16	8
5790	PTPRCAP	protein tyrosine phosphatase, receptor type, C-associated pr	0	16	8

80725	SNIP	SNAP25-interacting protein	0	16	8
5754	PTK7	PTK7 protein tyrosine kinase 7	0	16	8
64410	KLHL25	kelch-like 25 (Drosophila)	0	16	8
10610	ST6GALNAC	ST6 (alpha-N-acetyl-neuraminyl-2,3-beta-galactosyl-1,3)-N-a	0	16	8
11333	PDAP1	PDGFA associated protein 1	0	16	8
4862	NPAS2	neuronal PAS domain protein 2	0	16	8
653319	LOC653319	hypothetical protein LOC653319	0	16	8
6397	SEC14L1	SEC14-like 1 (S. cerevisiae)	0	16	8
79149	ZSCAN5	zinc finger and SCAN domain containing 5	0	16	8
65250	FLJ13231	hypothetical protein FLJ13231	0	16	8
9832	JAKMIP2	janus kinase and microtubule interacting protein 2	0	16	8
5290	PIK3CA	phosphoinositide-3-kinase, catalytic, alpha polypeptide	0	16	8
8514	KCNAB2	potassium voltage-gated channel, shaker-related subfamily,	0	16	8
57326	PBXIP1	pre-B-cell leukemia transcription factor interacting protein 1	0	16	8
9569	GTF2IRD1	GTF2I repeat domain containing 1	0	16	8
64092	SAMSN1	SAM domain, SH3 domain and nuclear localization signals 1	0	16	8
1384	CRAT	carnitine acetyltransferase	0	16	8
8942	KYNU	kynureninase (L-kynurenine hydrolase)	0	16	8
728537	LOC728537	hypothetical protein LOC728537	0	16	8
284805	FLJ33706	hypothetical protein FLJ33706	0	16	8
54882	ANKHD1	ankyrin repeat and KH domain containing 1	0	16	8
338799	LOC338799	hypothetical locus LOC338799	16	0	8
10135	PBEF1	pre-B-cell colony enhancing factor 1	16	0	8
79646	PANK3	pantothenate kinase 3	16	0	8
8226	HDHD1A	haloacid dehalogenase-like hydrolase domain containing 1A	16	0	8
4302	MLLT6	myeloid/lymphoid or mixed-lineage leukemia (trithorax homo	16	0	8
113402	SFT2D1	SFT2 domain containing 1	16	0	8
84966	IGSF21	immunoglobin superfamily, member 21	16	0	8
387103	C6orf173	chromosome 6 open reading frame 173	16	0	8
10000	AKT3	v-akt murine thymoma viral oncogene homolog 3 (protein kir	16	0	8
55670	PEX26	peroxisome biogenesis factor 26	16	0	8
4325	MMP16	matrix metallopeptidase 16 (membrane-inserted)	16	0	8
4637	MYL6	myosin, light chain 6, alkali, smooth muscle and non-muscle	16	0	8
10991	SLC38A3	solute carrier family 38, member 3	16	0	8
10005	ACOT8	acyl-CoA thioesterase 8	16	0	8
9816	KIAA0133	KIAA0133	16	0	8
23160	WDR43	WD repeat domain 43	16	0	8
730200	LOC730200	hypothetical protein LOC730200	16	0	8
115330	GPR146	G protein-coupled receptor 146	16	0	8
645	BLVRB	biliverdin reductase B (flavin reductase (NADPH))	16	0	8
79109	MAPKAP1	mitogen-activated protein kinase associated protein 1	16	0	8
494024	LOC494024	similar to 60S ribosomal protein L23a	16	0	8
10985	GCN1L1	GCN1 general control of amino-acid synthesis 1-like 1 (yeas	16	0	8
134145	LOC134145	hypothetical protein LOC134145	16	0	8
956	ENTPD3	ectonucleoside triphosphate diphosphohydrolase 3	16	0	8
147740	CPLP	complement C3 protein (GPC3) precursor-like	16	0	8
29	ABR	active BCR-related gene	13	2	7.5
2632	GBE1	glucan (1,4-alpha-), branching enzyme 1 (glycogen branchin	13	2	7.5
729092	CTGLF2	centaurin, gamma-like family, member 2	13	2	7.5
116448	OLIG1	oligodendrocyte transcription factor 1	13	2	7.5
10239	AP3S2	adaptor-related protein complex 3, sigma 2 subunit	13	2	7.5

4176	MCM7	MCM7 minichromosome maintenance deficient 7 (S. cerevis	12	3	7.5
51082	POLR1D	polymerase (RNA) I polypeptide D, 16kDa	12	3	7.5
10608	MXD4	MAX dimerization protein 4	12	3	7.5
1040	CDS1	CDP-diacylglycerol synthase (phosphatidate cytidylyltransfer	12	3	7.5
162962	FLJ16287	FLJ16287 protein	12	3	7.5
83544	DNAL1	dynein, axonemal, light chain 1	12	3	7.5
6304	SATB1	special AT-rich sequence binding protein 1 (binds to nuclear	12	3	7.5
23543	RBM9	RNA binding motif protein 9	12	3	7.5
84498	FAM120B	family with sequence similarity 120B	12	3	7.5
55102	C14orf103	chromosome 14 open reading frame 103	10	5	7.5
400618	FLJ37644	hypothetical gene supported by AK094963	10	5	7.5
284058	KIAA1267	KIAA1267	10	5	7.5
124540	MSI2	musashi homolog 2 (Drosophila)	10	5	7.5
51372	CCDC72	coiled-coil domain containing 72	10	5	7.5
23259	DDHD2	DDHD domain containing 2	10	5	7.5
57541	ZNF398	zinc finger protein 398	10	5	7.5
10013	HDAC6	histone deacetylase 6	8	7	7.5
80314	EPC1	enhancer of polycomb homolog 1 (Drosophila)	8	7	7.5
64080	RBKS	ribokinase	8	7	7.5
3778	KCNMA1	potassium large conductance calcium-activated channel, sub	8	7	7.5
79921	TCEAL4	transcription elongation factor A (SII)-like 4	8	7	7.5
54904	WHSC1L1	Wolf-Hirschhorn syndrome candidate 1-like 1	8	7	7.5
283846	DKFZp547E(	hypothetical gene LOC283846	8	7	7.5
90060	CCDC120	coiled-coil domain containing 120	8	7	7.5
5125	PCSK5	proprotein convertase subtilisin/kexin type 5	6	9	7.5
113510	HEL308	DNA helicase HEL308	6	9	7.5
5376	PMP22	peripheral myelin protein 22	6	9	7.5
2036	EPB41L1	erythrocyte membrane protein band 4.1-like 1	4	11	7.5
3017	HIST1H2BD	histone cluster 1, H2bd	4	11	7.5
339352	LOC339352	similar to ATP binding domain 3	4	11	7.5
79954	NOL10	nucleolar protein 10	4	11	7.5
11069	RAPGEF4	Rap guanine nucleotide exchange factor (GEF) 4	2	13	7.5
65220	NADK	NAD kinase	2	13	7.5
89886	SLAMF9	SLAM family member 9	2	13	7.5
56886	UGCGL1	UDP-glucose ceramide glucosyltransferase-like 1	2	13	7.5
115286	SLC25A26	solute carrier family 25, member 26	0	15	7.5
157739	TDH	L-threonine dehydrogenase	0	15	7.5
23252	OTUD3	OTU domain containing 3	0	15	7.5
55626	FLJ20294	hypothetical protein FLJ20294	0	15	7.5
728928	LOC728928	hypothetical protein LOC728928	0	15	7.5
9717	SEC14L5	SEC14-like 5 (S. cerevisiae)	0	15	7.5
57558	USP35	ubiquitin specific peptidase 35	0	15	7.5
9411	ARHGAP29	Rho GTPase activating protein 29	0	15	7.5
9747	KIAA0738	KIAA0738 gene product	0	15	7.5
6235	RPS29	ribosomal protein S29	0	15	7.5
401271	FLJ42177	FLJ42177 protein	0	15	7.5
642987	FLJ43080	hypothetical protein LOC642986	0	15	7.5
5500	PPP1CB	protein phosphatase 1, catalytic subunit, beta isoform	0	15	7.5
284716	FAM80A	family with sequence similarity 80, member A	0	15	7.5
345499	BRCTD1	BRCT domain containing 1	0	15	7.5
84219	WDR24	WD repeat domain 24	0	15	7.5

84749	USP30	ubiquitin specific peptidase 30	0	15	7.5
7265	TTC1	tetratricopeptide repeat domain 1	0	15	7.5
1984	EIF5A	eukaryotic translation initiation factor 5A	0	15	7.5
259232	VGCNL1	voltage gated channel like 1	0	15	7.5
333	APLP1	amyloid beta (A4) precursor-like protein 1	0	15	7.5
5743	PTGS2	prostaglandin-endoperoxide synthase 2 (prostaglandin G/H s	0	15	7.5
158427	C9orf97	chromosome 9 open reading frame 97	0	15	7.5
114885	OSBPL11	oxysterol binding protein-like 11	0	15	7.5
26093	CCDC9	coiled-coil domain containing 9	0	15	7.5
57511	COG6	component of oligomeric golgi complex 6	0	15	7.5
57221	KIAA1244	KIAA1244	0	15	7.5
28227	PPP2R3B	protein phosphatase 2 (formerly 2A), regulatory subunit B'', b	0	15	7.5
375346	TMEM110	transmembrane protein 110	0	15	7.5
54762	GRAMD1C	GRAM domain containing 1C	0	15	7.5
10641	TUSC4	tumor suppressor candidate 4	0	15	7.5
6301	SARS	seryl-tRNA synthetase	0	15	7.5
23658	LSM5	LSM5 homolog, U6 small nuclear RNA associated (S. cerevi	0	15	7.5
5911	RAP2A	RAP2A, member of RAS oncogene family	0	15	7.5
50618	ITSN2	intersectin 2	0	15	7.5
60491	NIF3L1	NIF3 NGG1 interacting factor 3-like 1 (S. pombe)	0	15	7.5
10299	MAR6	membrane-associated ring finger (C3HC4) 6	0	15	7.5
4281	MID1	midline 1 (Opitz/BBB syndrome)	0	15	7.5
643702	NA	NA	0	15	7.5
5582	PRKCG	protein kinase C, gamma	0	15	7.5
2160	F11	coagulation factor XI (plasma thromboplastin antecedent)	0	15	7.5
8562	DENR	density-regulated protein	0	15	7.5
57621	ZBTB2	zinc finger and BTB domain containing 2	0	15	7.5
55326	AGPAT5	1-acylglycerol-3-phosphate O-acyltransferase 5 (lysophosph	0	15	7.5
157807	MGC34646	hypothetical protein MGC34646	0	15	7.5
220972	MAR8	membrane-associated ring finger (C3HC4) 8	0	15	7.5
29081	METTL5	methyltransferase like 5	0	15	7.5
80854	SETD7	SET domain containing (lysine methyltransferase) 7	0	15	7.5
56926	NCLN	nicalin homolog (zebrafish)	0	15	7.5
64149	C17orf75	chromosome 17 open reading frame 75	0	15	7.5
144453	BEST3	bestrophin 3	0	15	7.5
9925	ZBTB5	zinc finger and BTB domain containing 5	0	15	7.5
51339	DACT1	dapper, antagonist of beta-catenin, homolog 1 (Xenopus lae	0	15	7.5
774	CACNA1B	calcium channel, voltage-dependent, L type, alpha 1B subun	0	15	7.5
9856	KIAA0319	KIAA0319	0	15	7.5
440900	LOC440900	hypothetical LOC440900	15	0	7.5
91461	LOC91461	hypothetical protein BC007901	15	0	7.5
114327	EFHC1	EF-hand domain (C-terminal) containing 1	15	0	7.5
11073	TOPBP1	topoisomerase (DNA) II binding protein 1	15	0	7.5
4729	NDUFV2	NADH dehydrogenase (ubiquinone) flavoprotein 2, 24kDa	15	0	7.5
4092	SMAD7	SMAD family member 7	15	0	7.5
22930	RAB3GAP1	RAB3 GTPase activating protein subunit 1 (catalytic)	15	0	7.5
79056	PRRG4	proline rich Gla (G-carboxyglutamic acid) 4 (transmembrane)	15	0	7.5
7374	UNG	uracil-DNA glycosylase	15	0	7.5
5196	PF4	platelet factor 4 (chemokine (C-X-C motif) ligand 4)	15	0	7.5
57217	TTC7A	tetratricopeptide repeat domain 7A	15	0	7.5
151525	WDSUB1	WD repeat, sterile alpha motif and U-box domain containing	15	0	7.5

57661	KIAA1542	CTD-binding SR-like protein rA9	15	0	7.5
6284	S100A13	S100 calcium binding protein A13	15	0	7.5
90231	KIAA2013	KIAA2013	15	0	7.5
285343	C3orf23	chromosome 3 open reading frame 23	15	0	7.5
84320	ACBD6	acyl-Coenzyme A binding domain containing 6	15	0	7.5
256471	MGC33302	hypothetical protein MGC33302	15	0	7.5
84108	PCGF6	polycomb group ring finger 6	15	0	7.5
92822	ZNF276	zinc finger protein 276	15	0	7.5
1010	CDH12	cadherin 12, type 2 (N-cadherin 2)	15	0	7.5
56950	SMYD2	SET and MYND domain containing 2	15	0	7.5
55500	ETNK1	ethanolamine kinase 1	15	0	7.5
400043	LOC400043	hypothetical gene supported by BC009385	15	0	7.5
528	ATP6V1C1	ATPase, H+ transporting, lysosomal 42kDa, V1 subunit C1	15	0	7.5
64359	NXN	nucleoredoxin	15	0	7.5
88455	ANKRD13A	ankyrin repeat domain 13A	15	0	7.5
345778	MTX3	metaxin 3	15	0	7.5
80185	C8orf41	chromosome 8 open reading frame 41	12	2	7
79820	C14orf161	chromosome 14 open reading frame 161	12	2	7
1131	CHRM3	cholinergic receptor, muscarinic 3	12	2	7
55869	HDAC8	histone deacetylase 8	12	2	7
378805	FLJ43663	hypothetical protein FLJ43663	12	2	7
55082	FLJ10154	hypothetical protein FLJ10154	12	2	7
84866	TMEM25	transmembrane protein 25	12	2	7
55252	ASXL2	additional sex combs like 2 (Drosophila)	12	2	7
2272	FHIT	fragile histidine triad gene	12	2	7
8243	SMC1A	structural maintenance of chromosomes 1A	8	6	7
440423	SUZ12P	suppressor of zeste 12 homolog pseudogene	6	8	7
441478	MGC61598	similar to ankyrin-repeat protein Nrarp	6	8	7
63908	NAPB	N-ethylmaleimide-sensitive factor attachment protein, beta	6	8	7
114787	GPRIN1	G protein regulated inducer of neurite outgrowth 1	6	8	7
58533	SNX6	sorting nexin 6	4	10	7
1386	ATF2	activating transcription factor 2	4	10	7
54820	NDE1	nudE nuclear distribution gene E homolog 1 (A. nidulans)	4	10	7
11056	DDX52	DEAD (Asp-Glu-Ala-Asp) box polypeptide 52	4	10	7
10102	TSFM	Ts translation elongation factor, mitochondrial	4	10	7
1390	CREM	cAMP responsive element modulator	2	12	7
79858	NEK11	NIMA (never in mitosis gene a)- related kinase 11	2	12	7
54468	FLJ20323	hypothetical protein FLJ20323	2	12	7
11146	GLMN	glomulin, FKBP associated protein	2	12	7
3981	LIG4	ligase IV, DNA, ATP-dependent	2	12	7
10055	SAE1	SUMO1 activating enzyme subunit 1	2	12	7
574036	C1orf133	chromosome 1 open reading frame 133	0	14	7
10735	STAG2	stromal antigen 2	0	14	7
23040	MYT1L	myelin transcription factor 1-like	0	14	7
6227	RPS21	ribosomal protein S21	0	14	7
1311	COMP	cartilage oligomeric matrix protein	0	14	7
51491	HSPC111	hypothetical protein HSPC111	0	14	7
1050	CEBPA	CCAAT/enhancer binding protein (C/EBP), alpha	0	14	7
55011	NOP17	NOP17	0	14	7
493826	HCG12	HLA complex group 12	0	14	7
347736	TXNDC6	thioredoxin domain containing 6	0	14	7

7322	UBE2D2	ubiquitin-conjugating enzyme E2D 2 (UBC4/5 homolog, yeas	0	14	7
645212	LOC645212	hypothetical LOC645212	0	14	7
90268	FAM105B	family with sequence similarity 105, member B	0	14	7
382	ARF6	ADP-ribosylation factor 6	0	14	7
814	CAMK4	calcium/calmodulin-dependent protein kinase IV	0	14	7
5715	PSMD9	proteasome (prosome, macropain) 26S subunit, non-ATPase	0	14	7
26061	HACL1	2-hydroxyacyl-CoA lyase 1	0	14	7
55623	THUMPD1	THUMP domain containing 1	0	14	7
5295	PIK3R1	phosphoinositide-3-kinase, regulatory subunit 1 (p85 alpha)	0	14	7
55827	IQWD1	IQ motif and WD repeats 1	0	14	7
5321	PLA2G4A	phospholipase A2, group IVA (cytosolic, calcium-dependent)	0	14	7
729096	LOC729096	similar to BMS1-like, ribosome assembly protein	0	14	7
23534	TNPO3	transportin 3	14	0	7
114803	MYSM1	myb-like, SWIRM and MPN domains 1	14	0	7
5796	PTPRK	protein tyrosine phosphatase, receptor type, K	14	0	7
9519	TBPL1	TBP-like 1	14	0	7
55039	TRMT12	tRNA methyltransferase 12 homolog (S. cerevisiae)	14	0	7
200162	SPAG17	sperm associated antigen 17	14	0	7
284900	KIAA1648	KIAA1648 protein	14	0	7
79033	PRNPIP	prion protein interacting protein	14	0	7
6890	TAP1	transporter 1, ATP-binding cassette, sub-family B (MDR/TAF	14	0	7
636	BICD1	bicaudal D homolog 1 (Drosophila)	14	0	7
2332	FMR1	fragile X mental retardation 1	14	0	7
79868	CXorf45	chromosome X open reading frame 45	14	0	7
51308	REEP2	receptor accessory protein 2	14	0	7
23446	SLC44A1	solute carrier family 44, member 1	14	0	7
642648	LOC642648	hypothetical LOC642648	14	0	7
22978	NT5C2	5'-nucleotidase, cytosolic II	14	0	7
56949	XAB2	XPA binding protein 2	14	0	7
9953	HS3ST3B1	heparan sulfate (glucosamine) 3-O-sulfotransferase 3B1	14	0	7
4487	MSX1	msh homeobox 1	14	0	7
83464	APH1B	anterior pharynx defective 1 homolog B (C. elegans)	14	0	7
25847	ANAPC13	anaphase promoting complex subunit 13	14	0	7
9424	KCNK6	potassium channel, subfamily K, member 6	14	0	7
124751	KRBA2	KRAB-A domain containing 2	14	0	7
5871	MAP4K2	mitogen-activated protein kinase kinase kinase kinase 2	14	0	7
1869	E2F1	E2F transcription factor 1	14	0	7
155368	WBSCR27	Williams Beuren syndrome chromosome region 27	14	0	7
170960	ZNF721	zinc finger protein 721	14	0	7
3642	INSM1	insulinoma-associated 1	14	0	7
3631	INPP4A	inositol polyphosphate-4-phosphatase, type I, 107kDa	14	0	7
8705	B3GALT4	UDP-Gal:betaGlcNAc beta 1,3-galactosyltransferase, polype	14	0	7
5334	PLCL1	phospholipase C-like 1	14	0	7
118813	ZFYVE27	zinc finger, FYVE domain containing 27	14	0	7
116442	RAB39B	RAB39B, member RAS oncogene family	14	0	7
57460	PPM1H	protein phosphatase 1H (PP2C domain containing)	14	0	7
55681	SCYL2	SCY1-like 2 (S. cerevisiae)	14	0	7
26628	OR7E47P	olfactory receptor, family 7, subfamily E, member 47 pseudo	14	0	7
80010	RMI1	RMI1, RecQ mediated genome instability 1, homolog (S. cer	14	0	7
147837	ZNF563	zinc finger protein 563	14	0	7
80153	EDC3	enhancer of mRNA decapping 3 homolog (S. cerevisiae)	14	0	7

2953	GSTT2	glutathione S-transferase theta 2	10	3	6.5
79781	IQCA	IQ motif containing with AAA domain	10	3	6.5
92002	FAM58A	family with sequence similarity 58, member A	10	3	6.5
9987	HNRPDL	heterogeneous nuclear ribonucleoprotein D-like	10	3	6.5
5930	RBBP6	retinoblastoma binding protein 6	8	5	6.5
57599	WDR48	WD repeat domain 48	8	5	6.5
8573	CASK	calcium/calmodulin-dependent serine protein kinase (MAGU	8	5	6.5
23523	CABIN1	calcineurin binding protein 1	8	5	6.5
8321	FZD1	frizzled homolog 1 (Drosophila)	8	5	6.5
29886	SNX8	sorting nexin 8	8	5	6.5
5932	RBBP8	retinoblastoma binding protein 8	8	5	6.5
9203	ZMYM3	zinc finger, MYM-type 3	8	5	6.5
3291	HSD11B2	hydroxysteroid (11-beta) dehydrogenase 2	8	5	6.5
126393	HSPB6	heat shock protein, alpha-crystallin-related, B6	6	7	6.5
9847	KIAA0528	KIAA0528	6	7	6.5
125058	TBC1D16	TBC1 domain family, member 16	6	7	6.5
89870	TRIM15	tripartite motif-containing 15	6	7	6.5
9777	TM9SF4	transmembrane 9 superfamily protein member 4	6	7	6.5
5784	PTPN14	protein tyrosine phosphatase, non-receptor type 14	6	7	6.5
80178	C16orf59	chromosome 16 open reading frame 59	6	7	6.5
219854	LOC219854	hypothetical protein LOC219854	6	7	6.5
64855	FAM129B	family with sequence similarity 129, member B	6	7	6.5
51002	TPRKB	TP53RK binding protein	6	7	6.5
4123	MAN2C1	mannosidase, alpha, class 2C, member 1	6	7	6.5
91433	RCCD1	RCC1 domain containing 1	4	9	6.5
84561	SLC12A8	solute carrier family 12 (potassium/chloride transporters), me	4	9	6.5
201475	RAB12	RAB12, member RAS oncogene family	2	11	6.5
6620	SNCB	synuclein, beta	2	11	6.5
55031	USP47	ubiquitin specific peptidase 47	2	11	6.5
55833	UBAP2	ubiquitin associated protein 2	2	11	6.5
130271	PLEKHH2	pleckstrin homology domain containing, family H (with MyTH	0	13	6.5
79716	NPEPL1	aminopeptidase-like 1	0	13	6.5
51434	ANAPC7	anaphase promoting complex subunit 7	0	13	6.5
26517	TIMM13	translocase of inner mitochondrial membrane 13 homolog (y	0	13	6.5
92960	PEX11G	peroxisomal biogenesis factor 11 gamma	0	13	6.5
9022	CLIC3	chloride intracellular channel 3	0	13	6.5
79148	MMP28	matrix metallopeptidase 28	0	13	6.5
79018	C17orf39	chromosome 17 open reading frame 39	0	13	6.5
84893	FBXO18	F-box protein, helicase, 18	0	13	6.5
9205	ZMYM5	zinc finger, MYM-type 5	0	13	6.5
27246	ZNF364	zinc finger protein 364	0	13	6.5
7114	TMSB4X	thymosin, beta 4, X-linked	0	13	6.5
284757	LOC284757	hypothetical protein LOC284757	0	13	6.5
55172	C14orf104	chromosome 14 open reading frame 104	0	13	6.5
23245	ASTN2	astrotactin 2	0	13	6.5
57088	PLSCR4	phospholipid scramblase 4	0	13	6.5
56977	STOX2	storkhead box 2	0	13	6.5
55278	QRSL1	glutaminyl-tRNA synthase (glutamine-hydrolyzing)-like 1	0	13	6.5
93589	CACNA2D4	calcium channel, voltage-dependent, alpha 2/delta subunit 4	0	13	6.5
163183	C19orf46	chromosome 19 open reading frame 46	0	13	6.5
90523	C6orf142	chromosome 6 open reading frame 142	0	13	6.5

23376	KIAA0776	KIAA0776	0	13	6.5
23032	USP33	ubiquitin specific peptidase 33	0	13	6.5
7428	VHL	von Hippel-Lindau tumor suppressor	0	13	6.5
57446	NDRG3	NDRG family member 3	0	13	6.5
26168	SENP3	SUMO1/sentrin/SMT3 specific peptidase 3	0	13	6.5
84641	HIATL1	hippocampus abundant transcript-like 1	0	13	6.5
51094	ADIPOR1	adiponectin receptor 1	0	13	6.5
284114	TMEM102	transmembrane protein 102	0	13	6.5
10602	CDC42EP3	CDC42 effector protein (Rho GTPase binding) 3	0	13	6.5
147650	LOC147650	hypothetical protein LOC147650	0	13	6.5
10948	STARD3	START domain containing 3	0	13	6.5
4600	MX2	myxovirus (influenza virus) resistance 2 (mouse)	0	13	6.5
2741	GLRA1	glycine receptor, alpha 1 (startle disease/hyperekplexia, stiff	0	13	6.5
5243	ABCB1	ATP-binding cassette, sub-family B (MDR/TAP), member 1	0	13	6.5
5537	PPP6C	protein phosphatase 6, catalytic subunit	0	13	6.5
9772	KIAA0195	KIAA0195	0	13	6.5
30815	ST6GALNAC	ST6 (alpha-N-acetyl-neuraminyl-2,3-beta-galactosyl-1,3)-N-a	0	13	6.5
56006	FLJ12886	hypothetical protein FLJ12886	13	0	6.5
55823	VPS11	vacuolar protein sorting 11 homolog (S. cerevisiae)	13	0	6.5
23671	TMEFF2	transmembrane protein with EGF-like and two follistatin-like	13	0	6.5
84820	MGC13098	hypothetical protein MGC13098	13	0	6.5
84717	HDGF2	hepatoma-derived growth factor-related protein 2	13	0	6.5
84659	RNASE7	ribonuclease, RNase A family, 7	13	0	6.5
10527	IPO7	importin 7	13	0	6.5
79789	CLMN	calmin (calponin-like, transmembrane)	13	0	6.5
79659	DYNC2H1	dynein, cytoplasmic 2, heavy chain 1	13	0	6.5
151903	CCDC12	coiled-coil domain containing 12	13	0	6.5
90865	IL33	interleukin 33	13	0	6.5
26354	GNL3	guanine nucleotide binding protein-like 3 (nucleolar)	13	0	6.5
23130	KIAA0404	hypothetical protein LOC23130	13	0	6.5
29090	C18orf55	chromosome 18 open reading frame 55	13	0	6.5
3574	IL7	interleukin 7	13	0	6.5
7750	ZMYM2	zinc finger, MYM-type 2	13	0	6.5
5877	RABIF	RAB interacting factor	13	0	6.5
64423	C14orf173	chromosome 14 open reading frame 173	13	0	6.5
5563	PRKAA2	protein kinase, AMP-activated, alpha 2 catalytic subunit	13	0	6.5
79852	ABHD9	abhydrolase domain containing 9	13	0	6.5
6590	SLPI	secretory leukocyte peptidase inhibitor	13	0	6.5
6452	SH3BP2	SH3-domain binding protein 2	13	0	6.5
9180	OSMR	oncostatin M receptor	13	0	6.5
6018	RLF	rearranged L-myc fusion	13	0	6.5
84897	TBRG1	transforming growth factor beta regulator 1	13	0	6.5
158234	RG9MTD3	RNA (guanine-9-) methyltransferase domain containing 3	13	0	6.5
5935	RBM3	RNA binding motif (RNP1, RRM) protein 3	10	2	6
10867	TSPAN9	tetraspanin 9	10	2	6
9937	DCLRE1A	DNA cross-link repair 1A (PSO2 homolog, S. cerevisiae)	10	2	6
126382	TRA16	TR4 orphan receptor associated protein TRA16	10	2	6
359948	IRF2BP2	interferon regulatory factor 2 binding protein 2	10	2	6
523	ATP6V1A	ATPase, H+ transporting, lysosomal 70kDa, V1 subunit A	10	2	6
388341	C17orf76	chromosome 17 open reading frame 76	10	2	6
203547	LOC203547	hypothetical protein LOC203547	10	2	6

1352	COX10	COX10 homolog, cytochrome c oxidase assembly protein, h	8	4	6
84206	RKHD3	ring finger and KH domain containing 3	8	4	6
1445	CSK	c-src tyrosine kinase	6	6	6
160418	TMTC3	transmembrane and tetratricopeptide repeat containing 3	6	6	6
8607	RUVBL1	RuvB-like 1 (E. coli)	4	8	6
644165	LOC644165	similar to Breakpoint cluster region protein (NY-REN-26 antig	4	8	6
113174	SAAL1	serum amyloid A-like 1	4	8	6
92906	HNRPLL	heterogeneous nuclear ribonucleoprotein L-like	4	8	6
84747	MGC5139	hypothetical protein MGC5139	4	8	6
10893	MMP24	matrix metallopeptidase 24 (membrane-inserted)	4	8	6
147007	C17orf32	chromosome 17 open reading frame 32	4	8	6
5163	PDK1	pyruvate dehydrogenase kinase, isozyme 1	4	8	6
85409	NKD2	naked cuticle homolog 2 (Drosophila)	4	8	6
23169	SLC35D1	solute carrier family 35 (UDP-glucuronic acid/UDP-N-acetylg	2	10	6
28231	SLCO4A1	solute carrier organic anion transporter family, member 4A1	2	10	6
124	ADH1A	alcohol dehydrogenase 1A (class I), alpha polypeptide	0	12	6
8322	FZD4	frizzled homolog 4 (Drosophila)	0	12	6
51192	CKLF	chemokine-like factor	0	12	6
160287	LDHAL6A	lactate dehydrogenase A-like 6A	0	12	6
898	CCNE1	cyclin E1	0	12	6
1268	CNR1	cannabinoid receptor 1 (brain)	0	12	6
11267	SNF8	SNF8, ESCRT-II complex subunit, homolog (S. cerevisiae)	0	12	6
54785	C17orf59	chromosome 17 open reading frame 59	0	12	6
7586	ZKSCAN1	zinc finger with KRAB and SCAN domains 1	0	12	6
2861	GPR37	G protein-coupled receptor 37 (endothelin receptor type B-lik	0	12	6
2683	B4GALT1	UDP-Gal:betaGlcNAc beta 1,4- galactosyltransferase, polype	0	12	6
9611	NCOR1	nuclear receptor co-repressor 1	0	12	6
115209	OMA1	OMA1 homolog, zinc metallopeptidase (S. cerevisiae)	0	12	6
5822	PWP2	PWP2 periodic tryptophan protein homolog (yeast)	0	12	6
55571	C2orf29	chromosome 2 open reading frame 29	0	12	6
51642	MRPL48	mitochondrial ribosomal protein L48	0	12	6
4208	MEF2C	MADS box transcription enhancer factor 2, polypeptide C (m	0	12	6
1105	CHD1	chromodomain helicase DNA binding protein 1	0	12	6
114987	WDR31	WD repeat domain 31	0	12	6
1678	TIMM8A	translocase of inner mitochondrial membrane 8 homolog A (y	0	12	6
4155	MBP	myelin basic protein	0	12	6
3840	KPNA4	karyopherin alpha 4 (importin alpha 3)	0	12	6
51614	ERGIC3	ERGIC and golgi 3	0	12	6
79643	CHMP6	chromatin modifying protein 6	0	12	6
283989	TSEN54	tRNA splicing endonuclease 54 homolog (S. cerevisiae)	0	12	6
6736	SRY	sex determining region Y	0	12	6
6446	SGK	serum/glucocorticoid regulated kinase	0	12	6
9531	BAG3	BCL2-associated athanogene 3	0	12	6
6483	ST3GAL2	ST3 beta-galactoside alpha-2,3-sialyltransferase 2	0	12	6
6358	CCL14	chemokine (C-C motif) ligand 14	12	0	6
148252	DIRAS1	DIRAS family, GTP-binding RAS-like 1	12	0	6
286075	ZNF707	zinc finger protein 707	12	0	6
84249	PSD2	pleckstrin and Sec7 domain containing 2	12	0	6
9480	ONECUT2	one cut domain, family member 2	12	0	6
3664	IRF6	interferon regulatory factor 6	12	0	6
9513	FXR2	fragile X mental retardation, autosomal homolog 2	12	0	6

83461	CDCA3	cell division cycle associated 3	12	0	6
5045	FURIN	furin (paired basic amino acid cleaving enzyme)	12	0	6
23469	PHF3	PHD finger protein 3	12	0	6
23230	VPS13A	vacuolar protein sorting 13 homolog A (S. cerevisiae)	12	0	6
79152	FA2H	fatty acid 2-hydroxylase	12	0	6
6571	SLC18A2	solute carrier family 18 (vesicular monoamine), member 2	12	0	6
10947	AP3M2	adaptor-related protein complex 3, mu 2 subunit	12	0	6
819	CAMLG	calcium modulating ligand	12	0	6
3797	KIF3C	kinesin family member 3C	12	0	6
64506	CPEB1	cytoplasmic polyadenylation element binding protein 1	12	0	6
57231	SNX14	sorting nexin 14	12	0	6
4257	MGST1	microsomal glutathione S-transferase 1	12	0	6
93058	COQ10A	coenzyme Q10 homolog A (S. cerevisiae)	12	0	6
162387	FLJ35773	hypothetical protein FLJ35773	12	0	6
84935	C13orf33	chromosome 13 open reading frame 33	12	0	6
114659	LRRC37B	leucine rich repeat containing 37B	12	0	6
26059	ERC2	ELKS/RAB6-interacting/CAST family member 2	12	0	6
60681	FKBP10	FK506 binding protein 10, 65 kDa	12	0	6
6871	TADA2L	transcriptional adaptor 2 (ADA2 homolog, yeast)-like	12	0	6
51460	SFMBT1	Scm-like with four mbt domains 1	12	0	6
114793	FMNL2	formin-like 2	12	0	6
51643	TMBIM4	transmembrane BAX inhibitor motif containing 4	12	0	6
55320	C14orf106	chromosome 14 open reading frame 106	12	0	6
23081	JMJD2C	jumonji domain containing 2C	12	0	6
51263	MRPL30	mitochondrial ribosomal protein L30	12	0	6
5382	PMS2L4	postmeiotic segregation increased 2-like 4	12	0	6
80267	EDEM3	ER degradation enhancer, mannosidase alpha-like 3	12	0	6
80184	CEP290	centrosomal protein 290kDa	12	0	6
5297	PIK4CA	phosphatidylinositol 4-kinase, catalytic, alpha polypeptide	12	0	6
80018	C12orf30	chromosome 12 open reading frame 30	12	0	6
284207	METRNL	meteorin, glial cell differentiation regulator-like	12	0	6
60489	APOBEC3G	apolipoprotein B mRNA editing enzyme, catalytic polypeptide	12	0	6
401504	LOC401504	hypothetical gene supported by AK091718	12	0	6
5782	PTPN12	protein tyrosine phosphatase, non-receptor type 12	12	0	6
64343	AZI2	5-azacytidine induced 2	12	0	6
5876	RABGGTB	Rab geranylgeranyltransferase, beta subunit	12	0	6
84759	PCGF1	polycomb group ring finger 1	12	0	6
9716	AQR	aquarius homolog (mouse)	12	0	6
55297	CCDC91	coiled-coil domain containing 91	12	0	6
113230	LOC113230	hypothetical protein LOC113230	12	0	6
1070	CETN3	centrin, EF-hand protein, 3 (CDC31 homolog, yeast)	12	0	6
57178	ZMIZ1	zinc finger, MIZ-type containing 1	12	0	6
245937	DEFB124	defensin, beta 124	12	0	6
93973	ACTR8	ARP8 actin-related protein 8 homolog (yeast)	12	0	6
153562	MARVELD2	MARVEL domain containing 2	12	0	6
27242	TNFRSF21	tumor necrosis factor receptor superfamily, member 21	12	0	6
84996	C21orf119	chromosome 21 open reading frame 119	12	0	6
57465	TBC1D24	TBC1 domain family, member 24	12	0	6
10084	PQBP1	polyglutamine binding protein 1	12	0	6
9871	SEC24D	SEC24 related gene family, member D (S. cerevisiae)	12	0	6
56983	C3orf9	chromosome 3 open reading frame 9	12	0	6

85021	REPS1	RALBP1 associated Eps domain containing 1	12	0	6
177	AGER	advanced glycosylation end product-specific receptor	12	0	6
123887	ZG16	zymogen granule protein 16	12	0	6
127253	TYW3	tRNA-yW synthesizing protein 3 homolog (S. cerevisiae)	12	0	6
9014	TAF1B	TATA box binding protein (TBP)-associated factor, RNA poly	12	0	6
11216	AKAP10	A kinase (PRKA) anchor protein 10	12	0	6
27327	TNRC6A	trinucleotide repeat containing 6A	12	0	6
5048	PAFAH1B1	platelet-activating factor acetylhydrolase, isoform Ib, alpha su	12	0	6
10402	ST3GAL6	ST3 beta-galactoside alpha-2,3-sialyltransferase 6	12	0	6
64921	CASD1	CAS1 domain containing 1	12	0	6
55607	PPP1R9A	protein phosphatase 1, regulatory (inhibitor) subunit 9A	12	0	6
220929	ZNF438	zinc finger protein 438	12	0	6
151987	PPP4R2	protein phosphatase 4, regulatory subunit 2	12	0	6
25924	MYRIP	myosin VIIA and Rab interacting protein	12	0	6
55924	C1orf183	chromosome 1 open reading frame 183	12	0	6
221079	ARL5B	ADP-ribosylation factor-like 5B	12	0	6
159195	USP54	ubiquitin specific peptidase 54	12	0	6
414918	FAM116B	family with sequence similarity 116, member B	12	0	6
83942	TSSK1	testis-specific serine kinase 1	12	0	6
29013	PRO1596	PRO1596 protein	12	0	6
388611	CD164L2	CD164 sialomucin-like 2	12	0	6
28951	TRIB2	tribbles homolog 2 (Drosophila)	12	0	6
1515	CTSL2	cathepsin L2	12	0	6
63917	GALNT11	UDP-N-acetyl-alpha-D-galactosamine:polypeptide N-acetylg	12	0	6
29098	RANGNRF	RAN guanine nucleotide release factor	12	0	6
9922	IQSEC1	IQ motif and Sec7 domain 1	12	0	6
55630	SLC39A4	solute carrier family 39 (zinc transporter), member 4	12	0	6
8795	TNFRSF10B	tumor necrosis factor receptor superfamily, member 10b	12	0	6
441204	LOC441204	hypothetical locus LOC441204	12	0	6
23150	FRMD4B	FERM domain containing 4B	12	0	6
11257	TP53AP1	TP53 activated protein 1	12	0	6
51062	SPG3A	spastic paraplegia 3A (autosomal dominant)	12	0	6
164284	APCDD1L	adenomatosis polyposis coli down-regulated 1-like	12	0	6
26276	VPS33B	vacuolar protein sorting 33 homolog B (yeast)	12	0	6
1740	DLG2	discs, large homolog 2, chapsyn-110 (Drosophila)	8	3	5.5
55917	CTTNBP2NL	CTTNBP2 N-terminal like	8	3	5.5
55291	SAPS3	SAPS domain family, member 3	8	3	5.5
10499	NCOA2	nuclear receptor coactivator 2	8	3	5.5
9215	LARGE	like-glycosyltransferase	8	3	5.5
81789	TIGD6	tigger transposable element derived 6	6	5	5.5
65072	ALS2CR10	amyotrophic lateral sclerosis 2 (juvenile) chromosome regior	6	5	5.5
80705	TSGA10	testis specific, 10	6	5	5.5
219293	ATAD3C	ATPase family, AAA domain containing 3C	6	5	5.5
128869	PIGU	phosphatidylinositol glycan anchor biosynthesis, class U	6	5	5.5
5429	POLH	polymerase (DNA directed), eta	6	5	5.5
8848	TSC22D1	TSC22 domain family, member 1	6	5	5.5
25840	METTL7A	methyltransferase like 7A	6	5	5.5
57834	CYP4F11	cytochrome P450, family 4, subfamily F, polypeptide 11	6	5	5.5
57333	RCN3	reticulocalbin 3, EF-hand calcium binding domain	6	5	5.5
57695	USP37	ubiquitin specific peptidase 37	6	5	5.5
647074	LOC647074	similar to ribosomal protein L10	6	5	5.5

3953	LEPR	leptin receptor	4	7	5.5
5921	RASA1	RAS p21 protein activator (GTPase activating protein) 1	4	7	5.5
57620	STIM2	stromal interaction molecule 2	4	7	5.5
10966	RAB40B	RAB40B, member RAS oncogene family	2	9	5.5
81849	ST6GALNAC	ST6 (alpha-N-acetyl-neuraminyl-2,3-beta-galactosyl-1,3)-N-a	2	9	5.5
114932	MRFAP1L1	Morf4 family associated protein 1-like 1	2	9	5.5
54516	MTRF1L	mitochondrial translational release factor 1-like	2	9	5.5
90624	LYRM7	Lyrm7 homolog (mouse)	2	9	5.5
9363	RAB33A	RAB33A, member RAS oncogene family	0	11	5.5
203197	C9orf91	chromosome 9 open reading frame 91	0	11	5.5
144110	TMEM86A	transmembrane protein 86A	0	11	5.5
157567	ANKRD46	ankyrin repeat domain 46	0	11	5.5
8899	PRPF4B	PRP4 pre-mRNA processing factor 4 homolog B (yeast)	0	11	5.5
6810	STX4	syntaxin 4	0	11	5.5
79829	NAT11	N-acetyltransferase 11	0	11	5.5
2887	GRB10	growth factor receptor-bound protein 10	0	11	5.5
57562	KIAA1377	KIAA1377	0	11	5.5
9040	UBE2M	ubiquitin-conjugating enzyme E2M (UBC12 homolog, yeast)	0	11	5.5
5119	PCOLN3	procollagen (type III) N-endopeptidase	0	11	5.5
116254	C6orf72	chromosome 6 open reading frame 72	0	11	5.5
6249	RSN	restin (Reed-Steinberg cell-expressed intermediate filament-	0	11	5.5
113451	ADC	arginine decarboxylase	0	11	5.5
80232	WDR26	WD repeat domain 26	0	11	5.5
9669	EIF5B	eukaryotic translation initiation factor 5B	0	11	5.5
140545	RNF32	ring finger protein 32	0	11	5.5
90417	C15orf23	chromosome 15 open reading frame 23	0	11	5.5
26235	FBXL4	F-box and leucine-rich repeat protein 4	0	11	5.5
123207	C15orf40	chromosome 15 open reading frame 40	0	11	5.5
57147	SCYL3	SCY1-like 3 (S. cerevisiae)	0	11	5.5
8487	SIP1	survival of motor neuron protein interacting protein 1	0	11	5.5
23348	DOCK9	dedicator of cytokinesis 9	0	11	5.5
147080	LOC147080	hypothetical protein LOC147080	0	11	5.5
23395	LARS2	leucyl-tRNA synthetase 2, mitochondrial	0	11	5.5
54834	GDAP2	ganglioside induced differentiation associated protein 2	0	11	5.5
55920	RCC2	regulator of chromosome condensation 2	0	11	5.5
5498	PPOX	protoporphyrinogen oxidase	0	11	5.5
119559	SFXN4	sideroflexin 4	0	11	5.5
79915	C17orf41	chromosome 17 open reading frame 41	0	11	5.5
943	TNFRSF8	tumor necrosis factor receptor superfamily, member 8	0	11	5.5
129138	ANKRD54	ankyrin repeat domain 54	0	11	5.5
94120	SYTL3	synaptotagmin-like 3	0	11	5.5
252983	STXBP4	syntaxin binding protein 4	0	11	5.5
79624	C6orf211	chromosome 6 open reading frame 211	0	11	5.5
6895	TARBP2	Tar (HIV-1) RNA binding protein 2	0	11	5.5
57678	GPAM	glycerol-3-phosphate acyltransferase, mitochondrial	0	11	5.5
1606	DGKA	diacylglycerol kinase, alpha 80kDa	0	11	5.5
11093	ADAMTS13	ADAM metallopeptidase with thrombospondin type 1 motif, 1	0	11	5.5
10670	RRAGA	Ras-related GTP binding A	0	11	5.5
163882	C1orf71	chromosome 1 open reading frame 71	0	11	5.5
646080	LOC646080	similar to Rab coupling protein isoform 1	0	11	5.5
8643	PTCH2	patched homolog 2 (Drosophila)	0	11	5.5

54994	C20orf11	chromosome 20 open reading frame 11	0	11	5.5
2957	GTF2A1	general transcription factor IIA, 1, 19/37kDa	0	11	5.5
3911	LAMA5	laminin, alpha 5	0	11	5.5
57129	MRPL47	mitochondrial ribosomal protein L47	0	11	5.5
164832	LONRF2	LON peptidase N-terminal domain and ring finger 2	0	11	5.5
10045	SH2D3A	SH2 domain containing 3A	0	11	5.5
4012	LNPEP	leucyl/cystinyl aminopeptidase	0	11	5.5
54795	TRPM4	transient receptor potential cation channel, subfamily M, mer	0	11	5.5
3633	INPP5B	inositol polyphosphate-5-phosphatase, 75kDa	0	11	5.5
7092	TLL1	tolloid-like 1	0	11	5.5
285755	PPIL6	peptidylprolyl isomerase (cyclophilin)-like 6	0	11	5.5
28985	MCTS1	malignant T cell amplified sequence 1	0	11	5.5
645688	LOC645688	similar to 60S ribosomal protein L12	0	11	5.5
3053	SERPIND1	serpin peptidase inhibitor, clade D (heparin cofactor), membe	0	11	5.5
26146	TRAF3IP1	TNF receptor-associated factor 3 interacting protein 1	0	11	5.5
5347	PLK1	polo-like kinase 1 (Drosophila)	0	11	5.5
27297	RCP9	calcitonin gene-related peptide-receptor component protein	0	11	5.5
4174	MCM5	MCM5 minichromosome maintenance deficient 5, cell divisio	0	11	5.5
11059	WWP1	WW domain containing E3 ubiquitin protein ligase 1	0	11	5.5
3101	HK3	hexokinase 3 (white cell)	0	11	5.5
1742	DLG4	discs, large homolog 4 (Drosophila)	0	11	5.5
11174	ADAMTS6	ADAM metallopeptidase with thrombospondin type 1 motif, 6	8	2	5
136051	ZNF786	zinc finger protein 786	8	2	5
120534	C11orf46	chromosome 11 open reading frame 46	8	2	5
29985	SLC39A3	solute carrier family 39 (zinc transporter), member 3	8	2	5
83694	RPS6KL1	ribosomal protein S6 kinase-like 1	8	2	5
9771	RAPGEF5	Rap guanine nucleotide exchange factor (GEF) 5	8	2	5
652968	LOC652968	hypothetical protein LOC652968	8	2	5
5099	PCDH7	BH-protocadherin (brain-heart)	8	2	5
84545	MRPL43	mitochondrial ribosomal protein L43	8	2	5
63826	SRR	serine racemase	8	2	5
1353	COX11	COX11 homolog, cytochrome c oxidase assembly protein (y	8	2	5
6387	CXCL12	chemokine (C-X-C motif) ligand 12 (stromal cell-derived fact	6	4	5
1454	CSNK1E	casein kinase 1, epsilon	4	6	5
22796	COG2	component of oligomeric golgi complex 2	4	6	5
677820	SNORA38	small nucleolar RNA, H/ACA box 38	4	6	5
54165	DCUN1D1	DCN1, defective in cullin neddylation 1, domain containing 1	2	8	5
10282	BET1	BET1 homolog (S. cerevisiae)	2	8	5
3632	INPP5A	inositol polyphosphate-5-phosphatase, 40kDa	2	8	5
143570	XRRA1	X-ray radiation resistance associated 1	2	8	5
6585	SLIT1	slit homolog 1 (Drosophila)	2	8	5
65109	UPF3B	UPF3 regulator of nonsense transcripts homolog B (yeast)	2	8	5
167691	C6orf152	chromosome 6 open reading frame 152	2	8	5
55653	BCAS4	breast carcinoma amplified sequence 4	2	8	5
55334	SLC39A9	solute carrier family 39 (zinc transporter), member 9	2	8	5
145957	NRG4	neuregulin 4	2	8	5
90488	C12orf23	chromosome 12 open reading frame 23	2	8	5
26135	SERBP1	SERPINE1 mRNA binding protein 1	2	8	5
57669	EPB41L5	erythrocyte membrane protein band 4.1 like 5	2	8	5
10767	HBS1L	HBS1-like (S. cerevisiae)	2	8	5
79041	TMEM38A	transmembrane protein 38A	0	10	5

23563	CHST5	carbohydrate (N-acetylglucosamine 6-O) sulfotransferase 5	0	10	5
341880	SLC35F4	solute carrier family 35, member F4	0	10	5
6760	SS18	synovial sarcoma translocation, chromosome 18	0	10	5
2013	EMP2	epithelial membrane protein 2	0	10	5
7410	VAV2	vav 2 oncogene	0	10	5
5990	RFX2	regulatory factor X, 2 (influences HLA class II expression)	0	10	5
146857	SLFN13	schlafen family member 13	0	10	5
57282	SLC4A10	solute carrier family 4, sodium bicarbonate transporter-like, r	0	10	5
2737	GLI3	GLI-Kruppel family member GLI3 (Greig cephalopolysyndact	0	10	5
343	AQP8	aquaporin 8	0	10	5
57552	AADACL1	arylacetamide deacetylase-like 1	0	10	5
145741	NLF1	nuclear localized factor 1	0	10	5
8455	ATRN	attractin	0	10	5
285927	LOC285927	hypothetical protein LOC285927	0	10	5
84230	LRRC8C	leucine rich repeat containing 8 family, member C	0	10	5
730057	LOC730057	hypothetical protein LOC730057	0	10	5
8672	EIF4G3	eukaryotic translation initiation factor 4 gamma, 3	0	10	5
7172	TPMT	thiopurine S-methyltransferase	0	10	5
120224	TMEM45B	transmembrane protein 45B	0	10	5
84823	LMNB2	lamin B2	0	10	5
10210	TOPORS	topoisomerase I binding, arginine/serine-rich	0	10	5
10512	SEMA3C	sema domain, immunoglobulin domain (Ig), short basic doma	0	10	5
9467	SH3BP5	SH3-domain binding protein 5 (BTK-associated)	0	10	5
84057	MND1	meiotic nuclear divisions 1 homolog (S. cerevisiae)	0	10	5
161835	FSIP1	fibrous sheath interacting protein 1	0	10	5
5934	RBL2	retinoblastoma-like 2 (p130)	0	10	5
51691	LSM8	LSM8 homolog, U6 small nuclear RNA associated (S. cerevi	0	10	5
10664	CTCF	CCCTC-binding factor (zinc finger protein)	0	10	5
6503	SLA	Src-like-adaptor	0	10	5
54073	C21orf41	chromosome 21 open reading frame 41	0	10	5
900	CCNG1	cyclin G1	0	10	5
9802	DAZAP2	DAZ associated protein 2	0	10	5
400945	FLJ41481	hypothetical gene supported by AK123475	0	10	5
10522	DEAF1	deformed epidermal autoregulatory factor 1 (Drosophila)	0	10	5
3845	KRAS	v-Ki-ras2 Kirsten rat sarcoma viral oncogene homolog	0	10	5
27085	MTBP	Mdm2, transformed 3T3 cell double minute 2, p53 binding pr	0	10	5
3991	LIPE	lipase, hormone-sensitive	0	10	5
8382	NME5	non-metastatic cells 5, protein expressed in (nucleoside-diph	0	10	5
51012	C20orf45	chromosome 20 open reading frame 45	0	10	5
3290	HSD11B1	hydroxysteroid (11-beta) dehydrogenase 1	0	10	5
51066	C3orf32	chromosome 3 open reading frame 32	0	10	5
57633	LRRN1	leucine rich repeat neuronal 1	0	10	5
114769	COP1	caspase-1 dominant-negative inhibitor pseudo-ICE	0	10	5
84924	ZNF566	zinc finger protein 566	0	10	5
2318	FLNC	filamin C, gamma (actin binding protein 280)	0	10	5
326624	RAB37	RAB37, member RAS oncogene family	0	10	5
63877	C10orf84	chromosome 10 open reading frame 84	0	10	5
1956	EGFR	epidermal growth factor receptor (erythroblastic leukemia vira	0	10	5
64421	DCLRE1C	DNA cross-link repair 1C (PSO2 homolog, S. cerevisiae)	0	10	5
865	CBFB	core-binding factor, beta subunit	0	10	5
29954	POMT2	protein-O-mannosyltransferase 2	0	10	5

89231	DPY19L1P1	dpy-19-like 1 pseudogene 1 (C. elegans)	0	10	5
63976	PRDM16	PR domain containing 16	0	10	5
23360	FNBP4	formin binding protein 4	0	10	5
51780	JMJD1B	jumonji domain containing 1B	10	0	5
80224	NUBPL	nucleotide binding protein-like	10	0	5
11269	DDX19B	DEAD (Asp-Glu-Ala-As) box polypeptide 19B	10	0	5
4988	OPRM1	opioid receptor, mu 1	10	0	5
401253	FLJ43752	FLJ43752 protein	10	0	5
84890	C10orf22	chromosome 10 open reading frame 22	10	0	5
23327	NEDD4L	neural precursor cell expressed, developmentally down-regu	10	0	5
23596	OPN3	opsin 3 (encephalopsin, panopsin)	10	0	5
9209	LRRFIP2	leucine rich repeat (in FLII) interacting protein 2	10	0	5
79758	DHRS12	dehydrogenase/reductase (SDR family) member 12	10	0	5
9556	C14orf2	chromosome 14 open reading frame 2	10	0	5
55350	VNN3	vanin 3	10	0	5
80095	ZNF606	zinc finger protein 606	10	0	5
645106	LOC645106	similar to kinesin-like motor protein C20orf23	10	0	5
138050	HGSNAT	heparan-alpha-glucosaminide N-acetyltransferase	10	0	5
283701	LOC283701	hypothetical protein LOC283701	10	0	5
672	BRCA1	breast cancer 1, early onset	10	0	5
57186	C20orf74	chromosome 20 open reading frame 74	10	0	5
114795	TMEM132B	transmembrane protein 132B	10	0	5
6623	SNCG	synuclein, gamma (breast cancer-specific protein 1)	10	0	5
11170	FAM107A	family with sequence similarity 107, member A	10	0	5
541468	C1orf190	chromosome 1 open reading frame 190	10	0	5
11103	KRR1	KRR1, small subunit (SSU) processome component, homolo	10	0	5
199221	DZIP1L	DAZ interacting protein 1-like	10	0	5
5469	PPARBP	PPAR binding protein	10	0	5
79905	TMC7	transmembrane channel-like 7	10	0	5
6654	SOS1	son of sevenless homolog 1 (Drosophila)	10	0	5
11116	FGFR1OP	FGFR1 oncogene partner	10	0	5
346329	LOC346329	similar to Guanine nucleotide-binding protein alpha-11 subur	10	0	5
93663	ARHGAP18	Rho GTPase activating protein 18	10	0	5
57461	KIAA1160	KIAA1160 protein	10	0	5
254528	C16orf73	chromosome 16 open reading frame 73	10	0	5
51125	GOLGA7	golgi autoantigen, golgin subfamily a, 7	10	0	5
1796	DOK1	docking protein 1, 62kDa (downstream of tyrosine kinase 1)	10	0	5
2005	ELK4	ELK4, ETS-domain protein (SRF accessory protein 1)	10	0	5
113277	TMEM106A	transmembrane protein 106A	10	0	5
60468	BACH2	BTB and CNC homology 1, basic leucine zipper transcriptior	10	0	5
8242	JARID1C	jumonji, AT rich interactive domain 1C	10	0	5
23516	SLC39A14	solute carrier family 39 (zinc transporter), member 14	10	0	5
91607	SLFN11	schlafen family member 11	10	0	5
8498	RANBP3	RAN binding protein 3	10	0	5
2784	GNB3	guanine nucleotide binding protein (G protein), beta polypep	10	0	5
9891	NUAK1	NUAK family, SNF1-like kinase, 1	10	0	5
790898	HYDIN2	hydrocephalus inducing homolog 2 (mouse)	10	0	5
339344	LOC339344	hypothetical protein LOC339344	10	0	5
2049	EPHB3	EPH receptor B3	10	0	5
284358	FLJ36070	likely ortholog of MEF2-activating SAP transcriptional regula	10	0	5
1789	DNMT3B	DNA (cytosine-5-)-methyltransferase 3 beta	10	0	5

10775	POP4	processing of precursor 4, ribonuclease P/MRP subunit (S. c	10	0	5
10308	ZNF267	zinc finger protein 267	10	0	5
11224	RPL35	ribosomal protein L35	10	0	5
23015	GOLGA8A	golgi autoantigen, golgin subfamily a, 8A	10	0	5
55665	URG4	up-regulated gene 4	10	0	5
9069	CLDN12	claudin 12	10	0	5
1022	CDK7	cyclin-dependent kinase 7 (MO15 homolog, Xenopus laevis,	10	0	5
677806	SNORA20	small nucleolar RNA, H/ACA box 20	10	0	5
57613	KIAA1467	KIAA1467	10	0	5
11130	ZWINT	ZW10 interactor	10	0	5
114907	FBXO32	F-box protein 32	10	0	5
24144	TFIP11	tuftelin interacting protein 11	10	0	5
10324	KBTBD10	kelch repeat and BTB (POZ) domain containing 10	10	0	5
283682	LOC283682	hypothetical protein LOC283682	10	0	5
26112	CCDC69	coiled-coil domain containing 69	10	0	5
5547	PRCP	prolylcarboxypeptidase (angiotensinase C)	10	0	5
54878	DPP8	dipeptidyl-peptidase 8	10	0	5
197407	ZNF553	zinc finger protein 553	10	0	5
79839	CCDC102B	coiled-coil domain containing 102B	10	0	5
26060	APPL	adaptor protein containing pH domain, PTB domain and leuc	10	0	5
7026	NR2F2	nuclear receptor subfamily 2, group F, member 2	10	0	5
79663	HSPBAP1	HSPB (heat shock 27kDa) associated protein 1	10	0	5
56963	RGMA	RGM domain family, member A	10	0	5
7913	DEK	DEK oncogene (DNA binding)	10	0	5
54566	EPB41L4B	erythrocyte membrane protein band 4.1 like 4B	10	0	5
26034	PIP3-E	phosphoinositide-binding protein PIP3-E	10	0	5
8717	TRADD	TNFRSF1A-associated via death domain	10	0	5
11137	PWP1	PWP1 homolog (S. cerevisiae)	10	0	5
5868	RAB5A	RAB5A, member RAS oncogene family	10	0	5
84612	PARD6B	par-6 partitioning defective 6 homolog beta (C. elegans)	10	0	5
713	C1QB	complement component 1, q subcomponent, B chain	10	0	5
27429	HTRA2	HtrA serine peptidase 2	10	0	5
54538	ROBO4	roundabout homolog 4, magic roundabout (Drosophila)	10	0	5
7115	TMSL1	thymosin-like 1	10	0	5
80052	FLJ12331	hypothetical protein FLJ12331	10	0	5
374354	NHLRC2	NHL repeat containing 2	10	0	5
6487	ST3GAL3	ST3 beta-galactoside alpha-2,3-sialyltransferase 3	10	0	5
81605	URM1	ubiquitin related modifier 1 homolog (S. cerevisiae)	10	0	5
7298	TYMS	thymidylate synthetase	10	0	5
7067	THRA	thyroid hormone receptor, alpha (erythroblastic leukemia vira	10	0	5
1280	COL2A1	collagen, type II, alpha 1 (primary osteoarthritis, spondyloepi	10	0	5
84138	SLC7A6OS	solute carrier family 7, member 6 opposite strand	10	0	5
133015	C4orf28	chromosome 4 open reading frame 28	10	0	5
8821	INPP4B	inositol polyphosphate-4-phosphatase, type II, 105kDa	10	0	5
55769	ZNF83	zinc finger protein 83	10	0	5
57605	PITPNM2	phosphatidylinositol transfer protein, membrane-associated 2	10	0	5
29841	GRHL1	grainyhead-like 1 (Drosophila)	10	0	5
79036	C19orf50	chromosome 19 open reading frame 50	10	0	5
64773	FAM113A	family with sequence similarity 113, member A	10	0	5
84706	GPT2	glutamic pyruvate transaminase (alanine aminotransferase) 2	6	3	4.5
25771	TBC1D22A	TBC1 domain family, member 22A	6	3	4.5

81853	TMEM14B	transmembrane protein 14B	6	3	4.5
58477	SRPRB	signal recognition particle receptor, B subunit	6	3	4.5
3175	ONECUT1	one cut domain, family member 1	6	3	4.5
8975	USP13	ubiquitin specific peptidase 13 (isopeptidase T-3)	6	3	4.5
6130	RPL7A	ribosomal protein L7a	6	3	4.5
55347	ABHD10	abhydrolase domain containing 10	6	3	4.5
5605	MAP2K2	mitogen-activated protein kinase kinase 2	6	3	4.5
25941	C18orf10	chromosome 18 open reading frame 10	6	3	4.5
5142	PDE4B	phosphodiesterase 4B, cAMP-specific (phosphodiesterase E	6	3	4.5
23473	CAPN7	calpain 7	6	3	4.5
4201	MEA1	male-enhanced antigen 1	6	3	4.5
2305	FOXM1	forkhead box M1	6	3	4.5
54876	C4orf30	chromosome 4 open reading frame 30	6	3	4.5
55744	C7orf44	chromosome 7 open reading frame 44	6	3	4.5
149013	KIAA1245	KIAA1245	6	3	4.5
51741	WWOX	WW domain containing oxidoreductase	6	3	4.5
29855	UBN1	ubinuclein 1	6	3	4.5
64714	PDIA2	protein disulfide isomerase family A, member 2	6	3	4.5
11011	TLK2	tousled-like kinase 2	4	5	4.5
1605	DAG1	dystroglycan 1 (dystrophin-associated glycoprotein 1)	4	5	4.5
5728	PTEN	phosphatase and tensin homolog (mutated in multiple advan	4	5	4.5
65008	MRPL1	mitochondrial ribosomal protein L1	4	5	4.5
55014	STX17	syntaxin 17	4	5	4.5
130013	ACMSD	aminocarboxymuconate semialdehyde decarboxylase	4	5	4.5
92070	MGC21675	hypothetical protein MGC21675	4	5	4.5
7353	UFD1L	ubiquitin fusion degradation 1 like (yeast)	4	5	4.5
9690	UBE3C	ubiquitin protein ligase E3C	4	5	4.5
51128	SAR1B	SAR1 gene homolog B (S. cerevisiae)	4	5	4.5
6788	STK3	serine/threonine kinase 3 (STE20 homolog, yeast)	4	5	4.5
85476	GFM1	G elongation factor, mitochondrial 1	4	5	4.5
132160	PPM1M	protein phosphatase 1M (PP2C domain containing)	4	5	4.5
717	C2	complement component 2	2	7	4.5
1059	CENPB	centromere protein B, 80kDa	2	7	4.5
29789	GTPBP9	GTP-binding protein 9 (putative)	2	7	4.5
64946	CENPH	centromere protein H	2	7	4.5
10529	NEBL	nebulette	2	7	4.5
79980	DSN1	DSN1, MIND kinetochore complex component, homolog (S.	2	7	4.5
11152	WDR45	WD repeat domain 45	0	9	4.5
4649	MYO9A	myosin IXA	0	9	4.5
1510	CTSE	cathepsin E	0	9	4.5
114790	STK11IP	serine/threonine kinase 11 interacting protein	0	9	4.5
9131	AIFM1	apoptosis-inducing factor, mitochondrion-associated, 1	0	9	4.5
9253	NUMBL	numb homolog (Drosophila)-like	0	9	4.5
10085	EDIL3	EGF-like repeats and discoidin I-like domains 3	0	9	4.5
23347	SMCHD1	structural maintenance of chromosomes flexible hinge doma	0	9	4.5
51268	PIPOX	pipecolic acid oxidase	0	9	4.5
64375	IKZF4	IKAROS family zinc finger 4 (Eos)	0	9	4.5
10673	TNFSF13B	tumor necrosis factor (ligand) superfamily, member 13b	0	9	4.5
84221	C21orf56	chromosome 21 open reading frame 56	0	9	4.5
54964	C1orf56	chromosome 1 open reading frame 56	0	9	4.5
56254	RNF20	ring finger protein 20	0	9	4.5

56606	SLC2A9	solute carrier family 2 (facilitated glucose transporter), memb	0	9	4.5
60370	AVPI1	arginine vasopressin-induced 1	0	9	4.5
5507	PPP1R3C	protein phosphatase 1, regulatory (inhibitor) subunit 3C	0	9	4.5
51762	RAB8B	RAB8B, member RAS oncogene family	0	9	4.5
8467	SMARCA5	SWI/SNF related, matrix associated, actin dependent regula	0	9	4.5
230	ALDOC	aldolase C, fructose-bisphosphate	0	9	4.5
54629	FAM63B	family with sequence similarity 63, member B	0	9	4.5
23304	UBR2	ubiquitin protein ligase E3 component n-recognin 2	0	9	4.5
10561	IFI44	interferon-induced protein 44	0	9	4.5
7678	ZNF124	zinc finger protein 124	0	9	4.5
868	CBLB	Cas-Br-M (murine) ecotropic retroviral transforming sequenc	0	9	4.5
9208	LRRFIP1	leucine rich repeat (in FLII) interacting protein 1	0	9	4.5
8396	PIP5K2B	phosphatidylinositol-4-phosphate 5-kinase, type II, beta	0	9	4.5
645818	NA	NA	0	9	4.5
10053	AP1M2	adaptor-related protein complex 1, mu 2 subunit	0	9	4.5
284948	SH2D6	SH2 domain containing 6	0	9	4.5
10884	MRPS30	mitochondrial ribosomal protein S30	0	9	4.5
79640	CTA-216E10	hypothetical protein FLJ23584	0	9	4.5
283065	C10orf41	chromosome 10 open reading frame 41	0	9	4.5
7994	MYST3	MYST histone acetyltransferase (monocytic leukemia) 3	0	9	4.5
22918	CD93	CD93 molecule	0	9	4.5
10907	TXNL4A	thioredoxin-like 4A	0	9	4.5
55108	BSDC1	BSD domain containing 1	0	9	4.5
27291	C10orf28	chromosome 10 open reading frame 28	0	9	4.5
51363	GALNAC4S-	B cell RAG associated protein	0	9	4.5
83986	ITFG3	integrin alpha FG-GAP repeat containing 3	0	9	4.5
11080	DNAJB4	DnaJ (Hsp40) homolog, subfamily B, member 4	0	9	4.5
115701	ALPK2	alpha-kinase 2	0	9	4.5
2239	GPC4	glypican 4	0	9	4.5
54063	C21orf53	chromosome 21 open reading frame 53	0	9	4.5
222171	PRR15	proline rich 15	0	9	4.5
4322	MMP13	matrix metallopeptidase 13 (collagenase 3)	0	9	4.5
56994	CHPT1	choline phosphotransferase 1	0	9	4.5
222546	RFXDC1	regulatory factor X domain containing 1	0	9	4.5
79899	FLJ14213	hypothetical protein FLJ14213	0	9	4.5
84457	PHYHIPL	phytanoyl-CoA 2-hydroxylase interacting protein-like	0	9	4.5
5332	PLCB4	phospholipase C, beta 4	0	9	4.5
400746	C1orf130	chromosome 1 open reading frame 130	6	2	4
652720	LOC652720	similar to synaptotagmin XV isoform a	6	2	4
90233	ZNF551	zinc finger protein 551	6	2	4
84540	KIAA1843	KIAA1843 protein	6	2	4
6144	RPL21	ribosomal protein L21	6	2	4
441167	LOC441167	hypothetical LOC441167	6	2	4
57538	ALPK3	alpha-kinase 3	6	2	4
9986	RCE1	RCE1 homolog, prenyl protein peptidase (S. cerevisiae)	6	2	4
158358	KIAA2026	KIAA2026	6	2	4
79674	VEPH1	ventricular zone expressed PH domain homolog 1 (zebrafish	6	2	4
4000	LMNA	lamin A/C	6	2	4
54069	C21orf45	chromosome 21 open reading frame 45	6	2	4
283254	C11orf77	chromosome 11 open reading frame 77	6	2	4
220382	MGC33846	hypothetical protein MGC33846	6	2	4

284406	ZNF545	zinc finger protein 545	6	2	4
9472	AKAP6	A kinase (PRKA) anchor protein 6	6	2	4
388730	TMEM81	transmembrane protein 81	6	2	4
84461	KIAA1787	KIAA1787 protein	4	4	4
51090	PLLP	plasma membrane proteolipid (plasmolipin)	4	4	4
9737	GPRASP1	G protein-coupled receptor associated sorting protein 1	2	6	4
55339	WDR33	WD repeat domain 33	2	6	4
10439	OLFM1	olfactomedin 1	2	6	4
84939	MUM1	melanoma associated antigen (mutated) 1	0	8	4
4261	CIITA	class II, major histocompatibility complex, transactivator	0	8	4
159090	FAM122B	family with sequence similarity 122B	0	8	4
56969	RPL23AP13	ribosomal protein L23a pseudogene 13	0	8	4
10106	CTDSP2	CTD (carboxy-terminal domain, RNA polymerase II, polypep	0	8	4
23604	DAPK2	death-associated protein kinase 2	0	8	4
9097	USP14	ubiquitin specific peptidase 14 (tRNA-guanine transglycosyla	0	8	4
11052	CPSF6	cleavage and polyadenylation specific factor 6, 68kDa	0	8	4
3216	HOXB6	homeobox B6	0	8	4
51379	CRLF3	cytokine receptor-like factor 3	0	8	4
10381	TUBB3	tubulin, beta 3	0	8	4
54438	GFOD1	glucose-fructose oxidoreductase domain containing 1	0	8	4
30011	SH3KBP1	SH3-domain kinase binding protein 1	0	8	4
84954	FLJ14981	hypothetical protein FLJ14981	0	8	4
133308	LOC133308	hypothetical protein BC009732	0	8	4
1230	CCR1	chemokine (C-C motif) receptor 1	0	8	4
25946	ZNF385	zinc finger protein 385	0	8	4
11157	LSM6	LSM6 homolog, U6 small nuclear RNA associated (S. cerevi	0	8	4
9788	MTSS1	metastasis suppressor 1	0	8	4
114784	CSMD2	CUB and Sushi multiple domains 2	0	8	4
152992	C4orf23	chromosome 4 open reading frame 23	0	8	4
1775	DNASE1L2	deoxyribonuclease I-like 2	0	8	4
283070	LOC283070	hypothetical protein LOC283070	0	8	4
219464	OR5T2	olfactory receptor, family 5, subfamily T, member 2	0	8	4
83786	FKSG44	FKSG44 gene	0	8	4
64167	LRAP	leukocyte-derived arginine aminopeptidase	0	8	4
139728	PNCK	pregnancy upregulated non-ubiquitously expressed CaM kin	0	8	4
10369	CACNG2	calcium channel, voltage-dependent, gamma subunit 2	0	8	4
81562	LMAN2L	lectin, mannose-binding 2-like	0	8	4
5824	PEX19	peroxisomal biogenesis factor 19	0	8	4
114815	SORCS1	sortilin-related VPS10 domain containing receptor 1	0	8	4
58510	PRODH2	proline dehydrogenase (oxidase) 2	0	8	4
3980	LIG3	ligase III, DNA, ATP-dependent	0	8	4
84928	FLJ14803	hypothetical protein FLJ14803	0	8	4
3038	HAS3	hyaluronan synthase 3	0	8	4
388662	SLC6A17	solute carrier family 6, member 17	0	8	4
80221	FLJ20920	hypothetical protein FLJ20920	0	8	4
54576	UGT1A8	UDP glucuronosyltransferase 1 family, polypeptide A8	0	8	4
129787	TMEM18	transmembrane protein 18	0	8	4
57636	ARHGAP23	Rho GTPase activating protein 23	0	8	4
6238	RRBP1	ribosome binding protein 1 homolog 180kDa (dog)	0	8	4
64105	CENPK	centromere protein K	0	8	4
49854	ZNF295	zinc finger protein 295	0	8	4

23114	NFASC	neurofascin homolog (chicken)	0	8	4
54977	SLC25A38	solute carrier family 25, member 38	0	8	4
4763	NF1	neurofibromin 1 (neurofibromatosis, von Recklinghausen dis	0	8	4
64409	WBSCR17	Williams-Beuren syndrome chromosome region 17	0	8	4
1124	CHN2	chimerin (chimaerin) 2	0	8	4
26053	AUTS2	autism susceptibility candidate 2	0	8	4
9219	MTA2	metastasis associated 1 family, member 2	0	8	4
51562	MBIP	MAP3K12 binding inhibitory protein 1	0	8	4
754	PTTG1IP	pituitary tumor-transforming 1 interacting protein	0	8	4
55435	C4orf16	chromosome 4 open reading frame 16	0	8	4
9689	BZW1	basic leucine zipper and W2 domains 1	0	8	4
7570	ZNF22	zinc finger protein 22 (KOX 15)	0	8	4
388886	C22orf36	chromosome 22 open reading frame 36	0	8	4
84216	TMEM117	transmembrane protein 117	0	8	4
95681	TSGA14	testis specific, 14	0	8	4
5516	PPP2CB	protein phosphatase 2 (formerly 2A), catalytic subunit, beta i	0	8	4
58500	ZNF250	zinc finger protein 250	0	8	4
54813	BTBD5	BTB (POZ) domain containing 5	0	8	4
2444	FRK	fyn-related kinase	0	8	4
51277	RBJ	Ras-associated protein Rap1	0	8	4
56252	YLPM1	YLP motif containing 1	0	8	4
91452	ACBD5	acyl-Coenzyme A binding domain containing 5	0	8	4
51201	ZDHHC2	zinc finger, DHHC-type containing 2	0	8	4
7164	TPD52L1	tumor protein D52-like 1	0	8	4
148867	SLC30A7	solute carrier family 30 (zinc transporter), member 7	0	8	4
5549	PRELP	proline/arginine-rich end leucine-rich repeat protein	0	8	4
55040	EPN3	epsin 3	0	8	4
2696	GIPR	gastric inhibitory polypeptide receptor	0	8	4
23678	SGK3	serum/glucocorticoid regulated kinase family, member 3	0	8	4
4192	MDK	midkine (neurite growth-promoting factor 2)	0	8	4
91603	CCDC16	coiled-coil domain containing 16	0	8	4
493	ATP2B4	ATPase, Ca++ transporting, plasma membrane 4	0	8	4
259282	FAM44A	family with sequence similarity 44, member A	0	8	4
9726	ZNF646	zinc finger protein 646	0	8	4
55704	KIAA1212	KIAA1212	0	8	4
392364	LOC392364	similar to nuclear pore membrane protein 121	0	8	4
3785	KCNQ2	potassium voltage-gated channel, KQT-like subfamily, memb	0	8	4
11226	GALNT6	UDP-N-acetyl-alpha-D-galactosamine:polypeptide N-acetylg	0	8	4
84288	EFCAB2	EF-hand calcium binding domain 2	0	8	4
84722	PSRC1	proline/serine-rich coiled-coil 1	0	8	4
219333	USP12	ubiquitin specific peptidase 12	0	8	4
27440	CECR5	cat eye syndrome chromosome region, candidate 5	0	8	4
732	C8B	complement component 8, beta polypeptide	0	8	4
140849	C20orf69	chromosome 20 open reading frame 69	0	8	4
729975	FLJ30403	hypothetical protein LOC729975	0	8	4
10090	UST	uronyl-2-sulfotransferase	0	8	4
23518	R3HDM1	R3H domain containing 1	0	8	4
444	ASPH	aspartate beta-hydroxylase	0	8	4
80012	PHC3	polyhomeotic homolog 3 (Drosophila)	0	8	4
148523	C1orf51	chromosome 1 open reading frame 51	0	8	4
2820	GPD2	glycerol-3-phosphate dehydrogenase 2 (mitochondrial)	0	8	4

2550	GABBR1	gamma-aminobutyric acid (GABA) B receptor, 1	0	8	4
51088	KLHL5	kelch-like 5 (Drosophila)	0	8	4
5144	PDE4D	phosphodiesterase 4D, cAMP-specific (phosphodiesterase E	0	8	4
138649	ANKRD19	ankyrin repeat domain 19	0	8	4
56848	SPHK2	sphingosine kinase 2	0	8	4
9643	MORF4L2	mortality factor 4 like 2	0	8	4
4638	MYLK	myosin, light chain kinase	0	8	4
202052	DNAJC18	DnaJ (Hsp40) homolog, subfamily C, member 18	0	8	4
4670	HNRPM	heterogeneous nuclear ribonucleoprotein M	0	8	4
1261	CNGA3	cyclic nucleotide gated channel alpha 3	0	8	4
84777	MGC11082	hypothetical protein MGC11082	0	8	4
10325	RRAGB	Ras-related GTP binding B	0	8	4
1241	LTB4R	leukotriene B4 receptor	0	8	4
50650	ARHGEF3	Rho guanine nucleotide exchange factor (GEF) 3	0	8	4
57645	POGK	pogo transposable element with KRAB domain	0	8	4
23122	CLASP2	cytoplasmic linker associated protein 2	0	8	4
55127	HEATR1	HEAT repeat containing 1	0	8	4
653189	LOC653189	similar to basic transcription factor 3-like 4	0	8	4
343099	CCDC18	coiled-coil domain containing 18	0	8	4
283489	C13orf8	chromosome 13 open reading frame 8	0	8	4
2222	FDFT1	farnesyl-diphosphate farnesyltransferase 1	0	8	4
26043	UBXD7	UBX domain containing 7	0	8	4
256356	GK5	glycerol kinase 5 (putative)	0	8	4
414245	C10orf103	chromosome 10 open reading frame 103	0	8	4
275	AMT	aminomethyltransferase	0	8	4
10133	OPTN	optineurin	0	8	4
286167	LOC286167	hypothetical protein LOC286167	0	8	4
55074	OXR1	oxidation resistance 1	0	8	4
55862	ECHDC1	enoyl Coenzyme A hydratase domain containing 1	0	8	4
8577	TMEFF1	transmembrane protein with EGF-like and two follistatin-like	8	0	4
412	STS	steroid sulfatase (microsomal), arylsulfatase C, isozyme S	8	0	4
8509	NDST2	N-deacetylase/N-sulfotransferase (heparan glucosaminyl) 2	8	0	4
284004	HEXDC	hexosaminidase (glycosyl hydrolase family 20, catalytic dom	8	0	4
9819	TSC22D2	TSC22 domain family, member 2	8	0	4
204851	HIPK1	homeodomain interacting protein kinase 1	8	0	4
51809	GALNT7	UDP-N-acetyl-alpha-D-galactosamine:polypeptide N-acetylg	8	0	4
27324	TNRC9	trinucleotide repeat containing 9	8	0	4
23351	KIAA0323	KIAA0323	8	0	4
10006	ABI1	abl-interactor 1	8	0	4
10011	SRA1	steroid receptor RNA activator 1	8	0	4
64770	CCDC14	coiled-coil domain containing 14	8	0	4
56907	SPIRE1	spire homolog 1 (Drosophila)	8	0	4
9477	TRFP	Trf (TATA binding protein-related factor)-proximal homolog (	8	0	4
55907	CMAS	cytidine monophosphate N-acetylneuraminic acid synthetase	8	0	4
328	APEX1	APEX nuclease (multifunctional DNA repair enzyme) 1	8	0	4
23004	KIAA0825	KIAA0825 protein	8	0	4
643921	LOC643921	similar to Tyrosine-protein phosphatase non-receptor type 1	8	0	4
51093	C1orf66	chromosome 1 open reading frame 66	8	0	4
26298	EHF	ets homologous factor	8	0	4
4327	MMP19	matrix metallopeptidase 19	8	0	4
1962	EHHADH	enoyl-Coenzyme A, hydratase/3-hydroxyacyl Coenzyme A d	8	0	4

7348	UPK1B	uroplakin 1B	8	0	4
114882	OSBPL8	oxysterol binding protein-like 8	8	0	4
79631	EFTUD1	elongation factor Tu GTP binding domain containing 1	8	0	4
22795	NID2	nidogen 2 (osteonidogen)	8	0	4
2296	FOXC1	forkhead box C1	8	0	4
80059	LRRTM4	leucine rich repeat transmembrane neuronal 4	8	0	4
55022	FLJ20701	hypothetical protein FLJ20701	8	0	4
51716	CES4	carboxylesterase 4-like	8	0	4
5029	P2RY2	purinergic receptor P2Y, G-protein coupled, 2	8	0	4
29799	YPEL1	yippee-like 1 (Drosophila)	8	0	4
9813	KIAA0494	KIAA0494	8	0	4
51550	CINP	cyclin-dependent kinase 2-interacting protein	8	0	4
55034	MOCOS	molybdenum cofactor sulfurase	8	0	4
11006	LILRB4	leukocyte immunoglobulin-like receptor, subfamily B (with TM	8	0	4
399671	HEATR4	HEAT repeat containing 4	8	0	4
2918	GRM8	glutamate receptor, metabotropic 8	8	0	4
1951	CELSR3	cadherin, EGF LAG seven-pass G-type receptor 3 (flamingo	8	0	4
123	ADFP	adipose differentiation-related protein	8	0	4
124093	CCDC78	coiled-coil domain containing 78	8	0	4
55795	PCID2	PCI domain containing 2	8	0	4
55206	SBNO1	strawberry notch homolog 1 (Drosophila)	8	0	4
25780	RASGRP3	RAS guanyl releasing protein 3 (calcium and DAG-regulated	8	0	4
57396	CLK4	CDC-like kinase 4	8	0	4
4665	NAB2	NGFI-A binding protein 2 (EGR1 binding protein 2)	8	0	4
57594	KIAA1443	KIAA1443	8	0	4
400657	LOC400657	hypothetical gene supported by BC036588	8	0	4
9232	PTTG1	pituitary tumor-transforming 1	8	0	4
65084	TMEM135	transmembrane protein 135	8	0	4
26233	FBXL6	F-box and leucine-rich repeat protein 6	8	0	4
9388	LIPG	lipase, endothelial	8	0	4
57453	DSCAML1	Down syndrome cell adhesion molecule like 1	8	0	4
11040	PIM2	pim-2 oncogene	8	0	4
57561	ARRDC3	arrestin domain containing 3	8	0	4
284440	MGC39821	hypothetical protein MGC39821	8	0	4
4901	NRL	neural retina leucine zipper	8	0	4
55054	ATG16L1	ATG16 autophagy related 16-like 1 (S. cerevisiae)	8	0	4
9792	SERTAD2	SERTA domain containing 2	8	0	4
5992	RFX4	regulatory factor X, 4 (influences HLA class II expression)	8	0	4
22841	RAB11FIP2	RAB11 family interacting protein 2 (class I)	8	0	4
29890	RBM15B	RNA binding motif protein 15B	8	0	4
904	CCNT1	cyclin T1	8	0	4
867	CBL	Cas-Br-M (murine) ecotropic retroviral transforming sequenc	8	0	4
63939	C20orf177	chromosome 20 open reading frame 177	8	0	4
653464	LOC653464	similar to SLIT-ROBO Rho GTPase-activating protein 2 (srG	8	0	4
9632	SEC24C	SEC24 related gene family, member C (S. cerevisiae)	8	0	4
23279	NUP160	nucleoporin 160kDa	8	0	4
5662	PSD	pleckstrin and Sec7 domain containing	8	0	4
84946	LTV1	LTV1 homolog (S. cerevisiae)	8	0	4
7266	DNAJC7	DnaJ (Hsp40) homolog, subfamily C, member 7	8	0	4
80237	ELL3	elongation factor RNA polymerase II-like 3	8	0	4
118924	C10orf4	chromosome 10 open reading frame 4	8	0	4

116150	NUS1	nuclear undecaprenyl pyrophosphate synthase 1 homolog (S	8	0	4
7637	ZNF84	zinc finger protein 84	8	0	4
29959	NRBP1	nuclear receptor binding protein 1	8	0	4
85007	AGXT2L2	alanine-glyoxylate aminotransferase 2-like 2	8	0	4
79984	FLJ21736	esterase 31	8	0	4
80263	TRIM45	tripartite motif-containing 45	8	0	4
4599	MX1	myxovirus (influenza virus) resistance 1, interferon-inducible	8	0	4
55086	CXorf57	chromosome X open reading frame 57	8	0	4
55149	PAPD1	PAP associated domain containing 1	8	0	4
253832	ZDHHC20	zinc finger, DHHC-type containing 20	8	0	4
1632	DCI	dodecenoyl-Coenzyme A delta isomerase (3,2 trans-enoyl-C	8	0	4
6876	TAGLN	transgelin	8	0	4
171425	CLYBL	citrate lyase beta like	8	0	4
51016	C14orf122	chromosome 14 open reading frame 122	8	0	4
8527	DGKD	diacylglycerol kinase, delta 130kDa	8	0	4
79596	C13orf7	chromosome 13 open reading frame 7	8	0	4
339500	ZNF678	zinc finger protein 678	8	0	4
3321	IGSF3	immunoglobulin superfamily, member 3	8	0	4
3619	INCENP	inner centromere protein antigens 135/155kDa	8	0	4
55835	CENPJ	centromere protein J	8	0	4
59342	SCPEP1	serine carboxypeptidase 1	8	0	4
84294	C8orf53	chromosome 8 open reading frame 53	8	0	4
2553	GABPB2	GA binding protein transcription factor, beta subunit 2	4	3	3.5
645757	LOC645757	hypothetical LOC645757	4	3	3.5
23142	DCUN1D4	DCN1, defective in cullin neddylation 1, domain containing 4	4	3	3.5
344595	LOC344595	hypothetical LOC344595	4	3	3.5
79745	CLIP4	CAP-GLY domain containing linker protein family, member 4	4	3	3.5
8861	LDB1	LIM domain binding 1	4	3	3.5
166793	ZNF509	zinc finger protein 509	4	3	3.5
132789	GNPDA2	glucosamine-6-phosphate deaminase 2	4	3	3.5
10606	PAICS	phosphoribosylaminoimidazole carboxylase, phosphoribosyla	4	3	3.5
47	ACLY	ATP citrate lyase	4	3	3.5
411	ARSB	arylsulfatase B	4	3	3.5
83440	ADPGK	ADP-dependent glucokinase	2	5	3.5
10134	BCAP31	B-cell receptor-associated protein 31	2	5	3.5
4892	NRAP	nebulin-related anchoring protein	2	5	3.5
10998	SLC27A5	solute carrier family 27 (fatty acid transporter), member 5	2	5	3.5
51651	PTRH2	peptidyl-tRNA hydrolase 2	2	5	3.5
645513	LOC645513	similar to septin 7	2	5	3.5
54868	TMEM104	transmembrane protein 104	2	5	3.5
1452	CSNK1A1	casein kinase 1, alpha 1	2	5	3.5
84376	HOOK3	hook homolog 3 (Drosophila)	0	7	3.5
84816	RTN4IP1	reticulon 4 interacting protein 1	0	7	3.5
5225	PGC	progastricsin (pepsinogen C)	0	7	3.5
8502	PKP4	plakophilin 4	0	7	3.5
649294	LOC649294	hypothetical protein LOC649294	0	7	3.5
84254	CAMKK1	calcium/calmodulin-dependent protein kinase kinase 1, alpha	0	7	3.5
6433	SFRS8	splicing factor, arginine/serine-rich 8 (suppressor-of-white-ap	0	7	3.5
64780	MICAL1	microtubule associated monoxygenase, calponin and LIM do	0	7	3.5
57654	KIAA1530	KIAA1530 protein	0	7	3.5
91272	FAM44B	family with sequence similarity 44, member B	0	7	3.5

340156	RP11-145H9	hypothetical protein LOC340156	0	7	3.5
55257	C20orf20	chromosome 20 open reading frame 20	0	7	3.5
2717	GLA	galactosidase, alpha	0	7	3.5
54965	PIGX	phosphatidylinositol glycan anchor biosynthesis, class X	0	7	3.5
5925	RB1	retinoblastoma 1 (including osteosarcoma)	0	7	3.5
10379	ISGF3G	interferon-stimulated transcription factor 3, gamma 48kDa	0	7	3.5
128553	TSHZ2	teashirt family zinc finger 2	0	7	3.5
55160	ARHGEF10L	Rho guanine nucleotide exchange factor (GEF) 10-like	0	7	3.5
121512	FGD4	FYVE, RhoGEF and PH domain containing 4	0	7	3.5
154822	LOC154822	hypothetical protein LOC154822	0	7	3.5
339665	SLC35E4	solute carrier family 35, member E4	0	7	3.5
6688	SPI1	spleen focus forming virus (SFFV) proviral integration oncog	0	7	3.5
8909	P11	26 serine protease	0	7	3.5
8505	PARG	poly (ADP-ribose) glycohydrolase	0	7	3.5
4779	NFE2L1	nuclear factor (erythroid-derived 2)-like 1	0	7	3.5
6926	TBX3	T-box 3 (ulnar mammary syndrome)	0	7	3.5
80173	IFT74	intraflagellar transport 74 homolog (Chlamydomonas)	0	7	3.5
3708	ITPR1	inositol 1,4,5-triphosphate receptor, type 1	0	7	3.5
23263	MCF2L	MCF.2 cell line derived transforming sequence-like	0	7	3.5
83989	C5orf21	chromosome 5 open reading frame 21	0	7	3.5
10181	RBM5	RNA binding motif protein 5	0	7	3.5
283820	NOMO2	NODAL modulator 2	0	7	3.5
22794	CASC3	cancer susceptibility candidate 3	0	7	3.5
4124	MAN2A1	mannosidase, alpha, class 2A, member 1	0	7	3.5
57506	VISA	virus-induced signaling adapter	0	7	3.5
2537	IFI6	interferon, alpha-inducible protein 6	0	7	3.5
90592	ZNF700	zinc finger protein 700	0	7	3.5
497256	LOC497256	hypothetical LOC497256	0	7	3.5
28952	CCDC22	coiled-coil domain containing 22	0	7	3.5
23514	KIAA0146	KIAA0146	0	7	3.5
10422	UBADC1	ubiquitin associated domain containing 1	0	7	3.5
114134	SLC2A13	solute carrier family 2 (facilitated glucose transporter), memb	0	7	3.5
81794	ADAMTS10	ADAM metallopeptidase with thrombospondin type 1 motif, 1	0	7	3.5
91442	C19orf40	chromosome 19 open reading frame 40	0	7	3.5
9493	KIF23	kinesin family member 23	0	7	3.5
1826	DSCAM	Down syndrome cell adhesion molecule	0	7	3.5
5203	PFDN4	prefoldin subunit 4	0	7	3.5
8501	SLC43A1	solute carrier family 43, member 1	0	7	3.5
57709	SLC7A14	solute carrier family 7 (cationic amino acid transporter, y+ sy	0	7	3.5
5533	PPP3CC	protein phosphatase 3 (formerly 2B), catalytic subunit, gamm	0	7	3.5
9557	CHD1L	chromodomain helicase DNA binding protein 1-like	0	7	3.5
9895	KIAA0329	KIAA0329	0	7	3.5
55214	LEPREL1	leprecan-like 1	0	7	3.5
11260	XPOT	exportin, tRNA (nuclear export receptor for tRNAs)	0	7	3.5
1595	CYP51A1	cytochrome P450, family 51, subfamily A, polypeptide 1	0	7	3.5
577	BAI3	brain-specific angiogenesis inhibitor 3	0	7	3.5
153364	LOC153364	similar to metallo-beta-lactamase superfamily protein	0	7	3.5
80144	FRAS1	Fraser syndrome 1	0	7	3.5
10478	SLC25A17	solute carrier family 25 (mitochondrial carrier; peroxisomal m	0	7	3.5
10579	TACC2	transforming, acidic coiled-coil containing protein 2	0	7	3.5
11098	PRSS23	protease, serine, 23	0	7	3.5

83451	ABHD11	abhydrolase domain containing 11	0	7	3.5
80167	C4orf29	chromosome 4 open reading frame 29	0	7	3.5
5997	RGS2	regulator of G-protein signalling 2, 24kDa	0	7	3.5
51185	CRBN	cereblon	0	7	3.5
92241	RCSD1	RCSD domain containing 1	0	7	3.5
80824	DUSP16	dual specificity phosphatase 16	0	7	3.5
8398	PLA2G6	phospholipase A2, group VI (cytosolic, calcium-independent)	0	7	3.5
84963	MGC15613	hypothetical protein MGC15613	0	7	3.5
4882	NPR2	natriuretic peptide receptor B/guanylate cyclase B (atrionatri	0	7	3.5
22941	SHANK2	SH3 and multiple ankyrin repeat domains 2	0	7	3.5
83869	TTTY14	testis-specific transcript, Y-linked 14	0	7	3.5
92703	TMEM183A	transmembrane protein 183A	0	7	3.5
23049	SMG1	PI-3-kinase-related kinase SMG-1	0	7	3.5
439921	MXRA7	matrix-remodelling associated 7	0	7	3.5
92667	C20orf72	chromosome 20 open reading frame 72	0	7	3.5
645644	FLJ42627	hypothetical protein LOC645644	0	7	3.5
25854	DKFZP564J1	DKFZP564J102 protein	4	2	3
729852	LOC729852	hypothetical protein LOC729852	4	2	3
105	ADARB2	adenosine deaminase, RNA-specific, B2 (RED2 homolog rat	4	2	3
10944	C11orf58	chromosome 11 open reading frame 58	4	2	3
729810	LOC729810	hypothetical protein LOC729810	4	2	3
2889	RAPGEF1	Rap guanine nucleotide exchange factor (GEF) 1	4	2	3
94134	ARHGAP12	Rho GTPase activating protein 12	4	2	3
4171	MCM2	MCM2 minichromosome maintenance deficient 2, mitotin (S.	4	2	3
9990	SLC12A6	solute carrier family 12 (potassium/chloride transporters), me	4	2	3
66036	MTMR9	myotubularin related protein 9	4	2	3
5611	DNAJC3	DnaJ (Hsp40) homolog, subfamily C, member 3	4	2	3
55148	C14orf130	chromosome 14 open reading frame 130	4	2	3
25945	PVRL3	poliovirus receptor-related 3	4	2	3
3188	HNRPH2	heterogeneous nuclear ribonucleoprotein H2 (H')	4	2	3
84905	ZNF341	zinc finger protein 341	4	2	3
4798	NFRKB	nuclear factor related to kappaB binding protein	4	2	3
861	RUNX1	runt-related transcription factor 1 (acute myeloid leukemia 1;	4	2	3
730021	LOC730021	hypothetical protein LOC730021	4	2	3
740	MRPL49	mitochondrial ribosomal protein L49	4	2	3
135293	ACY1L2	aminoacylase 1-like 2	4	2	3
154807	VKORC1L1	vitamin K epoxide reductase complex, subunit 1-like 1	4	2	3
22898	DENND3	DENN/MADD domain containing 3	2	4	3
79572	ATP13A3	ATPase type 13A3	0	6	3
4836	NMT1	N-myristoyltransferase 1	0	6	3
10919	EHMT2	euchromatic histone-lysine N-methyltransferase 2	0	6	3
23273	KIAA0367	KIAA0367	0	6	3
92017	LOC92017	similar to RIKEN cDNA 4933437K13	0	6	3
6513	SLC2A1	solute carrier family 2 (facilitated glucose transporter), memb	0	6	3
474384	F8A3	coagulation factor VIII-associated (intronic transcript) 3	0	6	3
66008	TRAK2	trafficking protein, kinesin binding 2	0	6	3
23078	RP11-125A7	KIAA0564 protein	0	6	3
55186	SLC25A36	solute carrier family 25, member 36	0	6	3
7922	SLC39A7	solute carrier family 39 (zinc transporter), member 7	0	6	3
11094	C9orf7	chromosome 9 open reading frame 7	0	6	3
5301	PIN1L	protein (peptidylprolyl cis/trans isomerase) NIMA-interacting	0	6	3

285800	MGC35308	hypothetical protein MGC35308	0	6	3
10863	ADAM28	ADAM metallopeptidase domain 28	0	6	3
7867	MAPKAPK3	mitogen-activated protein kinase-activated protein kinase 3	0	6	3
83931	STK40	serine/threonine kinase 40	0	6	3
54897	CASZ1	castor homolog 1, zinc finger (Drosophila)	0	6	3
25800	SLC39A6	solute carrier family 39 (zinc transporter), member 6	0	6	3
56957	OTUD7B	OTU domain containing 7B	0	6	3
2956	MSH6	mutS homolog 6 (E. coli)	0	6	3
55471	PRO1853	hypothetical protein PRO1853	0	6	3
374920	LOC374920	hypothetical protein LOC374920	0	6	3
1607	DGKB	diacylglycerol kinase, beta 90kDa	0	6	3
55279	ZNF654	zinc finger protein 654	0	6	3
7799	PRDM2	PR domain containing 2, with ZNF domain	0	6	3
9020	MAP3K14	mitogen-activated protein kinase kinase kinase 14	0	6	3
1837	DTNA	dystrobrevin, alpha	0	6	3
51684	SUFU	suppressor of fused homolog (Drosophila)	0	6	3
161436	EML5	echinoderm microtubule associated protein like 5	0	6	3
163590	TOR1AIP2	torsin A interacting protein 2	0	6	3
23250	ATP11A	ATPase, Class VI, type 11A	0	6	3
55273	TMEM100	transmembrane protein 100	0	6	3
284669	LOC284669	hypothetical protein LOC284669	0	6	3
8050	PDHX	pyruvate dehydrogenase complex, component X	0	6	3
8269	CXorf12	chromosome X open reading frame 12	0	6	3
57523	KIAA1305	KIAA1305	0	6	3
23321	TRIM2	tripartite motif-containing 2	0	6	3
83692	CD99L2	CD99 molecule-like 2	0	6	3
6427	SFRS2	splicing factor, arginine/serine-rich 2	0	6	3
7407	VARS	valyl-tRNA synthetase	0	6	3
9653	HS2ST1	heparan sulfate 2-O-sulfotransferase 1	0	6	3
9541	CIR	CBF1 interacting corepressor	0	6	3
10682	EBP	emopamil binding protein (sterol isomerase)	0	6	3
7006	TEC	tec protein tyrosine kinase	0	6	3
80256	KIAA1539	KIAA1539	0	6	3
57571	KIAA1394	KIAA1394 protein	0	6	3
9910	RABGAP1L	RAB GTPase activating protein 1-like	0	6	3
1002	CDH4	cadherin 4, type 1, R-cadherin (retinal)	0	6	3
55210	ATAD3A	ATPase family, AAA domain containing 3A	0	6	3
10301	DLEU1	deleted in lymphocytic leukemia, 1	0	6	3
389941	C1QL3	complement component 1, q subcomponent-like 3	0	6	3
11123	DSCR1L2	Down syndrome critical region gene 1-like 2	0	6	3
399668	MGC39606	hypothetical protein MGC39606	0	6	3
9830	TRIM14	tripartite motif-containing 14	0	6	3
8888	MCM3AP	MCM3 minichromosome maintenance deficient 3 (S. cerevis	0	6	3
571	BACH1	BTB and CNC homology 1, basic leucine zipper transcriptior	0	6	3
150709	ANKAR	ankyrin and armadillo repeat containing	0	6	3
136853	SRCRB4D	scavenger receptor cysteine rich domain containing, group E	0	6	3
143872	FLJ32810	hypothetical protein FLJ32810	0	6	3
150350	ENTHD1	ENTH domain containing 1	0	6	3
137994	LETM2	leucine zipper-EF-hand containing transmembrane protein 2	0	6	3
3485	IGFBP2	insulin-like growth factor binding protein 2, 36kDa	0	6	3
79890	RIN3	Ras and Rab interactor 3	0	6	3

10170	DHRS9	dehydrogenase/reductase (SDR family) member 9	0	6	3
9942	XYLB	xylulokinase homolog (H. influenzae)	0	6	3
57124	CD248	CD248 molecule, endosialin	0	6	3
5705	PSMC5	proteasome (prosome, macropain) 26S subunit, ATPase, 5	0	6	3
3752	KCND3	potassium voltage-gated channel, Shal-related subfamily, m	0	6	3
2870	GRK6	G protein-coupled receptor kinase 6	0	6	3
645323	LOC645323	hypothetical LOC645323	0	6	3
4017	LOXL2	lysyl oxidase-like 2	0	6	3
610	HCN2	hyperpolarization activated cyclic nucleotide-gated potassiur	0	6	3
79791	FBXO31	F-box protein 31	0	6	3
80307	FER1L4	fer-1-like 4 (C. elegans)	0	6	3
441893	LOC441893	similar to Glyceraldehyde-3-phosphate dehydrogenase (GAF	0	6	3
6712	SPTBN2	spectrin, beta, non-erythrocytic 2	6	0	3
80760	ITIH5	inter-alpha (globulin) inhibitor H5	6	0	3
3607	FOXK2	forkhead box K2	6	0	3
146346	LOC146346	hypothetical protein LOC146346	6	0	3
153222	LOC153222	adult retina protein	6	0	3
285147	LOC285147	hypothetical protein LOC285147	6	0	3
9693	RAPGEF2	Rap guanine nucleotide exchange factor (GEF) 2	6	0	3
80757	TMEM121	transmembrane protein 121	6	0	3
93134	ZNF561	zinc finger protein 561	6	0	3
442028	LOC442028	hypothetical LOC442028	6	0	3
51380	CSAD	cysteine sulfinic acid decarboxylase	6	0	3
26503	SLC17A5	solute carrier family 17 (anion/sugar transporter), member 5	6	0	3
51669	TMEM66	transmembrane protein 66	6	0	3
5826	ABCD4	ATP-binding cassette, sub-family D (ALD), member 4	6	0	3
50515	CHST11	carbohydrate (chondroitin 4) sulfotransferase 11	6	0	3
729620	LOC729620	hypothetical protein LOC729620	6	0	3
8515	ITGA10	integrin, alpha 10	6	0	3
1026	CDKN1A	cyclin-dependent kinase inhibitor 1A (p21, Cip1)	6	0	3
5678	PSG9	pregnancy specific beta-1-glycoprotein 9	6	0	3
23031	MAST3	microtubule associated serine/threonine kinase 3	6	0	3
84179	MFSD7	major facilitator superfamily domain containing 7	6	0	3
4745	NELL1	NEL-like 1 (chicken)	6	0	3
348110	C15orf38	chromosome 15 open reading frame 38	6	0	3
84133	ZNRF3	zinc and ring finger 3	6	0	3
22859	LPHN1	latrophilin 1	6	0	3
80124	VCPIP1	valosin containing protein (p97)/p47 complex interacting prot	6	0	3
92259	MRPS36	mitochondrial ribosomal protein S36	6	0	3
60672	RP5-1077B9	invasion inhibitory protein 45	6	0	3
7690	ZNF131	zinc finger protein 131	6	0	3
2968	GTF2H4	general transcription factor IIH, polypeptide 4, 52kDa	6	0	3
27031	NPHP3	nephronophthisis 3 (adolescent)	6	0	3
22915	MMRN1	multimerin 1	6	0	3
22902	RUFY3	RUN and FYVE domain containing 3	6	0	3
64324	NSD1	nuclear receptor binding SET domain protein 1	6	0	3
5093	PCBP1	poly(rC) binding protein 1	6	0	3
951	CD37	CD37 molecule	6	0	3
9980	DOPEY2	dopey family member 2	6	0	3
9648	GCC2	GRIP and coiled-coil domain containing 2	6	0	3
10023	FRAT1	frequently rearranged in advanced T-cell lymphomas	6	0	3

118421	C21orf100	chromosome 21 open reading frame 100	6	0	3
81602	CDADC1	cytidine and dCMP deaminase domain containing 1	6	0	3
13	AADAC	arylacetamide deacetylase (esterase)	6	0	3
25855	BRMS1	breast cancer metastasis suppressor 1	6	0	3
23154	NCDN	neurochondrin	6	0	3
6232	RPS27	ribosomal protein S27 (metallopanstimulin 1)	6	0	3
2863	GPR39	G protein-coupled receptor 39	6	0	3
27294	DHDH	dihydrodiol dehydrogenase (dimeric)	6	0	3
8303	SNN	stannin	6	0	3
905	CCNT2	cyclin T2	6	0	3
80319	CXXC4	CXXC finger 4	6	0	3
6608	SMO	smoothened homolog (Drosophila)	6	0	3
23394	ADNP	activity-dependent neuroprotector	6	0	3
9857	CEP350	centrosomal protein 350kDa	6	0	3
4584	MUC3A	mucin 3A, cell surface associated	6	0	3
11127	KIF3A	kinesin family member 3A	6	0	3
23729	CARKL	carbohydrate kinase-like	6	0	3
203111	C8orf47	chromosome 8 open reading frame 47	6	0	3
55154	MSTO1	misato homolog 1 (Drosophila)	6	0	3
90338	ZNF160	zinc finger protein 160	6	0	3
285761	DCBLD1	discoidin, CUB and LCCL domain containing 1	6	0	3
113263	GLCCI1	glucocorticoid induced transcript 1	6	0	3
79657	FLJ21908	hypothetical protein FLJ21908	6	0	3
646977	NA	NA	6	0	3
647217	LOC647217	hypothetical protein LOC647217	6	0	3
3164	NR4A1	nuclear receptor subfamily 4, group A, member 1	6	0	3
9440	CRSP6	cofactor required for Sp1 transcriptional activation, subunit 6	6	0	3
129790	C7orf13	chromosome 7 open reading frame 13	6	0	3
23603	CORO1C	coronin, actin binding protein, 1C	6	0	3
128	ADH5	alcohol dehydrogenase 5 (class III), chi polypeptide	6	0	3
26031	OSBPL3	oxysterol binding protein-like 3	6	0	3
11187	PKP3	plakophilin 3	6	0	3
467	ATF3	activating transcription factor 3	6	0	3
84186	ZCCHC7	zinc finger, CCHC domain containing 7	6	0	3
21	ABCA3	ATP-binding cassette, sub-family A (ABC1), member 3	6	0	3
51347	TAOK3	TAO kinase 3	6	0	3
6641	SNTB1	syntrophin, beta 1 (dystrophin-associated protein A1, 59kDa	6	0	3
51575	ESF1	ESF1, nucleolar pre-rRNA processing protein, homolog (S. c	6	0	3
6100	RP9	retinitis pigmentosa 9 (autosomal dominant)	6	0	3
255352	C10orf93	chromosome 10 open reading frame 93	6	0	3
55432	YOD1	YOD1 OTU deubiquinating enzyme 1 homolog (S. cerevisiae	6	0	3
10163	WASF2	WAS protein family, member 2	6	0	3
84913	ATOH8	atonal homolog 8 (Drosophila)	6	0	3
55114	ARHGAP17	Rho GTPase activating protein 17	6	0	3
145581	LRFN5	leucine rich repeat and fibronectin type III domain containing	6	0	3
339047	LOC339047	hypothetical protein LOC339047	6	0	3
126231	ZNF573	zinc finger protein 573	6	0	3
84876	TMEM142A	transmembrane protein 142A	6	0	3
1551	CYP3A7	cytochrome P450, family 3, subfamily A, polypeptide 7	6	0	3
81609	SNX27	sorting nexin family member 27	6	0	3
26128	KIAA1279	KIAA1279	6	0	3

196	AHR	aryl hydrocarbon receptor	6	0	3
79750	ZNF659	zinc finger protein 659	6	0	3
8883	APPBP1	amyloid beta precursor protein binding protein 1	6	0	3
729624	LOC729624	hypothetical protein LOC729624	6	0	3
150483	TEKT4	tektin 4	6	0	3
5565	PRKAB2	protein kinase, AMP-activated, beta 2 non-catalytic subunit	6	0	3
29960	FTSJ2	FtsJ homolog 2 (E. coli)	6	0	3
5250	SLC25A3	solute carrier family 25 (mitochondrial carrier; phosphate car	6	0	3
85019	C18orf45	chromosome 18 open reading frame 45	6	0	3
55709	KBTBD4	kelch repeat and BTB (POZ) domain containing 4	6	0	3
199953	RP13-15M17	hypothetical protein LOC199953	6	0	3
643866	CBLN3	cerebellin 3 precursor	6	0	3
51660	BRP44L	brain protein 44-like	6	0	3
26585	GREM1	gremlin 1, cysteine knot superfamily, homolog (Xenopus laev	6	0	3
1104	RCC1	regulator of chromosome condensation 1	6	0	3
10849	CD3EAP	CD3e molecule, epsilon associated protein	6	0	3
92454	PRR8	proline rich 8	6	0	3
140710	C20orf117	chromosome 20 open reading frame 117	6	0	3
9892	SNAP91	synaptosomal-associated protein, 91kDa homolog (mouse)	6	0	3
92979	MAR9	membrane-associated ring finger (C3HC4) 9	6	0	3
167227	DCP2	DCP2 decapping enzyme homolog (S. cerevisiae)	6	0	3
122553	TRAPPC6B	trafficking protein particle complex 6B	6	0	3
55632	KIAA1333	KIAA1333	6	0	3
6903	TBCC	tubulin folding cofactor C	6	0	3
80818	ZNF436	zinc finger protein 436	6	0	3
64766	S100PBP	S100P binding protein	6	0	3
5550	PREP	prolyl endopeptidase	6	0	3
114548	NLRP3	NLR family, pyrin domain containing 3	6	0	3
339977	LOC339977	similar to hypothetical protein MGC38937	6	0	3
5646	PRSS3	protease, serine, 3 (mesotrypsin)	6	0	3
5046	PCSK6	proprotein convertase subtilisin/kexin type 6	6	0	3
200634	KRTCAP3	keratinocyte associated protein 3	6	0	3
3508	IGHMBP2	immunoglobulin mu binding protein 2	6	0	3
22928	SEPHS2	selenophosphate synthetase 2	6	0	3
79728	PALB2	partner and localizer of BRCA2	6	0	3
389008	LOC389008	similar to KIAA1641 protein	6	0	3
84883	AIFM2	apoptosis-inducing factor, mitochondrion-associated, 2	6	0	3
23054	NCOA6	nuclear receptor coactivator 6	6	0	3
7482	WNT2B	wingless-type MMTV integration site family, member 2B	6	0	3
723788	MIG7	mig-7	2	3	2.5
55814	BDP1	B double prime 1, subunit of RNA polymerase III transcriptio	2	3	2.5
254887	ZDHHC23	zinc finger, DHHC-type containing 23	2	3	2.5
6938	TCF12	transcription factor 12 (HTF4, helix-loop-helix transcription fa	2	3	2.5
23341	DNAJC16	DnaJ (Hsp40) homolog, subfamily C, member 16	2	3	2.5
2166	FAAH	fatty acid amide hydrolase	2	3	2.5
26960	NBEA	neurobeachin	2	3	2.5
550631	LOC550631	hypothetical LOC550631	2	3	2.5
115	ADCY9	adenylate cyclase 9	2	3	2.5
5396	PRRX1	paired related homeobox 1	2	3	2.5
64478	CSMD1	CUB and Sushi multiple domains 1	2	3	2.5
4682	NUBP1	nucleotide binding protein 1 (MinD homolog, E. coli)	2	3	2.5

5216	PFN1	profilin 1	2	3	2.5
6840	SVIL	supervillin	2	3	2.5
81029	WNT5B	wingless-type MMTV integration site family, member 5B	2	3	2.5
60625	DHX35	DEAH (Asp-Glu-Ala-His) box polypeptide 35	2	3	2.5
10892	MALT1	mucosa associated lymphoid tissue lymphoma translocation	2	3	2.5
6586	SLIT3	slit homolog 3 (Drosophila)	2	3	2.5
8434	RECK	reversion-inducing-cysteine-rich protein with kazal motifs	0	5	2.5
90627	STARD13	START domain containing 13	0	5	2.5
55205	ZNF532	zinc finger protein 532	0	5	2.5
90806	ANGEL2	angel homolog 2 (Drosophila)	0	5	2.5
65059	RAPH1	Ras association (RalGDS/AF-6) and pleckstrin homology doi	0	5	2.5
79163	LENG6	leukocyte receptor cluster (LRC) member 6	0	5	2.5
246243	RNASEH1	ribonuclease H1	0	5	2.5
26289	AK5	adenylate kinase 5	0	5	2.5
29066	ZC3H7A	zinc finger CCCH-type containing 7A	0	5	2.5
6660	SOX5	SRY (sex determining region Y)-box 5	0	5	2.5
116236	LOC116236	hypothetical protein LOC116236	0	5	2.5
10681	GNB5	guanine nucleotide binding protein (G protein), beta 5	0	5	2.5
4928	NUP98	nucleoporin 98kDa	0	5	2.5
55299	BXDC2	brix domain containing 2	0	5	2.5
2312	FLG	filaggrin	0	5	2.5
55640	C14orf58	chromosome 14 open reading frame 58	0	5	2.5
348094	ANKDD1A	ankyrin repeat and death domain containing 1A	0	5	2.5
94032	CAMK2N2	calcium/calmodulin-dependent protein kinase II inhibitor 2	0	5	2.5
7134	TNNC1	troponin C type 1 (slow)	0	5	2.5
285958	C7orf40	chromosome 7 open reading frame 40	0	5	2.5
8045	RASSF7	Ras association (RalGDS/AF-6) domain family 7	0	5	2.5
1088	CEACAM8	carcinoembryonic antigen-related cell adhesion molecule 8	0	5	2.5
23398	PPWD1	peptidylprolyl isomerase domain and WD repeat containing 1	0	5	2.5
4683	NBN	nibrin	0	5	2.5
9779	TBC1D5	TBC1 domain family, member 5	0	5	2.5
1936	EEF1D	eukaryotic translation elongation factor 1 delta (guanine nucl	0	5	2.5
27125	AFF4	AF4/FMR2 family, member 4	0	5	2.5
155382	VPS37D	vacuolar protein sorting 37 homolog D (S. cerevisiae)	0	5	2.5
1102	RCBTB2	regulator of chromosome condensation (RCC1) and BTB (PO	0	5	2.5
55009	C19orf24	chromosome 19 open reading frame 24	0	5	2.5
644838	LOC644838	hypothetical LOC644838	0	5	2.5
84289	ING5	inhibitor of growth family, member 5	0	5	2.5
64132	XYLT2	xylosyltransferase II	0	5	2.5
140851	C20orf127	chromosome 20 open reading frame 127	0	5	2.5
1361	CPB2	carboxypeptidase B2 (plasma, carboxypeptidase U)	0	5	2.5
153657	FLJ25439	hypothetical protein FLJ25439	0	5	2.5
375607	FLJ39237	FLJ39237 protein	0	5	2.5
91355	LRP5L	low density lipoprotein receptor-related protein 5-like	0	5	2.5
161742	SPRED1	sprouty-related, EVH1 domain containing 1	0	5	2.5
79736	C17orf42	chromosome 17 open reading frame 42	0	5	2.5
84190	C12orf26	chromosome 12 open reading frame 26	0	5	2.5
4803	NGFB	nerve growth factor, beta polypeptide	0	5	2.5
23760	PITPNB	phosphatidylinositol transfer protein, beta	0	5	2.5
6558	SLC12A2	solute carrier family 12 (sodium/potassium/chloride transport	0	5	2.5
10199	MPHOSPH1	M-phase phosphoprotein 10 (U3 small nucleolar ribonucleop	0	5	2.5

65258	MPPE1	metallophosphoesterase 1	0	5	2.5
10644	IGF2BP2	insulin-like growth factor 2 mRNA binding protein 2	0	5	2.5
948	CD36	CD36 molecule (thrombospondin receptor)	0	5	2.5
124641	OVCA2	candidate tumor suppressor in ovarian cancer 2	0	5	2.5
653082	ZDHHC11B	zinc finger, DHHC-type containing 11B	0	5	2.5
493754	LOC493754	RAB guanine nucleotide exchange factor (GEF) 1 pseudoge	0	5	2.5
387640	FLJ45187	hypothetical protein LOC387640	0	5	2.5
81876	RAB1B	RAB1B, member RAS oncogene family	0	5	2.5
5682	PSMA1	proteasome (prosome, macropain) subunit, alpha type, 1	0	5	2.5
8412	BCAR3	breast cancer anti-estrogen resistance 3	0	5	2.5
641522	LOC641522	ADP-ribosylation factor-like protein	0	5	2.5
517	ATP5G2	ATP synthase, H+ transporting, mitochondrial F0 complex, s	0	5	2.5
123879	DCUN1D3	DCN1, defective in cullin neddylation 1, domain containing 3	0	5	2.5
348162	LOC348162	hypothetical protein 348162	0	5	2.5
1743	DLST	dihydrolipoamide S-succinyltransferase (E2 component of 2-	0	5	2.5
323	APBB2	amyloid beta (A4) precursor protein-binding, family B, memb	0	5	2.5
79091	C16orf68	chromosome 16 open reading frame 68	0	5	2.5
206938	C9orf94	chromosome 9 open reading frame 94	0	5	2.5
3692	ITGB4BP	integrin beta 4 binding protein	0	5	2.5
8310	ACOX3	acyl-Coenzyme A oxidase 3, pristanoyl	0	5	2.5
143666	LOC143666	hypothetical protein LOC143666	0	5	2.5
3292	HSD17B1	hydroxysteroid (17-beta) dehydrogenase 1	0	5	2.5
1730	DIAPH2	diaphanous homolog 2 (Drosophila)	0	5	2.5
64398	MPP5	membrane protein, palmitoylated 5 (MAGUK p55 subfamily r	0	5	2.5
80279	CDK5RAP3	CDK5 regulatory subunit associated protein 3	0	5	2.5
5267	SERPINA4	serpin peptidase inhibitor, clade A (alpha-1 antiproteinase, a	0	5	2.5
7572	ZNF24	zinc finger protein 24	0	5	2.5
79853	TM4SF20	transmembrane 4 L six family member 20	0	5	2.5
55303	GIMAP4	GTPase, IMAP family member 4	0	5	2.5
51426	POLK	polymerase (DNA directed) kappa	0	5	2.5
8404	SPARCL1	SPARC-like 1 (mast9, hevin)	0	5	2.5
2100	ESR2	estrogen receptor 2 (ER beta)	0	5	2.5
23732	C9orf4	chromosome 9 open reading frame 4	0	5	2.5
1953	MEGF6	multiple EGF-like-domains 6	0	5	2.5
146198	ZFP90	zinc finger protein 90 homolog (mouse)	0	5	2.5
54957	TXNL4B	thioredoxin-like 4B	0	5	2.5
595097	SNORD16	small nucleolar RNA, C/D box 16	0	5	2.5
6835	SURF2	surfeit 2	0	5	2.5
130	ADH6	alcohol dehydrogenase 6 (class V)	0	5	2.5
23271	CAMSAP1L1	calmodulin regulated spectrin-associated protein 1-like 1	0	5	2.5
64135	IFIH1	interferon induced with helicase C domain 1	0	5	2.5
89882	TPD52L3	tumor protein D52-like 3	0	5	2.5
57463	AMIGO1	adhesion molecule with Ig-like domain 1	0	5	2.5
152189	CMTM8	CKLF-like MARVEL transmembrane domain containing 8	0	5	2.5
54881	TEX10	testis expressed sequence 10	0	5	2.5
259230	TMEM23	transmembrane protein 23	0	5	2.5
166815	TIGD2	tigger transposable element derived 2	0	5	2.5
3371	TNC	tenascin C (hexabrachion)	0	5	2.5
201294	UNC13D	unc-13 homolog D (C. elegans)	0	5	2.5
57689	LRRC4C	leucine rich repeat containing 4C	0	5	2.5
414327	PS1TP4	HBV preS1-transactivated protein 4	0	5	2.5

4361	MRE11A	MRE11 meiotic recombination 11 homolog A (S. cerevisiae)	0	5	2.5
389136	VGLL3	vestigial like 3 (Drosophila)	0	5	2.5
283294	C11orf47	chromosome 11 open reading frame 47	0	5	2.5
3157	HMGCS1	3-hydroxy-3-methylglutaryl-Coenzyme A synthase 1 (soluble	0	5	2.5
6097	RORC	RAR-related orphan receptor C	0	5	2.5
55906	KIAA1166	KIAA1166	0	5	2.5
5795	PTPRJ	protein tyrosine phosphatase, receptor type, J	0	5	2.5
3135	HLA-G	HLA-G histocompatibility antigen, class I, G	0	5	2.5
22931	RAB18	RAB18, member RAS oncogene family	0	5	2.5
79573	TTC13	tetratricopeptide repeat domain 13	0	5	2.5
57214	KIAA1199	KIAA1199	0	5	2.5
9928	KIF14	kinesin family member 14	0	5	2.5
56911	C21orf7	chromosome 21 open reading frame 7	0	5	2.5
9858	KIAA0649	KIAA0649	0	5	2.5
79709	GLT25D1	glycosyltransferase 25 domain containing 1	0	5	2.5
400707	LOC400707	hypothetical gene supported by AK124070	0	5	2.5
11281	POU6F2	POU domain, class 6, transcription factor 2	0	5	2.5
57587	KIAA1430	KIAA1430	0	5	2.5
9684	LRRC14	leucine rich repeat containing 14	0	5	2.5
55619	DOCK10	dedicator of cytokinesis 10	0	5	2.5
4643	MYO1E	myosin IE	0	5	2.5
27255	CNTN6	contactin 6	0	5	2.5
728294	D2HGDH	D-2-hydroxyglutarate dehydrogenase	0	5	2.5
23148	KIAA0363	KIAA0363 protein	0	5	2.5
128061	C1orf131	chromosome 1 open reading frame 131	0	5	2.5
54973	CPSF3L	cleavage and polyadenylation specific factor 3-like	0	5	2.5
5928	RBBP4	retinoblastoma binding protein 4	0	5	2.5
54937	SOHLH2	spermatogenesis and oogenesis specific basic helix-loop-he	0	5	2.5
202374	STK32A	serine/threonine kinase 32A	0	5	2.5
3140	MR1	major histocompatibility complex, class I-related	0	5	2.5
57335	ZNF286	zinc finger protein 286	0	5	2.5
57544	KIAA1344	KIAA1344	0	5	2.5
10513	APPBP2	amyloid beta precursor protein (cytoplasmic tail) binding prot	0	5	2.5
64168	EFCBP1	EF-hand calcium binding protein 1	0	5	2.5
6594	SMARCA1	SWI/SNF related, matrix associated, actin dependent regula	0	5	2.5
3696	ITGB8	integrin, beta 8	0	5	2.5
4795	NFKBIL1	nuclear factor of kappa light polypeptide gene enhancer in B	0	5	2.5
79919	FLJ22671	hypothetical protein FLJ22671	0	5	2.5
8737	RIPK1	receptor (TNFRSF)-interacting serine-threonine kinase 1	0	5	2.5
83891	SNX25	sorting nexin 25	0	5	2.5
55857	C20orf19	chromosome 20 open reading frame 19	0	5	2.5
23126	POGZ	pogo transposable element with ZNF domain	0	5	2.5
8673	VAMP8	vesicle-associated membrane protein 8 (endobrevin)	0	5	2.5
65249	ZSWIM4	zinc finger, SWIM-type containing 4	0	5	2.5
8227	CXYorf3	chromosome X and Y open reading frame 3	0	5	2.5
121260	SLC15A4	solute carrier family 15, member 4	0	5	2.5
146517	LOC146517	hypothetical protein LOC146517	0	5	2.5
57502	NLGN4X	neuroligin 4, X-linked	0	5	2.5
57212	KIAA0495	KIAA0495	0	5	2.5
6707	SPRR3	small proline-rich protein 3	0	5	2.5
10186	LHFP	lipoma HMGIC fusion partner	0	5	2.5

84804	MGC11332	hypothetical protein MGC11332	0	5	2.5
53344	CHIC1	cysteine-rich hydrophobic domain 1	0	5	2.5
55831	TMEM111	transmembrane protein 111	0	5	2.5
51247	PAIP2	poly(A) binding protein interacting protein 2	0	5	2.5
644914	LOC644914	similar to H3 histone, family 3B	0	5	2.5
148741	ANKRD35	ankyrin repeat domain 35	0	5	2.5
126549	ANKRD41	ankyrin repeat domain 41	0	5	2.5
79632	C6orf60	chromosome 6 open reading frame 60	0	5	2.5
256126	SYCE2	synaptonemal complex central element protein 2	0	5	2.5
4189	DNAJB9	DnaJ (Hsp40) homolog, subfamily B, member 9	0	5	2.5
10068	IL18BP	interleukin 18 binding protein	0	5	2.5
115677	NOSTRIN	nitric oxide synthase trafficker	0	5	2.5
201163	FLCN	folliculin	0	5	2.5
5314	PKHD1	polycystic kidney and hepatic disease 1 (autosomal recessiv	0	5	2.5
317	APAF1	apoptotic peptidase activating factor 1	0	5	2.5
113278	C20orf54	chromosome 20 open reading frame 54	0	5	2.5
83874	TBC1D10A	TBC1 domain family, member 10A	0	5	2.5
10418	SPON1	spondin 1, extracellular matrix protein	0	5	2.5
339210	C17orf67	chromosome 17 open reading frame 67	0	5	2.5
401491	FLJ35024	hypothetical LOC401491	0	5	2.5
3265	HRAS	v-Ha-ras Harvey rat sarcoma viral oncogene homolog	0	5	2.5
7940	LST1	leukocyte specific transcript 1	0	5	2.5
23549	DNPEP	aspartyl aminopeptidase	0	5	2.5
286122	C8orf31	chromosome 8 open reading frame 31	0	5	2.5
1012	CDH13	cadherin 13, H-cadherin (heart)	0	5	2.5
80333	KCNIP4	Kv channel interacting protein 4	0	5	2.5
387750	CCDC35	coiled-coil domain containing 35	0	5	2.5
124045	C16orf55	chromosome 16 open reading frame 55	0	5	2.5
6570	SLC18A1	solute carrier family 18 (vesicular monoamine), member 1	0	5	2.5
53938	PPIL3	peptidylprolyl isomerase (cyclophilin)-like 3	0	5	2.5
83606	C22orf13	chromosome 22 open reading frame 13	0	5	2.5
440026	TMEM41B	transmembrane protein 41B	0	5	2.5
22873	DZIP1	DAZ interacting protein 1	0	5	2.5
79065	ATG9A	ATG9 autophagy related 9 homolog A (S. cerevisiae)	0	5	2.5
10280	OPRS1	opioid receptor, sigma 1	0	5	2.5
201853	NA	NA	0	5	2.5
84656	N-PAC	cytokine-like nuclear factor n-pac	0	5	2.5
144438	LOC144438	hypothetical protein LOC144438	0	5	2.5
729396	LOC729396	similar to GAGE-4 protein (G antigen 4)	0	5	2.5
56341	PRMT8	protein arginine methyltransferase 8	0	5	2.5
283638	KIAA0284	KIAA0284	0	5	2.5
7486	WRN	Werner syndrome	0	5	2.5
121340	SP7	Sp7 transcription factor	0	5	2.5
8545	CGGBP1	CGG triplet repeat binding protein 1	0	5	2.5
114132	SIGLEC11	sialic acid binding Ig-like lectin 11	0	5	2.5
130749	CPO	carboxypeptidase O	0	5	2.5
8869	ST3GAL5	ST3 beta-galactoside alpha-2,3-sialyltransferase 5	0	5	2.5
123283	TARSL2	threonyl-tRNA synthetase-like 2	0	5	2.5
55778	C14orf131	chromosome 14 open reading frame 131	0	5	2.5
8886	DDX18	DEAD (Asp-Glu-Ala-Asp) box polypeptide 18	0	5	2.5
1731	SEP1	septin 1	0	5	2.5

2315	MLANA	melan-A	0	5	2.5
1382	CRABP2	cellular retinoic acid binding protein 2	0	5	2.5
284186	TMEM105	transmembrane protein 105	0	5	2.5
6583	SLC22A4	solute carrier family 22 (organic cation transporter), member	0	5	2.5
317671	RFESD	Rieske (Fe-S) domain containing	0	5	2.5
23305	ACSL6	acyl-CoA synthetase long-chain family member 6	0	5	2.5
149951	COMMD7	COMM domain containing 7	0	5	2.5
222643	UNC5CL	unc-5 homolog C (C. elegans)-like	2	2	2
392	ARHGAP1	Rho GTPase activating protein 1	2	2	2
401024	FLJ44048	FLJ44048 protein	2	2	2
2894	GRID1	glutamate receptor, ionotropic, delta 1	2	2	2
401147	FLJ43963	similar to hypothetical protein	2	2	2
26047	CNTNAP2	contactin associated protein-like 2	2	2	2
10869	USP19	ubiquitin specific peptidase 19	2	2	2
10980	COPS6	COP9 constitutive photomorphogenic homolog subunit 6 (Ar	2	2	2
57700	KIAA1600	KIAA1600	2	2	2
10815	CPLX1	complexin 1	2	2	2
127396	ZNF684	zinc finger protein 684	2	2	2
957	ENTPD5	ectonucleoside triphosphate diphosphohydrolase 5	2	2	2
84100	ARL6	ADP-ribosylation factor-like 6	2	2	2
55793	FAM63A	family with sequence similarity 63, member A	2	2	2
56658	TRIM39	tripartite motif-containing 39	2	2	2
116224	FAM122A	family with sequence similarity 122A	2	2	2
25853	WDR40A	WD repeat domain 40A	2	2	2
132720	C4orf32	chromosome 4 open reading frame 32	2	2	2
23765	IL17RA	interleukin 17 receptor A	2	2	2
23357	ANGEL1	angel homolog 1 (Drosophila)	2	2	2
553137	LOC553137	hypothetical LOC553137	2	2	2
79752	ZFAND1	zinc finger, AN1-type domain 1	2	2	2
80381	CD276	CD276 molecule	0	4	2
7039	TGFA	transforming growth factor, alpha	0	4	2
79966	SCD5	stearoyl-CoA desaturase 5	0	4	2
153514	C14orf81	chromosome 14 open reading frame 81	0	4	2
6000	RGS7	regulator of G-protein signalling 7	0	4	2
55331	PHCA	phytoceramidase, alkaline	0	4	2
10620	ARID3B	AT rich interactive domain 3B (BRIGHT-like)	0	4	2
91431	LOC91431	prematurely terminated mRNA decay factor-like	0	4	2
56776	FMN2	formin 2	0	4	2
54540	FLJ10404	hypothetical protein FLJ10404	0	4	2
399687	MYO18A	myosin XVIIIA	4	0	2
55846	ITFG2	integrin alpha FG-GAP repeat containing 2	4	0	2
10220	GDF11	growth differentiation factor 11	4	0	2
79924	ADM2	adrenomedullin 2	4	0	2
84240	ZCCHC9	zinc finger, CCHC domain containing 9	4	0	2
55900	ZNF302	zinc finger protein 302	4	0	2
6744	SSFA2	sperm specific antigen 2	4	0	2
63971	KIF13A	kinesin family member 13A	4	0	2
9293	GPR52	G protein-coupled receptor 52	4	0	2
8111	GPR68	G protein-coupled receptor 68	4	0	2
80131	LRRC8E	leucine rich repeat containing 8 family, member E	4	0	2
23235	SNF1LK2	SNF1-like kinase 2	4	0	2

4076	GPIAP1	GPI-anchored membrane protein 1	4	0	2
64744	SMAP1L	stromal membrane-associated protein 1-like	4	0	2
57128	LYRM4	LYR motif containing 4	4	0	2
442871	C11orf32	chromosome 11 open reading frame 32	4	0	2
55254	TMEM39A	transmembrane protein 39A	4	0	2
91392	ZNF502	zinc finger protein 502	4	0	2
728473	LOC728473	hypothetical protein LOC728473	4	0	2
115024	NT5C3L	5'-nucleotidase, cytosolic III-like	4	0	2
148345	C1orf127	chromosome 1 open reading frame 127	4	0	2
57545	KIAA1345	KIAA1345 protein	4	0	2
59338	PLEKHA1	pleckstrin homology domain containing, family A (phosphoin	4	0	2
196294	IMMP1L	IMP1 inner mitochondrial membrane peptidase-like (S. cerev	4	0	2
57602	USP36	ubiquitin specific peptidase 36	4	0	2
25841	ABTB2	ankyrin repeat and BTB (POZ) domain containing 2	4	0	2
646670	LOC646670	similar to COMM domain containing 4	4	0	2
116412	LOC116412	hypothetical protein BC012365	4	0	2
29121	CLEC2D	C-type lectin domain family 2, member D	4	0	2
115950	ZNF653	zinc finger protein 653	4	0	2
10362	HMG20B	high-mobility group 20B	4	0	2
29117	BRD7	bromodomain containing 7	4	0	2
6171	RPL41	ribosomal protein L41	4	0	2
57156	TMEM63C	transmembrane protein 63C	4	0	2
253805	LOC253805	hypothetical protein LOC253805	4	0	2
90141	C14orf143	chromosome 14 open reading frame 143	4	0	2
26015	RPAP1	RNA polymerase II associated protein 1	4	0	2
22852	ANKRD26	ankyrin repeat domain 26	4	0	2
24137	KIF4A	kinesin family member 4A	4	0	2
5110	PCMT1	protein-L-isoaspartate (D-aspartate) O-methyltransferase	4	0	2
23242	COBL	cordon-bleu homolog (mouse)	4	0	2
9793	CKAP5	cytoskeleton associated protein 5	4	0	2
2530	FUT8	fucosyltransferase 8 (alpha (1,6) fucosyltransferase)	4	0	2
9439	CRSP3	cofactor required for Sp1 transcriptional activation, subunit 3	4	0	2
51654	CDK5RAP1	CDK5 regulatory subunit associated protein 1	4	0	2
8706	B3GALNT1	beta-1,3-N-acetylgalactosaminyltransferase 1 (globoside blo	4	0	2
116843	C6orf192	chromosome 6 open reading frame 192	4	0	2
79951	FLJ11783	hypothetical protein FLJ11783	4	0	2
80303	EFHD1	EF-hand domain family, member D1	4	0	2
147276	C18orf15	chromosome 18 open reading frame 15	4	0	2
7539	ZFP37	zinc finger protein 37 homolog (mouse)	4	0	2
114801	KIAA1913	KIAA1913	4	0	2
84892	C3orf39	chromosome 3 open reading frame 39	4	0	2
5509	PPP1R3D	protein phosphatase 1, regulatory subunit 3D	4	0	2
57097	PARP11	poly (ADP-ribose) polymerase family, member 11	4	0	2
114044	MCM3APAS	MCM3 minichromosome maintenance deficient 3 (S. cerevis	4	0	2
8407	TAGLN2	transgelin 2	4	0	2
51427	ZNF588	zinc finger protein 588	4	0	2
25929	GEMIN5	gem (nuclear organelle) associated protein 5	4	0	2
652972	C9orf29	chromosome 9 open reading frame 29	4	0	2
83538	TTC25	tetratricopeptide repeat domain 25	4	0	2
439911	LOC439911	hypothetical gene supported by NM_194304	4	0	2
132989	C4orf36	chromosome 4 open reading frame 36	4	0	2

4750	NEK1	NIMA (never in mitosis gene a)-related kinase 1	4	0	2
25850	ZNF345	zinc finger protein 345	4	0	2
2972	BRF1	BRF1 homolog, subunit of RNA polymerase III transcription i	4	0	2
6627	SNRPA1	small nuclear ribonucleoprotein polypeptide A'	4	0	2
644873	FLJ33630	hypothetical protein LOC644873	4	0	2
51279	C1RL	complement component 1, r subcomponent-like	4	0	2
3566	IL4R	interleukin 4 receptor	4	0	2
1159	CKMT1B	creatine kinase, mitochondrial 1B	4	0	2
9811	KIAA0427	KIAA0427	4	0	2
114569	MAL2	mal, T-cell differentiation protein 2	4	0	2
727773	LOC727773	similar to inhibitor of growth family, member 5	4	0	2
9214	FAIM3	Fas apoptotic inhibitory molecule 3	4	0	2
27254	CSDC2	cold shock domain containing C2, RNA binding	4	0	2
58516	FAM60A	family with sequence similarity 60, member A	4	0	2
93343	FAM125A	family with sequence similarity 125, member A	4	0	2
55737	VPS35	vacuolar protein sorting 35 homolog (S. cerevisiae)	4	0	2
151258	FLJ39822	hypothetical protein FLJ39822	4	0	2
28954	REM1	RAS (RAD and GEM)-like GTP-binding 1	4	0	2
57551	TAOK1	TAO kinase 1	4	0	2
221960	C7orf28B	chromosome 7 open reading frame 28B	4	0	2
113220	KIF12	kinesin family member 12	4	0	2
2618	GART	phosphoribosylglycinamide formyltransferase, phosphoribosy	4	0	2
55233	MOBK1B	MOB1, Mps One Binder kinase activator-like 1B (yeast)	4	0	2
27332	ZNF638	zinc finger protein 638	4	0	2
114781	BTBD9	BTB (POZ) domain containing 9	4	0	2
10274	STAG1	stromal antigen 1	4	0	2
401260	FLJ41649	FLJ41649 protein	4	0	2
5004	ORM1	orosomucoid 1	4	0	2
254251	LCORL	ligand dependent nuclear receptor corepressor-like	4	0	2
7157	TP53	tumor protein p53 (Li-Fraumeni syndrome)	4	0	2
10015	PDCD6IP	programmed cell death 6 interacting protein	4	0	2
1298	COL9A2	collagen, type IX, alpha 2	4	0	2
84808	C1orf170	chromosome 1 open reading frame 170	4	0	2
340075	ARSI	arylsulfatase family, member I	4	0	2
51148	CEECAM1	cerebral endothelial cell adhesion molecule 1	4	0	2
140290	TCP10L	t-complex 10 (mouse)-like	4	0	2
140901	STK35	serine/threonine kinase 35	4	0	2
331	BIRC4	baculoviral IAP repeat-containing 4	4	0	2
10848	PPP1R13L	protein phosphatase 1, regulatory (inhibitor) subunit 13 like	4	0	2
28990	ASTE1	asteroid homolog 1 (Drosophila)	4	0	2
8727	CTNNAL1	catenin (cadherin-associated protein), alpha-like 1	4	0	2
151050	FLJ23861	hypothetical protein FLJ23861	4	0	2
23594	ORC6L	origin recognition complex, subunit 6 like (yeast)	4	0	2
26515	FXC1	fracture callus 1 homolog (rat)	4	0	2
11145	HRASLS3	HRAS-like suppressor 3	4	0	2
54455	FBXO42	F-box protein 42	4	0	2
79983	POF1B	premature ovarian failure, 1B	4	0	2
347918	EP400NL	EP400 N-terminal like	4	0	2
10523	CHERP	calcium homeostasis endoplasmic reticulum protein	4	0	2
171568	POLR3H	polymerase (RNA) III (DNA directed) polypeptide H (22.9kD)	4	0	2
3824	KLRD1	killer cell lectin-like receptor subfamily D, member 1	4	0	2

84914	ZNF587	zinc finger protein 587	4	0	2
8493	PPM1D	protein phosphatase 1D magnesium-dependent, delta isofor	4	0	2
131965	METTL6	methyltransferase like 6	4	0	2
85364	ZCCHC3	zinc finger, CCHC domain containing 3	4	0	2
149832	LOC149832	hypothetical protein LOC149832	4	0	2
79917	FLJ21687	PDZ domain containing, X chromosome	4	0	2
147798	TMC4	transmembrane channel-like 4	4	0	2
1486	CTBS	chitobiase, di-N-acetyl-	4	0	2
23002	DAAM1	dishevelled associated activator of morphogenesis 1	4	0	2
8082	SSPN	sarcospan (Kras oncogene-associated gene)	4	0	2
2525	FUT3	fucosyltransferase 3 (galactoside 3(4)-L-fucosyltransferase,	4	0	2
7385	UQCRC2	ubiquinol-cytochrome c reductase core protein II	4	0	2
143458	LDLRAD3	low density lipoprotein receptor class A domain containing 3	4	0	2
26608	TBL2	transducin (beta)-like 2	4	0	2
285172	FAM126B	family with sequence similarity 126, member B	4	0	2
55322	C5orf22	chromosome 5 open reading frame 22	4	0	2
116832	RPL39L	ribosomal protein L39-like	4	0	2
1737	DLAT	dihydrolipoamide S-acetyltransferase (E2 component of pyru	4	0	2
401898	LOC401898	similar to hypothetical protein FLJ38281	4	0	2
9866	TRIM66	tripartite motif-containing 66	4	0	2
8520	HAT1	histone acetyltransferase 1	4	0	2
10159	ATP6AP2	ATPase, H+ transporting, lysosomal accessory protein 2	4	0	2
11133	KPTN	kaptin (actin binding protein)	4	0	2
51111	SUV420H1	suppressor of variegation 4-20 homolog 1 (Drosophila)	4	0	2
1608	DGKG	diacylglycerol kinase, gamma 90kDa	4	0	2
84159	ARID5B	AT rich interactive domain 5B (MRF1-like)	4	0	2
5515	PPP2CA	protein phosphatase 2 (formerly 2A), catalytic subunit, alpha	4	0	2
1436	CSF1R	colony stimulating factor 1 receptor, formerly McDonough fel	4	0	2
645781	LOC645781	hypothetical LOC645781	4	0	2
60598	KCNK15	potassium channel, subfamily K, member 15	4	0	2
28966	SNX24	sorting nexing 24	4	0	2
284944	FLJ37357	hypothetical protein FLJ37357	4	0	2
51479	ANKFY1	ankyrin repeat and FYVE domain containing 1	4	0	2
55008	HERC6	hect domain and RLD 6	4	0	2
91612	CHURC1	churchill domain containing 1	4	0	2
643599	LOC643599	hypothetical LOC643599	4	0	2
29091	STXBP6	syntaxin binding protein 6 (amisyn)	4	0	2
54556	ING3	inhibitor of growth family, member 3	4	0	2
23780	APOL2	apolipoprotein L, 2	4	0	2
983	CDC2	cell division cycle 2, G1 to S and G2 to M	4	0	2
147991	DPY19L3	dpy-19-like 3 (C. elegans)	4	0	2
4004	LMO1	LIM domain only 1 (rhombotin 1)	4	0	2
9746	CLSTN3	calsyntenin 3	4	0	2
55208	DCUN1D2	DCN1, defective in cullin neddylation 1, domain containing 2	4	0	2
84257	C8orf57	chromosome 8 open reading frame 57	4	0	2
3720	JARID2	jumonji, AT rich interactive domain 2	4	0	2
9001	HAP1	huntingtin-associated protein 1 (neuroan 1)	4	0	2
29904	EEF2K	eukaryotic elongation factor-2 kinase	4	0	2
5345	SERPINF2	serpin peptidase inhibitor, clade F (alpha-2 antiplasmin, pigr	4	0	2
10428	CFDP1	craniofacial development protein 1	4	0	2
121838	LOC121838	hypothetical protein LOC121838	4	0	2

2108	ETFA	electron-transfer-flavoprotein, alpha polypeptide (glutaric aci	4	0	2
23345	SYNE1	spectrin repeat containing, nuclear envelope 1	4	0	2
79175	ZNF343	zinc finger protein 343	4	0	2
6175	RPLP0	ribosomal protein, large, P0	4	0	2
23607	CD2AP	CD2-associated protein	4	0	2
8997	KALRN	kalirin, RhoGEF kinase	4	0	2
7373	COL14A1	collagen, type XIV, alpha 1 (undulin)	4	0	2
148789	B3GALNT2	beta-1,3-N-acetylgalactosaminyltransferase 2	4	0	2
203859	TMEM16E	transmembrane protein 16E	4	0	2
5504	PPP1R2	protein phosphatase 1, regulatory (inhibitor) subunit 2	4	0	2
55066	PDPR	pyruvate dehydrogenase phosphatase regulatory subunit	4	0	2
375295	LOC375295	hypothetical gene supported by BC013438	4	0	2
1129	CHRM2	cholinergic receptor, muscarinic 2	4	0	2
11328	FKBP9	FK506 binding protein 9, 63 kDa	4	0	2
7016	TESK1	testis-specific kinase 1	4	0	2
322	APBB1	amyloid beta (A4) precursor protein-binding, family B, memb	4	0	2
643549	FLJ40606	hypothetical protein LOC643548	4	0	2
4983	OPHN1	oligophrenin 1	4	0	2
84695	LOXL3	lysyl oxidase-like 3	4	0	2
3426	CFI	complement factor I	4	0	2
400352	LOC400352	similar to M-phase phosphoprotein 10	4	0	2
599	BCL2L2	BCL2-like 2	4	0	2
22920	KIFAP3	kinesin-associated protein 3	4	0	2
55284	UBE2W	ubiquitin-conjugating enzyme E2W (putative)	4	0	2
23099	ZBTB43	zinc finger and BTB domain containing 43	4	0	2
79068	FTO	fatso	4	0	2
1859	DYRK1A	dual-specificity tyrosine-(Y)-phosphorylation regulated kinase	4	0	2
3709	ITPR2	inositol 1,4,5-triphosphate receptor, type 2	4	0	2
54952	TRSPAP1	tRNA selenocysteine associated protein 1	4	0	2
10404	PGCP	plasma glutamate carboxypeptidase	4	0	2
80318	GKAP1	G kinase anchoring protein 1	4	0	2
4069	LYZ	lysozyme (renal amyloidosis)	4	0	2
83475	DOHH	deoxyhypusine hydroxylase/monooxygenase	4	0	2
10613	SPFH1	SPFH domain family, member 1	4	0	2
54793	KCTD9	potassium channel tetramerisation domain containing 9	4	0	2
2247	FGF2	fibroblast growth factor 2 (basic)	4	0	2
4338	MOCS2	molybdenum cofactor synthesis 2	4	0	2
163259	DENND2C	DENN/MADD domain containing 2C	4	0	2
5624	PROC	protein C (inactivator of coagulation factors Va and VIIIa)	4	0	2
65095	FLJ12949	hypothetical protein FLJ12949	4	0	2
4636	MYL5	myosin, light chain 5, regulatory	4	0	2
23396	PIP5K1C	phosphatidylinositol-4-phosphate 5-kinase, type I, gamma	4	0	2
474170	LRRC37A2	leucine rich repeat containing 37, member A2	4	0	2
11083	DIDO1	death inducer-obliterator 1	0	3	1.5
7398	USP1	ubiquitin specific peptidase 1	0	3	1.5
727792	LOC727792	similar to ribosomal protein L31	0	3	1.5
57050	SAS10	disrupter of silencing 10	0	3	1.5
83541	C20orf55	chromosome 20 open reading frame 55	0	3	1.5
9867	PJA2	praja 2, RING-H2 motif containing	0	3	1.5
50859	SPOCK3	sparc/osteonectin, cwcv and kazal-like domains proteoglycai	0	3	1.5
23151	DIP	death-inducing-protein	0	3	1.5

348793	WDR53	WD repeat domain 53	0	3	1.5
200933	FBXO45	F-box protein 45	0	3	1.5
11215	AKAP11	A kinase (PRKA) anchor protein 11	0	3	1.5
1164	CKS2	CDC28 protein kinase regulatory subunit 2	0	3	1.5
53615	MBD3	methyl-CpG binding domain protein 3	0	3	1.5
22881	ANKRD6	ankyrin repeat domain 6	0	3	1.5
145788	FLJ27352	hypothetical LOC145788	0	3	1.5
685	BTC	betacellulin	0	3	1.5
6670	SP3	Sp3 transcription factor	0	3	1.5
23421	ITGB3BP	integrin beta 3 binding protein (beta3-endonexin)	0	3	1.5
728570	LOC728570	hypothetical protein LOC728570	0	3	1.5
6189	RPS3A	ribosomal protein S3A	0	3	1.5
3223	HOXC6	homeobox C6	0	3	1.5
283335	LOC283335	hypothetical protein LOC283335	0	3	1.5
439	ASNA1	arsA arsenite transporter, ATP-binding, homolog 1 (bacterial	0	3	1.5
10745	PHTF1	putative homeodomain transcription factor 1	0	3	1.5
58480	RHOU	ras homolog gene family, member U	0	3	1.5
23125	CAMTA2	calmodulin binding transcription activator 2	0	3	1.5
122786	FRMD6	FERM domain containing 6	0	3	1.5
27250	PDCD4	programmed cell death 4 (neoplastic transformation inhibitor	0	3	1.5
118427	OLFM3	olfactomedin 3	0	3	1.5
205	AK3L1	adenylate kinase 3-like 1	0	3	1.5
26253	CLEC4E	C-type lectin domain family 4, member E	0	3	1.5
1945	EFNA4	ephrin-A4	0	3	1.5
1501	CTNND2	catenin (cadherin-associated protein), delta 2 (neural plakop	0	3	1.5
8828	NRP2	neuropilin 2	0	3	1.5
51071	DERA	2-deoxyribose-5-phosphate aldolase homolog (C. elegans)	0	3	1.5
55789	DEPDC1B	DEP domain containing 1B	0	3	1.5
10732	TCFL5	transcription factor-like 5 (basic helix-loop-helix)	0	3	1.5
10940	POP1	processing of precursor 1, ribonuclease P/MRP subunit (S. c	0	3	1.5
55754	TMEM30A	transmembrane protein 30A	0	3	1.5
55068	RP11-301I17	proliferation-inducing protein 38	0	3	1.5
135295	SRrp35	serine-arginine repressor protein (35 kDa)	0	3	1.5
112487	C14orf126	chromosome 14 open reading frame 126	0	3	1.5
84435	GPR123	G protein-coupled receptor 123	0	3	1.5
388732	C1orf132	chromosome 1 open reading frame 132	0	3	1.5
26037	SIPA1L1	signal-induced proliferation-associated 1 like 1	0	3	1.5
9575	CLOCK	clock homolog (mouse)	0	3	1.5
11108	PRDM4	PR domain containing 4	0	3	1.5
115290	FBXO17	F-box protein 17	0	3	1.5
2244	FGB	fibrinogen beta chain	0	3	1.5
80017	C14orf159	chromosome 14 open reading frame 159	0	3	1.5
55705	IPO9	importin 9	0	3	1.5
2256	FGF11	fibroblast growth factor 11	0	3	1.5
8875	VNN2	vanin 2	0	3	1.5
64397	ZFP106	zinc finger protein 106 homolog (mouse)	0	3	1.5
10813	UTP14A	UTP14, U3 small nucleolar ribonucleoprotein, homolog A (ye	0	3	1.5
2323	FLT3LG	fms-related tyrosine kinase 3 ligand	0	3	1.5
138255	C9orf135	chromosome 9 open reading frame 135	0	3	1.5
64282	PAPD5	PAP associated domain containing 5	0	3	1.5
646467	LOC646467	similar to Calponin-2 (Calponin H2, smooth muscle) (Neutral	0	3	1.5

170691	ADAMTS17	ADAM metallopeptidase with thrombospondin type 1 motif, 1	0	3	1.5
9470	EIF4E2	eukaryotic translation initiation factor 4E family member 2	0	3	1.5
59286	UBL5	ubiquitin-like 5	0	3	1.5
85461	TANC1	tetratricopeptide repeat, ankyrin repeat and coiled-coil contai	0	3	1.5
56623	INPP5E	inositol polyphosphate-5-phosphatase, 72 kDa	0	3	1.5
729178	LOC729178	hypothetical protein LOC729178	0	3	1.5
157922	CAMSAP1	calmodulin regulated spectrin-associated protein 1	0	3	1.5
9228	DLGAP2	discs, large (Drosophila) homolog-associated protein 2	0	3	1.5
284422	LOC284422	similar to HSPC323	0	3	1.5
2847	MCHR1	melanin-concentrating hormone receptor 1	0	3	1.5
5867	RAB4A	RAB4A, member RAS oncogene family	0	3	1.5
4714	NDUFB8	NADH dehydrogenase (ubiquinone) 1 beta subcomplex, 8, 1	0	3	1.5
51634	RBMX2	RNA binding motif protein, X-linked 2	0	3	1.5
646769	NA	NA	0	3	1.5
126272	EID2B	EP300 interacting inhibitor of differentiation 2B	0	3	1.5
10795	ZNF268	zinc finger protein 268	0	3	1.5
57336	ZNF287	zinc finger protein 287	0	3	1.5
1024	CDK8	cyclin-dependent kinase 8	0	3	1.5
83478	ARHGAP24	Rho GTPase activating protein 24	0	3	1.5
23331	TTC28	tetratricopeptide repeat domain 28	0	3	1.5
643313	LOC643313	similar to hypothetical protein LOC284701	0	3	1.5
729490	LOC729490	similar to nuclear receptor co-repressor 1	0	3	1.5
79414	LRFN3	leucine rich repeat and fibronectin type III domain containing	0	3	1.5
91768	CABLES1	Cdk5 and Abl enzyme substrate 1	0	3	1.5
10067	SCAMP3	secretory carrier membrane protein 3	0	3	1.5
283392	LOC283392	hypothetical protein LOC283392	0	3	1.5
253650	FLJ35740	FLJ35740 protein	0	3	1.5
90025	C6orf157	chromosome 6 open reading frame 157	0	3	1.5
54541	DDIT4	DNA-damage-inducible transcript 4	0	3	1.5
3783	KCNN4	potassium intermediate/small conductance calcium-activatec	0	3	1.5
11319	ECD	ecdysoneless homolog (Drosophila)	0	3	1.5
79969	C6orf134	chromosome 6 open reading frame 134	0	3	1.5
2188	FANCF	Fanconi anemia, complementation group F	0	3	1.5
145694	LOC145694	hypothetical protein LOC145694	0	3	1.5
7056	THBD	thrombomodulin	0	3	1.5
285352	FLJ39534	hypothetical protein FLJ39534	0	3	1.5
83715	ESPN	espin	0	3	1.5
10056	FARSLB	phenylalanine-tRNA synthetase-like, beta subunit	0	3	1.5
81565	NDEL1	nudE nuclear distribution gene E homolog like 1 (A. nidulans	0	3	1.5
57224	NHSL1	NHS-like 1	0	3	1.5
56181	FAM54B	family with sequence similarity 54, member B	0	3	1.5
4150	MAZ	MYC-associated zinc finger protein (purine-binding transcrip	0	3	1.5
55904	MLL5	myeloid/lymphoid or mixed-lineage leukemia 5 (trithorax hon	0	3	1.5
388228	SBK1	SH3-binding domain kinase 1	0	3	1.5
54014	BRWD1	bromodomain and WD repeat domain containing 1	0	3	1.5
91975	ZNF300	zinc finger protein 300	0	3	1.5
1978	EIF4EBP1	eukaryotic translation initiation factor 4E binding protein 1	0	3	1.5
10231	DSCR1L1	Down syndrome critical region gene 1-like 1	0	3	1.5
2300	FOXL1	forkhead box L1	0	3	1.5
8665	EIF3S5	eukaryotic translation initiation factor 3, subunit 5 epsilon, 47	0	3	1.5
7462	LAT2	linker for activation of T cells family, member 2	0	3	1.5

51063	FAM26B	family with sequence similarity 26, member B	0	3	1.5
4496	MT1H	metallothionein 1H	0	3	1.5
445	ASS1	argininosuccinate synthetase 1	0	3	1.5
51807	TUBA8	tubulin, alpha 8	0	3	1.5
80128	TRIM46	tripartite motif-containing 46	0	3	1.5
8224	SYN3	synapsin III	0	3	1.5
79875	THSD4	thrombospondin, type I, domain containing 4	0	3	1.5
4241	MFI2	antigen p97 (melanoma associated) identified by monoclona	0	3	1.5
255519	LOC255519	similar to high-mobility group box 3	0	3	1.5
2701	GJA4	gap junction protein, alpha 4, 37kDa (connexin 37)	0	3	1.5
84861	KLHL22	kelch-like 22 (Drosophila)	0	3	1.5
4615	MYD88	myeloid differentiation primary response gene (88)	0	3	1.5
10861	SLC26A1	solute carrier family 26 (sulfate transporter), member 1	0	3	1.5
26102	DKFZP434A	DKFZP434A062 protein	0	3	1.5
57574	MAR4	membrane-associated ring finger (C3HC4) 4	0	3	1.5
58488	PCTP	phosphatidylcholine transfer protein	0	3	1.5
79828	METTL8	methyltransferase like 8	0	3	1.5
2081	ERN1	endoplasmic reticulum to nucleus signalling 1	0	3	1.5
401145	MGC48628	similar to KIAA1680 protein	0	3	1.5
80777	CYB5B	cytochrome b5 type B (outer mitochondrial membrane)	0	3	1.5
124739	USP43	ubiquitin specific peptidase 43	0	3	1.5
376940	ZC3H6	zinc finger CCCH-type containing 6	0	3	1.5
84444	DOT1L	DOT1-like, histone H3 methyltransferase (S. cerevisiae)	0	3	1.5
9881	LBA1	lupus brain antigen 1	0	3	1.5
126068	ZNF441	zinc finger protein 441	0	3	1.5
6741	SSB	Sjogren syndrome antigen B (autoantigen La)	0	3	1.5
729	C6	complement component 6	0	3	1.5
441140	FLJ45422	FLJ45422 protein	0	3	1.5
54585	LZTFL1	leucine zipper transcription factor-like 1	0	3	1.5
112939	BTBD14B	BTB (POZ) domain containing 14B	0	3	1.5
4776	NFATC4	nuclear factor of activated T-cells, cytoplasmic, calcineurin-d	0	3	1.5
387700	SLC16A12	solute carrier family 16, member 12 (monocarboxylic acid tra	0	3	1.5
201595	STT3B	STT3, subunit of the oligosaccharyltransferase complex, hor	0	3	1.5
2313	FLI1	Friend leukemia virus integration 1	0	3	1.5
2762	GMDS	GDP-mannose 4,6-dehydratase	0	3	1.5
23633	KPNA6	karyopherin alpha 6 (importin alpha 7)	0	3	1.5
10845	CLPX	ClpX caseinolytic peptidase X homolog (E. coli)	0	3	1.5
400644	FLJ44255	hypothetical gene supported by AK126243	0	3	1.5
220004	C11orf66	chromosome 11 open reading frame 66	0	3	1.5
11252	PACSIN2	protein kinase C and casein kinase substrate in neurons 2	0	3	1.5
166336	PRICKLE2	prickle homolog 2 (Drosophila)	0	3	1.5
1509	CTSD	cathepsin D	0	3	1.5
10426	TUBGCP3	tubulin, gamma complex associated protein 3	0	3	1.5
1147	CHUK	conserved helix-loop-helix ubiquitous kinase	0	3	1.5
55212	BBS7	Bardet-Biedl syndrome 7	0	3	1.5
7433	VIPR1	vasoactive intestinal peptide receptor 1	0	3	1.5
85465	SELI	selenoprotein I	0	3	1.5
122509	FAM14B	family with sequence similarity 14, member B	0	3	1.5
55028	C17orf80	chromosome 17 open reading frame 80	0	3	1.5
78990	OTUB2	OTU domain, ubiquitin aldehyde binding 2	0	3	1.5
6543	SLC8A2	solute carrier family 8 (sodium-calcium exchanger), member	0	3	1.5

5069	PAPPA	pregnancy-associated plasma protein A, pappalysin 1	0	3	1.5
7096	TLR1	toll-like receptor 1	0	3	1.5
1479	CSTF3	cleavage stimulation factor, 3' pre-RNA, subunit 3, 77kDa	0	3	1.5
51601	LIPT1	lipoyltransferase 1	0	3	1.5
51361	HOOK1	hook homolog 1 (Drosophila)	0	3	1.5
23085	ERC1	ELKS/RAB6-interacting/CAST family member 1	0	3	1.5
145553	MDP-1	magnesium-dependent phosphatase 1	0	3	1.5
7347	UCHL3	ubiquitin carboxyl-terminal esterase L3 (ubiquitin thiolesteras	0	3	1.5
79696	C14orf140	chromosome 14 open reading frame 140	0	3	1.5
397	ARHGDIB	Rho GDP dissociation inhibitor (GDI) beta	0	3	1.5
6492	SIM1	single-minded homolog 1 (Drosophila)	0	3	1.5
729911	LOC729911	hypothetical protein LOC729911	0	3	1.5
10424	PGRMC2	progesterone receptor membrane component 2	0	3	1.5
441073	LOC441073	similar to 60S ribosomal protein L26 (Silica-induced gene 20	0	3	1.5
100	ADA	adenosine deaminase	0	3	1.5
7639	ZNF85	zinc finger protein 85	0	3	1.5
83660	TLN2	talin 2	0	3	1.5
730026	LOC730026	hypothetical protein LOC730026	0	3	1.5
79147	FKRP	fukutin related protein	0	3	1.5
4363	ABCC1	ATP-binding cassette, sub-family C (CFTR/MRP), member 1	0	3	1.5
285025	FLJ39502	hypothetical protein FLJ39502	0	3	1.5
54921	CTF8	chromosome transmission fidelity factor 8 homolog (S. cerev	0	3	1.5
79096	C11orf49	chromosome 11 open reading frame 49	0	3	1.5
27034	ACAD8	acyl-Coenzyme A dehydrogenase family, member 8	0	3	1.5
6668	SP2	Sp2 transcription factor	0	3	1.5
64285	RHBDF1	rhomboid 5 homolog 1 (Drosophila)	0	3	1.5
341208	HEPHL1	hephaestin-like 1	0	3	1.5
1767	DNAH5	dynein, axonemal, heavy chain 5	0	3	1.5
5378	PMS1	PMS1 postmeiotic segregation increased 1 (S. cerevisiae)	0	3	1.5
2258	FGF13	fibroblast growth factor 13	0	3	1.5
10352	WARS2	tryptophanyl tRNA synthetase 2 (mitochondrial)	0	3	1.5
54660	PCDHB18	protocadherin beta 18 pseudogene	0	3	1.5
11141	IL1RAPL1	interleukin 1 receptor accessory protein-like 1	0	3	1.5
1476	CSTB	cystatin B (stefin B)	0	3	1.5
729044	LOC729044	similar to procollagen, type III, alpha 1	0	3	1.5
4548	MTR	5-methyltetrahydrofolate-homocysteine methyltransferase	0	3	1.5
80022	MYO15B	myosin XVB pseudogene	0	3	1.5
8772	FADD	Fas (TNFRSF6)-associated via death domain	0	3	1.5
10110	SGK2	serum/glucocorticoid regulated kinase 2	0	3	1.5
54454	ATAD2B	ATPase family, AAA domain containing 2B	0	3	1.5
57194	ATP10A	ATPase, Class V, type 10A	0	3	1.5
7754	ZNF204	zinc finger protein 204	0	3	1.5
83746	L3MBTL2	l(3)mbt-like 2 (Drosophila)	0	3	1.5
3174	HNF4G	hepatocyte nuclear factor 4, gamma	0	3	1.5
1795	DOCK3	dedicator of cytokinesis 3	0	3	1.5
10494	STK25	serine/threonine kinase 25 (STE20 homolog, yeast)	0	3	1.5
25988	MIZF	MBD2-interacting zinc finger	0	3	1.5
4907	NT5E	5'-nucleotidase, ecto (CD73)	0	3	1.5
146760	RTN4RL1	reticulon 4 receptor-like 1	0	3	1.5
143686	SESN3	sestrin 3	0	3	1.5
57733	GBA3	glucosidase, beta, acid 3 (cytosolic)	0	3	1.5

3066	HDAC2	histone deacetylase 2	0	3	1.5
4744	NEFH	neurofilament, heavy polypeptide 200kDa	0	3	1.5
83990	BRIP1	BRCA1 interacting protein C-terminal helicase 1	0	3	1.5
785	CACNB4	calcium channel, voltage-dependent, beta 4 subunit	0	3	1.5
64919	BCL11B	B-cell CLL/lymphoma 11B (zinc finger protein)	0	3	1.5
10361	NPM2	nucleophosmin/nucleoplasmin, 2	0	3	1.5
7082	TJP1	tight junction protein 1 (zona occludens 1)	0	3	1.5
9154	SLC28A1	solute carrier family 28 (sodium-coupled nucleoside transpor	0	3	1.5
727845	LOC727845	hypothetical protein LOC727845	0	3	1.5
3667	IRS1	insulin receptor substrate 1	0	3	1.5
2054	STX2	syntaxin 2	0	3	1.5
4211	MEIS1	Meis1, myeloid ecotropic viral integration site 1 homolog (mc	0	3	1.5
57120	GOPC	golgi associated PDZ and coiled-coil motif containing	0	3	1.5
400713	LOC400713	zinc finger-like	0	3	1.5
55164	SHQ1	SHQ1 homolog (S. cerevisiae)	0	3	1.5
9917	FAM20B	family with sequence similarity 20, member B	0	3	1.5
26873	OPLAH	5-oxoprolinase (ATP-hydrolysing)	0	3	1.5
84419	C15orf48	chromosome 15 open reading frame 48	0	3	1.5
54102	CLIC6	chloride intracellular channel 6	0	3	1.5
114227	LOC114227	hypothetical protein LOC114227	0	3	1.5
80036	TRPM3	transient receptor potential cation channel, subfamily M, mer	0	3	1.5
54753	DKFZp434J1	hypothetical protein DKFZp434J1015	0	3	1.5
54521	WDR44	WD repeat domain 44	0	3	1.5
50649	ARHGEF4	Rho guanine nucleotide exchange factor (GEF) 4	0	3	1.5
85464	SSH2	slingshot homolog 2 (Drosophila)	0	3	1.5
10667	FARS2	phenylalanine-tRNA synthetase 2 (mitochondrial)	0	3	1.5
645079	FLJ41309	hypothetical protein LOC645079	0	3	1.5
313	AOAH	acyloxyacyl hydrolase (neutrophil)	0	3	1.5
145773	FAM81A	family with sequence similarity 81, member A	0	3	1.5
357	SHROOM2	shroom family member 2	0	3	1.5
192669	EIF2C3	eukaryotic translation initiation factor 2C, 3	0	3	1.5
57415	C3orf14	chromosome 3 open reading frame 14	0	3	1.5
27000	ZRF1	zuotin related factor 1	0	3	1.5
400509	RUNDC2B	RUN domain containing 2B	0	3	1.5
154043	CNKSR3	CNKSR family member 3	0	3	1.5
64065	PERP	PERP, TP53 apoptosis effector	0	3	1.5
3206	HOXA10	homeobox A10	0	3	1.5
1075	CTSC	cathepsin C	0	3	1.5
122809	SOCS4	suppressor of cytokine signaling 4	0	3	1.5
5026	P2RX5	purinergic receptor P2X, ligand-gated ion channel, 5	0	3	1.5
401431	LOC401431	hypothetical gene LOC401431	0	3	1.5
2773	GNAI3	guanine nucleotide binding protein (G protein), alpha inhibitir	0	3	1.5
147710	LOC147710	hypothetical LOC147710	0	3	1.5
241	ALOX5AP	arachidonate 5-lipoxygenase-activating protein	0	3	1.5
55032	SLC35A5	solute carrier family 35, member A5	0	3	1.5
130535	KCTD18	potassium channel tetramerisation domain containing 18	0	3	1.5
151648	SGOL1	shugoshin-like 1 (S. pombe)	0	3	1.5
401044	FLJ40712	FLJ40712 protein	0	3	1.5
22871	NLGN1	neuroligin 1	0	3	1.5
