# Supplementary material for: Detailed transcriptome atlas of the pancreatic beta cell
Source: BMC Med Genomics. 2009 Jan 15;2:3. doi: 10.1186/1755-8794-2-3 (PMC2635377; doi:10.1186/1755-8794-2-3)
Supplement: Additional file 5 — INS-1 cell microarray data analysis results. This file contains 1. MAS5-normalized expression data of rat β, α and INS cell. 2. Differential expression analysis of genes expressed in β vs α cells. 3. Differential expression analysis of genes expressed in β vs INS cells. [file 1755-8794-2-3-S5.pdf]

Expression Matrix

Probe_id	Gene_id	Symbol	Gene description	Beta	INS	Alpha
1387815_at	24505	Ins1	insulin 1	247585	149021.6	167251
1387660_at	24476	Iapp	islet amyloid polypeptide	169338.7	94392.62	108660.4
1370077_at	24506	Ins2	insulin 2	160970.3	106058.8	114615.7
1369897_s_at	24896	Gnas	GNAS complex locus	156433.2	131697.5	123477.5
1386921_at	25669	Cpe	carboxypeptidase E	152578.8	85968.61	140125.6
1388303_at	287417	Rpl26	ribosomal protein L26	147284.8	119688.2	155016.3
1367565_a_at	25319	Fth1	ferritin, heavy polypeptide 1	137011.2	55101.14	88907.15
1370283_at	25617	Hspa5	heat shock 70kDa protein 5 (glucose-r	136527.8	69038.12	112020.1
1367573_at	29304	Rps6	ribosomal protein S6	136466.8	81085.04	93086.45
1371327_a_at	287876	Actg1	actin, gamma, cytoplasmic 1	133781.5	82872.05	62927.22
1371504_at	500547	RGD15611	RGD1561181 (predicted)	132856.1	63361.39	131010
1386852_x_at	192255	Ubb	polyubiquitin	131781.5	103657.3	125243.9
1375219_a_at	83789	Rps2	ribosomal protein S2	131758.6	103609.4	67776.44
1370242_at	124323	Rps23	ribosomal protein S23	128544.5	105875.2	137633.1
1371297_at	296596	Rpl7a_pre	ribosomal protein L7a (predicted)	125312.7	122032.6	95154.96
1367709_at	29186	Cd63	CD63 antigen	122376.7	81625.69	61699.73
1398240_at	24468	Hspa8	heat shock protein 8	117408.3	77494.35	112990.6
1371301_at	29257	Rpl9	ribosomal protein L9	116899.7	92644.72	136930.3
1371344_at	293418	Rpl27a_pre	ribosomal protein L27a (predicted)	116228.9	93182.18	104294.5
1387890_at	25348	Rps29	ribosomal protein S29	112586.3	81583.98	165294.3
1388110_at	171361	Eef1a1	eukaryotic translation elongation factor	112001.9	84083.91	99422.98
1386886_at	83689	Cd164	CD164 antigen	111162.1	62763.91	77320.7
1388310_at	287703	Sui1-rs1_p	suppressor of initiator codon mutations	109611.9	68987.7	60096.33
1368344_at	24379	Gad1	glutamic acid decarboxylase 1	108848.2	6479.979	37996.07
1369952_at	171350	Pabpc1	poly(A) binding protein, cytoplasmic 1	108243.1	69334.53	30318.24
1398835_at	81822	Actb	actin, beta	107489.7	32297.7	23046.8
1367687_a_at	25508	Pam	peptidylglycine alpha-amidating monoo	106950.4	27454.88	73100.59
1367606_at	29288	Rps3a	ribosomal protein S3a	106528.2	100738.8	119414.4
1373067_at	84353	Ctnnb1	catenin (cadherin associated protein),	106393.1	64688.12	116113.4
1367559_at	29292	Ftl1	ferritin light chain 1	105469.1	17280.53	16543.56
1367645_at	29286	Rps17	ribosomal protein S17	101540.1	83985.68	110430.2
1371307_at	140661	Rplp1	ribosomal protein, large, P1	101212.7	49124.86	82402.92
1367904_at	50561	Resp18	regulated endocrine-specific protein 18	95216.64	53240.94	117129.9
1367717_at	94266	Rps27	ribosomal protein S27	94627.85	60361.16	127542.5
1367635_at	25506	P4hb	prolyl 4-hydroxylase, beta polypeptide	93943.57	36271.32	9525.45
1367657_at	29618	Btg1	B-cell translocation gene 1, anti-prolife	93929.52	58287.64	107830.4
1398324_at	290641	MGC72957	similar to 60S ribosomal protein L18a	92940.88	93671.41	41916.27
1398761_at	81763	Rpl5	ribosomal protein L5	92657.99	81439.62	110859.1
1388331_at	362862	Tra1_predi	tumor rejection antigen gp96 (predicte	91949.73	37200.85	27070.02
1369196_at	24677	Ppy	pancreatic polypeptide	91036.93	178.4137	216410.4
1398871_at	291434	Rpl17	ribosomal protein L17	90621.2	91661.31	94093.69
1367634_at	64298	Rpl31	ribosomal protein L31	89866.71	90841.27	134973.4
1368211_at	29284	Rps14	ribosomal protein S14	89735.3	92373.47	71130.4
1367625_at	81764	Rpl10	ribosomal protein L10	89408.3	81042.22	71048.37
1398885_at	29282	Rpl23	ribosomal protein L23	88694.86	86047.13	74239.07
1371308_at	29426	Rps4x	ribosomal protein S4, X-linked	88437.1	92272.32	123404.4
1367992_at	25719	Sgne1	secretory granule neuroendocrine prot	88215.95	68113.18	141253.4
1382882_x_at	500720	NA	NA	86816.41	27.87854	28390.21
1376845_at	299269	isg12(b)	putative ISG12(b) protein	86522.74	406.3228	17076.33

1399079_at	298617	RGD13078	similar to KIAA1096 protein (predicted)	84757.72	23037.92	83683.03
1371300_at	300079	Rpl3	ribosomal protein L3	84230.82	87176.52	90557.53
1367567_at	117042	Rpl6	ribosomal protein L6	84122.87	81311.6	35555.44
1398839_at	116484	Txn1	thioredoxin 1	83738.19	88656.01	48641.92
1367583_at	116646	Tpt1	tumor protein, translationally-controlled	83324.3	30365.32	99507.97
1375181_at	24949	Syn1	synapsin I	83244.31	69292.2	55389.42
1388311_at	282585	Mrfap1	Morf4 family associated protein 1	82867.1	71260.57	96375.11
1367617_at	24189	Aldoa	aldolase A	82726.03	16249.91	12860.8
1367640_at	65139	Rps12	ribosomal protein S12	82714.54	63447.76	100349.9
1367610_at	81767	Rpl19	ribosomal protein L19	82603.33	84796.18	96125.94
1384548_at	28298	Rpl32	ribosomal protein L32	82112.7	84344.87	108966
1398789_at	81770	Rpl37	ribosomal protein L37	79722.46	55506.98	106218.7
1398852_at	81775	Rps21	ribosomal protein S21	79684.65	85694.5	119317.8
1367557_s_at	24383	Gapdh	glyceraldehyde-3-phosphate dehydrog	79421.5	82043.8	6283.598
1371353_at	113894	Sqstm1	sequestosome 1	78778.65	27297.96	18699.94
1398775_at	117053	Rps15a	ribosomal protein S15a	77832.4	62877.05	113858.9
1386864_at	24642	Pgam1	phosphoglycerate mutase 1	75730.99	44115.12	21122.99
1371295_at	122772	Rps20	ribosomal protein S20	75608.65	69954.26	77872.53
1398917_at	297755	Rpl7	ribosomal protein L7	74871.21	66403	93039.72
1386895_at	84469	Maged1	melanoma antigen, family D, 1	74453.49	35647.24	40794.04
1371340_at	140662	Rplp2	ribosomal protein, large P2	74283.77	56482.39	79499.2
1368656_at	116635	Scg3	secretogranin III	74245.28	19174.39	98472.38
1367686_at	80881	RAMP4	ribosome associated membrane protei	74183.61	34229.78	57356.14
1367596_at	27139	Rps26	ribosomal protein S26	71790.11	72777.97	98379.07
1367641_at	24786	Sod1	superoxide dismutase 1	71462.15	57616.78	56763.81
1367597_at	65136	Rps8	ribosomal protein S8	70733.52	63720.74	75163.47
1370237_at	113965	Hadhsc	L-3-hydroxyacyl-Coenzyme A dehydro	70602.82	33717.14	5277.731
1398850_at	25518	Ppia	peptidylprolyl isomerase A	70488.82	106834.3	69571.6
1369966_a_at	81776	Rps24	ribosomal protein S24	69717.49	97234.14	117678.8
1372701_at	299331	Hspca	heat shock protein 1, alpha	69688.15	57271.74	106109.9
1398757_at	25498	Npm1	nucleophosmin 1	69667.23	48377.75	42400.44
1398774_at	64640	Rpl30	ribosomal protein L30	69625.75	61940.57	102137.3
1370859_at	286906	Pdia6	protein disulfide isomerase associated	69136.63	54235.65	17919.76
1369937_at	24242	Calm1	calmodulin 1	68950.08	45461.33	61947.11
1367630_at	81774	Rps11	ribosomal protein S11	67982.96	68983.84	63730.96
1370866_at	124440	Rpl41	ribosomal protein L41	67736.85	56080.81	88533.74
1388372_at	296709	Rpl35	ribosomal protein L35	67639.91	62566.36	105440.3
1398841_at	81754	Rab1	RAB1, member RAS oncogene family	66723.81	45360.34	78329.13
1371330_at	362631	Rpl11	ribosomal protein L11	66474.99	45558.98	68841.77
1388163_at	25176	Slc25a5	solute carrier family 25 (mitochondrial	66354.24	71721.16	26892.27
1367685_at	81777	Rps27a	ribosomal protein S27a	65722.6	75963.53	95821.7
1367623_at	81766	Rpl18	ribosomal protein L18	65301.1	66628.99	38745.22
1371761_at	362041	Rpl34_pred	ribosomal protein L34 (predicted)	64907.23	42893.13	100440.3
1367561_at	64306	Rpl27	ribosomal protein L27	64345.34	66976.67	80596.49
1398626_s_at	289820	Actr2	ARP2 actin-related protein 2 homolog	64170.07	41098.24	13091.51
1398760_at	57809	Rpl35a	ribosomal protein L35a	64136.18	82204.33	86175.71
1387228_at	25351	Slc2a2	solute carrier family 2 (facilitated gluco	63870.42	62032.24	3108.503
1398766_at	25596	Rpn1	ribophorin I	63478.18	44640.96	34797.42
1367653_a_at	24551	Mdh1	malate dehydrogenase 1, NAD (solubl	63291.96	46135.03	12253.58
1371377_at	29287	Rps19	ribosomal protein S19	63116.96	66531.33	35213.51
1398830_at	64638	Rpl28	ribosomal protein L28	63000.31	48178.75	69780.32

1367595_s_at	24223	B2m	beta-2 microglobulin	62659.68	16206.88	56869.99
1371305_at	26962	Rpl8	ribosomal protein L8	62604.69	56146.72	34485.93
1398749_at	64302	Rpl4	ribosomal protein L4	62432.21	38702.04	42440.63
1398751_at	29258	Rps7	ribosomal protein S7	61833.37	72029.52	48159.22
1367854_at	24159	Acly	ATP citrate lyase	61706.63	66184.05	37998.61
1367560_at	64205	Arbp	acidic ribosomal phosphoprotein P0	61168.86	63889.9	31666.9
1370156_at	24686	Prnp	prion protein	60713.15	47448.33	82168.33
1387888_at	81772	Rps9	ribosomal protein S9	60661.39	50047.35	32230.5
1375336_at	301252	Hspcb	heat shock 90kDa protein 1, beta	59776.93	53513.9	44533.83
1370810_at	64033	Ccnd2	cyclin D2	59503	24415.28	7551.182
1369939_at	25309	Cycs	cytochrome c, somatic	59257.34	49509.43	19711.06
1371299_at	140654	Rps3	ribosomal protein S3	59247.81	64650.59	26863.84
1398880_at	192269	Sub1	SUB1 homolog (S. cerevisiae)	59154.94	54256.98	92752.38
1367620_at	114630	Atp5g3	ATP synthase, H+ transporting, mitoch	59036.36	41901.98	37295.31
1398788_at	29468	Pdia3	protein disulfide isomerase associated	58958.23	29820.27	14505.09
1368247_at	24472	Hspa1a	heat shock 70kD protein 1A	58609.23	174.7521	72455.15
1369906_s_at	246117	Mcfd2	multiple coagulation factor deficiency 2	57892.53	9560.401	13366.98
1371316_at	29752	Fau	Finkel-Biskis-Reilly murine sarcoma vi	57830.03	69231.86	43001.76
1370190_at	81504	Grb2	growth factor receptor bound protein 2	57409.82	70416.3	126208.1
1375632_at	502741	RGD15616	similar to 60S ribosomal protein L38 (p	56644.61	57746.61	73073
1398315_at	245981	Rpl15	ribosomal protein L15	56191.19	75022.02	35294.65
1387288_at	29458	Neurod1	neurogenic differentiation 1	56135.95	38837.5	32334.63
1371571_at	54226	App	amyloid beta (A4) precursor protein	55959	43707.38	49279.11
1388314_at	360704	Hmgn1	high mobility group nucleosomal bindir	55761.08	64911.45	82221.4
1370073_at	63880	Dnajc3	DnaJ (Hsp40) homolog, subfamily C, r	55151.05	4983.834	4755.716
1370277_at	245959	Slc25a3	solute carrier family 25 (mitochondrial	55112.14	61983.65	49571.51
1370229_at	64457	Ndrg4	N-myc downstream regulated gene 4	54360.67	11264.65	9785.888
1368887_at	29182	Cdh22	cadherin 22	53898.85	41.06215	6866.365
1387154_at	24604	Npy	neuropeptide Y	53764.47	53317.92	405.7194
1370246_at	50663	Calm2	calmodulin 2	53756.9	70126.2	64376.32
1367624_at	79255	Atf4	activating transcription factor 4	53574.4	16802.75	46524.88
1398872_at	161477	Rps13	ribosomal protein S13	52925.81	54901.91	76193.53
1369931_at	25630	Pkm2	pyruvate kinase, muscle	52812.19	28434.59	5524.554
1388449_at	363241	Eef1b2_pre	eukaryotic translation elongation factor	52667.3	46100.08	77527.02
1386858_at	81765	Rpl13	ribosomal protein L13	52476.53	38434.86	43552.96
1398842_at	54241	Cltc	clathrin, heavy polypeptide (Hc)	52333.38	36482.98	64320.12
1370912_at	294254	Hspa1b_m	heat shock 70kD protein 1B (mapped)	51563.25	1019.988	53489.27
1371422_at	317413	Morf4l2	mortality factor 4 like 2	50950.15	52490.49	51792.88
1371642_at	303831	Eif4a2	eukaryotic translation initiation factor 4	50847.18	40466.42	81686.34
1367646_at	64529	Ctsb	cathepsin B	50756.57	6964.013	31909.17
1388777_at	81784	Ssr3	signal sequence receptor, gamma	50425.88	26055.53	51503.96
1398882_at	25538	Rps5	ribosomal protein S5	49671.75	57347.23	35051.37
1389857_at	294067	Wbp5_pre	WW domain binding protein 5 (predicte	49629.14	57026.14	60774.36
1370253_at	81768	Rpl22	ribosomal protein L22	49582.71	40292.7	59764.53
1398879_at	290796	Tmem66	transmembrane protein 66	49243.23	18021.12	34085.92
1367588_a_at	25060	Hk3	hexokinase 3	49071.94	25638.35	86307.73
1370426_a_at	29693	Atp2a2	ATPase, Ca++ transporting, cardiac m	48931.89	24232.58	23503.51
1398767_at	50522	Ubc	ubiquitin C	48475.24	43787.53	101581.1
1369888_at	24952	Gcg	glucagon	48438.12	5216.246	180115.8
1369953_a_at	25145	Cd24	CD24 antigen	48324.18	21.09743	4122.931
1386894_at	63868	Hspd1	heat shock protein 1 (chaperonin)	48073.55	42700.56	49885.43

1388076_at	246303	Serbp1	Serpine1 mRNA binding protein 1	47471.23	35000.91	35469.44
1368044_at	24765	Scg2	secretogranin 2	47287.43	19637.32	108358.2
1372241_at	25502	Oaz1	ornithine decarboxylase antizyme 1	47060.2	70820.84	32110.6
1369879_a_at	24822	Tegt	testis enhanced gene transcript	46710.82	18747.01	5581.496
1387862_at	25577	Ywhaq	tyrosine 3-monooxygenase/tryptophan	46692.98	55614.34	50500.89
1383455_at	289352	Eprs	glutamyl-prolyl-tRNA synthetase	46483.16	6735.692	7576.369
1371387_at	303393	Cox7b	cytochrome c oxidase subunit VIIb	46304.69	49287.49	50533.18
1371318_at	140655	Rps16	ribosomal protein S16	46015.22	53124.28	50131.17
1398770_at	81769	Rpl36a	large subunit ribosomal protein L36a	46004.45	24380.33	29816.25
1389973_a_at	619346	Surf4	surfeit 4	45684.28	15988.62	5337.512
1367469_at	171362	Eif4g2	eukaryotic translation initiation factor 4	44932.62	29808.3	54277.2
1386874_at	29285	Rps15	ribosomal protein S15	44832.11	54227.45	40199.99
1371386_at	291019	RGD13066	similar to Protein C9orf10 (predicted)	44278.56	41714.02	37593.62
1371688_at	312903	Tram1	translocation associated membrane pr	44169.55	31826.58	25584.79
1371843_at	361315	Yipf5	Yip1 domain family, member 5	43973.15	23383.89	21178.45
1370080_at	24451	Hmox1	heme oxygenase (decycling) 1	43852.46	612.6343	5620.417
1388318_at	24644	Pgk1	phosphoglycerate kinase 1	43790.45	28972.78	13556.8
1371375_at	502674	Dstn	NA	43500.34	40880.77	50353.38
1367459_at	64310	Arf1	ADP-ribosylation factor 1	43446.54	62554.63	12650.21
1370238_at	171069	Usmg5	upregulated during skeletal muscle grc	43328.27	81214.42	62903.3
1367569_at	29236	Rpsa	ribosomal protein SA	43289.67	35182.45	21471.66
1371507_at	296311	RGD13116	similar to 4921517L17Rik protein	42991.03	29829.44	41203.77
1398870_at	266601	Tomm20	translocase of outer mitochondrial mer	42928.76	19335.38	49775.55
1367618_a_at	83427	Gnb2l1	guanine nucleotide binding protein (G	42632.33	47605.71	38033.02
1368505_at	29480	Rgs4	regulator of G-protein signaling 4	42410.58	36.75228	27891.61
1367575_at	24333	Eno1	enolase 1, alpha	42183.63	47466.97	4079.525
1371320_at	290364	Itm2b	integral membrane protein 2B	42004.75	31497.19	75704.84
1375637_at	364154	RGD13111	similar to RIKEN cDNA 1110003E01	41828.95	10467.67	8023.838
1371427_at	290555	NA	NA	41822.74	45178.6	43851.22
1367582_at	29283	Rpl29	ribosomal protein L29	41667.99	40454.74	34395.06
1380191_s_at	140922	Txnl1	thioredoxin-like 1	41387.2	26146.52	41279.9
1388071_x_at	24737	RT1-Aw2	RT1 class Ib, locus Aw2	41072.59	3831.716	12605.82
1367590_at	84509	Ran	RAN, member RAS oncogene family	40783.51	56012.01	27673.74
1370803_at	257644	Zwint	ZW10 interactor	40556.42	50709.3	53125.23
1371573_at	292964	Rpl36a_pre	ribosomal protein L36a (predicted)	40264.13	33690.47	32555.07
1367619_at	291948	Pgrmc1	progesterone receptor membrane com	40218.87	31726.54	57529.35
1367455_at	116643	Vcp	valosin-containing protein	40175.11	29873.2	8768.62
1372352_at	315989	Armet_pre	arginine-rich, mutated in early stage tu	40078.62	12387.61	7135.732
1387599_a_at	24314	Nqo1	NAD(P)H dehydrogenase, quinone 1	40014.57	1458.571	365.6112
1369933_at	83531	Vdac2	voltage-dependent anion channel 2	39962.83	49411.04	25417.72
1367814_at	25650	Atp1b1	ATPase, Na+/K+ transporting, beta 1 p	39553.55	19011.54	15061.99
1388297_at	293725	Eef1g	eukaryotic translation elongation factor	39199.54	63866.19	30115.55
1370275_at	171374	Atp5b	ATP synthase, H+ transporting, mitoch	39189.78	43320.67	21373.81
1370000_at	59295	Nucb2	nucleobindin 2	39170.1	31248.03	44838.51
1367607_at	29445	Cox4i1	cytochrome c oxidase subunit IV isofor	39108.29	45279.87	26231.04
1379255_at	302526	Atp6ap2	ATPase, H+ transporting, lysosomal ac	38998.73	13893.8	22739.92
1387805_at	84480	Bnip3	BCL2/adenovirus E1B 19 kDa-interact	38964.44	11674.13	11019.43
1393138_at	24518	Jund	Jun D proto-oncogene	38955.98	17736.04	64252.26
1387155_at	25121	Pcsk2	proprotein convertase subtilisin/kexin t	38916.13	31482.87	55197.03
1388236_x_at	309600	RT1-CE12	RT1 class I, CE12	38873	961.2716	10610.79
1369242_at	25509	Pax6	paired box gene 6	38869.84	25315.94	54780.63

1371313_at	501709	NA	NA	38785.61	43614.05	91888.66
1373090_at	361233	Ssr1	signal sequence receptor, alpha	38673.4	25973.53	9028.28
1375119_at	25489	Nedd4a	neural precursor cell expressed, devel	38587.02	18317.61	7015.324
1398787_at	26954	Rheb	Ras homolog enriched in brain	38534.52	34822.81	47708.48
1367637_a_at	24669	Ppp1cc	protein phosphatase 1, catalytic subun	38400.91	41312.05	61447.27
1370010_at	24944	Lamp2	lysosomal membrane glycoprotein 2	38379.48	24877.05	32299.59
1373650_at	312826	Cmas	cytidine monophospho-N-acetylneuran	38350.76	23400.73	10726.52
1374426_at	362897	Uqcrb_pred	ubiquinol-cytochrome c reductase binc	38235.89	52788.36	49258.54
1373862_at	252889	Tmed7	transmembrane emp24 protein transpo	38050.49	21916.14	27137.88
1398759_at	25564	Tgfb1i4	transforming growth factor beta 1 indu	37987.19	14617.3	57557.15
1388369_at	361207	Tmed9	transmembrane emp24 protein transpo	37973.36	24528.9	10203.85
1370003_at	29565	Eef2	eukaryotic translation elongation factor	37854.45	33295.35	15606.79
1398855_at	171375	Atp5f1	ATP synthase, H+ transporting, mitoch	37826.88	45354.38	28640.21
1371783_at	290549	LOC29054	selenoprotein K	37498.44	15829.23	34262.17
1373365_at	298410	Cmpk	cytidylate kinase	37410.48	39708.88	32510.47
1387073_at	25012	Snap25	synaptosomal-associated protein 25	37381.68	25905.47	39923.27
1370213_at	500538	Ybx1	Y box protein 1	37233.39	43816.52	19399.63
1398831_at	58854	Psmb4	proteasome (prosome, macropain) sub	37181.35	45370.29	31809.14
1370442_at	286978	Tmsbl1	thymosin beta-like protein 1	37157.84	42729.33	115792.4
1370362_at	116660	Ptprn	protein tyrosine phosphatase, receptor	37005.72	7203.088	13492.36
1374712_at	303353	Psmd11_p	proteasome (prosome, macropain) 26S	36861.67	28007.6	9909.768
1399057_at	300891	Morf4l1	mortality factor 4 like 1	36608.38	24509.78	23631.47
1371583_at	114488	Rbm3	RNA binding motif protein 3	36574.86	39778.35	39874.19
1398797_at	117282	Hnrpk	heterogeneous nuclear ribonucleoprote	36467.9	37494.89	81698.78
1388519_at	298068	Sec61b_pr	Sec61 beta subunit (predicted)	36435.17	32734.78	24121.05
1371312_at	316643	Chchd2	coiled-coil-helix-coiled-coil-helix domai	36367.68	43857.84	26609.41
1399012_at	298308	RGD13103	similar to RIKEN cDNA 1110001M20 (	36362.69	47652.66	46190.96
1390048_at	302969	Srrm2_pred	serine/arginine repetitive matrix 2 (pred	36335.95	15107.59	22903.05
1371407_at	58823	Nckap1	NCK-associated protein 1	36324.51	23681.16	41190.98
1367599_at	29754	Atp5g1	ATP synthase, H+ transporting, mitoch	36292.03	48159.61	5381.919
1367603_at	24849	Tpi1	triosephosphate isomerase 1	36243.96	10934.28	4093.592
1398776_at	64701	Rpn2	ribophorin II	36211.9	33291.56	8004.406
1371435_at	288770	Naca_pred	nascent-polypeptide-associated compl	36082.43	48801.25	45597.22
1398846_at	501511	RGD15622	similar to Eukaryotic translation initiatio	35933.95	25177.04	13006.24
1371921_at	307505	Catna1	catenin (cadherin-associated protein),	35914.92	33462.82	24811.79
1389940_at	116455	Atp6v0a2_	ATPase, H+ transporting, lysosomal V	35742.62	32463	8917.865
1371837_at	287765	Ddx5	ddx5 gene	35669.79	42972.57	88529.39
1368441_at	60333	Msln	mesothelin	35507.2	172.935	10430.38
1372330_at	652955	LOC65295	goliath	35343.9	25020.71	50134.99
1367764_at	25405	Ccng1	cyclin G1	35259.95	18995.88	15255.35
1398758_at	79120	Arf4	ADP-ribosylation factor 4	35185.3	27458.81	26827.94
1398768_at	83712	Rbbp7	retinoblastoma binding protein 7	35180.16	48827.95	50869.44
1386902_at	83532	Vdac3	voltage-dependent anion channel 3	34924.63	52823.05	18029.26
1371989_at	113990	Hmgn3	high mobility group nucleosomal bindir	34749.49	36006.41	89034.23
1388377_at	364050	RGD13085	similar to expressed sequence AA408	34413.88	16214.09	23448.54
1377016_at	362978	Creld2	cysteine-rich with EGF-like domains 2	34320.81	7142.08	8032.476
1371838_at	494445	Sfrs2	similar to splicing factor, arginine/serin	34024.66	26953.97	45563.34
1386861_at	58940	H2afz	H2A histone family, member Z	33781.22	53485.16	55931.22
1367460_at	29662	Gdi2	GDP dissociation inhibitor 2	33732.71	36739.68	28027.74
1369502_a_at	497039	Amy2	NA	33718.55	10.81737	94023.38
1369930_at	29673	Psma6	proteasome (prosome, macropain) sub	33412.94	30377.43	27419.82

1367580_at	81729	Rpl10a	ribosomal protein L10A	33399.09	43423.59	18819.63
1398754_at	64156	Uba52	ubiquitin A-52 residue ribosomal protei	33375	43051.05	41960.88
1370384_a_at	24684	Prlr	prolactin receptor	33355.33	18379.15	1415.629
1371372_at	362809	NA	NA	33350.3	33434.59	40345.57
1386868_at	81773	Rps10	ribosomal protein S10	33163.48	43179.67	25919.44
1370804_at	58974	Gabarap	gamma-aminobutyric acid receptor ass	33080.97	20347.28	11679.95
1367613_at	117254	Prdx1	peroxiredoxin 1	32952.46	43977.31	14521.36
1382118_at	619476	Apeg3	antisense paternally expressed gene 3	32909.69	10097.05	6919.043
1398843_at	58857	Vapa	vesicle-associated membrane protein,	32736.19	19679.29	54634.15
1386866_at	56010	Ywhag	tyrosine 3-monooxygenase/tryptophan	32708.66	21237	35142.95
1371977_at	288669	Arpc3_pred	actin related protein 2/3 complex, subu	32678.45	40287.25	16935.48
1367629_at	29507	Cox7a2	cytochrome c oxidase, subunit VIIa 2	32638.66	45664.31	44799.73
1389023_at	363160	RGD13115	similar to Oligosaccharyl transferase 3	32439.41	15712.75	19521.88
1367724_a_at	94170	Atp6v0e1	ATPase, H+ transporting, V0 subunit E	32367.53	21135.24	12613.6
1398753_at	78959	Akr1a1	aldo-keto reductase family 1, member	32174.69	38904.74	33827.46
1367757_at	54322	Cox6c	cytochrome c oxidase, subunit VIc	32136.47	62481.27	45807.74
1383073_at	291796	Usp14	ubiquitin specific protease 14	32126.62	30328.68	18923.39
1371530_at	25626	Krt2-8	keratin complex 2, basic, gene 8	32126.05	43912.18	23021.26
1387915_at	286900	Sels	selenoprotein S	32104.81	17761.27	10499.44
1372242_at	317335	Ddx3x	DEAD/H (Asp-Glu-Ala-Asp/His) box po	32037.36	34388.09	70680.72
1371686_at	29144	Canx	calnexin	31895.06	21445.72	30265.87
1398827_at	25621	Cd81	CD 81 antigen	31878.84	37369.95	23950.14
1398618_s_at	364899	RGD13067	similar to hypothetical protein FLJ3274	31739.87	21628.75	16998.57
1393263_at	362256	RGD15639	RGD1563912 (predicted)	31713.26	103.6397	19346.77
1387235_at	24258	Chga	chromogranin A	31697.46	62612.36	43687.46
1398364_at	362626	RGD13595	similar to chromosome 1 open reading	31623.05	8407.225	61240.89
1367711_at	25581	Psmc2	proteasome (prosome, macropain) 26S	31259.95	39942.59	24442.32
1375877_at	64440	Syt4	synaptotagmin IV	31227.31	5082.539	18751.76
1370284_at	245958	Atp5e	ATP synthase, H+ transporting, mitoch	31008.86	51192.97	28224.9
1388444_at	304766	Ubxd2	UBX domain containing 2	30945.08	23164.16	15361.66
1370825_a_at	64465	Cdc42	cell division cycle 42 homolog (S. cere	30866.27	30864.85	32864.35
1387970_at	192208	Slc38a5	solute carrier family 38, member 5	30858.96	548.7886	17377.03
1374397_at	296302	Eif2s2	eukaryotic translation initiation factor 2	30823.65	26589.16	18717.01
1398796_at	84599	Tmed10	transmembrane emp24-like trafficking	30687.41	12494.1	6197.427
1398762_at	83841	Sdcbp	syndecan binding protein	30586.15	48918.19	82577.54
1371332_at	364716	Hist1h4a_p	histone 1, H4a (predicted)	30075.42	11128.65	28032.83
1377615_at	304280	LOC30428	similar to hypothetical protein	30039.34	19514.46	17661.29
1367549_a_at	314633	Ap3d1	adaptor-related protein complex 3, delt	30033.56	25731.19	37071.46
1388715_at	297113	Gars	glycyl-tRNA synthetase	29910.58	27549.85	4908.159
1398755_at	170667	Atp6v0c	ATPase, H transporting, lysosomal V0	29830.47	29118.47	16124.94
1386898_at	25462	Hspe1	heat shock 10 kDa protein 1 (chaperon	29743.39	35287.48	49671.67
1388325_at	299159	Atp6v1d	ATPase, H+ transporting, V1 subunit D	29666.1	12001.41	11538.47
1367512_at	297995	Chmp5	chromatin modifying protein 5	29590.07	27222.31	36781.22
1370817_at	266758	Sec11l3	Sec11-like 3 (S. cerevisiae)	29373.01	22523.87	9708.777
1371780_at	304290	Kdelr2	KDEL (Lys-Asp-Glu-Leu) endoplasmic	29242.02	25665.98	13178.82
1367668_a_at	83792	Scd2	stearoyl-Coenzyme A desaturase 2	29169.98	40794.65	14028.24
1368826_at	24267	Comt	catechol-O-methyltransferase	29132.67	2661.938	6797.437
1392681_at	65158	Rab2	RAB2, member RAS oncogene family	28917.75	39089.18	34620.24
1367476_at	296076	Srp14_pred	signal recognition particle 14 (predicte	28782.2	27008.88	30242.04
1388857_at	362226	Sec23b_pr	SEC23B (S. cerevisiae) (predicted)	28738.95	13863.2	13745.67
1370243_a_at	29222	Ptma	prothymosin alpha	28641.94	19503.7	11925.18

1368141_at	64530	Cnbp1	cellular nucleic acid binding protein 1	28430.03	43696.09	28487.91
1371381_at	306117	RGD1561€	similar to TBC1 domain family membe	28293.24	28085.48	23761.92
1371352_at	114637	Hmgn2	high mobility group nucleosomal bindi	28253.04	59143.54	56885.49
1398832_at	25135	Ncl	nucleolin	28180.07	13615.79	23146.2
1367766_at	83782	Nme2	expressed in non-metastatic cells 2	28177.77	30178.43	8201.102
1367784_a_at	24854	Clu	clusterin	28139.08	2337.718	86233.3
1388199_at	171577	Tacstd1	tumor-associated calcium signal trans	28039.23	20378.44	48580.45
1388861_at	316916	RGD1307€	similar to cisplatin resistance related p	27986.93	15301.9	8006.249
1367622_at	641434	Atp5h	ATP synthase, H+ transporting, mitoch	27944.48	52312.24	21449.24
1371505_at	290046	Hnrpc	heterogeneous nuclear ribonucleoprot	27919.74	21292.14	24654.8
1369934_at	64367	Ppib	peptidylprolyl isomerase B	27876.8	19349.24	5698.185
1371368_at	80843	Sec61a1	Sec61 alpha 1 subunit (S. cerevisiae)	27851.74	14232.52	5026.608
1371304_a_at	304447	RGD1559€	similar to myosin, light polypeptide 6, a	27814.39	17730.03	17813.6
1367586_at	24533	Ldha	lactate dehydrogenase A	27794.76	84.52685	857.4725
1388375_at	25725	Prkar1a	protein kinase, cAMP dependent regul	27769.7	39244.47	61944.2
1367579_a_at	300218	Tuba6	tubulin, alpha 6	27751.63	81723.9	19355.95
1398849_at	117056	H3f3b	H3 histone, family 3B	27615.48	61103.8	49550.95
1398764_at	79449	Rpl21	ribosomal protein L21	27267.42	31270.44	59784.94
1372437_at	287280	Skp1a	S-phase kinase-associated protein 1A	27258.34	40670.81	20126.02
1373044_at	295975	RGD1565€	similar to Dendritic cell protein GA17 (|	27102.2	35137.36	51912.98
1367762_at	24797	Sst	somatostatin	26840.35	123.3119	158421.3
1388898_at	288444	Hsph1	heat shock 105kDa/110kDa protein 1	26801.92	6237.999	41270.48
1368541_at	114511	Emb	embigin	26332.87	19274.68	10385.23
1371249_at	289754	Xbp1	X-box binding protein 1	26188.49	15722.17	16590.81
1367783_at	64670	Gabarapl2	GABA(A) receptor-associated protein l	26156.31	16552.72	32868.49
1371478_at	296315	RGD13077	similar to RIKEN cDNA 1110008F13	26153.54	16866.58	13306.3
1398845_at	56783	Eif5	eukaryotic translation initiation factor 5	26046.3	22580.77	41277.88
1388446_at	498696	RGD1563€	similar to heterogeneous nuclear ribon	26019.32	52346.77	28437.93
1368288_at	24384	Gc	group specific component	26018.23	54827.8	132015.5
1388622_at	501161	RGD1563€	similar to Nol5a_predicted protein (pre	26001.75	13913.08	8522.07
1386004_s_at	309220	LOC30922	NA	25994.73	24111.56	33992.34
1367465_at	192275	Dad1	defender against cell death 1	25933.86	6413.906	7067.751
1387887_at	65043	Rpl14	ribosomal protein L14	25886.67	25477.91	29641.85
1388351_at	288455	RGD1305€	similar to chromosome 13 open readin	25823.71	41707.12	11916.58
1374427_at	80977	Syt13	synaptotagmin XIII	25747.64	11491.54	13233.03
1370871_at	288146	RGD15611	hypothetical gene supported by Y1664	25698.64	30431.62	32681.53
1368034_at	24259	Chgb	chromogranin B	25683.85	81549.21	108145.6
1371417_at	497902	Qpc	low molecular mass ubiquinone-bindin	25663.67	39897.96	14569.87
1370163_at	24609	Odc1	ornithine decarboxylase 1	25649.81	14535.6	23735.88
1399090_at	252902	Dync1li1	dynein cytoplasmic 1 light intermediate	25634.44	20722.87	18242.33
1388113_at	171335	Cox8a	cytochrome c oxidase, subunit VIIIa	25626.36	48588.52	14380.02
1372443_at	292462	Lrp11_pre	low density lipoprotein receptor-relatec	25522.47	17835.42	26006.42
1371343_at	315548	Srpr	signal recognition particle receptor ('dc	25514.87	11380.33	11998.92
1388304_at	294964	Ndufb5_pr	NADH dehydrogenase (ubiquinone) 1	25425.04	29814.61	12723.21
1388320_at	292770	Spint2	serine protease inhibitor, Kunitz type 2	25394.88	20102.71	30850.67
1376654_at	310358	RGD1308€	similar to RIKEN cDNA B130016O10 g	25345.79	19777.63	3508.067
1398778_at	29668	Psma1	proteasome (prosome, macropain) sub	25163.29	22210.41	14185.2
1371321_at	502592	RGD15652	similar to Cytochrome c oxidase polyp	25151.3	30463.96	16473.48
1367666_at	140931	Hnrph1	heterogeneous nuclear ribonucleoprot	25043.6	25047.65	58331.36
1370193_at	29463	Ptp4a1	protein tyrosine phosphatase 4a1	25026.8	12653.68	95730.14
1398812_at	94198	Psmb1	proteasome (prosome, macropain) sub	24927.16	41098.99	10741.58

1375633_at	406864	Clic1	chloride intracellular channel 1	24833.61	17257.5	14883.89
1367979_s_at	25427	Cyp51	cytochrome P450, subfamily 51	24802.05	29280.07	20809.37
1373093_at	313729	Errfi1	ERBB receptor feedback inhibitor 1	24714.48	5120.974	30243.62
1368504_at	25328	Lamp1	lysosomal membrane glycoprotein 1	24697.15	11907.52	15736.68
1387116_at	24908	Dnajb9	DnaJ (Hsp40) homolog, subfamily B, n	24662.71	2607.001	43045.87
1371514_at	361577	Kdelr1	KDEL (Lys-Asp-Glu-Leu) endoplasmic	24644.29	11424.58	7069.009
1374385_at	500242	RGD15613	similar to Pol(yrC)-binding protein 1 (A	24620.84	21804.08	26396.71
1370751_at	257642	LOC25764	rRNA promoter binding protein	24611.11	11519.83	13735.16
1368266_at	29221	Arg1	arginase 1	24592.04	33232.34	14463.77
1371360_at	299923	Ndrg1	N-myc downstream regulated gene 1	24507.74	25976.44	38137.61
1399132_at	301555	Cul3_predi	cullin 3 (predicted)	24480.1	13925.15	28273.36
1374459_at	313231	Alg2	asparagine-linked glycosylation 2 hom	24456.64	10663.72	11037.95
1398752_at	113922	15-Sep	selenoprotein	24392.7	16237.2	25460.21
1370224_at	25125	Stat3	signal transducer and activator of trans	24352.79	9521.838	8989.949
1377630_at	366602	Tm4sf13	transmembrane 4 superfamily member	24292.5	11406.83	26791.03
1375705_at	24400	Gnb1	guanine nucleotide binding protein, be	24288.78	44397.46	12881.62
1398750_at	64202	Calr	calreticulin	24148.62	14208.46	6689.013
1389343_at	362641	RGD15601	similar to Hypothetical UPF0327 protei	24139.51	17626.97	21386.02
1370888_at	252934	Cox5a	cytochrome c oxidase, subunit Va	24103.4	25784.86	9484.415
1372704_at	362040	RGD15607	similar to RIKEN cDNA 2310008M10 (	23984.69	9551.003	6598.933
1367730_at	56042	Vdp	vesicle docking protein	23981.2	9304.285	9632.646
1371302_at	502663	LOC50266	NA	23974.17	19358.55	9809.222
1398790_at	24672	Ppp2ca	protein phosphatase 2 (formerly 2A), c	23961.02	25423.89	29551.32
1372077_at	297699	Strap	serine/threonine kinase receptor assoc	23947.99	21947.29	22832.55
1398854_at	64307	Rpl24	ribosomal protein L24	23751.4	42329.85	51710.42
1398296_at	60418	Mir16	membrane interacting protein of RGS1	23624.2	11641.62	14332.4
1398859_at	64474	Hdlbp	high density lipoprotein binding protein	23559.88	3841.704	4641.604
1377995_at	360502	RGD13114	similar to hypothetical protein DKFZp7	23469.09	40321.22	5265.845
1368438_at	63885	Pde10a	phosphodiesterase 10A	23402.2	5047.15	1960.887
1367800_at	25692	Plat	plasminogen activator, tissue	23350.71	249.3638	6400.684
1371357_at	289560	Igfbp7	insulin-like growth factor binding protei	23262.4	8610.259	86149.09
1367472_at	314432	LOC31443	similar to ubiquitin-protein ligase (EC 6	23171.6	20444.18	9916.276
1370918_a_at	116550	Atp5c1	ATP synthase, H+ transporting, mitoch	23030.56	47835.44	33783.2
1371335_at	300677	MGC72942	similar to CG6105-PA	22968.43	29270.29	23569.34
1367470_at	361842	Sar1a	SAR1 gene homolog A (S. cerevisiae)	22911.89	16681.08	20317.94
1371415_at	366448	Uqcrh	ubiquinol-cytochrome c reductase hing	22900.56	25036.25	28224.73
1367690_at	29435	Ssr4	signal sequence receptor 4	22883.5	36094.35	10352.38
1367456_at	81920	Ube2d3	ubiquitin-conjugating enzyme E2D 3 (U	22871.42	16674.98	31575.71
1387775_at	83828	Gtf2a2	general transcription factor IIa 2	22847	13944.49	34799.35
1399063_at	313658	Rbaf600	ZUBR1	22765.58	3284.977	4510.058
1389140_at	293142	Spcs2_pre	signal peptidase complex subunit 2 ho	22747.89	18779.89	7860.338
1371973_at	299872	Eif3s6	eukaryotic translation initiation factor 3	22637.83	41447.84	33490.05
1398819_at	65028	Dnaja1	DnaJ (Hsp40) homolog, subfamily A, n	22566.64	35808.97	69316.19
1376589_at	171064	Igsf6	immunoglobulin superfamily, member (	22536.9	24136.88	27711.65
1368819_at	24511	Itgb1	integrin beta 1 (fibronectin receptor be	22429.02	6632.36	21470.93
1388119_at	362152	Hnrpa3	heterogeneous nuclear ribonucleoprote	22384.33	25330.43	19784.36
1371237_a_at	24567	Mt1a	metallothionein 1a	22356.89	6867.152	86866.26
1367808_at	64372	Timm8b	translocase of inner mitochondrial mer	22276.12	10956.49	12003.72
1388307_at	294421	Serinc1	serine incorporator 1	22247.31	18868.27	50339.73
1369628_at	117556	Sv2b	synaptic vesicle glycoprotein 2b	22227.32	3600.24	59182.76
1371329_at	287444	Eif5a	eukaryotic translation initiation factor 5	22209.68	29441.85	5807.458

1388378_at	293484	Eif3s8	eukaryotic translation initiation factor 3	22194.18	17902.31	9134.355
1387797_at	29448	Rab7	RAB7, member RAS oncogene family	22177.16	18966.22	28317.15
1372131_at	317396	Ubqln2_pre	ubiquilin 2 (predicted)	22154.86	19088.12	46989.27
1368049_at	24818	Tcp1	t-complex protein 1	22128.61	27334.02	16280.22
1371644_at	315265	Ptk9	protein tyrosine kinase 9	22058.49	13933.49	12538.3
1371656_at	29374	Cct4	chaperonin subunit 4 (delta)	21962.62	33377.19	11703.1
1390022_at	360854	Arpc5	actin related protein 2/3 complex, subu	21958.26	18465.34	18664.38
1372067_at	362751	Txndc1	thioredoxin domain containing 1	21815.46	10675.23	15960.75
1387780_at	84026	Dnaja2	DnaJ (Hsp40) homolog, subfamily A, m	21743.76	13477.3	15570.53
1398893_at	291609	Ndfip1	Nedd4 family interacting protein 1	21741.65	27450.76	32351.61
1367706_at	83529	Vdac1	voltage-dependent anion channel 1	21739.92	22063.8	6764.888
1373041_at	301427	Ndufb3_pre	NADH dehydrogenase (ubiquinone) 1	21737.43	18321.08	18663.25
1367938_at	83472	Ugdh	UDP-glucose dehydrogenase	21734.13	5019.203	7598.628
1388770_at	365797	RGD13048	similar to RIKEN cDNA 1810045K17	21728.36	15503.07	11351.57
1373363_at	29456	Map1b	microtubule-associated protein 1b	21713.29	18390.33	17264.36
1388680_at	302499	C1galt1c1	C1GALT1-specific chaperone 1	21666.62	21494.88	18043.87
1388413_at	311483	Rrbp1_pre	ribosome binding protein 1 homolog 18	21631.71	5183.225	5406.162
1368151_at	29150	Matr3	matrin 3	21544.33	26772.89	44475.66
1372183_at	288064	Kpna1	karyopherin (importin) alpha 1	21516.54	19317.83	19346.71
1373013_at	361309	LOC36130	similar to polyadenylate-binding protei	21487.38	19176.94	41450.1
1368477_at	25391	Atp2a3	ATPase, Ca++ transporting, ubiquitous	21458.66	11330.23	11134.67
1371574_at	290596	Ghitm	growth hormone inducible transmembr	21453.75	13281.86	6594.803
1369948_at	117089	Ngfrap1	nerve growth factor receptor (TNFRSF	21422.37	53955.79	54506.33
1367694_at	171155	Hadhb	hydroxyacyl-Coenzyme A dehydrogena	21338.49	17757.88	15115.31
1369074_at	170573	Slc38a4	solute carrier family 38, member 4	21298.67	6124.14	5236.507
1371710_at	312828	Etnk1_pred	ethanolamine kinase 1 (predicted)	21282.79	8585.351	16180.52
1372333_at	360844	Snrpe_pred	small nuclear ribonucleoprotein E (pre	21238.18	27032.81	37403.07
1371869_at	29674	Psma7	proteasome (prosome, macropain) sub	21234.96	44660.93	8963.651
1398860_at	25490	Nedd8	neural precursor cell expressed, devel	21225.03	24603.25	11033.33
1388155_at	294853	Krt1-18	keratin complex 1, acidic, gene 18	21211.53	668.4305	24958.21
1367461_at	114023	Copb1	coatomer protein complex, subunit bet	21206.57	14746.49	14238.21
1387340_at	140945	Rtn3	reticulon 3	21202.38	30880.56	5974.183
1373206_at	294925	Fndc3b_pre	fibronectin type III domain containing 3	21197.33	6554.169	17763.91
1388612_at	289590	Ociad1	OCIA domain containing 1	21196.66	18509.86	15878.78
1368433_at	116482	Sacm1l	SAC1 (suppressor of actin mutations 1	21180.8	6989.617	12019.57
1367454_at	60384	Copb2	coatomer protein complex, subunit bet	20974.3	9589.655	19034.67
1383361_at	362293	Dnajb6	DnaJ (Hsp40) homolog, subfamily B, m	20973.65	26439.35	36231.92
1371905_at	288616	MGC94190	similar to 0610007L01Rik protein	20971.87	18474.91	13970.95
1371595_at	85242	Pola2	polymerase (DNA directed), alpha 2	20918.8	370.7864	3410.756
1369410_at	94189	Gosr1	golgi SNAP receptor complex member	20894.42	6151.209	6727.515
1398856_at	29669	Psma2	proteasome (prosome, macropain) sub	20846.6	46585.13	21595.81
1371391_at	306869	Txndc5_pre	thioredoxin domain containing 5 (predi	20841.99	13028	6598.159
1370320_at	171564	Mawbp	MAWD binding protein	20756.4	26701.45	12116.92
1399101_at	362251	Rnpc2	RNA-binding region (RNP1, RRM) con	20748.48	20152.06	68269.75
1388989_at	361923	Rpl22l1_pr	ribosomal protein L22 like 1 (predicted	20743.66	35722.25	60475.05
1392465_at	290284	Sap18	sin3-associated polypeptide, 18kDa	20715.79	15092.63	20221.97
1367463_at	114766	Phb2	prohibitin 2	20693.89	21312.46	8253.635
1371341_at	292688	Snrpd2_pre	small nuclear ribonucleoprotein D2 (pr	20620.7	26661.84	5938.359
1371936_at	287436	Eif4a1	eukaryotic translation initiation factor 4	20546.75	31205.69	3350.178
1398954_at	301529	Dnpep	NA	20532.49	11146.8	11324.2
1371701_at	299954	Ndufb9_pre	NADH dehydrogenase (ubiquinone) 1	20521.43	48721.99	12594.27

1369961_at	64369	Ppap2a	phosphatidic acid phosphatase 2a	20520.76	13930.38	4049.327
1398851_at	29753	Ywhae	tyrosine 3-monooxygenase/tryptophan	20489.63	30530.54	26541.81
1398829_at	25639	Fkbp1a	FK506 binding protein 1a	20485.48	24194.46	11479.17
1372123_at	298596	Sdhb_pred	succinate dehydrogenase complex, su	20472.65	11717.98	3250.416
1367670_at	24368	Fh1	fumarate hydratase 1	20329.84	9793.618	6014.326
1388550_at	298012	Rad23b	RAD23b homolog (S. cerevisiae)	20297.57	27176.78	18329.63
1375052_at	501015	LOC50101	NA	20258.88	714.9142	2609.15
1367736_at	117044	Rraga	Ras-related GTP binding A	20257.03	15999.99	17255.44
1369940_at	83688	Taldo1	transaldolase 1	20220.83	14502.31	7424.143
1368878_at	89784	Idi1	isopentenyl-diphosphate delta isomera	20199.84	26143.66	54065.64
1388327_at	294362	RGD13095	similar to DNA segment, Chr 10, ERAT	20185.67	28343.01	26356.27
1368173_at	60373	Nol5	nucleolar protein 5	20161.83	11770.34	32486.22
1389480_at	502084	Rwdd4a	RWD domain containing 4A	20145.13	20094.37	18334.52
1367712_at	116510	Timp1	tissue inhibitor of metalloproteinase 1	20055.72	279.4071	5102.964
1372612_at	140734	Dynll2	dynein light chain LC8-type 2	20055.59	12466.75	17478.82
1387031_at	117030	Erp29	endoplasmic reticulum protein 29	20054.57	12196.98	7155.039
1374437_at	291556	Nars	asparaginyl-tRNA synthetase	20023.09	9383.828	8984.5
1387855_at	25183	Gdi1	guanosine diphosphate dissociation in	19982.61	20632.97	17178.69
1369013_a_at	171061	Mrpl17	mitochondrial ribosomal protein L17	19961.5	16362.73	5455.449
1373346_at	289874	RGD13065	similar to hypothetical protein CL2508	19799.12	19694.28	21613.7
1388370_at	289500	Ccni_predi	cyclin I (predicted)	19792.18	35577.99	23974.7
1376739_at	373065	Ddx24	DEAD (Asp-Glu-Ala-Asp) box polypept	19769.77	7223.723	4047.808
1389832_at	114846	Gsto1	glutathione S-transferase omega 1	19751.56	37614.54	17576.66
1374413_at	363869	Ubl3	ubiquitin-like 3	19747.42	17494.19	54768.15
1398814_at	81830	Rab11a	RAB11a, member RAS oncogene fam	19733.54	10976.18	10437.97
1372878_at	365703	Zfr	zinc finger RNA binding protein	19621.23	27906.35	30352.97
1371653_at	24852	Tpm4	tropomyosin 4	19592.49	26196.54	17443.12
1371481_at	364838	Reep5_pre	receptor accessory protein 5 (predicte	19559.58	9461.734	6535.98
1388365_at	291969	Atp6v0d1	ATPase, H+ transporting, V0 subunit D	19559.35	10183.1	12403.43
1388995_at	619577	Rnf14	NA	19520.16	15653.89	17854.14
1392453_at	84360	Clcn3	chloride channel 3	19488.99	9877.428	9937.106
1371362_at	315133	Ddx17	DEAD (Asp-Glu-Ala-Asp) box polypept	19482.63	13843.44	22720.8
1371402_at	117596	Atp6v1b2	ATPase, H transporting, lysosomal V1	19386.27	18369.43	12863.95
1388394_at	292023	Aars	alanyl-tRNA synthetase	19380.6	6496.23	7276.904
1370928_at	65161	Litaf	LPS-induced TN factor	19380.18	13789.16	11422.91
1378925_at	25620	Crem	cAMP responsive element modulator	19333.13	8683.927	45744.56
1370378_at	65262	Atp5a1	ATP synthase, H+ transporting, mitoch	19325.23	50622.31	8813.648
1398959_at	25695	Cebpd	CCAAT/enhancer binding protein (C/E	19314.29	4711.708	15643.58
1374396_at	299971	Atp6v1c1	ATPase, H+ transporting, V1 subunit C	19204.15	13292.8	7418.015
1373815_at	290994	Lman2_pre	lectin, mannose-binding 2 (predicted)	19199.22	19349.52	2245.602
1388390_at	299899	Eif3s3	eukaryotic translation initiation factor 3	19163.67	31217.61	41378.7
1375421_a_at	192256	Pja2	praja 2, RING-H2 motif containing	19073.09	7356.381	9403.71
1398326_at	361824	MGC1056	similar to Nur77 downstream protein 2	19068.38	16873.94	6639.715
1372601_at	282840	Atf5	activating transcription factor 5	19057.03	1939.121	809.0085
1371585_at	24420	Gspt1	G1 to S phase transition 1	19019.62	19888.05	12151.75
1389965_at	192152	Tgoln2	trans-golgi network protein 2	18980.38	8871.613	30017.38
1392543_at	310511	RGD15636	similar to retinoblastoma binding prote	18970	11213.37	18719.47
1370180_at	94267	Nudt4	nudix (nucleoside diphosphate linked m	18960.59	13918.88	36520.09
1372810_at	305178	Hnrpdl	heterogeneous nuclear ribonucleoprot	18895.77	26733.34	33369.73
1371511_at	301511	Arpc2_pre	actin related protein 2/3 complex, subu	18881.95	13945.81	19032.84
1388568_at	362952	Eif3s7	eukaryotic translation initiation factor 3	18829.48	19313.32	9893.678

1387247_at	25204	Pcsk1	proprotein convertase subtilisin/kexin t	18824.14	5385.567	7745.562
1388666_at	294674	Enc1	ectodermal-neural cortex 1	18819.89	22154.79	7983.918
1372474_at	366595	Sypl	synaptophysin-like protein	18817.76	6307.219	20425.07
1387863_at	117180	Unr	upstream of NRAS	18814.07	15776.13	15367.26
1370189_at	117259	Sfrs10	splicing factor, arginine/serine-rich 10 (	18752.58	24662.05	71301.29
1372461_at	307947	Set_predic	SET translocation (predicted)	18678.78	16304.9	4904.443
1388391_at	363441	Ndufa1_pre	NADH dehydrogenase (ubiquinone) 1	18659.81	50445.04	33775.78
1368430_at	63865	Lgmn	legumain	18653.07	5215.418	5126.073
1376570_at	294864	Cct5	chaperonin subunit 5 (epsilon)	18642.37	31571.74	26021.38
1398781_at	116664	Atp6v1f	ATPase, H transporting, lysosomal V1	18631.29	25622.7	12016.44
1387016_a_at	56064	Sdfr1	stromal cell derived factor receptor 1	18571.17	16206.03	52405.1
1371337_at	298762	Cox7a2l_p	cytochrome c oxidase subunit VIIa poly	18545.4	22808.26	14900.93
1373319_at	84474	Ddx1	DEAD (Asp-Glu-Ala-Asp) box polypept	18544.27	22571.22	14538.85
1371392_at	292804	Gpi	glucose phosphate isomerase	18500.1	14948.32	3871.713
1367609_at	81683	Mif	macrophage migration inhibitory factor	18497.87	17859.6	4057.738
1367741_at	85430	Herpud1	homocysteine-inducible, endoplasmic	18467.27	5744.382	20418.21
1384866_at	316077	Entpd3	ectonucleoside triphosphate diphospho	18460.53	11090.72	4922.055
1388549_at	619385	Ncoa4_pre	nuclear receptor coactivator 4 (predicte	18445.57	9150.936	20570.83
1367971_at	85237	Ptp4a2	protein tyrosine phosphatase 4a2	18402.79	11592.61	10826.36
1373051_at	246046	Lmbrd1	LMBR1 domain containing 1	18400.41	13212.26	24003.58
1398896_at	300674	Arcn1	archain 1	18353.05	5500.875	11872.67
1388335_at	304983	Tagln2	transgelin 2	18313.65	14778.18	4325.355
1367496_at	306197	Tm9sf2	transmembrane 9 superfamily member	18273.81	10271.71	19524.92
1375441_at	266975	Sars1	seryl-aminoacyl-tRNA synthetase 1	18272.07	5705.915	4126.892
1391007_s_at	83535	Kcnj11	potassium inwardly rectifying channel,	18266.46	28167.02	14588.74
1372009_at	313047	Yars	tyrosyl-tRNA synthetase	18259.27	10719.02	7346.72
1370309_a_at	83498	Hnrpab	heterogeneous nuclear ribonucleoprote	18256.3	26727.49	9861.792
1371348_at	29425	Psmb5	proteasome (prosome, macropain) sub	18232.31	21728.14	8986.145
1388748_at	298875	Laptm4a	lysosomal-associated protein transmer	18207.83	29312.76	8081.542
1371371_at	288088	Ndufb4	NADH dehydrogenase (ubiquinone) 1	18151.03	38802.39	22074.34
1373408_at	366995	Tbca	tubulin cofactor a	18111.99	40371.79	27780.62
1371384_at	294680	Btf3	basic transcription factor 3	18105.09	37713.4	22874.77
1367578_at	29338	Prdx2	peroxiredoxin 2	18050.79	18234.54	13979.21
1372711_at	498750	LOC49875	NA	17965.5	14937.45	17578.25
1394940_at	300870	RGD13113	similar to hypothetical protein FLJ2003	17940.58	7946.687	13799.37
1398771_at	50567	Slc3a2	solute carrier family 3 (activators of dib	17934.13	6727.008	12211.64
1375516_at	293130	Ndufc2	NADH dehydrogenase (ubiquinone) 1,	17759.66	43904.96	19360.81
1398941_at	363005	Pcbp2	poly(rC) binding protein 2	17748.23	21624.08	34702.85
1368711_at	25099	Foxa2	forkhead box A2	17674.66	8007.303	15501.21
1368992_a_at	29667	Sfrs5	splicing factor, arginine/serine-rich 5	17630.41	14507.15	16303.6
1388975_at	361071	Sucla2_pre	succinate-Coenzyme A ligase, ADP-fo	17597.42	7749.375	8697.401
1398763_at	54312	Timm23	translocase of inner mitochondrial mer	17594.73	16560.85	7923.99
1375631_at	301337	Plekhb2_pr	pleckstrin homology domain containing	17579.13	10670.62	11130.91
1382721_at	360584	Zfp403	zinc finger protein 403	17532.89	16412.71	18375.42
1371690_at	64187	Arl1	ADP-ribosylation factor-like 1	17510.99	12894.03	9155.383
1368585_at	29131	Cart	cocaine and amphetamine regulated tr	17506.48	316.8831	12504.21
1386879_at	83781	Lgals3	lectin, galactose binding, soluble 3	17473.2	210.3882	5995.93
1398932_at	312393	Hint1_pred	histidine triad nucleotide binding protei	17443.94	56793.59	16208.09
1372627_at	290408	Sugt1	SGT1, suppressor of G2 allele of SKP	17442.85	7184.989	19910.88
1383075_at	58919	Ccnd1	cyclin D1	17329.64	198.2358	542.5276
1368212_at	81650	Csnk2b	casein kinase 2, beta subunit	17322.06	5961.507	40907.49

1368507_at	29670	Psma3	proteasome (prosome, macropain) sub	17305.18	16255.18	12787.6
1389454_at	292814	Pdcd5_pre	programmed cell death 5 (predicted)	17292.32	25361.75	20575.55
1372395_at	294862	RGD15657	similar to KIAA0597 protein (predicted)	17042.21	13170.38	19929.34
1387884_at	29672	Psma5	proteasome (prosome, macropain) sub	17032.16	23275.87	8173.461
1388906_at	498410	RGD15649	similar to novel protein similar to Tensi	17011.84	21250.5	23784.92
1398965_at	316052	LOC31605	eomesodermin	16983.4	3182.088	10794.76
1382203_at	306351	Gdf1_predi	growth differentiation factor 1 (predicte	16965.03	14450.46	5355.851
1375426_a_at	171137	Khsrp	KH-type splicing regulatory protein	16903.16	11525.08	3700.915
1388381_at	298700	Eif3s4	eukaryotic translation initiation factor 3	16826.42	21953.29	10559.15
1370244_at	25697	Ctsl	cathepsin L	16815.38	12430.35	22947.1
1389288_at	291660	Ndufa2_pre	NADH dehydrogenase (ubiquinone) 1	16793.53	12361.2	9264.549
1371409_at	498886	RGD15629	similar to cDNA sequence BC031181 (	16757.87	19687.98	8743.462
1374118_at	291624	Lars	leucyl-tRNA synthetase	16741.64	9985.143	10519.58
1367824_at	25318	Fnta	farnesyltransferase, CAAX box, alpha	16740.59	10652.51	9249.841
1370170_at	117280	Hnrpu	heterogeneous nuclear ribonucleoprote	16737.38	13234.91	32471.69
1386871_at	29328	Gpx4	glutathione peroxidase 4	16734.24	18478.34	5193.451
1373029_at	261736	Cops2	COP9 (constitutive photomorphogenic	16722.92	22479.44	10839.72
1370957_at	25205	Il6st	interleukin 6 signal transducer	16712.88	7846.751	4608.032
1372383_at	246254	Gpsm1	G-protein signalling modulator 1 (AGS	16710.43	1992.647	1229.754
1370341_at	24334	Eno2	enolase 2, gamma	16678.25	2071.113	4931.514
1372247_at	313648	Ddost_pred	dolichyl-di-phosphooligosaccharide-pro	16674.04	7488.881	8644.645
1370902_at	286921	Akr1b8	aldo-keto reductase family 1, member	16656.05	68.75537	72.08358
1398909_at	301124	LOC30112	NA	16632.38	25876.07	7228.617
1371350_at	171347	Mat2a	methionine adenosyltransferase II, alp	16629.61	11749.13	11771.5
1367585_a_at	24211	Atp1a1	ATPase, Na+/K+ transporting, alpha 1	16625.66	14103.92	5590.352
1367870_at	58815	Txnl2	thioredoxin-like 2	16618.19	25156.91	21111.89
1388562_at	296128	Stard7_pre	START domain containing 7 (predicted	16613.55	13769.68	8709.373
1375520_at	315345	Copz1_pre	coatomer protein complex, subunit zet	16536.28	16258.38	4214.473
1374431_at	360716	Atp6v1a1_	ATPase, H transporting, lysosomal V1	16490.56	22673.61	8222.088
1390234_at	84486	Sf3b1	splicing factor 3b, subunit 1	16486.87	16934.02	34693.48
1367671_at	25737	Pcna	proliferating cell nuclear antigen	16436.17	48327.52	63112.78
1398948_at	246244	Tax1bp1	Tax1 (human T-cell leukemia virus type	16333.27	21011.54	17614.91
1388134_at	300033	Eef1d	eukaryotic translation elongation factor	16321.88	16479.99	7279.005
1398765_at	116563	Ap2m1	adaptor-related protein complex 2, mu	16316.54	27998.08	9020.616
1371647_at	309475	RGD15646	similar to transmembrane protein TM9	16302.77	17535.55	4687.716
1371419_at	305614	Spnb2	spectrin beta 2	16272.63	24348.79	32944.56
1367589_at	79250	Aco2	aconitase 2, mitochondrial	16267.07	17821.51	3740.383
1370954_at	64475	P4ha1	procollagen-proline, 2-oxoglutarate 4-d	16232.88	1848.597	7664.179
1398904_at	317259	Nono	non-POU domain containing, octamer-	16169.71	25461.95	32186.85
1386968_at	58977	Ppp1r1a	protein phosphatase 1, regulatory (inhi	16143.46	38628.11	7729.561
1389968_at	292148	Eif3s10	eukaryotic translation initiation factor 3	16114.52	6519.944	21932.04
1388112_at	85333	Slc25a4	solute carrier family 25 (mitochondrial	16104.56	64112.07	12781.75
1387342_at	79218	Gng5	guanine nucleotide binding protein (G	16077.53	3210.253	16845.38
1382238_at	361849	RGD15603	similar to cell division cycle and apopto	15967.12	10588.78	8748.114
1372707_at	84379	Rab6a	RAB6A, member RAS oncogene family	15961.9	15335.6	17451.86
1373058_at	300857	Tmem30a	transmembrane protein 30A	15922.65	9719.427	16338.89
1387013_at	57395	Tmem27	transmembrane protein 27	15886.89	7331.256	111989.1
1379365_at	305236	Cxcl11	chemokine (C-X-C motif) ligand 11	15875.3	1375.565	3017.746
1371540_at	295243	Krtcap2_pr	keratinocyte associated protein 2 (pred	15872.21	10543.35	7847.023
1371626_at	363707	Srp68_pred	signal recognition particle 68 (predicted	15870.5	11677.06	4147.703
1388503_at	499882	RGD15627	similar to CREBBP/EP300 inhibitory pr	15843.43	13045.71	7741.351

1388389_at	117515	2-Sep	septin 2	15841.83	12069.16	17073.76
1371390_at	296554	Tubb2c	tubulin, beta 2c	15829.25	29877.8	12174.56
1391075_at	308118	Rgs17_pre	regulator of G-protein signaling 17 (pre	15825.18	5653.09	6596.291
1388681_at	287276	Sar1b	SAR1 gene homolog B (S. cerevisiae)	15812.42	16847.78	24324.92
1382322_a_at	308435	Cic_predict	capicua homolog (Drosophila) (predicte	15812.39	8481.231	6563.274
1374571_at	288041	Pigx	phosphatidylinositol glycan, class X	15811.32	21719.73	15265.11
1388767_at	308061	Pdcd6_pre	programmed cell death 6 (predicted)	15730.37	12125.71	15774.44
1370230_at	94271	Atp5j	ATP synthase, H+ transporting, mitoch	15727.05	25401.73	16617.61
1367884_at	94197	Rab14	RAB14, member RAS oncogene family	15719.29	7589.624	3004.439
1379420_at	299135	RGD1565C	similar to Dehydrogenase/reductase S	15712.61	3732.133	9059.701
1371311_at	289217	Sdhc	succinate dehydrogenase complex, su	15691.35	10470.53	2603.715
1388752_at	293017	Bclaf1	BCL2-associated transcription factor 1	15650.22	14407.78	24596.78
1372119_at	300968	Ube1dc1	ubiquitin-activating enzyme E1-domain	15630.37	4341.146	4357.878
1388563_at	294734	RGD13054	similar to RIKEN cDNA 1700034P14 (p	15590.87	11332.71	28215.68
1371564_at	297566	Atp6v1e1	ATPase, H+ transporting, V1 subunit E	15553.03	22244.4	4642.28
1368470_at	25455	Ggh	gamma-glutamyl hydrolase	15545.03	17391.8	56433.83
1388516_at	315970	LOC31597	similar to CDV-3B	15499.48	4727.629	13292.5
1398786_at	29675	Psmb2	proteasome (prosome, macropain) sub	15467.07	35895.34	9403.168
1398972_at	287367	Cops3	COP9 (constitutive photomorphogenic	15463.93	17377.02	6201.287
1370050_at	29598	Atp2b1	ATPase, Ca++ transporting, plasma m	15408.86	22247.74	14319.43
1384125_at	311968	Mll5	myeloid/lymphoid or mixed-lineage leu	15404.91	12488.57	24144.27
1371342_at	300047	Cyc1_pred	cytochrome c-1 (predicted)	15381.8	23439.55	4090.365
1388576_at	288516	Eif3s9	eukaryotic translation initiation factor 3	15381.55	8955.078	4194.016
1398800_at	56011	Ywhab	tyrosine 3-monooxygenase/tryptophan	15378.22	29006.92	24952.45
1371786_at	498538	Trim35	tripartite motif protein 35	15369.52	13373.7	41405.7
1386887_at	94194	Cox5b	cytochrome c oxidase subunit Vb	15355.19	34761.41	10513.19
1369991_at	65166	Sec11l1	Sec11-like 1 (S. cerevisiae)	15343.53	15986.66	8678.9
1372568_at	316021	Epm2aip1_	EPM2A (laforin) interacting protein 1 (p	15338.84	14600.78	23631.22
1398794_at	64525	Tceb1	transcription elongation factor B (SIII),	15334.14	26506.56	16056.77
1374188_at	294912	Tloc1	translocation protein 1	15303.73	6603.259	21330.67
1371403_at	295230	Cct3	chaperonin subunit 3 (gamma)	15253.44	20224.14	5648.377
1374464_at	360709	Tfg	Trk-fused gene	15230.07	15318.5	11790.38
1368158_at	54350	Scfd1	sec1 family domain containing 1	15173.53	8838.414	15404.26
1367873_at	83615	Atp6ap1	ATPase, H+ transporting, lysosomal ac	15122.96	13258.59	5310.289
1386915_at	170724	Anp32b	acidic nuclear phosphoprotein 32 famil	15079.4	21952.35	12592.27
1385921_at	362154	LOC36215	brain zinc finger protein	15044.42	13355.65	4905.049
1383615_a_at	362736	RGD15616	similar to HECT domain containing 1 (p	15032.15	2906.815	3752.235
1368511_at	117095	Bhlhb3	basic helix-loop-helix domain containin	15025.28	4757.346	22005.2
1371041_at	81728	Ndufv2	NADH dehydrogenase (ubiquinone) fla	15011.74	13741.05	3342.22
1398773_at	117268	Khdrbs1	KH domain containing, RNA binding, s	14959.09	20401.68	17948.4
1387774_at	25578	Ywhaz	tyrosine 3-monooxygenase/tryptophan	14954.66	27912.73	15926.84
1399003_at	303606	RGD13088	similar to adipocyte-specific protein 4	14922.67	8810.597	9473.731
1389323_at	363064	Wdr61	WD repeat domain 61	14912.88	8816.124	5894.606
1380500_s_at	293702	Fkbp2_pre	FK506 binding protein 2 (predicted)	14901.73	18877.92	11481.55
1373195_at	317385	Fus	fusion, derived from t(12;16) malignant	14894.79	20328.5	24500.87
1368035_a_at	360406	Ptprf	protein tyrosine phosphatase, receptor	14891.31	7377.22	3846.128
1386893_at	29143	Grn	granulin	14847.82	18849.99	7748.776
1373048_at	299121	Actr10	ARP10 actin-related protein 10 homolo	14800.34	10405.3	8557.078
1375006_at	362341	RGD13092	similar to RIKEN cDNA B130055L09 (p	14789.3	10820.45	16015.19
1398929_at	300069	Eif3s6ip	eukaryotic translation initiation factor 3	14776.03	26091.01	9739.784
1382150_at	291709	RGD1305C	similar to RIKEN cDNA 1200007B05 g	14740.69	14589.63	8441.262

1370339_at	117557	Tpm3	tropomyosin 3, gamma	14733.87	17571.27	7827.742
1374063_at	361814	Sfrs3_pred	splicing factor, arginine/serine-rich 3 (S	14725.15	29142.76	18864.43
1372929_at	83731	Kcnma1	potassium large conductance calcium-	14696.3	3367.188	8392.824
1369977_at	29545	Uchl1	ubiquitin carboxy-terminal hydrolase L	14683.69	20017.82	15421.8
1388313_at	122799	Rps25	ribosomal protein s25	14648.65	11140.61	26120.41
1367972_at	117152	Cand1	cullin associated and neddylation disas	14639.18	21544.45	17116.25
1388870_at	360596	RGD15603	similar to RNA-binding protein Musash	14620.67	11061.25	20427.5
1375146_at	360636	RGD13061	similar to RIKEN cDNA 3010027G13	14603.15	8431.474	4753.177
1383339_at	308537	RGD13103	similar to NNX3 (predicted)	14590.91	4344.803	4292.39
1386863_at	24668	Ppp1ca	protein phosphatase 1, catalytic subun	14589.36	37810.48	4439.621
1377593_at	296394	Dpm1_pre	dolichol-phosphate (beta-D) mannosyl	14578.29	7141.312	24787.5
1367787_at	81024	Ica1	islet cell autoantigen 1	14575.01	19535.1	12363.99
1388640_at	499417	LOC49941	similar to Ubiquitin-like protein SMT3A	14571.62	22160.18	7167.968
1372829_at	295284	Rbm8_pre	RNA binding motif protein 8 (predicted	14544.58	25750.64	19677.07
1372298_at	298066	Txndc4	thioredoxin domain containing 4 (endo	14510.2	6378.754	5170.255
1379101_at	310461	Dhx36_pre	DEAH (Asp-Glu-Ala-His) box polypepti	14506.32	7562.395	5579.792
1373362_at	293448	Uqcrc2	ubiquinol cytochrome c reductase core	14497.03	34294.68	20841.19
1375056_at	289589	NA	NA	14485.7	17890.6	8482.316
1383100_at	309146	RGD13063	similar to RIKEN cDNA 1810036I24 (p	14431.58	5527.828	13115.51
1383265_at	304019	Tbc1d23_p	TBC1 domain family, member 23 (pred	14424.38	15202.49	11042.1
1393216_at	64018	Slc33a1	solute carrier family 33 (acetyl-CoA tra	14413.51	5493.926	10582.77
1369457_a_at	140594	Sytl4	synaptotagmin-like 4	14404.44	44579.58	130.0653
1371544_at	503047	NA	NA	14397.25	15561.1	15579.93
1373397_at	114764	Mapre1	microtubule-associated protein, RP/EB	14396.37	27986.87	7258.204
1367452_at	171127	Sumo2	SMT3 suppressor of mif two 3 homolo	14378.12	38183.02	17850.37
1368042_a_at	25459	Hmgb1	high mobility group box 1	14363.97	27726.54	10871.53
1377457_a_at	300652	Sorl1_pred	sortilin-related receptor, L(DLR class)	14356.98	2909.732	5348.387
1383496_at	500409	RGD15604	similar to hypothetical protein FLJ2017	14336.07	9019.081	7168.897
1371495_at	312689	M6pr	mannose-6-phosphate receptor, catior	14334.82	13952.2	8412.63
1371718_at	252891	Sra1	steroid receptor RNA activator 1	14297.81	10902.4	7225.233
1367856_at	24377	G6pdx	glucose-6-phosphate dehydrogenase)	14292.89	5495.218	3046.036
1389868_at	500988	RGD15645	similar to RCK (predicted)	14292.82	3192.615	3670.762
1389531_at	361387	Zfp330_pre	zinc finger protein 330 (predicted)	14268.62	9025.156	23390.96
1368653_a_at	117287	Park7	Parkinson disease (autosomal recessiv	14250.75	10142.71	9109.694
1372464_at	297428	Copg	coatomer protein complex, subunit gar	14247.06	9105.302	4603.799
1373200_at	291057	Eef1e1_pre	eukaryotic translation elongation factor	14230.76	14620.75	8149.572
1374323_at	361666	Bccip_prec	BRCA2 and CDKN1A interacting prote	14230.22	14226.28	9319.865
1370278_at	245965	Atp5d	ATP synthase, H+ transporting, mitoch	14217.34	15792.8	3345.937
1383643_at	499317	RGD15603	similar to UPF0197 protein C11orf10 h	14151.84	27788.58	8658.352
1370179_at	170714	Dncl2a	dynein, cytoplasmic, light chain 2A	14101.18	21949.55	12752.8
1370007_at	116598	Pdia4	protein disulfide isomerase associated	14066.11	4125.876	3414.06
1388315_at	299310	Ndufb11_p	NADH dehydrogenase (ubiquinone) 1	14061.59	30422.58	18299.91
1398960_at	288620	Cct6a	chaperonin subunit 6a (zeta)	14061.02	22476.01	12386.01
1382326_at	83631	Dedd	death effector domain-containing	14060.21	7036.162	7211.771
1398923_at	287453	RGD13056	similar to D11Bwg0434e protein (predi	14035.43	21088.58	7574.592
1367713_at	54318	Eif2s1	eukaryotic translation initiation factor 2	13995.55	6326.862	4032.142
1386891_at	29542	Pebp1	phosphatidylethanolamine binding prot	13988.54	39309.34	8491.545
1388822_at	364981	Scoc	short coiled-coil protein	13964.87	11624.49	20121.08
1384253_at	364558	LOC36455	similar to palladin; CGI-151 protein	13929.65	82313.77	5812.205
1373718_at	291081	Tubb2b	tubulin, beta 2b	13915.5	29520.45	11990.29
1368123_at	25718	Igf1r	insulin-like growth factor 1 receptor	13893.4	2445.824	859.2515

1370838_s_at	64159	Spna2	alpha-spectrin 2	13881.17	16241.17	10159.73
1388997_at	140940	Arf3	ADP-ribosylation factor 3	13871.43	18026.87	12395.22
1371423_at	296551	Mrpl41	mitochondrial ribosomal protein L41	13855.21	20448.81	11445.86
1398919_at	302247	RGD13047	similar to Hypothetical protein CGI-99	13848.06	18297.97	11466.22
1369691_at	497770	Scn3a	sodium channel, voltage-gated, type II	13839.85	8304.155	6112.568
1387015_at	81531	Pfn2	profilin 2	13825.23	11379.68	8476.334
1398782_at	140673	Napa	N-ethylmaleimide sensitive fusion prot	13818.52	8409.177	6826.154
1398907_at	288783	Ormdl2_pr	ORM1-like 2 (S. cerevisiae) (predicted	13809.44	7640.262	3532.619
1375221_at	296182	Txndc13	thioredoxin domain containing 13	13805.12	26899.51	36339.22
1398772_at	83809	Nsfl1c	NSFL1 (p97) cofactor (p47)	13790.28	11374.56	2424.524
1371437_at	297522	Sec13l1	SEC13-like 1 (S. cerevisiae)	13772.18	20003.77	4626.375
1371742_at	361574	Snrp70_pr	U1 small nuclear ribonucleoprotein pol	13752.15	6314.871	6656.296
1371445_at	287633	Lrrc59	leucine rich repeat containing 59	13741.59	17560.28	3357.523
1370276_at	192241	Atp5o	ATP synthase, H+ transporting, mitoch	13702.63	20205.69	7248.747
1371802_at	290659	Cope_pred	coatomer protein complex, subunit eps	13688.32	11985.51	7216.77
1374846_at	498008	Clp1	cardiac lineage protein 1	13680.2	7427.473	33556.88
1388332_at	363875	Rac1	ras-related C3 botulinum toxin substra	13678.42	24177.59	16272.42
1374797_at	306261	Eaf1_predi	ELL associated factor 1 (predicted)	13639.68	3230.91	6046.817
1369927_at	81829	Mor1	malate dehydrogenase, mitochondrial	13620.88	20567.72	6154.677
1386926_at	94340	Acsl5	acyl-CoA synthetase long-chain family	13620.55	5929.939	7090.223
1371641_at	499323	RGD15628	similar to CCTeta, eta subunit of the ch	13529.94	23489.09	9133.403
1371596_at	287113	Rnps1	ribonucleic acid binding protein S1	13422.45	18747.76	8157.724
1372806_at	25479	Vps35_ma	vacuolar protein sorting 35 (mapped)	13400.98	12274.95	20251.18
1398889_at	192147	Grinl1a	glutamate receptor, ionotropic, N-meth	13394.27	8993.994	14326.05
1388521_at	361755	Pycs_predi	pyrroline-5-carboxylate synthetase (glu	13381.43	7714.648	4941.328
1386875_a_at	83800	Clta	clathrin, light polypeptide (Lca)	13377.92	21056.59	18037.91
1373063_at	289623	Hip2_predi	huntingtin interacting protein 2 (predict	13364.11	17056.48	13652.66
1389040_at	288165	LOC28816	similar to PEST-containing nuclear pro	13343.69	14760.1	23779.07
1383538_at	311115	RGD15652	similar to zinc finger protein 650 (predi	13320.8	9292.288	13399.67
1375338_at	50993	Rab10	RAB10, member RAS oncogene family	13310.2	12800.55	11270.81
1383651_a_at	360682	Wdr45l_pre	Wdr45 like (predicted)	13298.76	9767.758	22491.19
1367819_at	25721	Got2	glutamate oxaloacetate transaminase 2	13282.11	17469.17	6210.912
1388443_at	360804	Cdk2ap1_p	CDK2 (cyclin-dependent kinase 2)-ass	13274.73	29646.29	13703.03
1370890_at	81732	Actr3	ARP3 actin-related protein 3 homolog	13237.61	9911.337	7577.971
1369215_a_at	25306	Cpd	carboxypeptidase D	13212.61	17598.92	6814.366
1370296_at	25541	Scp2	sterol carrier protein 2	13198.99	21835.2	5752.763
1398989_at	304092	Son	Son cell proliferation protein	13186.32	12247.19	33268.47
1388960_at	294504	Pyp_mapp	pyrophosphatase (mapped)	13182.85	16962.9	6282.296
1388301_at	301011	Uqcrc1	ubiquinol-cytochrome c reductase core	13151.5	10303.84	6337.925
1371388_at	289950	Pdhb	pyruvate dehydrogenase (lipoamide) b	13143.62	22723.76	22981.15
1375548_at	298425	RGD13103	similar to RIKEN cDNA 4732418C07 (	13138.71	3460.859	1664.422
1367835_at	246333	Pcsk1n	proprotein convertase subtilisin/kexin t	13137.85	12651.18	49890.3
1371777_at	288398	RGD15624	similar to Pabpc4_predicted protein (pr	13136.3	7885.183	5981.195
1381428_a_at	29199	Hfe	hemochromatosis	13133.91	3823.604	22157.95
1388873_at	316575	Trip12	thyroid hormone receptor interactor 12	13117.27	5430.741	7118.02
1373185_at	295235	Ssr2_predi	signal sequence receptor, beta (predic	13109.18	16966.37	2753.226
1367509_at	502227	RGD15609	similar to general transcription factor II	13103.08	17813.01	11467.43
1368098_a_at	81781	Snrpn	small nuclear ribonucleoprotein N	13102.96	21505.33	4018.819
1371418_at	299809	Cct2	chaperonin containing TCP1, subunit 2	13092.47	29170.22	11398.5
1372741_at	305021	Sccpdh	saccharopine dehydrogenase (putative	13062.56	7003.234	8615.174
1398892_at	286898	Npc2	Niemann Pick type C2	13055.3	2270.916	20449.69

1370381_at	286988	Pnrc1	proline rich 2	13028.89	4101.838	44295.94
1398325_at	300733	Tspan3	tetraspanin 3	12998.58	16669.7	19722.05
1388376_at	293700	RGD13097	similar to RIKEN cDNA 0610038D11 (|	12994.15	18925.18	8439.848
1388333_at	300084	Rbx1	ring-box 1	12958.66	15451.6	5621.709
1371380_at	29554	Pdha1	pyruvate dehydrogenase E1 alpha 1	12947.98	10991.08	18083.33
1371464_at	293067	Za20d3	zinc finger, A20 domain containing 3	12944.4	16368.94	22249.08
1399022_at	301434	Clk1	CDC-like kinase 1	12923.6	7550.724	43175.22
1368391_at	25648	Slc7a1	solute carrier family 7 (cationic amino a	12908.13	3612.728	33188.93
1398219_at	500440	RGD15625	similar to RIKEN cDNA A930001M12 g	12889.31	22093.2	1426.497
1388645_at	296488	RGD13079	similar to RIKEN cDNA 2810409H07	12873.85	16437.68	13850.51
1387925_at	25612	Asns	asparagine synthetase	12848.65	15156.11	11295.68
1367760_at	170851	Map2k1	mitogen activated protein kinase kinas	12838.96	4585.96	8971.74
1373193_at	690089	LOC69008	NA	12822.59	20435.92	21760.17
1372497_at	303554	Nbr1	neighbor of Brca1 gene 1	12814.69	15481.53	16594.25
1398937_at	289693	Dhx15_pre	DEAH (Asp-Glu-Ala-His) box polypepti	12785.89	20680.51	20272.85
1398858_at	287984	Psmd2	proteasome (prosome, macropain) 26S	12777.05	16139.23	4139.651
1370861_at	25282	Cox6a1	cytochrome c oxidase, subunit VIa, po	12773.71	29533.78	12400.13
1388424_at	311371	Eif3s1_pre	eukaryotic translation initiation factor 3	12761.44	10882.87	11041.19
1374513_at	64551	7-Sep	septin 7	12748.32	10196.48	28378.85
1387911_at	25533	Rabggtb	RAB geranylgeranyl transferase, b sub	12737.25	9874.652	9488.153
1377143_at	287642	Slc35b1	solute carrier family 35, member B1	12730.98	17755.67	4172.282
1388789_at	362857	RGD15633	similar to hypothetical protein MGC179	12717.29	7712.314	15456.8
1385238_at	361342	Cplx4_pred	complexin 4 (predicted)	12710.33	10604.55	5380.35
1368029_at	25643	Gnai3	guanine nucleotide binding protein, alp	12704.69	14638.25	19210.95
1390178_at	300965	Srprb	signal recognition particle receptor, B s	12698.16	3656.512	5707.118
1398837_at	81807	Tceb2	transcription elongation factor B (SIII),	12690.24	29655.72	13039.61
1398930_at	298451	Atp6v0b_p	ATPase, H+ transporting, V0 subunit B	12687.65	15629.34	4517.328
1377608_a_at	298648	Tardbp	TAR DNA binding protein	12646.73	11151.5	17354.86
1367531_at	288599	Wbscr1	Williams-Beuren syndrome chromosor	12643.92	24178.41	12163.22
1382467_at	363498	LOC36349	similar to brain expressed X-linked pro	12636.16	39873.75	16327.15
1371702_at	363447	Tm4sf2_ma	transmembrane 4 superfamily member	12632.77	20021.5	33487.44
1398810_at	64527	Pdap1	PDGFA associated protein 1	12629.18	29219.92	4699.403
1373754_at	683591	LOC68359	NA	12618.35	3255.61	6271.484
1373160_at	498653	NA	NA	12613.25	6119.482	5988.813
1393547_at	498726	RGD15603	similar to hypothetical protein C630023	12601.37	1926.391	754.0748
1388531_at	361940	Pgrmc2	progesterone receptor membrane com	12584.41	4379.115	5949.291
1392627_x_at	64570	Nat8	N-acetyltransferase 8 (camello like)	12576.31	11183.83	5127.368
1388393_at	302562	Plp2_mapp	proteolipid protein 2 (mapped)	12576.1	11686.08	6904.528
1373877_at	89816	Picalm	phosphatidylinositol binding clathrin as	12572.14	11231.11	17524.13
1371454_at	287477	Tmem93_p	transmembrane protein 93 (predicted)	12559.17	13888.34	4546.369
1373133_at	500282	LOC50028	similar to ADP-ribosylation factor-like 1	12540.12	10227.52	15016.85
1367532_at	300235	Dazap2	DAZ associated protein 2	12526.37	31388.52	18020.45
1393165_at	305070	RGD13593	similar to RIKEN cDNA 2310028N02	12520.57	11547.56	6167.812
1399018_at	360602	RGD13079	similar to cisplatin resistance-associate	12499.9	9949.198	23642.79
1371965_at	362134	RGD13032	similar to RIKEN cDNA 2010311D03	12492.5	10739.21	8201.295
1388414_at	362588	Ndufs5b	NADH dehydrogenase (ubiquinone) Fe	12464.85	30852.59	15090.97
1375425_at	287541	RGD13094	hypothetical LOC287541 (predicted)	12448.74	6829.16	5658.427
1372947_at	81748	Pls3	plastin 3 (T-isoform)	12447.54	12941.71	5651.732
1370855_at	25307	Cst3	cystatin C	12442.4	10163.22	44490.89
1372446_at	192361	Ppp1r2	protein phosphatase 1, regulatory (inhi	12400.96	12417.93	28500.48
1370043_at	79559	Alcam	activated leukocyte cell adhesion mole	12395.65	6233.458	14600.91

1393048_at	25083	Adra2a	adrenergic receptor, alpha 2a	12375.75	3055.98	1257.02
1389282_at	360606	Itga3_pred	integrin alpha 3 (predicted)	12369.22	2480.143	3180.355
1388704_at	362160	Cugbp1	CUG triplet repeat, RNA binding protei	12357.94	8832.574	10095.61
1369868_at	116967	Iag2	implantation-associated protein	12342.74	3409.402	1718.895
1367643_at	25246	Bsg	basigin	12328.04	22093.79	10080.5
1371774_at	302642	Sat_mappe	spermidine/spermine N1-acetyl transfe	12318.03	1625.439	46750.86
1388417_at	288671	Anapc5_pr	anaphase-promoting complex subunit	12307.08	25132.45	6163.682
1375974_at	305240	RGD13095	similar to RNA-binding protein isoform	12296.95	9380.226	25269.72
1388404_at	360762	LOC36076	RNA polymerase 1-3	12252.35	12629.06	17781.58
1372214_at	296995	Mrps33_pr	mitochondrial ribosomal protein S33 (p	12220.12	14023.92	9987.337
1391346_at	292082	RGD1309C	similar to FKSG26 protein (predicted)	12218.13	4669.268	10755.55
1368641_at	84426	Wnt4	wingless-related MMTV integration site	12207.62	11883.05	7887.268
1387778_at	155173	Sdf4	stromal cell derived factor 4	12193.32	6961.743	12286.72
1375231_a_at	291670	Cxxc5	CXXC finger 5	12191.53	6658.122	8869.726
1388985_at	310926	LOC31092	hypothetical protein LOC310926	12183.83	18459.17	1544.535
1388802_at	501625	Bex1	brain expressed X-linked 1	12172.34	39376.94	13311.32
1388750_at	64678	Tfrc	transferrin receptor	12146.15	60381.6	9981.222
1372510_at	296271	Srxn1	sulfiredoxin 1 homolog (S. cerevisiae)	12146.14	1994.399	7213.517
1367987_at	81761	Rnpep	arginyl aminopeptidase (aminopeptida:	12142.38	10393.17	4343.337
1398848_at	81800	St13	suppression of tumorigenicity 13	12107.88	11204.13	15470.44
1368051_at	84013	Hsd17b12	hydroxysteroid (17-beta) dehydrogena	12055.24	12113.7	11273.35
1387790_at	140946	Paics	phosphoribosylaminoimidazole carbox	12050.4	12773.42	6139.718
1387086_at	81715	Camlg	calcium modulating ligand	12045.55	2819.111	1149.42
1398791_at	58819	Txnrd1	thioredoxin reductase 1	12039.9	8444.915	4322.406
1390407_at	288182	Cldnd1	claudin domain containing 1	12037.4	10893.12	8299.368
1367466_at	287530	Prpf8	pre-mRNA processing factor 8	12032.42	16745.11	7207.722
1370172_at	24787	Sod2	superoxide dismutase 2, mitochondrial	12014.8	5362.498	3354.805
1371254_at	291103	Uqcrfs1	ubiquinol-cytochrome c reductase, Rie	12006.31	37454.8	8496.211
1372251_at	300996	Rbm5	RNA binding motif protein 5	11986.26	6890.244	12202.71
1389308_at	360734	Dnajb11	DnaJ (Hsp40) homolog, subfamily B, n	11981.84	10951.07	2125.679
1371964_at	305256	Grsf1	G-rich RNA sequence binding factor 1	11977.62	17111.6	15677.85
1373934_at	500840	NA	NA	11977.15	3652.541	11374.58
1388507_at	305506	Itgb4bp	integrin beta 4 binding protein	11963.57	8839.675	5329.998
1388126_at	29688	Minpp1	multiple inositol polyphosphate histidin	11958.76	9699.921	11441.08
1367894_at	64194	Insig1	insulin induced gene 1	11951.69	14439.21	24118.24
1367769_at	117017	Polr2g	polymerase (RNA) II (DNA directed) pc	11946.69	35324.97	5147.642
1398824_at	65165	Tmed2	transmembrane emp24 domain traffick	11938.36	20416.57	2210.387
1387917_at	246314	Tor1aip1	torsin A interacting protein 1	11929.13	4738.676	5048.395
1367898_at	140923	Bnip3l	BCL2/adenovirus E1B 19 kDa-interact	11920.46	6201.393	9037.181
1387878_at	24399	Glud1	glutamate dehydrogenase 1	11896.67	14864.93	7793.136
1389815_at	259225	Ppp1r14b	protein phosphatase 1, regulatory (inhi	11894.43	17198.62	2386.524
1367908_at	171133	Gcsh	glycine cleavage system protein H (am	11886.35	9829.566	4345.359
1372341_at	501039	LOC50103	NA	11882.07	8355.273	26187.1
1393245_at	114209	Phyh	phytanoyl-CoA hydroxylase	11863.7	2213.034	12818.39
1398300_at	25390	Atp1b3	ATPase, Na+/K+ transporting, beta 3 p	11859.73	4360.037	7177.418
1373431_at	305131	Lrrc8d	leucine rich repeat containing 8 family,	11814.43	3799.987	7467.272
1370658_a_at	266680	St18	suppression of tumorigenicity 18	11811.75	4924.454	2909.57
1367931_a_at	29497	Ptbp1	polypyrimidine tract binding protein 1	11764.38	16377.45	5762.468
1388615_at	295347	Rap1a	RAS-related protein 1a	11758.63	21651.28	19021.47
1373499_at	81714	Gas5	growth arrest specific 5	11744.73	3675.772	28486.29
1374646_at	113927	Csnk1a1	casein kinase 1, alpha 1	11740.59	14933.7	28121.82

1383573_at	682217	LOC68221	NA	11685.86	20192.32	13727.74
1372182_at	60416	Pfkp	phosphofructokinase, platelet	11681.84	4563.438	2224.427
1372075_at	362264	LOC36226	similar to dJ862K6.2.2 (splicing factor,	11658.29	16418.18	8659.298
1369929_at	25524	Psap	prosaposin	11648.6	20010.39	7800.226
1370286_at	29642	Slc38a2	solute carrier family 38, member 2	11643.41	10492.82	40213.69
1399001_at	294030	RGD13054	hypothetical LOC294030	11627.86	23191.16	19570.15
1374387_at	66028	Arl6ip5	ADP-ribosylation factor-like 6 interactir	11626.9	8364.51	4250.537
1367677_at	113898	Prdx5	peroxiredoxin 5	11602.17	16972.26	2586.422
1368275_at	140910	Sc4mol	sterol-C4-methyl oxidase-like	11601.88	38128.33	24316.08
1372364_a_at	360462	RGD13051	similar to N-terminal asparagine amidc	11600.22	16272.28	20364.55
1367642_at	114597	Suclg1	succinate-CoA ligase, GDP-forming, a	11590.97	20710.58	6417.48
1390183_at	360990	LOC36099	similar to ubiquitin specific protease 34	11567.41	11355.93	13557.77
1388705_at	498398	RGD15650	similar to selenoprotein SelM (predicte	11555.66	3922.297	2791.327
1390113_a_at	303158	Larp1_prec	La ribonucleoprotein domain family, me	11553.07	8143.994	12631.24
1388388_at	363192	Klhdc3	kelch domain containing 3	11542.04	9727.285	9185.794
1398784_at	29681	C1qbp	complement component 1, q subcomp	11524.94	16153.64	5631.866
1368843_at	114217	Yme1l1	YME1-like 1 (S. cerevisiae)	11511.22	12370.35	10630.09
1370035_at	24525	Kras	v-Ki-ras2 Kirsten rat sarcoma viral onc	11510.28	17501.53	3156.656
1369976_at	58945	Dynll1	dynein light chain LC8-type 1	11509.82	39649.65	34367.77
1371841_at	79215	Mtpn	myotrophin	11491.22	15199.93	11773.74
1368706_at	116467	Tm4sf4	transmembrane 4 superfamily member	11489.67	26728.29	33497.64
1390514_at	288416	MGC9422	similar to hypothetical protein from BC	11482.52	6819.9	11609.7
1373675_at	114022	Glrx2	glutaredoxin 2 (thioltransferase)	11470.89	14176.74	10364.76
1389189_at	81634	Actn1	actinin, alpha 1	11458.16	4565.224	8210.242
1373447_at	360492	RGD13051	similar to HN1-like protein	11435.91	9877.872	3528.312
1371939_at	362173	Gpiap1	GPI-anchored membrane protein 1	11431.29	18831.37	10053.77
1399046_at	64550	Top1	topoisomerase (DNA) I	11430.74	5831.28	10703.47
1392669_at	306137	RGD13082	similar to CG10084-PA	11430.36	4013.237	22414.64
1388868_at	293960	Zfp216_pre	zinc finger protein 216 (predicted)	11415.2	22974	38138.37
1368844_at	29734	Stch	stress 70 protein chaperone, microsom	11404.62	7147.035	3920.242
1387262_at	81783	Ssb	Sjogren syndrome antigen B	11342.2	8219.625	15186.75
1398792_at	117263	Psmc1	peptidase (prosome, macropain) 26S s	11338.43	9847.074	3113.03
1382579_at	291908	Tnrc9_prec	trinucleotide repeat containing 9 (predi	11330.8	6066.141	20175.82
1372068_at	291259	Zmynd11	zinc finger, MYND domain containing 1	11294.29	15198.03	18320.96
1390423_at	290447	Phr1_predi	pam, highwire, rpm 1 (predicted)	11282.11	3411.948	16299.49
1398853_at	29676	Psmb3	proteasome (prosome, macropain) sub	11274.03	24493.05	6426.74
1370184_at	29271	Cfl1	cofilin 1, non-muscle	11262.04	13605.35	4632.609
1374581_at	362912	RGD13118	similar to RIKEN cDNA 1110021N07	11253.14	10544.88	10386.51
1369970_at	83730	Vamp8	vesicle-associated membrane protein 8	11236.22	11654.91	2456.256
1389089_at	294281	RT1-Ke4	RT1 class I, locus Ke4	11223.16	3628.463	4690.394
1368817_at	498433	Psme4	NA	11218.01	9217.321	6581.628
1377653_at	366669	LOC36666	similar to mKIAA1011 protein	11210.24	1859.594	5538.636
1389277_at	313196	RGD13061	similar to KIAA0368 (predicted)	11208.98	6516.194	10066.14
1369588_a_at	25392	Atpif1	ATPase inhibitory factor 1	11202.58	22300.1	17831.73
1367719_at	117060	Dd5	progestin induced protein	11201.65	11613.03	18451.19
1373508_at	365802	LOC36580	similar to Selenoprotein T	11200.32	15494.96	4912.958
1389623_at	315305	Atf1	activating transcription factor 1	11189.82	13084.13	18798.28
1388853_at	299628	Mrpl54_pre	mitochondrial ribosomal protein L54 (p	11189.81	17089.48	3288.29
1398910_at	287155	Stub1	STIP1 homology and U-Box containing	11151.56	11892.39	5023.308
1388629_at	301005	Impdh2	inosine 5-monophosphate dehydrogen	11137.96	7719.124	6802.525
1370678_s_at	29253	Maoa	monoamine oxidase A	11134.79	3111.252	3654.522

1375530_at	498486	RGD15631	similar to EMeg32 protein (predicted)	11119.81	5025.898	5786.236
1387653_at	64028	Tsnax	translin-associated factor X	11118.34	7875.72	10628.81
1386862_at	25673	Anxa5	annexin A5	11096.67	8203.206	13685.72
1375295_at	170587	Cs	citrate synthase	11085.29	22750.48	8614.816
1368145_at	25510	Pcp4	Purkinje cell protein 4	11083.92	15861.66	7389.522
1377742_at	366300	LOC36630	NA	11081.54	4066.238	5249.136
1393412_at	312159	Fam3c	family with sequence similarity 3, mem	11077.46	11959.73	9376.327
1367693_at	25576	Ywhah	tyrosine 3-monooxygenase/tryptophan	11073.24	21469.72	30393.66
1373870_at	313873	RGD13054	similar to RIKEN cDNA 2810405J04	11062.21	7699.153	4065.39
1367833_at	81827	Psmc5	peptidase (prosome, macropain) 26S s	11062.14	16690.2	4090.4
1389573_at	362196	Chac1_pre	ChaC, cation transport regulator-like 1	11061.65	1165.448	9875.714
1387770_at	170512	Ifi27l	interferon, alpha-inducible protein 27-li	11061.08	53269.95	3866.931
1373548_at	303602	Wdr68_pre	WD repeat domain 68 (predicted)	11046.25	18309.51	15429.91
1387807_at	83572	Pafah1b1	platelet-activating factor acetylhydrolas	11044.59	4969.882	5737.073
1388953_at	290556	Gnl3	guanine nucleotide binding protein-like	11036.68	11953.45	17329.33
1367933_at	81640	Amd1	S-adenosylmethionine decarboxylase	11024.51	7310.504	7629.521
1371894_at	299825	Gns	glucosamine (N-acetyl)-6-sulfatase	11011.74	8888.219	9289.619
1386897_at	60421	Hrmt1l2	heterogeneous nuclear ribonucleoprot	11003.96	17163.21	6073.075
1389245_at	307821	Psmd7_pre	proteasome (prosome, macropain) 26S	10999.42	7213.411	4741.409
1385387_at	366214	Nkx2-2_pre	NK2 transcription factor related, locus	10987.65	5348.62	8056.255
1370029_at	29382	Ctbp1	C-terminal binding protein 1	10981.85	9743.363	8004.397
1372460_at	296619	Pkn3	protein kinase N3	10956.93	15196.46	11137.6
1372145_at	294810	Tars	threonyl-tRNA synthetase	10940.53	4671.329	4822.959
1368712_at	25165	Znf386	zinc finger protein 386 (Kruppel-like)	10934	6363.129	9492.698
1367960_at	29308	Arl4a	ADP-ribosylation factor-like 4A	10930.87	2714.761	24153.36
1377379_at	364081	Irf6_predict	interferon regulatory factor 6 (predicted	10917.11	2385.084	12561.87
1386950_at	25594	Ppp1cb	protein phosphatase 1, catalytic subun	10901.96	13262.7	31889.52
1386947_at	83502	Cdh1	cadherin 1	10894.49	6598.878	20150.08
1371605_at	299739	Ndufa12_p	NADH dehydrogenase (ubiquinone) 1	10886.71	34823.45	17406.89
1388696_at	84478	Ufd1l	ubiquitin fusion degradation 1-like	10866.33	12278.47	7455.465
1372085_at	298757	Arl6ip2	ADP-ribosylation factor-like 6 interactir	10859.47	12929.17	18580.6
1367925_at	64681	Mvp	major vault protein	10847.48	1863.868	894.0621
1371587_at	362045	Map2k1ip1	mitogen-activated protein kinase kinas	10841.07	6157.212	6673.958
1398986_at	296350	Serinc3	serine incorporator 3	10829.49	15024.93	21807.21
1373042_at	79111	Slc27a5	solute carrier family 27 (fatty acid trans	10824.64	16244.04	9246.378
1371758_at	445268	Ufc1	Ufm1-conjugating enzyme 1	10818.54	5444.496	5032.148
1388452_at	499428	NA	NA	10800.42	4573.88	3001.973
1371835_at	293508	Prkacb_pre	protein kinase, cAMP dependent, cata	10750.67	13492.64	37467.75
1379346_at	304832	Cdc73	cell division cycle 73, Paf1/RNA polym	10748.96	2671.558	9330.14
1388294_at	363061	Sdhd	succinate dehydrogenase complex, su	10745.68	10998.07	8983.004
1386344_at	114506	Ank	progressive ankylosis homolog (mouse	10727.98	11937.88	11166.5
1398884_at	300257	Pfdn5_pred	prefoldin 5 (predicted)	10720.38	17177.98	9072.892
1399123_at	361057	Ddx26	DEAD/H (Asp-Glu-Ala-Asp/His) box po	10713.46	3827.993	19640.28
1367457_at	114558	Becn1	beclin 1 (coiled-coil, myosin-like BCL2-	10712.49	8899.703	7087.123
1371768_at	298681	Ssu72	Ssu72 RNA polymerase II CTD phospl	10708.94	3427.695	4656.419
1372547_at	294709	RGD13065	similar to RIKEN cDNA 2410002O22 g	10708.75	10769.48	5453.783
1388670_at	363288	Kif1a	kinesin family member 1A	10704.55	6496.271	12553.71
1388117_at	171365	Snrpb	small nuclear ribonucleoprotein polype	10693.21	17775.19	5143.272
1371446_at	289014	Mapkapk2	MAP kinase-activated protein kinase 2	10673.09	2275.457	2626.567
1388929_at	502635	RGD15597	similar to HMG-1 (predicted)	10668.73	4082.847	18517.58
1371512_at	362704	Bre	brain and reproductive organ-expresse	10663.09	15251.79	14380.87

1388504_at	314949	MGC11637	NA	10661.02	27529.15	20507.11
1383587_at	300783	RGD15654	similar to Butyrate-induced transcript 1	10619.79	3429.887	1376.418
1368566_a_at	64539	Ndufv3l	NADH dehydrogenase (ubiquinone) fla	10617.83	20925.62	4214.313
1372556_at	502374	LOC50237	hypothetical protein LOC502374	10615.46	13485.37	7499.417
1367485_at	362479	Tcea1	transcription elongation factor A (SII) 1	10614.38	18406.31	17677.09
1389168_at	311456	Mkks	McKusick-Kaufman syndrome protein	10613.73	10093.86	2722.225
1372787_at	305258	Crlz1	charged amino acid rich leucine zipper	10606.31	7841.034	6876.845
1370280_at	24465	Hprt	hypoxanthine guanine phosphoribosyl	10603.48	14033.35	12593.98
1376436_at	315923	Lysmd3	LysM, putative peptidoglycan-binding,	10600.38	2850.707	9055.913
1367503_at	293852	Bcap31	B-cell receptor-associated protein 31	10576.12	18580.95	6056.57
1389611_at	25696	Vldlr	very low density lipoprotein receptor	10561.3	5377.43	2552.814
1375414_at	373541	Taf9	TAF9 RNA polymerase II, TATA box b	10554.53	23652.1	13377.59
1369962_at	81643	Atic	5-aminoimidazole-4-carboxamide ribor	10546.8	5424.48	3973.601
1371558_at	306255	Nisch	nischarin	10545.53	11039.03	7962.099
1375524_at	297867	Arid1a_pre	AT rich interactive domain 1A (Swi1 lik	10538.23	5293.207	14279.07
1372653_at	300211	Fkbp11	FK506 binding protein 11	10515.05	4653.224	2636.999
1368228_at	58821	Zfp265	zinc finger protein 265	10505.37	6049.076	10580.63
1369632_a_at	25559	Abcc8	ATP-binding cassette, sub-family C (C	10497.33	8476.974	8720.834
1369984_at	89786	Cox17	cytochrome c oxidase, subunit XVII as	10493.61	12967.53	11577.43
1387919_at	64476	Mfn2	mitofusin 2	10483.19	4357.921	4924.081
1370250_at	25573	Ube2i	ubiquitin-conjugating enzyme E2I	10468.5	19263.63	8149.464
1388896_at	310960	Usp33	ubiquitin specific peptidase 33	10460.94	4335.132	28501.55
1388341_at	362965	Rangap1	RAN GTPase activating protein 1	10457.48	8993.396	1758.114
1367656_at	85492	Psmb7	proteasome (prosome, macropain) sub	10456.48	28258.91	8038.623
1372827_at	361967	Ppid	peptidylprolyl isomerase D (cyclophilin	10453.09	8165.471	26002.44
1373156_at	367903	Armcx2	armadillo repeat containing, X-linked 2	10444.1	7921.903	11166.5
1369670_at	24560	Cd200	Cd200 antigen	10427.97	17657.69	20797
1368886_at	25579	Map3k12	mitogen activated protein kinase kinas	10422.26	28004.46	3146.983
1388930_at	363013	Tmem123	transmembrane protein 123	10400.94	4204.742	4892.372
1374058_at	246301	Ascl3	achaete-scute complex homolog-like 3	10389.13	8594.014	3928.559
1388682_at	289994	Cnih_predi	cornichon homolog (Drosophila) (predi	10383.04	9354.184	7745.553
1370511_at	24366	Fgb	fibrinogen, B beta polypeptide	10379.23	14.70364	920.3351
1367827_at	24673	Ppp2cb	protein phosphatase 2 (formerly 2A), c	10376.06	16534.08	11704.28
1373955_at	306182	Ranbp5_pr	RAN binding protein 5 (predicted)	10370.45	4998.473	5355.15
1379264_at	362367	Znrf1_pred	zinc and ring finger 1 (predicted)	10368.09	29466.37	11671.43
1383264_at	304759	RGD13064	similar to RAB3 GTPase-activating pro	10360.88	5116.343	9108.313
1372080_at	312444	Immt	inner membrane protein, mitochondria	10356.95	16539.6	5577.808
1388658_at	619345	Surf2	surfeit 2	10354.1	5234.717	7380.167
1380334_at	305340	RGD13597	hypothetical RNA binding protein RGD	10352.96	2929.438	3961.883
1388660_at	302500	Mcts1	malignant T cell amplified sequence 1	10343.3	17300.37	10593.43
1368165_at	29562	Prps1	phosphoribosyl pyrophosphate synthet	10319.88	16370.19	8600.161
1372091_at	404280	Mig12	MID1 interacting G12-like protein	10286.97	23727.81	17964.53
1371366_at	360678	Arhgdia	Rho GDP dissociation inhibitor (GDI) a	10268.39	9334.529	2324.887
1382268_at	293024	Akap13	A kinase (PRKA) anchor protein 13	10264.8	4694.531	2557.555
1367704_at	140670	Ap2b1	adaptor-related protein complex 2, bet	10251.13	8702.205	5270.534
1371944_at	363836	Ube2l3_pre	ubiquitin-conjugating enzyme E2L 3 (p	10226.18	21073.03	4376.289
1376086_at	361151	RGD13111	similar to RIKEN cDNA 1810047C23	10221.88	13314.37	6389.207
1388983_at	296050	RGD1305C	similar to hypothetical protein (predicte	10215.16	14875.6	12970.08
1389292_at	307039	Rab18	RAB18, member RAS oncogene family	10206.72	13067.42	6454
1387221_at	29244	Gch	GTP cyclohydrolase 1	10190.29	5294.982	13139.52
1374177_at	310784	Taf13_prec	TAF13 RNA polymerase II, TATA box	10187.58	5604.45	5654.208

1367995_at	24248	Cat	catalase	10179.36	3435.106	4561.941
1371851_at	289924	Psmd6	proteasome (prosome, macropain) 26S	10173.09	13786.55	12466.97
1376079_at	291369	Dnajc1_pre	DnaJ (Hsp40) homolog, subfamily C, r	10172.47	2769.004	8668.14
1368998_at	65193	Nkx6-1	NK6 transcription factor related, locus	10170.5	3921.089	771.1274
1381100_at	367072	Arhgef12	Rho guanine nucleotide exchange fact	10170.4	1599.534	752.7151
1398883_at	499894	LOC49989	NA	10159.14	21645.81	27450.27
1398798_at	64370	Metap2	methionine aminopeptidase 2	10139.68	19981.06	10371.29
1377807_a_at	289181	RGD1561S	similar to IQ motif and WD repeats 1 (p	10127.61	5215.413	9057.431
1373767_at	360772	Zfand2a	zinc finger, AN1-type domain 2A	10120.87	2781.348	15426.78
1388851_at	291671	Hspa9a_pr	heat shock 70kDa protein 9A (predicte	10109.27	7010.275	5077.094
1386859_at	64524	Tkt	transketolase	10107.82	10519.14	2823.331
1388865_at	297486	Ppp4r2_pre	protein phosphatase 4, regulatory subu	10102.49	22231.84	18429.6
1373958_at	367902	LOC36790	similar to ALEX3 protein	10079.79	11946.95	9022.738
1373127_at	362172	Trim44	tripartite motif protein 44	10064.81	16752.49	12558.69
1370006_at	29478	Ndufs6	NADH dehydrogenase (ubiquinone) Fe	10052.47	19536.26	16942.76
1398900_at	362504	Dctn3_pre	dynactin 3 (predicted)	10052.45	19176.18	4652.166
1371465_at	60465	Cttn	cortactin	10024.17	9481.387	3475.453
1398799_at	117045	Eif4e	eukaryotic translation initiation factor 4	10019.68	12985.38	17013.27
1391393_at	361585	Ube3a_pre	ubiquitin protein ligase E3A (predicted)	10018.42	7251.995	8294.664
1388628_at	300888	Tmed3	transmembrane emp24 domain contai	10016.79	4360.019	5629.354
1386904_a_at	64001	Cyb5	cytochrome b-5	10004.37	17415.2	5648.912
1367530_at	362283	Stx16_pre	syntaxin 16 (predicted)	10003.08	2272.991	2691.708
1399005_at	312754	Ppp2r5a_p	protein phosphatase 2, regulatory subu	9991.384	3859.213	8295.584
1374976_a_at	81782	Soat1	sterol O-acyltransferase 1	9985.99	1671.004	2455.251
1371662_at	292028	Kars	lysyl-tRNA synthetase	9969.587	5384.697	3560.03
1398616_at	302671	Ap1s2_pre	adaptor-related protein complex 1, sig	9966.23	67366.47	20014.57
1388476_at	361655	Tial1_map	Tial1 cytotoxic granule-associated RN	9965.322	6922.23	14173.56
1393172_at	64824	Nab1	Ngfi-A binding protein 1	9957.649	2264.053	2931.223
1373866_at	301416	RGD13595	similar to hypothetical protein FLJ1344	9956.093	4100.967	18636.82
1368103_at	85264	Abcg1	ATP-binding cassette, sub-family G (W	9952.149	7048.915	9198.565
1367837_at	29671	Psma4	proteasome (prosome, macropain) sub	9941.469	8706.177	7008.531
1371996_at	297705	Aebp2_pre	AE binding protein 2 (predicted)	9940.396	5463.569	12473.61
1373391_at	289196	Tmco1	transmembrane and coiled-coil domair	9932.241	6313.594	3986.983
1367462_at	29156	Capns1	calpain, small subunit 1	9930.827	11740.61	4875.363
1371476_at	360915	Cops4	COP9 (constitutive photomorphogenic	9911.339	13361.68	13870.97
1367695_at	64192	Qdpr	quinoid dihydropteridine reductase	9909.67	9030.174	2706.085
1377375_at	296925	Aass_pred	aminoadipate-semialdehyde synthase	9905.447	72.40363	11925.88
1398994_at	288719	Tpst2	tyrosylprotein sulfotransferase 2	9898.96	6696.946	14975.95
1371509_at	25346	Ros1	v-ros UR2 sarcoma virus oncogene ho	9860.119	13649.78	5606.661
1398947_at	298874	Pum2	pumilio 2 (Drosophila)	9834.075	12266.6	25280.33
1368132_at	170842	Tob1	transducer of ErbB-2.1	9825.263	6678.489	23751.7
1397959_at	312248	RGD13107	similar to RIKEN cDNA D130059P03 g	9817.264	3734.871	3416.813
1387806_at	171337	Rap1b	RAS related protein 1b	9812.987	30002.88	18711.94
1398868_at	252928	Timm13	translocase of inner mitochondrial mer	9810.889	12303.75	3343.552
1388564_at	300206	RGD13031	similar to hypothetical protein FLJ2043	9810.286	12208.42	11628.75
1382063_at	363494	Gla_mappe	galactosidase, alpha (mapped)	9800.664	3683.463	3505.7
1389733_at	299851	Mars_pred	methionine-tRNA synthetase (predicte	9798.635	4376.192	4454.829
1388324_at	289222	Nit1	nitrilase 1	9794.125	1944.42	4724.864
1383662_at	500956	LOC50095	hypothetical protein LOC500956	9794.059	5014.245	8940.293
1398869_at	117262	Psmc4	proteasome (prosome, macropain) 26S	9786.256	11715.71	4759.926
1368255_at	50864	Hnt	neurotrimin	9781.158	1370.43	293.3299

1381970_at	361554	Lsm14a_pr	LSM14 homolog A (SCD6, S. cerevisia	9778.655	13135.85	14800.35
1371410_at	288925	RGD15640	similar to cDNA sequence BC056474 (	9764.178	8094.909	3639.178
1388552_at	308909	Smpd1	sphingomyelin phosphodiesterase 1, a	9761.185	5204.55	4442.369
1370262_at	170910	Mtdh	metadherin	9757.205	8941.824	3751.671
1373977_at	311024	Kif5c_pred	kinesin family member 5C (predicted)	9751.967	5182.954	5922.568
1387002_at	89819	G10	maternal G10 transcript	9750.015	13817.18	8885.582
1388974_at	291685	Srp19_pre	signal recognition particle 19 (predicte	9718.028	15133.4	7704.898
1387148_at	171407	Gprasp1	G protein-coupled receptor associated	9702.4	9147.922	33533.28
1367907_a_at	116561	Cltb	clathrin, light polypeptide (Lcb)	9701.184	13070.49	3045.597
1399027_at	117273	Rhoa	ras homolog gene family, member A	9693.213	164.3069	8683.912
1375916_at	311726	Pcmtd2_pr	protein-L-isoaspartate (D-aspartate) O	9681.707	6611.288	5631.442
1389088_at	64622	Adnp	activity-dependent neuroprotective pro	9680.287	20212.37	24901.47
1388362_at	290671	Ndufa13_p	NADH dehydrogenase (ubiquinone) 1	9671.815	21349.67	5952.152
1389012_at	362344	Ndufb2_pre	NADH dehydrogenase (ubiquinone) 1	9666.834	27652.11	16846.71
1389296_at	362905	RGD13104	similar to RIKEN cDNA 4921531G14 (	9647.409	15887.17	8673.133
1388529_at	295395	Rtcd1	RNA terminal phosphate cyclase doma	9644.783	10227.56	10437.53
1392468_at	289400	RGD13109	similar to KIAA1078 protein (predicted)	9624.317	6936.115	8517.821
1399098_at	294320	Glo1	glyoxylase 1	9603.589	17371.13	13696.7
1373399_at	301007	Wdr6	WD repeat domain 6	9602.271	6435.684	12054.97
1377664_at	171457	Kab	KARP-1 binding protein 1	9595.158	4343.942	16894.07
1399143_at	116725	Ube2n	ubiquitin-conjugating enzyme E2N	9593.701	11670.65	15019.56
1378170_at	303132	Aff4_predic	AF4/FMR2 family, member 4 (predicte	9592.406	3524.475	2410.907
1367927_at	25344	Phb	prohibitin	9584.078	18790.04	2218.422
1371411_at	315217	Plxnb2	plexin B2	9567.759	14279.82	5017.876
1369787_at	24889	Cckar	cholecystokinin A receptor	9567.3	609.4706	619.1757
1371648_at	64470	Ddb1	damage-specific DNA binding protein 1	9550.926	13686.4	4583.936
1374043_at	307288	Gramd3	GRAM domain containing 3	9538.511	8882.088	20845.36
1373345_at	300186	Amigo2	adhesion molecule with Ig like domain	9536.386	8715.661	15364.52
1389941_at	498910	Arl2bp	ADP-ribosylation factor-like 2 binding p	9529.364	7708.124	9140.327
1375537_at	114520	Strn3	striatin, calmodulin binding protein 3	9525.087	4709.477	8408.251
1371395_at	297093	Cbx3	chromobox homolog 3 (HP1 gamma h	9521.422	34452.32	21306.95
1388204_at	171052	Mmp13	matrix metallopeptidase 13	9520.085	110.5256	2107.714
1379041_at	170911	Pik3ca	phosphatidylinositol 3-kinase, catalytic	9510.901	4405.957	7163.149
1368888_a_at	83765	Rtn4	reticulon 4	9505.636	22473.62	23773.61
1372094_at	308472	Supt5h	suppressor of Ty 5 homolog (S. cerevi	9505.388	7182.143	5964.165
1368062_at	171126	Ap3m1	adaptor-related protein complex 3, mu	9495.551	14641.58	6261.908
1373842_at	300250	Kb1	type II keratin Kb1	9491.407	7280.891	11941.14
1367734_at	24192	Akr1b4	aldo-keto reductase family 1, member	9472.481	17700.25	5572.75
1367790_at	64635	Snd1	staphylococcal nuclease domain conta	9469.424	6180.186	3084.478
1374585_at	361465	Echdc1	enoyl Coenzyme A hydratase domain	9463.24	14720.1	11052.38
1371872_at	89825	Nap1l1	nucleosome assembly protein 1-like 1	9426.607	27199.67	12416.12
1375189_at	296570	Edf1_predi	endothelial differentiation-related facto	9424.149	14369.23	6819.343
1367795_at	29596	Ifrd1	interferon-related developmental regul	9412.208	3842.762	29369.58
1388445_at	290527	Anxa11	annexin A11	9408.925	5399.485	8838.979
1392918_at	288305	Cct8_predi	chaperonin subunit 8 (theta) (predicted	9406.92	15819.15	10220.47
1371763_at	363016	RGD13095	similar to RIKEN cDNA 4931406C07	9374.082	13848.05	8110.949
1388358_at	292845	Etfb	electron-transfer-flavoprotein, beta poly	9360.16	5948.626	4851.31
1370234_at	25661	Fn1	fibronectin 1	9349.488	23137.17	465.0679
1371334_at	301575	Itm2c	integral membrane protein 2C	9339.976	9470.558	7229.734
1375131_at	295922	Mtch2_pre	mitochondrial carrier homolog 2 (C. ele	9339.4	21061.09	7784.589
1372715_at	364678	Sfxn1	sideroflexin 1	9339.039	62.76561	4028.531

1367477_at	362388	Mrpl53_pre	mitochondrial ribosomal protein L53 (p	9337.972	13188.62	5396.323
1371978_at	298369	Osbpl9_pre	oxysterol binding protein-like 9 (predict	9332.694	5810.745	8518.728
1388371_at	300445	Prkcsh_pre	protein kinase C substrate 80K-H (pred	9317.845	9833.741	4712.433
1374766_at	314997	RGD15647	similar to mKIAA0493 protein (predicte	9312.023	5364.058	5925.924
1373968_at	364686	RGD15619	similar to KB07 protein (predicted)	9310.484	10842.66	27106.77
1388603_a_at	290985	Hbld2	HESB like domain containing 2	9297.883	9822.155	13749.65
1390785_at	362716	Ubxd4_pre	UBX domain containing 4 (predicted)	9289.497	6816.677	6505.009
1384022_at	363266	LOC36326	similar to HIV-1 Rev binding protein	9276.294	9319.698	9337.915
1373585_at	57022	Adam4	a disintegrin and metalloprotease dom	9275.596	6472.845	3268.96
1371724_at	300689	Rexo2	REX2, RNA exonuclease 2 homolog (S	9270.644	6548.014	4061.101
1377576_at	310856	Ppa2_pred	pyrophosphatase (inorganic) 2 (predict	9264.329	3116.786	4808.673
1374515_at	290303	RGD13064	similar to RIKEN cDNA 6330409N04	9261.397	4574.001	4620.883
1393186_at	499690	RGD15644	similar to RIKEN cDNA 2010200O16 (	9260.164	5360.024	4919.557
1367467_at	288740	RGD13095	similar to nitrogen fixation cluster-like (	9257.933	9811.8	9786.827
1377691_at	310710	Sec22l1	SEC22 vesicle trafficking protein-like 1	9254.925	4615.801	3661.422
1398801_at	171456	Cdk105	CDK105 protein	9237.02	19517.93	21511.26
1388574_at	314442	Wars	tryptophanyl-tRNA synthetase	9234.028	12649.99	1796.459
1383429_at	311078	LOC31107	NA	9200.701	9400.343	8319.523
1372262_at	500344	NA	NA	9191.888	11781	14857.25
1389170_at	64026	Casp7	caspase 7	9190.535	788.5312	1920.176
1389463_at	25521	Prkar1b	protein kinase, cAMP dependent regul	9173.719	7306.678	5702.535
1392912_at	289144	Cacybp	calcyclin binding protein	9169.76	5837.825	29930.64
1371796_at	295701	Tmx2	thioredoxin-related transmembrane pro	9169.067	10640.12	4893.117
1388903_at	363448	Tcte1l	T-complex associated-testis-expressed	9154.977	8679.403	14594.67
1373803_a_at	25235	Ghr	growth hormone receptor	9154.093	3112.934	6063.671
1371526_at	288710	Rnf10	ring finger protein 10	9153.569	6050.468	2613.343
1386923_at	81816	Ube2b	ubiquitin-conjugating enzyme E2B, RA	9146.971	9452.966	37442.88
1367710_at	29614	Psme2	proteasome (prosome, macropain) 28	9144.243	7617.563	4777.361
1399082_at	59303	Tmem33	transmembrane protein 33	9141.775	6683.832	8778.13
1389810_at	365668	RGD13059	similar to RIKEN cDNA 2610528A15	9122.381	6282.844	4936.929
1371799_at	367562	Gaa	glucosidase, alpha, acid	9120.973	7014.638	3824.436
1398807_at	24667	Ppm1b	protein phosphatase 1B, magnesium c	9119.266	9886.718	15416.73
1373820_at	360860	RGD13047	similar to constitutive photomorphogen	9105.892	3518.576	5249.177
1386321_s_at	246273	Trib3	tribbles homolog 3 (Drosophila)	9101.628	541.0949	1432.576
1367698_a_at	83788	9-Sep	septin 9	9100.444	5906.14	2656.22
1398803_at	29489	Dync1h1	dynein cytoplasmic 1 heavy chain 1	9083.414	4864.313	5912.963
1372671_at	499328	Rfk	riboflavin kinase	9078.574	12762.91	6466.335
1370004_at	29384	H2afy	H2A histone family, member Y	9077.506	18498.94	3166.748
1368067_at	58820	Zfp148	zinc finger protein 148	9071.035	18216.46	9737.876
1373277_at	292995	Tm2d3_pre	TM2 domain containing 3 (predicted)	9063.315	7298.224	7605.909
1388855_at	362356	Cul1_predi	cullin 1 (predicted)	9049.402	15490.57	12644.18
1378964_at	309196	Ttc9c	tetratricopeptide repeat domain 9C	9047.283	12538.95	8337.435
1372561_at	298500	Smap1l	stromal membrane-associated protein	9042.253	6683.913	4035.862
1398861_at	59087	Nxf1	nuclear RNA export factor 1	9039.698	4620.713	10535.81
1382056_at	502603	LOC50260	similar to splicing factor p54	9026.691	1591.929	4100.102
1382689_at	499242	NA	NA	9018.259	8400.172	11499.94
1374208_at	360466	Zc3h7a_pr	zinc finger CCCH type containing 7 A (	9018.134	6234.775	7860.783
1371338_at	292788	Psenen	presenilin enhancer 2 homolog (C. ele	9016.42	9808.124	6831.139
1388667_at	25478	Man2a1	mannosidase 2, alpha 1	9013.65	3485.358	5927.932
1374423_at	295398	Hiat1_pred	hippocampus abundant gene transcrip	8998.595	4729.214	22849.84
1371581_at	287615	Vezf1_prec	vascular endothelial zinc finger 1 (prec	8997.041	17466.45	8250.591

1371619_at	303122	RGD13103	similar to HTGN29 protein; keratinocyt	8992.581	9696.008	7435.768
1375280_at	289277	PNAS-4	CGI-146 protein	8986.423	4119.223	3989.235
1388431_at	361295	Ss18	synovial sarcoma translocation, Chrom	8971.479	9593.145	5330.768
1388353_at	288778	Pa2g4	proliferation-associated 2G4	8960.613	5509.711	1968.714
1370827_at	171015	Cyb5r4	cytochrome b5 reductase 4	8953.479	6668.224	4172.758
1373822_at	502367	RGD15653	similar to RIKEN cDNA 1110025L05 (p	8916.871	8526.275	2877.817
1387077_at	60336	Arpp19	cAMP-regulated phosphoprotein 19	8888.746	2917.59	8753.096
1388403_at	361596	LOC36159	NA	8888.38	14539.66	4561.387
1387900_at	192260	Cdipt	CDP-diacylglycerol--inositol 3-phospha	8880.696	7131.51	3961.874
1371657_at	308508	Uble1b	ubiquitin-like 1 (sentrin) activating enzy	8877.29	13364.95	13485.3
1372665_at	293820	Psat1	phosphoserine aminotransferase 1	8873.816	11797.07	3532.267
1367494_at	299198	RGD13108	similar to CGI-35 protein (predicted)	8854.085	13451.4	12448.31
1372473_at	292994	Tjp1_predi	tight junction protein 1 (predicted)	8847.399	4279.959	16686.58
1376418_a_at	306804	Iars_predic	isoleucine-tRNA synthetase (predicted	8826.944	3679.395	10456.24
1371091_at	207125	LOC20712	unknown protein	8821.803	6176.44	19626.89
1372255_at	287191	Rars_predi	arginyl-tRNA synthetase (predicted)	8821.428	11619.06	6346.046
1392471_at	114120	Gng12	guanine nucleotide binding protein (G	8811.801	5482.643	7010.509
1367667_at	83791	Fdps	farensyl diphosphate synthase	8802.72	35964.68	6180.319
1399102_at	303740	RGD13063	similar to hypothetical protein MGC155	8797.675	4659.794	3902.22
1388328_at	499619	RGD15604	similar to Eukaryotic translation initiatic	8794.119	18197.58	5178.562
1371488_at	499125	RGD15620	RGD1562079 (predicted)	8790.151	8430.278	2683.334
1390382_at	311359	RGD13114	similar to RIKEN cDNA 2310003F16 (p	8787.753	9265.294	7198.628
1388719_at	114590	Ubqln1	ubiquilin 1	8787.263	17861.04	8907.314
1371483_at	310378	Nnt_mappe	nicotinamide nucleotide transhydrogen	8780.046	10576.43	13825.61
1376650_at	299258	Golga5	golgi autoantigen, golgin subfamily a, 5	8772.185	3945.04	3577.984
1369982_at	81637	Ap2a2	adaptor protein complex AP-2, alpha 2	8761.303	2851.066	1524.896
1388869_at	361255	Tbce	tubulin-specific chaperone e	8755.511	8762.433	6541.439
1367958_at	79249	Abi1	abl-interactor 1	8755.465	5304.917	1690.513
1388352_at	362228	Nat5_predi	N-acetyltransferase 5 (ARD1 homolog	8754.453	9004.537	5199.727
1399095_at	301442	Sumo1	SMT3 suppressor of mif two 3 homolog	8753.732	9210.322	17357.75
1388508_at	498600	RGD15612	similar to RIKEN cDNA 2510049I19 (p	8742.898	5398.856	2137.999
1373959_at	315648	Ppp2r1b	protein phosphatase 2 (formerly 2A), re	8734.697	4558.691	8097.221
1393907_at	287730	Pyy_mappe	peptide YY (mapped)	8729.438	26.51651	54027.58
1389903_at	365548	Pttg1ip	pituitary tumor-transforming 1 interacti	8723.718	4624.986	9879.993
1371645_at	287470	Sdf2_predi	stromal cell derived factor 2 (predicted	8721.467	6877.802	4642.531
1388965_at	299147	Ppp2r5e_p	protein phosphatase 2, regulatory subu	8716.468	2928.278	11233.77
1386930_at	83499	Psmd4	proteasome (prosome, macropain) 26S	8707.743	10005.78	2100.315
1390392_at	304731	RGD13096	similar to RIKEN cDNA 2500001K11 (p	8705.332	8628.462	7058.311
1376733_at	303926	Igsf11	immunoglobulin superfamily, member	8704.933	1423.94	942.5822
1371633_at	296320	Ctnnbl1	beta catenin-like 1	8704.36	7415.892	3962.187
1389534_at	295686	Ube2e3_pr	ubiquitin-conjugating enzyme E2E 3, U	8695.824	18710.63	50232.54
1388651_at	289508	Rchy1	ring finger and CHY zinc finger domain	8691.697	12258.25	13067.43
1388754_at	296384	RGD13063	similar to Ab1-133	8688.786	5744.689	10192.6
1375636_at	116549	Csnk2a1	casein kinase II, alpha 1 polypeptide	8677.684	6338.252	5451.599
1388820_at	315792	RGD13075	similar to modulator of estrogen induce	8659.116	4972.719	10704.21
1398873_at	80846	Hnrpl	heterogeneous nuclear ribonucleoprote	8657.777	12613.85	13011.05
1375269_at	500355	NA	NA	8642.625	11042.05	4858.998
1368365_at	65183	Aldh3a2	aldehyde dehydrogenase family 3, sub	8641.21	14560.37	8139.67
1373040_at	293427	Eif3s5_pre	eukaryotic translation initiation factor 3	8637.906	16612.41	14281.36
1387076_at	29560	Hif1a	hypoxia inducible factor 1, alpha subur	8626.737	5987.442	18106.29
1371827_at	361712	Syvn1	synovial apoptosis inhibitor 1, synoviol	8622.849	2922.708	4364.77

1398779_at	81824	Arpc1a	actin related protein 2/3 complex, subu	8621.336	12249.78	7354.11
1377347_at	313554	Foxj3_prec	forkhead box J3 (predicted)	8621.161	10064.6	12790.55
1376298_at	313449	Upf3b_pre	UPF3 regulator of nonsense transcript	8620.595	5315.462	12906.25
1383798_at	313430	MGC9414	similar to CDNA sequence BC026682	8617.405	379.4215	1139.964
1398351_at	360471	Usp7	ubiquitin specific protease 7 (herpes vi	8612.772	6288.96	4062.532
1398783_at	117039	Gps1	G protein pathway suppressor 1	8607.847	11701.96	3016.199
1373983_at	360807	LOC36080	LOC360807	8607.326	10069.25	27750.19
1372773_at	296562	Npdc1	neural proliferation, differentiation and	8597.444	6387.929	4365.39
1398897_at	296390	Ube2v1_pr	ubiquitin-conjugating enzyme E2 varia	8597.093	17752.34	9843.418
1390125_at	361043	Tm9sf1	transmembrane 9 superfamily member	8578.622	4863.11	4079.154
1373474_at	361980	RGD15657	similar to RIKEN cDNA 2810403A07	8574.831	7265.357	14486.94
1393643_at	362182	Rcn1_pred	reticulocalbin 1 (predicted)	8564.215	8737.36	8438.143
1372115_at	363190	NA	NA	8552.173	3237.909	4971.932
1377125_at	313409	Dnajc6_pre	DnaJ (Hsp40) homolog, subfamily C, r	8536.252	13129.92	18504.91
1398865_at	192356	Unc50	unc-50 homolog (C. elegans)	8533.065	10924.82	9765.05
1398895_at	361171	Golga7	golgi autoantigen, golgin subfamily a, 7	8528.735	11965.07	8950.417
1392180_at	24790	Sp1	Sp1 transcription factor	8525.342	4404.804	2387.821
1368867_at	59117	Eif2c2	eukaryotic translation initiation factor 2	8495.51	2387.115	1375.846
1376209_at	315166	RGD13113	similar to hypothetical protein supporte	8492.158	18084.78	4935.062
1370176_at	171086	Trak2	trafficking protein, kinesin binding 2	8489.425	1368.235	1572.331
1399050_at	289276	Adss_pred	adenylosuccinate synthetase, non mus	8488.836	7294.855	15659.52
1389519_at	292766	Psmd8	proteasome (prosome, macropain) 26S	8482.008	13777.76	1949.571
1392905_at	80850	Gng2	guanine nucleotide binding protein, ga	8475.519	495.8919	686.2597
1388722_at	361384	Dnajb1_pre	DnaJ (Hsp40) homolog, subfamily B, r	8475.038	3772.664	26781.47
1392908_at	595134	LOC59513	NA	8464.248	13582.46	24138.6
1371365_at	292588	Ube2s_pre	ubiquitin-conjugating enzyme E2S (pre	8462.866	23855.64	4035.112
1383126_at	24185	Akt1	thymoma viral proto-oncogene 1	8457.462	6400.964	4182.98
1372706_at	294673	Hexb	hexosaminidase B	8454.052	8322.234	16225.29
1388323_at	362440	Ndufa9	NADH dehydrogenase (ubiquinone) 1	8453.398	22837.53	4682.177
1373179_at	305122	RGD13069	similar to mKIAA1107 protein (predicte	8449.506	8876.979	3205.069
1372938_at	303518	RGD13047	similar to RIKEN cDNA 6330509G02	8448.55	24965.18	24049.84
1387779_at	60571	Mybbp1a	MYB binding protein (P160) 1a	8446.154	6352.388	2800.683
1370295_at	191575	Nme1	expressed in non-metastatic cells 1	8433.237	22644.62	4959.19
1370684_s_at	501546	LOC50154	hypothetical protein LOC501546	8431.385	5408.293	6103.284
1395316_at	367767	Mageh1	melanoma antigen, family H, 1	8423.659	2957.143	42862.75
1368842_at	84382	Tcf4	transcription factor 4	8398.203	22.57637	4644.99
1388566_at	29278	Lasp1	LIM and SH3 protein 1	8393.678	14095.14	7481.725
1375641_at	296710	Arpc5l	actin related protein 2/3 complex, subu	8389.315	7251.897	2866.47
1371598_at	312670	Adipor2	adiponectin receptor 2	8380.537	7991.338	6535.541
1367484_at	361013	Ube2e2	ubiquitin-conjugating enzyme E2E 2 (U	8377.067	18652.76	13664.16
1375452_at	299078	Mgea6_pre	meningioma expressed antigen 6 (coile	8364.197	1808.874	14553.92
1374790_at	287134	Gnptg	N-acetylglucosamine-1-phosphotransfe	8351.07	5164.053	5066.122
1388948_at	293150	Stard10	START domain containing 10	8345.717	5680.562	3071.726
1367663_at	29630	Psme1	proteasome (prosome, macropain) 28	8342.221	6903.357	11678.06
1372659_at	297342	Kcmf1	potassium channel modulatory factor 1	8340.271	13329.51	8400.867
1370911_at	116633	Akap8	A kinase (PRKA) anchor protein 8	8335.34	6924.381	7916.282
1388751_at	116653	Cap2	CAP, adenylate cyclase-associated pro	8328.013	9679.831	7353.272
1370207_at	171082	Atp5g2	ATP synthase, H+ transporting, mitoch	8324.747	26176.55	3605.539
1370865_at	25179	Idh3g	isocitrate dehydrogenase 3 (NAD), gar	8318.777	21304.84	10695.68
1374472_at	290775	MGC11626	similar to DNA segment, Chr 8, ERATO	8316.361	4913.714	6418.469
1388867_at	361178	MGC11283	similar to transcription factor	8315.899	18734.32	14827.18

1389488_at	498544	LOC49854	hypothetical protein LOC498544	8314.836	4820.694	6429.341
1375630_at	300092	MGC7293	similar to NHP2-like protein 1 (High mo	8307.691	20553.44	6821.442
1368063_a_at	170956	Yt521	splicing factor YT521-B	8294.874	9630.82	19214.9
1370870_at	24552	Me1	malic enzyme 1	8291.425	7372.831	11204.04
1371346_at	297990	Ndufb6_pre	NADH dehydrogenase (ubiquinone) 1	8287.63	18477.71	9926.008
1392486_at	312132	Tmem106b	transmembrane protein 106B	8281.522	5092.046	8657.531
1383435_at	245956	Scn3b	sodium channel, voltage-gated, type II	8271.962	9824.981	22778.42
1368947_at	25112	Gadd45a	growth arrest and DNA-damage-induci	8269.255	8324.241	8337.605
1375874_at	361448	RGD13097	similar to cDNA sequence BC013529 (	8266.11	4358.903	6781.912
1373406_at	311854	Tor1b	torsin family 1, member B	8263.37	6955.689	2698.162
1372739_at	362890	Tspan31	tetraspanin 31	8259.706	5004.924	3712.385
1388792_at	291005	Gadd45g	growth arrest and DNA-damage-induci	8232.568	20618.64	5806.922
1387865_at	497778	Dut	deoxyuridine triphosphatase	8232.534	19349.66	10012.26
1398330_at	25558	Stxbp1	syntaxin binding protein 1	8220.398	11297.82	10298.97
1372127_at	313169	Ubap2_pre	ubiquitin-associated protein 2 (predicte	8215.246	8261.694	6561.313
1388528_at	292747	Fbl	fibrillarin	8213.76	17373.45	4134.257
1371590_s_at	500954	LOC50095	Ubiquitin-Like 5 Protein	8205.409	7019.605	16333.35
1381193_at	317456	LOC31745	hypothetical LOC317456	8192.954	2597.95	2368.574
1388926_at	316249	Enpp5	ectonucleotide pyrophosphatase/phos	8191.788	12569.56	12200.67
1376862_at	298652	Ube4b_pre	ubiquitination factor E4B, UFD2 homol	8188.544	2335.158	6169.519
1398925_at	309656	RGD13078	similar to RIKEN cDNA 1300018I05	8185.37	14627.05	2489.989
1373458_at	501624	Bex4	brain expressed X-linked 4	8180.756	13257.08	7976.898
1371948_at	288238	Pigp_predi	phosphatidylinositol glycan, class P (pr	8176.778	15025.16	12068.39
1373609_at	364906	Mrps21_pr	mitochondrial ribosomal protein S21 (p	8170.713	10533.11	8878.877
1370182_at	29714	Ptprn2	protein tyrosine phosphatase, receptor	8161.134	16673.68	18166.54
1367533_at	307545	Statip1	signal transducer and activator of trans	8157.007	13960.18	6612.374
1393136_at	313618	LOC31361	macoilin	8140.434	3981.929	2386.382
1398887_at	295234	RGD15625	similar to Late endosomal/lysosomal M	8112.028	13546.91	5601.529
1370030_at	29739	Gclm	glutamate cysteine ligase, modifier sub	8099.558	1529.236	4902.453
1374548_at	497901	LOC49790	similar to Liver-expressed antimicrobia	8097.479	7331.684	30860.85
1387196_at	64015	Khdrbs3	KH domain containing, RNA binding, s	8092.599	8516.011	8061.161
1373620_at	294400	RGD13078	similar to hypothetical protein D10Ertd	8083.009	7058.937	6909.748
1379243_at	315167	Ndufa6_pre	NADH dehydrogenase (ubiquinone) 1	8082.746	17476.05	10601.71
1373565_at	171379	Smarca4	SWI/SNF related, matrix associated, a	8070.915	7004.196	5884.886
1384308_at	305535	Meis1_pred	Meis1, myeloid ecotropic viral integrati	8063.422	468.2521	20544.82
1381925_x_at	282546	Arid1b	AT rich interactive domain 1B (Swi1 lik	8059.352	6446.704	18544.85
1395454_at	499474	LOC49947	similar to osteopetrosis associated tran	8058.022	4286.945	6071.267
1393364_at	266733	Slc12a8	solute carrier family 12 (potassium/chlo	8055.436	561.2049	817.3167
1371941_at	301563	RGD13102	similar to RIKEN cDNA 5230400G24	8051.985	6246.909	9020.031
1382384_at	305251	MGC11441	similar to Ras association (RalGDS/AF	8047.953	8131.143	1904.039
1388344_at	362819	RGD13056	similar to RIKEN cDNA 1110005A23	8042.808	18495.44	14700.35
1367932_at	29637	Hmgcs1	3-hydroxy-3-methylglutaryl-Coenzyme	8034.903	15636.06	33760.2
1389654_at	315926	Pls1_predi	plastin 1 (I isoform) (predicted)	8031.215	6811.781	14832.86
1372674_at	312603	Rybp_pred	RING1 and YY1 binding protein (predic	8026.02	20654.1	31289.37
1388118_at	63938	Hibadh	3-hydroxyisobutyrate dehydrogenase	8012.037	5939.864	9088.91
1371764_at	298689	MGC94113	similar to Ubc6p homolog	8010.358	3546.425	5678.828
1373874_at	81536	Sgpp1	sphingosine-1-phosphate phosphatase	8009.979	1568.764	5308.153
1372267_at	296651	Psmd5_pre	proteasome (prosome, macropain) 26S	8008.154	6625.789	8594.268
1367991_at	78947	Gcs1	glucosidase 1	8007.718	5937.666	1554.859
1368912_at	25569	Trh	thyrotropin releasing hormone	8002.58	49.98892	61.24318
1369630_at	25368	Adk	adenosine kinase	7999.426	12041.91	5758.676

1383986_at	54234	Cacna1e	calcium channel, voltage-dependent, L	7990.22	6250.763	6893.827
1383137_at	364712	Sox4_pred	SRY-box containing gene 4 (predicted	7983.8	19945.46	6855.985
1389518_at	362511	Fbxo10_pr	F-box only protein 10 (predicted)	7982.692	5668.953	4485.554
1390020_at	360975	LOC36097	similar to oxoglutarate dehydrogenase	7981.835	5830.346	3367.846
1388514_at	259229	Ppm1g	protein phosphatase 1G (formerly 2C),	7980.324	9027.666	2847.879
1375644_at	498824	RGD1562C	similar to WAC (predicted)	7979.329	4425.603	3981.808
1398777_at	29666	Psmb6	proteasome (prosome, macropain) sub	7966.293	18249.87	6852.615
1371513_at	500067	NA	NA	7954.271	7484.375	7719.109
1370745_at	25548	Slc34a1	solute carrier family 34 (sodium phosp	7951.238	4760.672	3838.915
1373861_at	361089	Ndfip2_pre	Nedd4 family interacting protein 2 (pre	7949.221	4616.028	20699.02
1398385_at	305714	RGD13054	similar to RIKEN cDNA 1500006O09 (	7936.442	15327.87	24805.95
1370055_at	140665	Rab3d	RAB3D, member RAS oncogene famil	7934.329	11406.29	2437.432
1371355_at	296658	Ndufa8	NADH dehydrogenase (ubiquinone) 1	7929.918	21482.67	4792.951
1398411_at	364357	NA	NA	7917.924	4598.028	3134.349
1375411_at	299643	Ndufa7_pr	NADH dehydrogenase (ubiquinone) 1	7900.411	19238.82	7197.667
1398903_at	290401	Esd_mapp	esterase D/formylglutathione hydrolase	7892.43	7669.355	6909.137
1372036_at	293505	Cd2bp2_pr	CD2 antigen (cytoplasmic tail) binding	7885.552	8100.655	8078.102
1398806_at	29525	Pitpna	phosphatidylinositol transfer protein, al	7882.496	9995.324	2713.168
1389188_at	24232	C3	complement component 3	7880.639	5286.316	2993.326
1375852_at	25675	Hmgcr	3-hydroxy-3-methylglutaryl-Coenzyme	7873.521	16196.89	39790.8
1372522_at	290912	RGD1310C	similar to hypothetical protein FLJ1015	7871.975	10945.25	13825.95
1373030_at	501594	NA	NA	7870.135	6679.741	10743.98
1388835_at	308392	Strn4_pred	striatin, calmodulin binding protein 4 (p	7870.003	11283.85	6121.098
1371567_at	291450	Aldh7a1	aldehyde dehydrogenase family 7, me	7864.908	11701.96	2561.218
1389444_at	305923	RGD13057	similar to RIKEN cDNA 5033406L14	7863.404	4259.463	3494.886
1373891_at	313436	Zcchc12	zinc finger, CCHC domain containing 1	7854.816	8959.828	17475.46
1372626_at	498994	NA	NA	7853.298	3729.698	1003.693
1373380_at	498247	RGD15621	similar to brain Zn-finger protein (predi	7846.365	9697.271	6511.095
1367681_at	64315	Cd151	CD151 antigen	7844.613	15215.32	7046.352
1383141_a_at	365407	RGD13054	similar to RIKEN cDNA 5730453I16	7842.42	7171.184	8592.65
1368083_at	84389	Ccnh	cyclin H	7837.623	5048.776	16042.01
1368122_at	84508	Rnf103	ring finger protein 103	7834.311	10900.15	10942.29
1383054_at	246282	Zfp91	zinc finger protein 91	7834.069	10463.63	2418.498
1371610_at	290794	Tnks_predi	tankyrase, TRF1-interacting ankyrin-re	7825.081	8751.642	16252.34
1373231_at	363452	Otud5	OTU domain containing 5	7821.619	4091.829	5205.864
1388382_at	361985	LOC36198	similar to NICE-3	7816.046	8876.839	14963.84
1368361_a_at	117063	Ptpn2	protein tyrosine phosphatase, non-rece	7815.89	6980.504	15879.73
1379481_at	116697	Pabpn1	poly(A) binding protein, nuclear 1	7813.856	10404.26	5632.572
1375527_at	65027	Chd8	chromodomain helicase DNA binding p	7813.476	3789.826	5012.707
1387019_at	140608	Atp5i	ATP synthase, H+ transporting, mitoch	7805.277	9833.677	7703.844
1374870_at	298101	Col27a1	procollagen, type XXVII, alpha 1	7783.572	1442.473	21088.07
1382254_at	64183	Pde4dip	phosphodiesterase 4D interacting prot	7783.13	4444.135	24917.68
1389912_at	60334	Ensa	endosulfine alpha	7777.109	3462.263	1896.473
1386882_at	83462	Tctex1	t-complex testis expressed 1	7767.634	27563.71	6779.75
1372720_at	293060	Btbd1	BTB (POZ) domain containing 1	7767.237	8814.837	20633.63
1373374_at	362051	Lmo4	LIM domain only 4	7759.69	2109.211	17080.18
1370305_at	171441	Yif1	Yip1 interacting factor homolog (S. cer	7755.297	4590.519	1953.984
1398894_at	291339	Commd3	COMM domain containing 3	7755.251	19769.92	16047.98
1378595_at	252855	Sfpq	splicing factor proline/glutamine rich (p	7751.39	7048.378	32501.09
1375211_at	292306	Rnaset2_p	ribonuclease T2 (predicted)	7750.703	7307.638	14994.13
1376705_a_at	361208	Rab24	RAB24, member RAS oncogene family	7748.782	5496.039	3690.106

1373024_at	302290	Ap3s1_pre	adaptor-related protein complex 3, sigı	7748.78	11996.38	11291.72
1376029_at	294283	Rab2l	RAB2, member RAS oncogene family-	7736.98	11694.19	10014.35
1375213_at	361042	Pck2_pred	phosphoenolpyruvate carboxykinase 2	7728.007	2078.566	1433.979
1370025_at	140607	Pip5k2c	phosphatidylinositol-4-phosphate 5-kin	7727.111	8862.182	3337.354
1367798_at	29443	Ahcy	S-adenosylhomocysteine hydrolase	7723.988	6606.29	2132.549
1372230_at	292792	RGD13047	similar to seven transmembrane doma	7717.098	12637.97	3081.67
1388408_at	363004	RGD13071	similar to RIKEN cDNA 1110020C13	7713.762	6288.357	4967.013
1373824_at	292027	Cfdp1	craniofacial development protein 1	7698.526	3837.65	3945.41
1388852_at	361578	RGD13052	similar to pM5 protein; DNA segment,	7694.407	7700.567	1683.056
1372815_at	298385	Magoh_pre	mago-nashi homolog, proliferation-ass	7693.848	16911.86	11722.19
1367759_at	24437	H1f0	H1 histone family, member 0	7688.755	6416.521	1058.358
1386996_at	50685	Mrlcb	myosin light chain, regulatory B	7687.992	7629.756	14022.77
1372431_at	303746	Mrpl12	ribosomal protein, mitochondrial, L12	7687.116	5450.776	2018.431
1375339_at	360515	Enth	enthoprotin	7669.155	6776.986	4160.396
1382045_at	366896	Tbc1d15	TBC1 domain family, member 15	7657.819	8317.114	34041.67
1379327_at	307766	Smarca5_p	SWI/SNF related, matrix associated, a	7653.572	3506.253	10465.79
1390257_at	60431	Vapb	vesicle-associated membrane protein,	7648.191	5049.625	2654.017
1367697_at	81649	Mapk14	mitogen activated protein kinase 14	7648.189	5958.526	6808.307
1375215_x_at	290648	Pgpep1	pyroglutamyl-peptidase I	7646.827	7702.798	6840.756
1368072_at	54230	Btg3	B-cell translocation gene 3	7644.903	12465.68	15212.02
1390777_at	114100	Sc5d	sterol-C5-desaturase (fungal ERG3, de	7642.781	3468.148	7155.912
1388685_at	360742	Dgcr2	DiGeorge syndrome critical region gen	7641.295	6130.714	3660.582
1372504_at	502017	RGD15618	similar to dolichyl-phosphate mannosy	7616.013	9738.672	4644.142
1398952_at	288920	RGD15640	similar to RIKEN cDNA 2310036O22 (	7613.146	4906.013	10517.64
1398417_at	316131	Fem1a	feminization 1 homolog a (C. elegans)	7606.209	3464.312	3505.364
1369085_s_at	113938	Snurf	SNRPN upstream reading frame	7597.95	22340.13	3149.345
1398975_at	301512	Aamp_pred	angio-associated migratory protein (pr	7585.837	4007.509	2342.046
1399033_at	361391	Cbfb	core binding factor beta	7584.061	10873.47	16668.72
1377577_at	295088	Gmps	guanine monphosphate synthetase	7582.977	4554.585	5046.414
1388728_at	315047	Laptm4b	lysosomal-associated protein transmer	7578.515	9728.863	6396.171
1368124_at	171109	Dusp5	dual specificity phosphatase 5	7576.142	672.1654	2287.735
1373985_at	308393	RGD15604	similar to KIAA1183 protein (predicted)	7570.296	11754.23	5342.869
1371244_at	292072	Aprt_predic	adenine phosphoribosyl transferase (p	7568.618	7835.805	7327.595
1398780_at	83583	Rabac1	Rab acceptor 1 (prenylated)	7564.701	12363.72	4890.867
1371549_at	305895	Wdr23	WD repeat domain 23	7563.959	3970.098	5025.501
1371646_at	362660	Pgd_mapp	phosphogluconate dehydrogenase (ma	7561.314	2000.206	1516.936
1388988_at	300983	Abhd14b	abhydrolase domain containing 14b	7547.918	8529.291	5383.203
1368215_at	83534	Tpp1	tripeptidyl peptidase I	7539.565	4140.798	4327.713
1377708_at	499339	LOC49933	hypothetical protein LOC499339	7529.849	3703.59	1335.233
1395590_at	501485	LOC50148	NA	7528.224	1314.559	3234.792
1373059_at	360823	Ankrd13	ankyrin repeat domain 13	7528.162	7733.883	6855.31
1388981_at	503165	LOC50316	similar to ADP-ribosylation factor GTPa	7526.54	2625.147	5678.787
1374842_at	362686	Cebpz_pre	CCAAT/enhancer binding protein zeta	7521.309	4785.323	10152.43
1389393_at	361601	RGD13082	similar to RIKEN cDNA 2210412D01	7517.275	8179.997	9820.353
1372459_at	361517	Vasp_pred	vasodilator-stimulated phosphoprotein	7504.192	4961.013	5109.262
1399043_at	493810	Capza2	capping protein (actin filament) muscle	7498.226	8172.501	9565.369
1367844_at	81664	Gnai2	guanine nucleotide binding protein, alp	7497.121	3685.682	2586.413
1379374_at	295401	Prg1	plasticity related gene 1	7493.62	9462.878	2248.612
1388346_at	291963	RGD15609	similar to HSPC171 protein (predicted)	7490.146	6364.811	2547.704
1382945_at	290500	RGD13047	similar to cDNA sequence BC006662	7480.394	3519.638	3343.66
1371584_at	362247	Trpc4ap	transient receptor potential cation char	7478.437	6422.302	2054.13

1374333_at	361224	RGD1306C	similar to RIKEN cDNA 1110007C09 (p	7471.023	17208.76	3172.654
1388136_at	171139	Timm9	translocase of inner mitochondrial mem	7469.059	3150.903	4955.925
1374602_at	29544	Tspyl	testis-specific protein, Y-encoded-like	7468.707	5783.713	29187.38
1372498_at	307649	Ciapin1	cytokine induced apoptosis inhibitor 1	7455.812	8005.587	3616.112
1377029_at	300807	Rora_predi	RAR-related orphan receptor alpha (pr	7452.028	19.19852	5933.777
1372865_at	362002	Zfp364_pre	zinc finger protein 364 (predicted)	7450.874	9994.707	10476.31
1373203_at	361213	Sptlc1_pre	serine palmitoyltransferase, long chain	7447.257	12591.98	5040.922
1373382_at	298675	RGD13063	similar to RER1 homolog	7441.146	5604.775	4250.409
1399013_at	296049	RGD13109	similar to RIKEN cDNA 2610318K02 (p	7438.287	10021.24	1965.208
1393317_at	500288	RGD15608	similar to THUMP domain containing 3	7437.277	4309.804	2334.713
1368402_at	81655	Dncli2	dynein, cytoplasmic, light intermediate	7418.297	6145.973	14728.99
1389062_at	291659	Ik	IK cytokine	7410.151	10401.27	6258.375
1392983_at	287772	Psmd12	proteasome (prosome, macropain) 26S	7405.496	10525.14	7492.478
1372767_at	501123	NA	NA	7404.624	8214.471	20333.72
1367525_at	313591	Thrap3	thyroid hormone receptor associated p	7400.97	7417.408	8859.816
1388797_at	308335	U2af2	U2 small nuclear ribonucleoprotein aux	7400.343	9509.192	5269.097
1372610_at	360526	P4ha2_pre	procollagen-proline, 2-oxoglutarate 4-d	7398.808	3539.244	2983.764
1398938_at	24161	Acp1	acid phosphatase 1, soluble	7398.233	6794.994	2890.102
1392916_at	293016	Mtap7_pre	microtubule-associated protein 7 (pred	7395.466	5244.645	7332.067
1377091_at	406167	Ng5	NG5 protein	7394.322	5203.92	9545.384
1372083_at	289561	Polr2b_pre	polymerase (RNA) II (DNA directed) po	7393.62	8636.008	5154.666
1367848_at	29167	Dctn1	dynactin 1	7393.243	14854.84	3044.324
1374825_at	308988	Sez6l2_pre	seizure related 6 homolog (mouse)-like	7385.293	5333.118	5727.134
1377731_at	289810	RGD15606	similar to small unique nuclear recepto	7382.779	8567.377	19308.14
1368405_at	81757	Rala	v-ral simian leukemia viral oncogene h	7379.971	5164.742	2998.252
1375212_at	362811	Ankrd52_p	ankyrin repeat domain 52 (predicted)	7367.906	8137.572	1271.14
1398957_at	315362	RGD15663	similar to cell division cycle 2-like 1 (pr	7361.684	2721.797	6709.527
1388343_at	361385	Ndufb7_pre	NADH dehydrogenase (ubiquinone) 1	7351.081	7404.963	6203.707
1389141_at	301430	Orc2l	origin recognition complex, subunit 2-li	7337.589	3427.98	11192.33
1388455_at	114119	Gng10	guanine nucleotide binding protein (G	7334.327	18858.82	26490.04
1371449_at	298696	Pin1_predi	protein (peptidyl-prolyl cis/trans isomer	7329.787	12711.59	2335.816
1373563_at	362392	RGD13109	similar to putative nucleic acid binding	7315.912	14180.42	7415.922
1370325_at	113961	Gorasp2	golgi reassembly stacking protein 2	7308.73	2679.838	3738.058
1398349_at	24184	Ak2	adenylate kinase 2	7304.75	12664.97	2829.182
1375934_at	315911	RGD15662	similar to RIKEN cDNA D330045A20 (	7301.43	1287.226	2430.065
1384394_at	79435	Ube2d2	ubiquitin-conjugating enzyme E2D 2	7287.809	5786.99	4552.233
1372150_at	307905	Usp10	ubiquitin specific protease 10	7282.297	4778.627	10299.36
1367939_at	25056	Rbp1	retinol binding protein 1, cellular	7278.383	1749.14	5611.152
1372726_at	64627	Hist1h4b	germinal histone H4 gene	7274.813	121.4982	1239.326
1372070_at	290644	Ifi30	interferon gamma inducible protein 30	7260.063	1815.774	2386.072
1375697_at	304543	RGD13077	similar to Hypothetical protein KIAA015	7259.424	2152.686	334.0323
1375901_at	606294	LOC60629	NA	7243.142	5363.099	13336.31
1372724_at	266668	Grina	glutamate receptor, ionotropic, N-meth	7237.079	12177.47	2054.882
1367832_at	25514	Lypla1	lysophospholipase 1	7236.081	10650.39	10038.39
1373878_at	81762	Rock1	Rho-associated coiled-coil forming kina	7234.578	7950.699	14408.34
1373432_at	25603	Marcks	myristoylated alanine rich protein kinas	7230.76	6236.988	10345.34
1388129_at	81785	Ssrp1	structure specific recognition protein 1	7229.421	13994.23	3980.403
1382351_at	297902	Gem_predi	GTP binding protein (gene overexpres	7218.896	1104.438	19150.87
1372735_at	292762	Eif3s12_pr	eukaryotic translation initiation factor 3	7214.529	7592.752	4323.984
1375185_at	308939	Ipo7_predi	importin 7 (predicted)	7211.371	6113.443	4273.09
1371542_at	316531	Tuba4	tubulin, alpha 4	7208.684	5242.707	12539.34

1373846_at	366434	RGD13117	similar to RIKEN cDNA 5730434I03 ge	7205.01	15853.05	9111.993
1367770_at	58970	Degs1	degenerative spermatocyte homolog 1	7201.572	5607.31	6387.12
1378352_at	310669	Golph3l	golgi phosphoprotein 3-like	7201.465	2383.002	4156.759
1372526_at	303185	Flcn	folliculin	7196.893	4176.97	7111.165
1382434_at	314312	Entpd5	ectonucleoside triphosphate diphosph	7195.517	1149.233	769.7039
1371547_at	297591	NA	NA	7190.81	5406.169	11508.4
1370554_at	114094	Uchl3	ubiquitin carboxyl-terminal esterase L3	7187.284	6931.437	4446.59
1372090_at	60661	Max	Max protein	7184.833	3100.321	9024.18
1368809_at	64185	Cap1	CAP, adenylate cyclase-associated pr	7183.889	11434.45	2350.897
1371930_at	290868	Qars	glutaminyl-tRNA synthetase	7179.857	4041.087	4411.184
1372252_at	287427	Trappc1	trafficking protein particle complex 1	7173.022	13384.69	6295.087
1373844_at	361613	Ppme1	protein phosphatase methylesterase 1	7170.82	7279.6	4546.339
1370314_at	81826	Slc20a1	solute carrier family 20 (phosphate tra	7169.456	7779.27	14140.82
1372791_at	116477	Prkwnk1	protein kinase, lysine deficient 1	7158.128	9579.979	12576.58
1372919_at	290843	Agpat6	1-acylglycerol-3-phosphate O-acyltran	7157.54	9152.809	3724.501
1375552_at	498351	LOC49835	similar to signal recognition particle,72	7157.257	5556.845	2940.158
1371734_at	298982	Maea	macrophage erythroblast attacher	7151.728	5883.045	2970.839
1371580_at	293939	Spfh1_pre	SPFH domain family, member 1 (predi	7137.716	9315.707	4244.051
1373020_at	287065	RGD15644	similar to mitochondria-associated gra	7132.364	7621.899	3142.659
1372056_at	316035	Cmtm6	CKLF-like MARVEL transmembrane d	7127.219	2846.118	10891.93
1372654_at	361674	Eps8l2_pre	EPS8-like 2 (predicted)	7122.89	448.477	2507.673
1367708_a_at	50671	Fasn	fatty acid synthase	7122.051	9659.74	7466.864
1373917_at	307503	Etf1	eukaryotic translation termination facto	7118.659	8319.276	11529.43
1372817_at	53982	Zfp260	zinc finger protein 260	7116.489	8815.631	7511.21
1374746_at	500877	LOC50087	Ab1-152	7110.445	2473.353	7442.211
1369992_at	83806	Psmd1	proteasome (prosome, macropain) 26S	7110.086	3336.335	7033.487
1386982_at	94273	Mgat2	mannoside acetylglucosaminyltransfer	7102.269	2481.268	6087.487
1388379_at	25622	Ptpn11	protein tyrosine phosphatase, non-rec	7090.982	12606.46	9065.945
1398795_at	116483	Dars	aspartyl-tRNA synthetase	7090.224	9774.835	10751.76
1369941_at	64322	Dap	death-associated protein	7089.513	5700.896	12090.03
1398793_at	85434	Cdc5l	cell division cycle 5-like (S. pombe)	7083.832	8960.25	7156.762
1373056_at	305502	Tmed4_pre	transmembrane emp24 protein transpo	7081.392	6857.923	9000.786
1388330_at	309004	Vkorc1	vitamin K epoxide reductase complex,	7075.584	14946.24	7109.738
1371393_at	313717	Clstn1	calsyntenin 1	7071.039	5582.558	2878.139
1373900_at	300242	Krt2-7	keratin complex 2, basic, gene 7	7070.54	7362.301	6302.348
1370304_at	54311	Timm17a	translocator of inner mitochondrial me	7055.902	13942.37	3416.014
1373335_at	302808	Zdhhc9	zinc finger, DHHC domain containing 9	7044.505	4991.919	1577.677
1399052_at	361677	Tollip_pred	toll interacting protein (predicted)	7027.211	7215.726	5082.878
1390148_a_at	305972	Zfp395_pre	zinc finger protein 395 (predicted)	7026.816	3443.621	19558.77
1368359_a_at	29461	Vgf	VGF nerve growth factor inducible	7026.685	5633.247	2966.519
1371515_at	315466	NA	NA	7023.86	7773.02	3617.981
1399058_at	292244	Mrpl18_pre	mitochondrial ribosomal protein L18 (p	7021.465	12686.59	2851.614
1370191_at	58961	Azin1	antizyme inhibitor 1	7012.539	12701.47	23180.74
1377970_at	294762	LOC29476	NA	7008.412	4459.459	7362.405
1367680_at	50681	Acox1	acyl-Coenzyme A oxidase 1, palmitoyl	7007.32	5998.438	6328.321
1388373_at	289036	Adipor1	adiponectin receptor 1	6998.836	8778.226	6971.881
1372421_at	290923	Aga	aspartylglucosaminidase	6988.476	5598.656	19547.71
1371888_at	295224	mrpl24	mitochondrial ribosomal protein L24	6988.401	4693.634	1457.892
1370281_at	140868	Fabp5	fatty acid binding protein 5, epidermal	6988.195	23514.23	4201.06
1374858_at	294741	Dhx29_pre	DEAH (Asp-Glu-Ala-His) box polypepti	6984.005	6106.492	7807.089
1388560_at	310769	Wdr77	WD repeat domain 77	6972.441	10077.96	4936.713

1371599_at	294154	LOC29415	similar to chromosome 6 open reading	6971.391	5466.138	4796.753
1370562_at	171519	Calcb	calcitonin-related polypeptide, beta	6968.354	18.60284	21978.42
1373381_at	309758	Herc4	hect domain and RLD 4	6958.545	2345.016	9083.84
1373087_at	311059	7-Mar	membrane-associated ring finger (C3H	6958.096	6649.622	26089.21
1388616_at	494346	MGC7295	NA	6954.003	9674.776	6541.022
1373481_at	304763	R3hdm1	R3H domain containing 1	6952.052	8301.813	4626.352
1383454_a_at	289342	Rps6kc1	ribosomal protein S6 kinase, polypepti	6931.697	383.3034	1046.787
1372028_at	306768	RGD13057	similar to Protein CGI-117 (Protein HS	6929.321	8757.391	3689.347
1371404_at	300253	Eif4b	eukaryotic translation initiation factor 4	6927.291	7405.381	6818.895
1387120_at	29677	Psmc3	proteasome (prosome, macropain) 26S	6924.659	10178.07	2270.686
1386867_at	171087	Brp44l	brain protein 44-like	6914.408	12995.86	13020.96
1375785_at	362520	Fcmd_prec	Fukuyama type congenital muscular dy	6898.155	8086.417	2210.162
1373812_at	83571	Cdkn1b	cyclin-dependent kinase inhibitor 1B	6892.042	20632.62	19124.93
1374878_at	360972	Fmip	Fms interacting protein	6880.084	8306.451	3261.584
1388614_at	293671	Sf3b2_prec	splicing factor 3b, subunit 2 (predicted)	6879.612	7674.326	6320.855
1379561_at	309532	Atad1	ATPase family, AAA domain containing	6863.391	15287.91	8683.203
1387857_at	60466	Stx7	syntaxin 7	6859.626	14894.18	14950.79
1371637_at	313647	Hp1bp3	heterochromatin protein 1, binding pro	6858.556	6673.354	4990.548
1373535_at	360891	Enah	enabled homolog (Drosophila)	6852.128	3012.899	6969.987
1388308_at	687090	LOC68709	NA	6851.167	6818.042	8341.892
1373845_at	246145	Pbp2	phosphatidylethanlomine binding prote	6850.581	9991.723	4247.481
1398864_at	64631	Ube2g1	ubiquitin-conjugating enzyme E2G 1 (U	6850.555	14481.04	8817.242
1367471_at	299610	Polr2e_pre	polymerase (RNA) II (DNA directed) po	6842.162	5192.005	4402.801
1389033_at	361805	RGD13069	similar to RIKEN cDNA 2900010M23 (	6835.125	13735.77	5016.58
1373591_at	293344	Arfip2	ADP-ribosylation factor interacting prot	6834.9	3392.175	1493.484
1386338_at	303201	Usp22_pre	ubiquitin specific protease 22 (predicte	6832.647	10022.41	6252.627
1375245_at	117281	Ppp2r1a	protein phosphatase 2 (formerly 2A), re	6825.08	9222.105	2525.803
1388575_at	499873	RGD15642	similar to Opa-interacting protein 5 (pre	6824.786	7844.127	10070.34
1389964_at	293453	Ndufab1_p	NADH dehydrogenase (ubiquinone) 1,	6821.897	15299.62	9704.435
1367497_at	314553	Ptdss1	phosphatidylserine synthase 1	6821.058	11629.64	2061.259
1374883_at	306490	Mtmr7_pre	myotubularin related protein 7 (predicte	6816.942	5968.068	3877.156
1389525_at	363222	Rnf149	ring finger protein 149	6811.135	6121.337	3697.217
1386890_at	81778	S100a10	S100 calcium binding protein A10 (calp	6804.191	27885.64	3468.713
1372982_at	29748	Ppp3r1	protein phosphatase 3, regulatory subu	6803.919	6033.836	3616.285
1387921_at	192359	Npuk68	nuclear protein UKp68	6803.805	6296.095	9246.135
1388488_at	297455	Lsm3_prec	LSM3 homolog, U6 small nuclear RNA	6803.64	15126.82	5198.288
1386994_at	29619	Btg2	B-cell translocation gene 2, anti-prolife	6800.803	1651.648	25726.35
1374401_at	291464	Snx2_pred	sorting nexin 2 (predicted)	6798.798	12724.9	19695.8
1383107_at	291794	Snrpd1_pre	small nuclear ribonucleoprotein D1 (pr	6791.683	3759.585	4570.31
1389526_at	362923	RGD13059	similar to RIKEN cDNA C920006C10 (	6791.368	6734.384	5259.755
1372100_at	298552	Tmem50a_	transmembrane protein 50A (predicted	6789.045	5313.705	5096.388
1375182_at	29484	Slc3a1	solute carrier family 3, member 1	6787.107	16013.48	6634.587
1381623_at	362612	RGD15613	similar to Sfrs4 protein (predicted)	6783.826	7287.965	9358.537
1398955_at	363283	Cops8	COP9 (constitutive photomorphogenic	6779.366	6768.061	6874.098
1399137_at	295500	Metap1_pr	methionyl aminopeptidase 1 (predicted	6779.232	6934.386	6087.173
1399135_at	300189	MGC9495	similar to RIKEN cDNA 2310042P20	6768.102	11090.24	10387.08
1375845_at	292486	RGD15629	similar to Aig1 protein (predicted)	6765.311	4282.075	2801.128
1387859_at	84594	Nfs1	nitrogen fixation gene 1 (S. cerevisiae)	6762.638	7686.052	4720.662
1387872_at	29578	Hnrpa1	heterogeneous nuclear ribonucleoprot	6762.01	21749.29	13679.19
1398874_at	170821	Atxn10	ataxin 10	6761.376	14103.12	9598.749
1374169_at	360480	RGD13106	similar to chromosome 16 open readin	6758.705	6639.855	3933.808

1398857_at	64463	Surf1	surfeit 1	6758.375	7530.88	6516.939
1374163_at	305420	Anapc4	anaphase promoting complex subunit	6758.276	8554.745	5994.078
1371988_at	294410	Man1a_pre	mannosidase 1, alpha (predicted)	6756.795	284.7208	7580.535
1373145_at	306991	RGD15605	similar to Vps41 protein (predicted)	6753.232	14917.53	7259.442
1380062_at	362359	Mpp6_prec	membrane protein, palmitoylated 6 (M	6747.591	6890.749	1889.746
1388817_at	310665	LOC31066	hypothetical protein LOC310665	6741.629	2061.134	789.8505
1393915_at	362434	Oact5	O-acyltransferase (membrane bound)	6740.288	10193.81	1688.221
1374067_at	365493	RGD13081	similar to 2700078E11Rik protein	6739.364	5627.249	8362.719
1385767_at	304000	LOC30400	cell adhesion molecule JCAM	6737.167	2005.623	3763.992
1372155_at	116698	Trim28	tripartite motif protein 28	6729.772	10844.5	5297.437
1367845_at	24588	Nef3	neurofilament 3, medium	6723.187	675.4652	1084.176
1387864_at	116478	Kidins220	kinase D-interacting substance 220	6717.886	8941.092	7794.745
1373244_at	303567	RGD13047	similar to RIKEN cDNA 2010008E23 g	6715.784	6886.088	3420.989
1382019_at	295051	Alg5	asparagine-linked glycosylation 5 hom	6714.445	7532.904	5043.574
1371911_at	287543	Tnfaip1	tumor necrosis factor, alpha-induced p	6704	3952.875	3135.835
1376848_at	310178	Myo10_pre	myosin X (predicted)	6698.481	13225.01	3437.273
1387798_a_at	54243	Crry	complement receptor related protein	6694.878	5187.663	10345.31
1373764_at	312491	Zfml_predi	zinc finger, matrin-like (predicted)	6694.583	5259.316	8395.254
1398811_at	29439	Jtb	jumping translocation breakpoint	6692.302	3802.865	4037.727
1379542_at	296377	RGD15619	similar to Small nuclear ribonucleoprot	6688.906	10697.36	7938.677
1390784_at	307075	Wdr37_pre	WD repeat domain 37 (predicted)	6683.639	1324.817	1788.43
1374731_at	50998	Coil	coilin	6672.501	7490.054	11631.24
1389126_at	361005	Chchd1_pr	coiled-coil-helix-coiled-coil-helix domai	6662.593	8377.252	7641.957
1391416_at	289521	RGD15623	similar to ankyrin repeat domain protei	6661.707	6853.393	5273.07
1367493_at	498890	RGD15602	similar to DNA segment, Chr 18, Wayr	6658.861	9600.081	1871.456
1372712_at	287208	Ttc1	tetratricopeptide repeat domain 1	6654.113	9274.915	2515.265
1367519_at	296461	Osbpl2	oxysterol binding protein-like 2	6646.28	6098.345	7342.267
1383165_at	362019	RGD13102	similar to KIAA1324 protein (predicted)	6640.776	5918.091	18377.39
1368216_at	117049	Rab28	RAB28, member RAS oncogene family	6636.171	9298.774	14521.78
1391625_at	360206	Asb15	ankyrin repeat and SOCS box-containi	6633.536	2622.176	2798.381
1383375_at	362490	Tmem55a	transmembrane protein 55A	6629.834	3191.368	8562.404
1388322_at	287986	Eif4g1	eukaryotic translation initiation factor 4	6625.677	5075.783	1144.346
1370158_at	79433	Myh10	myosin, heavy polypeptide 10, non-mu	6621.062	11726.11	2639.217
1368273_at	58840	Mapk6	mitogen-activated protein kinase 6	6614.461	4358.804	11231.91
1370907_at	25197	St6gal1	beta galactoside alpha 2,6 sialyltransfe	6608.582	209.0775	6215.545
1372143_at	287927	Ube2v2	ubiquitin-conjugating enzyme E2 varia	6600.799	8826.506	7899.638
1371929_at	360631	Mlx	MAX-like protein X	6596.901	9142.809	2048.21
1382303_at	306844	Phactr1	phosphatase and actin regulator 1	6584.648	3968.304	1873.182
1367453_at	114562	Cdc37	cell division cycle 37 homolog (S. cere	6582.63	10516.46	2915.922
1383477_at	360853	Uchl5	ubiquitin carboxyl-terminal hydrolase L	6573.169	6067.305	4515.326
1388799_at	362303	Klhl7	kelch-like 7 (Drosophila)	6572.246	14803.89	36325.58
1372485_at	29700	Pcbd1	pterin 4 alpha carbinolamine dehydrata	6554.171	27961.11	10636.71
1368379_at	117106	Scarb2	scavenger receptor class B, member 2	6552.09	2440.635	967.6455
1388586_at	85238	Synj1	synaptojanin 1	6547.474	6562.165	11204.66
1388412_at	307833	RGD13077	similar to RIKEN cDNA 2400003C14	6543.449	4593.524	11873.47
1376347_at	304809	RGD15656	similar to PLU1 (predicted)	6543.422	2542.289	1879.71
1373378_at	290986	Agtpbp1_p	ATP/GTP binding protein 1 (predicted)	6543.379	6473.132	3732.054
1374969_at	252886	Foxd4	forkhead box D4	6542.948	1409.698	6729.625
1384205_at	361014	LOC36101	NA	6540.405	4449.318	8387.161
1375003_at	291085	Serpinb6a	serine (or cysteine) peptidase inhibitor	6537.476	3291.09	3389.084
1368336_at	29189	Fdx1	ferredoxin 1	6526.803	2877.592	8437.404

1387892_at	29214	Tubb5	tubulin, beta 5	6525.697	31295.9	2694.54
1370886_a_at	171041	Kns2	kinesin 2	6525.332	8244.746	2992.59
1398928_at	294288	Cuta	divalent cation tolerant protein CUTA	6517.572	7770.751	3986.932
1372710_at	29631	Bet1	blocked early in transport 1 homolog (S	6515.191	5654.822	10096.6
1375575_at	24245	Camk2b	calcium/calmodulin-dependent protein	6504.654	14238.28	5962.607
1372501_at	292019	Sf3b3_pred	splicing factor 3b, subunit 3 (predicted)	6503.792	5436.845	3336.768
1370376_a_at	83807	Csda	cold shock domain protein A	6500.145	5805.444	3359.823
1389510_at	289707	Lyar	Ly1 antibody reactive clone	6489.401	4441.938	3595.332
1399049_at	299127	RGD13089	similar to RIKEN cDNA 1200003C05 (p	6488.544	3429.765	5117.728
1371820_at	308796	Mesdc2	mesoderm development candiate 2	6488.192	9500.555	9776.973
1387074_at	84583	Rgs2	regulator of G-protein signaling 2	6485.47	628.3023	28256.64
1371992_at	290631	MGC94542	similar to RIKEN cDNA 5430437P03	6484.098	8783.494	4733.005
1383410_at	116650	Srp54	signal recognition particle 54	6483.434	2193.763	4876.193
1383151_at	246150	Akap9	A kinase (PRKA) anchor protein (yotia	6481.332	6558.326	10548.52
1372099_at	297530	LOC29753	NP61201	6459.376	8070.225	8503.725
1373561_at	316533	D1bwg136	chondroitin polymerizing factor	6450.234	1022.493	2057.01
1388904_at	360863	Dd25	hypothetical protein Dd25	6448.616	1687.337	4982.924
1373080_at	314417	Papola_pre	poly (A) polymerase alpha (predicted)	6447.42	11624.21	7750.705
1383336_at	368070	LOC36807	NA	6446.648	2129.504	14168.46
1398916_at	298687	Aurkaip1	aurora kinase A interacting protein 1	6438.535	3375.615	2765.59
1374616_at	290771	Pdgfrl	platelet-derived growth factor receptor-	6435.605	4319.244	6913.862
1377854_at	311350	RGD13056	similar to hypothetical protein FLJ2337	6427.517	4653.717	2733.739
1371577_at	301458	Ndufs1	NADH dehydrogenase (ubiquinone) Fe	6417.447	6568.069	6503.903
1394567_at	170671	Lhx3	LIM homeobox protein 3	6406.931	3327.052	1042.831
1398971_at	303280	RGD13079	similar to CG14967-PA	6405.228	9776.722	2077.761
1388626_at	296162	RGD13099	similar to chromosome 20 open readin	6404.514	6692.143	2059.399
1383221_at	360478	RGD15635	RGD1563547 (predicted)	6403.818	5578.228	4958.263
1371502_at	361545	RGD13061	similar to RIKEN cDNA 3100004P22	6400.435	5889.098	5684.621
1387777_at	170922	Ilk	integrin linked kinase	6388.706	5586.358	1883.643
1373111_at	306260	Capn7	calpain 7	6382.409	7675.46	12179.26
1376304_at	301232	RGD15616	similar to AI661453 protein (predicted)	6374.842	1341.242	5540.713
1372286_at	302313	Tspan6	tetraspanin 6	6372.729	12356.74	9097.785
1377669_at	50645	Rab27a	RAB27A, member RAS oncogene fam	6371.995	1885.552	5170.461
1367675_at	81823	Cib1	calcium and integrin binding 1 (calmyri	6371.104	2705.829	1651.453
1372907_at	436582	Atp6v0e2	ATPase, H+ transporting, V0 subunit E	6366.258	11206.29	3391.632
1376636_at	29591	Tgfbr1	transforming growth factor, beta recep	6361.554	5938.003	19213.38
1369559_a_at	29364	Cd47	CD47 antigen (Rh-related antigen, inte	6359.355	9587.908	1837.773
1368321_at	24330	Egr1	early growth response 1	6353.735	1360.927	100405.4
1371960_at	313053	Ythdf2_pre	YTH domain family 2 (predicted)	6345.975	23457.65	3110.319
1388907_at	312694	RGD13060	similar to RIKEN cDNA 1200016B17	6341.962	5444.835	5145.438
1369926_at	64317	Gpx3	glutathione peroxidase 3	6340.357	162.2968	17595.89
1372052_at	361662	Bub3	budding uninhibited by benzimidazoles	6339.041	18316.01	4825.11
1367492_at	313035	Dnajc8	DnaJ (Hsp40) homolog, subfamily C, r	6333.083	8537.396	6961.563
1371474_at	294313	Mtch1	mitochondrial carrier homolog 1 (C. ele	6321.285	10167.43	5169.076
1383181_at	364240	Dnajc9_pre	DnaJ (Hsp40) homolog, subfamily C, r	6317.696	16319.02	2397.051
1398876_at	85493	Abcf1	ATP-binding cassette, sub-family F (G	6317.047	6448.413	3257.841
1377041_at	315119	Mfng	manic fringe homolog (Drosophila)	6314.072	1011.303	571.0635
1383115_at	309338	Cstf2t_pred	cleavage stimulation factor, 3' pre-RNA	6314.07	18857.5	12255.87
1372223_at	303010	Cpeb4_pre	cytoplasmic polyadenylation element b	6312.97	7758.869	18932.66
1379449_at	312927	Rb1cc1_pr	RB1-inducible coiled-coil 1 (predicted)	6300.484	6386.102	8228.896
1369681_at	64444	Isl1	ISL1 transcription factor, LIM/homeodo	6299.872	8911.262	4657.308

1398350_at	64160	Basp1	brain abundant, membrane attached s	6298.536	40114.95	24845.87
1388947_at	308306	Eif5b	eukaryotic translation initiation factor 5	6296.436	2815.378	4197.991
1388152_at	25595	Mtap2	microtubule-associated protein 2	6296.194	3816.568	14982.31
1399011_at	304343	Cops6_pre	COP9 (constitutive photomorphogenic	6293.03	17976.16	2793.448
1389289_at	289752	Ewsr1	Ewing sarcoma breakpoint region 1	6290.662	6665.913	11295.87
1388693_at	300142	RGD13108	similar to RIKEN cDNA 5730502D15 g	6281.194	2676.419	987.0341
1367510_at	307180	RGD13046	similar to hypothetical protein FLJ2262	6278.325	13959	6134.835
1370908_at	84577	Hdac2	histone deacetylase 2	6277.273	13309.78	15521
1371489_at	29274	Rnf4	ring finger protein 4	6273.569	10129.79	16074.24
1398448_at	298034	Tscot_pred	thymic stromal cotransporter (predicted	6272.423	3468.861	7531.576
1372645_at	287979	Psarl	presenilin associated, rhomboid-like	6267.945	7770.614	3601.501
1390129_at	308265	RGD15640	similar to LATS homolog 1 (predicted)	6255.994	4645.109	9819.059
1374487_at	300797	RGD13074	similar to RIKEN cDNA 5730536A07	6255.379	4231.663	4904.793
1370844_at	64200	Hnrpf	heterogeneous nuclear ribonucleoprote	6252.852	11226.01	8372.391
1391805_at	313111	RGD13103	similar to KIAA1900 protein (predicted)	6252.455	1981.118	1544.498
1373289_at	315287	Asb8_pred	ankyrin repeat and SOCS box-containi	6251.27	6772.263	3292.677
1383680_at	338474	Hspa5bp1	heat shock 70kDa protein 5 binding pr	6242.082	14503.71	8137.095
1379314_at	24675	Ppp3cb	protein phosphatase 3, catalytic subun	6235.336	5679.309	7268.197
1388684_at	311183	Fnbp4	formin binding protein 4	6233.152	3468.042	8348.776
1399036_at	308896	Usp47_pre	ubiquitin specific protease 47 (predicte	6231.062	5409.267	3936.826
1389575_at	293160	RGD13117	similar to sid2057p	6230.772	10147.7	5116.258
1378027_at	288124	Pvrl3_pred	poliovirus receptor-related 3 (predicted	6227.433	7827.716	4576.343
1389521_at	289089	Ivns1abp_p	influenza virus NS1A binding protein (p	6225.696	7753.851	6234.159
1389316_at	363445	Usp9x_pre	ubiquitin specific peptidase 9, X chrom	6225.159	7403.486	14327.92
1389482_at	312437	RGD13064	similar to EST AA792894	6224.909	5287.164	7471.536
1372877_at	288583	Plod3	procollagen-lysine, 2-oxoglutarate 5-di	6210.849	12670.47	4320.355
1387101_at	113976	Acsl4	acyl-CoA synthetase long-chain family	6206.349	10338.02	8409.131
1372017_at	288753	Diablo	diablo homolog (Drosophila)	6205.696	4386.699	4608.125
1380596_at	24856	Ttr	transthyretin	6204.78	10500.78	5071.92
1387928_at	303569	Rap2ip	Rap2 interacting protein	6197.099	5086.971	4489.449
1379395_a_at	313491	Hint2_pred	histidine triad nucleotide binding protei	6196.58	9558.513	4127.395
1371868_at	295334	Bcas2_pre	breast carcinoma amplified sequence 2	6191.033	6803.32	14465.9
1367755_at	81718	Cdo1	cysteine dioxygenase 1, cytosolic	6187.91	460.1003	3332.259
1383202_at	498934	RGD15619	similar to U6 snRNA-associated Sm-lik	6187.187	6863.976	5792.281
1378094_at	303953	RGD13080	similar to hypothetical protein FLJ1134	6186.083	2224.103	4438.755
1397380_at	306203	Pxk	PX domain containing serine/threonine	6185.734	571.7128	2740.908
1368025_at	140942	Ddit4	DNA-damage-inducible transcript 4	6184.482	1752.076	619.7053
1389329_at	116641	Lgals8	lectin, galactoside-binding, soluble 8	6182.984	7036.065	4403.721
1372270_at	300668	H2afx	dolichyl-phosphate (UDP-N-acetylgluc	6182.034	2658.206	2095.312
1373413_at	360952	RGD13104	similar to RIKEN cDNA 2810021O14 (	6181.719	6408.16	3737.76
1396556_at	498438	LOC49843	NA	6178.086	12.90879	1046.692
1371638_at	300948	Rnf7_predi	ring finger protein 7 (predicted)	6176.271	5926.313	5212.96
1387109_at	29441	Por	P450 (cytochrome) oxidoreductase	6170.134	3946.606	1872.124
1379353_at	300328	Aasdhppt_	aminoadipate-semialdehyde dehydrog	6165.701	3697.954	6377.556
1372209_at	312916	Cops5	COP9 (constitutive photomorphogenic	6164.917	15104.91	6081.934
1392806_at	290831	Whsc1l1_p	Wolf-Hirschhorn syndrome candidate 1	6154.355	5185.253	6815.875
1388150_at	85252	Xpo1	exportin 1, CRM1 homolog (yeast)	6152.796	11716.87	6223.032
1368903_at	84476	Strbp	spermatid perinuclear RNA binding pro	6146.905	3487.809	3091.813
1373332_at	64462	Csnk1d	casein kinase 1, delta	6144.28	8577.085	9382.276
1372217_at	303332	LOC30333	hypothetical protein LOC303332	6144.122	8537.846	4335.564
1370879_at	299201	Dlst	dihydrolipoamide S-succinyltransferase	6134.805	3078.232	1514.769

1374133_at	362586	Trit1_predi	tRNA isopentenyltransferase 1 (predic	6132.509	3156.272	3216.868
1374987_at	360511	Pank3_pre	pantothenate kinase 3 (predicted)	6132.306	13404.96	9826.913
1387485_a_at	117104	Ppp2r2a	protein phosphatase 2 (formerly 2A), r	6122.334	4514.64	4065.143
1371471_at	292624	Gltscr2	glioma tumor suppressor candidate reg	6121.748	7050.383	13494.46
1369954_at	24479	Idh1	isocitrate dehydrogenase 1 (NADP+), s	6119.912	15387.67	6995.787
1371704_at	362993	Rnd1	Rho family GTPase 1	6114.266	8871.673	3474.025
1371685_at	312372	Kbtbd2_pre	kelch repeat and BTB (POZ) domain c	6104.936	8267.734	14892.04
1373819_at	500855	LOC50085	hypothetical protein LOC500855	6093.85	12928.37	9724.574
1388814_at	292022	Ddx19	DEAD (Asp-Glu-Ala-Asp) box polypept	6087.142	3613.681	8085.827
1386970_at	117019	Eif2b4	eukaryotic translation initiation factor 2	6084.166	6161.635	4070.712
1371443_at	362671	RGD13045	similar to RIKEN cDNA A430005L14	6083.573	3296.062	3317.677
1372888_at	315608	Ube4a	ubiquitination factor E4A, UFD2 homol	6082.504	3045.424	1605.426
1372151_at	307474	Tcerg1_pre	transcription elongation regulator 1 (CA	6079.059	6988.149	9767.062
1383090_at	289143	Mrps14_pr	mitochondrial ribosomal protein S14 (p	6071.864	4892.65	5230.482
1372953_at	553106	Ncald	neurocalcin delta	6070.767	4855.503	10187.62
1379456_at	313241	Mcart1	mitochondrial carrier triple repeat 1	6070.126	13198.01	6325.08
1367678_at	157074	Sdha	succinate dehydrogenase complex, su	6064.082	8805.672	3640.569
1374004_at	298771	RGD13101	similar to RIKEN cDNA D030028O16	6052.913	14983.84	6709.75
1382064_at	292051	RGD13061	similar to 4933407C03Rik protein (pred	6052.041	3258.899	2965.587
1385266_at	365949	LOC36594	NA	6051.94	3774.296	4184.691
1386973_a_at	116457	Mapk8ip	mitogen activated protein kinase 8 inte	6050.621	5153.73	5361.951
1372759_at	362110	Cdk9	cyclin-dependent kinase 9 (CDC2-relat	6048.35	8465.797	6170.726
1388787_at	303478	Ube2z	ubiquitin-conjugating enzyme E2Z (put	6045.859	6366.525	2196.868
1380448_at	362766	Alkbh_prec	alkB, alkylation repair homolog (E. coli	6045.501	1825.772	3549.242
1374196_at	114515	Lancl1	lanC (bacterial lantibiotic synthetase co	6044.245	3564.518	1766.533
1389520_at	360950	Wdr1	NA	6030.547	5984.624	4519.999
1390592_at	688018	LOC68801	NA	6024.781	2616.832	785.5514
1372351_at	362844	Brd4	bromodomain containing 4	6020.03	4029.443	5413.958
1389063_at	293476	Xpo6	exportin 6	6016.881	10218.27	3591.149
1393244_at	140639	Nploc4	nuclear protein localization 4 homolog	6015.792	6762.28	1932.321
1392972_at	310192	Trio	triple functional domain (PTPRF intera	6010.072	7545.174	1826.914
1379330_s_at	502114	NA	NA	6007.577	7870.859	12698.6
1373905_at	319110	Hnrpr	heterogeneous nuclear ribonucleoprote	6004.426	7326.702	23022.15
1372520_at	60430	Mcl1	myeloid cell leukemia sequence 1	6002.037	5981.063	42275.59
1367523_at	292074	RGD13049	similar to hypothetical protein, 2-6 (pre	5998.896	6278.603	4852.461
1370226_at	25308	Cstb	cystatin B	5995.593	9179.642	8275.996
1388551_at	116782	Pcdhgc3	protocadherin gamma subfamily C, 3	5994.858	8467.086	7152.547
1371416_at	293655	Ndufv1	NADH dehydrogenase (ubiquinone) fla	5994.253	15470.68	2552.445
1383602_at	362609	Pum1_prec	pumilio 1 (Drosophila) (predicted)	5992.281	6768.209	23485.71
1387912_at	245957	Ddx46	DEAD (Asp-Glu-Ala-Asp) box polypept	5990.486	7564.921	4103.273
1372488_at	305177	RGD13090	similar to RIKEN cDNA 2310057D15	5987.971	4086.111	3182.787
1389208_at	363495	NA	NA	5982.347	6326.087	11937.2
1386925_at	54227	Arpc1b	actin related protein 2/3 complex, subu	5982.146	10409.94	1120.565
1373011_at	619558	LOC61955	hypothetical protein LOC619558	5980.319	6388.061	6856.875
1387883_a_at	81814	Tmsb4x	thymosin, beta 4	5979.759	24612.57	100085.1
1367721_at	24771	Sdc4	syndecan 4	5974.236	2535.948	4069.1
1389918_at	290704	LOC29070	NA	5973.873	2689.389	793.2213
1388776_at	301013	MGC94600	scotin	5973.831	3883.229	3821.669
1391812_at	315979	RGD13091	similar to RIKEN cDNA E330026B02 (	5959.918	459.4571	2077.964
1398877_at	192277	Stip1	stress-induced phosphoprotein 1	5956.753	19397.73	3875.478
1374792_at	498727	RGD15659	similar to nucleolar protein 7, 27kDa (p	5955.26	11821.73	11436.11

1384323_at	289990	Psmc6	proteasome (prosome, macropain) 26S	5949.198	4024.214	10185.96
1388642_at	300514	Ei24	etoposide induced 2.4 mRNA	5948.7	8095.111	3221.402
1371490_at	286899	Hsbp1	heat shock factor binding protein 1	5948.079	7515.479	4135.587
1371456_at	287982	Abcf3	ATP-binding cassette, sub-family F (G	5946.488	3090.906	2402.444
1371723_at	298514	Rragc_pred	Ras-related GTP binding C (predicted)	5941.884	6500.301	6826.614
1368427_at	25228	Akap11	A kinase (PRKA) anchor protein 11	5929.596	2786.015	9249.727
1399154_at	361700	Fbxl11_pre	F-box and leucine-rich repeat protein 1	5913.499	3309.974	9299.212
1367715_at	25625	Tnfrsf1a	tumor necrosis factor receptor superfa	5910.99	1693.9	2197.281
1373933_at	310533	Rapgef2_p	Rap guanine nucleotide exchange fact	5907.77	7767.042	11602.95
1372814_at	360868	RGD1310€	similar to RIKEN cDNA 2010005O13	5906.841	2536.45	3464.652
1373369_at	361103	Rai17_pred	retinoic acid induced 17 (predicted)	5905.329	6819.523	11486.26
1373299_at	114117	Gng3	guanine nucleotide binding protein (G	5902.437	10054.29	7766.438
1373500_at	313867	Lrpprc	leucine-rich PPR-motif containing	5897.593	12289.76	6364.221
1392051_at	362212	RGD13079	similar to hypothetical protein FLJ1468	5897.028	3943.739	1361.929
1372237_at	500441	RGD15619	similar to testes development-related N	5893.956	8360.437	7557.48
1372076_at	295357	Hbxip_pred	hepatitis B virus x interacting protein (p	5892.813	9170.911	3841.005
1376793_at	306687	RGD13083	similar to RIKEN cDNA 5730414C17 (p	5892.744	9707.69	11591.48
1370360_at	192229	RGD62135	similar to RIKEN cDNA 1500031L02	5891.494	7207.657	7251.414
1386872_at	25151	Igf2r	insulin-like growth factor 2 receptor	5889.59	4283.147	1326.422
1377872_at	297082	RGD13069	similar to chromosome 7 open reading	5888.712	7619.772	5880.574
1399133_at	366854	Fbxo7	F-box only protein 7	5887.122	7142.296	3157.814
1368057_at	25270	Abcd3	ATP-binding cassette, sub-family D (A	5887.073	2263.504	3383.78
1371985_a_at	361796	Bat5	HLA-B associated transcript 5	5878.185	7414.571	3034.317
1377812_a_at	300666	Tmem24	transmembrane protein 24	5875.489	2466.27	4402.887
1375027_at	363098	Ccpg1_pre	cell cycle progression 1 (predicted)	5870.327	1866.214	4882.98
1386394_at	314140	LOC31414	ribose-phosphate pyrophosphokinase	5869.251	3435.466	2931.105
1371873_at	361999	Anp32e	acidic (leucine-rich) nuclear phosphopr	5867.618	8434.305	7663.882
1389509_at	307527	Sap130_pr	Sin3A associated protein 130 (predicte	5866.26	4750.872	3388.578
1382001_at	502710	LOC50271	NA	5861.518	3429.977	20306.26
1373978_at	298075	Ncbp1	nuclear cap binding protein subunit 1,	5861.315	9788.675	1699.29
1397892_at	317163	Eif1a	eukaryotic translation initiation factor 1	5858.18	3297.391	8230.75
1368831_at	170577	Mark3	MAP/microtubule affinity-regulating kin	5854.94	6979.945	9107.667
1390409_at	361774	RGD13061	LOC361774 (predicted)	5853.814	2295.176	3779.533
1368552_at	79563	Grpel1	GrpE-like 1, mitochondrial	5851.632	6136.172	2516.503
1370174_at	171071	Myd116	myeloid differentiation primary respons	5851.445	823.8005	3397.224
1367718_at	29367	Chkb	choline kinase beta	5848.365	4775.074	4635.057
1393239_at	311403	Mtfr1_pred	mitochondrial fission regulator 1 (predi	5840.657	4126.992	5726.921
1379441_at	288308	Zfp294	zinc finger protein 294	5839.094	8468.184	5373.847
1367878_at	65134	Stx5a	syntaxin 5a	5838.282	4110.917	2022.853
1370220_at	114861	Scpep1	serine carboxypeptidase 1	5836.644	5736.389	1663.237
1369950_at	94201	Cdk4	cyclin-dependent kinase 4	5828.137	13203.02	4049.257
1382101_at	292155	Hs2st1	heparan sulfate 2-O-sulfotransferase 1	5822.086	3898.327	5288.737
1383068_at	301622	Dtymk_pre	deoxythymidylate kinase (predicted)	5821.029	4182.981	5986.764
1368436_at	29648	Nudc	nuclear distribution gene C homolog (A	5807.082	4168.529	6656.844
1371912_at	362837	Ndufs7	NADH dehydrogenase (ubiquinone) Fe	5806.028	6760.505	3729.138
1371692_at	295264	Mllt11	myeloid/lymphoid or mixed-lineage leu	5804.74	4180.731	2480.491
1367674_at	114561	Pitpnb	phosphatidylinositol transfer protein, be	5802.627	7672.242	4186.491
1399091_at	298584	Capzb	capping protein (actin filament) muscle	5801.71	5287.021	2602.124
1389372_at	313340	RGD13084	similar to hypothetical protein (predicte	5801.239	4946.586	10673.38
1371420_at	494529	LOC49452	92Aa-Protein	5801.085	5372.256	12377.19
1377869_at	171555	Ccrn4l	CCR4 carbon catabolite repression 4-l	5799.243	7044.427	17419.63

1375977_at	84593	Cetn2	centrin 2	5796.737	14848.45	13804.17
1373064_at	290370	Dnajc15_p	DnaJ (Hsp40) homolog, subfamily C, r	5795.926	3425.347	2911.907
1374078_at	362166	RGD15606	similar to PHF21A protein (predicted)	5794.025	2667.212	3410.318
1390788_a_at	361305	Wdr36_pre	WD repeat domain 36 (predicted)	5793.785	6055.438	3470.572
1389967_at	293551	Arl6ip1	ADP-ribosylation factor-like 6 interactir	5792.759	13250.15	8109.455
1370848_at	24778	Slc2a1	solute carrier family 2 (facilitated gluco	5787.641	1712.393	2706.254
1371463_at	192246	Phf5a	PHD finger protein 5A	5787.404	14237.56	3244.691
1383239_at	298086	Zcchc7_pre	zinc finger, CCHC domain containing 7	5784.904	2437.55	8866.985
1388000_at	84550	Slc24a2	solute carrier family 24 (sodium/potass	5784.51	3298.023	763.8539
1388487_at	24170	Add1	adducin 1 (alpha)	5782.689	4376.674	4186.957
1383135_at	25054	Ntrk2	neurotrophic tyrosine kinase, receptor,	5782.504	2366.557	1393.294
1387051_at	84496	Stau1	staufen RNA binding protein homolog	5782.04	9790.554	5035.685
1387503_at	365466	Cpn1	carboxypeptidase N, polypeptide 1, 50	5781.459	5503.206	7895.812
1371817_at	290651	LOC29065	similar to myo-inositol 1-phosphate syr	5778.125	6734.775	2442.079
1389658_at	361191	Nsun2_pre	NOL1/NOP2/Sun domain family, mem	5775.706	6363.349	4428.41
1388107_at	246255	Ppp2r2d	protein phosphatase 2, regulatory subu	5773.902	5995.031	8491.741
1383256_at	295419	Dnttip2_pre	deoxynucleotidyltransferase, terminal,	5770.725	2596.517	6206.481
1377654_at	501664	RGD15620	similar to RIKEN cDNA 1810037C20 (p	5760.93	6306.978	3591.214
1380909_at	310791	Slc25a24_p	solute carrier family 25 (mitochondrial c	5758.364	4021.018	7103.887
1388120_at	501083	Pdcd6ip	programmed cell death 6 interacting pr	5753.488	3311.736	1764.873
1385660_at	314388	RGD13090	similar to KIAA2010 protein (predicted)	5751.047	2354.465	10568.87
1388397_at	114021	Ebna1bp2	EBNA1 binding protein 2	5750.414	13409.8	5132.207
1384756_at	287532	Slc43a2_pr	solute carrier family 43, member 2 (pre	5747.807	3320.224	1511.766
1386952_a_at	116659	Dncic2	dynein, cytoplasmic, intermediate chai	5743.177	4811.577	10718.12
1371466_at	501550	RGD13069	similar to RIKEN cDNA 0610009B22	5734.983	3485.027	7372.1
1390604_s_at	362548	Itgb3bp	integrin beta 3 binding protein (beta3-e	5731.094	9388.019	4111.979
1374638_at	305581	Pex13_pre	peroxisomal biogenesis factor 13 (prec	5730.772	4631.693	7037.88
1389409_at	500040	LOC50004	similar to Testis derived transcript	5729.412	2166.618	6085.443
1388477_at	501281	LOC50128	NA	5726.029	6022.187	2911.407
1390278_at	500116	RGD15625	similar to RIKEN cDNA G430041M01 (	5722.207	5524.003	17718.05
1367654_at	83720	Fath	fat tumor suppressor homolog (Drosop	5720.142	17075.93	17825.3
1387035_a_at	63994	Arhgap17	Rho GTPase activating protein 17	5714.998	3157.17	10373.08
1386878_at	305880	Lrp10	low-density lipoprotein receptor-relatec	5714.308	2181.753	2050.772
1384302_at	613226	Slc6a17	NA	5710.826	5749.53	26557.01
1375162_at	295252	Ube2q_pre	ubiquitin-conjugating enzyme E2Q (pu	5707.294	6398.279	5998.791
1383290_at	311331	Spint1	serine peptidase inhibitor, Kunitz type	5707.179	3012.783	2621.016
1368839_at	83725	Wfs1	Wolfram syndrome 1 homolog (human	5705.24	1067.353	1363.845
1389845_at	360481	Dnaja3	DnaJ (Hsp40) homolog, subfamily A, n	5705.069	6726.519	3069.055
1371778_at	308051	Rnf146	ring finger protein 146	5705.003	7780.6	14071.52
1371809_at	294230	Mrps18b	mitochondrial ribosomal protein S18B	5697.713	6198.983	2316.982
1398863_at	81667	Gnb2	guanine nucleotide binding protein, be	5695.409	12079.12	2200.04
1389538_at	25493	Nfkbia	nuclear factor of kappa light chain gen	5694.884	1122.952	13083.54
1387219_at	25026	Adm	adrenomedullin	5692.826	32.16552	238.3257
1383368_at	304881	Tor1aip2	torsin A interacting protein 2	5690.792	6082.046	7865.662
1369990_at	360243	Top2a	topoisomerase (DNA) 2 alpha	5690.339	4279.568	3027.529
1388800_at	64633	Rab5a	RAB5A, member RAS oncogene family	5688.816	5581.331	18484.76
1389481_at	294311	RGD73506	similar to GI:13385412-like protein spli	5684.086	9834.85	2558.207
1390000_at	303242	DLP2	dynein-like protein 2	5683.673	3288.959	3264.265
1379186_at	304017	Tomm70a	translocase of outer mitochondrial mer	5678.07	5689.35	4721.106
1389325_at	494345	MGC72992	similar to programmed cell death 10	5677.789	11310.57	13104.6
1369969_at	25591	Parp1	poly (ADP-ribose) polymerase family, r	5677.252	7752.401	3315.055

1371631_at	293673	RGD1304€	similar to 2010003J03Rik protein	5677.209	4917.719	1288.17
1375438_at	64154	Gosr2	golgi SNAP receptor complex member	5673.97	5050.459	3453.006
1374903_at	306860	Gcnt2	glucosaminyl (N-acetyl) transferase 2,	5669.993	6544.747	23567.34
1370976_at	171092	G3bp	Ras-GTPase-activating protein SH3-d	5664.156	6643.256	11010.93
1368514_at	25750	Maob	monoamine oxidase B	5662.414	28022.32	22419.25
1389569_at	294799	Bxdc2	brix domain containing 2	5662.019	5452.937	3991.617
1386154_at	367235	RGD1304S	similar to KIAA1411 protein (predicted)	5659.983	7890.605	6760.318
1398867_at	246216	Prpf19	PRP19/PSO4 pre-mRNA processing fa	5659.266	14766.61	3555.609
1389265_at	288333	Gbe1	glucan (1,4-alpha-), branching enzyme	5657.837	2542.364	6766.518
1372480_at	313176	Zfp462_pre	zinc finger protein 462 (predicted)	5650.515	3179.511	2394.741
1370613_s_at	154516	Ugt1a7	UDP glycosyltransferase 1 family, poly	5650.245	3735.423	3433.134
1389685_at	360764	Zfp655	zinc finger protein 655	5645.429	5233.207	14152.32
1372948_at	498525	LOC49852	Bm403207	5645.084	5104.487	2345.486
1390688_at	361848	Ddx50	DEAD (Asp-Glu-Ala-Asp) box polypept	5641.176	4241.455	8805.884
1375663_at	363284	Ube2f	ubiquitin-conjugating enzyme E2F (put	5637.643	2669.324	2849.718
1375417_at	296374	Prkcbp1	protein kinase C binding protein 1	5636.996	2328.344	3657.264
1373123_at	306734	RGD1310€	similar to RIKEN cDNA 4932432N11 g	5635.193	11143.03	7237.721
1398963_at	293345	Taf10_prec	TAF10 RNA polymerase II, TATA box	5632.527	6558.035	3116.463
1371482_at	289218	Ndufs2	NADH dehydrogenase (ubiquinone) Fe	5631.251	5485.806	5084.58
1398976_at	54299	Ncor1	nuclear receptor co-repressor 1	5629.922	5209.917	10208.97
1374189_at	305848	Zfp219	zinc finger protein 219	5626.913	1375.026	3340.424
1371498_at	288480	MGC12527	JTV1	5625.571	6261.746	3183.109
1371715_at	500651	MGC11288	LOC500651	5624.097	4120.859	7320.125
1390109_at	363113	Syncrip	synaptotagmin binding, cytoplasmic RI	5616.498	3496.929	9023.194
1370925_at	291411	LOC29141	similar to Potential phospholipid-transp	5604.506	10135.82	1456.28
1367602_at	114490	Cited2	Cbp/p300-interacting transactivator, wi	5603.724	6667.309	39548.59
1371737_at	291171	Trim27_pre	tripartite motif protein 27 (predicted)	5601.632	4167.423	3680.95
1383144_at	312616	Trnt1	tRNA nucleotidyl transferase, CCA-add	5601.443	5874.158	4419.536
1369980_s_at	116504	Mrip	myosin phosphatase-Rho interacting p	5601.33	7096.322	3345.659
1389652_at	290706	RGD13117	similar to 2700029M09Rik protein (pre	5598.212	5384.519	11896.64
1376583_at	307907	RGD13048	similar to RIKEN cDNA 6430548M08 (	5594.669	1301.221	2838.075
1387909_at	286928	Abi2	abl-interactor 2	5587.609	4372.309	8038.503
1390208_at	292935	Htatip2_pre	HIV-1 tat interactive protein 2, homolog	5586.847	4677.883	906.5757
1398471_at	310411	Cog6	component of oligomeric golgi complex	5584.637	4122.238	3177.175
1369462_at	24380	Gad2	glutamic acid decarboxylase 2	5582.367	392.6228	103.0361
1390648_at	308669	Herc2_prec	hect (homologous to the E6-AP (UBE3	5581.02	3288.687	5052.59
1373898_at	360760	LOC36076	NA	5580.977	6066.602	13457.74
1369590_a_at	29467	Ddit3	DNA-damage inducible transcript 3	5573.088	1039.661	19222.75
1388732_at	288993	Slc35f5_pr	solute carrier family 35, member F5 (p	5571.971	3264.534	5881.522
1383056_a_at	499072	RGD15652	RGD1565210 (predicted)	5571.832	2959.301	836.9415
1390343_at	114839	Ccnc	cyclin C	5571.664	6660.956	10325.19
1372130_at	312915	Arfgef1_pr	ADP-ribosylation factor guanine nucleo	5567.303	6740.946	11943.47
1367591_at	64371	Prdx3	peroxiredoxin 3	5567.289	4479.874	3368.746
1371467_at	293103	LOC29310	similar to RIKEN cDNA 0610007P06	5566.01	7181.313	3691.653
1389165_at	498301	RGD1565	similar to myocardial ischemic precond	5561.089	5991.935	9302.128
1387080_at	29486	Cspg6	chondroitin sulfate proteoglycan 6	5557.654	9842.852	10976.14
1389001_at	361959	Kpna4	karyopherin (importin) alpha 4	5557.109	3269.303	4381.989
1388819_at	29521	Scamp1	secretory carrier membrane protein 1	5546.095	8254.871	9748.259
1389181_at	303141	Rapgef6_p	Rap guanine nucleotide exchange fact	5545.967	2691.094	4658.799
1371966_at	25604	Pcmt1	protein-L-isoaspartate (D-aspartate) O	5534.916	7903.146	5997.036
1374876_at	361160	Leprotl1	leptin receptor overlapping transcript-li	5531.358	4425.141	1552.999

1388542_at	290627	RGD13066	similar to RIKEN cDNA 2010315L10 (p	5528.975	5362.191	7428.641
1371614_at	361321	Atg12	autophagy-related 12 (yeast)	5528.251	7405.508	4111.346
1380102_at	293862	RGD15609	similar to XAP-5 protein (predicted)	5522.989	5977.141	5352.961
1372108_at	500199	LOC50019	similar to RIKEN cDNA 2810422B04	5505.919	5746.337	2686.35
1380371_at	294787	NIPBL	Nipped-B homolog (Drosophila)	5505.396	2834.674	2729.738
1368018_at	83536	Mkln1	muskelin 1, intracellular mediator conta	5505.303	4564.393	3723.867
1382526_at	313115	RGD13093	similar to RIKEN cDNA 1810074P20 (p	5498.774	3844.585	2744.403
1393647_at	296501	Hat1	histone aminotransferase 1	5494.924	9470.603	4184.099
1399039_at	501165	NA	NA	5493.154	1409.699	2266.16
1383478_at	84598	Jak1	Janus kinase 1	5492.824	12476.65	14676.9
1370199_at	84595	Nucb1	nucleobindin 1	5484.356	6168.838	3200.12
1373054_at	85254	Cdw92	CDW92 antigen	5482.28	3974.085	3075.621
1367491_at	362170	Api5_predi	apoptosis inhibitor 5 (predicted)	5478.668	10598.96	7407.139
1372160_at	171113	Blcap	bladder cancer associated protein hom	5475.472	3263.725	2010.667
1374788_at	296099	Trp53bp1_	transformation related protein 53 bindi	5470.137	3825.097	5667.896
1370968_at	81736	Nfkb1	nuclear factor of kappa light chain gen	5468.803	1071.914	8611.083
1376919_at	301388	RGD15623	similar to expressed sequence AW212	5464.651	9263.51	8770.938
1375892_at	363854	Elavl1_pre	ELAV (embryonic lethal, abnormal visi	5457.433	7910.601	6671.496
1387048_at	89827	Ddx39	DEAD (Asp-Glu-Ala-Asp) box polypept	5451.317	5369.535	1984.328
1374398_at	303281	Supt6h	suppressor of Ty 6 homolog (S. cerevi	5449.098	3381.927	4523.627
1398383_at	303601	Cyb561_pr	cytochrome b-561 (predicted)	5447.386	6483.178	3778.316
1373479_at	24674	Ppp3ca	protein phosphatase 3, catalytic subun	5435.285	4576.452	10805.45
1370098_at	85491	Sybl1	synaptobrevin-like 1	5435.106	8377.632	1333.839
1378361_at	312974	Chd7_pred	chromodomain helicase DNA binding p	5434.706	3816.858	4289.728
1371597_at	360533	Rnf187_pre	ring finger protein 187 (predicted)	5432.383	9867.119	3413.868
1374151_at	361600	Tm6sf1_pr	transmembrane 6 superfamily member	5425.123	3645.533	6122.936
1372283_at	299075	Trappc6b_	trafficking protein particle complex 6B	5421.521	2658.505	7796.016
1388364_at	295923	Ndufs3_pre	NADH dehydrogenase (ubiquinone) Fe	5418.971	8782.885	2908.595
1389577_at	291987	Cirh1a	cirrhosis, autosomal recessive 1A (hur	5413.95	2942.872	3730.811
1387905_at	619393	LOC61939	NA	5410.472	7787.537	10807.73
1368050_at	114121	Ccnl1	cyclin L1	5410.197	3822.781	35869.17
1371486_at	361808	Snrp1c_pre	U1 small nuclear ribonucleoprotein 1C	5402.233	12626.37	5061.422
1373954_at	360819	RGD13059	similar to FLJ00052 protein (predicted)	5401.52	7084.247	10943.84
1367793_at	29318	Ddt	D-dopachrome tautomerase	5401.36	4391.022	1339.41
1372808_at	313410	RGD15640	similar to methylenetetrahydrofolate de	5401.103	1061.202	2258.379
1367669_a_at	64862	Map1lc3b	microtubule-associated protein 1 light	5398.188	2626.988	2080.121
1371612_at	292534	TSEN34	tRNA splicing endonuclease 34 homol	5391.77	10615.95	4197.352
1367826_at	83619	Nfe2l2	nuclear factor, erythroid derived 2, like	5385.925	4338.86	5575.2
1376437_at	287464	Dhx33_pre	DEAH (Asp-Glu-Ala-His) box polypepti	5385.423	3525.327	1663.802
1388900_at	361797	RGD15661	RGD1566118 (predicted)	5384.617	6073.578	4216.165
1372776_at	305424	Fbxl5_prec	F-box and leucine-rich repeat protein 5	5383.638	3556.041	9611.002
1371890_at	116665	Rere	arginine-glutamic acid dipeptide (RE) r	5383.054	1940.385	15391.65
1371822_at	306012	Polr3d	polymerase (RNA) III (DNA directed) p	5381.679	3139.023	2262.333
1395426_at	499945	RGD15622	similar to ubiquitin-conjugating enzyme	5377.223	15644.16	2116.513
1372857_at	124461	Pacsin2	protein kinase C and casein kinase sul	5374.957	2828.882	4374.04
1388794_at	307779	Rbmxrt_pre	RNA binding motif protein, X chromoso	5367.916	7557.088	6830.952
1367941_at	83474	Tfam	transcription factor A, mitochondrial	5367.586	9694.631	7543.651
1399009_at	308837	RGD13050	similar to RIKEN cDNA 2610034N24 (p	5366.565	5049.486	2487.308
1389305_at	79124	Anxa4	annexin A4	5365.35	5381.454	9522.024
1373409_at	362294	RGD15599	similar to ubiquitin protein ligase E3C (	5361.248	6415	2553.156
1386984_at	50554	Smad4	MAD homolog 4 (Drosophila)	5357.834	8042.027	7640.472

1388810_at	361390	Abce1	ATP-binding cassette, sub-family E (O	5355.024	3784.1	2527.931
1372834_at	360834	Vps4b	vacuolar protein sorting 4b (yeast)	5351.615	4564.237	7792.825
1386960_at	29573	Slc37a4	solute carrier family 37 (glycerol-6-pho	5349.443	6757.15	2983.997
1367765_at	64365	Tcn2	transcobalamin 2	5345.908	3625.19	2907.385
1388160_a_at	94173	Idh3B	isocitrate dehydrogenase 3 (NAD+) be	5345.61	11448.41	3739.642
1370934_at	25281	Nup153	nucleoporin 153	5344.281	6318.76	17504.12
1368037_at	29224	Cbr1	carbonyl reductase 1	5343.818	9523.116	3296.866
1370815_at	24587	Nefh	neurofilament, heavy polypeptide	5341.644	7467.778	898.5314
1384314_at	305606	Mtif2	mitochondrial translational initiation fac	5341.437	5448.191	6426.728
1371784_at	303536	Dnajc7	DnaJ (Hsp40) homolog, subfamily C, r	5341.321	8874.418	3898.837
1385109_at	363017	Josd3	Josephin domain containing 3	5339.721	4373.388	3724.184
1368097_a_at	116644	Rtn1	reticulon 1	5338.486	4468.386	12993.47
1372543_at	363485	RGD15625	similar to RIKEN cDNA 2610029G23 (	5334.338	5038.541	6681.564
1388823_at	288779	Rab5b_pre	RAB5B, member RAS oncogene family	5333.874	5362.44	2101.71
1373462_at	293104	Eed_predic	embryonic ectoderm development (pre	5332.186	5650.21	5040.318
1389559_at	362919	NA	NA	5332.161	5185.427	3661.515
1388772_at	296913	Lsm8_prec	LSM8 homolog, U6 small nuclear RNA	5329.779	7320.575	6137.911
1367683_at	85245	Kpna2	karyopherin (importin) alpha 2	5328.705	19801.37	2415.636
1375884_at	500941	MGC10556	similar to Hypothetical protein BC0147	5328.494	3147.101	6984.865
1379317_a_at	309458	Pdcd11_pr	programmed cell death protein 11 (pre	5327.941	7171.411	2814.862
1388180_at	286917	Phax	phosphorylated adaptor for RNA expor	5326.24	7336.704	4462.69
1388354_at	296126	Ascc3l1	activating signal cointegrator 1 comple	5320.649	5067.447	3654.894
1376268_at	79121	Arf6	ADP-ribosylation factor 6	5320.013	4416.53	11281.32
1376687_at	313387	Usp1	ubiquitin specific peptdiase 1	5319.367	13477.75	2771.222
1373734_at	140915	Slco3a1	solute carrier organic anion transporter	5318.205	5740.841	2084.494
1399074_at	290875	Cdc16	CDC16 cell division cycle 16 homolog	5316.847	8323.475	4203.561
1373015_at	114559	Arhgef7	Rho guanine nucleotide exchange fact	5316.011	5950.285	17642.21
1375634_at	299707	Ccdc53_pr	coiled-coil domain containing 53 (predi	5315.97	19317.15	10966.34
1394414_at	362699	Yipf4	Yip1 domain family, member 4	5315.532	4218.932	14499
1383647_a_at	301112	Crb3	crumbs homolog 3 (Drosophila)	5315.079	3562.056	2446.1
1375550_at	297994	Bag1_pred	Bcl2-associated athanogene 1 (predict	5314.064	3550.326	2118.512
1391432_at	314545	Wdr75	WD repeat domain 75	5310.364	4140.781	5121.164
1392045_at	315957	Tmem22	transmembrane protein 22	5309.942	4979.915	3913.194
1371373_at	297337	MGC94464	similar to RIKEN cDNA 2500002L14; E	5309.697	4676.663	1849.386
1384244_at	313200	RGD13053	similar to RIKEN cDNA 2610207I16	5308.003	2778.086	7141.782
1381605_at	310306	Usp13_pre	ubiquitin specific protease 13 (isopepti	5295.069	1675.368	1782.239
1375958_at	304361	LOC30436	similar to Hypothetical protein MGC28	5291.87	1781.496	1724.896
1373915_at	308405	Dmpk_prec	dystrophia myotonica-protein kinase (p	5286.084	833.5903	2102.978
1382141_at	310668	Setdb1_pre	SET domain, bifurcated 1 (predicted)	5282.131	3300.152	4902.804
1371524_at	307642	Gtl3	gene trap locus 3	5281.261	8109.276	10124.05
1392484_at	299262	NA	NA	5277.544	7123.764	5910.961
1368848_at	116666	Lman1	lectin, mannose-binding, 1	5277.351	5512.169	984.0558
1399020_at	362012	LOC36201	similar to family with sequence similari	5276.753	3576.459	1981.775
1376676_a_at	290270	RGD13051	similar to Ab2-008	5272.788	2329.744	10113.72
1372563_at	501194	LOC50119	NA	5272.728	3267.753	12043.62
1383324_at	500121	NA	NA	5272.257	4728.685	2342.169
1398988_at	307347	LOC30734	hypothetical protein LOC307347	5267.093	6217.135	9067.52
1373507_at	307170	Acbd5	acyl-Coenzyme A binding domain cont	5257.626	4368.738	12745.74
1375526_at	287442	RGD15634	similar to novel protein of unknown fun	5255.051	4308.33	4018.99
1375043_at	314322	Fos	FBJ murine osteosarcoma viral oncoge	5253.368	137.5958	82257.84
1390047_at	361293	Riok3_prec	RIO kinase 3 (yeast) (predicted)	5253.036	1537.543	3800.03

1372813_at	360617	RGD13066	similar to RIKEN cDNA 1810046J19 (p	5252.656	9874.416	3573.09
1399075_at	313121	Map3k7_pr	mitogen activated protein kinase kinas	5250.03	9970.976	5628.93
1389334_at	314071	Ndufa10	NADH dehydrogenase (ubiquinone) 1	5246.369	5462.358	3555.803
1376191_at	79242	Hpgd	hydroxyprostaglandin dehydrogenase	5244.889	3703.554	4345.029
1398247_at	170916	Prss15	protease, serine, 15	5244.53	3438.595	1518.165
1375960_at	312516	Gmcl1	germ cell-less homolog 1 (Drosophila)	5243.268	5908.865	5004.839
1372338_at	366146	NA	NA	5242.806	4824.508	7724.512
1384525_at	313438	Dock11	dedicator of cytokinesis 11	5238.493	11934.54	47768.91
1382255_at	297508	Pbef1	pre-B-cell colony enhancing factor 1	5237.416	2239.678	10111.96
1367547_at	305913	LOC30591	NA	5236.141	3941.635	10470.07
1372243_at	301574	Cab39_pre	calcium binding protein 39 (predicted)	5233.002	2576.096	1469.189
1387732_at	85261	Mterf	mitochondrial transcription termination	5231.891	3752.142	5243.528
1368523_at	26989	Cadps	Ca2+-dependent secretion activator	5229.846	3422.829	26355.32
1371453_at	301544	Farslb	phenylalanine-tRNA synthetase-like, b	5225.877	4245.879	2000.455
1389629_at	366276	RGD13071	similar to RIKEN cDNA 1190003A07 (p	5224.719	4006.566	4807.143
1392399_at	362902	Wdsof1_pr	WD repeats and SOF domain containi	5224.594	5513.691	4782.095
1398834_at	58960	Map2k2	mitogen activated protein kinase kinas	5222.091	4928.467	2913.589
1368272_at	24401	Got1	glutamate oxaloacetate transaminase	5219.627	2272.9	3283.571
1371442_at	192235	Hyou1	hypoxia up-regulated 1	5218.398	223.6191	1319.037
1375347_at	303617	Falz_predic	fetal Alzheimer antigen (predicted)	5213.865	5429.818	13964.27
1368181_at	64300	Mthfd1	methylenetetrahydrofolate dehydroger	5210.703	3906.108	1961.831
1376641_at	291797	Thoc1	THO complex 1	5208.539	5044.733	8219.491
1375521_at	367909	Tceal8	transcription elongation factor A (SII)-li	5207.367	5989.903	5249.065
1375861_at	362377	Herc3_pred	hect domain and RLD 3 (predicted)	5200.934	3469.189	38807.69
1389294_at	308666	Cyfip1_pre	cytoplasmic FMR1 interacting protein 1	5195.793	5471.659	7606.592
1374599_at	315771	Herc1_pred	hect (homologous to the E6-AP (UBE3	5190.036	2183.427	3853.479
1375590_at	360845	RGD13082	similar to RIKEN cDNA 5730454B08 (p	5189.376	2883.353	1466.751
1368508_at	408248	Psma3l	NA	5189.027	5951.138	6392.014
1398424_at	288692	Wsb2	WD repeat and SOCS box-containing	5188.813	4877.952	4963.222
1388917_at	25485	Myo1d	myosin ID	5180.68	2459.33	5722.316
1374815_at	291182	Stard3nl	STARD3 N-terminal like	5180.279	3465.496	4106.309
1371683_at	290647	Lsm4_pred	LSM4 homolog, U6 small nuclear RNA	5173.505	10145.05	4002.712
1373222_at	300757	Hexa	hexosaminidase A	5171.6	6513.381	13269.56
1368194_at	170919	Agpat4	1-acylglycerol-3-phosphate O-acyltran	5166.818	3601.469	1078.557
1373465_at	361352	Pqlc1	PQ loop repeat containing 1	5165.936	2588.634	8621.364
1377685_at	498820	NA	NA	5162.805	5955.907	10480.33
1368276_at	24804	Syp	synaptophysin	5153.906	5175.818	3087.627
1383205_at	308212	Dact2_pred	dapper homolog 2, antagonist of beta-	5150.92	2071.857	6707.129
1372441_at	117535	Chd4	chromodomain helicase DNA binding p	5150.256	2837.557	1640.348
1373280_at	292907	Ruvbl2	RuvB-like 2 (E. coli)	5145.574	6881.321	1441.2
1390506_at	497991	RGD15595	similar to peroxisome proliferator-activ	5139.897	3758.97	1463.212
1376917_at	50552	Znf292	zinc finger protein 292	5138.573	3606.827	2593.667
1372722_at	295549	Dnajb4	DnaJ (Hsp40) homolog, subfamily B, m	5137.374	2971.122	15624.92
1392662_at	295457	RGD15662	similar to RIKEN cDNA 1500009M05 (	5135.377	2471.961	4237.937
1367750_at	64390	Prpsap1	phosphoribosyl pyrophosphate synthet	5134.881	3097.485	1864.426
1389476_at	294978	RGD13071	similar to RIKEN cDNA D630029K19	5134.296	3101.815	9319.768
1383089_at	299799	Rab21	RAB21, member RAS oncogene family	5131.145	4073.368	16963.35
1394760_at	314401	RGD13111	similar to KIAA1409 protein (predicted)	5120.556	5825.607	5881.784
1375022_at	307350	Afg3l2	AFG3(ATPase family gene 3)-like 2 (y	5117.771	2894.952	2315.19
1372477_at	294207	Ppp1r11	protein phosphatase 1, regulatory (inhi	5117.366	5755.537	3667.642
1372538_at	300083	Slc25a17_	solute carrier family 25 (mitochondrial	5111.22	4365.965	7682.728

1389883_at	500874	RGD15632	similar to 4930438D12Rik protein (pre	5105.641	6166.59	6318.685
1371940_at	362587	LOC36258	NA	5104.077	6087.09	4275.09
1371949_at	363232	Bzw1	basic leucine zipper and W2 domains	5091.506	5535.98	5121.215
1385671_at	303113	Phf15_pre	PHD finger protein 15 (predicted)	5075.512	5587.592	4052.676
1390311_at	171572	Ttl	tubulin tyrosine ligase	5071.813	4862.858	1958.334
1382556_a_at	290537	RGD13093	similar to DIP13 alpha (predicted)	5071.55	3399.029	4204.512
1367651_at	171293	Ctsd	cathepsin D	5069.335	8906.144	1543.609
1387110_at	25499	Nrd1	nardilysin, N-arginine dibasic converta	5068.789	4619.916	4458.457
1394397_at	302898	LOC30289	Ac1158	5058.414	4766.533	3473.741
1394330_at	361915	LOC36191	NA	5055.275	327.9852	5833.749
1376067_at	299805	Cnot2	CCR4-NOT transcription complex, sub	5053.244	7274.792	10757.78
1369641_at	64189	Pafah1b2	platelet-activating factor acetylhydrolas	5051.611	3609.468	2743.795
1378665_at	306091	Pcdh9_pre	protocadherin 9 (predicted)	5047.585	196.7841	3836.006
1372558_at	310399	Narg1_pre	NMDA receptor-regulated gene 1 (pre	5046.884	6526.919	4353.548
1367875_at	81659	Gak	cyclin G associated kinase	5045.809	4176.967	4277.955
1397556_at	288108	Mak3_pred	Mak3 homolog (S. cerevisiae) (predict	5043.655	2856.58	2425.23
1388511_at	361617	Centd2	centaurin, delta 2	5042.157	1370.266	4102.661
1390454_at	360971	Nipsnap1	4-nitrophenylphosphatase domain and	5040.959	5574.85	1943.208
1398927_at	305031	RGD13071	similar to 0610010K06Rik protein	5038.654	5150.737	5970.355
1370407_at	246302	Pcyox1	prenylcysteine oxidase 1	5037.806	2482.425	2846.52
1371425_at	313620	Srrm1_pre	serine/arginine repetitive matrix 1 (pre	5036.836	2392.691	6559.825
1388683_at	287429	RGD15596	similar to hypothetical protein MGC141	5034.478	10050.02	5151.822
1382084_at	500464	RGD15629	similar to EHM2 (predicted)	5032.587	1564.398	2338.019
1393162_at	291733	Slc39a6	solute carrier family 39 (metal ion trans	5030.622	4845.242	2781.85
1392894_at	84586	Fgl2	fibrinogen-like 2	5025.761	5811.995	6372.177
1389286_at	362776	Glrx5_pred	glutaredoxin 5 homolog (S. cerevisiae)	5022.537	8664.845	2517.842
1371812_at	362855	LOC36285	P55	5020.765	8599.745	5787.766
1388367_at	362385	Pole4_pred	polymerase (DNA-directed), epsilon 4	5008.216	8710.146	4214.955
1368967_at	171145	Eif2b3	eukaryotic translation initiation factor 2	5006.291	3546.986	1309.871
1392888_at	317322	Gpc4	glypican 4	4987.208	6829.851	8087.99
1367716_at	171083	Cda08	T-cell immunomodulatory protein	4986.58	4543.063	14373.79
1373940_at	300955	Nck1_pred	non-catalytic region of tyrosine kinase	4986.496	3132.773	6843.038
1371853_at	299743	Mrpl42_pre	mitochondrial ribosomal protein L42 (p	4982.504	10858.44	4166.794
1371957_at	316317	Imp4	IMP4, U3 small nucleolar ribonucleopr	4982.155	5279.323	2124.466
1372246_at	64040	Aldh9a1	aldehyde dehydrogenase family 9, sub	4979.559	5500.389	3039.255
1373624_at	312846	Rassf8_pre	Ras association (RalGDS/AF-6) doma	4978.489	4302.744	2365.144
1371479_at	315306	Mettl7a	methyltransferase like 7A	4976.514	2743.436	3617.602
1372185_at	362103	RGD13065	similar to RIKEN cDNA 2610205E22	4975.777	4365.894	3796.355
1373777_at	360857	Rgs16	NA	4974.35	4827.251	2380.939
1375853_at	314690	RGD13099	similar to CG13957-PA (predicted)	4973.632	4661.662	13950.07
1389587_at	288051	Umps	uridine monophosphate synthetase	4973.15	7071.016	1931.566
1388555_at	287474	Txnl5_pred	thioredoxin-like 5 (predicted)	4968.316	5763.632	1741.562
1391494_at	364033	Vamp4_pre	vesicle-associated membrane protein 4	4968.007	2066.927	2395.213
1388619_at	315655	Rdx	radixin	4965.702	2988.67	12217.57
1371421_at	361902	RGD13054	similar to FLJ40243 protein	4961.856	6670.921	11424.95
1372401_at	298071	Nans_pred	N-acetylneuraminic acid synthase (sial	4959.771	6397.089	3079.378
1375898_at	498642	RGD15610	similar to RNA binding protein gene wi	4958.638	5863.221	6666.667
1369030_at	24691	Prss1	pancreatic trypsin 1	4957.64	198.6385	12665.73
1389373_at	25671	Smad1	MAD homolog 1 (Drosophila)	4955.979	2806.105	6441.536
1373017_at	362404	Suclg2	succinate-Coenzyme A ligase, GDP-fo	4954.827	5548.531	7477.482
1399047_at	287635	Mrpl27_pre	mitochondrial ribosomal protein L27 (p	4951.454	12631.55	3435.47

1367834_at	84596	Srm	spermidine synthase	4950.42	2722.623	1295.009
1381903_at	314157	Fbxo33_pr	F-box only protein 33 (predicted)	4950.037	1916.335	21214.14
1371649_at	498406	RGD15646	similar to mitochondrial ribosomal prot	4948.627	5738.148	3997.552
1392936_at	497865	RGD15654	similar to RNA binding motif protein 25	4948.25	2340.647	2518.807
1389178_at	499191	Ngrn	NA	4947.038	4746.358	5921.726
1374484_at	288092	Tmem39a	transmembrane protein 39a	4940.832	3109.99	5032.968
1383829_at	303970	Bbx_predic	bobby sox homolog (Drosophila) (pred	4940.681	3164.298	3273.244
1392541_at	362368	RGD13048	similar to RIKEN cDNA A030007L17; E	4940.251	2504.783	4835.936
1377842_at	362728	Twistnb_pr	TWIST neighbor (predicted)	4939.757	3083.337	4631.89
1367520_at	295229	Apoa1bp_p	apolipoprotein A-I binding protein (pred	4938.388	8317.272	4369.8
1379348_at	366017	Exosc2_pre	exosome component 2 (predicted)	4938.333	2382.508	1933.764
1374209_at	290823	Spfh2_pred	SPFH domain family, member 2 (predi	4938.143	6681.658	5738.69
1371860_at	292640	RGD13050	similar to RIKEN cDNA 1110059P08	4935.857	8892.171	3618.195
1387784_at	116722	Psmd10	proteasome (prosome, macropain) 26S	4933.792	4840.195	3653.108
1374034_at	293638	Cars_predi	cysteinyl-tRNA synthetase (predicted)	4932.959	2369.313	3359.022
1372478_at	501065	LOC50106	NA	4929.751	468.8346	1535.619
1394793_at	503454	RGD15607	similar to LRRGT00057 (predicted)	4928.572	933.3311	4266.23
1388708_at	364531	NA	NA	4927.321	4178.458	1077.5
1372372_at	310201	RGD13069	similar to Ab2-225	4927.194	5892.059	6458.976
1389523_at	287119	Zfp598_pre	zinc finger protein 598 (predicted)	4922.806	2566.869	1594.266
1372989_at	499014	LOC49901	similar to zinc finger, DHHC domain co	4921.855	6711.449	9189.225
1371745_at	300361	RGD13106	hypothetical LOC300361	4921.826	10432.69	3925.756
1373161_at	303356	Tmem98	transmembrane protein 98	4916.341	3847.304	4132.949
1391474_at	317343	RGD15601	similar to cofactor required for Sp1 trar	4914.357	4524.479	7670.568
1372309_at	312251	Luc7l2_pre	LUC7-like 2 (S. cerevisiae) (predicted)	4914.001	4595.172	6702.189
1376098_a_at	289785	Myo1g	myosin IG	4911.235	2130.3	6156.2
1388450_at	84479	Ap1gbp1	AP1 gamma subunit binding protein 1	4905.836	2343.701	2401.037
1372505_at	64351	Ykt6	prenylated SNARE protein	4902.856	2686.772	847.4362
1371432_at	287721	Vat1	vesicle amine transport protein 1 homo	4897.786	1952.075	2445.544
1391495_at	294012	RGD13117	similar to RIKEN cDNA 2010012O05 (	4896.825	18575.49	2732.662
1371850_at	295443	Papss1_pre	3'-phosphoadenosine 5'-phosphosulfat	4896.52	3488.914	5271.912
1389021_at	299507	RGD13063	similar to HCV NS3-transactivated prot	4896.115	6157.428	4386.688
1388714_at	306347	Ell_predicte	elongation factor RNA polymerase II (p	4895.197	1928.115	4481.083
1393983_at	314879	Xpot_predi	exportin, tRNA (nuclear export recepto	4890.863	2296.801	2156.795
1371696_at	260326	Gpr56	G protein-coupled receptor 56	4889.479	3862.411	4505.641
1392462_at	499569	NA	NA	4888.019	3977.751	7398.962
1371661_at	360594	Mrps23_pre	mitochondrial ribosomal protein S23 (p	4887.364	9562.27	3093.374
1387206_at	65196	B4galt6	UDP-Gal:betaGlcNAc beta 1,4-galacto	4886.443	5095.245	12194.03
1397362_at	257650	LOC25765	hippyragranin	4884.111	2854.07	5481.293
1385904_at	81825	Cirbp	cold inducible RNA binding protein	4876.409	2102.357	5036.47
1399114_at	306516	Gtf2e2_pre	general transcription factor II E, polype	4875.35	6194.622	4443.558
1372071_at	362851	Cd320	CD320 antigen	4874.359	2203.558	1773.921
1377192_a_at	300786	Clpx	caseinolytic peptidase X (E.coli)	4871.187	3024.828	6981.51
1391062_at	499851	RGD15660	similar to elongation protein 4 homolog	4865.742	20715.3	3952.905
1372344_at	293408	Hbs1l	Hbs1-like (S. cerevisiae)	4864.79	3859.514	3142.21
1372259_at	306817	Dek	DEK oncogene (DNA binding)	4861.316	16508.06	17465.51
1370164_at	170670	Hadha	hydroxyacyl-Coenzyme A dehydrogena	4856.235	9467.773	2762.177
1373682_at	304570	Ddx51_pre	DEAD (Asp-Glu-Ala-Asp) box polypept	4855.177	3329.844	2399.134
1393096_at	292474	RGD15606	similar to ribosomal protein L27a (pred	4850.546	2966.624	9221.9
1387969_at	245920	Cxcl10	chemokine (C-X-C motif) ligand 10	4847.992	52.92582	22657.45
1371729_at	298792	Ypel5	yippee-like 5 (Drosophila)	4847.903	6630.922	12752.12

1368481_at	25024	Gipr	gastric inhibitory polypeptide receptor	4846.89	4644.051	12517.65
1368340_at	171458	Ipmk	inositol polyphosphate multikinase	4844.63	3341.555	3451.83
1374554_at	298557	RGD1308C	similar to HT014 (predicted)	4842.876	3319.241	4842.545
1371439_at	313052	RGD15647	similar to erythrocyte membrane protei	4842.675	1735.929	2283.045
1390027_at	296121	Usp8_pred	ubiquitin specific protease 8 (predicted	4842.04	4086.796	1845.616
1372702_at	287750	PRP-2	proline-rich protein	4839.829	624.7511	1502.038
1372500_at	300838	Tmod3	tropomodulin 3	4838.336	2927.567	6075.429
1390058_at	361990	LOC36199	similar to DKFZP547E1010 protein	4830.773	10579.8	6585.906
1368368_a_at	64355	Lsr	lipolysis stimulated lipoprotein receptor	4830.313	4880.386	1781.165
1367662_at	63864	Hadh2	hydroxyacyl-Coenzyme A dehydrogena	4828.733	8362.102	2291.78
1372433_at	305887	RGD13102	similar to CG11030-PA (predicted)	4827.582	3728.67	5495.706
1388636_at	360554	Rnf167	ring finger protein 167	4827.406	7784.27	2793.236
1367526_at	362216	Mrps26_pr	mitochondrial ribosomal protein S26 (p	4826.292	10772.15	5181.029
1369649_at	25399	Cacna2d1	calcium channel, voltage-dependent, a	4824.567	4186.536	1813.158
1388567_at	309041	Thumpd1	THUMP domain containing 1	4823.29	7172.358	8151.504
1379271_at	500616	RGD15649	similar to Suppressor of cytokine signa	4817.6	3201.853	10711.4
1378060_at	315546	Kirrel3_pre	kin of IRRE like 3 (Drosophila) (predict	4814.567	278.4332	104.2918
1392900_at	310756	LOC31075	NA	4812.169	9620.162	5406.107
1379810_at	114632	Scye1	small inducible cytokine subfamily E, m	4811.963	4070.771	1849.235
1388466_at	287670	Psmd3	proteasome (prosome, macropain) 26S	4810.998	5066.502	915.9738
1375220_at	367454	LOC36745	NA	4808.443	10913	3503.363
1393065_at	499935	RGD15625	similar to KIAA0406-like protein (predic	4804.86	4147.11	2317.836
1388526_at	292915	Sult2b1_pr	sulfotransferase family, cytosolic, 2B, r	4804.846	6510.696	6020.725
1367840_at	56084	Hgs	HGF-regulated tyrosine kinase substra	4804.629	3826.244	2402.328
1388788_at	364975	Gcdh_pred	glutaryl-Coenzyme A dehydrogenase (	4792.266	2442.986	4193.066
1368217_at	84014	Ralbp1	ralA binding protein 1	4788.136	2318.852	6273.46
1371385_at	288236	Dscr2_prec	Down syndrome critical region homolo	4788.12	12460.09	4159.261
1379693_at	84409	Robo2	roundabout homolog 2 (Drosophila)	4787.756	11563.24	14705.56
1369995_at	140657	Faf1	Fas-associated factor 1	4787.289	4368.408	2702.086
1370517_at	266777	Nptx1	neuronal pentraxin 1	4787.155	835.468	7378.21
1388428_at	362227	Hars2_prec	histidyl tRNA synthetase 2 (predicted)	4786.886	17417.13	8264.781
1372797_at	302557	Pqbp1	polyglutamine binding protein 1	4786.527	7560.058	3402.039
1387823_at	60376	Plrg1	pleiotropic regulator 1, PRL1 homolog	4773.025	7408.46	8041.945
1369516_at	29535	Pdx1	pancreatic and duodenal homeobox ge	4772.939	7092.656	668.8791
1377208_at	290494	Mbnl2_prec	muscleblind-like 2 (predicted)	4771.585	417.35	9200.486
1373383_at	299514	Mterfd1	MTERF domain containing 1	4770.746	10648.36	11107.26
1372166_at	361711	Dpf2_predi	D4, zinc and double PHD fingers famil	4765.294	6809.165	3686.914
1374660_at	293528	RGD1309C	similar to hypothetical protein FLJ2000	4762.825	16067.66	9126.183
1388713_at	305889	Thtpa	thiamine triphosphatase	4757.256	2749.434	5241.86
1371560_at	292892	Irf3	interferon regulatory factor 3	4754.014	1628.893	4657.153
1371983_at	315134	Josd1	Josephin domain containing 1	4753.838	4988.961	4523.445
1372313_at	362003	Gpr89_pre	G protein-coupled receptor 89 (predict	4751.334	3634.29	873.3425
1370192_at	65033	Stx12	syntaxin 12	4750.137	3917.122	1797.894
1379730_at	297797	Atp6v1h	vacuolar ATPase subunit H	4749.988	7177.058	4936.487
1388506_at	306871	Dsp	desmoplakin	4747.478	156.6989	3085.096
1372035_at	362265	Ift52_predi	intraflagellar transport 52 homolog (Ch	4746.623	6167.715	3597.181
1371772_at	362169	Alkbh3	alkB, alkylation repair homolog 3 (E. co	4744.14	5511.907	3137.889
1377644_at	291925	RGD13087	similar to RIKEN cDNA 4921524J17 (p	4741.775	1276.311	8640.296
1372156_at	303330	Tmem97	transmembrane protein 97	4740.074	7710.754	700.1776
1367801_at	94204	Ece1	endothelin converting enzyme 1	4734.476	3060.693	5612.343
1373412_at	312373	Nt5c3_prec	5'-nucleotidase, cytosolic III (predicted	4732.128	4821.618	3285.062

1374497_at	314648	Ncln	nicalin homolog (zebrafish)	4731.59	3726.444	1500.436
1373916_at	170915	Ep300	E1A binding protein p300	4731.171	1848.514	13438.77
1388127_at	266761	Loc266761	cytochrome P450-like protein	4731.142	956.2065	8391.953
1383674_at	308986	RGD15605	RGD1560566 (predicted)	4723.485	3523.178	2813.734
1376985_at	317418	NA	NA	4716.592	3547.2	3400.015
1377761_at	360518	Gfpt2	glutamine-fructose-6-phosphate transa	4713.109	371.4813	26150.49
1382189_at	25615	Sdc2	syndecan 2	4711.794	3952.938	17381.86
1386247_at	306471	Snx25	sorting nexin 25	4709.745	6316.98	6809.446
1387950_at	192180	Nip7	nuclear import 7 homolog (S. cerevisia	4709.556	2852.061	3891.321
1388368_at	305391	Med28_pre	mediator of RNA polymerase II transcr	4709.418	5047.129	7359.229
1385592_at	317346	Bcor_predi	Bcl6 interacting corepressor (predicted	4709.305	2311.431	7677.254
1368146_at	114856	Dusp1	dual specificity phosphatase 1	4703.683	8842.955	47778.64
1389684_at	314171	Prpf39_pre	PRP39 pre-mRNA processing factor 3	4702.932	2926.146	11992.03
1373794_at	308457	Egln2	EGL nine homolog 2 (C. elegans)	4698.354	2522.798	2267.869
1369720_at	117057	Myo1b	myosin Ib	4696.513	5685.402	1805.503
1367546_at	309440	Mrpl43_pre	mitochondrial ribosomal protein L43 (p	4696.183	8404.691	6052.804
1372512_at	360953	Stx18	syntaxin 18	4693.731	1804.324	1095.234
1371682_at	362245	Map1lc3a	microtubule-associated protein 1 light	4686.684	2114.578	3589.005
1389327_at	291206	Mrpl32_pre	mitochondrial ribosomal protein L32 (p	4684.265	7020.98	4613.499
1375433_at	311324	Disp2_pred	dispatched homolog 2 (Drosophila) (pr	4682.904	6992.232	10356.74
1370317_at	245960	LOC24596	potassium channel regulator 1	4681.955	7531.267	8562.753
1395875_at	294595	RGD1306C	similar to KIAA0372 gene product (pre	4670.261	1898.744	2544.544
1375848_at	296851	Pon2	paraoxonase 2	4662.087	1319.617	4786.835
1382598_at	64441	Hsf2	heat shock factor 2	4661.152	5775.511	6345.715
1375297_at	363474	RGD15652	similar to RIKEN cDNA 0610008C08 (	4659.667	7848.492	3904.047
1373466_at	25403	Cast	calpastatin	4656.599	3698.087	2651.114
1388766_at	288150	Mtx2	metaxin 2	4653.044	4890.467	9887.254
1379668_at	361174	RGD15647	similar to hypothetical protein B230397	4649.494	6428.592	1703.26
1377820_a_at	293526	Ate1_predi	arginine-tRNA-protein transferase 1 (p	4648.452	4762.144	8078.746
1398949_at	296410	RGD1311C	similar to 2410001C21Rik protein	4648.037	5801.634	3175.367
1376546_at	499157	RGD15654	similar to hypothetical protein (predicte	4646.793	5407.03	7121.33
1373705_at	497876	LOC49787	NA	4645.864	5265.47	2137.469
1374767_at	290963	RGD13095	similar to hypothetical protein FLJ1467	4643.451	4075.906	1669.147
1399002_at	288621	Mrps17_pr	mitochondrial ribosomal protein S17 (p	4639.713	11613.83	4435.133
1383965_at	288040	Ncbp2_pre	nuclear cap binding protein subunit 2 (	4639.41	4681.05	13780.83
1388410_at	289827	Ugp2	UDP-glucose pyrophosphorylase 2	4639.368	10239.92	7017.345
1370463_x_at	414819	RT1-CE16	RT1 class I, CE16	4632.313	2199.452	3310.146
1373241_at	309176	Mrpl49	mitochondrial ribosomal protein L49	4628.497	8651.958	3120.711
1371133_a_at	24679	Prkar2b	protein kinase, cAMP dependent regul	4628.181	7374.053	2879.141
1372064_at	497942	Cxcl16	similar to chemokine (C-X-C motif) liga	4626.98	2485.992	3547.012
1391461_at	298002	RGD13065	similar to hypothetical protein (predicte	4625.941	3151.466	1842.123
1372990_at	298400	Creb3	cAMP responsive element binding prot	4625.84	3875.539	1539.136
1374468_at	301059	Myd88	myeloid differentiation primary respons	4625.031	1181.753	1482.429
1370880_at	245964	Rnh1	ribonuclease/angiogenin inhibitor 1	4624.993	4606.555	2853.465
1374580_at	300860	Senp6_pre	SUMO/sentrin specific peptidase 6 (pr	4619.419	2059.237	11805.14
1372189_at	363127	Dnajc13_p	DnaJ (Hsp40) homolog, subfamily C, r	4618.878	2278.228	3713.526
1372284_at	362599	Trappc3	trafficking protein particle complex 3	4617.349	8094.013	3741.306
1391282_at	309570	RGD13069	similar to dJ55C23.6 gene product (pre	4616.782	2107.892	4001.571
1372607_at	287125	Nubp2	nucleotide binding protein 2	4612.051	6618.421	1666.598
1372941_at	296278	Pdrg1	p53 and DNA damage regulated 1	4608.47	2860.871	2016.472
1377668_at	24919	Yy1	YY1 transcription factor	4604.024	6696.876	19379.09

1374273_at	89843	Cxadr	coxsackie virus and adenovirus recept	4599.261	7801.636	13774.28
1372062_at	299334	RGD15633	similar to cyclin-dependent kinase 2-in	4594.904	4050.546	1892.978
1388695_at	299857	Shmt2	serine hydroxymethyl transferase 2 (m	4588.122	2955.577	792.6501
1388977_at	310773	Cept1	choline/ethanolamine phosphotransfer	4586.154	3496.189	3414.873
1379273_at	300472	LOC30047	NA	4585.403	5817.236	1627.557
1367468_at	362252	Scand1_pr	SCAN domain-containing 1 (predicted)	4583.851	5757.529	1098.082
1379703_at	315055	RGD13115	hypothetical LOC315055 (predicted)	4582.029	1930.765	3194.061
1388892_at	305853	Rab2b	RAB2B, member RAS oncogene famil	4581.92	2793.857	3022.511
1392885_at	291439	Mbd1	methyl-CpG binding domain protein 1	4581.242	8091.451	4269.72
1372169_at	303514	RGD15647	similar to RIKEN cDNA 4121402D02 (	4579.552	6856.506	7449.87
1376722_at	362335	Nup205_pr	nucleoporin 205kDa (predicted)	4571.998	10539.75	5745.491
1394884_s_at	54304	Ssbp1	single-stranded DNA binding protein 1	4569.06	6703.597	5444.56
1370298_at	252860	Zfp99	zinc finger protein 99	4568.69	8522.937	3644.644
1373629_at	307811	Slc7a6_pre	solute carrier family 7 (cationic amino a	4567.943	2003.988	3365.32
1399163_a_at	291202	Hs1bp1	HS1 binding protein	4567.702	3310.259	3992.595
1391643_at	78969	Trib1	tribbles homolog 1 (Drosophila)	4564.216	1363.925	2077.364
1374506_at	311835	Ddx31_pre	DEAD/H (Asp-Glu-Ala-Asp/His) box po	4561.088	1927.132	1901.679
1390097_at	309828	Tspyl4	TSPY-like 4	4559.457	6454.889	1876.031
1374406_at	64561	Phka1	phosphorylase kinase alpha 1	4559.213	5059.82	9315.735
1377676_at	64709	Nucks	nuclear ubiquitous casein kinase and c	4557.193	9560.907	4385.661
1388882_at	299104	Fkbp3_pre	FK506 binding protein 3 (predicted)	4553.388	6484.835	9088.115
1377613_at	300463	RGD13073	similar to RIKEN cDNA 5031400M07	4552.894	3052.835	8563.032
1389229_at	315939	Acpl2	acid phosphatase-like 2	4552.168	2317.006	8085.732
1372408_at	293455	Gga2	golgi associated, gamma adaptin ear c	4551.13	4496.194	2579.976
1388405_at	311191	Ckap5	cytoskeleton associated protein 5	4545.721	10773.4	5503.931
1389549_at	306544	Prosc_prec	proline synthetase co-transcribed (prec	4545.548	8767.521	4398.245
1371546_at	361128	LOC36112	similar to TR4 orphan receptor associa	4545.27	3792.065	574.8113
1388996_at	361351	RGD13085	similar to RIKEN cDNA 3110052N05	4543.189	9979.085	6001.682
1391158_a_at	288663	Atxn2_prec	ataxin 2 (predicted)	4542.445	4399.839	7694.431
1374436_at	288615	Sbds	Shwachman-Bodian-Diamond syndron	4539.148	4831.158	4900.177
1387226_at	24503	Inexa	internexin, alpha	4538.906	7983.521	13505.1
1369932_a_at	24703	Raf1	v-raf-1 murine leukemia viral oncogene	4533.222	7371.771	3717.984
1371473_at	292696	Clptm1_pre	cleft lip and palate associated transme	4530.361	4665.438	2424.331
1398441_at	316369	Nck2_pred	non-catalytic region of tyrosine kinase	4529.756	2253.953	1827.213
1386885_at	64526	Ech1	enoyl coenzyme A hydratase 1, peroxi	4524.514	5785.704	2236.605
1382596_a_at	362711	RGD13113	similar to hypothetical protein FLJ2025	4524.234	2450.395	1791.07
1372614_at	313346	RGD13118	similar to mKIAA1797 protein (predicte	4523.25	1725.4	3859.605
1371725_at	25745	Myh9	myosin, heavy polypeptide 9, non-mus	4516.398	3917.479	3153.704
1367966_at	114591	Dpp3	dipeptidylpeptidase 3	4516.153	8287.624	532.364
1392719_at	300886	Mthfs	5,10-methenyltetrahydrofolate syntheta	4514.45	3498.057	7443.781
1367632_at	24957	Glul	glutamate-ammonia ligase (glutamine	4513.086	3595.172	7209.391
1371253_at	300726	Etfa	electron transferring flavoprotein, alpha	4511.185	5114.632	4604.24
1374625_at	316626	Hes6	hairy and enhancer of split 6 (Drosoph	4509.511	3974.046	3444.672
1370001_at	117103	Rab8a	RAB8A, member RAS oncogene famil	4504.966	4875.746	2337.617
1375904_at	289883	Tmed5	transmembrane emp24 protein transpo	4503.928	1937.759	4169.518
1388321_at	315697	Imp3_pred	IMP3, U3 small nucleolar ribonucleopr	4502.277	2967.144	2008.16
1372731_at	303351	Rhot1_prec	ras homolog gene family, member T1 (	4501.454	6501.303	4647.469
1373016_at	298942	Dld	dihydrolipoamide dehydrogenase	4500.085	4090.39	2620.867
1373605_at	316085	RGD13078	similar to 106 kDa O-GlcNAc transfera	4492.574	3053.61	7764.889
1399159_a_at	29528	Vamp3	vesicle-associated membrane protein 3	4489.502	8064.464	11441.28
1389144_at	498559	RGD15607	similar to OTTHUMP00000018508 (pr	4488.882	1994.68	2641.476

1374508_at	296136	Nphp1_pre	nephronophthisis 1 (juvenile) homolog	4487.456	7233.54	3059.714
1371998_at	297387	MGC12488	NA	4481.261	3839.75	3574.208
1383172_at	294429	RGD1560C	similar to Ran-binding protein 2 (predic	4477.856	3174.408	12800.38
1388392_at	360564	Tax1bp3	Tax1 (human T-cell leukemia virus typ	4472.856	11170.75	1264.971
1388747_at	361643	Lcmt1	leucine carboxyl methyltransferase 1	4472.344	7369.896	6712.57
1369157_at	29516	Pde3b	phosphodiesterase 3B	4468.892	12971.85	5910.755
1367605_at	64303	Pfn1	profilin 1	4465.119	8103.432	2958.464
1387279_at	116479	F11r	F11 receptor	4464.112	6994.891	5234.422
1375307_at	315136	Cbx6	chromobox homolog 6	4461.467	4700.096	2011.946
1389074_at	685041	LOC68504	NA	4457.975	1756.999	5059.676
1387979_at	192243	Golgb1	golgi autoantigen, golgin subfamily b, r	4453.817	1909.842	1163.795
1370366_at	64464	Timm10	translocase of inner mitochondrial mer	4453.253	5313.532	2561.054
1373936_at	361212	Rnf44	ring finger protein 44	4447.182	6955.123	8509.461
1373757_at	114635	Fln29	FLN29 gene product	4445.446	3434.212	3799.683
1372507_at	306587	Tcta	T-cell leukemia translocation altered ge	4443.057	204.5101	2277.222
1387280_a_at	50719	Slc7a5	solute carrier family 7 (cationic amino a	4437.478	1110.78	1752.038
1372894_at	363136	Tmem115_	transmembrane protein 115 (predicted	4436.297	1516.313	1227.031
1398339_at	117855	Zfp162	zinc finger protein 162	4435.994	3411.345	4280.278
1388990_at	246042	Mki67ip	spermatogenesis-related protein (Srp)	4429.979	4210.42	4161.727
1388336_at	362164	RGD13106	similar to hypothetical protein D2Ertd3	4422.263	3299.764	1590.056
1372782_a_at	362015	LOC36201	NA	4420.856	5926.106	5435.328
1370060_at	64201	Slc25a11	solute carrier family 25 (mitochondrial	4416.256	8753.864	2384.472
1389728_at	311311	Meis2_pre	Meis1, myeloid ecotropic viral integrati	4415.931	14.95187	11271.25
1383768_at	286973	Elavl2	ELAV (embryonic lethal, abnormal visi	4414.567	1272.07	634.5858
1390383_at	298199	Adfp	adipose differentiation related protein	4411.516	790.6474	1827.305
1373209_at	289315	RGD13055	similar to hypothetical protein MGC306	4410.775	2425.3	4739.885
1378551_at	316435	Cyp20a1	cytochrome P450, family 20, subfamily	4409.307	3710.021	6383.814
1371959_at	365877	Hist2h2aa_	histone 2, H2aa (predicted)	4409.109	706.8998	1895.008
1390063_at	287299	Mfap3	microfibrillar-associated protein 3	4408.946	4579.962	971.8916
1370160_at	170751	Xpnpep1	X-prolyl aminopeptidase (aminopeptida	4408.777	8297.48	2279.669
1371325_at	296370	Ppgb	protective protein for beta-galactosidas	4407.633	5214.239	3457.363
1372136_at	306324	Tspan14_p	tetraspanin 14 (predicted)	4406.53	6059.579	1579.763
1374834_at	295270	Sf3b4	splicing factor 3b, subunit 4	4404.698	6588.418	4384.4
1372757_at	25124	Stat1	signal transducer and activator of trans	4402.081	4468.859	4840.533
1382584_at	500386	RGD15644	similar to mKIAA1321 protein (predicte	4400.452	1700.495	2742.201
1388420_at	361100	LOC36110	NA	4399.741	6147.149	15092.07
1388355_at	291295	Rbm17	RNA binding motif protein 17	4396.851	9524.573	9137.213
1392902_at	366203	RGD1306C	similar to chromosome 20 open readin	4396.366	3657.496	2108.673
1374692_at	315871	Snx14_pre	sorting nexin 14 (predicted)	4389.879	1334.08	11141.62
1367498_at	296467	Ythdf1	YTH domain family 1	4388.043	4551.643	9514.722
1372019_at	290939	RGD13101	similar to DNA segment, Chr 13, Wayr	4385.272	9014.79	1963.077
1398984_at	290833	Tm2d2	TM2 domain containing 2	4384.954	5795.851	1530.387
1373821_at	294698	Slc30a5_pr	solute carrier family 30 (zinc transporte	4381.485	2564.095	2553.575
1389384_at	362495	Hrpap20	hormone-regulated proliferation associ	4381.234	4511.059	4263.737
1389680_at	309918	Ell2	elongation factor RNA polymerase II 2	4377.849	1714.643	4432.674
1383689_at	303923	B4galt4	UDP-Gal:betaGlcNAc beta 1,4-galacto	4372.944	684.1704	1211.137
1377123_at	311839	Slc27a4	solute carrier family 27 (fatty acid trans	4372.553	2388.189	1204.942
1391170_at	297514	RGD13104	similar to mKIAA1757 protein (predicte	4370.499	1446.817	1789.67
1375961_at	295691	Frzb	frizzled-related protein	4366.329	103.1689	3711.207
1399094_at	299154	Churc1_pre	churchill domain containing 1 (predicte	4366.182	4833.49	10205.57
1369788_s_at	24516	Jun	Jun oncogene	4365.372	700.6522	46593.68

1398974_at	362273	Cse1l_pred	chromosome segregation 1-like (S. cer	4362.145	7727.207	3401.318
1376656_at	311856	Usp20_pre	ubiquitin specific protease 20 (predicte	4360.669	2556.828	2128.253
1372260_at	287061	MGC11614	leucine zipper domain protein	4359.255	2833.733	1524.543
1387904_at	50560	Ihpk1	inositol hexaphosphate kinase 1	4355.468	1912.112	3948.543
1399024_at	293684	Scyl1	SCY1-like 1 (S. cerevisiae)	4355.017	6172.339	2433.692
1371865_at	312640	Tmem111	transmembrane protein 111	4354.672	4476.584	2121.182
1372232_at	297381	Wbp1	WW domain binding protein 1	4354.478	6346.432	4360.629
1372553_at	362483	MGC94199	similar to RIKEN cDNA 2610301B20; E	4348.028	10037.59	6683.634
1388699_at	361378	Man2b1	mannosidase 2, alpha B1	4346.777	3991.461	2576.283
1369358_a_at	29430	Hap1	huntingtin-associated protein 1	4344.325	3758.41	8642.577
1372581_at	287645	Snf8	SNF8, ESCRT-II complex subunit, hom	4343.624	6466.123	1526.957
1371620_at	290995	RGD1308C	similar to px19-like protein	4339.917	8925.349	1341.144
1398601_at	307348	Spire1_pre	spire homolog 1 (Drosophila) (predicte	4339.85	2861.404	5516.076
1388596_at	361422	Cotl1_pred	coactosin-like 1 (Dictyostelium) (predic	4339.147	662.7223	2398.431
1367737_at	24375	Fuca	fucosidase, alpha-L- 1, tissue	4337.978	15879	5883.078
1389194_at	311323	RGD15655	similar to hypothetical protein (predicte	4337.202	6229.084	3324.072
1388643_at	432392	Fut8	fucosyltransferase 8 (alpha (1,6) fucos	4335.363	2423.805	1406.847
1386888_at	116636	Eif4ebp1	eukaryotic translation initiation factor 4	4332.948	195.9197	707.9135
1390858_at	286761	Vcpip1	valosin containing protein (p97)/p47 co	4332.55	2724.718	1029.013
1371706_at	306322	Sdccag3	serologically defined colon cancer anti	4331.312	4272.38	3867.685
1389717_at	293570	RGD13089	similar to KIAA0157 gene product is no	4330.844	3192.643	3713.034
1370807_at	192129	Tmem49	transmembrane protein 49	4328.764	4055.662	1501.758
1372000_at	307098	Net1	neuroepithelial cell transforming gene	4326.113	6089.176	2641.993
1389043_at	308453	Adck4	aarF domain containing kinase 4	4323.715	981.2533	1321.551
1388959_at	300105	Ttll12_pred	tubulin tyrosine ligase-like family, mem	4323.372	2221.118	1881.475
1391484_at	287659	Socs7_pre	suppressor of cytokine signaling 7 (pre	4323.323	2427.035	4103.042
1389990_at	498080	RGD15637	similar to Gene model 609 (predicted)	4321.678	9215.059	1822.367
1393248_at	310512	Nmd3_pred	NMD3 homolog (S. cerevisiae) (predic	4320.618	4848.907	7492.749
1374695_at	360609	Cbx1_pred	chromobox homolog 1 (Drosophila HP	4319.963	4593.512	4881.588
1371444_at	310667	Lass2	longevity assurance homolog 2 (S. cer	4319.858	5086.394	1571.487
1372184_at	315463	RGD13091	similar to hypothetical protein BC01183	4318.105	3653.733	4402.694
1388979_at	287768	Smndc1	survival motor neuron domain containi	4316.432	7827.622	6673.234
1368356_a_at	80897	Arts1	type 1 tumor necrosis factor receptor s	4314.577	11466.88	4012.844
1398249_at	117035	Slc25a20	solute carrier family 25 (mitochondrial c	4310.399	2312.296	1445.304
1389621_at	289582	Fip1l1	FIP1 like 1 (S. cerevisiae)	4307.989	3037.131	4703.429
1371961_at	361527	Pld3	phospholipase D family, member 3	4307.007	2593.446	2910.794
1368199_at	113929	Nup88	nucleoporin 88	4306.839	9314.483	7160.023
1390485_at	294079	March5_pr	membrane-associated ring finger (C3H	4305.651	6832.913	4605.539
1367495_at	364186	RGD15602	similar to prefoldin 4 (predicted)	4302.675	6605.731	6026.764
1370218_at	24534	Ldhb	lactate dehydrogenase B	4294.354	2265.297	13266.71
1373576_at	287249	RGD13107	similar to CCR4	4293.553	11558.06	11014.41
1388454_at	501454	RGD15629	similar to product is unknown~seizure-	4293.109	3144.663	3453.289
1388976_at	297388	RGD13059	similar to BolA domain-containing prote	4288.213	5127.868	2565.334
1393845_a_at	308310	Tmc4	transmembrane channel-like gene fam	4287.84	6147.349	3853.879
1382847_at	310638	Ash1l_pred	ash1 (absent, small, or homeotic)-like	4283.765	1221.151	1025.52
1373595_at	362401	Tmem43	transmembrane protein 43	4281.602	3283.381	3178.827
1398886_at	502642	RGD15633	similar to Selenoprotein H (predicted)	4281.129	10912.89	4376.934
1383652_at	81528	Ogg1	8-oxoguanine DNA-glycosylase 1	4278.396	4833.846	1501.186
1388534_at	366381	Cdc26	cell division cycle 26	4274.741	4365.002	1613.887
1376249_at	292485	Fuca2	fucosidase, alpha-L- 2, plasma	4273.711	5173.761	2503.064
1388565_at	300791	Spg21	spastic paraplegia 21 homolog (human	4270.136	1977.299	1836.297

1372432_at	361995	Prpf3_pred	PRP3 pre-mRNA processing factor 3 h	4268.712	2653.249	2205.058
1371982_at	286897	Aip1	AIP1	4267.361	15301.23	6185.342
1369975_at	65241	Secp43	tRNA selenocysteine associated prote	4262.758	8570.662	3675.161
1372269_at	299180	Med6_pred	mediator of RNA polymerase II transcr	4262.34	3941.943	3022.731
1371787_at	25562	Tceb3	transcription elongation factor B (SIII),	4260.268	857.507	6126.607
1390821_at	116741	Pcdha4	protocadherin alpha 4	4257.709	8351.462	4388.593
1398813_at	117553	Ube1c	ubiquitin-activating enzyme E1C	4257.497	8895.82	7134.494
1371639_at	288666	Vps29_pre	vacuolar protein sorting 29 (S. pombe)	4256.967	9705.618	8384.04
1370538_at	286966	Lama3	laminin, alpha 3	4256.02	107.5793	572.3432
1371830_at	308384	Uble1a	ubiquitin-like 1 (sentrin) activating enzy	4250.938	8908.775	2871.097
1371036_at	497815	Nrcam	neuron-glia-CAM-related cell adhesion	4247.23	1046.129	2916.693
1398508_at	314329	RGD13109	similar to polyglutamine-containing pro	4246.163	3334.702	5977.662
1372196_at	360801	Ncor2_pred	nuclear receptor co-repressor 2 (predic	4244.587	3953.076	3855.311
1387856_at	54321	Cnn3	calponin 3, acidic	4242.684	4088.603	5156.464
1371925_at	290673	Atp13a1_p	ATPase type 13A1 (predicted)	4241.428	4132.573	1116.732
1372393_at	291293	Fbxo18_pr	F-box only protein 18 (predicted)	4239.469	7800.286	3597.149
1372327_at	362207	Myef2	myelin basic protein expression factor	4239.331	14837.87	21190.59
1390010_at	313929	Ncoa1_pre	nuclear receptor coactivator 1 (predicte	4237.535	5318.553	6558.748
1373146_at	308023	Ssx2ip	synovial sarcoma, X breakpoint 2 inter	4237.322	1810.997	2438.707
1374938_at	363059	Zw10	ZW10 homolog, centromere/kinetocho	4236.021	1947.303	1037.984
1388122_at	29438	Gstp2	glutathione S-transferase, pi 2	4234.751	29.05964	2411.481
1371720_at	298684	RGD15623	similar to Mrpl20 protein (predicted)	4232.409	5854.991	3096.98
1368220_at	81673	Gtf2b	general transcription factor IIB	4226.34	5285.22	6028.544
1388780_at	307861	Terf2ip	telomeric repeat binding factor 2, intera	4225.731	3491.548	8082.429
1373100_at	499779	LOC49977	similar to RIKEN cDNA 2900010J23	4224.742	7173.498	4138.082
1371695_at	304862	Tpr	translocated promoter region	4224.061	3829.33	9102.35
1371885_at	292777	Ckap1_pre	cytoskeleton-associated protein 1 (pre	4222.833	13138.63	3479.863
1382464_at	363074	RGD13097	similar to ENSANGP00000021391 (pre	4221.564	4065.699	6411.478
1372310_at	364879	Isoc1	isochorismatase domain containing 1	4221.535	5720.279	3146.654
1391229_at	171358	Camk1g	calcium/calmodulin-dependent protein	4218.314	691.7439	1747.397
1374656_at	252881	Exoc3	exocyst complex component 3	4213.305	5785.497	4342.721
1384935_at	362178	Cstf3_pred	cleavage stimulation factor, 3' pre-RNA	4212.4	5494.962	3159.55
1398473_at	293938	Bloc1s2	biogenesis of lysosome-related organe	4211.332	11298.81	1558.735
1375854_at	503000	LOC50300	similar to beta-catenin-interacting prote	4209.124	2319.791	1640.29
1373907_at	367073	Trappc4	trafficking protein particle complex 4	4207.963	2996.905	1312.32
1373865_at	65178	Snap91	synaptosomal-associated protein, 91kD	4207.457	8191.87	8551.711
1371533_at	290798	Dctn6_pred	dynactin 6 (predicted)	4204.848	9399.561	5293.339
1398891_at	297799	Mrpl15_pre	mitochondrial ribosomal protein L15 (p	4204.846	6319.055	2696.316
1387113_at	81717	Ctbp2	C-terminal binding protein 2	4204.571	3342.468	3533.074
1372697_at	298517	Mrps15	mitochondrial ribosomal protein S15	4204.375	8972.63	2403.981
1373248_at	304529	RGD15658	similar to hypothetical protein FLJ2067	4203.669	3982.486	8126.766
1369942_at	63836	Actn4	actinin alpha 4	4203.303	2873.89	2281.812
1385088_at	304650	RGD13102	hypothetical LOC304650 (predicted)	4202.594	1911.445	610.8211
1388745_at	310630	Sema4a	sema domain, immunoglobulin domain	4200.885	2026.827	1366.907
1371461_at	298549	RGD13054	similar to RIKEN cDNA 2410166I05	4199.857	2735.37	4457.569
1371565_at	298370	Txndc12	thioredoxin domain containing 12 (end	4199.786	3100.845	6927.743
1371358_at	191576	Gpsn2	glycoprotein, synaptic 2	4195.304	12524.75	5412.638
1372882_at	296587	RGD13086	similar to CG12379-PA (predicted)	4192.473	5336.848	924.681
1367506_at	293666	mrpl11	mitochondrial ribosomal protein L11	4190.266	8233.23	909.5185
1371904_at	289372	Smyd2	SET and MYND domain containing 2	4186.424	2494.65	3946.074
1392906_at	293864	Ubl4a_pred	ubiquitin-like 4a (predicted)	4184.519	5684.463	1172.545

1369971_a_at	79256	Hnrpd	heterogeneous nuclear ribonucleoprot	4183.965	9307.461	7367.989
1383262_at	299800	Tmem19	transmembrane protein 19	4183.242	8994.942	2787.696
1384008_at	361275	RGD13058	similar to hypothetical protein FLJ4028	4180.546	5322.164	13576.62
1371770_at	309629	Ke2	MHC class II region expressed gene K	4180.232	8797.755	2690.084
1374579_at	306238	RGD15608	similar to RIKEN cDNA E230015L20 g	4179.188	2902.162	5115.373
1370195_at	64630	Snap23	synaptosomal-associated protein 23	4176.448	2247.147	4436.296
1389970_at	297728	RGD13106	similar to RIKEN cDNA 1200009B18; I	4174.731	2722.107	2506.201
1367763_at	25014	Acat1	acetyl-coenzyme A acetyltransferase 1	4174.695	2202.852	2813.294
1388860_at	287302	Mrpl22_pre	mitochondrial ribosomal protein L22 (p	4174.344	12736.17	4413.419
1374618_at	305152	Aff1_predic	AF4/FMR2 family, member 1 (predicte	4172.149	2539.482	7001.926
1374070_at	29326	Gpx2	glutathione peroxidase 2	4169.99	43.12113	653.4777
1382813_at	306549	RGD13108	similar to RIKEN cDNA 4930444A02	4169.279	2173.841	1141.702
1367744_at	113947	Maged2	melanoma antigen, family D, 2	4164.273	12815.25	9921.415
1388500_at	54260	Itpkb	inositol 1,4,5-trisphosphate 3-kinase B	4163.787	1513.783	395.521
1387012_at	29678	Sart1	squamous cell carcinoma antigen reco	4160.141	4193.93	3430.048
1371660_at	360784	Znhit1_pre	zinc finger, HIT domain containing 1 (p	4157.736	11597.71	1743.501
1372909_at	307302	RGD15656	similar to hypothetical protein FLJ3609	4157.245	3068.648	3766.982
1380824_at	306548	Hook3	hook homolog 3 (Drosophila)	4156.654	1029.424	1592.089
1398244_at	170933	Syf2	SYF2 homolog, RNA splicing factor (S	4154.876	3731.795	5692.1
1372066_at	293058	RGD13100	similar to RIKEN cDNA 2610204K14	4154.494	5702.404	4143.023
1375666_at	114485	Dmtf1	cyclin D binding myb-like transcription	4152.615	2353.654	5372.302
1371379_at	289182	RGD15634	similar to Brain protein 44 (predicted)	4151.442	4717.404	9056.256
1394654_at	316312	Zfp451	zinc finger protein 451	4148.229	3452.336	2203.003
1370875_at	54319	Vil2	villin 2	4143.56	4967.272	3590.078
1376587_at	301674	Fbxo11	F-box only protein 11	4142.907	5282.33	15525.04
1385727_at	362463	Fgfr1op2	FGFR1 oncogene partner 2	4140.82	2073.436	1580.669
1371782_at	313211	Nipsnap3a	nipsnap homolog 3A (C. elegans)	4140.742	12289.81	1722.755
1372448_at	362156	LOC36215	NA	4135.735	6720.392	2288.826
1367464_at	84401	Siahbp1	siah binding protein 1; FBP interacting	4133.733	6544.793	5160.75
1372369_at	361732	Tmem109	transmembrane protein 109	4132.278	7308.25	3929.991
1372688_at	316098	Exosc7	exosome component 7	4128.556	3442.574	1792.352
1371979_at	300095	Srebf2_pre	sterol regulatory element binding facto	4125.259	5767.848	3178.107
1377815_at	361020	Rpp14_pre	ribonuclease P 14 subunit homolog (hu	4121.382	2304.627	3821.1
1372548_at	170917	Cry2	cryptochrome 2 (photolyase-like)	4120.583	3148.405	6615.979
1367473_at	300075	Tomm22	translocase of outer mitochondrial mer	4118.771	12406.05	5433.835
1374135_at	290228	Ipo4_predi	importin 4 (predicted)	4117.298	2580.801	1977.407
1371848_at	24177	Afp	alpha-fetoprotein	4112.852	16166.55	5954.279
1389344_at	297336	Usp39_pre	ubiquitin specific protease 39 (predicte	4111.679	5460.831	1520.089
1376779_at	84482	Foxo1a	forkhead box O1A	4110.006	1689.741	1305.778
1377594_at	85385	Shc1	src homology 2 domain-containing trar	4109.332	1706.865	1374.424
1399014_at	246772	Vps4a	vacuolar protein sorting 4a (yeast)	4109.191	2727.526	1862.162
1389125_at	289491	Mrpl1_prec	mitochondrial ribosomal protein L1 (pre	4108.392	7957.162	4615.294
1382489_at	315843	RGD15649	similar to WD repeat domain 11 protein	4107.96	1114.205	3023.911
1396803_at	313308	Thoc2_pre	THO complex 2 (predicted)	4104.624	2815.812	1907.889
1389655_at	287593	RGD13068	similar to A230072I16Rik protein	4102.792	5494.9	629.5989
1378002_at	294993	Hspa4l_pre	heat shock 70kDa protein 4-like (predi	4092.958	2017.141	4907.135
1375699_at	619382	Centb2	centaurin, beta 2	4091.439	3305.765	851.4863
1382855_at	311715	RGD13058	similar to Protein C20orf158 (predicted	4087.105	156.9987	155.4188
1367746_a_at	83764	Flot2	flotillin 2	4086.942	2179.311	1044.153
1373400_at	29699	Prkar2a	protein kinase, cAMP-dependent, regu	4086.068	3902.381	4315.204
1377987_at	363858	RGD15639	similar to trafficking protein particle cor	4084.835	6516.004	1500.038

1378017_at	307081	Pitrm1_pre	pitrilysin metallepetidase 1 (predicted)	4083.467	4301.671	3087.069
1373848_at	289083	RGD13085	similar to RIKEN cDNA 5730449L18 (p	4081.541	3028.988	2401.554
1367481_at	300052	Vps28_pre	vacuolar protein sorting 28 (yeast) (pre	4078.345	4092.622	3257.726
1379282_at	301035	Lrrfip2	leucine rich repeat (in FLII) interacting	4072.152	1619.354	1841.388
1376073_at	314352	Sel1h	Sel1 (suppressor of lin-12) 1 homolog	4071.547	1486.879	494.564
1374505_at	502922	NA	NA	4066.425	1859.894	2372.721
1388440_at	300802	Aph1b	anterior pharynx defective 1b homolog	4064.988	2719.287	1464.574
1370920_at	296753	Srpk2_pred	serine/arginine-rich protein specific kin	4064.842	4638.774	8712.271
1388141_at	170895	Cetn3	centrin 3	4063.193	7029.117	5216.168
1372811_at	498487	NA	NA	4060.115	3732.859	5002.509
1371609_at	294326	RGD1303C	homolog of zebrafish ES1	4058.246	2411.957	2219.566
1387169_at	84424	Tle3	transducin-like enhancer of split 3, E(s	4057.23	1084.259	7072.837
1391944_at	361853	RGD13112	similar to Hypothetical protein C6orf60	4054.947	1904.199	7997.125
1398875_at	366277	Polr3k	NA	4054.78	4314.463	4734.948
1373547_at	307008	RGD13081	similar to expressed sequence AW209	4053.911	4821.077	2476.754
1373061_at	298836	Snx17	sorting nexin 17	4047.962	5513.74	2038.131
1375522_at	259274	Nmt1	N-myristoyltransferase 1	4043.912	5778.948	2515.409
1373228_at	361165	RGD15607	similar to D8Ertd354e protein (predicte	4038.557	4597.774	6625.858
1398918_at	361816	Zfand3	zinc finger, AN1-type domain 3	4038.499	3920.546	2226.483
1367969_at	94167	Prdx6	peroxiredoxin 6	4037.662	1170.913	825.0953
1398360_at	679532	LOC67953	NA	4035.76	5633.514	1813.184
1388779_at	246279	Zfp180	zinc finger protein 180	4025.594	5423.636	4191.512
1399045_at	79214	Galnt1	UDP-N-acetyl-alpha-D-galactosamine:	4022.344	9288.455	4302.541
1388937_at	362900	Rnf19_pred	ring finger protein (C3HC4 type) 19 (pr	4021.943	8653.582	4904.403
1372550_at	24917	Kpnb1	karyopherin (importin) beta 1	4020.946	6388.939	2007.02
1368314_at	81716	Ggcx	gamma-glutamyl carboxylase	4019.432	1734.736	1469.685
1397217_at	29428	Cugbp2	CUG triplet repeat, RNA binding protei	4017.022	703.6133	6053.886
1383091_at	303396	Appbp2	amyloid beta precursor protein (cytopla	4016.85	2731.795	1376.255
1371800_at	313038	Phc2	polyhomeotic-like 2 (Drosophila)	4016.693	4325.433	2798.159
1389468_at	362383	Rpia_predi	ribose 5-phosphate isomerase A (pred	4014.688	5242.357	4063.482
1370348_at	25338	Ninj1	ninjurin 1	4013.711	4440.009	2899.285
1367513_at	296279	Tm9sf4	transmembrane 9 superfamily protein r	4003.912	4026.04	2778.783
1369904_at	25450	Gabrb1	gamma-aminobutyric acid (GABA-A) re	4000.566	121.6414	4905.03
1389844_at	260321	Fkbp4	FK506 binding protein 4	4000.354	4322.837	1032.205
1373116_at	361055	Kpna3	karyopherin (importin) alpha 3	3997.95	3583.423	6383.086
1388105_at	116656	D123	D123 gene product	3997.839	10085.55	1676.312
1389010_at	299732	Lta4h	leukotriene A4 hydrolase	3997.447	8625.328	4624.801
1392559_at	363009	LOC36300	NA	3990.892	1510.213	2183.966
1380453_at	313524	Zswim5_pr	zinc finger, SWIM domain containing 5	3989.501	1807.415	1528.434
1373242_at	292173	Tbpl1_pred	TATA box binding protein-like 1 (predic	3988.134	6052.583	10244.83
1372149_at	361215	Auh_predic	AU RNA binding protein/enoyl-coenzyr	3985.116	5389.793	7137.998
1371709_at	300974	Mrpl3_pred	mitochondrial ribosomal protein L3 (pre	3984.339	5804.815	2922.042
1389600_at	301748	LOC30174	similar to RIKEN cDNA 1700001E04	3982.807	10665.42	6150.39
1372703_at	299677	Ube2r2_pr	ubiquitin-conjugating enzyme E2R 2 (p	3978.886	2201.1	2962.082
1398977_at	25700	Ide	insulin degrading enzyme	3978.859	10043.68	3210.279
1371622_at	497954	RGD15646	similar to candidate tumor suppressor	3975.508	5938.492	1445.377
1370878_at	29421	Urod	uroporphyrinogen decarboxylase	3974.448	5538.337	2394.542
1367482_at	498030	RGD15618	similar to anaphase promoting comple	3973.472	8087.133	1035.215
1390474_at	362899	RGD15643	similar to RIKEN cDNA 9330161F08 (p	3968.643	3816.616	1553.376
1376666_at	307200	Socs6_pre	suppressor of cytokine signaling 6 (pre	3968.462	6944.346	5433.984
1369986_at	24439	Hagh	hydroxyacyl glutathione hydrolase	3968.333	6773.295	1711.102

1398343_at	300721	Dnaja4	DnaJ (Hsp40) homolog, subfamily A, n	3964.614	3668.713	10732.76
1383206_at	361073	Cog3	component of oligomeric golgi complex	3964.522	1393.232	4658.502
1371020_at	266780	Rimbp2	RIM binding protein 2	3962.527	1698.912	1827.821
1372555_at	360488	RGD13093	similar to RIKEN cDNA E030034P13 (	3961.834	3343.217	2030.155
1386424_at	297066	Zfp212	Zinc finger protein 212	3959.739	4880.012	4911.436
1377926_at	316611	Centg2_pre	centaurin, gamma 2 (predicted)	3959.408	1081.032	686.5446
1373849_at	361925	Actl6a	actin-like 6A	3954.977	8439.297	4879.146
1388587_at	294235	Ier3	immediate early response 3	3953.607	1179.271	1638.203
1398367_at	287643	Spop	speckle-type POZ protein	3950.824	3504.549	3391.65
1398933_at	290999	Higd2a_pre	HIG1 domain family, member 2A (prec	3949.682	11966.06	3013.654
1382365_at	497961	RGD15616	similar to nemo like kinase (predicted)	3948.621	3565.472	1758.869
1372154_at	300782	RGD13596	similar to 2010321M09Rik protein	3946.762	4082.412	2517.309
1367728_at	60381	Tsn	translin	3941.859	6010.841	673.071
1389608_at	311959	Abcf2_prec	ATP-binding cassette, sub-family F (G	3939.23	3792.502	1415.053
1373813_at	295690	Dnajc10	DnaJ (Hsp40) homolog, subfamily C, n	3937.757	6703.426	2314.07
1372679_at	308759	Unc45a	unc-45 homolog A (C. elegans)	3937.413	2292.806	1941.082
1372873_at	307390	Fbxo38_pr	F-box protein 38 (predicted)	3930.201	3335.185	4530.34
1378543_at	312331	Nfe2l3_pre	nuclear factor, erythroid derived 2, like	3928.651	5793.146	8377.222
1390814_at	305549	Peli1	pellino homolog 1 (Drosophila)	3925.071	4213.326	16758.88
1383326_a_at	64031	Pdcd4	programmed cell death 4	3924.755	3676.657	14263.49
1398936_at	299027	Eif2s3x	eukaryotic translation initiation factor 2	3922.81	6717.745	2070.702
1371634_at	293113	RGD13056	similar to RIKEN cDNA 1810020E01	3922.451	6578.782	2508.587
1398268_at	25337	Nfyc	nuclear transcription factor-Y gamma	3922.028	5222.205	5109.256
1398458_at	306811	RGD13072	similar to protein kinase, lysine deficier	3921.459	2511.53	5824.308
1388858_at	303200	Map2k3	mitogen activated protein kinase kinas	3918.17	2770.802	2113.874
1386967_at	85428	Rhoq	ras homolog gene family, member Q	3917.474	5084.414	7224.519
1389507_at	291553	Nedd4l	neural precursor cell expressed, devel	3916.888	1471.887	2843.118
1389309_at	304470	Sbno1	sno, strawberry notch homolog 1 (Dros	3916.878	3380.166	6718.475
1376931_at	311575	RGD13050	similar to Hepatocellular carcinoma-as	3915.305	2875.052	1587.324
1372528_at	60355	Nsf	N-ethylmaleimide sensitive fusion prote	3914.697	5429.768	1108.056
1371255_at	293621	Hras	Harvey rat sarcoma viral (v-Ha-ras) on	3911.035	3925.439	1252.367
1376483_at	306222	Lsm5_prec	LSM5 homolog, U6 small nuclear RNA	3905.261	8508.114	7340.765
1398934_at	308267	Map3k7ip2	mitogen-activated protein kinase kinas	3903.061	4074.055	9278.139
1376974_at	362696	Ttc7	tetratricopeptide repeat domain 7	3899.821	899.2488	1466.471
1370921_at	65169	Scamp3	secretory carrier membrane protein 3	3898.454	2081.384	2965.542
1385856_at	291061	Riok1	RIO kinase 1 (yeast)	3898.366	3397.483	1884.521
1367499_at	311204	Slc35c1_pr	solute carrier family 35, member C1 (p	3897.177	3985.674	2117.57
1370321_at	83533	Pdcd8	programmed cell death 8	3896.875	3262.688	1147.531
1399130_at	502782	LOC50278	similar to RIKEN cDNA 2610022G08	3895.381	5585.247	3470.216
1387186_at	84589	Rab9	RAB9, member RAS oncogene family	3894.718	6803.932	4108.303
1383146_at	361948	RGD15626	similar to neurobeachin (predicted)	3892.986	5906.909	30161.31
1375161_at	287356	Mrpl55_pre	mitochondrial ribosomal protein L55 (p	3886.07	9120.374	2566.502
1387781_at	64198	Pmpcb	peptidase (mitochondrial processing) b	3880.724	10817.75	4052.24
1388366_at	363023	Mrpl4_prec	mitochondrial ribosomal protein L4 (pre	3879.996	7450.409	1613.73
1390719_at	365462	RGD13117	similar to DKFZP564P1916 protein (pr	3878.689	2165.84	3076.605
1374233_at	308009	RGD13083	similar to RIKEN cDNA 1810055E12 (p	3877.812	1974.647	3990.766
1374185_at	302899	Txndc11_p	thioredoxin domain containing 11 (prec	3870.677	1446.271	2959.193
1392910_at	300050	Bop1	block of proliferation 1	3870.223	2161.03	900.629
1373191_at	500897	NA	NA	3867.734	1306.043	2768.171
1374517_at	312824	Recql	RecQ protein-like	3865.362	5513.281	4392.267
1375855_at	291180	Epdr2	ependymin related protein 2 (zebrafish	3861.401	6628.537	5881.965

1383752_at	314969	Nol1_predi	nucleolar protein 1 (predicted)	3859.676	1906.521	2038.126
1391437_at	313771	MGC9433S	similar to BC002216 protein	3854.492	1203.93	850.786
1380283_at	307217	Sdccag33_	serologically defined colon cancer anti	3852.775	3218.471	2662.399
1372007_at	500569	RGD15629	similar to AOF2 protein (predicted)	3852.367	3511.316	8517.595
1398599_at	500972	RGD15657	similar to integral membrane protein 1	3851.854	2359.695	2413.455
1370209_at	117560	Klf9	Kruppel-like factor 9	3850.921	7259.394	19120.47
1372723_at	304817	Ipo9_predi	importin 9 (predicted)	3850.289	7655.484	4094.775
1388380_at	300111	Samm50	sorting and assembly machinery comp	3848.685	8489.832	1975.347
1375214_at	292090	Galnt2_pre	UDP-N-acetyl-alpha-D-galactosamine:	3848.327	2118.704	923.9927
1387929_at	171453	Pmf31	PMF32 protein	3846.92	3193.466	2643.955
1374562_at	360642	RGD13114	similar to KIAA1267 protein (predicted)	3838.657	3750.32	12005.43
1374197_at	304721	RGD15625	similar to expressed sequence AA415	3837.156	4759.063	3046.266
1373475_at	288065	Ccdc58_pr	coiled-coil domain containing 58 (predi	3835.675	5549.99	2809.405
1372487_at	619573	LOC61957	hypothetical protein LOC619573	3832.96	3100.285	3683.551
1374326_at	298699	Ppan	peter pan homolog (Drosophila)	3832.426	2529.824	1231.924
1374418_at	291914	RGD13088	similar to CG8009-PA (predicted)	3827.689	3185.196	5129.86
1367903_at	79239	Hmox2	heme oxygenase (decycling) 2	3825.214	4820.411	841.4674
1374253_at	309053	RGD13072	similar to RIKEN cDNA 2310035C23 (	3824.514	3740.085	3947.476
1386908_at	64045	Glrx1	glutaredoxin 1 (thioltransferase)	3823.142	4394.946	4333.469
1371617_at	365388	Psmd13_p	proteasome (prosome, macropain) 26S	3822.853	7377.54	1639.571
1388871_at	290829	Ash2l_pred	ash2 (absent, small, or homeotic)-like	3822.631	5333.552	4485.972
1376670_at	363026	Prmt4	protein arginine N-methyltransferase 4	3820.342	807.4746	1497.419
1388827_at	289784	H2afv_pred	H2A histone family, member V (predict	3820.158	23565.61	12608.58
1373573_at	246185	Pp3111	PP3111 protein	3815.913	4016.502	2192.984
1376576_at	297412	Dusp11	dual specificity phosphatase 11 (RNA/	3814.773	1715.819	1450.707
1371719_at	294276	Brd2	bromodomain containing 2	3810.046	1949.809	1759.085
1371797_at	24240	Cad_mapp	carbamyl phosphatate synthetase 2 (m	3806.11	2217.678	1072.494
1389251_at	361413	Nudt7_pred	nudix (nucleoside diphosphate linked r	3800.106	3516.185	3464.879
1389387_at	689852	LOC68985	NA	3799.881	4679.392	3148.979
1368598_at	171044	Sstr3	somatostatin receptor 3	3799.786	2898.106	2805.581
1368778_at	29464	Slc6a6	solute carrier family 6 (neurotransmitte	3798.505	6497.245	517.8957
1379645_at	306254	RGD15655	similar to polybromo-1 (predicted)	3798.268	5471.535	1925.335
1374442_at	288701	Sfrs9	splicing factor, arginine/serine rich 9	3796.762	6019.289	3370.92
1371611_at	311215	Ext2_predi	exostoses (multiple) 2 (predicted)	3789.213	8548.401	2148.152
1374430_at	363644	RGD13070	similar to RIKEN cDNA 2700085E05	3788.701	6507.885	3862.03
1369935_at	25193	Ccnd3	cyclin D3	3788.136	1991.329	1103.782
1369978_at	117272	Prpsap2	phosphoribosyl pyrophosphate synthet	3783.993	6611.798	5150.125
1376250_at	364430	Nufip1	nuclear fragile X mental retardation pro	3782.271	3386.405	10521.31
1372591_at	303196	Tmem11_p	transmembrane protein 11 (predicted)	3780.291	4560.681	3236.789
1388571_at	89815	Syngr2	synaptogyrin 2	3777.588	1677.682	2049.477
1374011_at	291703	Ercc3	excision repair cross-complementing r	3775.872	3856.963	2075.33
1371246_at	291981	NTF2	nuclear transport factor 2	3773.413	2745.408	1022.658
1398899_at	361365	Polr2c	polymerase (RNA) II (DNA directed) po	3771.924	6717.092	2250.594
1381174_at	307480	RGD73502	SEL1 domain containing protein RGD7	3771.056	1403.562	1679.427
1371828_at	288667	RGD13108	similar to RIKEN cDNA 1500011H22	3768.216	10578.94	5482.54
1367976_at	81815	Tpp2	tripeptidyl peptidase II	3765.292	4626.236	9470.203
1383103_at	294606	RGD13055	similar to Sperm 1 POU-domain transc	3763.867	4539.939	3529.213
1373213_at	116500	Snap29	synaptosomal-associated protein 29	3762.286	4447.917	2134.482
1368143_at	155423	Anxa7	annexin A7	3760.031	3278.719	2853.205
1387788_at	24517	Junb	Jun-B oncogene	3753.757	229.3555	5554.395
1389435_at	498366	RGD15602	similar to RIKEN cDNA 9130005N14 (	3752.617	1645.904	3887.031

1372764_at	498180	NA	NA	3752.152	6579.369	3538.528
1399117_at	301079	RGD13117	similar to RIKEN cDNA 1110059G10	3749.908	3039.55	3577.153
1387899_at	25415	Crmp1	collapsin response mediator protein 1	3746.186	13374.47	3705.594
1369699_at	25051	Glp1r	glucagon-like peptide 1 receptor	3746.153	3726.861	160.4627
1367722_at	83799	Dpp7	dipeptidylpeptidase 7	3745.499	5294.457	3547.016
1372876_at	308993	Sephs2	selenophosphate synthetase 2	3744.418	3987.888	2669.966
1372843_at	363020	RGD13094	LOC363020 (predicted)	3744.404	1149.863	1795.712
1371681_at	286990	RGD70844	epidermal Langerhans cell protein LCF	3742.52	2558.841	4979.159
1367487_at	494342	B4galt3	UDP-Gal:betaGlcNAc beta 1,4-galacto	3739.789	3292.764	1846.469
1373704_at	366163	Aqr_predic	aquarius (predicted)	3739.088	7953.369	1797.259
1374852_at	362592	RGD15599	RGD1559909 (predicted)	3738.318	3087.744	2025.807
1371875_at	310864	Manba	mannosidase, beta A, lysosomal	3736.89	888.2474	7907.109
1371962_at	293481	Tufm_pred	Tu translation elongation factor, mitoch	3731.627	4506.001	2247.215
1371717_at	192647	Mfn1	mitofusin 1	3731.404	2734.358	1411.105
1377791_at	117043	RragB	Ras-related GTP binding B	3729.813	7369.935	23132.04
1389002_at	313494	Tln1	talin 1	3729.754	2049.608	2148.769
1382074_at	25008	Lta	lymphotoxin A	3729.249	973.1514	1641.137
1392386_at	117279	Cflar	CASP8 and FADD-like apoptosis regu	3728.346	623.9993	3534.173
1367839_at	29580	Fdft1	farnesyl diphosphate farnesyl transfera	3726.921	5498.572	2630.615
1370036_at	81805	Suox	sulfite oxidase	3723.119	7615.313	4990.639
1382144_at	294963	Mrpl47	mitochondrial ribosomal protein L47	3721.153	2360.574	1462.632
1372552_at	289312	Acbd3	acyl-Coenzyme A binding domain cont	3721.143	1533.172	7852.033
1376034_at	362353	RGD15654	similar to mKIAA0738 protein (predicte	3719.762	5583.323	7525.031
1373294_at	289603	Commd8_p	COMM domain containing 8 (predicted	3716.767	6357.172	1704.966
1369996_at	83503	Polr2f	polymerase (RNA) II (DNA directed) po	3716.028	5214.799	2672.143
1389557_at	297392	Tex261	testis expressed gene 261	3715.239	5068.66	910.5287
1398316_at	288913	LOC28891	similar to LEYDIG CELL TUMOR 10 K	3714.819	2412.061	1312.705
1387081_at	29218	Rcn2	reticulocalbin 2	3710.484	3242.621	9802.92
1398980_at	305851	Supt16h_p	suppressor of Ty 16 homolog (S. cerev	3710.246	3298.526	1381.885
1398926_at	361310	Pfdn1_pred	prefoldin 1 (predicted)	3709.882	10131.13	3758.767
1373647_at	294846	Zfp622	zinc finger protein 622	3706.892	2507.505	5896.435
1376657_at	363058	Igsf4a	immunoglobulin superfamily, member 4	3705.559	3795.058	7707.925
1368450_at	25017	Myo5a	myosin Va	3704.272	812.885	498.9278
1388842_at	501099	RGD15597	similar to serum response factor (predi	3703.983	2964.575	8549.686
1374495_at	361975	Lrba_predi	LPS-responsive beige-like anchor (pre	3703.171	2387.63	3822.707
1389363_at	298934	Adi1	acireductone dioxygenase 1	3703.163	7792.058	3792.238
1395725_at	314075	RGD13114	similar to intracellular membrane-asso	3702.537	1867.157	3060.778
1367772_at	65160	Clns1a	chloride channel, nucleotide-sensitive,	3701.654	7452.26	3278.81
1382008_at	361751	RGD13098	similar to hypothetical protein FLJ1121	3698.541	3697.193	2645.758
1372575_at	297695	Wbp11	WW domain binding protein 11	3697.443	4561.547	7725.616
1383160_at	315447	Chordc1_p	cysteine and histidine-rich domain (CH	3693.579	3387.708	8364.73
1367761_at	170845	Ndel1	nudE nuclear distribution gene E homo	3688.252	2261.03	3035.552
1386877_at	65046	Ap2s1	adaptor-related protein complex 2, sig	3687.742	7455.467	1351.154
1390803_at	317612	Htatsf1_pre	HIV TAT specific factor 1 (predicted)	3684.715	7812.649	9373.127
1373888_at	309969	Ap3b1_pre	adaptor-related protein complex 3, bet	3684.361	3968.556	2942.428
1372312_at	361452	Ltv1	LTV1 homolog (S. cerevisiae)	3678.722	5694.05	1878.815
1388169_at	171120	Jmjd1c	jumonji domain containing 1C	3674.453	2822.109	26373.4
1384154_at	114765	Wbp4	WW domain binding protein 4	3670.148	1179.13	2831.914
1369008_a_at	93667	Olfm1	olfactomedin 1	3669.858	13623.63	2610.071
1383461_at	362914	RGD13113	similar to hypothetical protein FLJ1020	3668.391	2890.448	2314.579
1384465_at	305910	Pspc1	paraspeckle protein 1	3666.349	2771.696	5509.222

1382660_at	315912	RGD1563(	similar to FLJ20298 protein isoform a (	3663.596	972.9302	4336.232
1372113_at	287375	Fliih	flightless I homolog (Drosophila)	3663.558	6154.668	2118.184
1374532_at	311865	Ptges2_pre	prostaglandin E synthase 2 (predicted)	3662.87	1727.231	894.5359
1370223_at	117051	Arfrp1	ADP-ribosylation factor related protein	3661.27	5247.193	1193.532
1389228_at	297415	RGD13048	similar to RIKEN cDNA 2010309E21 (|	3660.628	4523.737	1266.565
1376843_at	140590	Bmpr2	bone morphogenic protein receptor, ty	3660.439	11.86926	681.68
1382390_at	362106	Fubp3	far upstream element (FUSE) binding		3660.327	4908.341	7517.686
1398913_at	308870	Numa1	nuclear mitotic apparatus protein 1	3658.77	3384.961	2104.588
1396831_at	310405	Glrp1_pred	glutamine repeat protein 1 (predicted)	3657.302	1225.647	1252.891
1386718_at	307096	RGD15629	similar to aldo-keto reductase family 1,	3657.065	5714.385	3808.041
1373411_at	301068	Eif1b_pred	eukaryotic translation initiation factor 1	3654.303	2667.486	6231.291
1394591_at	303763	Zfp207	zinc finger protein 207	3654.167	5582.97	10192.68
1374612_at	307745	Papd5_pre	PAP associated domain containing 5 (	3653.063	3491.753	9235.932
1373395_at	303088	Trim41_pre	tripartite motif protein 41 (predicted)	3652.383	2967.651	2414.66
1373372_at	501282	LOC50128	NA	3651.461	3855.789	903.5441
1367811_at	58835	Phgdh	3-phosphoglycerate dehydrogenase	3649.316	128.7597	162.5878
1385228_x_at	314961	Ddef1_pred	development and differentiation enhan	3648.01	6138.493	2716.23
1373835_at	306436	Fbxo8	F-box only protein 8	3647.157	5118.829	5857.165
1381461_at	502348	RGD15617	similar to StAR-related protein 1-4E (p	3645.752	1543.334	1837.042
1370356_at	64510	Rbm10	RNA binding motif protein 10	3644.651	3546.398	4180.057
1375037_at	309144	Saps3_pre	SAPS domain family, member 3 (predi	3644.31	5231.546	7948.821
1372516_at	293502	Kif22	kinesin family member 22	3639.794	7122.581	354.9638
1388633_at	309454	RGD13093	similar to RIKEN cDNA 4930538D17	3639.541	4387.525	1383.848
1373775_at	296731	Nub1	NEDD8 ultimate buster-1	3636.593	3293.515	2669.279
1391037_at	303812	RGD15644	RGD1564491 (predicted)	3636.482	4934.98	8960.489
1373398_at	360592	Trim37_pre	tripartite motif protein 37 (predicted)	3634.609	4503.541	2771.598
1373144_at	499654	NA	NA	3632.197	1042.393	1496.474
1398943_at	293863	RGD15624	similar to Eso3 protein (predicted)	3632.146	11767.6	1890.58
1391356_at	291705	RGD13047	similar to hypothetical protein FLJ1000	3630.996	4591.126	3647.625
1382175_at	499020	RGD15638	similar to Wilms tumor 1-associating pı	3629.78	4607.049	6299.322
1376703_at	299706	Nup37_pre	nucleoporin 37 (predicted)	3629.395	4304.603	3180.887
1388801_at	305466	RGD13054	similar to RIKEN cDNA 9030221M09 g	3626.381	3218.155	2854.423
1399097_at	361217	Spin	spindlin	3625.391	10371.88	9723.427
1371576_at	294696	Mrps36_pr	mitochondrial ribosomal protein S36 (p	3624.94	9793.453	2944.56
1371730_at	291922	RGD13054	similar to RIKEN cDNA 1300002A08	3623.233	3188.164	4113.235
1383296_a_at	290280	Xpo4_pred	exportin 4 (predicted)	3622.24	1742.107	1052.314
1370523_a_at	641452	Ubc2e	NA	3617.69	5250.828	15811.57
1368073_at	24508	Irf1	interferon regulatory factor 1	3614.288	1760.473	13859.49
1389407_at	290234	Dhrs1	dehydrogenase/reductase (SDR family	3612.705	1823.681	1104.914
1376057_at	308776	Pde8a	phosphodiesterase 8A	3611.269	1555.275	5374.884
1374007_at	317672	Slu7	step II splicing factor SLU7 (S. cerevis	3609.551	6276.319	5095.908
1367777_at	117543	Decr1	2,4-dienoyl CoA reductase 1, mitochor	3608.864	5675.93	3640.674
1370085_at	25676	Rasa1	RAS p21 protein activator 1	3608.778	2797.488	3161.772
1374995_at	291962	Elmo3	engulfment and cell motility 3, ced-12 l	3608.194	1213.901	1974.235
1398914_at	288588	Polr2j_pred	polymerase (RNA) II (DNA directed) po	3604.648	7943.546	2145.663
1373495_at	297961	Ube2j1_pre	ubiquitin-conjugating enzyme E2, J1 (p	3603.15	1982.512	2516.074
1381063_at	302950	Adcy9_pre	adenylate cyclase 9 (predicted)	3602.818	2912.327	1576.745
1372023_at	498674	NA	NA	3601.984	4048.093	4217.363
1371359_at	312709	Mlf2_predi	myeloid leukemia factor 2 (predicted)	3599.34	3557.299	3234.315
1381976_at	300158	Kif21a_pre	kinesin family member 21A (predicted)	3598.972	6584.632	9406.019
1399055_at	361374	Brd7_predi	bromodomain containing 7 (predicted)	3598.741	3024.977	3017.486

1372454_at	288704	RGD13118	similar to RIKEN cDNA 2210016L21 g	3598.185	5009.852	3623.348
1398921_at	56281	Mrpl37	mitochondrial ribosomal protein L37	3595.51	11786.94	2104.81
1373152_at	308807	Prss23	protease, serine, 23	3593.037	2145.628	35895.25
1383152_at	293628	RGD13089	similar to Cc1-9	3592.734	2000.529	1050.262
1383491_at	361594	Isg20l1_pre	interferon stimulated exonuclease gen	3592.394	1074.109	1416.944
1392165_at	290744	Ing1l_predi	inhibitor of growth family, member 1-lik	3591.058	4175.027	6185.368
1390430_at	259241	Nr1d2	nuclear receptor subfamily 1, group D,	3589.854	6580.909	27068.02
1389162_at	297416	Hirip5_prec	histone cell cycle regulation defective i	3589.694	3756.311	1880.014
1383396_at	306022	Fndc3a_pr	fibronectin type III domain containing 3	3589.186	1731.605	13372.03
1372740_at	303259	LOC30325	similar to Map4k6-pending protein	3588.331	2550.859	4803.873
1372714_at	312135	RGD13077	similar to RIKEN cDNA 8430437G11	3588.196	6145.199	4164.01
1389108_at	363309	LOC36330	NA	3587.681	4091.407	2316.212
1372801_at	361323	Commd10	COMM domain containing 10	3587.035	8358.288	5110.509
1374828_at	360722	Pdia5	protein disulfide isomerase-associated	3584.429	493.0865	1456.209
1371919_at	308518	Nudt19	nudix (nucleoside diphosphate linked r	3582.8	2906.919	3327.403
1398987_at	287608	Supt4h2_p	suppressor of Ty 4 homolog 2 (S. cere	3582.01	8418.589	3650.973
1375232_at	362892	Mbd6_prec	methyl-CpG binding domain protein 6 (	3580.932	1837.279	2518.867
1398968_at	360477	RGD13094	similar to RIKEN cDNA 3930401K13	3577.819	5491.158	5009.681
1371684_at	294754	Pelo	pelota homolog	3575.467	1426.18	2970.925
1378637_at	313166	Nfx1	nuclear transcription factor, X-box binc	3574.265	4941.383	7383.016
1377756_at	362394	RGD13076	similar to RIKEN cDNA 5830446M03	3570.264	2998.38	2125.943
1388749_at	312439	RGD13097	similar to hypothetical protein FLJ1391	3569.39	8875.513	10475.24
1378958_at	293738	MGC9472(	similar to hypothetical protein MGC257	3568.726	5511.847	4123.658
1371539_at	287273	Nola2_prec	nucleolar protein family A, member 2 (	3568.394	5509.516	1503.878
1368248_at	81925	Cds1	CDP-diacylglycerol synthase 1	3568.214	3060.781	1306.622
1372128_at	292758	Mrps12_pr	mitochondrial ribosomal protein S12 (p	3565.143	4810.808	1461.462
1373392_at	364137	Tparl	TPA regulated locus	3563.944	3251.281	3313.849
1398373_at	310508	B3galt3	UDP-Gal:betaGlcNAc beta 1,3-galacto	3563.739	9273.353	6284.331
1379164_at	316524	Znf142_pre	zinc finger protein 142 (clone pHZ-49)	3562.551	864.18	2080.765
1378275_at	361176	Upf3a	UPF3 regulator of nonsense transcript	3562.283	4644.494	5057.184
1398574_a_at	306998	Cdc2l5	cell division cycle 2-like 5 (cholinestera	3558.372	2061.788	2015.501
1372357_at	362237	Tbc1d20	TBC1 domain family, member 20	3557.517	1298.727	1751.299
1389338_at	293114	RGD13083	hypothetical LOC293114 (predicted)	3556.659	7116.854	5185.941
1381878_at	302935	Ubn1_pred	ubinuclein 1 (predicted)	3554.12	2161.83	1693.386
1387055_at	84019	Appbp1	amyloid beta precursor protein binding	3551.577	4776.233	5009.135
1372287_at	305935	Mtmr6_pre	myotubularin related protein 6 (predicte	3551.47	2089.103	7059.016
1373456_at	300813	Rnf111_pre	ring finger protein 111 (predicted)	3550.238	3412.46	4882.645
1374503_at	311876	Pbx3_pred	pre B-cell leukemia transcription factor	3548.422	4184.391	3491.652
1368668_at	116645	Plaa	phospholipase A2, activating protein	3548.247	1688.659	1772.531
1368303_at	63840	Per2	period homolog 2 (Drosophila)	3545.153	1855.101	37239.65
1399141_at	287269	Clk4	CDC like kinase 4	3545.101	3127.612	10802.82
1375878_at	311880	Gapvd1_pr	GTPase activating protein and VPS9 d	3544.182	3295.485	2744.253
1388457_at	290635	MGC72581	similar to RIKEN cDNA 1110012M11	3540.999	4413.9	1698.369
1376299_at	312678	LOC31267	NA	3538.387	1459.752	1229.843
1370311_at	64514	Eif2b1	eukaryotic translation initiation factor 2	3536.938	4970.639	2059.647
1376664_at	246187	LOC24618	liver regeneration-related protein	3536.433	1425.283	4079.775
1387115_at	140934	Ikbkap	inhibitor of kappa light polypeptide enh	3535.994	4758.265	2372.901
1388994_at	282581	Fzd6	frizzled homolog 6 (Drosophila)	3533.536	2041.592	2906.642
1383951_at	287212	RGD13095	similar to hypothetical protein FLJ3195	3530.988	5337.057	5687.083
1389535_at	295238	Dap3	death associated protein 3	3530.251	3889.088	5232.752
1391693_at	291555	Atp8b1_pre	ATPase, Class I, type 8B, member 1 (p	3529.233	2200.172	1093.326

1372331_at	302697	RGD15601	similar to eukaryotic translation initiatio	3524.401	9752.964	3390.298
1369974_at	24803	Vamp2	vesicle-associated membrane protein 2	3522.359	1748.874	1219.15
1372105_at	171433	Efha1	EF hand domain family A1	3519.91	1662.92	2914.423
1385020_at	294066	RGD13087	similar to DNA segment, Chr 19, Brigh	3518.973	2811.675	346.7215
1380163_at	309010	Sec23ip	SEC23 interacting protein	3516.493	2092.689	4448.024
1386927_at	25413	Cpt2	carnitine palmitoyltransferase 2	3514.351	4908.351	1136.486
1372571_at	362849	2-Mar	membrane-associated ring finger (C3H	3513.295	4401.281	6317.236
1391443_at	498489	RGD15599	similar to chromosome 14 open readin	3511.778	2252.397	7700.834
1389055_at	298508	Ppie	peptidylprolyl isomerase E (cyclophilin	3508.789	5200.903	2279.661
1368230_a_at	56769	RGD70854	nuclear protein E3-3	3507.655	2850.094	3604.91
1372386_at	78973	Senp2	SUMO/sentrin specific protease 2	3507.028	5067.739	3078.167
1388931_at	299938	Mrpl13	mitochondrial ribosomal protein L13	3506.344	9906.21	3900.216
1372325_at	298845	Emilin1_pr	elastin microfibril interfacer 1 (predicte	3505.608	200.5544	98.93002
1375337_at	57024	Adam9	a disintegrin and metalloproteinase do	3505.381	2104.978	2061.411
1370014_at	81803	Stx4a	syntaxin 4A (placental)	3502.75	3967.165	1679.338
1388360_at	50558	Npepps	aminopeptidase puromycin sensitive	3502.004	2521.746	2652.111
1393707_at	60434	Bcl2l2	Bcl2-like 2	3501.4	1688.492	4203.907
1393359_at	308777	Ap3b2_pre	adaptor-related protein complex 3, bet	3501.383	2388.073	5651.626
1388655_at	311802	Ssna1_pre	Sjogren's syndrome nuclear autoantige	3500.878	4988.192	1831.059
1389339_at	315222	Arsa	arylsulfatase A	3496.762	1918.771	2886.097
1397525_at	303193	Alkbh5_pre	alkB, alkylation repair homolog 5 (E. co	3495.999	971.7271	710.6774
1367571_a_at	24483	Igf2	insulin-like growth factor 2	3495.905	17596.23	101.0837
1368162_at	171096	Cst6	cystatin E/M	3494.636	17664.74	8463.299
1387017_at	29230	Sqle	squalene epoxidase	3491.328	9425.437	8679.829
1372744_at	295625	Pkp4_pred	plakophilin 4 (predicted)	3490.411	1435.303	6551.35
1376715_at	365567	Cbara1	calcium binding atopy-related autoanti	3490.005	1366.451	1144.703
1373441_at	308961	Dctn5	dynactin 5	3489.354	5952.349	3089.358
1373830_at	619574	LOC61957	hypothetical protein LOC619574	3489.021	3253.803	2692.774
1388217_a_at	64366	Calu	calumenin	3488.65	3058.706	1808.922
1371008_at	296588	Pmpca	peptidase (mitochondrial processing) a	3488.567	3096.565	961.8611
1372116_at	362094	Mrps2_pre	mitochondrial ribosomal protein S2 (pr	3488.254	5687.924	2531.22
1373966_at	303188	Rai1_predi	retinoic acid induced 1 (predicted)	3485.734	4972.16	4731.691
1368005_at	25679	Itpr3	inositol 1,4,5-triphosphate receptor 3	3483.482	1437.298	994.7151
1378380_at	292756	Pak4_pred	p21 (CDKN1A)-activated kinase 4 (pre	3483.308	5442.116	5134.573
1387089_at	171050	Prei3	preimplantation protein 3	3477.858	5183.564	7393.716
1390989_at	363463	RGD15639	similar to Mospd2 protein (predicted)	3477.004	4468.689	8969.829
1374331_at	301513	Rqcd1	rcd1 (required for cell differentiation) h	3476.861	3610.304	5561.178
1373106_at	298765	Zfp36l2	zinc finger protein 36, C3H type-like 2	3473.519	2034.432	21834.87
1392089_at	314704	Hcfc2	host cell factor C2	3473.113	3929.582	2933.322
1398951_at	291841	RGD13080	similar to KIAA1007 protein; adrenal gl	3472.495	4089.358	3363.393
1371469_at	64152	Chp	calcium binding protein p22	3471.397	5862.569	2593.638
1375887_at	501506	RGD15663	similar to kaiso protein (predicted)	3471.327	4724.603	7893.885
1389098_at	301038	Ubp1_pred	upstream binding protein 1 (predicted)	3470.833	1681.762	2742.977
1377868_at	317405	RGD15610	similar to hypothetical protein (predicte	3469.101	6595.04	23856.71
1390149_at	309025	Tacc2	transforming, acidic coiled-coil contain	3468.018	4136.367	3364.457
1382426_at	499950	RGD15637	similar to BC040823 protein (predicted	3461.566	3618.247	1475.582
1368401_at	29627	Gria2	glutamate receptor, ionotropic, AMPA2	3460.263	427.8752	5253.444
1372375_at	363278	Atg16l1_pr	ATG16 autophagy related 16-like 1 (S.	3458.683	1738.414	2583.187
1391347_at	266688	Rab8b	RAB8B, member RAS oncogene family	3454.188	364.2055	1106.016
1374139_at	308958	Cdr2	cerebellar degeneration-related 2	3453.326	2283.995	4660.188
1398804_at	64472	Mak10	MAK10 homolog, amino-acid N-acetylt	3452.77	8226.301	2352.05

1388625_at	290679	RGD13047	similar to RIKEN cDNA 4921521J11 (p	3450.994	3896.603	2416.48
1367550_a_at	362545	Tm2d1_pre	TM2 domain containing 1 (predicted)	3450.521	4485.297	6500.105
1387367_at	29476	Glg1	golgi apparatus protein 1	3450.401	2548.771	1420.449
1367727_at	116692	Pscd2	pleckstrin homology, Sec7 and coiled-c	3450.155	10384.81	2392.153
1389504_at	500847	RGD15661	similar to Deltex3 (predicted)	3450.032	6587.16	6256.535
1398327_at	289992	Plekhc1	pleckstrin homology domain containing	3449.603	3216.295	9148.45
1368686_at	25377	Ambp	alpha 1 microglobulin/bikunin	3448.262	15355.5	4404.217
1376156_at	316091	RGD13048	hypothetical LOC316091	3445.348	1705.243	1413.802
1399010_at	312710	Cops7a_pr	COP9 (constitutive photomorphogenic	3445.07	2366.376	1932.235
1382466_at	291060	RGD13090	similar to RIKEN cDNA 6530403A03	3443.393	2588.931	2732.731
1372554_at	316335	RGD13092	similar to RW1 protein (predicted)	3442.915	3951.162	8060.706
1378038_at	310820	Ptbp2	polypyrimidine tract binding protein 2	3441.375	478.8253	5771.401
1374086_at	307178	Arhgap21_	Rho GTPase activating protein 21 (pre	3440.718	5869.957	11603.52
1380728_at	25416	Dpysl2	dihydropyrimidinase-like 2	3439.961	12122.39	2939.323
1389028_at	116464	Ncoa6	nuclear receptor coactivator 6	3439.228	4626.079	4834.668
1387861_at	29466	Aes	amino-terminal enhancer of split	3439.179	3697.374	2261.492
1372179_at	50871	Hpcal1	hippocalcin-like 1	3436.46	4405.474	4932.463
1374424_at	364382	Prmt5_pred	protein arginine N-methyltransferase 5	3436.431	1787.844	1770.508
1398939_at	360618	LOC36061	similar to ORM1-like 3	3436.062	3371.106	3000.132
1371534_at	309111	Slc25a22	solute carrier family 25 (mitochondrial c	3435.62	1167.336	1819.845
1368022_at	65038	Inppl1	inositol polyphosphate phosphatase-lik	3432.811	3608.699	1382.226
1390524_at	317241	Rnf12	ring finger protein 12	3432.337	1578.784	2104.646
1371769_at	65168	Scamp2	secretory carrier membrane protein 2	3431.624	1026.034	1045.352
1381323_at	304794	Rbbp5_pre	retinoblastoma binding protein 5 (predi	3431.501	2395.867	757.6569
1389080_at	170900	Rbm14	RNA binding motif protein 14	3431.473	3535.704	3217.287
1376366_at	295217	Snapap	SNAP-associated protein	3429.457	7603.511	4386.571
1382065_at	362722	Tmem18	transmembrane protein 18	3428.8	1750.409	2292.734
1373117_at	309331	Uhrf2_prec	ubiquitin-like, containing PHD and RIN	3428.255	4719.983	3710.779
1376571_at	363171	Tmem42_p	transmembrane protein 42 (predicted)	3427.552	1135.424	1322.041
1393242_at	287170	RGD13109	similar to chromosome 16 open readin	3421.512	2896.054	1984.448
1368008_at	60357	Prom1	prominin 1	3419.308	218.7406	2345.956
1370337_at	83726	Ctcf	CCCTC-binding factor	3418.438	4168.707	5744.553
1374154_at	312030	LOC31203	NA	3417.172	2988.176	6490.654
1390507_at	293052	Isg20	interferon stimulated exonuclease 20	3416.254	4714.058	3906.908
1386945_a_at	83803	Prkab1	protein kinase, AMP-activated, beta 1	3415.739	2827.764	4135.647
1398805_at	171415	Apg3l	APG3 autophagy 3-like (S. cerevisiae)	3415.366	5965.416	6134.436
1372999_at	315405	Dcun1d5	DCN1, defective in cullin neddylation 1	3414.121	3.742291	5556.849
1379469_at	302711	Tbl1x_prec	transducin (beta)-like 1 X-linked (predi	3411.34	3559.652	605.788
1374584_at	361092	RGD15617	similar to Serine/threonine protein kina	3410.132	1402.666	3669.472
1368031_at	64896	Nolc1	nucleolar and coiled-body phosphopro	3407.741	5921.938	2084.906
1367511_at	289231	Ncstn	nicastrin	3406.96	3097.242	2773.469
1371968_at	362884	Tmbim4	transmembrane BAX inhibitor motif cor	3405.933	10453.49	3283.069
1374420_at	366227	RGD13088	similar to RIKEN cDNA 2310001A20	3405.052	5307.064	3830.488
1399113_at	299488	RGD15657	similar to chr2 synaptotagmin (predicte	3403.414	3712.226	5726.56
1389312_at	298787	RGD13099	similar to RIKEN cDNA 0610016J10 ge	3399.074	6298.835	5954.176
1372329_at	362037	RGD13114	similar to hypothetical protein PRO097	3398.865	2033.196	4541.694
1370086_at	24367	Fgg	fibrinogen, gamma polypeptide	3396.03	135.9129	987.7934
1377735_at	295674	Plekha3	pleckstrin homology domain-containing	3395.087	1726.472	4764.519
1367900_at	81675	Gyg1	glycogenin 1	3393.965	5314.12	4926.386
1384115_at	192272	Mte1	mitochondrial acyl-CoA thioesterase 1	3393.534	969.7898	2299.227
1370909_at	65274	Nup62	nucleoporin 62	3388.128	7106.766	3465.223

1367672_at	79244	Hsd17b4	hydroxysteroid (17-beta) dehydrogena	3385.577	4784.903	7520.099
1367820_at	114087	Banf1	barrier to autointegration factor 1	3384.716	6445.305	3142.223
1371834_at	299850	Dctn2	dynactin 2	3384.002	4599.437	1670.967
1371654_at	313699	RGD13053	similar to RIKEN cDNA 2510039O18 (	3382.282	1609.685	1447.636
1368775_at	171090	Giot1	gonadotropin inducible ovarian transcr	3379.494	343.6638	19580.99
1371967_at	293754	Mrpl16	mitochondrial ribosomal protein L16	3378.968	5064.032	1458.045
1388448_at	501691	NA	NA	3378.768	5427.19	3373.214
1388812_at	287550	Trp53i13_p	tumor protein p53 inducible protein 13	3377.91	898.0864	938.2546
1371952_at	311902	Rbm18_pre	RNA binding motif protein 18 (predicte	3377.902	6013.359	7049.121
1374412_at	501682	NA	NA	3375.546	7139.664	7443.112
1383267_at	291000	Ubxd8	UBX domain containing 8	3372.355	2913.792	1955.389
1383543_at	362460	Golt1b_pre	golgi transport 1 homolog B (S. cerevis	3370.19	2043.074	1743.55
1382021_at	498328	RGD15599	similar to polycystic kidney disease 2 (	3369.124	2743.799	3337.815
1389833_at	362409	Sumf1_pre	sulfatase modifying factor 1 (predicted	3367.017	2006.489	1222.49
1379398_at	289051	Fam31b_p	family with sequence similarity 31, mer	3362.786	2232.269	836.3019
1368528_at	171485	Mic2l1	MIC2 like 1	3362.54	3038.625	9076.729
1376419_at	300231	RGD13056	similar to expressed sequence AI3172	3361.828	1881.337	631.4759
1390730_at	303321	RGD13056	similar to 1810009O10Rik protein	3360.669	2224.685	1706.961
1373101_at	295543	Pigk	phosphatidylinositol glycan, class K	3360.578	4355.322	5851.983
1379368_at	303836	Bcl6_predi	B-cell leukemia/lymphoma 6 (predicted	3359.851	72.68854	4704.762
1371561_at	303565	G6pc3	glucose 6 phosphatase, catalytic, 3	3355.381	2848.105	1461.218
1376388_at	286937	Pex2	peroxin 2	3354.322	258.8607	841.2157
1376725_at	312781	Lrp6_predi	low density lipoprotein receptor-related	3351.956	997.0577	695.7653
1372265_at	299341	RGD13047	similar to RIKEN cDNA 2810002N01	3351.362	6115.601	4862.047
1376010_at	291078	Prpf4b	PRP4 pre-mRNA processing factor 4 h	3349.5	3485.406	6490.282
1371557_at	363291	Thap4	THAP domain containing 4	3348.561	1148.498	1019.638
1372366_at	192218	Htatip	HIV-1 tat interactive protein, homolog (	3347.833	6648.098	1703.585
1388590_at	361784	Znrd1	zinc ribbon domain containing, 1	3345.513	3731.083	4941.301
1368118_at	83477	Bcl10	B-cell CLL/lymphoma 10	3345.423	3409.255	4412.5
1393987_s_at	59076	Gprk6	G protein-coupled receptor kinase 6	3344.776	15696.4	3825.094
1389175_at	305455	Whsc2	Wolf-Hirschhorn syndrome candidate 2	3341.617	2601.479	1773.198
1385090_at	310034	Rad17	RAD17 homolog (S. pombe)	3340.334	2890.24	3610.692
1387771_a_at	50689	Mapk3	mitogen activated protein kinase 3	3340.091	4776.415	1857.055
1396866_s_at	29534	Pxmp3	peroxisomal membrane protein 3	3335.734	3987.738	1002.518
1391611_at	313254	Kif12	kinesin family member 12	3335.714	5302.262	8545.365
1376797_at	362224	Csrp2bp_p	cysteine and glycine-rich protein 2 binc	3335.28	1998.959	1289.798
1377263_at	303074	Crsp9_pred	cofactor required for Sp1 transcriptiona	3332.365	4206.508	5383.851
1398844_at	79462	Txn2	thioredoxin 2	3331.477	5183.269	1087.467
1373305_at	360725	Snx4_pred	sorting nexin 4 (predicted)	3329.279	10550.09	5967.026
1377616_at	297903	RGD13106	similar to RIKEN cDNA 6720467C03 (p	3327.285	8097.258	17028.1
1384428_at	502228	RGD15664	similar to OTTHUMP00000040081 (pre	3326.958	1895.931	545.6076
1374966_at	84394	Dcx	doublecortin	3324.719	7968.873	2276.763
1388499_at	303922	RGD13593	similar to hypothetical protein MGC753	3320.84	1792.809	1863.27
1398401_at	306066	Tdrd3	tudor domain containing 3	3318.2	585.7726	1433.766
1367807_at	116552	Plod1	procollagen-lysine, 2-oxoglutarate 5-di	3317.32	498.8075	801.1688
1374289_at	301246	Rpo1-1	RNA polymerase 1-1	3317.23	6881.194	3684.315
1383591_at	299620	Sf3a2	splicing factor 3a, subunit 2, 66kDa	3316.175	3077.62	2265.584
1388875_at	291440	Cxxc1	CXXC finger 1 (PHD domain)	3316.095	5441.926	1468.037
1393051_at	501619	LOC50161	similar to 40S ribosomal protein S29	3315.494	1899.077	12029.31
1381190_at	361084	LMO7	LIM domain only protein 7	3315.492	5671.525	10032.52
1371429_at	114489	Dag1	dystroglycan 1	3312.021	3659.206	2475.354

1374568_at	314330	RGD13094	similar to mKIAA1737 protein (predicte	3311.931	1774.088	4166.357
1389044_at	309451	RGD13071	similar to golgi-specific brefeldin A-resi	3309.406	2983.214	1022.934
1382135_at	315903	RGD13078	similar to CG9346-PA (predicted)	3309.393	2132.808	5061.372
1373196_at	364601	Efha2	EF hand domain family, member A2	3308.485	6224.324	7209.188
1370178_at	116600	Cacnb2	calcium channel, voltage-dependent, b	3305.161	3295.685	5011.985
1389723_at	363131	Pik3r4_pre	phosphoinositide-3-kinase, regulatory s	3305.083	1640.958	2498.846
1398432_at	306264	RGD15599	similar to Ankyrin repeat domain protei	3304.911	4125.05	3051.979
1376337_at	361745	Smarca2	SWI/SNF related, matrix associated, a	3303.043	4843.084	5190.151
1384609_a_at	363089	RGD13114	similar to RIKEN cDNA B230380D07 (	3302.896	915.4829	812.6341
1389128_at	305164	Wdfy3_pre	WD repeat and FYVE domain containi	3302.183	3020.46	4381.943
1388286_a_at	299602	Cdc34_pre	cell division cycle 34 homolog (S. cere	3302.046	2675.455	1221.264
1395399_at	500555	RGD15652	similar to PS1D protein (predicted)	3297.418	3173.559	424.9281
1368157_at	29246	Stmn3	stathmin-like 3	3297.219	8935.416	3255.021
1387875_at	25614	Ptk2	PTK2 protein tyrosine kinase 2	3293.706	3346.293	3146.483
1374322_at	499087	NA	NA	3293.412	3275.362	3768.393
1389358_at	498312	NA	NA	3292.816	3878.754	2125.45
1373560_at	303100	RGD15628	similar to N-acetylglucosaminyltrasnfer	3291.856	4420.25	3580.636
1381933_at	304528	Rfc5_predi	replication factor C (activator 1) 5 (pre	3291.374	15595.41	1580.231
1372943_at	361984	Atp8b2	Atpase, class I, type 8B, member 2	3291.325	3288.918	2484.08
1375714_at	365661	RGD15629	similar to Erbb2 interacting protein isof	3289.627	2200.394	3102.58
1386942_at	64528	Golga2	cis-Golgi matrix protein GM130	3288.983	2150.527	1376.083
1374428_at	296284	Kif3b_pred	kinesin family member 3B (predicted)	3285.863	5202.574	2260.041
1375686_at	301432	Ppil3	peptidylprolyl isomerase (cyclophilin)-li	3285.733	1662.459	907.5653
1370530_a_at	25096	Pld1	phospholipase D1	3285.187	41.85617	2492.111
1387131_at	116459	Serpini1	serine (or cysteine) peptidase inhibitor	3283.217	3713.806	4576.182
1398995_at	290997	RGD13070	similar to retinoid x receptor interacting	3282.866	5372.511	4061.314
1372932_at	309674	Nnp1	novel nuclear protein 1	3281.754	2610.765	2364.673
1390699_at	309307	RGD13115	similar to KIAA2026 protein	3280.826	8238.747	18307.94
1371516_at	361070	Xpo7	exportin 7	3279.353	2249.227	4811.715
1387045_at	29757	Atp6v0a1	ATPase, H+ transporting, lysosomal V	3274.11	2261.531	2197.31
1371608_at	287126	Mrps34_pr	mitochondrial ribosomal protein S34 (p	3273.361	5129.599	1425.208
1390218_at	362594	RGD13104	similar to hypothetical protein (predicte	3273.333	8452.864	3319.666
1372713_at	299295	RGD13095	similar to hypothetical protein D12Ertd	3272.744	3445.752	5975.618
1382261_at	287822	RGD13114	similar to CG8841-PA (predicted)	3270.817	3312.061	764.3951
1367915_at	84497	Dgat1	diacylglycerol O-acyltransferase 1	3268.452	2026.246	863.4789
1389406_at	369018	Ldhal6b	lactate dehydrogenase A-like 6B	3265.831	1928.061	1757.968
1379360_at	360821	RGD13096	similar to Putative protein 15E1.2 (pre	3265.364	2005.138	565.317
1389156_at	498606	LOC49860	hypothetical protein LOC498606	3263.165	1477.894	1600.248
1378098_at	302913	RGD13097	similar to CG4768-PA (predicted)	3256.993	3246.838	27424.24
1382699_s_at	114638	Hps1	Hermansky-Pudlak syndrome 1 homol	3255.792	2632.884	1185.011
1370287_a_at	24851	Tpm1	tropomyosin 1, alpha	3255.399	1425.106	6739.32
1379254_at	289034	RGD13098	similar to RIKEN cDNA 1300007B12; c	3249.312	5754.378	4843.251
1387908_at	64455	Rasd1	RAS, dexamethasone-induced 1	3248.668	2710.154	1292.003
1368350_at	25613	Ptprz1	protein tyrosine phosphatase, receptor	3247.691	5122.27	1973.136
1389416_at	498192	RGD15603	RGD1560398 (predicted)	3244.872	2621.21	1609.404
1373758_at	113917	Lenep	lens epithelial protein	3244.193	1155.093	694.1444
1389975_at	298359	RGD15619	similar to ELAV (embryonic lethal, abn	3242.925	2260.956	1876.281
1368055_a_at	60374	Lmna	lamin A	3236.354	2167.475	8040.51
1372914_at	297604	Ltbr	lymphotoxin B receptor	3233.161	34.32907	1692.841
1367935_at	117541	Smu1	smu-1 suppressor of mec-8 and unc-5	3229.466	6508.34	2620.657
1372732_at	311422	Itpa_mapp	inosine triphosphatase (nucleoside trip	3229.25	5196.811	2221.201

1372429_at	307798	LOC30779	similar to 24432 protein	3219.957	1925.991	3161.944
1375173_at	246120	LOC24612	RDCR-0918-3 protein	3219.718	3869.161	1888.401
1379013_at	296733	RGD13064	similar to mKIAA1402 protein (predicte	3219.408	1402.326	699.3464
1374538_at	362567	Pomgnt1	protein O-linked mannose beta1,2-N-a	3218.615	6036.252	868.9446
1371931_at	353256	Gtf2i	general transcription factor II I	3218.193	6835.91	4218.676
1377232_at	305332	RGD13052	similar to hypothetical protein (predicte	3217.661	2096.371	555.6575
1372453_at	289881	Dr1	down-regulator of transcription 1	3217.334	4385.791	4608.458
1369735_at	58935	Gas6	growth arrest specific 6	3216.882	12172.09	714.216
1367502_at	309140	Mrpl21_pre	mitochondrial ribosomal protein L21 (p	3214.737	6876.684	1645
1372580_at	94193	Madd	MAP-kinase activating death domain	3213.912	4615.259	4173.961
1388171_at	171150	Cdk7	cyclin-dependent kinase 7 (homolog of	3212.096	3949.949	1050.376
1388438_at	361684	Nap1l4	nucleosome assembly protein 1-like 4	3210.619	5689.807	1758.898
1390692_at	313560	Ctps_predi	cytidine 5'-triphosphate synthase (prec	3208.705	4680.975	1407.016
1383250_at	317579	Utp14a	UTP14, U3 small nucleolar ribonucleo	3207.882	2640.446	1594.73
1372215_at	499185	RGD15599	similar to mitochondrial ribosomal prot	3202.183	4000.48	1328.004
1394435_at	295328	Vangl1_pre	vang, van gogh-like 1 (Drosophila) (pre	3199.097	1636.912	408.2845
1367688_at	65170	Scamp4	secretory carrier membrane protein 4	3193.941	2156.763	803.0471
1371863_at	303698	RGD13050	similar to phosphatidylglycerophospha	3193.674	2454.175	1639.713
1378079_at	312077	Golga3_pre	golgi autoantigen, golgin subfamily a, 3	3193.271	1382.46	1016.405
1379550_a_at	246770	Gtf2ird1	general transcription factor II I repeat d	3191.321	6176.392	5361.157
1389478_at	303792	Klhl22_pre	kelch-like 22 (Drosophila) (predicted)	3187.857	1051.416	1459.777
1372988_at	316034	Cnot10	CCR4-NOT transcription complex, sub	3187.21	1220.689	1854.489
1376986_at	361035	Mettl3	methyltransferase-like 3	3186.703	893.0181	3663.895
1376726_at	360599	NA	NA	3186.012	260.6343	51.73854
1392557_at	361832	Bicc1_prec	bicaudal C homolog 1 (Drosophila) (pr	3185.504	4714.26	5723.033
1375205_at	301164	Pcaf	p300/CBP-associated factor	3183.611	6085.983	7763.005
1381972_at	297498	Crbn	cereblon	3182.98	6424.483	5214.89
1387817_at	25247	Nsg1	neuron specific gene family member 1	3182.689	13916.29	1035.824
1387122_at	25157	Plagl1	pleiomorphic adenoma gene-like 1	3181.469	5.761906	41751.95
1374770_at	84431	Asah1	N-acylsphingosine amidohydrolase 1	3181.202	2420.42	6243.378
1370318_at	64161	Pik4ca	phosphatidylinositol 4-kinase, catalytic	3181.055	3982.842	3240.086
1373498_at	315600	Vps11_pre	vacuolar protein sorting 11 (yeast) (pre	3180.145	1260.124	1753.866
1367486_at	362162	Zfp289	zinc finger protein 289	3179.413	3404.185	1279.441
1377235_a_at	309179	Ppp2r5b	protein phosphatase 2, regulatory subu	3178.371	2543.556	1980.059
1390146_at	360916	RGD13061	similar to RIKEN cDNA 2610318G18 (	3177.191	1382.858	1281.431
1369060_a_at	84578	Hdac3	histone deacetylase 3	3176.596	4052.902	1206.97
1388938_at	297593	Usp5_pred	ubiquitin specific protease 5 (isopeptid	3176.422	3848.967	1243.611
1379402_at	170924	Abcc4	ATP-binding cassette, sub-family C (C	3174.617	1880.978	1145.316
1399019_at	364380	Abhd4_pre	abhydrolase domain containing 4 (prec	3174.081	2266.182	1336.995
1388426_at	78968	Srebf1	sterol regulatory element binding facto	3172.497	3849.321	1447.021
1377903_at	313934	LOC31393	NA	3167.891	3362.194	5980.198
1389432_at	406164	Pbx2	pre-B-cell leukemia transcription factor	3167.513	3173.809	5455.295
1372593_at	308362	Zfp110	zinc finger protein 110	3165.572	744.9044	4107.943
1372506_at	25634	G6pc	glucose-6-phosphatase, catalytic	3165.527	7879.219	1519.842
1375867_at	287441	Zbtb4_prec	zinc finger and BTB domain containing	3164.957	2929.214	7980.755
1391415_at	289809	LOC28980	putatative 28 kDa protein	3163.531	3740.02	3516.619
1375368_at	287115	RGD13077	similar to RIKEN cDNA 1700012G19 g	3162.981	4322.048	1650.468
1368871_at	116667	Map3k1	mitogen activated protein kinase kinas	3160.307	1027.066	979.6817
1375450_at	299114	Sdccag1	serologically defined colon cancer anti	3159.657	1475.554	6836.403
1371553_at	364656	Mrpl36_pre	mitochondrial ribosomal protein L36 (p	3158.145	6487.961	4603.473
1370364_at	246310	Arfgap1	ADP-ribosylation factor GTPase activa	3154.054	2223.397	1508.126

1382521_at	24398	Gls	glutaminase	3153.222	24085.18	24491.63
1374481_at	364183	Cno	cappuccino homolog (mouse)	3151.284	3522.349	3652.83
1393161_at	362537	Snapc3	small nuclear RNA activating complex,	3151.032	1816.3	2978.235
1372132_at	291394	Cndp2	CNDP dipeptidase 2 (metallopeptidase	3150.346	4359.155	1656.025
1389054_at	498368	LOC49836	similar to RIKEN cDNA 0610040J01	3145.415	2145.811	4534.794
1388470_at	300849	Fbxo9	F-box only protein 9	3144.84	1312.327	5342.448
1370939_at	25288	Acsl1	acyl-CoA synthetase long-chain family	3142.629	9189.998	3945.539
1368472_at	83466	Celsr3	cadherin EGF LAG seven-pass G-type	3141.95	1309.337	2783.931
1375864_at	313478	Cc2d1b	coiled-coil and C2 domain containing 1	3141.859	2540.906	1502.498
1388595_at	367172	Dhx30	DEAH (Asp-Glu-Ala-His) box polypepti	3141.765	1557.991	2558.969
1373852_at	305291	RGD13088	similar to SRD5A2L	3140.873	2572.047	1639.364
1392590_at	305156	Arhgap24	Rho GTPase activating protein 24	3140.504	1623.258	8295.575
1388925_at	306840	Sirt5	sirtuin 5 (silent mating type information	3138.306	3349.537	4847.218
1371807_at	298841	RGD13116	similar to apoptosis related protein APl	3137.563	6573.76	2222.038
1398815_at	24206	Apeh	acylpeptide hydrolase	3137.336	2287.953	1363.075
1371586_at	293149	Mrpl48_pre	mitochondrial ribosomal protein L48 (p	3137.076	5910.202	2405.543
1393388_at	301081	Zdhhc3	zinc finger, DHHC domain containing 3	3136.722	2326.452	1013.704
1371389_at	306766	LOC30676	hypothetical LOC306766	3132.929	5077.672	9177.023
1367947_at	25520	Prkag1	protein kinase, AMP-activated, gamma	3131.653	3556.506	1970.01
1390628_at	360949	Cpeb2_pre	cytoplasmic polyadenylation element b	3130.438	4049.349	5331.434
1388634_at	24645	Pgm1	phosphoglucomutase 1	3128.807	4805.482	2021.377
1390217_at	56785	Garnl1	GTPase activating RANGAP domain-li	3128.745	1576.877	7699.754
1375357_at	266606	Dyt1	dystonia 1	3124.317	4989.063	1017.535
1389567_at	301024	Scap_pred	SREBP cleavage activating protein (pr	3122.766	1285.08	769.0181
1369182_at	25584	F3	coagulation factor III	3119.861	358.4348	2045.704
1370322_at	286927	Stk16	serine/threonine kinase 16	3119.296	3330.917	2138.866
1372854_at	311224	Ttc17	tetratricopeptide repeat domain 17	3118.515	1766.364	1407.43
1368004_at	64360	Mrpl23	mitochondrial ribosomal protein L23	3117.805	12660.42	3284.483
1383355_at	313210	Abca1	ATP-binding cassette, sub-family A (Al	3114.249	2554.771	2380.702
1369956_at	116465	Ifngr	interferon gamma receptor 1	3113.386	3279.355	2759.444
1389665_at	361196	Habp4_pre	hyaluronic acid binding protein 4 (pred	3111.027	3271.999	826.0451
1388739_at	314399	RGD13591	similar to chromosome 14 open readin	3110.99	6907.002	3732.813
1389317_at	311405	RGD13097	similar to hypothetical protein FLJ2050	3109.986	3111.377	1706.746
1398334_a_at	295956	Commd9	COMM domain containing 9	3109.974	5348.895	3910.156
1375910_at	313838	Cdc42ep3_	CDC42 effector protein (Rho GTPase	3109.67	1639.788	5295.483
1370820_at	192351	Fbxo6b	F-box only protein 6b	3109.466	2177.398	2376.178
1388847_at	360743	Pcqap_pre	positive cofactor 2, multiprotein comple	3107.147	1869.135	2450.483
1375259_at	361845	Eif4ebp2	eukaryotic translation initiation factor 4	3105.818	5519.456	2795.83
1382093_at	289382	RGD13080	similar to DKFZP434B168 protein (pre	3103.166	3892.888	1198.911
1397824_at	307029	RGD15624	similar to WAC (predicted)	3097.378	4949.153	1237.259
1373534_at	297942	RGD13073	similar to SR rich protein	3095.092	2156.404	8228.395
1387299_at	83422	Miz1	Msx-interacting-zinc finger	3093.038	9190.611	2606.311
1374835_at	266772	Klhl12	kelch-like 12 (Drosophila)	3093.017	3160.875	3220.314
1373730_at	362297	RGD13106	similar to hypothetical protein MGC204	3089.366	1945.039	3276.673
1391581_at	299012	Arhgap5	Rho GTPase activating protein 5	3088.735	723.3954	1786.917
1372203_at	260323	Snx27	sorting nexin family member 27	3088.363	2631.206	2253.858
1388430_at	292888	Ptov1	prostate tumor over expressed gene 1	3088.211	2241.59	2403.551
1374502_at	289350	Rab3gap2	RAB3 GTPase activating protein subui	3088.186	1010.701	1994.095
1377769_at	360785	Ap1s1_pre	adaptor protein complex AP-1, sigma 1	3088.09	8721.524	681.8568
1373806_at	289859	RGD13117	similar to hypothetical protein MGC154	3086.725	4814.754	5957.003
1387860_at	29154	Capn2	calpain 2	3082.734	1679.667	6661.857

1393214_at	499562	RGD15661	similar to hypothetical protein A230042	3082.347	2897.749	3228.118
1380570_at	56768	Pclo	piccolo (presynaptic cytomatrix protein	3079.699	1933.32	1996.956
1376573_at	360571	Rab34	RAB34, member of RAS oncogene far	3078.646	1759.531	1008.136
1387218_at	25563	Tff3	trefoil factor 3	3075.286	7552.83	179.6855
1374582_at	364410	Kctd9_prec	potassium channel tetramerisation dor	3073.762	2048.605	2001.943
1379312_at	294007	Pprc1_prec	peroxisome proliferative activated rece	3073.24	2181.454	993.7181
1371632_at	501841	RGD15644	similar to Coronin, actin binding protein	3072.053	7057.64	3752.746
1398996_at	308765	RGD1311C	hypothetical LOC308765 (predicted)	3070.45	5505.345	1222.809
1386957_at	113975	Pom121	nuclear pore membrane glycoprotein 1	3069.777	2494.734	3347.701
1382275_at	361232	MGC12501	similar to PAK/PLC-interacting protein	3064.716	3771.526	1857.937
1372177_at	294753	Mocs2	molybdenum cofactor synthesis 2	3064.47	4566.517	3266.323
1389729_at	288652	MGC94142	similar to cDNA sequence BC003324	3061.751	2489.608	3910.971
1369203_at	114557	Wif1	Wnt inhibitory factor 1	3061.107	13832.63	37518.36
1373768_at	362426	Bms1l	BMS1-like, ribosome assembly protein	3058.706	3078.032	3429.813
1374597_at	498733	NA	NA	3057.895	3724.239	2131.417
1370873_at	24244	Calm3	calmodulin 3	3057.715	6849.918	1525.005
1375042_at	293129	Alg8	asparagine-linked glycosylation 8 hom	3056.849	5264.701	2967.877
1368076_at	24874	Vhl	von Hippel-Lindau syndrome homolog	3056.365	3304.098	2758.577
1374711_at	298916	Cpsf3	cleavage and polyadenylation specifici	3054.958	4932.549	2662.307
1374953_at	500420	LOC50042	similar to CG12279-PA	3053.765	2544.429	2889.075
1389116_at	282584	Mtmr9	myotubularin related protein 9	3052.667	1186.055	884.0949
1399029_at	362636	Usp48	ubiquitin specific protease 48	3049.9	2334.129	4617.684
1373186_at	305310	RGD1311E	similar to RIKEN cDNA 5033405K12 (p	3048.824	3573.886	4354.788
1391459_at	367747	RGD15647	similar to nudix (nucleoside diphospha	3047.349	8080.734	10547.89
1372823_at	289278	RGD13091	similar to RIKEN cDNA 2310005N03 g	3045.719	1016.621	6080.997
1373904_at	300839	Lysmd2_pr	LysM, putative peptidoglycan-binding,	3045.714	1229.809	4382.724
1376016_at	288518	RGD1311E	similar to RIKEN cDNA 1110007L15 (p	3045.187	5355.43	5358.081
1382492_a_at	361802	Hsd17b8	hydroxysteroid (17-beta) dehydrogena	3045.067	1625.472	2785.251
1367515_at	306492	Cnot7_prec	CCR4-NOT transcription complex, sub	3043.457	4170.714	13982.55
1379717_at	360997	LOC36099	NA	3042.906	1496.41	2097.873
1393110_at	170551	Slc5a6	solute carrier family 5 (sodium-depend	3042.582	2608.156	3905.799
1372206_at	364298	RGD13074	similar to chromosome 14 open readin	3040.162	1918.623	2213.567
1388910_at	305493	Mrps24_pr	mitochondrial ribosomal protein S24 (p	3039.664	1341.625	2525.035
1373619_at	361183	Ankrd10	ankyrin repeat domain 10	3038.203	5060.525	4730.63
1367735_at	25287	Acadl	acetyl-Coenzyme A dehydrogenase, lo	3036.865	3124.133	2962.211
1386910_a_at	79116	Apex1	apurinic/apyrimidinic endonuclease 1	3036.102	4549.913	2348.456
1377061_at	315530	RICS_pred	RhoGAP involved in beta-catenin-N-ca	3035.7	1161.278	1158.538
1372348_at	500244	RGD15635	similar to AP2 associated kinase 1 (pre	3035.353	1604.357	1207.475
1389135_at	619580	Ctps2	cytidine triphosphate synthase II	3034.045	3729.385	3850.717
1374402_at	27137	Eif2ak1	eukaryotic translation initiation factor 2	3033.282	2698.42	1875.91
1371510_at	287097	Hcfc1r1	host cell factor C1 regulator 1 (XPO1-c	3031.209	7092.701	4036.286
1392610_at	362501	Topors_pre	topoisomerase I binding, arginine/serin	3030.911	4473.597	8938.905
1384427_at	314856	Mdm2_pre	transformed mouse 3T3 cell double mi	3030.494	2717.1	3385.844
1377701_at	362602	RGD15618	similar to RIKEN cDNA 9330177P20 (p	3028.091	1926.185	3615.401
1379377_at	362004	Gdap2	ganglioside-induced differentiation-ass	3023.466	2383.027	1727.897
1388570_at	498909	LOC49890	NA	3022.637	2707.175	1916.91
1372676_at	302980	Fahd1	fumarylacetoacetate hydrolase domain	3021.105	4971.652	4481.185
1387176_at	171123	Rph3al	rabphilin 3A-like (without C2 domains)	3019.768	2922.92	4661.285
1386856_a_at	308473	Samd4b	sterile alpha motif domain containing 4	3019.315	1558.369	4093.22
1368040_at	64531	Synj2bp	synaptojanin 2 binding protein	3018.582	1944.258	1834.99
1370357_at	64469	Slc30a4	solute carrier family 30 (zinc transporte	3018.412	4272.855	3453.644

1368274_at	83527	Dbnl	drebrin-like	3018.209	3604.238	1961.496
1372412_at	362667	Thap3_pre	THAP domain containing, apoptosis as	3018.019	828.9033	2046.579
1398531_at	315439	Slc36a4_pr	solute carrier family 36 (proton/amino a	3016.888	4711.368	8093.805
1371947_at	308690	Ndn	necdin	3015.101	99.31294	5863.916
1373448_at	299203	Acyp1_pre	acylphosphatase 1, erythrocyte (comm	3014.845	2704.711	2195.777
1379304_at	498171	LOC49817	NA	3012.949	1945.241	1827.975
1376274_at	308890	Btbd10	BTB (POZ) domain containing 10	3011.103	2880.626	3998.908
1388886_at	366693	Rbm25_pre	RNA binding motif protein 25 (predicte	3010.233	1968.62	3926.398
1390131_at	303306	Srr	serine racemase	3007.32	4710.792	1969.874
1388878_at	291996	Nob1p	nin one binding protein	3005.913	1933.18	1548.043
1396947_at	286994	Lgr4	leucine-rich repeat-containing G protei	3004.706	257.0085	691.1853
1374170_at	498749	LOC49874	similar to putative TRAF and TNF rece	3004.181	1444.132	6263.362
1376025_at	499420	Hrmt1l1	HMT1 hnRNP methyltransferase-like 1	3002.731	4541.539	1599.965
1370062_at	140937	Hig1	hypoxia induced gene 1	3002.234	6543.615	28850.27
1371916_at	685059	MGC10575	NA	3000.807	3717.478	1404.026
1372222_at	313493	RGD13117	similar to hypothetical protein F730001	2999.881	3125.345	2063.042
1370814_at	266686	Dhrs4	dehydrogenase/reductase (SDR family	2998.91	3954.82	847.3279
1379340_at	192362	Lamc2	lamimin, gamma 2	2998.256	49.51878	2003.068
1368863_at	85269	Nme3	non-metastatic cell expressed protein 3	2996.118	6666.01	3770.153
1372304_at	363068	Commd4_p	COMM domain containing 4 (predicted	2995.669	3371.639	1107.242
1389013_at	317275	RGD13084	similar to RIKEN cDNA 1500016H10 (p	2989.486	5417.332	1486.435
1374009_at	362320	RGD13063	similar to RIKEN cDNA 5830415L20	2988.368	5060.122	7191.833
1372377_at	314336	Ddx41_pre	DEAD (Asp-Glu-Ala-Asp) box polypept	2988.263	7511.649	2545.608
1373428_at	293668	Brms1	breast cancer metastasis-suppressor 1	2988.071	2443.502	1428.883
1375019_at	361838	Hnrph3_pre	heterogeneous nuclear ribonucleoprote	2986.008	4750.434	10853.31
1374414_at	363252	RGD13068	similar to RIKEN cDNA 1300010M03	2985.615	2564.595	1558.4
1371920_at	287544	Poldip2_pre	polymerase (DNA-directed), delta inter	2984.166	2612.245	1164.799
1398915_at	360776	RGD13097	similar to CG14977-PA (predicted)	2982.27	3496.853	2127.089
1368185_at	25740	Gnaz	guanine nucleotide binding protein, alp	2979.872	4439.298	3864.826
1387032_at	25298	Cck	cholecystokinin	2978.067	2553.96	2092.835
1398455_at	365252	Zdhhc13	zinc finger, DHHC domain containing 1	2976.048	5445.332	2480.465
1392477_at	362733	Etv1_predi	ets variant gene 1 (predicted)	2974.681	23437.16	32706.86
1392958_at	498035	NA	NA	2974.603	2440.224	4549.494
1368100_at	89841	Pcyt2	phosphate cytidylyltransferase 2, ethar	2972.343	2200.951	1265.667
1372420_at	290645	RGD13080	similar to FKSG24 (predicted)	2972.076	3012.87	935.9984
1392599_at	302678	Syap1	synapse associated protein 1	2967.222	2159.862	5075.093
1388347_at	362934	LOC36293	similar to lymphocyte antigen 6 comple	2966.058	8713.043	12248.43
1388993_at	282845	Rnf34	ring finger protein 34	2963.861	2334.233	1584.025
1369220_at	114114	Dnm1l	dynamin 1-like	2959.618	6855.337	1113.786
1382818_at	316415	RGD13097	similar to hypothetical protein D630010	2959.327	1116.379	5807.381
1372419_at	361565	Vrk3	vaccinia related kinase 3	2955.174	3443.883	1753.784
1372869_at	114300	Gtpbp4	GTP binding protein 4	2953.816	4303.741	2410.256
1373640_at	364064	Pycr2	pyrroline-5-carboxylate reductase fami	2953.08	3038.927	1544.742
1375545_at	362950	Rbm9_pre	RNA binding motif protein 9 (predicted	2950.868	3437.453	416.212
1377254_a_at	315036	Cohh1_pre	Cohen syndrome homolog 1 (predicted	2950.818	1271.073	9960.492
1389574_at	313914	Gtf3c2	general transcription factor IIIC, polype	2950.014	2577.909	2963.755
1373454_at	360461	RGD13058	similar to RIKEN cDNA 0610037P05	2949.604	6145.835	4275.284
1389390_at	500005	LOC50000	similar to ODAG protein	2947.467	5827.508	2311.163
1383155_at	363236	Als2cr13_p	amyotrophic lateral sclerosis 2 (juvenil	2946.624	1482.023	6267.901
1371503_at	287042	Nubp1	nucleotide binding protein 1	2944.983	5066.119	2097.756
1372254_at	295703	Serping1	serine (or cysteine) peptidase inhibitor	2943.074	374.0599	17722.59

1372409_at	316237	Mad2l1bp	MAD2L1 binding protein	2940.558	3998.648	2014.43
1389647_at	362731	Snx13_pre	sorting nexin 13 (predicted)	2940.026	2391.687	5794.16
1395533_at	307492	Dnd1	dead end homolog 1 (zebrafish)	2938.254	4101.109	609.4638
1383287_at	654496	Fubp1	NA	2936.895	1456.935	3039.825
1398425_at	500400	MGC1091	similar to hypothetical protein MGC393	2934.178	1279.942	519.8738
1369086_a_at	29716	Cacna1d	calcium channel, voltage-dependent, L	2933.975	1192.861	2254.491
1373364_at	298573	Eif4g3_pre	eukaryotic translation initiation factor 4	2933.263	1291.272	4680.625
1379526_at	24547	Mbp	myelin basic protein	2931.161	7550.28	9855.118
1375061_at	361449	Ppil4_pred	peptidylprolyl isomerase (cyclophilin)-li	2930.108	3240.653	6037.938
1377089_a_at	362048	Tspan5	tetraspanin 5	2929.949	2996.994	7364.029
1368291_at	60371	Birc2	baculoviral IAP repeat-containing 2	2929.511	2007.916	6801.946
1371002_at	58934	Pdcd2	programmed cell death 2	2929.167	2357.686	2873.262
1389008_at	305539	Spred2	sprouty-related, EVH1 domain contain	2927.468	3159.674	8309.227
1374299_at	304859	Dhx9_pred	DEAH (Asp-Glu-Ala-His) box polypepti	2927.163	2429.087	8989.411
1397223_at	500824	RGD15656	similar to FYVE, RhoGEF and PH dom	2926.868	254.7206	501.0783
1374109_at	305622	Dgkq_pred	diacylglycerol kinase, theta (predicted)	2926.698	2052.863	1852.201
1387175_a_at	94342	Bat3	HLA-B-associated transcript 3	2925.714	3454.112	1968.509
1382139_at	315804	RGD13087	similar to hypothetical protein FLJ1299	2925.266	3397.703	7412.778
1373052_at	316348	Pdcl3	phosducin-like 3	2923.534	11243.63	6427.6
1391714_at	297804	Plag1	pleiomorphic adenoma gene 1	2922.725	162.9846	1336.726
1375929_at	287521	Mnt_predic	max binding protein (predicted)	2922.285	1188.675	11374.74
1371754_at	246771	Slc25a25	solute carrier family 25 (mitochondrial	2922.212	2278.983	8859.1
1389963_at	652956	LOC65295	NA	2921.983	2262.712	1560.688
1388067_a_at	83635	Gmeb2	glucocorticoid modulatory element bin	2919.396	2812.647	4300.908
1398962_at	299617	RGD13596	uncharacterized protein family UPF022	2919.067	2168.209	1199.004
1371833_at	304284	Bri3	brain protein I3	2917.971	3049.267	3246.371
1392835_at	304337	Zfp68_pre	zinc finger protein 68 (predicted)	2916.068	7740.022	5243.334
1398606_at	310526	Golph4	golgi phosphoprotein 4	2915.72	2949.169	8638.264
1374956_at	81740	Pcm1	pericentriolar material 1	2913.893	6584.402	8562.048
1377703_at	309419	Pip5k1a	phosphatidylinositol-4-phosphate 5-kin	2912.587	2909.544	5253.73
1392514_at	294436	Bxdc1_pre	brix domain containing 1 (predicted)	2911.239	3209.437	1586.256
1377961_at	307829	RGD15602	similar to AT motif-binding factor (pred	2909.179	451.8048	2688.566
1377844_at	299194	Zadh1	zinc binding alcohol dehydrogenase, d	2907.235	683.1353	4319.118
1380979_a_at	361004	RGD13094	similar to KIAA0913 protein (predicted)	2906.2	2163.336	1531.414
1373492_at	361726	RGD13092	similar to hypothetical protein FLJ2048	2900.173	4477.077	1515.99
1373068_at	300994	Ifrd2_predi	interferon-related developmental regul	2897.185	1156.853	1165.367
1376038_at	303611	Tex2	testis expressed gene 2	2895.486	4629.685	1651.119
1373681_at	300741	Mpi_mappe	mannose phosphate isomerase (mapp	2894.538	1443.258	1262.912
1379595_at	296761	Tmem60_p	transmembrane protein 60 (predicted)	2894.326	5556.524	1740.803
1389066_at	140666	Dscr1l1	Down syndrome critical region gene 1-	2894.176	2880.014	4616.32
1387058_at	29510	Pctp	phosphatidylcholine transfer protein	2893.935	2014.832	742.0516
1387801_at	171121	Ppp6c	protein phosphatase 6, catalytic subun	2893.084	4284.695	3278.791
1368064_a_at	24311	Ddc	dopa decarboxylase	2892.281	8713.348	4860.398
1373047_at	84006	Prkci	protein kinase C, iota	2889.631	2201.064	4026.377
1388829_at	361399	LOC36139	similar to autoantigen	2889.038	1373.46	2906.571
1389705_at	295245	Rag1ap1_p	recombination activating gene 1 activa	2887.145	2352.678	3261.541
1371563_at	298594	Rcc2_pred	regulator of chromosome condensatio	2887.03	1873.821	1722.271
1370236_at	29411	Ppt1	palmitoyl-protein thioesterase 1	2885.845	5241.711	5078.251
1392960_at	287406	RGD13099	similar to RIKEN cDNA 2310004I24 ge	2885.554	2337.241	2782.342
1374567_at	314836	Psrc2	proline/serine-rich coiled-coil 2	2883.756	2065.584	2686.367
1388305_at	64363	Araf	v-raf murine sarcoma 3611 viral oncog	2883.353	1656.722	6440.79

1393352_at	300836	RGD13105	similar to hypothetical protein MGC389	2882.374	696.9893	11984.48
1373461_at	287446	DERP6	dermal papilla derived protein 6	2881.421	4200.171	3744.047
1373687_at	362963	Rutbc3	RUN and TBC1 domain containing 3	2880.718	1249.289	1007.346
1382264_at	54244	Crebbp	CREB binding protein	2879.142	2346.386	5504.931
1387060_at	58954	Klf6	Kruppel-like factor 6	2878.636	3371.293	5116.956
1372346_at	313913	Zfp513	zinc finger protein 513	2878.58	2106.071	1804.615
1372803_at	290529	RGD13056	similar to DNA segment, Chr 14, ERAT	2878.138	7583.607	1313.618
1376523_at	314205	Arid4a_pre	AT rich interactive domain 4A (Rbp1 lik	2877.733	752.4633	1309.935
1370893_at	60581	Acaca	acetyl-coenzyme A carboxylase alpha	2873.901	6352.421	3117.727
1379419_at	291946	Tmem34	transmembrane protein 34	2873.169	4206.874	5071.689
1368056_at	24855	Tsc2	tuberous sclerosis 2	2872.67	2408.169	2000.683
1383782_at	362700	Spast_prec	spastin (predicted)	2872.326	3869.041	2694.63
1390500_at	305544	RGD13119	similar to RIKEN cDNA 9130023F12 g	2868.471	3203.316	2637.231
1397916_s_at	24697	Ptpn1	protein tyrosine phosphatase, non-rece	2868.443	1821.489	675.3389
1390839_at	298906	Pqlc3	PQ loop repeat containing 3	2867.253	5437.676	2456.522
1371814_at	294331	Ube2g2_pr	ubiquitin-conjugating enzyme E2G 2 (p	2866.152	2823.864	1691.195
1373125_at	309159	Klc2_predi	kinesin light chain 2 (predicted)	2865.811	2395.159	1578.709
1372867_at	291534	Rnmt	RNA (guanine-7-) methyltransferase	2862.061	5672.746	7153.69
1392511_at	170844	Taf2	TAF2 RNA polymerase II, TATA box b	2861.267	4962.691	6031.508
1379603_at	500985	RGD15613	similar to CBL E3 ubiquitin protein liga	2860.876	833.8946	681.6845
1384399_at	501082	NA	NA	2860.272	3618.528	1151.066
1392987_at	289717	RGD15616	similar to hypothetical protein (predicte	2859.887	3536.921	3017.747
1370081_a_at	83785	Vegfa	vascular endothelial growth factor A	2859.369	3186.42	1128.675
1390527_at	500795	RGD15621	RGD1562114 (predicted)	2859.331	3033.492	1401.444
1377860_at	298185	RGD13069	similar to bM410K19.2.2 (novel protein	2856.792	505.4017	786.7364
1373430_at	317627	Baz2b_pre	bromodomain adjacent to zinc finger d	2856.141	2147.802	7204.037
1383572_at	361771	Zdhhc6	zinc finger, DHHC domain containing 6	2854.607	1878.94	3085.702
1372718_at	300963	LOC30096	similar to centrosome protein Cep63	2854.132	1984.651	5841.061
1387038_at	84485	Ccs	copper chaperone for superoxide dism	2854.034	4464.633	1884.134
1372396_at	363029	RGD13080	similar to 2310047B19Rik protein (pred	2853.527	2185.931	2199.671
1399152_at	313474	Eps15	epidermal growth factor receptor pathw	2851.1	1024.269	500.886
1387105_at	171110	Zfp422	zinc finger protein 422	2851.061	5873.67	5490.095
1367865_at	170946	Lkap	limkain b1	2850.713	1823.282	946.2888
1374156_at	314259	Mpp5_prec	membrane protein, palmitoylated 5 (MA	2848.848	1143.462	2603.92
1372020_at	361370	Tom1	target of myb1 homolog (chicken)	2846.878	2551.959	733.2203
1382420_at	296145	RGD13101	similar to hypothetical protein FLJ3744	2846.496	2803.078	7274.855
1388756_at	298490	Ppcs	phosphopantothenoylcysteine syntheta	2844.927	2618.706	838.6765
1375217_at	154968	Mgea5	meningioma expressed antigen 5 (hya	2844.657	1233.923	2789.957
1382196_at	360667	RGD13073	similar to hypothetical protein ET (pred	2842.98	1602.276	412.401
1370808_at	25035	Cyb5r3	cytochrome b5 reductase 3	2840.162	9044.783	4486.503
1399111_at	363137	Cyb561d2	cytochrome b-561 domain containing 2	2840.045	2307.055	361.8429
1390155_at	305795	Abhd6	abhydrolase domain containing 6	2839.575	1128.888	4829.803
1382105_at	83579	Gnb5	guanine nucleotide binding protein, bet	2838.465	1915.338	4892.614
1383633_at	259221	Osbpl1a	oxysterol binding protein-like 1A	2834.929	3997.412	3493.645
1389514_at	315691	Lrrn6a	leucine rich repeat neuronal 6A	2834.884	6947.391	3057.18
1368726_a_at	170902	Zfp347	zinc finger protein 347	2833.854	1538.664	3878.495
1367655_at	50665	Tmsb10	thymosin, beta 10	2832.821	6802.274	4499.406
1367853_at	83629	Slc12a2	solute carrier family 12, member 2	2832.429	6661.244	2675.153
1374301_at	303652	Cog1_pred	component of oligomeric golgi complex	2829.91	3440.677	3397.432
1373455_at	361531	Paf1	Paf1, RNA polymerase II associated fa	2826.736	1936.068	6876.38
1381685_a_at	287044	RGD13057	similar to CG12753-PA (predicted)	2826.188	3354.39	1865.448

1388182_at	246327	Prim1	DNA primase, p49 subunit	2825.129	25154.22	1511.766
1388744_at	288532	Mcm7	minichromosome maintenance deficien	2822.346	11677.72	1942.822
1398612_at	364773	LOC36477	similar to liver regeneration-related pro	2819.016	2593.245	2924.551
1368225_at	171455	Exoc2	exocyst complex component 2	2817.653	3116.354	3845.685
1375936_at	291760	Dsc2	desmocollin 2	2815.637	4382.213	3163.439
1377952_at	315722	Adpgk	ADP-dependent glucokinase	2815.386	413.4711	311.4474
1392506_at	288256	Cryzl1	crystallin, zeta (quinone reductase)-like	2814.991	3807.487	3648.7
1369989_at	64533	Pnpo	pyridoxine 5'-phosphate oxidase	2814.369	625.9906	1310.528
1398912_at	310653	mrpl9	mitochondrial ribosomal protein L9	2813.36	3813.27	3522.869
1375918_at	497967	LOC49796	similar to RIKEN cDNA 2410003C20	2811.097	2411.353	3182.265
1373121_at	361919	Mrps28	mitochondrial ribosomal protein S28	2809.372	5241.356	1672.416
1372353_at	288526	LOC28852	similar to DNA segment on chromosom	2808.17	2477.703	3845.424
1369414_at	114095	Stxbp3	syntaxin binding protein 3	2806.686	2018.765	3279.896
1376315_at	296304	RGD1303C	similar to putative alpha-mannosidase	2806.531	2203.56	613.7726
1397628_at	293500	RGD13055	similar to RIKEN cDNA 2900092E17	2806.309	1342.421	1292.006
1392717_at	117548	Kif1b	kinesin family member 1B	2803.901	632.1441	5216.257
1373837_at	291906	Fts	fused toes	2803.474	1841.756	1996.738
1374288_at	303608	Ftsj3	FtsJ homolog 3 (E. coli)	2799.477	1840.363	1397.015
1370231_at	171063	Gtf3c1	general transcription factor III C 1	2798.794	2669.585	2626.575
1369979_at	155183	Scap2	src family associated phosphoprotein 2	2797.763	6782.725	1558.016
1389052_at	292095	Ttc13	tetratricopeptide repeat domain 13	2797.423	2175.474	862.1785
1390307_at	498430	RGD15654	similar to echinoderm microtubule asso	2796.842	586.7146	227.6692
1375068_at	499762	RGD15648	similar to surfeit 5 isoform b (predicted	2795.804	1873.394	2525.164
1372316_at	313860	RGD13119	similar to AI115348 protein (predicted)	2795.771	1849.921	1139.568
1371535_at	302915	Pmm2_pre	phosphomannomutase 2 (predicted)	2794.847	1140.164	867.977
1367869_at	117520	Oxr1	oxidation resistance 1	2794.748	11026.17	11998.09
1388463_at	300988	Tex264	testis expressed gene 264 homolog (m	2794.666	793.4547	578.1406
1391252_at	362814	Rnf41	ring finger protein 41	2793.125	1734.945	1271.929
1374443_at	303673	Ict1_predic	immature colon carcinoma transcript 1	2790.224	3868.441	2576.841
1375554_at	297685	Ddx47	DEAD (Asp-Glu-Ala-Asp) box polypept	2787.928	5973.129	2049.79
1393094_at	498883	NA	NA	2786.876	969.2047	987.2122
1393346_at	499518	RGD15616	similar to RIKEN cDNA 5830436D01 (p	2785.655	158.3458	5685.714
1376609_at	291750	RGD13091	similar to TRS85 homolog (predicted)	2785.53	4660.963	5786.705
1382325_at	366959	Gcat	glycine C-acetyltransferase (2-amino-3	2785.015	1082.433	245.8956
1374651_at	304077	Dopey2_pr	dopey family member 2 (predicted)	2784.493	2217.142	1181.079
1389307_at	29572	Aplp1	amyloid beta (A4) precursor-like protei	2783.548	6093.605	5832.103
1392676_at	308326	Mip1	myocardial ischemic preconditioning u	2779.447	986.0133	2665.465
1367576_at	24404	Gpx1	glutathione peroxidase 1	2774.603	47.05274	6186.944
1376091_at	315150	Adsl_predi	adenylosuccinate lyase (predicted)	2773.991	3213.938	1723.238
1372194_at	360540	Akap10_pr	A kinase (PRKA) anchor protein 10 (pr	2773.184	3079.461	1247.694
1392454_at	292160	RGD13087	similar to hypothetical protein FLJ2072	2771.744	1503.603	3062.644
1375163_at	79434	Rab11b	RAB11B, member RAS oncogene fam	2771.149	6885.975	5846.836
1382797_at	296750	RGD15604	similar to 1500019C06Rik protein (pre	2770.984	658.5234	641.2108
1398663_at	499094	LOC49909	similar to zinc finger protein 61	2769.619	1309.438	5621.125
1373769_at	289632	Pgm1_pre	phosphoglucomutase 1 (predicted)	2769.01	3620.85	3811.343
1390827_at	25631	Smad3	MAD homolog 3 (Drosophila)	2768.1	2321.451	2086.465
1394392_at	311617	RGD13076	similar to dJ881L22.2 (novel protein) (p	2765.048	2115.025	1462.886
1372949_at	305227	Sept6_pre	septin 6 (predicted)	2762.895	2014.142	1633.363
1393218_at	314415	RGD13048	similar to 2410024A21Rik protein	2762.873	1913.314	5537.513
1371914_at	361825	Smarcb1	SWI/SNF related, matrix associated, a	2762.622	3786.134	1358.911
1367621_at	64391	Dapk3	death-associated protein kinase 3	2761.59	3193.194	1294.841

1388468_at	499672	Cdc42se1	CDC42 small effector 1	2761.488	2242.116	1732.454
1389174_s_at	288533	Taf6	TAF6 RNA polymerase II, TATA box b	2761.027	2628.775	2320.609
1389450_at	360830	LOC36083	NA	2760.204	3143.03	1577.331
1373964_at	364878	3-Mar	membrane-associated ring finger (C3H	2756.436	2600.658	2411.129
1382630_at	292139	RGD13115	similar to 4930506M07Rik protein (pre	2756.295	2291.213	852.2309
1389238_at	289740	LOC28974	similar to PES1 protein	2756.16	1836.744	1676.131
1398808_at	83523	Impa1	Inositol (myo)-1(or 4)-monophosphatas	2755.34	5088.078	2633.788
1370066_at	117519	Keap1	Kelch-like ECH-associated protein 1	2754.077	1527.514	1624.382
1391577_at	288731	Pgam5	phosphoglycerate mutase family meml	2753.604	3248.736	1729.304
1398395_at	298914	Itgb1bp1_p	integrin beta 1 binding protein 1 (predi	2753.387	4221.974	2030.567
1388897_at	362093	Wdr5	WD repeat domain 5	2753.181	3375.477	1452.528
1389252_at	64619	Zfp238	zinc finger protein 238	2752.973	1624.529	2337.908
1381259_at	315958	Stag1_pre	stromal antigen 1 (predicted)	2751.527	3000.391	3425.98
1372628_at	366618	Ap4s1_pre	adaptor-related protein complex AP-4,	2747.679	2169.223	2154.843
1398901_at	81738	Nup98	nucleoporin 98	2745.719	2127.073	1062.004
1387062_a_at	140583	Chek1	checkpoint kinase 1 homolog (S. pomb	2744.668	2648.37	1332.165
1387057_at	84551	Slc7a8	solute carrier family 7 (cationic amino a	2743.568	1091.21	1379.244
1372629_at	300768	LOC30076	similar to KIAA0925 protein	2742.331	1447.186	2219.76
1370326_at	81741	Pctk1	PCTAIRE-motif protein kinase 1	2742.011	2371.308	1699.774
1382076_at	294321	Slc37a1	solute carrier family 37 (glycerol-3-pho	2738.024	4686.607	2059.432
1367897_at	25363	Acadvl	acyl-Coenzyme A dehydrogenase, ver	2737.188	3865.109	993.9987
1398371_at	312083	RGD13047	similar to RIKEN cDNA 1700034M03 g	2737.005	5281.785	2858.993
1367877_at	25715	Slc11a2	solute carrier family 11 (proton-couple	2736.767	3357.695	2476.831
1388339_at	364052	Pea15	phosphoprotein enriched in astrocytes	2734.72	623.3807	1744.752
1383627_a_at	316123	Gtf2f1	general transcription factor IIF, polype	2733.713	4854.044	5717.613
1378155_at	360865	RGD1566C	similar to KIAA1096 protein (predicted)	2732.86	1045.844	1247.036
1373388_at	360822	RGD15628	similar to Hypothetical protein MGC75	2731.529	6203.624	4122.382
1372913_at	307652	RGD13074	similar to RIKEN cDNA 2310065K24	2730.122	3084.181	1165.034
1383834_at	362300	Centg3_pre	centaurin, gamma 3 (predicted)	2729.587	1788.676	2511.095
1372073_at	290669	Gatad2a	GATA zinc finger domain containing 2	2728.797	1959.985	1299.101
1373440_at	362498	Mdn1	midasin homolog (yeast)	2727.52	4495.016	1642.431
1372006_at	290350	Loxl2_pred	lysyl oxidase-like 2 (predicted)	2727.428	436.1209	1327.453
1388808_at	363633	Polr2a_ma	polymerase (RNA) II (DNA directed) po	2726.619	1939.199	3284.57
1367889_at	171503	Camk1	calcium/calmodulin-dependent protein	2724.82	2760.784	1555.741
1376013_at	500689	RGD15659	similar to mKIAA0227 protein (predicte	2724.496	1429.917	3020.514
1389204_at	361269	RGD15644	similar to chromosome 10 open readin	2723.398	3913.308	4272.126
1392469_at	311881	Fbxw2_pre	F-box and WD-40 domain protein 2 (pr	2720.13	2825.401	3720.195
1367872_at	29663	Ap1b1	adaptor protein complex AP-1, beta 1 s	2719.923	2742.715	919.2576
1375685_at	304740	Clasp1	cytoplasmic linker associated protein 1	2719.759	2474.692	3112.156
1380182_at	291356	RGD15634	similar to KIAA1217 (predicted)	2719.593	3060.845	17242.92
1388992_at	304569	Ep400	E1A binding protein p400	2719.023	3071.335	2937.106
1392498_at	500722	RGD15646	similar to protein phosphatase 2A B56	2718.198	6934.495	4054.726
1377141_at	498685	LOC49868	NA	2718.185	8250.924	8182.365
1371698_at	287739	Eftud2	elongation factor Tu GTP binding dom	2717.253	6148.612	1016.109
1389035_at	301245	Yipf3	Yip1 domain family, member 3	2716.485	2767.949	1930.862
1384252_at	301381	RGD1309C	similar to hypothetical protein BC0151	2716.05	1239.269	2126.839
1393046_at	311713	RGD13086	similar to Protein C20orf20	2715.035	4657.254	2101.544
1399088_at	171311	Tlk2	tousled-like kinase 2 (Arabidopsis)	2714.653	2538.887	5071.644
1368834_at	24246	Camk2d	calcium/calmodulin-dependent protein	2714.128	1400.242	10208.84
1381968_at	289185	Creg_predi	cellular repressor of E1A-stimulated ge	2711.299	1290.57	1397.288
1377829_at	315423	Cep57	centrosomal protein 57	2710.602	3300.287	9958.605

1389291_at	296966	Chchd3_pr	coiled-coil-helix-coiled-coil-helix domai	2710.076	5404.573	1976.607
1377625_at	311441	RGD13088	similar to CGI-09 protein (predicted)	2709.698	1956.704	228.0518
1377939_at	313391	LOC31339	NA	2708.871	1584.097	2033.63
1392855_x_at	361706	MGC10583	similar to Ras-related protein Rab-1B	2708.693	3471.164	1274.202
1368710_at	60328	Mark2	MAP/microtubule affinity-regulating kin	2708.32	1853.735	975.2778
1390933_a_at	298081	Rg9mtd3	RNA (guanine-9-) methyltransferase d	2707.032	3945.802	2731.842
1393101_at	304495	Fbxl10	F-box and leucine-rich repeat protein 1	2705.521	8578.725	3267.113
1390825_at	306866	Slc35b3_pr	solute carrier family 35, member B3 (p	2705.448	4108.001	4072.614
1368373_at	54296	Rgs7	regulator of G-protein signaling 7	2704.867	1318.096	8796.73
1370965_at	25705	Tcf8	transcription factor 8	2702.818	763.8752	4923.36
1368060_at	65151	Hrsp12	heat-responsive protein 12	2702.505	5389.046	4766.877
1386096_at	360905	Mtf2	metal response element binding transc	2702.404	5898.893	1520.935
1375988_at	58856	Ddx25	DEAD (Asp-Glu-Ala-Asp) box polypept	2702.194	1861.735	1147.904
1388816_at	25252	Dlgh1	discs, large homolog 1 (Drosophila)	2701.949	6270.47	7413.462
1399016_at	303470	Myst2	MYST histone acetyltransferase 2	2699.045	5497.426	2714.152
1372570_at	304579	Usp30_pre	ubiquitin specific protease 30 (predicte	2695.304	2897.054	2294.41
1373214_at	316370	Kdelc1	KDEL (Lys-Asp-Glu-Leu) containing 1	2694.827	2551.215	2178.76
1367551_a_at	360768	RGD13064	similar to CG14980-PB	2694.089	10256.4	6269.055
1389627_at	312303	RGD13062	similar to hypothetical protein FLJ3141	2693.529	2114.89	1599.341
1383081_at	652957	Csf2ra	NA	2693.264	3504.166	4563.937
1388467_at	64667	Sgta	small glutamine-rich tetratricopeptide r	2693.176	2435.098	1272.074
1372523_at	25283	Gclc	glutamate-cysteine ligase, catalytic sul	2692.025	1202.931	2072.981
1372373_at	317370	Wdr13_pre	WD repeat domain 13 (predicted)	2689.418	4898.931	6935.956
1376758_at	306626	Ing1	inhibitor of growth family, member 1	2689.082	3636.287	2635.853
1370300_at	58842	Preb	prolactin regulatory element binding	2686.332	1622.518	552.1424
1389983_at	313777	RGD13093	similar to cDNA sequence AF155546	2686.194	1441.42	1452.378
1388432_at	246294	Optn	optineurin	2684.268	1778.717	10512.04
1371326_at	307641	Csnk2a2_p	casein kinase II, alpha 2, polypeptide (	2683.714	2877.984	2581.535
1370437_at	245922	Nupl1	nucleoporin like 1	2683.562	1802.348	4586.965
1367993_at	65201	Rsn	restin (Reed-Steinberg cell-expressed	2683.061	1401.826	606.0452
1370351_at	85425	Tdrd7	tudor domain containing 7	2682.046	5393.174	6174.193
1393604_at	360810	Atpbd1c	ATP binding domain 1 family, member	2679.807	3612.982	1362.971
1392579_at	363227	RGD13066	similar to 5830411E10Rik protein	2679.802	759.6335	732.1332
1387222_at	56725	Cript	postsynaptic protein Cript	2678.111	4122.412	2478.492
1387759_s_at	113992	Ugt1a6	UDP glycosyltransferase 1 family, poly	2676.699	1902.091	795.5145
1372755_at	362911	Mal2	mal, T-cell differentiation protein 2	2675.268	6136.765	3201.299
1389247_at	361102	RGD13055	similar to polymerase (RNA) III (DNA c	2674.03	2705.994	1010.69
1367945_at	84355	Atox1	ATX1 (antioxidant protein 1) homolog	2671.597	8384.3	4791.672
1377787_at	315997	RGD15603	similar to Rbm6 protein (predicted)	2671.51	1087.406	2090.822
1379832_at	364834	Polr2d_pre	polymerase (RNA) II (DNA directed) po	2668.692	8549.758	6313.32
1389133_at	298786	Slc30a6_pr	solute carrier family 30 (zinc transporte	2668.467	2315.807	1655.352
1375315_at	361172	NA	NA	2665.657	1834.13	2755.99
1371131_a_at	117514	Txnip	upregulated by 1,25-dihydroxyvitamin	2664.941	19368.69	1972.699
1370913_at	65190	Best5	Best5 protein	2664.867	170.4816	5194.968
1372769_at	498225	LOC49822	similar to ligatin	2660.86	2689.794	1241.236
1380201_at	313981	Nol10	nucleolar protein 10	2659.517	3498.532	1566.448
1387636_a_at	171432	Cdtw1	P11 protein	2659.257	7341.011	1107.731
1388593_at	302983	Mapk8ip3	mitogen-activated protein kinase 8 inte	2658.432	771.0335	1249.251
1389907_at	297885	Zbtb8os_pr	zinc finger and BTB domain containing	2657.634	7123.295	1159.519
1372438_at	288174	Nit2	nitrilase family, member 2	2655.465	5774.219	1644.671
1389459_at	306994	RGD13066	similar to RIKEN cDNA 1600012F09 (p	2654.613	2318.118	1620.005

1392629_a_at	362990	Zcrb1	zinc finger CCHC-type and RNA bindir	2653.886	3956.831	2151.72
1373502_at	291433	Dym_predi	dymeclin (predicted)	2653.823	4405.728	1970.473
1368117_at	64845	Gphn	gephyrin	2652.804	1807.579	1015.895
1371874_at	361190	Usp19	ubiquitin specific protease 19	2651.884	1949.017	3368.023
1377662_at	363465	Pir	pirin	2650.837	4022.227	1755.804
1367982_at	65155	Alas1	aminolevulinic acid synthase 1	2650.232	1297.191	1288.633
1378128_at	313993	Grhl1_prec	grainyhead-like 1 (Drosophila) (predict	2650.02	1416.686	473.5607
1389069_at	361815	Rnf8	ring finger protein 8	2649.6	1800.002	2048.283
1389524_at	361920	Ythdf3_pre	YTH domain family 3 (predicted)	2647.309	3077.787	5984.951
1398356_at	291877	Cpsf5	cleavage and polyadenylation specific	2647.126	8693.213	3088.152
1375745_at	81666	Gnaq	guanine nucleotide binding protein, alp	2645.182	4235.136	3199.585
1376262_at	246232	Uxs1	UDP-glucuronate decarboxylase 1	2644.737	2030.215	857.9505
1370906_at	29711	Bckdhb	branched chain keto acid dehydrogena	2643.533	1812.15	1244.862
1367857_at	84575	Fads1	fatty acid desaturase 1	2639.718	21066.08	2130.933
1390168_a_at	362184	Zcsl3_pred	zinc finger, CSL-type containing 3 (pre	2638.944	4996.034	1824.38
1374964_at	315758	Dpp8_pred	dipeptidylpeptidase 8 (predicted)	2637.53	1492.302	1875.048
1389377_at	288985	Insig2	insulin induced gene 2	2636.287	2494.79	3972.385
1371876_at	291539	Tnfsf5ip1_p	tumor necrosis factor superfamily, mer	2632.071	2962.704	1890.503
1372519_at	291874	Nup93	nucleoporin 93	2631.495	7852.685	908.6869
1371383_at	293674	Drap1_pre	Dr1 associated protein 1 (negative cofa	2631.348	3275.001	1252.369
1372234_at	499677	LOC49967	NA	2630.235	935.0355	4875.53
1385526_at	365601	LOC36560	similar to autophagy 5-like	2629.786	1962.752	2603.104
1389016_at	290864	LOC29086	NA	2629.399	1066.331	1098.299
1377684_at	297969	Rarsl_pred	arginyl-tRNA synthetase-like (predicted	2624.872	3547.982	1821.292
1372705_at	290614	Cherp_pre	calcium homeostasis endoplasmic retic	2624.288	2786.53	3636.271
1369122_at	24887	Bax	Bcl2-associated X protein	2623.958	4257.85	1595.591
1389355_at	498256	Ier5	immediate early response 5	2623.934	1740.52	5815.091
1372747_at	29502	Slc20a2	solute carrier family 20, member 2	2621.111	2162.557	557.4692
1386880_at	170465	Acaa2	acetyl-Coenzyme A acyltransferase 2 (	2620.521	3633.88	3067.171
1376225_at	65199	Csen	calsenilin, presenilin binding protein, E	2619.629	4399.684	2243.936
1371027_at	171136	Cblb	Casitas B-lineage lymphoma b	2619.365	1605.796	1471.083
1392562_at	287929	RGD13081	similar to RIKEN cDNA 2610001E06 (p	2616.71	2366.211	1120.079
1392996_at	293056	Cpeb1_pre	cytoplasmic polyadenylation element b	2616.306	982.5731	2389.895
1390104_at	292708	Irgq_predic	immunity-related GTPase family, Q (pr	2612.631	2534.123	1396.617
1398833_at	89842	Mbtps1	membrane-bound transcription factor p	2611.977	2976.958	2364.915
1390110_at	499956	RGD15654	similar to Uridine-cytidine kinase 1-like	2611.679	4116.881	2955.863
1371909_at	309565	Crsp3	cofactor required for Sp1 transcriptiona	2609.875	4000.687	2206.096
1371470_at	29646	Adh4	alcohol dehydrogenase 4 (class II), pi p	2609.255	11266.1	3261.598
1393267_at	313323	Psip1	PC4 and SFRS1 interacting protein 1	2606.505	4200.79	7286.058
1375279_at	498423	Sertad2	SERTA domain containing 2	2602.384	1280.889	1504.513
1378250_at	311545	H13_predic	histocompatibility 13 (predicted)	2601.049	1742.108	3755.465
1372218_at	363237	Wdr12	WD repeat domain 12	2600.189	1577.637	1477.821
1389254_at	311266	Qser1_pre	glutamine and serine rich 1 (predicted)	2599.738	1580.288	2818.208
1384413_at	498081	RGD15643	similar to solute carrier family 35, mem	2598.596	3214.725	4163.238
1393213_at	298686	Ccnl2	cyclin L2	2598.154	1305.261	851.3683
1373437_at	313030	Ppp1r8_pre	protein phosphatase 1, regulatory (inhi	2597.486	4149.19	4492.026
1373425_at	365842	LOC36584	similar to CDC-like kinase 2	2596.714	1319.358	1088.503
1386951_at	25488	Ndufa5	NADH dehydrogenase (ubiquinone) 1	2596.272	4524.992	2896.601
1388553_at	290577	LOC29057	NA	2595.291	3379.088	5119.36
1371613_at	362271	Elmo2	engulfment and cell motility 2, ced-12 h	2594.196	5016.769	1923.952
1388893_at	306253	Glt8d1	glycosyltransferase 8 domain containir	2594.115	5301.904	6957.843

1376190_at	29741	Pik3r2	phosphatidylinositol 3-kinase, regulato	2592.412	2930.988	1062.972
1367508_at	497874	RGD15657	RGD1565784 (predicted)	2592.099	2717.121	762.6985
1368069_at	81859	Sharpin	SHANK-associated RH domain interac	2591.574	3348.451	1633.117
1371789_at	301117	LOC30111	NA	2589.256	4504.578	1390.138
1367584_at	56611	Anxa2	annexin A2	2588.192	2159.949	762.6176
1367921_at	64538	Ilkap	integrin-linked kinase-associated serin	2581.602	1639.792	2290.454
1371892_at	303190	Atpaf2_pre	ATP synthase mitochondrial F1 comple	2580.201	3706.757	1748.643
1371741_at	294010	Actr1a_pre	ARP1 actin-related protein 1 homolog	2579.421	7547.812	2155.551
1388669_at	313583	Sf3a3	splicing factor 3a, subunit 3	2579.147	7364.56	3279.576
1388725_at	24536	Lepr	leptin receptor	2577.948	3972.775	3174.993
1380943_at	313949	RGD13116	similar to hypothetical protein FLJ2182	2576.598	4511.355	2217.37
1377599_at	313977	Lpin1	lipin 1	2574.928	2904.271	13002.31
1377728_at	499567	RGD15656	RGD1565641 (predicted)	2574.476	3125.755	5058.017
1371907_at	288656	Arl6ip4	ADP-ribosylation factor-like 6 interactir	2573.769	3178.292	1085.267
1375249_at	313594	Eif2c1_pre	eukaryotic translation initiation factor 2	2572.951	2763.246	2325.234
1367725_at	64534	Pim3	serine/threonine-protein kinase pim-3	2572.815	1488.355	2543.103
1390128_at	24611	Gnal	guanine nucleotide binding protein, alp	2571.974	3391.709	3678.971
1388309_at	117062	Hmga1	high mobility group AT-hook 1	2569.449	1083.267	758.7604
1371528_at	290652	Fkbp8	FK506 binding protein 8	2567.649	1845.053	1139.492
1373174_at	497867	LOC49786	similar to 1110001K21Rik protein	2566.175	4081.032	1456.418
1372634_at	362600	Adprhl2_pr	ADP-ribosylhydrolase like 2 (predicted	2565.414	1940.407	1309.397
1388556_at	60562	Stx6	syntaxin 6	2563.872	957.1072	1962.671
1376718_at	304346	RGD13069	similar to CG9117-PA	2562.995	1486.789	733.1697
1373611_at	312679	Il17r_predi	interleukin 17 receptor (predicted)	2562.929	3081.434	966.1792
1390558_at	171500	Stau2	staufen, RNA binding protein, homolog	2562.349	4805.286	3335.989
1376362_at	81005	Nptxr	neuronal pentraxin receptor	2561.67	1484.799	2364.24
1391848_at	361317	Rbm27_pre	RNA binding motif protein 27 (predicte	2561.492	1196.597	1330.56
1374545_at	307271	Rkhd2_pre	ring finger and KH domain containing 2	2560.627	5222.379	9911.336
1387225_at	171116	Opa1	optic atrophy 1 homolog (human)	2560.098	2800.288	1131.495
1376343_at	361335	LOC36133	hypothetical protein LOC361335	2559.064	2092.713	1975.357
1370002_at	60323	Arhgef1	Rho guanine nucleotide exchange fact	2557.655	1637.6	1657.187
1375973_at	300756	Arih1	ariadne ubiquitin-conjugating enzyme I	2557.626	1211.342	4523.263
1367535_at	308404	Irf2bp1_pre	interferon regulatory factor 2 binding p	2557.374	1698.04	1073.725
1392588_at	304791	Ripk5	receptor interacting protein kinase 5	2556.937	2141.692	2937.093
1390846_at	366474	Col16a1	procollagen, type XVI, alpha 1	2556.554	1081.644	1035.767
1376992_a_at	315601	Foxr1_pred	forkhead box R1 (predicted)	2554.823	941.4991	2293.148
1387063_at	59268	Ihpk2	inositol hexaphosphate kinase 2	2553.655	2067.566	3836.187
1372174_at	297900	Peflin	PEF protein with a long N-terminal hyc	2553.31	3625.817	1329.996
1369792_at	83683	Gpr6	G protein-coupled receptor 6	2551.118	6662.117	75.86644
1389885_at	360646	Limd2	LIM domain containing 2	2550.862	6334.453	1493.421
1383495_at	292127	Dclre1a_pr	DNA cross-link repair 1A, PSO2 homo	2550.221	1166.416	1173.221
1373477_at	297372	Mrpl19	ribosomal protein, mitochondrial, L15	2550.051	4594.334	1369.943
1371650_at	303245	Senp3	SUMO/sentrin specific peptidase 3	2549.939	4581.489	1985.949
1367817_at	114499	Hdgf	hepatoma-derived growth factor	2548.643	2396.84	1389.558
1372918_at	498062	NA	NA	2546.763	4236.919	1796.291
1389447_at	301008	RGD13118	similar to prolyl-4-hydroxylase-alpha N	2545.746	1150.076	1039.974
1389273_at	316640	Atg4b	autophagy-related 4B (yeast)	2545.666	1576.765	2495.048
1374124_at	360498	RGD13073	similar to RIKEN cDNA 2610003J06	2544.516	807.2591	2264.411
1398301_at	293291	RGD15638	similar to ribosomal protein L36 (predic	2540.01	4954.954	1179.492
1375964_at	304429	Psph	phosphoserine phosphatase	2538.405	2329.916	3983.003
1398944_at	305884	Acin1	apoptotic chromatin condensation indu	2537.65	1398.467	2013.494

1376596_at	303607	Ddx42_pre	DEAD (Asp-Glu-Ala-Asp) box polypept	2537.02	3128.976	3943.581
1388154_at	116651	E2f5	E2F transcription factor 5	2536.88	2576.595	6726.865
1390416_at	361074	Slc25a30	solute carrier family 25, member 30	2534.698	1730.491	1810.023
1390806_at	295952	RGD13097	similar to RIKEN cDNA B230118H07 (	2532.889	2911.048	3943.725
1373976_at	300146	RGD15639	similar to 8430411H09Rik protein (pre	2532.189	1174.41	1046.795
1388709_at	362703	LOC36270	similar to WD-repeat protein 43	2532.131	2426.57	3366.488
1373132_at	499267	RGD15612	similar to CDNA sequence BC006909	2531.01	2273.427	3087.679
1376489_at	85384	Sos2	son of sevenless homolog 2 (Drosophi	2530.99	1799.964	6016.828
1386916_at	50655	Aco1	aconitase 1	2529.384	4299.005	2488.102
1369997_at	83721	Dvl1	dishevelled, dsh homolog 1 (Drosophil	2529.226	835.6451	883.936
1373003_at	304582	Sart3_pred	squamous cell carcinoma antigen reco	2526.499	6907.473	4297.2
1373366_at	293721	Ganab_pre	alpha glucosidase 2 alpha neutral subu	2525.458	4053.689	1698.775
1372933_at	303745	Sirt7_predi	sirtuin 7 (silent mating type information	2524.382	2582.612	1354.469
1371671_at	293546	Gprc5b_pr	G protein-coupled receptor, family C, g	2524.069	1213.517	1204.364
1372362_at	296117	LOC29611	similar to N-acetylgalactosamine kinas	2522.823	2867.253	2190.053
1373190_at	312227	Cnot4	CCR4-NOT transcription complex, sub	2522.596	1382.184	3314.473
1378753_at	83497	Tpmt	thiopurine methyltransferase	2521.626	7236.487	6398.048
1372295_at	360681	Narf	nuclear prelamin A recognition factor	2521.607	2976.378	844.4468
1376645_at	360757	RGD13073	similar to RIKEN cDNA 6330406I15 (p	2520.951	21.56566	1522.648
1396619_at	311362	RGD15615	similar to MGC14161 protein (predicte	2519.986	536.0788	436.7545
1387870_at	79426	Zfp36	zinc finger protein 36	2519.16	454.546	18023.6
1368330_at	114512	Aatf	apoptosis antagonizing transcription fa	2519.054	4072.365	1157.252
1371592_at	315707	Csk	c-src tyrosine kinase	2513.688	1506.528	2262.213
1369994_at	114205	Crcp	calcitonin gene-related peptide-recepto	2513.41	2810.022	3458.91
1383337_at	307913	RGD15626	similar to genetic suppressor element	2512.155	1416.606	3689.724
1393738_s_at	306508	Mfhas1_pr	malignant fibrous histiocytoma amplifie	2511.686	1356.088	1151.873
1374157_at	24626	Pde4b	phosphodiesterase 4B, cAMP specific	2511.451	1190.413	1298.839
1372112_at	308906	RGD13045	similar to 9230105E10Rik protein	2510.796	2711.613	4403.713
1372212_at	293588	Paox_pred	polyamine oxidase (predicted)	2509.923	939.2093	363.151
1389057_at	292097	Arv1_predi	ARV1 homolog (yeast) (predicted)	2509.119	2238.954	1635.54
1367756_at	114017	Gfm	G elongation factor	2506.823	2385.699	1257.121
1399151_at	360531	RGD13095	similar to hypothetical protein FLJ1152	2506.418	2720.238	2348.841
1372193_at	497198	Impact	NA	2506.201	1859.077	14378.82
1368909_a_at	116493	Gripap1	GRIP1 associated protein 1	2506.098	2684.716	1212.432
1398652_at	312952	Impad1	inositol monophosphatase domain con	2503.218	4954.253	1362.413
1391412_at	362229	Xrn2_predi	5'-3' exoribonuclease 2 (predicted)	2502.558	6321.311	2794.814
1377785_at	287595	Dhx40	DEAH (Asp-Glu-Ala-His) box polypepti	2501.951	5339.391	5151.688
1372845_at	406230	Rpp21	ribonuclease P 21 subunit (human)	2501.486	5797.926	2142.58
1372082_at	298598	Necap2	NECAP endocytosis associated 2	2500.919	1265.94	1402.99
1371805_at	299982	Ankrd46	ankyrin repeat domain 46	2499.079	5774.016	3854.517
1368043_at	84471	Snx1	sorting nexin 1	2497.972	1454.372	1696.59
1367478_at	297376	Prss25	protease, serine, 25	2497.742	4651.206	1942.479
1390863_at	289175	Slc19a2	solute carrier family 19 (thiamine trans	2492.093	1489.613	3388.065
1374433_at	501763	NA	NA	2492.025	2828.473	10452.18
1371932_at	361137	RGD13093	similar to hypothetical protein FLJ3848	2491.556	3787.464	1207.525
1367638_at	85239	Mlycd	malonyl-CoA decarboxylase	2490.876	1023.156	1061.522
1394597_at	305816	Ddhd1	DDHD domain containing 1	2490.844	882.1454	1276.653
1385101_a_at	308060	RGD73510	similar to RIKEN cDNA 0610011N22 g	2488.952	561.8216	935.8544
1376572_a_at	361256	Svil_predic	supervillin (predicted)	2487.794	3067.873	3236.234
1377834_at	307042	Epc1_pred	enhancer of polycomb homolog 1 (Dro	2487.693	3630.377	6315.333
1389197_at	288091	RGD13062	similar to RIKEN cDNA 9630046K23	2487.411	4347.681	1758.524

1375045_at	307855	Wdr59	WD repeat domain 59	2486.79	487.9993	1440.073
1388535_at	309069	RGD13068	similar to erythroid differentiation-relate	2485.889	1455.518	2578.364
1376340_a_at	361682	Tssc4	tumor-suppressing subchromosomal tr	2485.107	8337.698	1879.495
1375707_at	501745	RGD15651	similar to dendritic cell-derived immuno	2483.548	991.2348	3123.306
1387071_a_at	29477	Mapt	microtubule-associated protein tau	2483.313	14253.87	1591.247
1374033_at	291983	Psmb10	proteasome (prosome, macropain) sub	2480.555	2195.581	995.5909
1373272_at	246237	Plekha5	pleckstrin homology domain containing	2480.264	2247.452	2144.71
1371891_at	361038	RGD13084	similar to 1700123O20Rik protein (pre	2480.261	1751.58	3995.143
1379028_at	303260	Spag7_pre	sperm associated antigen 7 (predicted	2479.467	3412.32	2346.136
1389217_at	60433	Rfng	radical fringe gene homolog (Drosophi	2479.255	2627.412	849.931
1376115_at	312682	Bcl2l13_pr	BCL2-like 13 (apoptosis facilitator) (pre	2478.3	4179.869	2988.147
1393345_at	312981	Asph_pred	aspartate-beta-hydroxylase (predicted)	2478.099	3459.663	3196.572
1367861_at	79115	Evl	Ena-vasodilator stimulated phosphopro	2477.56	6165.68	5662.234
1376285_at	314543	Gulp1	GULP, engulfment adaptor PTB doma	2476.921	6978.097	6639.36
1373149_at	293491	RGD15645	similar to yippee-like 3 (predicted)	2475.823	2943.423	2961.605
1375299_at	287447	Dullard	Dullard homolog (Xenopus laevis)	2474.872	1215.886	687.4882
1371845_at	292831	Pop4	processing of precursor 4, ribonucleas	2473.356	4776.498	2053.228
1388716_at	361798	Ehmt2	euchromatic histone lysine N-methyltra	2472.928	3603.083	1316.24
1376177_at	497983	RGD15610	similar to RIKEN cDNA 5730593F17 (p	2471.554	2824.178	6271.598
1392332_at	362291	Myt1_predi	myelin transcription factor 1 (predicted	2470.056	6583.278	4265.046
1369743_a_at	29659	P2rx4	purinergic receptor P2X, ligand-gated i	2468.986	1799.966	1194.426
1393309_at	363030	NA	NA	2466.3	2452.88	2506.591
1372971_at	311855	RGD13051	similar to Hypothetical protein MGC11(	2466.232	4530.396	2509.885
1374486_at	78966	Arhgef11	Rho guanine nucleotide exchange fact	2466.097	1443.821	1185.367
1387344_at	81708	Aldh6a1	aldehyde dehydrogenase family 6, sub	2464.854	1479.324	2771.015
1394605_at	497960	RGD15661	similar to CDNA sequence BC017647	2462.996	1879.658	1171.229
1382316_at	363849	LOC36384	NA	2461.627	3191.691	3220.457
1374215_at	314634	Plekhj1	pleckstrin homology domain containing	2460.755	1514.483	1039.064
1398890_at	292878	MGC93975	similar to 2310044H10Rik protein	2457.832	5657.135	2285.787
1377480_at	300687	RGD13080	similar to RIKEN cDNA D030060M11	2457.749	1534.185	1525.278
1390577_at	361396	Ranbp10_p	RAN binding protein 10 (predicted)	2456.785	1112.27	2965.133
1383358_at	114124	Akap1	A kinase (PRKA) anchor protein 1	2456.284	2524.394	1355.403
1368991_at	94338	Smpd3	sphingomyelin phosphodiesterase 3, n	2455.191	1404.608	3693.368
1388912_at	311826	Rexo4	REX4, RNA exonuclease 4 homolog (S	2454.706	4706.299	1661.64
1392938_s_at	361624	RGD13069	similar to C11orf17 protein (predicted)	2453.695	7650.365	3413.237
1376273_at	310159	Rnasen	ribonuclease III, nuclear	2453.32	3064.153	1891.553
1372962_at	363006	Tarbp2	TAR (HIV) RNA binding protein 2	2452.791	1330.241	713.3346
1388436_at	292729	Snrpa	small nuclear ribonucleoprotein polype	2452.6	2938.263	441.0276
1374332_at	290660	Ddx49_pre	DEAD (Asp-Glu-Ala-Asp) box polypept	2451.386	1717.046	841.1792
1398967_at	619436	Thrap4	thyroid hormone receptor associated p	2450.799	3382.981	1992.889
1374446_at	310467	Tiparp_pre	TCDD-inducible poly(ADP-ribose) poly	2450.457	1701.943	22245.15
1398308_at	296883	Rpa3_pred	replication protein A3 (predicted)	2449.208	11172.43	4163.963
1398317_at	64473	Bpnt1	bisphosphate 3'-nucleotidase 1	2448.732	76.42504	1746.875
1372859_at	501167	Gmppa	GDP-mannose pyrophosphorylase A	2448.66	640.0175	2075.756
1382362_at	498682	RGD15663	similar to regulator of sex-limitation car	2448.66	1708.59	2200.558
1373918_at	362757	Rdh11	retinol dehydrogenase 11	2448.347	1379.832	660.9853
1392930_at	294948	Armc1_pre	armadillo repeat containing 1 (predicte	2447.281	5436.613	4412.013
1374617_at	287380	Dhrs7b	dehydrogenase/reductase (SDR family	2446.238	3782.43	631.557
1370842_at	29603	Bckdk	branched chain ketoacid dehydrogena	2446.075	5454.515	613.2549
1369999_a_at	94270	Nnat	neuronatin	2445.724	40141.34	5380.871
1377417_at	363328	LOC36332	NA	2445.538	548.3949	677.2435

1367831_at	24842	Tp53	tumor protein p53	2444.481	2636.163	3276.777
1370947_at	245975	Rda279	hypothetical protein RDA279	2443.881	4229.482	1189.757
1372147_at	499529	Ndufs4	NA	2443.113	2543.489	3001.315
1388407_at	360677	RGD13119	similar to BC003940 protein (predicted	2442.964	3769.896	1591.847
1374500_at	499691	LOC49969	similar to sarcoma antigen NY-SAR-27	2440.153	1796.383	4062.222
1388815_at	361502	Saps1_pre	SAPS domain family, member 1 (predi	2439.819	3013.263	1746.271
1388480_at	288707	Gltp_predic	glycolipid transfer protein (predicted)	2439.4	4342.247	1328.255
1372861_at	314641	Pip5k1c	phosphatidylinositol-4-phosphate 5-kin	2438.555	5738.615	4799.88
1390787_at	288259	Gart	phosphoribosylglycinamide formyltrans	2438.465	2741.577	837.7264
1374489_at	363195	Gtpbp2	GTP binding protein 2	2437.927	2004.819	950.0963
1374411_at	361037	Mrpl52_pre	mitochondrial ribosomal protein L52 (p	2436.638	2441.574	2277.217
1379594_at	308923	Ahi1	Abelson helper integration site 1	2436.071	300.5249	2662.926
1372413_at	300222	Mcrs1	microspherule protein 1	2435.926	4691.154	1109.746
1373273_at	298374	Prpf38a_pr	PRP38 pre-mRNA processing factor 3	2435.434	4317.102	4218.936
1382100_at	291320	RGD13096	similar to RIKEN cDNA 2310047O13 (	2433.94	3095.449	1713.285
1372045_at	306332	Ap1m1	adaptor-related protein complex AP-1,	2433.838	3940.768	1363.31
1368649_at	170944	Dkc1	dyskeratosis congenita 1, dyskerin	2433.108	976.5997	1965.59
1390214_a_at	296639	Ciz1_predi	CDKN1A interacting zinc finger protein	2432.543	1938.23	1362.474
1373420_at	300447	MGC94704	evolutionarily conserved signaling inter	2431.266	1115.156	669.246
1368488_at	114519	Nfil3	nuclear factor, interleukin 3 regulated	2430.214	696.3604	6224.656
1367980_at	54190	Rabep1	rabaptin, RAB GTPase binding effecto	2429.907	4631.247	3949.067
1372455_at	362326	Tm4sf12	transmembrane 4 superfamily member	2429.146	837.3444	3023.58
1372930_at	301570	Sp110	SP110 nuclear body protein	2427.362	3600.904	3327.158
1372228_at	288527	Asmtl_prec	acetylserotonin O-methyltransferase-lil	2424.405	3739.342	860.6415
1374857_at	499709	LOC49970	similar to nucleolar protein family A, m	2422.643	1805.705	1769.322
1389791_at	306619	Cln8	ceroid-lipofuscinosis, neuronal 8	2421.057	1960.066	7071.676
1369729_at	117050	Arl5a	ADP-ribosylation factor-like 5A	2421.039	1140.251	1186.701
1399108_at	363077	RGD13089	similar to expressed sequence AV3403	2420.675	1215.033	2759.599
1389139_at	314013	RGD15660	similar to Ttc15 protein (predicted)	2419.911	2689.234	1980.205
1371790_at	287656	Mrpl45_pre	mitochondrial ribosomal protein L45 (p	2419.42	3449.761	1460.907
1398337_at	290341	LOC29034	NA	2419.084	3702.345	4223.006
1389576_at	362223	Snrpb2_pre	U2 small nuclear ribonucleoprotein B (	2419.081	8228.862	3830.5
1389969_at	308416	Tomm40	translocase of outer mitochondrial mem	2418.437	1899.115	576.9667
1388337_at	290029	Np_mappe	nucleoside phosphorylase (mapped)	2418.122	2410.981	2095.337
1371997_at	307091	Akr1e1	aldo-keto reductase family 1, member	2418.084	7410.673	2202.165
1368045_at	171135	Slc31a1	solute carrier family 31 (copper transpo	2417.064	1521.703	651.9043
1376261_at	366734	Bag5	BCL2-associated athanogene 5	2417.034	2862.435	2681.597
1388679_at	360956	Tbc1d14	TBC1 domain family, member 14	2415.423	4351.872	1909.259
1372496_at	313786	RGD15612	similar to hypothetical protein MGC196	2415.322	1540.29	1186.596
1370964_at	25698	Ass	argininosuccinate synthetase	2415.15	1342.333	598.7428
1388863_at	298317	Ube2a	ubiquitin-conjugating enzyme E2A, RA	2415.091	4837.723	3615.782
1373099_at	303277	Pigs	phosphatidylinositol glycan, class S	2413.636	2723.332	1246.473
1382942_at	299907	LOC29990	NA	2413.105	159.8321	1061.643
1371536_at	260416	Carhsp1	calcium regulated heat stable protein 1	2411.474	3227.969	460.5198
1377102_at	289318	Tmem63a_	transmembrane protein 63a (predicted	2411.09	1771.361	1274.348
1382620_at	365023	Ankrd11_p	ankyrin repeat domain 11 (predicted)	2408.239	286.5352	1283.772
1376043_at	360550	MGC94192	similar to PHD zinc finger containing p	2407.325	3124.026	2191.406
1373546_at	246251	Ua20	putative UA20 protein	2406.462	2655.441	6588.29
1375767_at	314249	RGD13080	similar to common-site lymphoma leuk	2406.231	2071.588	3294.647
1373263_at	499312	NA	NA	2405.088	4160.064	1551.773
1368184_at	161475	Psmd9	proteasome (prosome, macropain) 26S	2404.093	3749.426	741.3657

1374364_at	498703	RGD15623	similar to mKIAA1931 protein (predicte	2403.064	2872.39	1444.018
1373433_at	365273	Nsbp1_pre	nucleosome binding protein 1 (predicte	2402.41	6765.464	9368.515
1372320_at	317464	Msl31	male-specific lethal-3 homolog 1 (Dros	2402.355	6995.668	2341.174
1369896_s_at	245926	Rbm16	RNA binding motif protein 16	2402.11	4464.964	11119.5
1395579_at	361667	Dhx32_pre	DEAH (Asp-Glu-Ala-His) box polypepti	2400.841	2687.636	918.4214
1370994_at	81917	Hip1r	huntingtin interacting protein 1 related	2400.323	2019.353	1092.287
1393114_at	407764	siat7D	alpha-2,6-sialyltransferase ST6GalNA(	2399.817	1199.857	2025.331
1367922_at	57027	Adam17	a disintegrin and metalloproteinase do	2399.636	2852.515	3242.268
1379578_at	288105	Zbtb20_pre	zinc finger and BTB domain containing	2399.326	629.3873	2818.184
1398344_at	300367	Crsp6	cofactor required for Sp1 transcriptiona	2396.923	2425.114	2571.399
1377011_at	304244	RGD13070	similar to hypothetical protein CG003 (	2396.288	95.04246	5248.743
1377400_at	314830	Hrb2_predi	HIV-1 Rev binding protein 2 (predicted	2394.965	3867.766	2819.553
1373404_at	287653	Lrrc46	leucine rich repeat containing 46	2393.35	2823.978	1272.131
1389152_at	309376	Zfyve27	zinc finger, FYVE domain containing 2	2393.161	4075.752	2310.271
1393009_at	308568	RGD13093	similar to RIKEN cDNA 2410002F23	2393.151	2321.281	1872.99
1387018_at	114901	Argbp2	Arg/Abl-interacting protein ArgBP2	2392.814	8180.174	3577.11
1372233_at	296306	Ergic3_pre	ERGIC and golgi 3 (predicted)	2390.763	4458.754	5095.18
1380582_at	78965	Csf1	colony stimulating factor 1 (macrophag	2389.143	1319.51	2046.14
1367773_at	29743	Slc25a1	solute carrier family 25, member 1	2383.105	6373.573	1185.824
1372900_at	296360	Pigt_predic	phosphatidylinositol glycan, class T (pr	2381.558	3724.891	452.1653
1383621_at	302705	Mbtps2	membrane-bound transcription factor p	2381.554	2569.814	2131.709
1383229_at	299609	Abca7	ATP-binding cassette, sub-family A (AI	2381.246	1513.408	1160.808
1374631_at	362813	Obfc2b	oligonucleotide/oligosaccharide-bindin	2378.073	5469.415	1556.769
1374719_at	309637	RGD13072	similar to hypothetical protein FLJ2030	2376.962	2459.86	1505.493
1379463_at	246776	Filip1	filamin A interacting protein 1	2376.288	548.7503	1106.275
1376801_at	294291	RGD15644	RGD1564450 (predicted)	2376.093	2249.761	1015.794
1374358_at	294004	RGD13076	similar to CG13901-PA	2375.539	7208.572	1749.356
1383962_at	362791	Siva_predi	Cd27 binding protein (Hindu God of de	2373.222	6297.843	2375.241
1388697_at	365382	Inpp5a_pre	inositol polyphosphate-5-phosphatase	2372.352	1542.323	3435.097
1383231_at	313588	Snip1	Smad nuclear interacting protein 1	2371.935	4282.472	2888.812
1367803_at	53372	Nup54	nucleoporin 54	2371.325	2927.701	3085.38
1388733_at	304709	Bfar	bifunctional apoptosis regulator	2371.05	2700.385	1007.128
1399072_at	282837	Fibp	fibroblast growth factor (acidic) intrace	2370.428	2354.373	1225.57
1383389_at	311842	Zyg11bl	zyg-11 homolog B (C. elegans)-like	2370.304	1646.764	1862.1
1398642_at	290947	Mtrr	5-methyltetrahydrofolate-homocysteine	2369.622	2879.019	2644.187
1374929_at	500117	RGD15611	similar to RIKEN cDNA 6330407D12 (p	2369.381	2948.886	3153.187
1373337_at	298085	Grhpr_pred	glyoxylate reductase/hydroxypyruvate	2368.978	1691.262	1121.217
1370264_at	499010	CPG2	CPG2 protein	2368.622	1759.078	7606.762
1373028_at	140585	Ryk	receptor-like tyrosine kinase	2365.912	2034.392	918.5239
1388148_a_at	116565	Lrpap1	low density lipoprotein receptor-related	2365.473	2894.871	2496.89
1372677_at	314644	RGD13047	similar to RIKEN cDNA 1110033C18	2365.363	1708.644	1243.303
1378096_at	117550	Kif5b	kinesin family member 5B	2364.396	2113.906	994.5013
1378274_at	291540	Cep76	centrosomal protein 76	2363.922	2519.175	1831.988
1375739_at	192204	Ehd4	EH-domain containing 4	2362.569	1124.691	702.5228
1389988_at	498024	RGD15660	similar to Potassium channel tetrameri	2362.139	2436.719	1981.54
1382046_at	303604	Map3k3_pr	mitogen activated protein kinase kinas	2361.067	1743.401	3642.358
1374911_at	296346	RGD13031	oxidative stress responsive gene	2361.055	3481.152	3582.056
1367659_s_at	29740	Dci	dodecenoyl-coenzyme A delta isomera	2360.152	1183.051	2584.798
1379385_at	362721	Tssc1	tumor suppressing subtransferable car	2355.546	4177.195	979.2822
1370017_at	25437	Emd	emerin	2354.405	2251.791	3593.364
1377060_at	361884	Mccc2	methylcrotonoyl-Coenzyme A carboxyl	2353.848	2125.822	1672.466

1376653_at	252962	Phtf1	putative homeodomain transcription fa	2353.483	4297.032	2477.171
1383179_at	311368	RGD13092	similar to hypothetical protein HSPC12	2353.39	2471.394	2932.042
1376714_at	302032	RGD13081	similar to F-box protein FBL2	2352.603	4446.079	3999.709
1372133_at	365355	Rras2	related RAS viral (r-ras) oncogene hon	2352.216	4446.88	2736.179
1371721_at	500532	LOC50053	NA	2350.596	2589.866	863.4724
1398309_at	192263	Pigl	phosphatidylinositol glycan, class L	2350.563	1389.399	1217.849
1382232_at	307759	Gpt2_predi	glutamic pyruvate transaminase (alanii	2349.647	585.5781	1053.757
1371005_at	24565	Abcc1	ATP-binding cassette, sub-family C (C	2348.234	1477.953	1017.763
1399068_at	290628	RGD15654	similar to RIKEN cDNA 9430098E02 (p	2346.859	2845.203	1671.767
1383826_at	303754	Rab40b_pr	Rab40b, member RAS oncogene fami	2346.721	2481.377	8029.189
1376359_at	361733	Ms4a8b_pr	membrane-spanning 4-domains, subfa	2345.548	1768.17	844.2195
1376578_at	362078	Ehmt1_pre	euchromatic histone methyltransferase	2344.206	2374.123	2068.683
1388909_at	306270	Oxnad1_pr	oxidoreductase NAD-binding domain c	2343.982	2023.727	2554.105
1389136_at	308845	Prkrir_pred	protein-kinase, interferon-inducible dou	2343.411	2893.494	2295.064
1386946_at	25757	Cpt1a	carnitine palmitoyltransferase 1a, liver	2342.937	2139.563	3032.052
1367483_at	298147	RGD13091	similar to RIKEN cDNA 3110001D03 (p	2339.709	2643.78	2038.122
1382191_at	300772	Pias1_prec	protein inhibitor of activated STAT 1 (p	2337.011	1246.283	3159.89
1373804_at	297480	Foxp1	forkhead box P1	2336.226	1192.626	3205.405
1390435_at	361111	RGD13075	LOC361111 (predicted)	2333.119	1526.722	1028.679
1373426_at	116590	Mapk1	mitogen activated protein kinase 1	2332.613	3875.731	3743.296
1368549_at	27080	Hbp1	high mobility group box transcription fa	2332.236	2387.536	3370.913
1395523_at	302855	LOC30285	similar to heterogeneous nuclear ribon	2332.16	3812.814	4948.241
1373719_at	170920	Map4k3	mitogen-activated protein kinase kinas	2331.243	2740.524	4605.468
1370919_at	116655	Hnrpm	heterogeneous nuclear ribonucleoprote	2330.849	4169.421	4482.99
1372061_at	296649	Rabepk	Rab9 effector protein with kelch motifs	2330.666	2133.334	1188.729
1374457_at	366697	Pomt2	protein-O-mannosyltransferase 2	2329.861	1183.879	961.6812
1367864_at	65152	Pfkm	phosphofructokinase, muscle	2328.21	8444.997	1866.833
1373600_at	300173	LOC30017	similar to CG9996-PA	2327.657	2689.472	3848.444
1371658_at	361425	Cox4nb	COX4 neighbor	2327.656	2742.784	969.7792
1373136_at	294390	RGD13076	hypothetical LOC294390	2327.407	2127.037	2555.539
1372363_at	291795	RGD15659	similar to polymerase (RNA) II (DNA d	2326.301	3326.548	2453.746
1373334_at	305458	RGD13108	similar to RIKEN cDNA 0610039G24 g	2326.208	2077.587	1367.444
1373004_at	116456	Dnajc2	DnaJ (Hsp40) homolog, subfamily C, r	2325.325	2164.357	1316.805
1371487_at	298544	Sh3bgrl3_p	SH3 domain binding glutamic acid-rich	2324.724	2343.444	1383.794
1389578_at	319113	Isrip	ischemia/reperfusion inducible protein	2324.36	2423.853	2071.227
1374296_at	311846	Lrrc8	leucine-rich repeat-containing 8	2324.014	3737.395	2397.271
1374701_at	360967	MGC10945	similar to 1700022N24Rik protein	2322.075	844.737	1021.4
1376519_at	315094	Brp16	brain protein 16	2321.659	1457.116	1430.477
1399127_at	361485	RGD13093	LOC361485	2321.508	1164.514	3308.819
1373685_at	361149	RGD15657	similar to low density lipoprotein recep	2321.443	5233.997	3607.855
1388883_at	361698	Pold4	polymerase (DNA-directed), delta 4	2321.363	4347.672	988.5943
1374074_at	293886	Cdc37l1	cell division cycle 37 homolog (S. cere	2320.993	2551.381	2610.451
1368854_at	24877	Vsnl1	visinin-like 1	2320.933	3569.039	7342.662
1372210_at	288557	Mospd3	motile sperm domain containing 3	2316.946	2351.416	1086.691
1383260_at	308992	RGD13059	similar to RIKEN cDNA G630024C07 g	2316.794	2643.605	1644.576
1372853_at	309165	Rela	v-rel reticuloendotheliosis viral oncoge	2314.956	1731.566	4015.075
1371582_at	310612	LOC31061	NA	2311.143	3789.68	3159.08
1372033_at	361501	RGD13090	similar to RIKEN cDNA 0610042E07	2310.891	271.9699	283.7068
1399021_at	171552	Pprf18	PRP18 pre-mRNA processing factor 1	2309.422	2671.529	4545.334
1390408_at	317444	Hccs_pred	holocytochrome c synthetase (predicte	2309.219	2093.406	2700.95
1398920_at	298536	NA	NA	2308.484	2647.537	853.0164

1367542_at	313519	LOC31351	similar to RIKEN cDNA 5330440M15	2308.344	2403.56	1912.882
1371779_at	362738	Snx6_pred	sorting nexin 6 (predicted)	2306.441	4545.505	1501.771
1371955_at	297334	Mrpl35_pre	mitochondrial ribosomal protein L35 (p	2305.605	3394.293	2062.473
1372828_at	361286	Msrb2	methionine sulfoxide reductase B2	2304.687	3965.469	1337.088
1393713_at	291095	Gmds	GDP-mannose 4, 6-dehydratase	2304.608	4241.688	966.1685
1368221_at	24413	Nr3c1	nuclear receptor subfamily 3, group C,	2304.371	5328.984	13401.82
1370420_at	24950	Srd5a1	steroid 5 alpha-reductase 1	2304.369	1780.188	549.8262
1388832_at	288525	Dhrsx_pred	dehydrogenase/reductase (SDR family	2303.969	3373.661	1287.326
1369654_at	78975	Prkaa2	protein kinase, AMP-activated, alpha 2	2302.227	3007.218	160.5582
1373112_at	304285	RGD13068	similar to RIKEN cDNA 2210010N04 g	2300.972	1311.666	997.4496
1372303_at	299909	RGD13076	similar to 0910001A06Rik protein (pred	2300.909	5434.619	8696.788
1389124_at	360603	RGD13106	similar to hypothetical protein MGC153	2299.75	3050.291	1979.251
1390794_at	291990	Cog8_pred	component of oligomeric golgi complex	2298.679	725.6041	1226.234
1372562_at	362946	MGC94207	similar to RIKEN cDNA C030006K11	2297.901	1690.962	836.0245
1375093_at	362706	Rbks_pred	ribokinase (predicted)	2296.707	1434.834	806.0264
1371679_at	499702	RGD15647	similar to Synaptopodin-2 (Myopodin) (	2295.326	3621.314	3821.953
1399087_at	360673	RGD13074	similar to hypothetical protein FLJ3152	2294.498	1828.928	1880.047
1367788_at	140671	Phkg2	phosphorylase kinase, gamma 2 (testis	2294.316	1479.448	1598.524
1385806_at	363032	Rp9h_pred	retinitis pigmentosa 9 homolog (human	2293.205	2521.513	2539.695
1368189_at	64191	Dhcr7	7-dehydrocholesterol reductase	2292.299	9347.481	1369.558
1372122_at	292925	Tsg101	tumor susceptibility gene 101	2291.728	3967.256	4195.815
1392495_at	315864	RGD13055	similar to dJ202D23.2 (novel protein si	2291.254	1141.645	3058.326
1381779_s_at	246136	Man2c1	mannosidase, alpha, class 2C, membe	2288.51	893.6695	4739.635
1379664_at	305955	RGD13077	similar to KIAA0853 protein (predicted)	2287.277	558.1441	1349.919
1387212_at	25334	Bhlhb8	basic helix-loop-helix domain containin	2287.187	445.6191	310.9297
1368071_at	171451	Mosc2	MOCO sulphurase C-terminal domain	2285.573	494.8092	883.6765
1372893_at	298312	Yipf1	Yip1 domain family, member 1	2285.437	5082.371	1577.053
1398785_at	29417	Men1	multiple endocrine neoplasia 1	2285.226	3199.412	756.8873
1388601_at	306960	Abt1	activator of basal transcription 1	2283.771	5102.812	852.4927
1368806_at	29360	Sepp1	selenoprotein P, plasma, 1	2282.271	11457.61	77708.85
1371785_at	302965	Tnfrsf12a	tumor necrosis factor receptor superfa	2280.923	1235.374	2009.935
1371541_at	288057	Mylk_predi	myosin, light polypeptide kinase (predi	2280.137	10471.28	984.6689
1379262_at	302640	LOC30264	similar to acyl-CoA thioesterase	2279.298	6321.341	2585.184
1374210_at	361645	RGD13077	similar to RIKEN cDNA 2510027N19	2277.841	2991.233	1152.861
1392986_at	29149	Strn	striatin, calmodulin binding protein	2277.68	3400.445	3169.785
1371882_a_at	89817	Sh2bpsm1	SH2-B PH domain containing signaling	2274.652	1567.01	1851.039
1368095_at	26956	Ak3	adenylate kinase 3	2274.515	3946.213	1265.641
1368669_at	54315	Ucp2	uncoupling protein 2 (mitochondrial, pr	2273.548	4844.7	2512.293
1371676_at	366984	Lass5_pred	longevity assurance homolog 5 (S. cer	2273.057	4150.593	1639.559
1388755_at	58817	Sec23a_pr	SEC23A (S. cerevisiae) (predicted)	2272.462	638.1397	4108.567
1388648_at	293454	RGD13066	similar to D7Wsu128e protein	2271.366	2020.349	4354.32
1390789_at	315973	Acad11_pr	acyl-Coenzyme A dehydrogenase fam	2271.317	29.53622	1095.953
1373491_at	295241	LOC29524	similar to Metaxin 1, isoform 2	2270.237	4059.893	829.4431
1398769_at	29474	Coro1b	coronin, actin-binding protein, 1B	2268.788	5187.477	791.6826
1377647_at	362581	Scmh1_pre	sex comb on midleg homolog 1 (predic	2267.313	2626.992	1347.129
1383723_at	361828	RGD13095	similar to mKIAA0376 protein	2266.938	2687.773	2715.185
1376164_at	290666	Sf4	splicing factor 4	2265.145	2084.873	1048.587
1382565_at	361028	RGD13114	similar to MAPK-interacting and spindle	2264.643	1053.803	4896.095
1384907_at	306096	LOC30609	NA	2264.387	4346.837	11563.86
1389362_at	362524	Ptpn3	protein tyrosine phosphatase, non-rece	2262.87	1422.37	833.0159
1389496_at	361458	Akap7	A kinase (PRKA) anchor protein 7	2262.299	9427.508	1553.61

1389091_at	363084	Usp3	ubiquitin specific peptidase 3	2261.984	2107.42	2412.664
1372928_at	500289	NA	NA	2261.651	4640.151	6180.955
1383624_at	498014	RGD1565(	similar to hypothetical protein LOC284	2261.281	6400.868	6189.078
1398924_at	499934	MGC1091	similar to mannosidase, beta A, lysoso	2261.015	4119.785	1294.391
1373938_at	360497	Wdr24	WD repeat domain 24	2259.462	1170.922	888.7344
1389361_at	363544	LOC36354	similar to 2610111M03Rik protein	2256.253	2614.8	2826.443
1383282_at	307806	Thap11_pr	THAP domain containing 11 (predictec	2255.408	1750.943	1852.844
1392472_at	309957	LOC30995	similar to myocyte enhancer factor 2C	2254.885	2232.633	1815.475
1382856_at	299828	LOC29982	NA	2254.683	6120.524	2029.55
1368082_at	24780	Slc4a2	solute carrier family 4, member 2	2253.715	1859.25	1094.964
1367934_at	25347	Rpl39	ribosomal protein L39	2253.605	1012.874	869.0971
1390384_at	500987	RGD15661	similar to Histone H2A.x (H2a/x) (predi	2253.327	2524.267	2368.844
1388121_at	64312	Aplp2	amyloid beta (A4) precursor-like protei	2252.897	4796.844	11547.34
1394876_at	314681	Ric8b	resistance to inhibitors of cholinesteras	2252.579	618.1255	1227.601
1367883_at	64301	Smn1	survival of motor neuron 1, telomeric	2251.172	2441.734	1643.01
1376833_at	313021	RGD13047	similar to hypothetical protein DKFZp5	2251.009	2020.967	2368.231
1377166_at	363235	Als2	amyotrophic lateral sclerosis 2 (juvenil	2250.526	1210.634	4729.911
1368133_at	29365	Mpdz	multiple PDZ domain protein	2249.836	1018.488	6707.715
1392956_at	296550	RGD1304ε	similar to RIKEN cDNA 2810443J12 (p	2248.581	1520.597	829.941
1380827_at	304851	RGD1307ε	similar to C1orf25	2247.35	1052.528	1898.292
1367702_at	24158	Acadm	acetyl-Coenzyme A dehydrogenase, m	2246.37	2794.796	4490.589
1392743_at	266670	Mina	myc induced nuclear antigen	2246.334	3857.699	1634.28
1392461_at	298095	Prpf4	PRP4 pre-mRNA processing factor 4 h	2246.314	3735.134	1748.91
1373607_at	64445	St3gal3	ST3 beta-galactoside alpha-2,3-sialyltr	2245.596	2452.643	1223.664
1384551_at	309326	Ranbp6_pr	RAN binding protein 6 (predicted)	2245.57	4545.604	6046.415
1389366_at	308991	RGD1561б	similar to Zinc finger protein 553 (predi	2245.302	2004.847	1664.09
1370162_at	140943	Ppp4r1	protein phosphatase 4, regulatory subu	2244.502	1648.912	1371.32
1372245_at	29231	Wdr39	WD repeat domain 39	2243.508	3493.311	1194.437
1373096_at	361107	Actr8_pred	ARP8 actin-related protein 8 homolog	2240.447	5446.268	2856.531
1373371_a_at	500087	MGC1128ξ	similar to RIKEN cDNA 1110001J03	2240.06	5515.888	2967.119
1367748_at	79117	Arf5	ADP-ribosylation factor 5	2239.977	5642.211	1451.707
1377270_a_at	500305	RGD15622	similar to decapping enzyme Dcp1b (p	2239.316	2334.435	2303.503
1387946_at	245955	Lgals3bp	lectin, galactoside-binding, soluble, 3 b	2239.177	7224.31	2603.202
1368019_at	56718	Frap1	FK506 binding protein 12-rapamycin a	2238.359	1481.477	1542.827
1377673_at	500715	LOC50071	NA	2237.967	5545.429	2451.656
1392488_at	307526	RGD1307ξ	similar to hypothetical protein MGC321	2237.742	3333.95	4328.661
1379278_at	290322	RGD1311ξ	similar to hypothetical protein FLJ1087	2237.678	1382.952	1922.441
1371993_at	313087	Cpne3_pre	copine III (predicted)	2237.072	2899.485	9441.648
1390529_at	361226	Cd83_pred	CD83 antigen (predicted)	2232.95	929.7526	10502.3
1393418_at	58814	Tmod2	tropomodulin 2	2232.742	929.8148	746.0075
1372423_at	292949	Perp_predi	PERP, TP53 apoptosis effector (predic	2232.267	3497.293	2404.556
1373668_at	292778	Polr2i_prec	polymerase (RNA) II (DNA directed) pc	2231.976	4976.762	2963.208
1396820_at	297893	Hdac1_pre	histone deacetylase 1 (predicted)	2231.497	13837.79	751.5403
1373893_at	291689	Cdc23	CDC23 (cell division cycle 23, yeast, h	2231.427	2804.488	1960.599
1385713_at	299569	Akap8l	A kinase (PRKA) anchor protein 8-like	2231.416	1028.102	1730.926
1388518_at	362081	Fbxw5	F-box and WD-40 domain protein 5	2230.962	1969.886	2150.418
1373811_at	365951	RGD1560ξ	similar to heat shock protein 8 (predict	2229.36	1542.089	881.195
1370175_a_at	171018	Zfp384	zinc finger protein 384	2228.751	3259.882	2133.076
1373094_at	361580	Gtf2h1_pre	general transcription factor II H, polype	2228.217	1969.404	4516.655
1374195_at	313325	Lad1_pred	ladinin (predicted)	2228.082	1435.145	374.8377
1389979_at	296954	Tnpo3	transportin 3	2227.901	1953.444	2181.075

1371607_at	367171	LOC36717	microtubule-associated protein 4	2226.813	1064.157	5939.171
1374632_at	360665	Ptdsr	phosphatidylserine receptor	2225.674	2588.943	2000.556
1389068_at	288498	Wipi2	WD repeat domain, phosphoinositide i	2225.384	916.1829	1584.131
1392925_at	296911	ST7	suppression of tumorigenicity 7	2224.602	175.4117	319.5904
1389679_at	500026	RGD15636	similar to testhymin (predicted)	2223.438	3825.047	12069.88
1392891_at	304218	Aprin_pred	androgen-induced proliferation inhibito	2221.377	3097.19	4189.404
1379476_at	295692	Nup35	nucleoporin 35	2221.104	1986.468	2506.575
1378740_at	304893	Rasal2_pre	RAS protein activator like 2 (predicted)	2220.805	937.6148	863.82
1373557_at	29728	Mcm4	minichromosome maintenance deficien	2219.522	3924.797	3566.668
1372074_at	294292	Nudt3	nudix (nucleotide diphosphate linked m	2218.769	2048.162	1421.877
1367747_at	64664	Arl3	ADP-ribosylation factor-like 3	2218.453	7845.253	5960.93
1373948_at	360505	RGD13086	similar to CGTHBA protein (-14 gene p	2218.231	1684.43	1292.367
1376585_at	362517	Mrpl50_pre	mitochondrial ribosomal protein L50 (p	2217.641	4471.456	1767.658
1374031_at	300759	RGD13109	similar to hypothetical protein (predicte	2216.56	2231.241	2130.29
1374945_at	314462	RGD13591	GCD14/PCMT domain containing prote	2213.917	1210.713	421.0616
1384330_at	303067	LOC30306	similar to hypothetical protein FLJ2054	2213.187	2357.849	774.48
1388108_at	171402	Elovl6	ELOVL family member 6, elongation of	2213.028	1849.119	1547.963
1383140_at	361840	Spock2_pre	sparc/osteonectin, cwcv and kazal-like	2212.954	522.9592	262.5411
1373442_at	362891	Os-9	amplified in osteosarcoma	2212.784	1745.154	818.8642
1382653_at	362763	RGD13067	similar to CG15929-PA (predicted)	2212.497	522.2096	2306.215
1368099_at	114514	Clasp2	CLIP associating protein 2	2211.763	1164.82	3284.505
1367522_at	501661	RGD15660	similar to Factor VIII associated protein	2210.679	3219.195	2597.356
1389065_at	307956	Rbm34	RNA binding motif protein 34	2210.645	1509.028	1830.185
1388637_at	316328	RGD13092	similar to RIKEN cDNA 4632411B12	2208.831	1650.468	1760.711
1371808_at	287552	Blmh	bleomycin hydrolase	2206.356	7800.489	1996.117
1372839_at	297727	Mrps35_pre	mitochondrial ribosomal protein S35 (p	2206.076	2314.758	2621.458
1376852_at	294972	Mccc1	methylcrotonoyl-Coenzyme A carboxyl	2205.365	1136.247	1793.584
1368152_at	170906	Zdhhc7	zinc finger, DHHC domain containing 7	2205.123	2164.679	1225.514
1391434_at	25531	Rab3a	RAB3A, member RAS oncogene family	2204.347	4840.245	1164.681
1379188_at	366980	Arid2_pred	AT rich interactive domain 2 (Arid-rfx li	2201.109	1720.367	1120.066
1382114_at	311118	Tlk1_predi	tousled-like kinase 1 (predicted)	2200.629	1678.827	5253.551
1372923_at	310682	Pex11b	peroxisomal biogenesis factor 11b	2200.201	2399.7	1618.778
1371896_at	288916	Gadd45gip	growth arrest and DNA-damage-induci	2198.875	1146.817	1675.432
1371836_at	287709	Rab5c_pre	RAB5C, member RAS oncogene famil	2198.755	1995.413	1199.428
1386883_at	50686	Gsk3a	glycogen synthase kinase 3 alpha	2196.605	1418.948	1339.192
1373589_at	305482	Mtmr3	myotubularin related protein 3	2195.922	3093.924	3466.023
1372305_at	360611	Copz2_pre	coatomer protein complex, subunit zeta	2195.403	635.0276	822.9327
1369029_at	117540	Plscr1	phospholipid scramblase 1	2195.311	74.56567	1346.723
1395404_at	303755	RGD13045	similar to Hypothetical protein 9030012	2195.294	2922.753	6089.251
1368096_at	171122	Rab7l1	RAB7, member RAS oncogene family-	2194.785	1378.129	3245.535
1389400_at	690911	LOC69091	NA	2194.705	775.9593	6558.626
1380110_at	24514	Jak2	Janus kinase 2	2194.071	2467.172	2913.384
1389227_at	308875	Rhog	Ras homolog gene family, member G	2193.85	1831.337	1361.597
1387978_at	245925	LOC24592	CTD-binding SR-like protein rA9	2193.711	1468.365	2151.169
1375135_at	304538	Gcn1l1_pre	GCN1 general control of amino-acid sy	2192.647	2239.223	243.8307
1389171_at	306327	Tmem38a_	transmembrane protein 38a (predicted	2191.867	4157.945	1250.785
1374669_at	309016	LOC30901	NA	2189.56	2877.379	2628.564
1382085_at	363289	Mterfd2	MTERF domain containing 2	2189.553	998.9638	1121.905
1381445_at	360896	Esrrg	estrogen-related receptor gamma	2187.887	1020.434	887.1676
1381982_at	296560	Uap1l1_pre	UDP-N-acteylglucosamine pyrophosph	2187.826	722.916	624.8342
1372289_at	311122	RGD15611	similar to solute carrier family 25 (mito	2187.687	1929.165	3321.098

1390271_at	360842	Plekha6_pr	pleckstrin homology domain containing	2187.183	1620.17	1780.247
1378119_at	311414	Zc3h8	zinc finger CCCH type containing 8	2186.959	1475.312	1853.434
1368606_at	80900	Slco1a5	solute carrier organic anion transporter	2186.682	137.2711	173.1139
1378022_at	313216	Rnf20_pred	ring finger protein 20 (predicted)	2186.656	3757.947	3889.351
1373623_at	308451	Itpkc	inositol 1,4,5-trisphosphate 3-kinase C	2186.43	386.161	789.566
1379323_at	311800	RGD13063	similar to hypothetical protein MGC143	2185.875	4042.766	1488.12
1388677_at	362502	Ubap1	ubiquitin-associated protein 1	2184.946	2137.929	2638.705
1371733_at	498234	RGD15659	similar to ADP-ribosylation factor-like 1	2184.796	2639.998	2739.793
1373618_at	362453	Crebl2	cAMP responsive element binding prot	2183.755	926.5374	1093.562
1371455_at	300089	Pmm1	phosphomannomutase 1	2182.392	3995.028	919.5471
1388502_at	362590	Inpp5b	inositol polyphosphate-5-phosphatase	2181.276	2619.528	2810.291
1373653_at	307050	Papd1_pre	PAP associated domain containing 1 (	2180.03	2736.651	1911.144
1374661_at	361571	LOC36157	similar to RIKEN cDNA 2410004H02	2179.835	2801.251	606.8733
1398582_at	314384	Rps6ka5_p	ribosomal protein S6 kinase, polypepti	2179.168	567.4326	1028.386
1389664_at	315988	Rbm15b_p	RNA binding motif protein 15B (predict	2178.74	1105.567	2175.353
1368614_at	171078	Wbscr14	Williams-Beuren syndrome chromosom	2178.249	5239.257	489.1095
1389087_at	296558	Anapc2	anaphase promoting complex subunit	2177.657	3995.835	2516.997
1389299_at	140544	Pcyt1a	phosphate cytidylyltransferase 1, choli	2177.532	1573.721	2726.68
1368010_at	116689	Ptpn6	protein tyrosine phosphatase, non-rece	2177.033	997.9088	815.9336
1390292_at	303004	Tmem8_pr	transmembrane protein 8 (five membra	2176.667	3071.974	1405.091
1387818_at	114555	Casp4	caspase 4, apoptosis-related cysteine	2175.601	74.28403	249.2486
1381966_at	361475	NA	NA	2174.98	842.7319	416.9266
1398355_at	114497	Trpm7	transient receptor potential-related pro	2174.584	3016.703	9905.483
1394854_at	297758	Terf1	telomeric repeat binding factor 1	2174.361	1722.639	3098.939
1371889_at	305886	Slc22a17	solute carrier family 22 (organic cation	2174.181	3620.493	17726.93
1370869_at	29592	Bcat1	branched chain aminotransferase 1, cy	2173.97	9.870241	90.72729
1384792_at	295607	Prpf40a_pr	pre-mRNA processing factor 40 homol	2173.744	1164.808	669.5976
1374202_at	296599	RGD13115	similar to chromosome 9 open reading	2172.942	1393.823	758.5205
1387907_at	25262	Itpr1	inositol 1,4,5-triphosphate receptor 1	2172.93	7186.438	15186.95
1378433_at	310200	RGD15642	similar to RIKEN cDNA 5730557B15 (p	2172.165	249.3956	712.4057
1373572_at	296462	LOC29646	similar to GTP binding protein 5	2171.261	2649.331	1538.634
1399078_at	299607	Thrap5_pre	thyroid hormone receptor associated p	2170.557	2774.843	809.451
1376440_at	315000	Rnf139_pre	ring finger protein 139 (predicted)	2168.797	5314.464	5778.676
1368526_at	83519	Pex3	peroxisomal biogenesis factor 3	2168.661	2491.658	2502.154
1368589_at	29645	Ptprj	protein tyrosine phosphatase, receptor	2168.473	1852.972	258.2989
1371897_at	296635	RGD13058	similar to RIKEN cDNA D830019K17	2167.657	2038.321	1239.114
1370261_at	81771	Rps6ka1	ribosomal protein S6 kinase polypeptic	2164.627	2460.653	811.4581
1373747_at	361705	Cnih2	cornichon homolog 2 (Drosophila)	2163.593	5206.035	2300.233
1376602_a_at	300724	Fbxo22	F-box only protein 22	2162.002	1169.955	1641.519
1377300_at	498003	RGD15600	similar to Dual specificity protein phosp	2161.958	1289.171	373.2024
1384956_at	499060	RGD15662	similar to hypothetical protein FLJ1434	2161.22	2160.453	2612.004
1390381_at	312560	Xpc_predic	xeroderma pigmentosum, complement	2160.47	2112.634	2093.731
1380513_at	305120	RGD13090	similar to Expressed sequence AW060	2160.301	3061.239	3719.776
1372584_at	364208	RGD13083	similar to DKFZP566K1924 protein	2159.414	5478.043	3883.297
1382332_at	313304	Stag2_pred	stromal antigen 2 (predicted)	2159.383	5977.902	3065.841
1374764_at	289724	RGD13056	similar to 2610033H07Rik protein (pre	2159.155	1552.402	1112.244
1373946_at	296758	RGD13059	similar to RIKEN cDNA 2810037C14	2159.136	2167.369	2099.971
1393572_at	293038	Zfp592_pre	zinc finger protein 592 (predicted)	2158.64	1646.119	5155.607
1390535_at	291414	Ctdp1_pred	CTD (carboxy-terminal domain, RNA p	2157.562	3166.814	2950.928
1368790_at	171154	Serpina10	serine (or cysteine) peptidase inhibitor	2157.296	221.5834	2496.381
1379765_at	363942	RGD15614	similar to nemo like kinase (predicted)	2157.244	1367.552	293.0747

1369017_at	116745	Kcnh6	potassium voltage-gated channel, subf	2156.818	1596.916	3672.69
1377992_at	300980	Dusp7	dual specificity phosphatase 7	2155.349	1118.5	3828.797
1371600_at	266709	Pkig	protein kinase inhibitor, gamma	2153.914	2737.858	4849.625
1383156_at	84391	Kif2	kinesin heavy chain family, member 2	2153.742	5360.692	3037.192
1373169_at	306582	Agpat5_pre	1-acylglycerol-3-phosphate O-acyltrans	2153.697	3027.073	1338.43
1373292_at	309001	RGD13116	similar to KIAA0339 protein	2152.934	1204.188	1158.04
1375084_at	313057	Serinc2	serine incorporator 2	2150.984	1639.458	534.1376
1374467_at	287069	Trap1	TNF receptor-associated protein 1	2150.168	4781.049	1793.81
1387792_at	65278	Csnk1g2	casein kinase 1, gamma 2	2150.091	1601.76	1326.56
1379876_at	293456	Cog7	component of oligomeric golgi complex	2149.458	2445.217	1605.556
1368879_a_at	50664	Gnao	guanine nucleotide binding protein, alp	2148.735	2924.129	1254.244
1368268_at	114521	Tdg	thymine-DNA glycosylase	2147.669	7521.06	1921.124
1373965_at	290642	RGD13109	similar to hypothetical protein BC0139	2146.411	2802.232	1442.371
1387897_at	25275	Cnp1	cyclic nucleotide phosphodiesterase 1	2145.773	2169.967	1578.226
1398908_at	298203	Stoml2	stomatin (Epb7.2)-like 2	2145.39	3843.859	1203.821
1372784_at	362566	Lrrc41	leucine rich repeat containing 41	2145.293	2166.067	1081.911
1390755_at	365243	RGD15634	similar to Gnefr protein (predicted)	2144.856	2337.438	1283.65
1375858_at	363138	Tusc4	tumor suppressor candidate 4	2144.851	1934.747	2058.021
1373077_at	287278	RGD13086	similar to hypothetical protein D11Ertd	2142.821	2785.2	1108.964
1393397_at	296959	Cpa2_pred	carboxypeptidase A2 (pancreatic) (pre	2141.055	1272.76	10912.3
1372946_at	292328	RGD15627	similar to RIKEN cDNA 1110012L19 (p	2138.878	2838.321	3060.836
1389357_at	289736	Zcwcc1_pr	zinc finger, CW-type with coiled-coil dc	2137.276	2993.682	1766.432
1397460_at	686523	LOC68652	NA	2134.257	60.73802	2640.934
1374534_at	287156	Rhot2	ras homolog gene family, member T2	2132.589	1457.126	894.6307
1371672_at	362962	Cbx7	chromobox homolog 7	2132.424	856.843	2681.551
1376104_at	316764	RGD13083	similar to KIAA0802 protein (predicted)	2130.923	751.9738	983.4182
1372008_at	363521	Taz_mappe	tafazzin (cardiomyopathy, dilated 3A (X	2130.394	2178.458	1108.947
1389090_at	282835	Wrnip1	Werner helicase interacting protein 1	2129.727	2779.426	2620.183
1391152_at	363306	LOC36330	hypothetical protein LOC363306	2126.805	254.8117	3102.598
1375058_at	501554	NA	NA	2125.99	919.5986	1813.189
1372043_at	298586	RGD13117	similar to ribosomal protein P0-like pro	2125.579	1210.628	708.2563
1398279_at	116469	Trpv5	transient receptor potential cation char	2124.272	964.2541	992.7637
1383273_a_at	294336	Pcbp3	poly(rC) binding protein 3	2122.974	4022.329	1148.194
1372253_at	363603	Cnot8	CCR4-NOT transcription complex, sub	2121.656	3308.424	1903.861
1373742_at	297592	Spsb2	splA/ryanodine receptor domain and S	2121.366	1234.89	1369.956
1388901_at	361810	Fkbp5	FK506 binding protein 5	2120.337	3214.368	3027.747
1372365_at	311494	Rin2_predi	Ras and Rab interactor 2 (predicted)	2119.122	1828.538	4516.539
1372656_at	293691	Snx15	sorting nexin 15	2118.578	2863.741	1968.81
1389249_at	293184	Sh2bp1	SH2 domain binding protein 1 (tetratric	2117.916	1846.553	3146.307
1381999_at	296580	RGD15650	similar to calmodulin regulated spectrir	2117.864	2555.761	2025.443
1389056_at	288914	Trmt1	TRM1 tRNA methyltransferase 1 homo	2117.772	1132.506	1913.664
1387964_a_at	171562	Ero1l	ERO1-like (S. cerevisiae)	2117.685	313.3546	694.777
1399093_at	293589	LOC29358	putative GTP-binding protein	2117.48	2889.053	2217.369
1371347_at	360698	Tmem50b	transmembrane protein 50B	2115.365	2767.086	3698.132
1390418_at	316333	Actr1b	ARP1 actin-related protein 1 homolog	2115.025	2943.383	996.0848
1388517_at	287962	Mrpl40	mitochondrial ribosomal protein L40	2114.202	2635.03	770.3015
1376753_at	310935	Fpgt	fucose-1-phosphate guanylyltransferas	2113.35	1257.604	3717.511
1373069_at	294767	Mrps30_pr	mitochondrial ribosomal protein S30 (p	2112.581	5165.715	3417.352
1383653_at	303493	Snx11	sorting nexin 11	2111.452	1367.553	452.4823
1368801_at	83824	Cxxc4	CXXC finger 4	2111.42	589.2953	516.6903
1370005_at	80773	Cyb5b	cytochrome b5 type B	2111.307	3034.978	1009.695

1373083_at	619549	Ppapdc2	phosphatidic acid phosphatase type 2	2111.215	3841.207	1940.1
1399054_at	498138	NA	NA	2109.665	4917.828	1508.828
1372798_at	654495	LOC65449	NA	2109.415	6208.552	1391.303
1388044_at	24640	Pfkfb2	6-phosphofructo-2-kinase/fructose-2,6	2109.19	3026.758	3078.472
1387021_at	64394	Wig1	wild-type p53-induced gene 1	2108.676	1985.921	1989.739
1374204_at	303336	Wsb1	WD repeat and SOCS box-containing	2108.056	2594.586	19737.55
1388434_at	300258	C12orf10	MYG1 protein	2107.91	3285.568	1487.811
1373484_at	313445	Klhl13	kelch-like 13 (Drosophila)	2107.906	1948.065	2505.765
1388934_at	296159	Vps16	vacuolar protein sorting 16 (yeast)	2107.553	3410.245	1802.062
1383431_at	305579	RGD13051	similar to KIAA1841 protein (predicted)	2107.439	5583.747	3244.593
1382643_at	64088	Snx16	sorting nexin 16	2106.699	1261.639	4564.482
1370061_at	81755	Rab3b	RAB3B, member RAS oncogene family	2106.562	2909.723	6083.247
1379422_at	300284	LOC30028	similar to RIKEN cDNA 4833435D08	2106.388	4565.01	3210.975
1389313_at	64520	Mta1	metastasis associated 1	2106.056	1773.01	1850.072
1383570_at	363210	Phf3_predi	PHD finger protein 3 (predicted)	2106.036	2042.529	3615.003
1373258_at	361704	Ctsf	cathepsin F	2105.918	4377.342	4201.958
1387147_at	171058	Rab3c	RAB3C, member RAS oncogene famil	2104.925	5184.146	1308.341
1395318_at	680231	LOC68023	NA	2104.244	457.0103	323.9533
1393082_at	171010	Ppp1r14c	protein phosphatase 1, regulatory (inhi	2103.653	401.2568	940.6126
1389431_at	360748	Prkdc_prec	protein kinase, DNA activated, catalytic	2103.122	1182.308	1551.017
1375955_at	362277	Zfp313	zinc finger protein 313	2102.111	4003.284	1822.773
1392865_at	25444	Fgf9	fibroblast growth factor 9	2101.337	96.53368	1034.035
1371568_at	113893	Sncb	synuclein, beta	2101.061	11007.88	1418.923
1367720_at	25374	Alad	aminolevulinate, delta-, dehydratase	2099.933	2633.409	573.4837
1377713_at	314374	Ches1_pre	checkpoint suppressor 1 (predicted)	2099.743	817.4082	3676.197
1382340_at	498863	NA	NA	2099.383	699.6802	790.9058
1383171_at	289307	Tfb2m	transcription factor B2, mitochondrial	2098.541	1168.233	1863.12
1374357_at	353304	Cdc91l1	CDC91 cell division cycle 91-like 1 (S.	2098.454	1444.38	988.0148
1373338_at	363327	Vmac	vimentin-type intermediate filament ass	2096.925	1938.764	1632.416
1394441_at	291860	RGD13088	similar to SPla/RYanodine receptor SP	2095.745	1816.047	1850.256
1373033_at	292305	MGC10550	similar to chromosome 6 open reading	2092.099	2603.658	1951.769
1379279_at	117087	Stim2_prec	stromal interaction molecule 2 (predicte	2091.919	1923.776	3579.442
1372207_at	291691	Brd8	bromodomain containing 8	2091.417	4426.549	7453.56
1374321_at	499309	RGD15601	similar to RIKEN cDNA 2700081O15 (	2090.199	6110.926	3848.211
1376450_at	299841	Tmem5	transmembrane protein 5	2089.749	2265.983	1760.921
1388877_at	296134	Mrps5_pre	mitochondrial ribosomal protein S5 (pre	2088.999	4963.406	1410.197
1372134_at	297436	Chchd6_pr	coiled-coil-helix-coiled-coil-helix domai	2088.46	4360.213	1572.587
1389876_at	287005	Camk2n1	calcium/calmodulin-dependent protein	2088.359	1478.694	1077.661
1376113_at	311631	Zswim1_pr	zinc finger, SWIM domain containing 1	2088.029	1241.963	1285.339
1382903_at	310958	LOC31095	NA	2086.948	1537.538	5171.404
1371480_at	686524	LOC68652	NA	2086.244	6850.277	401.5484
1372114_at	498839	NA	NA	2085.947	2489.644	3051.864
1367458_at	83510	Lypla2	lysophospholipase 2	2085.454	1548.753	531.0088
1374230_at	313061	RGD15599	similar to mKIAA1429 protein (predicte	2083.758	2869.108	2153.021
1392640_at	299691	Cry1	cryptochrome 1 (photolyase-like)	2082.089	1867.924	7958.167
1372792_at	292780	LOC29278	similar to hypothetical protein MGC156	2081.609	1503.324	769.3188
1373980_at	500619	NA	NA	2081.599	2710.73	951.8322
1377051_at	360463	Mpv17l_pre	Mpv17 transgene, kidney disease muta	2081.23	8024.752	495.8633
1372540_at	360514	Ublcp1	ubiquitin-like domain containing CTD p	2080.445	5093.245	3122.866
1368424_at	84351	Ikbkb	inhibitor of kappaB kinase beta	2080.304	1520.443	1138.261
1383350_at	311637	Slc35c2	solute carrier family 35, member C2	2078.696	1263.657	1042.697

1395899_at	501438	RGD1565C	similar to GTPase activating protein te	2078.641	1016.157	1650.659
1390205_at	310062	Zswim6	zinc finger, SWIM domain containing 6	2076.452	3201.285	5588.228
1375987_at	300129	Cerk_predi	ceramide kinase (predicted)	2076.066	1895.503	1304.366
1374686_at	310801	Slc30a7	solute carrier family 30 (zinc transporte	2075.51	1030.718	1362.53
1388429_at	499391	LOC49939	NA	2075.504	2003.624	994.4922
1367779_at	117028	Bin1	bridging integrator 1	2074.123	2093.394	1380.586
1398446_at	310640	RGD13061	similar to chromosome 1 open reading	2074.055	4087.354	1876.808
1393148_at	289287	Gpr137b_p	G protein-coupled receptor 137B (prec	2073.71	1682.609	1464.065
1376131_a_at	360552	Pelp1	proline, glutamic acid and leucine rich	2073.506	1748.392	1714.591
1399084_at	294232	Dhx16	DEAH (Asp-Glu-Ala-His) box polypepti	2073.244	2570.615	1018.926
1368500_a_at	29481	Rgs9	regulator of G-protein signaling 9	2071.833	1918.044	958.8757
1367676_at	29395	Hmgb2	high mobility group box 2	2070.189	22465.69	7341.518
1382965_at	316003	Amigo3	amphoterin induced gene and ORF 3	2067.197	396.9426	888.2108
1388584_at	315160	RGD13057	similar to D15Wsu75e protein	2066.336	5508.685	2898.764
1373553_at	287930	Top3b_pre	topoisomerase (DNA) III beta (predicte	2065.992	1828.867	1065.439
1377656_at	306526	Rbm13	RNA binding motif protein 13	2065.538	1921.997	1067.508
1375254_at	313139	Slc35a1_pi	solute carrier family 35 (CMP-sialic aci	2063.227	3399.031	1573.042
1370894_at	65132	Cldn7	claudin 7	2061.374	31.04127	410.4573
1374804_at	362593	Gnl2	guanine nucleotide binding protein-like	2060.294	3041.276	2421.238
1371578_at	25636	Prkaca	protein kinase, cAMP-dependent, cata	2060.227	1813.978	1030.56
1377436_at	292810	Gpatc1_pre	G patch domain containing 1 (predicte	2059.756	1452.237	2097.357
1394422_at	310982	RGD15661	similar to hypothetical protein FLJ2303	2059.099	2380.275	1441.773
1398354_at	298019	Ctnnal1_pr	catenin (cadherin associated protein),	2058.99	3367.915	2721.484
1373009_at	298384	RGD15597	similar to RIKEN cDNA 0610037L13	2057.621	2235.966	978.201
1374650_at	291044	Nedd9	neural precursor cell expressed, devel	2056.136	1453.274	4276.772
1373603_at	360494	RGD15657	similar to RIKEN cDNA 0610007P22 (ı	2056.108	1970.944	1302.861
1388730_at	303653	Cdc42ep4_	CDC42 effector protein (Rho GTPase	2056.045	1522.812	2276.778
1375701_at	360568	LOC36056	NA	2055.812	2599.275	1617.014
1372482_at	295928	Slc39a13	solute carrier family 39 (zinc transporte	2054.57	1847.114	1515.999
1374882_at	289928	RGD1306C	similar to HT021 (predicted)	2054.027	1387.91	2009.658
1391776_at	363162	RGD13052	similar to RIKEN cDNA 2010110K16 (ı	2053.077	2946.825	2617.05
1379369_at	315259	Prickle1	prickle-like 1 (Drosophila)	2051.631	1734.772	4107.402
1376720_at	312258	Adck2_pre	aarF domain containing kinase 2 (pred	2049.483	693.0479	656.6643
1372050_at	290637	Glt25d1_pr	glycosyltransferase 25 domain contain	2048.969	3623.506	862.9435
1383697_at	114507	Slc5a3	solute carrier family 5 (inositol transpo	2048.68	336.7079	16742.19
1373427_at	297960	Rragd_pre	Ras-related GTP binding D (predicted)	2048.53	637.2727	1649.427
1373060_at	363720	RGD13067	similar to CGI-84 protein (predicted)	2046.468	5518.058	3650.485
1373467_at	368042	RGD15641	similar to TBP-associated factor 172 (T	2045.267	3622.507	4787.874
1376668_at	298366	RGD13111	similar to RIKEN cDNA 4922503N01 (ı	2043.925	2710.459	2500.325
1372680_at	308309	Leng4_pre	leukocyte receptor cluster (LRC) meml	2042.655	2421.322	2440.15
1375412_at	25227	Arsb	arylsulfatase B	2042.226	7658.569	17598.67
1373375_at	308942	Rab6ip1_p	Rab6 interacting protein 1 (predicted)	2042.036	3823.266	2798.224
1383822_at	306809	Bicd2	bicaudal D homolog 2 (Drosophila)	2041.819	1798.781	2133.089
1370910_at	116468	Rfc2	replication factor C (activator 1) 2	2040.813	8090.611	1289.84
1380665_at	316122	LOC31612	CGI-58-like protein	2040.683	1505.095	2552.969
1372142_at	288919	Asna1	arsA arsenite transporter, ATP-binding	2040.301	2705.292	969.1791
1373953_at	298805	Slc4a1ap_	solute carrier family 4 (anion exchange	2039.005	2899.394	2453.126
1383399_at	117559	Sv2a	synaptic vesicle glycoprotein 2a	2038.229	1522.19	3049.983
1388980_at	308869	MGC7256C	Unknown (protein for MGC:72560)	2037.051	2370.679	1151.676
1376788_at	306722	Dapk1_pre	death associated protein kinase 1 (pre	2036.634	3960.277	5990.62
1383528_at	299305	RGD15639	similar to mKIAA0215 protein (predicte	2036.447	3571.777	3678.278

1371668_at	25271	Rxra	retinoid X receptor alpha	2036.271	1505.491	1674.253
1375967_a_at	361242	Dusp22_pr	dual specificity phosphatase 22 (predic	2034.426	2277.815	3582.424
1376605_at	363103	Slc17a5	solute carrier family 17 (anion/sugar tra	2033.81	780.0416	2153.099
1373702_at	300320	L3mbtl2	l(3)mbt-like 2 (Drosophila)	2033.179	2154.379	1587.546
1389041_at	307842	Vac14	Vac14 homolog (S. cerevisiae)	2033.02	1237.961	1127.722
1373201_at	29611	Dbt	dihydrolipoamide branched chain trans	2031.305	404.1389	2749.405
1376431_at	291673	Sil1	endoplasmic reticulum chaperone SIL1	2031.227	2724.204	1233.971
1387446_at	65044	C1galt1	core 1 UDP-galactose:N-acetylgalacto	2030.733	1084.514	808.1373
1384016_at	500790	RGD15636	similar to R31449_3 (predicted)	2030.627	1592.832	604.2107
1390360_a_at	301126	LOC30112	NA	2030.355	919.9195	1863.553
1367863_at	140932	Bnip1	BCL2/adenovirus E1B 19kDa-interactin	2029.644	3420.403	1214.009
1371963_at	54280	Pcca	propionyl-coenzyme A carboxylase, alp	2029.366	612.115	2291.961
1373706_at	316477	Smarcal1_	Swi/SNF related matrix associated, act	2029.226	1350.299	804.6012
1375532_at	25587	Id2	inhibitor of DNA binding 2	2029.092	1466.45	14725.83
1370187_at	24624	Pccb	propionyl coenzyme A carboxylase, be	2028.628	2064.02	1624.846
1398983_at	301352	Mrpl30_pre	mitochondrial ribosomal protein L30 (p	2027.355	4292.074	1951.278
1370903_a_at	296318	LOC29631	similar to Ndr3 protein	2026.339	6654.499	1005.349
1389200_at	359727	Bysl	bystin-like	2025.816	1121.131	722.0218
1372350_at	294385	Supv3l1	suppressor of var1, 3-like 1 (S. cerevis	2025.737	2724.074	2163.777
1372014_at	296612	RGD15649	similar to cofactor required for Sp1 tran	2023.571	4684.634	2267.289
1374305_at	290215	Ap1g2_pre	adaptor protein complex AP-1, gamma	2022.387	891.5632	449.7244
1373339_at	363003	NA	NA	2022.373	2172.033	2629.61
1386976_at	83628	Cd82	CD82 antigen	2022.323	1336.618	868.8806
1373791_at	299612	RGD13591	similar to RIKEN cDNA 2310011J03	2021.783	1963.472	1131.301
1399107_at	297971	RGD13051	similar to RIKEN cDNA 1810030N24 (p	2021.782	2994.972	2238
1383508_at	295041	Stoml3_pre	stomatin (Epb7.2)-like 3 (predicted)	2020.097	1554.301	1393.166
1382179_at	500629	RGD15651	similar to alcohol dehydrogenase PAN	2019.364	2194.242	2529.758
1374014_at	353233	Nsmaf	neutral sphingomyelinase (N-SMase) a	2018.924	1799.841	5631.664
1373238_at	360874	Tada1l	transcriptional adaptor 1 (HFI1 homolo	2018.647	1942.72	4955.223
1375922_at	309391	Cox15	COX15 homolog, cytochrome c oxidas	2018.484	4180.844	3420.72
1390779_at	361661	RGD15643	similar to phosphoseryl-tRNA kinase (p	2017.92	1893.833	9907.942
1368214_at	29357	Smad2	MAD homolog 2 (Drosophila)	2017.655	5613.964	1729.745
1373034_at	288233	Wrb	tryptophan rich basic protein	2017.397	3497.863	1265.602
1392770_at	81735	Neo1	neogenin	2017.3	2396.139	3845.615
1377945_at	308490	Ddx18	DEAD (Asp-Glu-Ala-Asp) box polypept	2016.215	1870.682	851.6185
1371591_at	301249	Mrps18a	mitochondrial ribosomal protein S18A	2015.663	7322.724	1366.864
1387126_at	170699	Atp2c1	ATPase, Ca++-sequestering	2015.004	946.9368	1364.58
1397758_at	362865	RGD15648	similar to mKIAA1208 protein (predicte	2014.284	2040.21	1433.797
1390501_at	311731	Scrn3	secernin 3	2012.717	2072.321	2588.435
1389202_at	501157	MGC12465	similar to Ribulose-5-phosphate-3-epir	2012.716	1610.823	2771.347
1392207_at	360503	Luc7l	LUC7-like (S. cerevisiae)	2012.544	2232.826	1548.754
1380548_at	303968	Ift57_predi	intraflagellar transport 57 homolog (Ch	2012.374	4377.998	3297.477
1373597_at	288564	Pop7_pred	processing of precursor 7, ribonucleas	2010.848	6708.105	1176.132
1387316_at	81503	Cxcl1	chemokine (C-X-C motif) ligand 1	2010.284	10.96003	6106.284
1368326_at	29702	Eif2ak3	eukaryotic translation initiation factor 2	2009.882	1446.312	7619.341
1373452_at	309301	Rcl1	RNA terminal phosphate cyclase-like 1	2009.767	3219.101	1258.801
1372837_at	305956	Wdfy2_pre	WD repeat and FYVE domain containi	2009.536	769.6204	1238.659
1371813_at	361650	Hirip3	HIRA interacting protein 3	2009.398	3286.56	2095.316
1367977_at	29219	Snca	synuclein, alpha	2008.986	1405.581	1565.147
1376835_at	316241	Slc35b2	solute carrier family 35, member B2	2008.792	1148.63	3026.247
1388483_at	366624	Cfl2_predic	cofilin 2, muscle (predicted)	2008.557	2732.408	2805.626

1374470_at	366532	Dhx57	DEAH (Asp-Glu-Ala-Asp/His) box poly	2008.28	1391.05	1933.065
1391410_at	290964	NA	NA	2007.173	7338.44	2916.723
1392513_at	361182	RGD1307(	similar to hypothetical protein FLJ1130	2007.086	2258.982	444.2656
1374010_at	303369	Lig3	ligase III, DNA, ATP-dependent	2002.026	2562.607	1186.739
1372051_at	360793	Rhbdl7_pre	rhomboid, veinlet-like 7 (Drosophila) (p	2001.555	2234.27	535.9018
1388306_at	288513	RGD13055	similar to RIKEN cDNA 1810042K04 (p	2001.122	8514.587	752.4534
1382082_at	25336	Nfyb	nuclear transcription factor-Y beta	2000.031	2780.07	5950.259
1373155_at	293054	Mrpl46	mitochondrial ribosomal protein L46	1998.421	2153.229	1273.813
1383616_at	304091	RGD15603	similar to class II cytokine receptor 4 (p	1998.279	1310.092	1236.884
1377638_at	296466	Bwk1	Bwk1 leukemia-related gene	1997.625	2065.477	2095.287
1384423_at	294016	Nt5c2_prec	5'-nucleotidase, cytosolic II (predicted)	1997.036	6887.751	797.7862
1379544_at	301447	RGD13113	similar to A530083I02Rik protein (pred	1996.494	195.8139	992.2301
1370358_at	246215	Tpcn1	two pore channel 1	1996.188	3052.298	1675.518
1377194_a_at	308820	RGD13086	similar to RIKEN cDNA 2310015N07	1995.983	6543.886	2385.636
1370901_at	296565	RGD13062	similar to hypothetical protein MGC368	1992.315	2861.295	1333.939
1377938_at	302941	RGD13594	hypothetical LOC302941	1992.277	1473.296	3463.175
1372030_at	362789	Zfyve21_pr	zinc finger, FYVE domain containing 2	1992.196	1119.685	526.994
1386918_a_at	29336	Oprs1	opioid receptor, sigma 1	1988.998	2914.364	646.4565
1373509_at	298426	Nsun4_pre	NOL1/NOP2/Sun domain family, mem	1988.385	2430.191	1174.262
1370232_at	24513	Ivd	isovaleryl coenzyme A dehydrogenase	1987.167	3378.508	633.0687
1383125_at	287953	Htf9c	HpaII tiny fragments locus 9c	1986.013	2222.977	1371.436
1388957_at	499427	NA	NA	1985.828	3431.812	2483.572
1388674_at	114851	Cdkn1a	cyclin-dependent kinase inhibitor 1A	1985.361	733.2261	1671.83
1372176_at	24680	Prkca	protein kinase C, alpha	1984.864	518.6329	390.1257
1391559_at	287472	Tlcd1	TLC domain containing 1	1983.207	1804.907	1924.346
1388544_at	296973	Bpgm	2,3-bisphosphoglycerate mutase	1983.199	4075.153	2070.784
1393450_at	360829	RGD13101	similar to Ab2-034	1983.001	2400.481	3580.318
1370989_at	24716	Ret	ret proto-oncogene	1981.999	2852.033	8234.148
1372157_at	365875	Bola1_prec	bolA-like 1 (E. coli) (predicted)	1981.097	2451.514	1356.736
1392920_at	296102	Ell3	elongation factor RNA polymerase II-lil	1980.503	1256.471	1027.737
1373194_at	313128	Casp8ap2_	caspase 8 associated protein 2 (predic	1979.672	5264.885	1972.933
1389061_at	288595	Nsun5_pre	NOL1/NOP2/Sun domain family, mem	1979.53	3044.457	914.2259
1371987_at	306672	Pols_predi	polymerase (DNA directed) sigma (pre	1979.501	5255.232	6137.558
1391269_at	317366	NA	NA	1979.188	5442.996	4670.259
1368515_at	116724	Epb4.1l3	erythrocyte protein band 4.1-like 3	1978.931	2074.951	3616.808
1386972_at	29515	Atn1	atrophin 1	1976.806	791.554	2090.145
1388533_at	363249	Ctdsp1	CTD (carboxy-terminal domain, RNA p	1976.041	702.7877	1457.508
1389009_at	361956	Rsrc1	arginine/serine-rich coiled-coil 1	1975.164	7315.963	2339.558
1369981_at	58845	Igbp1	immunoglobulin (CD79A) binding prote	1974.332	2784.941	4655.977
1389207_at	308913	Egln1	EGL nine homolog 1 (C. elegans)	1973.654	1030.418	1051.434
1376492_at	302987	RGD15652	similar to chromosome 16 open readin	1971.494	2425.648	1029.527
1383224_at	362279	Pard6b_pre	par-6 (partitioning defective 6) homolo(	1971.279	1532.581	3070.641
1382769_at	288264	Ifnar1_prec	interferon (alpha and beta) receptor 1 (	1969.768	2077.896	1686.246
1372950_at	54400	Bet1l	blocked early in transport 1 homolog (S	1968.905	1609.183	1037.131
1389067_at	171144	Slco4a1	solute carrier organic anion transporter	1967.239	2819.042	701.3847
1378977_at	362575	Med8_prec	mediator of RNA polymerase II transcr	1966.687	1204.515	839.8164
1382070_at	312800	Atf7ip_prec	activating transcription factor 7 interact	1965.466	2303.063	1680.54
1398341_at	287661	RGD15597	RGD1559720 (predicted)	1964.176	1026.229	880.4961
1383342_at	500900	MGC10888	similar to Leucine-rich repeat-containir	1963.726	1023.59	1250.068
1368399_a_at	58952	Pgcp	plasma glutamate carboxypeptidase	1963.292	22.09276	1899.428
1376260_at	295428	RGD13048	similar to KIAA1627 protein (predicted)	1962.82	1535.706	3255.032

1373737_at	367328	ORF19	open reading frame 19	1962.21	1213.362	1596.185
1373449_at	307927	Taf5l	TAF5-like RNA polymerase II, p300/CI	1961.468	1352.499	1431.05
1367943_at	81525	Nfkbib	nuclear factor of kappa light chain gen	1960.946	996.4755	1392.71
1374716_at	360895	RGD13061	similar to RIKEN cDNA 2810430M08	1960.005	830.658	442.4509
1372902_at	315212	Alg12_pred	asparagine-linked glycosylation 12 hor	1959.793	3112.051	917.474
1367967_at	114200	Lepre1	leprecan 1	1959.224	471.2559	421.2669
1375989_a_at	309452	Nfkb2	nuclear factor of kappa light polypeptic	1959.149	225.4879	860.2119
1383118_at	312200	RGD13096	similar to hypothetical protein FLJ1480	1959.045	2364.594	1217.337
1389993_at	307524	Wdr33_pre	WD repeat domain 33 (predicted)	1958.01	1215.364	1167.185
1387783_a_at	24157	Acaa1	acetyl-Coenzyme A acyltransferase 1	1957.148	1556.695	1263.648
1380185_at	313433	RGD15645	similar to hypothetical protein FLJ2296	1956.987	2970.115	3539.493
1376990_at	361044	RGD13093	similar to HCDI protein (predicted)	1956.247	724.9409	4561.405
1383996_at	306328	Slc35e1_pr	solute carrier family 35, member E1 (p	1955.125	3847.554	2681.104
1367577_at	24471	Hspb1	heat shock 27kDa protein 1	1954.416	9.455881	1331.074
1374112_at	499280	RGD15654	similar to MAPK-interacting and spindl	1953.937	1593.078	1942.458
1370257_at	29526	Pla2g1b	phospholipase A2, group IB	1953.644	335.2054	6164.855
1379816_at	304572	RGD15633	similar to RIKEN cDNA 2410025L10 (p	1952.672	1479.739	5499.282
1373046_at	362836	Dazap1	DAZ associated protein 1	1951.712	1906.59	890.384
1367701_at	58966	Ramp2	receptor (calcitonin) activity modifying	1951.656	1294.163	1023.774
1377657_at	501029	NA	NA	1949.93	806.3656	2857.572
1381201_at	365657	Marveld2_p	MARVEL (membrane-associating) dom	1948.671	1263.173	695.4775
1378791_at	308106	Map3k4_pr	mitogen activated protein kinase kinas	1948.481	2547.147	3202.041
1368899_at	81507	Bmpr1a	bone morphogenetic protein receptor,	1946.038	2231.26	1333.713
1375346_at	500993	RGD15639	similar to hypothetical protein FLJ2001	1945.401	1755.912	1067.496
1382603_at	309304	Pdcd1lg2_p	programmed cell death 1 ligand 2 (pre	1945.18	581.4531	7996.168
1374557_at	362065	LOC36206	CG6210-like	1944.969	3209.662	1082.214
1389137_at	83620	Cit	citron	1944.739	1050.703	1125.786
1373586_at	500518	NA	NA	1944.261	2746.266	1634.355
1372578_at	25009	Vars2	valyl-tRNA synthetase 2	1943.173	1314.947	1290.174
1388316_at	296207	RGD15663	RGD1566320 (predicted)	1942.163	4274.368	2323.692
1387026_at	63996	Smc1l1	structural maintenance of chromosome	1940.491	4590.338	2568.259
1374671_at	361857	MGC1128	NA	1940.37	529.9598	749.5845
1389034_at	312688	Usp18	ubiquitin specific peptidase 18	1940.214	433.9662	817.9054
1374048_at	84423	Nrtn	neurturin	1938.382	1234.497	256.3291
1395958_at	365869	RGD13082	similar to KIAA0460 protein (predicted)	1938.05	1145.029	1256.818
1367911_at	114096	Idh3a	isocitrate dehydrogenase 3 (NAD+) alp	1937.969	1992.298	1752.571
1383806_at	361249	RGD15663	similar to BAF57 (predicted)	1936.991	6086.415	1911.432
1368538_at	64632	Exoc7	exocyst complex component 7	1936.836	3156.447	629.2537
1398336_at	301515	Rnf25	ring finger protein 25	1936.533	1146.102	1087.112
1382345_at	314743	Pctk2	PCTAIRE-motif protein kinase 2	1936.099	2205.57	7043.272
1372557_at	363760	Arl6_predic	ADP-ribosylation factor-like 6 (predicte	1935.663	5665.417	5530.147
1391078_at	89809	Recc1	replication factor C 1	1935.069	2654.896	423.5086
1392481_at	363221	RGD15609	similar to DNA segment, Chr 1, Brigha	1935.047	1957.502	1474.192
1377303_at	302981	Spsb3_pre	splA/ryanodine receptor domain and S	1933.614	1000.211	2330.309
1374205_at	498407	pur-beta	transcription factor Pur-beta	1932.978	1888.647	2182.223
1372643_at	309557	RGD15639	similar to protein 4.1G (predicted)	1929.585	5381.959	6534.652
1373639_at	290925	RGD15656	similar to RIKEN cDNA 2410022L05 (p	1928.707	2620.772	4124.275
1374461_at	304291	Zdhhc4	zinc finger, DHHC domain containing 4	1926.695	2517.606	803.8874
1373748_at	312607	Pdzrn3_pre	PDZ domain containing RING finger 3	1926.213	1204.496	1634.982
1393492_at	499348	NA	NA	1925.65	1107.344	5004.126
1370303_at	257647	Slc35a4	solute carrier family 35, member A4	1925.285	3171.527	861.4377

1370521_at	64060	Vps33b	vacuolar protein sorting 33B (yeast)	1924.623	2020.674	385.6077
1368953_at	171129	Ugcgl1	UDP-glucose ceramide glucosyltransfe	1923.979	2501.948	156.3814
1373789_at	316087	RGD15624	similar to zinc finger protein 651 (predi	1923.55	1655.756	692.1852
1374462_at	289168	Kifap3_pre	kinesin-associated protein 3 (predicted	1921.276	1698.532	1389.285
1372087_at	298917	Harpb64	hypertrophic agonist responsive protei	1919.83	8808.831	2711.817
1389451_at	362942	RGD15604	similar to G protein-coupled receptor 1	1918.865	1073.227	431.7638
1368070_at	59074	Stx8	syntaxin 8	1918.221	1861.055	1631.996
1388889_at	373544	Fxna	putative aminopeptidase Fxna	1917.697	3663.967	1484.664
1375580_at	301130	LOC30113	NA	1916.967	1189.355	965.9659
1373741_at	304567	Pus1	pseudouridine synthase 1	1915.617	1435.164	750.73
1381162_at	309891	LOC30989	similar to septin 10 isoform 1	1915.028	3532.242	4968.706
1373616_at	499369	NA	NA	1914.432	2903.501	3101.707
1390264_at	360563	RGD13081	similar to RIKEN cDNA 1200014J11 (p	1914.194	3019.808	2403.497
1380867_a_at	303584	Plekhm1	pleckstrin homology domain containing	1914.023	1644.035	1808.374
1367541_at	502632	RGD15660	similar to 2810410A08Rik protein (pred	1914.021	2279.123	3434.592
1389639_at	362513	RGD15653	similar to Shb protein (predicted)	1912.857	1566.244	2708.908
1374980_at	304656	RGD13075	similar to RIKEN cDNA 4930527D15	1912.818	578.4626	665.1282
1379775_at	299347	Brf1_predi	BRF1 homolog, subunit of RNA polyme	1912.195	1398.432	1173.49
1391086_at	292464	Katna1	katanin p60 (ATPase-containing) subu	1911	3001.479	1909.942
1389446_at	292997	Snrpa1_pre	small nuclear ribonucleoprotein polype	1910.938	4414.219	3027.777
1390534_at	293967	Smc5l1_pr	SMC5 structural maintenance of chrom	1910.051	5463.059	7063.759
1398708_at	499614	NA	NA	1909.511	1539.276	1088.872
1375645_at	500575	RGD15602	similar to DNA segment, Chr 4, ERATO	1909.16	1213.551	657.3758
1378326_at	366012	LOC36601	similar to RIKEN cDNA G430055L02	1908.597	2581.753	788.2662
1376595_at	500006	RGD15599	similar to peroxisome biogenesis facto	1908.006	3691.665	1925.166
1389117_at	290028	Osgep	O-sialoglycoprotein endopeptidase	1906.034	1371.974	3066.798
1375915_at	300862	Irak1bp1_p	interleukin-1 receptor-associated kinas	1905.692	3191.423	7664.049
1367881_at	25528	Ptpns1	protein tyrosine phosphatase, non-rece	1904.808	1905.771	759.2823
1371881_at	309177	Znhit2_pre	zinc finger, HIT domain containing 2 (p	1904.403	1549.485	1051.264
1388363_at	296301	Raly	hnRNP-associated with lethal yellow	1903.554	5338.814	1303.618
1390488_a_at	361813	Stk38	serine/threonine kinase 38	1902.949	2433.207	1278.306
1388973_at	305104	Col9a1	procollagen, type IX, alpha 1	1902.776	3012.254	11320.32
1388594_at	312638	Creld1	cysteine-rich with EGF-like domains 1	1901.942	2208.571	780.6377
1389923_at	294318	Btbd9	BTB (POZ) domain containing 9	1901.414	680.6994	867.6767
1371012_at	85255	Phyh2	phytanoyl-CoA 2-hydroxylase 2	1901.402	1717.794	925.4884
1390531_at	296474	RGD13060	similar to Peroxisomal proliferator-activ	1898.522	424.4539	2712.878
1376121_at	292404	Phf10	PHD finger protein 10	1898.449	1461.4	1889.92
1398493_at	316457	LOC31645	NA	1898.341	1060.473	4696.335
1377244_at	304275	Zfp95_pred	zinc finger protein 95 (predicted)	1898.214	1894.605	2001.116
1376134_at	288912	RGD13077	similar to hypothetical protein MGC320	1897.948	1330.667	1036.055
1369007_at	54278	Nr4a2	nuclear receptor subfamily 4, group A,	1897.818	527.7855	14002.58
1393354_at	360560	RGD13047	similar to 4933427D14Rik protein (pred	1896.951	1861.098	2679.505
1374119_at	304815	Elf3	E74-like factor 3	1896.698	1052.732	958.9846
1367740_at	24264	Ckb	creatine kinase, brain	1895.795	403.664	768.8887
1388769_at	360491	Traf7_pred	Tnf receptor-associated factor 7 (predi	1895.471	1860.823	1062.291
1373150_at	305685	Comtd1_pr	catechol-O-methyltransferase domain	1895.437	790.9534	834.226
1374555_at	289125	Acbd6	acyl-Coenzyme A binding domain cont	1894.547	1534.945	3463.813
1383281_at	287918	RGD13062	similar to RIKEN cDNA 1110005A03	1893.801	1836.263	3196.113
1371431_at	312703	Pex5_pred	peroxisome biogenesis factor 5 (predic	1893.664	2214.011	3122.461
1369665_a_at	29197	Il18	interleukin 18	1893.538	663.5768	365.8648
1376407_a_at	362829	Lsm7_pred	LSM7 homolog, U6 small nuclear RNA	1893.249	3417.272	1671.109

1390893_at	619582	Tmem69	transmembrane protein 69	1892.458	2893.948	2612.951
1371037_at	81750	Pros1	protein S (alpha)	1892.331	4192.273	2610.656
1397676_at	362447	RGD15631	similar to osteoclast inhibitory lectin (pı	1889.526	184.6063	759.2689
1386971_at	65045	Ppp1r10	protein phosphatase 1, regulatory subı	1888.887	2116.924	849.3065
1377621_at	287398	Map2k4	mitogen activated protein kinase kinas	1888.795	4346.56	4218.302
1372734_at	300236	Smagp	small cell adhesion glycoprotein	1888.609	356.6331	2011.074
1399023_at	293614	Ric8a	resistance to inhibitors of cholinesteras	1887.157	1612.628	1936.269
1389445_at	293511	Zfp688_pre	zinc finger protein 688 (predicted)	1886.574	1844.232	834.2791
1389345_at	311264	RGD13107	similar to G2 (predicted)	1885.209	3983.515	3242.678
1373389_at	294973	Acad9	acyl-Coenzyme A dehydrogenase fam	1885.021	3411.91	1644.757
1381403_at	307270	Me2_predi	malic enzyme 2, NAD(+)-dependent, m	1884.569	2690.16	524.7659
1372924_at	497914	RGD1563€	similar to Mediator of RNA polymerase	1884.07	2292.516	1184.996
1385616_a_at	294408	Asf1a_prec	ASF1 anti-silencing function 1 homolog	1883.542	4658.635	1519.032
1390118_at	311166	RGD1307€	similar to ATP/GTP-binding protein	1881.625	2555.319	1166.461
1389300_at	365361	RGD15634	similar to RIKEN cDNA 4930404J24 (p	1881.506	1232.045	2037.567
1379829_at	298513	Mycbp_pre	c-myc binding protein (predicted)	1881.402	3478.041	3524.551
1390096_at	292156	Sh3glb1	SH3-domain GRB2-like B1 (endophilin	1881.148	1931.328	6763.994
1368094_at	79049	Secisbp2	SECIS binding protein 2	1881.117	2909.218	1588.065
1381294_at	309387	Cnnm1_pre	cyclin M1 (predicted)	1880.506	442.8874	710.9223
1373564_at	301384	Hibch	3-hydroxyisobutyryl-Coenzyme A hydr	1879.822	1742.523	2664.547
1368517_at	84354	Ssbp3	single stranded DNA binding protein 3	1877.688	1489.934	1822.084
1382858_at	306165	MGC9455	intimal thickness-related receptor	1875.365	1104.774	1465.986
1371857_at	494521	Kctd10	NA	1874.794	3310.731	1366.22
1377827_at	291469	Srfbp1	serum response factor binding protein	1873.535	1631.592	2297.141
1367893_a_at	170927	Clcc1	chloride channel CLIC-like 1	1873.44	1383.234	1268.718
1390801_at	361744	RGD13593	similar to hypothetical protein MGC347	1872.998	5086.141	754.0136
1387056_at	79257	Axin1	axin 1	1872.904	2848.028	1429.633
1391601_at	289020	Lrrn2_pred	leucine rich repeat protein 2, neuronal	1870.982	2815.749	1658.162
1386962_at	25031	Plcb4	phospholipase C, beta 4	1870.876	4748.898	5223.244
1389315_at	304546	Git2	G protein-coupled receptor kinase-inte	1870.031	3064.872	3379.571
1388941_at	360650	RGD13048	similar to X83328 protein	1868.21	3331.162	1007.985
1388387_at	362087	Ubadc1	ubiquitin associated domain containing	1866.94	2566.982	764.2734
1383430_at	312649	Tsen2	tRNA splicing endonuclease 2 homolo	1866.435	1482.274	907.2407
1393091_at	310086	RGD13112	similar to RIKEN cDNA D130064H19 (	1866.313	3937.117	11863.24
1390068_at	360610	Nfe2l1_pre	nuclear factor, erythroid derived 2,-like	1866.206	1168.488	751.9606
1390124_at	499866	RGD1564€	similar to hypothetical protein FLJ3842	1865.831	4822.511	3263.929
1384469_at	286970	Abca5	ATP-binding cassette, sub-family A (AI	1865.735	2412.054	1386.958
1381757_at	305552	RGD13095	hypothetical LOC305552 (predicted)	1865.321	1709.622	2284.872
1384327_at	246118	Tubb3	tubulin, beta 3	1864.723	1470.217	1434.413
1375659_at	361273	Sec61a2_p	Sec61, alpha subunit 2 (S. cerevisiae)	1864.676	3147.104	4055.128
1384172_at	314169	RGD13104	similar to KIAA0423 (predicted)	1863.934	1349.721	7246.397
1368126_at	65984	Aacs	acetoacetyl-CoA synthetase	1863.541	4388.353	2507.276
1388711_at	252963	Il13ra1	interleukin 13 receptor, alpha 1	1862.651	2741.531	4298.295
1384368_at	309762	Dna2l_prec	DNA2 DNA replication helicase 2-like (	1862.108	2612.634	3220.531
1385066_a_at	360811	RGD1305€	similar to hypothetical protein FLJ1308	1861.541	2172.569	2056.983
1393320_at	310019	RGD13109	similar to hypothetical protein FLJ1278	1861.125	2879.462	3769.643
1375191_at	499178	NA	NA	1860.041	1495.221	3072.424
1388631_at	316051	Azi2	5-azacytidine induced gene 2	1859.225	886.458	3891.267
1370344_at	266759	Hspa4	heat shock protein 4	1858.398	2006.995	743.3282
1388578_at	289378	LOC28937	NA	1857.272	789.3854	1120.068
1390185_at	266605	Dcps	decapping enzyme, scavenger	1857.039	3342.139	685.7335

1387654_at	65261	Myo1c	myosin IC	1856.579	1276.25	225.6504
1376040_at	361442	Sipa1l2	signal-induced proliferation-associated	1856.133	1305.013	2576.366
1393800_at	317403	NA	NA	1855.264	1074.538	5356.6
1391270_at	301345	Cnnm3_pre	cyclin M3 (predicted)	1855.145	3208.356	1243.37
1398429_at	311254	LOC31125	hypothetical protein LOC311254	1854.697	1724.639	1119.418
1393619_at	360917	Cnot6l_pre	CCR4-NOT transcription complex, sub	1853.589	3625.749	5108.596
1384925_at	290279	RGD13067	similar to RIKEN cDNA 2510005D08 (	1853.421	2990.966	3320.452
1367505_at	305127	Zfp644_pre	zinc finger protein 644 (predicted)	1852.24	4290.589	7615.444
1376812_at	363292	Ing5_predi	inhibitor of growth family, member 5 (p	1850.637	965.5257	1786.18
1372667_at	361740	RGD13591	similar to RIKEN cDNA 1110059E24	1850.454	4829.238	1594.397
1393276_at	287475	Med31_pre	mediator of RNA polymerase II transcr	1848.653	3547.428	8017.269
1387127_at	83526	Atrn	attractin	1847.491	2875.289	1495.238
1370302_at	192219	AF348365	thyroid hormone-response protein-1	1847.196	1622.878	1331.545
1390102_at	303902	Dirc2	disrupted in renal carcinoma 2 homolo	1846.031	5481.777	6944.238
1385335_at	309591	Trim39	tripartite motif protein 39	1845.189	622.2435	1237.659
1367829_at	140547	Echs1	enoyl Coenzyme A hydratase, short ch	1844.628	1709.285	1429.365
1383623_at	300470	Thyn1	thymocyte nuclear protein 1	1844.311	3084.903	952.6452
1398400_at	303605	Lyk5	protein kinase LYK5	1844.056	3226.132	843.6232
1372399_at	311796	RGD13078	similar to cofactor of BRCA1; negative	1843.09	2792.864	3523.292
1387670_at	25062	Gpd2	glycerol-3-phosphate dehydrogenase 2	1842.007	1184.613	1902.285
1371887_at	305373	RGD15644	similar to high mobility group protein h	1841.928	11379.69	1114.125
1372945_at	312154	Ing3	inhibitor of growth family, member 3	1841.106	2137.551	2402.775
1372675_at	288269	RGD13069	similar to RIKEN cDNA 1110004E09	1839.256	3499.858	1635.482
1399105_at	361065	Bin3	bridging integrator 3	1838.233	749.9104	1233.698
1397227_at	308134	Tmem35	transmembrane protein 35	1837.425	110.1069	455.358
1389027_at	301343	Lman2l_pr	lectin, mannose-binding 2-like (predict	1837.371	1561.841	1998.81
1379342_at	303164	Zfp692_pre	zinc finger protein 692 (predicted)	1837.254	1429.626	1944.316
1368591_at	81817	Usf2	upstream transcription factor 2	1834.563	1222.42	839.8938
1389183_at	288479	Pms2_pred	postmeiotic segregation increased 2 (S	1834.361	1925.123	1254.495
1382307_at	116670	Ppp1r12a	protein phosphatase 1, regulatory (inhi	1834.358	1310.817	848.4071
1375870_a_at	362138	Rbms1	RNA binding motif, single stranded inte	1834.097	690.0312	5429.97
1389744_at	499898	NA	NA	1833.77	2955.537	3799.945
1390026_at	293524	Bag3	Bcl2-associated athanogene 3	1833.2	3143.245	2324.236
1371594_at	288584	Fis1	fission 1 (mitochondrial outer membran	1831.11	6595.49	1853.891
1371923_at	361467	Aytl2_pred	acyltransferase like 2 (predicted)	1831.078	3440.931	2053.577
1399124_at	362643	RGD13104	similar to KIAA0090 protein (predicted)	1831.058	1073.02	3008.299
1373664_at	364032	Pigc	phosphatidylinositol glycan, class C	1828.006	3266.37	1814.237
1370818_at	64461	Decr2	2-4-dienoyl-Coenzyme A reductase 2,	1826.12	1036.483	1322.006
1388492_at	363599	Tnip1_pred	TNFAIP3 interacting protein 1 (predict	1825.447	3180.385	2187.827
1382161_at	293828	Mphosph10	M-phase phosphoprotein 10 (U3 small	1824.516	1498.582	1543.409
1372592_at	84581	Hdac6	histone deacetylase 6	1823.118	7729.773	3893.604
1374742_at	365216	Igsf4c_pre	immunoglobulin superfamily, member	1821.911	7282.88	758.4114
1379793_at	361888	RGD13053	similar to RIKEN cDNA 3110031B13	1821.532	1109.369	1029.872
1391205_at	304332	MGC12521	similar to hypothetical protein FLJ2039	1821.104	5920.898	1900.936
1371412_a_at	338475	Nrep	neuronal regeneration related protein	1820.643	14067.46	9854.043
1367997_at	65041	Clpb	ClpB caseinolytic peptidase B homolog	1820.013	1546.7	395.5357
1374107_at	282826	Elac2	elaC homolog 2 (E. coli)	1818.862	1534.061	667.3024
1367537_at	305468	RGD15609	similar to Clast4 protein (predicted)	1816.326	2177.865	3912.569
1384128_at	246295	LOC24629	glycine-, glutamate-, thienylcyclohexyl	1814.926	671.6049	2302.687
1377363_at	310632	Rab25_pre	RAB25, member RAS oncogene family	1813.643	130.2388	993.652
1392502_at	360886	Ahctf1_pre	AT hook containing transcription factor	1812.479	2000.385	3403.65

1389131_at	360629	Nt5c3l	5'-nucleotidase, cytosolic III-like	1810.943	3654.077	798.0306
1373198_at	362998	RGD1311(	similar to RIKEN cDNA 2810451A06	1810.258	8791.917	107.8819
1376805_at	304850	Rnf2	ring finger protein 2	1810.13	4253.968	7513.614
1390455_at	293050	Abhd2_pre	abhydrolase domain containing 2 (prec	1809.795	746.3905	351.8148
1373071_at	292787	RGD13089	similar to RIKEN cDNA 1810054G18 (	1808.814	1386.637	633.6068
1393034_at	293112	RGD15655	similar to tyrosine kinase-associated le	1808.593	3404.229	7020.199
1383465_at	312675	Fbxl14_pre	F-box and leucine-rich repeat protein 1	1808.402	1260.528	2252.38
1381469_a_at	304378	Perq1_pre(	PERQ amino acid rich, with GYF doma	1806.345	1325.082	452.0154
1370458_at	252941	Hdgfrp3	hepatoma-derived growth factor, relate	1805.356	3348.037	3096.616
1382725_at	363273	Cops7b_pr	COP9 (constitutive photomorphogenic	1803.686	1670.018	1057.368
1383077_at	297890	RGD13116	similar to RIKEN cDNA 1110063F24 (p	1803.61	1531.9	1772.762
1387244_at	116679	Cgrrf1	cell growth regulator with ring finger dc	1803.357	1281.255	1444.393
1384290_at	308968	Rbbp6	retinoblastoma binding protein 6	1802.731	5134.424	7134.006
1373552_at	296865	Las1l_pred	LAS1-like (S. cerevisiae) (predicted)	1801.957	3792.479	1739.892
1383341_at	290291	Cab39l	calcium binding protein 39-like	1801.466	695.9504	701.1441
1370113_at	78971	Birc3	baculoviral IAP repeat-containing 3	1800.503	370.6075	4090.662
1372879_at	292887	Akt1s1_pre	AKT1 substrate 1 (proline-rich) (predic	1800.339	2012.365	441.1322
1394097_at	65023	Kcnip1	Kv channel-interacting protein 1	1800.292	12893.72	3839.84
1389609_at	297725	Tm7sf3	transmembrane 7 superfamily member	1800.288	1761.449	923.4588
1378875_a_at	361789	RGD1303(	similar to RIKEN cDNA 2610110G12	1800.233	1138.178	2010.89
1379483_at	79431	Bhlhb2	basic helix-loop-helix domain containir	1800.201	7145.74	5284.05
1374847_at	303786	RGD1306(	similar to hypothetical protein FLJ3047	1800.079	1908.191	1075.806
1384002_at	361929	LOC36192	similar to Potential phospholipid-transp	1799.897	2449.977	4155.338
1376196_a_at	306030	Med4	mediator of RNA polymerase II transcr	1799.773	2597.382	1541.897
1391786_at	308650	LOC30865	similar to Murine homolog of human ftp	1797.626	1442.117	2694.535
1369726_at	25217	Tapbp	TAP binding protein	1797.415	1226.686	508.3195
1387024_at	116663	Dusp6	dual specificity phosphatase 6	1797.375	1024.2	923.4665
1371317_at	309447	Ldb1_pred	LIM domain binding 1 (predicted)	1796.801	3846.788	703.215
1389029_at	362092	Brd3_predi	bromodomain containing 3 (predicted)	1796.003	3093.114	2512.415
1386773_at	306262	Btd	biotinidase	1794.991	1058.261	345.0504
1398340_at	362201	Ccndbp1	cyclin D-type binding-protein 1	1794.33	5096.418	1700.812
1379378_at	289469	Mrps18c_p	mitochondrial ribosomal protein S18C	1793.945	2140.278	704.7577
1368012_at	64523	Tep1	telomerase associated protein 1	1792.348	630.2821	1698.861
1373660_at	361436	Afg3l1_pre	AFG3(ATPase family gene 3)-like 1 (y	1791.623	733.3511	682.3786
1389022_at	408200	Usp52	ubiquitin specific protease 52	1790.381	1802.255	2422.765
1370354_at	83507	Parg	poly (ADP-ribose) glycohydrolase	1790.088	1551.823	1998.542
1371555_at	363478	Snx12_pre	sorting nexin 12 (predicted)	1789.554	2187.616	603.615
1374108_at	314941	Lrp12_pre(	low density lipoprotein-related protein	1789.013	2305.932	1309.311
1371548_at	297459	Mrps25	mitochondrial ribosomal protein S25	1788.982	5873.993	1189.617
1382907_at	310721	LOC31072	similar to 4930431B09Rik protein	1787.296	1113.757	1021.687
1383839_at	295053	Spg20	spastic paraplegia 20, spartin (Troyer s	1786.655	1626.241	1298.03
1375486_at	317402	NA	NA	1786.04	1020.344	880.0504
1390189_at	298977	LOC29897	NA	1785.244	2276.588	2570.13
1373460_at	501103	Dsm-1	D-serine modulator-1	1784.322	3129.068	1205.762
1388803_at	288923	Dhps	deoxyhypusine synthase	1783.524	2015.379	877.0287
1369000_at	59109	Ntrk1	neurotrophic tyrosine kinase, receptor,	1783.02	3777.006	1654.919
1370809_at	252921	Tubg1	tubulin, gamma 1	1782.37	4670.62	1022.836
1387383_at	83633	Gabbr2	gamma-aminobutyric acid (GABA) B re	1781.859	3668.784	3489.221
1376598_at	311944	Paxip1_pre	PAX interacting (with transcription-acti	1781.608	3807.802	1474.426
1376561_at	305970	Fbxo16	F-box protein 16	1779.649	1343.717	1138.756
1369637_at	85248	Kif3c	kinesin family member 3C	1779.441	4186.293	1725.064

1377359_at	501665	LOC50166	similar to protein P3	1779.18	1013.128	672.79
1390973_at	295323	Trim45_pre	tripartite motif protein 45 (predicted)	1777.909	279.5862	776.2388
1368516_at	25167	Ptpra	protein tyrosine phosphatase, receptor	1776.958	1851.096	2058.732
1374807_at	305667	RGD13108	similar to RIKEN cDNA 1810063B07 g	1776.476	1125.569	809.4007
1373329_at	156435	Tmprss2	transmembrane protease, serine 2	1775.862	38.56691	319.9852
1382465_at	25330	Lipe	lipase, hormone sensitive	1774.812	611.5845	865.8954
1384295_at	307133	Hspa14	heat shock 70kDa protein 14	1774.468	5689.594	5834.931
1391648_at	288239	Dscr6_pred	Down syndrome critical region homolo	1773.595	8000.182	778.5987
1369209_at	171435	P34	p34 protein	1772.529	1706.343	216.1749
1370974_at	286932	Vps54	vacuolar protein sorting 54 (yeast)	1770.297	2543.233	2817.209
1383676_at	304431	Sfrs8	splicing factor, arginine/serine-rich 8 (s	1769.074	1735.126	1837.048
1398643_at	299284	Dicer1	Dicer1, Dcr-1 homolog (Drosophila)	1768.899	1721.832	909.8494
1376599_at	314993	Atad2_pred	ATPase family, AAA domain containing	1768.733	9844.679	1983.795
1388664_at	362938	RGD15650	similar to PDZ-domain protein scribble	1768.353	2367.688	1867.807
1373215_at	287537	Abr_predic	active BCR-related gene (predicted)	1767.045	1505.494	1699.548
1372824_at	362484	Plekhf2_pr	pleckstrin homology domain containing	1766.988	1674.624	4485.213
1388718_at	25566	Tmod1	tropomodulin 1	1766.594	1878.34	556.9151
1388646_at	287871	RGD13117	similar to p150 target of rapamycin (TC	1766.042	1585.775	917.8969
1379949_at	308140	Tfb1m	transcription factor B1, mitochondrial	1765.301	1240.585	875.9188
1388588_at	309170	Mtvr2	mammary tumor virus receptor 2	1764.982	5838.308	1854.717
1370252_at	171386	Avpi1	arginine vasopressin-induced 1	1764.604	1049.37	525.971
1379715_at	500042	RGD15662	similar to RIKEN cDNA 2610101N10 (p	1762.423	550.7249	1002.6
1379309_at	365755	Tbl1xr1_pr	transducin (beta)-like 1X-linked recept	1762.199	668.473	404.0909
1376580_at	499335	RGD15657	similar to frataxin (predicted)	1762.158	4963.806	1639.621
1389782_at	294499	RGD13055	similar to RIKEN cDNA 2010107G23 (	1761.404	9247.141	3433.369
1375536_at	29419	Numb	numb gene homolog (Drosophila)	1761.246	1138.176	2075.001
1374601_at	360697	Ifngr2_pred	interferon gamma receptor 2 (predicted	1761.234	2536.092	2447.157
1392988_at	309262	Nsdhl	NAD(P) dependent steroid dehydroger	1760.478	4363.843	1457.821
1383289_at	301431	Nif3l1	Ngg1 interacting factor 3-like 1 (S. pon	1760.466	1260.823	1426.485
1371570_at	366951	Scrt1_pred	scratch homolog 1, zinc finger protein	1760.111	1078.318	905.1604
1373038_at	361285	Mllt10	myeloid/lymphoid or mixed-lineage leu	1759.91	2306.68	2948.893
1368153_a_at	117536	Nelf	nasal embryonic LHRH factor	1759.533	2109.307	1201.756
1388326_at	293652	Ndufs8_pre	NADH dehydrogenase (ubiquinone) Fe	1758.902	5813.772	1836.142
1368235_at	171305	Clk3	CDC-like kinase 3	1758.687	1339.187	1462.349
1373141_at	316005	Arih2_pred	ariadne homolog 2 (Drosophila) (predi	1756.104	912.0822	1732.997
1368233_at	81674	Gtf2f2	general transcription factor IIF, polype	1755.205	1933.781	795.7208
1374678_at	293042	Sema4b	sema domain, immunoglobulin domain	1754.724	1062.808	1793.203
1372956_at	415064	Bat4	Bat4 gene	1753.994	1670.539	870.1423
1391739_at	303552	Rundc1_pr	RUN domain containing 1 (predicted)	1753.653	1166.708	321.712
1373709_at	289900	RGD13595	similar to KIAA0974 protein	1753.464	2376.2	3545.914
1390447_at	81802	Stx3	syntaxin 3	1752.754	1159.761	610.9704
1379393_at	316521	Vil1_predic	villin 1 (predicted)	1752.325	3897.092	405.7246
1370905_at	259237	Dock9	dedicator of cytokinesis 9	1751.509	1392.317	3878.289
1390620_at	312511	RGD13067	similar to Hypothetical protein MGC25	1750.998	2057.402	1951.061
1382041_at	294324	Agpat3_pre	1-acylglycerol-3-phosphate O-acyltran	1750.375	1598.915	592.3683
1389585_at	192183	Pvrl1	poliovirus receptor-related 1	1747.77	1113.507	827.9344
1389146_at	498796	LOC49879	hypothetical protein LOC498796	1747.539	3229.342	5905.207
1367644_at	25289	Adcy6	adenylate cyclase 6	1747.506	3247.596	3224.572
1395998_at	362214	Nol5a	nucleolar protein 5A	1745.078	2592.261	506.1324
1370181_at	25532	Rab4a	RAB4A, member RAS oncogene famil	1745.066	1471.891	1138.494
1398366_at	497968	NA	NA	1744.559	4479.434	3349.379

1399030_at	302559	Wdr45	WD repeat domain 45	1743.98	1345.352	2348.451
1377341_at	288545	RGD13054	similar to hypothetical protein FLJ1092	1743.392	1598.032	854.3933
1398342_at	501052	LOC50105	similar to Fus1 protein	1742.9	409.3991	443.0921
1389163_at	313264	Trim32	tripartite motif protein 32	1742.238	3687.274	2089.578
1399034_at	314288	Pcnx	pecanex homolog (Drosophila)	1741.481	1730.577	3283.588
1367771_at	83514	Tsc22d3	TSC22 domain family 3	1741.418	2639.352	6171.009
1372891_at	304330	RGD13056	similar to 2810437L13Rik protein (pred	1741.031	2424.18	1410.031
1377738_a_at	315097	Cyhr1	cysteine and histidine rich 1	1740.297	585.0711	1647.994
1374614_at	311185	Kbtbd4_pre	kelch repeat and BTB (POZ) domain c	1740.243	2972.079	1007.779
1374836_at	363134	Rnu3ip2_p	RNA, U3 small nucleolar interacting pr	1739.606	986.2958	794.0808
1372026_at	300240	RGD13593	similar to RIKEN cDNA 9430023L20	1737.476	2100.16	1002.193
1370975_at	312440	Jmjd1a	jumonji domain containing 1A	1737.253	2493.042	5830.423
1391703_at	295596	Orc4l	origin recognition complex, subunit 4-li	1734.115	2017.403	3957.532
1373472_at	314718	Actr6_pred	ARP6 actin-related protein 6 homolog	1733.684	3237.958	3118.549
1377571_at	361500	Zfp628_pre	zinc finger protein 628 (predicted)	1733.094	1317.35	840.4556
1375956_at	266713	Mnat1	menage a trois 1	1732.237	2733.984	2099.816
1378100_at	299810	Yeats4_pre	YEATS domain containing 4 (predicted	1730.997	3448.999	2757.129
1390851_at	297768	Lactb2	lactamase, beta 2	1730.126	1942.653	2172.562
1371523_at	498425	LOC49842	similar to U2 small nuclear ribonucleop	1728.748	4007.655	3087.937
1372211_at	246760	Mafk	v-maf musculoaponeurotic fibrosarcom	1728.14	983.6089	2553.042
1368229_at	84404	Sip1	survival of motor neuron protein intera	1727.433	992.6169	2361.626
1377889_at	498058	NA	NA	1727.229	1289.614	630.9839
1389025_at	309638	Taf11	TAF11 RNA polymerase II, TATA box	1726.577	5246.227	6066.544
1382269_at	294014	Cnnm2	cyclin M2	1726.109	465.6927	753.7538
1378092_at	290230	RGD13111	similar to magnesium-dependent phos	1725.77	1308.447	633.5399
1373095_at	503050	NA	NA	1725.746	1262.487	2117.309
1368179_at	171499	Tslpr	thymic stromal-derived lymphopoietin,	1725.561	3281.14	878.9104
1371673_at	310661	Vps72_pre	vacuolar protein sorting 72 (yeast) (pre	1724.519	2869.315	970.5593
1367536_at	295961	Apip_predi	APAF1 interacting protein (predicted)	1723.862	1786.956	1390.382
1371908_at	296219	Nxt1_predi	NTF2-related export protein 1 (predicte	1723.339	3795.152	1019.509
1376307_a_at	266631	Drb1	developmentally regulated RNA-bindin	1722.421	1595.086	2278.61
1377692_at	362697	RGD15653	similar to RIKEN cDNA 1110018J12 (p	1720.581	4266.346	1747.947
1387190_at	140866	Dgka	diacylglycerol kinase, alpha	1720.183	268.8635	1617.028
1389980_at	289324	RGD15597	similar to Protein HSPC163 (predicted	1719.953	2383.815	1639.781
1374525_at	363239	Raph1_pre	Ras association (RalGDS/AF-6) and p	1718.337	2140.406	3733.378
1387940_at	192234	Eif2b5	eukaryotic translation initiation factor 2	1717.945	1975.432	403.3455
1371713_at	311860	Abl1_mapp	v-abl Abelson murine leukemia viral or	1717.732	1431.323	1761.953
1377855_at	366872	RGD13595	similar to RIKEN cDNA 4921537D05	1716.474	1235.014	2360.337
1373531_at	366952	Cpsf1_pred	cleavage and polyadenylation specific	1715.474	1537.842	1071.498
1369993_at	171140	Camk2g	calcium/calmodulin-dependent protein	1715.295	1254.425	602.5894
1390144_at	313155	RGD13591	similar to RIKEN cDNA 3110043O21	1712.453	4294.049	2131.878
1370042_at	84510	Stmn2	stathmin-like 2	1710.924	27554.92	1045.833
1374690_at	362098	Gle1l	GLE1 RNA export mediator-like (yeast	1710.456	3052.049	1778.912
1370806_at	246298	Retsat	all-trans-13,14-dihydroretinol saturase	1710.051	4716.171	1433.675
1368977_a_at	84384	Fxc1	fractured callus expressed transcript 1	1709.405	4055.57	1130.238
1372973_at	81681	Lss	lanosterol synthase	1709.382	3787.053	2419.319
1392940_at	291022	Ptpdc1_pre	protein tyrosine phosphatase domain c	1709.086	1859.447	2534.765
1390786_at	310635	Arhgef2	rho/rac guanine nucleotide exchange f	1708.868	618.889	2063.024
1382461_at	361402	RGD13048	similar to arginine N-methyltransferase	1708.779	802.3408	703.0651
1370054_at	54238	Cdkn2c	cyclin-dependent kinase inhibitor 2C (p	1707.888	5539.274	782.7088
1392552_at	310614	Gatad2b	GATA zinc finger domain containing 2I	1707.122	390.7771	970.4532

1384391_at	353252	Rdh10	retinol dehydrogenase 10 (all-trans)	1706.656	769.3126	837.3556
1374848_at	361858	RGD13074	similar to RIKEN cDNA 2410016F19 (p	1705.526	1433.13	1127.698
1383299_at	500365	NA	NA	1705.004	972.1744	1928.277
1374550_at	500526	MGC11277	similar to map kinase interacting kinas	1704.433	696.7545	554.3463
1372786_at	287150	RGD13061	similar to hypothetical protein MGC249	1704.314	817.4898	1381.899
1390697_at	363462	Fam51a1	family with sequence similarity 51, mer	1703.597	2592.765	2615.369
1382285_at	315165	Naga	N-acetyl galactosaminidase, alpha	1703.468	1026.559	328.0858
1372661_at	287120	Tbl3	transducin (beta)-like 3	1703.312	2023.748	518.8851
1367929_at	25407	Cd59	CD59 antigen	1702.997	1226.998	6810.666
1387009_at	29153	Capn1	calpain 1	1701.256	366.0315	485.7616
1388717_at	309686	Pofut2_pre	protein O-fucosyltransferase 2 (predict	1697.475	1261.16	990.2814
1370433_at	246211	Hsd3b7	hydroxy-delta-5-steroid dehydrogenase	1696.175	1472.004	443.2496
1368550_at	64826	Foxq1	forkhead box Q1	1693.606	267.5335	562.1614
1373107_at	360475	Alg1_predi	asparagine-linked glycosylation 1 hom	1693.555	1113.024	421.7794
1387977_at	85482	Nbn	nibrin	1689.312	3163.156	2887.635
1399136_at	361807	Lemd2	LEM domain containing 2	1688.65	2052.899	1222.568
1386934_at	50690	Slc6a8	solute carrier family 6 (neurotransmitte	1688.354	3856.001	1908.28
1373763_at	311872	Zfp297b	zinc finger protein 297B	1688.152	988.1063	3977.238
1376202_at	293667	B3gnt6_pre	UDP-GlcNAc:betaGal beta-1,3-N-acety	1688.1	2357.921	1212.403
1372044_at	360738	RGD13103	similar to Ser/Thr-rich protein T10 in D	1687.046	1304.134	896.8999
1388760_at	296969	Slc35b4_pr	solute carrier family 35, member B4 (p	1686.776	2337.309	894.7828
1392534_at	311676	Tmepai_pr	transmembrane, prostate androgen ind	1685.845	245.5142	1278.795
1367767_at	79238	Hmgcl	3-hydroxy-3-methylglutaryl-Coenzyme	1685.657	1688.587	699.5792
1368554_at	25702	Pnlip	pancreatic lipase	1684.777	93.24521	13977.99
1380449_at	500367	RGD15609	similar to C230080I20Rik protein (prec	1684.014	1873.63	3347.939
1380459_at	296583	Btbd14a	BTB (POZ) domain containing 14A	1680.845	756.3027	1009.339
1386963_at	116717	Trip10	thyroid hormone receptor interactor 10	1680.283	490.1581	745.9812
1399051_at	361736	RGD13055	similar to expressed sequence AV3120	1679.275	2017.408	3131.895
1377745_at	310946	LOC31094	similar to hypothetical protein FLJ2033	1679.201	1788.905	1670.304
1380021_at	310772	Dennd2d_p	DENN/MADD domain containing 2D (p	1676.381	357.5468	36.9793
1384011_a_at	290946	RGD13097	similar to hypothetical protein MGC529	1676.336	1413.995	1784.467
1388991_at	295678	RGD15622	similar to SEC14 and spectrin domains	1676.318	1200.825	2298.446
1392911_at	503481	NA	NA	1675.419	1837.081	3699.524
1372088_at	690784	LOC69078	NA	1675.411	2422.006	6665.481
1372012_at	298298	Dhcr24	24-dehydrocholesterol reductase	1674.137	3982.008	2777.749
1388539_at	287925	Pkp2	plakophilin 2	1673.988	312.8876	593.3815
1368175_at	171159	Zhx1	zinc-fingers and homeoboxes 1	1673.972	1299.409	1939.353
1386061_at	305605	RGD13066	similar to hypothetical protein (predicte	1673.443	2098.536	1435.994
1388793_at	287159	Pigq	phosphatidylinositol glycan, class Q	1673.258	3099.879	1113.14
1372093_at	25701	Mxi1	Max interacting protein 1	1673.25	2290.025	2197.189
1373743_at	304132	RGD13050	similar to open reading frame 5	1671.849	1965.826	1045.826
1377690_at	292771	Sipa1l3	signal-induced proliferation-associated	1671.654	603.9583	916.2756
1373304_at	287711	Coasy	Coenzyme A synthase	1671.506	1164.491	485.1196
1373941_at	498112	RGD15661	similar to YEATS domain containing 2	1670.523	2458.036	2394.717
1386935_at	79240	Nr4a1	nuclear receptor subfamily 4, group A,	1669.633	467.3302	8381.029
1374549_at	474154	LOC47415	zinc responsive protein ZD7	1668.153	2813.128	2396.742
1376878_at	498967	RGD15598	similar to RIKEN cDNA 2310022B05 (p	1668.142	1359.281	1172.315
1368009_at	114711	Gne	glucosamine	1666.454	1140.235	624.2554
1382386_at	362295	RGD15639	similar to Lmbr1 protein (predicted)	1666.239	3031.076	1607.728
1370411_at	89821	Trpc1	transient receptor potential cation char	1666.053	681.9537	6562.636
1388922_at	282827	Aip	aryl-hydrocarbon receptor-interacting p	1665.782	4046.684	1524.496

1372376_at	316013	Kif9_predic	kinesin family member 9 (predicted)	1665.421	1360.35	3245.36
1381609_at	259270	Zfp335	zinc finger protein 335	1664.892	1217.932	1327.639
1390390_at	366964	Tcf20_map	transcription factor 20 (mapped)	1664.855	2040.071	1743.889
1376682_at	362261	LOC36226	NA	1664.69	1350.515	609.0118
1372389_at	494344	Ier2	immediate early response 2	1663.295	29.30309	17825.91
1367882_at	25152	Mtap1a	microtubule-associated protein 1 A	1663.203	1459.954	859.3111
1374050_at	360521	Rufy1	RUN and FYVE domain containing 1	1662.492	1720.1	3549.209
1367906_at	24162	Acp2	acid phosphatase 2, lysosomal	1662.207	2252.322	1265.08
1388885_at	365872	Aph1a	anterior pharynx defective 1a homolog	1661.409	2029.987	769.8351
1382066_at	362894	RGD1310C	similar to mKIAA1002 protein	1660.639	626.5363	1184.423
1367751_at	29640	Dpm2	dolichol-phosphate (beta-D) mannosyl	1660.197	4477.641	877.4905
1389347_at	361694	Pitpnm1	phosphatidylinositol transfer protein, m	1659.896	3089.023	1096.339
1377670_at	361605	Pcf11_prec	cleavage and polyadenylation factor su	1659.55	2134.995	13934.08
1389686_at	501563	Prkx	NA	1659.503	2120.41	1697.911
1376288_at	362906	Nudcd1_pr	NudC domain containing 1 (predicted)	1657.313	1901.598	1561.994
1376776_at	500528	LOC50052	NA	1656.92	1240.141	2011.9
1368241_a_at	64665	Flot1	flotillin 1	1656.41	1149.506	1839.861
1389788_at	293950	RGD13073	similar to hypothetical protein	1655.907	2787.251	3046.888
1369407_at	25341	Tnfrsf11b	tumor necrosis factor receptor superfa	1654.636	468.7012	371.38
1374516_at	364996	RGD13068	similar to RIKEN cDNA 5830457O10 (	1653.446	855.8976	672.8353
1374577_at	360977	Tbrg4	transforming growth factor beta regula	1651.957	2264.031	1201.885
1382068_at	499016	RGD15644	similar to tubby super-family protein (p	1651.632	1224.923	1131.509
1390478_at	252936	Orc4	origin recognition complex, subunit 4	1651.554	1419.031	5047.615
1387276_at	360341	Ania4	activity and neurotransmitter-induced e	1650.977	1129.541	3204.528
1372202_at	301374	RGD13105	similar to expressed sequence AI5974	1650.887	2443.567	3202.609
1373211_at	309444	Fbxw4_pre	F-box and WD-40 domain protein 4 (pr	1650.179	1917.36	1592.24
1389715_at	502643	Ypel4	yippee-like 4	1649.597	910.1286	13190.15
1370327_at	245974	Commd5	COMM domain containing 5	1649.446	702.7296	776.6898
1368525_at	79032	Mrs2l	MRS2-like, magnesium homeostasis fa	1648.988	1458.931	653.0028
1388149_at	24811	Tap1	transporter 1, ATP-binding cassette, su	1648.675	861.9784	698.5306
1373550_at	360534	Trim11_pre	tripartite motif protein 11 (predicted)	1647.039	1447.332	1337.15
1398573_at	306977	Zfp307	zinc finger protein 307	1646.245	1913.373	4059.184
1374137_at	85424	Elf1	E74-like factor 1	1645.725	338.1749	2840.086
1376451_at	362317	LOC36231	NA	1644.717	1653.596	5631.03
1388577_at	361418	Hsdl1	hydroxysteroid dehydrogenase like 1	1643.291	1348.317	2635.8
1375869_at	360827	Ulk1_mapp	unc-51-like kinase 1 (mapped)	1641.975	2984.24	3997.117
1372746_at	306348	RGD15662	similar to RIKEN cDNA 2810428I15 (p	1641.413	2551.635	836.0376
1376192_at	303669	Nat9_predi	N-acetyltransferase 9 (predicted)	1641.069	917.4713	856.3397
1395771_at	81647	Atf2	activating transcription factor 2	1640.269	333.2625	1977.948
1371862_at	365320	Rrm1_map	ribonucleotide reductase M1 (mapped)	1638.031	11140.98	1296.08
1372129_at	360520	RGD13101	similar to TBC1 domain family, membe	1637.832	1813.872	2398.63
1377205_at	290668	RGD13087	similar to KIAA0892 protein (predicted)	1637.791	1008.436	1071.257
1371525_at	308069	Slc12a7	solute carrier family 12 (potassium/chl	1637.192	1301.972	3116.253
1398377_at	303165	Zfp672	zinc finger protein 672	1636.48	1460.977	1608.959
1388609_at	313667	Plekhm2_p	pleckstrin homology domain containing	1634.313	791.2327	2174.405
1391444_at	503218	NA	NA	1634.079	882.2703	1685.296
1374647_at	310749	Rsbn1_pre	rosbin, round spermatid basic protein 1	1632.276	1233.394	6502.549
1377116_at	359726	Rnasel	ribonuclease L (2',5'-oligoisoadenylate	1631.94	311.4098	1124.156
1380035_at	500501	MGC1250C	similar to RIKEN cDNA 5830433M19	1631.289	1131.721	3258.699
1379333_at	363496	Armcx6	armadillo repeat containing, X-linked 6	1630.685	2381.14	1105.493
1384364_at	310363	Fbxo4_pre	F-box only protein 4 (predicted)	1630.457	681.5554	1009.649

1370365_at	25458	Gss	glutathione synthetase	1628.89	1161.091	241.0026
1373939_at	297393	Nagk	N-acetylglucosamine kinase	1627.618	1663.986	544.8276
1388334_at	360668	RGD15631	similar to SEC14-like 1 (predicted)	1627.525	776.4781	1083.696
1374161_at	314407	LOC31440	NA	1626.725	3405.261	1340.195
1389132_at	192154	Hip1	huntingtin interacting protein 1	1626.521	1986.432	800.5031
1397176_at	290564	Mettl6	methyltransferase like 6	1626.333	1354.622	1167.439
1398420_at	303614	Smurf2_pre	SMAD specific E3 ubiquitin protein liga	1625.919	1109.224	838.615
1378445_at	361197	RGD15615	similar to putative repair and recombin	1625.604	2509.511	1880.08
1374220_at	361261	RGD15659	similar to 5730405I09Rik protein (pred	1624.445	1340.033	704.3732
1373913_at	360992	Pnpt1	polyribonucleotide nucleotidyltransfera	1623.995	4281.052	3313.351
1372689_at	363150	Tmem103_	transmembrane protein 103 (predicted	1623.144	1336.984	690.3162
1371899_at	311130	Prkra	protein kinase, interferon inducible dou	1622.747	4426.563	6046.519
1368858_at	50555	Ugt8	UDP galactosyltransferase 8	1622.568	1995.529	543.956
1398084_at	501026	LOC50102	NA	1621.964	167.3485	1754.952
1378294_at	313782	Fbxl12	F-box and leucine-rich repeat protein 1	1621.963	785.2568	1415.152
1392701_at	363145	Gmppb_pr	GDP-mannose pyrophosphorylase B (	1621.177	544.9592	586.0657
1393740_at	298545	Cnksr1	connector enhancer of kinase suppres	1621.055	201.9531	736.1526
1373655_at	365215	RGD13098	similar to RIKEN cDNA 1500002O20	1619.884	1812.183	1228.051
1373828_at	310615	Crtc2	CREB regulated transcription coactiva	1619.815	895.232	1143.902
1387866_at	25486	Myo9b	myosin IXb	1619.321	858.9965	782.2797
1367514_at	361635	LOC36163	similar to RIKEN cDNA 9030624J02	1618.824	1805.463	849.5617
1372148_at	295064	Commd2_p	COMM domain containing 2 (predicted	1617.959	3190.235	991.0049
1392334_at	309621	RT1-Ba	RT1 class II, locus Ba	1616.212	485.1887	347.0732
1378387_at	290232	Tinf2	TERF1 (TRF1)-interacting nuclear fact	1615.577	1479.132	853.5934
1376774_at	116654	Exoc4	exocyst complex component 4	1614.725	1943.396	2090.306
1390244_at	300221	Spats2_pre	spermatogenesis associated, serine-ri	1614.364	2870.601	2423.512
1370811_at	192172	Mpst	mercaptopyruvate sulfurtransferase	1614.024	1304.786	1082.651
1376415_at	311903	Mrrf	mitochondrial ribosome recycling facto	1613.799	1002.236	963.2578
1373665_at	303238	RGD15631	similar to novel protein (predicted)	1613.379	2332.449	2589.521
1370197_a_at	25522	Prkcz	protein kinase C, zeta	1612.471	331.7878	854.3502
1381220_at	29263	Acvr2a	activin receptor IIA	1612.162	900.4599	3617.633
1387901_at	25529	Ptprd	protein tyrosine phosphatase, receptor	1611.985	8600.139	1284.436
1398878_at	252943	Cpsf4	cleavage and polyadenylation specific	1611.982	2109.376	1012.504
1389233_at	292944	Reps1_pre	RalBP1 associated Eps domain contai	1611.893	3003.687	3870.995
1389260_at	363332	LOC36333	NA	1611.619	2200.35	924.3625
1389982_at	257634	Lhx1	LIM homeobox protein 1	1610.746	39.8344	221.0969
1378825_a_at	361987	RGD15603	similar to brain specific protein 4 (pred	1610.578	848.4529	3213.524
1384186_at	297504	RGD15636	similar to mKIAA0212 protein (predicte	1610.054	2420.131	636.276
1390802_at	293888	RGD13068	similar to RIKEN cDNA 5033414D02 (|	1609.603	9470.859	1725.921
1374706_at	29454	Gdf11	growth differentiation factor 11	1609.273	4277.762	2231.697
1377137_at	314380	Tdp1	tyrosyl-DNA phosphodiesterase 1	1608.96	500.9064	1100.95
1376035_at	362768	Ttc7b_pred	tetratricopeptide repeat domain 7B (pr	1608.098	1015.911	2925.318
1377746_at	362250	LOC36225	NA	1607.75	1358.034	1811.084
1372966_at	298504	RGD13101	hypothetical LOC298504 (predicted)	1607.544	2519.396	313.7924
1392464_at	362281	Rae1	RAE1 RNA export 1 homolog (S. pomb	1607.292	3273.977	1636.702
1390661_at	314453	Wdr20	WD repeat domain 20	1606.697	2105.316	2534.881
1376690_at	312849	Surb7_pre	SRB7 (suppressor of RNA polymerase	1605.914	3070.429	2711.558
1373542_at	308589	Sphk2	sphingosine kinase 2	1605.878	1310.705	900.9118
1374997_at	309168	Map3k11	mitogen-activated protein kinase kinas	1605.424	1442.67	991.1849
1384220_at	316221	Tbcc_predi	tubulin-specific chaperone c (predicted	1605.351	1831.571	3247.96
1391497_at	294071	Tbc1d12_p	TBC1D12: TBC1 domain family, memb	1603.975	2403.323	3308.572

1375455_at	301233	Guca1a_pr	guanylate cyclase activator 1a (retina)	1603.782	36.8155	337.484
1377648_at	308218	RGD13103	similar to KIAA1838 protein (predicted)	1603.59	1119.885	2041.789
1375664_at	308971	Tnrc6_pred	trinucleotide repeat containing 6 (predi	1602.529	853.9662	3324.972
1372819_at	361407	Cog4_pred	component of oligomeric golgi complex	1602.438	1137.45	678.2648
1373875_at	299818	RGD13082	similar to RIKEN cDNA 1190005P17 (p	1602.231	1413.175	1257.115
1390647_at	296762	Phtf2_pred	putative homeodomain transcription fa	1601.613	3571.316	4228.928
1371697_at	361676	Pnpla2_pre	patatin-like phospholipase domain con	1601.381	1429.721	863.722
1395928_at	81812	Thra	thyroid hormone receptor alpha	1601.136	1388.662	2514.023
1391014_at	311791	Zmynd19	zinc finger, MYND domain containing 1	1600.125	1662.312	649.6796
1368717_at	29347	Faah	fatty acid amide hydrolase	1599.84	4609.18	925.3451
1372728_at	83576	Sort1	sortilin 1	1599.585	1240.718	1488.466
1374533_at	499883	RGD15603	similar to GA binding protein transcript	1599.124	2020.178	4394.129
1374032_at	303274	Phf12	PHD finger protein 12	1598.908	1823.949	6406.222
1388736_at	360637	Ccdc43	coiled-coil domain containing 43	1598.637	2821.576	992.4565
1387087_at	24253	Cebpb	CCAAT/enhancer binding protein (C/E	1597.857	736.7463	4187.452
1388402_at	361695	RGD13058	similar to 2410001H17Rik protein (pre	1597.22	4058.635	1337.234
1370452_at	24239	Cacna1c	calcium channel, voltage-dependent, L	1596.606	1219.338	3189.621
1395445_at	499617	LOC49961	similar to regulatory factor X-associate	1595.632	1417.87	2043.917
1373989_at	363140	Rassf1	Ras association (RalGDS/AF-6) doma	1594.954	644.9462	2457.116
1368169_at	84591	Pick1	protein interacting with C kinase 1	1592.613	2920.854	1452.567
1381057_at	364835	RGD15617	similar to hypothetical protein MGC539	1592.459	938.2397	443.8416
1382218_at	298077	RGD13058	hypothetical LOC298077	1591.234	2598.004	1698.616
1393072_at	363065	Ube2q2_pr	ubiquitin-conjugating enzyme E2Q (pu	1589.546	1490.084	2276.693
1382318_at	289019	Pctk3	PCTAIRE-motif protein kinase 3	1586.98	894.7413	561.8868
1372266_at	309812	Rev3l	REV3-like, catalytic subunit of DNA po	1586.677	4498.113	12733.52
1374047_at	291784	RGD13118	similar to RIKEN cDNA 2400010D15	1586.116	3215.862	3980.324
1382739_at	312787	Gpr19	G protein-coupled receptor 19	1586.072	1682.319	1352.001
1373326_at	295645	Slc4a10	solute carrier family 4, sodium bicarbo	1585.462	271.6625	651.5937
1371562_at	60443	Epn2	epsin 2	1585.013	2325.025	1191.398
1389391_at	362883	RGD15648	similar to Solute carrier family 35, mem	1584.891	4213.001	449.4999
1389716_at	501614	NA	NA	1584.352	1538.208	4650.09
1384152_at	363164	Wdr48_pre	WD repeat domain 48 (predicted)	1584.239	1616.556	1486.414
1384858_a_at	299900	RGD13071	similar to RIKEN cDNA D530033C11 (	1583.972	1510.672	439.2354
1367778_at	54281	Pcsk3	proprotein convertase subtilisin/kexin t	1583.425	1019.178	1758.809
1375430_at	303211	RGD13112	hypothetical LOC303211	1583.271	1128.516	1191.858
1379026_at	287556	Rhbdl4_pre	rhomboid, veinlet-like 4 (Drosophila) (p	1582.696	2160.343	4333.273
1390281_a_at	362810	Coq10a_pr	coenzyme Q10 homolog A (yeast) (pre	1582.065	2537.893	3061.838
1387800_at	140926	Daxx	Fas death domain-associated protein	1580.74	1388.215	803.6099
1376317_at	291927	Orc6l	origin recognition complex, subunit 6-li	1580.265	2414.928	1698.019
1389703_at	287476	Zzef1_pred	zinc finger, ZZ-type with EF hand doma	1579.4	1259.222	2020.211
1367890_at	64314	Casp2	caspase 2	1578.877	5537.083	4112.479
1384967_at	365583	Sart2_pred	squamous cell carcinoma antigen reco	1578.247	219.5247	1094.915
1370267_at	84027	Gsk3b	glycogen synthase kinase 3 beta	1577.228	1352.692	911.0948
1369057_at	81804	Stxbp2	syntaxin binding protein 2	1576.713	2117.165	618.0644
1374376_at	361882	Rgnef_pred	Rho-guanine nucleotide exchange fact	1576.579	493.2671	1195.263
1371519_at	295143	Etfdh	electron-transferring-flavoprotein dehy	1576.102	1806.21	1679.831
1381364_at	309126	Tnpo1	transportin 1	1575.681	1556.349	657.6357
1372016_at	299626	Gadd45b	growth arrest and DNA-damage-induci	1575.45	684.849	10164.75
1387312_a_at	24385	Gck	glucokinase	1574.793	3146.108	1102.449
1390440_at	302863	Slc9a6_pre	solute carrier family 9 (sodium/hydroge	1574.706	2138.704	1797.737
1374808_at	313525	RGD13098	similar to hypothetical protein FLJ2115	1574.3	1882.236	852.9681

1383467_at	362132	Epc2_pred	enhancer of polycomb homolog 2 (Dro	1573.712	1222.639	7417.497
1374715_at	294783	RGD13094	similar to hypothetical protein FLJ1023	1573.519	1508.711	2107.719
1390926_at	311630	Zswim3_pr	zinc finger, SWIM domain containing 3	1573.146	897.4363	595.0931
1383679_at	364386	Rnf31_pre	ring finger protein 31 (predicted)	1572.907	545.4395	1422.989
1375728_at	361063	Entpd4_pre	ectonucleoside triphosphate diphosph	1572.206	304.8424	558.4941
1383376_at	368190	LOC36819	NA	1571.981	1421.877	941.532
1372462_at	308100	Acat2	acetyl-Coenzyme A acetyltransferase	1571.799	5098.682	3653.164
1379461_at	301076	RGD15591	similar to ZFP (predicted)	1571.014	582.0531	555.9202
1376537_at	500465	NA	NA	1570.379	680.7806	709.4107
1380810_at	499061	NA	NA	1569.955	502.2126	3830.394
1370316_at	246146	Hspbp1	hsp70-interacting protein	1569.368	3119.978	1105.508
1373853_at	299647	RGD13081	similar to hypothetical protein MGC157	1569.137	3611.97	554.0815
1375563_at	287178	RGD13055	similar to dendritic cell-derived ubiquiti	1567.267	3054.182	1116.285
1382253_at	315549	RGD13071	similar to hypothetical protein FLJ2110	1565.904	1274.751	1054.963
1374851_at	292751	Ixl_predicte	intersex-like (Drosophila) (predicted)	1565.158	1184.615	1159.3
1398391_at	309593	Gnl1	guanine nucleotide binding protein-like	1563.89	1577.748	1386.667
1373464_at	366968	Tbc1d22a_	TBC1 domain family, member 22a (pre	1561.517	1028.043	870.4443
1368016_at	113956	Pecr	peroxisomal trans-2-enoyl-CoA reduct	1561.501	747.173	2094.489
1367901_at	24434	Gusb	glucuronidase, beta	1560.744	3211.735	1393.779
1370044_at	140930	Faim	Fas apoptotic inhibitory molecule	1559.98	1432.146	2890.525
1392244_at	498362	NA	NA	1559.232	2559.808	976.1039
1368955_at	29647	Cask	calcium/calmodulin-dependent serine	1558.81	3777.228	1697.59
1391706_at	501168	NA	NA	1558.355	576.3581	854.0062
1372458_at	498178	LOC49817	NA	1557.899	3857.143	806.2334
1371943_at	360230	Lyk4	Ser/Thr-like protein kinase lyk4	1555.792	1144.012	2594.064
1374564_at	304591	Dtx2	deltex 2 homolog (Drosophila)	1554.783	550.7287	1099.502
1392929_at	499891	RGD15656	RGD1565616 (predicted)	1554.559	2410.313	2894.958
1387891_at	85274	Prdx4	peroxiredoxin 4	1554.529	11860.83	7651.732
1371991_at	298201	RGD13112	similar to RIKEN cDNA B230312A22	1553.396	379.496	994.5464
1388185_at	24708	Rb1	retinoblastoma 1	1553.054	18.30084	4336.751
1386992_at	29355	Pkn1	protein kinase N1	1552.906	206.1098	620.8903
1371846_at	361094	Phgdhl1	phosphoglycerate dehydrogenase like	1552.228	1086.834	705.1891
1374518_at	362011	Tmem77	transmembrane protein 77	1551.665	2442.135	1158.851
1375677_at	315159	Tob2	transducer of ERBB2, 2	1551.034	921.3338	1276.047
1368242_at	25736	Kcnb1	potassium voltage gated channel, Sha	1550.868	789.5521	1360.082
1388964_at	361946	RGD13078	similar to transcription factor (p38 inter	1550.04	2347.898	2862.036
1368869_at	83425	Akap12	A kinase (PRKA) anchor protein (gravi	1549.142	2957.297	8821.588
1373435_at	287433	Fxr2h_pred	fragile X mental retardation gene 2, au	1548.026	1796.649	530.1085
1387205_at	24747	RT1-M3	RT1 class Ib, locus M3	1547.821	970.182	1658.796
1384340_a_at	363518	Ard1_predi	N-acetyltransferase ARD1 homolog (S	1547.752	1338.918	624.1748
1374224_at	114859	Eif2ak4_pr	eukaryotic translation initiation factor 2	1547.734	2970.887	10475.07
1374053_at	294339	Mcm3ap_p	minichromosome maintenance deficien	1546.063	1673.794	2587.173
1391109_x_at	619560	Rup2	urinary protein 2	1546.056	437.7806	1737.47
1372173_at	313056	RGD13091	similar to U5 snRNP-specific protein (F	1545.725	4057.968	1278.777
1374249_at	292781	RGD13045	similar to Hypothetical protein MGC38	1543.896	431.2345	319.1737
1393430_at	293571	Zranb1_pre	zinc finger, RAN-binding domain conta	1543.606	2067.109	1992.951
1389032_at	288371	Mcoln1_pre	mucolipin 1 (predicted)	1543.477	2688.871	1654.92
1372109_at	316516	Tmbim1	transmembrane BAX inhibitor motif co	1542.918	910.6088	1007.261
1395642_at	313744	Nol9	nucleolar protein 9	1542.014	1293.83	1047.714
1369958_at	64373	Rhob	ras homolog gene family, member B	1541.605	10143.71	15404.51
1383321_at	288617	Tpst1	tyrosylprotein sulfotransferase 1	1540.305	2654.947	12212.81

1389130_at	361944	Elf2	E74-like factor 2	1540.228	2355.902	3732.591
1373570_at	311671	Npepl1_pre	aminopeptidase-like 1 (predicted)	1540.163	19.87574	221.9612
1376189_at	313604	Zmym1_pr	zinc finger, MYM domain containing 1	1539.424	2049.42	2177.104
1382807_at	363254	RGD13104	similar to hypothetical protein	1538.303	1524.496	1065.708
1389176_at	309008	Inpp5f_pre	inositol polyphosphate-5-phosphatase	1538.048	3306.428	4551.203
1383673_at	317247	Nap1l2	nucleosome assembly protein 1-like 2	1537.981	6547.071	5627.523
1367768_at	59073	Lxn	latexin	1537.824	2628.19	506.2443
1374478_at	362576	RGD13053	similar to RIKEN cDNA 2610528J11 (p	1537.027	1631.263	636.8597
1383196_at	308339	Znf579_pre	zinc finger protein 579 (predicted)	1536.853	670.78	1146.591
1374152_at	309628	Wdr46	WD repeat domain 46	1536.384	1831.033	872.1973
1373997_at	499975	RGD15629	similar to Drctnnb1a (predicted)	1535.871	3280.23	2215.02
1388967_at	317376	Tcfe3_prec	transcription factor E3 (predicted)	1534.809	1656.391	3305.448
1374393_at	288058	Ptplb_pred	protein tyrosine phosphatase-like (prol	1534.594	3401.401	629.7972
1377187_at	313069	RGD13076	similar to 3000004N20Rik protein (pre	1534.298	1109.615	4583.2
1382865_at	500069	Tsga14	testis specific, 14	1533.889	2179.271	792.8396
1398607_at	301235	RGD13056	similar to KIAA0240 (predicted)	1533.614	1392.527	3484.375
1389469_at	310707	Chd1l_prec	chromodomain helicase DNA binding p	1533.27	2244.827	1092.237
1373816_at	171494	Ap1g1	adaptor-related protein complex 1, gar	1533.121	1802.774	1812.105
1372636_at	302998	RGD13071	similar to CG18661-PA	1532.495	1860.699	1061.445
1368177_at	114024	Acsl3	acyl-CoA synthetase long-chain family	1531.548	2842.858	1737.993
1372063_at	497938	LOC49793	similar to RIKEN cDNA 4933402P03	1530.602	1458.141	4150.428
1395410_at	363122	Ppp2r3a	protein phosphatase 2 (formerly 2A), r	1530.327	1023.13	1989.874
1372118_at	497878	MGC12520	HMP19 protein	1530.246	6321.109	2578.511
1374743_at	500509	RGD15642	similar to channel-interacting PDZ dom	1530.078	898.7984	1600.38
1374213_at	296380	Arfgef2	ADP-ribosylation factor guanine nucle	1530.047	1300.024	794.2409
1389024_at	498353	LOC49835	similar to Sec1 family domain containi	1529.81	1057.966	1000.233
1377473_at	296732	Smarcd3	SWI/SNF related, matrix associated, a	1529.796	1511.364	1004.838
1370916_at	84492	Tec	tec protein tyrosine kinase	1529.609	920.1937	603.1432
1389708_at	362191	RGD13100	similar to RIKEN cDNA 5730421E18	1529.534	2619.951	3407.626
1370112_at	50557	Pten	phosphatase and tensin homolog	1529.41	3229.05	2495.787
1372281_at	289357	Lyplal1_pre	lysophospholipase-like 1 (predicted)	1529.237	1278.662	1395.534
1371816_at	502684	LOC50268	hypothetical protein LOC502684	1529.066	1314.617	1902.856
1384282_at	304342	Zipro1	zinc finger proliferation 1	1528.611	2057.573	1993.195
1390997_at	64076	Slc26a1	solute carrier family 26 (sulfate transpo	1528.217	1300.449	2219.356
1372789_at	362425	Zfp637	zinc finger protein 637	1527.176	5974.166	2576.007
1383494_at	301665	RGD15627	similar to hypothetical protein (predicte	1526.344	2032.239	1650.207
1376762_at	361659	RGD15641	similar to Pleckstrin homology domain-	1526.333	11233.6	10400.55
1390439_at	299864	Ebag9	estrogen receptor-binding fragment-as	1526.318	2434.773	4023.08
1382430_at	315959	Msl2l1_pre	male-specific lethal 2-like 1 (Drosophila	1525.931	891.9995	2818.188
1371980_at	298682	Atad3a	ATPase family, AAA domain containing	1524.93	1027.391	456.9944
1382219_at	310348	RGD13075	similar to RIKEN cDNA 1700108L22	1524.512	1387.468	959.0936
1375654_at	362859	Ckap4_pre	cytoskeleton-associated protein 4 (pre	1524.478	949.2446	91.03203
1389455_at	295461	Sec24b_pr	SEC24 related gene family, member B	1524.294	1162.107	1640.385
1378661_x_at	499665	RGD15627	RGD1562704 (predicted)	1524.009	375.984	417.6439
1372010_at	498183	MGC11632	similar to MK-5 type 2	1522.652	2187.445	3594.566
1368877_at	24522	Zfp354a	zinc finger protein 354A	1522.28	1948.634	2210.506
1373532_at	308543	Plekhf1	pleckstrin homology domain containing	1521.736	235.9256	295.3917
1390200_at	362528	RGD13048	similar to mKIAA0674 protein (predicte	1521.641	1347.241	768.3609
1367946_at	54133	Pdlim1	PDZ and LIM domain 1 (elfin)	1521.172	2451.12	1940.485
1395748_at	362189	Slc12a6	solute carrier family 12, member 6	1520.942	359.5398	1082.784
1370418_s_at	192189	Bk	brain and kidney protein	1520.827	2077.185	3989.998

1393561_at	363087	RGD15603	similar to vacuolar protein sorting 13C	1520.736	646.4391	3660.731
1373001_at	312559	Chchd4	coiled-coil-helix-coiled-coil-helix domai	1520.598	1877.626	573.3465
1368234_at	83471	Prep	prolyl endopeptidase	1519.868	2667.544	676.6007
1372644_at	362949	NA	NA	1519.517	2977.749	900.1802
1368136_at	25359	Tmpo	thymopoietin	1519.121	2889.957	1481.796
1395155_at	60627	Exoc5	exocyst complex component 5	1518.798	1589.436	2249.693
1391462_at	361054	Ebpl_predi	emopamil binding protein-like (predicte	1518.449	33.37512	567.7772
1396142_at	301371	Mrps9	mitochondrial ribosomal protein S9	1517.606	6837.772	1117.63
1387554_at	83627	Galnt5	UDP-N-acetyl-alpha-D-galactosamine:	1517.327	229.8248	631.0327
1389215_at	291314	Sephs1	selenophosphate synthetase 1	1517.303	3454.391	2073.089
1394737_at	361201	RGD13113	similar to CG9752-PA	1516.639	3069.71	1510.853
1376171_at	408217	Usp11	ubiquitin specific protease 11	1514.754	3103.155	3432.037
1372302_at	311328	RGD13086	similar to hypothetical protein FLJ1057	1514.685	1331.955	1597.785
1377602_at	297998	Nudt2	nudix (nucleoside diphosphate linked r	1514.252	2879.206	1324.29
1386986_at	83525	Ogfr	opioid growth factor receptor	1513.718	3098.217	1135.534
1380045_at	246311	Pdp2	pyruvate dehydrogenase phosphatase	1513.129	1158.195	559.8182
1367886_at	29606	Pcsk7	proprotein convertase subtilisin/kexin t	1512.987	1274.743	847.8597
1382921_at	289504	Sdad1	SDA1 domain containing 1	1512.879	1104.971	605.241
1388691_at	310633	Ubqln4_pre	ubiquilin 4 (predicted)	1512.567	1747.516	1277.379
1370368_at	94165	Cabin1	calcineurin binding protein 1	1512.545	1552.798	1381.834
1372430_at	311428	RGD13117	similar to RIKEN cDNA 1700037H04	1512.525	2131.344	1998.808
1388765_at	25233	Akt2	thymoma viral proto-oncogene 2	1512.391	1064.626	907.8596
1369909_s_at	245966	Tm6p1	fasting-inducible integral membrane pr	1512.277	582.6712	570.6123
1373562_at	309200	RGD13093	similar to RIKEN cDNA 1110055N21 (|	1511.691	881.8137	309.2103
1372980_at	500065	RGD15609	similar to Hypothetical protein MGC30	1511.508	1112.838	1528.13
1386689_at	304669	Hook2	hook homolog 2 (Drosophila)	1509.855	643.2326	478.1596
1367876_at	116458	Ipo13	importin 13	1509.56	1042.083	483.5602
1388905_at	299608	RGD13111	membralin	1509.088	1754.167	1532.598
1390967_a_at	298688	RGD13068	similar to RIKEN cDNA 2410006F12	1508.392	1009.054	913.5404
1374689_at	81747	Pik4cb	phosphatidylinositol 4-kinase, catalytic	1507.347	1477.719	1448.05
1395520_at	312182	Rbm28_pre	RNA binding motif protein 28 (predicte	1507.02	1740.763	663.6177
1383534_at	498195	NA	NA	1506.445	1462.831	1632.848
1389261_at	303275	RGD13055	similar to hypothetical protein MGC232	1505.646	1525.828	476.7119
1392422_at	367083	Alg9_predi	asparagine-linked glycosylation 9 hom	1505.046	821.5268	1127.284
1371251_at	298003	Galt	galactose-1-phosphate uridyl transfera	1504.544	2936.647	2180.631
1371664_at	360820	Pxn	paxillin	1504.103	1451.448	3364.241
1384335_at	300673	Bcl9l_predi	B-cell CLL/lymphoma 9-like (predicted	1502.306	826.4233	2778.487
1374793_at	310720	Wdr3_pred	WD repeat domain 3 (predicted)	1501.811	2598.594	1533.112
1391262_at	679900	LOC67990	NA	1501.381	2731.909	1008.703
1373817_at	297597	Ing4	inhibitor of growth family, member 4	1499.681	4108.803	1220.058
1389335_at	314273	Wdr22	WD repeat domain 22	1498.602	1023.339	1579.084
1375041_at	685932	LOC68593	NA	1498.237	55.38895	972.3361
1381919_at	309446	Hps6	Hermansky-Pudlak syndrome 6	1497.94	2441.483	1026.796
1367714_at	84005	Eif2b2	eukaryotic translation initiation factor 2	1497.811	1633.56	999.1888
1374266_at	307481	Pcdh1_pre	protocadherin 1 (cadherin-like 1) (pred	1497.612	1433.525	565.6602
1367868_at	65138	Adrm1	adhesion regulating molecule 1	1497.217	950.1818	1643.57
1387957_a_at	84357	Sh3kbp1	SH3-domain kinase binding protein 1	1496.96	4058.454	1587.737
1369162_at	25711	Gucy2c	guanylate cyclase 2C	1494.937	3965.774	632.7927
1389562_at	291423	Setbp1_pre	SET binding protein 1 (predicted)	1492.415	9045.616	5674.57
1370258_at	171439	Bzw2	basic leucine zipper and W2 domains :	1491.435	1324.357	576.6633
1375460_at	287731	RGD13094	similar to hypothetical protein FLJ3065	1491.218	2207.419	1634.141

1388617_at	361239	Bphl	biphenyl hydrolase-like (serine hydrola	1490.711	1206.065	3259.575
1376579_at	289668	Lap3	leucine aminopeptidase 3	1488.6	862.0889	1646.678
1389382_at	297525	Mkrn2	makorin, ring finger protein, 2	1487.752	1668.002	1102.973
1367516_at	641528	Dtnbp1	NA	1486.676	1926.36	759.561
1383227_at	298658	NA	NA	1486.138	1981.851	542.4358
1381979_at	360800	Sumf2	sulfatase modifying factor 2	1484.436	1347.041	769.8024
1371038_at	25301	Cebpg	CCAAT/enhancer binding protein (C/E	1484.029	1634.856	618.3953
1380833_at	291132	Gpld1	glycosylphosphatidylinositol specific pl	1483.399	1539.787	549.9539
1368650_at	81813	Klf10	Kruppel-like factor 10	1482.543	15992.56	15634.06
1372762_at	308758	Hddc3_pre	HD domain containing 3 (predicted)	1481.886	1913.737	790.0179
1385670_at	360816	Sdsl_predi	serine dehydratase-like (predicted)	1481.504	1179.229	937.0865
1378080_at	497930	RGD15595	similar to SCO cytochrome oxidase de	1481.498	1138.483	1472.586
1373171_at	362622	Ccdc21	coiled-coil domain containing 21	1481.356	1777.921	1631.179
1390046_at	309098	Adam8_pre	a disintegrin and metalloprotease dom	1480.787	2758.563	1464.803
1370915_s_at	171437	Dnttip1	deoxynucleotidyltransferase, terminal,	1480.722	5368.718	1335.573
1380815_at	304923	LOC30492	similar to hypothetical protein FLJ1175	1480.076	516.8036	3599.636
1384376_at	499716	LOC49971	NA	1479.68	587.8965	1046.448
1370460_at	171329	Usp15	ubiquitin specific peptidase 15	1479.165	2178.682	1368.557
1387837_at	24205	Apc	adenomatosis polyposis coli	1478.259	1240.478	434.5867
1391921_at	313131	Rngtt_pred	RNA guanylyltransferase and 5'-phosp	1478	1850.968	909.0881
1390290_at	303076	Surf6_pred	surfeit gene 6 (predicted)	1477.815	2339.096	2335.308
1399032_at	292673	Ercc1_prec	excision repair cross-complementing r	1477.773	1608.276	865.6318
1382117_at	291844	RGD15605	similar to hypothetical protein FLJ2114	1477.479	863.8295	1347.725
1374441_at	288772	RGD13066	similar to RIKEN cDNA 4633402N23 g	1476.262	1190.146	576.2063
1373745_at	294236	Gtf2h4	general transcription factor II H, polype	1476.123	2715.241	618.8409
1370015_at	83709	Git1	G protein-coupled receptor kinase inte	1474.164	1603.79	1053.107
1374839_at	353231	Spg7	spastic paraplegia 7 homolog (human)	1473.884	946.4448	885.3761
1387030_at	116721	Abcc5	ATP-binding cassette, sub-family C (C	1473.699	1776.172	1096.108
1378257_at	301014	Trex1	three prime repair exonuclease 1	1473.466	1097.627	339.1013
1375927_at	499120	LOC49912	hypothetical protein LOC499120	1472.733	1018.56	748.8329
1390035_at	305076	LOC30507	similar to hypothetical protein MGC298	1471.164	1334.513	969.6181
1374854_at	305963	RGD15660	similar to Pin2-interacting protein X1 (p	1470.824	573.7652	373.8002
1386941_at	64204	Plec1	plectin 1	1469.792	1267.656	1210.427
1386892_at	83801	Ptms	parathymosin	1469.316	5945.073	988.7144
1373897_at	116685	Lmnb1	lamin B1	1469.171	14651.4	3420.884
1369650_at	29432	Pak2	p21 (CDKN1A)-activated kinase 2	1468.875	1565.832	730.072
1383774_at	289864	RGD13081	similar to RIKEN cDNA 2010316F05 (p	1468.199	676.269	1059.041
1371886_at	311849	Crat	carnitine acetyltransferase	1468.095	1025.354	612.9705
1377149_at	306469	RGD13073	similar to RIKEN cDNA 4933411K20	1467.539	2253.168	1802.993
1383279_at	290363	Rcbtb2	regulator of chromosome condensation	1467.079	110.0824	612.4372
1382349_at	303830	Crygs	crystallin, gamma S	1466.359	853.8689	2131.183
1371031_at	25331	Mat1a	methionine adenosyltransferase I, alph	1465.837	867.9444	1578.163
1371623_at	367234	NA	NA	1465.105	1821.69	3569.046
1396581_at	314717	Scyl2_prec	SCY1-like 2 (S. cerevisiae) (predicted)	1464.272	655.253	404.0696
1398516_at	498230	RGD15663	similar to RIKEN cDNA 0610012C01 (p	1464.103	976.0986	440.7029
1376832_at	314325	RGD13089	similar to FLJ20689 (predicted)	1463.014	639.0485	2889.896
1389472_at	289159	RGD13115	similar to RIKEN cDNA 5630401D24 (p	1463.005	634.6678	655.1119
1376883_at	295663	MGC93920	similar to RIKEN cDNA 1700048E23	1462.415	1366.411	1497.043
1371014_at	24654	Plcb1	phospholipase C, beta 1	1461.621	2610.692	7727.298
1389237_at	296957	Zc3hc1_pre	zinc finger, C3HC-type 1 (predicted)	1461.335	2162.61	1518.138
1385644_at	365319	LOC36531	NA	1459.91	1364.686	508.9839

1372361_at	317381	Ccdc22_pr	coiled-coil domain containing 22 (predi	1459.661	1253.822	616.1571
1390285_at	309112	RGD15609	similar to BC026645 protein (predicted	1459.176	726.0094	1506.133
1373732_at	295305	Acp6	acid phosphatase 6, lysophosphatidic	1459.038	1290.032	877.6842
1374416_at	499214	RGD15622	similar to E2-induced gene 2 protein (p	1458.969	1254.957	411.6764
1393088_at	304012	Rg9mtd1	RNA (guanine-9-) methyltransferase d	1458.926	1644.403	2282.688
1386857_at	29332	Stmn1	stathmin 1	1458.565	30583.78	5607.117
1392595_at	313253	Znf618_pre	zinc finger protein 618 (predicted)	1458.122	913.3134	870.783
1374036_at	312538	Mcm2_pre	minichromosome maintenance deficier	1457.963	5196.861	1289.391
1386229_at	362149	RGD13091	similar to CBF1 interacting corepresso	1457.193	1202.43	2821.13
1377627_at	314713	RGD15606	similar to down-regulated in metastasis	1457.146	1412.997	832.6831
1393641_at	499356	Blnk	B-cell linker	1456.795	476.6173	21632.41
1382993_at	317673	Bbc3	Bcl-2 binding component 3	1456.448	1475.162	2568.475
1373446_at	313742	Phf13_pred	PHD finger protein 13 (predicted)	1455.228	1325.426	1290.947
1397708_at	288978	LOC28897	NA	1454.478	2080.783	1409.614
1374861_at	299636	Tle2	transducin-like enhancer of split 2, hon	1454.378	705.976	1982.193
1380553_at	313085	RGD13063	similar to Protein C8orf1 (hT41) (predic	1454.283	2350.884	2469.603
1369081_at	24591	Neu1	neuraminidase 1	1454.153	1914.895	598.3644
1368245_at	116593	Upb1	ureidopropionase, beta	1454.018	745.0573	3700.153
1374751_at	24884	Yes1	Yamaguchi sarcoma viral (v-yes) onco	1453.942	29.63675	2804.331
1389682_at	360994	RGD13091	similar to RIKEN cDNA 2510006C20	1453.32	1127.402	3490.173
1374741_at	293701	Esrra	estrogen related receptor, alpha	1453.302	1667.95	139.7815
1375059_at	497984	RGD15663	similar to zinc finger protein 652 (predi	1452.684	3413.188	2861.363
1374117_at	117542	Baiap2	brain-specific angiogenesis inhibitor 1-	1451.495	1027.721	771.5118
1374603_at	500281	Setmar	SET domain and mariner transposase	1451.431	1996.627	722.2926
1384778_at	362653	Ctrc	chymotrypsin C (caldecrin)	1451.273	79.63685	7449.293
1371551_at	303285	Traf4_pred	Tnf receptor associated factor 4 (predi	1451.145	2078.11	1233.614
1394624_at	315151	Mkl1_predi	megakaryoblastic leukemia (translocat	1451.101	350.8921	667.3672
1376159_at	311508	RGD15623	similar to zinc finger protein 336 (predi	1450.395	2806.336	2759.342
1389330_at	361654	RGD13101	similar to cDNA sequence BC017158	1450.294	822.4952	2271.638
1398999_at	363675	LOC36367	similar to es 64	1449.926	2635.42	753.1579
1387000_at	56082	Gorasp1	golgi reassembly stacking protein 1	1449.412	535.3858	695.7584
1367885_at	29533	Pxmp2	peroxisomal membrane protein 2	1449.116	680.5992	538.9211
1375413_at	361532	Sirt2	sirtuin (silent mating type information r	1448.601	1728.134	777.9768
1372117_at	309255	RGD13059	similar to BB128963 protein	1447.437	3064.095	2103.146
1389580_at	499580	NA	NA	1446.603	1446.514	1214.161
1389385_at	287383	Eppb9_pre	endothelial precursor protein B9 (predi	1445.724	5538.145	1117.979
1377846_a_at	366547	Msh6_pred	mutS homolog 6 (E. coli) (predicted)	1444.949	5615.699	3109.527
1379366_a_at	64027	Bspry	B-box and SPRY domain containing	1444.333	1169.699	493.3393
1389663_at	316014	Nbeal2_pre	neurobeachin-like 2 (predicted)	1443.827	634.5897	1055.553
1387152_at	58839	Nrbf2	nuclear receptor binding factor 2	1443.6	1679.258	2253.346
1370343_at	245976	Xab2	XPA binding protein 2	1443.478	1970.426	701.3288
1376294_at	497916	RGD15607	similar to Smith-Magenis syndrome ch	1442.97	1347.353	752.4246
1379356_at	365903	RGD1310C	similar to RIKEN cDNA C230093N12 (	1442.786	3555.563	1851.65
1387107_at	117107	Zbtb7a	zinc finger and BTB domain containing	1442.157	648.8678	1632.804
1390237_at	84383	Timm8a	translocase of inner mitochondrial mer	1442.094	1117.136	669.7069
1374707_at	306007	RGD13099	similar to 2610301G19Rik protein (pre	1440.215	1281.865	915.0365
1370421_a_at	192226	Cand2	cullin-associated and neddylation-diss	1439.141	2108.121	2153.303
1381937_at	499344	RGD15624	similar to 60S ribosomal protein L7a (p	1439.022	1492.516	1262.407
1376893_at	291129	Vmp_predi	vesicular membrane protein p24 (pred	1438.993	4878.586	2924.392
1376052_at	292654	RGD15645	similar to hypothetical protein FLJ2051	1438.643	2369.127	553.3487
1367753_at	93646	Sec31l1	SEC31-like 1 (S. cerevisiae)	1438.588	697.978	3937.227

1367956_at	89791	Ncdn	neurochondrin	1438.191	1294.689	667.8118
1372342_at	309375	Mrvldc1	MARVEL (membrane-associating) don	1438.065	189.3206	1172.105
1377446_at	500132	RGD1563\9	similar to phosphoinositol 4-phosphate	1438.048	680.6644	333.493
1377314_at	297417	Gfpt1	glutamine fructose-6-phosphate transa	1437.983	192.9889	443.0366
1382228_at	499597	NA	NA	1437.962	1647.19	1350.508
1375378_at	266718	Qki	quaking homolog, KH domain RNA bir	1437.331	759.6752	2946.975
1382629_at	293173	Mlstd2	male sterility domain containing 2	1434.997	1332.853	1185.123
1384185_at	314300	RGD13077	similar to RIKEN cDNA 2410016O06 (	1434.89	910.168	880.5869
1385481_at	297865	RGD13077	similar to NCAG1 (predicted)	1434.357	2225.731	1139.825
1368849_at	64823	Csnk1g3	casein kinase 1, gamma 3	1433.935	1886.56	561.4237
1387268_at	83582	Rpo1-2	RNA polymerase 1-2	1432.943	833.4989	465.7114
1373496_at	296988	MGC94941	similar to Mkrn1 protein	1432.525	3146.876	2749.483
1370084_at	24271	Cpb1	carboxypeptidase B1 (tissue)	1432.049	38.40681	14026.89
1370973_at	64155	Scn7a	sodium channel, voltage-gated, type V	1432.018	53.31035	21020.41
1377743_at	296072	Spred1	sprouty protein with EVH-1 domain 1, r	1431.68	2936.157	2014.373
1376761_at	84579	Hdac4	histone deacetylase 4	1429.986	386.8563	550.8987
1367955_at	50866	Rab4b	RAB4B, member RAS oncogene famil	1429.934	1436.888	436.9322
1387824_at	56763	Sfrs12	splicing factor, arginine/serine-rich 12	1429.885	646.7784	3094.265
1390032_at	288771	Rbms2	RNA binding motif, single stranded inte	1429.532	173.2725	506.2648
1388813_at	79119	Arf2	ADP-ribosylation factor 2	1429.499	7426.886	3215.931
1382099_at	361846	Vps26	vacuolar protein sorting 26 (yeast)	1428.854	2640.511	1051.313
1372781_at	310785	Wdr47	WD repeat domain 47	1427.595	3499.477	2956.341
1387471_at	24332	Ela2	elastase 2	1427.186	30.33879	14462.54
1382080_at	498530	RGD15636	similar to KIAA0853 protein (predicted)	1426.879	551.0531	2437.326
1382498_x_at	311616	Gdap1l1_p	ganglioside-induced differentiation-ass	1426.135	2464.005	910.2394
1387300_at	116481	Crnkl1	Crn, crooked neck-like 1 (Drosophila)	1425.49	2751.556	1221.868
1392503_at	307410	Rbm22	RNA binding motif protein 22	1425.141	2827.306	3409.071
1369023_at	81684	Mipep	mitochondrial intermediate peptidase	1424.993	1203.559	886.6998
1374361_at	315173	RGD13066	similar to bK1191B2.3.1 (PUTATIVE n	1424.775	1305.854	778.3569
1368231_at	24918	Stat5a	signal transducer and activator of trans	1424.456	919.8938	580.7037
1367983_at	84490	Fen1	flap structure-specific endonuclease 1	1424.391	5760.729	426.3433
1377622_at	308839	Hbxap_pre	hepatitis B virus x associated protein (	1423.991	1689.804	2715.019
1374561_at	305452	RGD13096	hypothetical LOC305452 (predicted)	1423.942	1473.584	1285.161
1372961_at	362301	MGC72996	Unknown (protein for MGC:72996)	1423.729	1180.299	675.1285
1386959_a_at	29568	Map2k5	mitogen activated protein kinase kinas	1423.587	1168.324	986.8424
1388946_at	307235	RGD13119	similar to hypothetical p38 protein (pre	1423.214	2881.294	1346.454
1372698_at	304649	RGD13050	similar to CG2662-PA (predicted)	1423.08	829.709	1204.859
1382427_at	288353	Cggbp1_pr	CGG triplet repeat binding protein 1 (p	1421.961	1386.557	1034.367
1391064_at	308865	Atg16l2_pr	ATG16 autophagy related 16-like 2 (S.	1421.273	653.4904	1066.28
1388536_at	294250	Bat2	HLA-B associated transcript 2	1421.094	1229.382	958.2083
1390957_at	361030	RGD15620	similar to Protein C14orf101 homolog (	1420.983	409.7544	1463.035
1392815_at	305367	Centd1_pre	centaurin, delta 1 (predicted)	1420.409	786.2359	1007.672
1372434_at	313255	Cip98	CASK-interacting protein CIP98	1420.176	247.9351	528.4636
1367961_at	24594	Ngfg	nerve growth factor, gamma	1419.583	193.2087	626.7015
1370313_at	26759	Acot7	acyl-CoA thioesterase 7	1419.179	4884.449	2717.486
1399120_at	362754	Trmt5_pre	TRM5 tRNA methyltransferase 5 homo	1418.283	1006.202	628.3829
1382288_at	315769	Trip4_pred	thyroid hormone receptor interactor 4 (	1418.252	786.9391	739.0602
1388415_at	311163	Ctnnd1_pre	catenin (cadherin associated protein),	1417.25	670.4531	815.1649
1374264_at	362304	LOC36230	similar to ORC5-related protein	1417.082	1946.755	1450.667
1374239_at	316639	Farp2_pre	FERM, RhoGEF and pleckstrin domain	1416.625	428.3564	1201.895
1376335_at	288718	Tfip11	tuftelin interacting protein 11	1416.6	1613.435	1621.357

1375451_at	309167	Pcnxl3	pecanex-like 3 (Drosophila)	1416.214	1828.695	604.7874
1383347_at	316344	Rev1l_pred	REV1-like (S. cerevisiae) (predicted)	1415.719	1752.789	2584.596
1383236_at	361927	Fxr1h	fragile X mental retardation gene 1, au	1415.54	1748.151	4357.47
1374448_at	313488	Reck_pred	reversion-inducing-cysteine-rich protei	1414.564	1458.364	6011.065
1382312_at	309728	Arid5b_pre	AT rich interactive domain 5B (Mrf1 lik	1414.527	687.6118	170.0624
1392866_at	360791	RGD13073	LOC360791 (predicted)	1414.187	513.5589	2271.888
1386991_a_at	64639	Bad	bcl2-associated death promoter	1413.091	5257.901	1427.191
1380564_at	360567	RGD15622	similar to novel protein (predicted)	1411.73	2532.527	1265.33
1371950_at	302422	Pdzd11_pr	PDZ domain containing 11 (predicted)	1410.996	2481.688	1497.9
1373840_at	305149	Nudt9	nudix (nucleoside diphosphate linked r	1410.816	891.3627	494.5127
1397409_s_at	308937	Wee1	wee 1 homolog (S. pombe)	1409.707	3449.126	2644.706
1371669_at	292007	Dhx38_pre	DEAH (Asp-Glu-Ala-His) box polypepti	1409.439	752.4827	1100.579
1379245_at	280671	Txndc9	thioredoxin domain containing 9	1409.157	1948.883	1293.8
1370353_at	79463	Timm22	translocase of inner mitochondrial mer	1409.131	3126.781	1283.559
1376001_at	313245	Praf1_pred	polymerase (RNA) I associated factor	1407.237	1464.629	607.8155
1377830_a_at	293587	RGD13111	similar to RIKEN cDNA 1810014F10 g	1406.93	2054.648	986.6713
1393621_at	399684	Vkorc1l1	vitamin K epoxide reductase complex,	1406.412	836.3044	557.675
1383346_at	304719	RGD15603	similar to ERCC4_MOUSE (predicted)	1404.379	1284.062	680.1682
1393559_at	81801	Stc1	stanniocalcin 1	1404.187	33973.78	1617.173
1377439_at	310137	RGD13100	similar to hypothetical protein FLJ1323	1403.973	795.589	1874.212
1394095_at	316687	RGD13074	similar to RIKEN cDNA 4930429M06R	1403.303	1329.294	2421.153
1374590_at	301173	Plcl2_predi	phospholipase C-like 2 (predicted)	1403.179	2000.562	936.2551
1389595_at	291978	Dus2l_pred	dihydrouridine synthase 2-like, SMM1	1402.492	873.7272	1252.043
1368081_at	79248	Abca2	ATP-binding cassette, sub-family A (AI	1402.062	1393.443	687.1474
1376325_at	315523	Nfrkb_pred	nuclear factor related to kappa B bindi	1401.05	1974.058	2250.816
1373293_at	288029	Lsg1	large subunit GTPase 1 homolog (S. c	1400.916	2341.673	1115.76
1369177_at	114554	Pi4k2a	phosphatidylinositol 4-kinase type 2 al	1400.802	1079.735	465.8537
1392966_at	314310	Zfp410_pre	zinc finger protein 410 (predicted)	1399.961	512.3797	1483.817
1383427_at	499751	RGD15635	similar to Endoplasmic reticulum manr	1399.955	613.4594	469.5525
1375440_at	360746	Ppil2	peptidylprolyl isomerase (cyclophilin)-li	1399.677	3184.847	648.0709
1388951_at	361543	RGD13083	similar to Trithorax homolog 2 (Mixed l	1398.269	1053.971	1104.973
1389154_at	305348	Lias	lipoic acid synthetase	1398.137	1463.032	1940.698
1382145_at	303787	Slc7a4_pre	solute carrier family 7 (cationic amino a	1397.587	2253.124	293.8513
1372086_at	295161	RGD13119	similar to CG32384-PA (predicted)	1397.559	1539.957	1804.782
1371740_at	362007	RGD13113	similar to RIKEN cDNA 5730470L24	1396.882	3524.368	1794.26
1373045_at	293497	Kctd13	potassium channel tetramerisation dor	1395.705	1599.379	954.3048
1387975_at	83626	Ugcg	UDP-glucose ceramide glucosyltransfe	1395.503	632.8717	952.0387
1393521_at	295324	Ttf2_predic	transcription termination factor, RNA p	1395.367	1527.525	752.6922
1381838_at	316371	Bivm_pred	basic, immunoglobulin-like variable mo	1395.055	570.8902	1662.349
1368571_at	29264	Cyln2	cytoplasmic linker 2	1394.588	1325.625	1061.821
1384472_at	362683	LOC36268	similar to RIKEN cDNA D330050P16 g	1394.109	1084.228	1926.978
1368187_at	113955	Gpnmb	glycoprotein (transmembrane) nmb	1392.539	32.71487	657.7651
1370995_at	171068	Pou2f1	POU domain, class 2, transcription fac	1391.706	232.37	736.4071
1375226_at	365372	Tbc1d10b_	TBC1 domain family, member 10b (pre	1391.497	1524.788	734.7781
1399125_at	316376	Inpp1	inositol polyphosphate-1-phosphatase	1391.335	1096.782	2526.605
1385485_x_at	315297	RGD15615	similar to Riken cDNA C230021P08 (p	1390.488	898.2011	2057.77
1391236_at	305268	RGD13083	similar to RIKEN cDNA 5730469D23 (p	1390.356	854.8239	3222.943
1398436_at	288482	Usp42_pre	ubiquitin specific protease 42 (predicte	1389.728	559.7105	304.4886
1368244_at	140925	As3mt	arsenic (+3 oxidation state) methyltran	1387.031	58.26853	4569.75
1370585_a_at	25023	Prkcb1	protein kinase C, beta 1	1383.773	4239.949	9284.268
1393843_at	315745	Fem1b_pre	feminization 1 homolog b (C. elegans)	1383.492	255.8743	2737.133

1371424_at	308976	RGD13048	similar to RIKEN cDNA 3110005O21	1382.062	1506.691	626.6481
1378458_at	304947	Pbx1_pred	pre-B-cell leukemia transcription factor	1381.488	124.619	263.8146
1372138_at	116639	Cutl1	cut-like 1 (Drosophila)	1380.671	6230.412	1650.663
1378987_at	294560	LOC29456	NA	1380.387	354.6164	1146.23
1394511_at	298247	Mysm1_pre	myb-like, SWIRM and MPN domains 1	1379.727	236.8442	1079.497
1388462_at	113907	Sbk1	SH3-binding kinase 1	1379.05	1577.009	1025.754
1390857_at	316067	Xylb	xylulokinase homolog (H. influenzae)	1377.545	229.9915	2075.31
1373243_at	310645	Pmvk	phosphomevalonate kinase	1376.528	1863.639	1595.09
1373424_at	303794	Dgcr6_pre	DiGeorge syndrome critical region gen	1376.07	1705.976	2483.281
1374605_at	309953	RGD13096	similar to hypothetical protein MGC332	1375.677	1743.33	2956.627
1391475_at	313842	Hnrpll_pre	heterogeneous nuclear ribonucleoprot	1375.629	5255.471	7226.196
1384432_at	266716	Zfp96	zinc finger protein 96	1375.264	843.5703	1372.68
1389597_at	292098	Pgbd5_pre	piggyBac transposable element derive	1374.946	1520.136	7413.786
1370196_at	83614	Pias3	protein inhibitor of activated STAT 3	1374.794	2598.276	2191.323
1372588_at	266609	Bles03	basophilic leukemia expressed protein	1374.622	902.6018	807.7865
1374575_at	362165	Creb3l1	cAMP responsive element binding prot	1374.078	619.2358	1421.059
1392474_at	303206	Ulk2_predi	Unc-51 like kinase 2 (C. elegans) (pre	1373.72	2225.232	1637.944
1373581_at	303222	RGD13056	similar to KIAA0672 gene product	1373.677	1019.102	2354.629
1373086_at	362269	RGD13108	similar to RIKEN cDNA F730014I05 (p	1373.229	2729.169	2426.745
1391448_at	298744	Crim1_pre	cysteine-rich motor neuron 1 (predicte	1371.726	408.1472	2614.122
1377777_at	308067	Brd9_predi	bromodomain containing 9 (predicted)	1371.009	3491.295	2680.61
1368116_a_at	83840	Rps6kb1	ribosomal protein S6 kinase, polypepti	1369.882	1402.148	1124.691
1388130_at	114636	Zyx	zyxin	1369.802	669.8255	1053.545
1370699_a_at	24329	Egfr	epidermal growth factor receptor	1369.379	2227.68	1591.98
1390579_at	290686	RGD13052	similar to RIKEN cDNA 1810029B16 (p	1369.31	1976.68	517.7496
1387078_at	80849	Inpp4a	inositol polyphosphate-4-phosphatase,	1369.268	1332.895	2731.646
1393230_s_at	290381	RGD13087	similar to KIAA0564 protein (predicted)	1369.073	413.6771	1609.911
1373018_at	315327	RGD15615	similar to hypothetical protein FLJ1480	1368.938	1697.995	713.0646
1388151_at	192276	Coro7	coronin 7	1368.378	1254.263	566.5531
1390294_at	362726	NA	NA	1367.509	939.7709	1409.245
1374983_at	361426	Klhdc4_pre	kelch domain containing 4 (predicted)	1367.286	520.7467	1121.185
1389269_at	362778	RGD13084	similar to RIKEN cDNA 4933433P14 g	1367.018	1514.725	1289.193
1393370_at	310698	RGD13083	similar to RIKEN cDNA 6330415M09 (	1366.65	1813.582	4109.165
1376874_a_at	310746	Ap4b1_pre	adaptor-related protein complex AP-4,	1366.201	1351.695	824.1519
1374560_at	291444	MGC12482	similar to RIKEN cDNA 1190002C06	1365.95	561.0002	570.8496
1370606_at	25265	P2ry1	purinergic receptor P2Y, G-protein cou	1365.405	2791.613	350.0113
1373982_at	287554	RGD13065	similar to hypothetical protein	1364.401	2995.3	1011.986
1395064_at	500551	RGD15649	similar to 4930429A08Rik protein (pre	1364.158	2022.05	637.0155
1368159_at	140669	Abcb6	ATP-binding cassette, sub-family B (M	1363.909	960.8243	725.5929
1383313_at	288734	Chfr	checkpoint with forkhead and ring finge	1363.279	2622.103	1357.958
1377010_at	363000	RGD15654	similar to 2610317D23Rik protein (pre	1363.164	1321.437	884.2621
1387916_at	266689	Cyp4f6	cytochrome P450 4F6	1362.752	282.7522	1441.055
1389314_at	289323	Nvl_predic	nuclear VCP-like (predicted)	1362.506	990.6159	1096.336
1370823_at	83837	Bambi	BMP and activin membrane-bound inh	1362.244	3155.466	2910.085
1372551_at	296741	Fastk	Fas-activated serine/threonine kinase	1361.087	995.9859	627.7156
1388620_at	362414	LOC36241	similar to Tada3l protein	1360.515	2043.301	906.9036
1395621_at	305350	RGD15651	similar to Mitochondrial carrier triple re	1359.832	3459.548	981.5288
1393144_at	311021	Nmi	N-myc (and STAT) interactor	1359.444	715.3205	857.4363
1393745_at	313138	Orc3l	origin recognition complex, subunit 3-li	1358.84	1725.334	753.6619
1392330_at	288309	Hemk2_pre	HemK methyltransferase family memb	1358.66	2265.934	762.8296
1372527_at	308410	Rtn2	reticulon 2 (Z-band associated protein)	1358.329	2628.569	513.6511

1370282_at	29317	Csrp2	cysteine and glycine-rich protein 2	1357.835	12273.79	5006.437
1371825_at	304204	Snapc2	small nuclear RNA activating complex,	1356.945	1119.984	1355.806
1373961_at	304176	MGC9520{	similar to 4930453N24Rik protein	1356.575	2183.707	2034.154
1376119_at	313865	RGD1560E	similar to thyroid adenoma associated	1356.474	805.1602	1125.801
1373894_at	246324	Rab31	RAB31, member RAS oncogene family	1355.871	1491.922	708.4886
1372753_at	299195	Coq6	coenzyme Q6 homolog (yeast)	1355.709	1437.355	1274.617
1374840_at	362580	RGD1305E	similar to CG2919-PA (predicted)	1355.071	4141.411	619.6777
1392980_at	304109	Tiam1	T-cell lymphoma invasion and metasta	1354.768	872.4875	796.7146
1374573_at	298767	Dync2li1	dynein cytoplasmic 2 light intermediate	1354.264	3627.05	3162.122
1384498_at	315333	Atf7_predic	activating transcription factor 7 (predic	1353.415	673.6709	1301.556
1371451_at	364974	Rnaseh2a	ribonuclease H2, large subunit	1353.399	3217.437	1568.888
1373588_at	309172	RGD1310З	similar to RIKEN cDNA 1200004M23	1351.663	2885.605	1717.938
1369134_x_at	117101	Kcnc3	potassium voltage gated channel, Sha	1351.557	325.3128	438.3531
1379676_a_at	363522	Dnase1l1	deoxyribonuclease 1-like 1	1350.887	1286.755	659.8712
1387008_at	65042	Sfxn3	sideroflexin 3	1350.166	3626.014	1185.475
1372883_at	362217	Cenpb_pre	centromere autoantigen B (predicted)	1349.71	1578.369	690.6216
1391849_at	300665	Mizf_predic	MBD2-interacting zinc finger (predicted	1349.369	652.2095	844.6296
1374136_at	291947	RGD13061	similar to CG9882-PA (predicted)	1349.094	714.7138	1813.111
1379982_at	304157	Nrip1_pred	nuclear receptor interacting protein 1 (	1347.527	987.7672	3508.266
1368544_a_at	85383	Nol3	nucleolar protein 3 (apoptosis represso	1347.47	1063.167	333.9083
1390563_at	302973	Abca3	ATP-binding cassette, sub-family A (AI	1347.308	1491.238	887.2808
1382376_at	361167	Bag4	BCL2-associated athanogene 4	1346.914	1124.282	1029.806
1367879_at	80278	Cdk5rap3	CDK5 regulatory subunit associated pı	1345.84	1322.198	440.0464
1397749_at	25744	Nf2	neurofibromatosis 2	1345.655	523.6844	489.2229
1368026_at	171073	Hdgfrp2	hepatoma-derived growth factor, relate	1344.719	2323.734	1112.21
1386983_at	25709	Hmbs	hydroxymethylbilane synthase	1344.49	1214.576	780.8881
1393792_at	362694	NA	NA	1344.016	676.7094	2459.138
1389911_at	316842	Metrnl	meteorin, glial cell differentiation regula	1343.62	789.5734	856.7224
1373147_at	306129	Fbxl3	F-box and leucine-rich repeat protein 3	1342.907	1027.161	4670.228
1396082_at	315394	Kbtbd3_pre	kelch repeat and BTB (POZ) domain c	1342.632	775.1316	554.3186
1396013_at	305457	Letm1	leucine zipper-EF-hand containing trar	1342.554	1437.939	445.0362
1374529_at	445442	Thbs1	NA	1342.089	105.8299	4898.15
1392896_at	500951	Olr1188_pı	olfactory receptor 1188 (predicted)	1341.851	1040.485	1162.002
1376644_at	311165	Med19_pre	mediator of RNA polymerase II transcr	1341.277	1110.08	486.7447
1396939_at	116548	Pcsk5	proprotein convertase subtilisin/kexin t	1341.236	88.46494	222.5111
1383655_at	305451	Tnip2	TNFAIP3 interacting protein 2	1339.647	492.1657	967.995
1382276_at	304580	Coro1c_pre	coronin, actin binding protein 1C (pred	1339.297	773.9803	375.4444
1376486_at	311848	Sh3glb2	SH3-domain GRB2-like endophilin B2	1337.067	1652.445	1545.046
1385845_at	502617	RGD1564E	similar to divalent cation tolerant protei	1336.316	610.1927	632.6552
1388706_at	362678	RGD1308S	LOC362678 (predicted)	1335.983	748.845	1344.088
1369799_at	81632	Abat	4-aminobutyrate aminotransferase	1335.882	1456.715	361.6705
1378742_at	362363	NA	NA	1335.654	332.0521	597.7219
1381564_at	289437	Glmn	NA	1334.123	1062.983	1939.939
1391897_at	304914	RGD1304E	similar to rab6 GTPase activating prote	1332.959	868.687	4987.025
1372440_at	29366	Serpine2	serine (or cysteine) proteinase inhibito	1332.792	312.1178	706.3187
1373037_at	295704	Ube2l6	ubiquitin-conjugating enzyme E2L 6	1332.254	4903.886	1041.931
1374777_at	502894	LOC50289	hypothetical protein LOC502894	1332.178	6593.132	4371.923
1381775_at	499613	RGD15661	RGD1566102 (predicted)	1332.049	590.6546	3985.365
1385809_at	296161	RGD13054	similar to Rnf37-pending protein	1331.741	1792.194	1887.856
1371883_at	303439	Mmd	NA	1331.377	977.3307	2636.335
1375047_at	361935	Spata5_pre	spermatogenesis associated 5 (predict	1329.942	1317.131	1678.474

1389765_at	303372	Nle1_predi	notchless homolog 1 (Drosophila) (pre	1329.922	1378.611	715.8713
1399112_at	294260	Skiv2l	superkiller viralicidic activity 2-like	1329.847	1387.684	969.8231
1391702_at	308365	Zfp446_pre	zinc finger protein 446 (predicted)	1329.078	701.2689	891.659
1397781_at	500843	RGD15654	similar to Hypothetical protein LOC270	1328.594	815.1194	1278.692
1389336_at	117241	Pop5_pred	processing of precursor 5, ribonucleas	1327.947	2238.702	1676.389
1373662_at	362112	Tor2a	torsin family 2, member A	1327.507	1251.758	384.6626
1371976_at	497875	NA	NA	1326.943	784.6945	268.3216
1375490_at	498130	RGD15648	similar to Serologically defined colon c	1326.907	74.39535	365.6747
1384377_at	364995	Ddx28_pre	DEAD (Asp-Glu-Ala-Asp) box polypept	1326.522	826.2535	522.2768
1372514_s_at	300078	Dnalc4	dynein, axonemal, light chain 4	1325.59	2065.1	920.2882
1375170_at	445415	S100a11	S100 calcium binding protein A11 (cali	1325.268	198.9585	5481.116
1393833_at	362765	Angel1_pre	angel homolog 1 (Drosophila) (predicte	1324.716	185.6841	1034.871
1382688_at	306000	RGD13593	mitochondrial carrier domain containin	1324.239	372.4021	460.6124
1371604_at	290632	Mrpl34	mitochondrial ribosomal protein L34	1323.905	3333.965	1033.03
1389452_at	303796	Zdhhc8	zinc finger, DHHC domain containing 8	1323.609	1327.073	1624.762
1393955_at	246152	Wdr44	WD repeat domain 44	1323.003	1985.947	3018.777
1379830_at	308738	Chd2_pred	chromodomain helicase DNA binding p	1322.4	1861.968	1680.62
1382781_at	296078	Pak6_pred	p21 (CDKN1A)-activated kinase 6 (pre	1322.323	1532.278	1662.956
1370566_at	299511	Rdh2	retinol dehydrogenase 2	1321.602	98.98758	180.3339
1369636_at	24788	Sord	sorbitol dehydrogenase	1321.49	3928.703	1149.349
1388724_at	361859	Cdc40_pre	cell division cycle 40 homolog (yeast) (	1321.332	1317.226	1298.175
1378489_at	312203	Tsga13_pr	testis specific gene A13 (predicted)	1320.111	111.2054	599.331
1393027_at	292267	RGD13052	similar to RIKEN cDNA 1700052N19	1319.747	1941.916	2526.562
1397552_at	313861	Eml4_pred	echinoderm microtubule associated pr	1319.721	829.4244	932.1873
1372400_at	361181	RGD15638	similar to cullin 4A (predicted)	1318.922	3378.217	4040.616
1376804_at	315840	RGD15606	similar to Myosin VI (predicted)	1317.085	414.7875	3305.157
1388510_at	287873	Chmp6_pre	chromatin modifying protein 6 (predicte	1316.753	2744.764	1123.078
1392913_at	293044	Zfp710_pre	zinc finger protein 710 (predicted)	1315.55	1062.863	1406.887
1393458_s_at	500030	RGD15637	similar to PHD finger protein 14 isofor	1315.261	1568.365	6320.838
1383685_at	361262	Heatr1_pre	HEAT repeat containing 1 (predicted)	1315.207	1257.739	579.474
1388423_at	294231	RGD13029	hypothetical protein MGC:15854	1314.93	3419.3	1693.292
1374593_at	29340	Prkce	protein kinase C, epsilon	1313.955	1032.152	780.1623
1380265_at	314228	Snapc1_pr	small nuclear RNA activating complex,	1313.118	584.8066	2590.782
1376175_at	498174	LOC49817	similar to NipSnap2 protein (Glioblasto	1312.726	258.6427	106.971
1371752_at	287419	RGD15631	similar to Ran-interacting protein MOG	1312.215	1508.043	768.3187
1369571_at	78961	Golph3	golgi phosphoprotein 3	1312.087	1143.323	1118.538
1389760_at	309457	Pcgf6	polycomb group ring finger 6	1312.039	4109.585	2691.841
1372662_at	305830	Fbxo34_pr	F-box only protein 34 (predicted)	1311.07	1548.692	10807.41
1393367_at	308576	Pnkp	polynucleotide kinase 3'-phosphatase	1311.023	948.166	831.7716
1395760_at	303317	RGD13074	similar to membrane protein expresse	1310.876	582.7348	480.4198
1380410_at	502947	NA	NA	1310.472	761.3943	1500.703
1375439_at	314617	Wdr18	WD repeat domain 18	1310.129	1826.145	625.9824
1392476_at	500351	RGD15623	histone H4 variant H4-v.1 (predicted)	1310.122	364.8022	659.1095
1393233_at	306352	Armc6_pre	armadillo repeat containing 6 (predicte	1309.812	1088.565	593.1488
1390820_at	313481	Zcchc11_p	zinc finger, CCHC domain containing 1	1309.803	2154.522	7360.433
1397410_at	312192	RGD15648	similar to RIKEN cDNA 4631427C17 (p	1309.609	153.7335	597.0227
1379849_at	290519	Thoc3_pre	THO complex 3 (predicted)	1309.304	4205.34	939.5983
1378675_at	366189	RGD15618	similar to transglutaminase E3 (predict	1309.111	517.7626	244.4935
1379261_at	287598	RGD13070	similar to RIKEN cDNA 1110001A07 g	1308.7	2844.439	1327.434
1379623_at	365458	RGD13094	similar to chromosome 10 open readin	1308.201	1867.37	1409.209
1388923_at	290626	RGD13115	similar to 2410004L22Rik protein	1307.476	799.881	612.1399

1368642_at	83501	Cdh2	cadherin 2	1307.181	1282.729	5708.097
1388665_at	287452	RGD13081	similar to RIKEN cDNA 1110020A23 (|	1307.13	2356.406	999.8528
1368658_at	25707	Cntf	ciliary neurotrophic factor	1307.03	4001.603	727.4738
1378178_at	313961	Smc6l1_pr	SMC6 structural maintenance of chron	1306.352	3198.101	2175.409
1370507_at	286930	Dlgap4	discs, large homolog-associated protei	1306.105	1494.182	971.1683
1377598_at	498332	MGC12482	NA	1305.326	1288.225	1095.503
1374425_at	362533	Tle1_predi	transducin-like enhancer of split 1, hon	1304.708	626.371	1691.836
1384149_at	362685	RGD13114	similar to hypothetical protein FLJ3834	1304.612	1438.345	395.2533
1388471_at	314683	Tcp11l2	t-complex 11 (mouse) like 2	1304.132	2954.743	24844.8
1387061_at	81679	Jup	junction plakoglobin	1304.117	3136.07	1547.296
1368403_at	81758	Rbl2	retinoblastoma-like 2	1304.109	2471.936	4828.533
1388828_at	315617	Sidt2_pred	SID1 transmembrane family, member	1303.539	693.5735	3253.777
1394566_at	308004	RGD13055	similar to hypothetical protein FLJ1318	1302.919	1286.843	886.0395
1380562_at	362416	Rpusd3_pr	RNA pseudouridylate synthase domair	1302.245	793.7017	510.686
1374379_at	360881	Dusp23_pr	dual specificity phosphatase 23 (predic	1301.46	610.0923	622.3699
1372152_at	362031	RGD13120	similar to RIKEN cDNA 5430428G01	1301.411	1074.043	1202.833
1379307_at	266809	Sap1	sodium channel associated protein 1	1300.619	1215.209	4143.314
1377998_at	304024	Cpox	coproporphyrinogen oxidase	1299.79	1714.019	1704.013
1370577_at	286979	Zfp455	zinc finger protein 455	1299.483	949.9288	553.4767
1389547_at	366975	Pphln1_pre	periphilin 1 (predicted)	1298.613	1110.707	1459.033
1379321_at	305960	Xkr6	X Kell blood group precursor related fa	1298.569	942.3277	1896.996
1374780_at	360389	Zfp422_pre	zinc finger protein 422 (predicted)	1297.193	2969.77	1822.701
1393722_at	302288	Fem1c_pre	fem-1 homolog c (C.elegans) (predicte	1296.81	1119.465	2849.538
1382052_at	362630	Fusip1	FUS interacting protein (serine-arginin	1296.532	1222.94	3416.554
1389187_at	315126	RGD13596	similar to RIKEN cDNA 1700088E04	1296.284	1208.756	2447.967
1376810_at	361046	Adprtl1	ADP-ribosyltransferase (NAD+; poly (A	1295.771	351.7264	1616.789
1367752_at	25414	Bcar1	breast cancer anti-estrogen resistance	1295.709	339.8095	822.4512
1375436_at	315085	Naprt1	nicotinate phosphoribosyltransferase c	1295.589	229.0132	1027.142
1382661_at	311182	Nup160_pr	nucleoporin 160 (predicted)	1295.357	2992.088	1068.796
1387415_a_at	81022	Stxbp5	syntaxin binding protein 5 (tomosyn)	1295.129	732.1016	690.2371
1389561_at	300516	RGD13113	similar to RIKEN cDNA 1810021J13	1294.403	954.527	1548.783
1383370_at	286895	Brinp2	BMP/retinoic acid-inducible neural-spe	1294.151	768.1907	219.0624
1370476_at	171565	Stambp	Stam binding protein	1293.75	2199.35	1155.857
1373031_at	308181	Trim8_prec	tripartite motif protein 8 (predicted)	1293.485	1939.568	1941.732
1398689_at	299264	NA	NA	1292.856	414.5242	627.181
1383099_at	499526	NA	NA	1292.366	2260.687	1692.573
1371624_at	315158	Zc3h7b_pr	zinc finger CCCH-type containing 7B (	1291.971	3841.873	2097.334
1372137_at	288785	Bloc1s1_pr	biogenesis of lysosome-related organe	1291.939	8717.838	1945.728
1372846_at	361729	Cybasc3	cytochrome b, ascorbate dependent 3	1291.456	1004.727	709.559
1373636_at	306759	Spock1	sparc/osteonectin, cwcv and kazal-like	1290.182	3522.8	41.68494
1368782_at	54305	Sstr2	somatostatin receptor 2	1290.174	896.4579	11948.86
1373264_at	361881	Zbed3	zinc finger, BED domain containing 3	1290.078	1042.829	939.1391
1375008_at	292554	Aurkc_prec	aurora kinase C (predicted)	1289.918	1403.718	879.2473
1372800_at	361514	Meis3_pre	Meis1, myeloid ecotropic viral integrati	1289.243	786.3717	865.0232
1390412_at	170840	Slc40a1	solute carrier family 39 (iron-regulated	1289.126	1705.719	2541.922
1376597_at	360524	Zcchc10	zinc finger, CCHC domain containing 1	1289.054	1863.364	2419.569
1371074_a_at	29685	Mcm6	minichromosome maintenance deficier	1289.034	10134.41	1144.46
1380305_at	501101	nod3l	NOD3-like protein	1288.258	1800.228	593.1152
1386795_at	296846	RGD13056	similar to hypothetical protein FLJ2553	1287.573	6519.216	7428.942
1397537_at	363433	LOC36343	NA	1287.33	4382.072	1669.196
1390977_at	295344	St7l	suppression of tumorigenicity 7-like	1286.773	3231.262	4600.854

1373103_at	361724	Mta2	metastasis-associated gene family, me	1285.531	1729.433	1071.451
1383409_at	500504	NA	NA	1285.44	525.2806	3310.077
1373135_at	619440	LOC61944	similar to hypothetical protein MGC274	1284.97	2907.548	1914.065
1370505_at	25371	Adprh	ADP-ribosylarginine hydrolase	1284.802	1276.029	683.1327
1377837_at	314313	Znf183	zinc finger protein 183 (RING finger, C	1284.422	384.5615	4750.552
1373657_at	298091	Slc31a2	solute carrier family 31, member 2	1284.135	1354.179	2017.423
1374000_at	287773	Helz_predi	helicase with zinc finger domain (predi	1284.006	601.6117	763.0597
1383307_at	497863	MGC11437	similar to RIKEN cDNA 4432406C05	1283.585	1335.591	948.5413
1398384_at	294975	Exosc9	exosome component 9	1283.33	1876.903	1627.882
1392556_at	305230	RGD13104	similar to PDZ domain actin binding pr	1283.152	1363.226	1840.827
1387371_at	171102	Cdc25a	cell division cycle 25 homolog A (S. ce	1282.173	817.549	345.1582
1371625_at	25739	Pygb	brain glycogen phosphorylase	1282.168	1413.144	1275.925
1387177_at	29555	Vipr2	vasoactive intestinal peptide receptor 2	1281.58	82.3419	311.8111
1369070_at	116718	Pex12	peroxisomal biogenesis factor 12	1281.153	1221.115	913.5135
1382379_at	94196	Rnf138	ring finger protein 138	1280.951	2463.94	11259.57
1373593_at	298933	Rnaseh1	ribonuclease H1	1280.086	1531.701	755.4446
1374055_at	292721	Erf_predict	Ets2 repressor factor (predicted)	1280.005	953.6827	906.5497
1387821_at	29885	Rab3ip	RAB3A interacting protein	1279.65	1945.058	955.061
1368372_at	24800	Sts	steroid sulfatase	1279.647	392.2164	396.8737
1368120_at	81733	Nell1	NEL-like 1 (chicken)	1279.25	382.0282	3416.001
1368463_at	114111	Vegfc	vascular endothelial growth factor C	1279.064	342.9485	1182.216
1375894_at	316102	Lztfl1	leucine zipper transcription factor-like 1	1278.857	853.2162	2751.873
1383369_at	309586	Trim26	tripartite motif protein 26	1277.343	1315.583	1668.743
1373584_at	498100	RGD15596	similar to hypothetical protein A430031	1276.978	1541.907	1885.663
1383960_at	311203	Pex16	peroxisome biogenesis factor 16	1276.577	1520.933	408.4589
1388194_at	81654	Dlat	dihydrolipoamide S-acetyltransferase (	1275.549	644.1388	717.0113
1367802_at	29517	Sgk	serum/glucocorticoid regulated kinase	1275	1806.958	4771.491
1374949_at	499871	LOC49987	similar to Zinc finger, FYVE domain co	1274.81	981.1924	729.6808
1376652_at	298566	C1qa	complement component 1, q subcomp	1274.429	560.9333	321.9378
1372457_at	306487	Mtus1	mitochondrial tumor suppressor 1	1273.588	832.0105	3496.667
1377801_at	302953	RGD15660	similar to BTB (PO)Z domain containin	1272.649	1125.748	1372.632
1381832_at	300035	Pycrl	pyrroline-5-carboxylate reductase-like	1272.467	2311.257	863.2041
1378853_at	312634	RGD13048	similar to FLJ22405 protein (predicted)	1271.901	1196.22	1016.975
1387369_at	50556	Exoc6	exocyst complex component 6	1271.407	2006.691	1287.852
1370177_at	25066	PVR	poliovirus receptor	1270.98	25.10997	1039.661
1391421_at	367046	RGD15642	similar to Acyl-CoA dehydrogenase fa	1270.677	1096.374	624.9048
1373716_at	361653	Armc5	armadillo repeat containing 5	1270.59	1112.907	679.9404
1379443_at	24268	Cp	ceruloplasmin	1270.267	1250.441	1435.003
1370565_at	286991	LOC28699	putative retrovirus-related gag protein	1269.902	430.1407	707.8845
1374100_at	500243	RGD15608	similar to 2300003P22Rik protein (pre	1268.762	2799.904	3137.946
1383743_at	498751	NA	NA	1268.226	1257.622	2207.571
1391972_a_at	304655	Zswim4_pr	zinc finger, SWIM domain containing 4	1267.377	546.2081	1588.732
1387140_at	64666	Taok2	TAO kinase 2	1267.193	793.4385	621.2697
1372384_at	363412	RGD15616	similar to zinc finger protein 609 (predi	1266.861	1268.186	1406.339
1381992_at	308107	Kptn_predi	kaptin (actin binding protein) (predicte	1266.696	1871.609	873.9077
1388826_at	361649	Atxn2l_pre	ataxin 2-like (predicted)	1266.456	724.8941	1428.433
1376042_at	363460	Fgd1	FYVE, RhoGEF and PH domain conta	1266.203	2819.534	6737.206
1370930_at	113886	Kif1c	kinesin family member 1C	1265.836	367.3125	1280.688
1388621_at	287456	RGD15632	similar to RIKEN cDNA 1110030J09 (p	1265.244	2656.047	831.7409
1371824_at	29223	Ak3l1	adenylate kinase 3-like 1	1265.195	502.5768	308.5009
1390930_at	289362	Gpatc2	G patch domain containing 2	1264.875	661.1386	1534.48

1398259_at	117021	Nup155	nucleoporin 155	1264.492	2862.551	998.1981
1370829_at	64511	Fntb	farnesyltransferase, CAAX box, beta	1264.254	1112.609	690.2449
1378264_at	298441	Nasp	nuclear autoantigenic sperm protein (h	1263.689	8561.217	2586.263
1390226_at	498840	RGD15625	similar to hypothetical protein LOC340	1263.552	333.0927	419.5683
1370330_at	246212	Sipa1l1	signal-induced proliferation-associated	1263.36	447.6129	656.3107
1379704_at	361627	Zfp143	zinc finger protein 143	1262.804	1557.384	2741.826
1383157_at	301552	Mrpl44	mitochondrial ribosomal protein L44	1262.427	1243.54	1147.345
1383251_at	290027	Parp2_pred	poly (ADP-ribose) polymerase family, r	1260.039	1403.763	1965.494
1367775_at	25284	Amacr	alpha-methylacyl-CoA racemase	1259.804	1214.581	1322.073
1370594_at	302822	Igsf1	immunoglobulin superfamily, member	1259.71	12275.66	784.1735
1377760_at	360828	Noc4l	nucleolar complex associated 4 homol	1258.788	3259.709	1061.758
1372899_at	311651	Slc9a8	solute carrier family 9 (sodium/hydroge	1258.125	946.2737	383.4749
1375720_at	81657	Gabbr1	gamma-aminobutyric acid (GABA) B re	1258.046	1440.401	4777.431
1368204_at	81513	Lig1	ligase I, DNA, ATP-dependent	1257.618	3624.056	1351.272
1387039_at	58920	Gpc1	glypican 1	1257.517	104.0304	723.8667
1391005_at	313101	Fbxl4_pred	F-box and leucine-rich repeat protein 4	1257.391	1428.587	1609.431
1373490_at	113940	Gmfg	glia maturation factor, gamma	1256.63	1678.195	304.3138
1379827_at	497941	LOC49794	similar to G protein pathway suppresso	1255.74	2987.177	756.3774
1389638_at	293343	MGC94288	similar to 4632419K20Rik protein	1255.517	1069.257	919.7549
1392449_at	362412	Rad18_pre	RAD18 homolog (S. cerevisiae) (predi	1255.487	3982.858	2956.798
1382436_at	363487	RGD15662	similar to RIKEN cDNA 2610002M06 (	1254.846	1127.4	1735.483
1376448_at	301549	Wdfy1	WD repeat and FYVE domain containi	1254.26	1047.026	3373.344
1374917_at	309858	Sec63_pre	SEC63-like (S. cerevisiae) (predicted)	1254.115	1453.242	3454.678
1376187_at	298280	Slc35d1_pr	solute carrier family 35 (UDP-glucuron	1253.655	1097.752	1241.993
1393102_at	363931	Gtpbp6_pr	GTP binding protein 6 (putative) (predi	1253.572	1935.314	1822.243
1383721_at	266707	Cx39	connexin 39	1253.306	307.4241	578.1399
1370183_at	25255	Dyrk1a	dual-specificity tyrosine-(Y)-phosphory	1251.383	617.0308	825.6771
1393459_at	24948	Fmr1	fragile X mental retardation syndrome	1251.146	3205.911	8184.578
1376949_at	309153	Rce1	Ras and a-factor-converting enzyme 1	1251.09	2750.471	952.8042
1371804_at	361052	Cdadc1	cytidine and dCMP deaminase domain	1250.463	1374.437	3425.946
1379347_at	314730	RGD13054	similar to RIKEN cDNA 1700023M03	1249.116	962.5978	1221.095
1392111_at	313243	Exosc3_pr	exosome component 3 (predicted)	1249.037	839.8581	1045.528
1379525_at	366196	RGD13110	similar to chromosome 20 open readin	1248.771	1228.841	3968.94
1384523_at	494322	MGC72584	similar to postmeiotic segregation incre	1248.134	1991.275	1100.976
1389555_at	406195	Tcf19	transcription factor 19	1247.986	9998.015	996.7009
1385007_at	309986	Zcchc9	zinc finger, CCHC domain containing 9	1247.898	1819.933	1783.519
1373667_at	311844	Ccbl1	cysteine conjugate-beta lyase 1	1247.785	1174.304	660.9779
1375034_at	361401	Lypla3	lysophospholipase 3	1247.391	939.5189	2002.322
1368546_at	29721	Hivep2	human immunodeficiency virus type I e	1246.669	1581.157	1743.179
1387948_at	84411	Ick	intestinal cell kinase	1246.418	690.4032	647.0289
1391052_at	361799	Dom3z	DOM-3 homolog Z (C. elegans)	1246.226	450.0893	373.9111
1373131_at	294322	Pknox1	Pbx/knotted 1 homeobox	1245.774	1477.754	1294.377
1367825_at	29622	Ralgds	ral guanine nucleotide dissociation stin	1245.363	948.7712	1932.94
1388729_at	361568	Rras_predi	Harvey rat sarcoma oncogene, subgro	1245.294	597.8394	532.6173
1380320_at	305171	RGD13113	similar to hypothetical protein	1245.102	1796.791	1771.287
1392886_a_at	304965	RGD13061	similar to FCRL	1243.928	403.3881	731.4641
1368164_at	116599	Blvra	biliverdin reductase A	1243.712	1390.504	647.4135
1374524_at	363285	Scly	selenocysteine lyase	1243.277	291.6959	781.9524
1393368_at	361686	Osbpl5	oxysterol binding protein-like 5	1242.806	1072.304	2102.164
1389195_at	114214	Dffa	DNA fragmentation factor, alpha subur	1242.279	781.4578	977.1733
1369148_at	24817	Tcf1	transcription factor 1	1242.276	747.2562	799.93

1379977_at	309000	RGD13065	similar to hypothetical protein MGC13(	1242.245	834.3661	401.8311
1380239_at	310506	Ppm1l_pre	protein phosphatase 1 (formerly 2C)-lil	1241.998	118.5965	249.1901
1391134_at	306607	Tmco3_pre	transmembrane and coiled-coil domair	1241.747	716.4099	552.3529
1390165_at	304055	RGD73511	similar to RIKEN cDNA 5830404H04	1241.055	739.7701	915.0298
1374085_at	360961	Mxd4_prec	Max dimerization protein 4 (predicted)	1239.542	1571.798	497.2952
1370931_at	363247	Xrcc5	X-ray repair complementing defective r	1238.674	999.8117	1192.4
1369712_at	65189	Stk3	serine/threonine kinase 3 (STE20 hom	1238.62	2033.408	1945.124
1380100_at	294917	RGD15618	similar to Traf2 and NCK interacting kir	1238.243	2309.485	1110.009
1389494_at	361715	Rps6ka4_p	ribosomal protein S6 kinase, polypepti	1237.702	2208.385	1054.692
1390101_at	313496	RGD15602	similar to hypothetical protein MGC319	1237.672	1846.687	1894.223
1370159_at	83833	Smarcd2	SWI/SNF related, matrix associated, a	1237.349	1396.667	489.6234
1390188_at	297601	Mrpl51_pre	mitochondrial ribosomal protein L51 (p	1236.672	591.353	393.508
1390483_at	314441	Slc25a29	solute carrier family 25 (mitochondrial	1234.868	354.9948	319.1172
1379521_at	287983	Alg3	asparagine-linked glycosylation 3 hom	1234.186	2012.37	473.3849
1389389_at	289780	Ddx56	DEAD (Asp-Glu-Ala-Asp) box polypept	1233.875	1014.573	247.065
1371603_at	361788	Prr3	proline-rich polypeptide 3	1233.062	962.2158	1385.87
1367723_a_at	58838	Lnk	linker of T-cell receptor pathways	1231.839	2004.719	2021.748
1388908_at	291075	Peci	peroxisomal delta3, delta2-enoyl-Coen	1231.461	1841.085	1790.917
1371394_x_at	498989	NA	NA	1231.142	1826.863	1357.717
1391207_at	500915	RGD15621	similar to TAFA5 (predicted)	1230.131	1984.638	998.4886
1383098_at	360726	Fyttd1	forty-two-three domain containing 1	1230.045	6171.178	2093.599
1390602_a_at	499749	LOC49974	similar to RIKEN cDNA C430004E15	1229.704	635.1591	613.4931
1391827_at	306204	Flnb_predi	filamin, beta (predicted)	1229.05	559.6055	461.4564
1393197_at	306338	Abhd8_pre	abhydrolase domain containing 8 (prec	1228.745	1683.941	1385.095
1378614_at	29424	Hdh	Huntington disease gene homolog	1228.623	414.1522	2562.863
1369785_at	117544	Ppat	phosphoribosyl pyrophosphate amidot	1228.316	1953.284	853.1671
1376811_a_at	299811	Cpsf6_prec	cleavage and polyadenylation specific	1228.187	1646.059	3354.504
1367996_a_at	65096	Lphn1	latrophilin 1	1227.21	793.4055	611.1077
1389326_at	288414	Rfc3	replication factor C (activator 1) 3	1226.909	6326.388	628.4894
1372709_at	298943	Bcap29	B-cell receptor-associated protein 29	1226.678	2574.341	2651.994
1388687_at	298541	Dhdds	dehydrodolichyl diphosphate synthase	1226.613	356.009	799.6609
1390667_at	293156	RGD15658	similar to Hypothetical 55.1 kDa protein	1225.658	297.3959	115.6214
1377667_at	366169	Rtf1_predic	Rtf1, Paf1/RNA polymerase II complex	1224.805	1186.838	1177.619
1376050_at	294018	Taf5_predi	TAF5 RNA polymerase II, TATA box b	1224.547	2357.631	1809.179
1369063_at	25379	Anp32a	acidic (leucine-rich) nuclear phosphopr	1224.361	1126.636	385.7029
1391040_at	291787	RGD13088	similar to Retinoblastoma-binding prote	1223.771	2487.945	1327.997
1373912_at	301261	Enpp4_pre	ectonucleotide pyrophosphatase/phos	1223.702	2027.928	2045.135
1367661_at	85247	S100a6	S100 calcium binding protein A6 (calcy	1223.598	97.782	545.3693
1372170_at	300981	Acy1	aminoacylase 1	1221.567	548.0716	1171.066
1374761_at	300066	Gga1	golgi associated, gamma adaptin ear c	1221.04	908.5782	743.3588
1375157_at	303504	Mllt6_predi	myeloid/lymphoid or mixed lineage-leu	1220.913	455.0935	555.5433
1395806_at	501290	NA	NA	1220.746	160.7031	1647.736
1383635_at	289402	Ddx59	DEAD (Asp-Glu-Ala-Asp) box polypept	1220.362	2072.247	1199.153
1373613_at	300191	LOC30019	similar to RIKEN cDNA 4930570C03	1219.797	978.8481	577.6359
1374494_at	361237	LOC36123	similar to testis-specific chromodomair	1219.4	3140.005	1529.22
1374068_at	362199	Vps39	vacuolar protein sorting 39 (yeast)	1219.365	2777.237	1333.112
1378800_at	300115	Arhgap8	Rho GTPase activating protein 8	1218.563	1085.736	799.6416
1385803_at	294693	Gtf2h2_pre	general transcription factor II H, polype	1218.346	1195.548	853.9399
1372034_at	303903	RGD13104	similar to hypothetical protein MGC293	1218.14	358.5656	1359.037
1389257_at	362375	Lancl2_pre	LanC (bacterial lantibiotic synthetase c	1217.317	3472.903	2433.294
1385236_at	310660	NA	NA	1216.229	1181.879	338.436

1372663_at	293620	Ptdss2_pre	phosphatidylserine synthase 2 (predict	1216.026	1041.612	573.2896
1395652_at	311202	Gyltl1b	glycosyltransferase-like 1B	1215.963	1780.82	642.7711
1387256_at	56777	Adam1a	a disintegrin and metallopeptidase dom	1215.596	1261.735	1188.673
1370884_at	29270	Spr	sepiapterin reductase	1215.447	2493.805	483.9031
1379315_at	293623	Rassf7_pre	Ras association (RalGDS/AF-6) doma	1215.431	2045.571	1318.509
1378160_at	310810	Sdfr2_pred	stromal cell derived factor receptor 2 (p	1214.803	273.5756	75.37933
1374624_at	311952	Galnt11	UDP-N-acetyl-alpha-D-galactosamine:	1214.791	3015.155	2825.322
1389322_at	363082	Pdcd7_pre	programmed cell death protein 7 (pred	1214.361	834.9452	2075.507
1389222_at	691143	LOC69114	NA	1213.826	2664.835	812.3905
1368509_at	113948	Bbs2	Bardet-Biedl syndrome 2 homolog (hu	1211.887	2018.238	3867.572
1371714_at	361811	Srpk1	serine/arginine-rich protein specific kin	1211.224	2496.877	1126.846
1370045_at	85472	Polg	polymerase (DNA directed), gamma	1210.883	1686.891	801.0009
1372499_at	299639	Ankrd24_p	ankyrin repeat domain 24 (predicted)	1210.607	1189.146	1345.436
1370446_at	171566	Nme7	non-metastatic cells 7, protein express	1209.82	1529.277	1283.789
1373377_at	310662	Scnm1_pre	sodium channel modifier 1 (predicted)	1209.687	700.4228	703.3174
1391027_at	303684	RGD15594	similar to tripartite motif-containing 65	1209.072	884.7608	1350.363
1368389_at	83611	Apba3	amyloid beta (A4) precursor protein-bi	1208.826	801.1273	287.1464
1384540_at	308495	Lrfn3_pred	leucine rich repeat and fibronectin type	1208.069	852.3761	542.3377
1374838_at	316580	Sp140	SP140 nuclear body protein	1207.751	181.5304	1120.333
1376132_at	362129	LOC36212	glycosyltransferase-like protein	1207.016	1154.013	1086.195
1380327_at	84016	Grip1	glutamate receptor interacting protein	1204.729	2018.547	1629.781
1377126_at	296285	Commd7	COMM domain containing 7	1204.635	3386.735	1155.789
1369448_at	117108	B3gat1	beta-1,3-glucuronyltransferase 1 (gluc	1204.398	382.6878	266.1282
1375938_at	363151	Ccdc12_pr	coiled-coil domain containing 12 (predi	1203.438	1368.87	631.354
1383213_at	312812	Eps8_pred	epidermal growth factor receptor pathv	1202.597	927.1511	393.4346
1389489_at	501633	NA	NA	1201.788	1251.006	2387.399
1377948_at	363255	RGD13592	ankyrin repeat containing protein RGD	1200.918	536.8194	416.2072
1384384_at	362461	RGD13045	similar to KIAA0528 protein (predicted)	1200.768	1132.035	555.2812
1389622_at	362322	RGD15658	similar to citrin (predicted)	1200.002	3778.426	602.4177
1387242_at	54287	Prkr	Protein kinase, interferon-inducible dou	1199.863	2371.765	1001.698
1373510_at	25624	Vamp1	vesicle-associated membrane protein	1199.435	692.1064	843.2726
1368709_at	81919	Fut1	fucosyltransferase 1	1198.375	317.2179	91.7883
1379632_at	296813	RGD13087	similar to mitochondrial carrier family p	1197.97	1970.602	939.9027
1385590_at	353250	Gpr109a	G protein-coupled receptor 109A	1197.287	49.71941	405.6894
1391212_at	302593	Tceal1	transcription elongation factor A (SII)-li	1196.749	3189.429	7727.128
1394985_at	314764	Eea1_pred	early endosome antigen 1 (predicted)	1195.694	758.1325	453.534
1376441_at	500671	NA	NA	1195.691	542.548	4234.244
1371670_at	300045	Exosc4_pre	exosome component 4 (predicted)	1195.495	1631.545	604.8254
1368075_at	25055	Lip1	lysosomal acid lipase 1	1195.438	955.1059	1011.693
1390352_at	312480	Pcgf1	polycomb group ring finger 1	1194.968	1936.459	1658.189
1368489_at	25445	Fosl1	fos-like antigen 1	1194.944	48.60326	1688.952
1389537_at	291571	Tcof1_pred	Treacher Collins Franceschetti syndro	1194.937	1030.269	753.5831
1371363_at	60666	Gpd1	glycerol-3-phosphate dehydrogenase 1	1194.915	5877.052	777.1658
1381388_at	304984	Ccdc19	coiled-coil domain containing 19	1194.796	89.57541	236.9078
1374341_at	287944	Thap7	THAP domain containing 7	1193.585	1282.021	784.0372
1372866_at	301067	RGD13119	similar to hypothetical protein MGC188	1192.434	408.1079	351.3202
1383654_a_at	498034	Fnsk_predi	similar to fructosamine-3-kinase (predi	1192.312	70.10046	2276.208
1373527_at	293723	NA	NA	1191.759	1219.465	918.0781
1380008_at	306647	RGD13112	similar to Neurofilament triplet H protei	1191.197	843.1435	586.9291
1376353_at	362258	Actr5_pred	ARP5 actin-related protein 5 homolog	1190.97	1877.294	742.9763
1374921_at	362288	RGD13067	similar to helicase-like protein NHL iso	1189.551	2689.872	982.0378

1387673_a_at	79125	Anxa6	annexin A6	1188.955	553.26	2435.218
1368387_at	117099	Bdh1	3-hydroxybutyrate dehydrogenase, typ	1188.28	3323.269	69.84514
1390819_at	29362	Tef	thyrotroph embryonic factor	1187.996	1567.562	1747.177
1392884_at	360921	Ripx	rap2 interacting protein x	1186.894	601.8675	909.8176
1379909_at	361202	Gkap1	G kinase anchoring protein 1	1186.319	5788.265	5778.148
1368692_a_at	29194	Chka	choline kinase alpha	1185.098	488.2817	3488.005
1373153_at	24558	Mog	myelin oligodendrocyte glycoprotein	1185.022	135.6622	902.7438
1370107_at	117184	Ctrl	chymotrypsin-like	1184.351	124.1085	3316.711
1372188_at	300149	MGC11441	similar to hypothetical protein D15Ertd	1183.657	3615.567	1383.575
1378378_at	296167	Pank2_pre	pantothenate kinase 2 (Hallervorden-S	1183.292	1427.931	1335.617
1390340_a_at	54242	Cpa3	carboxypeptidase A3	1182.544	1071.504	134.3166
1380693_at	501560	RGD1563	similar to ribosomal protein S6 kinase	1182.535	127.0037	304.8727
1371655_at	303244	Mpdu1	mannose-P-dolichol utilization defect 1	1182.506	3738.068	705.7424
1384400_at	290646	Dpde1	phosphodiesterase 4C, cAMP-specific	1182.327	1006.618	1135.616
1386956_at	25073	Scarb1	scavenger receptor class B, member 1	1181.129	2507.364	639.2429
1379910_at	498272	RGD1561	similar to UDP-N-acteylglucosamine p	1180.161	577.2943	1738.103
1381381_at	311909	Mnab_prec	membrane associated DNA binding pr	1179.779	286.6967	548.9254
1375909_at	24423	Gstm1	glutathione S-transferase, mu 1	1179.094	2359.94	794.3162
1396752_at	316256	Tnfrsf21_p	tumor necrosis factor receptor superfa	1178.921	33.36954	255.7536
1376560_at	362652	LOC36265	similar to KIAA0962 protein	1178.475	656.6441	650.4862
1376787_at	313156	RGD13107	similar to hypothetical protein FLJ3181	1178.11	34.03674	563.8998
1368874_a_at	64188	Mafg	v-maf musculoaponeurotic fibrosarcom	1178.028	688.4796	582.119
1370578_at	207123	RGD62109	similar to RIKEN cDNA D230025D16R	1177.554	1301.524	985.0264
1372940_at	498107	RGD15623	RGD1562339 (predicted)	1177.504	1545.54	1787.13
1396052_at	287727	Dhx8	DEAH (Asp-Glu-Ala-His) box polypepti	1177.491	1193.219	449.3199
1384411_at	296884	LOC29688	hypothetical LOC296884	1177.193	339.077	2981.497
1385953_at	114510	Mllt3	myeloid/lymphoid or mixed-lineage leu	1175.918	1043.441	1528.764
1370828_at	246326	Zdhhc2	zinc finger, DHHC domain containing 2	1175.085	4244.599	4239.52
1379472_at	353255	Nadsyn1	NAD synthetase 1	1174.662	623.8815	557.5179
1393932_at	305684	Samd8	sterile alpha motif domain containing 8	1174.013	599.5799	552.3014
1377152_at	288906	Rfx1_predi	regulatory factor X, 1 (influences HLA	1172.761	1747.945	1602.044
1395845_at	312777	LOC31277	similar to TEL protein	1172.416	25.41718	465.1097
1383238_at	64016	Qtrt1	queuine tRNA-ribosyltransferase 1	1172.192	2375.252	1791.812
1397644_at	298227	Mtap_pred	methylthioadenosine phosphorylase (p	1171.787	451.5511	908.8132
1375049_at	83838	Ltbp3	latent transforming growth factor beta	1171.466	1576.625	1954.177
1373988_at	688591	LOC68859	NA	1171.197	566.2761	387.9563
1390003_at	361783	Zfp57	zinc finger protein 57	1171.127	33.89742	655.1084
1379085_at	311553	RGD15618	similar to mKIAA0978 protein (predicte	1170.844	2084.245	1685.932
1374479_at	291737	RGD13101	similar to RIKEN cDNA D030070L09	1170.637	2467.377	2305.969
1370186_at	24967	Psmb9	proteosome (prosome, macropain) sub	1170.177	1439.14	931.2264
1388952_at	360583	RGD13079	similar to Hypothetical protein MGC18	1170.122	532.7943	397.0449
1390133_at	300811	Bnip2_prec	BCL2/adenovirus E1B 19kDa-interacti	1170.004	451.6159	1308.78
1377268_at	294667	RGD13105	similar to RIKEN cDNA 1200014M14	1169.918	982.7306	410.2335
1375981_a_at	302553	Suv39h1_p	suppressor of variegation 3-9 homolog	1169.586	3699.611	1749.789
1379457_at	299730	Nedd1_pre	neural precursor cell expressed, devel	1169.542	547.4322	246.6121
1380619_at	361116	RGD15656	similar to RIKEN cDNA 3110001I22 (p	1169.253	2509.136	1969.65
1391478_at	307362	Znf532_pre	zinc finger protein 532 (predicted)	1168.703	2246.616	1377.641
1389588_at	246272	Cant1	calcium activated nucleotidase 1	1167.667	873.9	941.5812
1368109_at	83505	St3gal5	ST3 beta-galactoside alpha-2,3-sialyltr	1166.431	2804.691	493.2955
1393583_at	498420	RGD15609	similar to RIKEN cDNA 5730466H23 (	1166.373	1191.835	1369.794
1388186_at	286911	LOC28691	cationic trypsinogen	1165.226	139.6975	21609.48

1395383_at	296312	RGD1311C	similar to RIKEN cDNA 0610011L14 g	1164.788	1155.725	1225.399
1380186_at	305911	RGD13063	similar to Zinc finger protein 198 (Fuse	1163.673	1239.368	4636.98
1382348_at	65156	Dhodh	dihydroorotate dehydrogenase	1163.501	476.021	298.5343
1376281_at	500909	LOC50090	similar to hypothetical protein FLJ2234	1163.234	226.7588	424.4673
1385208_at	309029	RGD1305C	similar to RIKEN cDNA 2310057M21	1163.156	1997.79	741.5954
1390386_at	25402	Casp3	caspase 3, apoptosis related cysteine	1161.683	4414.515	2162.047
1377809_at	554364	LOC55436	NA	1160.506	1158.045	1090.806
1368267_at	84430	Pomt1	protein-O-mannosyltransferase 1	1159.589	1416.491	711.4679
1372804_at	289562	RGD13594	MMR_HSR1 domain containing protein	1158.743	1067.812	1239.662
1379759_at	361185	RGD15626	similar to RIKEN cDNA 0710008K08 (j	1158.373	2229.041	745.736
1388002_at	286993	Taok1	TAO kinase 1	1158.253	1682.428	478.5714
1373284_at	299116	Sav1_pred	salvador homolog 1 (Drosophila) (pred	1158.241	896.1178	1700.72
1394568_at	299700	RGD15633	similar to DNA segment, Chr 10, Wayr	1158.126	1705.125	1060.783
1391440_at	298065	Tex10_pre	testis expressed gene 10 (predicted)	1157.919	2150.719	1496.667
1375991_at	308751	Nmb_predi	neuromedin B (predicted)	1157.265	1045.179	549.4184
1370409_at	170567	Slc38a1	solute carrier family 38, member 1	1157.232	581.6709	195.1215
1373307_at	315999	Mon1a_pre	MON1 homolog A (yeast) (predicted)	1157.052	508.695	396.0866
1374670_at	362824	RGD13077	similar to hypothetical protein MGC207	1156.433	700.1141	725.4198
1373899_at	296190	RGD13098	similar to dJ842G6.1.1 (novel protein)	1156.423	2543.712	1459.837
1369714_at	114481	Dnajc14	DnaJ (Hsp40) homolog, subfamily C, r	1156.072	1982.088	416.6525
1367842_at	29722	Apbb1	amyloid beta (A4) precursor protein-bi	1156.042	4814.213	1437.517
1373574_at	363886	RGD13076	similar to hypothetical protein MGC404	1155.883	1542.431	1463.692
1368973_at	81635	Adar	adenosine deaminase, RNA-specific	1155.873	1087.594	314.9792
1376143_at	360815	RGD15625	similar to ATP-dependent RNA-helicas	1155.276	1869.926	600.8026
1398472_at	309441	Peo1_pred	progressive external ophthalmoplegia	1155.122	1063.544	1143.177
1393752_at	315606	Mll_mappe	myeloid/lymphoid or mixed-lineage leu	1154.951	262.679	162.1895
1383779_at	309673	RGD13056	similar to RIKEN cDNA 2600005C20 (j	1153.384	1024.216	506.8956
1393366_at	299821	RGD1561C	similar to Inner nuclear membrane prot	1152.424	927.1695	3134.829
1390738_at	378947	Bst2	bone marrow stromal cell antigen 2	1152.313	286.0761	553.8245
1368634_at	27150	Kcnh3	potassium voltage-gated channel, subf	1150.907	982.1097	604.3621
1380164_at	361358	RGD1562C	similar to Nedd4-binding brain specific	1150.633	803.7541	758.7782
1371462_at	360622	Igfbp4	insulin-like growth factor binding protei	1150.492	525.2535	2772.449
1377818_at	293057	RGD15635	similar to junction-mediating and regula	1150.287	1603.642	560.4087
1389004_at	292876	Josd2_pre	Josephin domain containing 2 (predicte	1150.233	1344.63	711.2944
1376118_at	314405	Otub2_pre	OTU domain, ubiquitin aldehyde bindir	1150.109	1622.443	261.8481
1397439_at	497978	LOC49797	similar to diacylglycerol kinase epsilon	1149.999	1456.671	655.9077
1377727_at	314126	Baz1a_pre	bromodomain adjacent to zinc finger d	1149.883	423.1453	542.0028
1389011_at	299957	RGD13051	similar to RIKEN cDNA 1110014D18	1149.557	1720.969	681.5906
1373429_at	406169	Crebl1	cAMP responsive element binding prot	1149.272	1414.409	294.0491
1383664_a_at	293689	RGD15605	similar to chromosome 11 open readin	1148.975	2407.474	1181.708
1388913_at	246115	Ppap2c	phosphatidic acid phosphatase type 2c	1148.957	319.432	503.7456
1397571_at	259170	Casc3	cancer susceptibility candidate 3	1148.774	1509.732	699.1785
1384254_at	498803	RGD15633	similar to OTU domain containing 1 (pr	1148.376	1091.62	4173.023
1377835_at	499337	LOC49933	similar to dedicator of cytokinesis 8	1147.972	1077.219	349.1959
1388179_at	25366	Acvr2b	activin receptor IIB	1147.834	428.1499	618.6103
1376117_at	294255	Slc44a4	solute carrier family 44, member 4	1147.107	1079.658	447.0358
1389191_at	290851	Vps36_pre	vacuolar protein sorting 36 (yeast) (pre	1146.567	2267.993	1460.466
1387172_a_at	81809	Tgfb2	transforming growth factor, beta 2	1146.513	315.2265	507.7549
1387204_at	59318	Negr1	neuronal growth regulator 1	1145.938	709.6623	1157.917
1395571_at	305624	Rnf3_predi	ring finger protein 3 (predicted)	1145.703	1114.449	691.4395
1370306_at	64460	Pex14	peroxisomal biogenesis factor 14	1145.681	968.6114	691.0226

1389081_at	288659	Vps37b_pr	vacuolar protein sorting 37B (yeast) (p	1145.411	1629.974	1494.557
1388406_at	363545	RGD13089	similar to RIKEN cDNA 1200013P24	1144.717	938.9078	450.1369
1373170_at	303248	Gps2_pred	G protein pathway suppressor 2 (predi	1143.725	1080.355	1131.426
1372181_at	287524	Rpa1	replication protein A1	1143.441	3078.492	1018.977
1398242_at	65179	Ppp5c	protein phosphatase 5, catalytic subun	1142.851	1962.577	626.8857
1389614_at	499288	RGD15629	similar to ovary-specific MOB-like prote	1142.65	1083.656	679.5387
1376160_at	499390	LOC49939	similar to SWAP2	1142.515	360.4282	1117.459
1372428_at	89811	Vegfb	vascular endothelial growth factor B	1142.292	982.3575	975.1433
1369268_at	25389	Atf3	activating transcription factor 3	1141.851	25.78513	22288.97
1388482_at	362115	RGD13070	similar to RIKEN cDNA 9130404D14	1140.694	2850.316	804.4654
1374227_at	298782	RGD13052	similar to hypothetical protein FLJ2027	1140.551	1849.949	1050.716
1388532_at	361301	RGD13105	similar to hypothetical protein	1140.199	5576.047	657.412
1387103_s_at	65162	Dio2	deiodinase, iodothyronine, type II	1140.007	2056.845	2053.677
1371663_at	315329	RGD13051	similar to expressed sequence AW556	1138.861	1497.015	643.3519
1367919_at	58958	Pom210	nuclear pore membrane glycoprotein 2	1136.769	3743.002	3158.581
1390995_at	406168	Fkbpl	FK506 binding protein-like	1136.764	914.1137	506.4133
1374556_at	308652	Smox_prec	spermine oxidase (predicted)	1136.567	462.0547	705.9377
1376465_at	310233	RGD15638	sim ilar to glucocorticoid induced gene	1136.134	4659.031	5170.809
1397705_at	117096	Nlgn2	neuroligin 2	1135.415	1086.112	2328.646
1379957_at	303378	Slfn8	schlafen 8	1134.99	263.2182	1245.779
1389709_at	499900	LOC49990	similar to Zinc finger protein 133	1134.659	962.2357	925.6726
1367930_at	29423	Gap43	growth associated protein 43	1133.898	6334.676	1973.442
1390333_at	171366	Ppp4c	protein phosphatase 4, catalytic subun	1133.213	989.8605	341.5528
1370235_at	25045	Dbi	diazepam binding inhibitor	1133.003	11327.47	3284.446
1393304_at	29215	Arg2	arginase 2	1132.96	577.7855	820.398
1390031_at	304496	RGD13118	similar to hypothetical protein FLJ1446	1131.534	860.6681	371.8703
1392494_at	499331	LOC49933	similar to hypothetical protein D030056	1131.178	1423.623	984.7184
1376087_at	360632	RGD15611	similar to RIKEN cDNA 1300010M03 (	1130.95	1927.733	1514.523
1371879_at	298309	Lrrc42	leucine rich repeat containing 42	1130.568	5418.843	1581.612
1389769_at	288514	RGD13089	similar to KIAA1440 protein (predicted)	1130.37	1375.164	788.2576
1379114_at	310040	RGD13101	similar to KIAA0303 (predicted)	1130.309	55.50925	325.9563
1379484_at	311299	Aven_pred	apoptosis, caspase activation inhibitor	1130.154	615.5794	363.8609
1374523_at	290636	Pgls_predi	6-phosphogluconolactonase (predicted	1129.383	3129.747	823.1727
1388137_a_at	94269	Fez2	fasciculation and elongation protein ze	1129.26	1815.942	985.4333
1373871_at	313905	RGD15622	similar to STAGA complex 65 gamma	1128.258	2153.546	807.3596
1389367_at	295105	Schip1	schwannomin interacting protein 1	1128.026	1484.524	5246.496
1371213_at	309627	RT1-A3	RT1 class I, A3	1127.785	43.58805	277.4774
1388675_at	361648	RGD13056	similar to spinster-like protein	1127.343	1475.804	1292.2
1389570_at	287533	LOC28753	similar to putative phosphoinositide 5-p	1127.188	1457.214	909.4244
1386435_at	360482	Tcfap4_pre	transcription factor AP4 (predicted)	1126.506	606.9152	1010.162
1381279_at	362491	Ripk2	receptor (TNFRSF)-interacting serine-t	1126.287	590.9416	1333.829
1369092_at	117513	Sec22l2	sec22 homolog	1126.029	1440.863	548.3852
1398691_at	499624	LOC49962	NA	1126.002	205.4556	2970.609
1373708_at	499314	MGC12503	similar to RNA binding motif protein 21	1125.694	710.8704	498.2095
1383295_at	311535	RGD13089	similar to RIKEN cDNA 5430432M24	1124.687	1328.9	1483.154
1372290_at	294258	Rdbp	RD RNA-binding protein	1123.879	2502.595	889.8063
1389471_at	311621	Tomm34_p	translocase of outer mitochondrial mer	1122.843	2825.899	1379.245
1374827_at	114002	Ndst2_prec	N-deacetylase/N-sulfotransferase (hep	1122.469	1175.085	638.8473
1380071_at	362343	Parp12_pre	poly (ADP-ribose) polymerase family, r	1122.273	1145.077	1650.957
1372470_at	303798	Arvcf_pred	armadillo repeat gene deleted in velo-c	1122.1	1303.585	503.0508
1374892_at	360547	Sat2_predi	spermidine/spermine N1-acetyl transfe	1121.858	676.1486	543.1486

1373073_at	301618	Ppp1r7	protein phosphatase 1, regulatory (inhi	1121.819	1897.337	482.8924
1378061_at	312651	Wdr10	WD repeat domain 10	1121.394	1872.126	1853.689
1389467_at	497979	MGC10877	similar to RIKEN cDNA 1810057C19	1120.361	5557.279	303.9261
1374886_at	301514	Bcs1l	BCS1-like (yeast)	1119.328	654.1356	578.3631
1392967_at	498830	MGC11612	similar to RIKEN cDNA 2700062C07	1119.103	2331.531	1117.118
1376328_at	301351	RGD13108	similar to putative protein (5S487) (pre	1118.459	754.6422	1197.82
1383876_at	313365	Ift74	intraflagellar transport 74 homolog (Ch	1118.353	965.3477	2306.481
1379500_at	498404	LOC49840	similar to cDNA sequence BC018601	1117.561	736.0288	1294.858
1372870_at	315131	Kdelr3_pre	KDEL (Lys-Asp-Glu-Leu) endoplasmic	1117.029	558.8651	1851.845
1371749_at	362975	RGD13060	similar to 2210021J22Rik protein (prec	1116.007	1511.379	725.6329
1373119_at	305055	Kctd3	potassium channel tetramerisation dor	1115.059	1649.677	1943.528
1370721_a_at	83686	Cngb1	cyclic nucleotide gated channel beta 1	1114.868	811.9446	379.0132
1384456_at	313427	Usp24_pre	ubiquitin specific protease 24 (predicte	1114.33	1042.759	753.8388
1371712_at	292030	NA	NA	1114.144	3071.432	640.7328
1392186_at	368088	RGD15633	similar to diacylglycerol kinase, delta 1	1113.928	600.9855	626.6642
1379560_at	306764	Nsd1_pred	nuclear receptor binding SET domain p	1113.897	996.4126	1029.047
1369813_at	79130	Dnajc5	DnaJ (Hsp40) homolog, subfamily C, r	1113.189	864.2749	208.9019
1379969_at	502886	RGD15651	similar to fork head transcription factor	1113.16	605.9393	1735.195
1367843_at	171445	Akr7a2	aldo-keto reductase family 7, member	1112.857	864.2197	1035.42
1392040_at	310807	Sass6_pre	spindle assembly 6 homolog (C. elega	1111.706	587.2372	370.9432
1376024_at	361618	Stim1_prec	stromal interaction molecule 1 (predicte	1110.471	2217.167	809.0507
1373669_at	289608	Gnpda2_pr	glucosamine-6-phosphate deaminase	1110.134	1268.557	4491.947
1383522_at	501513	NA	NA	1110.081	525.998	1962.397
1370075_at	24312	Dhfr	dihydrofolate reductase	1110.039	5012.124	713.8111
1368323_at	29436	Tfpi	tissue factor pathway inhibitor	1109.951	2658.788	13584.18
1367950_at	29726	Slc22a5	solute carrier family 22 (organic cation	1109.868	1068.848	1700.641
1392219_at	50659	Nr2c2	nuclear receptor subfamily 2, group C,	1109.5	292.3243	982.6028
1367963_at	64226	Gbl	G protein beta subunit-like	1109.473	1394.51	882.119
1368417_at	54309	Syt5	synaptotagmin V	1109.23	5315.921	902.849
1399086_at	316096	Zfp105	zinc finger protein 105	1108.985	873.4684	678.358
1368409_at	29487	Gstt2	glutathione S-transferase, theta 2	1108.447	437.794	496.7772
1388859_at	246768	Pgea1	PKD2 interactor, golgi and endoplasmi	1107.96	2250.543	908.4447
1374569_at	308592	Grwd1	glutamate-rich WD repeat containing 1	1107.916	1091.642	577.7161
1368331_at	81652	Ctbs	chitobiase, di-N-acetyl-	1107.046	4213.543	2617.05
1398569_at	293625	Lrdd_predi	leucine-rich and death domain contain	1106.986	813.0872	565.2106
1384029_at	298074	Xpa_predic	xeroderma pigmentosum, complement	1106.854	945.9047	1665.981
1398122_at	25681	Col10a1	procollagen, type X, alpha 1	1106.487	812.2074	270.5411
1379858_at	363687	Mettl2_pre	methyltransferase like 2 (predicted)	1106.438	1419.567	454.5395
1369564_at	64626	Rem2	rad and gem related GTP binding prote	1106.286	697.8476	755.9527
1383715_at	362521	RGD13057	similar to hypothetical protein D4Ertd8	1106.149	930.0039	849.3524
1382109_at	298342	2610020o0	nuclear NF-kappaB activating protein	1106.029	954.4644	223.3643
1376500_at	306771	Fbxo23	F-box only protein 23	1105.875	2360.258	797.5585
1377390_at	288227	Bace2	beta-site APP-cleaving enzyme 2	1105.582	6905.278	294.0545
1379784_at	308718	Pex7	peroxisome biogenesis factor 7	1105.545	1982.193	2337.116
1389792_at	303000	Solh_predi	small optic lobes homolog (Drosophila	1104.676	739.7822	862.5152
1376724_at	64562	Prkab2	protein kinase, AMP-activated, beta 2	1104.304	364.3099	2316.741
1389919_at	362973	Parvb_prec	parvin, beta (predicted)	1103.967	746.1929	145.4589
1371414_at	296654	Gsn	gelsolin	1103.736	2221.568	7204.871
1389320_at	364637	LOC36463	similar to F-box only protein 25	1103.427	2658.991	1396.308
1392548_at	362153	RGD15653	similar to mKIAA1604 protein (predicte	1103.213	760.6009	1889.645
1384289_at	361313	RGD13052	similar to RIKEN cDNA 4631403P03	1102.943	1024.603	1168.046

1387407_at	170914	Nap1l3	nucleosome assembly protein 1-like 3	1102.777	1069.429	5558.436
1375342_at	29228	Nfic	nuclear factor I/C	1102.581	1013.895	1303.753
1372618_at	362084	RGD13076	similar to hypothetical protein FLJ1304	1102.194	1544.831	1422.007
1397370_at	304375	Hrbl_predic	HIV-1 Rev binding protein-like (predicte	1101.194	140.3875	1128.618
1368889_at	65277	Vti1a	vesicle transport through interaction wi	1100.844	491.1208	402.4118
1385462_at	293694	Map4k2_pr	mitogen activated protein kinase kinas	1100.531	540.4725	550.9469
1387898_at	192245	Hspb6	heat shock protein, alpha-crystallin-rela	1100.008	1564.904	1845.137
1393799_at	60630	Unc5b	unc-5 homolog B (C. elegans)	1099.702	485.8785	504.9379
1374837_at	293514	Bcl7c_pred	B-cell CLL/lymphoma 7C (predicted)	1099.568	831.3139	681.9845
1385308_at	363261	Mogat1_pr	monoacylglycerol O-acyltransferase 1	1098.912	145.7358	274.4723
1393594_at	310658	Pogz_pred	pogo transposable element with ZNF d	1098.784	310.5876	1676.415
1372916_at	361883	Mrps27_pr	mitochondrial ribosomal protein S27 (p	1098.518	487.5044	717.0334
1380717_at	361529	Znf626_pre	zinc finger protein 626 (predicted)	1098.073	1178.072	676.4003
1370473_a_at	117552	Ptpn23	protein tyrosine phosphatase, non-rece	1097.866	318.825	1295.028
1390876_at	366174	Catsper2	cation channel, sperm associated 2	1097.728	1185.118	1226.511
1371738_at	301111	RGD13082	similar to hypothetical protein FLJ1037	1097.023	746.7007	676.5213
1374739_at	500727	Cdca4	cell division cycle associated 4	1096.248	1730.019	2771.284
1372250_at	499941	LOC49994	SCF apoptosis response protein 1	1096.119	4526.071	1417.794
1393205_at	498109	RGD15612	similar to polymerase (RNA) II (DNA d	1094.837	804.0065	39.22365
1380019_at	293650	Tcirg1	T-cell, immune regulator 1	1094.264	233.5376	850.6487
1381164_at	311919	Golga1_pre	golgi autoantigen, golgin subfamily a, 1	1094.042	362.4709	395.5067
1370812_at	24888	Bcl2l1	Bcl2-like 1	1094.02	857.5345	528.5368
1391604_at	315508	LOC31550	NA	1093.983	3791.673	1334.817
1383117_at	282634	Pxmp4	peroxisomal membrane protein 4	1093.806	2495.982	653.4188
1383558_at	500255	RGD15605	similar to MJ0495-like protein SelB (pr	1093.282	1406.607	574.4479
1391455_at	292067	Nhn1	conserved nuclear protein Nhn1	1093.196	756.0502	751.4304
1378962_at	287766	RGD13096	similar to RIKEN cDNA 4732496G21 g	1092.905	550.5222	1317.288
1393047_at	292188	Ibrdc1_pre	IBR domain containing 1 (predicted)	1091.832	1405.979	1999.036
1374962_at	309912	RGD15633	similar to NOGO-interacting mitochond	1091.522	218.2669	724.9668
1399092_at	362608	Tmem39b	transmembrane protein 39b	1090.921	1224.456	901.3447
1387023_at	81869	Gstm3	glutathione S-transferase, mu type 3	1090.744	2875.9	2545.072
1373832_at	315478	Snx19_pre	sorting nexin 19 (predicted)	1090.128	1722.274	819.1625
1372018_at	113960	Cdc42bpb	Cdc42 binding protein kinase beta	1090.106	1197.559	523.4893
1397349_at	290802	Ubxd6_pre	UBX domain containing 6 (predicted)	1090.088	1330.628	1360.574
1371520_at	117521	Znf291	zinc finger protein 291	1089.174	862.9217	1215.828
1373760_at	310672	Tarsl1	threonyl-tRNA synthetase-like 1	1089.073	1435.237	1429.057
1368308_at	24577	Myc	myelocytomatosis viral oncogene hom	1088.853	502.7202	621.1074
1390868_at	289088	RGD13086	similar to RIKEN cDNA 1200016B10 (p	1088.797	825.0446	974.2702
1379337_at	301463	RGD13078	similar to RIKEN cDNA 2810421I24	1087.873	1120.178	931.3502
1377768_at	291772	Kctd1	potassium channel tetramerisation dor	1087.868	1326.535	1256.68
1394086_at	288167	Senp7_pre	SUMO1/sentrin specific protease 7 (pr	1087.85	2092.572	4882.293
1371397_at	292894	Nosip_prec	nitric oxide synthase interacting protein	1087.456	1084.729	759.9518
1384565_at	362557	LOC36255	hypothetical protein LOC362557	1087.308	6614.185	1255.871
1399134_at	500054	LOC50005	similar to POT1-like telomere end-bind	1087.285	1795.727	1599.599
1376921_at	315547	Foxred1_p	FAD-dependent oxidoreductase doma	1087.135	944.3583	564.4313
1390653_at	314243	RGD13073	similar to hypothetical protein MGC990	1085.843	379.0871	479.7227
1376100_at	307351	Tubb6	tubulin, beta 6	1085.571	157.6986	758.3964
1390138_at	494320	MGC72567	similar to coiled-coil domain containing	1084.949	56.29094	706.9748
1375627_at	297968	RGD13077	similar to hypothetical protein FLJ1034	1084.534	3310.578	566.7465
1370375_at	192268	Gls2	glutaminase 2 (liver, mitochondrial)	1084.476	90.82164	1419.276
1388806_at	305837	Ttc5	tetratricopeptide repeat domain 5	1083.815	1030.531	1150.925

1370052_at	81745	Pdpk1	3-phosphoinositide dependent protein	1083.41	993.6349	1210.882
1367924_at	171444	Pacs1	phosphofurin acidic cluster sorting prot	1082.858	1396.533	1869.294
1373270_at	303630	Wipi1_prec	WD repeat domain, phosphoinositide i	1082.489	8501.252	1396.336
1372535_at	366431	LOC36643	similar to RIKEN cDNA 2210012G02	1082.073	1226.985	1325.576
1370260_at	25230	Add3	adducin 3 (gamma)	1081.96	1680.372	561.0688
1368183_at	25738	Plcg1	phospholipase C, gamma 1	1081.75	2479.571	1073.405
1373512_at	362843	Ilvbl_predic	ilvB (bacterial acetolactate synthase)-li	1080.598	2399.758	341.1087
1368837_at	84481	Arid4b	AT rich interactive domain 4B (Rbp1 lik	1079.326	641.4808	2706.397
1377797_at	312706	Emg1_prec	EMG1 nucleolar protein homolog (S. c	1078.862	955.9031	515.1088
1382137_at	291793	Abhd3_pre	abhydrolase domain containing 3 (prec	1078.492	760.3559	1761.058
1393956_at	170904	Stk17b	serine/threonine kinase 17b (apoptosis	1077.999	1473.229	828.7404
1372004_at	362454	Hebp1_pre	heme binding protein 1 (predicted)	1077.242	80.20187	214.457
1375846_at	289424	Xpr1_predi	xenotropic and polytropic retrovirus rec	1076.711	1670.024	2207.552
1390448_at	306630	RGD13083	similar to 1110065L07Rik protein (prec	1076.709	2732.363	779.9727
1373885_at	300266	Cbx5_pred	chromobox homolog 5 (Drosophila HP	1076.645	3166.199	1656.562
1379475_at	499895	RGD15631	similar to RIKEN cDNA 2210009G21 (	1076.441	1222.866	529.5385
1368343_at	117018	Kcnh2	potassium voltage-gated channel, subf	1076.314	600.3313	350.4797
1385869_at	305083	Zfp281	zinc finger protein 281	1075.573	1722.844	4383.044
1376297_at	366001	Arrdc1	arrestin domain containing 1	1075.241	1696.623	733.4971
1387059_at	54348	Stk39	serine/threonine kinase 39, STE20/SP	1075.188	2734.3	2653.791
1373744_at	311412	Anapc1_pr	anaphase promoting complex subunit	1075.162	1822.447	580.924
1376863_at	362257	RGD13068	similar to RIKEN cDNA B230339M05 g	1075.04	1563.036	1074.818
1372748_at	315880	Tbc1d2b	TBC1 domain family, member 2B	1074.532	818.6253	1254.488
1389180_at	361377	LOC36137	NA	1073.661	466.8448	2422.012
1368249_at	85497	Klf15	Kruppel-like factor 15	1073.382	38.33452	456.5704
1370247_a_at	24660	Pmp22	peripheral myelin protein 22	1072.997	1896.62	1072.675
1373138_at	361274	Nudt5	nudix (nucleoside diphosphate linked r	1071.894	1758.626	851.3897
1371374_at	315093	Maf1	MAF1 homolog (S. cerevisiae)	1071.829	3267.055	1536.564
1387185_at	117026	Apbb3	amyloid beta (A4) precursor protein-bir	1071.756	2401.927	944.8117
1372934_at	299209	RGD13073	similar to 1700019E19Rik protein (prec	1071.369	1138.556	1570.023
1398911_at	301240	Mrpl2	mitochondrial ribosomal protein L2	1070.785	2392.069	908.2257
1379257_at	307514	Epb4.1l4a_	erythrocyte protein band 4.1-like 4a (pr	1070.258	6192.677	3490.449
1372650_at	309362	LOC30936	NA	1070.209	612.7117	1921.299
1371803_at	282838	Gm2a	GM2 ganglioside activator protein	1069.62	666.8482	1284.636
1382265_at	306306	RGD13046	similar to KIAA1128 protein (predicted)	1069.428	535.3461	519.3177
1372451_at	316209	Trfp	Trf (TATA binding protein-related facto	1069.305	1690.818	964.7042
1399099_at	361522	Hnrpul1_pr	heterogeneous nuclear ribonucleoprote	1068.783	889.9394	596.134
1372692_at	303882	Tnk2	tyrosine kinase, non-receptor, 2	1068.285	2295.781	1837.686
1375865_at	316233	Tjap1_prec	tight junction associated protein 1 (pre	1068.265	628.7336	597.7004
1389764_at	298447	Dmap1	DNA methyltransferase 1-associated p	1068.03	2262.553	772.0625
1391425_at	301009	Nckipsd_pr	NCK interacting protein with SH3 doma	1067.976	408.3895	1186.349
1378215_at	293489	Giyd2	GIY-YIG domain containing 2	1067.826	710.1057	1457.702
1373327_at	316214	Tcfeb	transcription factor EB	1067.638	740.254	1235.501
1382389_at	140898	Arhgef5	Rho guanine nucleotide exchange fact	1067.475	603.1137	1547.355
1389620_at	308345	Suv420h2_	suppressor of variegation 4-20 homolo	1065.899	895.915	1270.309
1373143_at	316982	RGD13096	similar to hypothetical protein FLJ1065	1065.537	2491.008	1591.284
1398608_at	289150	RGD13118	similar to hypothetical gene supported	1063.416	2497.441	816.4208
1367786_at	24968	Psmb8	proteosome (prosome, macropain) sub	1063.131	1319.709	820.6465
1388572_at	301128	LOC30112	NA	1062.827	822.226	744.0781
1399040_at	298399	Gba2	glucosidase beta 2	1062.509	239.372	890.9787
1398953_at	300036	Tsta3_prec	tissue specific transplantation antigen	1062.379	1880.884	504.6769

1374146_at	313702	Mad2l2	MAD2 mitotic arrest deficient-like 2 (ye	1062.229	806.5584	663.6905
1367743_at	25741	Pfkl	phosphofructokinase, liver, B-type	1062.171	523.7816	1328.97
1389190_at	365468	Lzts2	leucine zipper, putative tumor suppres	1061.328	841.527	517.0125
1372738_at	314630	Rexo1	REX1, RNA exonuclease 1 homolog (S	1061.278	1420.802	985.088
1388453_at	369016	Myadm	myeloid-associated differentiation marl	1061.185	654.829	1307.398
1388943_at	315058	Chrac1_pre	chromatin accessibility complex 1 (pre	1060.326	2948.457	1113.184
1378217_at	305888	Zfhx2	zinc finger homeobox 2	1060.31	270.9119	493.141
1370307_at	25592	Agrn	agrin	1060.128	801.4287	710.5492
1373960_at	288591	RGD13114	similar to transmembrane protein induc	1059.916	483.8375	68.25599
1383831_at	309911	Qrsl1	glutaminyl-tRNA synthase (glutamine-l	1059.617	868.9055	431.1138
1372475_at	298575	Pink1_prec	PTEN induced putative kinase 1 (predi	1059.113	633.4945	1226.473
1367729_at	64313	Oat	ornithine aminotransferase	1058.361	5514.425	1716.675
1373833_at	293059	RGD13057	similar to RIKEN cDNA 3110040N11	1057.982	1941.473	635.5662
1386913_at	54320	Pdpn	podoplanin	1057.346	87.3677	2696.649
1372483_at	304302	Zfp469_pre	zinc finger protein 469 (predicted)	1057.16	473.9101	805.3215
1367828_at	64304	Acads	acetyl-Coenzyme A dehydrogenase, s	1056.413	1134.273	614.9872
1383785_at	161452	Lef1	lymphoid enhancer binding factor 1	1055.628	217.8123	364.8024
1383945_at	171570	Uck2	uridine-cytidine kinase 2	1055.373	1118.898	1198.228
1389657_at	365179	RGD15654	similar to zinc finger protein 524 (predi	1054.601	812.7582	486.343
1393199_at	317432	RGD15606	similar to Jumonji/ARID domain-contai	1054.6	303.6159	2361.671
1379665_at	294711	Ppwd1_pre	peptidylprolyl isomerase domain and V	1054.529	1597.68	1774.368
1391885_at	294594	Spata9_pre	spermatogenesis associated 9 (predict	1054.071	1236.22	2867.767
1372670_at	300675	RGD13076	similar to hypothetical protein FLJ2182	1054.061	912.1165	1964.315
1379287_at	312937	Rp1h	retinitis pigmentosa 1 homolog (humar	1053.997	1226.22	846.5359
1387322_at	84609	Sema6b	sema domain, transmembrane domair	1053.684	694.5956	480.5881
1389532_at	307189	Nebl_predi	nebulette (predicted)	1053.623	6393.254	392.0193
1388731_at	291403	Zadh2_pre	zinc binding alcohol dehydrogenase, d	1053.103	3107.112	682.1322
1392922_at	170923	Rap2b	RAP2B, member of RAS oncogene far	1052.701	1139.376	1947.619
1368676_at	65209	Dnch2	dynein, cytoplasmic, heavy polypeptide	1052.476	107.422	1150.214
1390366_at	291002	RGD15605	similar to PC-LKC gene product (predi	1051.47	983.9394	1532.693
1376498_at	304469	RGD13079	similar to 2900002H16Rik protein (pre	1051.141	1357.673	854.985
1372831_at	300114	RGD13064	similar to leucine zipper, down-regulate	1051.011	1603.918	1302.248
1378181_at	291071	Rpp40	ribonuclease P 40 subunit (human)	1050.941	1729.83	647.4218
1371915_at	363133	Pcbp4_pre	poly(rC) binding protein 4 (predicted)	1050.672	1741.499	1430.072
1382055_at	297383	Rtkn	rhotekin	1050.456	554.9679	820.8736
1376056_at	300043	NA	NA	1050.334	1158.969	1628.718
1374182_at	301020	Smarcc1_p	SWI/SNF related, matrix associated, a	1049.82	1274.331	1086.385
1378266_at	292686	Fbxo46	F-box protein 46	1049.509	355.8734	441.1589
1370816_at	252917	Nr1d1	nuclear receptor subfamily 1, group D,	1049.477	332.6059	7685.031
1381590_at	81918	Pard3	par-3 (partitioning defective 3) homolo	1048.485	216.5041	73.92419
1373074_at	307210	RGD13072	similar to RIKEN cDNA 2700002I20	1048.222	4607.326	1793.049
1368206_at	170588	Acot8	acyl-CoA thioesterase 8	1047.717	3064.595	410.2504
1388821_at	313974	RGD15644	similar to Tribbles homolog 2 (predicte	1047.556	2689.699	3464.681
1368129_at	58967	Sfmbt1	Scm-like with four mbt domains 1	1046.666	1835.946	1318.116
1389073_at	306014	Reep4	receptor accessory protein 4	1046.146	1549.73	658.0109
1384832_at	293645	Ppfia1_pre	protein tyrosine phosphatase, receptor	1045.839	1615.628	522.4279
1392847_at	314028	Cbll1_pred	Casitas B-lineage lymphoma-like 1 (pr	1045.261	827.0353	2770.136
1367789_at	94172	Slc27a1	solute carrier family 27 (fatty acid trans	1044.979	1099.849	820.4286
1375847_at	363129	Nudt16_pre	nudix (nucleoside diphosphate linked r	1044.734	291.0007	693.0074
1387003_at	79428	Luzp1	leucine zipper protein 1	1044.61	1284.567	619.72
1384352_at	361878	Papd4	PAP associated domain containing 4	1044.207	1131.86	1426.199

1374884_at	287585	Ppm1d_pre	protein phosphatase 1D magnesium-d	1043.726	2822.61	6036.061
1371035_at	246299	Gtf3a	general transcription factor III A	1043.589	2774.704	355.4997
1374734_at	289138	RGD13049	similar to RIKEN cDNA 2810025M15 (	1043.094	1692.273	672.6263
1374984_at	304733	Epb4.1l5	erythrocyte protein band 4.1-like 5	1042.414	213.3143	986.062
1381016_at	306808	Ippk	inositol 1,3,4,5,6-pentakisphosphate 2-	1041.842	965.384	778.3053
1392107_at	314619	Stno_predi	strawberry notch homolog (Drosophila	1041.668	346.5199	463.4643
1371081_at	252857	Rapgef4	Rap guanine nucleotide exchange fact	1041.668	481.147	5729.206
1387721_at	29290	Adora1	adenosine A1 receptor	1040.994	798.8673	229.1637
1370979_at	84473	Ddx20	DEAD/H (Asp-Glu-Ala-Asp/His) box po	1040.489	1734.011	1558.191
1376238_at	498411	LOC49841	NA	1040.248	696.4985	316.2371
1367818_at	29309	Coq3	coenzyme Q3 homolog, methyltransfer	1040.049	1493.912	396.2305
1392508_at	361159	Thex1	three prime histone mRNA exonucleas	1039.674	1226.762	1063.543
1374258_at	293509	RGD13069	similar to hypothetical protein MGC131	1039.565	569.2703	283.8111
1374654_at	361765	Btrc	beta-transducin repeat containing	1038.396	1391.151	739.6935
1398826_s_at	245980	Nr2f6	nuclear receptor subfamily 2, group F,	1038.389	1590.842	439.4338
1377663_at	295588	Rnd3	Rho family GTPase 3	1038.267	1041.324	1424.648
1389182_at	301419	RGD13112	similar to hypothetical protein FLJ3795	1037.894	1077.186	1503.693
1369938_at	171053	Pank4	pantothenate kinase 4	1037.408	791.6978	1261.854
1374853_at	287173	RGD13056	hypothetical LOC287173	1037.22	2069.697	1439.897
1389738_at	304577	Ung	uracil-DNA glycosylase	1036.638	2928.155	536.1139
1393033_at	287924	Yars2	tyrosyl-tRNA synthetase 2 (mitochondr	1036.367	1091.527	1476.303
1370203_at	116675	Frag1	FGF receptor activating protein 1	1035.509	860.7388	1042.132
1391085_at	246186	Fgl1	fibronigen-like protein 1	1035.012	1615.49	2072.095
1376497_a_at	305845	RGD15598	similar to FLJ20859 protein (predicted)	1034.206	1223.5	346.0197
1368224_at	24795	Serpina3n	serine (or cysteine) peptidase inhibitor	1034.053	116.4262	304.5696
1379263_at	308390	Fkrp	fukutin related protein	1033.973	1303.126	1121.501
1372101_at	192270	Ppap2b	phosphatidic acid phosphatase type 2E	1033.938	185.939	1641.834
1387141_at	65208	Dpysl5	dihydropyrimidinase-like 5	1033.865	2758.103	450.6923
1390807_at	302669	Ca5b	carbonic anhydrase VB, mitochondrial	1033.386	244.0945	1027.638
1373622_at	502414	LOC50241	hypothetical protein LOC502414	1033.265	842.465	940.1511
1373115_at	363213	RGD13046	similar to 2310061I09Rik protein (pred	1032.478	1454.146	952.7457
1373288_at	308944	St5_predic	suppression of tumorigenicity 5 (predic	1032.233	337.5788	756.7481
1380525_at	310326	Arse	arylsulfatase E (chondrodysplasia pun	1031.529	3789.032	2464.497
1368307_at	156275	Ggtl3	gamma-glutamyltransferase-like 3	1030.937	2579.59	183.4677
1372084_at	362930	Ptp4a3_pre	protein tyrosine phosphatase 4a3 (pre	1030.443	1914.342	1374.264
1373049_at	501195	RGD15621	similar to D1Ertd622e protein (predicte	1029.919	2217.519	3226.706
1387114_at	170538	Prkcd	protein kinase C, delta	1029.792	1555.728	1803.232
1372040_at	287462	Camta2_pr	calmodulin binding transcription activa	1029.093	1124.278	1408.881
1369026_at	60382	Arfip1	ADP-ribosylation factor interacting prot	1028.834	1112.817	806.7517
1382051_at	360826	RGD13110	similar to J-type co-chaperone HSC20	1028.471	2536.48	610.3144
1373543_at	498683	RGD15643	similar to Sprouty homolog 3 (Spry-3) (	1028.121	938.738	1091.22
1374802_at	305882	RGD13052	similar to chromosome 14 open readin	1027.963	2551.139	1077.993
1392973_at	499153	NA	NA	1026.551	699.4248	3684.786
1380969_at	309510	RGD15646	similar to mKIAA0940 protein (predicte	1026.519	160.8121	538.5593
1383343_at	292498	LOC29249	putative pheromone receptor V2R4	1026.032	631.9347	1272.563
1382200_at	501702	RGD15656	similar to Prr6 protein (predicted)	1025.928	2333.142	1419.322
1369338_at	58946	Robo1	roundabout homolog 1 (Drosophila)	1025.218	248.1268	306.1228
1372317_at	293692	Ehd1	EH-domain containing 1	1025.172	1386.91	416.1622
1369951_at	24291	Ctrb	Chymotrypsinogen B	1024.65	58.24679	6559.136
1375091_at	363055	RGD15612	similar to KIAA1052 protein (predicted)	1024.534	549.041	554.9811
1369972_at	116589	Serpinb5	serine (or cysteine) peptidase inhibitor	1024.523	947.3517	1659.672

1397241_at	313553	RGD13068	similar to hypothetical protein MGC478	1024.462	1214.865	233.7509
1369158_at	24247	Casr	calcium-sensing receptor	1024.223	260.0272	3667.753
1389636_at	362665	LOC36266	similar to KIAA0833 protein	1023.468	1444.545	1687.233
1382446_at	362749	Atp5s	mitochondrial ATP synthase regulatory	1023.079	270.9513	1293.371
1392214_at	317213	Brwd3_pre	bromodomain and WD repeat domain	1021.573	159.8206	31.6536
1381093_at	114101	Cds2	CDP-diacylglycerol synthase (phospha	1020.452	209.4381	535.8818
1368596_at	59329	Snf1lk	SNF1-like kinase	1020.442	301.6177	2587.667
1369963_at	114113	Pafah1b3	platelet-activating factor acetylhydrolas	1020.137	3344.109	848.6842
1395019_at	307764	Usp38_pre	ubiquitin specific protease 38 (predicte	1019.415	533.1708	1572.031
1390388_at	361338	Fech_pred	ferrochelatase (predicted)	1019.332	1717.216	1144.573
1378952_at	303564	Tmem101	transmembrane protein 101	1018.881	744.8726	1015.182
1390127_at	363062	Dixdc1	DIX domain containing 1	1018.655	657.7433	1675.969
1396733_at	309142	Mtl5	metallothionein-like 5, testis-specific (t	1018.636	426.2357	157.9052
1398363_at	500226	RGD15614	similar to D3Mm3e (predicted)	1018.183	2900.712	1480.852
1393103_at	287054	RGD13114	similar to cDNA sequence BC024814 (	1016.619	1811.512	1685.144
1393794_at	295654	RGD15651	similar to tetratricopeptide repeat doma	1015.762	776.1718	1719.876
1390811_at	366515	LOC36651	similar to Wdr8 protein	1015.728	868.1319	864.8584
1388638_at	299638	Sirt6	sirtuin 6 (silent mating type information	1014.728	1366.856	668.6122
1385395_at	252853	Stx17	syntaxin 17	1014.463	717.115	640.4164
1375819_at	298712	RGD13072	similar to RIKEN cDNA 1200011I18	1014.394	486.0311	758.6849
1378131_at	363115	RGD15607	similar to solute carrier family 9 (sodiur	1014.228	321.0307	773.9562
1373693_at	287805	Gprc5c	G protein-coupled receptor, family C, g	1014.031	1893.086	935.9742
1384024_at	315210	Brd1_predi	bromodomain containing 1 (predicted)	1013.992	1288.429	4237.288
1369703_at	29452	Epas1	endothelial PAS domain protein 1	1013.778	397.8618	86.41311
1374145_at	292898	RGD13098	similar to RIKEN cDNA 1110061L23	1013.316	1409.746	333.5274
1367650_at	94174	Lcn7	lipocalin 7	1013.175	46.30025	76.49782
1391602_at	305470	Drg1	developmentally regulated GTP bindin	1013.118	215.6103	423.5195
1383311_at	310677	RGD13081	similar to zinc finger protein Cezanne;	1012.746	1803.292	2170.307
1388411_at	361988	RGD13045	similar to expressed sequence C77668	1012.154	680.0098	301.8939
1381056_at	293619	Pkp3_pred	plakophilin 3 (predicted)	1011.984	115.0926	1135.033
1393057_at	308320	LOC30832	similar to 5730403M16Rik protein	1011.497	839.2861	1133.572
1374570_at	311821	Agpat2_pre	1-acylglycerol-3-phosphate O-acyltran	1010.79	37.50586	1185.62
1378724_at	361794	Nfkbil1	nuclear factor of kappa light polypeptic	1009.981	509.2885	705.5453
1375420_at	311209	Tp53i11_pr	tumor protein p53 inducible protein 11	1009.921	1513.887	984.9624
1372995_at	292658	Prkd2	protein kinase D2	1008.356	458.6478	869.8265
1387910_at	59317	Epb4.1l1	erythrocyte protein band 4.1-like 1	1008.32	1045.422	1121.217
1390862_at	308998	Zfp629	zinc finger protein 629	1008.296	946.0223	725.0667
1374028_at	500974	LOC50097	similar to CDNA sequence BC024479	1008.211	627.5699	2466.462
1390288_at	364993	Pskh1_pre	protein serine kinase H1 (predicted)	1007.964	274.3934	596.5614
1369661_at	25751	Dnm2	dynamin 2	1007.797	733.8326	156.4737
1390434_at	246756	Tradd	TNFRSF1A-associated via death doma	1007.386	6.489444	411.2938
1372403_at	315732	RGD15600	similar to Nuclear membrane binding p	1006.936	283.5389	1010.351
1370885_at	252929	Ctsz	cathepsin Z	1006.567	159.0208	1251.303
1375024_at	499731	RGD15596	similar to RIKEN cDNA 5230400J09 (p	1006.078	943.3513	1180.413
1382711_at	498160	MGC11636	zinc finger protein 36 (KOX 18)	1006.013	3378.705	319.6213
1375546_at	314642	Fzr1_predi	fizzy/cell division cycle 20 related 1 (Dr	1005.365	2118.164	542.2622
1383555_at	360799	RGD15602	similar to RIKEN cDNA 4930579G22 (	1004.19	1161.727	531.5739
1383428_at	315947	RGD15648	similar to Thioredoxin domain containir	1003.984	586.5307	1737.744
1377080_at	246143	Nradd	neurotrophin receptor associated deatl	1003.78	1352.168	822.8237
1367936_at	29398	Stk10	serine/threonine kinase 10	1003.385	1174.336	408.012
1374101_at	360968	Tbc1d10a	TBC1 domain family, member 10a	1002.499	302.2841	560.8416

1378578_at	501559	RGD15621	similar to chromosome X open reading	1001.118	2068.022	1487.11
1391506_at	310352	Phf17_pre	PHD finger protein 17 (predicted)	999.584	789.2179	1244.335
1386566_at	500652	NA	NA	999.2329	866.8736	715.596
1388165_at	286903	PORF-2	preoptic regulatory factor-2	998.7097	424.9685	1104.323
1387090_a_at	29524	Limk2	LIM motif-containing protein kinase 2	998.3769	1638.581	1142.738
1390357_at	308434	Zfp574	zinc finger protein 574	998.0717	992.5549	1110.987
1376964_at	302661	RGD15622	similar to Ofd1 protein (predicted)	997.7012	1181.129	1094.745
1390791_at	360973	Gas2l1_pre	growth arrest-specific 2 like 1 (predicte	997.2207	543.2919	948.2407
1377493_at	300852	Mto1_pred	mitochondrial translation optimization 1	997.0842	842.0288	1117.639
1369160_a_at	117955	Slc4a7	solute carrier family 4, sodium bicarbo	995.9103	317.5455	370.1888
1383601_at	308720	Dufd1_pre	DUF729 domain containing 1 (predicte	995.1866	4112.807	2175.85
1396235_at	313242	Wdr32_pre	WD repeat domain 32 (predicted)	994.9384	806.4227	1235.317
1378642_at	311502	RGD1307C	similar to uncharacterized hypothalamu	994.6978	2085.443	2286.61
1375276_at	317374	Timm17b_	translocase of inner mitochondrial mer	994.5478	1650.088	506.9957
1368341_at	29240	Polb	polymerase (DNA directed), beta	994.4265	2114.63	761.1622
1374621_at	361420	LOC36142	similar to TAFI95	994.0756	616.3535	451.7867
1380684_at	500086	LOC50008	hypothetical protein LOC500086	993.4022	1122.112	814.3098
1389185_at	24183	Ak1	adenylate kinase 1	992.7475	659.1848	546.825
1372436_at	296480	Tpd52l2	tumor protein D52-like 2	992.4429	2488.302	1387.935
1384768_at	499095	Zfp94	zinc finger protein 94	992.3956	617.2898	576.147
1369131_at	25549	Slc18a2	solute carrier family 18 (vesicular mon	992.3116	509.329	272.3515
1383976_at	362366	Fkbp14	FK506 binding protein 14	992.3072	138.5971	1452.507
1392550_at	500666	RGD1564C	similar to dapper 1 (predicted)	992.1357	1277.734	1823.291
1369067_at	58853	Nr4a3	nuclear receptor subfamily 4, group A,	991.8685	240.2607	15154.72
1372517_at	309651	Ppil1	peptidylprolyl isomerase (cyclophilin)-li	991.4957	1935.492	667.588
1376807_at	360720	Rabl3_pre	RAB, member of RAS oncogene family	991.2986	1680.706	650.7918
1372139_at	309109	Tmem80	transmembrane protein 80	990.8936	898.4868	1123.052
1373478_at	83708	Mybph	myosin binding protein H	990.6941	592.9028	102.2232
1382400_at	313566	Rlf_predict	rearranged L-myc fusion sequence (pr	990.3598	805.844	2730.383
1373890_at	296147	Chchd5_pr	coiled-coil-helix-coiled-coil-helix domai	990.2741	3064.188	606.8999
1370549_at	64516	Vps45	vacuolar protein sorting 45 (yeast)	990.093	3196.305	692.3603
1373471_at	365022	Rnf166	ring finger protein 166	989.1738	792.0382	1881.151
1370845_at	64467	Entpd2	ectonucleoside triphosphate diphosph	989.0998	161.8567	126.6389
1372447_at	79114	Fgfr1	Fibroblast growth factor receptor 1	989.086	984.662	3803.581
1384048_at	361607	RGD13107	similar to EMSY protein (predicted)	988.8657	1354.902	606.9862
1383138_at	365396	Ssh3	slingshot homolog 3 (Drosophila)	988.5299	3229.545	592.656
1391022_at	305078	Lamb3	laminin, beta 3	988.5252	1135.455	633.871
1393220_at	315949	Armc8_pre	armadillo repeat containing 8 (predicte	988.5164	854.875	3854.301
1367796_at	81519	Mgat1	mannoside acetylglucosaminyltransfer	988.1394	826.6776	726.9557
1397551_at	362274	Ddx27	DEAD (Asp-Glu-Ala-Asp) box polypept	987.8792	2080.1	1009.326
1372847_at	362323	Acn9	ACN9 homolog (S. cerevisiae)	987.8562	1135.903	1052.433
1373838_at	60670	Fut4	fucosyltransferase 4	987.7148	1132.291	1043.206
1381924_at	292078	RGD15615	similar to hypothetical protein FLJ3160	987.5837	348.4632	637.3113
1370413_at	252924	Psg4	pregnancy specific beta-1-glycoprotein	986.0413	1665.252	1981.149
1391101_at	307016	Arhgap12_	Rho GTPase activating protein 12 (pre	985.2415	951.3331	1437.688
1370535_at	116668	Myt1l	myelin transcription factor 1-like	984.3024	1447.421	1935.198
1370990_at	245962	Cd48	CD48 antigen	984.2019	1567.238	816.501
1378606_at	291694	RGD13104	similar to hypothetical protein MGC370	984.1269	2567.962	6609.267
1383448_at	305896	Isgf3g	interferon dependent positive acting tra	983.7426	656.7301	2099.881
1368087_a_at	171070	Ptpn21	protein tyrosine phosphatase, non-rece	982.6158	126.6998	627.8567
1387195_at	114093	St14	suppression of tumorigenicity 14 (colo	981.9709	3392.288	1188.056

1392036_at	306350	Rent1_prec	regulator of nonsense transcripts 1 (pr	981.9299	450.7807	633.054
1387441_at	29553	Kcnk3	potassium channel, subfamily K, meml	981.8025	436.2932	2648.872
1398375_at	298763	Mta3_pred	metastasis associated 3 (predicted)	981.7605	3130.201	556.2332
1375415_at	366468	LOC36646	NA	981.0586	2378.506	661.8436
1372871_at	316530	RGD73517	hypothetical protein MGC:72616	980.9881	380.6814	959.6752
1380167_at	50622	Slc23a2	solute carrier family 23 (nucleobase tra	979.7759	451.1378	222.7835
1369304_at	29498	Pts	6-pyruvoyl-tetrahydropterin synthase	979.2646	810.9812	503.3681
1373694_at	287432	Wdr79	WD repeat domain 79	979.0648	1032.953	667.0023
1382934_at	315435	Panx1	Pannexin 1	978.829	624.2349	529.3002
1392763_at	406165	Agpat1	1-acylglycerol-3-phosphate O-acyltran	978.416	830.4182	536.6204
1384315_at	296849	Pdk3_map	pyruvate dehydrogenase kinase, isoer	977.0392	2081.119	367.3215
1370248_at	63847	Fxyd6	FXYD domain-containing ion transport	976.9829	5913.461	7697.913
1373635_at	499090	RGD15651	similar to Myb protein P42POP (predic	976.588	534.3749	628.8841
1370478_at	192253	Myr8	myosin heavy chain Myr 8	975.9335	1338.604	81.7433
1381448_at	293128	RGD13051	similar to CG6796-PA	975.7008	43.88026	1431.93
1391537_at	360899	RGD15654	similar to SERTA domain containing 4	974.6906	2031.122	1069.357
1399035_at	300088	Polr3h_pre	polymerase (RNA) III (DNA directed) p	974.3928	2224.239	565.4902
1379104_at	499909	NA	NA	973.5902	948.2538	946.241
1393383_at	360500	Arhgdig_pr	Rho GDP dissociation inhibitor (GDI) g	973.2523	5416.282	322.1771
1384084_at	361719	RGD13081	LOC361719 (predicted)	972.9356	639.2744	482.1548
1370208_at	58851	Nr1h2	nuclear receptor subfamily 1, group H,	972.5194	1387.489	375.533
1374841_at	499724	RGD1560C	similar to RIKEN cDNA 2410004B18 (p	972.4408	1074.161	1814.93
1389641_at	362088	Snapc4_pr	small nuclear RNA activating complex,	971.5536	283.5802	432.3876
1388833_at	298098	Pole3	polymerase (DNA directed), epsilon 3	971.1103	2330.451	440.6162
1391489_at	303090	Irgm	immunity-related GTPase family, M	970.4924	836.8995	1185.87
1390403_at	288031	RGD13047	similar to CG8312-PA	970.3928	631.1632	3885.815
1380800_at	309407	Trpm3_pre	transient receptor potential cation char	970.3652	180.181	562.0334
1388639_at	363662	RGD15607	similar to Breast carcinoma amplified s	970.3604	718.2592	756.5076
1378492_at	306183	Farp1_prec	FERM, RhoGEF (Arhgef) and pleckstri	970.2931	204.6536	1138.678
1373810_at	362039	Pla2g12a_	phospholipase A2, group XIIA (predicte	969.9292	406.891	577.9861
1378490_at	304419	Auts2_prec	autism susceptibility candidate 2 (pred	969.1387	742.4176	376.083
1373394_at	303790	RGD13106	similar to RIKEN cDNA 4122402O22	969.1275	2445.462	878.9279
1396830_at	499979	RGD15621	RGD1562135 (predicted)	968.6867	209.9025	249.9191
1368074_at	114860	Gale	galactose-4-epimerase, UDP	967.6059	85.04704	133.5163
1389348_at	311670	Cstf1	cleavage stimulation factor, 3' pre-RNA	967.5719	1397.678	507.3938
1376592_at	293829	Mcee_pred	methylmalonyl CoA epimerase (predic	967.2631	3289.739	1970.078
1380309_at	287990	RGD13094	similar to mKIAA0804 protein (predicte	966.4262	1615.528	1006.308
1369543_s_at	192210	Gs3	putative regulation protein GS3	966.36	1187.329	1591.371
1398431_at	297814	Car8	carbonic anhydrase 8	966.1513	15455.9	1075.935
1379284_at	315579	RGD13103	similar to RIKEN cDNA 2810457I06 (p	966.1365	179.5761	877.1553
1395065_at	361879	Wdr41_pre	WD repeat domain 41 (predicted)	965.599	1763.229	909.5594
1386912_at	29569	Pcolce	procollagen C-endopeptidase enhance	965.3835	34.64107	577.6665
1369686_at	83825	Dcamkl1	double cortin and calcium/calmodulin-c	965.2803	1595.051	1018.109
1395974_at	303312	RGD13113	similar to hypothetical protein (predicte	964.9548	820.7091	585.9473
1371322_at	117036	Lamc1	laminin, gamma 1	964.9333	1057.404	1812.494
1377461_at	499936	NA	NA	963.9157	639.0074	977.3566
1389386_at	363169	RGD15607	similar to hypothetical protein DKFZp3	963.2628	688.1973	1321.149
1398370_at	25367	Adarb1	adenosine deaminase, RNA-specific, E	963.2168	2069.088	1197.25
1384344_at	295672	Fkbp7_pre	FK506 binding protein 7 (predicted)	962.9837	237.1479	843.1531
1379685_at	245924	Sfrs15	splicing factor, arginine/serine-rich 15	962.8354	1089.023	1206.544
1386990_at	117278	Ebp	phenylalkylamine Ca2+ antagonist (em	962.821	3254.881	482.1647

1383906_at	316326	LOC31632	similar to lung inducible neuralized-rela	962.7973	90.56989	1380.508
1387499_a_at	64013	Pdcl	phosducin-like	962.6421	2290.171	1352.713
1383475_at	24666	Ppm1a	protein phosphatase 1A, magnesium c	962.4425	750.6033	3079.508
1378611_at	498289	LOC49828	NA	962.1142	649.2651	1250.536
1384572_at	362360	RGD15642	similar to mKIAA0704 protein (predicte	961.5548	419.5229	788.4004
1391823_at	362886	Rassf3_pre	Ras association (RalGDS/AF-6) doma	961.4839	77.25743	283.1076
1395667_at	317628	RGD15659	similar to apurinic/apyrimidinic endonu	961.1719	818.5301	605.5518
1376221_at	292724	RGD13093	similar to DNA segment, Chr 7, ERATO	960.8846	498.5797	811.1536
1397600_at	299738	Vezt	vezatin, adherens junctions transmeml	959.553	2028.558	245.0192
1373240_at	313689	Dhrs3	dehydrogenase/reductase (SDR family	959.5352	153.7313	68.27845
1394802_at	59267	Syt7	synaptotagmin VII	959.495	696.892	359.2747
1370510_a_at	29657	Arntl	aryl hydrocarbon receptor nuclear tran	959.2457	902.0445	1655.349
1390793_at	294774	RGD15640	similar to osmosis responsive factor (p	958.9137	1462.716	914.7611
1375447_at	300077	Gtpbp1_pre	GTP binding protein 1 (predicted)	958.8458	765.7696	783.1043
1369839_at	60668	Amph1	amphiphysin 1	958.6026	1793.824	572.5835
1390662_at	500084	LOC50008	Ab2-427	958.5383	2130.419	2154.087
1393564_at	303963	RGD15632	similar to Ubiquitin ligase protein DZIP	958.2378	1395.426	404.2466
1388399_at	313819	Mast2_pre	microtubule associated serine/threonir	958.2367	985.0977	1236.557
1368741_at	117512	C9	complement component 9	958.0471	106.5163	143.8968
1391935_at	297481	LOC29748	NA	957.5953	2397.581	1881.307
1374199_at	687681	LOC68768	NA	957.5585	1936.248	2122.969
1398378_at	297029	Gstk1	glutathione S-transferase kappa 1	957.1087	1424.911	765.782
1388395_at	289388	G0s2	G0/G1 switch gene 2	956.4462	1824.894	506.4415
1376348_at	310025	Ptcd2_pred	pentatricopeptide repeat domain 2 (pre	955.4452	744.8789	729.9184
1385035_at	360763	Usp12_pre	ubiquitin specific protease 12 (predicte	955.2391	1146.035	277.4198
1381995_at	307540	Brunol4_pr	bruno-like 4, RNA binding protein (Dro	955.1587	3742.284	2143.289
1374993_at	287148	Rpusd1_pr	RNA pseudouridylate synthase domair	955.1175	734.3953	289.9371
1369655_at	65052	Pik3c3	phosphoinositide-3-kinase, class 3	954.7863	1777.64	941.8615
1372625_at	499076	LOC49907	NA	954.3869	956.1809	1452.256
1374042_at	299521	RGD13104	similar to hypothetical protein FLJ2345	953.0759	1255.944	1588.745
1391534_at	498728	Elovl2_pre	elongation of very long chain fatty acid	952.9823	4365.23	1070.679
1375278_at	361970	Trim2	tripartite motif protein 2	952.9717	2244.44	1735.1
1379150_at	303073	Cyfip2_pre	cytoplasmic FMR1 interacting protein 2	952.9109	50.24587	1029.179
1375660_at	303135	Sept8_pred	septin 8 (predicted)	952.0285	1841.097	315.876
1373205_at	361542	U2af1l4	U2 small nuclear RNA auxiliary factor	951.8168	3044.068	1414.474
1370953_at	360721	LOC36072	NA	951.6869	389.6613	238.9173
1389862_at	362955	RGD13053	similar to hypothetical protein MGC373	951.0122	954.3294	335.7828
1372491_at	313757	RGD15655	similar to Ski protein (predicted)	950.8956	1062.736	1975.643
1392227_at	298018	LOC29801	NA	950.0007	1466.554	561.4984
1367738_at	29402	Unc119	UNC-119 homolog (C. elegans)	949.9713	1702.372	647.4434
1373232_at	311772	RGD15660	similar to nidogen 2 (predicted)	949.8077	146.634	502.0526
1392435_at	50565	Hspb7	heat shock 27kD protein family, memb	949.6149	306.7003	651.2025
1368398_at	29717	Cacna1g	calcium channel, voltage-dependent, T	949.5159	1270.244	454.647
1374313_at	290640	Bpy2ip1_pr	BPY2 interacting protein 1 (predicted)	949.4117	889.4723	476.1863
1377204_at	305540	Slc1a4	solute carrier family 1 (glutamate/neutr	949.3759	181.4331	490.7989
1388703_at	300519	Esam	endothelial cell adhesion molecule	949.0788	2444.823	219.8984
1388274_at	311807	Bmyc_map	brain expressed myelocytomatosis onc	949.0252	943.5368	886.3082
1373752_at	305464	Depdc5_pr	DEP domain containing 5 (predicted)	948.8423	715.4169	635.48
1380499_at	304061	Brwd1_pre	bromodomain and WD repeat domain	948.7674	1514.879	1674.435
1383271_at	314799	Ccdc59_pr	coiled-coil domain containing 59 (predi	948.28	1967.051	3261.384
1372960_a_at	64134	Xylt2	xylosyltransferase II	947.3922	1547.402	652.5523

1390249_at	315702	RGD13054	similar to DKFZP434H132 protein	947.3808	400.9878	714.2247
1372630_at	361381	Rad23a	RAD23a homolog (S. cerevisiae)	947.3591	786.1288	1450.873
1371913_at	116487	Tgfbi	transforming growth factor, beta induce	947.3168	4097.408	4991.33
1370440_at	246280	Slc15a4	solute carrier family 15, member 4	947.2913	1576.771	915.4327
1374871_at	246307	Asrgl1	asparaginase like 1	947.1725	1188.307	1132.594
1394028_at	63995	Dusp10_pr	dual specificity phosphatase 10 (predic	946.9295	412.4464	1828.892
1391830_at	362988	Cpne8_pre	copine VIII (predicted)	946.9142	348.0235	767.263
1373230_at	361068	Nudt18	nudix (nucleoside diphosphate linked r	946.2927	351.5009	803.479
1372541_at	361728	MGC73003	Unknown (protein for MGC:73003)	945.6569	3319.22	773.1716
1382115_at	309486	RGD13051	similar to RIKEN cDNA 4930521E07 (p	945.1042	1173.42	944.0584
1383793_at	312654	RGD13119	hypothetical LOC312654 (predicted)	945.0878	475.8369	2039.167
1377848_at	289468	RGD13052	similar to RIKEN cDNA 3830405G04	944.6012	1212.842	410.9185
1391684_at	363206	Tmem14a_	transmembrane protein 14A (predicted	944.5366	4266.303	2373.501
1383981_at	305025	Trp53bp2_	transformation related protein 53 bindi	944.2818	502.8451	3190.972
1390739_at	315766	LOC31576	NA	943.6097	300.8926	392.705
1386988_at	83632	Deaf1	deformed epidermal autoregulatory fac	943.4614	1063.04	735.6513
1373688_at	498145	LOC49814	similar to RIKEN cDNA 2810453I06	942.8922	980.8851	1331.015
1374982_at	362944	Ppp1r16a_	protein phosphatase 1, regulatory (inhi	942.446	314.5206	474.7854
1370324_at	24218	Atp7b	ATPase, Cu++ transporting, beta polyp	942.4189	399.2257	601.2262
1391036_at	404781	Iqsec3	NA	942.2702	848.9321	742.9283
1391306_at	116684	Oplah	5-oxoprolinase (ATP-hydrolysing)	941.6343	1594.204	1013.901
1374415_at	361640	Polr3e_pre	polymerase (RNA) III (DNA directed) p	941.3158	1425.362	2092.886
1384110_at	366608	RGD15617	similar to mKIAA0716 protein (predicte	940.8472	792.6431	4623.169
1388037_at	29599	Atp2b3	ATPase, Ca++ transporting, plasma m	940.7325	3079.362	309.263
1372140_at	361454	RGD13103	similar to chromosome 6 open reading	940.3596	609.1722	818.0802
1385024_at	361760	Cutc_predi	cutC copper transporter homolog (E.co	938.4083	2406.845	1476.842
1371935_at	289046	Tmem9_pr	transmembrane protein 9 (predicted)	938.3324	5058.122	1037.653
1380478_at	501017	NA	NA	937.591	438.9651	173.1222
1382057_at	499378	NA	NA	937.3652	491.0574	1337.113
1389167_at	296648	Mapkap1	mitogen-activated protein kinase asso	937.1222	1033.295	480.0869
1373782_a_at	362100	Endog	endonuclease G	937.0043	702.4357	432.1788
1372939_at	287199	Nudcd2	NudC domain containing 2	936.2864	2612.927	1532.732
1377119_at	307498	Pura_predi	purine rich element binding protein A (	936.2224	394.2111	585.8673
1389801_at	25114	Fgfr4	fibroblast growth factor receptor 4	935.7163	2660.705	661.648
1395132_at	25600	Utrn	utrophin	934.9325	765.1662	463.6321
1379975_at	25530	Rab12	RAB12, member RAS oncogene family	934.6496	240.4575	626.1127
1376211_a_at	305792	Kctd6_prec	potassium channel tetramerisation dor	934.6309	556.7479	1582.174
1388212_a_at	294228	RT1-S3	RT1 class Ib, locus S3	933.9358	2751.92	2893.637
1375896_at	501146	RGD15594	similar to polyploidy associated proteir	933.877	1476.184	1647.878
1388547_at	304407	Cldn4	claudin 4	933.2921	55.0838	3101.873
1374294_at	293513	NA	NA	931.9648	922.0099	1049.105
1389264_at	362957	RGD13095	similar to RIKEN cDNA C730048E16	931.6385	1048.145	608.2195
1379425_at	313563	RGD13098	similar to RIKEN cDNA 3110037I16 (p	930.6748	1238.272	943.2704
1370837_at	245917	Sycn	syncollin	929.761	95.47037	2131.678
1377267_at	317232	Magee1_pr	melanoma antigen, family E, 1 (predict	929.7548	2809.956	3467.33
1374120_at	315054	RGD13099	similar to KIAA1126 protein (predicted)	929.3632	1174.251	735.4256
1367949_at	29237	Penk-rs	preproenkephalin, related sequence	929.3553	29.64245	4643.46
1377776_at	362137	Wdsub1	WD repeat, SAM and U-box domain co	929.0756	385.5396	1082.622
1378679_at	304150	Usp25_pre	ubiquitin specific protease 25 (predicte	929.0402	1026.387	13976.85
1378056_at	291137	Gmnn_pre	geminin (predicted)	928.8493	5337.816	676.2286
1379406_at	290235	RGD13083	similar to chromosome 14 open readin	928.202	593.486	504.028

1387942_at	266687	Slc35e4	solute carrier family 35, member E4	928.0761	885.7411	701.4129
1370548_at	170566	Slc16a10	solute carrier family 16 (monocarboxyl	927.9667	644.3531	4239.552
1377522_a_at	314639	RGD13047	similar to KIAA1086 protein (predicted)	927.6952	1429.204	1420.292
1379914_at	313994	Tcfcp2l2	transcription factor CP2-like 2	927.6194	2567.156	7161.343
1372609_at	363193	Ppp2r5d	protein phosphatase 2, regulatory subu	927.3746	1122.787	512.0956
1393899_at	192357	Gmpr2	guanosine monophosphate reductase	927.0762	395.4801	328.753
1368410_at	24561	Mpg	N-methylpurine-DNA glycosylase	927.0351	1055.675	287.1355
1390733_at	502205	NA	NA	926.3929	106.7011	941.8141
1377096_at	315763	Mtfmt	mitochondrial methionyl-tRNA formyltra	926.1375	1367.654	1737.694
1393150_at	114845	Klf3_mapp	Kruppel-like factor 3 (basic) (mapped)	925.9128	476.6282	893.57
1387098_at	83581	Rpo1-4	RNA polymerase 1-4	924.7746	643.8629	364.2935
1376701_a_at	297627	MGC94282	similar to 5930416I19Rik protein	924.4103	1802.212	1302.269
1391530_a_at	289934	RGD13110	similar to hypothetical protein FLJ2060	923.6761	2938.739	560.2388
1371953_at	29157	Ccng2_pre	cyclin G2 (predicted)	923.4692	3279.609	5900.774
1383018_at	298698	Angptl6_pr	angiopoietin-like 6 (predicted)	923.2862	669.2299	630.9216
1391297_at	314458	Rcor1_pre	REST corepressor 1 (predicted)	923.094	486.1175	459.6226
1389594_at	499913	LOC49991	similar to Protein C20orf22 homolog	922.8121	1024.998	478.323
1391855_at	690085	LOC69008	NA	922.5287	765.5961	318.6178
1371951_at	63839	Fhl2	four and a half LIM domains 2	921.5108	458.0092	1700.77
1388969_at	498962	LOC49896	NA	920.9668	786.9775	690.7458
1387951_at	64036	Daf1	decay accelarating factor 1	920.9561	2381.924	1516.976
1375859_a_at	308488	RGD15606	similar to zinc finger protein 565 (predi	920.6323	213.0887	789.1069
1370064_at	81751	Psen2	presenilin 2	919.474	346.9309	468.5582
1370543_at	26295	Ogt	O-linked N-acetylglucosamine (GlcNAc	919.3824	1398.392	680.3586
1378359_at	298316	RGD15639	similar to NF-kappa-B-repressing facto	919.0733	679.8027	1611.362
1368262_at	59265	Phlpp	PH domain and leucine rich repeat pro	918.9892	518.4414	840.8154
1370537_at	25019	Xrcc6	X-ray repair complementing defective r	918.8026	1700.087	1101.933
1388385_at	286925	Cryba2	crystallin, beta A2	916.8612	570.3073	4571.811
1395007_at	307839	Zfp612_pre	zinc finger protein 612 (predicted)	916.3736	514.7839	2234.733
1378180_at	290741	LOC29074	similar to 6030466N05Rik protein	916.2306	1584.506	601.5356
1374922_at	362614	Atpbd1b_p	ATP binding domain 1 family, member	916.1775	499.5089	536.7522
1382103_at	363109	Pgm3_pre	phosphoglucomutase 3 (predicted)	915.9458	319.6197	466.2324
1378639_at	316108	Rnut1	RNA, U transporter 1	915.6721	610.3689	589.3324
1368910_at	54705	Ppm2c	protein phosphatase 2C, magnesium c	914.948	3002.219	2000.336
1376446_at	311430	MGC93707	similar to RIKEN cDNA D430028G21	914.4739	592.1255	530.22
1378900_at	303539	Gcn5l2_pre	GCN5 general control of amino acid sy	914.3882	1072.68	276.8965
1370638_at	361833	Ank3	ankyrin 3, epithelial	914.023	1932.38	1179.822
1378049_at	297879	RGD13064	similar to Cas-associated zinc finger pr	913.8638	2200.304	3208.046
1374082_at	298748	RGD13115	similar to PRO1853 homolog	913.7202	1495.053	1012.428
1398399_at	303180	RGD13045	similar to RIKEN cDNA 2310033P09	913.6155	1573.616	1950.789
1383742_at	310815	Snx7	sorting nexin 7	913.4594	766.395	939.6494
1395273_at	362013	Ahcyl1_pre	S-adenosylhomocysteine hydrolase-lik	912.8339	2736.963	521.9362
1379556_a_at	291211	Ggps1	geranylgeranyl diphosphate synthase	912.678	1348.022	1061.504
1390338_at	361646	LOC36164	NA	912.1879	1145.174	929.7529
1388954_at	311257	RGD13067	similar to hypothetical protein MGC254	911.5757	654.9623	476.0475
1388791_at	316426	RGD13099	similar to 2810022L02Rik protein	911.473	1880.855	6938.971
1373863_at	301363	Map4k4_pr	mitogen-activated protein kinase kinas	911.3426	1036.596	527.3632
1383283_at	317576	Ocrl_mapp	oculocerebrorenal syndrome of Lowe (	910.7193	2630.549	7870.849
1381957_at	291577	RGD15608	similar to chondroitin sulfate synthase	910.7065	137.1152	775.2074
1375621_at	261737	Sfxn5	sideroflexin 5	910.3205	179.9659	174.9562
1390888_a_at	300989	Hemk1_pre	HemK methyltransferase family memb	910.2833	545.1529	535.1995

1387429_at	25238	Adrbk1	adrenergic receptor kinase, beta 1	910.2618	1137.164	385.2162
1389991_at	287836	Fbf1_predi	Fas (TNFRSF6) binding factor 1 (predi	910.2057	1108.66	516.9572
1367649_at	170673	Palm	paralemmin	909.9569	833.1013	520.3366
1388102_at	192227	Ltb4dh	leukotriene B4 12-hydroxydehydrogena	909.2365	304.5947	2214.753
1368965_at	80878	Slc16a3	solute carrier family 16 (monocarboxyl	908.8831	255.9026	148.1722
1381246_at	300117	Phf21b_pre	PHD finger protein 21B (predicted)	908.8218	567.8464	1871.025
1372597_at	301250	Mrpl14_pre	mitochondrial ribosomal protein L14 (p	908.5467	3544.474	294.963
1379987_at	298104	RGD13045	similar to RIKEN cDNA 6330416G13 g	908.3652	515.0766	898.1653
1370583_s_at	24646	Abcb1	ATP-binding cassette, sub-family B (M	907.9149	1003.904	48.26613
1374005_at	316008	Ccdc51	coiled-coil domain containing 51	907.8997	669.0489	678.227
1378881_at	117105	Kcnb2	potassium voltage gated channel, Sha	907.5277	244.3254	713.2277
1374565_at	360161	Nek6	NIMA (never in mitosis gene a)-relatec	906.6485	446.1927	555.3651
1387938_at	140720	Baalc	brain and acute leukemia, cytoplasmic	906.6469	738.3684	418.908
1378793_at	363185	Foxp4_pre	forkhead box P4 (predicted)	905.2514	384.9079	743.0864
1388686_at	266766	Dscr1	Down syndrome critical region homolo	905.1118	4425.409	453.7053
1369946_at	79227	Pdrp	PDRP protein	905.1095	2173.998	321.499
1376692_at	362342	Hipk2_prec	homeodomain interacting protein kinas	904.8076	353.5248	353.16
1368079_at	116551	Pdk1	pyruvate dehydrogenase kinase, isoer	904.8022	1547.548	336.2916
1368311_at	25332	Mgmt	O-6-methylguanine-DNA methyltransfe	904.6252	1085.562	830.3401
1383634_at	300446	Zfp653_pre	zinc finger protein 653 (predicted)	904.4476	697.0398	1070.229
1395214_at	497962	RGD15620	RGD1562012 (predicted)	904.3208	558.0545	736.3034
1372602_at	305234	RGD13118	similar to genethonin 1	904.0977	268.6374	359.2499
1391163_at	293063	NA	NA	903.3345	410.7574	780.8134
1374944_at	313548	Zfp691_pre	zinc finger protein 691 (predicted)	903.1935	378.6822	382.2999
1369717_at	63887	Nmu	neuromedin U	903.1014	91.15472	5165.886
1370398_at	192225	Spata7	spermatogenesis associated 7	902.7459	843.8549	2462.582
1395311_at	170818	Icmt	isoprenylcysteine carboxyl methyltrans	901.2703	367.2718	278.8473
1372968_at	300147	Sbf1_predi	SET binding factor 1 (predicted)	901.0532	1076.831	279.0061
1371832_at	300837	Leo1	Leo1, Paf1/RNA polymerase II comple	901.0314	450.0246	4579.127
1392856_at	502503	RGD15651	similar to small EDRK-rich factor 1 (pre	900.9465	2606.03	487.0502
1372880_at	313666	Zbtb17	zinc finger and BTB domain containing	900.3489	834.1431	971.4357
1391840_at	316238	RGD13106	similar to RIKEN cDNA 1700027N10 (p	899.8145	5104.331	2671.623
1377463_at	308757	Man2a2_pr	mannosidase 2, alpha 2 (predicted)	899.6045	892.0396	2097.06
1373721_at	307491	Zmat2	zinc finger, matrin type 2	898.8415	1430.046	1222.949
1376894_at	317630	Pldn	pallidin	898.8127	1359.975	1843.099
1383953_at	301382	Ercc5_map	excision repair cross-complementing r	898.5129	444.6987	849.2248
1374668_at	305479	Sf3a1_prec	splicing factor 3a, subunit 1 (predicted)	898.0409	1131.456	448.7483
1386097_at	316775	Ankrd12_p	ankyrin repeat domain 12 (predicted)	897.6855	326.4723	1132.729
1390284_at	312677	RGD13107	similar to RIKEN cDNA 2700091N06 (p	897.6555	1521.961	749.7099
1370526_at	83577	Itgae	integrin, alpha E, epithelial-associated	897.4523	1044.553	617.8472
1395982_at	296634	RGD13049	similar to mKIAA0023 protein (predicte	897.0568	579.1707	369.5603
1387793_at	59114	Slc9a3r1	solute carrier family 9 (sodium/hydroge	896.7806	3032.847	933.7808
1377687_at	367838	RGD15617	similar to cDNA sequence AK129302 (	896.0751	2814.375	1461.895
1395503_at	361641	RGD13079	similar to RIKEN cDNA 3230401I01 (p	895.6849	402.1897	85.46933
1387406_at	246332	Uhmk1	U2AF homology motif (UHM) kinase 1	895.5633	723.7328	361.9272
1373746_at	312225	RGD13066	similar to HSPC049 protein (predicted)	895.3316	686.0517	523.9816
1383994_at	25731	Syt3	synaptotagmin III	895.3167	568.4483	563.5368
1379565_at	362339	Creb3l2	cAMP responsive element binding prot	895.2734	316.0806	334.6491
1372608_at	313707	LOC31370	NA	895.2167	771.1662	712.2795
1374114_at	309453	Fbxl15_pre	F-box and leucine-rich repeat protein 1	895.2137	2232.216	820.0301
1377792_at	291445	Megf10	Megf10 protein	894.5673	120.8802	246.3795

1377933_at	314637	Slc39a3	solute carrier family 39 (zinc transporte	894.5575	820.6181	342.7685
1389148_at	317191	RGD15497	similar to RIKEN cDNA 9430083G14	894.3581	2325.847	1280.866
1371703_at	191572	Ahnak	AHNAK nucleoprotein (desmoyokin)	893.7435	213.7404	1061.232
1398352_at	362827	Pias4	protein inhibitor of activated STAT, 4	893.4902	544.0401	274.2011
1367742_at	25756	Cpt1b	carnitine palmitoyltransferase 1b, musc	893.162	1334.731	1583.856
1368840_at	171411	Lr8	LR8 protein	892.5991	454.7516	1365.536
1387462_at	24260	Chrm3	cholinergic receptor, muscarinic 3	892.4165	333.9444	648.5994
1388497_at	291135	Them2_pre	thioesterase superfamily member 2 (pr	891.9793	766.9834	491.192
1369331_a_at	64830	Unc13b	unc-13 homolog B (C. elegans)	891.8731	623.6858	217.4672
1370335_at	192126	Dab2ip	disabled homolog 2 (Drosophila) intera	891.2552	1627.154	976.9327
1384095_at	360034	Myrip	myosin VIIA and Rab interacting protei	890.9448	268.7867	68.15215
1395589_at	310808	Slc35a3	solute carrier family 35 (UDP-N-acetylg	890.8859	825.4498	304.6462
1395587_at	499210	RGD15612	similar to Alkaline phytoceramidase (al	890.8765	1054.915	405.2525
1383969_at	499143	RGD15618	similar to CDNA sequence BC043301	890.7802	149.0765	738.017
1385349_at	361934	Cetn4_pred	centrin 4 (predicted)	890.7095	451.3799	728.3162
1382062_at	310855	RGD13118	similar to hypothetical protein FLJ1327	890.5996	301.5684	569.6911
1393074_at	313595	RGD15630	similar to hypothetical protein FLJ3898	890.4628	1210.71	823.5742
1370512_at	192178	Cbl27	androgen receptor-related apoptosis-a	889.5269	877.7962	739.0041
1374231_at	366828	Klf16_pred	Kruppel-like factor 16 (predicted)	889.2499	862.0111	858.7618
1374979_at	301122	Dus3l	dihydrouridine synthase 3-like (S. cere	889.194	1206.213	555.6195
1377384_at	360634	Plekhh3	pleckstrin homology domain containing	888.8744	776.0983	381.3471
1392649_at	360818	Fbxo21_pr	F-box only protein 21 (predicted)	888.4257	435.6501	1394.739
1395489_at	361699	Ankrd13d_	ankyrin repeat domain 13 family, mem	888.0114	1271.579	775.16
1372493_at	362578	Ccdc23	coiled-coil domain containing 23	887.7872	1565.581	837.4392
1388209_at	252923	Tsga10	testis specific 10	885.8083	390.9012	816.2707
1374292_at	303749	RGD13050	similar to RIKEN cDNA 1110031I02	885.3252	3063.838	2489.106
1369285_at	81746	Pggt1b	protein geranylgeranyltransferase type	885.0977	1590.354	812.141
1395605_at	287942	Crkl	v-crk sarcoma virus CT10 oncogene h	885.039	780.8482	675.1957
1392593_a_at	361569	RGD13098	similar to KIAA1205 protein (predicted)	884.9624	540.6706	1223.352
1373949_at	362645	Atp13a2_p	ATPase type 13A2 (predicted)	884.7017	665.866	1049.411
1368904_at	63834	Capn10	calpain 10	884.5482	550.3292	741.7546
1380558_at	287638	Dlx3_predi	distal-less homeobox 3 (predicted)	884.2974	388.4295	535.2331
1377887_at	83522	Acox3	acyl-Coenzyme A oxidase 3, pristanoy	884.1967	571.2256	1014.566
1380915_at	311622	Stk4_predi	serine/threonine kinase 4 (predicted)	884.1004	178.9485	529.7791
1398357_at	64832	Cplx1	complexin 1	883.6602	2372.752	370.23
1372328_at	316226	Klc4	kinesin light chain 4	883.3085	1038.441	778.6361
1382399_at	303346	RGD13098	similar to hypothetical protein DKFZp4	883.0435	730.8735	1209.506
1394409_at	362838	Mum1_pre	melanoma associated antigen (mutate	882.9588	1759.829	1107.587
1372840_at	310330	NA	NA	882.1843	2187.277	1997.916
1374018_at	498402	RGD15614	similar to ASC-1 complex subunit P100	882.1329	666.3153	258.9449
1374732_at	303580	Hexim2_pr	hexamthylene bis-acetamide inducible	881.95	843.789	597.3207
1383803_at	310859	RGD13116	similar to KIAA1546 protein (predicted)	881.5792	44.63176	692.5046
1367733_at	54231	Ca2	carbonic anhydrase 2	881.3966	1130.302	2735.755
1369040_at	114116	Cdc42bpa	CDC42 binding protein kinase alpha	880.3146	494.6054	462.5608
1395013_at	313581	RGD15596	similar to CGI-94 protein (predicted)	880.1151	1063.391	867.2051
1398374_at	311346	RGD13071	similar to RIKEN cDNA 2810002D13 g	879.883	884.7767	1510.757
1372574_at	308582	Tead2_ma	TEA domain family member 2 (mappe	879.7508	326.3678	209.1258
1392463_at	317508	RGD15648	similar to hypothetical protein FLJ1450	879.4337	1208.356	4157.951
1382043_at	361689	Unc93b1	unc-93 homolog B1 (C. elegans)	878.8225	1761.718	590.6214
1390848_at	498036	RGD15621	similar to CKT2 (predicted)	878.301	1377.103	427.2714
1375175_at	360537	Tom1l2_pr	target of myb1-like 2 (chicken) (predict	877.9538	85.93484	368.6015

1369197_at	78963	Apaf1	apoptotic peptidase activating factor 1	877.1748	634.8522	704.2479
1387557_s_at	65081	Vps33a	vacuolar protein sorting 33A (yeast)	876.5495	1149.536	1265.835
1372299_at	246060	Cdkn1c	cyclin-dependent kinase inhibitor 1C (F	876.529	62.09737	809.0418
1378290_at	301010	Slc26a6_pr	solute carrier family 26, member 6 (pre	876.4962	581.3972	230.3712
1378408_at	294350	RGD15633	RGD1563351 (predicted)	876.3937	411.4442	1581.926
1378243_at	360985	Tmem17	transmembrane protein 17	874.0208	2563.842	1307.838
1374255_at	288917	Farsla	phenylalanine-tRNA synthetase-like, a	873.5073	979.5198	328.5981
1376710_at	503354	LOC50335	NA	873.1614	366.7466	877.243
1378310_at	499194	RGD15627	similar to neuromedin B precursor - rat	872.7317	312.3231	789.0216
1384089_at	360797	Rabgef1_p	RAB guanine nucleotide exchange fac	872.5917	1026.415	872.8074
1379538_at	305909	Cenpj_prec	centromere protein J (predicted)	871.5476	844.34	3270.439
1371635_at	85494	Gpr175	G protein-coupled receptor 175	871.4208	928.9271	1035.798
1374973_at	292889	Med25_pre	mediator of RNA polymerase II transcr	870.3595	992.6811	1129.788
1368562_at	58953	Sult4a1	sulfotransferase family 4A, member 1	870.2779	1779.867	1596.049
1386581_at	296456	Itgav_predi	integrin alpha V (predicted)	869.3872	193.8737	263.2344
1394590_at	305751	Nkiras1_pr	NFKB inhibitor interacting Ras-like pro	869.2515	932.5463	268.5878
1391498_at	363067	Sin3a_prec	transcriptional regulator, SIN3A (yeast	868.6325	855.1373	1465.237
1391970_at	308588	Car11	carbonic anhydrase 11	868.4785	813.3506	852.4642
1371917_at	362976	Trmu_pred	tRNA 5-methylaminomethyl-2-thiouridy	868.3533	321.2206	489.5783
1392839_at	498176	LOC49817	similar to BRI3-binding protein	867.986	463.8821	396.9134
1390954_at	314897	Ppm1h	protein phosphatase 1H (PP2C domair	867.2209	378.4718	303.3434
1382194_at	302470	F8	coagulation factor VIII	867.0485	789.9944	1049.787
1391071_at	499513	NA	NA	866.8474	3772.678	704.0446
1375600_at	313341	Pigo_predi	phosphatidylinositol glycan, class O (p	866.568	1319.831	316.3103
1383316_at	361462	Hddc2_pre	HD domain containing 2 (predicted)	866.3715	847.8719	47.97585
1391115_at	498229	LOC49822	similar to RIKEN cDNA 4930429O20	866.3028	366.99	289.4509
1377214_a_at	292764	RGD13031	hypothetical protein LOC292764	865.3524	1145.994	520.3139
1387368_at	25482	Mras	muscle and microspikes RAS	865.3237	369.1144	453.0387
1370876_at	116690	Nfasc	neurofascin	865.316	1635.581	1081.402
1380101_at	305586	Papolg_pre	poly(A) polymerase gamma (predicted	865.2457	2097.084	2225.282
1393322_at	287571	Taf15_prec	TAF15 RNA polymerase II, TATA box	864.7794	394.7838	1477.867
1367847_at	113900	Nupr1	nuclear protein 1	864.4114	2260.713	564.5372
1383309_at	304023	St3gal6	ST3 beta-galactoside alpha-2,3-sialyltr	864.0217	988.4425	7163.625
1375851_at	289457	Klhl8_pred	kelch-like 8 (Drosophila) (predicted)	864.0174	946.5263	2000.076
1399110_at	309630	Zfp297	zinc finger protein 297	863.7583	917.8309	451.111
1379354_at	310803	Extl2	exostoses (multiple)-like 2	863.5751	255.7919	477.9948
1388790_at	304542	RGD13108	similar to hypothetical protein D5Ertd3	863.4019	965.2854	315.6503
1368287_at	84030	Chn1	chimerin (chimaerin) 1	862.3038	1059.89	2887.492
1392576_at	361941	RGD13595	similar to protein C33A12.3	862.2476	910.5723	1031.147
1390029_at	361110	Tmem110	transmembrane protein 110	861.8034	2398.349	525.2108
1373750_at	297595	Leprel2_pr	leprecan-like 2 (predicted)	861.502	280.7162	2582.076
1376094_at	246769	Hint3	histidine triad nucleotide binding protei	860.8293	1730.24	1030.713
1377365_at	298694	RGD13110	similar to hypothetical protein DKFZp4	860.7705	102.1525	2007.842
1370069_at	171373	Slc12a5	solute carrier family 12, (potassium-ch	860.6899	1664.39	2004.94
1397500_x_at	58979	Gng7	guanine nucleotide binding protein, ga	859.6147	1204.835	1031.324
1382451_at	308632	Hebp2_pre	heme binding protein 2 (predicted)	858.7713	1110.393	1072.257
1370847_at	171569	Spon2	spondin 2, extracellular matrix protein	858.7622	269.9823	626.8194
1377218_at	296637	LOC29663	NA	858.3709	351.4608	782.9858
1373333_at	317409	MGC1093	similar to Microsomal signal peptidase	858.3426	12258.94	847.3268
1376751_at	361800	Stk19	serine/threonine kinase 19	858.3033	849.4435	181.5405
1377179_at	362642	Pqlc2_prec	PQ loop repeat containing 2 (predictec	858.0003	314.1768	119.4393

1393862_at	500238	RGD15635	similar to RIKEN cDNA 1700019G17 (	857.8913	985.3392	719.7145
1369667_at	25218	Vps52	vacuolar protein sorting 52 (yeast)	856.7085	1693.48	351.9834
1386685_at	305940	Sacs_pred	sacsin (predicted)	856.4189	713.6098	427.419
1383925_at	298643	LOC29864	NA	856.3239	539.5199	657.441
1382843_at	286896	Sgpl1	sphingosine phosphate lyase 1	856.323	1392.365	150.7882
1396280_at	302560	NA	NA	856.2514	754.0277	370.2901
1391664_at	366478	Pigv	phosphatidylinositol glycan, class V	856.1398	399.5777	362.2019
1368736_at	29391	Tsx	testis specific X-linked gene	856.0027	192.3302	1175.41
1389192_at	361520	MGC1091	similar to hypothetical protein FLJ1024	855.5017	946.5368	1115.436
1372069_at	309429	Ankrd15	ankyrin repeat domain 15	854.1316	951.9134	2111.646
1370099_at	64039	Fbxl20	F-box and leucine-rich repeat protein 2	853.5669	1165.783	869.2697
1382321_at	364535	RGD13077	similar to hypothetical protein FLJ2042	853.5201	1090.956	646.3269
1390490_at	301119	LOC30111	NA	852.3358	584.7626	339.7422
1370506_at	25249	Coq7	demethyl-Q 7	852.1674	3990.796	776.6434
1374748_at	287379	Shmt1	serine hydroxymethyl transferase 1 (sc	851.6749	3503.207	1527.013
1375954_at	295213	S100a13_p	S100 calcium binding protein A13 (pre	851.0945	677.5869	1201.616
1398605_at	309009	RGD13067	similar to cDNA sequence BC025641	850.6473	895.9256	465.283
1393461_at	500950	RGD15654	similar to zinc finger protein 75 (predic	850.6085	1129.52	1455.621
1387088_at	29141	Gal	galanin	850.5861	51.60572	71.85528
1368595_at	83513	Mmp24	matrix metallopeptidase 24	849.9188	2235.232	156.148
1368089_at	81743	Pde2a	phosphodiesterase 2A, cGMP-stimulat	849.6601	2305.8	287.7446
1368907_at	65171	Scamp5	secretory carrier membrane protein 5	849.5852	400.2407	128.3266
1369518_at	60664	Pik3r3	phosphatidylinositol 3 kinase, regulato	849.5582	516.0483	310.3035
1391825_at	497910	NA	NA	849.3204	1404.044	365.057
1387663_at	81661	Gmfb	glia maturation factor, beta	848.8795	1008.065	203.3374
1378739_at	500292	LOC50029	similar to cell death-inducing DFFA-like	848.8434	497.6081	17.66631
1368702_at	64513	Pawr	PRKC, apoptosis, WT1, regulator	848.7669	2072.767	3833.783
1382035_at	117526	Tbp	TATA box binding protein	848.4601	1641.266	1299.992
1378406_at	310315	Ccdc39_pr	coiled-coil domain containing 39 (predi	848.1607	509.3456	503.9463
1372895_at	361118	RGD13096	similar to RIKEN cDNA 5730469M10	847.4432	1744.474	3326.875
1382277_at	448830	Ly96	lymphocyte antigen 96	847.2566	400.1463	541.1991
1368011_at	79122	Fdxr	ferredoxin reductase	846.1412	786.1629	819.4056
1392496_at	312273	LOC31227	Trypsin V-A	845.95	31.44972	945.4811
1371040_at	292657	Slc1a5	solute carrier family 1 (neutral amino a	845.3547	935.3057	1931.908
1379349_a_at	288585	Rabl5	RAB, member of RAS oncogene family	845.3489	514.8964	545.2007
1377614_at	293494	CCDC95	similar to hypothetical protein FLJ9065	844.9371	555.0949	621.9913
1383110_at	303803	Klhl24	kelch-like 24 (Drosophila)	844.5699	1021.934	9347.944
1383049_at	498329	NA	NA	844.2704	806.385	836.5183
1374981_at	364136	RGD13111	similar to 2-aminoadipic 6-semialdehyd	844.0387	575.3431	615.2472
1374756_at	315088	RGD13111	similar to 1500031N24Rik protein (pre	843.8227	763.766	625.351
1368560_at	29713	Kcnj5	potassium inwardly-rectifying channel,	843.7694	170.4757	920.0034
1389830_at	25574	Ubtf	upstream binding transcription factor, I	843.6569	781.0989	719.4765
1373414_at	306349	RGD13055	similar to Brain specific membrane-anc	843.6231	2624.174	3551.121
1383230_at	360664	RGD1311C	LOC360664 (predicted)	843.6225	1588.516	123.1921
1370057_at	29276	Csrp1	cysteine and glycine-rich protein 1	843.2482	1466.971	609.7626
1391528_at	307236	RGD13082	similar to mKIAA0863 protein (predicte	842.8732	3154.179	1458.076
1373421_at	316742	Tgif	TG interacting factor	841.6574	244.5146	1896.933
1381695_at	317494	RGD15618	similar to Nance-Horan syndrome prot	841.6562	325.9347	469.5427
1368476_at	25672	Nr3c2	nuclear receptor subfamily 3, group C,	841.0232	320.9269	1131.281
1396834_at	114486	Braf	v-raf murine sarcoma viral oncogene h	839.7501	218.7906	974.3463
1398184_at	314212	Daam1_pr	dishevelled associated activator of mo	839.3521	219.0854	492.1505

1368426_at	83842	Crot	carnitine O-octanoyltransferase	839.0898	838.7098	1268.557
1391405_at	500032	RGD15662	similar to mKIAA0960 protein (predicte	838.5789	680.0062	532.7177
1388475_at	313535	LOC31353	NA	837.7637	1498.695	1070.772
1381355_at	287612	Mks1	Meckel syndrome, type 1	837.3189	1662.175	1695.511
1395559_at	360702	Ttc3_predi	tetratricopeptide repeat domain 3 (prec	837.0348	500.9459	611.7405
1398372_at	497900	MGC11268	similar to RIKEN cDNA 0610039K22	836.8169	808.6017	352.5051
1380981_at	314349	Ston2_prec	stonin 2 (predicted)	836.4397	86.01715	462.4808
1380009_at	362779	Vrk1	vaccinia related kinase 1	836.2715	6878.361	1756.191
1383618_at	303685	Mrpl38	mitochondrial ribosomal protein L38	836.2647	1037.149	1477.025
1376681_at	308911	RGD13083	similar to RIKEN cDNA 1500003O22	836.1731	553.1103	735.4688
1374914_at	25682	Ppard	peroxisome proliferator activated recep	835.7137	838.8346	1552.141
1376553_at	29541	Nthl1_pred	nth (endonuclease III)-like 1 (E.coli) (p	835.592	577.6895	285.9468
1369156_at	79209	Frk	fyn-related kinase	835.5161	75.78253	1310.802
1387826_at	83578	Pdxk	pyridoxal (pyridoxine, vitamin B6) kinas	835.4188	856.1374	316.1251
1387529_a_at	63837	Tagln3	transgelin 3	834.4532	545.0791	302.4807
1386280_at	366792	Mettl7b	methyltransferase like 7B	833.9942	296.4626	561.2751
1394216_at	314214	RGD13057	similar to RIKEN cDNA 2810055F11 (p	833.6012	409.2387	517.3688
1382168_at	282824	Elavl3	ELAV (embryonic lethal, abnormal visi	833.0023	468.4241	92.12953
1392963_at	502776	Scrn1	secernin 1	832.9322	402.1712	426.7648
1379927_at	365894	Trim33_pre	tripartite motif protein 33 (predicted)	832.4724	118.6574	383.586
1369042_at	79112	Pigm	phosphatidylinositol glycan, class M	832.4483	502.4265	400.6667
1386987_at	24499	Il6ra	interleukin 6 receptor, alpha	832.007	1738.671	2778.885
1385073_at	314232	NA	NA	831.586	1810.811	31192.56
1389106_at	288921	Fbxw9	F-box and WD-40 domain protein 9	831.184	835.6789	1456.038
1381215_at	292732	Numbl	numb-like	830.948	804.4656	619.0859
1393635_x_at	29213	Tubb4	tubulin, beta 4	830.9309	2992.5	1700.53
1392177_at	498458	NA	NA	830.0941	196.4291	125.6954
1393741_at	308766	Mesp1_pre	mesoderm posterior 1 (predicted)	829.8054	285.4228	335.6928
1387236_at	84428	Dctn4	dynactin 4	829.4993	1715.313	769.6622
1367745_at	81821	Dgkz	diacylglycerol kinase zeta	829.3339	1007.981	1557.074
1373823_at	498709	RGD15620	similar to Cyclin-dependent kinases re	828.9827	27788.57	3843.932
1370536_at	89820	Hrmt1l3	heterogeneous nuclear ribonucleoprote	828.4682	1423.072	332.0769
1392294_at	501285	LOC50128	NA	827.8844	503.9473	339.6926
1372942_at	308441	Exosc5_pre	exosome component 5 (predicted)	827.7475	1552.958	137.5334
1381305_at	308509	RGD13084	similar to RIKEN cDNA 4931406P16 (p	827.7059	1236.558	1531.244
1386266_at	500368	RGD15659	similar to male sterility domain contain	827.5337	570.5652	1430.6
1375504_at	303612	Polg2_prec	polymerase (DNA directed), gamma 2,	827.2268	835.8603	2192.02
1371382_at	293860	Flna_predi	filamin, alpha (predicted)	826.901	413.4586	318.7529
1382061_at	307858	Ldhd	lactate dehydrogenase D	826.8242	216.4807	370.0587
1377121_at	305645	Dlg5_predi	discs, large homolog 5 (Drosophila) (p	826.4747	2885.312	2080.472
1387260_at	114505	Klf4	Kruppel-like factor 4 (gut)	826.3589	458.7674	38541.75
1383606_at	500707	Mtac2d1	membrane targeting (tandem) C2 dom	826.2957	867.6827	1840.177
1389114_at	297332	RGD13091	similar to Hypothetical protein MGC59	826.0048	455.9438	1483.628
1386755_at	312863	LOC31286	similar to RIKEN cDNA 1810060J02	826.0041	2406.745	861.3777
1376392_at	287931	Ppm1f	protein phosphatase 1F (PP2C domain	825.9553	338.7101	526.9135
1376075_at	29192	Psen1	presenilin 1	825.7289	700.4558	516.1259
1380197_at	304813	Ppp1r12b_	protein phosphatase 1, regulatory (inhi	825.5185	247.2416	339.1917
1379488_at	362272	Trp53rk_pr	TP53 regulating kinase (predicted)	825.2226	1386.724	1448.629
1385690_at	301276	RGD15649	similar to Mut protein (predicted)	825.1512	811.3785	516.4018
1379674_at	361431	Cbfa2t3_pr	core-binding factor, runt domain, alpha	824.5261	340.5624	765.9671
1391501_at	295106	Ift80	intraflagellar transport 80 homolog (Ch	824.1955	2623.876	1178.161

1376730_at	360878	RGD1561C	similar to kelch/ankyrin repeat containi	824.18	632.6351	790.8134
1382235_at	365699	RGD13068	similar to hypothetical protein FLJ3059	823.6185	414.7684	1328.267
1370386_at	65137	Ruvbl1	RuvB-like protein 1	823.0703	2709.99	619.1705
1379626_at	316164	Satb1	special AT-rich sequence binding prote	823.038	2435.57	11454.13
1379235_x_at	287961	LOC28796	NA	822.9472	4115.097	586.5089
1367568_a_at	25333	Mgp	matrix Gla protein	822.8638	9.31803	11138.95
1384367_at	690148	LOC69014	NA	822.5431	144.001	394.8174
1388512_at	363272	Pde6d_pre	phosphodiesterase 6D, cGMP-specific	822.4969	1584.054	504.5405
1398434_at	362286	Datf1_pred	death associated transcription factor 1	821.9872	1299.624	2597.672
1382592_at	303037	RGD13069	similar to RIKEN cDNA 2600001J17	821.808	1898.39	373.8194
1392570_at	299196	Abcd4	ATP-binding cassette, sub-family D (A	821.5369	583.7971	375.0374
1387432_at	83515	Rod1	ROD1 regulator of differentiation 1 (S.	821.2184	596.742	484.5863
1370323_at	64517	Thop1	thimet oligopeptidase 1	821.1525	484.8551	181.4512
1393145_at	360833	Fvt1_predi	follicular lymphoma variant translocatic	821.0847	392.2666	388.8942
1389193_at	305438	Sorcs2_pre	sortilin-related VPS10 domain containi	821.0616	3806.218	2769.8
1395403_at	362895	Stac3_pred	SH3 and cysteine rich domain 3 (predi	820.8116	254.0555	327.6502
1391427_at	294718	RGD13117	similar to RIKEN cDNA 1500031M22 (	820.7956	1734.705	1567.955
1372324_at	497975	LOC49797	NA	820.7479	5450.597	1826.045
1383424_at	314004	Tyki_predic	thymidylate kinase family LPS-inducibl	820.5582	2431.856	3543.291
1393759_at	501191	NA	NA	820.0422	78.00512	762.0831
1371819_at	84580	Hdac5	histone deacetylase 5	819.9362	1288.654	2228.066
1398022_at	313928	RGD13108	similar to KIAA0953 protein (predicted)	819.4367	175.7632	2818.006
1377503_at	308201	Riok2	RIO kinase 2 (yeast)	819.3358	708.9078	970.972
1397811_at	363018	RGD13117	similar to KIAA1731 protein (predicted)	818.8514	1463.999	589.8501
1389078_at	362941	Fbxl6	F-box and leucine-rich repeat protein 6	818.4042	1514.018	647.0223
1374176_at	362535	RGD1308C	similar to DNA segment, Chr 4, Brigha	818.244	2483.211	2030.381
1396104_at	292884	RGD1305C	similar to Zinc finger protein 184 (predi	817.841	380.7368	356.0941
1390927_at	654482	LOC65448	NA	817.8357	308.4339	607.583
1383214_at	291309	Usp6nl_pre	USP6 N-terminal like (predicted)	817.818	80.22714	811.3986
1384143_at	365864	Tuft1_pred	tuftelin 1 (predicted)	817.7087	263.28	828.0572
1374127_at	303226	Zfp535	zinc finger protein 535	817.5437	1155.319	866.2278
1392109_at	311372	RGD15625	similar to hypothetical protein FLJ2143	816.0747	479.2508	1958.545
1377696_at	308795	Mesdc1	mesoderm development candidate 1	815.2674	1327.327	1468.901
1388884_at	291076	RGD13102	similar to RIKEN cDNA 1810022C23	815.2593	20.00481	1492.128
1387678_at	24605	Nras	neuroblastoma ras oncogene	815.0799	1482.253	725.8296
1376144_at	303905	Parp9_pred	poly (ADP-ribose) polymerase family, r	814.7098	508.6508	1631.81
1377402_at	361434	Cdk10	cyclin-dependent kinase (CDC2-like) 1	814.6145	468.2607	447.6557
1384791_at	305571	B3gnt1_pre	UDP-GlcNAc:betaGal beta-1,3-N-acety	814.5988	227.7953	128.6354
1377252_at	287596	RGD13068	similar to RIKEN cDNA 1200011M11 (	814.52	1264.563	539.6547
1398469_at	171378	Ppp3cc	protein phosphatase 3, catalytic subun	814.1055	324.7084	450.7989
1372178_at	497848	LOC49784	NA	814.0836	1138.236	824.2188
1370551_a_at	29744	Sema6c	sema domain, transmembrane domair	813.9309	333.8159	366.2265
1390573_a_at	305897	Nfatc4	nuclear factor of activated T-cells, cyto	813.4876	183.3976	396.1764
1385636_at	266715	Fzd3	frizzled homolog 3 (Drosophila)	813.4762	308.971	631.2035
1372617_at	500793	RGD1566C	similar to BTB (PO)Z domain containin	813.2962	1462.106	1064.479
1393423_at	309512	Tnks2_pred	tankyrase, TRF1-interacting ankyrin-re	812.2764	2233.309	634.1096
1398958_at	360496	Narfl	nuclear prelamin A recognition factor-li	811.6514	1350.734	340.1035
1388543_at	309014	RGD13062	similar to HTPAP protein (predicted)	811.4635	400.2871	489.0958
1384070_at	306357	Gmip_pred	Gem-interacting protein (predicted)	810.3551	1101.038	810.5964
1391216_at	305428	Zfp509_pre	zinc finger protein 509 (predicted)	810.2958	771.2681	661.2714
1389209_at	315218	RGD13062	similar to hypothetical protein BC0029	810.2807	1914.825	1039.719

1393055_at	207122	Pkn2	protein kinase N2	809.5549	847.8047	690.4249
1382284_at	306576	Nek3_pred	NIMA (never in mitosis gene a)-relatec	809.42	991.0281	940.3758
1374147_at	296478	Zgpat	zinc finger, CCCH-type with G patch d	809.0804	456.4296	426.202
1398610_at	619581	Nicn1	NA	808.8046	1513.238	1078.495
1385227_at	299897	Trps1_pred	trichorhinophalangeal syndrome I (pre	808.6563	386.5271	645.2255
1368883_at	81526	Nov	nephroblastoma overexpressed gene	808.6028	319.569	2931.523
1390426_at	25496	Notch1	Notch gene homolog 1 (Drosophila)	808.2579	445.4715	635.2083
1384957_at	304962	Atf6_predic	activating transcription factor 6 (predic	807.8329	367.5668	264.496
1373014_at	293722	B3gat3_pre	beta-1,3-glucuronyltransferase 3 (gluc	807.799	849.1078	357.0822
1390267_at	306879	Fars2	phenylalanine-tRNA synthetase 2 (mito	807.5098	1028.787	1103.384
1376018_at	299625	RGD15638	similar to Lmnb2 protein (predicted)	807.4974	1913.372	944.8739
1393011_at	361041	LOC36104	similar to hypothetical protein	807.2084	478.3635	964.0141
1368208_at	171084	Cml1	camello-like 1	806.9748	781.7742	1024.987
1396320_at	192350	Crkrs	Cdc2-related kinase, arginine/serine-ri	806.9516	531.4157	1317.48
1379778_at	298032	RGD13079	similar to HSPC043 protein (predicted)	806.9351	1602.767	1342.552
1377596_a_at	299905	Thrap6_pre	thyroid hormone receptor associated p	806.5648	1688.279	824.7273
1389746_at	360630	RGD15642	similar to Naglu (predicted)	806.2357	1212.245	319.5811
1389351_at	367314	LOC36731	similar to FLI-LRR associated protein-1	805.9822	367.2859	880.7333
1396420_at	368002	Baz1b	NA	805.7234	121.4623	478.6014
1370265_at	25388	Arrb2	arrestin, beta 2	805.6911	1032.049	325.6088
1391527_at	362896	Stat6_pred	signal transducer and activator of trans	805.6747	462.9934	1645.922
1395264_at	295602	RGD15624	similar to Rap1-interacting factor 1 (pre	805.6649	186.6545	118.1781
1387264_at	116491	Kcnk6	potassium channel, subfamily K, memb	805.4662	835.8951	304.3837
1398332_at	498118	NA	NA	805.1017	970.5193	462.8098
1368002_at	81709	Msh2	mutS homolog 2 (E. coli)	804.193	5819.897	961.8564
1374455_at	293679	NA	NA	804.0702	3455.02	791.2074
1384134_at	498626	NA	NA	803.2562	77.2753	615.4885
1382272_at	298646	Agtrap	angiotensin II, type I receptor-associate	803.1867	318.1071	161.6994
1386928_at	64203	Bcat2	branched chain aminotransferase 2, m	802.9904	90.59092	203.7475
1376140_at	363516	Abcd1_pre	ATP-binding cassette, sub-family D (A	802.9211	95.52601	233.2371
1384194_at	301440	Fzd7_pred	frizzled homolog 7 (Drosophila) (predic	801.7636	591.7436	930.5877
1384378_at	406196	Hcr	HCR (a-helix coiled-coil rod homolog)	801.6903	1338.728	770.1406
1389232_at	303907	Wdr5b	WD repeat domain 5B	800.8533	537.7224	395.6036
1395678_at	306285	Syt15	synaptotagmin 15	800.7965	381.2792	643.9175
1369796_at	50564	Gja9	gap junction membrane channel protei	799.8455	570.3646	93.22607
1371092_at	286960	LOC28696	preprotrypsinogen IV	799.6869	67.96761	162.9337
1374691_at	292077	Sult5a1_pr	sulfotransferase family 5A, member 1 (	799.5942	452.4358	351.4529
1374304_at	309995	Xrcc4	X-ray repair complementing defective r	799.1901	785.4863	1311.43
1390781_at	361439	Abcb10	ATP-binding cassette, sub-family B (M	799.1257	820.0181	621.8136
1387042_at	25297	Cacnb3	calcium channel, voltage-dependent, b	798.4648	4236.864	2367.417
1388396_at	373542	Stk25	serine/threonine kinase 25 (STE20 hor	797.7334	449.1594	795.8943
1382532_at	298567	Ela3b_pred	elastase 3B, pancreatic (predicted)	797.6679	46.4275	1591.714
1368306_at	24411	Grin2c	glutamate receptor, ionotropic, NMDA2	797.453	296.773	533.4484
1375498_at	304488	RGD13103	similar to T-cell activation protein phos	797.3056	200.6061	86.54628
1385801_at	291677	Dnajc18	DnaJ (Hsp40) homolog, subfamily C, m	797.2547	697.8539	550.6469
1374733_at	292683	Sympk	symplekin	797.2395	687.6509	751.0442
1367965_at	24782	Slc9a1	solute carrier family 9, member 1	796.6774	382.8198	573.5137
1396709_at	288204	RGD13056	similar to Hypothetical protein 5031404	796.491	238.7424	1005.065
1390272_at	295394	Dph5	DPH5 homolog (S. cerevisiae)	796.4408	553.0324	354.5057
1373670_at	288774	Stat2	signal transducer and activator of trans	796.1747	646.0315	1334.746
1370319_at	282819	Ppif	peptidylprolyl isomerase F (cyclophilin	795.9447	1449.872	1011.981

1374576_at	361546	NA	NA	795.4438	1328.202	657.3978
1392586_at	305861	RGD1308C	similar to RIKEN cDNA 4932432K03	795.0885	441.2208	527.4807
1390122_at	501096	RGD15651	similar to triggering receptor expressec	794.4414	983.4835	1009.988
1378127_at	361258	Cul2_predi	cullin 2 (predicted)	794.4036	2974.97	1540.602
1392932_at	361506	Leng8	leukocyte receptor cluster (LRC) meml	794.0584	166.6574	1667.522
1371262_at	314487	Igha_mapp	immunoglobulin heavy chain (alpha po	793.8882	145.9274	689.1486
1386059_at	295274	RGD15623	similar to histone H2b-613 (predicted)	793.8611	1287.1	3225.369
1370917_at	79245	Hsf1	heat shock transcription factor 1	793.6006	732.4573	1132.656
1370370_at	64468	Hyal2	hyaluronoglucosaminidase 2	793.5881	823.8195	452.9184
1378527_at	303590	LOC30359	NA	793.0114	30.07248	361.6383
1369073_at	60351	Nr1h4	nuclear receptor subfamily 1, group H,	792.6358	248.2329	1441.983
1387337_at	25305	Cort	cortistatin	791.4328	661.2811	567.5678
1373254_at	450225	Krt10	keratin 10	791.2195	2324.451	3233.738
1370966_at	114244	Hcn2	hyperpolarization activated cyclic nucle	790.9365	519.8604	560.4117
1379938_at	310230	Slc10a5	solute carrier family 10 (sodium/bile ac	790.492	508.194	1100.009
1372021_at	366016	Nup188	nucleoporin 188	790.3122	1098.232	488.5287
1378046_at	307848	Fuk_predic	fucokinase (predicted)	789.798	438.1866	513.3307
1373956_at	363442	Fundc1	FUN14 domain containing 1	789.7191	1425.167	3836.898
1384265_at	293144	Pold3	polymerase (DNA-directed), delta 3, ac	789.6251	1170.424	703.2639
1373247_at	29392	Bace1	beta-site APP cleaving enzyme 1	789.6019	881.114	1188.888
1370474_at	24831	Thrb	thyroid hormone receptor beta	789.3703	306.62	69.31953
1388280_a_at	58948	Dlgh3	discs, large homolog 3 (Drosophila)	788.7569	567.451	53.72165
1398840_at	89818	Vamp5	vesicle-associated membrane protein !	788.664	352.0936	412.2912
1389243_at	500483	NA	NA	788.6326	1762.771	1880.305
1378895_at	308581	RGD1561s	similar to hypothetical protein FLJ3265	788.2495	374.4053	306.4997
1377729_at	315851	Elovl4_pre	elongation of very long chain fatty acid	787.9932	1549.639	1992.561
1395596_at	500596	NA	NA	787.5599	239.4255	504.5525
1390566_a_at	29593	Ckmt1	creatine kinase, mitochondrial 1, ubiqu	787.3274	6497.396	1427.706
1398438_at	290633	Gtpbp3	GTP binding protein 3	787.2497	490.9458	714.1719
1387111_at	64157	Ddah1	dimethylarginine dimethylaminohydrola	786.9798	246.4034	1724.134
1382417_at	304798	Mdm4	transformed mouse 3T3 cell double mi	786.7753	663.6615	430.3673
1383258_at	360894	RGD13105	similar to hypothetical protein FLJ1414	786.7266	850.2122	1641.917
1390114_at	360871	Mpzl1	myelin protein zero-like 1	786.7213	1107.324	1634.435
1377624_at	312054	Cldn12_pre	claudin 12 (predicted)	786.5744	1030.206	761.022
1367918_at	81730	Fez1	fasciculation and elongation protein ze	786.4191	1177.073	3679.415
1385168_at	310775	RGD13065	similar to receptor-interacting factor 1	786.342	986.6325	2294.482
1393701_at	315951	RGD13102	similar to Alpha-1,4-N-acetylglucosami	786.1293	354.8305	1690.534
1373065_at	301333	Ptpn18	protein tyrosine phosphatase, non-rece	786.1193	1650.757	1638.855
1381252_at	24763	Sah	SA rat hypertension-associated gene	785.5224	1286.423	542.2248
1369031_at	84388	Il18bp	interleukin 18 binding protein	785.3473	363.9689	643.5996
1392526_at	498702	NA	NA	785.3227	1147.147	1048.176
1382201_at	362049	Unc5c	unc-5 homolog C (C. elegans)	784.9795	715.4149	537.8952
1370887_at	84574	Tgfb1i1	transforming growth factor beta 1 indu	784.432	377.4257	618.614
1369669_at	117041	Nln	neurolysin (metallopeptidase M3 family	784.2945	924.6706	640.3711
1375020_at	314397	Rin3_predi	Ras and Rab interactor 3 (predicted)	784.2457	55.16915	385.8752
1383301_at	498154	LOC49815	hypothetical protein LOC498154	783.6915	1308.01	990.7926
1392360_at	288240	RGD15653	similar to homolog of Human holocarb	783.6701	78.6233	235.6641
1382428_at	311468	Tasp1_pre	taspase, threonine aspartase 1 (predic	783.056	699.2716	2421.808
1372546_at	315994	Mapkapk3	mitogen-activated protein kinase-activa	782.898	61.02992	75.18703
1384264_at	308572	Myh14	myosin, heavy polypeptide 14	782.8883	2118.519	400.7513
1378466_at	362204	Casc4_pre	cancer susceptibility candidate 4 (pred	782.8264	119.7878	185.0909

1383792_at	297872	Sytl1	synaptotagmin-like 1	782.6346	50.53735	371.5038
1394991_at	363520	Irak1_pred	interleukin-1 receptor-associated kinas	782.3226	939.5829	284.092
1393887_at	362866	Chpt1	choline phosphotransferase 1	781.7427	543.7398	3065.606
1378394_at	361344	Mppe1_pre	metallophosphoesterase 1 (predicted)	780.8036	714.2415	2858.803
1393610_at	362618	RGD13051	similar to hypothetical protein BC0081	780.73	440.591	227.2772
1369820_at	117020	Mcf2l	mcf.2 transforming sequence-like	780.6373	1453.315	243.1953
1381847_at	500585	RGD15614	similar to BC021442 protein (predicted	780.1576	772.5706	1481.871
1376907_at	29457	Mtap6	microtubule-associated protein 6	778.4938	1316.313	738.026
1374849_at	315879	Adamts7_p	a disintegrin-like and metallopeptidase	778.3116	322.0002	420.7439
1368325_at	25313	Egf	epidermal growth factor	778.1365	259.0436	1488.611
1382743_at	641315	LOC64131	NA	778.1104	111.8082	914.4211
1387294_at	117186	Sh3bp5	SH3-domain binding protein 5 (BTK-as	777.6912	544.199	3645.989
1369814_at	29538	Ccl20	chemokine (C-C motif) ligand 20	777.6561	109.0534	185.0866
1372417_at	361526	Sertad1	SERTA domain containing 1	777.4485	249.8098	1212.224
1372398_at	499129	LOC49912	NA	777.3635	2015.996	844.4015
1377338_at	294800	Rad1_pred	RAD1 homolog (S. pombe) (predicted)	777.2378	1685.202	1192.633
1378860_at	502321	NA	NA	777.1676	1232.111	1154.863
1373556_at	298861	RGD15617	similar to CG14903-PA (predicted)	777.0623	5258.421	917.7544
1381118_at	311279	RGD13114	similar to RIKEN cDNA 2700007P21	776.7645	921.6379	173.8453
1379029_at	497894	RGD15601	similar to Zinc finger protein 62 homolo	776.7585	374.1747	1812.971
1373935_at	289758	Pold2	polymerase (DNA directed), delta 2, re	776.5297	2644.637	614.216
1372343_at	295050	Exosc8_pr	exosome component 8 (predicted)	776.3515	4323.93	2523.149
1373924_at	302890	RGD13065	similar to C530044N13Rik protein	776.0357	1924.594	853.7597
1390696_at	305448	RGD15664	similar to hypothetical protein A930013	775.8505	426.2408	273.8005
1393217_at	289453	Abcg3	ATP-binding cassette, sub-family G (W	775.6591	463.8052	839.6622
1389676_at	293488	RGD13106	similar to hypothetical protein BC0119	775.4686	2184.339	504.4763
1399056_at	365592	LOC36559	similar to CG10585-PA	775.3707	525.5313	622.3812
1388963_at	304900	Astn1	astrotactin 1	775.2467	2322.905	431.5995
1384407_at	287063	RGD13114	similar to RIKEN cDNA 1110025F24 (p	775.232	3311.838	1017.081
1393123_at	296545	C8g_predic	complement component 8, gamma pol	774.6984	792.3896	711.0598
1390289_at	309855	RGD13113	similar to RIKEN cDNA A530089I17 (p	774.6795	2282.784	1574.983
1389483_at	315496	Dpy19l1_p	dpy-19-like 1 (C. elegans) (predicted)	774.6438	1627.926	2292.279
1379289_at	364675	B4galt7	xylosylprotein beta1,4-galactosyltransf	774.3165	571.491	513.611
1374287_at	294508	Pald	paladin	774.0886	573.0413	408.7855
1372288_at	289783	RGD13092	similar to D11Bwg0280e protein	773.8739	1192.58	1601.326
1374388_at	298609	Efhd2	EF hand domain containing 2	773.2998	562.6304	602.1333
1370249_at	24230	Bzrp	benzodiazepine receptor, peripheral	772.2553	247.7941	140.4161
1394535_at	311478	Kif16b_pre	kinesin family member 16B (predicted)	772.1586	437.8817	296.4558
1371722_at	362967	RGD13115	similar to CGI-96 protein; gastric cance	772.1183	1153.159	363.8623
1377079_a_at	289219	Ppox_pred	protoporphyrinogen oxidase (predicted	771.6731	167.7692	331.2888
1391733_at	362556	Ttc4	tetratricopeptide repeat domain 4	771.2853	1354.683	351.9104
1389747_at	309646	Slc26a8_pr	solute carrier family 26, member 8 (pre	771.2727	603.4827	621.1468
1398646_at	500853	RGD15654	similar to DKFZP434I092 protein (pred	771.2252	2008.157	1642.947
1387876_at	25126	Stat5b	signal transducer and activator of trans	771.1864	519.5822	232.5777
1387375_at	25659	Khk	ketohexokinase	769.9937	445.5843	380.1739
1397316_at	299204	Nek9_pred	NIMA (never in mitosis gene a)- relate	769.8534	158.8959	784.826
1393155_at	58936	Plk3	polo-like kinase 3 (Drosophila)	769.3272	347.781	473.7147
1374620_at	81613	Ceacam1	CEA-related cell adhesion molecule 1	769.0327	4023.395	478.043
1385167_at	363194	Xpo5_pred	exportin 5 (predicted)	768.5412	774.8382	216.1665
1374639_at	303309	RGD13096	similar to KIAA0732 protein	767.6872	628.5874	601.5523
1379311_at	316327	Arid5a	AT rich interactive domain 5A (Mrf1 lik	766.2485	380.6609	414.067

1375382_at	309595	Mdc1	mediator of DNA damage checkpoint 1	766.0631	1201.753	207.1519
1380857_at	306231	RGD1310	similar to CG31759-PA	765.911	656.6537	512.756
1381543_at	291840	LOC29184	amino acid transporter	765.8019	433.3923	101.3154
1390937_at	171026	Akap5	A kinase (PRKA) anchor protein 5	764.6624	2382.155	2962.818
1370464_at	170913	Abcb1a	ATP-binding cassette, sub-family B (M	764.5532	42.97498	48.46165
1387812_at	25507	Pcsk6	proprotein convertase subtilisin/kexin t	763.8599	1457.567	586.9008
1376918_at	304917	Serpinc1	serine (or cysteine) peptidase inhibitor	763.4147	271.4407	777.6627
1374362_at	307834	RGD13066	similar to 4930566A11Rik protein (pred	762.269	1477.929	1632.892
1392517_at	498266	LOC49826	similar to Golgin 45 (Basic leucine zipp	761.8726	708.3711	530.4887
1388156_at	29322	Plcb3	phospholipase C, beta 3	761.5313	858.2695	194.7439
1373251_at	499671	LOC49967	similar to hypothetical protein MGC388	760.9024	666.4496	560.6743
1383961_a_at	362666	Dnajc11_p	DnaJ (Hsp40) homolog, subfamily C, r	760.6209	722.0861	344.3908
1383109_at	310687	Prcc_predi	papillary renal cell carcinoma (transloc	760.1702	971.0974	463.7451
1367957_at	54293	Rgs3	regulator of G-protein signalling 3	760.1362	1039.644	1336.515
1393116_at	287525	Smyd4_pre	SET and MYND domain containing 4 (	759.6104	596.7234	991.9774
1377864_at	297458	RGD13052	similar to RIKEN cDNA C130022K22 g	758.892	960.0565	842.3323
1398358_a_at	257645	Itgb5	integrin, beta 5	758.8325	706.2391	769.1402
1368695_at	24236	C4bpb	complement component 4 binding prot	758.7227	130.116	110.1698
1396924_at	362971	Mpped1_pr	metallophosphoesterase domain conta	758.5851	351.5482	107.7841
1378741_at	362739	RGD13092	similar to putative phosphatase subuni	758.4638	311.1407	756.0356
1376950_at	308775	Det1	de-etiolated homolog 1 (Arabidopsis)	758.118	512.6475	285.9796
1380561_at	364070	Iars2_pred	isoleucine-tRNA synthetase 2, mitocho	758.1143	563.7415	761.1604
1397378_at	288354	RGD13083	hypothetical LOC288354 (predicted)	758.0178	35.07282	548.5584
1392257_at	501706	LOC50170	hypothetical protein LOC501706	757.8181	681.209	141.6626
1397874_at	501688	RGD15655	similar to U7 snRNA-associated Sm-lik	757.8092	591.4391	731.7163
1377302_a_at	291939	Mmaa_pre	methylmalonic aciduria (cobalamin def	757.7672	172.6865	1169.651
1390736_at	309002	Zfp668_pre	zinc finger protein 668 (predicted)	757.5869	1161.844	291.1447
1369052_at	170849	Zfp111	zinc finger protein 111	757.3559	960.1445	629.9985
1373672_at	361241	MGC94010	similar to SPI6	757.3036	274.5494	2706.796
1381283_at	306765	Zfp346_pre	zinc finger protein 346 (predicted)	757.0141	500.6905	794.7627
1369567_at	113914	Taar1	trace-amine-associated receptor 1	756.8159	261.9643	578.0197
1387843_at	24373	Fst	follistatin	756.6788	27.29792	357.634
1372906_at	295930	RGD13095	similar to hypothetical protein MGC408	756.4173	4625.609	2144.015
1383319_at	311423	Slc4a11_pr	solute carrier family 4, sodium bicarbon	756.0503	407.5648	653.2528
1372985_at	292569	Zfp444_pre	zinc finger protein 444 (predicted)	755.492	488.5268	568.6908
1370111_at	54262	Kcnn2	potassium intermediate/small conducta	755.092	383.7633	1990.32
1387392_at	26955	Af6	afadin	754.6062	1304.156	419.2799
1378041_at	362302	Abcb8	ATP-binding cassette, sub-family B (M	754.4519	1306.01	237.754
1376072_at	301634	RGD13099	hypothetical LOC301634	753.4949	794.99	1857.013
1376792_at	500221	RGD15597	similar to CDNA sequence BC014699	753.2428	151.8158	53.61067
1382014_at	297411	Tprkb	Tp53rk binding protein	753.1234	1658.418	554.6655
1372795_at	292008	Txnl4b	thioredoxin-like 4B	753.1177	571.3014	1147.005
1390334_at	293493	RGD13082	similar to hypothetical protein DKFZp4	752.4118	1391.854	1227.33
1393223_a_at	288683	RGD13067	similar to RIKEN cDNA 1110008J03 (p	752.0919	1756.352	520.7048
1390700_s_at	362981	Hdac10	histone deacetylase 10	751.6144	513.4539	393.5542
1375951_at	83580	Thbd	thrombomodulin	751.1676	35.02578	729.885
1375427_at	360739	Ranbp1_pr	RAN binding protein 1 (predicted)	750.5425	707.4586	285.6729
1372780_at	313529	Tmem53_p	transmembrane protein 53 (predicted)	749.5835	1095.362	885.1838
1389103_at	290842	RGD13073	similar to RIKEN cDNA 2810037C03	748.6629	3069.615	1011.856
1380256_at	310685	Polr3c	polymerase (RNA) III (DNA directed) p	748.0978	819.1768	680.4721
1393127_at	360754	Zfp358_pre	zinc finger protein 358 (predicted)	748.0214	1108.576	855.6502

1390428_at	305244	Thap6_pre	THAP domain containing 6 (predicted)	747.2904	1447.916	937.6597
1393012_at	305467	RGD15606	similar to novel protein (predicted)	746.9031	327.058	608.7126
1372059_at	288176	RGD13094	similar to RIKEN cDNA 2610528E23	746.5148	6734.453	2123.542
1381825_at	313018	RGD13032	similar to chromosome 1 open reading	746.2423	659.0284	666.9253
1374799_at	362438	RGD15625	similar to mKIAA0159 protein (predicte	745.4942	3841.818	546.7287
1390847_at	308602	Tmem86a_	transmembrane protein 86A (predicted	745.4211	222.5953	255.0519
1374355_at	315283	RGD1561C	similar to MGC47262 protein	745.2435	767.8376	1104.849
1380650_at	362956	Triobp	TRIO and F-actin binding protein	745.2216	408.7412	305.7862
1391507_at	500110	LOC50011	similar to zinc finger protein EZI	745.2002	3001.965	851.1376
1392901_at	367113	LOC36711	similar to RIKEN cDNA A430093J20 g	744.7481	744.4866	3148.809
1386571_at	287622	RGD15626	similar to adaptor molecule SRCASM (	743.908	954.0634	536.7894
1389643_at	314598	Wiz_predic	widely-interspaced zinc finger motifs (p	743.6146	624.9728	2191.69
1383101_at	361389	Anapc10_p	anaphase promoting complex subunit	743.3229	792.229	1331.604
1371122_at	252961	Tank	TRAF family member-associated Nf-ka	743.2558	440.9172	1114.666
1397391_at	296777	NA	NA	743.0598	501.2999	483.2134
1375533_at	297523	Vgll4	vestigial like 4 (Drosophila)	742.7013	1263.648	1062.955
1383104_at	361606	RGD15614	similar to RIKEN cDNA 1810020D17 (p	742.3007	339.5854	991.1292
1384915_at	305918	Ift88_predi	intraflagellar transport 88 homolog (Ch	742.2626	532.4393	1181.06
1392727_at	300880	RGD13073	similar to KIAA1009 protein	742.2353	262.8937	950.1649
1374254_a_at	300441	RGD15632	RGD1563250 (predicted)	742.0365	1433.882	404.7236
1368309_at	50551	Txnrd2	thioredoxin reductase 2	742.0332	760.1898	429.9723
1373134_at	296131	Fahd2a_pr	fumarylacetoacetate hydrolase domain	741.9183	647.4148	787.0723
1369610_at	60442	Lin7c	lin-7 homolog C (C. elegans)	740.8857	1308.729	492.075
1372931_at	367743	Praf2_pred	PRA1 domain family 2 (predicted)	740.833	2710.122	994.9722
1388174_at	116466	Wnt2b	wingless-type MMTV integration site fa	740.3678	345.4721	404.5766
1399150_at	306229	RGD1306C	similar to RIKEN cDNA A630054L15; h	740.1522	2978.782	1536.682
1369944_at	81520	Marcksl1	MARCKS-like 1	739.8479	9483.862	3137.37
1370285_at	246047	Calcoco1	calcium binding and coiled coil domain	739.807	995.5762	1295.744
1381827_at	299317	RGD15658	similar to MGC4645 protein (predicted	739.7981	511.927	424.6815
1376638_at	302937	RGD13058	similar to RIKEN cDNA 2700067D09	739.734	1034.228	942.1994
1383608_at	306344	Arrdc2	arrestin domain containing 2	739.3034	917.8929	1032.118
1374234_at	362668	Hkr3	GLI-Kruppel family member HKR3	738.808	441.1841	710.4919
1370933_at	25484	Myo1e	myosin IE	738.4742	275.0829	659.901
1385252_at	293294	Trim34_pre	tripartite motif protein 34 (predicted)	737.5758	750.088	3063.834
1396421_at	365611	NA	NA	737.4857	439.4753	503.026
1378235_at	303407	Gdpd1_pre	glycerophosphodiester phosphodiester	737.1	1955.388	848.7514
1369404_a_at	60391	Nrxn1	neurexin 1	736.9221	1612.173	582.8879
1381985_at	312510	Tia1	cytotoxic granule-associated RNA binc	736.9132	1031.601	1505.612
1372048_at	297433	Podxl2_pre	podocalyxin-like 2 (predicted)	736.7241	969.3506	1202.55
1380226_at	499957	RGD15632	similar to Sterile alpha motif domain cc	736.5369	774.3855	537.9777
1381301_at	288010	LOC28801	similar to LIM domain containing prefe	735.3372	91.58695	305.9636
1383544_at	307482	Fchsd1_pr	FCH and double SH3 domains 1 (pred	735.1291	736.6599	455.158
1388987_at	307643	RGD13052	similar to expressed sequence AA9604	734.9211	798.0139	406.1313
1377323_at	311341	Pla2g4b_p	phospholipase A2, group IVB (cytosoli	734.7078	29.51379	75.19348
1388173_at	116695	Kcnd1	potassium voltage-gated channel, Sha	734.6059	787.5598	788.763
1380460_at	311429	RGD13112	similar to RIKEN cDNA 4931426K16 g	733.7645	306.8534	339.2584
1370522_at	24953	Gcgr	glucagon receptor	733.0294	1322.695	30.92501
1372898_at	497972	RGD15662	similar to 2700008B19Rik protein (prec	732.6713	682.4258	459.4842
1394411_at	500724	RGD15606	similar to novel protein (predicted)	732.1226	421.3496	303.7367
1376802_at	304547	Tchp_pred	trichoplein, keratin filament binding (pr	732.0962	1168.891	537.9836
1392017_at	311030	Stam2	signal transducing adaptor molecule (S	732.0459	142.8455	342.5066

1368058_at	64196	Safb	scaffold attachment factor B	731.2337	828.8753	421.583
1370750_a_at	25663	Il1r1	interleukin 1 receptor, type I	731.0771	1017.2	179.3031
1376704_a_at	309259	Ndnl2	necdin-like 2	731.0731	1152.997	1917.246
1388494_at	306628	Col4a2_pre	procollagen, type IV, alpha 2 (predicte	730.8565	348.6749	834.2383
1391857_at	363554	RGD15617	similar to hypothetical protein 4732467	730.8101	244.0459	589.1111
1381006_at	58947	Hgfac	hepatocyte growth factor activator	730.2303	789.5195	4666.188
1382672_a_at	301737	Flk	NA	729.9985	344.9599	561.5989
1396492_at	362966	Nfam1_pre	NFAT activating protein with ITAM mot	729.8802	642.0095	26.72481
1393377_at	500826	Galnt4	UDP-N-acetyl-alpha-D-galactosamine:	729.5966	1978.761	496.665
1390865_at	681395	LOC68139	NA	729.174	953.8566	267.1671
1389281_at	300439	RGD15635	similar to ankyrin repeat domain 25 (pr	729.097	578.556	852.5878
1389210_at	306071	Lcp1	lymphocyte cytosolic protein 1	729.0074	1837.249	661.334
1389212_at	58822	Csnk1e	casein kinase 1, epsilon	728.8728	729.4123	1015.451
1398459_at	25398	Cacna1a	calcium channel, voltage-dependent, F	728.4889	359.1932	366.283
1368584_a_at	116657	Cplx2	complexin 2	728.3761	1470.017	329.8079
1396561_x_at	363464	Piga_mapp	phosphatidylinositol glycan, class A (m	728.0014	75.93134	27.56876
1379367_at	362676	RGD13082	similar to RIKEN cDNA 2810405K02 (p	727.9557	630.7589	388.0878
1387514_at	171057	Cbwd1	COBW domain containing 1	727.8495	2112.315	425.2041
1397350_at	498029	LOC49802	similar to RIKEN cDNA A730011L01 g	727.642	491.9234	503.6792
1369270_at	84385	Nr1i2	nuclear receptor subfamily 1, group I, r	726.6848	349.2198	432.7063
1396639_at	499903	LOC49990	NA	726.2322	435.9311	480.772
1375907_at	294512	Ascc1	activating signal cointegrator 1 comple	726.1173	1410.845	559.3765
1390042_at	362334	MGC10949	similar to 1110007F12Rik protein	724.8079	48.18052	5715.311
1373274_at	297437	Txnrd3_pre	thioredoxin reductase 3 (predicted)	724.8055	995.9172	1096.663
1390804_at	499874	RGD15615	similar to mKIAA0518 protein (predicte	724.6635	574.1134	2816.982
1379621_at	499108	MGC10897	similar to replication protein-binding tra	723.6082	569.2404	508.5725
1389485_at	314859	LOC31485	similar to transformed mouse 3T3 cell	723.5036	2403.103	1454.953
1392816_at	297607	RGD13111	similar to DNA segment, Chr 6, Wayne	723.0645	2643.62	1048.363
1368327_at	171443	Slc12a9	solute carrier family 12 (potassium/chlo	722.9675	881.7955	286.9327
1393758_at	310314	Ttc14_prec	tetratricopeptide repeat domain 14 (pre	722.3114	365.1318	418.2446
1390120_a_at	309626	Ring1	ring finger protein 1	721.672	527.6841	1093.891
1392485_at	313551	RGD15648	similar to chromosome 1 open reading	721.1554	631.761	115.0353
1379299_at	310071	Ercc8_prec	excision repair cross-complementing ro	721.0485	2126.694	1062.937
1375678_at	501747	RGD15629	similar to hypothetical protein DKFZp4	720.9987	772.3046	880.7436
1372766_at	309650	Cpne5_pre	copine V (predicted)	720.861	653.7146	536.9925
1391685_at	361478	Pnldc1	poly(A)-specific ribonuclease (PARN)-l	720.8589	338.9596	303.1213
1374856_at	363001	RGD13064	similar to RIKEN cDNA 4930556P03 (p	720.6952	911.8158	513.2638
1383440_at	311218	RGD13093	similar to 2610203E10Rik protein (pred	720.6665	1505.369	427.3349
1381280_at	305248	RGD13108	similar to methylenetetrahydrofolate de	719.8339	131.5219	1256.745
1374221_at	353307	Slc29a3	solute carrier family 29 (nucleoside tra	719.181	379.5963	255.9948
1382484_at	307109	Taf3_predi	TAF3 RNA polymerase II, TATA box b	719.0592	508.9322	541.2913
1367507_at	293703	Nudt22	nudix (nucleoside diphosphate linked r	718.6163	1066.996	307.0178
1374891_at	306542	Brf2	BRF2, subunit of RNA polymerase III t	718.5731	1279.991	577.2727
1390232_at	309145	RGD13119	similar to RIKEN cDNA 1810055G02	718.379	1153.681	195.0077
1392974_at	294568	Wasf1	WAS protein family, member 1	718.1707	2066.503	2903.978
1390172_at	361272	Dhtkd1	dehydrogenase E1 and transketolase d	717.9853	372.6473	489.0506
1373772_at	84350	Dnmt1	DNA (cytosine-5-)-methyltransferase 1	717.8829	3292.998	1023.472
1397859_x_at	364376	NA	NA	717.593	25.77674	1047.761
1374062_x_at	59075	Gprk5	G protein-coupled receptor kinase 5	717.4918	923.4927	1748.53
1388419_at	286919	Cdk5rap2	CDK5 regulatory subunit associated pr	716.5316	968.987	481.2803
1389399_at	292100	RGD15622	similar to RIKEN cDNA 0610039J04	716.0263	713.1237	598.8116

1377383_at	290212	Efs_predict	embryonal Fyn-associated substrate (p	715.9057	68.81965	120.4395
1392654_at	361473	LOC36147	NA	715.8144	909.9798	1544.549
1373994_at	500247	RGD1565	similar to RIKEN cDNA 2010301N04 (p	715.2828	105.4698	594.7511
1375903_a_at	362989	Yaf2_predi	YY1 associated factor 2 (predicted)	714.8175	2141.815	1182.211
1380524_at	407760	Kb26	type II keratin Kb26	714.6329	113.9886	170.0437
1399073_at	293705	Otub1_pre	OTU domain, ubiquitin aldehyde bindir	713.437	3518.317	639.418
1378709_at	689991	LOC68999	NA	713.4342	61.40954	172.4589
1374409_at	309194	Taf6l_pred	TAF6-like RNA polymerase II, p300/CI	713.2837	941.3951	144.1867
1398297_at	60352	Mapk12	mitogen-activated protein kinase 12	712.4694	65.44135	247.1076
1382230_at	305096	Klhdc8a	kelch domain containing 8A	712.2812	4004.518	445.5647
1392617_at	362455	RGD13061	similar to hypothetical protein DKFZp7	712.2045	450.5753	166.8778
1369851_at	81518	Mef2d	myocyte enhancer factor 2D	711.8168	315.8328	309.3756
1376828_at	312790	Gprc5a	G protein-coupled receptor, family C, g	711.4042	209.3106	725.567
1387028_a_at	25261	Id1	inhibitor of DNA binding 1	711.256	979.8842	2152.342
1382771_at	296371	Pltp_predic	phospholipid transfer protein (predicte	710.751	414.7592	902.1137
1373715_at	304396	LOC30439	similar to hypothetical protein DKFZp4	709.5531	1157.019	743.1903
1373443_a_at	294679	Ankra2	ankyrin repeat, family A (RFXANK-like	708.643	1217.678	1534.381
1383248_at	246248	Fmo5	flavin containing monooxygenase 5	708.3172	267.2354	1141.619
1379831_at	360574	RGD1311C	similar to multiple hat domains (predict	708.1974	886.4272	430.06
1368196_at	25680	Clps	colipase, pancreatic	708.1431	34.45741	4012.262
1383013_at	499171	RGD1565C	similar to BTEB3 protein (predicted)	707.9888	444.5257	328.2546
1388927_at	300062	Rabl4_pred	RAB, member of RAS oncogene family	707.336	2143.748	477.8121
1393100_at	299050	RGD1305C	similar to 1110008L16Rik protein (pred	707.2353	517.1907	729.9252
1395990_at	361652	RGD15656	similar to Snf2-related CBP activator p	706.5503	337.1185	419.5609
1387046_at	116475	Slb	selective LIM binding factor, rat homol	706.3472	978.1387	1344.621
1381468_at	361394	Lrrc36	LRRC36 homolog (human)	706.2973	32.18847	646.1821
1393654_at	367090	Neil1	nei endonuclease VIII-like 1 (E. coli)	706.1898	208.7834	550.8871
1384943_at	360692	RGD15638	similar to DNA segment, Chr 16, ERAT	706.0728	715.3526	1244.362
1387412_at	116723	Pip5k2a	phosphatidylinositol-4-phosphate 5-kin	705.5585	458.4558	342.3892
1376139_at	309280	Plxna3_ma	plexin A3 (mapped)	705.458	488.0477	518.6905
1398426_at	498309	RGD15641	similar to RIKEN cDNA B130052G07 (	705.395	225.8598	205.4353
1379380_at	294981	Spry1_pred	sprouty homolog 1 (Drosophila) (predic	705.3319	319.0594	1161.297
1374256_at	291999	Wwp2_pre	WW domain containing E3 ubiquitin pr	704.9967	533.7364	270.8864
1381316_at	361243	Cdkal1_pre	CDK5 regulatory subunit associated pr	704.788	1450.175	1039.823
1377730_at	299207	RGD13107	similar to HSPC288 (predicted)	704.2295	678.8942	287.8033
1383449_at	361786	NA	NA	704.1846	547.8449	301.3921
1396254_at	83724	Ncoa2	nuclear receptor coactivator 2	704.1367	338.3307	661.9619
1392444_at	307859	RGD15611	similar to N-acetylglucosamine 6-O-su	704.0905	123.8196	181.1544
1367612_at	171341	Mgst1	microsomal glutathione S-transferase	703.9766	24024.62	646.3622
1369021_at	25593	Hcrtr1	hypocretin (orexin) receptor 1	703.8161	113.1022	263.9984
1383136_a_at	363459	NA	NA	702.8524	852.1043	2411.573
1374899_at	290877	RGD1566C	similar to hypothetical protein MGC205	702.4915	25.27756	43.28032
1383122_at	373545	Prkag2	protein kinase, AMP-activated, gamma	702.2863	899.2487	442.035
1393801_at	367808	LOC36780	similar to Sid3177p	701.9561	538.6117	669.2492
1387435_at	25547	St8sia3	ST8 alpha-N-acetyl-neuraminide alpha	701.7013	3129.634	744.2099
1374444_at	316009	Plxnb1_pre	plexin B1 (predicted)	701.5927	669.0923	930.8899
1387607_at	78964	Npffr2	neuropeptide FF receptor 2	701.3961	62.64723	227.1115
1392446_at	290692	Klhl2_pred	kelch-like 2, Mayven (Drosophila) (pre	701.115	2555.723	2015.198
1374925_at	314910	Nab2	Ngfi-A binding protein 2	700.5883	324.1603	1881.75
1369323_at	56766	Leprot	leptin receptor overlapping transcript	700.4072	1315.988	170.7978
1398435_at	310732	Slc22a15_	solute carrier family 22 (organic cation	700.1337	1209.145	626.1315

1393018_at	502072	RGD1565	similar to RING finger protein 33 (pred	700.0829	239.4734	254.0938
1382290_at	361664	RGD13063	similar to CG9643-PA (predicted)	700.0057	860.3612	1174.691
1396407_at	361438	Gas8	growth arrest specific 8	699.3886	420.0475	963.9677
1392299_at	192363	Capon	C-terminal PDZ domain ligand of neur	699.3254	264.688	715.8668
1372107_at	25177	Fhl1	four and a half LIM domains 1	699.007	1682.448	1070.796
1388177_at	25113	Ddn	dendrin	698.7546	451.0093	393.6091
1384837_at	29187	Cd69	CD69 antigen	698.7277	16.83568	39.2629
1372970_at	292735	Shkbp1_pr	Sh3kbp1 binding protein 1 (predicted)	698.7259	495.5352	445.0006
1368824_at	25687	Cald1	caldesmon 1	698.3982	311.7467	681.3284
1378104_at	296200	RGD13074	similar to RIKEN cDNA 8430406I07	698.3465	1634.415	853.369
1375667_at	311692	RGD13119	similar to Protein C20orf177 (predicted	698.3365	730.8735	70.17958
1382919_at	306009	Slc39a14_	solute carrier family 39 (zinc transporte	698.202	691.6195	210.4341
1393510_at	500865	RGD15643	similar to RIKEN cDNA 5730410E15 g	697.7421	492.4084	3050.405
1391239_at	303742	RGD13069	similar to hypothetical protein FLJ2217	697.5977	593.2973	566.9379
1367696_at	114709	Ifitm2	interferon induced transmembrane pro	697.4667	2267.039	1605.528
1375464_at	308556	RGD15649	RGD1564982 (predicted)	697.3545	401.0485	936.297
1367867_at	27100	Gfer	growth factor, erv1 homolog (S. cerevi	697.121	1269.104	501.3307
1385010_at	450227	Ka28	type I hair keratin KA28	696.7876	389.4166	469.8694
1368485_at	79253	Avil	advillin	696.4775	165.6362	681.6073
1384457_at	498713	NA	NA	696.313	1273.413	705.1056
1375814_at	360773	Unc84a	unc-84 homolog A (C. elegans)	696.2441	254.3337	851.9863
1368396_at	24254	Cel	carboxyl ester lipase	695.9672	88.93201	5045.8
1393554_at	361709	RGD15626	similar to hypothetical protein DKFZp7	695.8051	529.5927	417.45
1383853_at	304775	Dyrk3	dual-specificity tyrosine-(Y)-phosphory	695.5985	428.7161	3174.364
1373774_at	291713	Rit2	Ras-like without CAAX 2	695.0723	2074.756	2963.417
1374784_at	291355	Prtfdc1_pre	phosphoribosyl transferase domain co	695.0035	13293.31	4932.874
1389503_at	288748	NA	NA	694.9457	60.76922	1238.539
1372805_at	363015	RGD13104	LOC363015 (predicted)	694.6094	1715.488	724.5772
1371866_at	360570	LOC36057	similar to myosin XVIIIa	694.3984	565.9602	462.2176
1380687_at	316064	Oxsr1_pre	oxidative-stress responsive 1 (predicte	693.8074	109.2551	503.7044
1369928_at	29437	Acta1	actin, alpha 1, skeletal muscle	693.6156	989.2454	533.8635
1368795_at	65276	Nmur1	neuromedin U receptor 1	693.2382	317.1262	152.272
1392273_at	361169	Letm2	leucine zipper-EF-hand containing trar	692.7084	322.6604	2748.37
1369657_at	24269	Cpa1	carboxypeptidase A1	692.6756	125.2978	5445.183
1390486_at	303394	Usp32_pre	ubiquitin specific protease 32 (predicte	692.6612	355.5349	263.2453
1393085_at	363219	RGD13077	similar to hypothetical protein BC0184	692.6226	929.9881	746.6841
1372599_at	295037	Mgst2_pre	microsomal glutathione S-transferase	692.5265	349.5699	503.3754
1372606_at	311743	RGD15613	similar to MLTK-beta (predicted)	692.3286	2011.267	1010.953
1369768_at	201097	H1f4	NA	691.6579	163.0143	207.3984
1392491_at	293871	RGD13118	similar to RIKEN cDNA 2410127L17	691.456	1185.87	1304.777
1398131_at	192179	Gpm6b	glycoprotein m6b	691.1029	205.2715	590.9648
1378726_at	288587	Alkbh4_pre	alkB, alkylation repair homolog 4 (E. co	691.0637	723.613	262.5894
1374747_at	362316	Pftk1_pred	PFTAIRE protein kinase 1 (predicted)	690.5536	654.9063	215.7197
1393940_at	366954	Znf251_pre	zinc finger protein 251 (predicted)	690.45	218.5719	387.3197
1378319_at	362289	RGD15627	similar to Lck-interacting transmembra	690.4143	478.7198	408.7965
1373631_at	313644	Rap1ga1	RAP1, GTPase activating protein 1	690.2838	2336.67	1265.063
1393175_at	499137	RGD15620	similar to RIKEN cDNA 2810426N06 (	690.116	597.645	2109.868
1384937_at	288489	RGD15628	similar to RB-associated KRAB repres	690.101	930.8203	797.8133
1373850_at	313033	Xkr8	X Kell blood group precursor related fa	689.9252	329.1393	182.7961
1374693_at	315760	Parp16	poly (ADP-ribose) polymerase family, r	689.7384	208.2418	372.2706
1395781_at	298506	Mycl1_ma	v-myc myelocytomatosis viral oncoger	689.527	1965.69	635.6831

1387129_at	84495	Xrcc1	X-ray repair complementing defective r	689.3429	1117.763	1258.645
1384890_at	303547	Ezh1_pred	enhancer of zeste homolog 1 (Drosoph	689.3112	498.1104	160.6121
1368065_at	83823	Gipc1	GIPC PDZ domain containing family, m	689.2567	472.5922	208.7941
1387438_at	114097	Ltc4s	leukotriene C4 synthase	688.9618	523.4502	420.5984
1368902_at	29433	Pak3	p21 (CDKN1A)-activated kinase 3	688.6646	2669.23	1048.144
1378218_at	315309	Tcfcp2_pre	transcription factor CP2 (predicted)	688.5698	582.2121	504.5932
1379439_at	363035	Btbd15	BTB (POZ) domain containing 15	688.1813	773.7486	2032.71
1371242_at	60590	Grm8	glutamate receptor, metabotropic 8	687.5518	267.4282	308.8902
1367647_at	24648	Serpina1	serine (or cysteine) proteinase inhibito	687.1954	129.9688	5661.705
1389396_at	54283	Pfkfb4	6-phosphofructo-2-kinase/fructose-2,6-	687.1	843.8174	533.0606
1388984_at	362858	RGD15653	similar to RNA polymerase III subunit F	686.8496	861.7216	292.638
1373236_at	363069	Ppcdc_pre	phosphopantothenoylcysteine decarbo	686.7271	267.738	607.7781
1383372_at	58949	Ptafr	platelet-activating factor receptor	686.2758	423.5367	661.6685
1375351_at	364534	LOC36453	similar to RIKEN cDNA 1210002E11	686.211	1001.654	478.8392
1375216_at	308417	Pvrl2	poliovirus receptor-related 2 (herpesvi	686.0184	1304.337	680.1025
1398274_at	114210	Spata2	spermatogenesis associated 2	685.7794	470.2432	290.9814
1379868_at	310644	Pbxip1	pre-B-cell leukemia transcription factor	685.7543	10.4393	659.8221
1392708_at	291527	Stard6	STAR-related lipid transfer (START) do	685.5235	1226.693	492.2228
1387233_at	29540	Hsd17b7	hydroxysteroid (17-beta) dehydrogena	684.3494	583.4794	377.3796
1391616_at	246138	Ly6b	lymphocyte antigen 6 complex, locus B	684.1352	62.70049	326.7253
1368652_at	58918	Casp9	caspase 9	683.7336	662.7929	358.2441
1391310_at	308053	RGD13075	similar to intracellular protein transport	683.664	346.9799	305.336
1377600_at	502764	RGD15660	similar to KIAA1285 protein (predicted)	683.5775	921.3547	451.1136
1368119_at	171088	Pib5pa	phosphatidylinositol (4,5) bisphosphate	683.2493	1031.958	557.8822
1373683_at	25150	Fyn	fyn proto-oncogene	682.8341	1980.289	1670.407
1377183_at	502206	NA	NA	682.6843	584.1166	895.2848
1383314_at	500578	RGD15654	similar to BC003277 protein (predicted	681.9371	471.3981	368.7415
1386550_at	309031	Zfpn1a5_p	zinc finger protein, subfamily 1A, 5 (pre	681.8883	1668.425	1525.773
1372751_at	500936	NA	NA	681.2077	671.2942	385.1111
1385614_at	316620	Mlph	melanophilin	680.9752	259.5051	254.8636
1397611_at	501124	NA	NA	680.6096	236.055	220.058
1396987_at	311913	RGD13079	similar to RIKEN cDNA 6030446I19 ge	679.6666	164.7934	614.9307
1376502_at	298851	RGD13092	similar to putative protein, with at least	679.427	650.1165	371.2184
1378902_at	304423	Rsafd1_pre	radical S-adenosyl methionine and flav	678.8165	680.4124	362.096
1377209_at	293023	Klhl25	kelch-like 25 (Drosophila)	678.5499	1021.65	630.9285
1372874_at	305002	Sdccag8	serologically defined colon cancer anti	678.3913	1865.087	1008.785
1388493_at	362374	RGD13064	similar to Expressed sequence AW146	678.2941	1689.594	2064.968
1375063_at	287059	RGD15651	similar to MGC45438 protein (predicte	678.0935	188.4409	46.9986
1380118_at	315646	RGD15622	similar to hypothetical gene supported	678.0178	417.924	1219.312
1393306_at	64624	Cul5	cullin 5	677.8208	163.6334	525.4505
1382528_at	360947	RGD13110	similar to RIKEN cDNA 4933428G09 (	677.369	1566.81	935.4904
1387559_at	170796	Grin3b	glutamate receptor, ionotropic, N-meth	677.044	312.2253	170.5784
1389199_at	315891	RGD13090	similar to Ab2-095	676.2578	608.0085	2338.576
1389102_at	309081	RGD15660	similar to Dock1 protein (predicted)	676.1069	68.33708	3355.17
1376090_at	313851	Thumpd2	THUMP domain containing 2	675.6169	708.6724	720.7605
1394935_at	313024	Wasf2	WAS protein family, member 2	675.2993	23.99781	125.0145
1376293_at	303702	RGD13108	similar to RIKEN cDNA D230014K01 (	675.2989	293.8195	271.0757
1383120_at	365619	Arsk	arylsulfatase K	675.1776	925.387	1285.337
1386721_at	305687	Zfp503_pre	zinc finger protein 503 (predicted)	675.1106	1074.111	3446.069
1390861_at	362845	LOC36284	NA	674.7153	298.1616	429.6327
1381449_s_at	24827	Tgfa	transforming growth factor alpha	674.6692	1778.875	319.5155

1382552_at	307067	RGD15601	similar to mKIAA0934 protein (predicte	674.6046	702.9674	591.5365
1368088_at	140908	Cdk5	cyclin-dependent kinase 5	674.5307	3738.953	651.7957
1385204_at	499807	RGD15655	similar to RIKEN cDNA 4833418A01 (p	674.2892	259.7294	276.662
1389873_at	282817	Pycard	PYD and CARD domain containing	674.2437	1410.866	423.2667
1397173_at	317420	RGD15631	similar to Serine/threonine-protein kina	674.2028	2917.8	12457.3
1384827_at	289757	Polm	polymerase (DNA directed), mu	674.13	463.4394	498.0106
1386940_at	29543	Timp2	tissue inhibitor of metalloproteinase 2	674.0295	2015.709	3682.988
1390695_at	498265	LOC49826	similar to hypothetical protein FLJ1070	674.0283	459.6581	1715.891
1377918_at	300748	RGD15594	similar to Stomatin-like 1 (predicted)	673.9636	422.7795	202.4068
1372521_at	303553	Rnd2	Rho family GTPase 2	673.957	1035.442	968.331
1398068_at	314306	RGD13061	similar to transcriptional regulating pro	673.3396	224.5244	785.9333
1389037_at	499652	Rit1_predic	Ras-like without CAAX 1 (predicted)	673.3344	982.2029	464.7391
1376941_at	360796	Wbscr16_p	Williams-Beuren syndrome chromosom	673.1329	1291.789	527.778
1392655_at	363269	LOC36326	similar to Nuclear autoantigen Sp-100	673.1258	60.76067	1572.2
1381229_at	313678	Prdm2_ma	PR domain containing 2, with ZNF don	672.8061	507.3246	3677.9
1374910_at	83465	Celsr2	cadherin EGF LAG seven-pass G-type	672.6066	827.0777	1200.849
1368027_at	24886	Tbxas1	thromboxane A synthase 1	672.4611	632.8451	318.4358
1390644_at	307667	Hmgb2l1_p	high mobility group box 2-like 1 (predic	671.3223	182.8322	245.3248
1390036_at	303772	Slc16a6	solute carrier family 16 (monocarboxyl	671.1234	371.4127	619.0483
1387052_at	81670	Gpt1	glutamic pyruvic transaminase 1, solub	670.7711	186.1744	1169.364
1374720_at	301062	Endogl1_p	endonuclease G-like 1 (predicted)	670.6994	709.8397	516.6167
1391675_at	360867	RGD13097	similar to RIKEN cDNA 4930455F23	670.3271	538.6087	755.676
1369084_a_at	29884	Bok	Bcl-2-related ovarian killer protein	669.8825	733.9473	528.0061
1372868_at	304884	Tor3a	torsin family 3, member A	669.4411	332.9494	381.3024
1379812_at	300691	Nnmt_pred	nicotinamide N-methyltransferase (pre	669.4044	1959.669	289.5405
1396100_at	310005	Aggf1	angiogenic factor with G patch and FH	669.2671	1001.47	380.4832
1378864_at	312707	Lrrc23	leucine rich repeat containing 23	668.9397	917.3563	3312.452
1367888_at	93662	Pcdh21	MT-protocadherin	668.8493	2909.078	543.8411
1376135_at	304919	Dars2	aspartyl-tRNA synthetase 2 (mitochon	668.8217	648.3911	641.3374
1378073_at	50688	Cacnb1	calcium channel, voltage-dependent, b	668.517	550.3124	541.0188
1379805_at	362861	Slc41a2_pr	solute carrier family 41, member 2 (pre	667.6327	486.8384	189.9014
1378567_at	314640	Tjp3_predic	tight junction protein 3 (predicted)	667.548	441.7471	222.4892
1383083_at	292064	Banp_pred	Btg3 associated nuclear protein (predic	667.0904	71.34976	1273.146
1388425_at	315594	RGD13058	similar to RIKEN cDNA D130038B21	667.018	194.7512	469.9222
1389725_at	293688	Tm7sf2	transmembrane 7 superfamily member	666.9869	8585.165	525.4092
1391184_at	301121	LOC30112	NA	666.8646	508.1976	1548.929
1395473_at	60449	Gnb3	guanine nucleotide binding protein, be	666.444	481.4152	780.0646
1395268_at	297930	Wwp1	WW domain containing E3 ubiquitin pr	666.4117	706.1556	171.014
1368286_at	85256	Slc2a8	solute carrier family 2, (facilitated gluc	665.0003	1116.045	830.1306
1374806_at	313017	Sfn_predic	stratifin (predicted)	664.5636	26.9464	244.2421
1393321_at	314190	Klhdc1_pre	kelch domain containing 1 (predicted)	664.2715	359.824	460.2724
1379351_at	291034	RGD13076	similar to hypothetical protein MDS025	663.6755	834.5959	519.6405
1396480_at	300993	Hyal3	hyaluronoglucosaminidase 3	663.5263	171.909	899.1988
1382388_at	308593	RGD13050	similar to hypothetical protein MGC276	662.9425	739.7288	296.3723
1368320_at	24586	Ncam1	neural cell adhesion molecule 1	662.9028	1638.777	3104.726
1376455_at	500899	MGC11447	similar to 2310010G13Rik protein	662.6793	305.24	144.6803
1376581_at	366619	RGD13091	similar to hypothetical protein MGC991	662.5649	441.1501	677.3619
1388919_at	308108	Znf541_pre	zinc finger protein 541 (predicted)	662.4818	1880.181	1960.812
1378447_at	303403	Thrap1_pre	thyroid hormone receptor associated p	662.3305	957.3298	2999.175
1378605_at	309106	Sigirr	single immunoglobulin and toll-interleu	662.0251	767.829	311.777
1372205_at	305471	Zfp278	zinc finger protein 278	661.7639	1611.375	1958.377

1374306_at	362613	Zdhhc18	zinc finger, DHHC domain containing 1	661.7012	395.0213	77.40847
1369959_at	29344	Zfp36l1	zinc finger protein 36, C3H type-like 1	661.6021	219.4881	3305.524
1367574_at	81818	Vim	vimentin	661.549	84.29742	2807.519
1370611_at	25243	Arnt2	aryl hydrocarbon receptor nuclear tran	661.4843	280.7707	270.3112
1388459_at	85251	Col18a1	procollagen, type XVIII, alpha 1	660.7328	34.3056	176.4666
1395850_at	315554	Pus3_pred	pseudouridylate synthase 3 (predicted	660.5713	385.9402	208.0625
1371615_at	252900	Dgat2	diacylglycerol O-acyltransferase homo	660.448	1014.568	1256.569
1380655_at	362787	Rage	renal tumor antigen	660.3892	626.2745	400.516
1369161_at	24891	Abcb4	ATP-binding cassette, sub-family B (M	660.3695	1020.208	689.3257
1381472_at	315023	Slc25a32_	solute carrier family 25, member 32 (p	660.1095	223.307	483.3013
1377062_at	311573	RGD15622	similar to centrosomal Nek2-associate	659.6736	908.7477	762.3721
1369500_at	59324	Kcnk1	potassium channel, subfamily K, meml	659.3341	478.0788	510.5773
1379887_at	312474	RGD13047	similar to chromosome 2 open reading	659.032	674.0332	364.4235
1369965_at	81685	Mlh1	mutL homolog 1 (E. coli)	658.8675	928.6776	1404.863
1395671_at	288604	RGD1561S	similar to Williams-Beuren syndrome c	658.2123	308.5919	125.7964
1393142_at	367153	LOC36715	similar to p10-binding protein	657.8737	1124.56	4381.559
1379452_at	499156	RGD15631	similar to growth arrest-specific protei	657.7769	328.301	815.1839
1381480_at	287545	Sarm1_pre	sterile alpha and TIR motif containing	657.3994	1253.039	889.5943
1385504_at	282844	Rffl	ring finger and FYVE like domain cont	657.3094	375.7958	168.5208
1387241_at	64443	Gpr88	G-protein coupled receptor 88	657.2998	31.42073	97.68171
1385862_at	301579	Armc9_pre	armadillo repeat containing 9 (predicte	657.225	459.6144	949.5002
1367679_at	25599	Cd74	CD74 antigen (invariant polypeptide of	657.1961	112.6576	1849.979
1381184_at	171563	Nav2	neuron navigator 2	656.976	25.42821	678.9751
1368106_at	83722	Plk2	polo-like kinase 2 (Drosophila)	656.6122	383.4811	4891.69
1372057_at	619579	Nrbp	NA	656.3611	949.1898	450.9463
1395851_at	316088	Kbtbd5_pre	kelch repeat and BTB (POZ) domain c	656.3541	64.2087	360.0211
1378034_at	366856	Mterfd3	MTERF domain containing 3	655.8861	875.9463	1164.037
1382930_at	304486	RGD15662	similar to hypothetical protein FLJ2112	655.6121	428.6437	370.4225
1392715_at	291567	Ppargc1b	peroxisome proliferative activated rece	655.2809	106.1284	318.0989
1384878_at	291318	Nmt2	N-myristoyltransferase 2	655.1416	1131.397	405.9716
1377044_at	171438	Crtac1	cartilage acidic protein 1	653.979	2293.445	2928.828
1392558_at	500676	NA	NA	653.6137	333.0926	775.3829
1370020_at	170943	Slc25a10	solute carrier family 25 (mitochondrial	653.5368	3226.029	357.1002
1391619_at	501623	NA	NA	653.3024	1843.688	455.927
1369433_at	60347	Ip63	IP63 protein	653.1231	1984.485	1430.804
1377541_at	500726	LOC50072	NA	652.4369	233.2016	518.3929
1390649_at	366568	Slc30a3	solute carrier family 30 (zinc transporte	652.0965	553.4208	419.4253
1379027_at	303039	RGD13083	similar to KIAA0869 protein (predicted)	651.7408	633.0514	1041.68
1368346_at	171079	B3galt4	UDP-Gal:betaGlcNAc beta 1,3-galacto	651.7213	382.9484	53.04009
1395405_at	361540	Snx26_pre	sorting nexin 26 (predicted)	651.6752	1972.77	883.4993
1387357_at	170898	Tmlhe	trimethyllysine hydroxylase, epsilon	651.6533	2048.565	1010.234
1377178_at	288664	RGD13106	similar to hypothetical protein FLJ3235	651.5033	487.2127	192.0873
1391585_at	59319	Nyw1	ischemia related factor NYW-1	651.3319	2121.98	618.263
1377572_at	361091	RGD15635	similar to UDP-glucose ceramide gluco	651.064	73.27681	2450.749
1386277_at	301000	Ube1l_prec	ubiquitin-activating enzyme E1-like (pr	650.9592	406.751	387.4581
1373793_at	304979	Igsf8	immunoglobulin superfamily, member	650.9341	836.5341	707.1041
1388099_a_at	85423	Tfpt	TCF3 (E2A) fusion partner	650.8248	1264.585	487.1337
1393204_at	361778	Sfxn4_prec	sideroflexin 4 (predicted)	650.4995	326.3082	602.6305
1393247_at	367033	RGD15658	similar to zinc finger protein 560 (predi	650.3766	1502.259	2101.561
1394699_at	307376	RGD15646	similar to transcription factor ONECUT	649.8327	2595.342	208.6138
1392626_at	287101	Pkmyt1_pr	protein kinase, membrane associated	649.4407	1121.894	512.5645

1389550_at	116743	Sh3gl2	SH3-domain GRB2-like 2	649.2817	994.0333	3119.344
1376633_at	503166	NA	NA	648.7026	6047.576	654.9477
1389203_at	287306	RGD1307C	hypothetical LOC287306 (predicted)	648.5275	1078.875	564.7655
1372248_at	294518	Sesn1_pre	sestrin 1 (predicted)	648.332	1517.727	4286.456
1392131_at	499933	LOC49993	similar to RIKEN cDNA 1700022L09	648.0606	1860.193	440.6468
1375495_at	503009	LOC50300	NA	647.8593	1208.776	269.8334
1383867_at	310261	Eif5a2_pre	eukaryotic translation initiation factor 5	647.1465	2794.348	621.215
1379787_at	311864	Uck1_pred	uridine-cytidine kinase 1 (predicted)	646.7655	495.5272	337.8418
1374948_at	287722	Tmem106a	transmembrane protein 106A	646.4697	73.11239	549.7037
1376932_at	499747	RGD15608	similar to RIKEN cDNA 2310002J15 (p	646.3121	506.5151	196.0073
1379375_at	25266	Pdgfa	platelet derived growth factor, alpha	646.2356	605.8795	2288.026
1372410_at	315114	C1qtnf6	C1q and tumor necrosis factor related	646.1788	709.7014	481.0547
1389129_at	303002	RGD13089	similar to mKIAA0665 protein	646.1464	760.4631	938.8604
1372378_at	367307	RGD15601	similar to RIKEN cDNA 4930429A22 (p	645.5886	469.0364	286.4203
1374006_at	541589	Kat3	kynurenine aminotransferase III	645.4608	43.54433	443.2082
1376130_a_at	362715	Dtnb	dystrobrevin, beta	645.3465	880.7063	687.8099
1368475_at	29755	Colq	collagen-like tail subunit (single strand	645.294	340.2825	329.2506
1380872_at	366333	RGD15624	similar to hypothetical protein MGC281	645.1452	1016.793	2531.189
1377697_at	305338	RGD15624	similar to amyloid beta (A4) precursor	644.9615	532.8909	799.4029
1376900_at	259271	Aptx	aprataxin	644.8035	801.3137	289.2572
1375102_at	364945	LOC36494	NA	644.6652	109.1057	369.7044
1389493_at	297432	Abtb1	ankyrin repeat and BTB (POZ) domain	644.3654	857.5721	705.243
1380030_at	298546	Znf593_pre	zinc finger protein 593 (predicted)	644.0874	334.2171	519.1626
1391169_at	500921	RGD15625	similar to RIKEN cDNA B930062P21 g	644.0595	2101.378	2407.096
1387444_at	171125	Ptprh	protein tyrosine phosphatase, receptor	643.5798	239.6455	210.8031
1376639_at	314613	Rnf126	ring finger protein 126	643.0945	1008.12	184.9228
1383312_at	317377	RGD15615	similar to JM11 protein (predicted)	642.8418	441.22	673.1965
1370882_at	294273	Hla-dmb	major histocompatibility complex, class	642.8057	107.8837	517.7317
1384602_at	311857	Gpr107_pr	G protein-coupled receptor 107 (predic	642.6344	180.7693	214.8676
1376099_at	85490	Col5a1	procollagen, type V, alpha 1	641.8661	195.1599	651.1976
1370346_at	25203	Ccnb1	cyclin B1	641.8481	14908.74	287.4475
1376253_at	300025	Ly6h_predi	lymphocyte antigen 6 complex, locus H	641.7711	2232.674	266.6766
1368784_at	170912	Acf	apobec-1 complementation factor	641.4994	537.6486	83.31836
1387822_at	81662	Gna11	guanine nucleotide binding protein, alp	641.2898	428.7315	244.0231
1396096_at	367252	Mgat4a	mannoside acetylglucosaminyltransfer	641.2451	622.208	142.9591
1385002_at	315756	RGD15626	similar to c-myc promoter binding prote	641.0351	539.8736	1574.849
1376904_at	309158	RGD13106	similar to hypothetical protein MGC334	639.386	759.3742	678.5546
1384202_at	288689	RGD15663	similar to Tescalcin (predicted)	639.26	865.4339	87.24159
1381052_at	288417	RGD15661	similar to CG016 (predicted)	639.2329	170.5002	269.259
1389368_at	308113	Cnksr3	Cnksr family member 3	639.0915	474.9111	5779.658
1373445_at	361221	Nol8_predi	nucleolar protein 8 (predicted)	638.8771	770.3302	834.5561
1382471_at	304735	RGD13103	similar to hypothetical protein MGC109	638.6942	1035.386	300.5584
1378298_at	298591	RGD13101	hypothetical LOC298591 (predicted)	638.4441	246.4459	3227.696
1368646_at	50658	Mapk9	mitogen-activated protein kinase 9	637.9915	1040.773	81.70196
1389268_at	361767	LOC36176	NA	637.9866	999.5183	282.7219
1399083_at	360831	Wbscr21	Williams Beuren syndrome chromosom	637.8503	1554.262	301.7391
1377603_at	361328	Snx24	sorting nexing 24	637.7212	1205.119	1165.601
1381282_at	308996	NA	NA	637.3642	630.9372	329.1998
1369329_at	56761	Notch3	Notch gene homolog 3 (Drosophila)	637.1374	142.0878	257.0097
1367699_at	81922	Sh3gl1	SH3-domain GRB2-like 1	637.0359	710.3387	415.4241
1373290_at	312299	LOC31229	NA	636.9668	4052.931	671.7595

1396859_at	94188	Zfp423	zinc finger protein 423	636.8016	439.6044	338.7059
1395514_at	360866	RGD13089	similar to ezrin-binding partner PACE-1	636.6737	406.3389	488.1917
1377832_at	310344	Plk4_predi	polo-like kinase 4 (Drosophila) (predict	636.48	3233.695	1161.141
1375207_at	287949	Scarf2_pre	scavenger receptor class F, member 2	636.3955	581.25	262.4692
1378552_at	313022	Map3k6_pr	mitogen-activated protein kinase kinas	635.7832	230.0943	350.9012
1397503_at	303883	Lrch3_pred	leucine-rich repeats and calponin hom	635.7513	308.2181	668.3836
1393796_at	311391	Cep152_pr	centrosomal protein 152 (predicted)	635.6597	1004.72	1571.436
1374295_at	299604	Polrmt_pre	polymerase (RNA) mitochondrial (DNA	635.4738	566.7547	528.392
1383381_at	289728	RGD15603	RGD1560394 (predicted)	635.4176	2472.966	981.5415
1378968_at	500348	LOC50034	similar to Apoptosis facilitator Bcl-2-like	635.1412	1307.434	380.9062
1391635_at	301056	Ctdspl_pre	CTD (carboxy-terminal domain, RNA p	634.9248	101.4857	528.8143
1394617_at	303343	RGD13045	similar to abhydrolase domain containi	634.8276	55.01474	170.2972
1383471_at	288454	RGD13075	similar to RIKEN cDNA 1200006F02	634.3187	239.2645	555.9163
1394545_at	498964	NA	NA	634.2952	461.6945	762.8065
1391272_at	499942	LOC49994	NA	634.0745	125.0311	299.2677
1368772_at	24781	Slc4a3	solute carrier family 4, member 3	633.1246	440.8923	348.9449
1391754_at	192281	Oas1	2',5'-oligoadenylate synthetase 1, 40/4	633.0941	1022.94	448.9514
1382812_at	303764	RGD13104	similar to Protein Njmu-R1 (predicted)	633.0668	554.8233	240.7392
1382311_at	310877	LOC31087	TRAF2 binding protein	633.0028	93.27165	619.5285
1371918_at	652929	Cd99	NA	632.8701	2957.576	429.0665
1382614_at	499508	RGD15647	similar to Zinc finger, FYVE domain co	632.3753	441.627	793.2785
1376217_at	297720	Casc1_pre	cancer susceptibility candidate 1 (pred	632.266	317.5805	1324.255
1398197_at	500233	RGD15606	similar to Exocyst complex component	632.2036	154.7718	458.913
1373340_at	293847	Dusp9	dual specificity phosphatase 9	631.8774	43.6228	308.046
1378355_a_at	314396	Slc24a4_pr	solute carrier family 24 (sodium/potass	631.7845	294.8886	125.0615
1398611_at	302502	Cul4b_pred	cullin 4B (predicted)	631.7392	1485.645	672.8307
1368760_at	114105	Cxcl2	chemokine (C-X-C motif) ligand 2	631.7162	17.07703	1603.727
1386860_at	25277	Mfge8	milk fat globule-EGF factor 8 protein	631.5217	622.6677	895.535
1377497_at	304545	Oasl1	2'-5' oligoadenylate synthetase-like 1	631.4779	309.4897	919.6313
1382049_at	361727	RGD13046	similar to thymus atrophy-related prote	631.2113	1307.777	1045.954
1382758_at	291942	P7	P7 protein	631.0447	204.8877	116.5777
1383677_at	690043	LOC69004	NA	630.9226	1088.255	477.2166
1383931_at	360302	Ptprk	protein tyrosine phosphatase, receptor	630.8989	1291.626	1435.955
1383704_at	362298	RGD15657	similar to actin-related protein 3-beta (	630.0433	486.2313	535.535
1387803_at	60660	Ppp2r2b	protein phosphatase 2 (formerly 2A), r	629.3699	3654.404	6467.008
1393947_at	306574	Slc25a15	solute carrier family 25 (mitochondrial	628.7073	3582.027	173.5351
1376020_at	298452	Dph2	DPH2 homolog (S. cerevisiae)	628.6971	877.9455	491.7069
1378535_at	364510	Phf7	PHD finger protein 7	628.245	662.5791	259.3493
1388753_at	311642	Sulf2	sulfatase 2	628.242	2137.662	1866.529
1374991_at	287938	LOC28793	hypothetical LOC287938	628.1666	227.7833	257.7248
1389693_at	362514	RGD13108	similar to hypothetical protein 3010020	628.0687	677.3541	336.0318
1377454_at	294289	Zbtb9	zinc finger and BTB domain containing	628.0551	431.9139	382.1568
1381923_at	499839	RGD15646	similar to LOC387763 protein (predicte	627.4828	998.2738	649.2653
1379824_at	362481	Tox_predic	thymocyte selection-associated HMG b	627.3298	610.9909	5522.492
1394038_at	373066	Cdv1	carnitine deficiency-associated gene e	627.2384	1626.9	3720.597
1389341_at	312652	Plxnd1_pre	plexin D1 (predicted)	627.196	255.6433	84.39931
1388300_at	289197	Mgst3_pre	microsomal glutathione S-transferase 3	627.0755	1573.288	436.1956
1384117_at	499363	RGD15648	similar to 9130011E15Rik protein (pred	626.5977	1784.619	513.2269
1368651_at	24651	Pklr	pyruvate kinase, liver and red blood ce	626.5263	4959.599	1346.038
1385992_at	25629	Plin	perilipin	626.4169	337.6522	458.4301
1389436_at	289904	Zmynd17_	zinc finger, MYND domain containing 1	626.3944	308.6299	1139.88

1397645_at	287702	Ka17	type I keratin KA17	626.3222	351.6827	371.6803
1374642_at	311661	Zfp64	zinc finger protein 64	626.2325	102.9482	509.4813
1379972_at	309790	RGD13086	similar to 1700060H10Rik protein	626.1055	96.14575	2515.723
1383920_at	306586	Amt	aminomethyltransferase (glycine cleav	625.9066	45.75806	894.2075
1395212_at	290359	Npm2	nucleophosmin/nucleoplasmin 2	625.3863	440.8949	847.9568
1369206_at	113936	Cpb2	carboxypeptidase B2 (plasma)	625.011	105.5821	4231.46
1398513_at	499462	RGD15599	similar to Na+ dependent glucose trans	624.0642	1150.814	524.7475
1368687_at	29460	Tesk1	testis specific protein kinase 1	623.6984	663.8197	912.6159
1380565_at	304928	RGD13067	similar to 2810422O20Rik protein	623.6524	486.9253	260.1742
1385296_at	314999	Trmt12	tRNA methyltranferase 12 homolog (S	623.6257	703.0909	571.7944
1382745_at	310879	Alpk1_prec	alpha-kinase 1 (predicted)	623.5158	397.8141	580.3327
1390505_a_at	25104	Pc	Pyruvate carboxylase	623.1666	1171.073	1060.562
1380261_at	499806	LOC49980	similar to RIKEN cDNA 4933404M02	622.8724	579.0439	1583.852
1382722_at	303313	Scarf1_pre	scavenger receptor class F, member 1	622.4076	266.7098	432.0129
1398286_at	60356	Csad	cysteine sulfinic acid decarboxylase	622.3022	528.9406	1318.401
1382877_at	362260	Dhx35_pre	DEAH (Asp-Glu-Ala-His) box polypepti	622.0964	714.9394	478.8654
1368080_at	117183	Rgc32	response gene to complement 32	621.9553	367.3728	1136.81
1396070_at	361119	NA	NA	621.8668	212.8651	193.652
1371248_at	499660	LOC49966	NA	621.7405	138.5834	120.7682
1381702_at	57031	Adam19	a disintegrin and metalloproteinase do	621.5065	420.6688	445.0448
1381645_at	310652	RGD13111	similar to hypothetical protein	620.96	522.8499	379.7779
1393031_at	65270	Abo	ABO blood group (transferase A, alpha	620.7016	857.5976	273.5185
1383445_at	360204	Zfp191	zinc finger protein 191	620.4188	1876.236	1395.626
1379062_at	500994	NA	NA	620.0363	352.5628	302.749
1387963_a_at	114768	Uox	urate oxidase	619.8047	314.43	646.097
1388618_at	302248	Nid2	nidogen 2	619.548	253.8846	1251.383
1392157_at	289392	Plxna2_pre	plexin A2 (predicted)	619.3451	74.68243	541.5999
1395046_at	306570	Ank1	ankyrin 1, erythroid	619.2689	260.6782	316.3577
1375628_at	293991	Ndufb8_pre	NADH dehydrogenase (ubiquinone) 1	619.2581	4025.92	441.3895
1395989_at	294672	Gfm2	G elongation factor, mitochondrial 2	619.1985	819.3181	178.4615
1370315_a_at	79423	Stmn4	stathmin-like 4	618.6845	352.5427	717.5069
1398362_at	29492	Notch2	notch gene homolog 2 (Drosophila)	618.413	248.1991	2314.44
1383579_at	502282	NA	NA	618.3961	1628.695	1575.773
1378940_at	297483	Shq1_pred	SHQ1 homolog (S. cerevisiae) (predict	618.2628	491.4071	553.5283
1392881_at	363040	St3gal4	ST3 beta-galactoside alpha-2,3-sialyltr	618.0154	739.858	295.0251
1368667_at	81739	P2rx3	purinergic receptor P2X, ligand-gated i	617.865	352.1229	624.6424
1379580_at	311607	Chd6_pred	chromodomain helicase DNA binding p	617.747	1727.588	3574.845
1375493_at	289229	Vangl2_pre	vang-like 2 (van gogh, Drosophila) (pre	617.6173	553.8347	224.3273
1379409_at	361015	RGD15626	similar to spinocerebellar ataxia 7 hom	617.4862	89.95927	353.6366
1389644_at	689865	LOC68986	NA	617.0167	1269.987	2475.108
1368160_at	25685	Igfbp1	insulin-like growth factor binding protei	616.761	136.7431	5008.352
1392092_at	317580	Rab33a_pr	RAB33A, member of RAS oncogene fa	616.1868	933.0676	385.226
1379748_at	310968	LOC31096	NA	615.7604	790.9046	905.0591
1382151_at	361775	Trub1	TruB pseudouridine (psi) synthase hor	615.6093	577.5394	338.7945
1371376_at	500252	RGD15655	similar to Gene model 461 (predicted)	615.4476	1674.546	1559.503
1393026_at	364303	Rnase1_pr	ribonuclease, RNase A family, 1 (panc	614.9573	25.17782	15410.01
1369004_at	171111	Rab26	RAB26, member RAS oncogene family	614.1527	875.5173	423.9425
1383903_at	364901	St8sia5	ST8 alpha-N-acetyl-neuraminide alpha	614.0856	50.80651	19.62288
1398710_at	310848	Cyp2u1	cytochrome P450, family 2, subfamily u	613.9041	326.5999	1214.885
1397838_at	499976	RGD15647	RGD1564792 (predicted)	613.806	987.1028	2832.318
1377118_at	290722	RGD13085	similar to KIAA1712 protein	613.7243	1851.902	2672.158

1373451_at	361056	MGC9478(	similar to hypothetical protein FLJ1171	613.6703	1929.78	685.6683
1391107_at	300944	Xrn1_predi	5'-3' exoribonuclease 1 (predicted)	613.3579	136.4427	289.2756
1384769_a_at	314119	RGD15633	similar to Zinc finger X-linked protein Z	613.1677	527.4106	1332.601
1375994_at	24592	Nf1	neurofibromatosis 1	612.7084	492.6458	589.0886
1378750_at	680874	LOC68087	NA	611.8089	248.9268	347.0048
1383638_at	313434	RGD15663	similar to DNA segment on chromoson	611.7697	1207.892	1993.925
1371497_at	296627	Asb6	ankyrin repeat and SOCS box-containi	611.6385	1107.652	275.1102
1392689_at	287424	Aloxe3_pre	arachidonate lipoxygenase 3 (predicte	611.5482	238.8328	1390.638
1370308_at	192252	Rs21c6	RS21-C6 protein	611.446	5098.498	529.9944
1382025_at	288762	Mtm1	X-linked myotubular myopathy gene 1	611.2616	465.9584	511.5406
1390464_at	252858	Frmd4b	FERM domain containing 4B	611.2068	168.1888	723.6819
1390424_at	301044	NA	NA	610.9996	657.6494	275.0972
1376155_at	499507	RGD15611	similar to hypothetical protein 2BE212	610.1479	583.1234	2413.805
1369460_at	64554	Slc7a2	solute carrier family 7 (cationic amino a	609.8934	277.5499	28.76109
1391605_at	362263	Ptprt_predi	protein tyrosine phosphatase, receptor	609.876	1635.994	2842.206
1397017_at	66031	Wdr7	WD repeat domain 7	609.6335	241.4334	491.9596
1379318_at	498662	LOC49866	similar to RIKEN cDNA 2610019F03	609.6021	763.5309	1381.666
1392747_at	308283	Fbxo30	F-box protein 30	609.3294	1658.755	3317.625
1394700_at	171334	Il12rb2	interleukin 12 receptor, beta 2	608.8944	255.0483	473.3675
1381670_at	366512	NA	NA	608.8237	86.73238	455.0767
1379399_at	288908	RGD13061	similar to cDNA sequence BC016188	608.6247	282.0893	324.7018
1371121_at	246151	Sert1	Sertoli cell protein 1	608.4735	554.3569	302.5565
1390072_at	81651	Cspg4	chondroitin sulfate proteoglycan 4	608.4156	58.10148	241.0652
1383955_at	364144	RGD13113	similar to 2510002A14Rik protein (pre	608.1639	1279.689	1553.271
1370201_at	83839	Calb1	calbindin 1	608.0036	1700.48	9490.112
1397356_at	311193	Arhgap1_p	Rho GTPase activating protein 1 (pred	607.5531	257.2747	1973.749
1376603_at	362762	Wdr21_pre	WD repeat domain 21 (predicted)	607.4616	391.2572	670.781
1389691_at	308430	Znf575_pre	zinc finger protein 575 (predicted)	607.3629	131.2613	229.1247
1384336_at	293485	Cln3	ceroid lipofuscinosis, neuronal 3, juver	607.3295	486.4792	71.45059
1384118_at	308361	Zfp329_pre	zinc finger protein 329 (predicted)	607.2872	314.5057	419.2927
1387151_at	116555	Nup107	nucleoporin 107	607.2782	1454.223	552.3815
1389370_at	294287	Phf1	PHD finger protein 1	607.1295	424.2428	225.6529
1379197_at	114862	Cacna1h	calcium channel, voltage-dependent, T	606.1398	236.019	220.6602
1379577_at	500410	RGD15594	similar to MIC2L1 (predicted)	606.1058	519.1427	625.3161
1382077_at	140588	Gli3	GLI-Kruppel family member GLI3	605.9524	229.9569	358.8066
1387025_at	29564	Dync1i1	dynein cytoplasmic 1 intermediate cha	605.6604	4463.055	1091.533
1386846_at	291736	RGD13073	similar to hypothetical protein MGC363	605.4082	1375.81	251.9838
1384443_at	296083	Vps18_pre	vacuolar protein sorting 18 (yeast) (pre	605.3969	444.1677	279.068
1373177_x_at	287866	NA	NA	605.0016	728.0886	3569.363
1382821_at	140591	Ppfia3	protein tyrosine phosphatase, receptor	604.8942	729.4247	568.3207
1380990_at	362680	NA	NA	604.8229	234.4117	554.4487
1393860_at	365352	Mical2_pre	microtubule associated monoxygenase	604.8051	115.1246	175.9344
1395223_at	306589	MGC94736	similar to hypothetical protein MGC350	604.7239	976.4573	1495.376
1368251_at	25326	Jak3	Janus kinase 3	603.7417	201.8424	488.6769
1393089_at	299123	RGD13115	similar to Hypothetical protein KIAA058	602.7214	499.3953	627.1462
1370733_at	252964	Lipogenin	Lipogenin	602.17	123.3097	285.6679
1377991_at	311987	Rsbn1l_pre	round spermatid basic protein 1-like (p	602.0777	305.7486	725.5333
1387819_at	24331	Ela1	elastase 1, pancreatic	602.0404	17.2196	3719.83
1375976_a_at	293678	Mus81	MUS81 endonuclease homolog (yeast	601.9842	569.6815	461.9755
1367774_at	24421	Gsta3	glutathione S-transferase A3	601.9527	234.0507	752.9881
1376936_at	498185	LOC49818	similar to sodium/calcium exchanger p	601.8812	1065.486	419.9709

1385988_at	360960	Hmx1_pred	H6 homeo box 1 (predicted)	601.3898	2414.383	175.5421
1368174_at	54702	Egln3	EGL nine homolog 3 (C. elegans)	601.2392	294.6478	372.668
1374855_at	287422	Per1	period homolog 1 (Drosophila)	601.1207	447.3025	3371.785
1393464_at	315665	RGD13112	similar to RIKEN cDNA 4930550C14	600.5376	106.6228	550.6709
1385075_at	296081	Ccdc32	coiled-coil domain containing 32	600.3989	801.8551	239.4965
1373267_at	362724	Sh3yl1_pre	Sh3 domain YSC-like 1 (predicted)	600.3482	166.3733	986.2634
1387440_at	64831	Ireb2	iron responsive element binding protei	600.0313	990.6684	887.9466
1367604_at	338401	Crip2	cysteine-rich protein 2	599.9568	960.6907	303.2952
1395020_at	314262	Plekhh1_pr	pleckstrin homology domain containing	598.9231	239.7316	416.9853
1397007_at	501214	NA	NA	598.7896	84.86074	367.3892
1390629_at	312398	Smarcad1_	SWI/SNF-related, matrix-associated ac	598.7323	155.5085	210.8545
1383584_at	364973	Vmd2l1_pr	vitelliform macular dystrophy 2-like pro	598.672	390.4316	484.9735
1381206_at	498079	Cd96	CD96 antigen	598.3753	138.907	785.4474
1374165_at	288589	RGD15654	similar to Rasa4 protein (predicted)	598.2613	1438.119	1009.117
1373787_at	116509	Slc6a9	solute carrier family 6 (neurotransmitte	598.2601	515.3866	495.7512
1368564_at	84487	Slc17a6	solute carrier family 17 (sodium-depen	598.1754	140.7886	246.4932
1369899_s_at	58983	Rabggta	Rab geranylgeranyl transferase, a sub	597.8398	804.6034	792.2431
1375188_at	291852	Katnb1	katanin p80 (WD40-containing) subuni	597.3913	1326.26	442.5652
1381094_at	362118	RGD13097	similar to FLJ00022 protein (predicted)	597.3226	87.21593	328.2391
1374551_at	287719	Ifi35	interferon-induced protein 35	597.2267	1709.272	571.1891
1370854_at	246172	Nexn	nexilin	597.1044	132.8425	149.9941
1372306_at	292710	Ethe1_pred	ethylmalonic encephalopathy 1 (predic	597.0155	574.5827	329.9145
1387941_s_at	360426	Pla2g6	phospholipase A2, group VI	596.0253	1132.124	743.7494
1377636_at	498208	NA	NA	595.6786	858.3211	381.5257
1384872_at	499269	NA	NA	595.551	704.1085	246.1818
1375009_at	299346	Nudt14_pre	nudix (nucleoside diphosphate linked r	595.3913	5159.968	1025.822
1379659_at	289485	LOC28948	similar to BMP-2 inducible kinase	595.0072	449.0386	1022.73
1378523_at	300696	Ttc12	tetratricopeptide repeat domain 12	594.9939	722.3403	311.3922
1392512_at	64646	H2a	histone 2a	594.9881	7566.917	2002.943
1378994_at	316039	RGD15616	similar to oxysterol-binding protein-like	594.695	319.9252	422.2351
1369688_s_at	50646	Ptk2b	protein tyrosine kinase 2 beta	594.2816	288.9209	425.1502
1393043_at	303456	Wdr50_pre	WD repeat domain 50 (predicted)	594.2723	1305.911	3205.673
1376345_at	192349	Drd1ip	dopamine receptor D1 interacting prote	594.2151	706.886	1900.2
1383219_at	298801	Rsnl2	restin-like 2	594.0304	1812.401	835.26
1393896_at	362107	RGD13050	similar to ionized calcium binding adap	593.9784	469.5369	606.6981
1384971_at	295971	RGD13097	similar to Hypothetical protein MGC19	593.7853	195.3679	2898.862
1381513_at	315963	RGD13105	similar to RIKEN cDNA 1300017J02	593.7634	650.4945	720.2483
1390361_at	287094	Znf213_pre	zinc finger protein 213 (predicted)	593.223	862.3618	597.7973
1369973_at	497811	Xdh	xanthine dehydrogenase	593.1263	77.36098	466.2007
1375999_at	291580	RGD13120	similar to DD1 (predicted)	593.0422	62.64113	793.1735
1374785_at	296985	RGD15653	similar to CD69 antigen (p60, early T-c	592.9727	578.5183	292.9695
1382892_a_at	500898	NA	NA	592.9602	229.4131	122.1536
1381754_at	309162	NA	NA	592.8664	19.10594	671.6661
1390853_at	25435	Eef2k	eukaryotic elongation factor-2 kinase	592.2059	2021.639	840.1704
1377414_at	293953	RGD13079	similar to DNA segment, Chr 19, ERAT	592.137	879.5956	817.2366
1381580_at	309275	Trex2_pred	three prime repair exonuclease 2 (pred	590.7327	173.6053	275.873
1370862_at	25728	Apoe	apolipoprotein E	590.5951	139.2307	457.5677
1396997_at	290639	Fcho1_pre	FCH domain only 1 (predicted)	590.2707	411.3001	332.6591
1398361_at	498938	NA	NA	590.0275	420.0843	228.6927
1375935_at	54265	Llgl1	lethal giant larvae homolog 1 (Drosoph	589.3962	1219.985	595.9944
1372844_at	94268	Efna1	ephrin A1	588.7371	182.4829	1994.392

1368890_at	84470	Gnpat	glyceronephosphate O-acyltransferase	587.6242	840.8827	513.0882
1377744_at	307248	RGD1563C	similar to macrophage actin-associated	587.6107	183.3979	45.97463
1374440_at	289456	Dhrs8	dehydrogenase/reductase (SDR family	587.3992	542.2104	1104.245
1372952_at	292069	LOC29206	similar to RIKEN cDNA 2310061F22	587.2739	505.6683	507.001
1380360_at	298282	Oma1_pre	OMA1 homolog, zinc metallopeptidase	586.7948	537.3443	622.9231
1369912_at	54245	Crk	v-crk sarcoma virus CT10 oncogene h	586.6849	542.9931	255.5624
1387885_at	29558	Fcgrt	Fc receptor, IgG, alpha chain transport	586.6021	91.01775	339.4561
1389030_a_at	83805	Src	Rous sarcoma oncogene	585.8942	715.2606	636.8653
1390928_at	500150	RGD15596	similar to tigger transposable element	585.7709	850.8303	1349.297
1388591_at	310745	Dclre1b	DNA cross-link repair 1B, PSO2 homo	585.6178	1006.671	452.6843
1370561_at	171553	A3galt2	alpha 1,3-galactosyltransferase 2 (isog	585.4736	2720.708	197.2894
1379636_at	313840	LOC31384	similar to hypothetical protein FLJ3295	585.111	191.5247	669.635
1380721_at	292548	RGD13116	similar to RIKEN cDNA A230102I05 (p	584.9204	457.8818	353.8153
1395313_s_at	25291	Anxa3	annexin A3	584.5454	178.6014	336.0332
1395054_at	361495	Usp29_pre	ubiquitin specific protease 29 (predicte	584.4144	1003.401	150.103
1393689_at	296086	Ndufaf1_pr	NADH dehydrogenase (ubiquinone) 1	584.2093	461.4256	186.8945
1385967_at	303753	Foxk2_pre	forkhead box K2 (predicted)	584.1754	868.5725	304.0227
1395401_at	314622	Dos_predic	downstream of Stk11 (predicted)	583.9593	384.8348	322.2046
1387143_at	84686	Ppp1r9b	protein phosphatase 1, regulatory subu	583.8599	394.6179	477.3699
1387360_at	116470	Stx1a	syntaxin 1A (brain)	583.3968	1737.212	409.7648
1374003_at	498007	NA	NA	583.3362	1458.47	605.0056
1379657_a_at	293586	Znf511_pre	zinc finger protein 511 (predicted)	583.1373	765.0927	584.0371
1394530_at	301004	RGD1311C	similar to hypothetical protein FLJ2025	583.017	86.84622	993.71
1373089_at	116777	Cdh3	cadherin 3, type 1, P-cadherin (placen	583.0061	249.4966	269.0406
1385128_at	498356	RGD15657	similar to MGC68837 protein (predicte	582.9124	730.042	235.1759
1379579_at	307343	RGD13596	LEA_4 domain containing protein RGD	582.3121	321.7946	231.4132
1375026_at	315746	Cln6_predi	ceroid-lipofuscinosis, neuronal 6 (pred	581.8479	15157.16	1815.601
1384940_at	309478	RGD13053	similar to KIAA0335	581.2341	547.2454	1076.786
1383547_at	360753	RGD15646	similar to neuropathy target esterase h	581.0322	384.3713	363.4429
1383597_at	259171	Dclre1c	DNA cross-link repair 1C, PSO2 homo	580.9434	185.8036	231.5646
1373969_at	282843	Sorbs3	sorbin and SH3 domain containing 3	580.8575	99.20661	643.1565
1393611_at	289471	LOC28947	NA	580.3539	885.7436	390.1317
1390268_at	288700	Rab35	RAB35, member RAS oncogene family	580.0309	1259.828	1149.496
1392482_at	291565	RGD13115	similar to actin filament associated pro	579.4424	1522.075	873.5335
1383564_at	293624	Irf7	interferon regulatory factor 7	579.3123	447.8474	1046.088
1382949_at	314264	Rdh12_pre	retinol dehydrogenase 12 (predicted)	579.2382	22.52425	217.2968
1381526_at	307799	Pard6a	par-6 (partitioning defective 6,) homolo	579.2372	617.9411	938.0013
1392279_at	500442	LOC50044	similar to dynein, axonemal, intermedia	579.1562	378.6081	1632.027
1378634_at	363481	Hdac8_pre	histone deacetylase 8 (predicted)	579.1023	944.0345	696.4418
1378787_a_at	360741	Dgcr14	DiGeorge syndrome critical region gen	579.0197	248.0078	202.9416
1394548_at	314855	Cpm_predi	carboxypeptidase M (predicted)	578.8536	405.5868	316.3322
1376903_at	315117	RGD15596	similar to Hypothetical protein 6330514	578.5397	293.3802	321.4801
1368875_a_at	116595	Nrxn2	neurexin 2	577.1082	1876.646	383.1176
1392057_at	500893	LOC50089	similar to GLI-Kruppel family member (	576.8652	924.9069	728.8254
1383476_at	50693	Itih3	inter-alpha trypsin inhibitor, heavy chai	576.7334	1753.773	1635.275
1387998_at	245709	Exoc8	exocyst complex component 8	576.6479	284.0017	18.25923
1368565_at	29483	Slc1a3	solute carrier family 1 (glial high affinity	576.6361	70.28347	1131.171
1372370_at	315550	Rpusd4	RNA pseudouridylate synthase domain	576.2499	561.4994	682.1234
1371071_at	294962	Gnb4	guanine nucleotide binding protein bet	576.0761	1162.805	511.6442
1369120_a_at	25329	Lhb	luteinizing hormone beta	576.0452	353.6985	266.5287
1377171_at	266711	Lzts1	leucine zipper, putative tumor suppres	576.0448	673.9347	496.7609

1379258_at	305351	Klhl5	kelch-like 5 (Drosophila)	575.6133	3603.618	2374.878
1387001_at	116546	Ralb	v-ral simian leukemia viral oncogene h	575.4304	998.7408	627.8184
1383858_at	292597	Ttyh1_prec	tweety homolog 1 (Drosophila) (predic	574.8938	54.20308	444.7747
1390634_at	288106	RGD15663	similar to KIAA1407 (predicted)	574.8617	405.0472	64.8413
1387194_at	171097	Centa1	centaurin, alpha 1	574.0575	940.2229	391.2567
1369682_at	25640	Tcf2	transcription factor 2	573.9923	44.27416	243.7101
1377765_at	83718	Clic4	chloride intracellular channel 4	573.7693	137.0198	172.4995
1370008_at	140938	Psmc3ip	proteasome (prosome, macropain) 26S	573.5411	2358.296	423.83
1370484_at	404977	Olr1468	olfactory receptor 1468	573.5014	132.7363	187.2629
1380521_at	288077	Hcls1	hematopoietic cell specific Lyn substra	572.6449	293.3852	364.1124
1381014_at	310969	Ifi44	interferon-induced protein 44	572.4144	200.6751	965.3661
1381986_at	685707	LOC68570	NA	572.3725	738.1456	820.5404
1368292_at	140694	Dnm1	dynamin 1	572.2511	1653.592	785.6832
1394782_at	170903	Arntl2	aryl hydrocarbon receptor nuclear tran	572.0784	70.08498	170.3932
1393225_at	314655	LOC31465	NA	571.7733	542.1535	333.3834
1398866_at	245903	Magi3	membrane associated guanylate kinas	571.4828	3151.717	3213.774
1391255_at	287457	Zmynd15_	zinc finger, MYND domain containing 1	571.4022	159.5795	479.3751
1389244_x_at	60628	Cxcr4	chemokine (C-X-C motif) receptor 4	571.3442	217.7338	307.8561
1393128_at	295228	RGD1310	similar to RIKEN cDNA 2610029K21	570.8981	619.1959	397.7483
1372349_at	293844	RGD1562	similar to UCH37-interacting protein 1	570.8608	1893.961	671.9815
1387699_at	25411	Cnga2	cyclic nucleotide gated channel alpha 2	570.6785	211.0335	93.02684
1373514_at	303735	RGD13081	similar to chromosome 17 open readin	570.6301	741.0248	364.4808
1373417_at	360549	Plscr3	phospholipid scramblase 3	570.4955	1482.272	555.9612
1370482_at	24767	Scnn1b	sodium channel, nonvoltage-gated 1 b	570.4783	188.5491	171.1741
1371073_at	24390	B4galt1_m	UDP-Gal:betaGlcNAc beta 1,4- galact	570.3724	780.5831	595.3134
1383807_at	289657	Tbc1d19_p	TBC1 domain family, member 19 (pred	569.9768	844.1916	571.6142
1384083_at	367384	B3gntl1	UDP-GlcNAc:betaGal beta-1,3-N-acety	569.9506	198.2643	274.1033
1384879_at	293704	Trpt1_pred	tRNA phosphotransferase 1 (predicted	569.739	262.3399	665.804
1378802_at	309381	RGD13032	Phytn_dehydro and Pyr_redox domain	569.7067	109.5825	582.9164
1376818_at	361552	Wtip_predi	WT1-interacting protein (predicted)	569.4086	604.3655	432.7484
1376290_at	304201	RGD1561	similar to ecotropic viral integration site	569.0103	1229.785	388.6387
1397432_at	307024	RGD13072	similar to CG4854-PA (predicted)	568.8561	348.5775	349.9343
1384112_at	58813	Nt5e	5' nucleotidase, ecto	568.7375	564.8956	4474.638
1368588_at	85432	Ddx52	DEAD (Asp-Glu-Ala-Asp) box polypept	568.7278	1046.901	1007.6
1393590_at	353303	Col23a1	procollagen, type XXIII, alpha 1	568.6917	129.3426	186.449
1381709_at	313806	Ibrdc3_pre	IBR domain containing 3 (predicted)	568.5289	215.3986	292.2894
1372476_at	286922	Fads3	fatty acid desaturase 3	568.4726	1269.55	134.6732
1384398_at	498947	NA	NA	567.9941	523.6016	635.7269
1397581_at	171581	Rhov	ras homolog gene family, member V	567.3484	628.2215	2081.85
1367851_at	25526	Ptgds	prostaglandin D2 synthase	567.2507	33.73527	1278.885
1385062_at	304654	RGD1306	hypothetical LOC304654 (predicted)	566.6961	242.8663	261.3225
1381791_at	363391	RGD1562	similar to GTPase activating protein te	566.3556	131.871	255.6059
1389048_at	83470	Bmp1	bone morphogenetic protein 1	565.6629	508.2563	918.5463
1390979_at	499124	LOC49912	mouse zinc finger protein 14-like	565.5632	715.7754	798.769
1372729_at	362248	Procr	protein C receptor, endothelial	565.1751	32.82342	218.818
1373286_at	362650	Fblim1	filamin binding LIM protein 1	564.9261	1715.192	333.9802
1392170_at	65026	Rwdd3	RWD domain containing 3	564.8303	275.0275	841.1542
1368599_at	24783	Slc9a2	solute carrier family 9 (sodium/hydroge	564.5425	341.0796	611.3173
1384906_at	316232	Egfl9_pred	EGF-like-domain, multiple 9 (predicted	564.4363	118.0672	123.1829
1382263_at	308017	NA	NA	564.2343	800.6482	2052.102
1372603_at	311187	Pacsin3	protein kinase C and casein kinase sul	564.1814	365.5857	111.3526

1384550_at	317344	RGD1565€	similar to RIKEN cDNA 1810030O07 (	564.0088	941.6748	1573.706
1388433_at	360626	Krt1-19	keratin complex 1, acidic, gene 19	563.274	9.450577	1472.527
1368572_a_at	24408	Grin1	glutamate receptor, ionotropic, N-meth	563.122	205.5457	481.0486
1375074_at	266764	Tbkbp1	TBK1 binding protein 1	562.9969	804.9971	346.1853
1382765_at	292105	Cep27_pre	centrosomal protein 27 (predicted)	562.4757	651.5426	556.5274
1394539_at	64572	Vax2	ventral anterior homeobox 2	562.0886	148.0541	302.8334
1388219_at	79247	Htr5b	5-hydroxytryptamine (serotonin) recep	561.9236	350.1446	209.4065
1374959_at	291084	Nqo2	NAD(P)H dehydrogenase, quinone 2	561.4663	16.19999	601.7283
1375168_at	84582	Hdac7a	histone deacetylase 7A	561.4571	575.4336	526.6974
1373881_at	362456	Arhgdib	Rho, GDP dissociation inhibitor (GDI) I	561.413	222.3157	327.9174
1393427_s_at	245963	Egfl7	EGF-like domain 7	561.2972	855.8897	428.0897
1374718_at	498089	RGD15651	similar to deltex 3-like (predicted)	561.1523	396.7224	1940.508
1382039_at	362987	Rabl2a	RAB, member of RAS oncogene family	561.1288	1105.253	853.1465
1392797_at	361976	RGD13112	similar to CGI-41 protein	560.6956	496.9846	227.1441
1389758_at	360581	Tada2l	transcriptional adaptor 2 (ADA2 homol	560.2775	1136.261	530.283
1370136_at	89789	Lbr	lamin B receptor	559.9246	935.3	274.8884
1372695_at	260327	Fndc5	fibronectin type III domain containing 5	559.8044	553.3323	388.535
1373281_at	360495	RGD13101	similar to hypothetical protein FLJ1268	559.759	711.4794	429.7343
1375341_at	362278	Kua_predic	Kua homolog (predicted)	559.6881	1763.039	266.0999
1397632_at	306147	Slitrk1_pre	SLIT and NTRK-like family, member 1	559.6084	234.1315	185.4026
1387726_at	66019	Cdx2	caudal type homeo box 2	559.6059	362.1058	236.8492
1398171_at	292765	Ggn	gametogenetin	559.5602	171.4586	211.083
1387408_at	140593	Siah2	seven in absentia 2	559.5327	557.1433	1906.681
1381107_at	307924	Zfp276	zinc finger protein (C2H2 type) 276	559.315	243.2988	13.73819
1370895_at	85250	Col5a2	procollagen, type V, alpha 2	559.2894	157.0168	1921.387
1383973_at	307816	RGD15598	similar to expressed sequence AW413	558.9563	200.0341	359.7067
1394639_at	502327	RGD15639	similar to CDC42 small effector 2 (prec	558.3529	962.1715	370.7077
1376413_at	83589	Apba1	amyloid beta (A4) precursor protein-bi	558.2348	325.0212	490.5887
1378189_at	361489	NA	NA	558.0005	159.1951	514.6123
1384067_at	309129	Fcho2_pre	FCH domain only 2 (predicted)	557.7278	362.3661	1285.658
1380797_at	361268	Asb13_pre	ankyrin repeat and SOCS box-containi	557.5835	300.4087	255.9858
1393618_at	297073	Rarres2	retinoic acid receptor responder (tazar	557.4418	234.2201	220.7181
1390619_at	315216	RGD13119	hypothetical LOC315216 (predicted)	557.2953	299.029	46.59804
1388712_at	116693	Pscd3	pleckstrin homology, Sec7 and coiled-c	557.2432	1643.99	1029.604
1383017_at	29616	Ptprm	protein tyrosine phosphatase, receptor	557.0649	217.1302	430.8243
1397756_at	500623	RGD15637	similar to BC068281 protein (predicted	557.0175	272.5097	46.15672
1387350_at	114206	Tmf1	TATA element modulatory factor 1	556.8963	398.0987	272.7933
1377565_at	311039	Galnt13	UDP-N-acetyl-alpha-D-galactosamine:	556.4808	108.133	381.6678
1370435_a_at	207120	Nudt6	nudix (nucleoside diphosphate linked r	556.2691	378.5924	233.8795
1374628_at	362061	Cryz	crystallin, zeta	556.1243	454.8975	811.4036
1368020_at	81726	Mvd	mevalonate (diphospho) decarboxylase	555.8912	743.8961	278.2577
1397683_at	307494	RGD1305€	similar to RIKEN cDNA 2410080P20 (p	555.7825	466.7001	368.9859
1393780_at	310375	Zfp131	zinc finger protein 131	555.6899	690.0638	4648.708
1396815_at	501008	NA	NA	555.6079	315.5492	543.6924
1367658_at	59312	Shank3	SH3/ankyrin domain gene 3	555.5264	1205.853	454.6832
1371023_at	114029	Egfl4	EGF-like-domain, multiple 4	555.2665	684.3664	850.8017
1395440_at	498695	MGC12508	similar to RIKEN cDNA 5133401N09	555.26	670.5411	390.5338
1398310_at	192242	Akr1d1	aldo-keto reductase family 1, member	555.0826	223.2745	824.9081
1393967_at	498522	RGD15624	RGD1562422 (predicted)	554.9998	633.8878	355.1346
1374727_at	294513	LOC29451	similar to DnaJ (Hsp40) homolog, subf	554.9555	552.6656	689.6588
1382958_at	25708	Ucp3	uncoupling protein 3 (mitochondrial, pr	554.0098	135.6361	89.04002

1382370_at	117524	Ccnf	cyclin F	553.6358	2116.632	142.2371
1379971_at	311415	Zc3h6_pre	zinc finger CCCH type containing 6 (pr	553.6301	1179.107	1592.982
1390017_at	64057	Hapln2	hyaluronan and proteoglycan link prote	553.451	201.6003	329.1805
1378821_at	500709	LOC50070	NA	553.0409	538.8846	296.5757
1387596_at	116677	F2rl1	coagulation factor II (thrombin) recepto	552.8531	320.1381	412.8956
1367812_at	29211	Spnb3	spectrin beta 3	552.8524	242.0337	293.2637
1369445_at	64046	Mre11a	meiotic recombination 11 homolog A (S	552.8377	176.7224	32.12743
1374640_at	361992	Them4	thioesterase superfamily member 4	552.2412	976.0928	609.6455
1377034_at	291091	Serpinb1a	serine (or cysteine) proteinase inhibito	552.1059	32.17989	258.3901
1387669_a_at	25315	Ephx1	epoxide hydrolase 1, microsomal	552.0114	803.8076	475.1328
1388140_at	81756	Rab13	RAB13, member RAS oncogene family	551.9307	453.5485	377.2802
1373250_at	353229	Tmem23	transmembrane protein 23	551.6762	7612.951	968.4757
1373344_at	363471	RGD15622	similar to GS2 gene (predicted)	551.6115	647.5381	263.0048
1391681_at	362485	Ccne2_pre	cyclin E2 (predicted)	551.4987	487.9406	455.9529
1395624_at	314788	RGD13079	similar to RIKEN cDNA C430008C19	551.4031	381.9644	520.2074
1369276_at	59328	Smad5	MAD homolog 5 (Drosophila)	551.3092	1296.955	652.328
1367691_at	85332	Prkcdbp	protein kinase C, delta binding protein	550.8581	163.2385	212.7368
1381570_at	308496	RGD13080	similar to T-cell activation NFKB-like pr	550.4596	376.5258	1027.699
1393451_at	361416	RGD13109	similar to RIKEN cDNA 2610510J17	550.4356	2309.091	622.7585
1368703_at	64353	Pdlim5	PDZ and LIM domain 5	550.3763	167.391	109.6063
1397588_at	360483	RGD15617	RGD1561796 (predicted)	550.0439	682.8833	325.5691
1392292_at	287109	Kctd5_prec	potassium channel tetramerisation dor	549.9234	971.6059	323.093
1388950_at	362584	Col9a2_pre	procollagen, type IX, alpha 2 (predicte	549.6398	178.0136	306.7326
1377601_at	294333	RGD13112	similar to C21orf70 protein	549.4929	445.9652	306.3949
1369489_at	29427	Aif1	allograft inflammatory factor 1	549.3323	65.36617	131.9319
1367909_at	171408	Dcxr	dicarbonyl L-xylulose reductase	548.9092	140.4121	117.7232
1379450_at	310760	Cttnbp2nl_	CTTNBP2 N-terminal like (predicted)	548.6025	1646.529	1778.976
1368238_at	24618	Pap	pancreatitis-associated protein	548.4478	87.26406	397.6208
1382750_at	500687	NA	NA	548.3876	355.0313	634.0741
1370271_a_at	192266	Grpca	glutamine/glutamic acid-rich protein A	548.2807	209.669	149.8845
1383821_at	84472	Ilf3	interleukin enhancer binding factor 3	548.1137	1346.246	888.987
1369866_at	56825	LOC56825	prochymosin	548.0921	95.11072	81.40764
1382216_at	362688	Gemin6	gem (nuclear organelle) associated pro	547.859	1147.609	514.8194
1380736_at	117269	Rps6ka2	ribosomal protein S6 kinase polypeptic	547.6758	16.81804	62.66646
1379396_at	361251	Elmo1_pre	engulfment and cell motility 1, ced-12 l	547.5118	854.3729	557.4128
1374539_at	360932	LOC36093	NA	547.1457	909.1164	327.6319
1371032_at	25494	Nid1	nidogen 1	546.3069	798.8143	509.8424
1387329_at	171501	Rnf38	ring finger protein 38	545.959	288.2768	300.3969
1369663_at	65030	Ephx2	epoxide hydrolase 2, cytoplasmic	545.9466	1064.632	1056.646
1374738_at	361887	Sdccag10	serologically defined colon cancer anti	545.4702	998.9833	939.0917
1384808_at	306459	Stox2	storkhead box 2	545.1508	242.8212	141.4903
1387659_at	83585	Gda	guanine deaminase	544.9578	1715.408	331.7226
1398474_at	311352	LOC31135	similar to Adenosine deaminase CG11	544.7003	1098.916	502.7863
1388276_at	291159	Hist1h2ai_	histone 1, H2ai (predicted)	544.6212	723.3545	505.7907
1376564_at	308458	Spnb4	spectrin beta 4	543.8438	429.9052	971.9148
1369278_at	81663	Gna12	guanine nucleotide binding protein, alp	543.7879	724.0837	102.4613
1376445_at	116472	Il17b	interleukin 17B	543.6896	156.757	383.1005
1389662_at	287715	Wnk4	WNK lysine deficient protein kinase 4	543.2096	65.42786	880.6273
1384198_at	302817	Cova1_pre	cytosolic ovarian carcinoma antigen 1	543.1667	1063.08	1200.119
1398000_at	316313	Dst_predic	dystonin (predicted)	543.1624	143.1542	1699.721
1372039_at	287707	Nkiras2_pr	NFKB inhibitor interacting Ras-like pro	542.4939	1748.168	399.228

1397183_at	499669	RGD15631	similar to trinucleotide repeat containin	542.2987	330.8056	1135.399
1367999_at	29539	Aldh2	aldehyde dehydrogenase 2	542.2737	178.8489	236.6672
1373790_at	361998	Car14_pre	carbonic anhydrase 14 (predicted)	542.1348	118.8167	162.102
1374076_at	361409	RGD13052	similar to hypothetical protein FLJ3438	541.966	649.973	339.6291
1372904_at	362833	Mobkl2b_p	MOB1, Mps One Binder kinase activat	541.0904	537.4584	462.7432
1374025_at	363118	Nmnat3	nicotinamide nucleotide adenylyltransf	541.0576	347.5317	565.8791
1372684_at	289734	Smtn	smoothelin	541.0548	649.8061	708.1942
1390162_at	303915	Iqcb1_prec	IQ calmodulin-binding motif containing	541.0361	1314.115	2938.061
1372809_at	290595	LOC29059	hypothetical gene supported by AF152	540.7459	30.14922	523.6277
1392744_at	312647	Atg7	autophagy-related 7 (yeast)	540.6516	364.7879	632.3185
1387093_at	170698	Slco1a4	solute carrier organic anion transporte	540.5961	284.9253	354.3854
1377048_at	361730	RGD1311C	similar to cDNA sequence BC021917	540.5576	754.0432	440.2095
1383178_at	305105	Fbxo28_pr	F-box protein 28 (predicted)	540.5106	452.7112	290.276
1372799_at	297389	Dguok_pre	deoxyguanosine kinase (predicted)	540.4334	922.4707	1336.393
1367899_at	25439	F2r	coagulation factor II (thrombin) recepto	540.3595	3236.014	1006.303
1398290_at	64120	Kcnk13	potassium channel, subfamily K, meml	540.1812	157.3368	307.3042
1391522_at	293631	Brsk2	brain serine/threonine kinase 2	539.6004	310.4395	99.52681
1370328_at	171548	Dkk3	dickkopf homolog 3 (Xenopus laevis)	539.5133	305.8115	499.972
1396478_at	367763	RGD15632	similar to Expressed sequence AW547	539.5043	632.1673	367.7851
1387124_at	24504	Inha	inhibin alpha	539.4763	160.4644	706.5143
1389093_at	29431	Pak1	p21 (CDKN1A)-activated kinase 1	539.3936	3065.173	3059.387
1392965_a_at	292401	Smoc2_pre	SPARC related modular calcium bindir	539.3618	43.05785	1520.84
1384856_at	306340	RGD15639	similar to mKIAA1623 protein (predicte	539.3079	110.4005	107.5808
1373154_at	299208	RGD15635	similar to mKIAA0998 protein (predicte	538.5011	378.4301	935.5058
1373602_at	363172	Lars2_prec	leucyl-tRNA synthetase, mitochondrial	537.5938	408.5286	308.9913
1392720_at	500801	RGD15616	similar to Cytochrome P450 4F6 (CYP	537.2418	37.19725	695.4102
1377618_at	363243	Klf7_predic	Kruppel-like factor 7 (ubiquitous) (pred	537.2038	590.1604	1195.889
1382475_at	309157	Peli3_pred	pellino homolog 3 (Drosophila) (predic	536.5929	1109.071	515.8306
1397642_at	64012	Rad50	RAD50 homolog (S. cerevisiae)	536.3798	1335.476	503.9234
1373476_at	500251	LOC50025	hypothetical protein LOC500251	535.9968	1340.414	761.0006
1394665_at	365793	Rab33b_pr	RAB33B, member of RAS oncogene fa	535.9569	228.6879	405.42
1369698_at	140668	Abcc3	ATP-binding cassette, sub-family C (C	535.3935	1296.069	2052.187
1382995_at	81527	Nrp2	neuropilin 2	535.3662	73.20895	778.5592
1389996_at	290705	Nek1_pred	NIMA (never in mitosis gene a)-relatec	535.2866	1297.307	1754.243
1381391_at	287798	MGC9521(	hypothetical LOC287798	534.5147	290.8886	109.8571
1394764_at	304286	LOC30428	NA	533.99	411.4475	335.9888
1374831_at	309103	Athl1_pred	ATH1, acid trehalase-like 1 (yeast) (pr	533.4813	1143.055	1012.801
1368443_at	170928	Efcbp2	EF hand calcium binding protein 2	533.4347	1606.636	482.7274
1389628_at	287745	Plcd3_prec	phospholipase C, delta 3 (predicted)	532.9616	264.028	107.7111
1370970_at	276720	Kcnj14	potassium inwardly-rectifying channel,	532.8078	303.7567	843.0401
1398480_at	362945	NA	NA	532.6717	127.9847	54.57744
1393502_at	361410	RGD13061	similar to predicted CDS, putative prot	532.0655	555.7042	893.7799
1390542_at	497976	RGD15637	similar to RAD51L2/RAD51C protein (p	531.9839	1444.968	581.5859
1397045_at	314694	Chst11_pre	carbohydrate sulfotransferase 11 (prec	531.6325	177.7555	490.7147
1376239_at	362802	Atp6v1c2	ATPase, H+ transporting, V1 subunit C	531.4743	522.2197	1316.464
1387153_at	24915	Ril	reversion induced LIM gene	531.4029	356.7929	326.2401
1390974_a_at	502396	NA	NA	531.1224	633.1873	748.8599
1374023_at	305898	RGD13071	similar to KIAA1305 protein (predicted)	530.0887	286.0689	901.8123
1393266_at	500754	NA	NA	529.9497	230.7613	148.1518
1393874_at	367316	RGD15627	similar to aquaporin 12 (predicted)	529.5749	155.5381	481.1735
1390859_at	291921	RGD13051	similar to Nedd4 binding protein 1 (pre	529.3438	197.8747	659.1811

1367846_at	24615	S100a4	S100 calcium-binding protein A4	529.3036	219.8948	111.1878
1372976_at	300982	Abhd14a	abhydrolase domain containing 14A	529.1483	75.06322	288.4341
1373523_at	304966	Fcgr3a	Fc fragment of IgG, low affinity IIIa, rec	529.0023	104.7958	258.3629
1373253_at	303577	Acbd4	acyl-Coenzyme A binding domain cont	528.909	1110.982	601.6939
1385497_x_at	360926	Lnx1_predi	ligand of numb-protein X 1 (predicted)	528.6729	991.8048	2902.584
1397207_at	307774	Otud4	OTU domain containing 4	528.6482	360.2508	352.3509
1390607_at	363783	Zdhhc23	zinc finger, DHHC domain containing 2	528.5817	1024.887	235.4588
1398175_at	362876	Ppfia2_pre	protein tyrosine phosphatase, receptor	528.1758	121.4777	81.84899
1368510_at	25172	Gata1	GATA binding protein 1	528.1631	64.0278	186.9654
1377459_at	406866	Ly6g6e	lymphocyte antigen 6 complex, locus (	527.8633	298.3118	127.3983
1378197_at	300053	KIFC2	kinesin family member C2	527.6098	370.9211	728.0698
1369351_at	54279	Cntn3	contactin 3	527.294	125.5987	158.9221
1384488_at	494444	Cdkn2d	NA	527.2055	2997.037	683.4331
1392539_at	292737	Blvrb_pred	biliverdin reductase B (flavin reductase	526.9994	451.0443	312.391
1390011_at	304667	Rtbnd_prec	retbindin (predicted)	526.9736	352.9357	537.4719
1375749_at	305287	Sec3l1	SEC3-like 1 (S. cerevisiae)	526.8915	1167.845	111.8015
1368243_at	29530	Amhr2	anti-Mullerian hormone type 2 receptor	526.5502	288.3436	458.2712
1377387_a_at	498110	NA	NA	526.3117	268.1346	369.4237
1378926_at	78957	Shank1	SH3 and multiple ankyrin repeat doma	525.6463	489.6332	150.1164
1398306_at	25028	Ampd1	adenosine monophosphate deaminase	525.361	98.87689	555.9016
1374658_at	317433	Iqsec2_pre	IQ motif and Sec7 domain 2 (predicted	525.0454	177.7633	383.1994
1395519_at	297109	MGC95152	similar to B230212L03Rik protein	524.7263	795.275	638.4978
1383553_at	315599	RGD13112	similar to CDNA sequence BC034204	524.5738	730.384	516.4585
1387376_at	54349	Aox1	aldehyde oxidase 1	524.5461	54.60786	395.9445
1387092_at	64190	Fxyd4	FXYD domain-containing ion transport	524.4884	140.942	84.50862
1370987_at	24796	Spn	sialophorin	523.9952	1232.739	540.9187
1373896_at	25716	Syt1	synaptotagmin I	523.9856	665.2606	2197.961
1396521_at	291699	Stard4_pre	StAR-related lipid transfer (START) do	523.9676	1798.366	1199.485
1381298_at	307126	Mcm10_pr	minichromosome maintenance deficier	523.3981	1126.903	216.1884
1374309_at	304300	Papolb	poly (A) polymerase beta (testis specif	523.1982	388.2559	262.2826
1390397_at	362235	RGD13107	similar to chromosome 20 open readin	523.1942	169.386	494.4694
1372572_at	407765	Siat7F	sialyltransferase 7F	523.0796	542.5776	169.0714
1388962_at	287662	Pcgf2_prec	polycomb group ring finger 2 (predicted	522.9045	825.4592	630.6151
1381901_at	499586	RGD15631	RGD1563109 (predicted)	522.2221	208.8924	201.3811
1376971_at	361876	NA	NA	521.8627	123.4129	792.0367
1387223_at	29416	Aadat	aminoadipate aminotransferase	521.824	331.6204	339.5999
1388479_at	25418	Dpysl3	dihydropyrimidinase-like 3	521.538	1505.44	454.3707
1374245_at	363251	LOC36325	similar to 1700029B21Rik protein	521.0151	224.6626	298.5682
1389360_at	116831	Fxyd3	FXYD domain-containing ion transport	520.7665	154.7441	1285.067
1371109_at	313421	C8b_mapp	complement component 8, beta polype	520.4241	208.0792	259.8292
1380517_at	317260	Zmym3	zinc finger, MYM-type 3	520.3685	1031.616	602.7827
1395275_at	296751	Pus7_pred	pseudouridylate synthase 7 homolog (	520.2913	222.9767	238.8256
1374450_at	500225	RGD15622	similar to PAP-1 binding protein (predi	520.1054	477.8149	179.7091
1379906_at	24214	Atp1b2	ATPase, Na+/K+ transporting, beta 2 p	519.9857	344.992	1242.101
1378468_at	360575	RGD1564C	similar to novel protein (predicted)	519.239	674.6674	503.8894
1368354_at	25260	Gstt1	glutathione S-transferase theta 1	519.2251	1523.274	908.5743
1367682_at	81517	Mdk	midkine	518.8068	238.0904	363.222
1387146_a_at	50672	Ednrb	endothelin receptor type B	518.6396	139.8317	270.5731
1384500_at	316638	Sned1	insulin responsive sequence DNA binc	518.49	431.0558	2629.852
1393731_at	266712	Rnf40	ring finger protein 40	518.3111	289.2283	211.2416
1382891_at	302566	RGD15646	similar to ubiquitin specific protease 27	518.1482	370.6709	1955.277

1368232_at	81727	Mvk	mevalonate kinase	518.0731	1528.641	489.3073
1395336_at	501207	LOC50120	NA	517.4962	776.0218	787.0828
1392510_at	362336	RGD1308C	similar to hypothetical protein B230314	517.0929	175.4095	396.429
1384493_at	309465	Gsto2	glutathione S-transferase omega 2	517.0614	230.988	839.1632
1380389_at	24929	Ptger3	prostaglandin E receptor 3 (subtype EI	516.7347	715.7776	266.7168
1373945_at	314647	RGD1565C	similar to bruno-like 5, RNA binding pro	516.257	3275.37	1679.235
1387666_at	64020	Gpr85	G protein-coupled receptor 85	516.1093	813.133	4090.486
1388234_at	298210	Ifna1	interferon-alpha 1	515.6265	301.2839	309.9404
1369416_at	114245	Hcn3	hyperpolarization-activated cyclic nucle	514.9937	234.3013	308.2532
1368305_at	83584	Casp6	caspase 6	514.9691	586.4742	375.9115
1379945_at	500262	RGD15631	similar to T-cell activation kelch repeat	514.8837	452.5923	720.1429
1368928_at	83616	Trim3	tripartite motif protein 3	514.42	775.5907	361.69
1368351_at	29571	Scn10a	sodium channel, voltage-gated, type 10	514.4168	254.179	342.9154
1381893_at	294412	NA	NA	514.3661	449.2256	385.7538
1367905_at	54410	Enpp3	ectonucleotide pyrophosphatase/phos|	514.3216	3245.972	249.4929
1368374_a_at	116568	Ggt1	gamma-glutamyltransferase 1	514.1635	384.4641	296.6356
1376504_at	362931	Bai1_predi	brain-specific angiogenesis inhibitor 1	514.0369	733.3353	443.0856
1392944_at	315776	RGD15654	similar to talin 2 (predicted)	513.9343	315.6731	220.8235
1368115_at	65130	Cldn3	claudin 3	513.5901	122.4427	744.6177
1370569_at	24627	Pde4d	phosphodiesterase 4D, cAMP specific	513.4359	410.1739	493.35
1383203_at	311559	RGD15597	similar to Palate lung and nasal carcin	513.2206	218.6693	603.9951
1371452_at	300744	Bmsc-UbP	bone marrow stromal cell-derived ubiq	512.8926	527.5884	1281.172
1374708_at	306618	RGD1565C	similar to Rho guanine nucleotide exch	512.835	532.5439	1260.526
1396289_at	309659	Mdga1_pre	MAM domain containing glycosylphosp	512.8144	222.0539	440.7857
1378288_at	303879	RGD13111	similar to RIKEN cDNA D630035O19 (	512.6827	128.1968	99.28815
1368752_at	24808	Tacr3	tachykinin receptor 3	512.6461	91.27705	430.5503
1394727_at	362465	RGD15648	similar to ARG99 homolog (predicted)	512.3674	257.9349	372.2508
1387676_at	117016	Mark1	MAP/microtubule affinity-regulating kin	512.0683	884.7487	742.2668
1394039_at	84410	Klf5	Kruppel-like factor 5	511.8827	87.49613	4852.435
1381142_at	314587	Krim1	KRAB box containing zinc finger protei	511.8379	136.36	120.7109
1395105_at	362804	Cwf19l2_pr	CWF19-like 2, cell cycle control (S. po	511.1779	278.3837	340.3413
1383604_at	309051	Pign_predi	phosphatidylinositol glycan, class N (p	511.1107	766.6202	781.9579
1376973_at	311532	Sdcbp2	syndecan binding protein (syntenin) 2	511.0579	346.8698	342.719
1384544_at	312086	Pon3	paraoxonase 3	510.802	13.79326	740.6838
1377967_at	292071	Ris2_predi	retroviral integration site 2 (predicted)	510.4041	3097.193	306.5667
1393105_at	500989	RGD15621	similar to zinc finger protein (predicted	510.2321	323.139	282.9284
1395818_at	499110	LOC49911	NA	509.9787	379.6124	67.29518
1372426_at	310670	Adamtsl4	ADAMTS-like 4	509.9319	130.9204	647.8273
1372683_at	361300	Mocos_pre	molybdenum cofactor sulfurase (predic	509.7952	293.1662	261.3174
1387501_at	60324	Synpo	synaptopodin	509.5835	357.7323	102.6745
1368664_at	56764	LOC56764	dnaj-like protein	509.262	238.9667	294.3123
1380187_at	310674	RGD13114	similar to TNF intracellular domain-inte	509.0955	423.2458	486.2869
1394155_at	315634	Rbm7_pred	RNA binding motif protein 7 (predicted	508.978	837.5703	830.6101
1373204_at	297077	RGD13107	hypothetical LOC297077	508.9459	352.7651	2072.226
1374947_at	310838	Bcar3_pred	breast cancer anti-estrogen resistance	508.886	81.98823	193.8826
1375968_at	500214	RGD15596	similar to Catna2 protein (predicted)	508.5649	1697.222	2163.705
1387545_at	81522	Mtr	5-methyltetrahydrofolate-homocysteine	508.2417	57.94805	222.4788
1385617_at	312195	Nrf1_predi	nuclear respiratory factor 1 (predicted)	508.0108	280.4265	414.822
1368917_at	117260	Nudt1	nudix (nucleoside diphosphate linked r	507.9765	666.5878	296.8734
1370291_at	114108	Pdlim3	PDZ and LIM domain 3	507.9617	263.5085	559.4809
1390913_at	295671	NA	NA	507.712	405.5948	814.2208

1369104_at	65248	Prkaa1	protein kinase, AMP-activated, alpha 1	507.5451	275.0035	422.7897
1376037_at	300259	Aaas_pred	achalasia, adrenocortical insufficiency,	507.3794	912.9996	202.646
1373323_at	361120	LOC36112	similar to Epidermal growth factor rece	507.0637	516.9788	306.2416
1368748_at	170908	Tesk2	testis-specific kinase 2	506.3365	212.1566	469.0481
1367804_at	29339	Apcs	serum amyloid P-component	506.1796	204.2255	686.74
1367660_at	79131	Fabp3	fatty acid binding protein 3	506.1561	1271.601	322.4668
1371257_at	309288	Rorb_predi	RAR-related orphan receptor beta (pre	505.935	139.199	378.9552
1384788_at	304289	RGD13101	similar to KCCR13L (predicted)	505.7851	661.7757	357.7218
1393353_at	306504	RGD13048	similar to 6430573F11Rik protein (pred	505.6834	795.9577	602.0449
1392342_at	56816	Park2	parkin	505.6533	199.1681	342.4104
1372065_at	305235	Art3	ADP-ribosyltransferase 3	505.6458	127.1456	1140.967
1374017_at	365377	RGD15627	similar to tripartite motif protein 50 (pre	505.4477	544.0865	1698.21
1379648_at	307820	Nfat5_pred	nuclear factor of activated T-cells 5 (pr	505.2556	108.6476	724.678
1374488_at	300644	Gramd1b_	GRAM domain containing 1B (predicte	504.929	151.271	220.7595
1390879_at	301226	Apobec2_p	apolipoprotein B editing complex 2 (pre	504.4567	220.1483	316.4875
1387135_at	57025	Adam15	a disintegrin and metallopeptidase dom	503.9811	1504.714	5594.95
1382747_at	291377	Rttn_predic	rotatin (predicted)	503.9442	718.9703	302.8712
1376565_at	25638	Pde4a	phosphodiesterase 4A, cAMP specific	503.7009	487.4912	1597.859
1390107_at	361604	Sytl2_pred	synaptotagmin-like 2 (predicted)	503.3225	93.53728	2683.446
1376785_at	25561	Sycp3	synaptonemal complex protein 3	503.312	310.0657	962.3888
1385857_at	312108	Ophn1_pre	oligophrenin 1 (predicted)	503.2802	198.3536	256.8768
1394995_at	311088	Cobll1_pre	Cobl-like 1 (predicted)	502.8592	86.26648	773.0475
1378633_at	293783	Lpxn	leupaxin	502.7678	743.7361	395.8478
1390105_at	313536	B4galt2_pr	UDP-Gal:betaGlcNAc beta 1,4- galacto	502.4874	1261.128	664.1681
1380671_at	690093	LOC69009	NA	502.082	120.9483	172.1435
1383936_at	316736	Emilin2_pr	elastin microfibril interfacer 2 (predicte	501.6406	223.5677	237.7515
1389354_at	365960	LOC36596	similar to semaF cytoplasmic domain a	501.6357	2155.25	1633.568
1387923_at	24916	Zfp179	zinc finger protein 179	501.6253	2292.845	614.0731
1387187_a_at	116631	Nat1	N-acetyltransferase 1 (arylamine N-ac	501.4529	276.3351	334.1028
1393668_at	288369	Pex11c_pr	peroxisomal biogenesis factor 11c (pre	501.0494	445.3154	270.9749
1368654_at	25497	Npap60	nuclear pore associated protein	500.7057	1182.165	916.3577
1370153_at	29455	Gdf15	growth differentiation factor 15	500.4969	171.798	321.7078
1381993_at	294141	Clic2	chloride intracellular channel 2	500.338	4006.723	2330.767
1369359_at	24500	Il9r	interleukin 9 receptor	500.3071	206.4162	158.7436
1387414_at	79107	Duox2	dual oxidase 2	499.8522	140.6989	138.3018
1376081_at	296098	Lcmt2	leucine carboxyl methyltransferase 2	499.752	491.6055	244.1243
1391538_at	364388	Cideb_pred	cell death-inducing DNA fragmentation	499.5417	139.6222	184.6004
1384448_at	317486	RGD15658	similar to RIKEN cDNA 1700045I19 (p	499.2928	1813.587	2997.937
1398529_at	406160	Btnl8	butyrophilin-like 8	499.2871	211.3771	235.6483
1381789_at	287751	Lyzl6_pred	lysozyme-like 6 (predicted)	499.1438	423.5629	539.0684
1374211_at	501795	RGD15660	similar to Hypothetical protein LOC730	498.2793	926.1685	966.419
1377049_at	246246	Ntel1	neuropathy target esterase like 1	498.1578	57.20666	483.6551
1377090_at	367039	Dock6_pre	dedicator of cytokinesis 6 (predicted)	497.7097	793.8382	764.7781
1385071_at	363274	RGD15639	similar to transmembrane serine protea	497.2968	185.6906	275.5457
1368512_a_at	64017	Enpep	glutamyl aminopeptidase	497.2851	11.97331	7536.62
1393099_at	287621	Cox11_pre	COX11 homolog, cytochrome c oxidas	497.0849	367.146	36.90892
1376447_at	308341	RGD15657	similar to hypothetical protein A430110	496.8054	470.8659	297.6908
1386998_at	24191	Aldoc	aldolase C	496.748	448.3963	1023.598
1383617_at	306455	RGD13053	similar to RIKEN cDNA 4921511I16	496.6409	598.4985	5149.236
1377623_at	299613	Rkhd1_pre	ring finger (C3HC4 type) and KH doma	496.2787	1623.259	2064.294
1392520_at	58834	Dlc1	deleted in liver cancer 1	496.1172	191.4156	221.2968

1374527_at	298381	Echdc2_pr	enoyl Coenzyme A hydratase domain	496.0272	39.75637	949.7154
1371849_at	290558	Nt5dc2	5'-nucleotidase domain containing 2	495.9163	345.7242	862.6102
1383874_at	500640	RGD15608	RGD1560812 (predicted)	495.7012	257.9711	743.4417
1398252_at	29470	Mecr	mitochondrial trans-2-enoyl-CoA reduc	495.3028	1040.815	153.7665
1387642_at	155140	Il23a	Interleukin 23, alpha subunit p19	495.3005	225.423	358.0928
1397182_at	314393	LOC31439	NA	495.2039	394.6157	224.7567
1382197_at	293660	Rhod_pred	ras homolog gene family, member D (p	495.1232	758.1283	181.6499
1369624_at	63850	Prlh	prolactin releasing hormone	495.1034	298.4428	369.3277
1381049_at	313477	Zfyve9_pre	zinc finger, FYVE domain containing 9	494.9274	841.7432	796.2734
1370674_at	286915	LOC28691	putative pheromone receptor (Go-VN1	494.9215	26.01292	50.22546
1391902_at	24959	Pgam2	phosphoglycerate mutase 2	494.6398	260.6996	177.8299
1369438_at	64825	N5	DNA binding protein N5	494.6363	423.2442	499.226
1392677_at	315882	Zic4_predic	Zic family member 4 (predicted)	494.3699	276.5268	298.9825
1382458_at	305947	Kcnrg_pred	potassium channel regulator (predicted	494.3044	61.31463	120.6989
1398442_at	314484	RGD1311C	similar to chromosome 14 open readin	494.1693	1221.474	58.88211
1382180_at	314756	Cradd_pred	CASP2 and RIPK1 domain containing	494.153	763.7675	720.643
1392524_at	81002	Trim23	tripartite motif protein 23	494.1348	927.1827	1520.894
1394132_at	316976	MGC7297	Unknown (protein for MGC:72974)	494.0455	406.5691	107.9913
1370946_at	81524	Nfix	nuclear factor I/X	493.9675	189.731	248.3951
1369903_at	24922	Gabrb3	gamma-aminobutyric acid (GABA-A) re	493.8243	1917.327	360.3144
1391419_at	362617	Ahdc1_pre	AT hook, DNA binding motif, containin	493.6187	214.9341	568.8921
1369147_at	25690	Ahr	aryl hydrocarbon receptor	493.6169	26.5853	123.8393
1376871_at	288549	RGD15602	similar to cell surface receptor FDFAC	493.6048	192.3463	244.8748
1387874_at	24309	Dbp	D site albumin promoter binding protei	493.4823	1414.194	989.784
1379524_at	296618	Wdr34	WD repeat domain 34	493.2916	660.0481	562.8755
1374665_at	497958	RGD15637	similar to Gem (nuclear organelle) ass	493.2563	636.1142	125.5936
1372038_at	299618	Mknk2	MAP kinase-interacting serine/threonin	491.6851	1931.846	769.8562
1393336_at	293410	Swap70_pr	SWAP complex protein (predicted)	491.3361	526.6965	832.9187
1368756_at	64669	Thedc1	thioesterase domain containing 1	491.1074	584.8807	217.3401
1393092_at	308236	Zfp53_pred	zinc finger protein 53 (predicted)	491.0908	171.5614	382.5066
1368586_at	171449	Zg16	zymogen granule protein 16	490.8996	87.50747	1586.233
1368486_at	84021	Irs3	insulin receptor substrate 3	490.7946	75.99429	458.2214
1379790_at	311332	Dll4_predic	delta-like 4 (Drosophila) (predicted)	490.2699	168.2209	310.7618
1370567_at	24174	Adra2b	adrenergic receptor, alpha 2b	489.9572	64.77293	233.1744
1382419_at	294712	Solt_predic	SoxLZ/Sox6 leucine zipper binding pro	489.9025	5542.925	896.7877
1390001_at	306004	Rhobtb2	Rho-related BTB domain containing 2	489.838	178.7045	48.2229
1384056_at	84489	Fgfr3	fibroblast growth factor receptor 3	489.671	866.9281	3049.257
1382617_at	303419	RGD13048	similar to C330016O16Rik protein	489.5536	179.3883	526.2121
1388611_at	298559	Tcea3	transcription elongation factor A (SII), 3	488.5084	365.2322	230.7688
1378690_at	362935	Ly6a_predi	lymphocyte antigen 6 complex, locus A	488.1631	264.805	169.6714
1397025_at	84494	Trpc4	transient receptor potential cation char	487.9761	343.6544	268.7737
1379692_at	308501	Cd22_pred	CD22 antigen (predicted)	487.7705	360.2382	338.3222
1374697_at	310999	Plekhg5	pleckstrin homology domain containing	487.6489	485.6109	456.4563
1374812_at	498331	LOC49833	NA	487.4873	488.3537	2863.434
1368092_at	29383	Fah	fumarylacetoacetate hydrolase	487.0245	385.9117	1065.163
1370148_at	24464	Hp	haptoglobin	486.678	87.21729	134.0966
1395981_at	309887	Ascc3_pred	activating signal cointegrator 1 comple	486.6245	128.6209	202.0184
1372503_at	360548	Tnfsf12	tumor necrosis factor ligand superfami	486.5428	561.3551	2145.605
1386093_at	365192	Znf324_pre	zinc finger protein 324 (predicted)	486.5014	477.8934	407.087
1394523_at	297694	RGD13087	similar to RIKEN cDNA 1100001H23	486.181	134.2959	320.2667
1368202_a_at	79128	Dab2	disabled homolog 2 (Drosophila)	486.1375	172.1729	1050.379

1385519_at	362489	Cbfa2t1_pr	CBFA2T1 identified gene homolog (hu	485.7923	566.0592	58.32207
1376227_at	498440	RGD1561C	similar to myozenin 1 (predicted)	485.6555	224.2727	327.8542
1373749_at	362242	Snta1	syntrophin, acidic 1	485.42	269.8833	196.8115
1387270_at	79237	Hhex	hematopoietically expressed homeobo	485.3963	96.46808	4544.148
1368342_at	25095	Ampd3	adenosine monophosphate deaminase	485.237	23.69306	2785.803
1384965_at	54308	Slk	serine/threonine kinase 2	485.1481	2602.891	742.0927
1372125_at	298376	Gpx7_pred	glutathione peroxidase 7 (predicted)	485.0641	52.49978	693.5394
1380248_at	361764	Sema4g_p	sema domain, immunoglobulin domair	484.8225	352.5583	892.0421
1367849_at	25216	Sdc1	syndecan 1	484.3903	3371.107	319.2161
1378812_at	497944	RGD1565E	similar to expressed sequence AI8546	484.2493	395.9873	101.7393
1398721_at	301506	LOC30150	NA	484.1404	409.8431	305.5048
1381005_at	290295	NA	NA	484.1121	293.4419	311.1928
1371747_at	296470	RGD1310E	similar to RIKEN cDNA 2700038C09	483.948	4890.733	905.6874
1368111_at	171440	Abtb2	ankyrin repeat and BTB (POZ) domain	483.8099	206.6499	403.0647
1368633_at	64827	Crisp1	cysteine-rich secretory protein 1	483.4549	73.28296	32.23484
1368916_at	59085	Asl	argininosuccinate lyase	483.3801	547.2532	726.7769
1382038_at	362607	Kpna6	karyopherin (importin) alpha 6	483.3651	322.5464	265.3988
1375121_at	367100	Smad6_pre	MAD homolog 6 (Drosophila) (predicte	483.1664	306.8031	175.0242
1370216_at	25678	Ddr1	discoidin domain receptor family, mem	483.0604	491.6662	132.5753
1384354_at	293841	RGD15654	similar to X-linked lymphocyte regulate	482.9211	151.704	420.277
1387121_a_at	171114	Ndrg2	N-myc downstream regulated gene 2	482.3088	962.4455	473.716
1372494_a_at	362825	Hmg20b_p	high mobility group 20 B (predicted)	482.2689	1160.456	704.9243
1388144_at	25394	Bfsp1	beaded filament structural protein 1	482.1759	128.2706	356.6893
1373504_at	299783	Glipr1	GLI pathogenesis-related 1 (glioma)	481.8803	409.606	313.1759
1368583_a_at	171016	Hrg	histidine-rich glycoprotein	481.5513	143.0508	360.2513
1371762_at	25703	Rbp4	retinol binding protein 4, plasma	481.3683	28.22809	21995.46
1385148_at	25505	P2rx1	purinergic receptor P2X, ligand-gated i	481.2128	69.3774	507.103
1374143_at	366492	Epha2_pre	Eph receptor A2 (predicted)	481.0255	88.20786	735.7714
1371044_at	81744	Pde7a	phosphodiesterase 7A	480.9033	1360.258	926.7629
1392319_at	309208	RGD15649	similar to leucine rich repeat containing	480.8573	281.3997	71.40206
1396584_at	498281	RGD15598	similar to nectin 4 (predicted)	480.5929	50.24723	13.26201
1379059_at	288515	RGD13078	similar to FLJ23471 protein (predicted)	480.4482	395.8745	404.6819
1380515_at	361930	Bbs7	Bardet-Biedl syndrome 7	480.3248	484.0159	920.2925
1381171_at	364858	Stk32a_pre	serine/threonine kinase 32A (predicted	479.766	60.33217	126.5195
1388458_at	288003	Rfc4_predi	replication factor C (activator 1) 4 (pre	479.7064	6980.132	935.9348
1377920_at	294708	Sgtb	small glutamine-rich tetratricopeptide r	479.3969	356.8585	213.8427
1396494_at	89830	Ptch1	patched homolog 1 (Drosophila)	479.3924	157.9545	18.29932
1386120_at	500416	NA	NA	479.3918	446.9165	661.0971
1397618_at	360600	Spag9_pre	sperm associated antigen 9 (predicted	479.0512	1067.794	489.4281
1379734_at	362018	Mg29_prec	mitsugumin 29 (predicted)	478.6355	141.9292	333.4638
1367892_at	81530	Pdk2	pyruvate dehydrogenase kinase, isoer	478.3328	312.9617	279.7152
1386901_at	29184	Cd36	cd36 antigen	477.7065	187.8839	594.1879
1387011_at	170496	Lcn2	lipocalin 2	477.5078	37.76152	42.92131
1374354_at	499785	NA	NA	477.1436	1077.28	235.1915
1369061_at	116686	Gsr	glutathione reductase	477.1341	437.5945	122.3946
1369012_at	29200	Inhba	inhibin beta-A	476.8962	289.176	373.2026
1385217_at	80338	Zbtb10	zinc finger and BTB domain containing	476.8709	552.5499	5537.621
1394939_at	312381	Ppm1k_pre	protein phosphatase 1K (PP2C domai	476.4367	264.8468	332.2053
1377698_at	171369	Tnfrsf5	tumor necrosis factor receptor superfa	476.4109	27.89615	187.7057
1398719_at	292044	Cdyl2_prec	chromodomain protein, Y chromosome	476.0992	74.2871	242.3027
1376607_a_at	290811	RGD13104	similar to hypothetical protein FLJ2326	475.6713	937.0785	461.5449

1374198_at	315716	Cd276	CD276 antigen	474.6996	811.4397	1040.36
1390255_at	307395	RGD15651	similar to mKIAA0843 protein (predicte	474.3721	687.6731	1888.618
1378776_at	116545	Pou6f1	POU domain, class 6, transcription fac	473.7333	1167.906	1399.973
1389777_at	317431	Ribc1	RIB43A domain with coiled-coils 1	473.6759	414.4113	525.8557
1383567_at	366588	Colec11_p	collectin sub-family member 11 (predic	473.5493	190.5386	119.1835
1394320_at	362817	Cdk2	cyclin dependent kinase 2	473.4377	1241.565	405.3776
1390365_at	292535	Leng1_pre	leukocyte receptor cluster (LRC) meml	473.3672	257.993	460.8136
1388589_at	362831	Dot1l_pred	DOT1-like, histone H3 methyltransfera	473.0755	1143.135	1762.119
1387079_at	497757	Gucy1a3	guanylate cyclase 1, soluble, alpha 3	472.6262	115.1696	211.2869
1383678_at	362595	RGD13065	similar to RIKEN cDNA 1810007P19	472.5961	359.1597	299.124
1376207_at	287847	RGD15600	similar to ataxin 2-binding protein 1 iso	472.5506	262.3196	238.2778
1387387_at	29177	Hpca	hippocalcin	472.3781	570.2627	249.1357
1382439_at	311061	Itgb6	integrin, beta 6	472.3706	37.31043	981.9591
1368166_at	65142	Arl2	ADP-ribosylation factor-like 2	472.2992	532.9556	687.7848
1368013_at	140582	Ddit4l	DNA-damage-inducible transcript 4-like	472.2115	50.94667	155.5009
1371974_at	296621	Phyhd1	phytanoyl-CoA dioxygenase domain co	472.1863	310.4378	334.1356
1383380_at	291317	Rpp38	ribonuclease P/MRP 38 subunit (huma	472.0718	1327.767	799.5942
1370034_at	171103	Cdc25b	cell division cycle 25 homolog B (S. ce	471.9265	4548.232	153.4374
1368822_at	79210	Fstl1	follistatin-like 1	471.5857	33.43413	299.7221
1378841_at	292739	RGD13075	similar to CG16812-PA (predicted)	471.3566	531.5517	577.9216
1369494_a_at	25321	Ghrhr	growth hormone releasing hormone re	471.2236	183.9936	290.4307
1387068_at	54323	Arc	activity regulated cytoskeletal-associat	470.956	351.6446	892.7654
1387099_at	116564	Npr2	natriuretic peptide receptor 2	470.9345	154.5227	1290.222
1372658_at	308709	Dmn	desmuslin	470.7389	329.2858	374.4699
1388062_at	286997	Mapk15	mitogen-activated protein kinase 15	470.6516	463.7976	126.8283
1394949_at	502134	RGD15615	similar to TGFB-induced factor 2 (pred	470.5878	286.2047	442.6766
1387549_at	29633	Ndst1	N-deacetylase/N-sulfotransferase (hep	470.5799	499.4165	181.5969
1378745_at	78962	Per3	period homolog 3 (Drosophila)	470.4584	101.9745	7310.737
1370677_at	207119	Rin1	Ras and Rab interactor 1	470.2995	22.54143	58.35203
1396379_at	308431	RGD15611	similar to pleckstrin homology-like dom	470.1967	425.5325	272.9679
1398724_at	302946	Glis2_pred	GLIS family zinc finger 2 (predicted)	470.1721	352.417	336.3426
1378424_at	310641	Trim46_pre	tripartite motif protein 46 (predicted)	469.8548	922.4723	479.7866
1368949_at	116543	Ebf1	early B-cell factor 1	469.7581	311.9456	259.9484
1370297_at	25515	Plk1	polo-like kinase 1 (Drosophila)	469.6606	3555.21	94.34789
1385854_at	57033	Adam22	a disintegrin and metalloprotease dom	469.5739	221.8314	208.7829
1370103_at	84390	Hcn1	hyperpolarization-activated cyclic nucle	469.5569	399.6623	357.7209
1387180_at	117022	Il1r2	interleukin 1 receptor, type II	469.5139	2281.672	127.2373
1367984_at	56081	LOC56081	CTD-binding SR-like rA1	469.3552	369.1235	260.9899
1393820_at	681302	LOC68130	NA	469.3006	365.1698	109.4972
1387224_at	54248	Dgkb	diacylglycerol kinase, beta	469.2716	81.97961	1709.23
1396198_at	309060	RGD13107	similar to RIKEN cDNA A930008G19 (	468.9184	223.8548	234.3399
1383887_at	362220	RGD13069	similar to Protein C20orf103 precursor	468.6474	54.23143	40.8244
1372387_at	300751	RGD13118	hypothetical LOC300751 (predicted)	468.5918	399.1506	164.9533
1393466_at	362616	RGD15636	similar to EAPG6122 (predicted)	468.4846	298.9809	331.9417
1383519_at	25059	Hk2	hexokinase 2	468.1828	326.5566	398.7298
1368429_at	171152	Taf9l	TAF9-like RNA polymerase II, TATA bo	468.1355	915.0903	1165.469
1380727_at	304054	Prdm15_pr	PR domain containing 15 (predicted)	467.9865	177.4431	54.1576
1382868_at	361324	Sema6a_p	sema domain, transmembrane domain	467.9389	343.8996	348.2587
1381278_at	502885	RGD15596	similar to growth/differentiation factor (	467.7263	369.9317	670.5065
1378585_at	287776	RGD15641	similar to hypothetical protein MGC338	467.2531	78.96661	294.6723
1369777_a_at	171093	Shank2	SH3/ankyrin domain gene 2	467.2511	3631.324	2327.38

1372604_at	503164	LOC50316	hypothetical protein LOC503164	466.9482	235.8798	45.4032
1371468_at	312502	LOC31250	NA	466.8725	506.0994	440.7006
1369208_at	25647	Il7	interleukin 7	466.866	34.9168	644.1529
1389253_at	29142	Vnn1	vanin 1	466.7349	5.736757	759.8162
1391046_at	498295	LOC49829	similar to SET and MYND domain cont	466.6279	315.9512	811.5178
1395025_at	296757	NAPE-PLD	N-acyl-phosphatidylethanolamine-hydr	466.6017	169.3631	212.8891
1368460_at	65197	Slc2a5	solute carrier family 2, member 5	466.3946	911.429	358.5101
1381804_at	360551	RGD15631	similar to BAZF (predicted)	466.1847	213.1362	282.4075
1375619_at	84408	Cdh8	cadherin 8	465.7944	25.11681	576.343
1369680_at	171147	Slc2a13	solute carrier family 2 (facilitated gluco	465.6174	319.9321	72.54141
1387766_a_at	24710	Rbp2	retinol binding protein 2, cellular	465.5561	26.46325	43.75825
1387213_at	171085	Pcsk4	proprotein convertase subtilisin/kexin t	465.5136	506.0336	294.1952
1368315_at	85260	Entpd6	ectonucleoside triphosphate diphosph	465.3136	571.0566	178.4226
1392899_at	308761	Prc1_predi	protein regulator of cytokinesis 1 (pred	465.2467	12876.14	375.5309
1387250_at	29359	Pla2g10	phospholipase A2, group X	465.0329	300.5007	406.8751
1379700_at	313950	RGD13113	similar to HS1 binding protein 3 (predic	464.9541	247.9515	55.21158
1384445_at	298442	LOC29844	similar to RIKEN cDNA 0610037D15	464.5718	289.2918	290.2663
1390325_at	25668	Cd38	CD38 antigen	464.4525	352.3428	315.6564
1389546_at	65157	Amotl2	angiomotin like 2	464.1731	91.2048	651.4155
1377544_at	314386	Gpr68_pre	G protein-coupled receptor 68 (predict	463.9911	105.6193	297.8159
1390462_at	303542	RGD13088	similar to D11lgp1 (predicted)	463.985	304.9616	353.7388
1388006_at	207126	Muc13	mucin 13, epithelial transmembrane	463.9829	3075.892	69.47089
1374510_at	363453	Ppp1r3f_pr	protein phosphatase 1, regulatory (inhi	463.9222	530.7686	758.8437
1382770_at	503252	RGD1565C	similar to hypothetical protein MGC521	463.8315	286.6939	138.946
1387515_at	25264	Nmbr	neuromedin B receptor	463.2636	150.7838	614.9281
1376554_at	362917	Tatdn1_pre	TatD DNase domain containing 1 (pre	463.1711	282.055	1037.189
1374954_at	297453	Hdac11_pr	histone deacetylase 11 (predicted)	463.0223	396.5822	870.7387
1386933_at	171459	Gp2	glycoprotein 2 (zymogen granule mem	462.8832	106.7574	1267.575
1372672_at	293504	Qprt	quinolinate phosphoribosyltransferase	462.7844	321.8572	418.3177
1372427_at	298705	Raver1h	RAVER1 homolog (human)	462.7017	552.599	892.8215
1398024_at	316351	Npas2_pre	neuronal PAS domain protein 2 (predic	462.6054	59.28232	237.6171
1388784_at	307403	Csf1r	colony stimulating factor 1 receptor	462.5734	264.3836	330.2435
1398293_a_at	84022	Ghsr	growth hormone secretagogue recepto	462.4414	234.1104	237.245
1398295_at	63997	Slc29a1	solute carrier family 29 (nucleoside tra	462.2434	497.8898	977.1807
1387479_at	156726	Runx3	runt-related transcription factor 3	462.007	192.8425	218.2054
1371771_at	313641	LOC31364	perlecan	461.9745	226.6555	310.1355
1387184_at	29134	Axin2	axin2	461.8058	461.4881	205.9795
1395836_at	117242	Odz2	odd Oz/ten-m homolog 2 (Drosophila)	461.7073	714.1761	2152.064
1384886_at	500057	RGD15601	similar to hypothetical protein LOC168	461.6815	968.1732	2640.992
1395409_at	362102	Ppp2r4_pre	protein phosphatase 2A, regulatory su	461.2341	1888.775	350.0502
1381451_at	94339	Mmp23	matrix metallopeptidase 23	461.1628	300.2646	260.2321
1376962_at	363071	Stra6	stimulated by retinoic acid gene 6 hom	461.1244	201.3567	344.2356
1370904_at	294274	Hla-dma	major histocompatibility complex, class	461.0044	590.0619	207.038
1377397_at	298301	RGD15634	similar to hypothetical protein (predicte	460.8273	189.8186	161.2376
1387277_at	81515	Lyn	Yamaguchi sarcoma viral (v-yes-1) onc	460.2879	358.0321	401.1691
1378831_at	360840	RGD1566C	similar to KIAA0456 protein (predicted)	460.2264	181.181	279.4732
1393601_at	300034	Tigd5_prec	tigger transposable element derived 5	459.9975	194.6137	284.1941
1383860_at	25446	Fosl2	fos-like antigen 2	459.985	27.07017	688.9185
1398501_at	500111	RGD15618	similar to CDNA sequence BC027309	459.9658	243.1458	148.896
1396776_at	501909	NA	NA	459.9642	245.8876	105.577
1394913_at	361943	RGD15658	similar to OL-protocadherin isoform (pr	459.9091	421.1834	259.7588

1385420_at	303811	Dvl3_predi	dishevelled 3, dsh homolog (Drosophil	459.7033	186.9859	85.47119
1381393_at	303208	RGD13097	similar to sperm antigen HCMOGT-1	459.4021	76.12132	146.1019
1384797_at	309187	RGD13098	similar to RIKEN cDNA 5730596K20 (p	459.3556	1474.143	345.6566
1397211_at	498416	RGD15662	similar to Grb10 protein (predicted)	459.2733	30.12417	249.7345
1379250_at	313843	Galm	galactose mutarotase	459.0285	236.0403	284.4555
1393716_at	502421	RGD15595	similar to solute carrier family 35, mem	458.8675	210.9698	174.2887
1387390_at	29165	Gzmk	granzyme K	458.8356	324.5236	406.8706
1395123_at	363555	Wfikkn1_pr	WAP, follistatin/kazal, immunoglobulin	458.8249	364.0889	189.4174
1396628_at	502898	NA	NA	458.7597	270.3008	103.8032
1394033_at	29459	Rbbp9	retinoblastoma binding protein 9	458.4269	693.2836	572.6289
1386981_at	25027	Slc16a1	solute carrier family 16 (monocarboxyl	458.3598	375.7159	1818.433
1390222_at	305443	RGD13083	similar to RIKEN cDNA 2310079F23 (p	458.3217	461.0566	209.7689
1393443_a_at	290224	RGD13081	similar to CGI-112 protein	457.9462	465.7674	1217.532
1375209_at	303888	Osbpl11_p	oxysterol binding protein-like 11 (predi	457.9046	986.9189	501.5665
1369318_at	60398	Fhit	fragile histidine triad gene	457.7712	396.3829	772.8233
1382794_at	170501	Galnt10	UDP-N-acetyl-alpha-D-galactosamine:	457.6466	449.3028	309.7826
1374771_at	303019	RGD15649	similar to neuralized homolog (predicte	457.6083	7307.22	734.6183
1390918_at	361180	Grtp1	GH regulated TBC protein 1	457.0456	1601.385	162.3715
1374449_at	297594	Cdca3	cell division cycle associated 3	457.0053	4839.174	436.8776
1384842_s_at	246097	Tnfrsf6	Tumor necrosis factor receptor superfa	456.9993	457.646	407.4151
1393922_at	362549	RGD15618	similar to CDNA sequence BC020077	456.9202	990.3904	1680.527
1385061_at	500053	NA	NA	456.7727	861.3904	647.0635
1383371_at	367991	NA	NA	456.6798	566.8047	110.7877
1368156_at	60341	Camkk1	calcium/calmodulin-dependent protein	456.2609	1302.248	557.8242
1396575_at	367085	Arhgap20	Rho GTPase activating protein 20	456.0432	185.1653	406.8799
1385710_at	287034	Tnfrsf17_p	tumor necrosis factor receptor superfa	455.8645	192.6168	173.8536
1372841_at	362835	Reep6	receptor accessory protein 6	455.8585	3450.498	111.3518
1384804_at	444984	Dnmt3a	DNA methyltransferase 3A	455.803	519.9551	600.8888
1396825_at	304474	Pitpnm2_p	phosphatidylinositol transfer protein, m	455.2798	112.8913	113.2079
1387624_at	83586	Usf1	upstream transcription factor 1	455.2176	882.8552	286.5603
1385013_at	24881	Wnt1_map	wingless-type MMTV integration site fa	455.1597	156.3093	370.2988
1380830_at	499408	NA	NA	455.1508	175.5348	741.0778
1393030_at	291900	NA	NA	454.5514	1348.71	503.8267
1368254_a_at	170897	Sphk1	sphingosine kinase 1	454.4658	129.383	709.2431
1393982_at	299112	Pole2_prec	polymerase (DNA directed), epsilon 2	454.3093	1369.286	510.8539
1368990_at	25426	Cyp1b1	cytochrome P450, family 1, subfamily I	454.2873	271.506	353.7922
1369860_a_at	25187	Htr2c	5-hydroxytryptamine (serotonin) recept	454.1564	52.69885	80.78916
1385221_at	313063	RGD13065	similar to RAD54B homolog isoform 1;	454.0009	457.0331	290.4929
1370079_at	60414	Rhced	Rhesus blood group CE and D	453.973	213.3849	241.2909
1370239_at	25632	Hba-a1	hemoglobin alpha, adult chain 1	453.9144	26.34937	2830.743
1368466_a_at	29479	Odf2	outer dense fiber of sperm tails 2	453.7798	1629.702	171.1707
1368659_at	83784	Agxt2	alanine-glyoxylate aminotransferase 2	453.5206	74.44787	333.3521
1386899_at	25425	Ctsh	cathepsin H	453.4269	52.66113	10073.23
1376163_at	309082	Tmem12	transmembrane protein 12	453.2746	27.79178	432.3685
1378334_a_at	81678	Itpr2	inositol 1,4,5-triphosphate receptor 2	452.9054	178.0649	2165.545
1370038_at	60570	Rfrp	RFamide-related peptide	452.8902	275.8931	344.2038
1392685_at	315863	RGD13067	hypothetical LOC315863	452.8413	400.2063	369.0076
1368114_at	84488	Fgf13	fibroblast growth factor 13	452.6783	337.5517	405.052
1370544_at	192360	Eml2	echinoderm microtubule associated pr	452.3685	93.59144	365.9954
1378078_at	308387	Npas1_pre	neuronal PAS domain protein 1 (predic	452.2842	495.8498	443.5883
1390212_at	499974	RGD15609	similar to nucleoporin like 2 (predicted)	452.2193	1094.16	574.4337

1392120_at	498805	Thnsl1	threonine synthase-like 1	452.1297	831.4026	503.5001
1388836_at	81749	Prkch	protein kinase C, eta	452.0541	436.8138	212.2805
1376412_at	29629	Gria4	glutamate receptor, ionotropic, 4	451.827	237.2561	174.265
1377196_at	303205	Slc5a10_pı	solute carrier family 5 (sodium/glucose	451.5584	30.01788	224.992
1370049_at	83537	Smpd2	sphingomyelin phosphodiesterase 2, n	451.4118	499.4559	443.1086
1387036_at	29577	Hes1	hairy and enhancer of split 1 (Drosoph	451.377	454.0389	2519.85
1394982_at	302986	RGD1305	similar to 1200003M09Rik protein (pre	451.1998	45.0004	553.6003
1388661_at	289745	MGC9460	mitochondrial protein, 18 kDa	450.9266	263.7012	133.7282
1377354_at	499782	LOC49978	similar to 60S ribosomal protein L12	450.5878	445.4465	469.1414
1372657_at	315286	Tmem106c	transmembrane protein 106C	450.5255	1045.977	1404.886
1393452_at	313495	Car9_predi	carbonic anhydrase 9 (predicted)	450.0958	69.73577	94.58831
1370534_at	245921	Acvr1c	activin A receptor, type IC	449.4289	48.23346	22.30298
1367799_at	24799	Eef1a2	eukaryotic translation elongation factor	449.0189	5829.162	1736.42
1390251_at	362781	RGD1311	similar to KIAA1822 protein (predicted)	448.5858	333.765	172.4318
1384843_at	287557	Spaca3_pr	sperm acrosome associated 3 (predict	448.3417	140.694	67.99515
1383320_at	313050	Lck_mappe	lymphocyte protein tyrosine kinase (ma	448.0846	1376.992	429.8277
1375529_at	359725	Cbr4	carbonic reductase 4	448.0363	1682.895	1494.562
1383833_at	500991	LOC50099	hypothetical protein LOC500991	447.7703	168.4615	255.3864
1383143_at	313633	Ephb2_pre	Eph receptor B2 (predicted)	447.6005	470.155	819.8233
1393914_at	679519	LOC67951	NA	447.4529	628.7243	408.5849
1391503_at	361314	Centd3_pre	centaurin, delta 3 (predicted)	447.323	284.941	132.8274
1385411_at	303233	Usp43_pre	ubiquitin specific protease 43 (predicte	447.2906	704.72	283.7873
1389115_at	303687	Evpl_predi	envoplakin (predicted)	447.177	412.7714	750.6351
1395513_at	315708	Lsm16_pre	LSM16 homolog (EDC3, S. cerevisiae)	447.1049	386.4461	409.1632
1375715_at	259218	Rwdd1	RWD domain containing 1	446.5826	952.3047	16.61132
1390269_at	302552	Oatl1_pred	ornithine aminotransferase-like 1 (prec	446.5615	481.1901	185.8961
1381624_at	315666	Npat_predi	nuclear protein in the AT region (predic	446.1677	135.2384	908.0219
1387604_at	84359	Dffb	DNA fragmentation factor, beta subuni	446.1492	321.422	240.0052
1370609_a_at	29735	Slc16a7	solute carrier family 16 (monocarboxyl	445.8128	337.6311	709.3032
1370682_at	361493	Lilrb3	leukocyte immunoglobulin-like recepto	445.3176	179.9019	176.7484
1369102_at	25272	Mapk10	mitogen activated protein kinase 10	445.2252	516.7382	68.29452
1373486_at	360848	Tmem58_p	transmembrane protein 58 (predicted)	445.1848	931.4489	523.9238
1388484_at	296368	Ube2c_pre	ubiquitin-conjugating enzyme E2C (pre	445.0494	10552.1	595.648
1393832_at	311020	RGD1306	similar to RIKEN cDNA 0610033I05	444.9729	245.7358	767.4556
1370857_at	81633	Acta2	smooth muscle alpha-actin	444.7558	543.5754	334.5233
1370342_at	170899	Kcnk2	potassium channel, subfamily K, meml	444.4545	330.4282	242.7668
1383863_at	362176	Lmo2	LIM domain only 2	444.4366	355.4892	437.6615
1370065_at	58917	Hpx	hemopexin	444.1515	144.3094	42.86576
1374874_at	498386	RGD1561C	similar to RIKEN cDNA 5730509K17 g	443.6358	623.1491	4190.07
1368209_at	81916	Pdzk1ip1	PDZK1 interacting protein 1	443.3849	13402.61	918.664
1378057_at	366205	Flrt3_predi	fibronectin leucine rich transmembrane	443.2803	400.6747	759.4955
1379570_at	363563	RGD1562	similar to fibrillarin (predicted)	443.2173	91.1718	189.3764
1391679_at	366145	LOC36614	NA	442.9947	389.6882	119.8417
1375224_at	363989	Phlda3	pleckstrin homology-like domain, family	442.9706	333.5593	48.33628
1373658_at	315298	Racgap1_p	Rac GTPase-activating protein 1 (pred	442.7025	15190.47	1538.946
1381729_at	498675	LOC49867	NA	442.4499	170.1278	443.7575
1387585_at	65194	Slc29a2	solute carrier family 29 (nucleoside tra	442.4486	525.2027	182.7891
1369241_at	29462	Avpr1b	arginine vasopressin receptor 1B	442.1288	50.89804	1996.873
1383690_at	366473	LOC36647	similar to ornithine decarboxylase-like	441.7782	294.375	56.25831
1387611_at	24224	Bcl2	B-cell leukemia/lymphoma 2	441.7138	402.0214	105.4925
1369214_a_at	85483	C2ta	class II, major histocompatibility compl	441.5251	176.0666	293.5595

1387486_at	155812	Trim9	tripartite motif protein 9	441.3057	347.7866	334.8787
1376957_at	366507	Lzic	leucine zipper and CTNNBIP1 domain	441.0266	567.35	516.0289
1385673_at	287463	RGD13084	similar to hypothetical protein MGC418	440.9269	327.6827	200.3478
1370122_at	84590	Rab27b	RAB27B, member RAS oncogene fam	440.8308	204.6759	304.2175
1392427_at	500430	LOC50043	NA	440.7379	105.7179	110.9155
1376456_at	498252	NA	NA	440.7113	320.1538	136.493
1387586_at	64086	Csnk1g1	casein kinase 1, gamma 1	440.7074	67.14068	370.0353
1373773_at	306439	Gpm6a	glycoprotein m6a	440.5071	178.4304	8461.601
1387764_at	25410	Cebpe	CCAAT/enhancer binding protein , eps	440.2524	82.36568	199.663
1368195_at	171460	Hspbap1	Hspb associated protein 1	440.2369	768.5832	1091.632
1367859_at	25717	Tgfb3	transforming growth factor, beta 3	440.2045	47.52909	110.8885
1397383_at	500235	RGD1564C	similar to empty spiracles homolog 1 (p	440.133	157.8574	400.3965
1391960_at	302457	NA	NA	439.6372	58.8195	37.61769
1396983_at	289262	Fmn2_pred	formin 2 (predicted)	439.5556	28.70285	963.1114
1398347_at	308444	Axl	AXL receptor tyrosine kinase	439.4223	264.4012	590.5435
1397620_at	306417	Sh3md2	putative scaffolding protein POSH	439.3135	440.2054	961.6522
1369527_at	171056	Cx3cr1	chemokine (C-X3-C) receptor 1	439.2554	217.8927	107.9777
1378477_a_at	291964	LOC29196	NA	439.0878	112.0622	272.9819
1389421_at	304573	Pole_mapp	polymerase (DNA directed), epsilon (m	438.9498	1036.701	264.5071
1371029_at	24650	Pkd1	polycystic kidney disease 1 homolog	438.8692	268.2221	516.8277
1385558_at	363120	RGD15613	similar to hypothetical protein D93002	438.042	1147.015	582.5749
1370372_at	171099	Rasd2	RASD family, member 2	437.8682	56.93873	411.1132
1377916_at	303380	Slfn2_pred	schlafen 2 (predicted)	437.6882	109.0349	806.693
1374403_at	25186	Efnb1	ephrin B1	437.169	802.3384	506.1388
1389865_at	298443	Toe1_pred	target of EGR1, member 1 (nuclear) (p	437.0441	1438.269	70.79695
1378985_at	362681	LOC36268	similar to pseudouridylate synthase-lik	436.8547	173.9348	264.7783
1371284_at	362797	RGD15642	similar to immunoglobulin 4G6 heavy c	436.6994	103.6218	175.4635
1370672_a_at	171574	Dnm3	dynamin 3	436.5428	327.8433	98.85846
1388135_at	59102	Rpa2	replication protein A2	436.3864	3600.081	533.9302
1380037_at	170668	Rasgrp4	RAS guanyl releasing protein 4	436.2203	38.40832	109.1792
1393919_at	310849	RGD13057	similar to RIKEN cDNA 4933405A16	436.118	117.2706	274.7612
1368104_at	64521	Tspan2	tetraspanin 2	435.4926	588.5681	2155.097
1368382_at	114216	S100a3	S100 calcium binding protein A3	435.4488	141.7861	150.7776
1382589_at	361898	RGD13077	similar to hypothetical protein FLJ1179	435.2094	158.4972	195.4023
1376063_at	316526	Wnt6_pred	wingless-related MMTV integration site	435.1092	178.3183	351.4172
1380178_at	500922	NA	NA	434.9522	593.261	191.2506
1389755_at	619566	LOC61956	similar to transcription factor RAM2	434.2385	3757.806	1015.051
1382546_at	361051	Phf11	PHD finger protein 11	433.8117	171.6378	332.5434
1377940_at	287534	RGD13596	hypothetical LOC287534	433.7541	723.9348	372.316
1394731_at	362655	RGD15598	similar to casein kinase 1, gamma 3 (p	433.665	1393.381	486.4231
1370331_at	245983	Il11ra1	interleukin 11 receptor, alpha chain 1	433.4706	37.68046	765.0549
1370805_at	64466	Cited1	Cbp/p300-interacting transactivator wit	433.1625	1321.292	995.2633
1393254_at	301516	Stk36_pred	serine/threonine kinase 36 (fused hom	433.1416	255.7314	230.5763
1373163_at	315639	Usp28_pre	ubiquitin specific protease 28 (predicte	432.9044	943.3554	1124.623
1382122_at	303828	Etv5_predi	ets variant gene 5 (ets-related molecul	432.8571	199.9861	264.5778
1395434_at	362788	Traf3_pred	Tnf receptor-associated factor 3 (predi	432.7189	137.0841	194.0192
1398094_at	360506	RGD13113	similar to RIKEN cDNA 4930524B15 (p	432.6089	287.5357	286.9819
1372049_at	289753	RGD13118	similar to RIKEN cDNA 5730411O18 g	432.596	775.0544	25.91655
1385230_at	494219	Aer61	glycosyltransferase Aer61	432.5584	875.0918	416.515
1370881_at	25274	Tst	thiosulfate sulfurtransferase	432.452	239.4561	258.975
1372977_at	503027	RGD1562C	similar to mitogen-activated protein kin	432.4353	484.7597	610.3929

1372294_at	554172	Perc64	PE responsive protein c64	432.2638	1723.43	709.15
1384867_at	364147	RGD13107	similar to YIP1B (predicted)	432.1078	196.8702	180.8562
1397839_at	299156	Rab15	RAB15, member RAS onocogene fam	432.0952	342.3295	198.2432
1375725_at	363158	RGD15651	similar to Cartilage-associated protein	432.0316	194.4855	180.7508
1384960_at	24255	Cftr	cystic fibrosis transmembrane conduct	431.9788	71.6359	208.3848
1377639_at	286939	Asam	adipocyte-specific adhesion molecule	431.8467	128.7369	205.6212
1392008_at	301617	Pask	PAS domain containing serine/threonin	431.6906	216.7734	91.078
1370841_a_at	24391	Gh1	growth hormone 1	431.2878	46.73629	79.88097
1386598_at	308873	Art1_predic	ADP-ribosyltransferase 1 (predicted)	431.0981	364.4013	379.2041
1392128_at	303348	RGD15614	similar to novel protein (predicted)	430.995	1853.431	184.3771
1395550_at	307358	RGD15656	similar to collagen and calcium binding	430.965	188.7988	233.7306
1368921_a_at	25406	Cd44	CD44 antigen	430.6353	160.3681	472.8261
1389599_at	366698	Adck1_pre	aarF domain containing kinase 1 (pred	430.5164	456.5435	302.9626
1388109_at	245977	Gpr116	G protein-coupled receptor 116	430.3026	135.4558	800.9809
1387592_at	29414	Akt3	thymoma viral proto-oncogene 3	430.1515	259.7478	291.1163
1387986_at	24768	Scnn1g	sodium channel, nonvoltage-gated 1 g	430.1148	212.163	244.5512
1377881_at	308291	Phactr2	phosphatase and actin regulator 2	429.8924	576.6903	308.8155
1374772_at	498546	RGD15660	similar to Chromosome 13 open readin	429.8724	453.5611	308.6984
1385352_at	287451	Slc16a13	solute carrier family 16 (monocarboxyl	429.7019	77.83359	44.5749
1368860_at	29380	Phlda1	pleckstrin homology-like domain, family	429.5848	134.8941	171.9007
1371678_at	311840	RGD13065	similar to RIKEN cDNA 2900073H19 (	429.5649	517.3656	173.9647
1384627_at	501765	RGD15595	similar to RIKEN cDNA 2310057N15 (	429.2766	171.539	212.7958
1393400_at	301348	Zap70_ma	zeta-chain (TCR) associated protein ki	428.9453	76.16033	274.9014
1389378_at	361505	Cdc42ep5_	CDC42 effector protein (Rho GTPase	428.8817	190.265	386.3799
1368954_at	25097	Pld2	phospholipase D2	428.7276	181.9197	303.7365
1387027_a_at	25476	Lgals9	lectin, galactose binding, soluble 9	428.6425	276.2835	384.5589
1387379_at	25537	Rock2	Rho-associated coiled-coil forming kin	428.5664	533.989	260.0762
1390580_at	500934	NA	NA	428.3589	80.78871	69.695
1398276_at	64053	Dlgh2	discs, large homolog 2 (Drosophila)	428.2089	814.5445	371.8055
1386454_at	367298	Slc23a3_pr	solute carrier family 23 (nucleobase tra	428.1994	27.88563	569.0564
1391536_at	362324	RGD13084	similar to cDNA sequence BC020002 (	427.3851	944.9794	732.3549
1383490_at	315163	RGD15639	similar to Protein UNQ9166/PRO2863	426.5251	288.9273	181.1513
1383292_at	293733	Incenp_pre	inner centromere protein (predicted)	426.4981	1578.075	79.79646
1387981_at	170816	Olr59	olfactory receptor 59	425.72	252.8478	1496.286
1369330_at	64829	Unc13a	unc-13 homolog A (C. elegans)	425.6911	224.8835	113.6775
1377433_at	308336	Zfp580_pre	zinc finger protein 580 (predicted)	425.1832	603.1569	773.1497
1391810_at	309544	Rbm20_pre	RNA binding motif protein 20 (predicte	424.8353	358.5573	499.9446
1394492_at	498179	RGD15634	similar to hypothetical protein FLJ3866	424.5156	266.432	53.86426
1377405_at	304887	Ralgps2	Ral GEF with PH domain and SH3 bin	424.2454	768.2758	782.6314
1380031_at	171151	Slc25a21	solute carrier family 25 (mitochondrial	424.1876	252.9151	164.0398
1367964_at	29389	Tnni2	troponin I type 2 (skeletal, fast)	423.9734	208.7463	166.0583
1387893_at	192262	C1s	complement component 1, s subcomp	423.9384	98.48884	3845.063
1396056_at	307429	Dmxl1_pre	Dmx-like 1 (predicted)	423.3858	244.1903	268.3407
1385315_at	307845	RGD13097	similar to actin monomer-binding prote	422.5905	197.1292	274.4666
1398562_at	316412	Als2cr4_pr	amyotrophic lateral sclerosis 2 (juvenil	422.5358	757.9328	183.3216
1383897_at	361844	RGD15613	similar to macroH2A2 (predicted)	422.483	2033.29	413.4531
1394973_at	81742	Pde1c	phosphodiesterase 1C	422.2586	1376.996	561.9701
1381177_at	140935	Afap	actin filament associated protein	422.0795	94.19186	159.5116
1369333_a_at	116839	Rims2	regulating synaptic membrane exocyto	421.8967	386.0136	737.5524
1380243_at	313108	RGD13046	similar to CG14803-PA (predicted)	421.709	3931.532	222.4078
1395722_at	304833	Trove2_pre	TROVE domain family, member 2 (pre	421.7082	243.8621	298.3402

1394419_at	296060	RGD13091	similar to RIKEN cDNA 6530401L14 g	421.3841	15765.27	358.3822
1369159_at	24208	Ar	androgen receptor	421.3725	472.51	216.9144
1368532_at	84028	Pnliprp1	pancreatic lipase related protein 1	421.2158	29.52867	4246.291
1394537_at	317380	LOC31738	similar to Lmo6 protein	421.1564	192.8073	733.2296
1393513_at	117267	Slc26a2	solute carrier family 26 (sulfate transpo	420.9608	58.51948	169.1768
1368931_at	81921	Sh3gl3	SH3-domain GRB2-like 3	420.8356	635.7661	461.6339
1372682_at	362518	RGD13072	similar to RIKEN cDNA 2810432L12	420.2644	1176.884	677.579
1393215_at	362437	RGD13082	similar to intermediate filament-like pro	420.2254	260.734	152.7026
1389671_at	64573	Trpc2	transient receptor potential cation char	419.8391	1199.008	419.0918
1379196_at	498941	RGD15664	similar to F-box and leucine-rich repea	419.7698	319.3812	539.215
1368608_at	54246	Cyp2f2	cytochrome P450, family 2, subfamily f	419.5001	494.2271	312.2791
1389666_at	309201	Rom1	rod outer segment membrane protein 1	419.3114	637.0488	376.7532
1384023_at	317395	RGD15608	similar to Spindlin-like (predicted)	418.9602	681.8334	671.9267
1367791_at	58965	Ramp1	receptor (calcitonin) activity modifying	418.9069	255.9079	402.4939
1375018_at	311592	RGD15633	similar to hypothetical protein D630003	418.8544	238.2556	441.078
1383883_at	316624	RGD13096	similar to Hypothetical protein KIAA046	418.6755	283.1716	266.1418
1374704_at	315664	Kdelc2	KDEL (Lys-Asp-Glu-Leu) containing 2	418.5656	620.6929	729.9965
1381314_at	114588	Cdh4	cadherin 4	418.5067	212.237	344.1954
1367944_at	140568	Chst10	carbohydrate sulfotransferase 10	417.6179	1084.92	397.5694
1370787_at	64547	Bcl2l11	BCL2-like 11 (apoptosis facilitator)	417.5556	216.9056	255.2371
1385819_at	295237	Msto1_pre	misato homolog 1 (Drosophila) (predic	416.815	343.7813	329.1486
1398164_at	289615	Atp8a1_pre	ATPase, aminophospholipid transporte	416.7081	73.465	263.2335
1369024_at	80754	Rabep2	rabaptin, RAB GTPase binding effecto	416.3788	603.4923	152.5978
1384908_at	311349	Ttbk1_prec	tau tubulin kinase 1 (predicted)	416.207	297.6199	183.7467
1393724_at	362056	LOC36205	spermatogenesis associated 1	415.9076	306.3682	289.7163
1389757_at	304500	RGD13101	similar to acetyl-coA dehydrogenase -r	415.5131	369.9064	505.5184
1375100_at	494520	Ggnbp1	gametogenetin-binding protein 1	415.5092	137.4677	322.5377
1370374_at	170824	Steap3	STEAP family member 3	415.3689	283.0439	203.1805
1382410_at	288378	Cd209b	CD209b antigen	415.3647	121.4083	106.3777
1369857_a_at	54301	Slc14a1	solute carrier family 14 (urea transport	415.3551	250.1217	57.00368
1368978_at	64458	Scrg1	scrapie responsive gene 1	415.0944	126.6528	267.9234
1370940_at	115769	Tjp2	tight junction protein 2	415.0397	1928.27	1116.917
1385764_at	301114	LOC30111	NA	414.8895	175.036	73.30411
1372296_at	498066	RGD15635	similar to putative SH3BGR protein (pr	414.4646	202.4225	2281.059
1369124_at	29595	Htr2a	5-hydroxytryptamine (serotonin) recep	414.1907	208.6149	192.5596
1381783_at	312720	Akap3	A kinase (PRKA) anchor protein 3	414.0841	37.0276	246.8074
1370896_a_at	24582	Myh11	myosin, heavy polypeptide 11, smooth	413.9829	132.7398	234.2749
1384066_at	314168	RGD13076	hypothetical LOC314168 (predicted)	413.7999	287.2262	685.6272
1376755_at	24706	Rarb	retinoic acid receptor, beta	413.6944	83.44436	247.8016
1393646_at	297576	Dppa3	developmental pluripotency-associated	413.6792	61.39493	340.1161
1378589_at	502697	RGD15648	similar to prepro-endothelin-3 (predicte	413.491	1974.891	498.038
1370336_at	171493	Okl38	pregnancy-induced growth inhibitor	413.2726	133.8929	128.9833
1387349_at	25546	Shox2	short stature homeobox 2	412.6892	1923.04	206.0506
1368721_at	24209	Ascl2	achaete-scute complex homolog-like 2	411.8975	135.5293	58.36257
1387311_at	114243	Nox1	NADPH oxidase 1	411.7915	345.0982	286.6407
1369135_at	60568	Syt11	synaptotagmin XI	411.6688	319.9969	132.5172
1380222_at	83467	Slit3	slit homolog 3 (Drosophila)	411.4965	298.516	406.2989
1372536_at	360887	Cabc1	chaperone, ABC1 activity of bc1 comp	411.4776	273.0852	959.3231
1389959_at	54295	Rgs6	regulator of G-protein signaling 6	411.4579	287.2384	281.5735
1370836_at	246328	Serpina4	serine (or cysteine) proteinase inhibito	411.3064	281.7444	97.71059
1382869_at	309732	RGD13082	similar to hypothetical protein FLJ1454	411.231	234.941	160.0128

1381741_at	293498	RGD13066	hypothetical LOC293498 (predicted)	411.1062	207.5582	235.1511
1394008_x_at	313173	Cntfr	ciliary neurotrophic factor receptor	410.9884	547.3383	276.7701
1378220_at	497860	LOC49786	similar to RIKEN cDNA 4930517K11	410.9562	85.39488	690.321
1383891_a_at	287103	NA	NA	410.9276	2139.586	1040.493
1369167_at	25136	Gfra2	glial cell line derived neurotrophic facto	410.7844	96.0202	468.3543
1381139_at	498963	RGD15608	similar to RIKEN cDNA 2610039E05 (|	410.736	180.7241	1554.236
1389689_at	309596	Vars2l	valyl-tRNA synthetase 2-like	410.681	340.1941	417.8457
1383088_at	365314	RGD13052	similar to RIKEN cDNA 2610209A20 (|	410.6154	347.8312	183.984
1396592_at	367218	Runx2	runt related transcription factor 2	410.4299	84.18255	179.5851
1381663_at	361373	RGD15656	similar to cylindromatosis (turban tumc	410.2573	215.8851	210.3352
1394718_at	289095	Nmnat2_pr	nicotinamide nucleotide adenylyltransf	410.1608	188.637	650.7208
1368594_at	29387	Pla2g2c	phospholipase A2, group 2C	409.9531	42.21605	179.1275
1369236_at	170820	Prdm4	PR domain containing 4	409.9178	727.9367	178.3907
1368582_at	29485	Slc7a3	solute carrier family 7 (cationic amino a	409.8424	121.0977	80.55295
1374643_at	499600	NA	NA	409.7563	262.4563	349.4047
1398746_at	311530	Nanp	N-acetylneuraminic acid phosphatase	409.724	1452.351	372.0375
1375911_at	362219	RGD73514	hypothetical protein LK44	409.4793	270.4299	1587.715
1367978_at	81636	Adcy2	adenylate cyclase 2	409.2907	184.1248	760.0566
1371877_at	497857	RGD15649	similar to MYLE protein (Dexamethasc	409.2458	288.0231	618.9979
1368182_at	117243	Acsl6	acyl-CoA synthetase long-chain family	409.2414	1044.229	804.0211
1373324_at	360580	Dusp14_pr	dual specificity phosphatase 14 (predic	409.0557	504.0813	1110.935
1392970_at	300967	RGD13115	similar to mucin 7, salivary (predicted)	408.5875	190.2234	333.0247
1369708_a_at	81646	Creb1	cAMP responsive element binding prot	408.4166	353.4079	95.7966
1375060_at	366505	NA	NA	408.386	1358.428	776.5952
1397797_at	309174	Tigd3_prec	tigger transposable element derived 3	408.0042	970.8045	1025.704
1368435_at	81924	Cyp8b1	cytochrome P450, family 8, subfamily I	407.9612	208.0101	50.18789
1376659_at	299008	Nubpl_prec	nucleotide binding protein-like (predict	407.6493	146.7762	351.9402
1388936_at	84407	Cdh11	cadherin 11	407.4318	1000.574	1023.226
1381223_at	502579	NA	NA	407.1756	120.2637	173.6682
1378792_at	500633	RGD15640	similar to Kiaa0575 (predicted)	406.9823	15.97613	46.5613
1393706_at	297738	Steap1_pre	six transmembrane epithelial antigen c	406.978	220.9253	239.6752
1392737_at	117029	Ccr5	chemokine (C-C motif) receptor 5	406.8799	196.1645	321.0238
1387846_at	114493	Cacna1f	calcium channel, voltage-dependent, a	406.8651	28.63598	50.3686
1392043_at	24954	Insr	insulin receptor	406.7438	125.6064	822.2907
1395379_at	302670	RGD15597	similar to U2af1-rs2 (predicted)	406.6348	1015.027	361.8154
1373407_at	315203	RGD13099	similar to hypothetical protein 9930016	406.3365	1059.143	887.5838
1398254_at	81759	Renbp	renin binding protein	406.2185	86.42055	34.75866
1367973_at	24770	Ccl2	chemokine (C-C motif) ligand 2	405.9782	7.049029	8454.774
1389371_at	317189	Diap2_prec	diaphanous homolog 2 (Drosophila) (p	405.6664	1110.639	572.4043
1371114_at	116649	Kcnj4	potassium inwardly-rectifying channel,	405.4736	355.9343	256.1424
1389533_at	282583	Fbln2	fibulin 2	405.4719	268.2929	245.3039
1389099_at	361519	RGD15648	similar to hypothetical protein MGC510	405.4686	588.416	213.7993
1387490_at	64679	Tgm4	transglutaminase 4 (prostate)	405.4116	189.5318	40.99555
1370393_at	192228	Ccdc5	coiled-coil domain containing 5	405.209	3384.305	126.3606
1391013_at	64865	Pcdh8	protocadherin 8	405.1191	209.4507	3394.231
1375071_at	292085	Nup133_pr	nucleoporin 133 (predicted)	404.7869	551.6297	552.9754
1369744_at	24826	Tg	thyroglobulin	404.5999	75.79615	253.2465
1384923_at	300990	RGD13074	similar to RIKEN cDNA 6430571L13 g	404.1374	243.8762	433.4639
1379999_at	362427	Mical3	microtubule associated monoxygenase	403.7915	405.2532	619.1887
1374805_at	299933	RGD15617	similar to hypothetical protein MGC552	403.5565	5017.914	754.0358
1370392_at	171143	Trpm4	transient receptor potential cation char	403.3254	79.52641	115.2794

1382905_at	498011	Endo180	collagen-binding factor Endo180	403.1183	2105.628	730.6964
1370444_at	257648	Cacna1b	calcium channel, voltage-dependent, N	402.9925	1518.117	689.1253
1396832_at	366992	Kb15	type II keratin Kb15	402.9775	273.0592	288.1262
1387677_at	29589	Pou3f4	POU domain, class 3, transcription fac	402.7588	39.13395	359.7116
1376279_at	315045	Pop1_pred	processing of precursor 1, ribonucleas	402.7331	722.5335	179.1199
1385756_at	306251	Itih1_predi	inter-alpha trypsin inhibitor, heavy chai	402.5955	199.8699	1225.435
1385813_at	291675	RGD13102	similar to RIKEN cDNA 2010001M09	402.4927	171.1099	52.19828
1390838_at	501145	RGD15623	similar to KIAA1034-like DNA binding p	402.2846	206.1437	465.876
1369262_at	64044	Casp8	caspase 8	402.02	52.05602	209.3438
1369747_at	116632	Nat2	N-Acetyltransferase-2 (arylamine N-ac	401.3986	194.3607	245.4053
1367896_at	54232	Ca3	carbonic anhydrase 3	401.2813	109.0725	175.6491
1368284_at	56765	Plvap	plasmalemma vesicle associated prote	401.2319	264.9278	648.5207
1397952_at	365770	RGD15616	similar to novel protein (predicted)	401.1414	332.6733	431.713
1385277_x_at	303538	RGD13100	similar to hypothetical protein FLJ1135	401.1389	559.1398	485.6248
1368015_at	59103	Ptges	prostaglandin E synthase	400.9555	307.6015	284.6744
1381758_at	500870	RGD15656	similar to MDM2 Binding protein (predi	400.9108	889.6264	215.8521
1381836_at	502099	NA	NA	400.7535	224.3198	171.175
1378997_at	312275	Ephb6	Eph receptor B6	400.0199	386.4182	234.7332
1387593_at	171074	Pwwp1	hepatoma-derived growth factor, relate	399.9346	294.5069	328.8084
1395970_at	292684	Dmwd	dystrophia myotonica-containing WD r	399.8948	336.3156	282.1428
1368193_at	29440	Slc26a4	solute carrier family 26, member 4	399.8122	204.8619	67.75044
1368616_at	24361	Fancc	Fanconi anemia, complementation gro	399.7312	326.632	81.84813
1382176_at	287706	Klhl11_pre	kelch-like 11 (Drosophila) (predicted)	399.7149	104.8285	104.0565
1369015_at	24598	Nos1	nitric oxide synthase 1, neuronal	399.6843	251.9078	48.15594
1391512_at	317296	Mtmr1_pre	myotubularin related protein 1 (predicte	399.55	4839.481	134.0051
1397032_at	304074	Morc3_pre	microrchidia 3 (predicted)	399.0911	149.5775	129.2967
1390547_at	287920	St6galnac1	ST6 (alpha-N-acetyl-neuraminyl-2,3-be	399.0782	252.9076	289.236
1368186_a_at	25155	Syk	spleen tyrosine kinase	398.9836	306.1289	497.5463
1391727_at	501538	NA	NA	398.7714	3419.374	2240.479
1368851_at	24356	Ets1	v-ets erythroblastosis virus E26 oncog	398.7596	99.84777	746.5329
1368260_at	114592	Aurkb	aurora kinase B	398.4845	4401.208	440.6587
1369862_at	24649	Pim1	proviral integration site 1	398.4705	69.98074	191.9008
1377771_at	500258	RGD15602	similar to chromosome 3 open reading	398.222	123.5796	393.288
1387991_at	170808	Capn8	calpain 8	397.9367	203.6437	318.9783
1371500_at	292734	Ltbp4	latent transforming growth factor beta l	397.8501	51.82479	706.8898
1387209_at	89868	Lztr2	leucine zipper transcription regulator 2	397.5568	142.8009	416.4712
1393875_at	290959	Slc35d2_pr	solute carrier family 35, member D2 (p	397.3957	260.4183	463.1768
1396892_at	502211	RGD15637	similar to putative pheromone receptor	397.3133	211.0379	571.897
1398065_at	309860	RGD15604	similar to RIKEN cDNA 5330439J01 (p	396.8941	90.60451	70.04293
1395603_at	292625	RGD15654	RGD1565487 (predicted)	396.8257	67.66475	1257.742
1398552_a_at	500316	Acrbp	acrosin binding protein	396.5426	331.5247	985.4552
1388600_at	301295	LOC30129	NA	396.4613	679.2059	578.273
1397825_at	368062	LOC36806	NA	396.2201	773.0201	163.817
1392695_a_at	309070	Uros	uroporphyrinogen III synthase	395.8862	94.48295	56.25795
1369191_at	24498	Il6	interleukin 6	395.8725	42.60153	5100.568
1381512_at	309207	Nsddr	neural stem cell-derived dendrite regul	395.7798	58.17033	67.46265
1383243_at	24252	Cebpa	CCAAT/enhancer binding protein (C/E	395.7239	340.0188	874.1684
1369514_at	60663	Insrr	insulin receptor-related receptor	395.7157	217.1737	308.0219
1372029_at	316130	LOC31613	NA	395.658	146.2447	89.57846
1396383_at	309147	Aldh3b1	aldehyde dehydrogenase 3 family, me	395.6464	174.3155	379.1023
1372406_at	316273	Mcm3_pre	minichromosome maintenance deficier	395.6241	4611.589	260.1254

1370743_a_at	171105	Lnpep	leucyl/cystinyl aminopeptidase	395.5476	170.492	107.2873
1387202_at	25464	Icam1	intercellular adhesion molecule 1	395.4695	23.16277	657.1134
1390790_a_at	291437	Lipg	lipase, endothelial	395.3747	69.80551	122.5431
1383217_at	304761	RGD15649	similar to zinc finger, RAN-binding dom	395.27	484.7736	538.7348
1385783_at	498630	RGD15594	similar to BH3-only member B protein	395.2042	249.4897	266.0992
1373606_at	363253	Zfand2b	zinc finger, AN1 type domain 2B	395.1135	493.4639	484.6625
1384771_at	287564	RGD15655	similar to hypothetical protein (predicte	394.5676	184.7549	116.1352
1390987_at	498274	RGD15652	similar to novel protein (predicted)	394.4019	293.7427	888.2311
1368987_at	116638	Slc17a7	solute carrier family 17 (sodium-depen	394.3437	258.7501	167.9957
1393954_at	308854	Chrdl2_pre	chordin-like 2 (predicted)	394.3224	39.78731	84.02949
1382831_at	305477	Dusp18	dual specificity phosphatase 18	394.2277	435.6137	481.3702
1385114_at	499917	RGD15640	similar to Protein C20orf46 (predicted)	393.9893	214.55	140.205
1373287_at	500200	RGD15615	similar to bHLH factor Math6 (predicte	393.9017	129.694	58.0954
1369463_at	25689	Htr5a	5-hydroxytryptamine (serotonin) recep	393.7846	139.9058	174.9107
1395470_at	362240	RGD15635	similar to RIKEN cDNA 8430427H17 g	393.6637	222.0822	516.8708
1390374_at	360903	Fgfrl1	fibroblast growth factor receptor-like 1	393.4316	88.05855	65.58223
1382664_at	360643	Cdc27	cell division cycle 27 homolog (S. cere	393.4211	434.6022	318.8335
1369043_at	25469	Kcna4	potassium voltage-gated channel, shal	392.7196	392.0941	340.3345
1387266_at	140941	Siah1a	seven in absentia 1A	392.6438	611.21	1410.845
1368423_at	81712	Retnla	resistin like alpha	392.6424	202.7631	246.6038
1385398_at	295248	Zbtb7b_pre	zinc finger and BTB domain containing	392.415	131.945	112.3792
1389767_at	302982	RGD13049	similar to hypothetical protein FLJ3136	392.3492	233.7624	57.32616
1382986_at	29653	Gpam	glycerol-3-phosphate acyltransferase,	392.1359	313.8672	225.698
1394100_at	292794	Gpr43	G protein-coupled receptor 43	392.1096	75.75101	81.53662
1373795_at	502478	NA	NA	392.0563	276.67	391.483
1369128_at	24407	Grik5	glutamate receptor, ionotropic, kainate	391.9963	681.8374	465.8177
1377540_at	316229	RGD15595	similar to RP3-330M21.4 (predicted)	391.8737	175.8322	520.3245
1371269_at	366456	Olr857_pre	olfactory receptor 857 (predicted)	391.8306	250.3128	97.42415
1397481_at	297408	Alms1_pre	Alstrom syndrome 1 (predicted)	391.7835	139.6651	426.0047
1374277_at	303059	LOC30305	similar to novel protein	391.7352	283.9566	268.9149
1384560_x_at	298352	Rhox9	reproductive homeobox on X chromos	391.6584	232.4157	203.974
1383582_at	362605	Tmem54	transmembrane protein 54	390.7484	123.8221	64.17169
1395299_at	287675	Ka24	type I keratin KA24	390.6225	19.78557	119.7092
1376549_at	59320	Adcy10	adenylate cyclase 10 (soluble)	390.4822	97.92697	496.5989
1378772_at	79211	Gabrg3	gamma-aminobutyric acid (GABA) A re	390.2027	166.6322	198.6995
1395488_at	499505	Msh3	mutS homolog 3 (E. coli)	390.1899	682.2203	713.6137
1368698_at	24215	Atp2b2	ATPase, Ca++ transporting, plasma m	390.073	1246.075	461.3383
1376890_at	406868	G4	G4 protein	389.9987	17.0498	201.0878
1381300_at	498942	NA	NA	389.9488	212.7166	194.3351
1369644_at	171447	Lphn2	latrophilin 2	389.738	1010.262	449.045
1390884_a_at	316583	B3gnt7	UDP-GlcNAc:betaGal beta-1,3-N-acet	389.7075	233.8226	216.9592
1370347_at	286908	Pdlim7	PDZ and LIM domain 7	389.5184	1490.725	376.0857
1394599_at	362399	RGD15612	similar to cDNA sequence BC022133 (	389.498	482.5517	634.647
1373128_at	494125	Rcn3_pred	reticulocalbin 3, EF-hand calcium bind	389.4568	75.69727	1250.278
1373537_at	192348	Fnbp1	formin binding protein 1	389.142	431.9624	503.6846
1389484_at	366013	Coq4	coenzyme Q4 homolog (yeast)	388.9754	493.2048	379.7697
1390944_at	361916	MGC10877	Snf7 homologue associated with Alix 3	388.8848	39.46388	512.1796
1370962_at	286894	Cipar1	castration induced prostatic apoptosis-	388.8747	1127.827	557.7747
1369538_at	116671	Cdk5r1	cyclin-dependent kinase 5, regulatory s	388.8549	219.4763	25.97947
1374303_at	304578	Alkbh2_pre	alkB, alkylation repair homolog 2 (E. co	388.8482	559.2511	293.4843
1382490_at	301113	LOC30111	NA	388.7342	476.2613	292.7579

1368334_at	84427	Grb7	growth factor receptor bound protein 7	388.7174	897.6846	392.6794
1382709_at	498287	RGD15618	similar to lymphocyte antigen 108 isofo	388.6643	272.4315	426.4419
1392451_at	362659	NA	NA	388.6608	251.1562	215.5065
1394243_at	498273	RGD15599	similar to spermine synthase (predicte	388.6596	931.0523	567.3765
1368237_at	64104	Tnmd	tenomodulin	388.6003	177.653	301.9181
1375975_at	312667	RGD13107	similar to KIAA1074 protein (predicted)	388.5789	300.8478	553.1473
1379055_x_at	361869	RGD15650	similar to Rpl17 protein (predicted)	388.5065	22.03497	524.3921
1398542_at	295283	RGD13084	similar to hypothetical protein MGC467	388.4582	130.4672	231.6595
1398809_at	83836	Nde1	nuclear distribution gene E homolog 1	388.1444	255.9567	760.1387
1376368_at	294009	Cuedc2_pr	CUE domain containing 2 (predicted)	388.1194	1906.074	272.251
1387720_at	171394	Clstn2	calsyntenin 2	388.0964	435.2283	78.83686
1375419_at	361588	Lrrc28_pre	leucine rich repeat containing 28 (pred	387.9854	116.5651	303.0009
1379604_at	315108	Apol3	apolipoprotein L, 3	387.9243	351.4795	347.932
1391633_at	314903	RGD15662	similar to SLIT-ROBO Rho GTPase-ac	387.7183	40.64235	403.985
1384208_at	360922	Igj	immunoglobulin joining chain	387.6999	82.60779	246.9666
1368084_at	25633	Dnase1	deoxyribonuclease I	387.642	194.7235	32.84841
1382625_at	295496	LOC29549	NA	387.4233	549.7432	423.6004
1395037_at	500577	RGD15634	similar to RIKEN cDNA 2900090M10 (	387.2647	112.104	140.7857
1379955_at	313705	RGD13058	similar to KIAA1337 protein (predicted)	387.1889	165.2753	182.9721
1388624_at	499780	RGD15611	similar to Hypothetical UPF0184 protei	387.0749	2900.888	474.1203
1394359_at	364500	NA	NA	387.0585	1349.151	644.445
1387457_at	64014	Dusp12	dual specificity phosphatase 12	386.9264	425.6816	560.5216
1376695_at	293452	RGD13047	similar to hypothetical protein FLJ2181	386.7446	290.9901	225.2087
1376199_at	364666	Ccrk	cell cycle related kinase	386.6372	936.643	395.3075
1369548_at	83830	Gtf2a1	general transcription factor 2a, 1	386.6263	271.7842	338.1353
1380385_at	500063	NA	NA	386.3038	901.3063	1279.157
1376732_at	364529	Calr3	calreticulin 3	386.0957	113.5782	306.664
1396034_at	307660	LOC30766	carboxylesterase 615	386.0722	146.9163	199.3182
1371185_at	114517	Itga6	integrin, alpha 6	386.0626	218.1383	143.5805
1384667_x_at	29234	Galr2	galanin receptor 2	386.0545	414.5189	176.5198
1387066_a_at	54292	Rgs12	regulator of G-protein signaling 12	385.8912	503.6686	248.8331
1371066_at	170837	Snrk	SNF related kinase	385.7353	297.2514	98.79348
1393041_at	362519	Smc2l1_pr	SMC2 structural maintenance of chron	385.4604	3388.367	731.5937
1370127_at	59294	Pold1	polymerase (DNA directed), delta 1, ca	385.2781	1279.625	69.12761
1378526_at	297076	Gimap6	GTPase, IMAP family member 6	385.2682	263.1765	318.4222
1376026_at	288257	Donson	downstream neighbor of SON	384.7392	1755.346	1445.215
1368699_at	171119	Dscam	Down syndrome cell adhesion molecul	384.7043	291.819	460.6656
1382136_at	501925	LOC50192	NA	384.3115	276.175	374.797
1380293_at	361346	LOC36134	similar to chromosome 18 open readin	384.2871	1760.841	892.3912
1387934_at	25393	Bcan	brevican	384.0921	318.214	146.8837
1391621_at	314818	E2f7_predi	E2F transcription factor 7 (predicted)	383.9029	151.0324	429.8179
1384386_at	25597	Rasa2	RAS p21 protein activator 2	383.5965	160.2878	214.7122
1389659_at	498690	RGD15655	similar to ctla-2-beta protein (141 AA) (	383.3371	62.13913	381.4996
1388856_at	60427	Kitl	kit ligand	383.2699	652.189	2906.021
1376912_at	287388	RGD13103	hypothetical LOC287388 (predicted)	383.0462	468.7254	612.8026
1376120_at	362090	RGD13086	similar to pancreatitis-induced protein 4	382.9498	920.3765	533.2535
1387621_at	25572	Tnfrsf4	tumor necrosis factor receptor superfa	382.935	192.883	77.81748
1373386_at	394266	Gjb2	gap junction membrane channel protei	382.9335	25.55795	209.1986
1375206_at	287466	RGD13082	hypothetical LOC287466	382.7095	1204.504	691.7956
1385692_at	305309	Slc10a4	solute carrier family 10 (sodium/bile ac	382.4306	100.0452	129.4345
1368265_at	171380	Cyp2t1	cytochrome P450 monooxygenase CY	382.4168	19.61717	89.85449

1387396_at	84604	Hamp	hepcidin antimicrobial peptide	382.1162	178.4488	630.2854
1368964_at	81514	Lrrn3	leucine rich repeat protein 3, neuronal	381.8747	1470.268	9287.022
1380091_at	286998	Anubl1	testis-specific gene including a ubiquiti	381.6847	648.0789	862.9722
1375596_at	361586	Trpm1	NA	381.5523	427.2761	352.2179
1393844_at	306464	LOC30646	similar to myeloid leukemia factor 1 int	381.1294	1939.133	177.4511
1395517_at	316101	Limd1_pre	LIM domains containing 1 (predicted)	381.0482	324.6101	166.7363
1380262_at	171497	Sgk2	serum/glucocorticoid regulated kinase	380.8378	166.9898	260.2659
1383985_at	361432	Cdh15	cadherin 15	380.5582	298.5937	364.2395
1376082_at	294924	Evi1_predi	ecotropic viral integration site 1 (predic	380.4982	2502.481	1102.481
1371011_at	266778	Casrl1	calcium-sensing receptor like 1	380.4971	84.80204	357.8222
1382626_at	497925	NA	NA	380.4823	1005.426	267.4069
1383817_at	360987	NA	NA	380.2891	136.1703	642.5744
1383657_at	296947	Fscn3	fascin homolog 3, actin-bundling prote	380.2215	186.4257	368.2333
1393653_at	501174	RGD15607	similar to putative protein product of HI	380.1901	103.1555	699.2666
1387747_at	29585	Gjb3	gap junction membrane channel protei	380.0395	63.63217	36.57361
1376963_at	314862	Dyrk2_prec	dual-specificity tyrosine-(Y)-phosphory	379.9916	832.3556	1507.117
1368923_at	60417	Ecel1	endothelin converting enzyme-like 1	379.7492	142.4239	165.8303
1374328_at	499213	NA	NA	379.6806	552.6936	762.5002
1397867_at	64553	Akap6	A kinase (PRKA) anchor protein 6	379.5879	208.215	339.9056
1369773_at	25667	Bmp3	bone morphogenetic protein 3	379.5337	772.4424	134.0516
1395929_at	365511	LOC36551	NA	379.1381	275.8803	200.1992
1374606_at	310194	Myst1	MYST histone acetyltransferase 1	378.8302	534.4109	645.4898
1376008_at	303731	Cbx8	chromobox homolog 8 (Drosophila, Pc	378.5461	410.7559	272.2325
1369679_a_at	25492	Nfia	nuclear factor I/A	378.4499	400.1116	884.5052
1381098_at	500670	NA	NA	378.3707	76.0968	83.91511
1376970_at	361822	RGD13087	similar to RIKEN cDNA A130042E20; (	378.3085	309.9456	90.32639
1389374_at	307644	Kifc3	kinesin family member C3	378.0451	1851.498	954.1233
1397853_s_at	66013	Arhgef9	Cdc42 guanine nucleotide exchange fa	378.0056	617.0222	1354.89
1368210_at	170819	Il24	interleukin 24	377.8982	78.21578	85.65752
1369336_at	60563	hr	hairless homolog (mouse)	377.6097	222.4717	223.4871
1379663_at	298267	Cachd1_pr	cache domain containing 1 (predicted)	377.5405	1789.558	2375.417
1392563_at	315189	Arhgap8_p	Rho GTPase activating protein 8 (pred	377.119	538.1573	61.03269
1397836_at	304539	Sirt4_predi	sirtuin (silent mating type information r	376.9786	614.5149	250.3654
1370255_at	50683	Sftpc	surfactant associated protein C	376.8629	171.6427	202.4169
1383808_at	362969	Ttll1	tubulin tyrosine ligase-like 1	376.7534	374.9369	480.0922
1372660_at	306599	Tubgcp3	tubulin, gamma complex associated pr	376.6039	988.4158	403.2071
1385332_at	366593	Dus4l_prec	dihydrouridine synthase 4-like (S. cere	376.4119	238.333	207.2564
1375818_at	300803	Lactb_prec	lactamase, beta (predicted)	376.3755	210.3106	255.5362
1397121_at	415057	RT1-T24-2	RT1 class I, T24, gene 2	376.019	153.4661	106.8709
1380450_at	287609	Bzrap1	benzodiazapine receptor associated pr	376.0063	277.8452	269.6174
1387587_at	25385	Faslg	Fas ligand (TNF superfamily, member	375.9984	221.4462	260.3324
1381802_at	501808	RGD15604	similar to Leukosialin precursor (Leuco	375.8518	79.59558	89.00665
1378807_at	50592	Gria1	glutamate receptor, ionotropic, AMPA1	375.8374	311.4354	239.1559
1383695_at	24875	Vipr1	vasoactive intestinal peptide receptor 1	375.7971	380.6459	3139.807
1378796_at	450224	Omg	oligodendrocyte-myelin glycoprotein	375.7897	283.265	126.444
1377867_at	313837	RGD15622	similar to Glutaminyl-peptide cyclotrans	375.1686	2448.05	5861.159
1384950_at	305419	Pi4k2b	phosphatidylinositol 4-kinase type 2 be	375.1592	547.4395	80.1717
1369087_at	54251	Flt1	FMS-like tyrosine kinase 1	375.0979	260.0778	182.1121
1398302_at	64361	Prlpf	prolactin-like protein F	375.0054	56.32596	157.3859
1376791_at	497903	RGD15646	similar to hypothetical protein A830006	374.8105	29.33142	143.5969
1370635_at	171412	LOC17141	E-3 epididymal fluid protein	374.5671	173.3979	291.8232

1368618_at	58844	Grb14	growth factor receptor bound protein 1	374.479	417.5445	659.1423
1387649_at	59314	Camk2n2	calcium/calmodulin-dependent protein	374.4712	243.3834	245.291
1391998_at	314964	LOC31496	similar to PHD finger protein 20-like 1 i	373.6194	306.7407	126.3807
1396822_at	308126	Zbtb2_prec	zinc finger and BTB domain containing	373.4443	198.1403	95.94189
1374291_at	311329	Dnajc17_p	DnaJ (Hsp40) homolog, subfamily C, r	373.3025	1066.852	479.8873
1391756_at	365207	RGD15605	similar to carcinoembryonic antigen-re	373.2459	241.9418	226.0994
1382138_at	688240	LOC68824	NA	373.2363	336.4652	303.1469
1369035_a_at	25743	Kcnj6	potassium inwardly-rectifying channel,	373.096	704.6778	610.1179
1373105_at	315461	Il1rl1l	interleukin 1 receptor-like 1 ligand	373.0287	908.0513	71.31951
1380828_at	29705	Gabra1	gamma-aminobutyric acid A receptor,	372.7639	88.06638	104.2572
1380391_at	362687	Sfrs7	splicing factor, arginine/serine-rich 7	372.7459	298.2098	191.1863
1385404_at	307102	Ankrd16	ankyrin repeat domain 16	372.7083	400.941	422.8605
1371119_at	360231	LOC36023	MHC class I RT1.O type 149 processe	372.5517	221.1665	342.1153
1387472_at	25710	Cd3d	CD3 antigen delta polypeptide	372.5487	15.71679	78.37474
1397745_at	307594	Mib1_predi	mindbomb homolog 1 (Drosophila) (pr	371.9846	88.366	327.8169
1391038_at	252834	Gdf6	growth differentiation factor 6	371.9749	48.09819	182.3229
1387504_at	171106	Il1rl2	interleukin 1 receptor-like 2	371.6	257.1753	244.2052
1382486_at	302995	Haghl	hydroxyacylglutathione hydrolase-like	371.5911	433.181	435.9877
1376420_at	500059	RGD15598	similar to hypothetical gene supported	371.5622	336.2859	519.625
1387227_at	117538	Waspip	Wiskott-Aldrich syndrome protein intera	371.2896	53.56205	330.3668
1371937_at	501007	RGD15626	similar to RIKEN cDNA 6030419C18 g	371.1623	242.4166	204.918
1369189_at	29471	Ppyr1	pancreatic polypeptide receptor 1	370.9846	152.5917	119.2167
1391787_at	360900	Traf3ip3	TRAF3 interacting protein 3	370.6829	201.8425	342.293
1376684_at	289997	Dlg7_predi	discs, large homolog 7 (Drosophila) (p	370.575	2721.866	221.0052
1368150_at	65192	Slc27a2	solute carrier family 27 (fatty acid trans	370.538	128.3137	238.9001
1397456_at	291940	RGD15606	similar to hypothetical protein (predicte	370.3988	250.0516	408.2552
1374057_at	366083	RGD15618	similar to Homeobox protein Hox-D8 (H	370.2358	158.144	248.6271
1397252_at	307989	RGD15657	similar to actin-binding LIM protein 1 lo	370.1111	118.5442	235.4317
1396580_at	63883	Kcne3	potassium voltage-gated channel, Isk-r	370.0479	81.7726	40.66103
1384941_at	362205	RGD15627	similar to Shb-like adapter protein, Shf	370.024	447.9486	339.6413
1396737_at	316732	RGD13072	similar to RIKEN cDNA 4931400A14 (p	369.9636	179.5697	348.9968
1396511_at	363924	Rad9b	RAD9 homolog B (S. cerevisiae)	369.8698	290.483	149.812
1387618_at	114552	Bcl2l10	Bcl2-like 10	369.7269	130.5894	133.8972
1380701_at	311146	Ssfa2_prec	sperm specific antigen 2 (predicted)	369.6068	517.2132	68.20998
1380451_at	364378	RGD13113	similar to T cell receptor V delta 6	369.5211	164.1329	115.4155
1385394_at	293739	Ms4a10_pr	membrane-spanning 4-domains, subfa	369.2633	200.2647	183.1567
1388093_at	246219	Tas2r41	taste receptor, type 2, member 41	368.9887	49.70589	98.65825
1368803_at	50546	Insl6	insulin-like 6	368.8947	79.74076	623.4891
1388094_at	25447	Fshb	follicle stimulating hormone beta	368.6862	230.6902	269.4475
1388175_at	79430	Clcnkb	chloride channel Kb	368.5944	114.048	204.9023
1393347_at	308995	Itgal	integrin alpha L	368.5639	357.6815	429.6216
1368085_at	171128	Gchfr	GTP cyclohydrolase I feedback regulat	368.0775	470.3647	320.2503
1392957_at	361877	RGD15629	similar to Single-stranded DNA-binding	368.0457	2695.502	1784.228
1391228_at	361818	Tsga2	testis specific gene A2	367.9002	970.5469	569.7785
1392927_at	302967	Paqr4	progestin and adipoQ receptor family r	367.8301	410.345	167.3426
1378718_at	362910	RGD15614	similar to sterile alpha motif domain co	367.8173	31.84384	72.41278
1389295_at	304960	Olfml2b_pr	olfactomedin-like 2B (predicted)	367.8067	165.8589	592.015
1384007_at	362750	Spg3a	spastic paraplegia 3A homolog (humar	367.7602	148.005	582.1699
1383940_at	304951	Cdca1	cell division cycle associated 1	367.7282	7429.96	374.8939
1368337_at	25258	Glycam1	glycosylation dependent cell adhesion	367.6167	200.116	372.2976
1368983_at	25433	Hbegf	heparin-binding EGF-like growth factor	367.5392	967.2335	199.1573

1368662_at	171387	Rnf39	ring finger protein 39	367.5155	506.5851	3359.551
1370981_at	83574	Rxrg	retinoid X receptor gamma	367.4624	244.6002	252.807
1367600_at	64362	Des	desmin	367.2848	202.3746	218.7131
1394919_at	24446	Hgf	hepatocyte growth factor	366.8785	93.74105	273.0973
1370334_at	64471	Plekhb1	pleckstrin homology domain containing	366.7884	147.644	1421.825
1394924_at	307035	Mpp7	membrane protein, palmitoylated 7 (M/	366.7827	20.82413	165.6749
1374206_at	291975	RGD13073	similar to hypothetical protein DKFZp4	366.7565	515.534	325.7065
1396381_at	688736	MGC1162(	NA	366.6159	656.1568	26.69477
1381514_at	307974	LOC30797	NA	366.6121	68.16649	281.8701
1369988_at	24336	Epor	erythropoietin receptor	366.2808	266.5778	283.5588
1389276_at	309550	L3mbtl3_pr	l(3)mbt-like 3 (Drosophila) (predicted)	366.234	297.6339	884.4584
1376471_at	307916	Jph3_predi	junctophilin 3 (predicted)	366.1628	110.6151	39.05893
1377385_at	303583	Arhgap27	Rho GTPase activating protein 27	366.0638	267.4066	351.0204
1373617_at	360468	Emp2	epithelial membrane protein 2	365.654	57.99042	178.9354
1368700_at	84587	Plcl1	phospholipase C-like 1	365.4614	1939.033	10172.24
1388032_a_at	171551	Gm1960	gene model 1960, (NCBI)	365.3897	217.5221	318.7246
1388226_at	286959	Olr1271	olfactory receptor 1271	365.3179	59.39592	112.7781
1368915_at	59113	Kmo	kynurenine 3-monooxygenase (kynure	365.3063	56.12445	100.7357
1368732_at	24812	Tap2	transporter 2, ATP-binding cassette, su	365.2694	247.1791	471.2945
1370547_at	252922	Pzp	pregnancy-zone protein	365.2112	175.3476	130.2978
1374951_at	338458	Obscn	obscurin, cytoskeletal calmodulin and t	365.1904	337.1878	49.25973
1391929_at	306038	RGD13061	similar to hypothetical protein FLJ3184	365.1522	59.55588	216.6333
1375622_at	312562	Zfyve20_pr	zinc finger, FYVE domain containing 2	365.1121	389.8957	306.7062
1387468_at	25354	Sstr5	somatostatin receptor 5	365.0121	210.6344	77.92244
1375812_at	309252	RGD15637	similar to Cezanne 2 protein (predictec	364.4525	143.474	191.8216
1383395_at	298607	Agmat	agmatine ureohydrolase (agmatinase)	364.4188	140.1008	23.93105
1373245_at	290905	Col4a1	procollagen, type IV, alpha 1	364.3173	68.4337	1659.287
1393126_at	309373	Ubtd1	ubiquitin domain containing 1	363.9987	348.5963	469.2396
1394140_at	450228	Ka25	type I hair keratin KA25	363.961	183.9705	276.3451
1381574_at	362732	RGD1312C	similar to putative protein, with at least	363.9321	237.0031	208.6866
1370490_at	25133	Pcdh3	protocadherin 3	363.7823	121.3035	121.2801
1370089_at	83516	Ppargc1a	peroxisome proliferative activated rece	363.6073	75.25764	473.1756
1378970_at	310782	RGD13105	similar to Myosin-binding protein H (My	363.5677	94.46022	181.2295
1393460_at	303875	Lrrc33	leucine rich repeat containing 33	363.5555	309.4455	290.259
1376423_at	308999	Fbxl19_pre	F-box and leucine-rich repeat protein 1	363.5334	383.5582	244.7859
1387935_at	246144	Il3ra	interleukin 3 receptor, alpha chain	363.4964	2724.434	421.2389
1391509_at	494343	Tacstd2	tumor-associated calcium signal transc	363.4788	102.8262	67.40008
1369479_at	81820	Doc2b	double C2, beta	363.4748	88.41788	31.09034
1374759_at	362760	Galntl1	UDP-N-acetyl-alpha-D-galactosamine:	363.3508	99.37559	65.92045
1368661_at	65202	Slc13a2	solute carrier family 13 (sodium-depen	363.2645	78.29748	331.3611
1377407_at	361637	RGD13095	similar to Aa2-174	363.2471	103.6197	232.5341
1380107_a_at	289727	RGD13057	similar to putative N-acetyltransferase	363.1729	117.535	66.07289
1384103_at	500536	RGD15614	similar to novel protein (HT036) (predic	363.1453	852.4232	855.7405
1379907_at	50872	Hpcal4	hippocalcin-like 4	363.0565	140.3156	54.05809
1383145_at	303801	Lamp3	lysosomal-associated membrane prote	363.0164	125.3838	70.68607
1384741_at	289733	Pla2g3_pre	phospholipase A2, group III (predicted	362.8317	179.6689	155.816
1388970_at	292912	Rasip1_pre	Ras interacting protein 1 (predicted)	362.4748	268.773	348.2339
1367871_at	25086	Cyp2e1	cytochrome P450, family 2, subfamily e	362.4625	162.84	169.1945
1376895_at	116996	Il16_mappe	interleukin 16 (mapped)	362.4414	167.5255	232.1351
1367673_at	140927	Selenbp1	selenium binding protein 2	362.4393	191.5959	193.3313
1387318_at	66016	Kcnmb4	potassium large conductance calcium-	362.4282	1043.904	272.398

1378856_at	501628	RGD15625	similar to transcription elongation facto	362.3492	9937.194	916.5579
1389858_at	24834	Tk1	thymidine kinase 1	362.3058	5689.026	215.1504
1371527_at	25314	Emp1	epithelial membrane protein 1	361.8899	130.295	2417.614
1397696_at	304863	RGD13078	similar to cDNA sequence BC003331 (	361.2195	265.7644	108.8264
1369390_a_at	29272	Dpp6	dipeptidylpeptidase 6	360.9831	1868.912	665.8454
1379182_at	316219	Trerf1_pred	transcriptional regulating factor 1 (pred	360.8709	507.6426	1088.998
1397111_at	304827	Kcnt2	potassium channel, subfamily T, meml	360.8439	73.44912	164.7221
1384272_at	499425	Zfp365	zinc finger protein 365	360.8411	456.4997	601.5954
1373010_at	360625	Krt1-12	keratin complex 1, acidic, gene 12	360.8124	183.431	403.0544
1370415_at	54355	Rassf5	Ras association (RalGDS/AF-6) doma	360.7602	328.0514	1994.264
1368258_at	58812	Apln	apelin, AGTRL1 ligand	360.6748	291.1485	230.8027
1367566_at	25575	Scgb1a1	secretoglobin, family 1A, member 1 (ut	360.6548	67.98858	216.949
1394675_at	362635	Zbtb40_pre	zinc finger and BTB domain containing	360.5052	297.4035	283.8371
1395117_at	311348	Cdan1_pre	congenital dyserythropoietic anemia, ty	360.175	122.1986	69.75727
1389566_at	363088	Ccnb2	cyclin B2	360.112	7651.548	456.052
1377534_at	365381	Stk32c_pre	serine/threonine kinase 32C (predicted	359.813	845.0646	1820.775
1393376_at	293165	Sox6	SRY-box containing gene 6	359.6973	56.36966	510.5139
1394561_at	308773	Hapln3	hyaluronan and proteoglycan link prote	359.6465	152.1629	376.0589
1388920_at	25644	Bmp6	bone morphogenetic protein 6	359.5907	40.76486	207.0371
1368786_a_at	80840	Gpcr12	G-protein coupled receptor 12	359.0998	43.47969	40.36627
1378317_at	685652	LOC68565	NA	358.6046	153.2331	378.2207
1385835_at	303505	Arl12_pred	ADP-ribosylation factor-like 12 (predict	358.1478	287.3942	128.961
1379159_at	304592	RGD13102	similar to chromosome 7 open reading	357.7197	230.104	106.5078
1397462_at	305135	RGD15634	similar to T-cell activation leucine repe	357.6407	60.32104	109.6505
1369519_at	24323	Edn1	endothelin 1	357.5463	118.9724	175.4797
1368259_at	24693	Ptgs1	prostaglandin-endoperoxide synthase	357.529	705.3237	460.552
1389836_a_at	25358	Timp3	tissue inhibitor of metalloproteinase 3 (	357.5204	102.9186	840.5204
1370142_at	24631	Pem	placentae and embryos oncofetal gene	357.4903	84.21653	60.15132
1373722_at	361308	Kif20a_pre	kinesin family member 20A (predicted)	357.2509	9851.016	219.8081
1394027_at	300923	RGD15643	similar to Nucleoporin 62 (predicted)	357.2012	214.9812	320.2379
1397681_at	309456	RGD13057	similar to hypothetical protein FLJ2015	357.19	540.3761	179.1313
1376259_at	85420	PRKCQ	protein kinase C, theta	357.0111	154.0343	73.17189
1383902_at	304007	Lrriq2_pred	leucine-rich repeats and IQ motif conta	356.9479	359.3411	147.1438
1369740_at	50599	Kcnj3	potassium inwardly-rectifying channel,	356.8573	194.4489	490.8134
1371205_at	54302	Slc14a2	solute carrier family 14 (urea transport	356.8232	205.5579	265.2134
1378389_at	307231	RGD15602	similar to nuclear factor of activated T-	356.5702	3229.664	602.5024
1387296_at	65210	Cyp2j4	cytochrome P450, family 2, subfamily .	356.3013	128.3802	317.2783
1395168_at	304558	LOC30455	NA	356.1793	206.0598	209.5542
1380531_at	300908	Slc9a9_pre	solute carrier family 9 (sodium/hydroge	356.1546	241.4322	46.02843
1369169_at	50621	Slc23a1	solute carrier family 23 (nucleobase tra	356.1223	121.5637	158.2793
1383908_at	307148	Nsun6_pre	NOL1/NOP2/Sun domain family, mem	355.9966	752.16	429.2921
1375281_at	303398	Tbx2_pred	T-box 2 (predicted)	355.9061	284.9182	129.5513
1377675_at	498261	NA	NA	355.8079	203.4879	424.8146
1382822_at	309927	LOC30992	NA	355.7883	142.1202	205.0582
1382088_at	84025	Ryr2	ryanodine receptor 2, cardiac	355.7759	824.0029	5053.063
1379343_at	301418	MGC94335	similar to hypothetical protein FLJ2255	355.6222	343.46	683.2007
1370404_at	266776	LOC26677	cystatin TE-1	355.5107	79.52969	12.18691
1395884_at	305035	Angel2_pre	angel homolog 2 (Drosophila) (predicte	355.4203	204.0595	231.0864
1368003_at	116676	Aldh1a2	aldehyde dehydrogenase family 1, sub	355.148	291.1365	404.9689
1376861_at	317312	LOC31731	similar to RIKEN cDNA 1810018L05	355.0698	403.4094	503.7475
1368473_at	50563	Gja5	gap junction membrane channel protei	354.9371	97.3557	238.7974

1380944_at	311613	RGD13073	similar to KIAA0681 protein (predicted)	354.7098	179.7358	188.3059
1368453_at	83512	Fads2	fatty acid desaturase 2	353.988	3782.46	215.4326
1391847_at	25747	Ppara	peroxisome proliferator activated recep	353.8834	225.5643	223.1786
1376093_at	301221	Mocs1_pre	molybdenum cofactor synthesis 1 (pre	353.8049	177.1895	243.778
1374794_at	353302	Kif15	kinesin family member 15	353.7489	1947.89	343.5938
1369704_at	113918	Xtrp3	X transporter protein 3	353.3764	467.8645	298.4382
1388220_at	116544	Pou2f3	POU domain, class 2, transcription fac	353.3465	148.0564	62.08485
1369837_at	60671	Gulo	L-gulonolactone oxidase	353.275	33.30077	821.5012
1369301_at	83518	Agtrl1	angiotensin receptor-like 1	353.2416	73.48017	131.1092
1383391_a_at	24231	C2	complement component 2	353.2353	360.7249	1451.657
1397107_at	502070	NA	NA	353.1636	76.78645	409.568
1387809_at	114495	Map2k6	mitogen-activated protein kinase kinas	353.1316	143.258	146.8685
1368383_at	60337	Npff	neuropeptide FF-amide peptide precur	353.0899	331.4974	751.2074
1388026_at	25300	Cd3z	CD3 antigen, zeta polypeptide	353.0028	182.0009	245.1834
1391199_at	266610	Fadd	Fas (TNFRSF6)-associated via death	352.8997	507.5492	450.8027
1368951_at	24412	Grin2d	glutamate receptor, ionotropic, N-meth	352.8449	288.8031	169.8377
1390092_at	54268	Mapk4	mitogen-activated protein kinase 4	352.8175	389.9942	740.9603
1369408_at	140610	Dbccr1	deleted in bladder cancer chromosome	352.7724	325.1238	644.9718
1386964_at	54303	Smgb	neonatal submandibular gland protein	352.7678	679.6636	454.0233
1387990_at	432361	Nrg2	neuregulin 2	352.7139	117.6473	243.632
1396722_at	140928	Pde11a	phosphodiesterase 11A	352.4924	122.1244	124.3325
1392785_at	364458	Kctd12_pre	potassium channel tetramerisation dor	352.3965	557.2042	926.2997
1393693_at	312317	Doxl2	diamine oxidase-like protein 2	352.2335	112.043	192.5593
1383414_at	361066	RGD13081	similar to 9930012K11Rik protein (pre	352.127	171.6238	301.0588
1369045_at	114705	Rgs14	regulator of G-protein signaling 14	352.0584	271.2473	567.3397
1379361_at	85249	Pex11a	peroxisomal biogenesis factor 11A	352.0404	605.01	570.1506
1381871_at	500261	LOC50026	NA	351.9068	615.8189	715.1256
1369150_at	89813	Pdk4	pyruvate dehydrogenase kinase, isoer	351.847	200.7751	509.0546
1398511_at	294335	Susd2_pre	sushi domain containing 2 (predicted)	351.8401	578.4797	623.9594
1375261_at	295319	Man1a2_pr	mannosidase, alpha, class 1A, membe	351.7611	134.8612	73.55391
1381009_at	60669	Cmklr1	chemokine-like receptor 1	351.3439	161.2025	216.3285
1393891_at	304021	Col8a1_pre	procollagen, type VIII, alpha 1 (predict	351.3419	64.40206	476.3867
1384696_at	367620	MGC11619	similar to RIKEN cDNA 1700001E04	351.2884	31.37333	335.2639
1382783_at	308755	Blm_predic	Bloom syndrome homolog (human) (pr	351.0661	1993.468	608.3481
1395166_at	361512	Ehd2	EH-domain containing 2	350.9223	149.9302	289.1549
1398580_at	298096	Wdr31	WD repeat domain 31	350.5248	590.9303	213.4725
1395710_at	313418	RGD15623	similar to mesoderm induction early re	350.51	656.1124	429.9386
1390625_at	312301	RGD13048	similar to Zinc finger protein 398 (Zinc	350.4595	837.3418	272.7726
1369523_at	140473	Ccbp2	chemokine binding protein 2	350.4026	88.59289	76.98127
1368310_at	29148	Myog	myogenin	350.314	260.5653	338.137
1370059_at	83613	Nefl	neurofilament, light polypeptide	349.724	1518.917	404.8932
1389786_at	500419	LOC50041	similar to RIKEN cDNA 2410005O16	349.64	424.6082	323.1549
1378541_at	315264	Pus7l_prec	pseudouridylate synthase 7 homolog (	349.6273	599.5248	249.0636
1382553_at	361746	Rfx3	regulatory factor X, 3 (influences HLA	349.5825	136.9405	356.1772
1384783_at	282825	Zfp161	zinc finger protein 161	349.5197	257.4053	194.2978
1387005_at	50654	Ctss	cathepsin S	349.5099	66.66116	415.853
1392466_at	293098	RGD13099	similar to CG11737-PA	349.4526	515.2773	570.9258
1384995_at	315073	Jrk_predict	jerky homolog (mouse) (predicted)	349.37	250.8578	155.5939
1383008_at	295107	Smc4l1	SMC4 structural maintenance of chron	349.2575	876.7663	643.3106
1376628_at	313219	Zfp189_pre	zinc finger protein 189 (predicted)	349.1911	2130.956	695.0306
1396163_at	303163	Igtp	interferon gamma induced GTPase	349.1767	303.5992	275.5333

1368462_at	81677	Itpka	inositol 1,4,5-trisphosphate 3-kinase A	349.1585	277.6394	450.5994
1369428_a_at	79246	Htr3a	5-hydroxytryptamine (serotonin) recep	349.0628	159.7262	172.9087
1372213_at	500300	LOC50030	similar to hypothetical protein MGC683	349.0194	158.0228	420.9592
1378300_at	299186	Dpf3_predi	D4, zinc and double PHD fingers, fami	349.0158	349.8736	282.4688
1380907_at	314456	RGD13075	similar to Hypothetical protein KIAA029	348.9581	265.1885	415.6148
1371049_at	25417	Dpysl4	dihydropyrimidinase-like 4	348.9422	542.2324	189.7615
1392915_at	25654	Col11a1	procollagen, type XI, alpha 1	348.9122	170.6188	191.484
1371194_at	84397	Tnfaip6	tumor necrosis factor alpha induced pr	348.8878	124.3049	680.8497
1383549_at	296840	RGD13107	similar to RIKEN cDNA C030048B08	348.8004	1195.184	470.3324
1369298_at	29170	Aqp6	aquaporin 6	348.636	177.5676	142.3112
1386989_at	29415	Edg5	endothelial differentiation, sphingolipid	348.6171	194.892	351.2699
1397659_at	361809	Zfp523_pre	zinc finger protein 523 (predicted)	348.4155	398.1124	467.8335
1373873_at	306896	RGD15653	similar to OTTMUSP00000000621 (pre	348.4096	88.77753	162.2611
1375236_at	315409	RGD15635	similar to mKIAA1377 protein (predicte	348.213	103.3778	129.2618
1387150_at	54282	Padi1	peptidyl arginine deiminase, type I	347.8443	159.4612	168.7447
1387484_at	29610	Tgfbr3	transforming growth factor, beta recep	347.7475	264.1777	121.1845
1370648_a_at	259242	Cr16	SH3 domain binding protein CR16	347.6874	88.20566	319.6877
1389449_at	290223	RGD13069	similar to CG10671-like (predicted)	347.6357	149.914	221.2637
1381390_at	360885	NA	NA	347.6289	434.4477	821.7437
1397027_at	65271	Mgat5	mannoside acetylglucosaminyltransfer	347.5354	154.8378	289.0386
1395358_at	309922	Rhobtb3_p	Rho-related BTB domain containing 3	347.4713	74.11049	59.33962
1368601_at	24898	Slc6a3	solute carrier family 6 (neurotransmitte	347.2506	175.5962	112.4629
1370883_at	294269	RT1-Da	RT1 class II, locus Da	347.2426	95.26314	1546.011
1382929_at	292596	Leng9_pre	leukocyte receptor cluster (LRC) meml	347.0589	189.7001	267.8403
1368207_at	60338	Fxyd5	FXYD domain-containing ion transport	346.9828	126.9359	136.2165
1386145_at	300900	Plscr4	phospholipid scramblase 4	346.7936	105.008	481.0506
1385825_at	296115	RGD15599	similar to mKIAA0256 protein (predicte	346.7813	414.4962	172.31
1384580_at	24237	C6	complement component 6	346.7482	158.279	39.8638
1369615_at	114560	Rap2a	RAS related protein 2a?	346.7033	308.1016	184.2856
1381475_at	360652	Sdk2_pred	sidekick homolog 2 (chicken) (predicte	346.5562	411.9962	324.42
1384251_at	499281	RGD15599	similar to leucine rich repeat containing	346.4555	312.9347	182.9146
1383568_at	361856	Tube1_pre	tubulin, epsilon 1 (predicted)	346.3968	1416.217	688.6199
1369237_at	117100	Slc6a7	solute carrier family 6 (neurotransmitte	346.3788	172.1091	185.9616
1368375_a_at	25670	Il15	interleukin 15	346.3701	711.2796	671.8817
1368763_at	24495	Il3	interleukin 3	346.2777	108.9168	181.1877
1372860_at	361663	MGC95092	similar to phospholysine phosphohistic	346.0835	2083.594	1423.171
1376334_at	679664	LOC67966	NA	346.0537	111.2584	585.4148
1387104_at	25122	Scnn1a	sodium channel, nonvoltage-gated 1 a	346.0169	50.12879	143.4555
1367626_at	24265	Ckm	creatine kinase, muscle	345.7647	192.5513	104.672
1369765_at	64186	Ascl1	achaete-scute complex homolog-like 1	345.753	160.2711	571.1747
1387229_at	83624	Ppig	peptidylprolyl isomerase G	345.5124	417.0432	1999.116
1378656_at	362142	Bbs5_pred	Bardet-Biedl syndrome 5 homolog (hu	345.4066	716.3619	1343.384
1388460_at	297339	Capg	capping protein (actin filament), gelsoli	344.9759	201.8197	179.2989
1386903_at	25742	S100b	S100 protein, beta polypeptide	344.736	97.76658	274.9414
1384262_at	192280	Ppp1r3b	protein phosphatase 1, regulatory (inhi	344.5701	692.4175	446.4956
1387168_at	84398	C1qr1	complement component 1, q subcomp	344.287	166.521	153.8521
1368999_a_at	79146	Begain	brain-enriched guanylate kinase-assoc	344.2235	337.9909	334.0847
1384305_at	246258	Slc45a1	solute carrier family 45, member 1	343.977	1040.17	766.1718
1376663_at	304653	RGD13048	similar to hypothetical protein 6720484	343.8723	879.8596	113.0864
1370254_at	94272	Clic5	chloride intracellular channel 5	343.6091	263.657	676.3934
1380029_at	315037	RGD13057	similar to mKIAA0431 protein	343.6003	73.88196	88.80168

1373697_at	292879	Mybpc2_pr	myosin binding protein C, fast-type (pr	343.4359	69.37971	344.5088
1371110_at	287004	LOC28700	Mg1 protein	343.4248	125.6478	135.8309
1380285_at	117275	Chrd	chordin	342.8995	371.308	191.647
1389821_at	117582	Ris1	Ras-induced senescence 1	342.639	46.3786	283.9654
1387630_at	171400	Elovl5	ELOVL family member 5, elongation o	342.46	578.3912	259.8675
1391791_at	313339	Asah3l_pre	N-acylsphingosine amidohydrolase 3-l	342.4315	86.76318	287.9992
1374244_at	501038	LOC50103	Ab2-060	342.0874	539.6046	387.1029
1367614_at	25380	Anxa1	annexin A1	342.0368	39.13835	6997.056
1382902_at	362376	Herc6	potential ubiquitin ligase	341.9076	73.7847	2335.465
1369886_a_at	171051	Cabp1	calcium binding protein 1	341.8402	262.0988	30.94734
1398282_at	116682	Kynu	kynureninase (L-kynurenine hydrolase	341.2182	208.6545	118.9005
1393408_at	500137	RGD15627	similar to NEX-1 (predicted)	341.072	38.17991	43.3081
1374452_at	191569	Pde9a	phosphodiesterase 9A	340.9428	2077.463	475.7185
1369573_at	54270	Mcpt4	mast cell protease 4	340.6727	46.11492	124.4901
1393757_at	498131	LOC49813	NA	340.6213	127.131	736.3091
1387334_at	29268	Mcpt6	mast cell protease 6	340.3078	88.52176	161.858
1381908_at	314555	Tspyl5_pre	TSPY-like 5 (predicted)	340.1904	91.15634	37.58783
1394626_at	305968	RGD13067	similar to hypothetical protein FLJ2161	340.1506	63.54076	228.149
1393061_at	300676	MGC95155	similar to cDNA sequence BC021608	339.9714	269.3746	265.9552
1374936_at	302972	RGD13046	similar to RIKEN cDNA 5730457F11	339.9169	406.5534	133.1733
1385897_at	641386	Kiaa0415	NA	339.8446	342.6084	405.9219
1393931_at	363276	RGD13114	LOC363276 (predicted)	339.7542	96.91123	336.268
1382569_at	315904	Paqr9_pre	progestin and adipoQ receptor family r	339.7276	158.9186	342.5745
1394494_at	362648	Arhgef19_p	Rho guanine nucleotide exchange fact	339.5514	117.8705	141.8235
1373991_at	29719	Kcnj16	potassium inwardly-rectifying channel,	339.389	40.18556	10.90975
1372897_at	300901	Plod2	procollagen lysine, 2-oxoglutarate 5-di	339.358	82.26106	793.2959
1377288_at	291960	Hsf4_predi	heat shock transcription factor 4 (predi	339.3519	285.6096	295.7493
1375025_at	83506	Camkk2	calcium/calmodulin-dependent protein	339.3514	1363.702	384.0759
1381286_at	502544	RGD15637	similar to late envelope protein 7 (LEP	339.2818	290.1079	24.47066
1393129_at	361612	P4ha3	procollagen-proline, 2-oxoglutarate 4-d	339.2453	175.5803	173.5234
1383729_at	303566	Asb16	ankyrin repeat and SOCS box-containi	338.8648	569.1405	118.3471
1370019_at	83783	Sult1a1	sulfotransferase family 1A, phenol-pref	338.8276	73.93738	282.9259
1384079_at	361503	Eps8l1_pre	EPS8-like 1 (predicted)	338.7552	264.1738	67.68456
1368127_at	29204	Neu2	neuraminidase 2	338.423	608.982	384.4517
1385649_at	315346	Itga5_map	integrin alpha 5 (mapped)	338.2185	155.7622	195.9581
1393790_at	288025	Hrasls_pre	HRAS-like suppressor (predicted)	338.1719	135.8876	169.4432
1382478_at	311462	Btbd3_pre	BTB (POZ) domain containing 3 (predi	338.1368	804.3068	1028.109
1398021_at	502047	RGD15653	similar to hypothetical protein 4930474	338.1324	150.1929	240.7855
1387932_at	25550	Slc1a1	solute carrier family 1 (neuronal/epithe	338.0813	1151.425	280.0702
1369363_at	25162	Sp4	Sp4 transcription factor	338.049	443.6219	251.1015
1384437_at	317575	Smarca1_p	SWI/SNF related, matrix associated, a	337.9604	2290.499	6535.276
1384382_at	314548	Osgepl1	O-sialoglycoprotein endopeptidase-like	337.8245	452.5384	420.4933
1368285_at	24775	Shbg	sex hormone binding globulin	337.7914	306.8218	121.8431
1382450_at	362185	C11orf8h	putative C11orf8 homolog (human)	337.6949	97.43239	210.8193
1368192_at	84475	Cxcr3	chemokine (C-X-C motif) receptor 3	337.5701	75.31786	97.67136
1382053_at	295293	RGD13056	similar to MEGF12 (predicted)	337.5015	353.7006	314.2848
1385269_s_at	304743	LOC30474	hypothetical protein LOC304743	337.2984	190.2329	200.2403
1388161_at	29650	Adam10	a disintegrin and metalloprotease dom	337.2848	633.5098	168.314
1372569_at	313582	Fhl3_predi	four and a half LIM domains 3 (predict	337.273	993.0989	250.6638
1392078_at	499787	NA	NA	337.035	179.9967	277.9674
1373663_at	361548	RGD15615	similar to 1110014F24Rik protein (pred	337.034	271.3161	324.6639

1374889_at	296468	RGD15628	similar to livin inhibitor of apoptosis iso	337.0321	167.3501	206.8808
1391456_at	315509	Jam3	junctional adhesion molecule 3	336.9048	336.0612	553.1299
1368855_at	29532	Ighmbp2	immunoglobulin mu binding protein 2	336.8461	555.1599	114.7909
1387651_at	25240	Aqp1	aquaporin 1	336.8448	389.6119	327.4715
1386966_a_at	60383	Pkcbpb15	protein kinase C-binding protein Beta1	336.8286	1110.377	624.8652
1381127_at	500758	RGD15640	similar to ubiquitin-conjugating enzyme	336.4697	4485.461	435.3455
1387083_at	29201	Ctf1	cardiotrophin 1	336.2704	160.5995	192.0681
1380502_at	361096	Zic2_predi	Zic family member 2 (odd-paired homo	335.7401	92.82817	140.8968
1374906_at	313450	LOC31345	similar to RIKEN cDNA 2810428C21	335.718	485.3378	582.8689
1382792_at	361977	Isg20l2	interferon stimulated exonuclease gen	335.5567	325.9285	270.8764
1377519_a_at	308934	RGD15594	similar to SET binding factor 2 (predict	335.4843	292.4477	400.3921
1368557_s_at	65039	Vegp1	von Ebners gland protein 1	335.4195	212.9987	210.1312
1393425_at	682100	LOC68210	NA	335.3251	176.111	244.5087
1393385_at	498405	RGD15602	RGD1560273 (predicted)	335.262	116.9873	392.5803
1376355_at	317423	RGD15642	similar to Protein CXorf17 homolog (pr	335.0484	113.971	177.4471
1384979_at	117097	Gpr50	G protein-coupled receptor 50	334.9874	9.312244	22.50435
1392811_at	316599	Usp40	ubiquitin specific protease 40	334.9662	283.9148	56.10387
1373781_a_at	292793	RGD15623	similar to suprabasal-specific protein s	334.8695	333.7684	484.1644
1368655_at	56782	Pgsg	proteoglycan peptide core protein	334.8051	156.3083	486.1117
1375553_at	291676	RGD13117	similar to RIKEN cDNA 5133400G04	334.7996	665.7508	222.706
1388837_at	363024	Slc44a2_pr	solute carrier family 44, member 2 (pre	334.6802	1228.079	673.9594
1383911_at	298144	Jmjd2c_pre	jumonji domain containing 2C (predicte	334.679	103.3508	628.1792
1369833_at	56806	Adam2	a disintegrin and metalloprotease dom	334.6789	90.51298	134.0584
1367553_x_at	24440	Hbb	hemoglobin beta chain complex	334.5276	241.4478	592.1189
1370294_a_at	64515	Cdc20	cell division cycle 20 homolog (S. cere	334.4304	6123.456	317.4743
1378914_a_at	308990	LOC30899	hypothetical protein LOC308990	334.1912	503.5961	660.2382
1393754_at	360937	Tbc1d1_pr	TBC1 domain family, member 1 (predi	334.1189	697.4394	260.3367
1385430_at	499443	RGD15607	similar to LIM and senescent cell antig	333.9978	262.566	104.5643
1372699_at	312309	RGD13049	similar to hypothetical protein C130032	333.9972	188.5643	340.6376
1368739_s_at	500892	Cyp11b1	cytochrome P450, subfamily 11B, poly	333.9493	43.67473	71.20516
1396626_at	360928	Dcun1d4_p	DCN1, defective in cullin neddylation 1	333.9353	41.84596	320.6325
1391797_at	498607	LOC49860	similar to Myocyte-specific enhancer fa	333.6942	305.6787	307.0107
1370705_at	266774	Zfp597	zinc finger protein 597	333.6691	511.3736	145.0358
1387442_at	25129	Egr4	early growth response 4	333.594	141.8821	596.2476
1384594_at	311337	Ltk_predict	leukocyte tyrosine kinase (predicted)	333.3512	183.256	564.9849
1392065_at	362186	RGD15617	similar to Kinesin family member 18A (	333.2639	934.9665	156.0808
1388340_at	300795	Ns5atp9	NS5A (hepatitis C virus) transactivated	333.2535	9494.388	397.4946
1380168_at	681360	LOC68136	NA	333.1877	19.79204	31.62764
1376795_at	294048	Pik3ap1_pr	phosphoinositide-3-kinase adaptor pro	333.1476	654.0305	1132.308
1367860_a_at	81707	Mmp14	matrix metallopeptidase 14 (membrane	332.9805	36.49813	482.7209
1378151_at	140729	Cacng8	calcium channel, voltage-dependent, g	332.8837	76.16013	160.5405
1394893_at	311342	RGD13052	similar to phospholipase A2, group IVE	332.8259	21.59979	63.48465
1398467_at	360644	NA	NA	332.7967	736.4501	207.8122
1395099_at	29750	Galnt7	UDP-N-acetyl-alpha-D-galactosamine:	332.5813	616.8255	278.1677
1398283_at	64019	Cbp	Csk binding protein	332.4486	35.53117	73.9788
1369581_at	25511	Pemt	phosphatidylethanolamine N-methyltra	332.3594	74.36619	19.30915
1369309_a_at	24806	Tac1	tachykinin 1	332.3442	57.37056	1171.822
1374864_at	306141	Spry2	sprouty homolog 2 (Drosophila)	332.2419	357.7202	4475.845
1368302_at	81710	Msx1	homeo box, msh-like 1	332.2351	19.56594	62.74228
1387328_at	29277	Cyp2c	Cytochrome P450, subfamily IIC (mep	332.0864	191.5062	131.5341
1379863_at	65180	Kcnd2	potassium voltage gated channel, Sha	331.9712	583.3844	489.8396

1370104_at	114248	Pde6h	phosphodiesterase 6H, cGMP-specific	331.9378	265.3675	168.5317
1372959_at	619572	Nme4	NA	331.7842	21.32407	281.9937
1374190_at	306198	Clybl	citrate lyase beta like	331.5841	445.1782	349.8519
1395042_at	305556	Ehbp1_pre	EH domain binding protein 1 (predicted	331.5625	133.897	165.5809
1376039_at	261730	Stk6	serine/threonine kinase 6	331.4696	3570.953	66.82161
1385765_at	360888	LOC36088	NA	331.4219	1336.627	1521.815
1377753_at	307237	Pard6g_pre	par-6 partitioning defective 6 homolog	331.3305	1789.339	507.1536
1398270_at	29373	Bmp2	bone morphogenetic protein 2	331.0596	378.3988	3252.591
1390588_at	303375	Rad51l3_p	RAD51-like 3 (S. cerevisiae) (predicted	330.484	116.0679	214.0826
1392672_at	29313	Clec11a	C-type lectin domain family 11, membe	330.4627	475.8273	467.4471
1392994_at	499330	LOC49933	similar to Nicotinamide riboside kinase	330.2256	467.0453	237.12
1376781_at	316033	Glb1_mapp	galactosidase, beta 1 (mapped)	330.0263	199.1044	314.5655
1369230_at	29695	Gabrr2	gamma-aminobutyric acid (GABA-C) re	329.9658	33.40644	190.824
1369911_at	29363	Blr1	Burkitt lymphoma receptor 1	329.8969	66.27335	52.06885
1380523_at	361354	Fbxo15_pre	F-box protein 15 (predicted)	329.8732	174.7744	13.10339
1369022_at	171442	Svop	SV2 related protein	329.8324	1372.206	224.4734
1382667_at	305449	RGD1311S	similar to tetracycline transporter-like p	329.5647	89.48673	173.1981
1387395_at	29316	Adora2b	adenosine A2B receptor	329.5366	2623.374	451.1335
1370397_at	298423	Cyp4a14	cytochrome P450, family 4, subfamily a	329.5253	121.7816	365.5111
1368872_a_at	29547	Homer2	homer homolog 2 (Drosophila)	329.4574	292.5216	152.5061
1369260_a_at	58808	Mpp4	membrane protein, palmitoylated 4 (MA	329.4463	207.0245	323.8364
1378476_at	301254	Aarsl_pred	alanyl-tRNA synthetase like (predicted	329.3375	261.9854	374.2169
1369027_at	63888	A4galt	alpha 1,4-galactosyltransferase	329.2044	57.34315	77.90439
1369517_at	116691	Pscd1	pleckstrin homology, Sec7 and coiled-c	329.1636	368.524	142.0976
1384425_at	304131	LOC30413	similar to C21ORF7	329.1345	120.0889	66.29318
1368482_at	170929	Bcl2a1	B-cell leukemia/lymphoma 2 related pr	329.0782	37.13546	508.7602
1367631_at	64032	Ctgf	connective tissue growth factor	328.3261	147.2885	784.1293
1390175_at	309161	RGD15653	similar to Delta-interacting protein A (H	328.1146	142.4046	175.1224
1385573_at	29548	Homer3	homer homolog 3 (Drosophila)	327.8691	409.5337	76.4305
1368406_at	25557	Star	steroidogenic acute regulatory protein	327.7419	301.0837	307.0773
1397476_at	315852	Ttk_predict	Ttk protein kinase (predicted)	327.5894	715.3564	85.81349
1371226_at	25412	Col2a1	procollagen, type II, alpha 1	327.3162	233.5095	53.75017
1390415_at	292206	Trip13	thyroid hormone receptor interactor 13	327.1259	2319.84	181.4621
1390882_at	313575	Heyl_predi	hairy/enhancer-of-split related with YR	327.0445	123.6886	140.628
1369565_at	64546	Il12b	interleukin 12b	326.7575	182.0406	16.82551
1368918_at	94203	Pgf	placental growth factor	326.6329	192.6172	44.72126
1393823_at	316235	Polh_predi	polymerase (DNA directed), eta (RAD	326.5729	367.0305	64.78933
1370329_at	171522	Cyp2d22	cytochrome P450, family 2, subfamily c	326.4281	408.1836	506.1841
1378088_at	291967	LOC29196	NA	326.3112	366.6296	493.0701
1398262_at	24689	Prps2	phosphoribosyl pyrophosphate synthet	326.2091	1185.234	472.2419
1384206_at	297805	RGD15659	similar to short chain dehydrogenase r	326.2018	123.8029	424.1389
1395449_at	363099	RGD13097	similar to ribosomal protein L24-like; 60	326.1743	168.1412	263.5332
1391713_at	298020	RGD13089	similar to chromosome 9 open reading	326.0619	194.6481	304.8663
1374594_at	363060	LOC36306	similar to RIKEN cDNA 1600029D21	326.0068	1133.868	427.2856
1382323_at	365217	RGD15635	similar to cornifelin (predicted)	325.9041	234.2639	367.602
1378265_at	406162	Notch4	Notch homolog 4	325.7507	32.33981	71.36964
1385426_at	295462	RGD13053	similar to hypothetical protein FLJ2064	325.595	1356.014	1171.501
1392568_at	303433	RGD15622	similar to TAF11 RNA polymerase II, T	325.5817	1440.541	508.5025
1381537_at	171549	Klc3	kinesin light chain 3	325.5237	164.1628	888.737
1388081_at	405339	Olr1172	olfactory receptor 1172	325.4649	31.1919	166.2966
1394638_at	310704	Bcl9_predi	B-cell CLL/lymphoma 9 (predicted)	325.3744	296.8866	149.3644

1384130_at	367949	RGD15601	similar to PRO0149 protein (predicted)	325.3209	203.0404	116.1356
1386547_at	303599	RGD13092	similar to KIAA1636 protein (predicted)	325.1376	179.7625	117.363
1384988_at	292263	Fbxo5_pre	F-box only protein 5 (predicted)	324.9093	1418.529	218.5981
1392824_at	361994	Bnipl_pred	BCL2/adenovirus E1B 19kD interacting	324.8972	19.50447	117.8491
1369091_at	24261	Chrnb1	cholinergic receptor, nicotinic, beta pol	324.8803	66.65251	465.6367
1393563_at	25466	Il1rap	interleukin 1 receptor accessory protei	324.8333	266.9162	405.3294
1373317_at	364774	Calm4_pre	calmodulin 4 (predicted)	324.6823	151.1133	199.1716
1376166_at	361192	RGD13085	LOC361192 (predicted)	324.4226	140.0437	148.1611
1374492_at	363188	LOC36318	NA	324.4049	102.2074	223.4501
1396711_at	363259	RGD13060	similar to sallimus CG1915-PC (predic	324.3563	96.42918	312.0359
1382830_at	364785	Suv39h2_p	suppressor of variegation 3-9 homolog	324.3261	782.5145	358.0793
1395043_at	287036	NA	NA	324.2888	169.9708	48.39732
1397734_at	367846	Sp3	Sp3 transcription factor	324.2885	420.5253	1095.785
1368492_at	58962	Ptgds2	prostaglandin D2 synthase 2	324.2862	183.525	188.893
1392099_at	310463	Plcl3_predi	phospholipase C-like 3 (predicted)	324.2496	92.90208	1023.638
1372002_at	24392	Gja1	gap junction membrane channel protei	323.6574	52.14748	653.0031
1398230_at	299285	Clmn_pred	calmin (predicted)	323.5626	168.0573	251.2154
1384970_at	361340	NA	NA	323.4951	277.0542	326.3614
1385541_at	297852	Hspcal3_pr	heat shock 90kDa protein 1, alpha-like	323.3085	182.8922	278.2631
1378732_at	114031	Fstl3	follistatin-like 3	323.2573	555.5205	350.4819
1388277_at	363828	RGD15643	similar to immunoglobulin light chain va	323.254	171.6263	67.35913
1381369_at	306013	Lgi3_predi	leucine-rich repeat LGI family, membe	322.9509	198.4129	277.2567
1396155_at	501756	NA	NA	322.9139	100.605	201.5809
1396942_at	301267	NA	NA	322.8687	66.16618	89.7653
1383134_at	361184	RGD13116	similar to hypothetical protein FLJ1211	322.2411	483.9662	283.7212
1369266_at	171060	Il13ra2	interleukin 13 receptor, alpha 2	321.7555	114.0792	329.121
1367794_at	24153	A2m	alpha-2-macroglobulin	321.4316	66.8908	832.4502
1367994_at	81656	Dpyd	dihydropyrimidine dehydrogenase	321.1039	49.59009	558.3999
1397287_at	500294	RGD15659	similar to 4931417G12Rik protein (pre	321.0991	295.7535	448.6743
1396933_s_at	191574	LOC19157	3-alpha-hydroxysteroid dehydrogenase	320.8258	34.78966	150.5906
1371149_at	57029	Adam18	a disintegrin and metallopeptidase dom	320.5496	294.2286	175.9316
1387947_at	54264	Mafb	v-maf musculoaponeurotic fibrosarcom	320.2811	304.9846	3182.502
1388809_at	294422	Smpdl3a	sphingomyelin phosphodiesterase, aci	319.9655	49.51984	2274.381
1387718_at	29665	P2rx7	purinergic receptor P2X, ligand-gated i	319.9286	59.53024	131.0217
1379411_at	306366	RGD15638	similar to ENSANGP00000020885 (pre	319.8964	437.6845	176.4968
1370379_at	192107	Prss8	protease, serine, 8 (prostasin)	319.7238	80.46852	98.16268
1389997_at	315609	Cd3e_pred	CD3 antigen, epsilon polypeptide (pred	319.7054	36.19466	136.6752
1383432_at	362136	RGD13096	LOC362136 (predicted)	319.6603	20.72687	302.2314
1369454_at	24873	Vdr	vitamin D receptor	319.5193	192.5181	187.3792
1369250_at	116642	Slc28a1	solute carrier family 28 (sodium-couple	319.3494	33.88319	50.88924
1383015_at	361697	RGD13114	similar to DKFZP434P1750 protein (pr	319.1992	247.1435	54.8583
1386979_at	170907	Tpo1	developmentally regulated protein TPO	318.9476	574.6472	2108.095
1387526_at	65275	Gpr27	G protein-coupled receptor 27	318.7549	449.7708	160.8022
1368246_at	140667	Ap3m2	adaptor-related protein complex 3, mu	318.7281	650.866	618.1225
1391519_at	687921	LOC68792	NA	318.5543	184.9268	67.03958
1372271_at	366262	Rnpc1_pre	RNA-binding region (RNP1, RRM) con	318.4635	394.4822	638.6741
1395764_at	499797	RGD15602	similar to formin-like 2 isoform B (predi	318.4631	110.5041	199.1058
1370071_at	24165	Ada	adenosine deaminase	318.3637	363.2803	114.7522
1392005_at	362773	Prima1_pre	proline rich membrane anchor 1 (predi	318.331	60.76505	186.484
1393540_at	29557	Myh7	myosin, heavy polypeptide 7, cardiac r	318.2856	271.1024	156.457
1387198_at	54259	Inpp5d	inositol polyphosphate-5-phosphatase	318.2595	158.7262	230.9232

1383286_at	314260	Plek2_prec	pleckstrin 2 (predicted)	318.1367	612.9398	391.0788
1369745_at	24410	Grin2b	glutamate receptor, ionotropic, N-meth	317.9293	177.7394	242.1625
1369623_at	64548	Morp1	morphine related protein-1 (RefSeq = I	317.7886	176.5429	237.173
1395072_at	289399	RGD13118	similar to hypothetical protein FLJ1090	317.7597	61.99999	36.45218
1371135_at	64133	Xylt1	xylosyltransferase 1	317.5191	159.9009	54.13003
1369412_a_at	29723	Slc19a1	solute carrier family 19, member 1	317.3997	1345.888	16.12531
1370734_a_at	25254	Dspp	dentin sialophosphoprotein	317.3558	150.6449	242.793
1384210_at	293180	RSB-11-77	RSB-11-77 protein	317.2911	442.7578	648.1274
1387404_at	140448	Slc8a3	solute carrier family 8 (sodium/calcium	317.2265	68.05942	247.6761
1397548_at	314618	Hmha1_pre	histocompatibility (minor) HA-1 (predic	317.0714	211.0421	266.2788
1369709_at	25049	Atxn1	ataxin 1	316.973	40.2229	51.77147
1395408_at	311111	Nostrin	nitric oxide synthase trafficker	316.9722	825.3948	188.0964
1380682_at	308790	Rkhd3_pre	ring finger and KH domain containing 3	316.7263	1618.942	1765.01
1399076_at	313706	Ubiad1_pre	UbiA prenyltransferase domain contair	316.6639	206.5034	298.9405
1380259_at	294519	RGD13113	similar to RIKEN cDNA 2410017P07	316.5242	609.8868	67.87331
1393036_at	691380	MGC12504	NA	316.4399	1135.351	130.8746
1368494_at	116547	S100a8	S100 calcium binding protein A8 (calgr	316.4334	205.9684	129.4436
1368536_at	84050	Enpp2	ectonucleotide pyrophosphatase/phos	316.2705	55476.2	5423.676
1387374_at	25720	Tcf12	transcription factor 12	315.9074	522.6117	256.5468
1370701_at	65187	Gabrq	gamma-aminobutyric acid A receptor,	315.7626	272.4648	239.3919
1368636_at	114700	Cyp27b1	cytochrome P450, family 27, subfamily	315.5909	122.1225	325.5774
1385357_at	502311	NA	NA	315.5687	176.8279	135.8359
1395761_at	363142	Sema3b_p	sema domain, immunoglobulin domair	315.5043	136.1934	341.9054
1374491_at	301045	Cmtm8	CKLF-like MARVEL transmembrane d	315.2636	1452.086	1951.877
1376124_at	296936	Wasl	Wiskott-Aldrich syndrome-like (human)	315.0934	23.45873	834.2331
1394662_at	499283	RGD15624	RGD1562481 (predicted)	314.9826	130.961	280.4847
1390286_at	288559	RGD15624	similar to FLJ00248 protein (predicted)	314.9774	162.9126	63.75064
1395135_at	360794	NA	NA	314.781	246.4259	191.4496
1371033_at	309622	RT1-Bb	RT1 class II, locus Bb	314.583	34.79627	626.792
1373530_at	25729	Ccne1	cyclin E	314.4497	2773.482	83.91602
1379036_at	291324	Dnmt2	DNA methyltransferase 2	314.3678	639.3246	367.3482
1377018_at	311252	RGD13087	similar to E430002G05Rik protein (pre	314.2236	36.17067	645.7076
1368061_at	65198	Kcnh1	potassium voltage-gated channel, subf	313.8665	353.3042	226.1662
1368355_at	25132	Myo5b	myosin 5B	313.6908	615.7711	625.6432
1381911_at	500267	RGD15649	similar to leiomodin 3 (fetal) (predicted	313.6355	73.23692	235.1682
1393067_at	89804	Tek	endothelial-specific receptor tyrosine k	313.4862	88.7831	369.2879
1387665_at	81508	Bhmt	betaine-homocysteine methyltransfera	313.4652	107.0327	128.9234
1387574_at	170945	Chrna2	cholinergic receptor, nicotinic, alpha po	313.3325	102.4037	142.1235
1368299_at	140595	Gpr83	G protein-coupled receptor 83	313.1306	140.9981	184.6933
1368484_at	63886	Abcb9	ATP-binding cassette, sub-family B (M	313.06	325.278	202.1034
1381876_at	289486	Fras1_prec	Fraser syndrome 1 homolog (human) (	312.6128	41.49988	143.7028
1387075_at	25085	Th	tyrosine hydroxylase	312.5483	163.2954	170.3775
1390763_at	170901	Efna3	ephrin A3	312.1548	1415.936	364.3683
1387674_at	25248	Cnr1	cannabinoid receptor 1 (brain)	312.0599	175.1344	303.0136
1368499_at	83820	Sycp2	synaptonemal complex protein 2	311.852	332.1245	191.5449
1396896_at	303130	Fstl4_predi	follistatin-like 4 (predicted)	311.6189	751.3418	933.1207
1368293_at	83575	Cpz	carboxypeptidase Z	311.542	156.5267	50.50712
1391483_at	314638	Creb3l3	cAMP responsive element binding prot	311.5285	179.1753	430.7766
1369227_at	24942	Chm	choroidermia	310.7884	865.5936	324.1955
1367564_at	24602	Nppa	natriuretic peptide precursor type A	310.7369	74.18617	143.6409
1379709_at	363739	Krtap14_pr	keratin associated protein 14 (predicte	310.2482	198.8231	184.8723

1375518_at	84015	Ttn	titin	310.1459	143.5368	88.70757
1382034_at	296972	Akr1b10	aldo-keto reductase family 1, member	309.7102	183.1374	129.7914
1387331_at	24217	Atp4b	ATPase, H+/K+ exchanging, beta poly	309.6805	89.08791	24.20444
1379780_at	306934	RGD13069	similar to mKIAA0386 protein	309.6754	132.8942	254.4373
1377309_at	503293	LOC50329	NA	309.6218	255.5404	505.9445
1384292_at	312477	Dok1	docking protein 1	309.5545	710.2079	108.4261
1370445_at	85311	Pspla1	phosphatidylserine-specific phospholip	309.5104	159.7021	356.8446
1383007_at	300754	Bbs4_pred	Bardet-Biedl syndrome 4 homolog (hu	309.494	259.8106	36.89661
1379568_at	294091	Ifit2	interferon-induced protein with tetratric	309.4926	75.12689	242.7945
1392602_at	361195	Cdc14b_pr	CDC14 cell division cycle 14 homolog	309.3868	114.5966	250.3725
1387516_at	117554	Pnliprp2	pancreatic lipase-related protein 2	309.2389	52.36633	2634.263
1379148_at	58942	Cacnb4	calcium channel, voltage-dependent, b	309.2127	140.926	83.22395
1370813_at	64352	Gstm5	glutathione S-transferase, mu 5	309.1159	4052.079	1124.767
1377145_at	294614	RGD15621	similar to very large G-protein coupled	309.054	939.4338	502.809
1390560_at	360912	LOC36091	similar to DNA helicase HEL308	308.857	300.6185	197.8102
1369420_at	117185	Neu3	neuraminidase 3	308.8434	235.4771	443.5937
1384660_at	308068	Nkd2_pred	naked cuticle 2 homolog (Drosophila) (	308.7497	159.4161	155.2065
1382511_at	399489	E2f1	E2F transcription factor 1	308.5205	1731.832	206.7387
1368740_at	25041	P2rxl1	purinergic receptor P2X-like 1, orphan	308.4494	136.46	38.61995
1397995_at	313060	RGD15609	similar to SPOC domain containing 1 (	308.4092	327.4791	254.9567
1376612_at	296119	Dtwd1	DTW domain containing 1	308.3967	588.4475	371.3026
1379009_at	501048	RGD15650	similar to RIKEN cDNA 2510040D07 (	308.3898	179.405	187.6937
1384723_at	286971	Fut11	fucosyltransferase 11	308.2693	407.6139	238.95
1368253_at	25257	Gamt	guanidinoacetate methyltransferase	308.1836	300.7468	630.7682
1390627_a_at	353305	Tbx3	T-box 3	308.1126	250.2093	229.5523
1368762_at	29168	Ubd	ubiquitin D	307.9109	182.3943	150.9356
1378423_at	362826	Atcay_prec	ataxia, cerebellar, Cayman type (cayta	307.9033	112.0845	150.0149
1395127_at	361931	RGD13099	similar to RIKEN cDNA 4932438A13 (	307.7461	241.0827	611.2985
1379183_at	308492	RGD13067	similar to hypothetical protein	307.7048	381.449	225.8803
1391130_at	308318	RGD13087	similar to Zinc finger protein OZF (POZ	307.5661	173.7432	229.6609
1390830_at	499602	LOC49960	hypothetical protein LOC499602	307.5347	42.13203	197.7851
1387511_at	24894	Cyp2a1	Cytochrome P450 IIA1 (hepatic steroic	307.3764	128.0116	192.4456
1397155_at	29710	Scn8a	sodium channel, voltage-gated, type V	307.3312	178.0363	246.1701
1392679_at	365176	RGD13105	similar to TTF-I interacting peptide 20;	307.2541	219.213	90.789
1368927_at	29579	Mbc2	membrane bound C2 domain containir	307.2136	322.0895	228.4064
1382113_at	494340	MGC72612	similar to expressed sequence AI4491	307.0477	556.5264	1119.097
1393677_at	307459	Arhgap26_	Rho GTPase activating protein 26 (pre	307.0169	159.7158	132.6969
1371266_at	282708	Afm	afamin	307.0014	909.1663	1641.448
1379650_at	308000	Pdzk8_pre	PDZ domain containing 8 (predicted)	306.8292	147.9354	144.9454
1378612_at	313202	RGD13109	similar to RIKEN cDNA E130308A19 (	306.8242	87.13385	68.08506
1393975_a_at	313774	RGD13070	similar to RIKEN cDNA 1110035L05 (	306.7861	335.6931	539.7661
1398260_a_at	79224	Serpind1	serine (or cysteine) peptidase inhibitor	305.9833	15.21348	252.1458
1374308_at	116486	Sec14l2	SEC14-like 2 (S. cerevisiae)	305.7438	518.9298	204.3835
1377172_at	362021	RGD15609	similar to Pins (predicted)	305.4122	2052.417	421.0988
1387445_at	29353	Phkg1	phosphorylase kinase gamma 1	305.2493	1266.952	255.1265
1370469_at	246781	Ptpn7	protein tyrosine phosphatase, non-rece	305.237	239.6919	43.03098
1378312_at	316356	RGD15622	similar to RIKEN cDNA 4931419K03 (	305.025	179.4	15.54546
1368612_at	25724	Itgb4	integrin beta 4	304.9357	51.72425	152.3618
1377015_at	361602	Me3_predi	malic enzyme 3, NADP(+)-dependent,	304.7224	767.0199	1035.538
1396763_at	301455	LOC30145	NA	304.7055	111.0692	411.8816
1381228_at	303287	Fbxo39	F-box protein 39	304.4465	470.0109	1313.411

1381654_at	313866	RGD13049	similar to CG12467-PA (predicted)	303.9388	73.8609	1391.671
1368678_at	24225	Bdnf	brain derived neurotrophic factor	303.8755	291.812	278.5231
1393518_at	296352	Wfdc5_pre	WAP four-disulfide core domain 5 (pre	303.8608	118.1423	149.0771
1393090_at	503246	NA	NA	303.8389	183.6963	291.8248
1368524_at	25327	Kcnc1	potassium voltage gated channel, Sha	303.8141	292.3294	441.2947
1372282_at	294744	RGD13075	similar to RIKEN cDNA 2310016C16 (p	303.5464	169.8638	713.0421
1377631_at	362285	Col9a3_pre	procollagen, type IX, alpha 3 (predicte	303.483	31.75635	547.3177
1371756_at	369017	Krt2-5	keratin complex 2, basic, gene 5	303.4751	175.327	65.5594
1376850_a_at	362505	Ccl27_prec	chemokine (C-C motif) ligand 27 (pred	303.289	481.811	467.1699
1369552_at	170637	Samsn1	SAM domain, SH3 domain and nuclea	303.1858	37.63227	37.37813
1376769_at	363135	Rad54l2_p	Rad54 like 2 (S. cerevisiae) (predicted	303.0494	227.7402	361.164
1388972_at	113912	Rtn4r	reticulon 4 receptor	302.9879	494.4291	406.7064
1386969_at	83834	Nrn1	neuritin	302.7615	255.1392	324.5551
1368358_a_at	94202	Ptprr	protein tyrosine phosphatase, receptor	302.7231	814.6323	2993.475
1388197_at	79241	Hoxa5	homeo box A5	302.6189	191.8726	399.0772
1389214_at	309816	RGD15600	similar to Laminin alpha-4 chain precu	302.4574	117.2844	180.795
1394826_at	499155	RGD15614	similar to Fanconi anemia, complemen	302.1687	355.6956	209.5874
1369320_at	81510	Mia1	melanoma inhibitory activity 1	302.128	194.5258	235.0792
1384561_at	65188	Muc5ac	mucin 5, subtypes A and C, tracheobro	302.1172	259.3115	259.1479
1398150_at	364385	RGD15652	similar to 40S ribosomal protein S17 (p	302.1032	1915.73	57.1067
1374365_at	292041	Wwox_pre	WW domain-containing oxidoreductase	302.0828	220.4828	72.31982
1385797_at	29275	Actc1	actin alpha cardiac 1	301.9256	348.4491	33.55384
1371015_at	24575	Mx1	myxovirus (influenza virus) resistance	301.7709	255.4632	333.32
1391082_at	295936	RGD13056	similar to PR-domain protein 11 (predi	301.713	69.98187	40.88383
1370664_a_at	286986	LOC28698	putative pheromone receptor Go-VN13	301.5512	104.0199	202.4714
1370390_at	245982	Coro6	coronin, actin binding protein 6	301.1822	211.4573	519.9899
1382874_at	304860	LOC30486	similar to N-acetylneuraminate pyruvat	301.1696	6.953579	416.9547
1378349_at	304872	NA	NA	300.9813	16.49158	38.41041
1375653_at	116508	Nrxn3	neurexin 3	300.9677	142.9683	196.9315
1392418_at	116554	Mapk8	mitogen-activated protein kinase 8	300.855	84.55567	158.9809
1384068_at	306575	Ckap2_pre	cytoskeleton associated protein 2 (pre	300.8172	8102.045	100.8954
1388084_at	24933	Klra22	killer cell lectin-like receptor subfamily	300.7281	31.91385	74.78027
1387255_at	25120	Aanat	arylalkylamine N-acetyltransferase	300.6479	173.5517	20.74078
1368725_at	29146	Jag1	jagged 1	300.5317	51.45338	139.212
1389350_at	287774	Apoh	apolipoprotein H	300.4046	96.83858	2559.86
1369280_at	84429	Kcnk9	potassium channel, subfamily K, meml	300.386	188.3649	71.9175
1377189_at	292680	RGD13073	similar to BC282485_1 (predicted)	300.2779	720.5601	42.21198
1382803_at	292763	Map4k1_pr	mitogen activated protein kinase kinas	300.2373	76.41492	238.4795
1379061_at	498517	RGD15656	similar to mKIAA0323 protein (predicte	300.1633	83.79478	180.0358
1379752_at	310909	Rap1gds1_	RAP1, GTP-GDP dissociation stimulat	299.8762	39.53975	232.1826
1369511_at	24326	Ednra	endothelin receptor type A	299.8562	287.6382	299.1425
1369633_at	24772	Cxcl12	chemokine (C-X-C motif) ligand 12	299.7968	338.2322	2134.943
1379228_at	499148	NA	NA	299.789	505.8072	646.6811
1371310_s_at	29345	Serpinh1	serine (or cysteine) proteinase inhibito	299.7879	170.8348	609.1824
1372042_at	291813	Cmtm3_pre	CKLF-like MARVEL transmembrane d	299.7455	35.29247	150.0988
1371025_at	64459	Masp2	mannan-binding lectin serine peptidas	299.6756	98.1141	52.28464
1379489_at	361595	NA	NA	299.6228	147.905	195.5758
1378586_at	83681	Cish	cytokine inducible SH2-containing prot	299.5057	1526.747	939.4178
1382266_at	498153	RGD15607	similar to G protein-coupled receptor 1	299.5033	94.45607	110.8655
1370497_at	353498	Cyp11b3	cytochrome P450, subfamily 11B, poly	299.167	26.989	124.1005
1370755_at	140725	Cacng4	calcium channel, voltage-dependent, g	299.1097	276.4344	177.6786

1384476_at	361245	Cmah	cytidine monophospho-N-acetylneuran	298.8871	78.14004	73.05529
1369368_at	140933	Trpc5	transient receptor potential cation char	298.7633	71.78097	164.7066
1378767_at	310781	RGD1307(	hypothetical LOC310781	298.5899	235.6696	196.1689
1387756_s_at	113882	Hemgn	hemogen	298.3234	74.11425	121.5782
1392804_at	287114	Dnase1l2_	deoxyribonuclease 1-like 2 (predicted)	298.2517	157.4811	249.9312
1397865_at	502580	NA	NA	298.2315	293.2191	161.8934
1385290_at	499865	RGD15652	similar to RIKEN cDNA 1700095F04 (p	298.0579	71.33175	139.8318
1367628_at	56646	Lgals1	lectin, galactose binding, soluble 1	298.0462	114.2393	591.3753
1368674_at	64035	Pygl	liver glycogen phosphorylase	298.0406	718.5373	587.0852
1371268_at	290050	Olr1641_pr	olfactory receptor 1641 (predicted)	297.6936	125.7985	240.7513
1392406_at	298439	Ipp_predict	IAP promoted placental gene (predicte	297.639	573.0043	278.6716
1371258_at	361969	Fga	fibrinogen, alpha polypeptide	297.6092	26.84708	63.5667
1387548_at	25694	Has2	hyaluronan synthase 2	297.5121	60.10139	230.9082
1387210_at	29495	Dlgh4	discs, large homolog 4 (Drosophila)	297.4124	558.8661	201.6721
1369700_at	29233	Clcn7	chloride channel 7	297.1145	497.0715	69.62672
1370612_at	25468	Kcna2	potassium voltage-gated channel, shal	297.0336	129.8961	348.096
1396173_at	361625	Nrip3_pred	nuclear receptor interacting protein 3 (	296.8169	443.4533	27.76206
1368657_at	171045	Mmp3	matrix metallopeptidase 3	296.812	108.6756	41.14665
1376403_at	259227	Vof16	ischemia related factor vof-16	296.8016	186.6844	340.8383
1370448_at	171517	Gpc2	glypican 2 (cerebroglycan)	296.7751	145.0552	115.9064
1368478_at	24316	Drd1a	dopamine receptor D1A	296.7171	38.33317	211.4506
1368458_at	25428	Cyp7a1	cytochrome P450, family 7, subfamily a	296.6018	27.85724	102.9244
1370126_at	25052	Prss2	protease, serine, 2	296.3661	13.81803	944.3333
1395083_at	298992	Nova1	neuro-oncological ventral antigen 1	296.3112	12.88229	308.1395
1367948_a_at	25589	Kdr	kinase insert domain protein receptor	296.3055	501.1736	635.0633
1369572_at	29265	Mcpt1	mast cell protease 1	296.2016	157.3015	208.9254
1387605_at	156117	Casp12	caspase 12	296.0185	115.7239	301.8513
1387505_at	25686	Gnai1	guanine nucleotide binding protein, alp	295.9906	1874.636	1318.345
1396726_at	361087	RGD13086	similar to 9630044O09Rik protein	295.9532	24.40404	58.15318
1387589_at	65025	Rims3	regulating synaptic membrane exocyto	295.8947	215.0602	248.4878
1368539_at	78956	Scn9a	sodium channel, voltage-gated, type IX	295.8425	99.4943	5790.541
1367866_at	29158	Fbln5	fibulin 5	295.8134	93.25629	304.6173
1380538_at	503242	NA	NA	295.6154	466.8669	350.8565
1387987_at	286961	Slc22a19	solute carrier family 22 (organic anion	295.5029	95.97255	279.4405
1389527_at	378467	LOC37846	promethin	295.4591	1907.308	1445.096
1374572_at	360969	RGD13047	similar to opposite strand transcription	295.4366	202.2453	276.4841
1397062_at	298120	NA	NA	295.3843	198.01	203.7788
1371356_at	315326	Tenc1_pre	tensin like C1 domain containing phosp	295.2695	251.297	88.19714
1371117_at	361170	Adam32	a disintegrin and metalloprotease dom	295.2612	100.7464	29.08474
1369763_at	59077	Gprk2l	G protein-coupled receptor kinase 2, g	295.0301	202.3796	372.8559
1392765_at	680415	LOC68041	NA	294.9482	60.17384	29.82795
1369470_at	60332	Akap14	A kinase (PRKA) anchor protein 14	294.914	72.76603	151.0595
1381576_at	289021	Pik3c2b_pr	phosphoinositide-3-kinase, class 2, be	294.8091	268.4386	316.7277
1388258_at	112400	Nrg1	neuregulin 1	294.6188	156.4677	156.5689
1369584_at	89829	Socs3	suppressor of cytokine signaling 3	294.3982	160.28	186.1485
1396677_at	362984	NA	NA	294.386	29.43982	115.4859
1393020_at	500548	NA	NA	294.2324	2986.834	938.2767
1368793_at	29712	Kcnj2	potassium inwardly-rectifying channel,	294.2195	105.5284	232.6675
1393620_at	315427	Sesn3_pre	sestrin 3 (predicted)	294.0672	262.6993	78.21475
1371199_at	63881	Rapgef1	Rap guanine nucleotide exchange fact	294.0046	58.84395	110.0721
1384499_at	361638	Bucs1_pre	butyryl Coenzyme A synthetase 1 (pre	293.7065	88.17759	39.237

1385041_at	294043	Sorcs3_pre	sortilin-related VPS10 domain containi	293.6307	189.8405	147.9515
1369121_at	59304	Gdf9	growth differentiation factor 9	293.5547	148.4012	147.0587
1392209_at	286976	Gcnt3	glucosaminyl (N-acetyl) transferase 3,	293.5306	56.27497	242.6336
1394408_at	501185	RGD15636	similar to hypothetical protein FLJ2267	293.1042	117.5657	90.68543
1397578_at	297333	Smyd1_pre	SET and MYND domain containing 1 (	292.9254	164.7828	61.09638
1376449_at	304971	NA	NA	292.9157	40.72135	320.121
1369377_at	25605	Hcrtr2	hypocretin (orexin) receptor 2	292.7667	72.78308	65.17124
1390891_at	499350	NA	NA	292.7587	6020.045	334.9357
1381352_at	366035	RGD15625	similar to cis-Golgi matrix protein GM1	292.5984	197.4539	78.75961
1387728_at	117539	Il10ra	interleukin 10 receptor, alpha	292.5737	177.6089	142.2642
1388288_at	140860	Slco2b1	solute carrier organic anion transporte	292.3992	76.39108	353.2415
1380281_at	362559	RGD13078	similar to KIAA1730 protein (predicted)	292.0386	189.7942	31.94563
1389548_at	362474	Adhfe1	alcohol dehydrogenase, iron containin	291.8785	48.47423	72.89943
1392845_at	497898	RGD1560C	similar to KID2 (predicted)	291.6209	223.3462	211.783
1377395_at	308668	Nipa1_pred	non imprinted in Prader-Willi/Angelman	291.345	20.42483	138.8469
1384173_at	286975	Scap1	src family associated phosphoprotein 1	291.1732	149.5523	786.4016
1387650_at	192205	Prok1	prokineticin 1	290.9942	123.3678	179.6107
1390812_a_at	502916	RGD15628	similar to RAS-like, estrogen-regulatec	290.7359	50.21062	384.2787
1374342_at	294241	Ly6g6c	lymphocyte antigen 6 complex, locus C	290.6886	111.5577	233.7502
1380536_at	300207	RGD13059	hypothetical LOC300207 (predicted)	290.6621	70.40907	61.34151
1387474_at	24807	Tacr1	tachykinin receptor 1	290.5273	180.9767	178.5239
1378390_at	291802	RGD15656	similar to zinc finger protein 124 (predi	290.4783	274.2434	516.4323
1370619_at	117551	Ccl22	chemokine (C-C motif) ligand 22	290.2671	247.2671	113.9775
1371137_at	252898	Acox2	acyl-Coenzyme A oxidase 2, branchec	289.7897	81.19802	119.3158
1381622_at	365546	RGD15631	similar to chromosome 21 open readin	289.7123	80.74056	108.4966
1391634_at	501766	RGD1562C	similar to RIKEN cDNA 2310057N15 (	289.5987	166.9676	97.77111
1398688_at	361319	Spink5_pre	serine protease inhibitor, Kazal type 5	289.2937	127.7054	102.28
1396357_at	304725	LOC30472	NA	289.1857	107.8972	230.4312
1370997_at	29546	Homer1	homer homolog 1 (Drosophila)	289.149	218.4061	9257.066
1368882_at	29758	St6galnac3	ST6 (alpha-N-acetyl-neuraminyl-2,3-be	289.0873	118.2297	233.4203
1391083_at	306279	Arhgap22_	Rho GTPase activating protein 22 (pre	289.0743	405.7416	32.69716
1369803_at	117034	Ptf1a	pancreas specific transcription factor, 1	288.8003	55.70772	239.8702
1387253_at	64055	Guca2b	guanylate cyclase activator 2b	288.6913	268.8412	152.9439
1390469_at	361791	Nrm	nurim (nuclear envelope membrane pr	288.3589	564.6206	224.5358
1379281_at	266803	Sostdc1	sclerostin domain containing 1	288.2685	125.9525	316.8528
1376313_at	309139	Tpcn2_pre	two pore segment channel 2 (predictec	288.1673	130.1082	43.00072
1368626_at	54263	Kcnn3	potassium intermediate/small conducta	288.0663	125.1755	68.47013
1387958_at	171516	Akr1c18	aldo-keto reductase family 1, member	287.8671	14.05409	24.84202
1384942_at	314164	Lrfn5_pred	leucine rich repeat and fibronectin type	287.8524	40.29158	81.87423
1379877_at	362925	Zfp406_pre	zinc finger protein 406 (predicted)	287.7142	591.3138	432.9852
1373436_at	361834	RGD13067	similar to RIKEN cDNA 1700040L02 (p	287.6613	550.9989	716.2519
1369921_at	57298	Gstm4	glutathione S-transferase M4	287.6129	157.4229	71.3437
1391924_at	361187	LOC36118	similar to ankyrin repeat and SOCs bo	287.5581	172.6133	244.8203
1369422_at	192203	Fap	fibroblast activation protein	287.4444	98.49742	256.7917
1378983_at	498944	RGD15653	similar to translin-associated factor X (	287.4339	818.0704	227.5328
1379075_at	313997	Oact2	O-acyltransferase (membrane bound)	287.4058	706.9503	829.6712
1370707_at	246271	Fev	FEV (ETS oncogene family)	287.1554	291.4075	1570.742
1387852_at	25357	Thrsp	thyroid hormone responsive protein	287.0637	17.65771	64.47956
1375502_at	498858	RGD15619	similar to mutated in bladder cancer 1	286.9576	172.3577	43.05049
1387333_at	114103	Il5ra	interleukin 5 receptor, alpha	286.8564	61.52788	82.77005
1387717_at	85240	Plcb2	phospholipase C, beta 2	286.7694	105.1329	116.0241

1394885_at	291535	RGD15654	RGD1565482 (predicted)	286.656	196.6415	199.383
1369205_at	29410	Neurod3	neurogenic differentiation 3	286.4176	30.39932	207.7037
1392857_at	500549	RGD15629	similar to Zfp31 protein (predicted)	286.3296	185.5582	325.3617
1371106_at	25713	Itgb7	integrin, beta 7	286.2858	242.3495	250.5371
1388545_at	314280	Smoc1	SPARC-related modular calcium bindi	286.2362	877.6878	163.5305
1395649_at	353230	Lhfpl4	lipoma HMGIC fusion partner-like prote	286.0488	132.8416	75.6913
1371120_s_at	25245	Bdkrb2	bradykinin receptor, beta 2	286.0118	177.9989	157.3664
1370922_at	29145	Ctxn	cortexin	286.0117	766.1979	162.8339
1376646_at	360718	Popdc2	popeye domain containing 2	285.9952	232.3435	294.7455
1394501_at	292844	Siglec10_p	sialic acid binding Ig-like lectin 10 (pre	285.9268	118.1186	244.112
1378452_at	497040	LOC49704	NA	285.7968	169.0905	203.6384
1378482_at	362325	Mdfic_pred	MyoD family inhibitor domain containi	285.7066	206.3371	239.9498
1388133_at	266600	Csdc2	cold shock domain containing C2, RN/	285.6081	369.6307	197.1261
1374219_at	289338	Disp1_pred	dispatched homolog 1 (Drosophila) (pr	285.4462	29.00117	234.4467
1368643_at	171413	Spata6	spermatogenesis associated 6	285.3757	1116.702	846.6916
1393170_at	500125	RGD15614	similar to homeobox-containing transc	285.2928	22.8582	214.4131
1368176_at	24705	Rara	retinoic acid receptor, alpha	285.1848	167.1415	308.2107
1379430_at	311437	Rassf2	Ras association (RalGDS/AF-6) doma	285.1543	31.97184	6.743756
1387789_at	170909	Erg	v-ets erythroblastosis virus E26 oncog	284.9845	119.4376	135.4991
1370937_a_at	81008	Itga7	integrin alpha 7	284.9679	155.3064	231.5942
1387851_at	63852	Pter	phosphotriesterase related	284.924	308.7728	11.85934
1378430_at	294119	Moxd1	monooxygenase, DBH-like 1	284.7823	370.6856	349.2348
1370960_at	25285	Igfbp5	insulin-like growth factor binding protei	284.7314	273.7689	275.8101
1392189_at	500818	RGD15620	similar to regulatory factor X 4 variant	284.3711	14.44964	388.1656
1397161_a_at	29491	Itsn1	intersectin 1	284.3672	578.9288	183.2376
1385001_at	315084	Gsdmdc1_	gasdermin domain containing 1 (predic	284.3017	21.7505	355.5077
1398267_at	89776	Slc22a7	solute carrier family 22 (organic anion	284.118	172.7782	90.0515
1372297_at	300850	Gsta4	glutathione S-transferase, alpha 4	283.9856	308.4069	1150.25
1389274_at	360639	Dcakd	dephospho-CoA kinase domain contai	283.9283	1776.435	741.0737
1379147_at	60444	Kcnt1	potassium channel, subfamily T, meml	283.8635	323.3283	432.2724
1395955_at	311184	C1qtnf4_pr	C1q and tumor necrosis factor related	283.743	224.3464	142.675
1377645_at	360479	LOC36047	similar to hypothetical protein	283.6193	203.0638	209.4052
1385441_at	365550	Vpreb3_pre	pre-B lymphocyte gene 3 (predicted)	283.5667	23.83491	313.4701
1389877_at	364706	LOC36470	NA	283.5611	269.3532	167.1786
1367852_s_at	116728	5-Sep	septin 5	283.5524	2347.183	690.6028
1396467_at	361843	Amid_pred	apoptosis-inducing factor (AIF)-like mit	283.2872	206.04	122.6521
1391941_at	304237	NA	NA	283.1012	454.0427	425.4956
1368023_at	84491	Qscn6	quiescin Q6	283.0806	662.4418	1467.239
1393888_at	25378	Amh	anti-Mullerian hormone	282.9625	124.8612	317.6715
1370860_at	297081	Svs1	seminal vesicle secretion 1	282.9353	146.5228	198.5246
1377223_at	24883	Wt1	Wilms tumor 1	282.8101	77.74642	231.1417
1378728_at	60589	Epha8	Eph receptor A8	282.475	178.2845	90.63306
1392779_at	500822	NA	NA	282.4683	156.3293	238.6395
1386681_at	497934	LOC49793	similar to hypothetical protein FLJ2001	282.4604	325.8164	206.4946
1388049_a_at	65191	Gabre	gamma-aminobutyric acid A receptor,	282.406	12.97656	121.3458
1390934_at	27064	Ankrd1	ankyrin repeat domain 1 (cardiac musc	282.2442	76.84809	172.9183
1381544_at	501576	RGD15645	similar to CHCHD4 protein (predicted)	282.203	45.24082	60.64212
1387091_at	29511	Padi2	peptidyl arginine deiminase, type II	282.0245	295.0047	408.6751
1369048_at	29689	Gabrd	gamma-aminobutyric acid A receptor,	282.0111	3031.541	379.2177
1373036_at	364885	RGD15614	similar to Ras GTPase-activating-like p	282.0088	859.2055	433.0897
1393201_at	500361	RGD15620	similar to lymphoid-restricted membrar	281.9459	198.1829	226.0193

1368563_at	79251	Aspa	aspartoacylase	281.9402	39.84453	51.0168
1387007_at	25454	Gfra1	glial cell line derived neurotrophic facto	281.8138	330.1182	534.9528
1396979_at	500566	MGC1144	similar to RIKEN cDNA 4930555I21	281.7871	60.53424	55.56651
1368281_at	94199	Dpep1	dipeptidase 1 (renal)	281.7776	249.5063	119.9915
1393278_at	310616	Creb3l4	cAMP responsive element binding prot	281.7648	41.40349	149.6548
1373717_at	116597	Opcml	opioid binding protein/cell adhesion mo	281.7533	160.9856	196.8792
1375080_at	309173	NA	NA	281.5962	175.8797	65.65076
1369105_a_at	24678	Pkib	protein kinase inhibitor beta, cAMP de	281.5837	57.30245	748.6842
1369173_at	84007	C3ar1	complement component 3a receptor 1	281.4416	95.17013	125.2781
1392755_at	289443	Lrrc8c	leucine rich repeat containing 8 family,	281.3409	342.2133	625.2389
1380331_at	499205	RGD15646	similar to A830059I20Rik protein (pred	281.1379	92.41742	110.9651
1374338_at	361993	RGD1562C	similar to RIKEN cDNA 1110038F21 (p	280.9042	222.6838	15.78126
1370589_at	266773	Znf14	zinc finger protein 14 (KOX 6)	280.6364	531.4982	312.0496
1370090_at	155918	Lcp2	lymphocyte cytosolic protein 2	280.1731	202.6205	175.8715
1393581_at	289054	Aspm_prec	asp (abnormal spindle)-like, microceph	279.6776	3752.136	431.483
1369089_at	24681	Prkcc	protein kinase C, gamma	279.4612	114.9789	148.9556
1388422_at	361303	Lims2	LIM and senescent cell antigen like do	279.2999	43.85965	62.51952
1370408_at	286910	Nid67	putative small membrane protein NID6	279.2308	25.06379	926.794
1368691_at	29628	Gria3	glutamate receptor, ionotropic, AMPA3	279.1441	14.52859	1524.981
1392801_at	498580	RGD15611	similar to novel protein (predicted)	279.1176	141.954	133.2748
1369895_s_at	192181	Podxl	podocalyxin-like	279.0239	296.1521	435.4548
1387984_at	245978	Cklf	chemokine-like factor	278.7926	541.8167	34.6763
1384408_at	293015	Map3k5_pr	mitogen-activated protein kinase kinas	278.7109	422.1373	939.4551
1376867_at	360983	RGD13074	similar to RIKEN cDNA 1110067D22 (	278.5655	955.9672	723.2023
1369469_s_at	192209	Spdy1	speedy homolog 1 (Drosophila)	278.2487	207.8689	411.8799
1370763_at	404968	Olr1496	olfactory receptor 1496	277.9461	58.66573	175.6547
1398380_at	298683	Vwa1	von Willebrand factor A domain contai	277.8573	34.09749	110.4212
1393196_at	311114	Klhl23_pre	kelch-like 23 (Drosophila) (predicted)	277.7576	1190.934	117.2292
1385425_at	293449	RGD13116	similar to RIKEN cDNA A230074B11 g	277.4966	90.74259	317.3024
1373282_at	313720	RGD13066	similar to CG7744-PA (predicted)	277.4856	1141.503	4489.637
1385209_at	306571	Myst3	MYST histone acetyltransferase (mon	277.4083	118.2997	155.2937
1395391_at	498677	NA	NA	277.3817	70.72417	224.3695
1370400_at	24750	RT1-N3	RT1 class Ib gene, H2-TL-like, grc reg	277.3092	69.49658	116.141
1391018_at	315820	Myo5c_pre	myosin VC (predicted)	277.1857	116.6293	167.2448
1396158_at	366232	Rem1	rad and gem related GTP binding prote	277.1492	112.5072	133.9836
1396703_at	289502	RGD13085	similar to RIKEN cDNA 4932413O14 g	277.131	36.16039	187.6397
1371070_at	171091	Zbp1	Z-DNA binding protein 1	277.0608	60.8058	152.2248
1372967_at	314904	Geft	RAC/CDC42 exchange factor	277.0438	1059.395	631.3775
1368701_at	24213	Atp1a3	ATPase, Na+/K+ transporting, alpha 3	277.0211	621.8012	50.8912
1394470_at	298890	RGD13059	similar to hypothetical protein DKFZp5	276.8589	94.45477	174.7554
1373503_at	360779	Lrch4_prec	leucine-rich repeats and calponin hom	276.7317	436.8507	750.6405
1398176_at	309859	Scml4_pre	sex comb on midleg-like 4 (Drosophila	276.5799	204.7428	215.9678
1374175_at	317368	RGD15649	similar to porcupine-D (predicted)	276.4687	884.9254	229.5438
1380802_at	288504	Snx8_pred	sorting nexin 8 (predicted)	276.4419	40.24147	28.42644
1380352_at	309077	Dock1_pre	dedicator of cyto-kinesis 1 (predicted)	276.3704	30.52953	223.1324
1370108_a_at	85327	Lin7a	lin-7 homolog a (C. elegans)	276.2877	733.1308	25.24475
1371279_at	498753	RGD15647	Histone H2a (predicted)	276.2214	442.3448	193.2353
1370462_at	25460	Hmmr	hyaluronan mediated motility receptor	275.9476	4370.75	341.5946
1391545_at	288111	Ccdc52	coiled-coil domain containing 52	275.8432	886.2911	339.4298
1387570_at	140808	Manea	mannosidase, endo-alpha	275.7415	262.1642	325.7445
1378045_at	363686	C1ql1_prec	complement component 1, q subcomp	275.6748	114.5634	156.39

1368579_at	116474	Prlpm	prolactin-like protein M	275.6573	49.29159	84.90108
1369248_a_at	63879	Birc4	baculoviral IAP repeat-containing 4	275.6485	506.6364	117.293
1369608_at	60464	Fgf16	fibroblast growth factor 16	275.5616	283.3444	125.5773
1372936_at	304195	Pcp2	Purkinje cell protein 2 (L7)	275.5418	144.2313	192.8787
1369238_at	83711	Inhbe	inhibin beta E	275.5142	175.7002	214.7617
1380577_at	312382	Abcg2	ATP-binding cassette, sub-family G (W	275.4759	159.1449	342.2611
1380443_at	362856	Pwp1_prec	PWP1 homolog (S. cerevisiae) (predic	275.4025	753.6626	443.5744
1379226_at	298543	Aim1l_prec	absent in melanoma 1-like (predicted)	275.204	201.0972	412.918
1371017_at	24821	Tcrg	T-cell receptor gamma chain	275.0533	245.5277	152.5228
1398277_at	79558	Acvr1	activin A receptor, type 1	274.9877	285.6989	21.53135
1379885_at	246247	Fmo4	flavin containing monooxygenase 4	274.9353	134.0884	161.6269
1381708_at	299188	Zfyve1_pre	zinc finger, FYVE domain containing 1	274.8133	62.24106	418.264
1387495_at	25565	Tle4	transducin-like enhancer of split 4, E(s	274.4876	1454.58	181.3803
1393121_at	298961	Agr2_predi	anterior gradient 2 (Xenopus laevis) (p	274.3218	152.3215	69.0765
1373357_at	313504	Foxd2_pre	forkhead box D2 (predicted)	274.2886	663.9269	174.3518
1376697_at	304322	Chst12	carbohydrate sulfotransferase 12	274.2763	242.449	481.0979
1397231_at	541462	Sprn	shadow of prion protein	274.2555	72.09197	198.5585
1381821_at	314180	Mamdc1	MAM domain containing glycosylphosp	273.9415	44.63997	880.8311
1370897_at	25244	Bckdha	branched chain ketoacid dehydrogena	273.8802	377.0879	333.2466
1383119_at	316290	Ogfrl1	opioid growth factor receptor-like 1	273.856	632.543	182.2643
1367555_at	24186	Alb	albumin	273.8192	70.31589	287.2437
1395635_at	361685	Tnfrsf26_p	tumor necrosis factor receptor superfa	273.8099	99.38807	202.9572
1388048_a_at	116699	Inpp4b	inositol polyphosphate-4-phosphatase,	273.7959	193.7473	429.6915
1396118_at	298409	RGD15649	RGD1564942 (predicted)	273.7726	221.7772	76.17764
1388831_at	116501	Slc9a3r2	solute carrier family 9 (sodium/hydroge	273.7151	387.1557	108.9216
1370852_at	171573	LOC17157	spleen protein 1 precursor	273.412	72.9665	86.18646
1374763_at	296616	Ceecam1	cerebral endothelial cell adhesion mole	273.3356	316.2928	639.9087
1387694_at	85258	Cnga4	cyclic nucleotide gated channel alpha 4	273.1451	122.5837	39.60152
1391865_at	302977	Rnf151_pre	ring finger protein 151 (predicted)	272.8974	400.2693	131.5589
1390502_at	289084	RGD13091	similar to RIKEN cDNA 1700025G04 g	272.883	2474.628	888.4441
1368751_at	83588	Kcns3	potassium voltage-gated channel, dela	272.8231	164.8971	373.8237
1394848_at	498725	NA	NA	272.6632	153.4013	149.7234
1390061_at	24837	Tnnt2	troponin T2, cardiac	272.5866	18.47173	155.9382
1384312_at	306659	Irx1_predic	Iroquois related homeobox 1 (Drosoph	272.5224	133.7017	19275.16
1372807_at	360735	NA	NA	272.5134	513.2588	408.7615
1379950_at	59300	Cml2	Camello-like 2	272.5005	454.3605	187.3299
1371062_at	291133	Aldh5a1	aldehyde dehydrogenase family 5, sub	272.4814	456.9493	137.8982
1368226_at	171047	RGD62038	Nucleoside 2-deoxyribosyltransferase	272.4804	1689.674	430.3616
1369865_at	25660	Cd28	CD28 antigen	272.4734	147.0181	124.7297
1387765_at	24548	Mbl1	mannose binding lectin 1, protein A	272.4263	59.87434	165.9969
1377271_at	303373	Unc45b_pr	unc-45 homolog B (C. elegans) (predic	272.3339	76.43037	116.2714
1387746_at	56821	Olr414_pre	olfactory receptor 414 (predicted)	272.2416	108.8094	120.9493
1376197_at	363595	Tcf7_predi	transcription factor 7, T-cell specific (p	272.24	101.828	204.5264
1375544_at	363878	NA	NA	272.1834	198.3786	172.159
1368592_at	24493	Il1a	interleukin 1 alpha	272.1303	48.45787	304.6585
1386884_at	65164	Htra1	HtrA serine peptidase 1	272.1091	41.81236	462.3244
1372015_at	306562	Tacc1	transforming, acidic coiled-coil contain	271.9951	2622.881	4224.984
1389324_at	89806	Tie1	tyrosine kinase with immunoglobulin-lik	271.9471	63.25855	209.3668
1374098_at	406166	Ng3	Ng3 protein	271.8322	525.5986	678.1111
1387894_at	54254	Gata4	GATA binding protein 4	271.6765	923.8457	127.9651
1385489_at	56003	3-Sep	septin 3	271.6398	461.0898	182.8632

1381550_at	498015	LOC49801	NA	271.5891	167.101	106.7777
1387536_at	25665	Scn5a	sodium channel, voltage-gated, type V	271.5674	145.2874	145.9342
1398158_at	498334	NA	NA	271.4664	233.2404	110.3885
1369499_at	29261	Tyms	thymidylate synthase	271.3728	1263.837	469.351
1369495_at	64680	Crhr2	corticotropin releasing hormone recept	271.0051	161.5918	238.4631
1387645_at	29151	Ucn	urocortin	270.9887	125.9074	181.9053
1367816_at	171160	Hod	homeobox only domain	270.9604	2197.582	1883.136
1387200_at	60394	Olig1	oligodendrocyte transcription factor 1	270.8779	205.6892	295.9487
1398685_at	500611	NA	NA	270.7343	305.0464	148.635
1397034_at	301742	RGD15662	similar to Coatomer gamma-2 subunit	270.7229	218.2012	276.9014
1372055_at	311647	RGD13065	similar to P-Rex1 (predicted)	270.671	2082.792	671.0209
1368787_at	170841	Mutyh	mutY homolog (E. coli)	270.511	245.4181	148.9346
1387802_at	65040	Dlgap1	discs, large (Drosophila) homolog-asso	270.5066	365.3653	220.2619
1375073_at	500601	NA	NA	270.4241	228.7024	1169.322
1397790_at	500001	NA	NA	270.2226	196.6967	228.3166
1368236_at	25684	Mep1a	meprin 1 alpha	270.1387	211.3503	166.9877
1375833_at	246116	Ptpn4	protein tyrosine phosphatase, non-rece	270.0498	175.7112	179.2677
1369496_at	117255	Ptpn12	protein tyrosine phosphatase, non-rece	269.9351	430.993	97.85653
1383585_s_at	297096	Snx10	sorting nexin 10	269.8486	3222.445	1639.655
1376105_at	314981	Col14a1_p	procollagen, type XIV, alpha 1 (predict	269.5644	112.5839	4303.427
1370753_at	299593	Olr1078	olfactory receptor 1078	269.3886	179.6127	158.1941
1372144_at	313811	Dnajb5_pre	DnaJ (Hsp40) homolog, subfamily B, m	269.2364	470.2264	130.819
1387719_at	25688	Clcn1	chloride channel 1	269.2254	150.5843	170.1553
1387034_at	24616	Pah	phenylalanine hydroxylase	269.2083	261.0023	2644.407
1383765_at	501668	MGC11438	similar to RIKEN cDNA 4921520P21	269.1941	750.867	954.6214
1385499_at	308850	RGD15658	similar to DRE1 protein (predicted)	269.0444	95.92417	111.6073
1372611_at	298377	RGD13052	similar to RIKEN cDNA 2010305A19 (p	268.965	1410.851	64.33338
1391986_at	307070	Larp5_pred	La ribonucleoprotein domain family, me	268.9624	113.553	107.1671
1387533_at	25525	Pspn	persephin	268.9043	147.1601	183.3533
1369287_at	60564	Syt9	synaptotagmin IX	268.8698	537.5733	181.6554
1369362_at	60445	Tsc1	tuberous sclerosis 1	268.8142	243.8666	277.3641
1384805_at	641632	Defb24	beta-defensin 24	268.7498	43.33468	72.64191
1394980_at	365924	Tmem56_p	transmembrane protein 56 (predicted)	268.6475	121.9206	233.274
1377853_at	308523	Zfp537_pre	zinc finger protein 537 (predicted)	268.1996	209.4727	919.5817
1369458_at	84477	Gab2	growth factor receptor bound protein 2	267.8771	161.6725	48.50996
1396880_at	307960	LOC30796	NA	267.8661	209.5761	314.8708
1370938_at	300067	Sh3bp1	NA	267.6845	980.4627	129.8957
1369352_at	83617	Hipk3	homeodomain interacting protein kinas	267.6395	489.7876	313.8833
1372646_at	363225	RGD13056	similar to RIKEN cDNA 1500015O10 (	267.5981	481.1652	203.9271
1368914_at	50662	Runx1	runt related transcription factor 1	267.5935	2495.556	103.3911
1369443_at	171100	Angptl2	angiopoietin-like 2	267.517	159.7423	75.66281
1389741_at	304208	Lass4_pred	longevity assurance homolog 4 (S. cer	267.4749	15.08193	129.0566
1392817_at	24171	Add2	adducin 2 (beta)	267.4738	429.3056	281.4204
1397100_at	361230	LOC36123	Ac1258	267.3806	39.78683	193.6238
1383252_at	292759	Sars2_pred	seryl-tRNA synthetase 2 (predicted)	267.1462	548.9666	107.0038
1376765_at	361348	RGD15598	similar to maestro (predicted)	267.1236	305.0071	54.23487
1368269_at	25474	Lgals4	lectin, galactose binding, soluble 4	267.069	135.8675	492.0882
1397411_at	309653	Fgd2_pred	FYVE, RhoGEF and PH domain conta	267.0559	48.94967	143.6829
1369732_a_at	64442	St3gal2	ST3 beta-galactoside alpha-2,3-sialyltr	266.635	640.652	745.6984
1373554_at	313722	Spsb1_pre	splA/ryanodine receptor domain and S	266.5285	137.7152	1580.687
1370088_at	85244	Spa17	sperm autoantigenic protein 17	266.2426	2794.386	1468.897

1377239_at	307171	Apbb1ip	amyloid beta (A4) precursor protein-bi	266.1076	30.41008	364.0843
1389891_at	294279	Col11a2_m	procollagen, type XI, alpha 2 (mapped	266.0273	225.5066	327.9904
1393910_at	362378	RGD13098	similar to Fam13a1 protein	266.0046	108.292	267.3858
1377901_at	304522	Fbxw8_pre	F-box and WD-40 domain protein 8 (pr	265.969	371.2363	226.2734
1369694_at	29482	Slc1a2	solute carrier family 1 (glial high affinity	265.9011	44.37186	131.8028
1387433_a_at	85262	Slc25a27	solute carrier family 25, member 27	265.8831	325.69	216.0802
1396263_at	299356	RGD13045	similar to RIKEN cDNA 4831426I19 (p	265.8136	525.7345	288.0109
1390336_at	360695	RGD13075	similar to Protein C21orf63 homolog pr	265.7944	38.26849	323.2069
1369050_at	116720	Pik3c2g	phosphatidylinositol 3-kinase, C2 doma	265.7566	193.4966	100.855
1377353_a_at	287437	Tnfsf13	tumor necrosis factor (ligand) superfan	265.6873	145.1011	526.2013
1368404_at	81653	Dbn1	drebrin 1	265.6599	4354.515	501.8126
1377902_a_at	297561	Rad52_pre	RAD52 homolog (S. cerevisiae) (predi	265.5517	244.2398	384.9983
1375519_at	287167	LOC28716	globin, alpha	265.5052	97.46196	170.4627
1389051_at	361801	Rxrb	retinoid X receptor beta	265.4527	474.8318	211.8222
1397180_at	360792	Styxl1	serine/threonine/tyrosine interacting-lik	265.367	38.06073	94.85006
1377278_at	25118	Itga1	integrin alpha 1	264.8553	107.4509	48.53041
1381277_at	303488	Hoxb3_pre	homeo box B3 (predicted)	264.7828	473.0363	310.131
1379480_at	362338	Dgki	diacylglycerol kinase, iota	264.7178	263.6728	122.458
1389423_at	83573	Ddr2	discoidin domain receptor family, mem	264.6333	38.26481	367.3553
1370096_at	50669	Prf1	perforin 1 (pore forming protein)	264.5145	22.75221	50.00016
1381397_at	366138	Ldlrad3_pr	low density lipoprotein receptor class A	264.4442	272.5657	304.4223
1389349_s_at	362417	Il17re	interleukin 17 receptor E	264.3768	69.3165	58.67794
1369835_at	24612	Omp	olfactory marker protein	264.3064	165.7673	248.8516
1369864_a_at	25044	Sds	serine dehydratase	264.0269	72.00103	92.56625
1394937_at	299762	Tmtc2_pre	transmembrane and tetratricopeptide r	263.9249	85.13826	332.4683
1387647_at	117270	Tff1	trefoil factor 1	263.9203	28.53261	298.434
1382181_at	311984	LOC31198	similar to RIKEN cDNA A530088I07 ge	263.8363	247.4613	441.3266
1372013_at	293618	Ifitm1_prec	interferon induced transmembrane pro	263.6979	154.7485	958.7928
1386090_at	499437	RGD15605	similar to minichromosome maintenanc	263.6388	318.4272	325.6005
1387163_at	25723	Hcrt	hypocretin	263.552	162.3674	153.0234
1387487_a_at	116506	Calcr	calcitonin receptor	263.53	22.42596	223.0283
1369440_at	155192	Abcg8	ATP-binding cassette, sub-family G (W	263.3625	89.53439	13.62308
1372264_at	362282	Pck1	phosphoenolpyruvate carboxykinase 1	263.2431	186.6576	144.2991
1393648_at	311718	Btbd4_prec	BTB (POZ) domain containing 4 (predi	263.1845	272.0015	54.30723
1378136_at	89787	Lrp3	low density lipoprotein receptor-relatec	263.0274	607.0759	1205.263
1370602_at	29600	Atp2b4	ATPase, Ca++ transporting, plasma m	262.7882	294.7437	172.5036
1381997_at	246253	Adipoq	adiponectin, C1Q and collagen domair	262.7205	175.4921	163.2471
1391824_at	294368	RGD15647	similar to RIKEN cDNA 0610012D17 (p	262.636	92.6681	260.0861
1380591_at	298076	NA	NA	262.5811	85.64395	430.23
1380105_at	312226	RGD15653	similar to Solute carrier family 23, mem	262.2477	172.1679	243.6217
1394022_at	291023	Id4	inhibitor of DNA binding 4	262.1597	7540.078	1316.177
1392754_at	499285	RGD15663	similar to cysteine-rich glycoprotein (pr	261.9162	98.40135	156.8332
1368861_a_at	29409	Mag	myelin-associated glycoprotein	261.8284	182.247	230.9458
1376061_at	497895	RGD15640	similar to RIKEN cDNA 3010026O09 (	261.7963	363.9396	300.6608
1392376_at	499527	NA	NA	261.7792	51.95814	95.82012
1391378_at	316737	Lpin2_prec	lipin 2 (predicted)	261.6789	38.05953	810.0472
1369832_at	57021	Adam3	a disintegrin and metalloprotease dom	261.6019	213.4474	218.5709
1371048_at	192274	Foxe1	forkhead box E1 (thyroid transcription	261.2388	157.5276	127.7942
1396431_at	500718	NA	NA	261.2222	160.0516	161.8831
1380442_at	368178	Hoxc8_ma	homeo box C8 (mapped)	261.04	225.5536	172.2451
1386521_at	298024	Akap2	A kinase (PRKA) anchor protein 2	260.9566	212.6581	245.8638

1377340_at	286926	Tfpi2	tissue factor pathway inhibitor 2	260.8763	62.21452	4481.16
1378233_at	362606	Sync_pred	syncoilin (predicted)	260.7967	1614.099	42.30248
1379505_at	292381	Lix1_predic	limb expression 1 homolog (chicken) (p	260.7447	126.3478	126.7134
1375757_at	114483	Cdk6	cyclin-dependent kinase 6	260.7083	36.98855	244.9293
1387492_at	24546	Slco2a1	solute carrier organic anion transporter	260.6435	105.0741	37.68584
1384972_at	688336	LOC68833	NA	260.5554	378.8434	36.99451
1368745_at	29500	Slc10a2	solute carrier family 10, member 2	260.5044	179.323	163.1243
1384054_at	309133	Mrgprg	MAS-related G-protein coupled recepto	260.4297	46.9141	382.6306
1370713_at	252879	Cdc2l1	cell division cycle 2 homolog (S.pombe	259.9298	323.1006	334.9929
1393588_at	304073	Cldn14	claudin 14	259.9107	166.4963	20.35847
1386749_at	408243	Ece2	endothelin-converting enzyme 2	259.7251	204.2923	564.6836
1375789_at	56813	Pthr1	parathyroid hormone receptor 1	259.7094	314.2535	278.744
1387178_a_at	24250	Cbs	cystathionine beta synthase	259.602	145.9885	109.1513
1370439_a_at	246153	Kcnc2	potassium voltage gated channel, Sha	259.5283	382.5475	791.5559
1371086_at	499168	NA	NA	259.4035	103.6954	48.09279
1369587_at	59325	Ereg	epiregulin	259.045	43.09142	235.7023
1390651_at	366360	RGD13098	similar to KIAA1161 protein (predicted)	258.9168	312.2312	96.7029
1380769_at	500083	NA	NA	258.8069	64.53655	313.707
1370874_at	24283	Csh2	chorionic somatomammotropin hormon	258.7517	41.07188	72.56629
1369305_at	171452	Rab3il1	RAB3A interacting protein (rabin3)-like	258.7324	27.77981	162.9793
1396182_at	313130	Acy1l2_pre	aminoacylase 1-like 2 (predicted)	258.5655	108.7045	172.3303
1370832_at	116637	Ccl4	chemokine (C-C motif) ligand 4	258.3218	77.88228	995.1762
1394956_at	361327	RGD15645	similar to mesenchymal stem cell prote	258.2711	14.52132	117.1772
1380372_at	311551	Pofut1	protein O-fucosyltransferase 1	258.1684	362.2051	164.654
1381410_a_at	362402	Fgd5_pred	FYVE, RhoGEF and PH domain conta	258.1492	231.8378	189.6258
1379005_at	499798	LOC49979	similar to ADP-ribosylation-like factor 6	258.0486	167.0104	323.0384
1390585_at	64023	Masp1	mannan-binding lectin serine peptidase	257.876	180.5941	390.0105
1367988_at	83790	Cyp2c23	cytochrome P450, family 2, subfamily c	257.5457	159.2019	196.8503
1368696_at	63848	Fxyd7	FXYD domain-containing ion transport	257.5037	22.78382	63.02379
1369099_at	58976	Slc30a1	solute carrier family 30 (zinc transporte	257.479	494.286	432.7957
1379655_at	361480	NA	NA	257.387	35.48925	316.3455
1393968_at	497956	NA	NA	257.3589	26.66911	82.69151
1382083_at	362735	Coch_pred	coagulation factor C homolog (Limulus	257.2574	67.86739	411.5556
1382834_at	498494	NA	NA	257.0541	184.0032	321.06
1392364_at	361284	NA	NA	257.0304	226.7591	586.3606
1368742_at	113959	C5r1	complement component 5, receptor 1	256.7827	103.3088	245.1103
1377521_at	361126	Sfrs14_pre	splicing factor, arginine/serine-rich 14 (	256.5759	154.0882	144.4609
1383245_at	362740	Mbip_predi	MAP3K12 binding inhibitory protein 1 (	256.4395	449.4624	858.0581
1387159_at	81722	Ager	advanced glycosylation end product-sp	256.3823	318.7652	377.2966
1368780_at	25645	Adrb3	adrenergic receptor, beta 3	256.2301	213.1083	254.5629
1385000_at	497957	RGD15656	RGD1565611 (predicted)	256.1312	295.5159	257.6131
1388496_at	362332	Flnc_predic	filamin C, gamma (actin binding protein	256.0641	74.4384	169.5518
1369687_at	58981	Kcnab3	potassium voltage-gated channel, shal	256.0365	276.9481	65.11963
1394928_at	297801	XRG4	XK-related protein 4	255.9664	154.7342	450.8168
1382729_at	499783	RGD15640	similar to GTPase activating RANGAP	255.9032	1003.257	568.4532
1388295_s_at	316213	Frs3	fibroblast growth factor receptor substr	255.8769	181.9663	462.3139
1388961_at	317258	Itgb1bp2_p	integrin beta 1 binding protein 2 (predic	255.8754	154.4962	82.42166
1388945_at	361238	RGD13113	similar to 1300014I06Rik protein	255.7696	94.3791	372.8071
1369005_at	29682	Kcnq3	potassium voltage-gated channel, subf	255.7064	194.1491	343.6933
1380274_at	297402	RGD15607	similar to Sepiapterin reductase (SPR)	255.6424	222.678	386.1232
1394673_at	499142	NA	NA	255.4625	105.1831	148.61

1371076_at	361523	Cyp2b2	Cytochrome P450, family 2, subfamily	255.3715	57.46592	161.0275
1385080_s_at	360619	LOC36061	NA	255.3605	483.5934	105.9719
1376993_at	315430	Amotl1_pre	angiomotin-like 1 (predicted)	255.2658	149.8753	10.80458
1374391_at	367086	Sln	sarcolipin	255.0454	164.785	100.6262
1387808_at	83509	Slc7a7	solute carrier family 7 (cationic amino a	254.9852	145.8438	394.5122
1379960_at	306810	Susd3_pre	sushi domain containing 3 (predicted)	254.8799	130.9384	154.3268
1389017_at	305633	LOC30563	similar to Antxr2 protein	254.7544	748.6638	307.6011
1384273_at	287479	Carkl	carbohydrate kinase-like	254.6958	2185.858	148.8315
1368574_at	24173	Adra1b	adrenergic receptor, alpha 1b	254.6084	209.0306	129.1995
1387065_at	140693	Plcd4	phospholipase C, delta 4	254.5923	13.35438	14.17448
1387044_at	171158	Gpha2	glycoprotein hormone alpha 2	254.3767	90.0714	15.40431
1370898_at	29140	Snn	stannin	254.282	1606.727	2978.674
1387606_at	54250	Fgf2	fibroblast growth factor 2	254.2716	154.1039	289.7323
1370718_at	60567	Syt10	synaptotagmin X	254.1143	580.576	79.29928
1383600_at	266998	Slc13a5	solute carrier family 13 (sodium-depen	254.1126	43.2522	22.1989
1368926_at	29745	Sema4f	sema domain, immunoglobulin domair	254.0465	615.2284	488.393
1378409_at	114503	Pou4f1	POU domain, class 4, transcription fac	254.0129	132.7763	36.97466
1390411_at	298487	Cldn19	claudin 19	253.8084	178.4688	245.9642
1387889_at	171049	Folr1	folate receptor 1 (adult)	253.7406	159.2023	243.9192
1378947_at	303517	Tns4	tensin 4	253.5703	130.846	221.0592
1391946_at	25651	Selp	selectin, platelet	253.5465	100.0662	30.84433
1372168_s_at	25641	Igfbp6	insulin-like growth factor binding protei	253.5265	163.6249	485.1734
1377156_at	365486	NA	NA	253.471	680.3814	928.0782
1370021_at	24717	Rho	rhodopsin	253.4645	173.5909	135.4385
1390180_at	305198	Prdm8_pre	PR domain containing 8 (predicted)	253.4428	255.1986	318.8137
1379285_at	360733	Rtp4_predi	receptor transporter protein 4 (predicte	253.4372	49.18079	378.0152
1387188_at	171080	Slc17a1	solute carrier family 17 (sodium phosp	253.4013	93.58836	116.2468
1387995_a_at	361673	Ifitm3	NA	253.3712	46.02683	1698.5
1380218_at	501054	NA	NA	252.8382	72.54899	34.83706
1390584_at	307251	RGD15607	similar to ring finger protein 111 (predi	252.7087	1687.334	679.0196
1387053_at	25256	Fmo1	flavin containing monooxygenase 1	252.6698	210.0489	352.1801
1368689_at	29586	Gjb5	gap junction membrane channel protei	252.5853	106.8406	205.7422
1397937_at	315591	Lrrc35	leucine rich repeat containing 35	252.4139	190.3067	474.8131
1371840_at	29733	Edg1	endothelial differentiation sphingolipid	252.3968	10.42545	257.1885
1389898_at	362275	B4galt5_pr	UDP-Gal:betaGlcNAc beta 1,4-galacto	252.3958	127.99	154.0191
1387566_at	24653	Pla2g4a	phospholipase A2, group IVA (cytosoli	252.3308	26.15844	206.8742
1393958_at	25108	Avpr2	arginine vasopressin receptor 2	252.2152	85.28787	74.25262
1378712_at	313785	Icam5_pre	intercellular adhesion molecule 5, teler	252.1964	354.1408	73.25471
1383420_at	502479	LOC50247	similar to lactation elevated 1	252.0584	424.8584	395.7304
1388602_at	54249	Cfd	complement factor D (adipsin)	251.9233	22.10914	27.86052
1388598_at	691996	LOC69199	NA	251.872	125.4823	86.50156
1382202_at	687661	LOC68766	NA	251.8679	790.0153	385.5355
1369544_a_at	25607	Hoxa1	homeo box A1	251.6824	177.1366	28.43814
1368531_at	24658	Prlpc1	prolactin-like protein C 1	251.5125	32.68505	83.77297
1371045_at	79123	Accn2	amiloride-sensitive cation channel 2, n	251.3995	685.4504	809.5518
1393301_at	307992	RGD15634	similar to mKIAA0534 protein (predicte	251.2618	212.973	191.4429
1393187_at	689152	LOC68915	NA	251.2597	131.2418	145.8743
1380223_at	287127	Cramp1l_p	Crm, cramped-like (Drosophila) (predic	251.1369	105.8186	64.86112
1382111_at	302554	RGD15645	similar to RIKEN cDNA 2010001H14 (|	251.0738	38.29125	216.1053
1391201_at	305827	Wdhd1_pre	WD repeat and HMG-box DNA binding	250.9027	1259.851	693.9192
1380462_at	500612	RGD15621	RGD1562146 (predicted)	250.8294	128.6402	72.35466

1370268_at	25470	Kcna5	potassium voltage-gated channel, shal	250.7992	428.6033	1799.032
1377427_at	305064	Ptpn14_pre	protein tyrosine phosphatase, non-rece	250.6448	68.133	417.2884
1370491_a_at	24443	Hdc	histidine decarboxylase	250.45	51.56491	543.696
1380270_at	288038	Mfi2_predic	antigen p97 (melanoma associated) id	250.4187	383.245	39.28922
1368317_at	29171	Aqp7	aquaporin 7	250.415	75.98385	197.9649
1385136_at	311803	RGD13083	similar to hypothetical protein MGC375	250.3823	202.0505	172.106
1371303_at	290315	Tdh_predic	L-threonine dehydrogenase (predicted	250.3074	31.02992	61.71209
1383472_at	298079	Aldh1b1	aldehyde dehydrogenase 1 family, me	250.2124	35.44439	363.5436
1374142_at	312358	RGD13115	similar to RIKEN cDNA E130201N16 (	249.9778	71.29781	386.5399
1370355_at	246074	Scd1	stearoyl-Coenzyme A desaturase 1	249.9508	62.69128	54.10317
1369829_at	117055	Gjb4	gap junction membrane channel protei	249.9456	33.14084	90.15941
1385640_at	298296	Pcsk9	proprotein convertase subtilisin/kexin t	249.9403	213.1825	204.2516
1390319_at	308173	Kif13a_pre	kinesin family member 13A (predicted)	249.8196	168.7957	334.1621
1369325_at	85419	Lyst	lysosomal trafficking regulator	249.4454	322.6553	78.69481
1368548_at	25065	Slc12a1	solute carrier family 12, member 1	249.4004	126.7439	170.7058
1370225_at	114491	Cited4	Cbp/p300-interacting transactivator, wi	249.2139	470.9094	171.4246
1368294_at	116687	Dnase1l3	deoxyribonuclease I-like 3	249.1457	111.6823	200.6858
1378658_at	362053	Clca6	chloride channel calcium activated 6	248.9637	53.15702	276.0384
1384502_at	362646	Fbxo42_pr	F-box protein 42 (predicted)	248.9599	79.15844	114.2414
1382548_at	303499	Sp2_mapp	Sp2 transcription factor (mapped)	248.8214	225.8793	237.6417
1377025_at	84392	Kif3a	kinesin family member 3a	248.6164	936.1488	510.8916
1377099_at	303817	RGD15605	similar to RIKEN cDNA 2310042E22 (|	248.453	105.0334	72.17563
1393240_at	293677	Efemp2	EGF-containing fibulin-like extracellula	247.8532	117.4113	257.0168
1397205_at	299131	Dhrs7	dehydrogenase/reductase (SDR family	247.6958	9.243853	17.83743
1394571_at	300074	RGD13059	hypothetical LOC300074	247.6493	99.13151	915.4128
1395988_at	299211	Zdhhc22	zinc finger, DHHC-type containing 22	247.6386	213.6834	170.3891
1385563_at	308868	RGD15615	similar to Catechol O-methyltransferas	247.3827	617.3777	365.053
1378285_at	294251	Ncr3	natural cytotoxicity triggering receptor :	246.9773	137.2461	83.24914
1368746_a_at	171028	Atp12a	ATPase, H+/K+ transporting, nongastr	246.9721	147.4697	62.04497
1384710_at	292813	RGD15627	similar to RIKEN cDNA 2410004F06 (|	246.6611	85.66818	47.78696
1370683_at	156873	Kcnk15	potassium channel, subfamily K, meml	246.3886	104.8841	141.7118
1367700_at	64507	Fmod	fibromodulin	246.3174	319.1275	175.7851
1391227_at	294329	Trpm2	transient receptor potential cation char	246.284	965.4681	210.9376
1387238_at	116648	Phox2a	paired-like homeobox 2a	246.1714	157.9549	214.9824
1387845_at	171011	Kcng3	potassium voltage-gated channel, subf	246.164	74.52712	154.0627
1381485_at	306685	NA	NA	246.0195	17.51291	104.3056
1394258_at	503264	NA	NA	245.9347	118.0661	41.26275
1369453_at	117277	Epn1	Epsin 1	245.7635	426.5686	20.45193
1390575_at	501803	RGD15625	similar to beta 3-glycosyltransferase-lik	245.6111	36.57758	82.05338
1396612_at	362036	Ank2	ankyrin 2, neuronal	245.5116	180.7684	534.3122
1382291_at	363698	NA	NA	245.4629	1719.018	1775.477
1372752_at	293627	Tspan4	tetraspanin 4	245.3652	2924.437	66.83181
1393804_at	362472	RGD1307C	similar to hypothetical protein FLJ2249	245.2398	260.04	513.7443
1397346_at	366504	NA	NA	245.2221	209.0782	289.6785
1368988_at	29209	Casq2	calsequestrin 2	245.2006	149.4149	215.4877
1389280_at	309900	Tulp1_prec	tubby like protein 1 (predicted)	245.1949	75.38854	143.9387
1393802_x_at	312793	Gsg1	germ cell associated 1	245.1768	333.0678	186.0221
1377943_at	56822	Cd86	cd86 antigen	245.0816	36.69655	133.3082
1370206_at	63882	Accn4	amiloride-sensitive cation channel 4, p	244.9697	38.28295	194.1743
1369107_at	24773	Sftpa1	surfactant, pulmonary-associated prote	244.8826	7.892991	134.2214
1382924_at	294088	Pank1_pre	pantothenate kinase 1 (predicted)	244.8453	315.9291	324.4338

1398657_at	502834	RGD15601	similar to immunoglobulin kappa-chain	244.7307	66.37722	236.494
1393260_at	360228	LOC36022	WDNM1 homolog	244.3436	142.6093	216.8549
1369770_at	25033	Sstr1	somatostatin receptor 1	244.2935	813.1717	595.1916
1373577_at	246331	Nrp1	neuropilin 1	244.2204	664.4373	3633.433
1370735_at	286913	Olr1278	olfactory receptor 1278	244.1429	22.77766	147.7396
1372404_at	366957	Rac2	RAS-related C3 botulinum substrate 2	244.1171	127.839	59.45197
1394251_x_at	307721	Irx3_predic	Iroquois related homeobox 3 (Drosoph	244.0976	55.09611	33.70844
1372886_at	360962	Tacc3	transforming, acidic coiled-coil contain	243.8975	4916.29	225.6679
1369223_at	113911	Gucy2d	guanylate cyclase 2d	243.8744	75.18486	132.0869
1381935_at	500364	RGD15597	similar to Sspn protein (predicted)	243.7107	195.3314	95.33776
1393637_at	301525	Glb1l_pred	galactosidase, beta 1-like (predicted)	243.5667	124.4085	24.65577
1368490_at	60350	Cd14	CD14 antigen	243.563	79.05311	507.5266
1387303_at	29503	Slc22a2	solute carrier family 22 (organic cation	243.4408	26.44512	57.50539
1368934_at	24307	Cyp4b1	cytochrome P450, family 4, subfamily I	243.3468	130.5975	31.95466
1393329_at	64545	Icos	inducible T-cell co-stimulator	243.3319	181.4359	214.0191
1368660_at	59326	Rapgef3	Rap guanine nucleotide exchange fact	243.1993	79.09977	131.4849
1369204_at	25734	Hck	hemopoietic cell kinase	243.0614	281.5931	134.9161
1380377_at	367901	Btk	Bruton agammaglobulinemia tyrosine I	243.0542	53.34663	72.52908
1387230_at	54300	Slc12a3	solute carrier family 12, member 3	242.908	212.7116	363.8185
1377369_at	295669	Cybrd1	cytochrome b reductase 1	242.6605	247.1738	199.0361
1370475_at	286953	Cyp2b3	cytochrome P450IIB3	242.575	42.92951	45.17418
1373957_at	24718	Reln	reelin	242.5306	70.70024	289.7767
1390219_at	305349	Wdr19_pre	WD repeat domain 19 (predicted)	242.4867	1278.675	953.556
1396716_at	367242	Rab23_pre	RAB23, member RAS oncogene family	242.3953	210.6332	100.4089
1387136_at	64576	Ptprv	protein tyrosine phosphatase, receptor	242.1572	359.264	294.0721
1379056_at	361437	RGD1310C	similar to RIKEN cDNA 2810427I04; D	242.0955	385.2274	562.4129
1388004_at	252939	Gpr37l1	G protein-coupled receptor 37-like 1	242.0743	248.383	155.6292
1395384_at	308959	Usp31_pre	ubiquitin specific protease 31 (predicte	241.9485	165.6123	84.03367
1368090_at	78960	Prx	periaxin	241.9132	153.6403	313.0179
1384885_at	298532	Tekt2	tektin 2	241.9094	269.0084	1382.789
1391055_at	296199	Snx5_pred	sorting nexin 5 (predicted)	241.8291	245.2617	90.07747
1369210_at	81574	Scn1a	sodium channel, voltage-gated, type I,	241.6234	144.5737	517.637
1389221_at	304301	Mmd2_pre	monocyte to macrophage differentiatio	241.5531	220.3185	452.8203
1394122_at	305341	Rhoh	ras homolog gene family, member H	241.4533	30.06829	295.0231
1396620_at	25236	Gpc3	glypican 3	241.2376	62.68439	143.3444
1376693_at	500011	RGD1563C	similar to OEF2 (predicted)	241.1717	954.6603	1373.132
1388811_at	29179	Syn2	synapsin II	241.1228	1595.631	48.92099
1369883_at	24694	Pth	parathyroid hormone	240.9266	48.65797	56.22195
1370483_at	64025	Cd244	CD244 natural killer cell receptor 2B4	240.8887	91.46061	222.4934
1398602_at	297176	Mad2l1_pr	MAD2 (mitotic arrest deficient, homolo	240.8597	5191.609	229.5435
1397648_at	314850	Frs2_predi	fibroblast growth factor receptor substr	240.6486	293.5288	48.08771
1385601_at	361156	Frg1_predi	FSHD region gene 1 (predicted)	240.5733	1021.78	157.5426
1370786_at	257643	LOC25764	cystatin SC	240.4367	109.2753	64.61386
1374320_at	304929	F5_mappe	coagulation factor 5 (mapped)	240.1965	401.4913	1468.782
1391132_at	29242	Ca4	carbonic anhydrase 4	239.9443	94.15811	127.3596
1376051_at	290277	Cryl1	crystallin, lamda 1	239.7618	242.098	448.5419
1374933_at	78967	Mcam	melanoma cell adhesion molecule	239.7272	29.68207	301.6519
1396665_at	362384	Reep1_pre	receptor accessory protein 1 (predicte	239.6987	30.3254	34.55732
1372407_at	295261	Tmod4_pre	tropomodulin 4 (predicted)	239.6794	138.7973	481.4915
1392221_at	498819	RGD15623	similar to GREB1 protein isoform a (pr	239.6424	279.6254	691.6159
1374073_at	303333	RGD13094	similar to DNA segment, Chr 11, ERAT	239.32	181.3246	44.21652

1370767_at	286962	Cox6c1	cytochrome c oxidase subunit VIc-1	239.2325	310.9966	377.5659
1388085_at	259233	Gpx6	glutathione peroxidase 6	239.152	33.27094	18.36802
1368638_at	64107	Npffr1	neuropeptide FF receptor 1	239.1506	69.53733	308.6436
1387489_at	56819	Extl3	exostoses (multiple)-like 3	239.0975	116.0017	88.10136
1390060_at	294715	RGD13093	hypothetical LOC294715	239.0497	95.15925	186.6976
1372585_at	499318	RGD15662	RGD1566254 (predicted)	239.0148	158.8921	1542.426
1370279_at	24273	Cryaa	crystallin, alpha A	238.8901	59.65261	297.9239
1396791_at	288093	Cdgap_pre	Cdc42 GTPase-activating protein (pre	238.8159	41.78652	177.4696
1369397_at	170634	Tas1r3	taste receptor, type 1, member 3	238.7551	183.7734	31.52505
1368942_at	79225	Hes5	hairy and enhancer of split 5 (Drosoph	238.6098	206.167	238.5757
1368442_at	29251	F2	coagulation factor II	238.3444	173.4158	142.8059
1379300_at	367145	Chst2_pre	carbohydrate sulfotransferase 2 (predi	238.1385	117.5162	176.2791
1376381_at	497083	LOC49708	XK-related protein 5	238.1151	971.9973	162.0788
1381168_at	308449	Hipk4	homeodomain interacting protein kinas	237.9295	136.5876	225.9021
1383457_at	498228	RGD15660	similar to hypothetical protein DKFZp7	237.9077	516.8943	234.5003
1389966_at	367313	Col6a3_pre	procollagen, type VI, alpha 3 (predicte	237.7811	108.6754	1274.201
1374451_at	64637	Hand2	heart and neural crest derivatives expr	237.6307	186.0515	276.1048
1378780_at	315663	Exph5_pre	exophilin 5 (predicted)	237.5178	24.78316	203.7823
1368624_at	24152	A	agouti	237.4091	89.24264	155.381
1373847_at	295061	Tm4sf1_pr	transmembrane 4 superfamily member	237.3951	88.58674	279.7388
1368518_at	24251	Cd53	CD53 antigen	237.0285	41.91002	277.3898
1385263_at	498834	RGD15611	similar to myosin-VIIb (predicted)	236.8641	243.4112	135.2462
1386807_at	114487	Wnt2	wingless-related MMTV integration site	236.7803	137.0521	213.2134
1369302_at	171104	Gpr30	G protein-coupled receptor 30	236.6262	87.91291	158.1828
1387620_a_at	64565	Tmprss11d	transmembrane protease, serine 11d	236.6021	89.43287	132.7079
1380579_at	297894	Matn1	matrilin 1, cartilage matrix protein	236.5553	150.7546	179.96
1381376_at	299339	LOC29933	NA	236.4417	79.71043	41.89623
1398453_at	498957	LOC49895	similar to cDNA sequence BC025816	236.2481	258.5362	429.5021
1380318_at	499196	NA	NA	236.1806	59.03201	173.6295
1378719_at	364891	NA	NA	236.1199	131.1983	243.5065
1387556_at	27256	Cntn6	contactin 6	235.9426	92.26974	136.5265
1393070_at	360612	Scrn2	secernin 2	235.8788	319.9326	187.9382
1379479_at	84393	Kif4	kinesin family member 4	235.8295	3124.253	84.48548
1391133_at	297520	Il17rc_pred	interleukin 17 receptor C (predicted)	235.7493	200.4286	132.9956
1373032_at	290553	Mustn1	musculoskeletal, embryonic nuclear pr	235.6878	417.7026	601.045
1387326_at	117037	Spam	sperm adhesion molecule	235.6625	36.41674	33.03259
1368773_at	25448	Fshprh1	FSH primary response 1	235.4802	1230.308	251.4541
1389119_at	316071	Cmya1_pre	cardiomyopathy associated 1 (predicte	234.9201	62.25348	62.80573
1384192_at	295934	Chst1	carbohydrate (keratan sulfate Gal-6) su	234.722	518.0699	544.1391
1373188_at	315611	Scn4b	sodium channel, voltage-gated, type IV	234.6925	135.3004	200.6196
1396949_at	298139	LOC29813	similar to RIKEN cDNA 2310003M01	234.6426	5.585329	57.989
1370205_at	84511	Slco1c1	solute carrier organic anion transporter	234.5293	51.19065	12.05505
1367940_at	84348	Cmkor1	chemokine orphan receptor 1	234.4288	192.241	853.6769
1390310_at	360647	Icam2	intercellular adhesion molecule 2	234.3538	186.676	47.98451
1377410_at	308607	E2f8	E2F transcription factor 8	234.2724	1303.982	271.488
1392082_a_at	303747	Cd7_predic	CD7 antigen (predicted)	234.1983	210.8861	116.9217
1387703_a_at	115771	Usp2	ubiquitin specific peptidase 2	234.1803	214.3966	721.8443
1370422_at	246240	Ripk3	receptor-interacting serine-threonine k	234.1616	68.93195	171.3599
1375148_at	691504	LOC69150	NA	234.16	142.8302	78.59438
1398269_at	114523	Ntn1	netrin 1	234.1293	273.1568	177.9732
1395055_at	309724	Tmem26_p	transmembrane protein 26 (predicted)	234.1225	31.32984	80.69522

1370453_at	207113	Tex101	testis expressed gene 101	234.0532	176.9354	173.9109
1381004_at	292711	RGD15643	similar to BC049730 protein (predicted	233.9604	44.12924	251.2086
1394433_at	288449	Katnal1	katanin p60 subunit A-like 1	233.8712	435.6884	77.53129
1386911_at	24212	Atp1a2	ATPase, Na+/K+ transporting, alpha 2	233.7622	563.683	413.7598
1397855_at	367181	RGD15651	similar to cysteine sulfinic acid decarbo	233.5336	7.100641	777.962
1388082_at	60587	Dusp4	dual specificity phosphatase 4	233.464	66.95245	74.72832
1387999_at	25693	Slc18a1	solute carrier family 18 (vesicular mon	233.4291	53.84789	154.4622
1392261_at	312401	RGD13110	similar to RIKEN cDNA A930038C07 (	233.3624	71.1737	193.4784
1384530_at	690217	LOC69021	NA	233.3166	336.0862	158.4875
1377732_at	361658	RGD13114	similar to hypothetical protein FLJ2535	233.2855	34.39214	140.2586
1398556_at	305454	Zfyve28_pr	zinc finger, FYVE domain containing 2	233.1332	44.69955	103.5093
1388911_at	301323	Prim2	DNA primase, p58 subunit	233.1275	1623.635	302.2909
1370094_at	60353	Acrv1	acrosomal vesicle protein 1	233.0449	37.38967	19.45506
1391765_at	287371	Lrrc48	leucine rich repeat containing 48	232.7049	90.07503	575.6738
1379253_at	303918	Gtf2e1	general transcription factor II E, polype	232.6838	185.6957	375.5652
1369943_at	56083	Tgm2	transglutaminase 2, C polypeptide	232.6611	1551.116	2037.708
1378256_at	311338	Rpap1	NA	232.5944	418.1121	359.5042
1370581_at	63851	Fgf14	fibroblast growth factor 14	232.5914	229.808	491.9263
1369822_at	64030	Kit	v-kit Hardy-Zuckerman 4 feline sarcom	232.5017	1824.449	126.0163
1387006_at	24902	Smp2a	rat senescence marker protein 2A gen	232.4153	28.84554	90.90618
1389783_s_at	292746	RGD13119	similar to Fc fragment of IgG binding p	232.3014	49.30812	739.913
1373122_at	85265	Jub	ajuba homolog (Xenopus laevis)	232.2913	120.4504	130.4314
1376987_at	300763	Lrrc49_pre	leucine rich repeat containing 49 (pred	232.2901	586.7515	397.8905
1387069_a_at	24816	Tbxa2r	thromboxane A2 receptor	232.2178	271.504	101.9238
1397271_at	500910	RGD15608	similar to tumor necrosis factor recepto	231.8748	13.7872	42.05426
1369673_at	113995	P2rx5	purinergic receptor P2X, ligand-gated i	231.8526	45.83596	56.11481
1393750_at	299002	RGD13102	similar to KIAA1333 protein (predicted)	231.7164	463.2548	126.6481
1383186_at	299338	RGD13077	similar to RIKEN cDNA 1600013K19 (p	231.7022	299.8923	251.7578
1387751_at	114596	Wap	whey acidic protein	231.6133	73.62065	25.63229
1387707_at	25551	Slc2a3	solute carrier family 2 (facilitated gluco	231.3558	54.74151	45.19358
1385405_at	301460	Adam23_p	a disintegrin and metallopeptidase don	231.2749	94.70771	127.8967
1377035_at	317161	Mospd4_pr	motile sperm domain containing 4 (pre	231.2707	48.30731	46.52842
1379747_at	315866	Prss35	protease, serine, 35	231.1834	33.09815	183.2875
1396169_at	303589	RGD13054	similar to 4921510J17Rik protein	231.1779	101.1696	205.9213
1384094_at	363076	RGD15640	similar to timeless-interacting protein (p	230.9838	95.71377	56.85936
1397158_at	259269	Cmbp	cytomatrix protein p110	230.9441	136.8647	216.0204
1398261_at	29635	Timm44	translocator of inner mitochondrial mer	230.6102	53.96667	90.01535
1375106_at	314459	Amn_predi	amnionless (predicted)	230.544	262.51	61.68299
1375776_at	295062	MGC11609	transcriptional co-activator with PDZ-b	230.4894	22.23401	366.9123
1369513_at	114492	Ccl28	chemokine (C-C motif) ligand 28	230.4343	43.88312	248.218
1373419_at	171357	Ptprg	protein tyrosine phosphatase, receptor	230.3588	20.63318	242.6489
1373990_at	294881	Slc7a12	solute carrier family 7 (cationic amino a	230.351	156.2624	169.6781
1378953_at	312310	RGD15597	similar to Zinc finger and SCAN domai	230.1262	173.8095	108.3619
1383848_at	24925	Adrb1	adrenergic receptor, beta 1	230.0525	54.13962	384.7392
1369468_at	64558	Fzd4	frizzled homolog 4 (Drosophila)	230.0217	142.0183	243.3216
1378140_at	364396	Arl11	ADP-ribosylation factor-like 11	229.964	36.44288	176.8378
1385245_at	305103	LOC30510	cell surface glycoprotein gp42	229.8879	124.4142	82.76088
1378443_at	289235	Slamf9_pre	SLAM family member 9 (predicted)	229.8538	142.3781	126.9883
1396259_at	307767	RGD15627	similar to Fras1 related extracellular m	229.8284	114.6684	203.7477
1369752_a_at	25050	Camk4	calcium/calmodulin-dependent protein	229.7541	1276.649	127.8558
1392086_at	362918	Mtss1_pre	metastasis suppressor 1 (predicted)	229.7161	213.3429	112.5099

1383422_at	362564	LOC36256	NA	229.6933	545.1791	1056.457
1379744_at	365245	Saa4	serum amyloid A 4	229.6331	83.79421	182.2246
1394748_at	313592	Col8a2_pre	procollagen, type VIII, alpha 2 (predicte	229.6046	118.4374	60.41974
1392926_at	316758	Lama1_pre	laminin, alpha 1 (predicted)	229.528	86.92664	55.11388
1367937_at	252899	Miox	myo-inositol oxygenase	229.4033	118.4159	89.69231
1368958_at	29704	Pacsin1	protein kinase C and casein kinase sul	229.394	412.7377	98.05871
1389568_at	294019	RGD13082	similar to RIKEN cDNA 2810048G17 g	229.3583	67.63981	222.4182
1394508_at	311604	Zhx3	zinc fingers and homeoboxes 3	229.3425	102.7638	288.9354
1368737_at	64675	Smr2	SMR2	229.3093	122.644	108.6719
1396810_at	500531	RGD15637	RGD1563714 (predicted)	229.2958	153.3238	302.0519
1384757_at	307657	RGD13088	similar to mKIAA1612 protein (predicte	229.1662	151.1752	180.0193
1369490_at	60432	Glp2r	glucagon-like peptide 2 receptor	229.0533	131.971	14.14932
1393462_at	306278	RGD13117	similar to RIKEN cDNA 4930442L21	228.8778	130.915	72.33674
1377972_at	310045	NA	NA	228.773	133.9206	136.7006
1393872_at	360945	RGD15628	similar to RIKEN cDNA 2310045A20 (p	228.6809	145.9987	140.8808
1389811_at	360519	Rasgef1c_	RasGEF domain family, member 1C (p	228.5258	1054.181	120.1233
1376661_at	313672	LOC31367	similar to CG11206-PA	228.388	67.5983	868.757
1395116_at	24930	Cd8a	CD8 antigen, alpha chain	228.3341	126.1484	136.8292
1370628_at	171528	Gzmb	granzyme B	228.3085	117.3893	145.2179
1398478_at	314320	Mlh3_predi	mutL homolog 3 (E. coli) (predicted)	228.204	243.4554	106.4731
1368452_at	81642	Abcc6	ATP-binding cassette, sub-family C (C	228.1081	262.164	245.0523
1380258_at	498488	NA	NA	227.9814	66.21275	340.8813
1393542_at	500626	NA	NA	227.9551	188.9704	116.8034
1375624_at	300177	Irak4_pred	interleukin-1 receptor-associated kinas	227.7882	15.83543	110.3717
1371354_at	290561	Tnnc1	troponin C type 1 (slow)	227.5736	269.5154	73.23913
1387820_at	24523	Klk7	NA	227.5184	118.5462	126.8669
1377326_at	315308	RGD13089	similar to TGF-beta induced apoptosis	227.4774	18.78913	186.4475
1370555_at	266760	Vgcnl1	voltage gated channel like 1	227.4756	161.5367	9261.149
1390948_at	365390	Phemx	pan hematopoietic expression	227.3073	18.85947	78.38064
1397018_at	54286	Prkg1_map	protein kinase, cGMP-dependent, type	227.3003	95.65557	272.8224
1385115_at	309775	RGD13077	similar to RIKEN cDNA 5730521E12 (p	227.2425	58.53021	64.28276
1374013_at	315598	C1qtnf5	C1q and tumor necrosis factor related	227.2294	163.7076	229.3277
1384055_at	500570	NA	NA	227.2169	38.50826	79.04197
1390253_at	314457	Ankrd9	ankyrin repeat domain 9	227.2135	144.4411	144.4673
1370417_at	25194	Gnrh1	gonadotropin-releasing hormone 1	226.8587	52.6484	53.94659
1385769_at	365387	Odf3_predi	outer dense fiber of sperm tails 3 (prec	226.8354	66.84244	67.09441
1370124_at	117038	Mt3	metallothionein 3	226.6974	9242.756	108.4842
1371984_at	498587	RGD15648	similar to mKIAA0613 protein (predicte	226.5778	210.888	467.033
1369532_at	192649	Prokr2	prokineticin receptor 2	226.5105	62.7974	86.10049
1370219_at	79129	Cyba	cytochrome b-245, alpha polypeptide	226.4911	228.5102	286.8037
1381523_at	303304	Rutbc1_pre	RUN and TBC1 domain containing 1 (p	226.3907	72.62641	170.9014
1367703_at	24278	Crygd	crystallin, gamma D	226.3834	38.47841	31.43316
1376084_a_at	315330	Espl1_prec	extra spindle poles like 1 (S. cerevisiae	226.263	2498.05	59.62423
1387506_at	25100	Foxa3	forkhead box A3	226.1873	198.1225	70.586
1368734_at	54240	Chrnd	cholinergic receptor, nicotinic, delta po	226.1077	84.9708	254.4781
1387306_a_at	114090	Egr2	early growth response 2	226.0635	188.3865	1358.781
1377510_at	500911	RGD15633	similar to serine hydolase like protein,	225.8719	229.6303	64.56206
1391214_at	294430	RGD13048	similar to chromosome 6 open reading	225.8319	110.7069	160.8449
1370145_at	78972	Zfp354c	zinc finger protein 354C	225.4901	549.7769	205.5375
1398659_at	501231	LOC50123	NA	225.2893	60.80139	7036.128
1381054_at	311529	RGD13061	similar to KIAA0980 protein (predicted)	225.2868	363.627	247.8816

1376106_at	362691	MGC94782	similar to hypothetical protein MGC339	225.0621	2038.997	691.6913
1370133_at	59293	Rgs19	regulator of G-protein signaling 19	224.961	729.4711	68.18453
1376243_at	304692	Serpinb12_	serine (or cysteine) peptidase inhibitor	224.8464	73.1076	131.8749
1368412_a_at	50677	Ptpro	protein tyrosine phosphatase, receptor	224.7725	143.6964	59.88858
1394640_at	311033	Fmnl2_pre	formin-like 2 (predicted)	224.7639	29.22476	16.77538
1394666_at	311029	Neb_predic	nebulin (predicted)	224.754	49.47172	196.3143
1368693_at	79113	Fgr	Gardner-Rasheed feline sarcoma viral	224.7067	160.3966	163.7089
1368263_a_at	25037	Mobp	myelin-associated oligodendrocytic ba	224.6261	27.06314	55.66507
1392939_at	641603	Slc41a3	NA	224.5733	1302.621	535.1165
1368804_at	60584	Lif	leukemia inhibitory factor	224.5305	52.48633	34.34165
1369805_at	59101	Sc65	synaptonemal complex protein SC65	224.4867	488.0672	128.5331
1375889_at	363469	Sms	spermine synthase	224.4676	1801.382	1216.624
1378324_at	499814	LOC49981	NA	224.395	400.7714	3122.011
1391948_at	314423	Bcl11b_pre	B-cell leukemia/lymphoma 11B (predic	223.9585	22.97064	89.37051
1370923_at	58964	Nme6	expressed in non-metastatic cells 6, pr	223.9255	269.686	230.7825
1394385_s_at	25432	Drd4	dopamine receptor D4	223.8768	104.6705	210.6223
1393729_at	307757	Neto2_pre	neuropilin (NRP) and tolloid (TLL)-like	223.8764	896.1087	386.1782
1385235_at	302920	Boll_predic	bol, boule-like (Drosophila) (predicted)	223.8071	398.3518	94.38931
1370423_at	89788	Gna15	guanine nucleotide binding protein, alp	223.7634	54.37059	119.6645
1385144_at	301155	St6gal2	beta galactoside alpha 2,6 sialyltransfe	223.7126	63.91822	183.988
1368167_at	25424	Ctse	cathepsin E	223.6424	122.2586	107.1646
1385372_at	297418	Gkn1	gastrokine 1	223.5635	137.0125	150.6249
1396325_at	308561	RGD13095	similar to RIKEN cDNA 4933405K07	223.4925	60.26401	324.3785
1389779_at	306376	Sh2d4a	SH2 domain containing 4A	223.3507	97.5834	277.6601
1390591_at	266730	Slc17a3	solute carrier family 17 (sodium phosp	223.2849	39.67485	249.9214
1385391_at	306872	Ctag3	cancer/testis antigen 3	223.2291	141.093	340.3225
1387679_at	58978	Slco1b2	solute carrier organic anion transporter	223.1144	191.4972	33.11414
1367914_at	81505	Emp3	epithelial membrane protein 3	223.084	268.9246	294.3368
1388282_s_at	297666	Klra5	killer cell lectin-like receptor, subfamily	223.0312	54.01391	207.2585
1385383_at	291847	RGD15657	similar to hypothetical protein 4933409	223.0223	97.61553	1437.301
1371095_at	171291	Kif6	kinesin family member 6	223	68.87533	174.469
1386948_at	25491	Nes	nestin	222.9561	174.1793	69.62777
1368754_at	117264	P2ry6	pyrimidinergic receptor P2Y, G-protein	222.8898	279.8103	174.4101
1374596_at	309681	RGD13095	similar to RIKEN cDNA 1810043G02; I	222.5737	814.4561	305.8267
1391556_at	310207	Sema5a_p	sema domain, seven thrombospondin	222.5127	53.74168	71.92753
1379656_a_at	313235	Coro2a	coronin, actin binding protein 2A	222.506	2426.376	135.2183
1368360_at	85253	Plg	plasminogen	222.3591	202.6608	35.71108
1381148_at	498235	NA	NA	222.2247	52.93008	22.62051
1377803_at	300663	RGD13053	hypothetical LOC300663	222.188	172.7802	342.1212
1368456_at	29694	Gabrr1	gamma-aminobutyric acid (GABA-C) re	222.1624	67.76115	73.69773
1375941_at	304282	Baiap2l1	BAI1-associated protein 2-like 1	222.1492	1242.597	875.2502
1393890_at	499818	RGD15620	similar to protein phosphatase 1, regul	222.0795	132.9747	138.7823
1391767_at	309809	RGD13104	similar to KIAA1919 protein (predicted)	221.8982	120.7004	189.2533
1384799_at	302396	RGD15619	similar to KIAA2022 protein (predicted)	221.5491	229.6391	261.7968
1384871_at	311129	Osbpl6_pre	oxysterol binding protein-like 6 (predict	221.4633	236.64	353.1594
1383128_at	360906	RGD13085	similar to RIKEN cDNA 2900024C23 (p	221.4209	312.9342	481.0207
1383605_at	360919	LOC36091	similar to alpha-fetoprotein	221.3837	687.4659	446.158
1376766_at	287746	Fmnl1_pre	formin-like 1 (predicted)	221.3737	392.7842	578.2546
1369540_at	64169	Efcbp1	EF hand calcium binding protein 1	221.3694	184.5714	171.755
1369413_at	29375	Uncx4.1	Unc4.1 homeobox (C. elegans)	221.0889	106.4049	92.07074
1397709_at	691397	LOC69139	NA	221.0344	221.4467	206.736

1384491_at	316424	RGD1311S	similar to aldehyde oxidase structural I	220.8824	51.92129	27.75545
1375081_at	287368	Nt5m_pred	5',3'-nucleotidase, mitochondrial (predi	220.7753	392.1521	309.9279
1388782_at	252856	Tcf21	transcription factor 21	220.6494	74.5951	267.995
1388939_at	298069	Col15a1	procollagen, type XV	220.4059	213.6428	880.2106
1369165_at	60395	Trpc3	transient receptor potential cation char	220.2716	224.764	118.3562
1377660_at	293613	RGD13093	similar to transthyretin (4L369) (predict	220.2574	14.57392	1094.709
1370068_at	29354	Pla2g5	phospholipase A2, group V	220.2117	100.5405	42.01613
1377486_at	499013	NA	NA	220.1304	59.8081	45.74948
1388168_a_at	25022	Fgfr2	fibroblast growth factor receptor 2	220.0217	140.777	76.06135
1376925_at	310811	Palmd	palmdelphin	219.608	50.56347	271.7701
1368316_at	29172	Aqp8	aquaporin 8	219.5086	47.24274	60.16614
1393660_at	301118	LOC30111	NA	219.4633	306.2949	103.0214
1382950_at	685067	LOC68506	NA	219.312	1638.713	1034.896
1392322_at	500113	Gimap7	GTPase, IMAP family member 7	219.3041	20.34965	103.9136
1369750_at	25653	Tshb	thyroid stimulating hormone, beta subu	219.2369	67.32425	83.32207
1377074_at	291138	LOC29113	NA	219.179	44.59892	66.45694
1384466_at	493574	LOC49357	notch1-induced protein	219.0847	198.602	791.5422
1373971_at	296048	Agpat7_pre	1-acylglycerol-3-phosphate O-acyltrans	218.9934	1113.009	117.1815
1387043_at	60378	Lypd3	Ly6/Plaur domain containing 3	218.9879	484.2558	147.1184
1375328_at	353251	Prss21	protease, serine, 21	218.9717	109.5191	134.7991
1391330_at	252959	Bm259	BM259 protein	218.8604	6.562283	914.3239
1368422_at	29279	Meox2	mesenchyme homeobox 2	218.7549	23.38071	189.4232
1387385_at	117258	Cntn1	contactin 1	218.0339	433.8291	774.6136
1368797_at	65035	Nr1i3	nuclear receptor subfamily 1, group I, r	218.029	210.7567	30.90441
1390016_at	298867	Kbtbd9_pre	kelch repeat and BTB (POZ) domain c	218.0185	136.5864	88.52635
1370486_a_at	252958	Iiig9	IIIG9 protein	217.9797	257.8023	147.6583
1376284_at	361623	LOC36162	NA	217.8298	403.2184	271.8597
1373302_at	500488	NA	NA	217.7217	76.98685	218.9804
1388224_at	64543	Sec14l3	SEC14-like 3 (S. cerevisiae)	217.693	185.2339	36.44636
1370706_a_at	313375	Cyp2j9	cytochrome P450, family 2, subfamily j	217.5282	60.80767	183.1041
1369119_a_at	65032	Htr7	5-hydroxytryptamine (serotonin) recep	217.4738	113.9061	136.1044
1376908_at	309526	Ifit3	interferon-induced protein with tetratric	217.4586	69.3563	179.9274
1377110_at	497990	RGD15631	similar to ADP-ribosylation-like factor 1	217.4034	26.03555	389.1948
1379932_at	60586	Clcn4-2	putative chloride channel 4-2	217.3961	135.4263	407.5064
1369213_at	50687	L1cam	L1 cell adhesion molecule	217.1407	461.0017	130.2987
1391612_at	444986	Il22ra2	interleukin 22 receptor, alpha 2	217.0549	82.37079	455.6588
1389688_at	288016	Leprel1	leprecan-like 1	217.0111	131.4657	117.1377
1375508_at	364670	RGD1559S	similar to hypothetical protein (predicte	216.9863	249.9929	107.7549
1388721_at	113906	Hspb8	heat shock 22kDa protein 8	216.8552	50.30886	284.7376
1375456_at	309399	RGD13052	similar to Cgi67 serine protease precur	216.7847	426.9976	139.2409
1376920_at	500013	LOC50001	NA	216.6779	28.9628	568.3382
1395643_at	317170	Slc9a7_pre	solute carrier family 9 (sodium/hydroge	216.6633	382.4289	297.1377
1388097_at	140726	Cacng5	calcium channel, voltage-dependent, g	216.6146	37.20323	95.56794
1380546_at	298250	LOC29825	similar to hypothetical protein FLJ1098	216.5855	457.343	2337.065
1369891_at	60462	Jdp1	Jun dimerization protein 1	216.4536	453.5597	300.81
1370110_at	116489	Kcnk4	potassium channel, subfamily K, meml	216.417	46.93007	77.65547
1387283_at	286918	Mx2	myxovirus (influenza virus) resistance	216.395	62.55713	333.6672
1368707_at	54404	Itih4	inter alpha-trypsin inhibitor, heavy chai	216.2834	130.9806	167.22
1387232_at	25296	Bmp4	bone morphogenetic protein 4	216.1109	137.3408	238.3645
1369867_at	25280	St8sia1	ST8 alpha-N-acetyl-neuraminide alpha	216.0397	105.4251	56.15898
1382018_at	296158	RGD13088	similar to chromosome 20 open readin	216.0189	372.858	391.2817

1382563_at	499112	RGD15608	similar to hypothetical protein FLJ3894	215.8416	446.5046	332.9646
1393659_at	310846	Tram1l1_p	translocation associated membrane pr	215.6519	522.5215	1356.389
1373257_at	363154	RGD13072	similar to protein phosphatase 1, regul	215.3275	125.4147	151.3344
1370582_a_at	29160	Amelx	amelogenin X chromosome	215.2605	28.65175	8.51501
1384968_at	305450	Sh3bp2	SH3-domain binding protein 2	215.0518	144.9916	97.53666
1396578_at	117253	Dnah11	dynein, axonemal, heavy polypeptide 1	215.0485	125.3915	25.1625
1387989_at	24419	Grm6	glutamate receptor, metabotropic 6	214.9213	111.0064	145.5034
1378815_at	362203	RGD13069	similar to Gamma-tubulin complex com	214.7617	135.0471	39.56764
1377067_at	363217	NA	NA	214.4543	201.735	647.256
1389263_at	294804	Rai14	retinoic acid induced 14	214.4345	865.462	249.2128
1381073_at	308478	RGD13117	similar to SPRED-3 (predicted)	214.4336	217.3254	214.979
1370615_at	286989	LOC28698	UDP-glucuronosyltransferase	214.3005	65.11325	92.34666
1375402_at	307128	Frmd4a_pr	FERM domain containing 4A (predicte	214.2836	84.27247	311.2338
1374475_at	313917	Abhd1	abhydrolase domain containing 1	214.1711	197.5397	35.20574
1385434_at	81752	Ptger2	prostaglandin E receptor 2, subtype EI	214.1176	50.51951	686.8062
1369530_at	57233	Isl2	insulin related protein 2 (islet 2)	213.9967	187.4213	154.6352
1395695_at	305494	Aebp1_pre	AE binding protein 1 (predicted)	213.9698	144.6536	127.2472
1371142_at	25251	Cyp2g1	cytochrome P450, subfamily 2G, polyp	213.9047	140.6484	157.4617
1397518_at	498057	NA	NA	213.7774	637.872	88.08108
1385286_at	309449	Elovl3_pre	elongation of very long chain fatty acid	213.7609	64.71043	41.29604
1394218_s_at	314930	Zfpm2_pre	zinc finger protein, multitype 2 (predict	213.7439	753.2973	900.0169
1393390_at	316507	LOC31650	NA	213.6687	139.1191	208.5837
1387467_at	29718	Kcnj10	potassium inwardly-rectifying channel,	213.5838	31.64891	176.6318
1385295_at	315465	RGD13100	similar to hypothetical protein MGC209	213.5232	206.5085	255.2239
1389770_at	311548	LOC31154	similar to RIKEN cDNA 4930509O20	213.4544	70.5552	66.52604
1371227_at	116630	Csf2	colony stimulating factor 2 (granulocyte	213.4296	48.76861	241.6908
1372468_at	361383	Cd97	CD97 antigen	213.4007	289.832	375.6762
1387709_at	360457	Figf	c-fos induced growth factor	213.2953	15.33397	92.72179
1388074_at	286912	Krt20	keratin 20	213.2921	265.2262	178.0961
1387713_a_at	25047	Fcer1a	Fc receptor, IgE, high affinity I, alpha p	213.1732	19.56432	118.3642
1387638_a_at	63835	Ctla4	cytotoxic T-lymphocyte-associated pro	213.142	48.09354	206.6551
1384479_at	366061	Galnt3	UDP-N-acetyl-alpha-D-galactosamine:	212.8848	180.1681	237.3899
1377286_at	286938	Gimap4	GTPase, IMAP family member 4	212.8618	138.8466	35.10607
1391327_at	363181	LOC36318	similar to RIKEN cDNA 1700001E04	212.8451	91.64037	339.788
1376452_at	501077	NA	NA	212.8333	173.2055	81.93303
1382387_at	309135	Tmem16a_	transmembrane protein 16A (predicted	212.8296	80.39158	73.77841
1378245_at	315789	RGD13119	similar to 6430514L14Rik protein (pred	212.7824	565.9403	128.9299
1370151_at	497840	Cps1	carbamoyl-phosphate synthetase 1, m	212.6047	66.52353	110.0722
1382217_at	294747	RGD15611	similar to BC067074 protein (predicted	212.5129	122.7581	78.75433
1376165_at	85267	Slc24a3	solute carrier family 24 (sodium/potass	212.5037	518.6822	2035.227
1382339_a_at	308215	Chd1_pred	chromodomain helicase DNA binding p	212.4667	178.8469	232.4742
1388145_at	25602	Tnxa	tenascin XA	212.366	124.1162	280.8861
1375151_at	366733	RGD15651	similar to RAP2A, member of RAS onc	212.3586	142.9632	16.95863
1397773_at	315953	LOC31595	similar to claudin-18A1.2	212.1693	115.8114	134.1088
1387097_at	58924	Fut2	fucosyltransferase 2 (secretor status ir	212.1077	199.0732	297.9733
1398160_at	297406	Cct7_predi	chaperonin subunit 7 (eta) (predicted)	212.0832	88.54812	72.2834
1387748_at	25608	Lep	leptin	212.053	11.64445	17.93158
1369815_at	25542	Ccl3	chemokine (C-C motif) ligand 3	211.9979	115.6196	555.313
1391955_at	500088	LOC50008	similar to RAB19, member RAS oncog	211.9498	191.191	243.6757
1391730_at	362996	Mll2_predi	myeloid/lymphoid or mixed-lineage leu	211.7718	919.5315	917.9799
1369477_at	170636	Pnma1	paraneoplastic antigen MA1	211.7697	288.1625	90.44871

1371138_at	59299	Trdn	triadin	211.7497	52.67471	93.75533
1371361_at	29136	Tns	tensin	211.5775	131.8848	11.40796
1374366_at	300051	Slc39a4_pr	solute carrier family 39 (zinc transporte	211.457	120.3707	146.4632
1368873_at	24452	Hoxa2	homeo box A2	211.3885	20.83614	23.75999
1393592_at	294449	Hs3st5_pre	heparan sulfate (glucosamine) 3-O-sul	211.3325	182.9246	268.5728
1369662_at	24766	Scn2a1	sodium channel, voltage-gated, type 2	211.2782	136.8373	101.9117
1387454_at	63912	Niban	niban protein	211.1298	156.9674	97.00847
1376540_at	501104	NA	NA	211.0448	56.67457	459.2929
1369356_at	116556	Gucy2f	guanylate cyclase 2f	211.0012	56.65822	30.8262
1379504_at	367131	NA	NA	210.9137	13.74404	155.2434
1369983_at	81780	Ccl5	chemokine (C-C motif) ligand 5	210.8896	133.3027	198.6557
1369846_at	60583	Ivl	involucrin	210.8853	18.37442	49.94438
1389107_at	315795	RGD13046	similar to KIAA1749 protein (predicted)	210.8388	56.38677	1442.422
1368421_at	29644	Ptpn5	protein tyrosine phosphatase, non-rece	210.8179	58.97221	196.8115
1387741_at	25075	Htr1b	5-hydroxytryptamine (serotonin) recep	210.5819	356.0376	292.6559
1377642_at	363425	Cav2	caveolin 2	210.3844	13.56855	128.6475
1374080_at	308406	Six5_predi	sine oculis-related homeobox 5 homol	210.3088	148.0935	250.3808
1376889_at	619550	Gpr153	G protein-coupled receptor 153	210.3058	875.6806	774.5059
1384583_at	446171	Smgc	neonatal submandibular gland protein	210.2158	26.02562	36.48377
1393682_at	554353	GPR34_pr	G-protein-coupled receptor GPR34 (pr	210.2099	39.58563	88.23818
1388218_at	300438	Ldlr	low density lipoprotein receptor	210.1437	831.479	203.6978
1369592_at	192645	Wbp2	WW domain binding protein 2	209.9806	351.3575	92.84233
1386141_at	498167	RGD15661	similar to Zinc finger, CW type with PW	209.8421	45.83125	190.2674
1398604_at	305475	Osbp2_pre	oxysterol binding protein 2 (predicted)	209.8249	309.2742	620.2841
1391309_at	498797	NA	NA	209.7986	69.5968	293.5807
1367594_at	25181	Bgn	biglycan	209.7232	55.56061	728.8547
1388720_at	361225	NA	NA	209.6328	667.9077	172.1912
1387310_at	171496	Atp2c2	ATPase, Ca++ transporting, type 2C, r	209.4831	723.4362	28.46599
1374496_at	362364	RGD13108	similar to RIKEN cDNA 1200009O22; l	209.4084	107.607	92.23617
1383851_at	289716	RGD13051	similar to RIKEN cDNA 4921513E08 (p	209.2202	453.9857	1086.774
1370720_at	266771	LOC26677	putative pheromone receptor VN6	209.0867	56.22743	191.2543
1397780_at	685648	LOC68564	NA	209.0811	147.5441	214.3963
1385550_at	304555	Hps4_pred	Hermansky-Pudlak syndrome 4 homol	209.0516	210.1159	163.3498
1390940_at	311071	Zfhx1b	zinc finger homeobox 1b	209.042	110.691	172.5133
1396796_at	310091	RGD13118	similar to hypothetical protein FLJ9070	209.0009	54.36099	231.82
1368495_at	25616	Rln1	relaxin 1	208.9525	197.9827	587.2681
1378955_at	303692	St6galnac2	ST6 (alpha-N-acetyl-neuraminyl-2,3-be	208.9183	139.343	41.38757
1368637_at	64171	Card9	caspase recruitment domain family, me	208.8476	540.4607	64.3553
1393696_at	499856	LOC49985	hypothetical protein LOC499856	208.5767	12.09986	188.3259
1369175_a_at	25376	Ambn	ameloblastin	208.5614	29.25305	208.4817
1384017_at	308703	Lrrk1_pred	leucine-rich repeat kinase 1 (predicted	208.5474	90.19442	279.5635
1377721_at	499021	RGD15610	similar to PACRG (predicted)	208.5392	247.4551	486.2556
1387449_at	25360	Tshr	thyroid stimulating hormone receptor	208.4918	109.7857	74.08014
1369365_at	50678	Pde3a	phosphodiesterase 3A	208.4141	67.97102	41.22798
1370361_at	245918	Cgref1	cell growth regulator with EF hand dom	208.4029	34.91124	304.4047
1369510_at	66020	Gapdhs	glyceraldehyde-3-phosphate dehydrog	208.3983	381.7749	145.5091
1370824_at	252919	Slc38a3	solute carrier family 38, member 3	208.2981	222.3551	96.70226
1369190_at	497761	Cd2	CD2 antigen	208.1753	110.6781	17.45573
1370959_at	84032	Col3a1	procollagen, type III, alpha 1	208.1512	137.8002	10304.19
1385132_at	297783	Mybl1_pre	myeloblastosis oncogene-like 1 (predic	208.0662	3903.308	1063.012
1386009_at	298518	Csf3r_pred	colony stimulating factor 3 receptor (gr	207.9867	93.84519	100.8315

1379499_at	361795	Ltb	lymphotoxin B	207.841	37.41112	351.09
1379006_at	308337	RGD1565	similar to zinc finger protein ZFP (pred	207.8406	148.0329	218.3717
1370572_at	192251	Gpr149	G protein-coupled receptor 149	207.731	214.9471	150.59
1371259_at	310738	Ngfb_mapp	nerve growth factor, beta (mapped)	207.4637	48.33356	204.9276
1374895_at	316740	Myom1	myomesin 1 (skelemin) 185kDa	207.4381	78.6458	20.39477
1381531_at	365834	RGD1565C	similar to RIKEN cDNA 4632419K20 (p	207.3969	20.83959	55.35259
1373331_at	311866	RGD15644	similar to Leucine rich repeat and steri	207.331	405.2661	650.112
1384729_at	304799	Ppp1r15b_	protein phosphatase 1, regulatory (inhi	207.2753	18.9942	12.06283
1393747_at	408233	Spink4	Kazal type serine protease inhibitor 4	207.1859	59.01701	60.68224
1370499_at	362443	Klrb1a_ma	killer cell lectin-like receptor subfamily	207.0449	5.104633	118.4941
1393663_at	246235	Slc36a2	tramdorin 1	206.8685	44.56072	113.344
1369264_at	24298	Cyp21a1	cytochrome P450, subfamily 21A, poly	206.8657	121.7795	52.39766
1397515_at	309105	RGD1310C	similar to hypothetical protein FLJ4036	206.7927	1013.439	336.7496
1387289_at	83610	Apba2	amyloid beta (A4) precursor protein-bi	206.659	515.5931	97.83191
1369245_at	81645	Chrm2	cholinergic receptor, muscarinic 2	206.6039	86.12604	104.3787
1375744_at	171299	Foxd1	forkhead box D1	206.4526	24.22331	58.98061
1373151_at	499615	RGD15601	similar to RIKEN cDNA 2810489O06 (	206.421	508.1412	650.9125
1377153_a_at	287974	Klhl6_pred	kelch-like 6 (Drosophila) (predicted)	206.3382	26.57061	245.0004
1393417_at	500468	NA	NA	206.2952	215.293	303.8364
1377950_at	307415	RGD13093	similar to interferon-inducible GTPase	206.2587	168.7451	211.0117
1369660_at	83687	Defb1	defensin beta 1	206.1996	110.4076	56.6899
1376305_at	308857	Dnajb13	DnaJ (Hsp40) related, subfamily B, me	206.1187	99.72466	30.6912
1388092_at	363640	Olr1493	olfactory receptor gene Olr1493	206.0234	127.9458	57.18829
1398304_at	64512	Fzd2	frizzled homolog 2 (Drosophila)	206.0109	389.6302	121.2574
1368671_at	64316	Srpx	sushi-repeat-containing protein	205.9587	31.2829	656.0905
1397039_at	360465	Ercc4_prec	excision repair cross-complementing r	205.8673	324.7393	284.5028
1396039_at	365398	Slc22a12	solute carrier family 22 (organic anion/	205.8237	37.42021	78.65538
1370241_at	29298	Cyp2c7	cytochrome P450, family 2, subfamily c	205.7807	2574.73	113.3149
1370762_at	287001	Olr287	olfactory receptor 287	205.7339	148.13	213.653
1367563_at	24791	Sparc	secreted acidic cysteine rich glycoprot	205.6071	285.1578	759.0465
1393076_at	313825	Vps13d_pr	vacuolar protein sorting 13D (yeast) (p	205.5945	45.8388	272.5094
1388111_at	25043	Eln	elastin	205.5633	199.7785	304.1591
1377718_at	501552	RGD15615	similar to CDNA sequence BC022692	205.4074	633.6565	352.0211
1368970_at	114102	Cdh23	cadherin 23 (otocadherin)	204.7406	553.598	173.0364
1381511_at	140447	Slc8a2	solute carrier family 8 (sodium/calcium	204.7175	91.57164	731.9212
1393913_at	311852	RGD1311C	similar to 1700113K14Rik protein (prec	204.6475	132.5983	157.8296
1370943_at	171072	Sult1c2	sulfotransferase family, cytosolic, 1C, r	204.5482	22.22369	143.6729
1380688_at	293181	Galntl4_pre	UDP-N-acetyl-alpha-D-galactosamine:	204.4588	933.9517	867.8178
1382889_s_at	59313	Psgb1	pregnancy-specific beta 1-glycoprotein	204.4326	157.9048	142.8749
1376958_at	306892	RGD15628	similar to serine (or cysteine) proteinas	204.3627	138.1039	215.2335
1397646_at	499813	NA	NA	204.3608	13.73297	186.8165
1369659_at	116700	Cga	glycoprotein hormones, alpha subunit	204.25	90.6754	33.93773
1367652_at	24484	Igfbp3	insulin-like growth factor binding protei	204.0938	2657.821	569.5073
1368799_at	64041	Birc5	baculoviral IAP repeat-containing 5	204.0405	3132.391	217.7649
1397716_at	299113	Klhdc2	kelch domain containing 2	203.8998	149.8028	254.3402
1394037_at	502011	NA	NA	203.8857	421.3446	241.5734
1397264_at	499679	NA	NA	203.6593	108.4479	598.032
1391068_at	415052	E030032D1	E030032D13Rik gene	203.5303	136.792	27.09083
1368376_at	117274	Nr0b2	nuclear receptor subfamily 0, group B,	203.4384	105.7576	133.3653
1368979_at	84009	Kalrn	kalirin, RhoGEF kinase	203.3997	2207.672	276.5814
1387735_at	63849	Mmp8	matrix metallopeptidase 8	203.391	12.52634	79.80052

1383197_at	619374	Jam2	junction adhesion molecule 2	203.1086	91.7439	1479.671
1393681_at	408244	Tessp5	testis-specific serine protease-5	203.0645	24.25521	53.23012
1388281_at	309584	RT1.M4_pr	MHC class Ib antigen (predicted)	203.0493	25.83284	144.3527
1379582_a_at	114494	Ccna2	cyclin A2	202.96	9146.167	198.3699
1368219_at	29232	Clcn2	chloride channel 2	202.9497	319.0813	166.2483
1380025_at	287954	Dgcr8_pre	DiGeorge syndrome critical region gen	202.7541	131.5549	57.7535
1385173_at	361668	Ebf3_predi	early B-cell factor 3 (predicted)	202.5928	766.8301	427.1573
1381633_at	367832	Zfx_predict	zinc finger protein X-linked (predicted)	202.5151	35.42625	123.1746
1395367_at	305478	RGD13107	similar to RIKEN cDNA 0610009J22 (p	202.4794	649.9332	221.1131
1396238_at	313878	Galnt14	UDP-N-acetyl-alpha-D-galactosamine:	202.2913	2325.591	1586.963
1376481_at	312566	Adamts9_p	a disintegrin-like and metalloprotease (	202.2682	17.07602	998.3157
1377680_at	84422	Gfra3	glial cell line derived neurotrophic facto	202.1683	81.8313	256.7475
1369228_at	81680	Lifr	leukemia inhibitory factor receptor	202.0169	154.3095	217.1803
1368980_at	114633	Plce1	phospholipase C, epsilon 1	202.0122	187.3477	180.1652
1370696_at	207121	LOC20712	membrane and microfilament-associat	201.9469	23.34389	71.41866
1393496_at	299599	Theg	testicular haploid expressed gene	201.9076	210.9801	37.71083
1375640_at	297123	Fkbp9	FK506 binding protein 9	201.7694	30.10708	1027.033
1384351_at	363493	Taf7l_pred	TAF7-like RNA polymerase II, TATA b	201.7548	62.94754	55.96488
1391442_at	192249	Ehd3	EH-domain containing 3	201.7257	748.0516	71.49155
1370443_at	171575	Dnase2	deoxyribonuclease II	201.6788	181.5625	149.4018
1387197_at	83717	Omd	osteomodulin	201.6726	101.8711	344.8592
1368622_at	114508	Fbp2	fructose-1,6-bisphosphatase 2	201.5952	155.0919	261.2669
1380839_at	499977	RGD15597	similar to lipoma HMGIC fusion partne	201.5669	163.2492	178.8624
1397623_at	500072	NA	NA	201.5136	20.54136	153.6558
1376777_at	24711	Rbp3	retinol binding protein 3, interstitial	201.4534	114.9751	141.5953
1369692_at	25567	Tnr	tenascin R	201.3691	407.2307	392.7165
1390633_at	296229	P22k15	cystatin related protein 2	201.3302	46.39204	36.55495
1388003_at	29508	Nfya	nuclear transcription factor-Y alpha	201.1057	562.2633	205.2548
1385056_at	294252	Msh5	mutS homolog 5 (E. coli)	201.0499	40.83783	171.9852
1390287_at	366988	Bin2	bridging integrator 2	201.0488	39.72989	328.2034
1368413_at	65029	Abp1	amiloride binding protein 1 (amine oxid	201.0412	51.22795	59.98601
1390510_at	293749	Ms4a6b	membrane-spanning 4-domains, subfa	201.0399	110.2886	100.1409
1385018_at	681331	LOC68133	NA	200.9944	92.41883	13.80237
1389470_at	294257	Cfb	complement factor B	200.8919	29.41306	14.49436
1382519_at	362151	Lnp_predic	limb and neural patterns (predicted)	200.8406	380.0292	368.8602
1369393_at	116596	Map3k8	mitogen-activated protein kinase kinas	200.7857	104.4448	3740.022
1370765_at	266704	Gzmg	granzyme G	200.7498	41.81661	93.71805
1379741_at	296981	Atp6v0a4_	ATPase, H+ transporting, lysosomal V	200.7089	164.4434	191.4943
1394775_at	365652	Col4a3bp_	procollagen, type IV, alpha 3 (Goodpa	200.4504	54.95751	24.81933
1380142_at	24457	Hoxb8_ma	homeo box B8 (mapped)	200.3044	51.9666	5.092112
1379537_at	363231	RGD13073	similar to 3222401M22Rik protein (pre	200.2875	44.57858	99.94544
1387182_at	117549	Gpr37	G protein-coupled receptor 37	200.2866	178.0988	224.0214
1389605_at	305434	MGC12521	multiple coiled-coil GABABR1-binding	200.2106	1474.98	89.62887
1374104_at	65131	Cldn5	claudin 5	200.0975	26.76896	23.56317
1370056_at	56778	Ly6c	Ly6-C antigen	200.08	121.9309	171.9503
1384744_at	500925	MGC11446	NA	199.775	292.7871	272.0999
1373780_at	298436	Tspan1	tetraspanin 1	199.7473	157.0104	350.7102
1393433_at	365691	RGD13065	similar to Agrin (predicted)	199.7402	7.546166	12.30091
1381947_at	295622	RGD15602	RGD1560210 (predicted)	199.6385	8.891003	37.79593
1394257_at	303214	Trim16_pre	tripartite motif protein 16 (predicted)	199.6111	49.97274	48.86332
1368332_at	171164	Gbp2	guanylate nucleotide binding protein 2	199.5783	29.47117	391.2689

1387265_at	25666	Dgkg	diacylglycerol kinase, gamma	199.5546	239.2903	191.7405
1380817_at	312912	Depdc2_pr	DEP domain containing 2 (predicted)	199.5489	11.66075	120.7128
1384568_at	363681	Hspb9_pre	heat shock protein, alpha-crystallin-rela	199.5086	41.78015	122.0131
1393818_s_at	361564	RGD13111	similar to hypothetical protein FLJ3279	199.479	258.5766	323.0045
1367809_at	24656	Prlpa	prolactin-like protein A	199.4764	108.6758	212.998
1368483_a_at	65047	Slit1	slit homolog 1 (Drosophila)	199.4487	541.4518	102.2271
1394057_at	363443	Ndph_pred	Norrie disease homolog (human) (prec	199.3105	98.34255	63.22643
1367942_at	25732	Acp5	acid phosphatase 5, tartrate resistant	199.2667	70.41341	55.25808
1396404_at	361288	RGD15609	similar to FUN14 domain containing 2	199.2089	763.2741	162.1449
1386239_at	313352	Dmrta1_pr	doublesex and mab-3 related transcrip	199.1439	130.8333	121.7891
1395863_at	113984	Nr2f2	nuclear receptor subfamily 2, group F,	199.0811	233.3017	88.78075
1370447_at	192259	Phlpb	phospholipase B	199.0543	134.174	111.347
1389701_at	25154	Pgr	progesterone receptor	198.9809	344.1139	236.0766
1370033_at	56781	Mlc3	fast myosin alkali light chain	198.9568	81.78828	113.9983
1397945_at	499461	RGD15641	similar to WNT1 inducible signaling pa	198.8932	75.16136	103.938
1376487_at	293048	Rhcg	Rhesus blood group-associated C glyc	198.8801	247.0056	80.48312
1389824_at	25400	Camk2a	calcium/calmodulin-dependent protein	198.8159	234.7213	333.4184
1370039_at	25345	Prm2	protamine 2	198.6123	43.91122	62.27834
1371077_at	58963	Htr3b	5-hydroxytryptamine (serotonin) recep	198.5661	24.90594	58.10834
1376439_at	296968	RGD13082	similar to hypothetical protein FLJ3278	198.4973	154.5217	42.36084
1382827_at	295052	Ccna1	cyclin A1	198.4916	663.2989	232.696
1369025_at	54236	Cd5	CD5 antigen	198.3641	475.6428	132.3778
1369884_at	29348	Fgf7	fibroblast growth factor 7	198.2976	123.9546	173.6163
1383630_at	306760	Dok3_pred	docking protein 3 (predicted)	198.0352	119.4364	242.1087
1386865_at	25434	Sparcl1	SPARC-like 1 (mast9, hevin)	198.0273	54.02511	2404.241
1387945_at	502131	NA	NA	197.9303	99.20827	29.45207
1375646_at	289280	Efcab2_pre	EF-hand calcium binding domain 2 (pr	197.919	232.6842	1007.769
1384485_at	116680	Ptpru	protein tyrosine phosphatase, receptor	197.8642	242.6395	215.8132
1384283_at	367785	Asb9_pred	ankyrin repeat and SOCS box-containi	197.8129	178.7163	13.01747
1370843_at	245986	Gng8	guanine nucleotide binding protein (G	197.7739	99.34471	81.70576
1385316_at	363780	NA	NA	197.7612	335.9511	61.99089
1369171_at	24566	Mst1	Macrophage stimulating 1 (hepatocyte	197.6732	65.48207	79.21431
1370588_a_at	29715	Slc8a1	solute carrier family 8 (sodium/calcium	197.6186	283.7583	49.25065
1369580_at	78980	Tas2r16	taste receptor, type 2, member 16	197.5467	55.82783	65.99254
1376819_at	315019	RGD15622	similar to breast cancer membrane pro	197.4444	138.5325	187.9275
1374934_at	288995	Gpr39	G protein-coupled receptor 39	197.4352	153.2822	294.565
1381166_at	308486	Znf383_pre	zinc finger protein 383 (predicted)	197.3814	90.1476	167.9262
1367841_a_at	171406	Prlpc2	prolactin-like protein C 2	197.3225	25.31577	155.3123
1384700_at	502300	RGD15619	similar to chromosome 6 open reading	197.3192	124.7684	240.8047
1396898_at	313262	Pappa_pre	pregnancy-associated plasma protein	197.3177	184.0627	108.0345
1370387_at	171352	Cyp3a13	cytochrome P450, family 3, subfamily a	197.2244	136.116	143.1033
1392032_at	363616	Grap	GRB2-related adaptor protein	197.1103	84.06853	90.74471
1369216_a_at	114110	Flt4	fms-related tyrosine kinase 4	197.0825	43.54492	90.32309
1387181_at	25714	Myf6	myogenic factor 6	197.0772	140.9665	144.7011
1392248_at	79219	Grid1	glutamate receptor, ionotropic, delta 1	196.9833	278.7905	212.7539
1369455_at	114628	Abcg5	ATP-binding cassette, sub-family G (W	196.8659	145.8667	57.41567
1369277_at	29386	Mecp2	methyl CpG binding protein 2	196.8444	151.2828	140.9488
1369802_at	29731	Kcna3	potassium voltage-gated channel, shal	196.6356	19.3757	91.68629
1391737_at	500904	LOC50090	NA	196.4426	175.8356	65.09839
1392834_at	303395	RGD13101	similar to Chromodomain-helicase-DN	196.409	103.7679	394.3319
1391925_at	362506	Ccl19_prec	chemokine (C-C motif) ligand 19 (pred	196.3497	111.3175	177.1868

1370775_a_at	24241	Calca	calcitonin/calcitonin-related polypeptide	196.311	99.86058	8633.901
1368188_at	29531	Hpd	4-hydroxyphenylpyruvic acid dioxygena	196.2482	49.40863	20.51822
1394743_at	362010	NA	NA	196.1029	19.93648	125.5219
1393622_at	291209	RGD1561C	similar to HECT type E3 ubiquitin ligas	196.0099	80.04206	64.65386
1373987_at	56817	Kcnip2	Kv channel-interacting protein 2	195.9341	130.2454	318.7801
1370742_at	64029	Rsd6	RSD-6	195.8179	64.84728	38.53251
1372639_at	362708	Trim54	tripartite motif-containing 54	195.8154	22.79423	136.024
1387704_at	24890	Esr1	estrogen receptor 1	195.6683	34.843	32.50277
1369549_at	24934	Klrk1	killer cell lectin-like receptor subfamily	195.5936	142.4954	149.3344
1392791_at	25148	Egr3	early growth response 3	195.5845	10.37598	2637.382
1384255_at	307382	Napg	N-ethylmaleimide-sensitive factor attac	195.5837	1269.348	235.7138
1387706_at	140674	Gabrg1	gamma-aminobutyric acid A receptor,	195.4366	114.3438	200.7408
1395617_at	50692	Plaur	plasminogen activator, urokinase rece	195.4189	273.7742	16.35358
1377974_at	362800	Itgb8_pred	integrin beta 8 (predicted)	195.3661	69.23361	303.2564
1376831_at	360559	RGD13087	similar to hypothetical protein FLJ1015	195.2569	4034.959	87.23471
1369748_at	171149	Serpini2	serine (or cysteine) peptidase inhibitor	195.1923	75.76629	848.7784
1395644_at	303637	Abca8b_pr	ATP-binding cassette, sub-family A (Al	195.1081	356.6798	1510.168
1395182_at	304920	Klhl20_pre	kelch-like 20 (Drosophila) (predicted)	195.0804	190.9532	320.9103
1367616_at	25105	Nppb	natriuretic peptide precursor type B	194.9876	42.62367	73.05106
1368911_at	25472	Kcnj8	potassium inwardly-rectifying channel,	194.9288	14.0284	68.18146
1396150_at	65129	Cldn1	claudin 1	194.7658	237.7795	138.7347
1389637_at	300097	NA	NA	194.4487	3986.939	619.5897
1391380_at	316663	Fbxl17_pre	F-box and leucine-rich repeat protein 1	194.2977	60.60818	193.7356
1398584_at	287698	Krt1-5	keratin complex 1, acidic, gene 5	194.2404	37.14799	60.58042
1381909_at	311427	Hspa12b_p	heat shock protein 70kDa 12B (predict	194.0369	162.8019	78.37729
1391104_at	303856	RGD13067	similar to putative ATPase (predicted)	193.9275	59.03651	115.763
1369858_at	24938	Grpr	gastrin releasing peptide receptor	193.8986	40.31057	60.37905
1380060_at	300029	Top1mt	DNA topoisomerase I, mitochondrial	193.824	554.9131	26.52753
1369388_at	81725	Musk	muscle, skeletal, receptor tyrosine kina	193.6791	170.2719	106.0261
1368163_at	25253	Dpp4	dipeptidylpeptidase 4	193.5003	286.4872	316.744
1374672_at	295531	Cark	cardiac ankyrin repeat kinase	193.4897	1257.808	284.5358
1386829_at	308463	Map3k10	mitogen activated protein kinase kinas	193.4626	249.6218	17.99157
1388098_at	246267	LOC24626	resection-induced TPI (rs11)	193.4239	25.62446	96.3284
1371928_at	500545	Cdca8	cell division cycle associated 8	193.2219	2686.813	104.1619
1397357_at	500124	LOC50012	similar to RIKEN cDNA 4921507P07	193.1381	73.97561	94.64598
1385518_at	313776	RGD13049	similar to RIKEN cDNA 2310042D19	193.0135	35.84273	43.63081
1397232_at	500280	Lrrn1	leucine rich repeat neuronal 1	192.9465	256.7149	329.1442
1381268_at	287185	Foxi1_prec	forkhead box I1 (predicted)	192.9403	58.33549	226.7107
1384764_at	499126	RGD15635	similar to Hypothetical protein MGC30	192.8329	310.3892	435.5735
1368107_at	58937	Prlpi	prolactin-like protein 1	192.7967	148.3012	160.9136
1398272_at	64828	B4galnt1	beta-1,4-N-acetyl-galactosaminyl trans	192.5377	715.1942	86.11656
1396106_at	307548	RGD15619	similar to dystrobrevin alpha isoform 1	192.5165	637.0871	505.944
1378752_at	314721	RGD15655	similar to cajalin 2 isoform a (predicted	192.4498	188.5138	144.3873
1389971_at	432360	Sgce	sarcoglycan, epsilon	192.4142	368.4068	1889.549
1368304_at	84493	Fmo3	flavin containing monooxygenase 3	192.3349	9.877375	46.46669
1396518_at	361223	Fgd3_pred	FYVE, RhoGEF and PH domain conta	192.3117	107.261	191.885
1384739_at	313163	RGD13061	similar to RAN protein	192.1649	121.2635	56.88612
1393773_at	303519	Krt25A	keratin 25A	192.1616	235.6028	262.8275
1370580_a_at	29296	Cyp2c37	cytochrome P450, 2c37	192.1516	10.38832	224.1343
1378013_at	361199	RGD15643	RGD1564391 (predicted)	192.0227	169.852	22.0546
1377305_at	310764	RGD13065	hypothetical LOC310764 (predicted)	191.9918	198.0591	119.8712

1388001_at	246214	Lrrc21	leucine rich repeat containing 21	191.9647	167.143	47.9781
1369553_at	117182	Hsd17b3	hydroxysteroid (17-beta) dehydrogena	191.8609	181.1709	139.767
1379716_at	312701	Cd163_pre	CD163 antigen (predicted)	191.7651	99.34723	150.1118
1385142_at	499365	NA	NA	191.7001	18.94885	144.2317
1392210_at	361244	RGD13074	similar to mKIAA0319 protein (predicte	191.6984	196.1582	62.74416
1389675_at	499772	Ier5l	immediate early response 5-like	191.622	37.57711	98.87942
1373485_at	502091	RGD15612	similar to ring finger protein 122 homol	191.5223	52.8914	621.6985
1387842_at	60566	Syt8	synaptotagmin 8	191.5135	439.2359	294.6199
1376429_at	313183	Actl7b	actin-like 7b	191.4805	119.3302	114.5712
1387531_at	29447	Msra	methionine sulfoxide reductase A	191.4143	505.7843	200.7511
1397187_at	315723	Senp8	SUMO/sentrin specific protease family	191.2026	97.8499	57.96021
1370266_at	57341	Parva	parvin, alpha	191.1846	1206.878	186.4072
1394804_at	309760	Mypn_prec	myopalladin (predicted)	190.8037	12.92405	147.2741
1389408_at	362720	Rrm2_map	ribonucleotide reductase M2 (mapped)	190.513	30486.69	341.1264
1367998_at	84386	Slpi	secretory leukocyte peptidase inhibitor	190.2429	122.9607	141.7089
1368190_at	24715	Ren1	renin 1	190.2207	147.3958	251.7968
1382267_at	366140	Fjx1_predi	four jointed box 1 (Drosophila) (predict	190.2193	206.2049	249.2665
1393586_at	363081	Nope_pred	neighbor of Punc E11 (predicted)	190.2176	35.21062	128.0304
1368353_at	24387	Gfap	glial fibrillary acidic protein	190.0763	52.7727	249.457
1375195_at	499876	NA	NA	190.024	113.1576	118.4569
1370403_at	192224	Prlpk	prolactin-like protein K	189.9205	63.51266	30.23422
1385297_at	300057	Recql4_pre	RecQ protein-like 4 (predicted)	189.8682	357.0568	8.728282
1387525_at	59316	Nxph4	neurexophilin 4	189.8322	263.3171	213.3757
1369194_a_at	25163	Cdkn2a	cyclin-dependent kinase inhibitor 2A	189.7971	47.9112	68.12773
1384752_at	362446	RGD15630	similar to osteoclast inhibitory lectin (pi	189.5813	94.61154	264.4532
1387567_at	50572	Slc21a1	solute carrier family 21, member 1	189.1861	177.5945	153.3719
1377207_at	497672	Brca1	breast cancer 1	189.1093	363.7266	192.5082
1398503_at	114213	Rax	retina and anterior neural fold homeob	189.0621	117.6498	45.5161
1368543_at	85431	Nox4	NADPH oxidase 4	188.9541	118.5201	29.68072
1380666_at	307868	Mon1b_pre	MON1 homolog b (yeast) (predicted)	188.8703	42.20668	246.1225
1383453_at	361621	Olfml1	olfactomedin-like 1	188.6284	1433.616	143.9154
1379593_at	304103	Olig2_pred	oligodendrocyte transcription factor 2 (	188.5782	16.88695	112.8419
1394865_at	316018	Tmem7_pr	transmembrane protein 7 (predicted)	188.5023	98.06419	90.22096
1369286_at	25268	Proc	protein C	188.4049	14.31187	378.74
1377083_at	360595	RGD15624	similar to RIKEN cDNA 0610013E23 (p	188.3408	35.95215	124.2524
1377643_at	303991	Hoxd10_pr	homeo box D10 (predicted)	188.0571	94.03195	63.70877
1375446_at	363872	Znf498_pre	zinc finger protein 498 (predicted)	188	127.0637	326.3341
1380472_at	683587	LOC68358	NA	187.9533	73.88023	32.72016
1381958_at	315969	RGD15629	similar to mKIAA0259 protein (predicte	187.9297	186.4747	26.09344
1368502_at	25320	Gast	gastrin	187.8577	349.9992	886.993
1389852_at	315164	RGD13073	similar to proliferation associated nucle	187.8424	1774.977	200.6092
1380775_at	309523	Mphosph1_	M-phase phosphoprotein 1 (predicted)	187.6719	7200.84	239.8967
1397712_at	307595	LOC30759	NA	187.6601	70.60116	217.1914
1390033_at	360882	Igsf4b_pre	immunoglobulin superfamily, member	187.509	136.7869	370.9376
1390119_at	310552	Sfrp2	secreted frizzled-related protein 2	187.4907	112.5689	295.1172
1388223_at	286924	Gnat3	guanine nucleotide binding protein, alp	187.4568	10772.61	1429.532
1373903_at	360872	Rcsd1_pre	RCSD domain containing 1 (predicted)	187.4447	145.8698	311.6175
1387734_at	59302	Bmp15	bone morphogenetic protein 15	187.3217	15.52078	58.90479
1368161_a_at	25373	Ahsg	alpha-2-HS-glycoprotein	187.1872	84.04537	126.448
1392088_at	309962	Pde8b	phosphodiesterase 8B	187.1527	65.77246	211.6997
1389645_at	361538	Prodh2	proline dehydrogenase (oxidase) 2	187.1091	42.26866	71.25295

1381874_at	499593	RGD15656	similar to SOX2 protein (predicted)	187.0165	288.5729	21.25937
1378247_at	266787	Eaf2	ELL associated factor 2	186.9301	251.3002	224.1565
1382740_at	291961	RGD13111	similar to RIKEN cDNA E430013E20 g	186.8824	11.28227	131.8152
1377088_at	311536	RGD13046	similar to RIKEN cDNA 2310046K01	186.8818	212.4323	245.3681
1371118_a_at	299351	Ighe	immunoglobulin heavy chain (epsilon p	186.7251	94.73115	164.4843
1369225_at	25087	LOC25087	NA	186.6034	16.13781	116.4992
1387315_at	25350	Sftpd	surfactant associated protein D	186.4743	116.4147	322.9846
1397607_at	303016	RGD13108	similar to adult retina protein (predicted	186.4103	26.89067	296.4842
1384178_at	308571	Lrrc4b_pre	leucine rich repeat containing 4B (prec	186.2908	1261.41	926.2384
1398237_at	360566	NA	NA	186.2673	137.6412	224.3703
1369579_at	63878	Stc2	stanniocalcin 2	186.214	15.95539	55.51605
1368735_a_at	29465	Trpv2	transient receptor potential cation char	186.0461	106.4692	204.7241
1393191_at	500557	RGD15612	similar to RIKEN cDNA 2610200G18 (	185.9729	635.7065	112.1657
1368191_a_at	24904	Slc22a1	solute carrier family 22 (organic cation	185.9463	89.51831	108.2927
1379022_at	300475	Adamts8_p	a disintegrin-like and metallopeptidase	185.9129	156.2879	111.3477
1391907_at	303563	Nags_pred	N-acetylglutamate synthase (predicted	185.8346	65.77613	216.8831
1394977_at	289440	Abhd7_pre	abhydrolase domain containing 7 (prec	185.7857	120.7196	61.06586
1367916_at	24655	Plcd1	phospholipase C, delta 1	185.6642	131.9322	168.31
1377356_at	499211	RGD15596	similar to hypothetical protein PP1665	185.5681	269.1042	131.8655
1387254_at	59301	Ghrl	ghrelin precursor	185.4493	71.79196	3961.349
1379331_at	304913	Tnn_predic	tenascin N (predicted)	185.4089	42.44746	46.44063
1387386_at	116557	Foxj1	forkhead box J1	185.2368	70.81525	186.1153
1369465_at	24470	Hsd3b	steroid delta-isomerase, 3 beta	185.1868	28.08387	48.09833
1386677_at	641521	Lrrc4	leucine rich repeat containing 4 protein	185.1778	70.1566	76.10459
1383749_at	287644	Phospho1_	phosphatase, orphan 1 (predicted)	185.1729	40.89316	134.1168
1374775_at	291234	Mki67_pred	antigen identified by monoclonal antibo	184.9651	7754.263	730.0388
1369908_at	29625	Crhbp	corticotropin releasing hormone bindin	184.7389	23.54099	120.786
1387295_at	50676	Slc6a12	solute carrier family 6 (neurotransmitte	184.7376	47.29062	116.6553
1368078_at	64536	Esm1	endothelial cell-specific molecule 1	184.5256	43.31994	646.4577
1374978_at	303730	LOC30373	NA	184.3897	608.1786	548.6006
1377804_at	315806	RGD13071	similar to DDM36	184.2722	147.2909	72.17001
1382541_at	266802	Alk	anaplastic lymphoma kinase	184.2424	220.6001	154.422
1387022_at	24188	Aldh1a1	aldehyde dehydrogenase family 1, me	184.0182	1797.76	567.5622
1382474_at	499431	LOC49943	NA	183.9502	33.06578	201.6725
1397567_at	360849	Kif14_pred	kinesin family member 14 (predicted)	183.8073	731.9577	116.977
1368201_at	24603	Npr1	natriuretic peptide receptor 1	183.7476	41.53978	30.85327
1371089_at	494500	Yc2	NA	183.7381	118.7343	20.72474
1377033_at	287527	Serpinf2	serine (or cysteine) peptidase inhibitor	183.6114	59.90034	84.83048
1395087_at	300359	MGC94183	similar to hypothetical protein FLJ2351	183.6085	201.047	385.0244
1373521_at	307393	RGD13090	similar to RIKEN cDNA D430044G18	183.5665	57.88439	70.22596
1385078_at	361095	Zic5_predi	zinc finger protein of the cerebellum 5	183.4643	9.852185	17.30207
1393386_at	287346	RGD15619	similar to novel protein (predicted)	183.3906	10480.96	2371.264
1368108_at	116601	Atp2a1	ATPase, Ca++ transporting, cardiac m	183.3159	60.74991	51.73024
1387541_at	58982	Cspg3	chondroitin sulfate proteoglycan 3	183.1827	86.52498	63.59106
1385815_at	25381	Apeg1	aortic preferentially expressed gene 1	183.0051	194.6118	379.5427
1368530_at	117033	Mmp12	matrix metallopeptidase 12	182.9793	59.23123	611.7966
1387102_at	29335	Oprk1	opioid receptor, kappa 1	182.961	199.7349	130.1634
1396423_at	315059	RGD13094	similar to KIAA1882 protein	182.9482	54.59008	87.27206
1369261_at	94341	Kcnj13	potassium inwardly-rectifying channel,	182.9383	184.5296	112.5641
1393280_at	291359	Ly86_predi	lymphocyte antigen 86 (predicted)	182.9021	34.64496	159.3723
1371970_at	499322	RGD15609	similar to expressed sequence AW413	182.8438	8229.798	794.2295

1384320_at	301309	Bai3_predi	brain-specific angiogenesis inhibitor 3	182.7638	221.6399	2201.778
1388456_at	295214	S100a1	S100 calcium binding protein A1	182.757	451.2956	203.8625
1388055_at	286905	Cyp4f5	cytochrome P450 4F5	182.6804	50.12725	343.7805
1373005_at	65186	Krtdap	keratinocyte differentiation associated	182.5314	198.6438	57.86923
1387398_at	114906	Pkia	protein kinase inhibitor, alpha	182.4942	2372.813	1191.69
1370018_at	161476	Hspb2	heat shock 27kDa protein 2	182.4868	90.67083	150.9656
1377264_at	301291	Il17f	interleukin 17F	182.2117	37.07855	49.60226
1372986_at	116674	Jundp2	Jun dimerization protein 2	182.1856	107.2805	477.9349
1369046_at	60565	Syt6	synaptotagmin VI	182.1461	115.5718	129.1098
1396538_at	365297	RGD13096	similar to FLJ00364 protein (predicted)	182.0776	254.9089	103.5291
1369315_at	84405	Il12a	interleukin 12a	182.0494	36.68305	51.06616
1378734_at	114500	Gbx2	gastrulation brain homeobox 2	181.9961	7.712427	113.5073
1375476_at	24701	Pygm	muscle glycogen phosphorylase	181.977	170.8178	144.0628
1382292_at	361366	NA	NA	181.8398	111.9164	49.03268
1398112_at	499418	LOC49941	similar to Putative protein C21orf56 ho	181.8313	51.759	219.2103
1370147_at	171385	Acmsd	2-amino-3-carboxymuconate-6-semial	181.6348	125.1262	347.0181
1394637_at	502020	LOC50202	NA	181.588	126.8505	145.6489
1394007_at	24685	Prm1_map	protamine 1 (mapped)	181.4743	48.22405	12.99035
1385686_at	502543	NA	NA	181.2876	122.8686	168.5814
1378260_at	24172	Adh1	alcohol dehydrogenase 1 (class I)	181.252	76.64914	281.5654
1391384_at	24835	Tnf	tumor necrosis factor (TNF superfamily	181.2407	23.63953	390.3143
1398101_at	501143	RGD15595	similar to boule (predicted)	181.2275	45.55107	157.0218
1385369_at	315952	Dzip1l	DAZ interacting protein 1-like	181.2254	78.27476	717.7958
1387832_at	65036	Bche	butyrylcholinesterase	181.1943	78.46787	310.4161
1384063_at	282836	Cthrc1	collagen triple helix repeat containing 1	180.8964	70.00393	568.5534
1382375_at	64566	Wnt5a	wingless-type MMTV integration site 5A	180.8772	273.2231	224.1101
1387794_at	83517	Fcna	ficolin A	180.8002	198.8996	24.14676
1378819_at	292875	RGD13101	similar to RIKEN cDNA 0610012D14	180.7454	76.40073	217.8479
1378710_at	315438	Ccdc67	coiled-coil domain containing 67	180.7286	14.28635	102.7086
1369239_at	25749	Clcn5	chloride channel 5	180.7003	104.2863	125.7259
1392064_at	296500	Dlx1	distal-less homeobox 1	180.6942	167.0075	212.356
1368765_at	79425	Clcnk1	chloride channel K1	180.6299	169.5757	211.2647
1382954_at	289596	Corin	corin	180.5832	141.404	857.9597
1385759_at	266775	Serpinb10	serine (or cysteine) peptidase inhibitor	180.1112	90.78026	65.08775
1390878_at	24571	Muc1	mucin 1, transmembrane	180.0187	411.8487	287.0098
1389166_at	300719	Cib2	calcium and integrin binding family me	179.9964	223.524	197.9231
1391203_at	29738	Kcnab2	potassium voltage-gated channel, shal	179.9366	180.8556	164.8541
1377780_at	312728	Tspan9_pr	tetraspanin 9 (predicted)	179.8922	142.4685	96.08902
1385057_at	362420	NA	NA	179.8033	99.19178	115.0439
1390922_at	363220	Aff3_predic	AF4/FMR2 family, member 3 (predicte	179.6842	120.4997	215.875
1375141_at	497881	RGD15641	similar to HOX11L2 (predicted)	179.6304	96.40709	37.68986
1371088_at	116694	Capn9	calpain 9 (nCL-4)	179.615	94.58161	61.02618
1387591_at	25704	Il2ra	interleukin 2 receptor, alpha chain	179.6019	13.61542	64.01079
1367587_at	24282	Csh1l1	chorionic somatomammotropin hormon	179.418	194.409	123.7935
1371067_at	192271	Adam6	a disintegrin and metallopeptidase don	179.3321	110.3289	18.68737
1385450_at	170896	Ucn2	urocortin 2	179.3225	22.39307	137.3316
1370996_at	192213	Rasgrf1	RAS protein-specific guanine nucleotic	178.9974	484.7637	619.1548
1377146_at	117064	Vip	vasoactive intestinal polypeptide	178.9288	83.25542	24.33335
1370051_at	60335	Tgm1	transglutaminase 1	178.8978	47.40414	103.3608
1376799_a_at	290655	Crlf1_predi	cytokine receptor-like factor 1 (predicte	178.8892	850.5096	142.8609
1369875_at	54258	Il8ra	interleukin 8 receptor, alpha	178.8599	45.31196	51.71494

1382210_at	302495	LOC30249	NA	178.5755	203.8621	838.7861
1391245_at	309613	Ng35	Ng35 pseudogene	178.5602	333.444	265.0067
1395235_at	306342	Txnl6_prec	thioredoxin-like 6 (predicted)	178.5229	26.00655	16.84567
1376372_at	498083	NA	NA	178.3112	155.8758	48.29184
1375803_at	25637	Ptger1	prostaglandin E receptor 1	178.2958	123.5418	111.7453
1387930_at	171162	Reg3a	regenerating islet-derived 3 alpha	178.2059	15.36353	287.3491
1391296_at	502520	NA	NA	178.1827	68.70896	187.6746
1390795_at	25571	Ttpa	tocopherol (alpha) transfer protein	178.1462	107.9018	141.9511
1368621_at	65054	Aqp9	aquaporin 9	178.0194	42.36143	65.04871
1387543_at	85426	Slc5a7	solute carrier family 5 (choline transpo	177.9639	39.49509	55.80971
1390211_at	295219	Slc27a3_pr	solute carrier family 27 (fatty acid trans	177.6243	148.4038	199.3423
1373724_at	315323	Kb4	type II keratin Kb4	177.585	96.24945	182.054
1368487_at	60325	Serpinb2	serine (or cysteine) proteinase inhibito	177.5644	27.7736	78.13771
1368570_at	64047	Lrat	lecithin-retinol acyltransferase (phosph	177.5515	48.63743	81.75659
1394129_at	113970	Magi2	membrane associated guanylate kinas	177.3849	1435.993	814.5703
1390707_at	54290	Rgs10	regulator of G-protein signalling 10	177.163	1245.779	327.1347
1390881_at	286965	Abra	actin-binding Rho activating protein	177.0607	114.4775	25.91403
1398649_at	310519	Slitrk3_pre	SLIT and NTRK-like family, member 3	177.0311	31.91312	44.96396
1375136_at	291665	NA	NA	177.026	606.9993	799.463
1370299_at	24190	Aldob	aldolase B	176.986	151.2275	164.3402
1381185_at	311658	Nfatc2_pre	nuclear factor of activated T-cells, cyto	176.9814	239.7511	313.3211
1368102_at	25117	Hsd11b2	hydroxysteroid 11-beta dehydrogenase	176.757	451.364	74.42538
1391490_at	25590	Chrna4	cholinergic receptor, nicotinic, alpha po	176.7092	184.9969	129.8821
1391920_at	362832	RGD15647	similar to C19orf36 protein (predicted)	176.6397	94.8793	82.35043
1387811_at	24179	Agt	angiotensinogen (serpin peptidase inh	176.5675	57.71597	1697.575
1385125_at	246297	Myocd	transcription factor myocardin	176.5592	105.2713	182.3346
1369968_at	24924	Ptn	pleiotrophin	176.2797	530.1292	751.5626
1382623_at	359959	Rhebl1	Ras homolog enriched in brain like 1	176.2604	1189.415	337.6671
1389145_at	309175	Cdc42ep2	CDC42 effector protein (Rho GTPase	176.2561	238.5461	70.52452
1396863_at	300950	Spsb4_pre	splA/ryanodine receptor domain and S	176.1827	112.8829	737.269
1374863_at	362662	RGD15621	similar to retinoid binding protein 7 (pre	175.9264	34.54129	119.0992
1380928_at	304554	RGD15636	similar to Seizure 6-like protein precurs	175.8022	81.03452	166.7148
1393892_at	360508	RGD13066	LOC360508 (predicted)	175.7896	375.8742	24.62763
1382147_at	363915	LOC36391	NA	175.7673	16.12948	149.3904
1369163_at	24406	Grik4	glutamate receptor, ionotropic, kainate	175.751	89.22784	124.5667
1368474_at	25361	Vcam1	vascular cell adhesion molecule 1	175.7449	49.21788	747.5419
1371233_at	114122	Cspg2	chondroitin sulfate proteoglycan 2	175.7143	15.75877	47.96312
1394512_at	500803	NA	NA	175.5769	194.6743	77.12507
1391332_at	498397	NA	NA	175.5545	274.366	243.5323
1372590_at	303701	C1qtnf1	C1q and tumor necrosis factor related	175.4641	4596.958	433.1665
1375980_at	360577	Nxn_predic	nucleoredoxin (predicted)	175.438	1114.513	416.5012
1390901_at	310448	Igsf10	immunoglobulin superfamily, member	175.4367	130.8554	812.8028
1386851_a_at	54398	Ppt2	palmitoyl-protein thioesterase 2	175.3593	376.7372	126.3727
1386450_at	303236	Odf4	outer dense fiber of sperm tails 4	175.337	137.4827	41.37047
1378550_at	292699	Cblc	Casitas B-lineage lymphoma c	175.3154	90.14754	82.95236
1383812_at	501046	LOC50104	NA	175.0441	11.82685	63.09053
1372835_at	299145	Rhoj	ras homolog gene family, member J	174.8919	56.93325	237.8491
1367902_at	64199	Gng11	guanine nucleotide binding protein (G	174.7446	17.38165	245.0256
1379518_at	306873	Rreb1_pre	ras responsive element binding proteir	174.6767	196.8553	221.7887
1382479_at	311236	Lrrc4c_pre	leucine rich repeat containing 4C (prec	174.67	125.1098	224.8904
1378023_at	290847	NA	NA	174.6675	42.25837	51.42698

1387760_a_at	25231	Onecut1	one cut domain, family member 1	174.5653	22.64505	129.7647
1387145_at	29584	Gjb1	gap junction membrane channel protei	174.3258	11.13293	71.20219
1380063_at	309527	Ch25h	cholesterol 25-hydroxylase	174.3127	85.63533	390.3143
1385038_at	291936	RGD15641	similar to hedgehog-interacting protein	174.3097	262.9195	93.01209
1387257_at	24769	Sct	secretin	174.1822	574.2355	238.0404
1374634_at	315645	RGD13105	similar to RIKEN cDNA 2700059L22 (p	174.1421	298.9168	531.3986
1370794_at	246305	Spag11	sperm associated antigen 11	174.08	92.33953	114.6842
1385657_at	314251	LOC31425	erythroid spectrin beta	173.9996	36.60178	27.75663
1376027_at	362598	RGD1305C	similar to CD2-associated protein (pre	173.9757	1183.217	774.2383
1370263_at	114125	Dll3	delta-like 3 (Drosophila)	173.9204	177.1052	76.77572
1393565_at	497935	RGD15634	similar to RIKEN cDNA A030009H04 (	173.9047	177.3276	82.02167
1380617_at	246774	Gimap5	GTPase, IMAP family member 5	173.8948	107.0929	154.2689
1391457_a_at	306657	Irx2	Iroquois related homeobox 2 (Drosoph	173.8249	28.72246	10178.41
1385255_at	501069	LOC50106	NA	173.8192	35.96402	295.7156
1391775_at	363676	Zpbp2	zona pellucida binding protein 2	173.4868	282.8907	531.572
1370495_s_at	171521	Cyp2c13	cytochrome P450 2c13	173.3729	9.281515	41.17942
1369038_at	64350	Itgad	integrin, alpha D	173.0293	83.47011	81.87514
1393645_at	317274	LOC31727	hypothetical protein LOC317274	173.0224	24.88175	149.4799
1369387_at	25156	Vav1	vav 1 oncogene	172.9352	36.97839	44.42531
1369118_a_at	81668	Gnrhr	gonadotropin releasing hormone recep	172.9148	164.7627	35.41742
1385707_at	361205	Lect2_prec	leukocyte cell-derived chemotaxin 2 (p	172.616	10.41485	42.80664
1367593_at	25545	Sepw1	selenoprotein W, muscle 1	172.5762	917.1718	1164.578
1369913_at	89810	Opn1mw	opsin 1 (cone pigments), medium-wave	172.4992	33.98288	59.82816
1390348_at	293154	Folr2_pred	folate receptor 2 (fetal) (predicted)	172.4654	247.3228	206.0355
1395310_at	316628	Asb1_pred	ankyrin repeat and SOCS box-containi	172.3816	186.1261	67.27638
1369108_at	246334	Trp63	transformation related protein 63	172.3286	60.53169	232.3107
1388427_at	313770	1200013a0	limitrin	172.2886	174.494	529.485
1391141_at	499024	NA	NA	172.2275	182.2957	298.2298
1385994_at	366261	Spo11_pre	sporulation protein, meiosis-specific, S	172.2049	25.47322	514.8151
1368771_at	171396	Sulf1	sulfatase 1	171.9071	85.13079	84.11199
1379683_at	315593	RGD1565C	similar to hypothetical protein MGC178	171.8381	55.01688	142.4085
1380636_at	287454	Alox12_pre	arachidonate 12-lipoxygenase (predict	171.71	43.78175	119.8565
1375494_a_at	171297	Nlgn3	neuroligin 3	171.6027	230.9473	103.4002
1369111_at	24360	Fabp1	fatty acid binding protein 1, liver	171.3902	7.877138	96.44811
1381447_at	313775	RGD13115	similar to RIKEN cDNA 9430015G10	171.3826	504.4869	230.9973
1379978_at	313749	LOC31374	similar to mKIAA0673 protein	171.204	165.2444	66.19962
1370646_at	246306	Rmt1	mammary cancer associated protein R	171.1228	154.751	58.22996
1378966_at	498061	NA	NA	171.0392	29.02328	486.4682
1388958_a_at	25139	Slc2a4	solute carrier family 2 (facilitated gluco	171.0176	502.779	40.41165
1385829_at	363257	NA	NA	170.9881	120.8395	148.7531
1398177_at	308178	Vps37c_pr	vacuolar protein sorting 37C (yeast) (p	170.9042	188.0835	49.6825
1379967_at	306695	Zfp367	zinc finger protein 367	170.6392	4963.567	721.7005
1369492_at	57300	Aadac	arylacetamide deacetylase (esterase)	170.4717	69.99278	142.6665
1392547_at	302884	MGC10564	hypothetical LOC302884	170.4338	190.8312	291.073
1380658_at	29210	Epha3	Eph receptor A3	170.2932	119.5171	104.5924
1396951_at	500908	LOC50090	NA	170.284	68.97457	69.54741
1393185_at	499875	NA	NA	170.167	289.1615	140.1676
1369456_at	29581	Htr2b	5-hydroxytryptamine (serotonin) recep	170.1389	44.13124	80.71408
1374065_at	24553	Met	met proto-oncogene	169.9681	767.7935	9170.925
1380512_at	291984	Dpep2	dipeptidase 2	169.9549	130.109	12.81386
1368059_at	117024	Crym	crystallin, mu	169.9342	189.7469	201.0785

1378816_a_at	365410	Osbp_pred	oxysterol binding protein (predicted)	169.9222	278.7951	26.60138
1394681_at	361267	Akr1cl1_pr	aldo-keto reductase family 1, member	169.8064	71.53602	152.8867
1369764_at	24235	C4bpa	complement component 4 binding prot	169.7722	58.97128	59.87366
1382320_at	362793	RGD13073	LOC362793	169.6806	374.0937	401.981
1396624_at	499968	Galntl5	UDP-N-acetyl-alpha-D-galactosamine:	169.6251	45.33876	80.0215
1387834_at	60450	Matk	megakaryocyte-associated tyrosine kir	169.5185	298.5174	28.21397
1368794_at	56823	Haao	3-hydroxyanthranilate 3,4-dioxygenase	169.4545	91.96118	230.7978
1380816_at	307926	RGD15632	similar to 1700054N08Rik protein (pre	169.0885	187.9236	56.12538
1392648_at	291327	Mrc1_pred	mannose receptor, C type 1 (predicted	168.9924	22.29217	131.6955
1393137_at	362553	Tctex1d1_	Tctex1 domain containing 1 (predicted	168.9054	26.71179	61.54456
1397484_at	299193	RGD13096	similar to Cytosolic acyl coenzyme A tl	168.8271	340.1511	257.2641
1392780_at	501621	LOC50162	similar to nuclear RNA export factor 7	168.7747	57.81362	10.96541
1374207_at	89805	Angpt2	angiopoietin 2	168.7295	64.33559	297.142
1374284_at	362423	Rassf4	Ras association (RalGDS/AF-6) doma	168.6952	116.2684	255.4415
1367785_at	65204	Cnn1	calponin 1	168.5932	109.9358	150.4874
1392952_at	619561	LOC61956	hypothetical protein LOC619561	168.5592	209.3921	93.71845
1397086_at	500789	RGD15632	similar to PDZ-domain protein Gipc3 (p	168.5266	99.72973	45.11462
1373975_at	368066	LOC36806	NA	168.4507	109.0712	438.9889
1390676_at	313593	Eif2c3_pre	eukaryotic translation initiation factor 2	168.4437	119.7207	105.5207
1382453_at	367944	RGD15612	similar to Zinc finger protein ZIC 3 (Zin	168.4254	40.96064	98.67371
1384784_at	362052	Clca2	chloride channel calcium activated 2	168.3702	41.02216	66.76036
1395689_at	310013	LOC31001	NA	168.3609	235.4776	198.0038
1383855_at	314438	Degs2	degenerative spermatocyte homolog 2	168.3312	105.4617	224.3921
1368112_at	25539	Sag	retinal S-antigen	168.2637	41.20034	32.54071
1387424_at	25356	Cntn2	contactin 2	168.217	41.72939	170.1378
1380618_at	502709	NA	NA	168.2155	234.5355	303.2432
1371178_at	24497	Il5	interleukin 5	168.1879	32.82165	159.9362
1367953_at	25232	Tyro3	TYRO3 protein tyrosine kinase 3	168.1673	166.5736	7.640676
1383917_at	303476	Abi3	ABI gene family, member 3	168.1514	71.3557	95.76445
1372587_at	295490	Emcn	endomucin	168.0252	86.08037	479.8406
1388279_at	25221	Sry	sex determining region on Y	167.9876	81.5978	141.8728
1391884_at	306761	F12	coagulation factor XII (Hageman factor	167.9693	127.8058	117.2061
1376015_at	297748	Jph1_predi	junctophilin 1 (predicted)	167.4768	277.4325	375.4853
1383010_at	305589	Bcl11a	B-cell CLL/lymphoma 11A (zinc finger	167.408	360.3205	27.19659
1377494_at	315532	Fli1	Friend leukemia integration 1	167.1904	150.0996	161.8559
1368630_at	64822	Fabp9	fatty acid binding protein 9, testis	167.1298	17.99085	20.98607
1396219_at	302022	LOC30202	NA	167.067	18.97361	351.789
1385141_at	293012	Olig3_pred	oligodendrocyte transcription factor 3 (	167.0272	77.40681	179.2781
1369907_at	24197	Alpi	Alkaline phosphatase 1, intestinal, defi	167.0188	66.04028	204.9767
1398009_at	305476	RGD13087	similar to RIKEN cDNA 4921536K21 (p	167.0092	66.81808	53.84384
1380444_at	291312	RGD13089	similar to RIKEN cDNA 1110017I16 (p	167.0019	88.29041	37.73415
1367608_at	64348	Cryba4	crystallin, beta A4	166.9598	249.8408	110.0223
1380911_at	360590	RGD13115	LOC360590 (predicted)	166.941	174.3789	193.6822
1374310_at	295341	Ppm1j	protein phosphatase 1J	166.8637	44.77203	170.6915
1369602_at	29368	Fgf17	fibroblast growth factor 17	166.8419	49.70639	82.64279
1394960_at	307897	RGD13084	similar to RIKEN cDNA 4632417N05 (p	166.6782	195.3513	25.36053
1390283_at	312451	Tcf3_predi	transcription factor 3 (predicted)	166.6412	85.28379	143.4553
1367986_at	29602	Ptgfrn	prostaglandin F2 receptor negative reg	166.5408	1384.992	568.4527
1387189_at	29504	Slc22a3	solute carrier family 22, member 3	166.4191	75.30973	137.766
1384307_at	363985	Slc41a1_pi	solute carrier family 41, member 1 (pre	166.387	88.87293	151.494
1395089_at	24473	Htr1a	5-hydroxytryptamine (serotonin) recep	166.346	378.9412	135.5825

1386980_at	55939	Apom	apolipoprotein M	166.2754	62.80114	97.23928
1369716_s_at	25475	Lgals5	lectin, galactose binding, soluble 5	166.275	40.72625	592.3722
1390358_at	306243	Cacna2d3	calcium channel, voltage-dependent, a	166.166	909.1154	122.6557
1377453_at	315578	RGD13091	similar to hypothetical protein FLJ2355	165.8641	173.4538	635.5275
1377499_a_at	292905	Hrc	histidine rich calcium binding protein	165.7386	41.00344	163.8877
1370671_at	245708	Gucy2g	guanylate cyclase 2g	165.7305	134.2119	24.62689
1380424_at	192207	Ubxd5	UBX domain containing 5	165.688	171.3543	494.7125
1379495_at	361282	Plxdc2_pre	plexin domain containing 2 (predicted)	165.6255	42.0021	208.739
1382913_at	282587	Cttnbp2	cortactin binding protein 2	165.6246	136.9534	426.526
1368491_at	59296	Dnase2b	deoxyribonuclease II beta	165.5825	25.51804	135.8941
1384763_at	362319	RGD13066	similar to RIKEN cDNA 4930500J03	165.5596	95.09991	769.8691
1384742_at	246284	Atrx	alpha thalassemia/mental retardation s	165.4632	695.4572	1028.856
1379894_at	361032	RGD13101	similar to 3632451O06Rik protein (pre	165.4314	148.4162	1746.738
1369019_at	25102	Chrna5	cholinergic receptor, nicotinic, alpha po	165.4024	131.0868	174.0176
1383824_at	289664	Ldb2_pred	LIM domain binding 2 (predicted)	165.3295	119.2786	223.2499
1387004_at	50594	Nbl1	neuroblastoma, suppression of tumorig	165.2176	1112.506	213.3696
1374830_at	498795	RGD15594	similar to Coiled-coil domain containing	165.1549	20.56451	181.0917
1369786_at	53949	Chrm5	cholinergic receptor, muscarinic 5	164.9669	88.33935	145.7899
1369960_at	58971	Fxyd1	FXYD domain-containing ion transport	164.872	406.251	188.2151
1384910_at	312668	RGD15620	similar to putative voltage-gated calciu	164.796	676.9529	22.50798
1369780_at	114513	Rasgrf2	RAS protein-specific guanine nucleotic	164.6383	115.127	213.598
1378486_at	306055	Pcdh17_pr	protocadherin 17 (predicted)	164.4483	175.7837	293.3629
1370048_at	116744	Edg2	endothelial differentiation, lysophospha	164.4135	473.616	321.7152
1384623_at	287785	NA	NA	164.4003	105.639	40.8683
1370093_at	60448	Htr1f	5-hydroxytryptamine (serotonin) recep	164.2322	22.85738	172.2604
1377679_at	305408	Gpr125_pr	G protein-coupled receptor 125 (predic	164.2233	1380.738	659.3336
1396835_at	498892	NA	NA	164.0549	59.29479	35.27818
1368540_at	83684	Tpbg	trophoblast glycoprotein	163.9388	248.9993	2324.988
1369041_at	116647	Nlgn1	neuroligin 1	163.8357	213.7547	1298.093
1387761_at	24687	Prp15	proline-rich protein 15	163.8345	30.79891	55.16325
1372915_at	500873	RGD15656	similar to hypothetical protein MGC141	163.6742	75.97618	38.48927
1394966_at	310812	RGD13095	similar to hypothetical protein FLJ2030	163.6725	2915.885	156.1546
1388243_at	117257	Gpr176	G protein-coupled receptor 176	163.4681	1044.184	1327.591
1380851_at	315996	Sema3f_pr	sema domain, immunoglobulin domain	163.4014	259.0617	160.7738
1369794_a_at	117276	Pfkfb3	6-phosphofructo-2-kinase/fructose-2,6	163.1972	320.5571	128.6154
1369232_at	65272	Kcnk10	potassium channel, subfamily K, meml	163.1712	116.5094	535.9185
1371315_at	289759	Myl7_predi	myosin, light polypeptide 7, regulatory	163.1265	115.8331	97.90101
1382863_at	311287	Tmem16c_	transmembrane protein 16C (predicted	162.6084	151.7106	66.72399
1367581_a_at	25353	Spp1	secreted phosphoprotein 1	162.6004	82.60827	599.5543
1396922_at	361086	Scel_predi	sciellin (predicted)	162.3418	26.63372	86.68348
1382047_at	301624	RGD13079	similar to AI325464 protein (predicted)	162.2735	348.1966	191.0671
1374726_at	308099	Fndc1	fibronectin type III domain containing 1	162.1711	94.57835	346.1072
1392525_at	25652	Ptgfr	prostaglandin F receptor	162.1529	158.4977	979.0289
1375381_at	289380	Nenf	neuron derived neurotrophic factor	162.071	37.04371	136.3755
1368535_at	117271	Bid3	BH3 interacting (with BCL2 family) dor	161.702	174.9344	246.1552
1382948_at	301709	RGD15598	similar to hypothetical protein E130310	161.5962	195.2898	248.0623
1380798_at	304005	Nfkbiz_pre	nuclear factor of kappa light polypeptic	161.5458	90.23067	43.41945
1391394_s_at	24629	Pdgfrb	platelet derived growth factor receptor,	161.4628	63.97582	138.4454
1388050_at	286914	LOC28691	putative pheromone receptor (Go-VN5	161.3645	35.97045	51.59896
1383397_at	362161	Rapsn_pre	receptor-associated protein of the syna	161.2286	223.1896	278.1958
1385656_at	499516	LOC49951	similar to hypothetical protein MGC130	161.1868	24.49706	123.9352

1367627_at	81660	Gatm	glycine amidinotransferase (L-arginine	161.1412	2074.623	684.0592
1376775_at	362171	RGD13099	similar to RIKEN cDNA 2600010E01	161.1111	92.19978	37.35275
1374540_at	311742	Cdca7	cell division cycle associated 7	160.9499	1417.711	76.33924
1368727_at	116726	Slc7a9	solute carrier family 7 (cationic amino a	160.941	35.79546	117.4833
1397718_at	308742	RGD15629	similar to multiple C2-domains with two	160.8769	34.12403	25.77388
1390961_at	502858	Gp9	glycoprotein 9	160.8435	49.93179	90.06834
1370778_at	298107	Mup5	major urinary protein 5	160.6966	26.56315	54.82033
1369306_at	25110	Klrd1	killer cell lectin-like receptor, subfamily	160.6412	67.29742	17.64232
1387179_at	29241	Adcy8	adenylate cyclase 8	160.6323	401.3195	198.0462
1393866_at	500592	LOC50059	NA	160.4957	151.2632	209.8846
1387509_at	29475	Dio3	deiodinase, iodothyronine, type III	160.2962	158.5532	139.6899
1386943_at	64364	Pllp	plasma membrane proteolipid	160.2813	1810.769	239.8263
1369870_at	29307	Admr	adrenomedullin receptor	160.189	132.3242	126.9797
1386656_at	298062	LOC29806	plasticity-related protein 3	159.5996	257.054	306.3902
1379386_at	300126	RGD15631	similar to B99 protein (predicted)	159.3945	2419.619	65.58524
1387847_at	85243	Pik3cb	phosphatidylinositol 3-kinase, catalytic	159.273	332.5392	63.03389
1396065_at	305842	Rnase6	ribonuclease, RNase A family, 6	159.1233	35.89482	165.0823
1383474_at	362418	Irak2	interleukin-1 receptor-associated kinas	159.0988	172.1426	451.2829
1383893_at	289407	Atp6v1g3_	ATPase, H transporting, lysosomal V1	158.9801	134.3532	52.95705
1384507_at	310190	LOC31019	similar to hypothetical protein FLJ1112	158.8351	353.7251	408.6986
1377666_at	290551	Chdh	choline dehydrogenase	158.7311	188.5094	180.2264
1397425_at	311911	Gpr21_pre	G protein-coupled receptor 21 (predicte	158.5092	35.22514	194.7077
1368688_at	64636	Ntsr2	neurotensin receptor 2	158.4548	9.937408	221.5928
1380991_at	286972	Ptar1	protein prenyltransferase alpha subuni	158.3064	159.8577	68.48025
1387413_at	114553	Ncf1	neutrophil cytosolic factor 1	157.9207	180.1857	40.09429
1387702_at	50577	Galr1	galanin receptor 1	157.8354	269.2348	196.6137
1379460_at	313413	RGD15649	similar to RIKEN cDNA 3110007P09 (p	157.818	480.8298	72.71257
1370215_at	29687	C1qb	complement component 1, q subcomp	157.7668	74.53437	70.45916
1374229_at	362828	RGD13085	similar to SPPL2b; presenilin-like prote	157.5905	657.8196	209.2471
1370140_a_at	83630	Pax4	paired box gene 4	157.5177	137.317	95.1315
1369507_at	497787	V1rb5_pred	vomeronasal V1r-type receptor V1rb5	157.5078	107.1133	50.56255
1370301_at	81686	Mmp2	matrix metallopeptidase 2	157.4953	136.6695	343.1457
1370470_at	286958	Np4	defensin NP-4 precursor	157.4751	65.07994	70.19291
1368620_at	83623	Spag4	sperm associated antigen 4	157.4731	193.0426	217.7321
1391279_at	298975	Scin	scinderin	157.3241	168.1927	486.0927
1385479_at	499759	RGD15655	similar to hypothetical protein 4932418	157.263	156.0909	69.38196
1392732_at	311336	Nusap1_pr	nucleolar and spindle associated prote	157.2538	11238.67	344.2862
1387632_at	64038	LOC64038	sertolin	157.2068	155.8431	170.1626
1387922_at	171547	Crispld2	cysteine-rich secretory protein LCCL d	156.9983	53.18288	60.91099
1386152_at	365564	Atoh7_pred	atonal homolog 7 (Drosophila) (predict	156.8495	202.7986	1130.037
1370564_at	25699	Dbh	dopamine beta hydroxylase	156.6838	54.87003	213.5911
1377962_at	296344	Mybl2_pred	myeloblastosis oncogene-like 2 (predic	156.6717	1558.664	152.6393
1369467_a_at	24638	Pfkfb1	6-phosphofructo-2-kinase/fructose-2,6-	156.4947	256.0899	138.308
1376327_at	366518	Tnfrsf14	tumor necrosis factor receptor superfa	156.4845	602.1095	15.57469
1369645_at	29256	Oprl1	opioid receptor-like 1	156.4256	25.72278	200.3147
1387537_at	360417	Ptprq	protein tyrosine phosphatase, receptor	156.3563	48.42857	28.59726
1367792_at	50585	Psp	parotid secretory protein	156.3168	91.35376	175.3098
1374046_at	293451	Hs3st2	heparan sulfate (glucosamine) 3-O-sul	156.3162	472.3136	214.3381
1387595_at	29319	Gif	gastric intrinsic factor	156.2649	206.5043	168.9802
1393202_a_at	312320	Igf2bp3	insulin-like growth factor 2, binding pro	156.0853	36.35217	25.05691
1381331_at	307616	Cklfsf1	chemokine-like factor super family 1	156.0664	100.9824	32.75514

1380554_at	288486	RGD15643	similar to hypothetical protein C530015	156.0451	366.7153	385.2032
1384918_at	362433	RGD13079	LOC362433 (predicted)	155.9873	178.1486	41.51183
1377610_at	296935	LOC29693	NA	155.9498	51.20371	171.5067
1383641_at	498937	RGD15594	RGD1559432 (predicted)	155.897	22.85622	316.5287
1390737_at	303824	RGD13056	similar to IGF-II mRNA-binding protein	155.7984	61.67433	104.2308
1369878_at	60451	Olr1654_pr	olfactory receptor 1654 (predicted)	155.7336	30.75992	139.3024
1398264_at	25362	Slc30a2	solute carrier family 30 (zinc transporte	155.6576	58.74191	93.78972
1374779_at	60327	F13a1	coagulation factor XIII, A1 subunit	155.4642	124.2098	67.6166
1369332_a_at	84556	Rims1	regulating synaptic membrane exocyto	155.2314	60.51233	102.2423
1381417_at	305066	Prox1_pred	prospero-related homeobox 1 (predicte	155.1727	148.687	116.7538
1387973_at	286904	Cyp4f4	cytochrome P450, family 4, subfamily f	155.1489	89.28558	135.4912
1377289_at	315748	Lbxcor1_pr	ladybird homeobox 1 homolog (Drosop	155.1242	836.4568	179.3315
1391983_at	500227	LOC50022	hypothetical gene supported by BC079	154.8858	90.42196	86.72645
1368394_at	89803	Sfrp4	secreted frizzled-related protein 4	154.8403	83.47051	229.364
1386023_at	252892	Lgi1	leucine-rich repeat LGI family, membe	154.7106	68.28163	40.43934
1382406_at	500690	RGD15621	similar to mixed-lineage protein kinase	154.5028	119.8501	70.49405
1387355_at	58968	Agc1	aggrecan 1	154.4362	292.3633	103.6432
1370636_at	246358	Cxcl7	chemokine (C-X-C motif) ligand 7	154.2322	158.1579	85.07981
1383460_at	360790	Upk3b_pre	uroplakin 3B (predicted)	154.1569	152.0564	153.9411
1381776_at	291991	Tmed6_pre	transmembrane emp24 protein transpo	153.9804	78.77466	90.79669
1378773_at	313663	Crocc_pred	ciliary rootlet coiled-coil, rootletin (pred	153.7064	66.16416	196.1779
1369949_at	78958	Bcam	basal cell adhesion molecule	153.5939	236.2602	1284.609
1384981_at	25752	Cd6	CD6 antigen	153.5105	253.8852	48.49541
1397490_at	305938	Spata13_p	spermatogenesis associated 13 (predi	153.4968	108.157	36.47211
1369725_at	56826	Centa2	centaurin, alpha 2	153.4105	575.1279	483.4332
1372652_at	302415	RGD15612	similar to forkhead protein AFXH (pred	153.3175	236.3079	396.9935
1368370_at	54223	Adcy4	adenylate cyclase 4	153.2777	217.9128	26.83321
1397499_at	24838	Tnnt3	troponin T3, skeletal, fast	153.2086	78.89585	146.8672
1372975_at	502541	RGD15599	RGD1559993 (predicted)	153.1368	252.9536	110.3368
1384760_at	361708	RGD15605	similar to Sorting nexin 6 (TRAF4-asso	153.0692	365.5096	230.4669
1393132_at	501097	RGD15602	similar to inhibitor of MyoD family-a (pr	153.0655	282.7337	120.779
1375983_at	309410	Mamdc2	MAM domain containing 2	153.0307	86.38023	84.53044
1368767_at	29679	Cst8	cystatin 8 (cystatin-related epididymal	152.9111	87.04778	69.26346
1391557_at	363632	Sox15_pre	SRY-box containing gene 15 (predicte	152.9019	96.93722	125.273
1380333_at	295338	Ptpn22_pre	protein tyrosine phosphatase, non-rece	152.7985	10.2118	32.08683
1383131_at	309684	Itgb2	integrin beta 2	152.745	274.8413	353.2547
1372700_at	297822	Trp53inp1	transformation related protein 53 induc	152.7031	35.28235	439.5754
1368148_at	24596	Ngfr	nerve growth factor receptor (TNFR su	152.59	1238.929	243.7224
1375908_at	300679	Eva1_pred	epithelial V-like antigen 1 (predicted)	152.4636	31.57045	98.22838
1368290_at	83476	Cyr61	cysteine rich protein 61	152.456	102.2775	195.986
1368974_at	66012	Gucy1a2	guanylate cyclase 1, soluble, alpha 2	152.3764	32.68708	108.8151
1384325_at	246238	Plunc	palate, lung, and nasal epithelium carc	152.3621	160.4791	151.2518
1371047_at	171148	Slc6a5	solute carrier family 6 (neurotransmitte	152.2448	57.93428	86.68157
1379813_at	498958	RGD15656	RGD1565689 (predicted)	152.2229	65.01208	171.5217
1383220_at	259243	Kcnip4	Kv channel interacting protein 4	152.2119	318.4276	442.0889
1387437_at	85273	Fbxo2	F-box only protein 2	152.039	762.1824	81.91902
1370466_at	124451	Lhx5	LIM homeobox protein 5	151.8569	181.3591	112.013
1369113_at	50566	Grem1	gremlin 1 homolog, cysteine knot supe	151.7876	58.59563	530.5294
1381251_at	363483	LOC36348	similar to RIKEN cDNA 4921520P21; I	151.5235	214.9045	248.5692
1377488_at	315220	Mapk8ip2	mitogen-activated protein kinase 8 inte	151.4022	52.98712	28.65868
1398185_at	306424	Mfap3l	microfibrillar-associated protein 3-like	151.3571	21.18636	242.3572

1386396_at	361679	Dusp8_pre	dual specificity phosphatase 8 (predict	151.1016	270.1752	1294.502
1370457_at	286916	Testin	testin gene	150.951	120.0248	19.23173
1387477_at	64119	Kcnk12	potassium channel, subfamily K, meml	150.9439	463.3369	425.1359
1387302_at	24167	Adcyap1r1	adenylate cyclase activating polypeptic	150.842	566.226	60.61976
1368322_at	25352	Sod3	superoxide dismutase 3, extracellular	150.7638	265.564	176.555
1390632_at	498997	RGD15632	similar to thrombospondin, type I, dom	150.4954	12.06412	260.936
1387643_at	170580	Fgf21	fibroblast growth factor 21	150.3736	227.7364	225.554
1375996_at	293507	1-Sep	septin 1	150.3264	260.6613	534.4615
1369100_at	171390	Nalp6	NACHT, leucine rich repeat and PYD c	150.3185	36.62711	17.96979
1381672_at	500564	RGD15634	similar to LDL receptor adaptor protein	150.2629	175.3065	20.12781
1378948_at	361104	RGD13061	similar to hypothetical protein FLJ9079	150.0362	195.3014	23.32658
1377108_at	315141	MGC94326	hypothetical LOC315141	149.9927	150.2249	158.3906
1368533_at	117240	Heph	hephaestin	149.9601	75.61973	24.31979
1391063_at	315740	Kif23_pred	kinesin family member 23 (predicted)	149.8519	1483.071	295.9103
1367780_at	64193	Pttg1	pituitary tumor-transforming 1	149.8195	11456.91	409.4119
1368371_at	84020	Kcnq1	potassium voltage-gated channel, subf	149.8006	774.7099	14.60214
1387731_at	79217	Gja3	gap junction membrane channel protei	149.7352	212.1491	70.5029
1395961_at	366274	Ntsr1_pred	neurotensin receptor 1 (predicted)	149.3307	21.17125	139.0114
1390984_at	362083	RGD15604	similar to Gene model 996 (predicted)	149.2618	30.33918	49.03025
1397613_at	363197	Tmem63b_	transmembrane protein 63b (predicted	149.1782	240.4795	148.2498
1382130_at	317183	Pcdh19_pr	protocadherin 19 (predicted)	149.1728	228.0519	50.21921
1390051_at	498136	RGD15625	similar to mKIAA0774 protein (predicte	149.1345	26.57154	477.72
1395428_at	313713	RGD15635	similar to novel protein (predicted)	149.1132	224.8092	88.75296
1386907_at	25438	Eno3	enolase 3, beta	149.0353	196.5981	580.2278
1385844_at	498056	RGD15647	RGD1564720 (predicted)	148.9624	167.9283	482.9787
1394462_at	287353	RGD13057	hypothetical LOC287353 (predicted)	148.9166	17.86973	75.55121
1390762_at	296293	Cbfa2t2_pr	core-binding factor, runt domain, alpha	148.9021	195.3288	314.6307
1389403_at	85272	Bmp7	bone morphogenetic protein 7	148.84	181.5412	521.1341
1398638_at	289777	Fignl1	fidgetin-like 1	148.8382	258.9565	222.503
1385203_at	501996	RGD15616	similar to IL25 (predicted)	148.815	46.0061	82.02395
1390618_at	500131	RGD15661	similar to cAMP responsive element bi	148.6305	13.68627	203.7604
1368714_at	24315	Dtprp	decidual/trophoblast prolactin-related p	148.4392	155.1983	81.01656
1368648_at	84683	Cox4i2	cytochrome c oxidase subunit IV isofor	148.3834	72.29523	56.58203
1370761_at	287000	Olr1361	olfactory receptor 1361	148.3554	199.947	99.53075
1367887_at	24530	Lcat	lecithin cholesterol acyltransferase	148.2813	95.13388	32.8033
1383200_at	308810	RGD13067	similar to hypothetical protein FLJ2351	148.2513	67.45733	21.2273
1385788_at	287989	Ephb3_pre	Eph receptor B3 (predicted)	148.1419	176.0313	353.5431
1368971_a_at	84018	Synj2	synaptojanin 2	148.1191	98.4828	143.7395
1369569_at	65140	Olr226	olfactory receptor 226	148.0942	87.09463	145.6209
1387635_at	84017	Hmga2	high mobility group AT-hook 2	148.0658	29.05016	58.55515
1388241_at	114215	Insl3	insulin-like 3	147.9596	110.2175	215.7474
1395431_at	78974	Six3	sine oculis homeobox homolog 3 (Dros	147.9584	895.3076	89.10247
1385718_at	300442	RGD13080	similar to RIKEN cDNA 2510048L02	147.9382	596.7817	379.2859
1375159_at	498724	NA	NA	147.8913	84.91126	287.2056
1369405_a_at	25103	Chrnb4	cholinergic receptor, nicotinic, beta pol	147.7927	112.3198	229.4172
1384999_at	499664	RGD15610	RGD1561089 (predicted)	147.7803	68.16298	82.13216
1386080_at	155437	Hey1	hairy/enhancer-of-split related with YR	147.7486	130.7979	918.3441
1371659_at	295342	Rhoc_pred	ras homolog gene family, member C (p	147.5554	427.504	381.0097
1397866_at	364705	Serpinb6b	serine (or cysteine) proteinase inhibito	147.4774	97.56405	89.85464
1385469_at	303477	Igf2bp1	insulin-like growth factor 2, binding pro	147.4449	18.6395	74.99492
1387672_at	25134	Gnmt	glycine N-methyltransferase	147.2624	76.06546	61.0227

1370796_at	246073	Foxi2	forkhead box I2	147.1009	33.21409	32.80358
1392182_at	171410	Acsbg1	acyl-CoA synthetase bubblegum family	147.099	33.67302	158.7941
1374374_x_at	287285	RGD1308C	similar to KIAA1960 protein (predicted)	147	49.19904	187.3588
1378449_at	432393	Kprp	keratinocytes proline-rich protein	146.9847	168.0233	121.3129
1368264_at	117265	Pex6	peroxisomal biogenesis factor 6	146.9283	348.165	162.4206
1393426_at	307401	Pde6a_pre	phosphodiesterase 6A, cGMP-specific	146.8942	19.22721	99.50421
1379539_at	363036	NA	NA	146.7595	116.9361	136.8705
1388172_at	192273	Slc22a9	solute carrier family 22 (organic anion/	146.7441	88.58183	93.8018
1379363_at	312272	Mgam_pre	maltase-glucoamylase (predicted)	146.7358	377.5629	225.9579
1379596_at	306168	Sox21_pre	SRY-box containing gene 21 (predicte	146.609	25.02844	66.18947
1368791_at	24613	Oprd1	opioid receptor, delta 1	146.4144	207.7641	75.73785
1387461_at	25601	Oprm1	opioid receptor, mu 1	146.403	124.8122	185.2483
1388227_at	25119	Hlals_map	MHC class I-like sequence (mapped)	146.3742	16.03061	90.52427
1394607_at	308003	Rab11fip2_	RAB11 family interacting protein 2 (cla	146.179	37.70048	126.6338
1375567_at	361363	RGD13086	similar to hypothetical protein LOC929	146.0869	141.1968	97.0705
1397630_at	316395	LOC31639	NA	145.927	298.7319	20.7553
1396547_at	362477	LOC36247	NA	145.8871	22.77711	109.0942
1378901_at	499496	NA	NA	145.8401	277.3261	122.7056
1384877_at	286758	Aqp11	aquaporin 11	145.8361	371.9415	205.279
1398365_at	291966	RGD1305C	similar to RIKEN cDNA 2700055K07	145.7574	281.6902	791.7321
1368829_at	83727	Fbn1	fibrillin 1	145.7256	51.46135	1138.699
1388416_at	299858	Lrp1	low density lipoprotein receptor-relatec	145.7157	552.6723	374.0879
1372579_at	497010	Eng	NA	145.6852	39.29748	114.7024
1370629_at	293152	Art2b	ADP-ribosyltransferase 2b	145.6821	98.57234	23.63232
1387161_at	84012	Slc1a6	solute carrier family 1 (high affinity asp	145.6484	14.86605	18.42171
1388293_at	363925	Mlc2	myosin regulatory light chain 2, ventric	145.4856	19.91663	38.48352
1390694_at	301242	Srf_predict	serum response factor (predicted)	145.4758	148.6369	427.6989
1378410_at	361769	Sufu	suppressor of fused	145.4426	307.4429	502.3498
1368469_at	25241	Aqp5	aquaporin 5	145.423	178.6298	14.11976
1384929_at	499264	RGD15651	similar to apolipoprotein B48 receptor (	145.3414	47.56245	235.3662
1370622_at	25635	Mc4r	melanocortin 4 receptor	145.3238	50.77699	70.96991
1384905_at	360751	NA	NA	145.2829	51.71782	91.69027
1373931_at	312711	RGD13049	similar to RIKEN cDNA C530028O21 (	145.1396	389.9126	454.3165
1395754_at	310418	Frem2_pre	Fras1 related extracellular matrix prote	145.0356	22.85938	43.73792
1371129_at	299247	Cpg1	candidate plasticity gene 1	145.0086	130.6276	401.2457
1393978_at	288070	Stfa2_pred	stefin A2 (predicted)	144.9324	17.21998	15.76644
1387980_at	246147	Ctsq	cathepsin Q	144.8188	31.02325	132.288
1368683_at	140914	Oldlr1	oxidized low density lipoprotein (lectin-	144.7095	9.413355	188.9864
1378698_at	29605	Myh13	myosin, heavy polypeptide 13, skeleta	144.6209	6.45983	91.9087
1387994_at	286964	Hsd17b9	hydroxysteroid (17-beta) dehydrogena	144.5301	107.4425	31.07848
1384811_at	363667	Hlf	hepatic leukemia factor	144.4601	132.9379	40.94974
1369211_at	56827	Cacna1i	calcium channel, voltage-dependent, a	144.3897	100.6112	61.49769
1398820_at	117558	Mylk2	myosin light chain kinase 2, skeletal m	144.292	389.09	422.3107
1393339_at	315179	RGD1309C	similar to RIKEN cDNA 4931407K02 (p	143.9846	20.79125	36.15422
1369370_s_at	66026	Trpv4	transient receptor potential cation char	143.9073	8.797055	14.86813
1384625_at	366244	RGD13062	similar to RIKEN cDNA 2410116G06 (	143.8756	19.18811	15.20667
1370741_at	294158	Olr1696	olfactory receptor 1696	143.8591	148.2403	180.1144
1385914_at	498918	LOC49891	iroquois homeobox protein 5	143.8182	14.38226	167.2282
1380962_at	302668	Ace2	angiotensin I converting enzyme (pept	143.8177	100.1007	9001.75
1380183_at	408247	Try10	trypsin 10	143.7485	41.82838	425.5207
1393926_at	305269	RGD13115	similar to stem cell adaptor protein ST/	143.6675	19.66853	17.9507

1369062_at	54266	Madcam1	mucosal vascular addressin cell adhes	143.5415	145.7787	37.91017
1369149_at	65172	Limk1	LIM motif-containing protein kinase 1	143.3268	252.9176	94.21135
1368369_at	25516	Pnoc	prepronociceptin	143.3163	363.5689	77.92492
1369807_at	81509	Bdkrb1	bradykinin receptor B1	143.1101	122.7417	33.05058
1373654_at	306283	Anxa8	annexin A8	143.0434	155.3725	49.81656
1394587_at	29203	Foxd3	forkhead box D3	143.0234	46.00416	88.9
1370154_at	25211	Lyz	lysozyme	142.8959	22.41659	1217.07
1378327_at	309430	Dmrt2_pre	doublesex and mab-3 related transcrip	142.8953	37.13346	84.39045
1385912_at	502685	NA	NA	142.6203	474.4976	93.48198
1382848_at	25098	Foxa1	forkhead box A1	142.522	483.4623	595.018
1387308_at	24335	Epo	erythropoietin	142.518	94.41751	66.46764
1384175_at	310643	Efna4_pre	ephrin A4 (predicted)	142.2844	26.45363	55.60072
1368969_at	80722	Sost	sclerostin	142.2832	52.53844	16.89544
1375937_a_at	307805	RGD13093	similar to RIKEN cDNA G630055P03 g	142.2748	884.2864	27.26394
1368605_at	114203	Aps	adaptor protein with pleckstrin homolog	142.1882	318.5145	140.9698
1391049_at	306720	Ctsm	cathepsin M	141.8941	19.57485	176.7435
1391911_at	500422	NA	NA	141.6559	275.1261	130.6966
1397233_at	316023	Dcamkl3_p	doublecortin and CaM kinase-like 3 (pr	141.6406	210.9998	1378.819
1368522_at	83508	Timeless	timeless homolog (Drosophila)	141.6237	816.0958	112.5359
1385574_at	361253	RGD13081	similar to cDNA sequence AF397014	141.5209	134.0592	374.3441
1387403_at	54297	Rgs8	regulator of G-protein signaling 8	141.5133	120.016	68.8198
1370016_at	81734	Nell2	nel-like 2 homolog (chicken)	141.4975	641.9328	424.3116
1380196_at	361016	LOC36101	similar to RIKEN cDNA 4933406L09	141.4029	77.70736	26.70314
1369836_at	56824	Ifit1	interferon-induced protein with tetratric	141.3922	249.943	227.4128
1369253_at	114107	Kremen1	kringle containing transmembrane prot	141.3614	26.47084	100.0121
1384564_at	499520	RGD15646	similar to hypothetical protein MGC524	141.3014	57.08788	138.5249
1389058_at	300656	Trim29_pre	tripartite motif protein 29 (predicted)	141.1531	108.1784	78.47097
1388166_at	299357	RGD13592	similar to immunoglobulin heavy chain	141.022	124.1125	141.551
1375010_at	287435	Cd68	CD68 antigen	140.902	153.0671	183.5002
1385361_at	365266	Atp10a	ATPase, class V, type 10A	140.8669	64.54116	217.2755
1391780_at	313019	RGD13089	similar to hypothetical protein MGC164	140.8313	140.8752	59.27441
1370037_at	83728	Fbn2	fibrillin 2	140.7728	107.7982	43.7654
1384949_at	363229	Slc39a10_	solute carrier family 39 (zinc transporte	140.6945	49.70473	108.9407
1369730_a_at	66023	Gfra4	glial cell line derived neurotrophic facto	140.623	234.6376	175.2436
1369224_at	117048	Cdh17	cadherin 17	140.5733	161.0198	54.26241
1388698_at	116662	Ecm1	extracellular matrix protein 1	140.5386	123.9176	218.9194
1397320_at	306104	RGD13050	similar to Progesterone-induced blocki	140.4918	89.46994	130.5257
1398067_at	497798	RGD15613	similar to LIM domain only 3 (predicted	140.2452	121.7702	48.77723
1376233_at	286923	Dlgap3	discs, large (Drosophila) homolog-asso	140.127	154.1931	212.2011
1368556_at	94106	Vegp2	von Ebners gland protein 2	140.0071	147.8466	46.56565
1380968_at	497926	RGD15646	similar to novel protein (predicted)	140.0052	108.3901	6.602048
1371202_a_at	29227	Nfib	nuclear factor I/B	139.7736	203.4626	657.7399
1394220_at	316012	Klhl18_pre	kelch-like 18 (Drosophila) (predicted)	139.6426	152.5831	37.26804
1380422_at	304297	RGD15606	similar to sidekick 1 (predicted)	139.6139	33.59111	292.7924
1370592_at	171179	Keg1	kidney expressed gene 1	139.3936	43.41089	37.31968
1398234_at	499612	NA	NA	139.1791	32.55929	42.09833
1376648_at	298894	Mycn_map	v-myc myelocytomatosis viral related o	139.1478	1085.671	1437.83
1387157_at	171414	Pmfbp1	polyamine modulated factor 1 binding	139.1233	111.0325	56.17698
1380123_at	310626	RGD13098	similar to RIKEN cDNA 2310042N02	139.0959	123.9674	161.906
1370369_at	29252	Gzmm	granzyme M (lymphocyte met-ase 1)	139.0078	157.3483	129.6544
1368785_a_at	54284	Pitx2	paired-like homeodomain transcription	138.8196	22.87147	87.60652

1369512_at	84468	Chst3	carbohydrate (chondroitin 6/keratan) s	138.8156	29.29131	57.41394
1382274_at	310486	Rarres1	retinoic acid receptor responder (tazar	138.8126	55.25083	219.5464
1379134_at	311882	RGD1565C	similar to beta-1,3-N-acetylglucosamin	138.8103	65.88405	49.89067
1368468_at	29680	Cyp11a1	cytochrome P450, family 11, subfamily	138.7774	190.4531	22.40643
1388014_at	192267	Obp1f	odorant binding protein I f	138.7584	135.8197	245.4248
1396271_at	316304	Gluld1	glutamate-ammonia ligase (glutamine	138.7014	95.3221	49.79814
1384855_at	498278	RGD15626	similar to RIKEN cDNA 1700009P17 (p	138.5618	156.8859	367.3062
1373006_at	293634	Prp2	proline-rich protein PRP2	138.5459	119.326	67.62187
1384976_at	313596	Tcfap2e_pr	transcription factor AP-2, epsilon (pred	138.4279	129.8428	210.4312
1382356_at	302507	Efhc2_prec	EF-hand domain (C-terminal) containir	138.4131	124.3374	255.6007
1384695_at	315190	Upk3a_pre	uroplakin 3A (predicted)	138.2799	55.39928	136.2501
1381557_at	309242	Gna14	guanine nucleotide binding protein, alp	138.2104	451.9192	214.6706
1376623_at	287129	LOC28712	claudin-like protein 24	138.1446	37.25348	288.6726
1369481_at	89814	Tnfsf4	tumor necrosis factor (ligand) superfan	138.1022	83.2438	79.04976
1387314_at	64305	Sult1b1	sulfotransferase family 1B, member 1	138.0565	31.66779	9.13936
1391095_at	304608	Mmp19_pr	matrix metalloproteinase 19 (predicted	138.0374	19.15604	72.54542
1370673_at	246264	Znf382	zinc finger protein 382	138.0265	158.3073	42.3674
1369566_at	78979	Tas2r1	taste receptor, type 2, member 1	138.0228	51.51191	101.7217
1382828_at	499103	NA	NA	137.8795	110.8591	225.3424
1376270_at	500694	RGD15632	similar to HESB like domain containing	137.8787	67.0032	150.7804
1370047_at	85496	Enpp1	ectonucleotide pyrophosphatase/phosp	137.7581	454.3374	237.172
1391806_at	498793	LOC49879	similar to inter-alpha-inhibitor H2 chain	137.7086	28.72272	51.9776
1369350_at	170848	Kcnq2	potassium voltage-gated channel, subf	137.6272	159.9009	93.87379
1380317_at	29181	Cdh10	cadherin 10	137.605	103.1745	21.27542
1387539_at	497756	Si	Sucrase-isomaltase	137.598	76.24171	20.41528
1387239_a_at	29512	Padi4	peptidyl arginine deiminase, type IV	137.598	28.19569	86.33205
1389515_at	306353	Rfxank	regulatory factor X-associated ankyrin-	137.3699	41.33223	201.188
1378252_at	288289	Chodl_prec	chondrolectin (predicted)	137.3245	94.59328	281.0593
1368816_at	24564	Mpz	myelin protein zero	137.3236	50.80388	140.3582
1392714_at	304689	Serpinb11_	serine (or cysteine) peptidase inhibitor	137.2286	65.75847	96.6632
1384836_at	307100	Calml3	calmodulin-like 3	137.1746	34.04294	83.76797
1381130_at	296178	Mcm8_prec	minichromosome maintenance deficier	137.0952	745.1015	257.2388
1394720_at	313507	Tal1_predi	T-cell acute lymphocytic leukemia 1 (p	136.9532	235.0014	13.39133
1387298_at	60372	Pga5	pepsinogen 5, group I (pepsinogen A)	136.94	39.4085	81.16411
1370977_at	50545	Neud4	neuronal d4 domain family member	136.9376	141.0024	110.969
1379726_at	361131	Edg4_pred	endothelial differentiation, lysophospha	136.9307	234.3417	89.8144
1368733_at	25355	Ste	sulfotransferase, estrogen preferring	136.9168	57.30503	86.79842
1390756_at	498667	RGD15594	similar to expressed sequence AW121	136.8809	249.2766	48.86113
1377122_at	81529	Pde1a	phosphodiesterase 1A, calmodulin-dep	136.6765	128.2768	150.6871
1384796_at	361060	Ebf2_predi	early B-cell factor 2 (predicted)	136.6258	9.759508	28.29237
1386361_at	664710	Teddm1	NA	136.5336	41.06624	62.00981
1379392_at	290880	Adprhl1	ADP-ribosylhydrolase like 1	136.5148	254.7389	115.82
1382182_at	300264	Hoxc9_pre	homeo box C9 (predicted)	136.4596	252.0056	21.7213
1371895_at	287701	Krt1-14	keratin complex 1, acidic, gene 14	136.4106	22.04483	53.27472
1393755_at	366508	Pik3cd_pre	phosphatidylinositol 3-kinase catalytic	136.3408	29.93832	262.2811
1370702_at	192258	Gabrr3	gamma-aminobutyric acid (GABA) rec	136.2299	96.82707	114.6906
1375069_at	362983	RGD1308C	similar to RIKEN cDNA A630054L15; h	135.9987	92.40776	203.4231
1368759_at	84347	Cacng2	calcium channel, voltage-dependent, g	135.9679	371.0718	133.2249
1379921_at	308779	Fsd2_pred	fibronectin type III and SPRY domain c	135.9163	29.52368	105.0448
1390190_at	317316	Plac1	placenta-specific 1	135.916	91.88825	128.4927
1370927_at	25683	Col12a1	procollagen, type XII, alpha 1	135.7982	50.83082	192.7025

1368377_at	171290	Gzmc	granzyme C	135.7858	132.5839	269.3114
1368590_at	65205	Mmp16	matrix metalloproteinase 16	135.781	410.734	351.2647
1386974_at	171434	Phldb1	pleckstrin homology-like domain, famil	135.7634	164.2857	633.3902
1387754_at	25007	Tacr2	tachykinin receptor 2	135.7372	28.87464	11.52791
1369881_at	54256	Gja6	gap junction membrane channel protei	135.6972	100.4461	193.5424
1396146_at	313490	RGD1310C	similar to NAG-5 protein (predicted)	135.6426	358.0231	64.33513
1369129_at	29434	Rasgrp1	RAS guanyl releasing protein 1	135.6161	66.88844	18.52256
1389739_at	311633	Neurl2_pre	neuralized-like 2 (Drosophila) (predicte	135.5485	102.1871	112.2453
1390828_at	29358	Npy1r	neuropeptide Y receptor Y1	135.5406	233.7177	336.9595
1373723_at	300027	RGD15642	similar to high density lipoprotein-bindi	135.5389	30.41421	221.9538
1369591_at	29188	Csn10	casein kappa	135.5049	103.1811	45.14789
1371094_at	117555	Lhx2	LIM homeobox protein 2	135.4448	557.3277	69.12323
1379204_at	691764	LOC69176	NA	135.324	44.22045	69.77843
1369430_at	114106	Bcmo1	beta-carotene 15,15'-monooxygenase	135.2422	103.4131	231.58
1369697_at	29385	Il8rb	interleukin 8 receptor, beta	135.2275	51.74479	119.1818
1372640_at	499410	NA	NA	135.0781	244.5792	763.9828
1369226_at	24903	Kng1	kininogen 1	134.9005	68.9895	8.664014
1383846_at	502694	RGD15628	similar to Docking protein 5 (Downstre	134.8841	46.89427	414.0349
1382687_at	314221	Six6_predi	sine oculis-related homeobox 6 homol	134.5112	86.76031	89.31641
1368155_at	25011	Cyp2c40	cytochrome P450, family 2, subfamily	134.233	30.8477	83.45013
1392820_at	25317	Fgf1	fibroblast growth factor 1	134.0773	55.21952	185.9508
1387037_at	80848	Cubn	cubilin (intrinsic factor-cobalamin rece	133.908	14.6805	225.9229
1390238_at	308015	Clca3_prec	chloride channel calcium activated 3 (	133.8689	76.69542	205.7411
1395915_at	366783	RGD13046	similar to CG17807-PA (predicted)	133.6686	101.6773	38.69182
1388932_at	140433	Lama5	laminin, alpha 5	133.6321	505.3131	697.0804
1368527_at	29527	Ptgs2	prostaglandin-endoperoxide synthase	133.6149	94.12174	2941.575
1390766_at	500377	LOC50037	similar to Tubulin alpha-8 chain (Alpha	133.4464	112.7839	132.6158
1371264_at	502359	Hbe2_pred	hemoglobin, epsilon 2 (predicted)	133.3022	21.0813	116.2144
1382590_at	501095	RGD15633	similar to RIKEN cDNA 2310015N21 (	133.2864	171.7016	584.901
1397177_at	29162	Cdh7	cadherin 7, type 2	133.2746	345.4825	41.08884
1371147_at	299276	Serpina3m	serine (or cysteine) proteinase inhibito	133.2304	100.9598	87.01106
1376485_at	367167	Traip_pred	TRAF-interacting protein (predicted)	133.1038	761.6324	155.8886
1385791_at	301057	Vill_predict	villin-like (predicted)	133.0729	77.39509	263.2119
1370092_at	25153	Mas1	MAS1 oncogene	132.952	60.08251	120.9274
1388018_at	25544	Sele	selectin, endothelial cell	132.9312	4.747769	10.87583
1389768_at	301266	Gpr110_pr	G protein-coupled receptor 110 (predic	132.7018	132.5231	60.56264
1397167_at	500902	RGD15653	similar to RIKEN cDNA 2210421G13 (	132.6609	65.78077	82.94914
1369179_a_at	25664	Pparg	peroxisome proliferator activated rece	132.5896	67.43094	93.28072
1388924_at	362850	Angptl4	angiopoietin-like 4	132.5439	14.37445	56.77538
1367862_at	83521	Rrad	Ras-related associated with diabetes	132.5203	6.522641	76.79362
1371043_a_at	192109	Pou3f3	POU domain, class 3, transcription fac	132.3866	179.4418	23.47907
1384824_at	295027	Pcdh18_pr	protocadherin 18 (predicted)	132.274	40.22844	290.343
1370211_at	64356	Nrgn	neurogranin	132.1822	400.3408	53.66611
1388060_at	191595	Syt12	synaptotagmin XII	132.0344	574.263	85.78475
1390312_at	500012	RGD15614	similar to mKIAA2005 protein (predicte	131.8997	136.471	479.4035
1392693_at	295714	RGD13112	similar to fatty acid desaturase 2; linol	131.8961	103.4948	17.28643
1369088_at	29496	Erbb3	v-erb-b2 erythroblastic leukemia viral	131.8917	67.76314	59.71982
1393863_at	294706	Cd180_pre	CD180 antigen (predicted)	131.8375	45.12361	12.41378
1389071_at	309169	LOC30916	tangerin	131.7995	38.4436	294.5969
1370459_at	246323	Aard	alanine and arginine rich domain conta	131.3641	83.37319	51.51463
1391963_at	29737	Kcnab1	potassium voltage-gated channel, sha	131.3376	131.3055	32.88164

1384077_at	498913	RGD15621	RGD1562170 (predicted)	131.2809	147.7669	167.6996
1374237_at	304816	Lmod1_pre	leiomodin 1 (smooth muscle) (predicte	131.1529	50.43481	173.793
1370516_at	246239	Slc15a3	solute carrier family 15, member 3	131.0767	9.400844	13.29331
1369648_at	25029	Calcrl	calcitonin receptor-like	131.0763	155.8929	118.3913
1378723_at	502499	NA	NA	131.0581	559.7336	37.72444
1387644_at	64022	Btc	betacellulin	130.927	182.1572	31.30748
1385583_at	308800	Tyr_mappe	tyrosinase (albino coat color) (mapped	130.8384	79.43872	73.29063
1370749_at	286892	V1rb7	vomeronasal 1 receptor, B7	130.8298	27.12288	24.93586
1380866_at	295545	LOC29554	NA	130.8255	23.54915	462.6679
1388178_at	84584	Ncoa3	nuclear receptor coactivator 3	130.8245	325.4364	60.51749
1398169_at	81811	Thpo	thrombopoietin	130.7764	63.23078	16.62533
1392411_at	300846	Tinag	tubulointerstitial nephritis antigen	130.5174	15.73769	121.7332
1369751_at	25570	Trhr	thyrotropin releasing hormone recepto	130.4807	114.4196	63.1133
1382601_at	498638	RGD15643	similar to scavenger receptor type A S	130.3937	58.12936	12.56458
1396655_at	116560	Kcnj9	potassium inwardly-rectifying channel,	130.366	89.9208	116.8631
1369279_at	170635	Dhrs9	dehydrogenase/reductase (SDR family	130.3341	42.76475	185.0521
1394505_at	362398	RGD15608	similar to plexin 1 (predicted)	130.1817	82.91396	126.2614
1369340_at	56029	Snip	SNAP25-interacting protein	130.1786	148.0191	243.9931
1370117_at	29349	Fgf8	fibroblast growth factor 8	130.1493	42.443	40.54376
1378623_at	499263	RGD15622	similar to KTSR5831 (predicted)	130.1342	124.5625	84.00452
1387768_at	59108	Mb	myoglobin	130.0812	85.59836	77.79996
1380519_at	301378	RGD15614	similar to RIKEN cDNA 4832428D23 g	130.0714	42.99698	78.47828
1383684_at	304648	Asf1b_prec	ASF1 anti-silencing function 1 homolog	129.9808	886.4086	125.3794
1387609_at	54233	Ca5a	carbonic anhydrase 5a, mitochondrial	129.9752	15.73228	67.69265
1384881_at	288045	Zdhhc19	zinc finger, DHHC domain containing 1	129.9295	69.66658	71.23793
1370432_at	192110	Pou3f1	POU domain, class 3, transcription fac	129.754	96.27313	122.2323
1383268_at	364204	RGD13089	similar to cDNA sequence BC027127;	129.6979	18.50375	47.28768
1387010_s_at	29686	Scn1b	sodium channel, voltage-gated, type I,	129.5397	254.6299	194.9196
1378376_at	83835	Chic2_prec	cysteine-rich hydrophobic domain 2 (p	129.4416	274.2749	23.09808
1388914_at	303250	Ybx2_pred	Y box protein 2 (predicted)	129.432	556.9944	62.89691
1369389_at	170955	Zfp483	zinc finger protein 483	129.401	20.97149	61.61519
1385629_at	296608	RGD13062	similar to hypothetical protein MGC297	129.4009	52.06172	76.56046
1385770_at	363167	RGD15657	similar to hypothetical protein A730098	129.1941	61.49435	32.52135
1398221_at	29643	Sv2c	synaptic vesicle glycoprotein 2c	129.1157	161.7859	295.503
1382997_at	316062	Slc22a13_	solute carrier family 22 (organic cation	129.0154	170.4511	49.27735
1383875_at	303924	Upk1b	uroplakin 1B	128.9446	31.04919	125.9894
1382912_at	314746	Usp44_pre	ubiquitin specific protease 44 (predicte	128.8946	21.91959	47.96606
1385927_at	24459	Hoxc4_ma	homeo box C4 (mapped)	128.8686	133.9434	76.27659
1370698_at	286954	Udpgtr2	liver UDP-glucuronosyltransferase, ph	128.7973	125.4811	255.6863
1395169_at	288661	Zcchc8_pre	zinc finger, CCHC domain containing 8	128.7643	422.6338	231.5693
1395879_at	298186	RGD13057	similar to hypothetical protein FLJ3386	128.7347	68.52559	31.07918
1381856_at	501962	NA	NA	128.6716	200.1327	248.4399
1374099_at	307982	Nrap_predi	nebulin-related anchoring protein (pred	128.5822	138.808	63.628
1371364_a_at	25030	Andpro	androgen regulated 20 kDa protein	128.5609	3.210395	20.92924
1385325_at	298936	Sntg2_prec	syntrophin, gamma 2 (predicted)	128.5576	13.62334	66.10689
1382328_at	312312	Gimap1	GTPase, IMAP family member 1	128.4718	26.02521	81.4557
1386936_at	117130	Grifin	galectin-related inter-fiber protein	128.3963	97.56017	213.4211
1396867_at	266997	Rln3	relaxin 3	128.372	67.29965	36.41264
1379065_at	191570	Serpina12	serine (or cysteine) peptidase inhibitor	128.2284	54.26204	38.89617
1379746_at	304530	JIK	JNK/SAPK-inhibitory kinase	128.1816	392.8096	906.3157
1397081_at	287988	Polr2h_pre	polymerase (RNA) II (DNA directed) po	128.0077	261.9816	42.61614

1391314_at	503247	NA	NA	127.9313	54.31133	232.8207
1390123_at	315524	Tmem45b	transmembrane protein 45b	127.8747	20.29919	126.533
1387307_at	29301	Hal	histidine ammonia lyase	127.817	58.19241	41.01559
1392467_at	282636	Impa2	inositol (myo)-1(or 4)-monophosphatas	127.7907	674.5226	278.5611
1368749_at	117023	Kcns1	K+ voltage-gated channel, subfamily S	127.7286	59.76301	206.2933
1370892_at	24233	C4a	complement component 4a	127.5865	175.1352	510.8761
1380757_at	85421	Prkcm	protein kinase C, mu	127.4673	12.96504	68.51797
1378906_at	499204	RGD1559€	similar to hypothetical protein FLJ2541	127.4517	1042.122	271.8747
1369840_at	171397	UST4r	integral membrane transport protein U	127.4281	57.45271	204.5372
1385837_at	288152	Hoxd3_ma	homeo box D3 (mapped)	127.376	45.65065	161.3915
1382554_at	298288	C8a_predic	complement component 8, alpha polyp	127.3466	32.59655	51.70877
1389807_at	287935	RGD1308C	hypothetical LOC287935 (predicted)	127.175	74.77733	46.11746
1388139_at	360543	Myh4	myosin, heavy polypeptide 4, skeletal r	127.0284	126.1991	298.2737
1391260_at	366962	Grap2	GRB2-related adaptor protein 2	127.0134	102.6171	117.9993
1396455_at	365227	Cox6b_pre	cytochrome c oxidase, subunit VIb (pre	126.98	6.906151	84.22926
1371708_at	298657	Car6	carbonic anhydrase 6	126.5495	29.24031	116.824
1369706_at	29658	Cacng1	calcium channel, voltage-dependent, g	126.5324	57.10497	69.36612
1369542_at	192648	Prokr1	prokineticin receptor 1	126.4707	64.61547	82.04261
1371057_at	29707	Gabra5	gamma-aminobutyric acid (GABA-A) re	126.2938	25.03031	239.9049
1369151_at	114587	Dlk1	delta-like 1 homolog (Drosophila)	126.2304	30.37875	25.79925
1388091_at	405281	Olr1500	olfactory receptor 1500	126.2135	79.53427	21.17805
1385502_at	308901	Trim21_pre	tripartite motif protein 21 (predicted)	126.0892	257.0495	72.87069
1379177_at	303384	Mmp28_pr	matrix metallopeptidase 28 (epilysin) (p	126.0049	23.30634	16.59921
1375731_at	315615	Dscaml1_p	Down syndrome cell adhesion molecul	125.8548	36.99153	154.1232
1372158_at	246233	Lrp16	LRP16 protein	125.8295	1308.64	536.3334
1376567_at	304323	Ftsj2_predi	FtsJ homolog 2 (E. coli) (predicted)	125.7637	918.1424	601.1049
1372111_at	25404	Cav	caveolin	125.7022	20.23623	398.0215
1374558_at	499415	RGD15627	similar to B7-like protein GL50-B (pred	125.662	696.6701	23.111
1371282_at	442921	Prm3	NA	125.65	84.42211	55.065
1388008_at	252859	Trhr2	thyrotropin releasing hormone recepto	125.5667	53.97447	84.82448
1368846_at	79011	Camkv	CaM kinase-like vesicle-associated	125.5411	200.2789	251.1802
1369529_at	25610	Csf3	colony stimulating factor 3 (granulocyte	125.4589	71.30606	102.8766
1369604_at	25443	Fgf10	fibroblast growth factor 10	125.3677	220.0455	30.57924
1393929_at	308163	Ccr6	chemokine (C-C motif) receptor 6	125.281	13.1068	195.0988
1385787_at	308900	rnf141	ring finger protein 141	125.2326	533.5144	230.1327
1375057_at	311210	Tspan18_p	tetraspanin 18 (predicted)	125.2223	156.4093	38.12203
1387972_at	171554	Mucdhl	mucin and cadherin like	125.1508	235.4407	204.3345
1374367_at	498355	RGD1564€	similar to CG12206-PA, isoform A (pre	125.1415	65.56196	145.3138
1387211_at	117232	Barhl1	BarH-like 1 (Drosophila)	125.0657	532.7585	20.00031
1397737_at	499846	RGD15623	similar to RIKEN cDNA C130096D04 g	125.0571	72.18544	178.9799
1368768_at	29701	Scn11a	sodium channel, voltage-gated, type X	125.016	60.50194	225.4477
1377452_at	316099	Clec3b_pre	C-type lectin domain family 3, member	124.9823	201.2102	132.6162
1386664_at	312664	Rasgef1a_	RasGEF domain family, member 1A (p	124.8575	94.7401	176.8366
1370608_at	365909	LOC36590	similar to Heme oxygenase 3 (HO-3)	124.8073	140.2311	138.6642
1389026_at	293117	Ankrd42_p	ankyrin repeat domain 42 (predicted)	124.7875	420.8348	974.8991
1385213_at	498547	RGD15632	similar to epithelial stromal interaction	124.773	12.99118	121.9242
1381502_at	363093	Mns1	meiosis-specific nuclear structural prot	124.7286	266.0213	327.8726
1394174_at	613224	Defa8	defensin alpha 8	124.5333	139.4991	201.3411
1388264_at	59306	Spnb1	short form of beta II spectrin	124.4703	270.3353	26.55196
1368690_a_at	24417	Grm4	glutamate receptor, metabotropic 4	124.293	420.653	175.2445
1393846_at	361018	RGD13063	similar to downregulated in renal cell c	124.2591	91.12146	44.82966

1370114_a_at	25513	Pik3r1	phosphatidylinositol 3-kinase, regulato	124.2241	258.0735	46.06248
1369339_at	25311	Dcc	deleted in colorectal carcinoma	124.0788	55.04179	37.61962
1387628_at	78981	Tas2r10	taste receptor, type 2, member 10	124.0161	91.19339	85.45698
1398398_at	368057	RGD15646	similar to Homeobox protein A10 (prec	123.8671	1257.058	51.7528
1377988_at	362974	RGD13046	similar to CG9646-PA	123.7613	99.30579	15.24574
1370412_at	171409	Tnnt1	troponin T1, skeletal, slow	123.7363	104.5121	118.8193
1390568_at	287592	Tubd1_pre	tubulin, delta 1 (predicted)	123.6977	58.57587	34.36909
1382932_at	361419	Lrrc50	leucine rich repeat containing 50	123.6142	40.25995	93.35157
1370732_at	297439	V1ra12_pre	vomeronasal 1 receptor, a12 (predicte	123.4605	54.12945	62.56153
1387082_at	83928	Fetub	fetuin beta	123.404	45.25431	67.422
1392041_at	301230	RGD15659	RGD1565959 (predicted)	123.3372	29.03902	11.74882
1368615_a_at	60422	Slc18a3	solute carrier family 18 (vesicular acety	123.3049	28.62448	110.1846
1392981_at	306655	Irx4_predic	Iroquois related homeobox 4 (Drosoph	123.1785	94.80049	260.8937
1394117_at	311186	Ddb2_pred	damage specific DNA binding protein 2	123.1662	280.8572	57.76498
1376942_at	308663	Tubgcp5_p	tubulin, gamma complex associated pr	123.1563	257.7978	192.6078
1379344_at	66021	Cybb	cytochrome b-245, beta polypeptide	123.1486	110.7104	93.49925
1378047_at	314711	Spic_predi	Spi-C transcription factor (Spi-1/PU.1 r	123.0909	23.93586	109.9383
1368800_at	84349	Cd40lg	CD40 ligand	122.9376	123.0755	86.59448
1370115_at	114518	Slc7a10	solute carrier family 7 (cationic amino a	122.7594	100.8054	76.7068
1376228_at	830009	AT4G3852	NA	122.6937	103.1806	427.5253
1370667_at	24785	Slc9a4	solute carrier family 9, member 4	122.6753	10.95787	132.9519
1392104_at	362748	RGD13062	similar to chromosome 14 open readin	122.6634	142.7822	90.52535
1374778_at	25423	Ctsc	cathepsin C	122.663	75.96342	543.9157
1394918_at	360645	Ccdc44_pr	coiled-coil domain containing 44 (predi	122.6339	228.3912	94.05562
1398621_at	314416	Ak7_predic	adenylate kinase 7 (predicted)	122.5564	22.57393	94.77167
1398540_at	54289	Rgs1	regulator of G-protein signaling 1	122.5098	30.38056	744.5035
1373992_at	307414	MGC10882	similar to interferon-inducible GTPase	122.4469	57.40808	53.68534
1375101_at	117092	Serpinb7	serine (or cysteine) proteinase inhibito	122.4209	12.82196	11.41008
1391472_at	364475	Dzip1	DAZ interacting protein 1	122.4159	68.45707	787.9093
1370834_at	84406	Hs3st1	heparan sulfate (glucosamine) 3-O-sul	122.4091	299.9688	872.8394
1380058_at	316228	RGD15620	similar to p53-associated parkin-like cy	122.3658	82.10739	73.9175
1390565_at	291618	RGD15598	similar to potassium channel tetrameris	122.2803	97.18428	506.8025
1376291_at	499412	NA	NA	122.2683	696.4199	147.1359
1369020_at	114613	Slc5a5	solute carrier family 5 (sodium iodide s	122.1813	126.3233	52.72906
1370202_at	24913	Hrasls3	HRAS like suppressor 3	122.0935	257.9579	505.2326
1369570_at	24912	Sult2a1	sulfotransferase family 2A, dehydroepi	122.0792	20.85189	43.74014
1379144_at	361829	NA	NA	122.0666	1361.67	364.5528
1370788_at	116499	Fgf4	fibroblast growth factor 4	121.9977	192.9025	20.39176
1385247_at	24862	Ugt2b	UDP glycosyltransferase 2 family, poly	121.9841	7.849944	27.93229
1369789_at	114516	Glra3	glycine receptor, alpha 3 subunit	121.7835	6.216771	110.5281
1389855_at	117256	Ppp2r2c	protein phosphatase 2 (formerly 2A), re	121.772	27.53545	134.8544
1394141_at	502382	LOC50238	NA	121.7693	55.75111	48.71829
1369537_at	83567	Mchr1	melanin-concentrating hormone recept	121.6765	194.4815	113.8272
1392280_at	310553	Tlr2	toll-like receptor 2	121.6491	32.13781	99.62794
1369096_at	171287	Epha7	Eph receptor A7	121.37	162.7699	176.335
1391504_at	310587	RGD13076	similar to hornerin (predicted)	121.1292	24.66033	22.59517
1368681_at	24695	Pthlh	parathyroid hormone-like peptide	121.1058	318.9985	346.3021
1398482_at	292700	Bcl3_predi	B-cell leukemia/lymphoma 3 (predicted	121.004	90.95297	906.7587
1373295_at	294025	Obfc1	oligonucleotide/oligosaccharide-bindin	120.9709	3037.125	512.5321
1369801_at	29259	Sell	selectin, lymphocyte	120.8452	55.26941	245.356
1375808_at	287108	Prss27	protease, serine 27	120.8276	167.5801	40.36748

1392515_at	494205	Ly49i9	Ly49 inhibitory receptor 9	120.7524	26.34736	363.7236
1392990_at	312936	Sox17_pre	SRY-box containing gene 17 (predicte	120.5941	17.11965	227.5181
1387571_at	81808	Nr2f1	nuclear receptor subfamily 2, group F,	120.302	360.2813	65.43524
1369711_at	24182	Agtr2	angiotensin II receptor, type 2	120.1155	56.46707	162.7217
1375557_at	294640	Otp	orthopedia homolog (Drosophila)	120.0063	61.71499	77.99148
1380946_at	302405	RGD15663	RGD1566367 (predicted)	119.9882	855.8601	557.5667
1385817_at	306252	Nek4	NIMA (never in mitosis gene a)-relatec	119.7581	191.9843	149.1753
1369605_at	29567	Hes2	hairy and enhancer of split 2 (Drosoph	119.6649	46.37631	103.3426
1378318_at	498097	RGD15595	similar to WD repeat domain 53 (predi	119.6141	745.3291	463.4497
1387275_at	84046	Sox11	SRY-box containing gene 11	119.5992	26.79809	51.01239
1372200_at	406163	Gpsm3	G-protein signalling modulator 3 (AGS	119.5646	34.95668	51.4471
1387982_at	29260	Tlr4	toll-like receptor 4	119.4068	126.3284	49.3718
1393332_at	307801	RGD13111	similar to RIKEN cDNA 5730466C23 (	119.365	34.50857	122.4786
1390472_at	497009	Asahl_prec	N-acylsphingosine amidohydrolase (ac	119.3264	635.7076	318.2947
1379766_at	338477	Sla	src-like adaptor	119.32	18.6442	12.07159
1370441_at	266782	Plpi	prolactin like protein I	119.2809	36.72555	34.38851
1367962_at	171009	Actn3	actinin alpha 3	119.1945	67.18063	262.5556
1387191_at	54193	Pbsn	probasin	119.118	19.45575	25.29514
1393335_at	317470	Egfl6	EGF-like-domain, multiple 6	119.0625	55.69751	445.2366
1370941_at	25267	Pdgfra	platelet derived growth factor receptor,	118.958	700.6598	655.7756
1394075_at	361435	Fanca_pre	Fanconi anemia, complementation gro	118.816	340.4855	244.4874
1368632_at	24370	Foxg1	forkhead box G1	118.7975	86.71417	139.4964
1368802_at	24659	Pmch	pro-melanin-concentrating hormone	118.7709	47.85263	86.27154
1394633_at	300228	eplin	epithelial protein lost in neoplasm	118.7461	181.0586	151.8528
1381192_at	364976	RGD13118	similar to RIKEN cDNA 1700013H19 (	118.6081	48.1261	72.48408
1367850_at	116591	Fcgr3	Fc receptor, IgG, low affinity III	118.5898	6.93062	390.4079
1384417_at	83418	Apoc4	apolipoprotein C-IV	118.406	37.81906	209.6789
1369771_at	25467	Irs1	insulin receptor substrate 1	118.3777	90.5512	276.5499
1369731_at	59322	Cnksr2	connector enhancer of kinase suppres	118.365	28.03031	101.7311
1369905_at	140675	Gabra4	gamma-aminobutyric acid (GABA-A) re	118.2129	1186.707	98.10201
1368561_at	84356	Abcd2	ATP-binding cassette, sub-family D (A	118.1437	55.29335	133.9593
1368471_at	25656	Guca2a	guanylate cyclase activator 2a (guanyl	118.0146	27.5963	103.7237
1376615_at	294299	Tead3	TEA domain family member 3	117.9033	419.1147	245.7961
1381374_at	361549	Lgi4	leucine-rich repeat LGI family, membe	117.8425	23.64574	155.8365
1370660_at	286983	LOC28698	putative pheromone receptor (Go-VN3	117.6693	32.54232	134.5539
1382819_at	298182	Tyrp1_map	tyrosinase-related protein 1 (mapped)	117.6264	57.3222	141.0645
1391278_at	366213	Nkx2-4_pre	NK2 transcription factor related, locus	117.6054	151.9319	27.43147
1381310_at	367103	LOC36710	NA	117.4373	52.86007	481.8968
1373544_at	246759	Cxcl9	chemokine (C-X-C motif) ligand 9	117.3816	77.86873	221.8767
1394999_at	501854	RGD15626	similar to RAB7-like protein (predicted)	117.3399	51.70928	150.6354
1374389_at	25202	Gucy1b3	guanylate cyclase 1, soluble, beta 3	117.3032	1362.49	1388.529
1387744_at	114593	Nppc	natriuretic peptide precursor type C	117.2443	47.9598	85.35452
1371339_at	29388	Tnni1	troponin I, skeletal, slow 1	117.2397	243.507	62.52601
1386403_at	313270	Egfl5_pred	EGF-like-domain, multiple 5 (predicted	117.188	80.14752	221.5005
1373798_at	362020	RGD13091	similar to CG5435-PA (predicted)	117.1825	131.9127	120.043
1387547_a_at	64124	Eltd1	EGF, latrophilin and seven transmemb	117.1672	29.35095	96.78283
1373674_at	362429	Mfap5_pre	microfibrillar associated protein 5 (prec	117.1324	60.00421	456.4918
1377097_at	654441	Cox6b2	NA	117.1113	45.25544	36.21536
1385465_at	292843	Siglec5_pr	sialic acid binding Ig-like lectin 5 (predi	116.9594	90.46898	117.0598
1368455_at	171062	Nkg7	natural killer cell group 7 sequence	116.939	18.15008	26.01719
1389756_at	362510	Melk_predi	maternal embryonic leucine zipper kina	116.7613	4840.066	185.2694

1368378_at	64392	Fthfd	formyltetrahydrofolate dehydrogenase	116.726	110.5183	198.3403
1368779_a_at	25206	Gucy1b2	guanylate cyclase 1, soluble, beta 2	116.7075	105.6749	12.19486
1367912_at	59107	Ltbp1	latent transforming growth factor beta l	116.6477	66.35714	257.2261
1398278_at	24683	Prl	prolactin	116.5374	136.1635	28.77347
1380076_at	316614	Asb18_pre	ankyrin repeat and SOCS box-containi	116.4454	182.5191	123.4451
1383425_at	499786	NA	NA	116.4278	218.0419	108.7708
1370624_at	29636	F2rl2	coagulation factor II (thrombin) recepto	116.3858	15.87415	52.42631
1391151_at	303631	Arsg	arylsulfatase G	116.2713	52.27992	44.82652
1367952_at	29216	Lrp2	low density lipoprotein receptor-relatec	115.9373	321.1426	650.1812
1387853_at	24163	Acr	acrosin	115.8471	19.88364	161.2856
1384664_at	192155	Sftpb	surfactant associated protein B	115.837	146.0341	100.277
1396861_at	500527	RGD1566C	similar to DNA segment, Chr 4, Brigha	115.7791	47.8588	69.51076
1368640_at	171403	Spas1	spermatogenic specific-gene1	115.6594	122.8218	247.4459
1370157_at	64672	Pln	phospholamban	115.4847	48.77587	133.2057
1388263_at	64358	Kcna6	potassium voltage gated channel, shal	115.4361	121.7857	32.982
1371292_at	25483	Msx2	msh homeo box homolog 2 (Drosophil	115.3831	130.3315	95.39037
1395472_at	502715	Lrrc17	leucine rich repeat containing 17	115.3261	20.48622	10.55059
1384576_at	301285	Tcfap2b_pr	transcription factor AP-2 beta (predicte	115.2395	100.0675	186.4649
1376632_at	494021	Lmcd1_pre	LIM and cysteine-rich domains 1 (pred	115.2087	30.90066	159.347
1394486_at	500249	Rab43	Ras-related protein RAB43	115.027	80.7013	228.7108
1388176_at	113892	Cml3	camello-like 3	115.0206	97.31146	32.63178
1395376_at	316767	Ddx11_pre	DEAD/H (Asp-Glu-Ala-Asp/His) box pc	114.9473	1236.027	820.1884
1369450_at	171398	Ust5r	integral membrane transport protein U	114.9392	82.77945	265.7301
1386200_at	291926	RGD13058	similar to Myosin light chain kinase 2, s	114.6836	59.82795	208.2646
1397276_at	306767	Arl10	ADP-ribosylation factor-like 10	114.6169	45.32771	78.74669
1382608_at	288563	Actl6b_pre	actin-like 6B (predicted)	114.5561	485.4418	500.1206
1391637_at	295635	LOC29563	similar to RIKEN cDNA 5830480G12	114.5289	61.04706	91.82715
1388272_at	299352	Igh-1a	immunoglobulin heavy chain 1a (serun	114.5276	60.00095	12.1818
1385419_at	302619	Gpr143_pr	G protein-coupled receptor 143 (predic	114.4218	15.44429	64.22226
1377693_at	313244	Frmpd1_pr	FERM and PDZ domain containing 1 (	114.3617	329.5171	52.46082
1397704_at	498536	NA	NA	114.2888	1440.652	201.0385
1370956_at	29139	Dcn	decorin	114.2728	32.88032	5831.24
1379681_at	298300	Ttc22_prec	tetratricopeptide repeat domain 22 (pre	114.1954	28.22422	679.1625
1387324_at	25677	Mak	male germ cell-associated kinase	114.1377	115.4462	44.25439
1369841_at	60460	Hspa2	heat shock 70kDa protein 2	114.1273	99.80626	397.2005
1398521_at	114246	Trpv6	transient receptor potential cation char	113.9	351.9068	280.7996
1387886_at	84400	Prelp	proline arginine-rich end leucine-rich re	113.8823	40.78796	284.009
1371156_a_at	25674	Glra1	glycine receptor, alpha 1 subunit	113.8495	152.8813	37.06754
1396246_at	361831	Ube2d1_pr	ubiquitin-conjugating enzyme E2D 1, U	113.8468	291.1114	152.1465
1383706_at	360459	RGD13057	similar to RIKEN cDNA 2900011O08	113.7652	173.165	840.9532
1391267_at	498151	NA	NA	113.5836	127.1655	56.06805
1370146_at	25456	Glrb	glycine receptor, beta subunit	113.5786	73.37017	417.7164
1369010_at	114212	Chek2	CHK2 checkpoint homolog (S. pombe)	113.5772	508.0821	171.5355
1393060_at	311827	RGD13054	similar to KIAA0605 gene product (pre	113.5204	86.66137	388.343
1369360_at	79222	Gucy2e	guanylate cyclase 2e	113.4905	33.96475	36.14836
1397202_at	499334	RGD15602	similar to RIKEN cDNA 1700028P14 (p	113.4295	395.2395	16.06079
1370119_at	64569	Lst1	leucocyte specific transcript 1	113.323	101.0993	65.00625
1391354_at	296395	Kcng1	potassium voltage-gated channel, subf	113.2223	234.5374	94.95052
1391345_at	300455	Bmper_pre	BMP-binding endothelial regulator (pre	113.1845	46.7404	54.39001
1372559_at	406170	Ng23	Ng23 protein	113.1231	86.64838	223.4203
1381334_at	305675	RGD15654	similar to synaptopodin 2-like (predicte	113.0526	66.89216	31.69864

1385447_at	311491	RGD13083	similar to RIKEN cDNA 1700010M22 (	113.0404	88.58515	91.47003
1370998_at	24415	Grm2	glutamate receptor, metabotropic 2	113.0042	517.7351	33.86209
1392703_at	303399	Tbx4_pred	T-box 4 (predicted)	112.8254	21.94877	15.82836
1378720_at	140589	Gli1	GLI-Kruppel family member GLI1	112.7737	27.65842	25.92092
1394259_at	304388	Cldn15_pre	claudin 15 (predicted)	112.6752	146.7751	86.72155
1378358_at	445541	Repin1	replication initiator 1	112.6458	89.75469	42.04151
1381722_at	501283	LOC50128	NA	112.6129	18.22255	59.78925
1367572_at	24585	Myl3	myosin, light polypeptide 3	112.5518	18.95879	99.09504
1393158_at	363198	LOC36319	similar to 2610528M18Rik protein	112.5484	2903.69	310.2453
1387491_at	79223	Gyk	glycerol kinase	112.4944	180.6534	405.2974
1378377_at	362046	Dapp1_pre	dual adaptor for phosphotyrosine and :	112.3075	59.20456	142.0016
1373401_at	116640	Tnc	tenascin C	112.2963	38.19016	47.42613
1370009_at	24207	Apoc3	apolipoprotein C-III	112.232	206.6688	85.77914
1390765_at	286888	Wfdc2	WAP four-disulfide core domain 2	111.8819	814.6635	1117.128
1382966_at	360348	Hsd3b1_pr	hydroxysteroid dehydrogenase-1, delta	111.8399	82.31558	55.64841
1387581_at	65053	Pamci	peptidylglycine alpha-amidating monoo	111.8025	19.57745	43.40828
1368555_at	29185	Cd37	CD37 antigen	111.801	161.9278	86.91795
1368367_at	117179	Cuzd1	CUB and zona pellucida-like domains	111.6457	19.35003	637.983
1387640_at	170582	Fgf15	fibroblast growth factor 15	111.6306	167.999	64.28715
1373026_at	363028	Spbc24_pr	spindle pole body component 24 homo	111.5942	4851.759	617.9485
1389677_at	362842	Syde1_pre	synapse defective 1, Rho GTPase, hor	111.5499	115.7523	126.969
1368348_at	25553	Slc6a4	solute carrier family 6 (neurotransmitte	111.5124	34.8669	88.67791
1371801_at	295426	Myoz2_pre	myozenin 2 (predicted)	111.4878	7.400847	33.20753
1381382_at	361875	RGD15649	similar to Vacuolar ATP synthase subu	111.4789	113.1051	217.3809
1369925_at	245916	Cst11	cystatin 11	111.3967	157.4752	179.3923
1372323_at	114123	Sardh	sarcosine dehydrogenase	111.2521	810.7981	411.4988
1384431_at	300866	RGD13085	similar to RIKEN cDNA 4930431B11 g	111.244	4.491041	355.7088
1388116_at	29393	Col1a1	procollagen, type 1, alpha 1	111.2354	12.42067	1349.658
1371050_at	84024	Pon1	paraoxonase 1	111.2148	28.25718	61.24799
1394874_at	360707	RGD13109	similar to Dermal papilla derived protei	111.2096	126.3883	76.00462
1385354_at	84381	Itga8	integrin alpha 8	111.1833	24.06808	84.36239
1397877_at	310131	Fyb_predic	FYN binding protein (predicted)	111.1285	108.1101	50.66361
1393675_at	407781	Btnl3	butyrophilin-like 3	110.8779	123.0106	54.81007
1381169_at	503301	NA	NA	110.7904	262.2346	166.1933
1385591_at	309071	Fank1	fibronectin type 3 and ankyrin repeat d	110.7391	34.25593	21.44904
1384628_at	308129	RGD13092	similar to RIKEN cDNA 0610009A07	110.6484	121.8816	145.852
1376174_at	362774	LOC36277	similar to serine proteinase inhibitor A1	110.5425	366.8795	76.73411
1374445_at	362436	Gpr162_pr	G protein-coupled receptor 162 (predic	110.535	589.1843	143.9582
1382639_at	501406	LOC50140	NA	110.4989	61.07265	116.602
1368180_s_at	24422	Gsta2	glutathione-S-transferase, alpha type2	110.4934	104.2084	92.89793
1380411_at	306540	RGD13071	similar to hypothetical protein MGC333	110.4367	11.15949	13.02992
1398567_at	501127	RGD15607	similar to FLJ42986 protein (predicted)	110.3657	70.75439	75.12361
1370858_at	309203	Scgb1d2	secretoglobin, family 1D, member 2	110.3632	4.464037	29.50319
1391749_a_at	364679	NA	NA	110.2898	400.0562	248.713
1369595_at	170583	Fgf23	fibroblast growth factor 23	110.2471	53.09926	16.93954
1394489_at	311936	Rnf32	ring finger protein 32	110.1176	240.1071	40.38538
1377554_at	353218	Tnfsf9	tumor necrosis factor (ligand) superfan	110.0196	121.3308	256.237
1394125_at	499291	RGD15658	RGD1565859 (predicted)	109.8989	16.51186	10.85006
1369439_at	81638	Agtr1b	angiotensin receptor 1b	109.8599	56.13283	128.4971
1371815_at	313662	Mfap2_pre	microfibrillar-associated protein 2 (prec	109.8134	60.19824	241.7404
1395003_at	362430	Clec4a1	C-type lectin domain family 4, member	109.7722	64.30198	233.2627

1386929_at	25058	Hk1	hexokinase 1	109.6669	125.6373	279.1356
1385662_at	444985	Dnmt3b	DNA methyltransferase 3B	109.5541	82.63512	109.7402
1372818_at	361289	Colec12	collectin sub-family member 12	109.5491	225.5941	767.2443
1382678_at	289057	Cfhl1	complement component factor h-like 1	109.5308	109.595	123.5885
1368066_at	116502	Bak1	BCL2-antagonist/killer 1	109.4175	238.7109	122.8743
1386514_at	361129	Hapln4_pre	hyaluronan and proteoglycan link prote	109.3304	502.7868	413.0724
1374302_at	192223	Opn4	opsin 4 (melanopsin)	109.3117	158.1325	30.39591
1391430_at	365345	Cyb5r2	cytochrome b5 reductase 2	109.3035	238.2196	20.97786
1389690_at	301100	RGD13111	similar to RIKEN cDNA 2410146L05 (p	109.2333	56.0119	157.6976
1385635_at	310693	Cd5l	CD5 antigen-like	109.1711	20.28217	121.7835
1369292_at	25322	Hsd17b1	hydroxysteroid (17-beta) dehydrogena	109.1427	239.7831	179.2567
1369893_at	24828	Hist1h2aa	testis-specific histone 2a	109.1086	99.0231	54.77479
1393307_at	362284	Phactr3	phosphatase and actin regulator 3	109.0953	168.7161	211.1516
1368996_at	24926	Ceacam3	carcinoembryonic antigen-related cell a	109.0621	135.5875	100.9555
1393112_at	309641	Tcp11	t-complex protein 11 (mouse)	109.044	34.87032	47.07248
1377317_at	501959	NA	NA	108.9535	55.9162	51.54976
1387924_at	246217	Ngef_predi	neuronal guanine nucleotide exchange	108.8965	701.0737	185.2929
1370228_at	24825	Tf	transferrin	108.8944	47.33063	765.0311
1391087_at	365389	Lrrc56	leucine rich repeat containing 56	108.759	381.497	26.75352
1374103_at	65153	Freq	frequenin homolog (Drosophila)	108.6762	799.0191	797.2467
1369578_at	78983	Tas2r7	taste receptor, type 2, member 7	108.6412	98.13129	277.5415
1368629_at	24714	Reg1	regenerating islet-derived 1	108.6247	170.0362	1386.877
1397110_at	306110	Klf12_pred	Kruppel-like factor 12 (predicted)	108.607	55.18555	185.215
1371163_at	65273	Cdk107	CDK107	108.3882	84.98969	38.08917
1385320_at	317379	LOC31737	similar to Pdz-containing protein	108.3575	60.92403	186.6414
1373756_at	361675	RGD13094	similar to BM88 antigen	108.3055	118.6198	40.16119
1396208_at	29566	Ggtla1	gamma-glutamyltransferase-like activit	108.2503	42.31834	2674.661
1394398_at	499944	NA	NA	108.1653	241.9892	275.8182
1379606_at	308821	Rab30	RAB30, member RAS oncogene family	108.1302	452.3514	462.304
1392805_at	311345	NA	NA	108.0637	334.258	83.60043
1369193_at	25164	Cdkn2b	cyclin-dependent kinase inhibitor 2B (p	108.0305	33.66725	42.74821
1390638_at	316539	RGD15605	similar to Eph receptor A4 (predicted)	107.9511	122.7045	872.7999
1386742_at	367754	RGD15626	similar to leucine-rich repeats and calp	107.8515	275.4847	353.2207
1387286_at	24414	Grm1	glutamate receptor, metabotropic 1	107.8453	149.6849	163.7485
1384830_at	298524	Dnali1	dynein, axonemal, light intermediate po	107.8387	83.48866	106.4746
1381904_at	309611	G7c	G7c protein	107.8359	33.68254	296.8865
1368439_at	29361	Sox10	SRY-box containing gene 10	107.7401	61.98131	103.4083
1370723_at	192254	Grasp	GRP1 (general receptor for phosphoin	107.6049	44.62978	17.57895
1380594_at	315174	Scube1	signal peptide, CUB domain, EGF-like	107.5465	255.028	123.1768
1396537_at	303596	Rnf190	ring finger protein 190	107.4806	32.96155	68.46315
1387133_at	117059	Calb2	calbindin 2	107.2263	27.6577	209.5501
1383078_at	360546	Efnb3_pred	ephrin B3 (predicted)	107.1995	332.4769	1014.161
1380271_at	498114	NA	NA	107.1582	114.473	120.2117
1377722_at	368044	Atp6v1g2	ATPase, H+ transporting, V1 subunit G	107.1196	56.66994	211.7068
1374226_at	301012	Col7a1_pre	procollagen, type VII, alpha 1 (predicte	107.1186	49.08781	101.9119
1394302_at	501106	RGD15652	similar to G-protein coupled receptor 1	107.101	90.88572	39.24952
1387580_at	64677	Srd5a2	steroid 5-alpha-reductase 2	107.0845	87.65063	120.1873
1390982_at	288242	Chaf1b	chromatin assembly factor 1, subunit B	106.8285	1157.802	31.04653
1387203_at	25658	Gckr	glucokinase regulatory protein	106.8138	48.87321	38.11764
1378214_at	286974	GalNAc4S6	N-acetylgalactosamine 4-sulfate 6-O-s	106.6996	62.02076	534.3245
1368257_at	24256	Cgm3	Carcinoembryonic antigen gene family	106.6156	59.19731	155.9676

1374434_at	498222	LOC49822	similar to specifically androgen-regulat	106.6065	17.42807	195.958
1390780_at	305705	Lrrc3b_pre	leucine rich repeat containing 3B (prec	106.494	15.80331	133.5548
1387705_at	25555	Sstr4	somatostatin receptor 4	106.4101	145.6803	87.66559
1388017_at	252882	Mamdc4	MAM domain containing 4	106.4	246.4112	161.8964
1379992_at	293490	Gdpd3_pre	glycerophosphodiester phosphodiester	106.3569	101.4717	156.8932
1378365_at	297386	Slc4a5	solute carrier family 4, sodium bicarbo	106.3561	99.4002	63.76303
1369259_at	25430	Dio1	deiodinase, iodothyronine, type I	106.3555	188.943	238.1315
1369478_at	58850	Nr0b1	nuclear receptor subfamily 0, group B,	106.3202	157.3138	235.7761
1369155_at	116658	Cntn4	contactin 4	106.3093	124.4087	45.64489
1368223_at	79252	Adamts1	a disintegrin-like and metallopeptidse (	106.2188	83.84684	1247.245
1379190_at	366463	Oxct2a	3-oxoacid CoA transferase 2A	106.1619	36.35824	169.8202
1375364_at	314621	Stk11_prec	serine/threonine kinase 11 (predicted)	106.0005	45.48617	94.63483
1383614_at	289419	Nuak2	NUAK family, SNF1-like kinase, 2	105.9772	56.33522	260.9362
1387208_at	85382	Ngb	neuroglobin	105.9609	95.26519	288.9222
1398463_at	664630	B0at1	NA	105.9143	95.10394	84.56674
1376231_at	499914	RGD15622	similar to Hypothetical UPF0080 protei	105.8383	2465.851	176.9171
1397950_at	360855	Smg7_prec	Smg-7 homolog, nonsense mediated r	105.7025	154.9702	27.2239
1393785_at	316616	RGD13053	similar to hypothetical protein FLJ2252	105.7006	105.9342	262.2147
1385435_at	303148	Slc36a3	solute carrier family 36 (proton/amino a	105.6329	67.76763	39.07757
1370710_at	246281	Asmt	acetylserotonin O-methyltransferase	105.6292	60.03947	76.60744
1383426_at	300732	Pstpip1_pr	proline-serine-threonine phosphatase-	105.6003	119.9899	153.5108
1385751_at	292406	Thbs2	thrombospondin 2	105.5704	55.90076	40.85592
1368610_at	59105	Mca32	mast cell antigen 32	105.5285	154.1031	169.9108
1368609_at	24777	Slc10a1	solute carrier family 10 (sodium/bile ac	105.5144	19.02526	38.12131
1378150_at	304314	Card11_pr	caspase recruitment domain family, m	105.4991	45.83253	61.25917
1391878_at	498982	RGD1560C	similar to Myb proto-oncogene protein	105.4734	402.1535	93.52364
1381756_at	295498	Adh6a_pre	alcohol dehydrogenase 6A (class V) (p	105.4389	81.76234	128.187
1378514_at	366311	RGD15642	similar to hypothetical protein MGC346	105.4321	54.10075	25.12874
1390209_at	302975	Syngr3_pre	synaptogyrin 3 (predicted)	105.43	312.4763	90.2571
1383840_at	306375	RGD13076	similar to chondroitin beta1,4 N-acetylg	105.3715	52.413	414.8096
1377689_at	304477	Kntc1_prec	kinetochore associated 1 (predicted)	105.2852	4826.147	223.8951
1378748_at	502031	NA	NA	105.2324	104.3229	99.63502
1369898_a_at	25040	Gip	gastric inhibitory polypeptide	105.1637	285.3344	199.7715
1388053_at	252827	Cdk5rap1	CDK5 regulatory subunit associated pr	105.0827	44.24031	122.4017
1393756_at	25312	Dmp1	dentin matrix protein 1	105.0449	29.1558	288.2298
1385174_at	408245	Tessp6	testis-specific serine protease-6	104.9463	56.02024	22.87265
1389404_at	311547	Fkhl18	forkhead-like 18 (Drosophila)	104.8922	53.04182	113.7135
1392204_at	498945	LOC49894	similar to NK10	104.887	417.0237	1381.457
1382690_at	24257	Cgm4	carcinoembryonic antigen gene family	104.8707	108.5071	258.3305
1379566_at	288321	Rbm11_pre	RNA binding motif protein 11 (predicte	104.8245	1558.759	546.5626
1373889_at	303666	RGD15617	similar to dendritic cell-derived immuno	104.7397	138.9231	36.01126
1383898_at	289949	Oit1_predic	oncoprotein induced transcript 1 homo	104.6991	11.66986	24.30926
1372159_at	296345	Jph2	junctophilin 2	104.6345	301.3288	218.8795
1382378_at	293676	Ctsw	cathepsin W	104.628	36.03152	27.22339
1374768_at	362994	LOC36299	NA	104.6115	23.277	376.1486
1370078_at	60377	Lin7b	lin-7 homolog b (C. elegans)	104.3604	153.1073	478.043
1375730_at	362441	RGD13119	similar to expressed sequence AI6467	104.2582	78.96404	94.66105
1398520_at	500685	RGD15661	similar to pleckstrin homology domain	104.194	275.3397	53.25014
1373102_at	192248	Cdh13	cadherin 13	104.171	37.70919	98.59017
1371274_at	296234	CysS	cystatin S	104.0886	55.15956	7.005731
1371349_at	294337	Col6a1_pre	procollagen, type VI, alpha 1 (predicte	104.0613	55.28648	1850.156

1392407_at	498282	RGD15614	similar to RIKEN cDNA 6030405P05 g	104.0446	26.04332	220.3951
1368575_at	29323	Slc6a18	solute carrier family 6 (neurotransmitte	103.9536	18.307	30.06964
1386800_at	311655	Mocs3_pre	molybdenum cofactor synthesis 3 (pre	103.8539	22.23512	147.0951
1386132_at	500511	RGD15654	similar to hypothetical protein MGC351	103.6384	75.67202	39.45322
1385477_at	367734	RGD15631	similar to RIKEN cDNA 1700054O13 (	103.5714	19.82787	11.68065
1368580_at	116673	Sdc3	syndecan 3	103.5593	139.7765	117.1767
1397989_at	282635	Mbnl1	muscleblind-like 1 (Drosophila)	103.398	58.15414	100.3676
1393209_at	192675	bsnd	Bartter syndrome, infantile, with senso	103.3697	38.11278	204.0489
1393232_at	498911	RGD15655	RGD1565500 (predicted)	103.3131	37.13461	32.0096
1387297_at	58975	Klrg1	killer cell lectin-like receptor subfamily	103.2455	23.22229	155.7253
1396060_at	316353	RGD15640	similar to cellular repressor of E1A-stin	103.091	227.5328	79.95491
1397568_at	362246	Trp53inp2	tumor protein p53 inducible nuclear pro	102.9123	205.2466	314.5013
1382327_at	293654	Doc2g	double C2, gamma	102.6874	68.01358	222.4098
1368121_at	26760	Akr7a3	aldo-keto reductase family 7, member	102.5165	47.77804	49.08293
1369401_at	84608	Slc21a13	solute carrier family 21, member 13	102.3399	75.51963	151.3561
1386437_at	308622	Txlnb_pred	taxilin beta (predicted)	102.2972	17.51417	26.32934
1386938_at	81641	Anpep	alanyl (membrane) aminopeptidase	102.2342	232.8384	196.3143
1392739_a_at	315500	RGD13074	similar to RIKEN cDNA 2310005P05	102.2226	566.1358	606.0395
1370023_at	25655	Gja4	gap junction membrane channel protei	102.1834	55.23457	233.7272
1387967_at	266602	Spink3	serine protease inhibitor, Kazal type 3	102.0311	56.9236	618.2668
1378545_at	360737	Tbx1_pred	T-box 1 (predicted)	101.788	52.18236	54.79152
1389448_at	287606	4-Sep	septin 4	101.7726	174.4622	524.6755
1381318_at	113901	Chia	chitinase, acidic	101.7677	159.4714	152.3768
1379639_at	310647	NA	NA	101.687	61.68234	62.25004
1381487_at	89807	Angpt1	angiopoietin 1	101.6199	28.02088	28.95601
1397580_at	291669	RGD15645	similar to 40S ribosomal protein S16 (p	101.5792	142.4507	79.18953
1374912_at	171529	Kif2c	kinesin family member 2C	101.4771	2067.067	43.6607
1379727_at	500760	NA	NA	101.4733	77.50936	143.1246
1387804_at	140939	Trim63	tripartite motif protein 63	101.2064	20.48581	35.87085
1370546_at	286931	Unc13c	unc-13 homolog C (C. elegans)	101.0683	22.09636	10.9527
1380621_at	361597	RGD15643	similar to tyrosine kinase Fps/Fes (pre	100.9853	203.7822	24.40309
1388657_at	308912	Dchs1_pre	dachsous 1 (Drosophila) (predicted)	100.9783	89.75608	107.5709
1385139_at	500118	LOC50011	similar to RIKEN cDNA D330028D13	100.8917	931.8995	74.1275
1368497_at	25303	Abcc2	ATP-binding cassette, sub-family C (C	100.888	128.2675	73.89698
1395205_at	500901	LOC50090	NA	100.8807	70.95253	270.1683
1369244_at	25242	Arnt	aryl hydrocarbon receptor nuclear tran	100.8365	36.55721	74.51969
1368631_at	59116	Dbil5	diazepam binding inhibitor-like 5	100.8268	67.83053	96.75722
1391626_at	295160	RGD13090	similar to RIKEN cDNA 2610034E18 g	100.818	841.677	282.5071
1398660_at	300713	Slc35f2_pr	solute carrier family 35, member F2 (p	100.7834	324.0233	178.048
1387937_at	64509	Tpbpa	trophoblast specific protein alpha	100.7768	153.1974	304.8638
1369347_s_at	192211	Prom2	prominin 2	100.7252	119.1711	565.7142
1383367_at	287637	Samd14	sterile alpha motif domain containing 1	100.71	206.2638	76.84417
1387281_a_at	29660	Pnck	pregnancy upregulated non-ubiquitous	100.6737	417.3518	71.63225
1385687_at	498586	NA	NA	100.5562	61.45313	145.758
1398215_at	361459	RGD13068	similar to hypothetical protein MGC346	100.4792	124.2475	62.97457
1369818_at	25451	Gabrb2	gamma-aminobutyric acid (GABA-A) re	100.4608	17.40648	17.67103
1385463_at	498921	NA	NA	100.4355	911.0884	146.1725
1387158_at	25727	Mep1b	meprin 1 beta	100.3994	30.71522	10.53094
1397919_at	297444	Ccdc37_pr	coiled-coil domain containing 37 (predi	100.2492	11.90168	19.07252
1384598_at	501190	NA	NA	100.0025	76.88259	32.16137
1376961_at	316205	Lrfn2	leucine rich repeat and fibronectin type	99.96645	71.54172	99.91005

1387936_at	361510	Sult2a2_pr	sulfotransferase family 2A, dehydroepi	99.93959	19.04879	13.44819
1369574_at	78984	Tas2r13	taste receptor, type 2, member 13	99.7349	31.75707	43.10333
1390953_at	304010	Zbtb11_pre	zinc finger and BTB domain containing	99.67484	173.5783	15.00212
1370587_at	298521	Grik3	glutamate receptor, ionotropic, kainate	99.56736	95.83547	64.72429
1381556_at	498673	NA	NA	99.54022	24.05152	129.8533
1369715_at	79213	Slc6a11	solute carrier family 6 (neurotransmitte	99.52537	31.47528	71.17077
1383895_at	361415	Dynlrb2_pr	dynein light chain roadblock-type 2 (pr	99.48602	201.5946	1020.524
1375638_at	316384	Sdpr	serum deprivation response protein	99.44418	55.12561	383.7872
1369441_at	171495	Capn5	calpain 5	99.43444	269.7372	189.9337
1377085_at	291819	Car7_predi	carbonic anhydrase 7 (predicted)	99.31496	188.9312	83.73888
1385840_at	498415	Zpbp	zona pellucida binding protein	99.2377	129.207	209.7412
1374330_at	297025	RGD15654	similar to Kell protein (predicted)	99.21738	137.2305	210.9219
1394962_at	503151	NA	NA	99.11417	53.76392	180.8112
1397533_at	360920	Mobkl1a_p	MOB1, Mps One Binder kinase activat	99.08867	145.9605	35.31394
1385397_at	499991	LOC49999	Ab1-219	99.08771	25.16965	362.387
1389670_at	500127	RGD15664	similar to homeobox protein (predicted	99.08037	56.87991	41.01515
1387988_at	29632	Hsd3b1	hydroxy-delta-5-steroid dehydrogenas	99.01695	163.7341	69.42768
1369957_at	54294	Rgs5	regulator of G-protein signaling 5	98.89127	31.12012	340.2607
1369849_at	57302	Cnr2	cannabinoid receptor 2 (macrophage)	98.80499	40.96405	64.70434
1385623_at	361576	Tulp2	tubby-like protein 2	98.44201	98.50848	82.72593
1375033_at	308579	Cpt1c	carnitine palmitoyltransferase 1c	98.42424	993.3637	142.7731
1387128_at	64508	Adcy3	adenylate cyclase 3	98.16289	150.1551	185.713
1369090_at	25523	Prkg2	protein kinase, cGMP-dependent, type	98.12124	84.83455	151.0294
1392050_at	24338	Ephb1	Eph receptor B1	98.08655	120.5592	193.758
1369546_at	64564	Bbox1	butyrobetaine (gamma), 2-oxoglutarate	98.04461	108.1707	37.5799
1384911_at	303252	Alox12e_pr	arachidonate lipoxygenase, epidermal	98.03232	45.63162	131.5337
1368324_at	360254	Brca2	breast cancer 2	97.82095	442.8504	320.512
1385587_at	292168	Mcoln2	mucolipin 2	97.80294	21.86057	58.3586
1372153_at	287700	Ka15	type I keratin KA15	97.67989	242.5031	128.4664
1391922_at	498633	RGD1564C	similar to Heslike (predicted)	97.65686	15.74005	59.65356
1370703_at	266762	Mrgprf	MAS-related GPR, member F	97.63703	21.00838	25.07536
1369442_at	64556	Norp	nucleolin-related protein	97.562	79.01793	126.2994
1387931_at	24279	Cryge	crystallin, gamma E	97.54815	23.63182	59.71726
1367648_at	25662	Igfbp2	insulin-like growth factor binding protei	97.52252	98.04186	897.333
1367913_at	170520	Cygb	cytoglobin	97.49614	158.802	519.0306
1369186_at	25166	Casp1	caspase 1	97.39461	89.14234	116.432
1387423_at	25477	Lhcgr	luteinizing hormone/choriogonadotropi	97.34685	133.6357	211.1704
1383804_at	313484	RGD15609	similar to doublesex and mab-3 relatec	97.25268	22.55811	95.11194
1388740_at	309186	RGD13101	similar to cDNA sequence BC032204 (	97.18743	19.03752	171.1812
1386697_at	25501	Nxph1	neurexophilin 1	97.15461	89.55803	367.5806
1369606_at	66017	Fgf20	fibroblast growth factor 20	97.12405	29.3418	20.49443
1372685_at	289993	Cdkn3_pre	cyclin-dependent kinase inhibitor 3 (pr	97.02184	10781.15	349.6891
1383787_at	641520	LOC64152	NA	96.86546	41.33162	403.6791
1389179_at	291541	Cidea_prec	cell death-inducing DNA fragmentation	96.86477	113.3508	200.7847
1382669_at	494244	Bin2a	beta-galactosidase-like protein	96.81107	20.44674	155.1924
1397501_at	140584	Wnt11	wingless-type MMTV integration site fa	96.80578	53.47577	119.3114
1374477_at	113931	Prrx2_pred	paired related homeobox 2 (predicted)	96.7871	45.67886	61.65075
1385536_at	365214	RGD15607	similar to zinc finger protein ZFP235 (p	96.76145	62.66673	183.4523
1392976_at	500450	MGC10951	similar to tropomyosin 1, embryonic fib	96.74665	71.90615	69.6432
1392308_at	298579	Pla2g2d	phospholipase A2, group IID	96.70454	51.94933	42.9425
1372190_at	25293	Aqp4	aquaporin 4	96.59195	16.96876	455.4089

1384301_at	498697	RGD15652	similar to Kelch-like protein 3 (predicte	96.4793	716.5159	992.7814
1387214_at	29225	Ces1	carboxylesterase 1	96.43248	49.25873	14.75855
1376308_at	29254	Mgll	monoglyceride lipase	96.42684	45.86357	61.23411
1386518_at	290749	Irf2_predict	interferon regulatory factor 2 (predicted	96.42336	14.67973	15.68738
1388025_at	81644	Opn1sw	opsin 1 (cone pigments), short-wave-s	96.42161	14.6985	10.3176
1377298_at	362393	Antxr1	anthrax toxin receptor 1	96.38384	22.37254	16.9304
1395873_at	315992	Dock3_pre	dedicator of cyto-kinesis 3 (predicted)	96.32654	113.7916	27.92348
1378106_at	293637	Phlda2_pre	pleckstrin homology-like domain, family	96.25476	61.14654	64.69916
1376709_at	295455	Slc39a8	solute carrier family 39 (metal ion trans	96.25348	13.23062	333.0307
1392067_at	498269	RGD15643	similar to RIKEN cDNA C030014K22 g	96.22159	100.8873	126.498
1369618_at	116553	Il13	interleukin 13	96.12524	60.1438	101.7692
1379529_at	501603	NA	NA	95.9527	621.3172	272.6362
1371436_at	294239	Ddah2	dimethylarginine dimethylaminohydrola	95.92402	1678.388	564.6087
1397736_at	306454	Cldn22_pre	claudin 22 (predicted)	95.74022	114.0668	34.14115
1384969_at	499723	RGD15655	similar to collagen, type XXIV, alpha 1	95.72689	162.3359	401.312
1387839_at	24748	RT1-N1	RT1 class Ib gene, H2-TL-like, grc reg	95.70186	31.59073	50.19796
1368250_at	85270	Tekt1	tektin 1	95.60227	122.232	200.4366
1371260_at	29266	Mcpt2	mast cell peptidase 2	95.56271	20.81657	72.6941
1369622_at	192206	Prok2	prokineticin 2	95.53485	43.64881	131.8639
1398245_at	64347	Sncg	synuclein, gamma	95.48199	44.9912	84.98111
1383241_at	312705	C1r	complement component 1, r subcompo	95.45337	231.6699	1072.471
1377772_at	63845	Tmeff1	transmembrane protein with EGF-like a	95.3767	578.4284	95.9286
1376873_at	498922	LOC49892	similar to cerebellin 1 precursor protein	95.37296	1056.806	100.7238
1380495_at	313417	Wdr78	WD repeat domain 78	95.36937	131.5665	27.98281
1386965_at	24539	Lpl	lipoprotein lipase	95.36772	1328.717	8980.041
1369795_at	117052	Kcnj12	potassium inwardly-rectifying channel,	95.36559	140.6295	196.8873
1392825_at	499256	RGD15596	RGD1559600 (predicted)	95.32758	8.01278	35.1824
1395397_at	309514	Hectd2_pre	HECT domain containing 2 (predicted)	95.28577	60.1774	180.5575
1368593_at	25109	Cd1d1	CD1d1 antigen	95.06352	449.4113	477.6262
1374679_at	287738	Higd1b_pre	HIG1 domain family, member 1B (prec	95.04909	99.47218	297.2846
1370105_at	170905	Lfng	lunatic fringe gene homolog (Drosophi	95.0182	73.80865	186.8104
1376738_at	308708	Ttc23	tetratricopeptide repeat domain 23	94.92791	104.7275	346.3205
1374280_at	291388	Cbln2	cerebellin 2 precursor protein	94.90888	274.2848	173.3227
1396177_at	303635	RGD13063	similar to cDNA sequence BC029169	94.8379	123.4576	346.6976
1395462_at	305999	Nkx3-1	NK-3 transcription factor, locus 1 (Dros	94.7221	133.8532	132.324
1370026_at	25420	Cryab	crystallin, alpha B	94.70013	112.3622	330.3663
1368675_at	84031	Chn2	chimerin (chimaerin) 2	94.62311	519.964	29.11293
1391818_at	313824	LOC31382	NA	94.54118	16.85881	65.65808
1379847_at	312492	Dysf_predi	dysferlin (predicted)	94.51258	108.1713	182.4658
1369923_at	58922	Og9x	OG9 homeobox gene	94.37716	12.43682	23.37673
1391020_at	303660	RGD15633	similar to CG1841-PA, isoform A (pred	94.35575	65.90031	75.61286
1397901_at	502987	RGD15614	similar to RIKEN cDNA 2900010J23 (p	94.21655	45.36266	226.3561
1375066_at	293632	RGD15633	similar to RIKEN cDNA 6330512M04 g	94.12849	93.15801	180.8749
1384329_at	302971	Prss22_pre	protease, serine, 22 (predicted)	94.07356	92.56954	212.9799
1387416_at	83618	Rest	RE1-silencing transcription factor	94.0692	84.27625	58.48219
1387394_at	25746	Il2rb	interleukin 2 receptor, beta chain	94.05252	193.6727	288.0143
1381150_at	295695	RGD13052	similar to hypothetical protein (predicte	94.04955	73.38087	165.5164
1376899_at	499071	RGD15662	similar to HSU79303 protein (predicted	94.02102	265.0887	238.0092
1392751_at	317224	RGD15638	similar to Gene model 784 (predicted)	93.95839	17.80721	36.50534
1388191_at	25111	Chrm4	cholinergic receptor, muscarinic 4	93.84925	41.14185	226.8957
1382163_at	85471	Gata3	GATA binding protein 3	93.69674	741.9271	149.9979

1375046_at	289786	LOC28978	hypothetical protein LOC289786	93.61452	125.0547	114.2171
1387873_at	171112	Wfdc1	WAP four-disulfide core domain 1	93.51481	23.79724	58.75187
1369634_at	24779	Slc4a1	solute carrier family 4, member 1	93.49282	175.7334	124.9475
1395515_at	317407	Bhlhb9	basic helix-loop-helix domain containir	93.47783	355.1877	165.5886
1391074_at	25061	Crabp1_ma	cellular retinoic acid binding protein 1 (	93.40578	2024.791	88.94595
1382973_at	301027	RGD15625	similar to testis serine protease2 (pred	93.29293	14.05794	37.69913
1378101_at	266705	Gsbs	G substrate	93.24759	929.3923	70.08528
1369176_at	155205	Slc36a1	solute carrier family 36 (proton/amino a	93.15331	82.43342	55.14707
1374222_at	309131	Slc22a18	solute carrier family 22 (organic cation	93.08796	224.4915	205.7918
1381553_at	353227	Zbtb16	zinc finger and BTB domain containing	93.06711	47.65466	159.4433
1370784_a_at	140727	Cacng6	calcium channel, voltage-dependent, g	92.9882	308.9553	125.4354
1372195_at	296369	Tnnc2	troponin C type 2 (fast)	92.81529	132.891	32.40507
1369144_a_at	65195	Kcnd3	potassium voltage gated channel, Sha	92.754	527.8899	39.07431
1368312_at	25504	Oxt	oxytocin	92.70156	16.07937	94.30019
1377978_at	501767	RGD15627	similar to RIKEN cDNA 2310057N15 (p	92.65949	33.35117	63.20786
1387608_at	66029	Indo	indoleamine-pyrrole 2,3 dioxygenase	92.65313	76.69359	183.72
1397461_at	366859	RGD15604	similar to glycosyltransferase 8 domair	92.65119	12.99421	66.14969
1370716_at	85435	Smad9	MAD homolog 9 (Drosophila)	92.65041	54.71486	64.80512
1372565_at	360959	Htra3_prec	HtrA serine peptidase 3 (predicted)	92.3171	91.04595	845.3647
1369719_at	25512	Phex	phosphate regulating gene with homol	92.29477	31.55898	144.3304
1378693_at	314612	Shc2_pred	src homology 2 domain-containing trar	92.2751	341.6451	30.67871
1393299_at	363972	Dpp10	dipeptidylpeptidase 10	92.2722	1420.645	502.6632
1388080_a_at	85268	Hrh3	histamine receptor H3	92.2549	36.77386	54.36242
1381307_at	499177	NA	NA	92.22978	18.47379	91.06007
1370102_at	54261	Kcnn1	potassium intermediate/small conducta	92.16486	52.16778	79.37273
1395262_at	79208	Epha5	EphA5	92.11797	34.29086	403.4691
1391156_at	288907	RGD13077	similar to podocan protein (predicted)	92.07765	31.93592	21.78052
1387954_a_at	171571	Grip2	glutamate receptor interacting protein 2	91.9407	183.5798	145.9144
1393830_at	310688	Sh2d2a	SH2 domain protein 2A	91.94019	55.65561	98.71415
1368431_at	29135	Hpn	hepsin	91.8264	139.6256	68.79158
1368006_at	89783	Laptm5	lysosomal-associated protein transmer	91.79839	55.90153	214.9522
1383442_at	298676	Morn1	MORN repeat containing 1	91.74662	48.41368	374.6459
1385353_at	361614	RGD13089	LOC361614 (predicted)	91.73347	210.7635	185.8303
1384838_at	246234	Slc34a3	solute carrier family 34 (sodium phosp	91.42779	21.90628	141.2947
1369498_at	25726	Mc5r	melanocortin 5 receptor	91.3855	43.16585	185.9801
1398687_at	297110	NA	NA	91.34411	90.0389	70.01588
1389553_at	362431	Dcir3	dendritic cell inhibitory receptor 3	91.32275	36.84053	113.3904
1375531_at	299830	RGD15612	similar to Leucine-rich and immunoglol	91.00615	65.82884	280.9321
1388238_at	290856	Defcr4	defensin related cryptdin 4	90.97148	53.16014	71.25836
1382106_at	287910	Ccl6	chemokine (C-C motif) ligand 6	90.871	37.92155	107.0235
1384973_at	304688	Serpinb3	serine protease inhibitor B3	90.86879	19.78263	228.7338
1368576_at	25401	Cart1	cartilage homeo protein 1	90.77063	65.62174	43.16715
1372300_at	361364	Dok4_pred	docking protein 4 (predicted)	90.76261	591.3684	441.3666
1370137_at	84114	Agps	alkylglycerone phosphate synthase	90.74979	270.4882	153.2069
1388880_at	303377	Slfn5_pred	schlafen 5 (predicted)	90.73122	60.41423	344.8788
1375917_at	292594	Gp49b	glycoprotein 49b	90.72634	20.73758	128.8386
1370256_at	58868	Fzd1	frizzled homolog 1 (Drosophila)	90.69902	134.2045	609.0593
1382096_at	290214	Cklfsf5_pre	chemokine-like factor super family 5 (p	90.67006	19.33718	14.85816
1378788_at	307310	RGD13116	similar to RIKEN cDNA 4933409D10 (p	90.64345	18.42938	74.05758
1371277_at	364883	Cdx1_pred	caudal type homeo box 1 (predicted)	90.59766	34.86576	235.5263
1385763_at	362231	Cst9_predi	cystatin 9 (predicted)	90.56407	24.70947	25.58342

1375398_at	360839	RGD13062	similar to hypothetical protein DKFZp5	90.45865	27.95561	273.733
1383956_at	362442	RGD15657	similar to ovostatin-2 (predicted)	90.38289	49.93053	36.53166
1381212_at	361954	Veph1	ventricular zone expressed PH domair	90.33338	152.1683	137.6488
1378764_at	501122	RGD15604	similar to RIKEN cDNA 9430069J07 g	90.252	123.7459	27.76084
1388485_at	306748	Cxcl14	chemokine (C-X-C motif) ligand 14	90.14069	28.24915	427.0853
1384865_at	367956	RGD15656	similar to leucine zipper, down-regulate	90.04184	35.91026	54.07592
1387243_at	24297	Cyp1a2	cytochrome P450, family 1, subfamily a	89.94235	41.08137	64.68634
1387881_at	60326	Kcnv1	potassium channel, subfamily V, meml	89.93603	34.69518	21.52301
1387418_a_at	83511	Slc6a2	solute carrier family 6 (neurotransmitte	89.89577	89.81463	150.5747
1385044_at	311093	RGD13062	similar to TGF-beta induced apotosis p	89.89329	31.56973	66.73674
1387523_at	63889	Ptgdr	prostaglandin D receptor	89.7725	79.08808	211.9351
1387743_at	60667	Gpr20	G protein-coupled receptor 20	89.72541	47.10958	56.98489
1387443_at	171454	Btbd14b	BTB (POZ) domain containing 14B	89.63489	121.0958	7.3014
1370312_at	64456	Spon1	spondin 1	89.56842	32.74832	716.999
1390640_at	287146	Chtf18_pre	CTF18, chromosome transmission fide	89.56149	590.4865	41.05637
1396025_at	310780	Gpr61_pre	G protein-coupled receptor 61 (predict	89.41478	34.69722	27.08464
1374970_at	282582	Wnt5b	wingless-related MMTV integration site	89.40971	185.638	60.40692
1385498_at	316557	RGD13064	hypothetical LOC316557	89.39929	73.56609	12.09124
1385309_at	288593	Ccl24	chemokine (C-C motif) ligand 24	89.27197	16.49683	37.79539
1398636_at	298072	RGD13054	similar to Nef associated protein 1	89.03676	397.1545	292.9869
1394613_at	499774	NA	NA	88.99157	142.3832	20.45997
1368171_at	24914	Lox	lysyl oxidase	88.88911	86.58546	831.8472
1387494_at	170925	Slco6b1	solute carrier organic anion transporter	88.86747	41.44563	55.51876
1368635_at	25069	Tnfrsf8	tumor necrosis factor receptor superfa	88.85075	24.54174	215.0894
1367990_at	64349	Crybb3	crystallin, beta B3	88.68353	436.146	163.2172
1373192_at	314523	RGD13064	similar to hypothetical protein FLJ1030	88.65089	541.6973	517.7474
1368613_at	114522	Stag3	stromal antigen 3	88.63273	158.6721	74.47027
1387577_at	54276	Neurod2	neurogenic differentiation 2	88.62844	763.1919	227.537
1369701_at	24538	Lipc	lipase, hepatic	88.60528	59.17287	41.206
1391402_at	301436	RGD15648	similar to potassium channel tetrameris	88.57636	48.83877	43.79434
1369288_at	113983	Pitx1	paired-like homeodomain transcription	88.50613	43.83282	54.1776
1370712_at	286957	Vnr2	vomeronasal receptor 2	88.43464	15.10257	43.60051
1368028_at	24688	Prph1	peripherin 1	88.41956	33862.68	225.9013
1394730_at	295362	Gstm6_pre	glutathione S-transferase, mu 6 (predic	88.30843	24.08903	28.56057
1373219_at	116490	Snai1	snail homolog 1 (Drosophila)	88.30775	240.1357	101.2722
1373567_at	299637	RGD15615	similar to Tle6 protein (predicted)	88.27978	48.75203	150.9718
1397629_at	498721	NA	NA	88.23403	24.63287	103.003
1369652_at	24832	Thy1	thymus cell antigen 1, theta	88.23339	2435.943	445.1369
1394761_at	300350	NA	NA	88.16333	165.3162	622.565
1368796_at	78976	Kiss1r	KISS1 receptor	88.15543	13.16019	7.958733
1393655_at	298429	Rad54l_pre	RAD54 like (S. cerevisiae) (predicted)	88.0592	1265.486	127.1308
1371169_at	289526	Vcsa2	variable coding sequence A2	87.88505	31.90379	13.36078
1387562_at	29520	Padi3	peptidyl arginine deiminase, type III	87.8746	46.30692	41.64593
1370456_at	191571	Fat3	FAT tumor suppressor homolog 3 (Dro	87.81445	24.38958	88.97846
1370926_at	303887	Muc4	mucin 4	87.77626	22.69101	10.53357
1378702_at	365446	NA	NA	87.76285	243.3728	62.29451
1368534_at	29413	Adra1d	adrenergic receptor, alpha 1d	87.72894	67.47288	72.87645
1368459_at	79216	Gdf10	growth differentiation factor 10	87.68833	91.10952	277.5995
1392267_at	312257	RGD13061	similar to RIKEN cDNA B930096L08 (p	87.47252	161.7952	591.5496
1392172_at	360579	Ccl9	chemokine (C-C motif) ligand 9	87.4668	6.697304	51.48292
1370391_at	29563	Crabp2	cellular retinoic acid binding protein 2	87.32291	55.64926	326.9956

1387542_at	24784	Slc9a3	solute carrier family 9 (sodium/hydroge	87.23314	30.96375	52.44061
1379760_at	310663	Tnfaip8l2	tumor necrosis factor, alpha-induced p	87.20972	106.9898	39.29791
1377175_at	316602	RGD13058	similar to hypothetical protein A730008	87.1624	1707.819	128.5094
1390518_at	498403	RGD15658	similar to putative emu1 protein (predic	87.15885	148.7634	90.32607
1389441_at	207118	Abp10	annexin V-binding protein ABP-10	87.07761	8.771447	130.4215
1385086_at	296137	Bub1_pred	budding uninhibited by benzimidazoles	86.91967	5697.324	283.5769
1379035_at	500308	RGD15641	similar to Cat eye syndrome critical reg	86.9037	118.9733	38.33519
1372359_at	306527	RGD13100	similar to 2310043K02Rik protein	86.88835	591.5163	256.2245
1376377_at	502024	NA	NA	86.88173	414.8942	190.5744
1368283_at	171142	Ehhadh	enoyl-Coenzyme A, hydratase/3-hydro	86.86421	14.94692	69.98009
1378803_at	309095	Nkx6-2_pre	NK6 transcription factor related, locus	86.84974	9.103328	11.86522
1370414_at	252916	Rab38	Rab38, member of RAS oncogene fam	86.83911	48.36539	42.40548
1398650_at	500519	NA	NA	86.8024	10.88843	139.1951
1380812_at	304885	RGD13111	similar to RIKEN cDNA C530043G21 g	86.7696	92.65214	53.46461
1370471_at	24657	Prlpb	prolactin-like protein B	86.73281	30.20059	95.67529
1369910_at	246075	Prlhr	prolactin releasing hormone receptor	86.59147	39.05792	42.13179
1380929_at	302996	RGD15602	similar to hypothetical protein FLJ3451	86.43534	169.3034	246.3259
1392694_at	295337	RGD15623	similar to Gm566 protein (predicted)	86.38352	22.24154	103.2657
1391722_at	499413	RGD15658	similar to heat shock transcription facto	86.36501	112.3138	136.7409
1385182_at	304822	Pkp1_pred	plakophilin 1 (predicted)	86.35563	125.4273	22.47831
1369289_at	25735	Hnf4a	hepatocyte nuclear factor 4, alpha	86.3501	545.613	162.6156
1370436_at	246263	LOC24626	kidney-specific protein (KS)	86.23181	141.3578	159.1088
1387112_at	24943	Plp	proteolipid protein	86.17825	258.0059	1394.918
1372047_at	499906	RGD15599	similar to Eukaryotic translation initiatio	86.13826	10.38883	6.251919
1369556_at	114098	Ltb4r2	leukotriene B4 receptor 2	86.0459	65.95458	23.65693
1369759_at	25552	Slc5a1	solute carrier family 5 (sodium/glucose	85.99757	9.802307	21.82172
1385232_at	313045	LOC31304	NA	85.9809	256.7842	272.0957
1371545_at	29583	Pecam	platelet/endothelial cell adhesion mole	85.93896	96.06618	248.1469
1389781_at	315670	Elmod1_pr	ELMO domain containing 1 (predicted)	85.87215	642.1745	7427.643
1380958_at	114850	Wnt7a	wingless-related MMTV integration site	85.7978	47.70024	42.11898
1393865_at	366791	Rdh5_pred	retinol dehydrogenase 5 (predicted)	85.75474	45.3236	27.66109
1393319_a_at	503269	RGD15624	similar to RAB17, member RAS oncog	85.7513	28.35924	91.99726
1392798_at	287164	Pdia2_prec	protein disulfide isomerase associated	85.67183	48.88091	139.0606
1386869_at	25365	Actg2	actin, gamma 2	85.60073	150.743	147.5853
1370676_at	155012	Cfh	complement component factor H	85.54535	1157.133	34.77442
1392726_at	307852	RGD13083	similar to pyruvate dehydrogenase pho	85.521	231.823	166.7183
1390822_at	307614	Cdh16	cadherin 16	85.50131	33.26859	72.53205
1393691_at	314473	Kif26a_pre	kinesin family member 26A (predicted)	85.49723	999.322	84.37009
1385525_at	500336	RGD15635	similar to C-type lectin-like receptor 2 (	85.44893	85.34188	45.39463
1369850_at	63867	Ugt2a1	UDP glucuronosyltransferase 2 family,	85.44023	89.50376	63.17883
1371677_at	301016	RGD13071	similar to Esophagus cancer-related ge	85.42039	116.1537	38.9837
1372794_at	300799	Dapk2	death-associated kinase 2	85.32328	65.04263	60.26464
1386205_at	314323	LOC31432	transporter	85.17548	53.04144	231.7098
1368349_at	64535	Fgfbp1	fibroblast growth factor binding protein	85.01327	18.3138	75.08116
1387688_at	64354	Htr6	5-hydroxytryptamine (serotonin) recep	84.71386	119.5942	43.29217
1387166_at	59110	Aipl1	aryl hydrocarbon receptor-interacting p	84.66296	85.36972	116.6084
1378942_at	499627	RGD15646	RGD1564666 (predicted)	84.61164	102.1019	140.8123
1391446_at	309217	Ms4a1_pre	membrane-spanning 4-domains, subfa	84.60296	21.54908	17.70877
1392274_at	79429	Pdgfc	platelet-derived growth factor, C polyp	84.52503	98.03648	876.475
1387949_at	171518	Cyp2c70	cytochrome P450, family 2, subfamily	84.52119	9.100852	15.74046
1377982_at	293774	Dtx4	deltex 4 homolog (Drosophila)	84.47094	166.4796	82.28039

1368205_at	79126	Cfi	complement factor I	84.37929	104.8991	253.5707
1387220_at	54272	Mcpt9	mast cell protease 9	84.3397	22.00579	17.52548
1395824_at	499528	RGD15652	similar to uracil-DNA glycosylase 2 (pr	84.25693	124.7149	81.85195
1398404_at	408201	Ctsql2	cathepsin Q-like 2	84.21099	20.39253	143.4718
1387216_at	29634	Ldhc	lactate dehydrogenase C	84.17456	39.3276	32.14686
1369480_at	65200	Slc16a8	solute carrier family 16, member 8	84.14942	19.17353	36.83468
1396999_at	170570	Acot12	acyl-CoA thioesterase 12	84.1087	152.6971	22.08936
1395497_at	499310	RGD15608	similar to cell division cycle associated	84.096	59.67784	227.0042
1384748_at	313755	Mell1_pred	mel transforming oncogene-like 1 (pred	84.08087	41.77172	217.1098
1392950_at	291605	Myot_predi	myotilin (predicted)	84.06054	155.3801	28.44023
1396499_at	300201	NA	NA	83.95821	115.8698	24.31215
1393850_at	501121	RGD15621	similar to CRYPTIC (predicted)	83.80699	39.93522	33.91702
1380756_at	498317	NA	NA	83.7712	51.50272	45.60428
1381425_at	303901	Sema5b_p	sema domain, seven thrombospondin	83.72118	48.2537	37.24409
1377367_at	293997	Kazald1	Kazal-type serine peptidase inhibitor d	83.71143	186.58	350.4846
1397341_at	290326	Pbk_predic	PDZ binding kinase (predicted)	83.59992	3666.982	127.7451
1385243_at	54267	Maf	v-maf musculoaponeurotic fibrosarcom	83.59586	156.9417	443.9862
1384782_at	361551	Abpa	androgen binding protein, alpha	83.54283	17.56573	20.52548
1387528_at	64668	Mbl2	mannose binding lectin 2 (protein C)	83.47844	119.8791	54.41017
1369672_at	29624	Alox5ap	arachidonate 5-lipoxygenase activating	83.43796	61.78382	60.67156
1369137_at	29422	Chrne	cholinergic receptor, nicotinic, epsilon	83.43271	56.3222	226.8951
1373296_a_at	293155	RGD13116	similar to RIKEN cDNA 3200002M19 (	83.42813	1582.882	296.6087
1369282_at	117516	Tnfsf11	tumor necrosis factor (ligand) superfan	83.40233	98.14425	102.3654
1397860_at	299741	NA	NA	83.39431	65.58806	75.45883
1387733_at	29238	Drd3	dopamine receptor D3	83.26661	177.5825	115.4781
1369486_at	170578	Asz1	ankyrin repeat, SAM and basic leucine	83.22401	115.1081	48.20216
1382167_at	500449	LOC50044	similar to SHP2-interacting transmemb	83.16463	170.7457	159.6157
1391919_at	361669	RGD15624	similar to transcription elongation regu	83.10439	384.3267	255.3122
1396443_at	360875	NA	NA	83.09269	31.70006	25.14329
1369596_at	116562	Il2	interleukin 2	83.09033	39.59874	82.56491
1394911_at	298383	NA	NA	82.94221	12.53509	58.89074
1374965_at	364190	RGD13061	similar to RRP22 (predicted)	82.9127	232.0777	282.9985
1384241_at	296730	Crygn_pred	crystallin, gamma N (predicted)	82.8812	42.9443	30.94178
1369781_at	81672	Grm7	glutamate receptor, metabotropic 7	82.8725	19.59531	22.33194
1376955_at	301562	Col4a4	procollagen, type IV, alpha 4	82.86442	66.44562	75.25072
1368298_at	64532	Adcy5	adenylate cyclase 5	82.77317	325.7987	54.86436
1378418_at	364674	RGD13116	similar to MGC37193 protein	82.7506	7.931125	98.97174
1367782_at	25278	Cox6a2	cytochrome c oxidase, subunit VIa, po	82.73927	102.2515	79.65725
1392397_at	498333	Bmp2k	BMP-2 inducible kinase	82.63834	136.8682	477.8874
1388439_at	360627	LOC36062	similar to 65kDa FK506-binding protein	82.6035	46.4481	93.94642
1394559_at	315473	RGD15614	similar to RIKEN cDNA 9530077C05 (	82.58851	12.57961	166.3871
1393657_at	293118	Prcp_predi	prolylcarboxypeptidase (angiotensinas	82.57866	288.085	74.41998
1381572_at	308429	RGD15657	similar to RIKEN cDNA 1810065E05 (	82.48591	38.90785	39.4926
1384444_at	499102	NA	NA	82.45567	15.95029	52.6651
1382073_at	313152	Ifnk_predic	interferon, kappa (predicted)	82.40295	31.34211	106.3402
1384098_at	361296	Rnf125_pre	ring finger protein 125 (predicted)	82.39755	38.53349	342.6816
1381971_at	311723	Sox18	SRY-box containing gene 18	82.31821	95.31596	169.2856
1369274_a_at	60396	Cdkl3	cyclin-dependent kinase-like 3	82.27169	35.95566	289.0436
1390421_at	293949	RGD13104	similar to RIKEN cDNA 0610010D20 (	82.2651	8.58416	428.8753
1382926_s_at	499533	NA	NA	82.26454	36.68096	136.1457
1369464_at	85271	Zp1	zona pellucida glycoprotein 1	82.2572	6.63866	56.44635

1368896_at	81516	Madh7	MAD homolog 7 (Drosophila)	82.11219	228.688	1576.103
1370647_at	245919	Impg2	interphotoreceptor matrix proteoglycar	82.07102	15.15124	77.85656
1384789_at	302991	Tekt4	tektin 4	81.96997	226.5734	135.1183
1381849_at	500329	RGD1560(	similar to tubby like protein 3 (predicte	81.95137	106.4963	92.81488
1371082_at	171107	Arr3	arrestin 3, retinal	81.94768	48.93052	27.95501
1374730_at	361537	Tyrobp	Tyro protein tyrosine kinase binding pr	81.89353	127.5984	270.2387
1387313_at	81523	Myoc	myocilin	81.83718	175.672	238.7921
1388257_at	117284	Lrrc7	leucine rich repeat containing 7	81.75473	86.27532	179.7321
1369843_at	79557	Chrna1	cholinergic receptor, nicotinic, alpha po	81.71942	10.68753	26.03347
1369575_at	78982	Tas2r5	taste receptor, type 2, member 5	81.65309	31.54669	23.39334
1384111_at	367264	Stat4	signal transducer and activator of trans	81.64407	157.7935	59.52139
1374097_at	366126	Sfpi1	SFFV proviral integration 1	81.61397	94.82594	95.09095
1387551_at	170739	Kcnh7	potassium voltage-gated channel, subf	81.56728	168.3406	14.21012
1368364_at	24899	Mcsp	mitochondrial capsule selenoprotein	81.56248	288.6112	118.6575
1375709_at	361710	Sipa1	signal-induced proliferation-associated	81.53662	756.3015	91.99044
1367554_at	25010	Scgb2a1	secretoglobin, family 2A, member 1	81.48209	96.746	19.55123
1391856_at	290562	Sema3g	sema domain, immunoglobulin domair	81.47456	759.0408	391.246
1375367_at	290354	Pdlim2	PDZ and LIM domain 2	81.46763	118.3467	39.95956
1376509_at	500919	NA	NA	81.37657	266.4019	70.32808
1385950_at	500102	LOC50010	hypothetical protein LOC500102	81.20274	164.391	84.97673
1394464_at	500352	RGD1560€	RGD1560652 (predicted)	81.19965	21.12756	61.89118
1380628_at	303528	Krt1-4	keratin complex 1, acidic, gene 4	81.14145	55.5681	140.9084
1387040_at	25263	Mal	myelin and lymphocyte protein, T-cell (	81.12264	33.03303	22.17905
1396673_at	367184	NA	NA	81.04949	76.52069	344.3273
1395447_at	295619	RGD13083	similar to KIAA1189 protein	80.91554	21.04214	48.4569
1368905_at	171118	Ces2	carboxylesterase 2 (intestine, liver)	80.83468	128.8206	44.25761
1369664_at	25107	Avpr1a	arginine vasopressin receptor 1A	80.72722	25.5732	32.49441
1369141_at	53950	Csh1	chorionic somatomammotropin hormor	80.68703	29.72135	21.32316
1368770_at	64043	Gcnt1	glucosaminyl (N-acetyl) transferase 1,	80.66941	85.69177	129.0698
1387769_a_at	25585	Id3	inhibitor of DNA binding 3	80.53962	165.1748	549.6903
1393744_at	304181	Tssk2	testis-specific serine kinase 2	80.49219	117.0783	31.26349
1390304_at	292808	Pepd_map	peptidase D (mapped)	80.22511	28.10413	125.9757
1369106_at	29575	Tcea2	transcription elongation factor A (SII), 2	80.14962	748.0991	314.5232
1370185_at	84008	Cntnap1	contactin associated protein 1	80.14596	79.51139	87.55697
1377429_at	287610	Lpo_predic	lactoperoxidase (predicted)	80.12057	18.80648	47.21972
1369053_at	24805	Syt2	synaptotagmin II	79.93457	162.539	211.456
1385842_at	362076	Il1f8_predi	interleukin 1 family, member 8 (predict	79.87148	51.23867	65.22508
1391761_at	289032	Chit1_pred	chitinase 1 (chitotriosidase) (predicted)	79.86747	28.18363	191.3505
1368448_at	59106	Ltbp2	latent transforming growth factor beta l	79.81604	38.93868	93.36778
1378196_at	311168	Slc43a1_pr	solute carrier family 43, member 1 (pre	79.79117	12.55997	87.89072
1390137_at	311325	Traf4af1	TRAF4 associated factor 1	79.24437	10448.29	61.87958
1387993_at	292728	Cyp2b13	cytochrome P450, family 2, subfamily l	79.20276	23.85036	29.33658
1393607_at	79220	Grid2	glutamate receptor, ionotropic, delta 2	79.1546	275.657	711.1911
1370424_at	257651	Prpg2	proline-rich proteoglycan 2	79.03717	98.69388	56.40995
1379883_at	315870	Tbx18_pre	T-box18 (predicted)	78.96187	38.73234	113.0395
1387838_at	24528	Lalba	lactalbumin, alpha	78.90832	83.27412	104.0164
1369459_at	89812	Pip5k2b	phosphatidylinositol-4-phosphate 5-kin	78.83585	199.8654	14.7303
1379246_at	362120	RGD13087	similar to Complement C5 precursor (p	78.7112	92.96644	45.61332
1398241_a_at	29229	Spt1	salivary protein 1	78.6065	49.0122	97.09762
1374626_at	367455	Lrg1	leucine-rich alpha-2-glycoprotein 1	78.43917	102.833	116.961
1368697_at	25440	Fabp6	fatty acid binding protein 6, ileal (gastr	78.32336	26.99309	21.81078

1369328_at	116719	Acacb	acetyl-Coenzyme A carboxylase beta	78.30068	9.123542	14.06252
1387568_at	65146	Pirb	paired-Ig-like receptor B	78.26463	11.72074	175.5164
1371280_at	497995	LOC49799	NA	78.21957	37.85359	88.80669
1381034_at	316527	Wnt10a_pr	wingless related MMTV integration site	78.19761	189.0733	11.38812
1387439_at	65049	Egfl3	EGF-like-domain, multiple 3	78.19682	113.652	84.21109
1383413_at	301073	Gup1_prec	Gup1, glycerol uptake/transporter hom	78.16044	26.43381	96.28601
1368454_at	84377	Plfr	proliferin related protein	78.12589	138.536	118.3774
1378692_at	81819	Pax8	paired box gene 8	78.11052	179.6262	104.8845
1372162_at	296259	Acss1_pre	acyl-CoA synthetase short-chain family	77.99649	35.04368	707.5624
1372621_at	362597	RGD13088	similar to 2610027C15Rik protein (pre	77.99364	47.04013	280.862
1369616_at	170579	Fgf22	fibroblast growth factor 22	77.99343	77.96712	32.55421
1368007_at	170568	Dmbt1	deleted in malignant brain tumors 1	77.99212	18.34379	30.73784
1370425_at	298109	LOC29810	NA	77.98573	56.30429	21.58664
1369685_at	59327	Twist2	twist homolog 2 (Drosophila)	77.98268	12.96458	9.265375
1390167_at	361528	RGD13062	similar to hypothetical protein FLJ3037	77.95349	19.87047	92.87764
1369139_at	25343	Pdc	phosducin	77.94873	44.77398	23.78054
1384978_at	292858	Klk1c10	T-kininogenase	77.88135	99.07103	27.34585
1369612_at	60591	LOC60591	calcium-activated potassium channel b	77.81967	100.6937	47.00743
1369713_at	25706	Cckbr	cholecystokinin B receptor	77.59688	62.17945	47.33565
1371566_at	363083	RGD13118	similar to F-box protein FBL2 (predicte	77.57459	64.96908	45.82019
1391317_at	291441	RGD13107	similar to RIKEN cDNA 2810433K01 (p	77.41736	3223.198	50.56211
1369016_at	50938	Cdon	cell adhesion molecule-related/down-re	77.35792	132.9327	61.32373
1381738_at	314213	Gpr135	G protein-coupled receptor 135	77.30894	54.11219	24.78596
1368757_at	114639	Zp3	zona pellucida glycoprotein 3	77.30313	99.68844	27.79584
1382955_at	308376	Gpr126_pr	G protein-coupled receptor 126 (predic	77.21209	94.48435	159.5438
1388138_at	29220	Thbs4	thrombospondin 4	77.19032	48.94076	426.9847
1373596_at	361112	RGD13104	similar to hypothetical protein FLJ3173	77.17433	8.748668	80.73615
1370012_at	25527	Ptgis	prostaglandin I2 (prostacyclin) synthas	77.17013	37.19513	168.5306
1369394_at	60629	Unc5a	unc-5 homolog A (C. elegans)	77.14369	341.9099	74.70761
1374356_at	302999	LOC30299	NA	77.05658	61.43701	176.6924
1380055_at	498951	LOC49895	NA	77.03526	35.56968	155.5504
1373493_at	300016	Slurp1_pre	secreted Ly6/Plaur domain containing	77.01439	20.90151	7.145096
1387273_at	25556	Il1rl1	interleukin 1 receptor-like 1	76.90414	90.05105	214.0739
1377058_at	500973	RGD15641	similar to solute carrier family 37 (glyce	76.85376	36.91765	35.12558
1371369_at	361821	Col6a2	procollagen, type VI, alpha 2	76.83281	210.8332	852.038
1378165_at	85489	Twist1	twist gene homolog 1 (Drosophila)	76.83099	915.2577	97.85351
1385451_at	116696	St8sia4	ST8 alpha-N-acetyl-neuraminide alpha	76.75921	62.98589	19.40633
1368465_at	25364	Accn1	amiloride-sensitive cation channel 1, n	76.74163	368.1381	41.8319
1373666_at	362799	Rapgef5	Rap guanine nucleotide exchange fact	76.6366	1668.421	723.9096
1387680_at	29691	Pde1b	phosphodiesterase 1B, Ca2+calmodul	76.62665	63.99314	20.4684
1368178_at	65144	Pdzk1	PDZ domain containing 1	76.54814	95.87886	63.00014
1382776_at	498741	RGD15655	similar to O-acyltransferase (membran	76.53364	1024.136	204.6252
1374247_at	290559	Stab1_pre	stabilin 1 (predicted)	76.51817	25.60097	114.9228
1393788_at	366219	Cst10_pre	cystatin 10 (chondrocytes) (predicted)	76.50979	11.03193	119.4866
1369436_at	64574	Chrna10	cholinergic receptor, nicotinic, alpha po	76.3822	35.89947	76.20533
1393246_at	363139	Zmynd10	zinc finger, MYND domain-containing 1	76.34578	24.76578	445.7788
1380354_at	302962	Zfp206_pre	zinc finger protein 206 (predicted)	76.33001	42.48093	148.4005
1376928_at	287899	RGD15659	similar to A disintegrin-like and metallo	76.31935	133.7668	89.88825
1398589_at	363897	LOC36389	NA	76.10757	46.17566	115.8079
1393840_at	365901	Gnat2_pre	guanine nucleotide binding protein, alp	76.06757	34.92003	34.09057
1368723_at	81511	Lat	linker for activation of T cells	76.01586	73.13259	287.5271

1379179_at	317253	RGD15641	similar to novel protein similar to multic	75.99446	219.3173	279.6987
1368666_a_at	170641	Lphn3	latrophilin 3	75.9821	113.3797	69.8208
1368300_at	25369	Adora2a	adenosine A2a receptor	75.95895	466.2812	523.1115
1390705_at	366517	RGD13048	similar to RIKEN cDNA B230396O12 (	75.95158	16.00968	21.24788
1371168_at	85275	Mpp2	membrane protein, palmitoylated 2 (M/	75.59697	262.9954	135.6514
1385083_at	500434	RGD15630	similar to hypothetical protein (predicte	75.50253	59.63703	738.3165
1368461_at	83500	Slc22a8	solute carrier family 22 (organic anion	75.49222	43.0667	163.7578
1384679_at	365946	RGD13050	similar to RIKEN cDNA 4933425K02 (p	75.48401	19.95862	16.17393
1382996_at	499867	RGD15662	similar to RIKEN cDNA 1810007E14 (p	75.47178	107.7193	105.9297
1387727_at	25753	Chrng	cholinergic receptor, nicotinic, gamma	75.24982	46.86295	36.04765
1392977_at	497933	LOC49793	NA	75.12156	139.3774	158.2523
1377898_at	362812	Slc39a5_pr	solute carrier family 39 (metal ion trans	75.08624	114.7806	15.89695
1369403_at	25372	Adrbk2	adrenergic receptor kinase, beta 2	75.08499	221.1491	30.12329
1367552_at	24802	Svp4	seminal vesicle protein 4	75.01359	96.03742	99.22937
1376544_at	310806	Cdc14a_pr	CDC14 cell division cycle 14 homolog	74.98767	6.031269	29.05271
1375040_at	366998	Nfe2	nuclear factor, erythroid derived 2	74.94855	21.46636	37.58659
1378859_at	365900	Alx3	aristaless 3	74.94806	40.20792	23.82175
1381799_at	498764	RGD15620	similar to OTTHUMP00000046255 (pre	74.8123	63.25735	45.04882
1380706_at	310538	RGD15621	similar to mKIAA1450 protein (predicte	74.73376	106.7903	17.65083
1371942_at	499422	RGD15627	similar to Glutathione S-transferase, th	74.69373	31.40773	289.7467
1382734_at	29147	Jag2	jagged 2	74.68943	1697.005	224.094
1385288_at	298398	RGD13104	hypothetical LOC298398	74.6702	152.5377	169.6455
1371128_at	287287	Il4	interleukin 4	74.65645	21.46135	24.69198
1370132_at	58950	Fkbp1b	FK506 binding protein 1b	74.56137	488.1515	64.96826
1395663_at	362211	Mall	mal, T-cell differentiation protein-like	74.48734	24.07011	189.9981
1392298_at	313772	Centb5_pre	centaurin, beta 5 (predicted)	74.4841	346.6745	52.90489
1368731_at	24614	Orm1	orosomucoid 1	74.46201	105.5487	50.35625
1389007_at	309415	RGD13075	similar to Friedreich ataxia region gene	74.45814	329.49	17.86486
1379732_at	292483	Stx11	syntaxin 11	74.39178	116.3939	337.2584
1390349_at	117088	Adarb2	adenosine deaminase, RNA-specific, B	74.37124	81.6757	292.7713
1367813_at	114004	Ppp1r14a	protein phosphatase 1, regulatory (inhi	74.36285	339.3412	57.55492
1391736_at	300215	Lmbr1l	limb region 1-like homolog (mouse)	74.27662	161.388	49.3175
1387576_at	64702	Trim17	tripartite motif protein 17	74.27458	14.13891	75.16054
1380160_at	497847	NA	NA	74.18109	101.069	76.42421
1367570_at	25123	Tagln	transgelin	74.1714	8.781891	35.25297
1384669_at	287796	Slc39a11	solute carrier family 39 (metal ion trans	74.02396	36.30682	88.0339
1379491_at	288271	Mrap_pred	melanocortin 2 receptor accessory pro	74.0104	8.434587	18.45166
1387478_at	25646	Otx1	orthodenticle homolog 1 (Drosophila)	73.97728	138.4952	35.224
1374469_at	502689	RGD15613	similar to C20orf118 (predicted)	73.94898	124.4894	44.76504
1393742_at	300121	Smc1l2_pr	SMC (structural maintenace of chromo	73.90138	62.02974	84.01449
1391137_at	295265	RGD13593	similar to hypothetical protein FLJ2051	73.88184	81.52538	26.70806
1394323_at	501164	RGD15625	similar to cyclin-dependent kinase 5 ac	73.87945	59.84963	124.9692
1370125_at	29331	Hapln1	hyaluronan and proteoglycan link prote	73.87058	27.40934	66.82032
1371700_at	287382	Mfap4	microfibrillar-associated protein 4	73.84621	18.1621	152.8407
1377796_at	317674	Fzd5	frizzled homolog 5 (Drosophila)	73.76758	17.97811	127.4074
1391468_at	292264	Myct1_pred	myc target 1 (predicted)	73.66132	19.61625	87.79368
1381474_at	302492	Mbnl3_pred	muscleblind-like 3 (Drosophila) (predic	73.60344	162.5895	27.3901
1368708_a_at	24318	Drd2	dopamine receptor 2	73.59737	136.1989	100.9101
1385129_at	296130	RGD13049	similar to mitochondrial glycerol 3-phos	73.55115	95.00237	147.443
1376185_at	294286	Kifc1	kinesin family member C1	73.45333	5925.87	319.0909
1370726_at	286893	Vnr1	vomeronasal receptor 1	73.39911	32.58863	22.39303

1387742_at	60463	Ccr2	chemokine (C-C motif) receptor 2	73.38983	86.7017	53.69933
1387434_at	64037	Slc22a4	solute carrier family 22 (organic cation	73.36728	20.67587	102.3841
1387813_at	24337	Erbb2	v-erb-b2 erythroblastic leukemia viral c	73.28573	60.97673	26.77777
1368135_at	59115	Ninj2	ninjurin 2	73.27368	25.73702	18.44115
1387648_at	60665	Cxcl5	chemokine (C-X-C motif) ligand 5	73.00059	20.75845	141.6893
1370729_at	192352	Ss18l1	synovial sarcoma translocation gene o	72.97143	26.07804	17.16288
1390355_at	114207	Ryr1	ryanodine receptor 1, skeletal muscle	72.931	78.11754	96.6083
1369534_at	171040	Il11	interleukin 11	72.7281	230.1808	38.4622
1387633_at	58826	Prg2	proteoglycan 2, bone marrow	72.7093	18.60365	89.58144
1380181_at	360858	Lhx4_predi	LIM homeobox protein 4 (predicted)	72.6911	81.61166	67.73715
1384722_at	296290	Bpil1_pred	bactericidal/permeability-increasing pr	72.60855	37.6593	18.56404
1376898_at	361140	RGD1561€	similar to hypothetical protein FLJ2003	72.44268	32.52152	158.0946
1390404_at	309368	Lama2_pre	laminin, alpha 2 (predicted)	72.40889	12.93275	601.7582
1384612_at	24664	Pomc	pro-opiomelanocortin	72.34934	26.47789	82.57061
1370027_a_at	497794	Mug1	Murinoglobulin 1 homolog (mouse)	72.23953	20.50159	1538.366
1388356_at	361991	S100a16_p	S100 calcium binding protein A16 (pre	72.17497	85.12611	208.9612
1374471_at	498972	LOC49897	NA	72.156	370.1429	138.0722
1392264_s_at	24617	Serpine1	serine (or cysteine) peptidase inhibitor	72.12495	32.45748	165.697
1370221_at	65154	Wisp1	WNT1 inducible signaling pathway pro	72.03397	40.85604	17.22266
1375457_at	311748	Metapl1_pr	methionine aminopeptidase-like 1 (pre	71.98723	118.0978	296.5093
1392329_at	260325	Homez	homeodomain leucine zipper-encoding	71.76565	92.22036	167.3172
1386162_at	305311	Txk	TXK tyrosine kinase	71.63557	171.2156	36.90431
1391198_at	363085	Car12	carbonic anyhydrase 12	71.59709	52.64813	84.37418
1370289_x_at	292868	Klks3	kallikrein, submaxillary gland S3	71.56613	71.39242	34.48533
1386691_at	363268	Fbxo36_pr	F-box only protein 36 (predicted)	71.32708	26.44608	183.0205
1387193_a_at	24833	Spink1	serine protease inhibitor, Kazal type 1	71.25902	14.05266	782.4973
1384570_at	308584	Plekha4	pleckstrin homology domain containing	71.24901	52.38368	39.69666
1372097_at	292060	Irf8	interferon regulatory factor 8	71.23217	46.01991	263.4967
1388275_at	24820	Tcrb	T-cell receptor beta chain	71.20035	108.8745	31.38478
1374683_at	305941	Sgcg	sarcoglycan, gamma (dystrophin-asso	71.16686	38.07173	24.11937
1372613_at	295458	Bdh2_pred	3-hydroxybutyrate dehydrogenase, typ	71.14819	57.22694	37.12183
1398576_at	310212	NA	NA	71.00541	75.93418	156.5476
1398291_at	83819	Slc26a5	solute carrier family 26, member 5	70.99951	151.8191	31.32506
1391669_at	314843	Ptprb_pred	protein tyrosine phosphatase, receptor	70.96566	26.10684	78.75795
1375830_at	315668	Rab39_pre	RAB39, member RAS oncogene family	70.95709	17.32267	130.0145
1382967_at	266735	Gpr64	G protein-coupled receptor 64	70.82369	243.0407	463.7969
1369168_a_at	60447	Clock	clock homolog (mouse)	70.77559	244.4127	24.63696
1385311_at	312907	Slco5a1_pr	solute carrier organic anion transporter	70.53207	48.11413	38.87421
1387914_at	301517	Cyp27a1	cytochrome P450, family 27, subfamily	70.48516	9.126171	507.1341
1369295_at	24864	Pgc	progastricsin (pepsinogen C)	70.45551	23.00561	193.1467
1380403_at	363110	Rwdd2_pre	RWD domain containing 2 (predicted)	70.42975	142.9497	203.2065
1368357_at	114032	Kcnh4	potassium voltage-gated channel, subf	70.3848	24.23648	171.6678
1380484_at	294312	Pi16_predi	protease inhibitor 16 (predicted)	70.34549	46.06665	56.59575
1370013_at	85259	Cnga1	cyclic nucleotide gated channel alpha	70.07547	740.036	23.69588
1369036_at	54257	Grik2	glutamate receptor, ionotropic, kainate	69.97968	122.896	556.8341
1377308_a_at	29613	Ntrk3	neurotrophic tyrosine kinase, receptor,	69.96703	2595.334	113.2919
1368892_at	24166	Adcyap1	adenylate cyclase activating polypeptic	69.92908	23.3622	69.97521
1378714_at	363112	RGD13098	similar to hypothetical protein BC0100	69.88851	193.7798	278.3832
1391782_at	365881	RGD13055	similar to hypothetical protein FLJ2512	69.83687	19.37948	42.54171
1378402_at	298555	Tfcp2l4_pr	transcription factor CP2-like 4 (predicte	69.75649	129.926	57.6607
1367692_at	25540	Sbp	spermine binding protein	69.70859	78.8754	21.11501

1368130_at	25375	Aldh3a1	aldehyde dehydrogenase family 3, me	69.6649	17.15785	94.02936
1395194_at	362591	Mtf1_predi	metal response element binding transc	69.64611	14.66436	38.85401
1387267_at	81737	Ntf3	neurotrophin 3	69.63589	162.5286	75.22558
1378374_at	290372	RGD15601	similar to expressed sequence AU021	69.59413	91.05109	157.6331
1368789_at	56780	Acpp	acid phosphatase, prostate	69.47816	11.75366	16.50007
1391653_at	29709	Gabrg2	gamma-aminobutyric acid A receptor,	69.38299	625.9995	893.8604
1383513_at	295475	Neurog2_p	neurogenin 2 (predicted)	69.38191	8.836613	39.66893
1368134_a_at	25084	Il4ra	interleukin 4 receptor, alpha	69.38052	17.82021	91.46505
1376287_at	362701	Capn13	calpain 13	69.28372	124.0986	234.4664
1389453_at	287726	Rdm1_pred	RAD52 motif 1 (predicted)	69.14122	65.64706	266.6156
1376210_at	294515	Foxo3a_pr	forkhead box O3a (predicted)	69.05457	12.25542	147.9772
1370601_a_at	191573	Grin3a	glutamate receptor, ionotropic, N-meth	69.05142	107.5289	73.55542
1390481_a_at	360847	Ube2t_pred	ubiquitin-conjugating enzyme E2T (put	68.92961	3057.634	213.197
1396519_at	24418	Grm5	glutamate receptor, metabotropic 5	68.8795	31.7457	70.55549
1393778_at	500286	RGD15620	similar to Fls485 protein (predicted)	68.83864	138.5488	83.82453
1369987_at	114109	Nkx2-5	NK2 transcription factor related, locus	68.80081	46.36635	245.4729
1380356_at	305846	Slc39a2_pr	solute carrier family 39 (zinc transporte	68.77743	48.60098	151.3793
1388569_at	287526	Serpinf1	serine (or cysteine) peptidase inhibitor	68.77519	73.92705	559.1379
1387317_at	24221	Avp	arginine vasopressin	68.67397	39.59299	62.55256
1369526_at	25618	Acadsb	acyl-Coenzyme A dehydrogenase, sho	68.58792	89.99439	67.35328
1370553_at	25130	Epim	epimorphin	68.55789	277.4268	173.5052
1387274_at	25431	Dlx5	distal-less homeobox 5	68.50577	156.1039	80.83261
1370593_at	266682	Cyp3a11	cytochrome P450, family 3, subfamily a	68.46598	8.68517	12.652
1389812_at	494538	LOC49453	ABP beta	68.46378	67.53162	35.73842
1375876_at	293690	RGD1308C	similar to RIKEN cDNA 2410004C24 (p	68.40165	616.1184	107.8164
1377311_at	499380	RGD15647	similar to empty spiracles-like protein 2	68.34454	61.76515	26.69531
1387469_at	29235	Galr3	galanin receptor 3	68.31226	55.0695	32.68312
1381114_at	500204	RGD15625	similar to RIKEN cDNA 4931417E11 (p	68.11181	38.64164	161.1873
1369828_at	171081	Csf2rb1	colony stimulating factor 2 receptor, be	68.02179	63.12118	65.01103
1368623_at	116711	Ceacam9	CEA-related cell adhesion molecule 9	68.00839	90.77742	125.0052
1388043_at	24216	Atp4a	ATPase, H+/K+ exchanging, alpha pol	67.76401	13.47835	135.4457
1386931_at	29248	Tnni3	troponin I type 3 (cardiac)	67.76108	197.9705	58.64335
1368467_at	56266	Cyp4f2	cytochrome P450, family 4, subfamily f	67.74124	447.4742	17.93338
1397735_at	311558	Bpil3_pred	bactericidal/permeability-increasing pro	67.72002	54.91951	184.8792
1397236_at	500808	RGD15613	RGD1561305 (predicted)	67.59311	37.88839	66.4029
1375582_at	310250	Zfhx4_pred	zinc finger homeodomain 4 (predicted)	67.45006	397.0004	292.2648
1395637_at	364948	Asphd2	aspartate beta-hydroxylase domain co	67.37348	912.873	208.8476
1374735_at	246249	Arhgap4	Rho GTPase activating protein 4	67.36896	60.94445	78.44603
1377434_at	315597	Mfrp_predi	membrane frizzled-related protein (pre	67.36365	184.9368	120.2446
1370709_at	246296	Lrrc15	leucine rich repeat containing 15	67.20935	115.1956	173.888
1388222_at	301965	Tert	telomerase reverse transcriptase	67.17167	63.38877	158.6701
1375400_at	307585	Cables1_p	Cdk5 and Abl enzyme substrate 1 (pre	67.15338	38.96242	80.70002
1379935_at	287561	Ccl7	chemokine (C-C motif) ligand 7	67.11381	43.72552	952.5094
1384555_at	363326	LOC36332	NA	67.08809	49.65787	304.6873
1376012_at	500128	RGD15644	similar to hypothetical gene supported	67.06542	58.74016	53.99111
1398298_at	25323	Htr1d	5-hydroxytryptamine (serotonin) recep	66.9348	142.7232	18.29214
1397733_at	288657	RGD13105	similar to RIKEN cDNA 5730405M13 (	66.92568	42.29456	47.26821
1371732_at	289178	Dpt_predic	dermatopontin (predicted)	66.9209	55.85652	4002.268
1385740_at	361670	Lrrc27_pre	leucine rich repeat containing 27 (pred	66.72899	140.6009	66.24923
1385040_at	292757	Fbxo17	F-box only protein 17	66.72122	53.45876	41.38908
1370388_at	192215	Slc9a5	solute carrier family 9 (sodium/hydroge	66.57128	159.712	106.848

1376702_at	315215	Mlc1_predi	megalencephalic leukoencephalopathy	66.47908	147.149	14.487
1383888_at	307495	LOC30749	NA	66.43396	24.99689	172.2546
1383322_at	305302	Rasl11b	RAS-like family 11 member B	66.37166	73.78412	445.0519
1399089_at	299139	Slc38a6	solute carrier family 38, member 6	66.34997	367.0931	385.3247
1388231_at	290244	Mcpt3	mast cell peptidase 3	66.30854	46.96842	93.11401
1369826_at	60331	Atxn3	ataxin 3	66.21459	52.5249	34.09271
1395889_at	361707	Tsga10ip	testis specific 10 interacting protein	66.10989	13.65826	86.56324
1379756_at	294945	RGD1562890	RGD1562890 (predicted)	66.10265	52.70217	198.7497
1396674_at	309212	Gpr44	G protein-coupled receptor 44	66.07538	50.90284	36.50176
1389785_at	293653	Acy3	aspartoacylase (aminoacylase) 3	66.05087	34.90755	110.0035
1391147_at	367343	NA	NA	65.9853	16.70365	33.44568
1371030_at	94168	Spp2	secreted phosphoprotein 2	65.87192	1068.238	159.3889
1393210_at	291018	Ecm2_prec	extracellular matrix protein 2, female o	65.80516	87.2367	417.832
1378959_at	300742	NA	NA	65.79018	77.83295	36.06047
1374649_at	361714	Rasgrp2_p	RAS guanyl releasing protein 2 (calciu	65.69886	576.5187	107.7673
1376427_a_at	309312	Gldc_predi	glycine decarboxylase (predicted)	65.69061	50.27797	76.03179
1384353_at	317439	RGD1560€	similar to KIAA1280 protein (predicted)	65.68512	125.9075	66.88739
1367838_at	24962	Cth	cystathionase (cystathionine gamma-ly	65.59657	34.10746	10.00915
1369819_at	29138	Bsn	bassoon	65.58767	73.2996	46.44504
1394403_at	360604	Spata20	spermatogenesis associated 20	65.56581	166.9322	24.31673
1370430_at	245979	Lmo3	LIM domain only 3	65.54002	1345.311	144.188
1397972_at	311862	Lamc3_pre	laminin gamma 3 (predicted)	65.51825	15.92528	42.32889
1385175_at	303480	Hoxb13_pr	homeo box B13 (predicted)	65.50248	507.7776	13.28052
1384954_at	303218	Hs3st3b1_	heparan sulfate (glucosamine) 3-O-sul	65.42781	18.02044	61.94062
1390691_at	500359	RGD1561E	similar to Hypothetical protein MGC75(	65.40412	261.6516	188.3822
1381353_at	317297	MGC11442	similar to melanoma antigen family A,	65.36746	59.23766	118.3779
1398078_at	313040	RGD15657	similar to CUB and Sushi multiple dom	65.34744	68.41465	184.9368
1369284_at	65050	Loc65050	BarH-class homeodomain transcriptior	65.3001	99.06508	44.42366
1368713_at	117061	Mmp10	matrix metallopeptidase 10	65.29202	25.32378	21.7187
1381562_at	308587	RGD15651	similar to hypothetical protein BC0186	65.27473	66.14448	353.5046
1384964_at	301083	RGD15663	similar to Solute carrier family 6 (neurc	65.25889	153.2352	192.4807
1380956_at	317070	LOC31707	NA	65.20896	17.4844	26.60446
1381022_at	306356	Cilp2_pred	cartilage intermediate layer protein 2 (p	65.20206	104.3282	75.58801
1384754_at	362712	MGC94915	similar to hypothetical protein	65.18012	42.84081	201.4709
1368805_at	29180	Uts2	urotensin 2	65.16132	25.31486	24.35416
1380629_at	362000	NA	NA	65.07433	51.03583	9.788579
1385722_at	304071	Sim2_pred	single-minded 2 (predicted)	65.07058	33.92532	34.86519
1393416_at	360814	Rasal1_pre	RAS protein activator like 1 (GAP1 like	65.03492	197.0508	27.2038
1387671_at	81779	Sctr	secretin receptor	65.021	67.98575	533.7954
1391136_at	292866	Klk6_predi	kallikrein 6 (predicted)	64.99065	81.48916	99.1068
1368684_at	65048	Fath2	fat tumor suppressor homolog 2 (Dros	64.97648	12.53372	60.96771
1373108_at	309513	Ppp1r3c	protein phosphatase 1, regulatory (inhi	64.92143	142.2367	101.1412
1393447_at	293047	Kif7_predic	kinesin family member 7 (predicted)	64.89758	792.6518	158.2438
1389668_at	295661	Spbc25	spindle pole body component 25 homo	64.822	9283.304	317.6086
1392718_at	307380	RGD13068	similar to Hypothetical protein KIAA02	64.7203	28.38683	66.34381
1391032_at	192247	Sez6	seizure related 6 homolog (mouse)	64.69042	2161.886	333.2202
1375992_at	499765	RGD15641	similar to FLJ46082 protein (predicted)	64.50177	677.9402	569.5314
1378763_at	500947	LOC50094	hypothetical gene supported by BC088	64.47749	159.5322	272.3895
1370631_at	24620	Reg3g	regenerating islet-derived 3 gamma	64.40994	113.2332	75.5314
1374902_at	310621	Iqgap3_pre	IQ motif containing GTPase activating	64.38093	866.7366	249.3872
1369483_at	24932	Cd4	CD4 antigen	64.35679	97.08395	123.8164

1392810_at	315137	Apobec3	apolipoprotein B editing complex 3	64.24801	28.34856	244.8938
1397684_at	295445	Dkk4_pred	dickkopf homolog 4 (Xenopus laevis) (	64.22864	15.76347	88.21131
1394790_at	295586	Clcn6_pred	chloride channel 6 (predicted)	64.12382	337.0988	32.50118
1377758_at	305150	Hsd17b13	hydroxysteroid (17-beta) dehydrogena	64.07016	65.80592	148.2258
1368774_a_at	56227	Espn	espin	64.06558	1142.038	230.2019
1390892_at	310074	Depdc1b_p	DEP domain containing 1B (predicted)	64.01647	755.2018	45.44381
1392800_at	362516	RGD13081	similar to hypothetical protein MGC173	63.84563	280.0034	45.91547
1370782_a_at	257652	Prpg1	proline-rich proteoglycan 1	63.83073	14.24929	19.31386
1368704_a_at	50568	Cspg5	chondroitin sulfate proteoglycan 5	63.8268	98.37838	22.41144
1376263_at	287151	Metrn	meteorin, glial cell differentiation regula	63.67608	112.8329	125.7666
1385650_at	361476	Rshl2_pred	radial spokehead-like 2 (predicted)	63.61749	82.87347	193.303
1377168_at	297516	Cpne9	copine family member IX	63.57113	1336.861	100.601
1385064_at	298483	Lao1_pred	L-amino acid oxidase 1 (predicted)	63.56328	44.5409	61.30118
1379640_at	316432	LOC31643	Ica69-related protein	63.48082	63.84571	173.5981
1379614_at	25409	Cdh6	cadherin 6	63.3591	969.2607	133.4528
1387139_at	84029	Hao2	hydroxyacid oxidase 2 (long chain)	63.33183	29.12734	19.5191
1384734_at	288280	Ncam2	neural cell adhesion molecule 2	63.30604	160.483	253.0943
1385055_at	306883	RGD15634	similar to D0H6S2654E protein (predic	63.25197	19.40315	12.4007
1387162_at	54271	Tpsab1	tryptase alpha/beta 1	63.18914	17.18743	36.79564
1373025_at	362634	C1qg	complement component 1, q subcomp	63.01983	45.97534	42.62658
1369902_at	246142	Bmf	Bcl2 modifying factor	63.00086	38.13004	70.86829
1390230_at	25480	Mip	major intrinsic protein of eye lens fiber	62.90777	21.40002	27.30592
1388115_at	192239	Svs3	seminal vesicle secretion 3	62.90524	19.92358	66.00277
1375834_at	293711	Hrasls5	HRAS-like suppressor family, member	62.68134	30.07169	34.19583
1391041_at	363218	NA	NA	62.62209	17.53358	54.74984
1397882_at	313049	Zbtb8_pred	zinc finger and BTB domain containing	62.61153	545.0183	350.8616
1397555_at	499162	NA	NA	62.60627	83.18279	270.3315
1384064_at	287678	Krt1-23	keratin complex 1, acidic, gene 23	62.36855	37.29509	200.9726
1371225_at	66027	Drp2	dystrophin related protein 2	62.33148	186.6101	66.29791
1389092_at	140924	Il2rg	interleukin 2 receptor, gamma (severe	62.07029	14.49491	121.3922
1377023_at	311406	Dusp2	dual specificity phosphatase 2	62.06192	44.66341	97.02692
1373970_at	361749	RGD13111	similar to RIKEN cDNA 9230117N10	62.04614	388.1045	166.7577
1371144_at	60576	Treh	trehalase (brush-border membrane gly	61.99236	28.23948	17.27234
1375481_at	361630	Tead1	TEA domain family member 1	61.94859	42.79999	151.9916
1373210_at	298941	Lamb1_pre	laminin, beta 1 (predicted)	61.79311	1214.615	852.6477
1385113_at	315338	Hoxc10	homeo box C10	61.7083	561.6131	58.46606
1368464_at	64195	Mgl1	macrophage galactose N-acetyl-galact	61.61011	10.03852	28.69053
1382185_at	497886	RGD15610	similar to C1q and tumor necrosis facto	61.5961	46.36618	119.3685
1369475_x_at	114115	P2rx2	purinergic receptor P2X, ligand-gated i	61.58548	49.44983	51.74082
1376193_at	363455	Chrdl1	kohjirin	61.43295	114.5324	269.1533
1371924_at	310743	Olfml3_pre	olfactomedin-like 3 (predicted)	61.39217	20.48538	281.9149
1369920_at	24438	Hist1h1t	histone 1, H1t	61.38346	124.5334	67.64756
1369756_a_at	84484	Slc4a4	solute carrier family 4, member 4	61.37182	712.9692	48.98124
1372531_at	308918	Ppfibp2	protein tyrosine phosphatase, receptor	61.35321	282.8423	106.6729
1387738_at	24581	Mycs	myc-like oncogene, s-myc protein	61.31951	4.320381	32.32067
1392203_at	303489	Hoxb2_pre	homeo box B2 (predicted)	61.31272	83.70773	132.0934
1386385_at	361544	Etsrp71_pr	ets related protein 71 (predicted)	61.27874	83.80722	56.19081
1379319_at	502320	RGD15647	similar to RIKEN cDNA C230052I12 (p	61.1511	427.2264	46.07265
1376914_at	689490	LOC68949	NA	61.09042	1701.377	199.0205
1369558_at	64549	Inhbc	inhibin beta C	61.01495	38.68909	10.53724
1393487_at	298850	RGD15636	similar to centromere protein A (predic	61.00975	747.0763	120.8731

1387796_at	81639	Alox15	arachidonate 15-lipoxygenase	60.95592	303.0483	115.5612
1387033_at	24860	Ucp1	uncoupling protein 1 (mitochondrial, pr	60.94213	75.62113	20.00774
1387723_at	29751	Sema3a	sema domain, immunoglobulin domair	60.87356	16.1135	47.26394
1368333_at	25128	Umod	uromodulin	60.74398	43.5202	174.5781
1368077_at	24362	Fbp1	fructose-1,6- biphosphatase 1	60.71879	1447.757	117.698
1387332_at	64042	Nmur2	neuromedin U receptor 2	60.60166	24.03514	48.78955
1397263_at	310903	Adh6	alcohol dehydrogenase 6 (class V)	60.55316	113.2322	17.49585
1372280_at	299266	Asb2	ankyrin repeat and SOCS box-containi	60.54831	45.59696	77.90322
1378878_at	300517	RGD13068	similar to hypothetical protein FLJ2553	60.49829	63.157	26.71691
1375098_at	363041	Svs7_pred	seminal vesicle protein, secretion 7 (pr	60.39854	46.40312	151.3903
1389112_at	364952	Nkd1_pred	naked cuticle 1 homolog (Drosophila) (	60.3584	119.6155	177.895
1370568_at	24175	Adra2c	adrenergic receptor, alpha 2c	60.24881	28.58509	22.43171
1383991_at	304203	Lrrc8e	leucine rich repeat containing 8 family,	60.21399	19.61334	284.4378
1376959_at	310575	Sprr3_pred	small proline-rich protein 3 (predicted)	60.17774	84.67054	49.03374
1398258_at	25239	Apod	apolipoprotein D	60.09768	110.4219	26.68119
1391277_at	295211	S100a5_pr	S100 calcium binding protein A5 (pred	60.02204	29.82848	16.13248
1370932_at	83469	Lrp4	low density lipoprotein receptor-relatec	60.01989	93.50674	185.9791
1373085_at	304078	Cbr3_predi	carbonyl reductase 3 (predicted)	59.99974	1340.27	10.055
1368569_at	116463	Akr1b7	aldo-keto reductase family 1, member	59.96575	18.33385	21.37696
1391698_at	353118	MAST1	microtubule associated serine/threonir	59.96408	529.5222	29.28385
1370394_at	367586	IgG-2a	gamma-2a immunoglobulin heavy cha	59.91569	47.60146	134.2694
1375383_at	291105	RGD15616	similar to E2f3 protein (predicted)	59.89798	169.5707	9.459416
1387500_at	54252	Mid1	midline 1	59.82897	135.7968	54.0525
1387959_at	246266	LOC24626	lysophospholipase	59.67405	36.64948	29.58745
1391772_at	362337	NA	NA	59.65354	56.20776	38.23126
1390055_at	308631	RGD13081	similar to KIAA1357 protein (predicted)	59.58567	64.01739	60.92507
1369852_at	29243	F10	coagulation factor X	59.58242	18.83366	110.2015
1397739_at	361938	Pdzk6_pre	PDZ domain containing 6 (predicted)	59.51721	21.09766	24.93825
1393936_at	292890	Stk22s1	serine/threonine kinase 22 substrate 1	59.46974	34.99402	42.75697
1369874_at	25457	Gpr1	G protein-coupled receptor 1	59.37867	74.81887	43.67709
1385529_at	304050	RGD13116	similar to open reading frame 9	59.27039	9.434404	26.74742
1396456_at	315798	RGD15607	similar to suppressor of hairy wing hon	59.24298	6.182773	80.1937
1377438_at	366826	Diras1_pre	DIRAS family, GTP-binding RAS-like 1	59.22087	21.27865	80.46794
1394892_at	361516	Ceacam12	CEA-related cell adhesion molecule 12	59.14294	56.31629	64.51925
1388949_at	500992	RGD15662	similar to protein of unknown function (	59.08936	28.57724	189.082
1385120_at	302328	Pof1b_prec	premature ovarian failure 1B (predictec	58.99545	118.7688	105.4383
1370455_a_at	252920	Olfm3	olfactomedin 3	58.91404	73.98477	480.9575
1368168_at	84395	Slc34a2	solute carrier family 34 (sodium phosp	58.85194	31.92804	174.1005
1396352_at	362879	RGD15621	similar to chromosome 11 open readin	58.83122	67.74317	36.56873
1368425_at	140722	Caskin1	CASK interacting protein 1	58.81165	260.8622	247.8269
1387372_at	171163	Slc6a13	solute carrier family 6 (neurotransmitte	58.77576	17.45807	10.47077
1379899_at	289081	Glt25d2_pr	glycosyltransferase 25 domain contain	58.74486	101.7513	12.56195
1387156_at	79243	Hsd17b2	hydroxysteroid (17-beta) dehydrogena	58.74012	23.04492	41.43315
1384549_at	366790	Wibg_pred	within bgcn homolog (Drosophila) (pre	58.67576	101.7055	25.20408
1369577_at	84607	Socs2	suppressor of cytokine signaling 2	58.60171	586.4908	265.3266
1385586_at	296600	Slc2a6_pre	solute carrier family 2 (facilitated gluco	58.57875	9.926789	190.4845
1391072_at	171075	Fcer2a	Fc receptor, IgE, low affinity II, alpha p	58.47105	38.74079	20.35615
1376030_at	287443	Centb1_pre	centaurin, beta 1 (predicted)	58.46145	23.73485	34.83763
1395429_at	25302	Chrna7	cholinergic receptor, nicotinic, alpha po	58.40453	161.5938	60.13252
1394789_at	499812	NA	NA	58.39324	63.00172	37.4776
1392206_at	305865	RGD15611	similar to solute carrier family 35, mem	58.33953	129.1158	1191.306

1371166_at	24600	Nos3	nitric oxide synthase 3, endothelial cell	58.31087	13.89652	58.97905
1380852_at	294883	RGD13058	hypothetical LOC294883	58.14603	474.4687	268.3919
1392948_at	304081	CLIC6	chloride intracellular channel 6	58.13034	127.3479	213.2744
1388228_at	362657	Mthfr_pred	5,10-methylenetetrahydrofolate reduct	58.11356	37.51494	43.64626
1388840_at	292750	Plekhg2_pr	pleckstrin homology domain containing	58.10178	122.622	161.29
1369683_at	64625	Bid	BH3 interacting domain death agonist	58.09336	556.9194	56.75071
1398166_at	501183	RGD15660	RGD1566074 (predicted)	57.9571	30.07383	223.2714
1377086_at	294806	C1qtnf3_pr	C1q and tumor necrosis factor related	57.9454	52.65898	201.3267
1373590_at	296655	Stom	stomatin	57.92531	383.8783	190.5554
1370600_at	246174	Fgd4	FYVE, RhoGEF and PH domain conta	57.87616	92.63245	67.9904
1369585_at	64620	Tore	trispanning orphan	57.87593	12.77116	17.90601
1368577_at	84403	Gjb6	gap junction membrane channel protei	57.85688	4.931535	81.34244
1371447_at	360914	Plac8_pred	placenta-specific 8 (predicted)	57.84759	24034.34	266.7316
1396672_at	290156	RGD15621	similar to RIKEN cDNA A430107P09 g	57.78644	91.49787	114.0956
1385348_at	290876	LOC29087	similar to RIKEN cDNA 1700029H14	57.76046	106.978	25.95172
1398256_at	24494	Il1b	interleukin 1 beta	57.69369	76.92274	839.2989
1398008_at	364384	Znf409_pre	zinc finger protein 409 (predicted)	57.66338	29.66904	86.10483
1391112_at	246766	Ggta1	glycoprotein galactosyltransferase alpl	57.66216	104.8425	116.9777
1369399_at	25316	Ms4a2	membrane-spanning 4-domains, subfa	57.65788	100.0832	138.8464
1375857_at	309499	RGD15642	similar to Myoferlin (Fer-1 like protein 3	57.5618	124.7193	137.4918
1369827_at	171393	Clstn3	calsyntenin 3	57.55346	482.5504	119.2186
1378240_at	312641	Fancd2	Fanconi anemia D2 protein	57.49782	1829.542	292.832
1368200_at	89808	Cx3cl1	chemokine (C-X3-C motif) ligand 1	57.45801	47.69016	170.079
1380419_at	305386	RGD13096	similar to hypothetical protein FLJ9001	57.28273	199.9568	15.66845
1388188_at	25429	Cyp7b1	cytochrome P450, family 7, subfamily I	57.2318	46.14326	130.5481
1368728_at	64803	P2ry12	purinergic receptor P2Y, G-protein cou	57.22851	22.79586	72.8131
1382657_at	315824	Bmp5_pred	bone morphogenetic protein 5 (predicte	57.1352	16.4911	34.32659
1368113_at	116592	Tff2	trefoil factor 2 (spasmolytic protein 1)	57.10671	132.0304	522.2451
1393969_at	498307	NA	NA	57.06457	182.938	117.966
1387522_at	65207	Rhag	Rhesus blood group-associated A glyc	57.0586	12.63996	59.05127
1368604_at	58923	Mefv	Mediterranean fever	57.02043	18.95613	26.94183
1380083_at	292850	Klk10	kallikrein 10	56.95632	58.22595	26.34722
1368547_at	113937	Ocil	osteoclast inhibitory lectin	56.91728	304.5682	311.4819
1369497_at	24906	LOC24906	NA	56.90137	20.36816	51.72695
1369484_at	29576	Wisp2	WNT1 inducible signaling pathway pro	56.83908	69.42393	153.1773
1384033_at	309772	Vgll2_pred	vestigial like 2 homolog (Drosophila) (p	56.80738	11.64242	136.7115
1368827_at	29300	Gata6	GATA binding protein 6	56.79416	402.4195	1718.478
1379872_at	500844	NA	NA	56.53906	87.20743	87.79166
1369508_at	170847	Kcnj15	potassium inwardly-rectifying channel,	56.48955	55.59376	34.18124
1369314_at	60662	Fgf5	fibroblast growth factor 5	56.40739	22.24668	19.19187
1376910_at	292820	RGD15633	similar to Zfp536 protein (predicted)	56.39745	18.33166	20.89789
1371010_at	25628	Titf1	thyroid transcription factor 1	56.38593	51.37914	220.4667
1378871_at	313058	Bai2_predi	brain-specific angiogenesis inhibitor 2	56.34624	1024.287	481.9266
1380066_at	288562	Trfr2_predi	transferrin receptor 2 (predicted)	56.34362	108.6109	205.6163
1371069_at	64559	Nritp	ion transporter protein	56.29967	104.5189	75.22334
1374344_at	314614	Prg-2	plasticity-related protein PRG-2	56.29278	2212.257	130.9911
1368139_s_at	25586	Alpl	alkaline phosphatase, tissue-nonspeci	56.25054	22.52883	310.1222
1370797_at	171302	Foxe3	forkhead box E3	56.23756	269.0399	24.78454
1368625_at	60574	Prap1	proline-rich acidic protein 1	56.19535	80.11497	11.29333
1388278_at	24963	Hspa1l_ma	heat shock 70kD protein 1-like (mappe	56.11978	7.034337	24.18838
1390569_at	307212	Cndp1	carnosine dipeptidase 1 (metallopeptic	56.07898	119.9357	251.3234

1371413_x_at	301468	Crygb_map	crystallin, gamma B (mapped)	56.07099	10.61502	43.49152
1369808_at	117027	Ccr3	chemokine (C-C motif) receptor 3	56.02892	80.39572	83.49694
1368788_at	29195	Chad	chondroadherin	56.00688	49.85241	60.41506
1369613_at	64623	Jwa	vitamin A responsive; cytoskeleton rela	56.00513	142.9795	32.25889
1396069_at	317178	Utx_predic	ubiquitously transcribed tetratricopepti	55.98981	131.3502	191.2967
1376011_at	501789	NA	NA	55.98393	405.8294	53.10113
1375467_at	366994	NA	NA	55.89772	253.332	22.72234
1379184_at	499684	NA	NA	55.79237	41.98941	64.36343
1398280_at	66014	Impg1	interphotoreceptor matrix proteoglycar	55.78885	28.06004	13.81521
1390998_at	298732	Glis1_pred	GLIS family zinc finger 1 (predicted)	55.77345	495.2806	67.75833
1382513_at	301227	Trem2_pre	triggering receptor expressed on myel	55.75699	27.06853	24.07886
1395160_at	502873	RGD15624	similar to RIKEN cDNA B230206N24 (	55.70556	245.0711	178.1909
1380493_at	500229	RGD15619	similar to MGC22014 protein (predicte	55.67156	25.82059	112.1094
1374989_at	503446	Asb12	ankyrin repeat and SOCS box-containi	55.59236	29.13559	26.75089
1382481_a_at	311425	Adam33_p	a disintegrin and metallopeptidase dor	55.57552	70.01234	166.0412
1376114_at	307234	Kcng2	potassium voltage-gated channel, subf	55.49406	102.888	241.1627
1397447_at	500108	RGD15621	similar to zinc finger protein 398 (predi	55.42379	216.6212	224.8261
1374757_at	292666	LOC29266	similar to pregnancy-specific beta 1-gly	55.21242	31.32496	42.31462
1392171_at	89824	Chi3l1	chitinase 3-like 1	55.16981	145.109	239.3776
1382207_at	288979	LOC28897	NA	55.16758	68.98931	72.29856
1387123_at	25146	Cyp17a1	cytochrome P450, family 17, subfamily	55.13549	48.99305	133.4151
1384993_at	308894	RGD13098	similar to peptidylglycine alpha-amidat	55.1063	61.26564	490.5015
1378804_at	295649	Fign_predi	fidgetin (predicted)	55.09625	4.395051	24.32467
1370095_at	59264	Ltb4r	leukotriene B4 receptor	54.95785	81.00598	19.25254
1398255_at	60577	Slc15a2	solute carrier family 15 (H+/peptide tra	54.95562	1736.749	618.9247
1396418_at	317626	NA	NA	54.84013	63.18352	22.8825
1388051_at	114629	Slc26a3	solute carrier family 26, member 3	54.82344	94.43707	50.28499
1369080_at	25534	Rds	retinal degeneration, slow	54.792	16.23811	231.7684
1394031_at	311598	RGD15655	similar to Protein C20orf129 homolog (	54.75332	1134.994	404.7917
1395464_at	300317	RGD13111	similar to hypothetical protein FLJ1224	54.73467	286.5841	98.50812
1385229_at	306081	Pcdh20_pr	protocadherin 20 (predicted)	54.65372	468.3633	161.8972
1385207_at	289530	RGD13103	hypothetical LOC289530 (predicted)	54.62809	85.55533	49.80098
1378348_at	501201	RGD15617	similar to KIAA1913 (predicted)	54.62142	13.55086	133.4398
1376321_at	361430	Fam38a_p	family with sequence similarity 38, mer	54.56145	154.7045	45.79073
1387382_at	81676	Hnmt	histamine N-methyltransferase	54.51318	28.86385	70.06307
1398390_at	498335	LOC49833	similar to Small inducible cytokine B13	54.47959	56.10142	49.02345
1384153_at	307479	RGD15597	similar to Hypothetical protein KIAA05	54.46954	532.2032	86.50767
1395450_at	317314	RGD15615	similar to RIKEN cDNA 1700001F22 (p	54.4544	21.19779	160.2414
1397242_at	314996	LOC31499	hypothetical protein LOC314996	54.42905	43.15629	35.66497
1369598_at	25453	Gdnf	glial cell line derived neurotrophic facto	54.35239	32.39148	79.99979
1369115_at	24176	Adrb2	adrenergic receptor, beta 2	54.34484	23.12417	252.0216
1376711_at	84588	Cldn11	claudin 11	54.33701	259.6738	498.5671
1369447_at	140944	Slc28a3	solute carrier family 28 (sodium-couple	54.27305	49.00395	188.8358
1369398_at	83568	Naaladl1	N-acetylated alpha-linked acidic dipept	54.24836	46.81689	23.47604
1372208_at	360616	Ppp1r1b	protein phosphatase 1, regulatory (inhi	54.16879	47.58516	23.84078
1387697_at	171146	Kcnh5	potassium voltage-gated channel, subf	54.12453	13.26451	48.89838
1394812_at	316017	RGD13052	similar to RN49018 (predicted)	54.09307	23.41927	24.07838
1383926_at	171576	Bub1b	budding uninhibited by benzimidazoles	54.02041	2895.317	57.13116
1385654_at	362085	RGD15644	similar to helix-loop-helix protein NOHI	53.9832	64.57105	29.65622
1377930_at	306073	Kbtbd7	kelch repeat and BTB (POZ) domain c	53.87638	63.33353	45.60109
1388848_at	362790	RGD13083	similar to hypothetical protein MGC132	53.81842	89.5547	55.50721

1369322_at	171138	Kcne2	potassium voltage-gated channel, Isk-r	53.72883	224.8138	122.3349
1387106_at	64634	Sh3bp4	SH3-domain binding protein 4	53.68249	31.62484	114.8774
1370477_at	25503	Ocm	oncomodulin	53.67857	39.04664	13.74814
1369296_at	65185	Sult1c1	sulfotransferase family, cytosolic, 1C, r	53.67529	91.94934	24.08297
1395986_at	360272	Slit2	slit homolog 2 (Drosophila)	53.62718	172.1548	43.18072
1392218_at	366631	RGD15605	similar to C14orf25 protein (predicted)	53.62281	144.0249	72.24827
1393880_at	497989	NA	NA	53.60315	5.482717	48.27072
1369381_a_at	117261	Slc15a1	solute carrier family 15 (oligopeptide tr	53.49832	149.5241	132.8914
1375568_at	252971	Socs1	suppressor of cytokine signaling 1	53.42613	92.49958	55.52489
1374375_at	501196	RGD15609	similar to 2610034M16Rik protein (pre	53.42538	121.8884	181.281
1393221_at	498789	RGD15648	similar to 20-alpha-hydroxysteroid deh	53.41749	2268.822	863.939
1385012_at	500800	LOC50080	NA	53.36825	35.51017	87.47429
1370024_at	80841	Fabp7	fatty acid binding protein 7, brain	53.26283	72.57423	71.92457
1367684_at	25422	Crybb2	crystallin, beta B2	53.26255	75.53519	19.37942
1387234_at	25294	Azgp1	alpha-2-glycoprotein 1, zinc	53.19843	110.3124	105.9541
1383581_at	499752	RGD15655	similar to 60S ribosomal protein L7a (p	53.18616	1092.257	94.34229
1368384_at	29245	Klk6	kallikrein 6	53.07321	168.0065	73.0564
1384603_at	310836	Abca4_pre	ATP-binding cassette, sub-family A (AI	53.00991	42.54688	70.16788
1388940_at	500246	RGD15621	similar to Rho-GTPase-activating prote	52.99424	27.74182	12.75738
1397635_at	498209	RGD15628	similar to squamous cell carcinoma an	52.95872	91.37455	114.825
1368627_at	25106	Rgn	regucalcin	52.86287	35.16269	360.0851
1391533_at	362188	RGD13116	similar to MGC52019 protein (predicte	52.85923	48.38886	52.40186
1368761_at	29749	Ppp3r2	protein phosphatase 3, regulatory subu	52.81782	44.07682	141.8498
1369335_at	64555	Kpl2	KPL2 protein	52.7876	54.68386	8.466797
1380242_at	293649	Lrp5_predi	low density lipoprotein receptor-relatec	52.74845	183.1049	92.75039
1369095_at	84685	Ppp1r9a	neurabin 1	52.6843	69.15828	21.02927
1370744_at	192153	Gpr26	G protein-coupled receptor 26	52.50355	103.8494	62.98733
1387365_at	58852	Nr1h3	nuclear receptor subfamily 1, group H,	52.4973	26.89728	48.84018
1369011_at	140638	Apoa5	apolipoprotein A-V	52.45022	87.84275	134.4429
1369272_at	25370	Adora3	adenosine A3 receptor	52.39994	69.00469	50.37161
1369055_at	66025	Edg7	putative G protein-coupled receptor sn	52.36939	5.387413	17.44092
1369707_at	171296	Myo9a	myosin IXA	52.3621	58.22048	33.00083
1372374_at	310218	Car1_predi	carbonic anhydrase 1 (predicted)	52.35253	87.0908	71.04709
1368607_at	266674	Cyp4a12	cytochrome P450, 4a12	52.31687	2254.534	27.91352
1387711_at	25325	Il10	interleukin 10	52.2149	13.58132	118.4952
1368271_a_at	79451	Fabp4	fatty acid binding protein 4, adipocyte	52.18461	32.87493	41.31192
1389420_at	363334	Stap2	signal-transducing adaptor protein-2	52.17583	11.59639	43.5461
1369375_a_at	29155	Capn3	calpain 3	52.13961	12.07404	11.77967
1387799_at	29639	Fxyd2	FXYD domain-containing ion transport	51.99194	35.88589	38.15547
1398253_at	24937	Kap	kidney androgen regulated protein	51.93018	14.11844	85.27982
1374818_at	59315	Nxph3	neurexophilin 3	51.8507	245.637	438.5795
1386811_at	362540	Ptplad2_pr	protein tyrosine phosphatase-like A do	51.83508	19.87069	82.31627
1385160_at	691926	LOC69192	NA	51.81186	665.5834	50.10298
1388190_at	54225	Apob	apolipoprotein B	51.80994	41.90487	627.4289
1379154_at	359728	Rab40c	Rab40c, member RAS oncogene famil	51.74492	11.63708	14.61811
1383870_at	497986	RGD15621	similar to homeotic protein Hox 2.2 - m	51.72015	80.35633	157.6802
1385576_at	311167	Smtnl1_pre	smoothelin-like 1 (predicted)	51.70996	54.7565	55.80472
1376770_at	501181	RGD15595	similar to EF hand domain containing 1	51.69752	109.6989	214.8869
1371052_at	25495	Nog	noggin	51.69745	125.086	100.4706
1393489_at	296610	Rsb66	Rsb-66 protein	51.53257	68.19594	272.8285
1370731_at	245985	Sgp158	proline-rich glycoprotein (sgp158)	51.4814	7.146627	9.819829

1370936_at	245961	Dmgdh	dimethylglycine dehydrogenase precur	51.4375	25.47798	34.07144
1375890_at	499088	RGD15638	similar to MGC15476 protein (predicte	51.35094	402.6552	283.617
1388287_at	497985	R1b	homeobox protein	51.25759	43.35807	64.83928
1368568_at	25386	Aqp2	aquaporin 2	51.21361	27.46823	22.89184
1395908_at	362841	RGD13072	similar to KIAA1193 protein (predicted)	51.20366	119.999	55.87532
1387473_at	24839	Tnp1	transition protein 1	51.20067	6.226402	23.48643
1370479_x_at	259247	Obp3	alpha-2u globulin PGCL4	51.19617	22.16912	188.4535
1386762_at	310097	Snag1_pre	sorting nexin associated golgi protein 1	51.12673	325.9546	338.0729
1369620_at	60580	Hint1	histidine triad nucleotide binding protei	50.95186	11.37079	46.69908
1376369_at	300219	Troap_pred	trophinin associated protein (tastin) (pr	50.86685	1962.645	23.42496
1394882_at	501043	RGD15661	similar to hypothetical protein MGC349	50.84995	30.42911	43.96381
1375132_at	300017	Lypd2_pre	Ly6/Plaur domain containing 2 (predict	50.82111	156.418	147.4347
1387597_at	114498	Dmrt1	doublesex and mab-3 related transcrip	50.81233	47.12093	27.55201
1398591_at	316019	Ccrl2_pred	chemokine (C-C motif) receptor-like 2	50.78068	139.4438	588.4
1372622_at	316001	RGD15607	similar to RIKEN cDNA 6530418L21 (p	50.77248	44.1645	230.1588
1372273_at	364837	Gypc	glycophorin C (Gerbich blood group)	50.75135	109.0993	202.0688
1382033_at	304666	Klf1_predic	Kruppel-like factor 1 (erythroid) (predic	50.74971	49.58095	139.3336
1370480_at	171556	Prlpl	prolactin-like protein L	50.73105	10.70212	47.6307
1393918_at	360851	RGD15660	similar to novel protein (predicted)	50.665	776.9306	400.4629
1370161_at	64387	Ssg1	steroid sensitive gene 1	50.62942	82.78239	1239.615
1392301_at	305441	Sh3tc1_pre	SH3 domain and tetratricopeptide repe	50.58945	95.80043	39.07505
1381630_at	362175	RGD15632	similar to RIKEN cDNA A930018P22 (	50.57302	171.8656	29.80072
1387829_at	56814	Slc24a1	solute carrier family 24 (sodium/potass	50.56083	28.69591	20.72967
1369411_at	24388	Gfi1	growth factor independent 1	50.51558	58.08811	18.79512
1394483_at	304135	Adamts5	a disintegrin-like and metallopeptidase	50.43702	50.41457	341.6586
1387400_at	24409	Grin2a	glutamate receptor, ionotropic, N-meth	50.4266	165.7031	235.6361
1377307_at	361441	RGD13098	similar to mammary tumor virus recept	50.36896	672.265	419.3708
1385781_at	317252	RGD15657	similar to SNF2/RAD54 family protein (	50.36586	783.8756	68.09353
1382806_at	363806	NA	NA	50.31345	9.77618	152.7892
1385440_at	474146	Hcst	hematopoietic cell signal transducer	50.23048	29.23515	64.88423
1370083_at	57301	Ccr1	chemokine (C-C motif) receptor 1	50.21817	81.56837	33.34326
1389853_at	308443	RGD15661	similar to CDNA sequence BC028440	50.16874	163.0314	56.62881
1387675_at	25619	Plau	plasminogen activator, urokinase	50.13376	22.64808	612.1997
1386295_at	293148	RGD13073	similar to CG32425-PA (predicted)	50.09926	35.94383	14.76533
1371499_at	24936	Cd9	CD9 antigen	50.01217	10.78295	359.1821
1374191_at	303008	Rhbdf1	rhomboid family 1 (Drosophila)	49.84162	198.1681	441.879
1390835_at	360539	RGD13111	similar to 1300013J15Rik protein	49.76663	44.408	39.87157
1374862_at	290356	Phyhip	phytanoyl-CoA hydroxylase interacting	49.73282	24.22398	62.06537
1384928_at	309374	Ankrd2_pre	ankyrin repeat domain 2 (stretch respo	49.69443	53.69556	72.47356
1397292_at	500333	NA	NA	49.67609	247.2175	43.7605
1381759_at	291903	RGD15659	RGD1565975 (predicted)	49.63035	955.604	106.8334
1382081_at	364405	Scara3_pre	scavenger receptor class A, member 3	49.56217	68.22548	1354.63
1389893_at	363449	RGD15607	similar to RIKEN cDNA B630019K06 (	49.53947	25.66598	94.5544
1385678_at	171525	Polk	polymerase (DNA directed) kappa	49.53409	191.9507	59.84902
1391465_at	500572	RGD15604	similar to novel protein (predicted)	49.51581	47.48356	72.15178
1390571_at	308408	Gpr4	G protein-coupled receptor 4	49.49387	41.17569	81.34495
1389095_at	360715	Boc_predic	biregional cell adhesion molecule-relat	49.48434	14.2967	354.8235
1369914_at	25461	Hrh2	histamine receptor H 2	49.4748	47.87973	78.6283
1384864_at	84380	Dhh	desert hedgehog homolog (Drosophila	49.45282	74.31808	62.44977
1370270_at	59305	Tcam1	testicular cell adhesion molecule 1	49.45157	20.08808	35.5372
1397427_at	113902	Ces3	carboxylesterase 3	49.44903	39.9689	174.6269

1393038_at	295279	Fcgr1	Fc receptor, IgG, high affinity I	49.41089	109.1209	49.39082
1369861_at	29708	Gabra6	gamma-aminobutyric acid (GABA-A) re	49.34018	219.0455	206.2307
1385696_at	500561	RGD1561C	similar to BC013712 protein (predicted	49.27652	15.3594	147.7482
1384749_at	360467	RGD15664	similar to RIKEN cDNA 1810036I24 (p	49.22499	131.1096	31.88376
1384442_at	287098	Cldn6_pre(	claudin 6 (predicted)	49.17491	17.87781	74.22951
1391273_at	337920	Naglt1	Na+ dependent glucose transporter 1	49.16056	34.34153	392.5375
1384880_at	295483	RGD13067	hypothetical LOC295483 (predicted)	49.01904	13.14756	31.38625
1384392_at	312495	Cyp26b1	cytochrome P450, family 26, subfamily	48.99436	383.5907	269.5974
1387362_at	25722	Scn4a	sodium channel, voltage-gated, type IV	48.98198	196.1869	77.82796
1392478_at	363123	Rab6b_pre	RAB6B, member RAS oncogene family	48.96093	330.2452	35.84902
1380313_at	297645	Klre1	killer cell lectin-like receptor, family E,	48.82269	27.89368	191.9718
1393723_at	314627	RGD1305C	similar to Serine/threonine-protein kina	48.73943	2433.172	252.7409
1379586_at	361332	Adamts19_	a disintegrin-like and metallopeptidase	48.66563	52.21235	45.87212
1371271_at	296678	Olr416_pre	olfactory receptor 416 (predicted)	48.51657	9.659331	107.4363
1376373_at	406159	Btnl7	butyrophilin-like 7	48.48714	77.71107	23.87009
1386013_at	308348	NA	NA	48.4735	265.2045	40.53195
1384803_at	499663	RGD15656	RGD1565626 (predicted)	48.45384	35.31648	66.39222
1369324_at	84605	Sval2	seminal vesicle antigen-like 2	48.40736	32.80665	163.5336
1396445_at	300122	Ribc2	RIB43A domain with coiled-coils 2	48.34131	21.80941	13.18227
1387389_at	56820	Ramp3	receptor (calcitonin) activity modifying	48.32803	34.63719	29.57162
1370269_at	24296	Cyp1a1	cytochrome P450, family 1, subfamily a	48.24247	14.19767	11.4746
1375648_at	287736	RGD13079	similar to hypothetical protein (predicte	48.19336	195.1494	225.4439
1387378_at	114091	Fcnb	ficolin B	48.18019	17.09011	27.19072
1396915_at	314131	Insm2_pre(	insulinoma-associated 2 (predicted)	48.07746	154.3722	92.93433
1376110_at	315705	Rpp25	ribonuclease P 25 subunit (human)	48.0564	35.35126	35.28208
1393997_at	500595	RGD15642	similar to hypothetical protein FLJ3282	47.97404	24.87527	40.76819
1387245_at	50682	Lipf	lipase, gastric	47.8347	144.0635	53.47679
1370754_at	287003	Tas2r123	taste receptor, type 2, member 123	47.80753	20.93746	51.54922
1368776_at	25290	Alox5	arachidonate 5-lipoxygenase	47.74949	34.46091	135.1058
1369382_at	65037	Mertk	c-mer proto-oncogene tyrosine kinase	47.72098	294.5781	15.82676
1387600_at	81658	Gabrp	gamma-aminobutyric acid A receptor,	47.64568	83.03911	52.61325
1398092_at	498694	NA	NA	47.58001	32.95073	27.42188
1368718_at	29651	Aldh1a4	aldehyde dehydrogenase family 1, sub	47.53039	38.83323	24.29969
1387496_a_at	85257	Cnga3	cyclic nucleotide gated channel alpha 3	47.5171	61.8718	34.96431
1370985_at	114509	Mapk7	mitogen-activated protein kinase 7	47.48272	361.1726	230.1562
1369300_at	117547	Ncr1	natural cytotoxicity triggering receptor	47.42587	35.47363	52.74237
1384503_at	308565	Klk8	kallikrein 8 (neuropsin/ovasin)	47.36661	17.43774	14.08737
1367781_at	64673	Pip	prolactin induced protein	47.28005	11.34045	28.83518
1387497_at	25340	Npy5r	neuropeptide Y receptor Y5	47.21084	109.6418	21.57053
1369056_at	89826	Rpe65	retinal pigment epithelium 65	47.12315	20.07997	45.29647
1381660_at	291974	RGD13077	similar to mKIAA1930 protein	47.09745	45.89227	164.4606
1386953_at	25116	Hsd11b1	hydroxysteroid 11-beta dehydrogenase	47.09348	43.73329	885.6979
1377537_at	499158	RGD15599	RGD1559980 (predicted)	47.05372	23.18491	28.78837
1369848_at	66022	Kcns2	potassium voltage-gated channel, dela	46.98889	51.36501	51.75331
1376184_at	300018	Lynx1_pre(	Ly6/neurotoxin 1 (predicted)	46.94688	892.2591	378.9089
1380679_at	502347	NA	NA	46.91875	29.92411	19.32794
1384831_at	313089	Slc7a13	solute carrier family 7, (cationic amino	46.8901	23.76549	228.0115
1372691_at	289801	Upp1	uridine phosphorylase 1	46.86225	35.50989	26.99998
1387814_at	29161	Cav3	caveolin 3	46.8615	18.18377	16.02975
1384672_at	304252	RGD1561C	similar to RIKEN cDNA 4930434E21 (p	46.82592	134.8846	23.23998
1369887_at	25730	Ntf5	neurotrophin 5	46.79793	53.20263	24.50051

1387831_at	171371	Xcl1	chemokine (C motif) ligand 1	46.77447	23.25608	58.27088
1368705_at	60399	Edg8	endothelial differentiation, sphingolipid	46.68587	33.85015	34.10209
1385549_at	500256	NA	NA	46.6765	27.98297	129.3689
1374248_at	362867	Mybpc1	myosin binding protein C, slow type	46.63219	13.11162	12.86283
1370727_at	66018	Pdgfd	platelet-derived growth factor, D polype	46.58348	27.46277	27.65557
1380288_at	500984	RGD15638	RGD1563821 (predicted)	46.5635	75.62401	53.81883
1368328_at	25623	Gys2	glycogen synthase 2	46.5455	6.943716	605.4917
1393297_at	503206	NA	NA	46.4781	43.41064	79.09545
1397291_at	313790	RGD13101	similar to RIKEN cDNA 9030625A04 (p	46.44533	21.13853	16.02597
1369599_at	64568	Galp	galanin-like peptide precursor	46.40534	32.26887	104.716
1385766_at	501147	LOC50114	similar to RIKEN cDNA 1700052H20	46.3933	87.66057	64.83845
1370675_at	83810	Trpv1	transient receptor potential cation char	46.35849	2548.05	138.3902
1369760_a_at	25149	Esr2	estrogen receptor 2 beta	46.34058	6.601508	17.51129
1369594_at	116683	Efna5	ephrin A5	46.32555	127.6185	9.450839
1369853_at	60329	Neurog3	neurogenin 3	46.31534	17.67603	34.5208
1370165_at	84416	Smpx	small muscle protein, X-linked	46.27654	6.213414	22.81553
1383610_at	156826	Eya2	eyes absent 2 homolog (Drosophila)	46.23866	157.0156	170.2001
1369985_at	25421	Crybb1	crystallin, beta B1	46.23571	14.91454	41.72209
1396982_at	362603	NA	NA	46.17453	19.17756	45.75971
1389079_at	287411	Dhrs7c_pre	dehydrogenase/reductase (SDR family	46.16131	91.56107	52.63338
1396797_at	292681	Nova2_pre	neuro-oncological ventral antigen 2 (pr	46.13574	208.9803	138.9197
1369555_at	171054	Ccr4	chemokine (C-C motif) receptor 4	46.11866	117.7228	19.49811
1387745_at	64357	Mox2r	antigen identified by monoclonal antibo	46.1124	97.37586	119.2184
1369183_at	29513	Mapk13	mitogen activated protein kinase 13	46.09056	323.3284	72.90466
1380746_at	498999	Ahrr	aryl-hydrocarbon receptor repressor	46.03082	196.5174	69.49002
1390161_at	304138	LOC30413	similar to cysteine and tyrosine-rich pro	46.01164	27.55242	148.1318
1393711_at	361599	NA	NA	45.96955	55.62099	77.86845
1385331_at	500534	RGD15629	similar to hypothetical protein MGC454	45.94823	25.66682	50.49971
1376897_at	305012	RGD15600	similar to Hypothetical protein 483242(	45.73997	12.2773	22.34192
1388087_at	286956	V1rb9	vomeronasal 1 receptor, B9	45.72076	52.32784	57.6894
1376435_at	309380	Loxl4_pred	lysyl oxidase-like 4 (predicted)	45.7153	7.394984	31.62727
1372991_at	361388	Gab1_prec	growth factor receptor bound protein 2	45.53017	11.21644	25.58153
1387667_at	24599	Nos2	nitric oxide synthase 2, inducible	45.48918	35.63989	62.56959
1384526_at	290803	Tex15_pre	testis expressed gene 15 (predicted)	45.40594	296.6026	224.5963
1371083_at	299282	Spin2a	Serine protease inhibitor	45.35326	30.20622	29.92308
1368930_at	65206	Kcnn4	potassium intermediate/small conducta	45.27896	29.79754	25.04668
1369947_at	29175	Ctsk	cathepsin K	45.26697	20.78371	368.5966
1374630_at	296566	Clic3	chloride intracellular channel 3	45.23088	121.6948	53.34909
1369600_at	170632	Fgf11	fibroblast growth factor 11	45.1932	186.5815	24.11061
1368755_at	114598	Clec4f	C-type lectin domain family 4, member	45.18678	47.10103	21.78592
1393573_at	289878	Pde6b_pre	phosphodiesterase 6B, cGMP, rod rec	45.13351	15.88719	33.45799
1393638_at	84023	Ptger4	prostaglandin E receptor 4 (subtype EI	45.08297	67.15007	502.6018
1388329_at	287699	Ka13	type I keratin KA13	45.05991	74.09903	9.928815
1387134_at	114247	Slfn3	schlafen 3	45.05289	183.9273	357.7603
1371788_at	501926	RGD15597	similar to RIKEN cDNA 2610510L01 (p	45.05033	654.0507	425.8711
1374816_at	363091	LOC36309	similar to hypothetical protein FLJ3097	45.03025	13.03164	20.54795
1371162_at	252960	Mrga10	nuclear receptor MrgA10 RF-amide G	45.0158	34.12354	44.84487
1370942_at	29372	Rasa3	RAS p21 protein activator 3	44.97484	1021.746	426.3493
1387483_at	29337	Plcg2	phospholipase C, gamma 2	44.95146	40.84855	265.3993
1370152_at	25259	Gp5	glycoprotein 5, platelet	44.93799	44.91609	132.2986
1385298_at	366909	RGD15648	similar to putative anion transporter (pr	44.92085	18.58034	34.7197

1370999_at	252918	Spag5	sperm associated antigen 5	44.89384	5343.24	201.6095
1373720_at	361135	March1_pr	membrane-associated ring finger (C3H	44.87312	57.28826	67.84286
1378085_at	360605	Epn3	epsin 3	44.80343	159.0357	69.70123
1369054_at	171039	Rph3a	rabphilin 3A homolog (mouse)	44.76555	31.80169	134.6198
1383315_at	308843	Eiih	hepatic protein EIIH	44.75842	130.0771	63.55243
1383732_at	293656	RGD13076	similar to hypothetical protein MGC379	44.7454	18.01201	45.47908
1375267_at	291463	Ppic	peptidylprolyl isomerase C	44.7113	23.78428	252.9672
1387854_at	84352	Col1a2	procollagen, type I, alpha 2	44.67295	20.73213	2287.961
1381398_at	296516	RGD15604	similar to Cystatin S precursor (LM pro	44.63883	56.1612	7.814994
1374928_at	360954	NA	NA	44.62964	34.75202	103.5885
1397861_at	361560	RGD13062	similar to contains transmembrane (TM	44.58921	892.3826	161.4009
1368278_at	171134	Lgals2	lectin, galactoside-binding, soluble 2	44.58741	10127.26	681.6311
1393680_at	84592	Cetn1	centrin 1	44.5558	145.0854	74.57395
1384787_at	300678	Cd3g	CD3 antigen, gamma polypeptide	44.54456	22.64962	229.6387
1393650_at	362972	LOC36297	NA	44.48726	98.18962	223.8561
1378939_at	363682	Gpr2_pred	G protein-coupled receptor 2 (predicte	44.47605	46.90194	17.38141
1387014_at	65182	Muc10	mucin 10, submandibular gland salivar	44.40038	32.73974	47.24253
1397171_at	304521	RGD15601	similar to hypothetical protein (predicte	44.36496	83.40203	122.5407
1387217_a_at	29446	Ghrh	growth hormone releasing hormone	44.33845	175.3988	157.9822
1380927_at	687814	LOC68781	NA	44.33386	16.45684	46.20821
1387828_at	65218	Centg1	centaurin, gamma 1	44.2723	107.6124	198.3362
1390541_at	308596	Ush1c	Usher syndrome 1C homolog (human)	44.26878	36.6499	229.6181
1368407_at	64537	Hpse	heparanase	44.24578	1201.167	174.2336
1369800_at	116498	F2rl3	coagulation factor II (thrombin) recepto	44.24117	27.02288	21.86033
1372321_at	304663	Lyl1	lymphoblastic leukemia derived seque	44.14994	17.47012	113.0164
1381039_at	171339	Dnah1	dynein, axonemal, heavy polypeptide 1	44.14595	70.54859	15.61801
1369838_at	64345	Hif3a	hypoxia inducible factor 3, alpha subur	44.0883	29.73491	19.36804
1378295_at	312052	Steap2_pre	six transmembrane epithelial antigen c	44.08262	38.18059	58.40059
1371080_at	292861	LOC29286	kallikrein	44.04453	30.68776	101.3821
1371408_at	25583	Cryba1	crystallin, beta A1	44.03485	73.1718	19.61578
1387240_at	360420	Rdh7	retinol dehydrogenase 7	43.99772	12.8213	7.705082
1387327_at	170843	Khdrbs2	KH domain containing, RNA binding, s	43.99105	152.1289	104.9544
1379081_at	302976	Noxo1_pre	NADPH oxidase organizer 1 (predicted	43.95371	69.48699	42.13053
1388183_at	24284	CSN1S1	casein alpha s1	43.94911	63.23705	25.87406
1388846_at	361567	Bcl2l12_pr	BCL2-like 12 (proline rich) (predicted)	43.93481	21.68378	32.46892
1380456_at	307923	RGD15651	similar to chromosome 16 open readin	43.93306	131.8196	183.9195
1380149_at	314716	Gas2l3_pre	growth arrest-specific 2 like 3 (predicte	43.83397	17.74052	11.09727
1369353_at	59323	Erbb4	v-erb-a erythroblastic leukemia viral or	43.80521	20.04712	16.37952
1393893_at	362122	RGD13077	similar to CG3306-PA	43.79307	50.70421	99.24339
1371000_at	116652	Cacna1s	calcium channel, voltage-dependent, L	43.77838	19.33997	64.94682
1386914_at	117533	Gmpr	guanosine monophosphate reductase	43.77419	571.4357	578.9759
1380104_at	24946	F9	coagulation factor IX	43.72855	13.12334	12.9369
1377699_at	304127	Bach1_pre	BTB and CNC homology 1 (predicted)	43.64157	20.83109	18.27483
1385599_at	297173	RSA-14-44	RSA-14-44 protein	43.61697	25.04653	176.9178
1369509_a_at	140656	A1bg	alpha-1-B glycoprotein	43.56692	9379.482	49.16225
1368611_at	171101	Grp	gastrin releasing peptide	43.56293	32.32832	164.9547
1390554_at	311137	Zfp533_pre	zinc finger protein 533 (predicted)	43.54206	17.88002	885.0824
1374384_at	24277	Crygc	crystallin, gamma C	43.49754	77.13906	17.11142
1368380_at	29169	Vtn	vitronectin	43.3421	11.7445	91.33557
1394609_at	360958	Ablim2	actin-binding LIM protein 2	43.31101	193.1054	141.7628
1390715_at	366366	Igfbpl1_pre	insulin-like growth factor binding protei	43.2661	60.20114	31.90706

1395812_at	500493	NA	NA	43.22519	92.90469	43.08354
1371061_at	29588	Pou3f2	POU domain, class 3, transcription fac	43.21652	31.30775	75.07497
1369136_at	24299	Cyp2a3a	cytochrome P450, family 2, subfamily .	43.11713	30.68116	126.4629
1369533_a_at	25324	Htr4	5-hydroxytryptamine (serotonin) recep	43.02093	217.0677	62.58459
1377635_at	246245	Fmo2	flavin containing monooxygenase 2	42.94131	128.6193	196.61
1396272_at	361292	NA	NA	42.92431	245.03	23.42594
1368335_at	25081	Apoa1	apolipoprotein A-I	42.82099	29.96278	277.8038
1385299_at	302378	Gpr23_pre	G protein-coupled receptor 23 (predict	42.79967	9.879181	63.19402
1385225_at	680918	LOC68091	NA	42.68872	22.72369	42.39586
1384102_at	298884	RGD15597	similar to RIKEN cDNA 5830483C08 g	42.6351	77.66994	13.065
1376569_at	306330	Klf2_predic	Kruppel-like factor 2 (lung) (predicted)	42.55334	3097.106	4364.134
1369257_at	81760	Grk1	G protein-coupled receptor kinase 1	42.55266	55.17659	69.07506
1369524_at	64618	Zic1	zinc finger protein of the cerebellum 1	42.49413	82.10965	48.11806
1375831_at	365344	LOC36534	NA	42.46819	76.92087	96.9076
1370118_at	117518	Ccl17	chemokine (C-C motif) ligand 17	42.42746	43.12127	233.8659
1378419_at	474169	LOC47416	pre-eosinophil-associated ribonucleas	42.41044	3.960075	48.42359
1387137_at	25304	Comp	cartilage oligomeric matrix protein	42.39977	96.28046	160.33
1392326_at	287845	Dnahc11	beta heavy chain of outer-arm axonem	42.35618	68.60661	142.2636
1369506_at	29394	Gcm1	glial cells missing homolog 1 (Drosoph	42.18617	36.90276	9.984847
1392233_at	289395	Cr2_predic	complement receptor 2 (predicted)	42.17144	28.20295	156.0294
1368125_at	29501	Slc12a4	solute carrier family 12, member 4	42.16246	275.2152	105.343
1390764_at	292620	NA	NA	42.14616	46.70999	15.58418
1368017_at	29518	Lgals7	lectin, galactose binding, soluble 7	42.09923	16.43591	18.1452
1368131_at	83685	Capn6	calpain 6	42.07524	67.45812	38.31452
1392802_at	288469	RGD13097	similar to RIKEN cDNA 4930431E10 (|	42.03208	110.4032	23.75001
1371153_a_at	24397	Glra2	glycine receptor, alpha 2 subunit	41.87469	48.99227	25.30102
1378209_a_at	293854	Stk23	serine/threonine kinase 23	41.77911	43.48584	614.3815
1387138_at	29191	Tac2	tachykinin 2	41.74823	98.24105	165.7772
1398387_at	310540	MGC72614	Unknown (protein for MGC:72614)	41.74532	53.91495	761.3376
1389937_at	307206	RGD15662	similar to Neuropilin- and tolloid-like pr	41.69873	44.59888	18.66461
1376976_at	287884	Sectm1	secreted and transmembrane 1	41.66846	94.52608	29.74716
1384240_at	24180	Agtr1a	angiotensin II receptor, type 1 (AT1A)	41.60235	10.98308	525.5363
1386274_at	306950	Slc17a2_pi	solute carrier family 17 (sodium phosp	41.54069	40.64164	28.18709
1382559_at	362741	LOC36274	similar to transcription factor	41.53332	14.48733	19.87424
1398524_at	305185	RGD15622	similar to GPI-gamma 4; GPIgamma4	41.47918	40.3554	214.1642
1375465_at	305858	Otx2	orthodenticle homolog 2 (Drosophila)	41.33025	11.34958	33.40154
1368777_at	64557	Bard1	BRCA1 associated RING domain 1	41.25995	485.8729	43.80599
1374908_at	498891	RGD15596	similar to hypothetical protein B230399	41.24938	30.7789	145.2239
1370560_at	286996	Hit39	zinc finger protein HIT-39	41.20986	94.12798	175.2146
1368270_at	25383	Apobec1	apolipoprotein B editing complex 1	41.20183	10.21435	30.73416
1376538_at	498020	NA	NA	41.12552	53.35857	28.10033
1385400_at	312488	Atp6v1b1_	ATPase, H transporting, lysosomal V1	41.07775	21.5341	69.80751
1391326_at	497987	RGD15622	similar to homeotic protein Hox B5 - m	41.02945	150.3228	85.50822
1379168_at	311786	Traf2_pred	Tnf receptor-associated factor 2 (predi	40.99649	14.01751	17.44632
1396354_at	502145	LOC50214	NA	40.97399	113.1189	141.9083
1383952_at	294520	Mical1_pre	microtubule associated monoxygenase	40.9589	46.56193	18.36451
1369845_at	81721	Chrna6	cholinergic receptor, nicotinic, alpha po	40.94627	48.15599	36.57466
1392821_at	315074	RGD13081	similar to secreted Ly6/uPAR related p	40.91999	67.78098	24.73679
1382026_at	362893	Arhgap9	Rho GTPase activating protein 9	40.89683	18.61281	76.14525
1389607_at	305811	Rcor2	REST corepressor 2	40.81012	440.7331	28.25948
1384762_at	363174	LOC36317	NA	40.76431	2718.353	121.1331

1387816_at	79438	Igfals	insulin-like growth factor binding protei	40.747	16.67537	36.83087
1369018_at	58921	Foxm1	forkhead box M1	40.73117	1188.553	223.1563
1377324_at	293925	RGD15645	RGD1564599 (predicted)	40.64008	13.26311	20.62044
1391629_at	362677	NA	NA	40.5585	38.2011	114.8381
1369586_at	29269	Mcpt8	mast cell protease 8	40.51763	35.6993	38.15182
1379224_at	363502	Mid2_predi	midline 2 (predicted)	40.46993	14.24353	48.47988
1369812_at	63843	P2ry4	pyrimidinergic receptor P2Y, G-protein	40.40168	57.89796	19.69777
1376271_at	294284	AA926063	AA926063gene	40.33801	6.741028	19.42705
1381963_at	315728	RGD15662	similar to RIKEN cDNA B230114P05 (	40.3353	76.32441	23.4344
1368414_at	64522	Slc5a2	solute carrier family 5 (sodium/glucose	40.32808	81.45026	53.7318
1387309_a_at	29559	Grik1	glutamate receptor, ionotropic, kainate	40.21388	26.50456	221.1571
1396551_at	314798	RGD13102	similar to CG33154-PB (predicted)	40.20892	5.268885	120.2008
1387968_at	282712	Slc6a15	solute carrier family 6 (neurotransmitte	40.14619	1096.892	24.6456
1379293_at	266708	Gzma	granzyme A	40.12614	20.01204	31.4473
1370333_a_at	24482	Igf1	insulin-like growth factor 1	39.95837	2109.584	581.2842
1384558_at	361105	Plac9_prec	placenta-specific 9 (predicted)	39.95348	32.1261	49.60783
1397671_at	305142	RGD15647	similar to ATP-binding cassette, sub-fa	39.93982	6.638973	84.82207
1378554_at	501049	RGD15601	similar to Probable G-protein coupled r	39.81838	28.95691	21.69779
1371146_at	26296	Tfec	transcription factor EC	39.79214	17.99534	152.7168
1387902_a_at	500180	LOC50018	similar to IG KAPPA CHAIN V-V REGI	39.75865	13.19617	19.53141
1380982_at	246755	Bcas1	breast carcinoma amplified sequence	39.68937	96.28379	316.1731
1368769_at	83569	Abcb11	ATP-binding cassette, sub-family B (M	39.65425	63.5039	119.3028
1389766_at	292874	RGD1309C	hypothetical LOC292874 (predicted)	39.65374	287.2532	53.31929
1370839_at	286920	Accn3	amiloride-sensitive cation channel 3	39.64818	8.645442	15.152
1387293_at	81828	Zp2	zona pellucida glycoprotein 2	39.64719	15.2096	144.1652
1384278_x_at	362187	RGD15605	similar to 2810027O19Rik protein (pre	39.56708	769.2071	34.4736
1387100_at	65133	Aqp3	aquaporin 3	39.51869	37.15975	182.0047
1394824_at	315744	Itga11_pre	integrin, alpha 11 (predicted)	39.46876	107.453	168.261
1373262_at	361790	RGD13095	similar to 2310014H01Rik protein (pre	39.44105	136.4917	42.27896
1387305_s_at	24294	Cyp11b2	cytochrome P450, family 11, subfamily	39.41625	11.0222	20.67821
1370653_at	286985	LOC28698	putative pheromone receptor (Go-VN7	39.33978	38.58629	54.5994
1380133_at	315039	Osr2	odd-skipped related 2 (Drosophila)	39.31234	44.44019	159.4676
1392819_at	361735	Ms4a11_pr	membrane-spanning 4-domains, subfa	39.30813	67.6419	69.56819
1386920_x_at	59088	Prlph	prolactin-like protein H	39.30361	26.67647	4.355882
1369273_a_at	25339	Npr3	natriuretic peptide receptor 3	39.25375	19.84042	35.84159
1393229_at	362818	Silv_predic	silver homolog (mouse) (predicted)	39.2073	122.057	38.60929
1387532_at	170633	Fgf3	fibroblast growth factor 3	39.15678	21.69558	157.092
1369882_at	29190	Pdyn	prodynorphin	39.14591	121.6009	133.866
1368938_at	84010	Dll1	delta-like 1 (Drosophila)	39.13511	58.70143	303.3291
1381023_at	296469	RGD13058	similar to chromosome 20 open readin	39.07622	31.6931	92.01744
1368764_at	24829	Hist1h2ba	histone 1, H2ba	39.03985	23.78146	35.88686
1369684_at	171046	Tcfe2a	transcription factor E2a	38.98159	42.29724	30.60572
1398492_at	500401	NA	NA	38.95765	33.75804	20.43263
1387877_at	89833	Ftcd	formiminotransferase cyclodeaminase	38.8071	29.99203	248.2316
1382581_at	295965	Ehf_predic	ets homologous factor (predicted)	38.65131	24.78267	57.71631
1389490_at	293669	Cd248_pre	CD248 antigen, endosialin (predicted)	38.64485	34.05193	275.3259
1391186_at	498250	NA	NA	38.63666	963.4806	847.6586
1393532_at	308905	Ubqln3_pre	ubiquilin 3 (predicted)	38.61709	17.80954	14.43072
1387451_at	155696	Dcbld2	discoidin, CUB and LCCL domain cont	38.53219	326.8274	82.47487
1387722_at	29295	Cyp2b15	cytochrome P450, family 2, subfamily I	38.51835	11.30486	93.56231
1379203_at	300947	Tcfdp2_pre	transcription factor Dp 2 (predicted)	38.4998	254.007	143.3726

1370371_a_at	287009	Ceacam10	CEA-related cell adhesion molecule 10	38.37217	795.5578	47.92694
1387835_at	60582	Il1rn	interleukin 1 receptor antagonist	38.36228	16.15296	19.12674
1380930_at	290569	NA	NA	38.32923	33.19131	93.95073
1374904_at	114634	Six1	sine oculis homeobox homolog 1 (Dros	38.28931	33.12581	75.75127
1396339_at	500266	RGD15630	similar to TAFA1 protein (predicted)	38.21254	24.33633	69.04159
1390783_at	303638	Abca8a_pr	ATP-binding cassette, sub-family A (AI	38.19367	28.29914	2186.537
1368052_at	171048	Tspan8	tetraspanin 8	38.17236	10.1268	400.4732
1388056_at	246268	Oas1b	2-5 oligoadenylate synthetase 1B	38.16473	36.85399	245.7116
1367739_at	25250	Cox8h	Cytochrom c oxidase subunit VIII-H (h	38.08763	65.28174	11.19249
1368093_at	29556	Myh6	myosin, heavy polypeptide 6, cardiac r	37.98966	31.67755	60.91266
1388012_at	252880	Prrxl1	paired related homeobox protein-like 1	37.98506	10.73847	7.810308
1390782_at	314148	Clec14a	C-type lectin domain family 14, membe	37.81992	28.1865	67.41396
1383686_at	29205	Syngr1	synaptogyrin 1	37.81867	239.2362	23.97229
1381578_at	361002	Dusp13	dual specificity phosphatase 13	37.71244	18.7442	44.38726
1394112_at	311446	Hao1_map	hydroxyacid oxidase 1 (mapped)	37.58905	107.486	170.1658
1376172_at	300664	Abcg4	ATP-binding cassette, sub-family G (W	37.50693	575.9276	139.7776
1388031_x_at	259245	LOC25924	alpha-2u globulin PGCL5	37.20514	12.08444	85.0867
1369563_at	64571	Vax1	ventral anterior homeobox 1	37.14368	13.69896	23.3008
1370757_at	140724	Cacng3	calcium channel, voltage-dependent, g	37.08681	93.38515	19.73613
1382906_at	317590	MGC11452	similar to melanoma antigen family A,	37.08235	77.03451	21.77967
1393403_at	502970	Angptl3	angiopoietin-like 3	37.06124	33.20846	28.71357
1398054_at	288744	RGD13065	similar to hypothetical protein A530094	37.01624	83.4616	57.85793
1387683_at	58959	Crhr1	corticotropin releasing hormone recept	37.01173	2686.104	62.41755
1387830_at	25419	Crp	C-reactive protein, pentraxin-related	37.01077	227.6559	633.974
1380260_at	364680	Barx1_prec	BarH-like homeobox 1 (predicted)	36.97214	38.57222	19.60116
1370467_at	58980	Slc13a1	solute carrier family 13 (sodium/sulfate	36.97203	88.77395	41.66002
1390955_at	315567	Panx3	pannexin 3	36.90458	45.21422	24.14475
1369727_at	25649	Apoa2	apolipoprotein A-II	36.82455	26.37529	43.80316
1393924_s_at	498117	NA	NA	36.72605	68.12101	51.68152
1385421_at	499836	RGD15632	similar to RIKEN cDNA 1700029I15 (p	36.70849	37.94864	10.9787
1395148_at	313278	RGD15610	similar to protein tyrosine phosphatase	36.70182	52.3408	280.4815
1394851_at	309389	Nkx2-3_pre	NK2 transcription factor related, locus	36.69334	15.91244	27.70176
1368415_at	24583	Myh3	myosin, heavy polypeptide 3, skeletal r	36.66153	32.55779	14.14313
1370632_at	286933	Obp2b	odorant binding protein 2B	36.64372	26.1928	15.02341
1389727_at	314848	Lrrc10_pre	leucine-rich repeat-containing 10 (prec	36.57399	28.3236	23.81743
1388046_at	25021	Itgam	integrin alpha M	36.54992	16.98735	20.36713
1384385_at	361914	LOC36191	similar to solute carrier family 7 (cation	36.50061	14.00312	37.60254
1369366_at	114589	Cntn5	contactin 5	36.47627	39.99468	36.91418
1386604_at	114202	Mpp3	membrane protein, palmitoylated 3 (MA	36.47267	224.0239	237.9357
1368913_at	29173	Csn2	casein beta	36.31297	12.51313	132.1151
1374378_at	289565	Arl9	ADP-ribosylation factor-like 9	36.23533	54.51989	85.84771
1369392_at	79254	Akap4	A-kinase anchor protein 4	36.18996	14.35699	27.68422
1371276_at	613222	Defa7	defensin alpha 7	36.11874	9.882059	99.15209
1370768_at	286992	LOC28699	peptide HP (rs14)	36.08946	32.79871	9.570487
1387955_at	266685	Ugt2b3	UDP glycosyltransferase 2 family, mer	36.03578	9.411788	180.1441
1375578_at	499701	NA	NA	36.02875	36.76216	29.45397
1378640_at	316129	Uhrf1_map	ubiquitin-like, containing PHD and RIN	36.00585	1387.128	117.7287
1393379_a_at	364403	Blk	B lymphoid kinase	35.97791	77.45212	35.75116
1385785_at	313451	LOC31345	reproductive homeobox on X chromos	35.971	23.7576	36.7488
1393953_at	295361	Eps8l3_pre	EPS8-like 3 (predicted)	35.87952	35.83562	148.035
1384522_at	499017	RGD15619	similar to synaptotagmin-like protein 3-	35.81096	24.92944	47.66193

1396572_at	499255	RGD1565C	similar to TSG118.1 (predicted)	35.78753	72.22476	25.75103
1393708_at	365748	Bhlhb5_pre	basic helix-loop-helix domain containir	35.67003	38.8832	232.102
1385969_at	306664	Adamts16_	a disintegrin-like and metallopeptidase	35.65522	50.94637	79.67891
1384592_at	502986	NA	NA	35.62754	31.06382	16.25385
1388095_at	286987	LOC28698	hemiferrin, transferrin-like protein	35.58062	51.60051	8.35921
1368362_a_at	29403	Asgr2	asialoglycoprotein receptor 2	35.52402	17.18261	170.4183
1392646_at	299714	Tmem16d_	transmembrane protein 16D (predicted	35.51546	252.6751	114.5603
1384440_at	498993	NA	NA	35.44364	102.6121	157.5493
1390322_at	501014	NA	NA	35.37545	96.37277	20.19258
1397627_at	307483	Diap1_prec	diaphanous homolog 1 (Drosophila) (p	35.37152	11.04839	21.71819
1390420_at	296156	Cpxm1_pre	carboxypeptidase X 1 (M14 family) (pr	35.35976	74.91208	222.2922
1367880_at	25473	Lamb2	laminin, beta 2	35.29882	18.92868	246.1838
1367749_at	81682	Lum	lumican	35.27551	10.42041	5858.701
1370550_at	29561	Lsamp	limbic system-associated membrane p	35.24882	65.42275	24.88042
1382358_at	25560	Abcc9	ATP-binding cassette, sub-family C (C	35.21317	11.09925	334.8203
1371270_at	295713	Olr442_pre	olfactory receptor 442 (predicted)	35.1845	89.40648	91.67841
1384721_at	499659	NA	NA	35.16527	14.48953	73.56356
1373856_at	314467	RGD13056	hypothetical LOC314467 (predicted)	35.12064	50.59522	23.73471
1391352_at	297698	RGD15653	similar to RIKEN cDNA 1810033M07 (	35.03124	13.40475	78.7866
1384677_at	301556	RGD15619	similar to Dedicator of cytokinesis prot	34.83644	57.24646	27.61025
1378528_at	294789	RGD13075	similar to Hypothetical protein FLJ2542	34.829	54.49801	39.27205
1369140_at	140936	Rcvrn	recoverin	34.81736	197.1591	111.8335
1378826_at	498535	NA	NA	34.80231	81.12084	84.28754
1368428_at	117522	Xpnpep2	X-prolyl aminopeptidase (aminopeptida	34.79121	47.2913	62.84594
1387164_at	81512	Lect1	leukocyte cell derived chemotaxin 1	34.75947	27.69141	33.27528
1396365_at	501056	RGD15645	RGD1564575 (predicted)	34.74895	18.77757	66.00567
1370659_at	286981	LOC28698	putative pheromone receptor (Go-VN2	34.70275	9.575536	49.14335
1379670_at	316425	Sgol2_prec	shugoshin-like 2 (S. pombe) (predicted	34.60207	24.85678	46.56373
1373698_at	295176	S100vp	S100 calcium-binding protein, ventral p	34.59094	16.53316	17.51792
1377112_at	362638	Cda_predic	cytidine deaminase (predicted)	34.51581	24.07029	52.98202
1391301_at	306969	Hist1h2bp_	histone 1, H2bp (predicted)	34.47785	3.088463	142.0462
1368347_at	60379	Col5a3	procollagen, type V, alpha 3	34.43317	29.56967	42.43158
1398139_at	315072	RGD13119	hypothetical LOC315072 (predicted)	34.34056	98.98672	19.1103
1394651_at	310689	RGD13094	similar to hypothetical protein FLJ3288	34.04783	13.44968	122.0426
1386160_at	310588	Thh_predic	trichohyalin (predicted)	33.92046	22.63405	137.7306
1370310_at	24450	Hmgcs2	3-hydroxy-3-methylglutaryl-Coenzyme	33.884	32.24115	93.31945
1398418_at	303382	Rasl10b_p	RAS-like, family 10, member B (predic	33.81475	26.23798	41.55061
1370670_at	115768	Zfp37	zinc finger protein 37	33.76815	68.25283	99.02461
1397037_at	361651	RGD15641	similar to hypothetical protein (predicte	33.66927	11.78436	16.1304
1375900_at	500590	LOC50059	NA	33.5352	65.89523	44.24012
1392771_at	312246	RGD13062	similar to KIAA1549 protein (predicted)	33.52017	22.5803	9.793006
1379563_at	296953	Irf5_predict	interferon regulatory factor 5 (predicted	33.51028	34.72708	94.5392
1385179_at	291077	RGD13075	similar to RIKEN cDNA 4933417A18	33.44428	12.9034	94.70401
1378102_at	307949	RGD13058	similar to cDNA sequence AF096286;	33.39724	86.57979	34.62006
1372598_at	361424	RGD13096	similar to 1190005I06Rik protein (pred	33.28634	128.5661	45.88523
1381082_at	502306	RGD15643	RGD1564357 (predicted)	33.24189	32.05621	18.53029
1392553_at	314626	Adamtsl5_	ADAMTS-like 5 (predicted)	33.23865	79.39715	46.66415
1380967_at	299757	Nts_predic	neurotensin (predicted)	33.19977	16.54983	61.00849
1373686_at	299270	Serpina6_r	serine (or cysteine) proteinase inhibito	33.19368	74.21934	87.96427
1368496_at	24610	Odf1	outer dense fiber of sperm tails 1	33.14381	166.4894	21.08845
1369406_at	114104	Asah2	N-acylsphingosine amidohydrolase 2	33.07764	37.5996	60.78845

1384535_at	498868	NA	NA	32.91284	34.30106	71.88131
1385461_at	500675	NA	NA	32.84736	10.01411	37.84523
1388902_at	315714	Loxl1	lysyl oxidase-like 1	32.70705	22.27267	634.6248
1369918_at	246043	Klrh1	killer cell lectin-like receptor subfamily	32.7016	62.62866	70.66012
1373489_at	363333	LOC36333	NA	32.63316	2337.275	76.49816
1387047_at	78951	Hspb3	heat shock 27kDa protein 3	32.60181	53.69179	129.4993
1369294_at	81506	Bst1	bone marrow stromal cell antigen 1	32.58877	57.60402	18.47724
1380267_at	287392	Tekt3	tektin 3	32.58765	21.83054	16.62821
1397827_at	288909	Nanos3_pr	nanos homolog 3 (Drosophila) (predict	32.57573	77.85222	33.31278
1391597_at	498434	RGD15663	similar to G protein-coupled receptor G	32.57449	43.17716	174.4105
1387787_at	24584	Myl2	myosin, light polypeptide 2	32.53958	183.6375	44.1313
1374745_at	311629	NA	NA	32.53547	501.8403	229.1248
1369889_at	24481	Ifnb1	interferon beta 1, fibroblast	32.50866	10.47201	18.07747
1368553_at	25237	Acvrl1	activin A receptor type II-like 1	32.45613	34.22847	12.24754
1369444_at	25147	Cyp19a1	cytochrome P450, family 19, subfamily	32.41895	25.10838	11.70825
1385097_at	266729	Dab1	disabled homolog 1 (Drosophila)	32.4028	203.6029	49.59008
1393629_at	364069	Hlx1_predi	H2.0-like homeo box 1 (Drosophila) (p	32.34201	63.44151	36.84738
1387215_at	24792	Agxt	alanine-glyoxylate aminotransferase	32.34028	45.31736	37.72171
1391150_at	500047	Wnt16	wingless-related MMTV integration site	32.30386	85.5714	99.78644
1383418_at	360638	Adam11_p	a disintegrin and metallopeptidase dom	32.2976	63.06484	133.2734
1391652_at	293779	Glyat	glycine-N-acyltransferase	32.24787	49.96804	10.32923
1372240_at	303468	Sgca_pred	sarcoglycan, alpha (dystrophin-associa	32.23429	27.215	54.95927
1372903_at	303575	RGD13103	similar to 3000004C01Rik protein	32.18094	3894.988	83.78531
1367917_at	25053	Cyp2d26	cytochrome P450, family 2, subfamily d	32.11502	52.19205	66.31071
1388147_at	24573	Muc3	mucin 3	32.01811	29.04704	19.80587
1384706_at	305835	RGD15658	similar to Pellino protein homolog 2 (Pe	31.98563	7.79256	33.73756
1368047_at	64846	Slc13a3	solute carrier family 13 (sodium-depen	31.98163	46.8462	21.9703
1369607_at	170700	Fgf6	fibroblast growth factor 6	31.85126	29.29397	61.57702
1380329_at	361757	Tmem10	transmembrane protein 10	31.82602	50.4979	9.104791
1391595_at	315731	Larp6_prec	La ribonucleoprotein domain family, me	31.78932	132.4227	294.4384
1389160_at	293522	Eraf_predic	erythroid associated factor (predicted)	31.76109	16.92845	23.04902
1394757_at	314787	RGD13116	similar to Hypothetical protein KIAA037	31.68689	13.73333	105.8456
1377037_at	50559	Cte1	cytosolic acyl-CoA thioesterase 1	31.68394	209.6549	41.47368
1388047_at	286982	LOC28698	putative pheromone receptor (Go-VN6	31.65278	41.52056	109.0638
1368864_at	66030	Synpr	synaptoporin	31.56477	165.92	67.25899
1385483_at	289236	RGD15624	similar to hypothetical protein A030011	31.50122	33.75265	149.7808
1380417_at	498902	RGD15650	similar to chemokine-like factor super f	31.48507	57.4294	49.40802
1386387_at	140586	Sox9	SRY-box containing gene 9	31.48437	7.740307	12.57984
1381554_at	313653	RGD13085	similar to hypothetical protein FLJ3278	31.25846	33.61128	223.4065
1387583_at	154985	Cyp26a1	cytochrome P450, family 26, subfamily	31.23689	168.6225	191.9214
1383571_at	303515	LOC30351	hypothetical protein LOC303515	31.17472	293.4915	585.9718
1381536_at	292690	RGD15620	similar to expressed sequence C79127	30.97915	17.38417	29.92328
1387544_at	171436	Mk1	Mk1 protein	30.94887	39.32956	50.74792
1391188_at	313311	RGD15607	RGD1560720 (predicted)	30.83402	65.40788	53.05853
1370332_at	192177	Unc13d	unc-13 homolog D (C. elegans)	30.79765	37.25882	36.24236
1391268_at	259167	Art5	ADP-ribosyltransferase 5	30.74146	15.92354	75.6728
1368297_at	25159	Gata2	GATA binding protein 2	30.72903	108.9628	114.9272
1369823_at	29641	Adam7	a disintegrin and metalloprotease dom	30.72336	76.06165	40.40476
1381922_at	252854	Slc5a11	solute carrier family 5 (sodium/glucose	30.63373	153.7149	18.22693
1387552_at	116681	Dlgap2	discs, large (Drosophila) homolog-asso	30.60086	87.72028	45.75037
1387422_at	84387	Pglyrp1	peptidoglycan recognition protein 1	30.56837	55.59722	66.92275

1369536_at	24324	Edn2	endothelin 2	30.55618	27.57616	59.51907
1369767_a_at	29747	Kcnmb1	potassium large conductance calcium-	30.41276	59.07988	19.42208
1370771_at	140728	Cacng7	calcium channel, voltage-dependent, g	30.40138	151.514	147.2187
1389234_at	116669	Vwf	von Willebrand factor	30.40137	18.31433	54.99048
1383272_at	302666	Asb11_pre	ankyrin repeat and SOCS box-containi	30.38102	13.75384	81.6696
1379326_at	361228	RGD13056	similar to 9530008L14Rik protein	30.23253	41.70572	47.37055
1385827_at	365395	Clcf1	cardiotrophin-like cytokine factor 1	30.18764	70.36989	81.64709
1369797_at	29412	Adra1a	adrenergic receptor, alpha 1a	30.14013	45.30531	33.69448
1369561_at	114099	Cysltr1	cysteinyl leukotriene receptor 1	30.09211	58.21044	17.54251
1369327_at	65034	Pdzk3	PDZ domain containing 3	30.07735	144.9917	11.81877
1387125_at	94195	S100a9	S100 calcium binding protein A9 (calgr	30.02606	16.79468	31.92054
1388103_at	245953	Tmem37	transmembrane protein 37	29.83106	18.23026	214.7329
1393775_at	303112	RGD13083	similar to Hypothetical protein KIAA03	29.82971	67.14878	47.80883
1394755_at	301334	LOC30133	NA	29.82788	23.01432	37.64961
1377882_at	140635	Flt3	FMS-like tyrosine kinase 3	29.81505	14.28074	36.54509
1370756_at	287002	Olr1082	olfactory receptor 1082	29.67281	119.2097	16.05094
1368521_at	60575	Napsa	napsin A aspartic peptidase	29.63648	12.89253	16.08282
1387974_a_at	80899	Slc21a4	kidney specific organic anion transport	29.62736	28.71647	44.0705
1368329_at	29509	Slc22a6	solute carrier family 22 (organic anion	29.60658	37.71447	15.06368
1396343_at	499463	RGD15617	similar to Na+ dependent glucose tran:	29.54261	34.75461	87.38882
1387749_at	171055	Cd79b	CD79B antigen	29.50401	18.24603	25.81656
1383708_at	498564	LOC49856	similar to integrin, beta-like 1	29.49916	96.38163	508.7594
1370128_at	59112	Hand1	heart and neural crest derivatives expr	29.49645	123.4016	203.2675
1398138_at	266765	Ush2a	Usher syndrome 2A (autosomal recess	29.45488	10.8866	51.32696
1394507_at	362719	Ddef2_pre	development and differentiation enhan	29.4299	11.21778	135.5092
1396347_at	361629	RGD15659	RGD1565926 (predicted)	29.37619	124.385	38.82501
1369423_at	29130	Syn3	synapsin III	29.30191	19.7914	47.19697
1370101_at	60446	Crx	cone-rod homeobox protein	29.29958	20.53711	22.27823
1396089_at	297626	NA	NA	29.25952	41.68907	15.8226
1372595_at	291245	Actn2_pre	actinin alpha 2 (predicted)	29.24306	120.2389	29.37057
1380899_at	311550	RGD13052	similar to Protein C20orf160 (predicted	29.12609	126.2434	158.4303
1384753_at	498311	RGD15616	similar to hypothetical protein (predicte	29.03495	51.67946	65.54633
1382488_at	296184	Ankrd5_pre	ankyrin repeat domain 5 (predicted)	29.01002	34.47324	188.9676
1372725_at	315883	LOC31588	similar to phospholipid scramblase 2	29.0069	15.89569	162.8414
1369755_at	64544	B3gat2	beta-1,3-glucuronyltransferase 2 (gluc	28.98944	34.88875	32.73911
1371155_at	29683	Klrc1	killer cell lectin-like receptor subfamily	28.91136	34.52645	76.50473
1369361_at	83504	Kl	Klotho	28.89971	44.12816	9.356756
1372852_at	499300	LOC49930	similar to protein tyrosine phosphatase	28.86212	101.0464	109.0101
1368520_at	25080	Apoa4	apolipoprotein A-IV	28.85443	13.10951	44.34934
1396199_at	500341	NA	NA	28.85324	13.0591	31.13058
1390112_at	305604	Efemp1	epidermal growth factor-containing fibu	28.83821	15.10564	264.5238
1383916_at	295315	Tbx15_pre	T-box 15 (predicted)	28.80926	239.9887	17.41798
1396231_at	299567	LOC29956	similar to Peptidoglycan recognition pr	28.72482	44.76409	19.7794
1375390_at	303885	RGD13054	similar to mKIAA0226 protein (predicte	28.6046	25.93615	41.82858
1387524_at	170926	Cysltr2	cysteinyl leukotriene receptor 2	28.5663	11.74828	38.10962
1384606_at	287111	NA	NA	28.5539	604.5497	75.04094
1369653_at	81810	Tgfbr2	transforming growth factor, beta recep	28.53549	11.69932	20.52213
1370625_at	246274	Faim2	Fas apoptotic inhibitory molecule 2	28.53411	25.77802	66.32942
1385733_at	305000	Exo1_pred	exonuclease 1 (predicted)	28.51068	590.6623	41.90771
1375775_at	500917	RGD15643	similar to shippo 1 (predicted)	28.43245	10.15051	177.934
1369842_at	63866	Accn5	amiloride-sensitive cation channel 5, ir	28.4048	11.4425	118.2463

1370626_at	25223	Tspy	testis specific protein, Y-linked	28.38489	7.360264	15.62351
1371370_a_at	362267	Svs6_pred	seminal vesicle secretion 6 (predicted)	28.28299	21.16954	17.80333
1371084_at	292872	LOC29287	kallikrein	28.24675	34.56367	87.39838
1369348_at	171384	Trpm8	transient receptor potential cation char	28.21304	38.81445	21.03467
1382663_at	299206	Batf_predic	basic leucine zipper transcription facto	28.17419	10.5234	135.7244
1369810_at	24907	Dmd	dystrophin, muscular dystrophy	28.13502	13.53186	44.9972
1390687_at	364206	Plek	pleckstrin	28.12452	17.13857	126.6177
1380134_at	295322	Vtcn1	V-set domain containing T cell activatio	28.07652	40.34518	29.27743
1377104_at	498608	RGD15632	similar to BC028663 protein (predicted	28.02314	10.73215	18.72928
1394368_x_at	362346	RGD13118	similar to RIKEN cDNA 1700016G05 (	27.95223	55.48829	91.68658
1393591_at	290300	RGD15649	similar to tumor necrosis factor recepto	27.91628	12.44753	33.39845
1369427_at	64552	Mpeg1	macrophage expressed gene 1	27.754	35.37277	12.74112
1385500_at	500945	RGD15617	similar to RIKEN cDNA 1700012B09 (p	27.7104	20.34728	8.572036
1386212_at	289257	Spna1	spectrin alpha 1	27.68184	13.95496	16.57421
1371294_at	287168	Hbz	hemoglobin, zeta	27.55734	19.38661	17.44331
1369551_at	64021	Gpr173	G-protein coupled receptor 173	27.52761	70.90743	51.54798
1385063_at	314436	Elk1	ELK1, member of ETS oncogene famil	27.45132	199.4194	36.37654
1378297_at	499927	RGD15615	similar to chromosome 20 open readin	27.41748	272.2771	72.97923
1387582_a_at	140929	Pde7b	phosphodiesterase 7B	27.38989	11.92221	14.30377
1372492_at	290485	Cldn10_pre	claudin 10 (predicted)	27.37094	75.11777	133.7964
1393895_a_at	501592	RGD15641	RGD1564177 (predicted)	27.32672	15.57407	27.50068
1384884_at	295019	RGD13075	similar to RIKEN cDNA 1700018B24 (p	27.27166	19.33291	12.71516
1395348_at	304315	Ttyh3_prec	tweety homolog 3 (Drosophila) (predic	27.23741	100.7193	120.3519
1388181_at	641523	LOC64152	NA	27.23488	17.35008	36.045
1378193_at	293744	Ms4a7_pre	membrane-spanning 4-domains, subfa	27.21581	59.38248	76.6855
1393436_at	309100	Scgb1c1_p	secretoglobin, family 1C, member 1 (p	27.2157	15.04856	17.65998
1374619_at	360576	Tusc5	tumor suppressor candidate 5	27.20601	42.10519	31.73308
1388673_at	361680	Lsp1	lymphocyte specific 1	27.15608	16.9964	25.46952
1385251_at	500638	LOC50063	hypothetical protein LOC500638	27.09664	16.91476	268.7538
1368338_at	117054	Cd52	CD52 antigen	27.01239	61.99064	14.07089
1395493_at	296411	RGD13078	hypothetical LOC296411 (predicted)	26.99611	49.84234	195.1908
1370082_at	59086	Tgfb1	transforming growth factor, beta 1	26.94767	12.90469	37.5122
1367985_at	25748	Alas2	aminolevulinic acid synthase 2	26.92006	59.1115	43.0408
1388069_at	25192	Klrb1b	killer cell lectin-like receptor subfamily	26.86783	111.1934	206.6154
1387736_at	25229	Chrm1	cholinergic receptor, muscarinic 1	26.80987	31.14807	21.32858
1381701_at	312519	Aak1_pred	AP2 associated kinase 1 (predicted)	26.70541	114.6625	12.8311
1389834_at	361692	Nudt8_prec	nudix (nucleoside diphosphate linked r	26.69576	17.49662	11.91852
1387739_at	24931	Cd8b	CD8 antigen, beta chain	26.67063	65.64726	17.78463
1373911_at	361945	Postn_prec	periostin, osteoblast specific factor (pre	26.63621	236.6421	547.0954
1389473_at	306307	Rgr_predic	retinal G protein coupled receptor (pre	26.58742	23.32936	12.94743
1369188_at	171043	Fbxo32	F-box only protein 32	26.57452	112.4883	63.12154
1370222_at	29609	Pitx3	paired-like homeodomain transcription	26.50874	9.808061	21.5436
1376937_at	304020	RGD15659	similar to 4631422O05Rik protein (pre	26.46146	27.59617	554.897
1381460_at	362964	NA	NA	26.44459	38.83444	87.76515
1383365_at	294027	Col17a1_p	procollagen, type XVII, alpha 1 (predic	26.41608	34.08124	32.33278
1387686_at	24559	Mos	v-mos moloney murine sarcoma viral c	26.24178	18.26713	18.51132
1368352_at	24923	Stx1b2	syntaxin 1B2	26.23642	19.97687	125.6037
1390842_at	306862	Tcfap2a_pr	transcription factor AP-2, alpha (predic	26.19011	12.82715	23.03169
1370791_at	498659	RatNP-3b	NA	26.11795	42.97051	71.7132
1391417_at	365972	LOC36597	similar to betaine-homocysteine methy	26.11185	85.13397	71.18122
1367776_at	54237	Cdc2a	cell division cycle 2 homolog A (S. pon	26.09504	13669.05	174.4961

1382618_at	362202	Epb4.2_pre	erythrocyte protein band 4.2 (predicted	26.07633	38.98394	31.40248
1387173_at	25627	Cma1	chymase 1, mast cell	26.07324	16.61859	26.11047
1387626_at	79127	Dck	deoxycytidine kinase	26.04821	149.5543	141.0608
1368339_at	24249	S100g	S100 calcium binding protein G	26.04342	89.01886	74.99441
1373827_at	306929	LOC30692	prolactin-like protein-F beta	25.98062	113.5587	4.863526
1390596_at	293890	Mlana_pre	melan-A (predicted)	25.97853	10.07886	22.24533
1387960_at	64389	Defb3	defensin beta 3	25.94029	58.67426	12.72883
1381801_at	303741	Fscn2_pre	fascin homolog 2, actin-bundling prote	25.90077	43.86236	40.19944
1387584_at	29499	Shh	sonic hedgehog homolog (Drosophila)	25.87065	24.11255	55.11896
1381345_at	310377	RGD13062	similar to 4833420G17Rik protein	25.84205	110.0255	44.8255
1369311_at	25349	Scn2b	sodium channel, voltage-gated, type II	25.78883	26.17293	18.49247
1376296_at	296323	RGD15624	similar to C20orf95 (predicted)	25.76205	58.87661	28.88953
1385058_at	304124	Cldn8	claudin 8	25.7432	8.516676	158.5607
1369461_at	81753	Pthr2	parathyroid hormone receptor 2	25.70248	45.98311	47.71224
1387284_at	65135	Dpys	dihydropyrimidinase	25.69077	8.898901	7.693583
1371026_at	140592	Ppfia4	protein tyrosine phosphatase, receptor	25.6597	27.92378	42.92299
1387965_at	286934	Havcr1	kidney injury molecule 1	25.6184	76.89987	26.2626
1384350_at	315348	Nckap1l_pr	NCK associated protein 1 like (predicte	25.61014	153.7794	37.30341
1374942_at	293566	Cpxm2_pre	carboxypeptidase X 2 (M14 family) (pr	25.58036	12.42976	23.532
1369749_a_at	25013	Svs2	seminal vesicle protein, secretion 2	25.46681	14.70562	46.21622
1370496_at	24303	Cyp2d13	cytochrome P450, family 2, subfamily c	25.40084	41.59148	52.48973
1383480_at	501597	RGD15616	similar to Gene model 784 (predicted)	25.32741	26.96135	36.80633
1369184_at	155268	Cldn16	claudin 16	25.26589	58.74761	28.43919
1389542_at	362044	Cenpe_pre	centromere protein E (predicted)	25.25931	882.2149	13.18211
1368948_at	81521	Msn	moesin	25.25122	146.8889	21.30481
1382192_at	293186	Xlkd1_prec	extracellular link domain-containing 1 (	25.11019	92.63293	64.89229
1368493_at	114903	Lim2	lens intrinsic membrane protein 2	25.08606	49.97187	87.99785
1391058_at	305307	RGD13109	similar to RIKEN cDNA C130090K23 (	25.03422	4.0888	50.30351
1378483_at	266804	Prlpn	prolactin-like protein N	24.99219	9.641438	42.15486
1369762_at	25517	Pou1f1	POU domain, class 1, transcription fac	24.93536	38.2757	77.39689
1369671_at	25611	Otc	ornithine transcarbamylase	24.68123	19.20051	19.19604
1369342_at	24941	Atp7a	ATPase, Cu++ transporting, alpha poly	24.66591	122.5757	21.43585
1368170_at	79212	Slc6a1	solute carrier family 6 (neurotransmitte	24.55239	81.11888	351.8625
1380906_at	313499	NA	NA	24.4766	9.160384	60.61182
1395041_at	310900	Mttp	microsomal triglyceride transfer protein	24.46281	74.59916	12.69228
1389475_at	25273	Smo	smoothened homolog (Drosophila)	24.41696	53.40848	103.8276
1369550_at	29152	Gdf8	growth differentiation factor 8	24.41015	5.386112	8.769106
1370022_at	24840	Tnp2	transition protein 2	24.36398	9.315385	32.30644
1398043_at	497651	Rnase11	ribonuclease 11	24.35171	11.28002	113.6382
1370377_at	266684	Cyp2d9	cytochrome P450, family 2, subfamily c	24.24725	38.531	41.23
1376311_at	295382	RGD15634	similar to netrin G1 (predicted)	24.24708	7.535293	98.24673
1392074_at	500046	LOC50004	similar to hypothetical protein FLJ2198	24.2452	12.19327	209.0561
1388123_at	293731	Psbpc2	prostatic steroid-binding protein C2	24.24231	45.91711	112.6367
1387572_at	171381	Psd	pleckstrin and Sec7 domain containing	24.14699	31.17109	36.72567
1378015_at	298006	Ccl21b	chemokine (C-C motif) ligand 21b (ser	24.10688	47.0945	16.95106
1369856_at	25195	Drd5	dopamine receptor D5	24.03109	62.71953	10.58534
1397842_at	84402	Sfrp1	secreted frizzled-related protein 1	24.01678	10.12387	229.8909
1387868_at	29469	Lbp	lipopolysaccharide binding protein	24.00965	68.29744	36.65767
1369964_at	155151	Coro1a	coronin, actin binding protein 1A	23.98312	1492.333	8.714754
1387613_at	66024	Npy2r	neuropeptide Y receptor Y2	23.85433	41.23368	30.17028
1369201_at	29582	Mgat3	mannoside acetyl glucosaminyltransfe	23.83994	102.155	20.65711

1383937_at	362280	Tcfap2c	transcription factor AP-2, gamma	23.80727	16.52937	13.85079
1387610_at	29333	Mcp	membrane cofactor protein	23.68784	136.0602	127.3058
1387363_at	85309	Folh1	folate hydrolase	23.62723	54.06693	183.1595
1394128_at	25642	Cyp3a3	cytochrome P450, subfamily 3A, polyp	23.58229	12.8121	188.4959
1378599_at	303181	Wnt3a_pre	wingless-related MMTV integration site	23.5408	10.32188	36.24454
1397402_at	499096	NA	NA	23.53946	153.6168	50.8455
1384951_at	311716	RGD15639	similar to Protein KIAA1510 precursor	23.49326	70.46836	109.7963
1387321_at	84396	Atp1b4	ATPase, (Na+)/K+ transporting, beta 4	23.41857	28.85806	31.94676
1368766_at	25335	Mmp7	matrix metallopeptidase 7	23.40969	45.63494	22.39204
1380806_at	309533	Sorcs1_pre	VPS10 domain receptor protein SORC	23.39104	115.3731	130.0373
1367968_at	171161	LOC17116	common salivary protein 1	23.38575	3.885364	28.6511
1385189_at	311374	RGD13066	similar to cDNA sequence BC019755 (	23.3272	32.24211	360.528
1370774_at	257653	Dcm5	Dcm5 protein	23.25014	62.93042	59.96875
1378484_at	315762	Rasl12_pre	RAS-like, family 12 (predicted)	23.14036	79.78198	221.9263
1377031_at	366225	LOC36622	NA	23.129	44.53902	20.07925
1396609_at	498655	NA	NA	23.09078	29.21021	8.054108
1387758_at	64621	Alpi2	intestinal alkaline phosphatase-II (IAP-	23.0874	30.64556	15.83828
1371054_at	24661	Pnmt	phenylethanolamine-N-methyltransfera	23.00808	10.51926	41.7463
1368416_at	24477	Ibsp	integrin binding sialoprotein	23.00024	7.778809	35.97046
1367858_at	25481	Mmp11	matrix metallopeptidase 11	22.74341	175.4029	191.8455
1398243_at	117505	Csrp3	cysteine and glycine-rich protein 3	22.63583	22.00338	61.51648
1387330_at	79110	Mepe	matrix extracellular phosphoglycoprote	22.61945	50.09506	25.88665
1388235_at	498341	VCS-beta1	PR-Vbeta1	22.59784	10.52005	146.6632
1382787_at	314906	Kif5a	kinesin family member 5A	22.58272	6.801549	15.1624
1368617_at	65051	Serpina5	serine (or cysteine) peptidase inhibitor	22.55514	42.64437	34.81921
1394334_at	308511	Chst8_prec	carbohydrate (N-acetylgalactosamine 4	22.52986	120.7552	22.28027
1384735_at	498267	RGD15605	similar to C130085G02Rik protein (pre	22.51582	34.24536	17.46469
1398514_at	360719	Hgd	homogentisate 1, 2-dioxygenase	22.4241	91.9293	138.2315
1393771_at	338418	Scgb3a1	secretoglobin, family 3A, member 1	22.39241	11.70334	23.9099
1369593_at	170669	Cgi94	comparative gene identification transcı	22.36097	18.39036	26.23094
1391429_at	310681	Hfe2	hemochromatosis type 2 (juvenile) hor	22.32388	21.162	51.03589
1380726_at	306805	LOC30680	similar to asporin precursor	22.3191	3.242407	750.5728
1393897_at	363948	Cdh20	cadherin 20	22.25704	26.90581	23.4318
1384775_s_at	287106	Tmprss8	transmembrane protease, serine 8 (int	22.23566	131.8803	184.7632
1384825_at	499089	LOC49908	NA	22.22655	9.163533	15.78255
1369916_at	308586	RGD15624	similar to GDP-L-fucose:beta-D-galact	22.2094	25.77266	18.65016
1388189_at	24416	Grm3	glutamate receptor, metabotropic 3	22.1806	45.65346	36.04215
1368252_at	117537	Kbtbd10	kelch repeat and BTB (POZ) domain c	22.08414	32.43688	84.3996
1398307_at	252931	Cyp3a18	cytochrome P450, 3a18	22.05041	98.81262	29.86624
1383943_at	252893	Dnah7	dynein, axonemal, heavy polypeptide 7	22.03482	20.66939	148.9032
1379202_at	313668	RGD13118	similar to RIKEN cDNA 1700027M01	21.9767	20.18294	45.91742
1395778_at	308064	RGD15663	similar to RIKEN cDNA 4933440J22 (p	21.97242	97.12506	10.89195
1387249_at	114496	Bik	Bcl2-interacting killer	21.95743	44.5986	74.75686
1387518_at	81923	Crisp2	cysteine-rich secretory protein 2	21.95461	29.47955	33.3418
1377873_at	497982	NA	NA	21.94085	74.58404	79.06702
1369303_at	81648	Crh	corticotropin releasing hormone	21.92504	29.89388	129.4502
1393228_at	313521	Gloxd1	glyoxalase domain containing 1	21.89149	66.55949	30.29544
1385336_at	290361	Dok2_pred	docking protein 2 (predicted)	21.87595	22.93115	32.80315
1369576_at	78985	Tas2r105	taste receptor, type 2, member 105	21.76853	90.54955	20.2374
1384924_at	113910	Prnd	prion protein dublet	21.76774	4.866341	44.42703
1381833_at	305984	Cdca2	cell division cycle associated 2	21.73243	1190.302	67.69761

1369790_at	24813	Tat	tyrosine aminotransferase	21.62619	9.406422	14.94035
1393902_at	307092	Akr1c6	aldo-keto reductase family 1, member	21.61393	13.00619	8.869117
1368128_at	29692	Pla2g2a	phospholipase A2, group IIA (platelets	21.54227	9.140824	46.92409
1391265_at	498236	LOC49823	LRRGT00186	21.51751	91.90959	26.94522
1378703_at	499295	NA	NA	21.50282	47.14023	67.99373
1375750_at	499919	NA	NA	21.478	52.30914	61.13025
1393737_at	297419	RGD1311	similar to RIKEN cDNA 1810036H07 (	21.40215	66.37125	111.1795
1373410_at	499497	RGD15631	similar to MADS box transcription enha	21.36214	25.43001	210.4912
1370214_at	25269	Pvalb	parvalbumin	21.25842	11.66381	12.55448
1371475_at	56759	Rnase4	ribonuclease, RNase A family 4	21.21709	56.08115	1090.587
1371628_at	292668	LOC29266	pregnancy-specific beta 1-glycoprotein	21.19562	12.38878	31.39731
1370416_at	252915	Mxd3	Max dimerization protein 3	21.1536	842.9823	116.9682
1386122_at	293990	Wnt8b_pre	wingless related MMTV integration site	21.09363	38.38497	61.47841
1369869_at	29324	Capza3	capping protein (actin filament) muscle	21.06165	15.31777	19.61548
1382184_at	548326	Faim3	Fas apoptotic inhibitory molecule	21.03337	30.67647	257.2725
1387573_a_at	60349	Nr5a2	nuclear receptor subfamily 5, group A,	20.88845	24.46011	20.74919
1381311_at	316137	Emr1	EGF-like module containing, mucin-like	20.81554	4.442956	118.2497
1382153_at	474143	Clecsf6	C-type (calcium dependent, carbohydr	20.6952	107.2834	83.96608
1373000_at	317181	Srpx2_pre	sushi-repeat-containing protein, X-link	20.67227	16.51814	42.39749
1375772_at	366604	NA	NA	20.63635	21.04979	114.6781
1393632_at	305423	C1qtnf7_pr	C1q and tumor necrosis factor related	20.56876	5.921361	33.57458
1387508_at	29725	Baat	bile acid-Coenzyme A: amino acid N-a	20.52522	15.6164	11.56797
1369001_at	25101	Chrna3	cholinergic receptor, nicotinic, alpha p	20.50494	41.00532	102.3245
1368227_at	60423	Slc28a2	solute carrier family 28 (sodium-couple	20.33465	44.14463	37.72314
1376017_at	361188	LOC36118	NA	20.32942	31.18862	31.9147
1370777_at	192264	Ear11	eosinophil-associated, ribonuclease A	20.31616	109.8282	70.79353
1388013_at	25408	Cd80	CD80 antigen	20.26784	12.33402	153.3959
1398720_at	297026	NA	NA	20.13025	80.49623	8.433345
1368884_at	64519	Entpd1	ectonucleoside triphosphate diphosph	20.12435	22.28599	46.01608
1398655_at	337868	Myod1	myogenic differentiation 1	19.96828	8.460676	13.31947
1391121_at	306506	RGD13117	similar to DNA segment, Chr 8, ERAT	19.95431	20.08297	42.15993
1388557_at	117517	C7	complement component 7	19.942	8.614943	5799.599
1373276_at	499657	RGD15622	similar to S100 calcium-binding protein	19.88494	17.06699	6.127337
1387791_at	24310	Ace	angiotensin I converting enzyme (pept	19.79861	126.9982	204.961
1369364_at	29132	Atp1a4	ATPase, Na+/K+ transporting, alpha 4	19.76959	46.35671	14.5483
1367615_at	171027	Svs5	seminal vesicle secretion 5	19.73735	17.30341	14.88971
1384020_at	316275	RGD13117	similar to putative membrane steroid re	19.73414	196.56	206.0401
1387943_at	286995	Defa	defensin, alpha 5, Paneth cell-specific	19.58369	12.22077	30.85054
1384713_at	316318	RGD1561	similar to TESP2 (predicted)	19.54529	12.85916	6.991741
1374061_at	295629	Cd302	CD302 antigen	19.53795	60.78378	803.4182
1387615_at	117523	St8sia2	ST8 alpha-N-acetyl-neuraminide alpha	19.5237	8.940223	23.81267
1384834_at	305497	Cobl_predi	cordon-bleu (predicted)	19.50212	953.6839	724.262
1374148_at	498009	NA	NA	19.48107	92.27262	24.91191
1369871_at	29183	Areg	amphiregulin	19.46998	210.5804	2027.116
1386053_at	500554	RGD15641	similar to RIKEN cDNA 1700025K23 (	19.39807	25.7698	33.34286
1387590_at	170704	Hrh4	histamine H4 receptor	19.34221	31.61571	19.439
1370700_at	286936	Pcyt1b	phosphate cytidylyltransferase 1, choli	19.30824	258.3646	54.26359
1368968_at	25387	Arrb1	arrestin, beta 1	19.30178	92.78769	40.08184
1385748_at	366491	Spata21	spermatogenesis associated 21	19.26987	26.92515	54.05454
1370227_at	286889	LOC28688	prolactin-like protein C related	19.26971	28.10032	51.60385
1368628_at	24841	Ton	tonin	19.18773	45.7794	12.83784

1370072_at	24590	Mme	membrane metallo endopeptidase	19.04857	230.8443	112.1381
1395811_at	289656	RGD15627	similar to 60S ribosomal protein L23a (	18.98588	8.234492	26.21395
1398551_at	303534	RGD1311{	similar to hypothetical protein DKFZp4	18.97704	1005.791	170.5614
1369229_at	171115	Pde5a	phosphodiesterase 5A, cGMP-specific	18.95241	105.2463	30.8784
1369047_at	60393	Sult1d1	sulfotransferase family 1D, member 1	18.8667	102.1059	88.55879
1387712_at	64628	Hes3	hairy and enhancer of split 3 (Drosoph	18.82409	22.72012	32.84661
1393603_at	316010	Camp	cathelicidin antimicrobial peptide	18.78248	36.04913	51.8393
1370545_at	24520	Kcna1	potassium voltage-gated channel, shal	18.6445	8.103475	14.69045
1388042_at	246325	Kcnh8	NA	18.53724	15.26009	80.91417
1396935_at	363079	RGD15637	similar to MEGF11 protein (predicted)	18.41483	37.24874	52.52646
1369491_at	114027	Dao1	D-amino acid oxidase 1	18.39147	20.88346	53.11154
1387603_at	29684	Klrc2	killer cell lectin-like receptor subfamily	18.37297	23.44397	28.62807
1387319_at	29397	Ccl11	chemokine (C-C motif) ligand 11	18.35088	38.337	124.102
1382531_at	317468	Tlr7_predic	toll-like receptor 7 (predicted)	18.3499	12.8145	94.92607
1389934_at	315833	RGD15605	similar to hypothetical protein D930047	18.33473	58.30356	16.46002
1370645_at	286984	LOC28698	putative pheromone receptor (Go-VN4	18.32902	29.13598	27.11621
1377655_at	170630	Fgf12	fibroblast growth factor 12	18.32857	609.1405	55.2712
1369784_at	54314	Tpo	thyroid peroxidase	18.32461	12.83368	114.1291
1381446_at	353306	LOC35330	vitamin A-deficient testicular protein 11	18.26659	46.67777	6.597725
1387251_at	29311	Msmb	beta-microseminoprotein	18.24698	21.19443	15.02296
1387304_at	57305	Uts2r	urotensin 2 receptor	18.21833	41.33651	11.89589
1387553_at	25276	Sycp1	synaptonemal complex protein 1	18.14723	8.386061	49.55329
1398275_at	81687	Mmp9	matrix metallopeptidase 9	18.01572	32.08347	17.14434
1381340_at	288478	RGD1311{	hypothetical LOC288478	17.98026	7.140135	14.92188
1393826_at	304603	RGD1311{	similar to apolipoprotein F-like	17.75981	60.92617	57.308
1369395_at	64560	Tpc1808	tropic 1808	17.63651	33.91829	26.29966
1385522_at	313479	Orc1l	origin recognition complex, subunit 1-li	17.60349	108.7247	196.202
1369844_at	24947	Gabra3	gamma-aminobutyric acid (GABA-A) re	17.53495	10.72001	14.78197
1369317_at	29552	Erabp	epididymal retinoic acid-binding protei	17.3367	8.152211	73.89652
1387997_at	59266	Hcn4	hyperpolarization-activated, cyclic nucl	17.33397	22.05818	17.97895
1396873_at	366889	LOC36688	NA	17.19983	5.328914	35.41603
1380894_at	308350	RGD15663	similar to PIRB1 (predicted)	17.1508	10.90096	123.4885
1371115_at	114767	Ptpre	protein tyrosine phosphatase, receptor	17.14056	11.53499	53.89059
1385130_at	305719	Zfp312_pre	zinc finger protein 312 (predicted)	17.11988	58.79843	31.92269
1381573_at	363205	RGD15621	similar to class-alpha glutathione S-tra	17.00498	11.69045	75.59306
1378470_at	315741	Paqr5	progestin and adipoQ receptor family r	16.8813	41.73366	39.28386
1387710_at	25342	Oxtr	oxytocin receptor	16.8483	26.17261	152.1494
1369153_at	64563	Nphs1	nephrosis 1 homolog, nephrin (human)	16.82467	45.17459	129.9862
1368587_at	25292	Apoc1	apolipoprotein C-I	16.70026	11.32289	18.98931
1392731_at	156767	Tnfrsf1b	tumor necrosis factor receptor superfa	16.69472	17.03577	24.43392
1386354_at	502002	RGD15648	similar to natural killer cell protease 7 (	16.65308	31.0347	22.69208
1370963_at	85246	Gas7	growth arrest specific 7	16.65195	416.7189	52.58606
1378534_at	308394	LOC30839	similar to brain carcinoembryonic antig	16.64946	23.45823	7.023414
1369432_at	54239	Chrnb2	cholinergic receptor, nicotinic, beta pol	16.62351	46.45656	32.61245
1369769_at	25471	Kcne1	potassium voltage-gated channel, Isk-r	16.526	15.77865	26.73702
1393589_at	317196	RGD15654	similar to KIAA1687 protein (predicted)	16.46892	13.47262	21.63515
1369195_at	25598	Fabp2	fatty acid binding protein 2, intestinal	16.42922	8.24906	85.69794
1369142_at	25295	Bglap2	bone gamma-carboxyglutamate protei	16.37838	20.11195	20.15373
1380077_at	499271	RGD15611	similar to mFLJ00114 protein (predicte	16.37067	28.51296	112.6784
1371105_at	114005	Epm2a	epilepsy, progressive myoclonic epilep	16.35582	15.26591	21.18899
1370139_a_at	89823	Trpc6	transient receptor potential cation char	16.34555	52.47127	92.09963

1396035_at	297757	RGD15597	similar to RPE-spondin (predicted)	16.33654	98.60389	128.6501
1368110_a_at	192117	Syngap1	synaptic Ras GTPase activating protei	16.3161	24.59854	25.20848
1370795_at	171356	Foxc2	forkhead box C2	16.23455	19.77032	127.1649
1368940_at	29597	P2ry2	purinergic receptor P2Y, G-protein cou	16.06929	20.3645	81.6436
1391357_at	500913	RGD1566C	similar to mKIAA1644 protein (predicte	16.0627	50.42272	217.4059
1378762_at	306451	Odz3_pred	odd Oz/ten-m homolog 3 (Drosophila)	15.89214	27.92353	64.11105
1385702_at	304988	Mnda	myeloid cell nuclear differentiation anti	15.66799	6.815983	438.1171
1394309_at	363538	RGD1307C	similar to hypothetical protein FLJ3287	15.59846	49.52861	39.73801
1390413_at	308794	RGD13103	similar to RIKEN cDNA 1700026D08	15.50948	9.709121	7.93331
1391059_at	259224	LOC25922	prepro-Neuropeptide W polypeptide	15.50441	39.24776	50.23362
1384952_at	315907	Nrk_predic	Nik related kinase (predicted)	15.35772	6.157018	12.08921
1391371_at	29281	Prodh	proline dehydrogenase	15.32446	18.70811	154.6474
1387380_at	83612	Slc32a1	solute carrier family 32 (GABA vesicula	15.19847	139.1927	22.29757
1369855_at	24448	Hrh1	histamine receptor H 1	15.13689	43.87115	13.18047
1381753_at	361907	Fbxl7_prec	F-box and leucine-rich repeat protein 7	15.12776	67.83824	173.8833
1369143_at	171131	Chrnb3	cholinergic receptor, nicotinic, beta pol	15.12642	7.487885	10.1402
1388605_at	296366	Wfdc3_pre	WAP four-disulfide core domain 3 (pre	15.06743	7.291217	10.32725
1380845_at	300384	Naalad2_p	N-acetylated alpha-linked acidic dipept	15.06332	7.312896	41.20564
1395416_at	296372	Ncoa5_pre	nuclear receptor coactivator 5 (predicte	14.93527	541.0631	163.0444
1371250_at	360918	Cxcl4	chemokine (C-X-C motif) ligand 4	14.75944	112.8139	161.2706
1374317_at	497932	RGD15608	similar to Expressed sequence BB220	14.6025	26.83608	10.16909
1387323_at	25048	Klkb1	kallikrein B, plasma 1	14.59863	26.34425	166.4583
1377659_at	310483	Mlf1_predi	myeloid leukemia factor 1 (predicted)	14.39142	599.1984	8970.268
1386623_at	307850	Exosc6_pr	exosome component 6 (predicted)	14.37552	17.92242	35.15128
1376286_at	298602	RGD1566C	similar to hypothetical protein MGC240	14.35795	47.44495	58.78967
1370790_at	25712	Ifng	interferon gamma	14.20571	17.75012	74.27368
1378779_at	367874	RGD15635	similar to 60S ribosomal protein L29 (F	14.18393	19.95303	23.48861
1397048_at	501710	NA	NA	14.08662	7.376365	11.54449
1370149_at	24210	Asgr1	asialoglycoprotein receptor 1	14.03145	32.75909	11.91172
1390236_at	311858	RGD15659	similar to hypothetical protein (predicte	14.00666	9.501023	22.29457
1392419_at	298563	RGD13118	similar to RIKEN cDNA 4930549C01 (p	13.97864	70.36339	35.29806
1375485_at	266813	Prrx1	paired related homeobox 1	13.93429	14.50086	164.5808
1377139_at	304514	Tbx5	T-box 5	13.84097	11.68944	15.85638
1390798_at	24699	Ptprc	protein tyrosine phosphatase, receptor	13.72264	40.6112	137.7823
1377557_at	503453	NA	NA	13.71919	48.4555	26.07186
1393791_at	498075	MGC1250C	similar to T cell receptor interacting mo	13.70186	25.07033	13.83678
1368048_at	24794	Spin2b	Serine protease inhibitor	13.59841	24.58377	17.95298
1395094_at	114502	Pax3	paired box gene 3	13.53616	27.84069	75.32499
1393139_at	292697	Apoc2_pre	apolipoprotein C-II (predicted)	13.39203	32.49485	13.05255
1393473_at	315652	RGD15638	RGD1563866 (predicted)	13.27592	4.885105	55.10855
1369424_at	24895	Cyp2a2	cytochrome P450, subfamily 2A, polyp	13.16055	10.57618	38.0193
1371079_at	289211	Fcgr2b	Fc receptor, IgG, low affinity IIb	13.0715	29.4956	25.51028
1370106_at	29369	Fgf18	fibroblast growth factor 18	13.05667	127.3507	34.18812
1375752_at	365603	Bves	NA	12.72257	55.01253	22.44791
1369114_at	24867	Vcsa1	variable coding sequence A1	12.71139	60.36291	51.23093
1392565_at	312855	Ppfibp1_pr	PTPRF interacting protein, binding pro	12.60637	19.52196	58.1893
1396605_at	300141	Mov10l1_p	Moloney leukemia virus 10-like 1 (prec	12.57781	14.51239	7.791633
1387534_at	25310	Cyct	cytochrome c, testis	12.29306	23.98591	131.7657
1385685_at	288075	Stfa3_pred	stefin A3 (predicted)	12.22708	95.86584	15.82858
1379852_at	311721	Urkl1_pred	uridine kinase-like 1 (predicted)	11.90934	22.77896	110.7211
1369466_at	65024	Chrna9	cholinergic receptor, nicotinic, alpha po	11.90856	34.95179	47.75294

1380364_at	500686	RGD15611	similar to Gene model 1568 (predicted	11.74427	17.56619	14.85165
1377775_at	295190	Rptn_predi	repetin (predicted)	11.56989	44.25685	6.269897
1394412_at	690914	LOC69091	NA	11.48485	28.54256	40.86732
1369283_at	25609	Tub	tubby homolog (mouse)	11.43585	11.58445	31.57379
1387715_at	171059	Expi	extracellular peptidase inhibitor	11.14118	41.5819	105.2777
1385300_at	303533	Klhl10	kelch-like 10 (Drosophila)	11.01869	139.9669	20.84086
1374125_at	499951	LOC49995	similar to Transcription factor GATA-5	10.90652	41.35888	32.70281
1369072_at	171178	Adh7	alcohol dehydrogenase 7 (class IV), m	10.90231	30.59208	8.834812
1370449_at	171108	P2ry14	purinergic receptor P2Y, G-protein cou	10.71602	149.1507	55.4326
1377585_at	363508	Vgll1_pred	vestigial like 1 homolog (Drosophila) (p	10.61014	11.64971	13.52454
1396932_at	366403	Fam29a_p	family with sequence similarity 29, mei	10.48946	5.906416	3.803694
1371164_at	54269	Mcpt10	mast cell protease 10	10.48128	9.412844	66.44725
1377014_at	310376	RGD13081	similar to hypothetical protein MGC421	10.43448	14.22836	1022.897
1397856_at	361974	Pet112l_pr	PET112-like (yeast) (predicted)	10.41317	245.0176	131.6718
1368720_at	64206	Tdo2	tryptophan 2,3-dioxygenase	10.21147	39.40716	336.1074
1394297_at	288151	Hoxd1_pre	homeo box D1 (predicted)	9.951849	46.57117	60.2549
1374520_at	498875	RGD15644	similar to heart alpha-kinase (predicted	9.621443	9.806758	205.6642
1369429_at	117098	Pdha2	pyruvate dehydrogenase E1 alpha 2	9.056278	3.755658	6.790658
1385392_at	494192	Mycbpap	Mycbp associated protein	8.55688	13.95119	28.66377
1369451_a_at	24521	Kcnj1	potassium inwardly-rectifying channel,	8.441656	69.78944	138.3314
1371143_at	81806	Serpina7	serine (or cysteine) peptidase inhibitor	8.377861	5.268819	21.60127
1385736_at	315650	Btg4	B-cell translocation gene 4	8.356812	6.79013	12.35448
1392455_at	361021	NA	NA	8.317322	24.03542	114.9966
1373805_at	290975	Ctsr	cathepsin R	7.451827	28.54625	86.79215
1370359_at	24203	Amy1	amylase 1, salivary	6.834894	25.28961	72.22729
1397360_at	365009	Clecsf1_pr	C-type (calcium dependent, carbohydr	6.193258	8.75524	4.862681
1385824_at	246304	Cap350	centrosome-associated protein 350	5.557781	22.11182	106.0073
1369258_at	84597	Fut9	fucosyltransferase 9	5.545745	20.79474	69.3237
1387827_x_at	64647	Hist1h2bl	histone 1, H2bl	4.205652	50.73252	87.65298
1385248_a_at	291015	Ogn_predic	osteoglycin (predicted)	4.20486	22.3187	1561.393
1379653_a_at	501502	RGD15624	similar to SH2 domain protein 1A (Sigr	3.982018	78.33488	11.69701

Differential Expression Analysis: Beta cells vs Alpha cells

probes	entrez_ger	symbols	gene_names	logFC	Pvalue	AdjP Value
1387154_at	24604	Npy	neuropeptide Y	7.050027	6.85E-07	0.009065
1370384_a_at	24684	Prlr	prolactin receptor	4.558402	3.80E-06	0.009185
1387599_a_at	24314	Nqo1	NAD(P)H dehydrogenase, quinone 1	6.774071	4.74E-06	0.009185
1383075_at	58919	Ccnd1	cyclin D1	4.997401	5.32E-06	0.009185
1370959_at	84032	Col3a1	procollagen, type III, alpha 1	-5.629455	6.41E-06	0.009185
1381100_at	367072	Arhgef12	Rho guanine nucleotide exchange fact	3.756129	1.05E-05	0.009185
1369453_at	117277	Epn1	Epsin 1	3.586962	1.06E-05	0.009185
1385073_at	314232	NA	NA	-5.229193	1.21E-05	0.009185
1385020_at	294066	RGD13087	similar to DNA segment, Chr 19, Brigh	3.343305	1.52E-05	0.009185
1370898_at	29140	Snn	stannin	-3.550169	1.62E-05	0.009185
1368650_at	81813	Klf10	Kruppel-like factor 10	-3.398547	1.81E-05	0.009185
1374864_at	306141	Spry2	sprouty homolog 2 (Drosophila)	-3.751854	1.82E-05	0.009185
1384312_at	306659	Irx1_predic	Iroquois related homeobox 1 (Drosoph	-6.144224	1.86E-05	0.009185
1398256_at	24494	Il1b	interleukin 1 beta	-3.862699	1.95E-05	0.009185
1377867_at	313837	RGD15622	similar to Glutaminyl-peptide cyclotrans	-3.965575	2.01E-05	0.009185
1369699_at	25051	Glp1r	glucagon-like peptide 1 receptor	4.5451	2.01E-05	0.009185

1377952_at	315722	Adpgk	ADP-dependent glucokinase	3.176272	2.06E-05	0.009185
1388471_at	314683	Tcp11l2	t-complex 11 (mouse) like 2	-4.251782	2.09E-05	0.009185
1372383_at	246254	Gpsm1	G-protein signalling modulator 1 (AGS	3.764307	2.09E-05	0.009185
1367635_at	25506	P4hb	prolyl 4-hydroxylase, beta polypeptide	3.301935	2.10E-05	0.009185
1390001_at	306004	Rhobtb2	Rho-related BTB domain containing 2	3.344514	2.11E-05	0.009185
1369203_at	114557	Wif1	Wnt inhibitory factor 1	-3.615471	2.13E-05	0.009185
1398659_at	501231	LOC50123	NA	-4.964931	2.25E-05	0.009185
1381107_at	307924	Zfp276	zinc finger protein (C2H2 type) 276	5.347398	2.30E-05	0.009185
1380021_at	310772	Dennd2d_p	DENN/MADD domain containing 2D (p	5.502488	2.34E-05	0.009185
1375412_at	25227	Arsb	arylsulfatase B	-3.107253	2.45E-05	0.009185
1372601_at	282840	Atf5	activating transcription factor 5	4.558024	2.71E-05	0.009185
1375992_at	499765	RGD15641	similar to FLJ46082 protein (predicted)	-3.142365	2.77E-05	0.009185
1377659_at	310483	Mlf1_predi	myeloid leukemia factor 1 (predicted)	-9.283798	2.79E-05	0.009185
1370795_at	171356	Foxc2	forkhead box C2	-2.969562	2.82E-05	0.009185
1387883_a_at	81814	Tmsb4x	thymosin, beta 4	-4.064997	2.93E-05	0.009185
1382088_at	84025	Ryr2	ryanodine receptor 2, cardiac	-3.828118	3.26E-05	0.009185
1382379_at	94196	Rnf138	ring finger protein 138	-3.135864	3.36E-05	0.009185
1372389_at	494344	Ier2	immediate early response 2	-3.42186	3.36E-05	0.009185
1368323_at	29436	Tfpi	tissue factor pathway inhibitor	-3.613359	3.45E-05	0.009185
1368123_at	25718	Igf1r	insulin-like growth factor 1 receptor	4.015175	3.51E-05	0.009185
1397856_at	361974	Pet112l_pr	PET112-like (yeast) (predicted)	-3.660466	3.66E-05	0.009185
1389655_at	287593	RGD13068	similar to A230072I16Rik protein	2.704101	3.69E-05	0.009185
1369787_at	24889	Cckar	cholecystokinin A receptor	3.949691	3.78E-05	0.009185
1376726_at	360599	NA	NA	5.944369	3.80E-05	0.009185
1387260_at	114505	Klf4	Kruppel-like factor 4 (gut)	-5.54351	3.81E-05	0.009185
1388000_at	84550	Slc24a2	solute carrier family 24 (sodium/potass	2.920826	3.85E-05	0.009185
1370234_at	25661	Fn1	fibronectin 1	4.329374	4.08E-05	0.009185
1387998_at	245709	Exoc8	exocyst complex component 8	4.980993	4.23E-05	0.009185
1378098_at	302913	RGD13097	similar to CG4768-PA (predicted)	-3.073839	4.26E-05	0.009185
1389918_at	290704	LOC29070	NA	2.912871	4.27E-05	0.009185
1370073_at	63880	Dnajc3	DnaJ (Hsp40) homolog, subfamily C, r	3.535654	4.29E-05	0.009185
1367599_at	29754	Atp5g1	ATP synthase, H+ transporting, mitoch	2.75346	4.30E-05	0.009185
1369943_at	56083	Tgm2	transglutaminase 2, C polypeptide	-3.130645	4.31E-05	0.009185
1379663_at	298267	Cachd1_pr	cache domain containing 1 (predicted)	-2.653477	4.32E-05	0.009185
1370555_at	266760	Vgcnl1	voltage gated channel like 1	-5.347407	4.37E-05	0.009185
1372266_at	309812	Rev3l	REV3-like, catalytic subunit of DNA po	-3.004551	4.45E-05	0.009185
1376184_at	300018	Lynx1_pre	Ly6/neurotoxin 1 (predicted)	-3.01275	4.60E-05	0.009185
1375548_at	298425	RGD13103	similar to RIKEN cDNA 4732418C07 (p	2.98073	4.69E-05	0.009185
1369931_at	25630	Pkm2	pyruvate kinase, muscle	3.256941	4.76E-05	0.009185
1374195_at	313325	Lad1_pred	ladinin (predicted)	2.571464	4.98E-05	0.009185
1384437_at	317575	Smarca1_p	SWI/SNF related, matrix associated, a	-4.273322	4.99E-05	0.009185
1390208_at	292935	Htatip2_pre	HIV-1 tat interactive protein 2, homolog	2.623535	5.12E-05	0.009185
1370080_at	24451	Hmox1	heme oxygenase (decycling) 1	2.963909	5.31E-05	0.009185
1367966_at	114591	Dpp3	dipeptidylpeptidase 3	3.08461	5.34E-05	0.009185
1369879_a_at	24822	Tegt	testis enhanced gene transcript	3.065033	5.37E-05	0.009185
1368303_at	63840	Per2	period homolog 2 (Drosophila)	-3.39292	5.43E-05	0.009185
1369982_at	81637	Ap2a2	adaptor protein complex AP-2, alpha 2	2.522435	5.58E-05	0.009185
1388715_at	297113	Gars	glycyl-tRNA synthetase	2.607402	5.60E-05	0.009185
1393091_at	310086	RGD13112	similar to RIKEN cDNA D130064H19 (	-2.668235	5.62E-05	0.009185
1393641_at	499356	Blnk	B-cell linker	-3.892325	5.62E-05	0.009185
1373815_at	290994	Lman2_pre	lectin, mannose-binding 2 (predicted)	3.095874	5.63E-05	0.009185

1377868_at	317405	RGD1561(	similar to hypothetical protein (predicte	-2.781762	5.72E-05	0.009185
1367953_at	25232	Tyro3	TYRO3 protein tyrosine kinase 3	4.460053	5.85E-05	0.009185
1391212_at	302593	Tceal1	transcription elongation factor A (SII)-li	-2.690812	5.86E-05	0.009185
1390430_at	259241	Nr1d2	nuclear receptor subfamily 1, group D,	-2.914592	5.89E-05	0.009185
1383140_at	361840	Spock2_pr	sparc/osteonectin, cwcv and kazal-like	3.075359	6.05E-05	0.009185
1382521_at	24398	Gls	glutaminase	-2.95739	6.14E-05	0.009185
1385217_at	80338	Zbtb10	zinc finger and BTB domain containing	-3.537596	6.15E-05	0.009185
1388695_at	299857	Shmt2	serine hydroxymethyl transferase 2 (m	2.533148	6.21E-05	0.009185
1370845_at	64467	Entpd2	ectonucleoside triphosphate diphosph	2.965396	6.27E-05	0.009185
1389781_at	315670	Elmod1_pr	ELMO domain containing 1 (predicted)	-6.43457	6.29E-05	0.009185
1372626_at	498994	NA	NA	2.96798	6.35E-05	0.009185
1370201_at	83839	Calb1	calbindin 1	-3.964273	6.49E-05	0.009185
1374389_at	25202	Gucy1b3	guanylate cyclase 1, soluble, beta 3	-3.565243	6.56E-05	0.009185
1368806_at	29360	Sepp1	selenoprotein P, plasma, 1	-5.089537	6.62E-05	0.009185
1381543_at	291840	LOC29184	amino acid transporter	2.918118	6.64E-05	0.009185
1369788_s_at	24516	Jun	Jun oncogene	-3.415958	6.66E-05	0.009185
1383316_at	361462	Hddc2_pre	HD domain containing 2 (predicted)	4.174605	6.71E-05	0.009185
1371368_at	80843	Sec61a1	Sec61 alpha 1 subunit (S. cerevisiae)	2.47011	6.74E-05	0.009185
1367958_at	79249	Abi1	abl-interactor 1	2.372723	6.74E-05	0.009185
1367557_s_at	24383	Gapdh	glyceraldehyde-3-phosphate dehydrog	3.659867	6.77E-05	0.009185
1367925_at	64681	Mvp	major vault protein	3.600841	6.78E-05	0.009185
1375212_at	362811	Ankrd52_p	ankyrin repeat domain 52 (predicted)	2.535131	6.84E-05	0.009185
1369516_at	29535	Pdx1	pancreatic and duodenal homeobox ge	2.835061	6.86E-05	0.009185
1396238_at	313878	Galnt14	UDP-N-acetyl-alpha-D-galactosamine:	-2.971763	6.94E-05	0.009185
1370237_at	113965	Hadhsc	L-3-hydroxyacyl-Coenzyme A dehydro	3.741736	6.97E-05	0.009185
1375545_at	362950	Rbm9_pre	RNA binding motif protein 9 (predicted	2.825749	7.10E-05	0.009185
1376042_at	363460	Fgd1	FYVE, RhoGEF and PH domain conta	-2.411642	7.17E-05	0.009185
1371646_at	362660	Pgd_mapp	phosphogluconate dehydrogenase (ma	2.317477	7.20E-05	0.009185
1372123_at	298596	Sdhb_pred	succinate dehydrogenase complex, su	2.655002	7.30E-05	0.009185
1387013_at	57395	Tmem27	transmembrane protein 27	-2.81745	7.39E-05	0.009185
1374741_at	293701	Esrra	estrogen related receptor, alpha	3.37809	7.43E-05	0.009185
1370815_at	24587	Nefh	neurofilament, heavy polypeptide	2.571643	7.49E-05	0.009185
1375119_at	25489	Nedd4a	neural precursor cell expressed, devel	2.459534	7.55E-05	0.009185
1387803_at	60660	Ppp2r2b	protein phosphatase 2 (formerly 2A), r	-3.361118	7.55E-05	0.009185
1384834_at	305497	Cobl_predi	cordon-bleu (predicted)	-5.214809	7.62E-05	0.009185
1368379_at	117106	Scarb2	scavenger receptor class B, member 2	2.759405	7.63E-05	0.009185
1367991_at	78947	Gcs1	glucosidase 1	2.364608	7.64E-05	0.009185
1379469_at	302711	Tbl1x_pred	transducin (beta)-like 1 X-linked (predi	2.493454	7.70E-05	0.009185
1387791_at	24310	Ace	angiotensin I converting enzyme (pept	-3.371879	7.71E-05	0.009185
1372942_at	308441	Exosc5_pr	exosome component 5 (predicted)	2.589409	7.74E-05	0.009185
1368589_at	29645	Ptprj	protein tyrosine phosphatase, receptor	3.069566	7.74E-05	0.009185
1376073_at	314352	Sel1h	Sel1 (suppressor of lin-12) 1 homolog	3.041348	7.75E-05	0.009185
1367653_a_at	24551	Mdh1	malate dehydrogenase 1, NAD (solubl	2.368819	7.85E-05	0.009185
1397233_at	316023	Dcamkl3_p	doublecortin and CaM kinase-like 3 (pr	-3.283126	7.85E-05	0.009185
1395318_at	680231	LOC68023	NA	2.699444	7.90E-05	0.009185
1374249_at	292781	RGD13045	similar to Hypothetical protein MGC38	2.274162	7.91E-05	0.009185
1388574_at	314442	Wars	tryptophanyl-tRNA synthetase	2.361804	8.22E-05	0.009185
1374224_at	114859	Eif2ak4_pr	eukaryotic translation initiation factor 2	-2.75873	8.59E-05	0.009185
1367559_at	29292	Ftl1	ferritin light chain 1	2.672479	8.66E-05	0.009185
1392477_at	362733	Etv1_predi	ets variant gene 1 (predicted)	-3.458786	8.71E-05	0.009185
1368104_at	64521	Tspan2	tetraspanin 2	-2.307033	8.71E-05	0.009185

1368778_at	29464	Slc6a6	solute carrier family 6 (neurotransmitte	2.874698	8.73E-05	0.009185
1388817_at	310665	LOC31066	hypothetical protein LOC310665	3.093446	8.77E-05	0.009185
1377791_at	117043	RragB	Ras-related GTP binding B	-2.632717	8.81E-05	0.009185
1372156_at	303330	Tmem97	transmembrane protein 97	2.759117	8.84E-05	0.009185
1369412_a_at	29723	Slc19a1	solute carrier family 19, member 1	4.298902	8.88E-05	0.009185
1398277_at	79558	Acvr1	activin A receptor, type 1	3.674856	8.88E-05	0.009185
1393459_at	24948	Fmr1	fragile X mental retardation syndrome	-2.709657	8.90E-05	0.009185
1389973_a_at	619346	Surf4	surfeit 4	3.097459	8.99E-05	0.009185
1387907_at	25262	Itpr1	inositol 1,4,5-triphosphate receptor 1	-2.805118	9.02E-05	0.009185
1383697_at	114507	Slc5a3	solute carrier family 5 (inositol transpor	-3.030721	9.08E-05	0.009185
1374784_at	291355	Prtfdc1_pre	phosphoribosyl transferase domain co	-2.827336	9.10E-05	0.009185
1367617_at	24189	Aldoa	aldolase A	2.68536	9.18E-05	0.009185
1378128_at	313993	Grhl1_prec	grainyhead-like 1 (Drosophila) (predict	2.484382	9.19E-05	0.009185
1389367_at	295105	Schip1	schwannomin interacting protein 1	-2.217554	9.38E-05	0.009185
1370088_at	85244	Spa17	sperm autoantigenic protein 17	-2.46392	9.39E-05	0.009185
1382325_at	366959	Gcat	glycine C-acetyltransferase (2-amino-3	3.501567	9.39E-05	0.009185
1383768_at	286973	Elavl2	ELAV (embryonic lethal, abnormal visi	2.798385	9.40E-05	0.009185
1386762_at	310097	Snag1_pre	sorting nexin associated golgi protein 1	-2.725184	9.44E-05	0.009185
1371311_at	289217	Sdhc	succinate dehydrogenase complex, su	2.591326	9.59E-05	0.00921
1367969_at	94167	Prdx6	peroxiredoxin 6	2.290887	9.65E-05	0.00921
1382434_at	314312	Entpd5	ectonucleoside triphosphate diphosph	3.224723	9.73E-05	0.00921
1392957_at	361877	RGD15629	similar to Single-stranded DNA-binding	-2.277343	9.75E-05	0.00921
1368146_at	114856	Dusp1	dual specificity phosphatase 1	-3.344503	9.85E-05	0.00921
1395644_at	303637	Abca8b_pr	ATP-binding cassette, sub-family A (AI	-2.952363	9.88E-05	0.00921
1367575_at	24333	Eno1	enolase 1, alpha	3.37021	0.000104	0.009543
1393048_at	25083	Adra2a	adrenergic receptor, alpha 2a	3.299436	0.000104	0.009543
1390699_at	309307	RGD13115	similar to KIAA2026 protein	-2.480339	0.000105	0.009543
1373152_at	308807	Prss23	protease, serine, 23	-3.320517	0.000107	0.009567
1387914_at	301517	Cyp27a1	cytochrome P450, family 27, subfamily	-2.846976	0.000107	0.009567
1371832_at	300837	Leo1	Leo1, Paf1/RNA polymerase II comple	-2.345423	0.000108	0.009567
1389815_at	259225	Ppp1r14b	protein phosphatase 1, regulatory (inhi	2.317303	0.000109	0.009567
1398626_s_at	289820	Actr2	ARP2 actin-related protein 2 homolog	2.293269	0.000109	0.009567
1376762_at	361659	RGD15641	similar to Pleckstrin homology domain-	-2.768517	0.000109	0.009567
1395533_at	307492	Dnd1	dead end homolog 1 (zebrafish)	2.269347	0.000111	0.009567
1372516_at	293502	Kif22	kinesin family member 22	3.358113	0.000111	0.009567
1388006_at	207126	Muc13	mucin 13, epithelial transmembrane	2.739591	0.000112	0.009567
1370522_at	24953	Gcgr	glucagon receptor	4.567025	0.000112	0.009567
1375267_at	291463	Ppic	peptidylprolyl isomerase C	-2.500239	0.000113	0.009611
1389308_at	360734	Dnajb11	DnaJ (Hsp40) homolog, subfamily B, n	2.494854	0.000115	0.009623
1369654_at	78975	Prkaa2	protein kinase, AMP-activated, alpha 2	3.841862	0.000115	0.009623
1373577_at	246331	Nrp1	neuropilin 1	-3.895078	0.000117	0.009623
1382291_at	363698	NA	NA	-2.85463	0.000117	0.009623
1367677_at	113898	Prdx5	peroxiredoxin 5	2.165365	0.000117	0.009623
1375697_at	304543	RGD13077	similar to Hypothetical protein KIAA015	4.441796	0.000118	0.009633
1390779_at	361661	RGD15643	similar to phosphoseryl-tRNA kinase (p	-2.295716	0.00012	0.009633
1375532_at	25587	Id2	inhibitor of DNA binding 2	-2.859442	0.000122	0.009633
1382902_at	362376	Herc6	potential ubiquitin ligase	-2.772031	0.000122	0.009633
1383146_at	361948	RGD15626	similar to neurobeachin (predicted)	-2.95375	0.000123	0.009633
1393780_at	310375	Zfp131	zinc finger protein 131	-3.064478	0.000123	0.009633
1384320_at	301309	Bai3_predi	brain-specific angiogenesis inhibitor 3	-3.590616	0.000123	0.009633
1371546_at	361128	LOC36112	similar to TR4 orphan receptor associa	2.983206	0.000124	0.009633

1398796_at	84599	Tmed10	transmembrane emp24-like trafficking	2.307906	0.000124	0.009633
1383455_at	289352	Eprs	glutamyl-prolyl-tRNA synthetase	2.61713	0.000124	0.009633
1382843_at	286896	Sgpl1	sphingosine phosphate lyase 1	2.505632	0.000126	0.009723
1375699_at	619382	Centb2	centaurin, beta 2	2.264553	0.000127	0.009731
1371366_at	360678	Arhgdia	Rho GDP dissociation inhibitor (GDI) a	2.142978	0.000132	0.009883
1382045_at	366896	Tbc1d15	TBC1 domain family, member 15	-2.152296	0.000132	0.009883
1389368_at	308113	Cnksr3	Cnksr family member 3	-3.17689	0.000132	0.009883
1367589_at	79250	Aco2	aconitase 2, mitochondrial	2.120697	0.000133	0.009883
1367856_at	24377	G6pdx	glucose-6-phosphate dehydrogenase X	2.230293	0.000133	0.009883
1367515_at	306492	Cnot7_pre	CCR4-NOT transcription complex, sub	-2.199845	0.000134	0.009883
1379603_at	500985	RGD15613	similar to CBL E3 ubiquitin protein liga	2.069281	0.000136	0.009898
1368512_a_at	64017	Enpep	glutamyl aminopeptidase	-3.921773	0.000136	0.009898
1371445_at	287633	Lrrc59	leucine rich repeat containing 59	2.03308	0.000139	0.009898
1370775_a_at	24241	Calca	calcitonin/calcitonin-related polypeptid	-5.458799	0.000141	0.009898
1374204_at	303336	Wsb1	WD repeat and SOCS box-containing	-3.226958	0.000142	0.009898
1376315_at	296304	RGD1303C	similar to putative alpha-mannosidase	2.193012	0.000142	0.009898
1368700_at	84587	Plcl1	phospholipase C-like 1	-4.798775	0.000143	0.009898
1383587_at	300783	RGD15654	similar to Butyrate-induced transcript 1	2.947765	0.000144	0.009898
1381118_at	311279	RGD13114	similar to RIKEN cDNA 2700007P21	2.159673	0.000145	0.009898
1368839_at	83725	Wfs1	Wolfram syndrome 1 homolog (human	2.064608	0.000145	0.009898
1370098_at	85491	Sybl1	synaptobrevin-like 1	2.026723	0.000146	0.009898
1388121_at	64312	Aplp2	amyloid beta (A4) precursor-like protei	-2.357707	0.000146	0.009898
1376165_at	85267	Slc24a3	solute carrier family 24 (sodium/potass	-3.25963	0.000146	0.009898
1380982_at	246755	Bcas1	breast carcinoma amplified sequence	-2.99389	0.000149	0.009898
1369958_at	64373	Rhob	ras homolog gene family, member B	-3.320848	0.00015	0.009898
1368539_at	78956	Scn9a	sodium channel, voltage-gated, type IX	-4.290797	0.00015	0.009898
1369462_at	24380	Gad2	glutamic acid decarboxylase 2	5.759655	0.000151	0.009898
1377708_at	499339	LOC49933	hypothetical protein LOC499339	2.495529	0.000151	0.009898
1393386_at	287346	RGD15619	similar to novel protein (predicted)	-3.692665	0.000151	0.009898
1374602_at	29544	Tspyl	testis-specific protein, Y-encoded-like	-1.966414	0.000152	0.009898
1373905_at	319110	Hnrpr	heterogeneous nuclear ribonucleoprot	-1.938925	0.000154	0.009898
1382384_at	305251	MGC11441	similar to Ras association (RalGDS/AF	2.079559	0.000155	0.009898
1387407_at	170914	Nap1l3	nucleosome assembly protein 1-like 3	-2.333538	0.000156	0.009898
1373414_at	306349	RGD13055	similar to Brain specific membrane-an	-2.073604	0.000156	0.009898
1387828_at	65218	Centg1	centaurin, gamma 1	-2.163471	0.000156	0.009898
1383110_at	303803	Klhl24	kelch-like 24 (Drosophila)	-3.46836	0.000156	0.009898
1383617_at	306455	RGD13053	similar to RIKEN cDNA 4921511I16	-3.374083	0.000157	0.009898
1370411_at	89821	Trpc1	transient receptor potential cation char	-1.977841	0.000157	0.009898
1374938_at	363059	Zw10	ZW10 homolog, centromere/kinetocho	2.028926	0.000158	0.009898
1371993_at	313087	Cpne3_pre	copine III (predicted)	-2.077427	0.000158	0.009898
1372674_at	312603	Rybp_pred	RING1 and YY1 binding protein (predi	-1.962916	0.000159	0.009898
1382855_at	311715	RGD13058	similar to Protein C20orf158 (predicted	4.716846	0.000159	0.009898
1367671_at	25737	Pcna	proliferating cell nuclear antigen	-1.941059	0.00016	0.009898
1386930_at	83499	Psmd4	proteasome (prosome, macropain) 26S	2.051693	0.00016	0.009898
1386773_at	306262	Btd	biotinidase	2.379098	0.00016	0.009898
1373372_at	501282	LOC50128	NA	2.014807	0.000162	0.009924
1388693_at	300142	RGD13108	similar to RIKEN cDNA 5730502D15 g	2.669867	0.000162	0.009924
1387818_at	114555	Casp4	caspase 4, apoptosis-related cysteine	3.125757	0.000163	0.009924
1392910_at	300050	Bop1	block of proliferation 1	2.103412	0.000164	0.009924
1367884_at	94197	Rab14	RAB14, member RAS oncogene family	2.387369	0.000165	0.009924
1372662_at	305830	Fbxo34_pr	F-box only protein 34 (predicted)	-3.043204	0.000165	0.009924

1392463_at	317508	RGD15648	similar to hypothetical protein FLJ1450	-2.241226	0.000166	0.00993
1390255_at	307395	RGD15651	similar to mKIAA0843 protein (predicte	-1.99324	0.000167	0.00995
1387891_at	85274	Prdx4	peroxiredoxin 4	-2.299308	0.000168	0.00995
1388708_at	364531	NA	NA	2.193115	0.000169	0.00995
1383396_at	306022	Fndc3a_pr	fibronectin type III domain containing 3	-1.897489	0.00017	0.00995
1371631_at	293673	RGD13046	similar to 2010003J03Rik protein	2.139859	0.000171	0.00995
1372296_at	498066	RGD15635	similar to putative SH3BGR protein (pr	-2.460383	0.000171	0.00995
1388557_at	117517	C7	complement component 7	-8.183999	0.000172	0.00995
1393142_at	367153	LOC36715	similar to p10-binding protein	-2.735562	0.000173	0.00995
1368536_at	84050	Enpp2	ectonucleotide pyrophosphatase/phos	-4.10004	0.000174	0.00995
1388169_at	171120	Jmjd1c	jumonji domain containing 1C	-2.843482	0.000175	0.00995
1375861_at	362377	Herc3_pre	hect domain and RLD 3 (predicted)	-2.8995	0.000175	0.00995
1376587_at	301674	Fbxo11	F-box only protein 11	-1.905881	0.000175	0.00995
1388353_at	288778	Pa2g4	proliferation-associated 2G4	2.186344	0.000177	0.00997
1392640_at	299691	Cry1	cryptochrome 1 (photolyase-like)	-1.934404	0.000177	0.00997
1395399_at	500555	RGD15652	similar to PS1D protein (predicted)	2.956046	0.000178	0.009982
1390232_at	309145	RGD13119	similar to RIKEN cDNA 1810055G02	1.881214	0.000179	0.009982
1374884_at	287585	Ppm1d_pre	protein phosphatase 1D magnesium-d	-2.531864	0.000179	0.009984
1371756_at	369017	Krt2-5	keratin complex 2, basic, gene 5	2.210704	0.000182	0.009985
1397525_at	303193	Alkbh5_pre	alkB, alkylation repair homolog 5 (E. co	2.298438	0.000182	0.009985
1378324_at	499814	LOC49981	NA	-3.798363	0.000183	0.009985
1369934_at	64367	Ppib	peptidylprolyl isomerase B	2.290491	0.000183	0.009985
1379765_at	363942	RGD15614	similar to nemo like kinase (predicted)	2.879849	0.000183	0.009985
1367759_at	24437	H1f0	H1 histone family, member 0	2.860922	0.000186	0.010028
1367967_at	114200	Lepre1	leprecan 1	2.217476	0.000187	0.010028
1388280_a_at	58948	Dlgh3	discs, large homolog 3 (Drosophila)	3.876005	0.000188	0.010028
1372900_at	296360	Pigt_predic	phosphatidylinositol glycan, class T (pr	2.396983	0.000188	0.010028
1371626_at	363707	Srp68_pre	signal recognition particle 68 (predicte	1.935963	0.000189	0.010028
1374617_at	287380	Dhrs7b	dehydrogenase/reductase (SDR family	1.95358	0.00019	0.010028
1389679_at	500026	RGD15636	similar to testhymin (predicted)	-2.440548	0.00019	0.010028
1373433_at	365273	Nsbp1_pre	nucleosome binding protein 1 (predicte	-1.963338	0.00019	0.010028
1370094_at	60353	Acrv1	acrosomal vesicle protein 1	3.58239	0.000192	0.010029
1372088_at	690784	LOC69078	NA	-1.992194	0.000193	0.010029
1370409_at	170567	Slc38a1	solute carrier family 38, member 1	2.568233	0.000194	0.010029
1389451_at	362942	RGD15604	similar to G protein-coupled receptor 1	2.151939	0.000195	0.010029
1368106_at	83722	Plk2	polo-like kinase 2 (Drosophila)	-2.897219	0.000196	0.010029
1389550_at	116743	Sh3gl2	SH3-domain GRB2-like 2	-2.264326	0.000196	0.010029
1390307_at	498430	RGD15654	similar to echinoderm microtubule asso	3.618788	0.000198	0.010029
1372352_at	315989	Armet_pre	arginine-rich, mutated in early stage tu	2.489699	0.0002	0.010029
1367465_at	192275	Dad1	defender against cell death 1	1.875514	0.000203	0.010029
1370828_at	246326	Zdhhc2	zinc finger, DHHC domain containing 2	-1.851135	0.000203	0.010029
1367927_at	25344	Phb	prohibitin	2.111106	0.000206	0.010029
1384910_at	312668	RGD15620	similar to putative voltage-gated calciu	2.872173	0.000206	0.010029
1377670_at	361605	Pcf11_pre	cleavage and polyadenylation factor su	-3.069754	0.000207	0.010029
1376654_at	310358	RGD13084	similar to RIKEN cDNA B130016O10 g	2.852998	0.000208	0.010029
1392905_at	80850	Gng2	guanine nucleotide binding protein, ga	3.626475	0.000208	0.010029
1398866_at	245903	Magi3	membrane associated guanylate kinas	-2.491487	0.00021	0.010029
1387854_at	84352	Col1a2	procollagen, type I, alpha 2	-5.678517	0.00021	0.010029
1388369_at	361207	Tmed9	transmembrane emp24 protein transpo	1.895874	0.000211	0.010029
1376175_at	498174	LOC49817	similar to NipSnap2 protein (Glioblasto	3.617274	0.000212	0.010029
1386940_at	29543	Timp2	tissue inhibitor of metalloproteinase 2	-2.449993	0.000214	0.010029

1367845_at	24588	Nef3	neurofilament 3, medium	2.632546	0.000215	0.010029
1369886_a_at	171051	Cabp1	calcium binding protein 1	3.465435	0.000216	0.010029
1390592_at	688018	LOC68801	NA	2.939131	0.000216	0.010029
1398219_at	500440	RGD15625	similar to RIKEN cDNA A930001M12 g	3.175627	0.000216	0.010029
1369871_at	29183	Areg	amphiregulin	-6.702033	0.000216	0.010029
1398776_at	64701	Rpn2	ribophorin II	2.177597	0.000218	0.010029
1373177_x_at	287866	NA	NA	-2.560656	0.000218	0.010029
1391475_at	313842	Hnrpll_pred	heterogeneous nuclear ribonucleoprote	-2.393145	0.000218	0.010029
1367766_at	83782	Nme2	expressed in non-metastatic cells 2	1.780668	0.000219	0.010029
1388791_at	316426	RGD13099	similar to 2810022L02Rik protein	-2.92845	0.00022	0.010029
1384428_at	502228	RGD15664	similar to OTTHUMP00000040081 (pr	2.608268	0.00022	0.010029
1396822_at	308126	Zbtb2_pred	zinc finger and BTB domain containing	1.96066	0.00022	0.010029
1375224_at	363989	Phlda3	pleckstrin homology-like domain, family	3.196033	0.000223	0.010029
1370942_at	29372	Rasa3	RAS p21 protein activator 3	-3.244846	0.000223	0.010029
1375043_at	314322	Fos	FBJ murine osteosarcoma viral oncoge	-3.968839	0.000225	0.010029
1388322_at	287986	Eif4g1	eukaryotic translation initiation factor 4	2.533545	0.000226	0.010029
1371976_at	497875	NA	NA	2.306071	0.000227	0.010029
1371329_at	287444	Eif5a	eukaryotic translation initiation factor 5	1.93521	0.000228	0.010029
1371514_at	361577	Kdelr1	KDEL (Lys-Asp-Glu-Leu) endoplasmic	1.801674	0.000229	0.010029
1387086_at	81715	Camlg	calcium modulating ligand	3.389522	0.000231	0.010029
1388856_at	60427	Kitl	kit ligand	-2.922613	0.000232	0.010029
1376419_at	300231	RGD13056	similar to expressed sequence AI3172	2.412446	0.000232	0.010029
1388500_at	54260	Itpkb	inositol 1,4,5-trisphosphate 3-kinase B	3.39607	0.000233	0.010029
1388703_at	300519	Esam	endothelial cell adhesion molecule	2.109691	0.000233	0.010029
1376567_at	304323	Ftsj2_predi	FtsJ homolog 2 (E. coli) (predicted)	-2.256902	0.000233	0.010029
1372119_at	300968	Ube1dc1	ubiquitin-activating enzyme E1-domain	1.842655	0.000233	0.010029
1373650_at	312826	Cmas	cytidine monophospho-N-acetylneuram	1.838073	0.000235	0.010029
1390219_at	305349	Wdr19_pre	WD repeat domain 19 (predicted)	-1.975412	0.000235	0.010029
1368523_at	26989	Cadps	Ca2+-dependent secretion activator	-2.333254	0.000236	0.010029
1377051_at	360463	Mpv17l_pre	Mpv17 transgene, kidney disease muta	2.069422	0.000237	0.010029
1389557_at	297392	Tex261	testis expressed gene 261	2.028679	0.000237	0.010029
1377417_at	363328	LOC36332	NA	1.852405	0.000237	0.010029
1373087_at	311059	7-Mar	membrane-associated ring finger (C3H	-1.906689	0.000237	0.010029
1388413_at	311483	Rrbp1_pred	ribosome binding protein 1 homolog 18	2.000471	0.000237	0.010029
1374712_at	303353	Psmd11_p	proteasome (prosome, macropain) 26S	1.895198	0.000238	0.010029
1389534_at	295686	Ube2e3_pr	ubiquitin-conjugating enzyme E2E 3, U	-2.530228	0.000239	0.010029
1371526_at	288710	Rnf10	ring finger protein 10	1.808438	0.00024	0.010029
1377599_at	313977	Lpin1	lipin 1	-2.336165	0.00024	0.010029
1369013_a_at	171061	Mrpl17	mitochondrial ribosomal protein L17	1.871451	0.000241	0.010029
1378606_at	291694	RGD13104	similar to hypothetical protein MGC370	-2.747574	0.000241	0.010029
1372297_at	300850	Gsta4	glutathione S-transferase, alpha 4	-2.018058	0.000243	0.010029
1391013_at	64865	Pcdh8	protocadherin 8	-3.066667	0.000244	0.010029
1370189_at	117259	Sfrs10	splicing factor, arginine/serine-rich 10 (	-1.926839	0.000244	0.010029
1387340_at	140945	Rtn3	reticulon 3	1.827413	0.000244	0.010029
1371342_at	300047	Cyc1_pred	cytochrome c-1 (predicted)	1.910923	0.000244	0.010029
1389940_at	116455	Atp6v0a2_	ATPase, H+ transporting, lysosomal V	2.002875	0.000247	0.010029
1373280_at	292907	Ruvbl2	RuvB-like 2 (E. coli)	1.836061	0.000248	0.010029
1388335_at	304983	Tagln2	transgelin 2	2.082029	0.000248	0.010029
1375852_at	25675	Hmgcr	3-hydroxy-3-methylglutaryl-Coenzyme	-2.337354	0.000248	0.010029
1376739_at	373065	Ddx24	DEAD (Asp-Glu-Ala-Asp) box polypept	2.288083	0.000249	0.010029
1370810_at	64033	Ccnd2	cyclin D2	2.978188	0.000249	0.010029

1374048_at	84423	Nrtn	neurturin	2.918784	0.00025	0.010029
1371246_at	291981	NTF2	nuclear transport factor 2	1.883547	0.000251	0.010029
1398835_at	81822	Actb	actin, beta	2.221559	0.000253	0.010029
1373090_at	361233	Ssr1	signal sequence receptor, alpha	2.098819	0.000253	0.010029
1370035_at	24525	Kras	v-Ki-ras2 Kirsten rat sarcoma viral onc	1.866454	0.000254	0.010029
1371564_at	297566	Atp6v1e1	ATPase, H+ transporting, V1 subunit E	1.74429	0.000255	0.010029
1395416_at	296372	Ncoa5_pre	nuclear receptor coactivator 5 (predicte	-3.44847	0.000255	0.010029
1371889_at	305886	Slc22a17	solute carrier family 22 (organic cation	-3.027399	0.000256	0.010029
1367455_at	116643	Vcp	valosin-containing protein	2.19588	0.000256	0.010029
1383615_a_at	362736	RGD1561€	similar to HECT domain containing 1 ((	2.002229	0.000257	0.010029
1376805_at	304850	Rnf2	ring finger protein 2	-2.053413	0.000257	0.010029
1367482_at	498030	RGD15618	similar to anaphase promoting comple:	1.94047	0.000258	0.010029
1386863_at	24668	Ppp1ca	protein phosphatase 1, catalytic subun	1.716409	0.000259	0.010029
1386925_at	54227	Arpc1b	actin related protein 2/3 complex, subu	2.416437	0.00026	0.010029
1384866_at	316077	Entpd3	ectonucleoside triphosphate diphosph	1.907111	0.00026	0.010029
1386923_at	81816	Ube2b	ubiquitin-conjugating enzyme E2B, RA	-2.033326	0.00026	0.010029
1388455_at	114119	Gng10	guanine nucleotide binding protein (G	-1.852713	0.000261	0.010029
1397600_at	299738	Vezt	vezatin, adherens junctions transmeml	1.969468	0.000261	0.010029
1378864_at	312707	Lrrc23	leucine rich repeat containing 23	-2.307951	0.000261	0.010029
1388223_at	286924	Gnat3	guanine nucleotide binding protein, alp	-2.930913	0.000261	0.010029
1368848_at	116666	Lman1	lectin, mannose-binding, 1	2.423002	0.000263	0.010029
1388705_at	498398	RGD1565C	similar to selenoprotein SelM (predicte	2.049576	0.000263	0.010029
1389597_at	292098	Pgbd5_pre	piggyBac transposable element derive	-2.430835	0.000263	0.010029
1397842_at	84402	Sfrp1	secreted frizzled-related protein 1	-3.258835	0.000265	0.010029
1372461_at	307947	Set_predic	SET translocation (predicted)	1.929239	0.000266	0.010029
1370997_at	29546	Homer1	homer homolog 1 (Drosophila)	-5.00067	0.000266	0.010029
1368170_at	79212	Slc6a1	solute carrier family 6 (neurotransmitte	-3.841076	0.000267	0.010029
1389391_at	362883	RGD15648	similar to Solute carrier family 35, mem	1.817991	0.000268	0.010029
1378739_at	500292	LOC50029	similar to cell death-inducing DFFA-like	5.586426	0.000268	0.010029
1390804_at	499874	RGD15615	similar to mKIAA0518 protein (predicte	-1.958767	0.000268	0.010029
1369067_at	58853	Nr4a3	nuclear receptor subfamily 4, group A,	-3.933475	0.000268	0.010029
1387034_at	24616	Pah	phenylalanine hydroxylase	-3.296149	0.000269	0.010029
1393915_at	362434	Oact5	O-acyltransferase (membrane bound)	1.997306	0.000269	0.010029
1389562_at	291423	Setbp1_pre	SET binding protein 1 (predicted)	-1.926863	0.000272	0.01008
1382813_at	306549	RGD13108	similar to RIKEN cDNA 4930444A02	1.868612	0.000272	0.01008
1379360_at	360821	RGD13096	similar to Putative protein 15E1.2 (pre	2.530112	0.000273	0.01008
1398907_at	288783	Ormdl2_pr	ORM1-like 2 (S. cerevisiae) (predicted	1.966844	0.000273	0.01008
1371041_at	81728	Ndufv2	NADH dehydrogenase (ubiquinone) fla	2.167213	0.000275	0.010088
1369970_at	83730	Vamp8	vesicle-associated membrane protein 8	2.193624	0.000275	0.010088
1368886_at	25579	Map3k12	mitogen activated protein kinase kinas	1.727627	0.000277	0.010088
1371584_at	362247	Trpc4ap	transient receptor potential cation char	1.864209	0.000278	0.010088
1384290_at	308968	Rbbp6	retinoblastoma binding protein 6	-1.984528	0.000278	0.010088
1387870_at	79426	Zfp36	zinc finger protein 36	-2.838873	0.00028	0.010088
1397173_at	317420	RGD15631	similar to Serine/threonine-protein kina	-4.207665	0.000281	0.010088
1386721_at	305687	Zfp503_pre	zinc finger protein 503 (predicted)	-2.351756	0.000282	0.010088
1390506_at	497991	RGD15595	similar to peroxisome proliferator-activ	1.812601	0.000284	0.010088
1375968_at	500214	RGD15596	similar to Catna2 protein (predicted)	-2.089	0.000285	0.010088
1388092_at	363640	Olr1493	olfactory receptor gene Olr1493	1.849016	0.000286	0.010088
1390820_at	313481	Zcchc11_p	zinc finger, CCHC domain containing 1	-2.490441	0.000286	0.010088
1387759_s_at	113992	Ugt1a6	UDP glycosyltransferase 1 family, poly	1.750495	0.000286	0.010088
1390881_at	286965	Abra	actin-binding Rho activating protein	2.772438	0.000287	0.010088

1391459_at	367747	RGD15647	similar to nudix (nucleoside diphospha	-1.791328	0.000288	0.010088
1379626_at	316164	Satb1	special AT-rich sequence binding prote	-3.798765	0.000288	0.010088
1370229_at	64457	Ndrg4	N-myc downstream regulated gene 4	2.473789	0.000288	0.010088
1398355_at	114497	Trpm7	transient receptor potential-related pro	-2.187488	0.000289	0.010088
1377995_at	360502	RGD13114	similar to hypothetical protein DKFZp7	2.156025	0.000291	0.010088
1377189_at	292680	RGD13073	similar to BC282485_1 (predicted)	2.830574	0.000293	0.010088
1378394_at	361344	Mppe1_pre	metallophosphoesterase 1 (predicted)	-1.87238	0.000293	0.010088
1368544_a_at	85383	Nol3	nucleolar protein 3 (apoptosis represso	2.012729	0.000294	0.010088
1398859_at	64474	Hdlbp	high density lipoprotein binding protein	2.343636	0.000294	0.010088
1388347_at	362934	LOC36293	similar to lymphocyte antigen 6 comple	-2.045978	0.000294	0.010088
1389168_at	311456	Mkks	McKusick-Kaufman syndrome protein	1.963074	0.000296	0.010088
1387663_at	81661	Gmfb	glia maturation factor, beta	2.061684	0.000296	0.010088
1372520_at	60430	Mcl1	myeloid cell leukemia sequence 1	-2.816301	0.000296	0.010088
1369735_at	58935	Gas6	growth arrest specific 6	2.17123	0.000296	0.010088
1367584_at	56611	Anxa2	annexin A2	1.762913	0.000299	0.010088
1371014_at	24654	Plcb1	phospholipase C, beta 1	-2.402395	0.0003	0.010088
1393352_at	300836	RGD13105	similar to hypothetical protein MGC389	-2.055838	0.000303	0.010088
1380682_at	308790	Rkhd3_pre	ring finger and KH domain containing 3	-2.478368	0.000303	0.010088
1398772_at	83809	Nsfl1c	NSFL1 (p97) cofactor (p47)	2.507878	0.000304	0.010088
1385088_at	304650	RGD13102	hypothetical LOC304650 (predicted)	2.782458	0.000305	0.010088
1367508_at	497874	RGD15657	RGD1565784 (predicted)	1.764936	0.000305	0.010088
1369538_at	116671	Cdk5r1	cyclin-dependent kinase 5, regulatory s	3.903788	0.000307	0.010088
1375135_at	304538	Gcn1l1_pre	GCN1 general control of amino-acid sy	3.168722	0.000307	0.010088
1373015_at	114559	Arhgef7	Rho guanine nucleotide exchange fact	-1.730615	0.000307	0.010088
1372051_at	360793	Rhbdl7_pre	rhomboid, veinlet-like 7 (Drosophila) (p	1.901081	0.000309	0.010088
1374086_at	307178	Arhgap21_	Rho GTPase activating protein 21 (pre	-1.753781	0.00031	0.010088
1399101_at	362251	Rnpc2	RNA-binding region (RNP1, RRM) con	-1.718241	0.000311	0.010088
1370381_at	286988	Pnrc1	proline rich 2	-1.76546	0.000311	0.010088
1369896_s_at	245926	Rbm16	RNA binding motif protein 16	-2.210717	0.000311	0.010088
1399152_at	313474	Eps15	epidermal growth factor receptor pathw	2.508964	0.000312	0.010088
1388973_at	305104	Col9a1	procollagen, type IX, alpha 1	-2.572737	0.000312	0.010088
1368050_at	114121	Ccnl1	cyclin L1	-2.728992	0.000313	0.010088
1391437_at	313771	MGC94339	similar to BC002216 protein	2.179672	0.000313	0.010088
1396830_at	499979	RGD15621	RGD1562135 (predicted)	1.954569	0.000314	0.010088
1373420_at	300447	MGC94704	evolutionarily conserved signaling inter	1.861099	0.000314	0.010088
1370055_at	140665	Rab3d	RAB3D, member RAS oncogene famil	1.702746	0.000314	0.010088
1369018_at	58921	Foxm1	forkhead box M1	-2.453849	0.000316	0.010088
1377623_at	299613	Rkhd1_pre	ring finger (C3HC4 type) and KH doma	-2.056426	0.000318	0.010088
1382847_at	310638	Ash1l_prec	ash1 (absent, small, or homeotic)-like	2.062524	0.000319	0.010088
1372259_at	306817	Dek	DEK oncogene (DNA binding)	-1.84509	0.000319	0.010088
1382276_at	304580	Coro1c_pre	coronin, actin binding protein 1C (pred	1.834805	0.000319	0.010088
1377014_at	310376	RGD13081	similar to hypothetical protein MGC421	-6.615158	0.000322	0.010088
1388799_at	362303	Klhl7	kelch-like 7 (Drosophila)	-2.466527	0.000322	0.010088
1387229_at	83624	Ppig	peptidylprolyl isomerase G	-2.532552	0.000322	0.010088
1371647_at	309475	RGD15646	similar to transmembrane protein TM9	1.79816	0.000323	0.010088
1383056_a_at	499072	RGD15652	RGD1565210 (predicted)	2.734953	0.000323	0.010088
1372193_at	497198	Impact	NA	-2.520371	0.000326	0.010088
1371361_at	29136	Tns	tensin	4.213073	0.000326	0.010088
1374305_at	290215	Ap1g2_pre	adaptor protein complex AP-1, gamma	2.168946	0.000326	0.010088
1388576_at	288516	Eif3s9	eukaryotic translation initiation factor 3	1.874797	0.000327	0.010088
1384056_at	84489	Fgfr3	fibroblast growth factor receptor 3	-2.638573	0.000328	0.010088

1392273_at	361169	Letm2	leucine zipper-EF-hand containing trar	-1.988256	0.000328	0.010088
1375686_at	301432	Ppil3	peptidylprolyl isomerase (cyclophilin)-li	1.856142	0.000329	0.010088
1392512_at	64646	H2a	histone 2a	-1.751189	0.00033	0.010088
1387036_at	29577	Hes1	hairy and enhancer of split 1 (Drosoph	-2.480933	0.000331	0.010088
1370191_at	58961	Azin1	antizyme inhibitor 1	-1.724918	0.000332	0.010088
1367869_at	117520	Oxr1	oxidation resistance 1	-2.102015	0.000332	0.010088
1376451_at	362317	LOC36231	NA	-1.775559	0.000334	0.010088
1367798_at	29443	Ahcy	S-adenosylhomocysteine hydrolase	1.856767	0.000334	0.010088
1370278_at	245965	Atp5d	ATP synthase, H+ transporting, mitoch	2.087169	0.000336	0.010088
1392180_at	24790	Sp1	Sp1 transcription factor	1.836063	0.000336	0.010088
1383339_at	308537	RGD1310З	similar to NNX3 (predicted)	1.765217	0.000337	0.010088
1383391_a_at	24231	C2	complement component 2	-2.039	0.000338	0.010088
1383283_at	317576	Ocrl_mapp	oculocerebrorenal syndrome of Lowe (	-3.111441	0.000338	0.010088
1371888_at	295224	mrpl24	mitochondrial ribosomal protein L24	2.261078	0.000339	0.010088
1369577_at	84607	Socs2	suppressor of cytokine signaling 2	-2.178755	0.000339	0.010088
1391730_at	362996	Mll2_predic	myeloid/lymphoid or mixed-lineage leu	-2.115952	0.000339	0.010088
1388466_at	287670	Psmd3	proteasome (prosome, macropain) 26S	2.392958	0.00034	0.010088
1374538_at	362567	Pomgnt1	protein O-linked mannose beta1,2-N-a	1.889104	0.00034	0.010088
1389919_at	362973	Parvb_pre	parvin, beta (predicted)	2.924014	0.00034	0.010088
1373562_at	309200	RGD1309З	similar to RIKEN cDNA 1110055N21 (p	2.289503	0.000342	0.010088
1394039_at	84410	Klf5	Kruppel-like factor 5	-3.244824	0.000342	0.010088
1387148_at	171407	Gprasp1	G protein-coupled receptor associated	-1.78918	0.000342	0.010088
1369813_at	79130	Dnajc5	DnaJ (Hsp40) homolog, subfamily C, r	2.413801	0.000343	0.010088
1382118_at	619476	Apeg3	antisense paternally expressed gene 3	2.249868	0.000345	0.010088
1373490_at	113940	Gmfg	glia maturation factor, gamma	2.045929	0.000345	0.010088
1376795_at	294048	Pik3ap1_pr	phosphoinositide-3-kinase adaptor pro	-1.765033	0.000345	0.010088
1372503_at	360548	Tnfsf12	tumor necrosis factor ligand superfami	-2.140746	0.000346	0.010088
1373049_at	501195	RGD15621	similar to D1Ertd622e protein (predicte	-1.647531	0.000346	0.010088
1377625_at	311441	RGD13088	similar to CGI-09 protein (predicted)	3.570699	0.000349	0.010088
1387135_at	57025	Adam15	a disintegrin and metallopeptidase dom	-3.472684	0.000349	0.010088
1371353_at	113894	Sqstm1	sequestosome 1	2.074771	0.000349	0.010088
1392974_at	294568	Wasf1	WAS protein family, member 1	-2.015632	0.00035	0.010088
1396866_s_at	29534	Pxmp3	peroxisomal membrane protein 3	1.734376	0.00035	0.010088
1388622_at	501161	RGD15635	similar to Nol5a_predicted protein (pre	1.609333	0.00035	0.010088
1399013_at	296049	RGD13109	similar to RIKEN cDNA 2610318K02 (p	1.920288	0.000351	0.010088
1373822_at	502367	RGD15653	similar to RIKEN cDNA 1110025L05 (p	1.631563	0.000351	0.010088
1367907_a_at	116561	Cltb	clathrin, light polypeptide (Lcb)	1.671436	0.000351	0.010088
1368221_at	24413	Nr3c1	nuclear receptor subfamily 3, group C,	-2.539984	0.000353	0.010127
1373758_at	113917	Lenep	lens epithelial protein	2.224552	0.000356	0.010173
1367728_at	60381	Tsn	translin	2.550046	0.000358	0.010176
1368034_at	24259	Chgb	chromogranin B	-2.074041	0.000359	0.010176
1368098_a_at	81781	Snrpn	small nuclear ribonucleoprotein N	1.705049	0.00036	0.010176
1372505_at	64351	Ykt6	prenylated SNARE protein	2.532446	0.00036	0.010176
1398255_at	60577	Slc15a2	solute carrier family 15 (H+/peptide tra	-3.493425	0.000361	0.010176
1375441_at	266975	Sars1	seryl-aminoacyl-tRNA synthetase 1	2.146513	0.000363	0.010176
1387915_at	286900	Sels	selenoprotein S	1.612477	0.000363	0.010176
1383981_at	305025	Trp53bp2_	transformation related protein 53 bindi	-1.756706	0.000363	0.010176
1386859_at	64524	Tkt	transketolase	1.840002	0.000363	0.010176
1369041_at	116647	Nlgn1	neuroligin 1	-2.986072	0.000364	0.010176
1383467_at	362132	Epc2_pred	enhancer of polycomb homolog 2 (Dro	-2.236761	0.000364	0.010176
1376718_at	304346	RGD13069	similar to CG9117-PA	1.805612	0.000365	0.01018

1368389_at	83611	Apba3	amyloid beta (A4) precursor protein-bi	2.073748	0.000367	0.010198
1383602_at	362609	Pum1_prec	pumilio 1 (Drosophila) (predicted)	-1.970606	0.000368	0.010218
1389567_at	301024	Scap_pred	SREBP cleavage activating protein (pr	2.021735	0.000371	0.010249
1384525_at	313438	Dock11	dedicator of cytokinesis 11	-3.188848	0.000371	0.010249
1371391_at	306869	Txndc5_pr	thioredoxin domain containing 5 (predi	1.659358	0.000372	0.010249
1388432_at	246294	Optn	optineurin	-1.969442	0.000372	0.010249
1398385_at	305714	RGD13054	similar to RIKEN cDNA 1500006O09 (	-1.644122	0.000373	0.010255
1396619_at	311362	RGD15615	similar to MGC14161 protein (predicte	2.528521	0.000377	0.010273
1379909_at	361202	Gkap1	G kinase anchoring protein 1	-2.284115	0.000377	0.010273
1386979_at	170907	Tpo1	developmentally regulated protein TPC	-2.724549	0.000378	0.010273
1377837_at	314313	Znf183	zinc finger protein 183 (RING finger, C	-1.886976	0.000378	0.010273
1390814_at	305549	Peli1	pellino homolog 1 (Drosophila)	-2.094135	0.000378	0.010273
1371620_at	290995	RGD1308C	similar to px19-like protein	1.694203	0.000379	0.010273
1388643_at	432392	Fut8	fucosyltransferase 8 (alpha (1,6) fucos	1.623688	0.00038	0.010273
1387109_at	29441	Por	P450 (cytochrome) oxidoreductase	1.720625	0.00038	0.010273
1387666_at	64020	Gpr85	G protein-coupled receptor 85	-2.986524	0.000381	0.010273
1391078_at	89809	Recc1	replication factor C 1	2.191922	0.000383	0.010311
1394038_at	373066	Cdv1	carnitine deficiency-associated gene e	-2.568448	0.000385	0.010321
1370521_at	64060	Vps33b	vacuolar protein sorting 33B (yeast)	2.31937	0.000385	0.010321
1381229_at	313678	Prdm2_ma	PR domain containing 2, with ZNF dor	-2.45062	0.000386	0.010335
1374874_at	498386	RGD1561C	similar to RIKEN cDNA 5730509K17 g	-3.239527	0.000388	0.010351
1383653_at	303493	Snx11	sorting nexin 11	2.222302	0.000389	0.010351
1387294_at	117186	Sh3bp5	SH3-domain binding protein 5 (BTK-as	-2.229041	0.000391	0.010357
1367915_at	84497	Dgat1	diacylglycerol O-acyltransferase 1	1.920375	0.000391	0.010357
1375720_at	81657	Gabbr1	gamma-aminobutyric acid (GABA) B re	-1.92505	0.000391	0.010357
1369792_at	83683	Gpr6	G protein-coupled receptor 6	5.071524	0.000392	0.010371
1387180_at	117022	Il1r2	interleukin 1 receptor, type II	1.883646	0.000394	0.010377
1384302_at	613226	Slc6a17	NA	-2.217321	0.000395	0.010377
1377143_at	287642	Slc35b1	solute carrier family 35, member B1	1.609435	0.000396	0.010377
1376733_at	303926	Igsf11	immunoglobulin superfamily, member	3.207143	0.000398	0.010377
1379824_at	362481	Tox_predic	thymocyte selection-associated HMG I	-3.138024	0.000398	0.010377
1376993_at	315430	Amotl1_pre	angiomotin-like 1 (predicted)	4.562286	0.000399	0.010377
1372270_at	300668	H2afx	dolichyl-phosphate (UDP-N-acetylgluc	1.560917	0.0004	0.010377
1372441_at	117535	Chd4	chromodomain helicase DNA binding p	1.650642	0.0004	0.010377
1388827_at	289784	H2afv_prec	H2A histone family, member V (predict	-1.722702	0.000401	0.010377
1380968_at	497926	RGD15646	similar to novel protein (predicted)	4.406423	0.000401	0.010377
1367865_at	170946	Lkap	limkain b1	1.59097	0.000401	0.010377
1393175_at	499137	RGD1562C	similar to RIKEN cDNA 2810426N06 (p	-1.612242	0.000403	0.01038
1373773_at	306439	Gpm6a	glycoprotein m6a	-4.263694	0.000404	0.01038
1369799_at	81632	Abat	4-aminobutyrate aminotransferase	1.885045	0.000404	0.01038
1370235_at	25045	Dbi	diazepam binding inhibitor	-1.535498	0.000408	0.010464
1379398_at	289051	Fam31b_p	family with sequence similarity 31, mer	2.007561	0.000411	0.010519
1368721_at	24209	Ascl2	achaete-scute complex homolog-like 2	2.81917	0.000413	0.010557
1382196_at	360667	RGD13073	similar to hypothetical protein ET (prec	2.785285	0.000414	0.010568
1388420_at	361100	LOC36110	NA	-1.7783	0.000415	0.010578
1368184_at	161475	Psmd9	proteasome (prosome, macropain) 26S	1.697236	0.000419	0.010645
1370859_at	286906	Pdia6	protein disulfide isomerase associated	1.947899	0.00042	0.010645
1393276_at	287475	Med31_pre	mediator of RNA polymerase II transcr	-2.116636	0.000423	0.01068
1374532_at	311865	Ptges2_pre	prostaglandin E synthase 2 (predicted)	2.033763	0.000424	0.01068
1388853_at	299628	Mrpl54_pre	mitochondrial ribosomal protein L54 (p	1.766777	0.000425	0.01068
1381388_at	304984	Ccdc19	coiled-coil domain containing 19	2.334366	0.000425	0.01068

1379013_at	296733	RGD13064	similar to mKIAA1402 protein (predicte	2.202716	0.000426	0.01068
1368858_at	50555	Ugt8	UDP galactosyltransferase 8	1.576717	0.000426	0.01068
1376636_at	29591	Tgfbr1	transforming growth factor, beta recep	-1.59466	0.000427	0.01068
1391739_at	303552	Rundc1_pr	RUN domain containing 1 (predicted)	2.446522	0.000428	0.01068
1372248_at	294518	Sesn1_pre	sestrin 1 (predicted)	-2.724981	0.000431	0.010684
1370223_at	117051	Arfrp1	ADP-ribosylation factor related protein	1.617107	0.000431	0.010684
1373185_at	295235	Ssr2_predi	signal sequence receptor, beta (predic	2.251382	0.000433	0.010684
1390937_at	171026	Akap5	A kinase (PRKA) anchor protein 5	-1.954075	0.000434	0.010684
1393136_at	313618	LOC31361	macoilin	1.770281	0.000435	0.010684
1367811_at	58835	Phgdh	3-phosphoglycerate dehydrogenase	4.488335	0.000436	0.010684
1368288_at	24384	Gc	group specific component	-2.343112	0.000436	0.010684
1387042_at	25297	Cacnb3	calcium channel, voltage-dependent, b	-1.568013	0.000437	0.010684
1368606_at	80900	Slco1a5	solute carrier organic anion transporter	3.65895	0.000437	0.010684
1386872_at	25151	Igf2r	insulin-like growth factor 2 receptor	2.150627	0.000438	0.010684
1393364_at	266733	Slc12a8	solute carrier family 12 (potassium/chl	3.300996	0.000438	0.010684
1387817_at	25247	Nsg1	neuron specific gene family member 1	1.619468	0.000439	0.010684
1376118_at	314405	Otub2_pre	OTU domain, ubiquitin aldehyde bindir	2.134969	0.000439	0.010684
1369944_at	81520	Marcksl1	MARCKS-like 1	-2.084255	0.000439	0.010684
1398270_at	29373	Bmp2	bone morphogenetic protein 2	-3.296427	0.00044	0.010684
1383089_at	299799	Rab21	RAB21, member RAS oncogene family	-1.725069	0.000441	0.010684
1387779_at	60571	Mybbp1a	MYB binding protein (P160) 1a	1.592516	0.000442	0.010697
1388463_at	300988	Tex264	testis expressed gene 264 homolog (m	2.273184	0.000445	0.010754
1397630_at	316395	LOC31639	NA	2.813695	0.000447	0.010786
1383476_at	50693	Itih3	inter-alpha trypsin inhibitor, heavy chai	-1.503557	0.000448	0.010793
1374661_at	361571	LOC36157	similar to RIKEN cDNA 2410004H02	1.844752	0.000451	0.010827
1388749_at	312439	RGD13097	similar to hypothetical protein FLJ1391	-1.553234	0.000452	0.010827
1398606_at	310526	Golph4	golgi phosphoprotein 4	-1.566889	0.000452	0.010827
1378679_at	304150	Usp25_pre	ubiquitin specific protease 25 (predicte	-3.911154	0.000454	0.010843
1385522_at	313479	Orc1l	origin recognition complex, subunit 1-li	-3.478407	0.000455	0.010843
1367568_a_at	25333	Mgp	matrix Gla protein	-3.758816	0.000456	0.010848
1398971_at	303280	RGD13079	similar to CG14967-PA	1.624221	0.000457	0.010854
1382268_at	293024	Akap13	A kinase (PRKA) anchor protein 13	2.004868	0.000459	0.010883
1384886_at	500057	RGD15601	similar to hypothetical protein LOC168	-2.51611	0.00046	0.010896
1386918_a_at	29336	Oprs1	opioid receptor, sigma 1	1.621417	0.000461	0.010896
1389116_at	282584	Mtmr9	myotubularin related protein 9	1.787797	0.000463	0.010897
1398975_at	301512	Aamp_pred	angio-associated migratory protein (pr	1.695539	0.000465	0.010897
1370817_at	266758	Sec11l3	Sec11-like 3 (S. cerevisiae)	1.59713	0.000466	0.010897
1371890_at	116665	Rere	arginine-glutamic acid dipeptide (RE) r	-1.515651	0.000466	0.010897
1371449_at	298696	Pin1_predi	protein (peptidyl-prolyl cis/trans isomer	1.649844	0.000466	0.010897
1398858_at	287984	Psmd2	proteasome (prosome, macropain) 26S	1.625974	0.000466	0.010897
1368321_at	24330	Egr1	early growth response 1	-3.982089	0.000468	0.010897
1393588_at	304073	Cldn14	claudin 14	3.674315	0.000469	0.010897
1391856_at	290562	Sema3g	sema domain, immunoglobulin domain	-2.263654	0.000469	0.010897
1379580_at	311607	Chd6_pred	chromodomain helicase DNA binding p	-2.532793	0.00047	0.010897
1374876_at	361160	Leprotl1	leptin receptor overlapping transcript-li	1.832577	0.00047	0.010897
1390257_at	60431	Vapb	vesicle-associated membrane protein,	1.526941	0.000471	0.010897
1384971_at	295971	RGD13097	similar to Hypothetical protein MGC19	-2.287473	0.000472	0.01091
1387228_at	25351	Slc2a2	solute carrier family 2 (facilitated gluco	4.360856	0.000474	0.010937
1375213_at	361042	Pck2_pred	phosphoenolpyruvate carboxykinase 2	2.430073	0.000477	0.010979
1367609_at	81683	Mif	macrophage migration inhibitory factor	2.188612	0.000479	0.010994
1370193_at	29463	Ptp4a1	protein tyrosine phosphatase 4a1	-1.935499	0.000479	0.010994

1383054_at	246282	Zfp91	zinc finger protein 91	1.69565	0.000481	0.01101
1378376_at	83835	Chic2_prec	cysteine-rich hydrophobic domain 2 (p	2.486457	0.000483	0.01101
1368509_at	113948	Bbs2	Bardet-Biedl syndrome 2 homolog (hur	-1.674173	0.000484	0.01101
1398425_at	500400	MGC1091S	similar to hypothetical protein MGC393	2.496723	0.000485	0.01101
1380182_at	291356	RGD15634	similar to KIAA1217 (predicted)	-2.664542	0.000485	0.01101
1369976_at	58945	Dynll1	dynein light chain LC8-type 1	-1.578192	0.000486	0.01101
1369226_at	24903	Kng1	kininogen 1	3.960717	0.000486	0.01101
1382189_at	25615	Sdc2	syndecan 2	-1.883234	0.000487	0.011031
1375520_at	315345	Copz1_pre	coatomer protein complex, subunit zet	1.97221	0.000489	0.011037
1376465_at	310233	RGD15638	sim ilar to glucocorticoid induced gene	-2.186257	0.00049	0.011037
1367716_at	171083	Cda08	T-cell immunomodulatory protein	-1.527318	0.000491	0.011037
1398426_at	498309	RGD15641	similar to RIKEN cDNA B130052G07 (	1.779747	0.000491	0.011037
1389912_at	60334	Ensa	endosulfine alpha	2.035915	0.000492	0.011037
1372303_at	299909	RGD13076	similar to 0910001A06Rik protein (prec	-1.918279	0.000493	0.011038
1393547_at	498726	RGD15603	similar to hypothetical protein C630023	4.062729	0.000494	0.011041
1368967_at	171145	Eif2b3	eukaryotic translation initiation factor 2	1.934317	0.000497	0.011062
1380828_at	29705	Gabra1	gamma-aminobutyric acid A receptor,	1.838115	0.000498	0.011062
1372528_at	60355	Nsf	N-ethylmaleimide sensitive fusion prot	1.82087	0.000499	0.011062
1373978_at	298075	Ncbp1	nuclear cap binding protein subunit 1,	1.786292	0.0005	0.011062
1379273_at	300472	LOC30047	NA	1.494341	0.0005	0.011062
1372464_at	297428	Copg	coatomer protein complex, subunit gar	1.629767	0.0005	0.011062
1372702_at	287750	PRP-2	proline-rich protein	1.688035	0.000501	0.011062
1374014_at	353233	Nsmaf	neutral sphingomyelinase (N-SMase) a	-1.479974	0.000504	0.011114
1371762_at	25703	Rbp4	retinol binding protein 4, plasma	-5.513921	0.000505	0.011114
1392747_at	308283	Fbxo30	F-box protein 30	-2.444857	0.000505	0.011114
1367929_at	25407	Cd59	CD59 antigen	-1.99972	0.000509	0.011165
1378136_at	89787	Lrp3	low density lipoprotein receptor-relatec	-2.196062	0.00051	0.011171
1369074_at	170573	Slc38a4	solute carrier family 38, member 4	2.024086	0.000511	0.011171
1398930_at	298451	Atp6v0b_p	ATPase, H+ transporting, V0 subunit E	1.489883	0.000511	0.011171
1368702_at	64513	Pawr	PRKC, apoptosis, WT1, regulator	-2.175328	0.000514	0.011201
1398792_at	117263	Psmc1	peptidase (prosome, macropain) 26S s	1.86483	0.000515	0.011201
1369559_a_at	29364	Cd47	CD47 antigen (Rh-related antigen, inte	1.790922	0.000516	0.011201
1370004_at	29384	H2afy	H2A histone family, member Y	1.519294	0.000516	0.011201
1375426_a_at	171137	Khsrp	KH-type splicing regulatory protein	2.191339	0.000517	0.011201
1380062_at	362359	Mpp6_prec	membrane protein, palmitoylated 6 (M/	1.83618	0.000518	0.011201
1370989_at	24716	Ret	ret proto-oncogene	-2.054663	0.000522	0.011231
1398769_at	29474	Coro1b	coronin, actin-binding protein, 1B	1.518928	0.000522	0.011231
1368320_at	24586	Ncam1	neural cell adhesion molecule 1	-2.227597	0.000523	0.011231
1390031_at	304496	RGD13118	similar to hypothetical protein FLJ1446	1.605409	0.000524	0.011231
1374980_at	304656	RGD13075	similar to RIKEN cDNA 4930527D15	1.523995	0.000524	0.011231
1368642_at	83501	Cdh2	cadherin 2	-2.126551	0.000525	0.011231
1377761_at	360518	Gfpt2	glutamine-fructose-6-phosphate transa	-2.472087	0.000526	0.011231
1395316_at	367767	Mageh1	melanoma antigen, family H, 1	-2.347205	0.000527	0.011231
1384112_at	58813	Nt5e	5' nucleotidase, ecto	-2.975936	0.000528	0.011231
1383172_at	294429	RGD15600	similar to Ran-binding protein 2 (predic	-1.515307	0.000528	0.011231
1390534_at	293967	Smc5l1_pr	SMC5 structural maintenance of chron	-1.886825	0.000529	0.011231
1388517_at	287962	Mrpl40	mitochondrial ribosomal protein L40	1.456618	0.00053	0.011252
1388120_at	501083	Pdcd6ip	programmed cell death 6 interacting pr	1.704873	0.000534	0.011305
1367746_a_at	83764	Flot2	flotillin 2	1.968689	0.000535	0.011307
1387226_at	24503	Inexa	internexin, alpha	-1.573088	0.000536	0.011307
1370268_at	25470	Kcna5	potassium voltage-gated channel, shal	-2.842616	0.000536	0.011307

1367903_at	79239	Hmox2	heme oxygenase (decycling) 2	2.184561	0.000537	0.011307
1372313_at	362003	Gpr89_pre	G protein-coupled receptor 89 (predict	2.443713	0.00054	0.011341
1392906_at	293864	Ubl4a_pre	ubiquitin-like 4a (predicted)	1.835419	0.000541	0.011341
1367771_at	83514	Tsc22d3	TSC22 domain family 3	-1.825244	0.000541	0.011341
1393489_at	296610	Rsb66	Rsb-66 protein	-2.404438	0.000543	0.01136
1368314_at	81716	Ggcx	gamma-glutamyl carboxylase	1.451485	0.000544	0.01136
1369940_at	83688	Taldo1	transaldolase 1	1.445546	0.000546	0.011372
1370678_s_at	29253	Maoa	monoamine oxidase A	1.60732	0.000547	0.011375
1374545_at	307271	Rkhd2_pre	ring finger and KH domain containing 2	-1.952582	0.00055	0.011382
1374468_at	301059	Myd88	myeloid differentiation primary respons	1.6415	0.00055	0.011382
1371574_at	290596	Ghitm	growth hormone inducible transmembr	1.701829	0.000552	0.011382
1388341_at	362965	Rangap1	RAN GTPase activating protein 1	2.572435	0.000553	0.011382
1372888_at	315608	Ube4a	ubiquitination factor E4A, UFD2 homol	1.921709	0.000554	0.011382
1372653_at	300211	Fkbp11	FK506 binding protein 11	1.995486	0.000555	0.011382
1398788_at	29468	Pdia3	protein disulfide isomerase associated	2.023134	0.000555	0.011382
1390784_at	307075	Wdr37_pre	WD repeat domain 37 (predicted)	1.90194	0.000555	0.011382
1378334_a_at	81678	Itpr2	inositol 1,4,5-triphosphate receptor 2	-2.257449	0.000555	0.011382
1388508_at	498600	RGD15612	similar to RIKEN cDNA 2510049I19 (p	2.03185	0.000558	0.011382
1367706_at	83529	Vdac1	voltage-dependent anion channel 1	1.684209	0.00056	0.011382
1382001_at	502710	LOC50271	NA	-1.792578	0.000561	0.011382
1374032_at	303274	Phf12	PHD finger protein 12	-2.002386	0.000562	0.011382
1398984_at	290833	Tm2d2	TM2 domain containing 2	1.518665	0.000562	0.011382
1388236_x_at	309600	RT1-CE12	RT1 class I, CE12	1.873237	0.000562	0.011382
1370300_at	58842	Preb	prolactin regulatory element binding	2.282525	0.000563	0.011382
1368889_at	65277	Vti1a	vesicle transport through interaction wi	1.451866	0.000565	0.011382
1394935_at	313024	Wasf2	WAS protein family, member 2	2.433431	0.000566	0.011382
1371754_at	246771	Slc25a25	solute carrier family 25 (mitochondrial	-1.600099	0.000566	0.011382
1369209_at	171435	P34	p34 protein	3.035538	0.000566	0.011382
1368844_at	29734	Stch	stress 70 protein chaperone, microson	1.540604	0.000566	0.011382
1368005_at	25679	Itpr3	inositol 1,4,5-triphosphate receptor 3	1.808175	0.000566	0.011382
1378656_at	362142	Bbs5_pred	Bardet-Biedl syndrome 5 homolog (hu	-1.959504	0.000567	0.011382
1371357_at	289560	Igfbp7	insulin-like growth factor binding protei	-1.888836	0.000568	0.011399
1368035_a_at	360406	Ptprf	protein tyrosine phosphatase, receptor	1.952992	0.000572	0.011453
1373254_at	450225	Krt10	keratin 10	-2.031053	0.000574	0.011475
1387016_a_at	56064	Sdfr1	stromal cell derived factor receptor 1	-1.496642	0.000575	0.011475
1371987_at	306672	Pols_predi	polymerase (DNA directed) sigma (pre	-1.632528	0.000577	0.011493
1384016_at	500790	RGD15636	similar to R31449_3 (predicted)	1.748802	0.000578	0.011493
1389519_at	292766	Psmd8	proteasome (prosome, macropain) 26S	2.121249	0.000578	0.011493
1388852_at	361578	RGD13052	similar to pM5 protein; DNA segment,	2.192727	0.000579	0.011493
1386080_at	155437	Hey1	hairy/enhancer-of-split related with YR	-2.635891	0.000584	0.011573
1367793_at	29318	Ddt	D-dopachrome tautomerase	2.011725	0.000586	0.011592
1392214_at	317213	Brwd3_pre	bromodomain and WD repeat domain	5.012278	0.000587	0.011592
1388185_at	24708	Rb1	retinoblastoma 1	-1.481506	0.000588	0.011592
1371392_at	292804	Gpi	glucose phosphate isomerase	2.256489	0.00059	0.011609
1393317_at	500288	RGD15608	similar to THUMP domain containing 3	1.671529	0.00059	0.011609
1368025_at	140942	Ddit4	DNA-damage-inducible transcript 4	3.318999	0.000592	0.011623
1385308_at	363261	Mogat1_pr	monoacylglycerol O-acyltransferase 1	2.001343	0.000593	0.011623
1375929_at	287521	Mnt_predic	max binding protein (predicted)	-1.960665	0.000594	0.011623
1375853_at	314690	RGD13099	similar to CG13957-PA (predicted)	-1.4879	0.000594	0.011624
1386059_at	295274	RGD15623	similar to histone H2b-613 (predicted)	-2.022506	0.000597	0.01165
1388800_at	64633	Rab5a	RAB5A, member RAS oncogene famil	-1.700136	0.000601	0.01171

1375958_at	304361	LOC30436	similar to Hypothetical protein MGC28	1.617268	0.000604	0.011764
1382203_at	306351	Gdf1_predi	growth differentiation factor 1 (predicte	1.663376	0.000605	0.011764
1373591_at	293344	Arfip2	ADP-ribosylation factor interacting prot	2.194238	0.000606	0.011766
1382254_at	64183	Pde4dip	phosphodiesterase 4D interacting prot	-1.678748	0.000608	0.011771
1374903_at	306860	Gcnt2	glucosaminyl (N-acetyl) transferase 2,	-2.05537	0.000609	0.011771
1391897_at	304914	RGD13046	similar to rab6 GTPase activating prote	-1.903546	0.000609	0.011771
1371255_at	293621	Hras	Harvey rat sarcoma viral (v-Ha-ras) on	1.642893	0.00061	0.011771
1370957_at	25205	Il6st	interleukin 6 signal transducer	1.858738	0.000611	0.011774
1371481_at	364838	Reep5_pre	receptor accessory protein 5 (predicted	1.5814	0.000612	0.011776
1377744_at	307248	RGD15630	similar to macrophage actin-associated	3.675951	0.000614	0.011788
1370548_at	170566	Slc16a10	solute carrier family 16 (monocarboxyl	-2.191767	0.000617	0.011788
1370855_at	25307	Cst3	cystatin C	-1.838245	0.000619	0.011788
1367872_at	29663	Ap1b1	adaptor protein complex AP-1, beta 1 s	1.565025	0.000619	0.011788
1375146_at	360636	RGD13061	similar to RIKEN cDNA 3010027G13	1.619316	0.000619	0.011788
1371557_at	363291	Thap4	THAP domain containing 4	1.715484	0.000619	0.011788
1368973_at	81635	Adar	adenosine deaminase, RNA-specific	1.875654	0.000619	0.011788
1378690_at	362935	Ly6a_predi	lymphocyte antigen 6 complex, locus A	1.524619	0.000621	0.011788
1371437_at	297522	Sec13l1	SEC13-like 1 (S. cerevisiae)	1.573803	0.000621	0.011788
1387255_at	25120	Aanat	arylalkylamine N-acetyltransferase	3.857533	0.000622	0.011788
1398785_at	29417	Men1	multiple endocrine neoplasia 1	1.594187	0.000622	0.011788
1377041_at	315119	Mfng	manic fringe homolog (Drosophila)	3.466848	0.000624	0.0118
1371936_at	287436	Eif4a1	eukaryotic translation initiation factor 4	2.616601	0.000626	0.011821
1372243_at	301574	Cab39_pre	calcium binding protein 39 (predicted)	1.832619	0.000629	0.011853
1375911_at	362219	RGD73514	hypothetical protein LK44	-1.95509	0.000631	0.011874
1368809_at	64185	Cap1	CAP, adenylate cyclase-associated pro	1.611554	0.000632	0.011878
1387979_at	192243	Golgb1	golgi autoantigen, golgin subfamily b, r	1.936206	0.000637	0.011941
1383652_at	81528	Ogg1	8-oxoguanine DNA-glycosylase 1	1.510967	0.000638	0.011941
1375019_at	361838	Hnrph3_pr	heterogeneous nuclear ribonucleoprote	-1.861845	0.000638	0.011941
1397959_at	312248	RGD13107	similar to RIKEN cDNA D130059P03 g	1.52267	0.000639	0.011941
1370804_at	58974	Gabarap	gamma-aminobutyric acid receptor ass	1.501968	0.00064	0.011941
1374491_at	301045	Cmtm8	CKLF-like MARVEL transmembrane do	-2.630232	0.000641	0.011941
1371373_at	297337	MGC94464	similar to RIKEN cDNA 2500002L14; E	1.521583	0.000641	0.011941
1381903_at	314157	Fbxo33_pr	F-box only protein 33 (predicted)	-2.099515	0.000644	0.011967
1390619_at	315216	RGD13119	hypothetical LOC315216 (predicted)	3.580101	0.000647	0.012003
1372015_at	306562	Tacc1	transforming, acidic coiled-coil contain	-3.957294	0.000649	0.012003
1370842_at	29603	Bckdk	branched chain ketoacid dehydrogena	1.99591	0.000649	0.012003
1371266_at	282708	Afm	afamin	-2.418652	0.000649	0.012003
1375347_at	303617	Falz_predic	fetal Alzheimer antigen (predicted)	-1.421315	0.00065	0.012007
1393510_at	500865	RGD15643	similar to RIKEN cDNA 5730410E15 g	-2.128235	0.000655	0.012014
1368867_at	59117	Eif2c2	eukaryotic translation initiation factor 2	2.626382	0.000655	0.012014
1375641_at	296710	Arpc5l	actin related protein 2/3 complex, subu	1.549278	0.000656	0.012014
1382285_at	315165	Naga	N-acetyl galactosaminidase, alpha	2.37633	0.000656	0.012014
1389467_at	497979	MGC10877	similar to RIKEN cDNA 1810057C19	1.882171	0.000656	0.012014
1384742_at	246284	Atrx	alpha thalassemia/mental retardation s	-2.63646	0.000658	0.012014
1369868_at	116967	Iag2	implantation-associated protein	2.844109	0.00066	0.012014
1374413_at	363869	Ubl3	ubiquitin-like 3	-1.471673	0.00066	0.012014
1371929_at	360631	Mlx	MAX-like protein X	1.687425	0.00066	0.012014
1388186_at	286911	LOC28691	cationic trypsinogen	-4.212983	0.00066	0.012014
1392972_at	310192	Trio	triple functional domain (PTPRF intera	1.717974	0.000661	0.012014
1393892_at	360508	RGD13066	LOC360508 (predicted)	2.8355	0.000664	0.012058
1367997_at	65041	Clpb	ClpB caseinolytic peptidase B homolog	2.202069	0.000665	0.012058

1373242_at	292173	Tbpl1_prec	TATA box binding protein-like 1 (predic	-1.361111	0.000667	0.012058
1379375_at	25266	Pdgfa	platelet derived growth factor, alpha	-1.823972	0.000668	0.012058
1370321_at	83533	Pdcd8	programmed cell death 8	1.763785	0.000669	0.012058
1370261_at	81771	Rps6ka1	ribosomal protein S6 kinase polypeptic	1.41553	0.000669	0.012058
1374747_at	362316	Pftk1_pred	PFTAIRE protein kinase 1 (predicted)	1.678596	0.00067	0.012058
1374387_at	66028	Arl6ip5	ADP-ribosylation factor-like 6 interactir	1.45175	0.000672	0.012089
1372792_at	292780	LOC29278	similar to hypothetical protein MGC156	1.436046	0.000674	0.012089
1373853_at	299647	RGD13081	similar to hypothetical protein MGC157	1.501802	0.000674	0.012089
1386864_at	24642	Pgam1	phosphoglycerate mutase 1	1.84207	0.000676	0.012101
1388626_at	296162	RGD13099	similar to chromosome 20 open readin	1.636866	0.00068	0.012116
1388861_at	316916	RGD13078	similar to cisplatin resistance related p	1.805555	0.00068	0.012116
1371341_at	292688	Snrpd2_pre	small nuclear ribonucleoprotein D2 (pr	1.795957	0.000681	0.012116
1385252_at	293294	Trim34_pre	tripartite motif protein 34 (predicted)	-2.054475	0.000681	0.012116
1368255_at	50864	Hnt	neurotrimin	5.059409	0.000682	0.012116
1373911_at	361945	Postn_prec	periostin, osteoblast specific factor (pre	-4.360331	0.000682	0.012116
1384448_at	317486	RGD15658	similar to RIKEN cDNA 1700045I19 (p	-2.586012	0.000683	0.012116
1371797_at	24240	Cad_mapp	carbamyl phosphatate synthetase 2 (m	1.827348	0.000684	0.012116
1375245_at	117281	Ppp2r1a	protein phosphatase 2 (formerly 2A), re	1.434104	0.000685	0.012116
1371899_at	311130	Prkra	protein kinase, interferon inducible dou	-1.897667	0.000687	0.012119
1393820_at	681302	LOC68130	NA	2.099619	0.000687	0.012119
1386987_at	24499	Il6ra	interleukin 6 receptor, alpha	-1.739839	0.000688	0.012119
1374945_at	314462	RGD13591	GCD14/PCMT domain containing prote	2.394498	0.000688	0.012119
1375646_at	289280	Efcab2_pre	EF-hand calcium binding domain 2 (pr	-2.348183	0.000691	0.012143
1368373_at	54296	Rgs7	regulator of G-protein signaling 7	-1.70141	0.000695	0.012198
1375785_at	362520	Fcmd_prec	Fukuyama type congenital muscular dy	1.642059	0.000697	0.012213
1384008_at	361275	RGD13058	similar to hypothetical protein FLJ4028	-1.699361	0.000698	0.01222
1387212_at	25334	Bhlhb8	basic helix-loop-helix domain containir	2.878914	0.000701	0.012252
1367459_at	64310	Arf1	ADP-ribosylation factor 1	1.78008	0.000703	0.01228
1380334_at	305340	RGD13597	hypothetical RNA binding protein RGD	1.385785	0.000705	0.01228
1367834_at	84596	Srm	spermidine synthase	1.934588	0.000707	0.01228
1397838_at	499976	RGD15647	RGD1564792 (predicted)	-2.206128	0.000708	0.01228
1370523_a_at	641452	Ubc2e	NA	-2.12784	0.00071	0.01228
1376441_at	500671	NA	NA	-1.82426	0.000711	0.01228
1399097_at	361217	Spin	spindlin	-1.423328	0.000711	0.01228
1372990_at	298400	Creb3	cAMP responsive element binding prot	1.587594	0.000712	0.01228
1373916_at	170915	Ep300	E1A binding protein p300	-1.506131	0.000712	0.01228
1378312_at	316356	RGD15622	similar to RIKEN cDNA 4931419K03 (p	4.294362	0.000712	0.01228
1375063_at	287059	RGD15651	similar to MGC45438 protein (predicte	3.850794	0.000713	0.01228
1372962_at	363006	Tarbp2	TAR (HIV) RNA binding protein 2	1.781774	0.000715	0.01228
1397241_at	313553	RGD13068	similar to hypothetical protein MGC478	2.131823	0.000715	0.01228
1372431_at	303746	Mrpl12	ribosomal protein, mitochondrial, L12	1.929208	0.000715	0.01228
1374533_at	499883	RGD15603	similar to GA binding protein transcript	-1.458295	0.000716	0.012281
1367695_at	64192	Qdpr	quinoid dihydropteridine reductase	1.87263	0.000719	0.012303
1382139_at	315804	RGD13087	similar to hypothetical protein FLJ1299	-1.341446	0.000721	0.012303
1371702_at	363447	Tm4sf2_ma	transmembrane 4 superfamily member	-1.406449	0.000721	0.012303
1368438_at	63885	Pde10a	phosphodiesterase 10A	3.577066	0.000721	0.012303
1373447_at	360492	RGD13051	similar to HN1-like protein	1.696521	0.000722	0.012303
1389407_at	290234	Dhrs1	dehydrogenase/reductase (SDR family	1.709145	0.000725	0.012334
1381006_at	58947	Hgfac	hepatocyte growth factor activator	-2.675821	0.000726	0.012334
1370658_a_at	266680	St18	suppression of tumorigenicity 18	2.021345	0.000727	0.012338
1373546_at	246251	Ua20	putative UA20 protein	-1.452991	0.000729	0.012354

1367647_at	24648	Serpina1	serine (or cysteine) proteinase inhibito	-3.042444	0.00073	0.012354
1368469_at	25241	Aqp5	aquaporin 5	3.364468	0.000734	0.012398
1372343_at	295050	Exosc8_pr	exosome component 8 (predicted)	-1.700443	0.000736	0.012398
1396722_at	140928	Pde11a	phosphodiesterase 11A	1.503388	0.000737	0.012398
1373561_at	316533	D1bwg136	chondroitin polymerizing factor	1.648802	0.000739	0.012398
1399159_a_at	29528	Vamp3	vesicle-associated membrane protein :	-1.349621	0.00074	0.012398
1373870_at	313873	RGD13054	similar to RIKEN cDNA 2810405J04	1.444174	0.000741	0.012398
1371786_at	498538	Trim35	tripartite motif protein 35	-1.429757	0.000741	0.012398
1371925_at	290673	Atp13a1_p	ATPase type 13A1 (predicted)	1.925267	0.000742	0.012398
1373282_at	313720	RGD1306€	similar to CG7744-PA (predicted)	-4.016114	0.000742	0.012398
1382261_at	287822	RGD13114	similar to CG8841-PA (predicted)	2.09726	0.000742	0.012398
1370127_at	59294	Pold1	polymerase (DNA directed), delta 1, ca	2.478566	0.000743	0.012398
1372602_at	305234	RGD1311€	similar to genethonin 1	1.331491	0.000744	0.012403
1374976_a_at	81782	Soat1	sterol O-acyltransferase 1	2.024035	0.000748	0.012444
1388331_at	362862	Tra1_predi	tumor rejection antigen gp96 (predicte	1.764149	0.000749	0.012444
1393388_at	301081	Zdhhc3	zinc finger, DHHC domain containing 3	1.629622	0.000749	0.012444
1388452_at	499428	NA	NA	1.847105	0.00075	0.012445
1370062_at	140937	Hig1	hypoxia induced gene 1	-3.264476	0.000752	0.012452
1369959_at	29344	Zfp36l1	zinc finger protein 36, C3H type-like 1	-2.320843	0.000754	0.012452
1367807_at	116552	Plod1	procollagen-lysine, 2-oxoglutarate 5-di	2.04984	0.000755	0.012452
1387031_at	117030	Erp29	endoplasmic reticulum protein 29	1.486899	0.000755	0.012452
1371465_at	60465	Cttn	cortactin	1.528209	0.000755	0.012452
1373147_at	306129	Fbxl3	F-box and leucine-rich repeat protein 3	-1.798134	0.000758	0.012483
1383309_at	304023	St3gal6	ST3 beta-galactoside alpha-2,3-sialyltr	-3.05155	0.000763	0.012525
1385236_at	310660	NA	NA	1.84546	0.000763	0.012525
1372478_at	501065	LOC50106	NA	1.682695	0.000764	0.012525
1384792_at	295607	Prpf40a_pr	pre-mRNA processing factor 40 homol	1.698816	0.000765	0.012528
1383181_at	364240	Dnajc9_pre	DnaJ (Hsp40) homolog, subfamily C, r	1.398138	0.000768	0.012528
1368566_a_at	64539	Ndufv3l	NADH dehydrogenase (ubiquinone) fla	1.333119	0.000768	0.012528
1383690_at	366473	LOC36647	similar to ornithine decarboxylase-like	2.973184	0.00077	0.012528
1389403_at	85272	Bmp7	bone morphogenetic protein 7	-1.807893	0.00077	0.012528
1388325_at	299159	Atp6v1d	ATPase, H+ transporting, V1 subunit D	1.362363	0.000771	0.012528
1369939_at	25309	Cycs	cytochrome c, somatic	1.587988	0.000771	0.012528
1383435_at	245956	Scn3b	sodium channel, voltage-gated, type II	-1.461366	0.000771	0.012528
1387777_at	170922	Ilk	integrin linked kinase	1.761998	0.000773	0.012531
1375715_at	259218	Rwdd1	RWD domain containing 1	4.748688	0.000773	0.012531
1384172_at	314169	RGD13104	similar to KIAA0423 (predicted)	-1.958913	0.000776	0.012565
1385248_a_at	291015	Ogn_predi	osteoglycin (predicted)	-8.53656	0.000782	0.012644
1387851_at	63852	Pter	phosphotriesterase related	4.586481	0.000784	0.012653
1368826_at	24267	Comt	catechol-O-methyltransferase	2.099575	0.000786	0.012653
1367585_a_at	24211	Atp1a1	ATPase, Na+/K+ transporting, alpha 1	1.5724	0.000787	0.012653
1390478_at	252936	Orc4	origin recognition complex, subunit 4	-1.611778	0.000787	0.012653
1388989_at	361923	Rpl22l1_pr	ribosomal protein L22 like 1 (predicted	-1.543669	0.000788	0.012653
1382370_at	117524	Ccnf	cyclin F	1.96064	0.000789	0.012653
1388589_at	362831	Dot1l_pred	DOT1-like, histone H3 methyltransfera	-1.897169	0.000792	0.012692
1367670_at	24368	Fh1	fumarate hydratase 1	1.757124	0.000793	0.012692
1373991_at	29719	Kcnj16	potassium inwardly-rectifying channel,	4.95925	0.000797	0.012738
1398989_at	304092	Son	Son cell proliferation protein	-1.335114	0.000798	0.012741
1386152_at	365564	Atoh7_pre	atonal homolog 7 (Drosophila) (predict	-2.848918	0.000801	0.01275
1368300_at	25369	Adora2a	adenosine A2a receptor	-2.783827	0.000801	0.01275
1370333_a_at	24482	Igf1	insulin-like growth factor 1	-3.862674	0.000801	0.01275

1370176_at	171086	Trak2	trafficking protein, kinesin binding 2	2.432762	0.000803	0.012756
1368470_at	25455	Ggh	gamma-glutamyl hydrolase	-1.860107	0.000804	0.012756
1370879_at	299201	Dlst	dihydrolipoamide S-succinyltransferase	2.01792	0.000806	0.012756
1370560_at	286996	Hit39	zinc finger protein HIT-39	-2.088061	0.000807	0.012756
1376440_at	315000	Rnf139_pre	ring finger protein 139 (predicted)	-1.413844	0.000807	0.012756
1370973_at	64155	Scn7a	sodium channel, voltage-gated, type V	-3.875669	0.000807	0.012756
1368525_at	79032	Mrs2l	MRS2-like, magnesium homeostasis fa	1.33642	0.000809	0.01276
1370305_at	171441	Yif1	Yip1 interacting factor homolog (S. cer	1.988763	0.00081	0.01276
1389715_at	502643	Ypel4	yippee-like 4	-2.999276	0.000811	0.01276
1396494_at	89830	Ptch1	patched homolog 1 (Drosophila)	4.711345	0.000813	0.01276
1390162_at	303915	Iqcb1_prec	IQ calmodulin-binding motif containing	-2.441068	0.000814	0.01276
1373956_at	363442	Fundc1	FUN14 domain containing 1	-2.280529	0.000815	0.01276
1388037_at	29599	Atp2b3	ATPase, Ca++ transporting, plasma m	1.60495	0.000815	0.01276
1370707_at	246271	Fev	FEV (ETS oncogene family)	-2.451543	0.000817	0.01276
1373983_at	360807	LOC36080	LOC360807	-1.688861	0.000818	0.01276
1384308_at	305535	Meis1_pre	Meis1, myeloid ecotropic viral integrati	-1.34931	0.000818	0.01276
1388985_at	310926	LOC31092	hypothetical protein LOC310926	2.979724	0.000818	0.01276
1382596_a_at	362711	RGD13113	similar to hypothetical protein FLJ2025	1.336852	0.000821	0.012783
1374058_at	246301	Ascl3	achaete-scute complex homolog-like 3	1.403003	0.000822	0.012787
1386689_at	304669	Hook2	hook homolog 2 (Drosophila)	1.658846	0.000825	0.012802
1387081_at	29218	Rcn2	reticulocalbin 2	-1.401604	0.000825	0.012802
1379457_at	299730	Nedd1_pre	neural precursor cell expressed, devel	2.245629	0.000827	0.012812
1371456_at	287982	Abcf3	ATP-binding cassette, sub-family F (G	1.307535	0.000829	0.012838
1371414_at	296654	Gsn	gelsolin	-2.706578	0.00083	0.012838
1388171_at	171150	Cdk7	cyclin-dependent kinase 7 (homolog of	1.61261	0.000831	0.012838
1372020_at	361370	Tom1	target of myb1 homolog (chicken)	1.957062	0.000833	0.012848
1377267_at	317232	Magee1_pr	melanoma antigen, family E, 1 (predict	-1.898903	0.000836	0.012871
1397223_at	500824	RGD15656	similar to FYVE, RhoGEF and PH dom	2.54625	0.000836	0.012871
1373106_at	298765	Zfp36l2	zinc finger protein 36, C3H type-like 2	-2.652164	0.000839	0.012898
1380186_at	305911	RGD13063	similar to Zinc finger protein 198 (Fuse	-1.9945	0.000841	0.01292
1396052_at	287727	Dhx8	DEAH (Asp-Glu-Ala-His) box polypepti	1.389901	0.000842	0.01292
1388336_at	362164	RGD13106	similar to hypothetical protein D2Ertd3	1.475707	0.000845	0.01294
1383960_at	311203	Pex16	peroxisome biogenesis factor 16	1.644018	0.000847	0.012955
1385504_at	282844	Rffl	ring finger and FYVE like domain conta	1.963646	0.000849	0.012985
1376569_at	306330	Klf2_predic	Kruppel-like factor 2 (lung) (predicted)	-6.680279	0.000854	0.013023
1383673_at	317247	Nap1l2	nucleosome assembly protein 1-like 2	-1.871462	0.000855	0.013023
1378170_at	303132	Aff4_predic	AF4/FMR2 family, member 4 (predicte	1.992317	0.000856	0.013023
1384408_at	293015	Map3k5_pr	mitogen-activated protein kinase kinas	-1.753055	0.000856	0.013023
1384295_at	307133	Hspa14	heat shock 70kDa protein 14	-1.717329	0.000857	0.013023
1372070_at	290644	Ifi30	interferon gamma inducible protein 30	1.605345	0.000861	0.013039
1387947_at	54264	Mafb	v-maf musculoaponeurotic fibrosarcom	-3.312751	0.000862	0.013039
1374612_at	307745	Papd5_pre	PAP associated domain containing 5 (	-1.338151	0.000863	0.013039
1392610_at	362501	Topors_pre	topoisomerase I binding, arginine/serir	-1.560347	0.000864	0.013039
1374692_at	315871	Snx14_pre	sorting nexin 14 (predicted)	-1.343706	0.000865	0.013039
1385189_at	311374	RGD13066	similar to cDNA sequence BC019755 (	-3.950026	0.000865	0.013039
1389025_at	309638	Taf11	TAF11 RNA polymerase II, TATA box	-1.81296	0.000867	0.013039
1368888_a_at	83765	Rtn4	reticulon 4	-1.322506	0.000867	0.013039
1382153_at	474143	Clecsf6	C-type (calcium dependent, carbohydr	-2.02051	0.000868	0.013039
1372327_at	362207	Myef2	myelin basic protein expression factor	-2.321515	0.000868	0.013039
1370585_a_at	25023	Prkcb1	protein kinase C, beta 1	-2.746181	0.000871	0.013043
1373329_at	156435	Tmprss2	transmembrane protease, serine 2	2.472442	0.000871	0.013043

1376177_at	497983	RGD1561C	similar to RIKEN cDNA 5730593F17 (p	-1.343415	0.000872	0.013043
1373968_at	364686	RGD15619	similar to KB07 protein (predicted)	-1.541725	0.000872	0.013043
1368541_at	114511	Emb	embigin	1.342332	0.000873	0.013044
1367835_at	246333	Pcsk1n	proprotein convertase subtilisin/kexin t	-1.92503	0.000874	0.013044
1398362_at	29492	Notch2	notch gene homolog 2 (Drosophila)	-1.90402	0.000876	0.013044
1367833_at	81827	Psmc5	peptidase (prosome, macropain) 26S s	1.435317	0.000876	0.013044
1370007_at	116598	Pdia4	protein disulfide isomerase associated	2.042663	0.000878	0.013044
1371913_at	116487	Tgfbi	transforming growth factor, beta induce	-2.397506	0.000881	0.013044
1388436_at	292729	Snrpa	small nuclear ribonucleoprotein polype	2.475371	0.000881	0.013044
1368181_at	64300	Mthfd1	methylenetetrahydrofolate dehydroger	1.409277	0.000881	0.013044
1367986_at	29602	Ptgfrn	prostaglandin F2 receptor negative reg	-1.771165	0.000882	0.013044
1398806_at	29525	Pitpna	phosphatidylinositol transfer protein, al	1.538674	0.000882	0.013044
1373734_at	140915	Slco3a1	solute carrier organic anion transporter	1.351242	0.000883	0.013048
1393221_at	498789	RGD15648	similar to 20-alpha-hydroxysteroid deh	-4.015545	0.000885	0.013061
1387048_at	89827	Ddx39	DEAD (Asp-Glu-Ala-Asp) box polypept	1.457954	0.000886	0.013061
1370442_at	286978	Tmsbl1	thymosin beta-like protein 1	-1.639802	0.000893	0.013142
1377834_at	307042	Epc1_pred	enhancer of polycomb homolog 1 (Dro	-1.34405	0.000893	0.013142
1370184_at	29271	Cfl1	cofilin 1, non-muscle	1.281571	0.0009	0.013215
1399020_at	362012	LOC36201	similar to family with sequence similari	1.412857	0.000901	0.013215
1369935_at	25193	Ccnd3	cyclin D3	1.779033	0.000902	0.013215
1381605_at	310306	Usp13_pre	ubiquitin specific protease 13 (isopepti	1.570959	0.000903	0.013215
1385167_at	363194	Xpo5_pred	exportin 5 (predicted)	1.829979	0.000904	0.013215
1372421_at	290923	Aga	aspartylglucosaminidase	-1.48395	0.000904	0.013215
1369489_at	29427	Aif1	allograft inflammatory factor 1	2.057886	0.00091	0.013268
1388893_at	306253	Glt8d1	glycosyltransferase 8 domain containir	-1.423398	0.00091	0.013268
1388896_at	310960	Usp33	ubiquitin specific peptidase 33	-1.446028	0.000912	0.013268
1382351_at	297902	Gem_predi	GTP binding protein (gene overexpres	-1.407559	0.000913	0.013268
1389400_at	690911	LOC69091	NA	-1.579367	0.000913	0.013268
1371403_at	295230	Cct3	chaperonin subunit 3 (gamma)	1.433226	0.000914	0.013271
1380385_at	500063	NA	NA	-1.727385	0.000921	0.01335
1398521_at	114246	Trpv6	transient receptor potential cation char	-1.301774	0.000922	0.013361
1399111_at	363137	Cyb561d2	cytochrome b-561 domain containing 2	2.972478	0.000925	0.013389
1391371_at	29281	Prodh	proline dehydrogenase	-3.335074	0.000927	0.0134
1374475_at	313917	Abhd1	abhydrolase domain containing 1	2.604881	0.000932	0.013435
1374461_at	304291	Zdhhc4	zinc finger, DHHC domain containing 4	1.261063	0.000933	0.013435
1386899_at	25425	Ctsh	cathepsin H	-4.473512	0.000935	0.013435
1368964_at	81514	Lrrn3	leucine rich repeat protein 3, neuronal	-4.604045	0.000935	0.013435
1398783_at	117039	Gps1	G protein pathway suppressor 1	1.512921	0.000936	0.013435
1395426_at	499945	RGD15622	similar to ubiquitin-conjugating enzyme	1.345172	0.000937	0.013435
1370264_at	499010	CPG2	CPG2 protein	-1.683234	0.000937	0.013435
1378079_at	312077	Golga3_pre	golgi autoantigen, golgin subfamily a, 3	1.651559	0.000938	0.013435
1374716_at	360895	RGD13061	similar to RIKEN cDNA 2810430M08	2.147268	0.000942	0.013481
1368953_at	171129	Ugcgl1	UDP-glucose ceramide glucosyltransfe	3.620952	0.000947	0.013544
1371734_at	298982	Maea	macrophage erythroblast attacher	1.267421	0.000955	0.013595
1367795_at	29596	Ifrd1	interferon-related developmental regul	-1.641717	0.000957	0.013595
1373071_at	292787	RGD13089	similar to RIKEN cDNA 1810054G18 (	1.513384	0.000957	0.013595
1379312_at	294007	Pprc1_prec	peroxisome proliferative activated rece	1.628852	0.000958	0.013595
1377594_at	85385	Shc1	src homology 2 domain-containing trar	1.580076	0.000958	0.013595
1394086_at	288167	Senp7_pre	SUMO1/sentrin specific protease 7 (pr	-2.16608	0.000959	0.013595
1370209_at	117560	Klf9	Kruppel-like factor 9	-2.311843	0.000959	0.013595
1386877_at	65046	Ap2s1	adaptor-related protein complex 2, sigi	1.448546	0.000959	0.013595

1384805_at	641632	Defb24	beta-defensin 24	1.88739	0.000962	0.013609
1371454_at	287477	Tmem93_p	transmembrane protein 93 (predicted)	1.465955	0.000962	0.013609
1370721_a_at	83686	Cngb1	cyclic nucleotide gated channel beta 1	1.556553	0.000966	0.01365
1369129_at	29434	Rasgrp1	RAS guanyl releasing protein 1	2.872173	0.000967	0.01365
1389140_at	293142	Spcs2_pre	signal peptidase complex subunit 2 ho	1.533069	0.00097	0.013673
1394435_at	295328	Vangl1_pre	vang, van gogh-like 1 (Drosophila) (pre	2.970018	0.000973	0.013693
1398249_at	117035	Slc25a20	solute carrier family 25 (mitochondrial	1.576449	0.000973	0.013693
1390447_at	81802	Stx3	syntaxin 3	1.520449	0.000975	0.013696
1385702_at	304988	Mnda	myeloid cell nuclear differentiation anti	-4.805424	0.000977	0.01371
1398247_at	170916	Prss15	protease, serine, 15	1.788485	0.000979	0.013733
1377300_at	498003	RGD15600	similar to Dual specificity protein phos	2.534309	0.000983	0.013766
1393220_at	315949	Armc8_pre	armadillo repeat containing 8 (predicte	-1.963132	0.000984	0.013766
1387120_at	29677	Psmc3	proteasome (prosome, macropain) 26S	1.608615	0.000986	0.013775
1368358_a_at	94202	Ptprr	protein tyrosine phosphatase, receptor	-3.305751	0.000989	0.013775
1371769_at	65168	Scamp2	secretory carrier membrane protein 2	1.714903	0.000989	0.013775
1374196_at	114515	Lancl1	lanC (bacterial lantibiotic synthetase co	1.774642	0.000991	0.013775
1379101_at	310461	Dhx36_pre	DEAH (Asp-Glu-Ala-His) box polypepti	1.378398	0.000991	0.013775
1373790_at	361998	Car14_pre	carbonic anhydrase 14 (predicted)	1.74175	0.000992	0.013775
1377572_at	361091	RGD15635	similar to UDP-glucose ceramide gluco	-1.912351	0.000992	0.013775
1386950_at	25594	Ppp1cb	protein phosphatase 1, catalytic subun	-1.548495	0.000993	0.013775
1398316_at	288913	LOC28891	similar to LEYDIG CELL TUMOR 10 K	1.500749	0.001001	0.01387
1369993_at	171140	Camk2g	calcium/calmodulin-dependent protein	1.50921	0.001006	0.013931
1378049_at	297879	RGD13064	similar to Cas-associated zinc finger p	-1.811644	0.001008	0.013934
1367993_at	65201	Rsn	restin (Reed-Steinberg cell-expressed	2.146383	0.001008	0.013934
1373636_at	306759	Spock1	sparc/osteonectin, cwcv and kazal-like	4.951904	0.001012	0.013968
1377731_at	289810	RGD15606	similar to small unique nuclear recepto	-1.386974	0.001018	0.014035
1389261_at	303275	RGD13055	similar to hypothetical protein MGC232	1.659193	0.001019	0.014035
1371442_at	192235	Hyou1	hypoxia up-regulated 1	1.984122	0.00102	0.014035
1393034_at	293112	RGD15655	similar to tyrosine kinase-associated le	-1.956644	0.001021	0.014039
1387087_at	24253	Cebpb	CCAAT/enhancer binding protein (C/E	-1.389934	0.001025	0.014067
1386865_at	25434	Sparcl1	SPARC-like 1 (mast9, hevin)	-3.601811	0.001025	0.014067
1369177_at	114554	Pi4k2a	phosphatidylinositol 4-kinase type 2 al	1.588305	0.001029	0.014094
1383321_at	288617	Tpst1	tyrosylprotein sulfotransferase 1	-2.987107	0.00103	0.014094
1374818_at	59315	Nxph3	neurexophilin 3	-3.080403	0.001032	0.014114
1393713_at	291095	Gmds	GDP-mannose 4, 6-dehydratase	1.254174	0.001039	0.014166
1372720_at	293060	Btbd1	BTB (POZ) domain containing 1	-1.409524	0.00104	0.014166
1367505_at	305127	Zfp644_pre	zinc finger protein 644 (predicted)	-2.039657	0.001042	0.014166
1367844_at	81664	Gnai2	guanine nucleotide binding protein, alp	1.535384	0.001042	0.014166
1390311_at	171572	Ttl	tubulin tyrosine ligase	1.372875	0.001042	0.014166
1372420_at	290645	RGD13080	similar to FKSG24 (predicted)	1.666893	0.001043	0.014166
1367790_at	64635	Snd1	staphylococcal nuclease domain conta	1.61825	0.001044	0.014166
1392908_at	595134	LOC59513	NA	-1.511888	0.001047	0.014166
1390439_at	299864	Ebag9	estrogen receptor-binding fragment-as	-1.398244	0.001047	0.014166
1375845_at	292486	RGD15629	similar to Aig1 protein (predicted)	1.272149	0.001048	0.014166
1371662_at	292028	Kars	lysyl-tRNA synthetase	1.485644	0.001048	0.014166
1368450_at	25017	Myo5a	myosin Va	2.892287	0.00105	0.014166
1367693_at	25576	Ywhah	tyrosine 3-monooxygenase/tryptophan	-1.456693	0.00105	0.014166
1383695_at	24875	Vipr1	vasoactive intestinal peptide receptor 1	-3.06265	0.001056	0.014209
1388619_at	315655	Rdx	radixin	-1.298888	0.001057	0.014209
1395299_at	287675	Ka24	type I keratin KA24	1.706242	0.001059	0.014209
1368662_at	171387	Rnf39	ring finger protein 39	-3.192391	0.001059	0.014209

1375221_at	296182	Txndc13	thioredoxin domain containing 13	-1.396324	0.00106	0.014209
1372767_at	501123	NA	NA	-1.457376	0.00106	0.014209
1371692_at	295264	Mllt11	myeloid/lymphoid or mixed-lineage leu	1.226605	0.00106	0.014209
1389844_at	260321	Fkbp4	FK506 binding protein 4	1.954398	0.001062	0.014221
1367878_at	65134	Stx5a	syntaxin 5a	1.529153	0.001065	0.014223
1383152_at	293628	RGD1308S	similar to Cc1-9	1.774332	0.001066	0.014223
1398350_at	64160	Basp1	brain abundant, membrane attached s	-1.979918	0.00107	0.014223
1370061_at	81755	Rab3b	RAB3B, member RAS oncogene famil	-1.529951	0.00107	0.014223
1376788_at	306722	Dapk1_pre	death associated protein kinase 1 (pre	-1.556518	0.001071	0.014223
1388440_at	300802	Aph1b	anterior pharynx defective 1b homolog	1.47277	0.001072	0.014223
1373915_at	308405	Dmpk_pre	dystrophia myotonica-protein kinase (p	1.329766	0.001073	0.014223
1388753_at	311642	Sulf2	sulfatase 2	-1.570965	0.001073	0.014223
1377141_at	498685	LOC49868	NA	-1.589874	0.001074	0.014223
1373907_at	367073	Trappc4	trafficking protein particle complex 4	1.681003	0.001075	0.014223
1392204_at	498945	LOC49894	similar to NK10	-3.719282	0.001075	0.014223
1395589_at	310808	Slc35a3	solute carrier family 35 (UDP-N-acetyl	1.548106	0.001076	0.014223
1383826_at	303754	Rab40b_pr	Rab40b, member RAS oncogene fami	-1.774608	0.001078	0.014236
1371302_at	502663	LOC50266	NA	1.28927	0.001079	0.014236
1374100_at	500243	RGD1560S	similar to 2300003P22Rik protein (pre	-1.306399	0.00108	0.014236
1376725_at	312781	Lrp6_predi	low density lipoprotein receptor-relatec	2.268331	0.001081	0.014236
1370934_at	25281	Nup153	nucleoporin 153	-1.711627	0.001082	0.014236
1387112_at	24943	Plp	proteolipid protein	-4.016713	0.001084	0.014245
1374846_at	498008	Clp1	cardiac lineage protein 1	-1.294519	0.001089	0.014254
1399141_at	287269	Clk4	CDC like kinase 4	-1.607509	0.001089	0.014254
1376843_at	140590	Bmpr2	bone morphogenic protein receptor, ty	2.42485	0.001091	0.014254
1398925_at	309656	RGD13078	similar to RIKEN cDNA 1300018I05	1.716908	0.001092	0.014254
1371444_at	310667	Lass2	longevity assurance homolog 2 (S. cer	1.458854	0.001093	0.014254
1374956_at	81740	Pcm1	pericentriolar material 1	-1.555008	0.001093	0.014254
1389716_at	501614	NA	NA	-1.553366	0.001097	0.014254
1371389_at	306766	LOC30676	hypothetical LOC306766	-1.550514	0.001097	0.014254
1375739_at	192204	Ehd4	EH-domain containing 4	1.749739	0.001098	0.014254
1373210_at	298941	Lamb1_pre	laminin, beta 1 (predicted)	-3.786432	0.001099	0.014254
1371729_at	298792	Ypel5	yippee-like 5 (Drosophila)	-1.395304	0.001099	0.014254
1376311_at	295382	RGD15634	similar to netrin G1 (predicted)	-2.018599	0.001099	0.014254
1370323_at	64517	Thop1	thimet oligopeptidase 1	2.178069	0.0011	0.014254
1367799_at	24799	Eef1a2	eukaryotic translation elongation factor	-1.951268	0.001101	0.014254
1398791_at	58819	Txnrd1	thioredoxin reductase 1	1.477917	0.001101	0.014254
1372867_at	291534	Rnmt	RNA (guanine-7-) methyltransferase	-1.321633	0.001103	0.014254
1398938_at	24161	Acp1	acid phosphatase 1, soluble	1.35606	0.001104	0.014254
1394414_at	362699	Yipf4	Yip1 domain family, member 4	-1.447668	0.001104	0.014254
1392847_at	314028	Cbll1_pred	Casitas B-lineage lymphoma-like 1 (pr	-1.406094	0.001106	0.014266
1371555_at	363478	Snx12_pre	sorting nexin 12 (predicted)	1.567899	0.001108	0.01427
1374646_at	113927	Csnk1a1	casein kinase 1, alpha 1	-1.260186	0.001108	0.01427
1375915_at	300862	Irak1bp1_p	interleukin-1 receptor-associated kinas	-2.007792	0.001111	0.014273
1370718_at	60567	Syt10	synaptotagmin X	1.680098	0.001112	0.014273
1368372_at	24800	Sts	steroid sulfatase	1.688994	0.001114	0.014273
1399058_at	292244	Mrpl18_pre	mitochondrial ribosomal protein L18 (p	1.299993	0.001114	0.014273
1383638_at	313434	RGD15663	similar to DNA segment on chromosom	-1.704551	0.001115	0.014273
1382891_at	302566	RGD15646	similar to ubiquitin specific protease 27	-1.915936	0.001115	0.014273
1398810_at	64527	Pdap1	PDGFA associated protein 1	1.426211	0.001117	0.014273
1370248_at	63847	Fxyd6	FXYD domain-containing ion transport	-2.978062	0.001118	0.014273

1368969_at	80722	Sost	sclerostin	3.07406	0.001118	0.014273
1398473_at	293938	Bloc1s2	biogenesis of lysosome-related organe	1.433901	0.00112	0.014273
1388392_at	360564	Tax1bp3	Tax1 (human T-cell leukemia virus typ	1.822092	0.001121	0.014273
1375052_at	501015	LOC50101	NA	2.956903	0.001121	0.014273
1390846_at	366474	Col16a1	procollagen, type XVI, alpha 1	1.303501	0.001123	0.014283
1389523_at	287119	Zfp598_pre	zinc finger protein 598 (predicted)	1.626588	0.001124	0.014283
1393918_at	360851	RGD1566C	similar to novel protein (predicted)	-2.982607	0.001132	0.014343
1386604_at	114202	Mpp3	membrane protein, palmitoylated 3 (M/	-2.705684	0.001132	0.014343
1372254_at	295703	Serping1	serine (or cysteine) peptidase inhibitor	-2.590193	0.001136	0.014343
1389344_at	297336	Usp39_pre	ubiquitin specific protease 39 (predicte	1.435571	0.001136	0.014343
1374647_at	310749	Rsbn1_pre	rosbin, round spermatid basic protein 1	-1.994121	0.001136	0.014343
1388868_at	293960	Zfp216_pre	zinc finger protein 216 (predicted)	-1.740287	0.001137	0.014343
1368801_at	83824	Cxxc4	CXXC finger 4	2.030842	0.001139	0.014343
1392051_at	362212	RGD13079	similar to hypothetical protein FLJ1468	2.114337	0.001139	0.014343
1386994_at	29619	Btg2	B-cell translocation gene 2, anti-prolife	-1.91947	0.001141	0.014343
1384110_at	366608	RGD15617	similar to mKIAA0716 protein (predicte	-2.29685	0.001141	0.014343
1398824_at	65165	Tmed2	transmembrane emp24 domain traffick	2.433233	0.001142	0.014343
1372207_at	291691	Brd8	bromodomain containing 8	-1.833449	0.001142	0.014343
1395836_at	117242	Odz2	odd Oz/ten-m homolog 2 (Drosophila)	-2.220671	0.001147	0.01439
1393418_at	58814	Tmod2	tropomodulin 2	1.581554	0.001149	0.014396
1398972_at	287367	Cops3	COP9 (constitutive photomorphogenic	1.318267	0.001149	0.014396
1373534_at	297942	RGD13073	similar to SR rich protein	-1.410629	0.001151	0.014407
1369118_a_at	81668	Gnrhr	gonadotropin releasing hormone recep	2.287531	0.001156	0.014441
1369451_a_at	24521	Kcnj1	potassium inwardly-rectifying channel,	-4.034459	0.001158	0.014441
1369457_a_at	140594	Sytl4	synaptotagmin-like 4	6.791134	0.00116	0.014441
1368326_at	29702	Eif2ak3	eukaryotic translation initiation factor 2	-1.922556	0.001161	0.014441
1381874_at	499593	RGD15656	similar to SOX2 protein (predicted)	3.136994	0.001161	0.014441
1374423_at	295398	Hiat1_pred	hippocampus abundant gene transcrip	-1.344412	0.001163	0.014441
1372724_at	266668	Grina	glutamate receptor, ionotropic, N-meth	1.816352	0.001165	0.014441
1391840_at	316238	RGD13106	similar to RIKEN cDNA 1700027N10 (|	-1.570017	0.001165	0.014441
1398868_at	252928	Timm13	translocase of inner mitochondrial mer	1.553002	0.001167	0.014441
1380815_at	304923	LOC30492	similar to hypothetical protein FLJ1175	-1.28218	0.001167	0.014441
1372260_at	287061	MGC11614	leucine zipper domain protein	1.515705	0.001167	0.014441
1367603_at	24849	Tpi1	triosephosphate isomerase 1	3.146302	0.001168	0.014441
1382082_at	25336	Nfyb	nuclear transcription factor-Y beta	-1.57293	0.001168	0.014441
1367551_a_at	360768	RGD13064	similar to CG14980-PB	-1.218451	0.001173	0.014474
1374065_at	24553	Met	met proto-oncogene	-5.753732	0.001173	0.014474
1372059_at	288176	RGD13094	similar to RIKEN cDNA 2610528E23	-1.50823	0.001174	0.014475
1390954_at	314897	Ppm1h	protein phosphatase 1H (PP2C domai	1.515448	0.001175	0.014475
1392791_at	25148	Egr3	early growth response 3	-3.753242	0.001185	0.014577
1388823_at	288779	Rab5b_pre	RAB5B, member RAS oncogene famil	1.34362	0.001189	0.01461
1374396_at	299971	Atp6v1c1	ATPase, H+ transporting, V1 subunit C	1.372313	0.00119	0.01461
1370888_at	252934	Cox5a	cytochrome c oxidase, subunit Va	1.345606	0.001192	0.01461
1373585_at	57022	Adam4	a disintegrin and metalloprotease dom	1.504608	0.001194	0.01461
1376497_a_at	305845	RGD15598	similar to FLJ20859 protein (predicted)	1.579598	0.001195	0.01461
1372938_at	303518	RGD13047	similar to RIKEN cDNA 6330509G02	-1.509252	0.001196	0.01461
1377616_at	297903	RGD13106	similar to RIKEN cDNA 6720467C03 (|	-2.3555	0.001196	0.01461
1397356_at	311193	Arhgap1_p	Rho GTPase activating protein 1 (pred	-1.699856	0.001196	0.01461
1379309_at	365755	Tbl1xr1_pr	transducin (beta)-like 1X-linked recept	2.124625	0.001199	0.014623
1371488_at	499125	RGD15620	RGD1562079 (predicted)	1.711861	0.0012	0.014623
1367784_a_at	24854	Clu	clusterin	-1.61567	0.001207	0.014699

1367468_at	362252	Scand1_pr	SCAN domain-containing 1 (predicted)	2.061575	0.001208	0.014699
1372446_at	192361	Ppp1r2	protein phosphatase 1, regulatory (inhi	-1.200534	0.001211	0.014724
1382630_at	292139	RGD13115	similar to 4930506M07Rik protein (pre	1.693414	0.001213	0.014737
1397611_at	501124	NA	NA	1.628944	0.001218	0.014749
1367675_at	81823	Cib1	calcium and integrin binding 1 (calmyri	1.947808	0.001218	0.014749
1398883_at	499894	LOC49989	NA	-1.434042	0.001218	0.014749
1372230_at	292792	RGD13047	similar to seven transmembrane doma	1.324346	0.00122	0.014749
1388002_at	286993	Taok1	TAO kinase 1	1.275145	0.001221	0.014749
1367724_a_at	94170	Atp6v0e1	ATPase, H+ transporting, V0 subunit E	1.359567	0.001221	0.014749
1383227_at	298658	NA	NA	1.454044	0.001223	0.014749
1368010_at	116689	Ptpn6	protein tyrosine phosphatase, non-rece	1.415839	0.001224	0.014749
1383165_at	362019	RGD13102	similar to KIAA1324 protein (predicted)	-1.468508	0.001224	0.014749
1368878_at	89784	Idi1	isopentenyl-diphosphate delta isomera	-1.420368	0.00123	0.014799
1399094_at	299154	Churc1_pre	churchill domain containing 1 (predicte	-1.224913	0.001233	0.014808
1371835_at	293508	Prkacb_pre	protein kinase, cAMP dependent, cata	-1.801223	0.001233	0.014808
1369665_a_at	29197	Il18	interleukin 18	2.371702	0.001234	0.014808
1380045_at	246311	Pdp2	pyruvate dehydrogenase phosphatase	1.434505	0.001237	0.014812
1387654_at	65261	Myo1c	myosin IC	3.040485	0.001237	0.014812
1375084_at	313057	Serinc2	serine incorporator 2	2.009713	0.001237	0.014812
1367913_at	170520	Cygb	cytoglobin	-2.412403	0.001239	0.014816
1370282_at	29317	Csrp2	cysteine and glycine-rich protein 2	-1.882477	0.001241	0.014827
1369590_a_at	29467	Ddit3	DNA-damage inducible transcript 3	-1.786266	0.001246	0.014873
1388514_at	259229	Ppm1g	protein phosphatase 1G (formerly 2C),	1.486559	0.001247	0.014873
1371685_at	312372	Kbtbd2_pre	kelch repeat and BTB (POZ) domain c	-1.286493	0.001248	0.014873
1381193_at	317456	LOC31745	hypothetical LOC317456	1.790365	0.001251	0.014897
1373576_at	287249	RGD13107	similar to CCR4	-1.359149	0.001253	0.014897
1387837_at	24205	Apc	adenomatosis polyposis coli	1.766183	0.001254	0.014897
1368430_at	63865	Lgmn	legumain	1.863487	0.001255	0.014897
1390858_at	286761	Vcpip1	valosin containing protein (p97)/p47 cc	2.073955	0.001256	0.014897
1388521_at	361755	Pycs_predi	pyrroline-5-carboxylate synthetase (glu	1.437262	0.001257	0.014897
1391885_at	294594	Spata9_pre	spermatogenesis associated 9 (predict	-1.443956	0.001259	0.0149
1398762_at	83841	Sdcbp	syndecan binding protein	-1.432871	0.001259	0.0149
1368009_at	114711	Gne	glucosamine	1.416573	0.001261	0.0149
1383955_at	364144	RGD13113	similar to 2510002A14Rik protein (pred	-1.352777	0.001263	0.014912
1373467_at	368042	RGD15641	similar to TBP-associated factor 172 (T	-1.227096	0.001266	0.014931
1377926_at	316611	Centg2_pre	centaurin, gamma 2 (predicted)	2.527859	0.001267	0.014931
1386962_at	25031	Plcb4	phospholipase C, beta 4	-1.481232	0.001268	0.014931
1368706_at	116467	Tm4sf4	transmembrane 4 superfamily member	-1.543721	0.001272	0.01494
1368481_at	25024	Gipr	gastric inhibitory polypeptide receptor	-1.368833	0.001273	0.01494
1382797_at	296750	RGD15604	similar to 1500019C06Rik protein (pred	2.111528	0.001273	0.01494
1376775_at	362171	RGD13099	similar to RIKEN cDNA 2600010E01	2.10877	0.001275	0.01494
1367873_at	83615	Atp6ap1	ATPase, H+ transporting, lysosomal ac	1.509878	0.001275	0.01494
1368117_at	64845	Gphn	gephyrin	1.384766	0.001275	0.01494
1394022_at	291023	Id4	inhibitor of DNA binding 4	-2.327835	0.001277	0.01494
1376052_at	292654	RGD15645	similar to hypothetical protein FLJ2051	1.378448	0.001278	0.01494
1372049_at	289753	RGD13118	similar to RIKEN cDNA 5730411O18 g	4.061074	0.001279	0.01494
1383895_at	361415	Dynlrb2_pr	dynein light chain roadblock-type 2 (pr	-3.358672	0.001281	0.01494
1369278_at	81663	Gna12	guanine nucleotide binding protein, alp	2.407965	0.001283	0.01494
1371040_at	292657	Slc1a5	solute carrier family 1 (neutral amino a	-1.192398	0.001283	0.01494
1371535_at	302915	Pmm2_pre	phosphomannomutase 2 (predicted)	1.687041	0.001284	0.01494
1378740_at	304893	Rasal2_pre	RAS protein activator like 2 (predicted)	1.36228	0.001284	0.01494

1371410_at	288925	RGD1564C	similar to cDNA sequence BC056474 (	1.423886	0.001286	0.014949
1377534_at	365381	Stk32c_pre	serine/threonine kinase 32C (predictec	-2.339233	0.001287	0.014949
1380962_at	302668	Ace2	angiotensin I converting enzyme (pept	-5.967893	0.001289	0.014949
1379914_at	313994	Tcfcp2l2	transcription factor CP2-like 2	-2.948625	0.001292	0.014949
1373660_at	361436	Afg3l1_pre	AFG3(ATPase family gene 3)-like 1 (y	1.392623	0.001294	0.014949
1383296_a_at	290280	Xpo4_pred	exportin 4 (predicted)	1.783317	0.001294	0.014949
1381164_at	311919	Golga1_pre	golgi autoantigen, golgin subfamily a, 1	1.467895	0.001296	0.014949
1373662_at	362112	Tor2a	torsin family 2, member A	1.787054	0.001296	0.014949
1382041_at	294324	Agpat3_pre	1-acylglycerol-3-phosphate O-acyltran	1.563098	0.001297	0.014949
1374202_at	296599	RGD13115	similar to chromosome 9 open reading	1.51839	0.001298	0.014949
1369981_at	58845	Igbp1	immunoglobulin (CD79A) binding prote	-1.237719	0.001299	0.014949
1371536_at	260416	Carhsp1	calcium regulated heat stable protein 1	2.38858	0.001299	0.014949
1374634_at	315645	RGD13105	similar to RIKEN cDNA 2700059L22 (p	-1.60953	0.001302	0.014959
1397853_s_at	66013	Arhgef9	Cdc42 guanine nucleotide exchange fa	-1.841696	0.001302	0.014959
1389448_at	287606	4-Sep	septin 4	-2.366076	0.001303	0.014959
1370286_at	29642	Slc38a2	solute carrier family 38, member 2	-1.788173	0.001307	0.014992
1378409_at	114503	Pou4f1	POU domain, class 4, transcription fac	2.780293	0.00131	0.015
1368709_at	81919	Fut1	fucosyltransferase 1	3.706626	0.001311	0.015
1398980_at	305851	Supt16h_p	suppressor of Ty 16 homolog (S. cerev	1.424877	0.001312	0.015
1369060_a_at	84578	Hdac3	histone deacetylase 3	1.396092	0.001312	0.015
1384330_at	303067	LOC30306	similar to hypothetical protein FLJ2054	1.514825	0.00132	0.015072
1381976_at	300158	Kif21a_pre	kinesin family member 21A (predicted)	-1.385999	0.001322	0.015073
1377016_at	362978	Creld2	cysteine-rich with EGF-like domains 2	2.095167	0.001322	0.015073
1398531_at	315439	Slc36a4_pı	solute carrier family 36 (proton/amino a	-1.423757	0.001326	0.015109
1374580_at	300860	Senp6_pre	SUMO/sentrin specific peptidase 6 (pr	-1.353631	0.001332	0.015142
1398947_at	298874	Pum2	pumilio 2 (Drosophila)	-1.362154	0.001333	0.015142
1368940_at	29597	P2ry2	purinergic receptor P2Y, G-protein cou	-2.345034	0.001333	0.015142
1371446_at	289014	Mapkapk2	MAP kinase-activated protein kinase 2	2.022728	0.001334	0.015142
1373499_at	81714	Gas5	growth arrest specific 5	-1.278254	0.001335	0.015143
1390027_at	296121	Usp8_pred	ubiquitin specific protease 8 (predicted	1.391512	0.001336	0.015143
1386871_at	29328	Gpx4	glutathione peroxidase 4	1.688038	0.00134	0.015174
1382345_at	314743	Pctk2	PCTAIRE-motif protein kinase 2	-1.863093	0.001343	0.015187
1389323_at	363064	Wdr61	WD repeat domain 61	1.339091	0.001343	0.015187
1381958_at	315969	RGD15629	similar to mKIAA0259 protein (predicte	2.848434	0.001346	0.015191
1382144_at	294963	Mrpl47	mitochondrial ribosomal protein L47	1.347183	0.001346	0.015191
1371967_at	293754	Mrpl16	mitochondrial ribosomal protein L16	1.212548	0.001349	0.015199
1388387_at	362087	Ubadc1	ubiquitin associated domain containing	1.288515	0.00135	0.015199
1385835_at	303505	Arl12_pred	ADP-ribosylation factor-like 12 (predict	1.47362	0.001351	0.015199
1372661_at	287120	Tbl3	transducin (beta)-like 3	1.714856	0.001351	0.015199
1387876_at	25126	Stat5b	signal transducer and activator of trans	1.729367	0.001357	0.015244
1388149_at	24811	Tap1	transporter 1, ATP-binding cassette, su	1.238911	0.00136	0.015253
1393172_at	64824	Nab1	Ngfi-A binding protein 1	1.764302	0.00136	0.015253
1379439_at	363035	Btbd15	BTB (POZ) domain containing 15	-1.562543	0.001361	0.015253
1387017_at	29230	Sqle	squalene epoxidase	-1.313891	0.001363	0.015253
1376347_at	304809	RGD15656	similar to PLU1 (predicted)	1.799535	0.001363	0.015253
1371944_at	363836	Ube2l3_pre	ubiquitin-conjugating enzyme E2L 3 (p	1.224488	0.001367	0.015285
1389008_at	305539	Spred2	sprouty-related, EVH1 domain contain	-1.505061	0.00137	0.015294
1380872_at	366333	RGD15624	similar to hypothetical protein MGC281	-1.972119	0.001372	0.015294
1377668_at	24919	Yy1	YY1 transcription factor	-2.073534	0.001372	0.015294
1371980_at	298682	Atad3a	ATPase family, AAA domain containing	1.738495	0.001373	0.015294
1396947_at	286994	Lgr4	leucine-rich repeat-containing G protei	2.120079	0.001374	0.015294

1387830_at	25419	Crp	C-reactive protein, pentraxin-related	-4.098406	0.001377	0.01531
1370611_at	25243	Arnt2	aryl hydrocarbon receptor nuclear tran	1.291086	0.001378	0.01531
1371539_at	287273	Nola2_pred	nucleolar protein family A, member 2 (	1.246587	0.001379	0.01531
1398175_at	362876	Ppfia2_pre	protein tyrosine phosphatase, receptor	2.689982	0.001384	0.015347
1388071_x_at	24737	RT1-Aw2	RT1 class Ib, locus Aw2	1.704086	0.001385	0.015347
1371489_at	29274	Rnf4	ring finger protein 4	-1.357392	0.001387	0.015347
1369667_at	25218	Vps52	vacuolar protein sorting 52 (yeast)	1.283297	0.001388	0.015347
1368998_at	65193	Nkx6-1	NK6 transcription factor related, locus	3.721278	0.001389	0.015347
1378060_at	315546	Kirrel3_pre	kin of IRRE like 3 (Drosophila) (predict	5.528708	0.001389	0.015347
1371313_at	501709	NA	NA	-1.244365	0.001391	0.015353
1389791_at	306619	Cln8	ceroid-lipofuscinosis, neuronal 8	-1.546415	0.001392	0.015356
1373335_at	302808	Zdhhc9	zinc finger, DHHC domain containing 9	2.158696	0.001393	0.015356
1368022_at	65038	Inppl1	inositol polyphosphate phosphatase-lik	1.312396	0.001395	0.015356
1391037_at	303812	RGD15644	RGD1564491 (predicted)	-1.301034	0.001395	0.015356
1367453_at	114562	Cdc37	cell division cycle 37 homolog (S. cere	1.174712	0.001397	0.015361
1384315_at	296849	Pdk3_map	pyruvate dehydrogenase kinase, isoer	1.411373	0.001399	0.015362
1373988_at	688591	LOC68859	NA	1.594018	0.0014	0.015362
1387908_at	64455	Rasd1	RAS, dexamethasone-induced 1	1.330239	0.001401	0.015362
1368540_at	83684	Tpbg	trophoblast glycoprotein	-3.825994	0.001403	0.015379
1378240_at	312641	Fancd2	Fanconi anemia D2 protein	-2.348494	0.001407	0.015402
1378447_at	303403	Thrap1_pre	thyroid hormone receptor associated p	-2.178943	0.001408	0.015406
1377015_at	361602	Me3_predi	malic enzyme 3, NADP(+)-dependent,	-1.764813	0.001409	0.015408
1371817_at	290651	LOC29065	similar to myo-inositol 1-phosphate syn	1.242491	0.001411	0.015411
1368538_at	64632	Exoc7	exocyst complex component 7	1.621988	0.001419	0.015477
1387940_at	192234	Eif2b5	eukaryotic translation initiation factor 2	2.090595	0.00142	0.015477
1396820_at	297893	Hdac1_pre	histone deacetylase 1 (predicted)	1.570089	0.001421	0.015477
1374433_at	501763	NA	NA	-2.068414	0.001422	0.015477
1383600_at	266998	Slc13a5	solute carrier family 13 (sodium-depen	3.516908	0.001424	0.01548
1394220_at	316012	Klhl18_pre	kelch-like 18 (Drosophila) (predicted)	1.905728	0.001424	0.01548
1370239_at	25632	Hba-a1	hemoglobin alpha, adult chain 1	-2.640688	0.001426	0.015485
1398764_at	79449	Rpl21	ribosomal protein L21	-1.132604	0.001428	0.015492
1375382_at	309595	Mdc1	mediator of DNA damage checkpoint 1	1.886774	0.001431	0.015492
1390647_at	296762	Phtf2_pred	putative homeodomain transcription fa	-1.400766	0.001432	0.015492
1398934_at	308267	Map3k7ip2	mitogen-activated protein kinase kinas	-1.24923	0.001435	0.015492
1372704_at	362040	RGD15607	similar to RIKEN cDNA 2310008M10 (	1.861809	0.001435	0.015492
1375867_at	287441	Zbtb4_pred	zinc finger and BTB domain containing	-1.334339	0.001436	0.015492
1383007_at	300754	Bbs4_pred	Bardet-Biedl syndrome 4 homolog (hu	3.068351	0.001437	0.015492
1379378_at	289469	Mrps18c_p	mitochondrial ribosomal protein S18C	1.347936	0.001437	0.015492
1388385_at	286925	Cryba2	crystallin, beta A2	-2.317991	0.001439	0.015492
1371957_at	316317	Imp4	IMP4, U3 small nucleolar ribonucleopr	1.229669	0.00144	0.015492
1367720_at	25374	Alad	aminolevulinate, delta-, dehydratase	1.872519	0.00144	0.015492
1369085_s_at	113938	Snurf	SNRPN upstream reading frame	1.270558	0.001441	0.015492
1371617_at	365388	Psmd13_p	proteasome (prosome, macropain) 26S	1.221331	0.001444	0.015511
1367918_at	81730	Fez1	fasciculation and elongation protein ze	-2.226106	0.001445	0.015511
1377869_at	171555	Ccrn4l	CCR4 carbon catabolite repression 4-l	-1.586778	0.001446	0.015513
1374326_at	298699	Ppan	peter pan homolog (Drosophila)	1.637345	0.001447	0.015513
1399033_at	361391	Cbfb	core binding factor beta	-1.136101	0.001455	0.015572
1373491_at	295241	LOC29524	similar to Metaxin 1, isoform 2	1.452628	0.001455	0.015572
1370474_at	24831	Thrb	thyroid hormone receptor beta	3.509368	0.001457	0.015576
1370351_at	85425	Tdrd7	tudor domain containing 7	-1.202917	0.001458	0.015576
1389833_at	362409	Sumf1_pre	sulfatase modifying factor 1 (predicted	1.461649	0.001459	0.015576

1367802_at	29517	Sgk	serum/glucocorticoid regulated kinase	-1.903943	0.00146	0.015576
1371117_at	361170	Adam32	a disintegrin and metalloprotease dom	3.343657	0.001465	0.015591
1376779_at	84482	Foxo1a	forkhead box O1A	1.654231	0.001465	0.015591
1377314_at	297417	Gfpt1	glutamine fructose-6-phosphate transa	1.698549	0.001465	0.015591
1368429_at	171152	Taf9l	TAF9-like RNA polymerase II, TATA b	-1.315913	0.001469	0.015623
1380736_at	117269	Rps6ka2	ribosomal protein S6 kinase polypeptic	3.127557	0.001472	0.015641
1387446_at	65044	C1galt1	core 1 UDP-galactose:N-acetylgalacto	1.329328	0.001474	0.015649
1384399_at	501082	NA	NA	1.313182	0.001475	0.015649
1373898_at	360760	LOC36076	NA	-1.269847	0.00148	0.015666
1368796_at	78976	Kiss1r	KISS1 receptor	3.469439	0.001481	0.015666
1398750_at	64202	Calr	calreticulin	1.852075	0.001481	0.015666
1391357_at	500913	RGD1566C	similar to mKIAA1644 protein (predicte	-3.758605	0.001482	0.015666
1372581_at	287645	Snf8	SNF8, ESCRT-II complex subunit, hon	1.50824	0.001483	0.01567
1385921_at	362154	LOC36215	brain zinc finger protein	1.616889	0.00149	0.015725
1388346_at	291963	RGD15609	similar to HSPC171 protein (predicted)	1.555796	0.001495	0.015764
1375869_at	360827	Ulk1_mapp	unc-51-like kinase 1 (mapped)	-1.283528	0.001503	0.01584
1371561_at	303565	G6pc3	glucose 6 phosphatase, catalytic, 3	1.199305	0.001505	0.015847
1371480_at	686524	LOC68652	NA	2.377262	0.001509	0.01587
1373020_at	287065	RGD15644	similar to mitochondria-associated grai	1.182395	0.00151	0.01587
1397756_at	500623	RGD15637	similar to BC068281 protein (predicted	3.59311	0.001511	0.01587
1385251_at	500638	LOC50063	hypothetical protein LOC500638	-3.310099	0.001512	0.015875
1389527_at	378467	LOC37846	promethin	-2.290135	0.001515	0.015897
1389088_at	64622	Adnp	activity-dependent neuroprotective pro	-1.363109	0.001517	0.015898
1394985_at	314764	Eea1_pred	early endosome antigen 1 (predicted)	1.398566	0.001518	0.015898
1394720_at	313507	Tal1_predi	T-cell acute lymphocytic leukemia 1 (p	3.354312	0.001521	0.015917
1379484_at	311299	Aven_pred	apoptosis, caspase activation inhibitor	1.635061	0.001522	0.015919
1376026_at	288257	Donson	downstream neighbor of SON	-1.909331	0.001524	0.015923
1396082_at	315394	Kbtbd3_pre	kelch repeat and BTB (POZ) domain c	1.276276	0.001529	0.015967
1371412_a_at	338475	Nrep	neuronal regeneration related protein	-2.436268	0.001537	0.016039
1390692_at	313560	Ctps_predi	cytidine 5'-triphosphate synthase (prec	1.189352	0.001539	0.016041
1367960_at	29308	Arl4a	ADP-ribosylation factor-like 4A	-1.143816	0.00154	0.016041
1378092_at	290230	RGD13111	similar to magnesium-dependent phos	1.445733	0.001543	0.016054
1384340_a_at	363518	Ard1_predi	N-acetyltransferase ARD1 homolog (S	1.310153	0.001546	0.016074
1393800_at	317403	NA	NA	-1.529693	0.001548	0.016085
1391812_at	315979	RGD13091	similar to RIKEN cDNA E330026B02 (	1.520122	0.001554	0.016111
1377948_at	363255	RGD13592	ankyrin repeat containing protein RGD	1.528763	0.001554	0.016111
1367530_at	362283	Stx16_prec	syntaxin 16 (predicted)	1.893851	0.001554	0.016111
1367897_at	25363	Acadvl	acyl-Coenzyme A dehydrogenase, ver	1.461379	0.001559	0.016147
1395617_at	50692	Plaur	plasminogen activator, urokinase recep	3.578891	0.001563	0.016154
1367654_at	83720	Fath	fat tumor suppressor homolog (Drosop	-1.639803	0.001563	0.016154
1373750_at	297595	Leprel2_pr	leprecan-like 2 (predicted)	-1.583605	0.001564	0.016154
1385238_at	361342	Cplx4_prec	complexin 4 (predicted)	1.240229	0.001564	0.016154
1377453_at	315578	RGD13091	similar to hypothetical protein FLJ2355	-1.937953	0.001567	0.016167
1377829_at	315423	Cep57	centrosomal protein 57	-1.87733	0.001569	0.016177
1367816_at	171160	Hod	homeobox only domain	-2.796984	0.00157	0.016177
1372557_at	363760	Arl6_predic	ADP-ribosylation factor-like 6 (predicte	-1.51449	0.001573	0.01618
1383585_s_at	297096	Snx10	sorting nexin 10	-2.60317	0.001573	0.01618
1384756_at	287532	Slc43a2_pr	solute carrier family 43, member 2 (pre	1.926777	0.001582	0.016235
1384024_at	315210	Brd1_predi	bromodomain containing 1 (predicted)	-2.063095	0.001583	0.016235
1389608_at	311959	Abcf2_prec	ATP-binding cassette, sub-family F (G	1.477058	0.001583	0.016235
1398349_at	24184	Ak2	adenylate kinase 2	1.36845	0.001584	0.016235

1375889_at	363469	Sms	spermine synthase	-2.438304	0.001584	0.016235
1382056_at	502603	LOC50260	similar to splicing factor p54	1.138537	0.001587	0.016242
1382903_at	310958	LOC31095	NA	-1.309161	0.001588	0.016242
1372519_at	291874	Nup93	nucleoporin 93	1.534027	0.00159	0.016256
1372212_at	293588	Paox_pred	polyamine oxidase (predicted)	2.789002	0.001591	0.016256
1391236_at	305268	RGD1308	similar to RIKEN cDNA 5730469D23 (	-1.212924	0.001598	0.01629
1388105_at	116656	D123	D123 gene product	1.25393	0.001598	0.01629
1390146_at	360916	RGD13061	similar to RIKEN cDNA 2610318G18 (	1.309996	0.001599	0.01629
1370908_at	84577	Hdac2	histone deacetylase 2	-1.306012	0.0016	0.01629
1375280_at	289277	PNAS-4	CGI-146 protein	1.171635	0.001606	0.016338
1370816_at	252917	Nr1d1	nuclear receptor subfamily 1, group D,	-2.872381	0.001613	0.016392
1398844_at	79462	Txn2	thioredoxin 2	1.61519	0.001613	0.016392
1374708_at	306618	RGD1565	similar to Rho guanine nucleotide exch	-1.297459	0.001616	0.016403
1367776_at	54237	Cdc2a	cell division cycle 2 homolog A (S. pon	-2.741347	0.001619	0.016413
1393366_at	299821	RGD1561	similar to Inner nuclear membrane pro	-1.443716	0.001619	0.016413
1388333_at	300084	Rbx1	ring-box 1	1.204836	0.00162	0.016413
1373383_at	299514	Mterfd1	MTERF domain containing 1	-1.219216	0.001622	0.016419
1390502_at	289084	RGD13091	similar to RIKEN cDNA 1700025G04 g	-1.702999	0.001624	0.01642
1371758_at	445268	Ufc1	Ufm1-conjugating enzyme 1	1.104259	0.001629	0.016466
1367885_at	29533	Pxmp2	peroxisomal membrane protein 2	1.427027	0.001638	0.016534
1393492_at	499348	NA	NA	-1.377772	0.00164	0.016534
1383326_a_at	64031	Pdcd4	programmed cell death 4	-1.861653	0.001641	0.016534
1379766_at	338477	Sla	src-like adaptor	3.305147	0.001642	0.016534
1373669_at	289608	Gnpda2_pr	glucosamine-6-phosphate deaminase	-2.016606	0.001642	0.016534
1372062_at	299334	RGD1563	similar to cyclin-dependent kinase 2-in	1.279377	0.001647	0.016572
1389228_at	297415	RGD1304	similar to RIKEN cDNA 2010309E21 (	1.531171	0.001651	0.016586
1388601_at	306960	Abt1	activator of basal transcription 1	1.421659	0.001651	0.016586
1398900_at	362504	Dctn3_pred	dynactin 3 (predicted)	1.111572	0.001659	0.016629
1374446_at	310467	Tiparp_pre	TCDD-inducible poly(ADP-ribose) poly	-3.182368	0.001659	0.016629
1374701_at	360967	MGC1094	similar to 1700022N24Rik protein	1.184867	0.00166	0.016629
1372160_at	171113	Blcap	bladder cancer associated protein hom	1.445309	0.001661	0.016629
1374085_at	360961	Mxd4_pred	Max dimerization protein 4 (predicted)	1.317633	0.001663	0.016644
1381398_at	296516	RGD1560	similar to Cystatin S precursor (LM pro	2.513982	0.001674	0.016736
1398360_at	679532	LOC67953	NA	1.154315	0.001678	0.016755
1370365_at	25458	Gss	glutathione synthetase	2.756769	0.001679	0.016755
1383851_at	289716	RGD13051	similar to RIKEN cDNA 4921513E08 (	-2.376958	0.00168	0.016755
1376797_at	362224	Csrp2bp_p	cysteine and glycine-rich protein 2 bin	1.370663	0.001683	0.01678
1375433_at	311324	Disp2_pred	dispatched homolog 2 (Drosophila) (pr	-1.145094	0.001689	0.016825
1383840_at	306375	RGD1307	similar to chondroitin beta1,4 N-acetyl	-1.976965	0.001691	0.016827
1375934_at	315911	RGD1566	similar to RIKEN cDNA D330045A20 (	1.587184	0.001696	0.016847
1368216_at	117049	Rab28	RAB28, member RAS oncogene family	-1.129795	0.001696	0.016847
1387372_at	171163	Slc6a13	solute carrier family 6 (neurotransmitte	2.488854	0.001696	0.016847
1388390_at	299899	Eif3s3	eukaryotic translation initiation factor 3	-1.110515	0.001702	0.016888
1372373_at	317370	Wdr13_pre	WD repeat domain 13 (predicted)	-1.366801	0.001703	0.016891
1372215_at	499185	RGD1559	similar to mitochondrial ribosomal prot	1.269796	0.001706	0.016907
1375037_at	309144	Saps3_pre	SAPS domain family, member 3 (predi	-1.125095	0.001708	0.01691
1387408_at	140593	Siah2	seven in absentia 2	-1.768769	0.00171	0.016915
1373896_at	25716	Syt1	synaptotagmin I	-2.068566	0.001718	0.016988
1370827_at	171015	Cyb5r4	cytochrome b5 reductase 4	1.101447	0.001723	0.017011
1373222_at	300757	Hexa	hexosaminidase A	-1.359438	0.001724	0.017011
1368052_at	171048	Tspan8	tetraspanin 8	-3.391105	0.001725	0.017011

1376751_at	361800	Stk19	serine/threonine kinase 19	2.241196	0.001727	0.017023
1379525_at	366196	RGD1311C	similar to chromosome 20 open readin	-1.668245	0.001733	0.01707
1372145_at	294810	Tars	threonyl-tRNA synthetase	1.181692	0.001737	0.01708
1376105_at	314981	Col14a1_p	procollagen, type XIV, alpha 1 (predict	-3.996784	0.001738	0.01708
1372013_at	293618	Ifitm1_prec	interferon induced transmembrane pro	-1.862333	0.001741	0.01708
1367493_at	498890	RGD15602	similar to DNA segment, Chr 18, Wayr	1.831115	0.001742	0.01708
1384885_at	298532	Tekt2	tektin 2	-2.515043	0.001742	0.01708
1380562_at	362416	Rpusd3_pr	RNA pseudouridylate synthase domair	1.350493	0.001744	0.01708
1390102_at	303902	Dirc2	disrupted in renal carcinoma 2 homolo	-1.91139	0.001744	0.01708
1383798_at	313430	MGC94145	similar to CDNA sequence BC026682	2.918265	0.001744	0.01708
1398441_at	316369	Nck2_pred	non-catalytic region of tyrosine kinase	1.309789	0.001747	0.01709
1373198_at	362998	RGD1311C	similar to RIKEN cDNA 2810451A06	4.06867	0.001752	0.017104
1388984_at	362858	RGD15653	similar to RNA polymerase III subunit F	1.230877	0.001752	0.017104
1397916_s_at	24697	Ptpn1	protein tyrosine phosphatase, non-rece	2.086585	0.001753	0.017104
1389217_at	60433	Rfng	radical fringe gene homolog (Drosophi	1.544489	0.001753	0.017104
1388012_at	252880	Prrxl1	paired related homeobox protein-like 1	2.281981	0.001755	0.017104
1388378_at	293484	Eif3s8	eukaryotic translation initiation factor 3	1.280807	0.001758	0.017106
1370224_at	25125	Stat3	signal transducer and activator of trans	1.437702	0.001759	0.017106
1384907_at	306096	LOC30609	NA	-2.352431	0.00176	0.017106
1388718_at	25566	Tmod1	tropomodulin 1	1.665441	0.001761	0.017106
1372688_at	316098	Exosc7	exosome component 7	1.203783	0.001761	0.017106
1399078_at	299607	Thrap5_pre	thyroid hormone receptor associated p	1.42305	0.001763	0.017113
1368382_at	114216	S100a3	S100 calcium binding protein A3	1.530081	0.001765	0.01712
1369160_a_at	117955	Slc4a7	solute carrier family 4, sodium bicarbor	1.427755	0.001772	0.017154
1368514_at	25750	Maob	monoamine oxidase B	-1.985249	0.001774	0.017154
1368231_at	24918	Stat5a	signal transducer and activator of trans	1.294537	0.001775	0.017154
1389389_at	289780	Ddx56	DEAD (Asp-Glu-Ala-Asp) box polypept	2.320234	0.001775	0.017154
1369961_at	64369	Ppap2a	phosphatidic acid phosphatase 2a	2.34133	0.001775	0.017154
1367762_at	24797	Sst	somatostatin	-2.561291	0.001778	0.017163
1398863_at	81667	Gnb2	guanine nucleotide binding protein, be	1.37227	0.001779	0.017163
1375518_at	84015	Ttn	titin	1.805818	0.001781	0.017163
1372895_at	361118	RGD13096	similar to RIKEN cDNA 5730469M10	-1.972979	0.001782	0.017163
1381294_at	309387	Cnnm1_pre	cyclin M1 (predicted)	1.403357	0.001783	0.017163
1370003_at	29565	Eef2	eukaryotic translation elongation factor	1.278289	0.001786	0.017163
1387058_at	29510	Pctp	phosphatidylcholine transfer protein	1.963441	0.001786	0.017163
1370577_at	286979	Zfp455	zinc finger protein 455	1.231343	0.001787	0.017163
1393051_at	501619	LOC50161	similar to 40S ribosomal protein S29	-1.859258	0.001791	0.017179
1374459_at	313231	Alg2	asparagine-linked glycosylation 2 hom	1.147754	0.001792	0.017179
1372747_at	29502	Slc20a2	solute carrier family 20, member 2	2.233214	0.001793	0.017179
1372010_at	498183	MGC11632	similar to MK-5 type 2	-1.239231	0.001793	0.017179
1376299_at	312678	LOC31267	NA	1.524617	0.001797	0.017197
1389193_at	305438	Sorcs2_pre	sortilin-related VPS10 domain containi	-1.754219	0.001799	0.017204
1387122_at	25157	Plagl1	pleiomorphic adenoma gene-like 1	-3.714079	0.001801	0.01721
1380167_at	50622	Slc23a2	solute carrier family 23 (nucleobase tra	2.136809	0.001804	0.017223
1387074_at	84583	Rgs2	regulator of G-protein signaling 2	-2.123307	0.001804	0.017223
1378209_a_at	293854	Stk23	serine/threonine kinase 23	-3.878281	0.001807	0.01723
1383422_at	362564	LOC36256	NA	-2.201454	0.001809	0.017243
1379832_at	364834	Polr2d_pre	polymerase (RNA) II (DNA directed) po	-1.242266	0.001811	0.017244
1376644_at	311165	Med19_pre	mediator of RNA polymerase II transcr	1.46237	0.001812	0.017244
1372069_at	309429	Ankrd15	ankyrin repeat domain 15	-1.305838	0.001814	0.017252
1372882_at	296587	RGD13086	similar to CG12379-PA (predicted)	2.180774	0.001816	0.017252

1373918_at	362757	Rdh11	retinol dehydrogenase 11	1.889118	0.001817	0.017252
1368869_at	83425	Akap12	A kinase (PRKA) anchor protein (gravi	-2.509569	0.00182	0.017252
1375529_at	359725	Cbr4	carbonic reductase 4	-1.738035	0.00182	0.017252
1389245_at	307821	Psmd7_pre	proteasome (prosome, macropain) 26S	1.21404	0.001821	0.017252
1389043_at	308453	Adck4	aarF domain containing kinase 4	1.710039	0.001824	0.017268
1373163_at	315639	Usp28_pre	ubiquitin specific protease 28 (predicte	-1.377321	0.001825	0.017268
1369952_at	171350	Pabpc1	poly(A) binding protein, cytoplasmic 1	1.836018	0.001828	0.017282
1393433_at	365691	RGD13065	similar to Agrin (predicted)	4.021288	0.001832	0.017305
1377854_at	311350	RGD13056	similar to hypothetical protein FLJ2337	1.233386	0.001833	0.017305
1395982_at	296634	RGD13049	similar to mKIAA0023 protein (predicte	1.27939	0.001837	0.017332
1372242_at	317335	Ddx3x	DEAD/H (Asp-Glu-Ala-Asp/His) box po	-1.141561	0.00184	0.017349
1375357_at	266606	Dyt1	dystonia 1	1.618463	0.001843	0.017368
1371128_at	287287	Il4	interleukin 4	1.596224	0.001851	0.017425
1382052_at	362630	Fusip1	FUS interacting protein (serine-arginine	-1.397884	0.001852	0.017425
1369974_at	24803	Vamp2	vesicle-associated membrane protein 2	1.530666	0.001862	0.017507
1367747_at	64664	Arl3	ADP-ribosylation factor-like 3	-1.425983	0.001864	0.017515
1389868_at	500988	RGD15645	similar to RCK (predicted)	1.961139	0.001868	0.017528
1369948_at	117089	Ngfrap1	nerve growth factor receptor (TNFRSF	-1.347305	0.001868	0.017528
1387770_at	170512	Ifi27l	interferon, alpha-inducible protein 27-li	1.516232	0.001878	0.017605
1387022_at	24188	Aldh1a1	aldehyde dehydrogenase family 1, me	-1.62493	0.00188	0.017609
1381364_at	309126	Tnpo1	transportin 1	1.260615	0.001881	0.017609
1394567_at	170671	Lhx3	LIM homeobox protein 3	2.619128	0.001883	0.017612
1368133_at	29365	Mpdz	multiple PDZ domain protein	-1.576002	0.001885	0.017622
1382589_at	361898	RGD13077	similar to hypothetical protein FLJ1179	1.155262	0.001891	0.017646
1377044_at	171438	Crtac1	cartilage acidic protein 1	-2.163007	0.001891	0.017646
1371819_at	84580	Hdac5	histone deacetylase 5	-1.442208	0.001892	0.017646
1398962_at	299617	RGD13596	uncharacterized protein family UPF022	1.283671	0.001893	0.017646
1374333_at	361224	RGD13060	similar to RIKEN cDNA 1110007C09 (p	1.235615	0.001899	0.01768
1393267_at	313323	Psip1	PC4 and SFRS1 interacting protein 1	-1.483022	0.001899	0.01768
1382365_at	497961	RGD15616	similar to nemo like kinase (predicted)	1.1667	0.001904	0.017707
1372654_at	361674	Eps8l2_pre	EPS8-like 2 (predicted)	1.506113	0.001905	0.017707
1383965_at	288040	Ncbp2_pre	nuclear cap binding protein subunit 2 (	-1.570649	0.00191	0.017746
1374401_at	291464	Snx2_pred	sorting nexin 2 (predicted)	-1.534536	0.001913	0.01776
1384178_at	308571	Lrrc4b_pre	leucine rich repeat containing 4B (prec	-2.313827	0.001916	0.01776
1374412_at	501682	NA	NA	-1.140785	0.001916	0.01776
1367932_at	29637	Hmgcs1	3-hydroxy-3-methylglutaryl-Coenzyme	-2.070971	0.001922	0.017803
1368827_at	29300	Gata6	GATA binding protein 6	-4.919245	0.001926	0.017803
1372304_at	363068	Commd4_p	COMM domain containing 4 (predicted	1.435908	0.001927	0.017803
1379810_at	114632	Scye1	small inducible cytokine subfamily E, m	1.379697	0.001927	0.017803
1377118_at	290722	RGD13085	similar to KIAA1712 protein	-2.122343	0.001928	0.017803
1377340_at	286926	Tfpi2	tissue factor pathway inhibitor 2	-4.102435	0.001929	0.017803
1395472_at	502715	Lrrc17	leucine rich repeat containing 17	3.450323	0.00193	0.017803
1393621_at	399684	Vkorc1l1	vitamin K epoxide reductase complex,	1.334523	0.001931	0.017803
1384609_a_at	363089	RGD13114	similar to RIKEN cDNA B230380D07 (	2.023054	0.001941	0.017876
1367651_at	171293	Ctsd	cathepsin D	1.715489	0.001942	0.017876
1388351_at	288455	RGD13058	similar to chromosome 13 open readin	1.115725	0.001945	0.017886
1390403_at	288031	RGD13047	similar to CG8312-PA	-2.001576	0.001946	0.017886
1383831_at	309911	Qrsl1	glutaminyl-tRNA synthase (glutamine-h	1.297402	0.001947	0.017886
1373570_at	311671	Npepl1_pre	aminopeptidase-like 1 (predicted)	2.794704	0.001949	0.017889
1388948_at	293150	Stard10	START domain containing 10	1.441987	0.001951	0.0179
1388426_at	78968	Srebf1	sterol regulatory element binding facto	1.132533	0.001954	0.017915

1373276_at	499657	RGD15622	similar to S100 calcium-binding protein	1.698344	0.001956	0.017916
1367908_at	171133	Gcsh	glycine cleavage system protein H (am	1.451759	0.001966	0.01799
1371005_at	24565	Abcc1	ATP-binding cassette, sub-family C (C	1.206174	0.001967	0.01799
1389247_at	361102	RGD13055	similar to polymerase (RNA) III (DNA c	1.403675	0.001968	0.01799
1384254_at	498803	RGD15633	similar to OTU domain containing 1 (pr	-1.861497	0.00197	0.01799
1384787_at	300678	Cd3g	CD3 antigen, gamma polypeptide	-2.366045	0.001972	0.01799
1395760_at	303317	RGD13074	similar to membrane protein expressed	1.448164	0.001972	0.01799
1381753_at	361907	Fbxl7_prec	F-box and leucine-rich repeat protein 7	-3.522849	0.00198	0.01805
1388883_at	361698	Pold4	polymerase (DNA-directed), delta 4	1.231522	0.001985	0.018085
1375659_at	361273	Sec61a2_p	Sec61, alpha subunit 2 (S. cerevisiae)	-1.120823	0.00199	0.018097
1372128_at	292758	Mrps12_pr	mitochondrial ribosomal protein S12 (p	1.286548	0.001991	0.018097
1376609_at	291750	RGD13091	similar to TRS85 homolog (predicted)	-1.05479	0.001991	0.018097
1370807_at	192129	Tmem49	transmembrane protein 49	1.527303	0.001993	0.018097
1391733_at	362556	Ttc4	tetratricopeptide repeat domain 4	1.132056	0.001995	0.018097
1367730_at	56042	Vdp	vesicle docking protein	1.3159	0.001995	0.018097
1389444_at	305923	RGD13057	similar to RIKEN cDNA 5033406L14	1.169909	0.001996	0.018097
1396556_at	498438	LOC49843	NA	2.561323	0.002002	0.018138
1382848_at	25098	Foxa1	forkhead box A1	-2.061749	0.002008	0.018172
1376359_at	361733	Ms4a8b_pr	membrane-spanning 4-domains, subfa	1.474235	0.002008	0.018172
1370561_at	171553	A3galt2	alpha 1,3-galactosyltransferase 2 (isog	1.569291	0.002011	0.018186
1369057_at	81804	Stxbp2	syntaxin binding protein 2	1.351091	0.002013	0.018189
1371622_at	497954	RGD15646	similar to candidate tumor suppressor	1.459693	0.002019	0.018218
1372665_at	293820	Psat1	phosphoserine aminotransferase 1	1.32896	0.002019	0.018218
1391115_at	498229	LOC49822	similar to RIKEN cDNA 4930429O20	1.581553	0.002027	0.01828
1375645_at	500575	RGD15602	similar to DNA segment, Chr 4, ERATO	1.538148	0.00203	0.018297
1372879_at	292887	Akt1s1_pre	AKT1 substrate 1 (proline-rich) (predic	2.028986	0.002039	0.018345
1372316_at	313860	RGD13119	similar to AI115348 protein (predicted)	1.294759	0.002039	0.018345
1389052_at	292095	Ttc13	tetratricopeptide repeat domain 13	1.69804	0.002041	0.018345
1390125_at	361043	Tm9sf1	transmembrane 9 superfamily member	1.072476	0.002041	0.018345
1370439_a_at	246153	Kcnc2	potassium voltage gated channel, Sha	-1.608799	0.002045	0.018351
1382711_at	498160	MGC11636	zinc finger protein 36 (KOX 18)	1.654213	0.002045	0.018351
1393659_at	310846	Tram1l1_p	translocation associated membrane pr	-2.652995	0.002046	0.018351
1375504_at	303612	Polg2_prec	polymerase (DNA directed), gamma 2,	-1.405906	0.002048	0.018357
1391625_at	360206	Asb15	ankyrin repeat and SOCS box-containi	1.245186	0.002052	0.018375
1377178_at	288664	RGD13106	similar to hypothetical protein FLJ3235	1.76201	0.002056	0.018379
1371717_at	192647	Mfn1	mitofusin 1	1.402894	0.002057	0.018379
1380453_at	313524	Zswim5_pr	zinc finger, SWIM domain containing 5	1.384154	0.002059	0.018379
1379526_at	24547	Mbp	myelin basic protein	-1.749401	0.002059	0.018379
1369445_at	64046	Mre11a	meiotic recombination 11 homolog A (S	4.104978	0.002061	0.018379
1372233_at	296306	Ergic3_pre	ERGIC and golgi 3 (predicted)	-1.091662	0.002061	0.018379
1385727_at	362463	Fgfr1op2	FGFR1 oncogene partner 2	1.389381	0.002062	0.018379
1374767_at	290963	RGD13095	similar to hypothetical protein FLJ1467	1.476087	0.002068	0.018398
1374854_at	305963	RGD15660	similar to Pin2-interacting protein X1 (p	1.976285	0.002068	0.018398
1392525_at	25652	Ptgfr	prostaglandin F receptor	-2.593996	0.002068	0.018398
1371416_at	293655	Ndufv1	NADH dehydrogenase (ubiquinone) fla	1.2317	0.002071	0.018412
1398916_at	298687	Aurkaip1	aurora kinase A interacting protein 1	1.219145	0.002077	0.018454
1387811_at	24179	Agt	angiotensinogen (serpin peptidase inh	-3.265184	0.002081	0.018463
1389685_at	360764	Zfp655	zinc finger protein 655	-1.325883	0.002082	0.018463
1386321_s_at	246273	Trib3	tribbles homolog 3 (Drosophila)	2.667513	0.002082	0.018463
1386878_at	305880	Lrp10	low-density lipoprotein receptor-related	1.478412	0.002085	0.018463
1370362_at	116660	Ptprn	protein tyrosine phosphatase, receptor	1.455606	0.002085	0.018463

1376571_at	363171	Tmem42_p	transmembrane protein 42 (predicted)	1.374411	0.002087	0.018464
1368405_at	81757	Rala	v-ral simian leukemia viral oncogene h	1.299494	0.002093	0.018471
1372480_at	313176	Zfp462_pre	zinc finger protein 462 (predicted)	1.238513	0.002093	0.018471
1384979_at	117097	Gpr50	G protein-coupled receptor 50	3.895831	0.002093	0.018471
1372607_at	287125	Nubp2	nucleotide binding protein 2	1.468502	0.002094	0.018471
1395403_at	362895	Stac3_prec	SH3 and cysteine rich domain 3 (predi	1.324895	0.002094	0.018471
1399011_at	304343	Cops6_pre	COP9 (constitutive photomorphogenic	1.171708	0.0021	0.018497
1384384_at	362461	RGD13045	similar to KIAA0528 protein (predicted)	1.112666	0.0021	0.018497
1372050_at	290637	Glt25d1_pr	glycosyltransferase 25 domain contain	1.24756	0.002101	0.018497
1386795_at	296846	RGD13056	similar to hypothetical protein FLJ2553	-2.528503	0.002108	0.018523
1372827_at	361967	Ppid	peptidylprolyl isomerase D (cyclophilin	-1.314718	0.002108	0.018523
1373945_at	314647	RGD15650	similar to bruno-like 5, RNA binding pro	-1.701643	0.00211	0.018523
1371780_at	304290	Kdelr2	KDEL (Lys-Asp-Glu-Leu) endoplasmic	1.149822	0.00211	0.018523
1398351_at	360471	Usp7	ubiquitin specific protease 7 (herpes vi	1.084098	0.002111	0.018523
1393244_at	140639	Nploc4	nuclear protein localization 4 homolog	1.63842	0.002125	0.018603
1389200_at	359727	Bysl	bystin-like	1.488389	0.002126	0.018603
1391611_at	313254	Kif12	kinesin family member 12	-1.357146	0.002126	0.018603
1393213_at	298686	Ccnl2	cyclin L2	1.609632	0.002126	0.018603
1372819_at	361407	Cog4_pred	component of oligomeric golgi complex	1.240348	0.00213	0.018613
1373391_at	289196	Tmco1	transmembrane and coiled-coil domain	1.316822	0.00213	0.018613
1389316_at	363445	Usp9x_pre	ubiquitin specific peptidase 9, X chrom	-1.202647	0.002142	0.018699
1392099_at	310463	Plcl3_predi	phospholipase C-like 3 (predicted)	-1.658529	0.002143	0.018699
1371788_at	501926	RGD15597	similar to RIKEN cDNA 2610510L01 (p	-3.240807	0.002144	0.018701
1378155_at	360865	RGD15660	similar to KIAA1096 protein (predicted)	1.131908	0.002147	0.018714
1389665_at	361196	Habp4_pre	hyaluronic acid binding protein 4 (pred	1.913099	0.002153	0.018747
1398819_at	65028	Dnaja1	DnaJ (Hsp40) homolog, subfamily A, m	-1.619001	0.002154	0.018747
1391605_at	362263	Ptprt_predi	protein tyrosine phosphatase, receptor	-2.220423	0.002157	0.018747
1371420_at	494529	LOC49452	92Aa-Protein	-1.093289	0.002159	0.018747
1372948_at	498525	LOC49852	Bm403207	1.267108	0.002159	0.018747
1376845_at	299269	isg12(b)	putative ISG12(b) protein	2.341081	0.00216	0.018747
1376250_at	364430	Nufip1	nuclear fragile X mental retardation pro	-1.475989	0.00217	0.018821
1385856_at	291061	Riok1	RIO kinase 1 (yeast)	1.048672	0.002171	0.018824
1371453_at	301544	Farslb	phenylalanine-tRNA synthetase-like, b	1.385345	0.002173	0.018824
1374917_at	309858	Sec63_pre	SEC63-like (S. cerevisiae) (predicted)	-1.461882	0.002175	0.018829
1382921_at	289504	Sdad1	SDA1 domain containing 1	1.321715	0.002176	0.018831
1377945_at	308490	Ddx18	DEAD (Asp-Glu-Ala-Asp) box polypept	1.24337	0.002181	0.018854
1398815_at	24206	Apeh	acylpeptide hydrolase	1.202675	0.002185	0.018874
1367506_at	293666	mrpl11	mitochondrial ribosomal protein L11	2.203867	0.002186	0.018874
1373160_at	498653	NA	NA	1.074598	0.00219	0.018901
1387371_at	171102	Cdc25a	cell division cycle 25 homolog A (S. ce	1.893261	0.002197	0.018943
1372298_at	298066	Txndc4	thioredoxin domain containing 4 (endo	1.48876	0.002199	0.018943
1373478_at	83708	Mybph	myosin binding protein H	3.276717	0.002201	0.018943
1395094_at	114502	Pax3	paired box gene 3	-2.47631	0.002201	0.018943
1398909_at	301124	LOC30112	NA	1.202203	0.002202	0.018943
1388318_at	24644	Pgk1	phosphoglycerate kinase 1	1.6916	0.002207	0.018976
1387161_at	84012	Slc1a6	solute carrier family 1 (high affinity asp	2.983011	0.00221	0.018987
1391169_at	500921	RGD15625	similar to RIKEN cDNA B930062P21 g	-1.902028	0.002213	0.019001
1383409_at	500504	NA	NA	-1.364602	0.002218	0.01903
1388163_at	25176	Slc25a5	solute carrier family 25 (mitochondrial	1.302997	0.002223	0.019058
1388375_at	25725	Prkar1a	protein kinase, cAMP dependent regul	-1.157458	0.002232	0.019121
1367498_at	296467	Ythdf1	YTH domain family 1	-1.116584	0.002233	0.019121

1377769_at	360785	Ap1s1_pre	adaptor protein complex AP-1, sigma 1	2.179174	0.002235	0.019123
1389643_at	314598	Wiz_predic	widely-interspaced zinc finger motifs (p	-1.559417	0.002238	0.019139
1398763_at	54312	Timm23	translocase of inner mitochondrial mer	1.150844	0.002243	0.019172
1371837_at	287765	Ddx5	ddx5 gene	-1.311454	0.002247	0.01919
1390097_at	309828	Tspyl4	TSPY-like 4	1.281179	0.002255	0.019243
1388411_at	361988	RGD13045	similar to expressed sequence C77668	1.745315	0.00226	0.019243
1390096_at	292156	Sh3glb1	SH3-domain GRB2-like B1 (endophilin	-1.846262	0.00226	0.019243
1387721_at	29290	Adora1	adenosine A1 receptor	2.183511	0.00226	0.019243
1371989_at	113990	Hmgn3	high mobility group nucleosomal bindir	-1.357368	0.00226	0.019243
1388552_at	308909	Smpd1	sphingomyelin phosphodiesterase 1, a	1.135727	0.002265	0.01926
1392701_at	363145	Gmppb_pr	GDP-mannose pyrophosphorylase B (	1.467907	0.002265	0.01926
1371774_at	302642	Sat_mappe	spermidine/spermine N1-acetyl transfe	-1.924221	0.002267	0.019261
1391461_at	298002	RGD13065	similar to hypothetical protein (predicte	1.328377	0.002269	0.019269
1373812_at	83571	Cdkn1b	cyclin-dependent kinase inhibitor 1B	-1.472451	0.002274	0.019293
1387900_at	192260	Cdipt	CDP-diacylglycerol--inositol 3-phospha	1.16449	0.002279	0.019313
1370975_at	312440	Jmjd1a	jumonji domain containing 1A	-1.746792	0.002279	0.019313
1376704_a_at	309259	Ndnl2	necdin-like 2	-1.390948	0.002282	0.019329
1374506_at	311835	Ddx31_pre	DEAD/H (Asp-Glu-Ala-Asp/His) box po	1.262104	0.002287	0.019359
1373507_at	307170	Acbd5	acyl-Coenzyme A binding domain cont	-1.277532	0.00229	0.019362
1384292_at	312477	Dok1	docking protein 1	1.513481	0.00229	0.019362
1375590_at	360845	RGD13082	similar to RIKEN cDNA 5730454B08 (p	1.822937	0.002297	0.019393
1371008_at	296588	Pmpca	peptidase (mitochondrial processing) a	1.858734	0.002299	0.019393
1395520_at	312182	Rbm28_pre	RNA binding motif protein 28 (predicte	1.183274	0.0023	0.019393
1390474_at	362899	RGD15643	similar to RIKEN cDNA 9330161F08 (p	1.353239	0.002302	0.019393
1392171_at	89824	Chi3l1	chitinase 3-like 1	-2.117337	0.002303	0.019393
1370025_at	140607	Pip5k2c	phosphatidylinositol-4-phosphate 5-kin	1.211225	0.002303	0.019393
1398562_at	316412	Als2cr4_pr	amyotrophic lateral sclerosis 2 (juvenil	1.204697	0.002304	0.019393
1380451_at	364378	RGD13113	similar to T cell receptor V delta 6	1.67882	0.002309	0.019416
1382064_at	292051	RGD13061	similar to 4933407C03Rik protein (pre	1.029104	0.002318	0.019464
1381403_at	307270	Me2_predi	malic enzyme 2, NAD(+)-dependent, m	1.844489	0.002319	0.019464
1396521_at	291699	Stard4_pre	StAR-related lipid transfer (START) do	-1.194866	0.002319	0.019464
1378675_at	366189	RGD15618	similar to transglutaminase E3 (predict	2.42072	0.002322	0.019465
1387047_at	78951	Hspb3	heat shock 27kDa protein 3	-1.989921	0.002322	0.019465
1390333_at	171366	Ppp4c	protein phosphatase 4, catalytic subun	1.730239	0.002328	0.019504
1367463_at	114766	Phb2	prohibitin 2	1.326104	0.00233	0.019513
1389044_at	309451	RGD13071	similar to golgi-specific brefeldin A-resi	1.693859	0.002332	0.019513
1369303_at	81648	Crh	corticotropin releasing hormone	-2.561746	0.002333	0.019513
1397632_at	306147	Slitrk1_pre	SLIT and NTRK-like family, member 1	1.593756	0.002337	0.019524
1396863_at	300950	Spsb4_pre	splA/ryanodine receptor domain and S	-2.065119	0.002339	0.019524
1388833_at	298098	Pole3	polymerase (DNA directed), epsilon 3	1.140112	0.002341	0.019524
1374520_at	498875	RGD15644	similar to heart alpha-kinase (predicted	-4.417894	0.002341	0.019524
1374406_at	64561	Phka1	phosphorylase kinase alpha 1	-1.030885	0.002342	0.019524
1392513_at	361182	RGD13070	similar to hypothetical protein FLJ1130	2.175608	0.002348	0.019529
1379330_s_at	502114	NA	NA	-1.079815	0.002348	0.019529
1378753_at	83497	Tpmt	thiopurine methyltransferase	-1.343278	0.002348	0.019529
1388324_at	289222	Nit1	nitrilase 1	1.051644	0.00235	0.019529
1367666_at	140931	Hnrph1	heterogeneous nuclear ribonucleoprote	-1.21983	0.002351	0.019529
1386518_at	290749	Irf2_predict	interferon regulatory factor 2 (predicted	2.619778	0.002353	0.019529
1391014_at	311791	Zmynd19	zinc finger, MYND domain containing 1	1.300384	0.002353	0.019529
1388952_at	360583	RGD13079	similar to Hypothetical protein MGC187	1.559285	0.002356	0.019532
1374573_at	298767	Dync2li1	dynein cytoplasmic 2 light intermediate	-1.223384	0.002356	0.019532

1394571_at	300074	RGD13059	hypothetical LOC300074	-1.886124	0.00236	0.019548
1380239_at	310506	Ppm1l_pre	protein phosphatase 1 (formerly 2C)-lil	2.317344	0.002364	0.01957
1383579_at	502282	NA	NA	-1.349457	0.002369	0.019573
1388930_at	363013	Tmem123	transmembrane protein 123	1.088108	0.00237	0.019573
1373683_at	25150	Fyn	fyn proto-oncogene	-1.290592	0.00237	0.019573
1390787_at	288259	Gart	phosphoribosylglycinamide formyltrans	1.541422	0.00237	0.019573
1367472_at	314432	LOC31443	similar to ubiquitin-protein ligase (EC 6	1.224488	0.002374	0.019576
1381174_at	307480	RGD73502	SEL1 domain containing protein RGD7	1.167	0.002374	0.019576
1369448_at	117108	B3gat1	beta-1,3-glucuronyltransferase 1 (gluc	2.178119	0.002375	0.019576
1374332_at	290660	Ddx49_pre	DEAD (Asp-Glu-Ala-Asp) box polypept	1.543113	0.002377	0.01958
1382303_at	306844	Phactr1	phosphatase and actin regulator 1	1.813616	0.002379	0.01958
1369274_a_at	60396	Cdkl3	cyclin-dependent kinase-like 3	-1.812819	0.00238	0.01958
1371317_at	309447	Ldb1_pred	LIM domain binding 1 (predicted)	1.353393	0.002384	0.019602
1367669_a_at	64862	Map1lc3b	microtubule-associated protein 1 light	1.375808	0.002387	0.019609
1367750_at	64390	Prpsap1	phosphoribosyl pyrophosphate synthet	1.461599	0.002388	0.019609
1367919_at	58958	Pom210	nuclear pore membrane glycoprotein 2	-1.474337	0.00239	0.019609
1380101_at	305586	Papolg_pre	poly(A) polymerase gamma (predicted	-1.362807	0.002391	0.019609
1388938_at	297593	Usp5_pred	ubiquitin specific protease 5 (isopeptid	1.352867	0.002395	0.019619
1391269_at	317366	NA	NA	-1.238594	0.002395	0.019619
1374605_at	309953	RGD13096	similar to hypothetical protein MGC332	-1.103811	0.002399	0.019633
1368212_at	81650	Csnk2b	casein kinase 2, beta subunit	-1.239754	0.0024	0.019633
1392810_at	315137	Apobec3	apolipoprotein B editing complex 3	-1.930433	0.002402	0.019633
1393738_s_at	306508	Mfhas1_pre	malignant fibrous histiocytoma amplifie	1.124674	0.002403	0.019633
1369063_at	25379	Anp32a	acidic (leucine-rich) nuclear phosphop	1.666467	0.002404	0.019633
1382426_at	499950	RGD15637	similar to BC040823 protein (predicted	1.230141	0.002406	0.019634
1398846_at	501511	RGD15622	similar to Eukaryotic translation initiatio	1.466144	0.002408	0.019641
1392780_at	501621	LOC50162	similar to nuclear RNA export factor 7	3.944068	0.00242	0.019724
1373865_at	65178	Snap91	synaptosomal-associated protein, 91kI	-1.023265	0.002428	0.019777
1369927_at	81829	Mor1	malate dehydrogenase, mitochondrial	1.146065	0.002431	0.019788
1368871_at	116667	Map3k1	mitogen activated protein kinase kinas	1.68968	0.002438	0.019818
1370205_at	84511	Slco1c1	solute carrier organic anion transporte	4.282058	0.002441	0.019818
1370834_at	84406	Hs3st1	heparan sulfate (glucosamine) 3-O-sul	-2.834005	0.002442	0.019818
1393094_at	498883	NA	NA	1.497217	0.002443	0.019818
1383876_at	313365	Ift74	intraflagellar transport 74 homolog (Ch	-1.044317	0.002449	0.019818
1367535_at	308404	Irf2bp1_pre	interferon regulatory factor 2 binding p	1.252039	0.00245	0.019818
1367676_at	29395	Hmgb2	high mobility group box 2	-1.826316	0.002452	0.019818
1382074_at	25008	Lta	lymphotoxin A	1.18419	0.002452	0.019818
1388134_at	300033	Eef1d	eukaryotic translation elongation facto	1.164994	0.002454	0.019818
1372324_at	497975	LOC49797	NA	-1.153711	0.002455	0.019818
1368124_at	171109	Dusp5	dual specificity phosphatase 5	1.727543	0.002456	0.019818
1374807_at	305667	RGD13108	similar to RIKEN cDNA 1810063B07 g	1.134092	0.00246	0.019818
1367767_at	79238	Hmgcl	3-hydroxy-3-methylglutaryl-Coenzyme	1.268752	0.002461	0.019818
1389282_at	360606	Itga3_pred	integrin alpha 3 (predicted)	1.959496	0.002462	0.019818
1368063_a_at	170956	Yt521	splicing factor YT521-B	-1.211933	0.002462	0.019818
1375977_at	84593	Cetn2	centrin 2	-1.251791	0.002462	0.019818
1374671_at	361857	MGC11284	NA	1.372169	0.002463	0.019818
1376164_at	290666	Sf4	splicing factor 4	1.111157	0.002463	0.019818
1376990_at	361044	RGD13093	similar to HCDI protein (predicted)	-1.22139	0.002463	0.019818
1389199_at	315891	RGD13090	similar to Ab2-095	-1.789985	0.002464	0.019818
1367713_at	54318	Eif2s1	eukaryotic translation initiation factor 2	1.795349	0.002471	0.019858
1376448_at	301549	Wdfy1	WD repeat and FYVE domain containi	-1.427343	0.002476	0.019874

1389483_at	315496	Dpy19l1_p	dpy-19-like 1 (C. elegans) (predicted)	-1.565177	0.002476	0.019874
1372506_at	25634	G6pc	glucose-6-phosphatase, catalytic	1.058525	0.002478	0.019879
1386953_at	25116	Hsd11b1	hydroxysteroid 11-beta dehydrogenase	-4.233215	0.002482	0.019898
1376720_at	312258	Adck2_pre	aarF domain containing kinase 2 (pred	1.642032	0.002483	0.0199
1379816_at	304572	RGD15633	similar to RIKEN cDNA 2410025L10 (p	-1.493794	0.002488	0.019922
1370182_at	29714	Ptprn2	protein tyrosine phosphatase, receptor	-1.154442	0.002491	0.01993
1375427_at	360739	Ranbp1_pr	RAN binding protein 1 (predicted)	1.39357	0.002492	0.01993
1374415_at	361640	Polr3e_pre	polymerase (RNA) III (DNA directed) p	-1.152743	0.002494	0.019936
1385397_at	499991	LOC49999	Ab1-219	-1.870753	0.002501	0.019984
1388444_at	304766	Ubxd2	UBX domain containing 2	1.010376	0.002504	0.019993
1389969_at	308416	Tomm40	translocase of outer mitochondrial mer	2.067515	0.002506	0.019999
1381993_at	294141	Clic2	chloride intracellular channel 2	-2.21983	0.00251	0.020014
1370296_at	25541	Scp2	sterol carrier protein 2	1.198101	0.002511	0.020014
1398904_at	317259	Nono	non-POU domain containing, octamer-	-0.993178	0.002524	0.020104
1372019_at	290939	RGD13101	similar to DNA segment, Chr 13, Wayr	1.15955	0.002528	0.020105
1383606_at	500707	Mtac2d1	membrane targeting (tandem) C2 dom	-1.155114	0.002528	0.020105
1388117_at	171365	Snrpb	small nuclear ribonucleoprotein polype	1.055936	0.00253	0.020105
1388828_at	315617	Sidt2_pred	SID1 transmembrane family, member	-1.319682	0.002532	0.020105
1375621_at	261737	Sfxn5	sideroflexin 5	2.379381	0.002534	0.020105
1391827_at	306204	Flnb_predi	filamin, beta (predicted)	1.413277	0.002535	0.020105
1375205_at	301164	Pcaf	p300/CBP-associated factor	-1.285951	0.002535	0.020105
1388932_at	140433	Lama5	laminin, alpha 5	-2.383058	0.002542	0.020152
1388640_at	499417	LOC49941	similar to Ubiquitin-like protein SMT3A	1.023525	0.002545	0.020163
1398801_at	171456	Cdk105	CDK105 protein	-1.219593	0.002547	0.020164
1369410_at	94189	Gosr1	golgi SNAP receptor complex member	1.634973	0.002549	0.020164
1376171_at	408217	Usp11	ubiquitin specific protease 11	-1.179982	0.002551	0.020164
1390272_at	295394	Dph5	DPH5 homolog (S. cerevisiae)	1.167758	0.002551	0.020164
1398302_at	64361	Prlpf	prolactin-like protein F	1.252605	0.002557	0.020181
1372043_at	298586	RGD13117	similar to ribosomal protein P0-like pro	1.585513	0.002558	0.020181
1375421_a_at	192256	Pja2	praja 2, RING-H2 motif containing	1.020237	0.00256	0.020181
1370854_at	246172	Nexn	nexilin	1.993078	0.00256	0.020181
1372498_at	307649	Ciapin1	cytokine induced apoptosis inhibitor 1	1.043926	0.002562	0.020181
1373406_at	311854	Tor1b	torsin family 1, member B	1.614753	0.002563	0.020181
1380371_at	294787	NIPBL	Nipped-B homolog (Drosophila)	1.012084	0.002565	0.020181
1388154_at	116651	E2f5	E2F transcription factor 5	-1.406879	0.002566	0.020181
1387429_at	25238	Adrbk1	adrenergic receptor kinase, beta 1	1.240613	0.002567	0.020181
1398324_at	290641	MGC72957	similar to 60S ribosomal protein L18a	1.148803	0.002571	0.020189
1376931_at	311575	RGD13050	similar to Hepatocellular carcinoma-as	1.302527	0.002573	0.020189
1375022_at	307350	Afg3l2	AFG3(ATPase family gene 3)-like 2 (y	1.144385	0.002573	0.020189
1390486_at	303394	Usp32_pre	ubiquitin specific protease 32 (predicte	1.395742	0.002579	0.02019
1383136_a_at	363459	NA	NA	-1.778681	0.002579	0.02019
1376285_at	314543	Gulp1	GULP, engulfment adaptor PTB doma	-1.422497	0.002579	0.02019
1394640_at	311033	Fmnl2_pre	formin-like 2 (predicted)	3.743992	0.00258	0.02019
1388902_at	315714	Loxl1	lysyl oxidase-like 1	-4.27823	0.002584	0.02019
1387076_at	29560	Hif1a	hypoxia inducible factor 1, alpha subur	-1.069604	0.002585	0.02019
1373705_at	497876	LOC49787	NA	1.120044	0.002585	0.02019
1376650_at	299258	Golga5	golgi autoantigen, golgin subfamily a, 5	1.293789	0.002585	0.02019
1398797_at	117282	Hnrpk	heterogeneous nuclear ribonucleoprote	-1.163687	0.00259	0.020209
1372575_at	297695	Wbp11	WW domain binding protein 11	-1.063122	0.002591	0.020209
1371349_at	294337	Col6a1_pre	procollagen, type VI, alpha 1 (predicte	-4.152142	0.002592	0.020209
1367688_at	65170	Scamp4	secretory carrier membrane protein 4	1.991781	0.002596	0.02023

1391184_at	301121	LOC30112	NA	-1.215805	0.002603	0.020268
1392901_at	367113	LOC36711	similar to RIKEN cDNA A430093J20 g	-2.079982	0.002607	0.020287
1379318_at	498662	LOC49866	similar to RIKEN cDNA 2610019F03	-1.180469	0.002609	0.020291
1379565_at	362339	Creb3l2	cAMP responsive element binding prot	1.419679	0.002613	0.020304
1391805_at	313111	RGD13103	similar to KIAA1900 protein (predicted)	2.017285	0.002614	0.020304
1370426_a_at	29693	Atp2a2	ATPase, Ca++ transporting, cardiac m	1.057899	0.002615	0.020304
1381982_at	296560	Uap1l1_pre	UDP-N-acteylglucosamine pyrophosph	1.807953	0.002625	0.02037
1371348_at	29425	Psmb5	proteasome (prosome, macropain) sub	1.020723	0.00263	0.020391
1372824_at	362484	Plekhf2_pr	pleckstrin homology domain containing	-1.343885	0.002634	0.020391
1381469_a_at	304378	Perq1_pred	PERQ amino acid rich, with GYF doma	1.99863	0.002635	0.020391
1367769_at	117017	Polr2g	polymerase (RNA) II (DNA directed) po	1.214627	0.002635	0.020391
1372843_at	363020	RGD13094	LOC363020 (predicted)	1.06018	0.002637	0.020391
1372063_at	497938	LOC49793	similar to RIKEN cDNA 4933402P03	-1.439161	0.002637	0.020391
1380512_at	291984	Dpep2	dipeptidase 2	3.729375	0.00264	0.020406
1374651_at	304077	Dopey2_pr	dopey family member 2 (predicted)	1.237309	0.002642	0.020408
1398920_at	298536	NA	NA	1.4363	0.002647	0.020423
1379374_at	295401	Prg1	plasticity related gene 1	1.736628	0.002649	0.020423
1393722_at	302288	Fem1c_pre	fem-1 homolog c (C.elegans) (predicte	-1.135761	0.002649	0.020423
1399022_at	301434	Clk1	CDC-like kinase 1	-1.740196	0.002652	0.020433
1387240_at	360420	Rdh7	retinol dehydrogenase 7	2.513546	0.002665	0.020514
1372130_at	312915	Arfgef1_pre	ADP-ribosylation factor guanine nucleo	-1.101171	0.002665	0.020514
1373878_at	81762	Rock1	Rho-associated coiled-coil forming kin	-0.993924	0.002669	0.020529
1370925_at	291411	LOC29141	similar to Potential phospholipid-transp	1.944299	0.002672	0.020542
1398343_at	300721	Dnaja4	DnaJ (Hsp40) homolog, subfamily A, m	-1.43677	0.00268	0.020592
1399019_at	364380	Abhd4_pre	abhydrolase domain containing 4 (pred	1.247344	0.002682	0.020592
1371475_at	56759	Rnase4	ribonuclease, RNase A family 4	-5.683735	0.002685	0.020598
1378543_at	312331	Nfe2l3_pre	nuclear factor, erythroid derived 2, like	-1.092438	0.002687	0.020598
1371698_at	287739	Eftud2	elongation factor Tu GTP binding dom	1.419093	0.002689	0.020598
1371478_at	296315	RGD13077	similar to RIKEN cDNA 1110008F13	0.974896	0.002689	0.020598
1374739_at	500727	Cdca4	cell division cycle associated 4	-1.337981	0.002692	0.020598
1378595_at	252855	Sfpq	splicing factor proline/glutamine rich (p	-2.067961	0.002692	0.020598
1370027_a_at	497794	Mug1	Murinoglobulin 1 homolog (mouse)	-4.412467	0.002693	0.020598
1383236_at	361927	Fxr1h	fragile X mental retardation gene 1, au	-1.622138	0.002698	0.020618
1389176_at	309008	Inpp5f_pre	inositol polyphosphate-5-phosphatase	-1.565147	0.002701	0.020622
1368368_a_at	64355	Lsr	lipolysis stimulated lipoprotein receptor	1.439296	0.002701	0.020622
1388417_at	288671	Anapc5_pr	anaphase-promoting complex subunit	0.997625	0.00271	0.020675
1377268_at	294667	RGD13105	similar to RIKEN cDNA 1200014M14	1.51189	0.002711	0.020675
1399084_at	294232	Dhx16	DEAH (Asp-Glu-Ala-His) box polypepti	1.024841	0.002717	0.020705
1367668_a_at	83792	Scd2	stearoyl-Coenzyme A desaturase 2	1.05615	0.002729	0.020789
1371237_a_at	24567	Mt1a	metallothionein 1a	-1.958076	0.002733	0.020802
1368410_at	24561	Mpg	N-methylpurine-DNA glycosylase	1.690892	0.002736	0.020815
1389093_at	29431	Pak1	p21 (CDKN1A)-activated kinase 1	-2.503832	0.002737	0.020815
1379538_at	305909	Cenpj_pred	centromere protein J (predicted)	-1.907833	0.002749	0.020881
1368045_at	171135	Slc31a1	solute carrier family 31 (copper transpo	1.890524	0.002749	0.020881
1396492_at	362966	Nfam1_pre	NFAT activating protein with ITAM mot	4.771408	0.002751	0.020881
1367995_at	24248	Cat	catalase	1.157927	0.002758	0.020907
1391643_at	78969	Trib1	tribbles homolog 1 (Drosophila)	1.135613	0.002758	0.020907
1393370_at	310698	RGD13083	similar to RIKEN cDNA 6330415M09 (	-1.588201	0.002762	0.020907
1389208_at	363495	NA	NA	-0.99668	0.002763	0.020907
1371393_at	313717	Clstn1	calsyntenin 1	1.296786	0.002766	0.020907
1373409_at	362294	RGD15599	similar to ubiquitin protein ligase E3C (	1.070287	0.002767	0.020907

1374870_at	298101	Col27a1	procollagen, type XXVII, alpha 1	-1.437923	0.00277	0.020907
1369726_at	25217	Tapbp	TAP binding protein	1.822117	0.002771	0.020907
1378458_at	304947	Pbx1_pred	pre-B-cell leukemia transcription factor	2.388627	0.002772	0.020907
1376811_a_at	299811	Cpsf6_pred	cleavage and polyadenylation specific	-1.449569	0.002772	0.020907
1373196_at	364601	Efha2	EF hand domain family, member A2	-1.123666	0.002774	0.020907
1399014_at	246772	Vps4a	vacuolar protein sorting 4a (yeast)	1.141876	0.002775	0.020907
1370269_at	24296	Cyp1a1	cytochrome P450, family 1, subfamily a	2.071859	0.002776	0.020907
1375425_at	287541	RGD13094	hypothetical LOC287541 (predicted)	1.137527	0.002777	0.020907
1370947_at	245975	Rda279	hypothetical protein RDA279	1.038507	0.002782	0.020907
1393233_at	306352	Armc6_pre	armadillo repeat containing 6 (predicte	1.142894	0.002784	0.020907
1376705_a_at	361208	Rab24	RAB24, member RAS oncogene family	1.070307	0.002784	0.020907
1372712_at	287208	Ttc1	tetratricopeptide repeat domain 1	1.403536	0.002784	0.020907
1398434_at	362286	Datf1_pred	death associated transcription factor 1	-1.660031	0.002785	0.020907
1373455_at	361531	Paf1	Paf1, RNA polymerase II associated fa	-1.282512	0.002786	0.020907
1388307_at	294421	Serinc1	serine incorporator 1	-1.178066	0.002787	0.020907
1388457_at	290635	MGC72581	similar to RIKEN cDNA 1110012M11	1.060007	0.002791	0.020921
1373442_at	362891	Os-9	amplified in osteosarcoma	1.434167	0.002802	0.020995
1394824_at	315744	Itga11_pre	integrin, alpha 11 (predicted)	-2.091918	0.002806	0.021013
1371804_at	361052	Cdadc1	cytidine and dCMP deaminase domain	-1.45404	0.00281	0.021026
1372512_at	360953	Stx18	syntaxin 18	2.099496	0.002812	0.021029
1369331_a_at	64830	Unc13b	unc-13 homolog B (C. elegans)	2.036041	0.002817	0.021058
1367861_at	79115	Evl	Ena-vasodilator stimulated phosphopro	-1.192452	0.002821	0.021064
1371718_at	252891	Sra1	steroid receptor RNA activator 1	0.984678	0.002821	0.021064
1373624_at	312846	Rassf8_pre	Ras association (RalGDS/AF-6) doma	1.07378	0.002823	0.021065
1389362_at	362524	Ptpn3	protein tyrosine phosphatase, non-rece	1.441738	0.002825	0.02107
1381775_at	499613	RGD15661	RGD1566102 (predicted)	-1.581065	0.002827	0.02107
1392562_at	287929	RGD13081	similar to RIKEN cDNA 2610001E06 (p	1.224153	0.002828	0.02107
1371822_at	306012	Polr3d	polymerase (RNA) III (DNA directed) p	1.250245	0.00283	0.021071
1367497_at	314553	Ptdss1	phosphatidylserine synthase 1	1.72647	0.002832	0.021071
1371395_at	297093	Cbx3	chromobox homolog 3 (HP1 gamma ho	-1.162075	0.002833	0.021073
1387827_x_at	64647	Hist1h2bl	histone 1, H2bl	-4.381402	0.002837	0.021086
1367567_at	117042	Rpl6	ribosomal protein L6	1.242428	0.002849	0.021161
1394129_at	113970	Magi2	membrane associated guanylate kinas	-2.199156	0.00285	0.021161
1371660_at	360784	Znhit1_pre	zinc finger, HIT domain containing 1 (p	1.253811	0.002853	0.021173
1372341_at	501039	LOC50103	NA	-1.14007	0.002857	0.021176
1394591_at	303763	Zfp207	zinc finger protein 207	-1.47992	0.002857	0.021176
1374513_at	64551	7-Sep	septin 7	-1.15451	0.00286	0.021176
1387270_at	79237	Hhex	hematopoietically expressed homeobo	-3.226775	0.002861	0.021176
1390185_at	266605	Dcps	decapping enzyme, scavenger	1.437284	0.002862	0.021176
1373811_at	365951	RGD15605	similar to heat shock protein 8 (predict	1.339096	0.002866	0.021193
1376893_at	291129	Vmp_predi	vesicular membrane protein p24 (pred	-1.023077	0.002874	0.021241
1398436_at	288482	Usp42_pre	ubiquitin specific protease 42 (predicte	2.190342	0.002876	0.021247
1368883_at	81526	Nov	nephroblastoma overexpressed gene	-1.858147	0.002882	0.021278
1374033_at	291983	Psmb10	proteasome (prosome, macropain) sub	1.317038	0.002884	0.021278
1384377_at	364995	Ddx28_pre	DEAD (Asp-Glu-Ala-Asp) box polypept	1.344762	0.002891	0.021314
1374416_at	499214	RGD15622	similar to E2-induced gene 2 protein (p	1.825367	0.002892	0.021314
1388272_at	299352	Igh-1a	immunoglobulin heavy chain 1a (serum	3.232895	0.002895	0.021328
1383324_at	500121	NA	NA	1.170576	0.002897	0.021329
1372295_at	360681	Narf	nuclear prelamin A recognition factor	1.578265	0.002901	0.021345
1390068_at	360610	Nfe2l1_pre	nuclear factor, erythroid derived 2,-like	1.311379	0.002904	0.021355
1388531_at	361940	Pgrmc2	progesterone receptor membrane com	1.080848	0.002909	0.021381

1392617_at	362455	RGD13061	similar to hypothetical protein DKFZp7	2.093499	0.00291	0.021381
1398326_at	361824	MGC1056	similar to Nur77 downstream protein 2	1.521989	0.002917	0.021382
1370464_at	170913	Abcb1a	ATP-binding cassette, sub-family B (M	3.979702	0.002918	0.021382
1390696_at	305448	RGD15664	similar to hypothetical protein A93001	1.502653	0.002919	0.021382
1370606_at	25265	P2ry1	purinergic receptor P2Y, G-protein cou	1.963856	0.00292	0.021382
1373052_at	316348	Pdcl3	phosducin-like 3	-1.136567	0.00292	0.021382
1387949_at	171518	Cyp2c70	cytochrome P450, family 2, subfamily	2.424835	0.00292	0.021382
1373824_at	292027	Cfdp1	craniofacial development protein 1	0.964407	0.002923	0.021391
1398607_at	301235	RGD13056	similar to KIAA0240 (predicted)	-1.183964	0.002927	0.021391
1370364_at	246310	Arfgap1	ADP-ribosylation factor GTPase activa	1.064451	0.002928	0.021391
1388018_at	25544	Sele	selectin, endothelial cell	3.611483	0.002928	0.021391
1373425_at	365842	LOC36584	similar to CDC-like kinase 2	1.254342	0.002932	0.02141
1371719_at	294276	Brd2	bromodomain containing 2	1.114983	0.002935	0.021416
1390987_at	498274	RGD15652	similar to novel protein (predicted)	-1.171269	0.002939	0.021433
1372136_at	306324	Tspan14_p	tetraspanin 14 (predicted)	1.479935	0.00294	0.021434
1398910_at	287155	Stub1	STIP1 homology and U-Box containing	1.150536	0.002947	0.021471
1392912_at	289144	Cacybp	calcyclin binding protein	-1.706668	0.002955	0.021483
1390928_at	500150	RGD15596	similar to tigger transposable element	-1.203799	0.002955	0.021483
1374603_at	500281	Setmar	SET domain and mariner transposase	1.00682	0.002959	0.021483
1393887_at	362866	Chpt1	choline phosphotransferase 1	-1.971407	0.002964	0.021483
1378264_at	298441	Nasp	nuclear autoantigenic sperm protein (h	-1.033227	0.002964	0.021483
1368910_at	54705	Ppm2c	protein phosphatase 2C, magnesium d	-1.12848	0.002965	0.021483
1372376_at	316013	Kif9_predic	kinesin family member 9 (predicted)	-0.962492	0.002965	0.021483
1371648_at	64470	Ddb1	damage-specific DNA binding protein 1	1.059054	0.002966	0.021483
1379475_at	499895	RGD15631	similar to RIKEN cDNA 2210009G21 (	1.023462	0.002967	0.021483
1371447_at	360914	Plac8_prec	placenta-specific 8 (predicted)	-2.20506	0.002968	0.021483
1371439_at	313052	RGD15647	similar to erythrocyte membrane protei	1.084845	0.002969	0.021483
1371953_at	29157	Ccng2_pre	cyclin G2 (predicted)	-2.675768	0.00297	0.021483
1368918_at	94203	Pgf	placental growth factor	2.868637	0.002971	0.021483
1372182_at	60416	Pfkp	phosphofructokinase, platelet	2.392761	0.002971	0.021483
1372462_at	308100	Acat2	acetyl-Coenzyme A acetyltransferase 2	-1.21673	0.002976	0.021502
1374486_at	78966	Arhgef11	Rho guanine nucleotide exchange fact	1.056895	0.002977	0.021502
1371732_at	289178	Dpt_predic	dermatopontin (predicted)	-5.902217	0.002983	0.02153
1374515_at	290303	RGD13064	similar to RIKEN cDNA 6330409N04	1.003061	0.002997	0.021619
1370920_at	296753	Srpk2_pre	serine/arginine-rich protein specific kin	-1.09985	0.003001	0.021631
1379982_at	304157	Nrip1_pred	nuclear receptor interacting protein 1 (	-1.380444	0.003002	0.021631
1367691_at	85332	Prkcdbp	protein kinase C, delta binding protein	1.372611	0.003004	0.021635
1375854_at	503000	LOC50300	similar to beta-catenin-interacting prote	1.359569	0.003006	0.021639
1370994_at	81917	Hip1r	huntingtin interacting protein 1 related	1.135876	0.003014	0.021685
1389089_at	294281	RT1-Ke4	RT1 class I, locus Ke4	1.258698	0.003027	0.021743
1367690_at	29435	Ssr4	signal sequence receptor 4	1.144346	0.003027	0.021743
1368403_at	81758	Rbl2	retinoblastoma-like 2	-1.888521	0.003029	0.021743
1394251_x_at	307721	Irx3_predic	Iroquois related homeobox 3 (Drosoph	2.856276	0.00303	0.021743
1373554_at	313722	Spsb1_pre	splA/ryanodine receptor domain and S	-2.568189	0.00303	0.021743
1377918_at	300748	RGD15594	similar to Stomatin-like 1 (predicted)	1.735413	0.003037	0.021778
1371540_at	295243	Krtcap2_pr	keratinocyte associated protein 2 (prec	1.016286	0.003041	0.021793
1367936_at	29398	Stk10	serine/threonine kinase 10	1.298191	0.00305	0.021846
1387892_at	29214	Tubb5	tubulin, beta 5	1.276093	0.003067	0.021958
1390977_at	295344	St7l	suppression of tumorigenicity 7-like	-1.838144	0.003073	0.021987
1387857_at	60466	Stx7	syntaxin 7	-1.12402	0.003077	0.021992
1372131_at	317396	Ubqln2_pre	ubiquilin 2 (predicted)	-1.084708	0.003077	0.021992

1391855_at	690085	LOC69008	NA	1.533767	0.003086	0.022042
1373430_at	317627	Baz2b_pre	bromodomain adjacent to zinc finger d	-1.334739	0.003087	0.022042
1371843_at	361315	Yipf5	Yip1 domain family, member 5	1.054026	0.00309	0.022051
1372108_at	500199	LOC50019	similar to RIKEN cDNA 2810422B04	1.035336	0.003092	0.022051
1397227_at	308134	Tmem35	transmembrane protein 35	2.012613	0.0031	0.022101
1373064_at	290370	Dnajc15_p	DnaJ (Hsp40) homolog, subfamily C, r	0.993075	0.003108	0.022146
1396280_at	302560	NA	NA	1.209379	0.003111	0.022152
1373933_at	310533	Rapgef2_p	Rap guanine nucleotide exchange fact	-0.973806	0.003119	0.022195
1389684_at	314171	Prpf39_pre	PRP39 pre-mRNA processing factor 3	-1.350443	0.003121	0.022203
1393823_at	316235	Polh_predi	polymerase (DNA directed), eta (RAD	2.333577	0.003125	0.022215
1392654_at	361473	LOC36147	NA	-1.109528	0.003127	0.022216
1384002_at	361929	LOC36192	similar to Potential phospholipid-transp	-1.207051	0.003128	0.022216
1378926_at	78957	Shank1	SH3 and multiple ankyrin repeat doma	1.80801	0.003134	0.022245
1368795_at	65276	Nmur1	neuromedin U receptor 1	2.1867	0.003141	0.022285
1392503_at	307410	Rbm22	RNA binding motif protein 22	-1.258274	0.003147	0.022311
1371799_at	367562	Gaa	glucosidase, alpha, acid	1.253941	0.003152	0.022336
1370953_at	360721	LOC36072	NA	1.993976	0.003157	0.022364
1386550_at	309031	Zfpn1a5_p	zinc finger protein, subfamily 1A, 5 (pre	-1.161933	0.003167	0.022409
1393907_at	287730	Pyy_mappe	peptide YY (mapped)	-2.629735	0.003167	0.022409
1376437_at	287464	Dhx33_pre	DEAH (Asp-Glu-Ala-His) box polypepti	1.694576	0.003172	0.022429
1373428_at	293668	Brms1	breast cancer metastasis-suppressor 1	1.064327	0.003177	0.022448
1368391_at	25648	Slc7a1	solute carrier family 7 (cationic amino a	-1.362422	0.003179	0.022448
1368285_at	24775	Shbg	sex hormone binding globulin	1.471108	0.003181	0.022448
1388151_at	192276	Coro7	coronin 7	1.272184	0.003181	0.022448
1378466_at	362204	Casc4_pre	cancer susceptibility candidate 4 (pred	2.080458	0.003191	0.022505
1387184_at	29134	Axin2	axin2	1.164785	0.003193	0.022505
1367554_at	25010	Scgb2a1	secretoglobin, family 2A, member 1	2.059224	0.003197	0.022522
1388733_at	304709	Bfar	bifunctional apoptosis regulator	1.23528	0.003205	0.022567
1370894_at	65132	Cldn7	claudin 7	2.328303	0.003207	0.022567
1367931_a_at	29497	Ptbp1	polypyrimidine tract binding protein 1	1.029667	0.003215	0.022614
1367987_at	81761	Rnpep	arginyl aminopeptidase (aminopeptida	1.483175	0.003217	0.022614
1386857_at	29332	Stmn1	stathmin 1	-1.942709	0.00322	0.022614
1372501_at	292019	Sf3b3_pred	splicing factor 3b, subunit 3 (predicted)	0.96283	0.003221	0.022614
1374357_at	353304	Cdc91l1	CDC91 cell division cycle 91-like 1 (S.	1.086722	0.003223	0.022614
1393458_s_at	500030	RGD15637	similar to PHD finger protein 14 isoforr	-2.264767	0.003224	0.022614
1382954_at	289596	Corin	corin	-2.248246	0.003226	0.022614
1372552_at	289312	Acbd3	acyl-Coenzyme A binding domain cont	-1.07732	0.003229	0.022614
1388787_at	303478	Ube2z	ubiquitin-conjugating enzyme E2Z (put	1.460499	0.00323	0.022614
1376293_at	303702	RGD13108	similar to RIKEN cDNA D230014K01 (	1.31683	0.003231	0.022614
1371081_at	252857	Rapgef4	Rap guanine nucleotide exchange fact	-2.45944	0.003232	0.022614
1369999_a_at	94270	Nnat	neuronatin	-1.137578	0.003235	0.022621
1387472_at	25710	Cd3d	CD3 antigen delta polypeptide	2.248968	0.003244	0.022676
1390783_at	303638	Abca8a_pr	ATP-binding cassette, sub-family A (AI	-5.83917	0.003253	0.022726
1380265_at	314228	Snapc1_pr	small nuclear RNA activating complex,	-0.980391	0.003257	0.022738
1384791_at	305571	B3gnt1_pre	UDP-GlcNAc:betaGal beta-1,3-N-acety	2.662802	0.003261	0.022757
1374299_at	304859	Dhx9_pred	DEAH (Asp-Glu-Ala-His) box polypepti	-1.618723	0.003263	0.022757
1373073_at	301618	Ppp1r7	protein phosphatase 1, regulatory (inhi	1.216066	0.003268	0.022781
1398516_at	498230	RGD15663	similar to RIKEN cDNA 0610012C01 (p	1.732138	0.003278	0.02283
1368502_at	25320	Gast	gastrin	-2.239283	0.003278	0.02283
1371327_a_at	287876	Actg1	actin, gamma, cytoplasmic 1	1.088122	0.003282	0.02284
1374009_at	362320	RGD13063	similar to RIKEN cDNA 5830415L20	-1.267002	0.003286	0.022857

1370836_at	246328	Serpina4	serine (or cysteine) proteinase inhibito	2.073627	0.003291	0.022877
1369007_at	54278	Nr4a2	nuclear receptor subfamily 4, group A,	-2.883279	0.003292	0.022877
1392911_at	503481	NA	NA	-1.142818	0.003294	0.022879
1391229_at	171358	Camk1g	calcium/calmodulin-dependent protein	1.271459	0.0033	0.022905
1387807_at	83572	Pafah1b1	platelet-activating factor acetylhydrolas	0.944953	0.003307	0.022945
1377853_at	308523	Zfp537_pre	zinc finger protein 537 (predicted)	-1.777671	0.003314	0.022961
1387000_at	56082	Gorasp1	golgi reassembly stacking protein 1	1.05881	0.003314	0.022961
1387268_at	83582	Rpo1-2	RNA polymerase 1-2	1.621474	0.003315	0.022961
1385018_at	681331	LOC68133	NA	3.864167	0.003319	0.022976
1389416_at	498192	RGD15603	RGD1560398 (predicted)	1.011635	0.00332	0.022976
1370014_at	81803	Stx4a	syntaxin 4A (placental)	1.060595	0.003328	0.023021
1399095_at	301442	Sumo1	SMT3 suppressor of mif two 3 homolog	-0.98761	0.003339	0.023067
1374338_at	361993	RGD15620	similar to RIKEN cDNA 1110038F21 (p	4.153794	0.00334	0.023067
1373741_at	304567	Pus1	pseudouridine synthase 1	1.351443	0.00334	0.023067
1392936_at	497865	RGD15654	similar to RNA binding motif protein 25	0.974178	0.003351	0.023124
1371639_at	288666	Vps29_pre	vacuolar protein sorting 29 (S. pombe)	-0.97782	0.003352	0.023124
1372854_at	311224	Ttc17	tetratricopeptide repeat domain 17	1.147796	0.003354	0.023124
1391497_at	294071	Tbc1d12_p	TBC1D12: TBC1 domain family, memb	-1.044557	0.003356	0.023124
1372643_at	309557	RGD15639	similar to protein 4.1G (predicted)	-1.75982	0.003373	0.023229
1374742_at	365216	Igsf4c_pred	immunoglobulin superfamily, member 4	1.2644	0.003374	0.023229
1372860_at	361663	MGC95092	similar to phospholysine phosphohistic	-2.039917	0.003377	0.023234
1371809_at	294230	Mrps18b	mitochondrial ribosomal protein S18B	1.298136	0.003379	0.023236
1387224_at	54248	Dgkb	diacylglycerol kinase, beta	-1.864852	0.003383	0.023252
1390289_at	309855	RGD13113	similar to RIKEN cDNA A530089I17 (p	-1.023665	0.00339	0.023286
1392973_at	499153	NA	NA	-1.843775	0.003398	0.023323
1372176_at	24680	Prkca	protein kinase C, alpha	2.347029	0.0034	0.023323
1387089_at	171050	Prei3	preimplantation protein 3	-1.0881	0.003402	0.023323
1393320_at	310019	RGD13109	similar to hypothetical protein FLJ1278	-1.018253	0.003402	0.023323
1371916_at	685059	MGC10575	NA	1.095781	0.003407	0.023345
1374497_at	314648	Ncln	nicalin homolog (zebrafish)	1.656944	0.00341	0.023354
1371432_at	287721	Vat1	vesicle amine transport protein 1 homo	1.001975	0.003412	0.023355
1387218_at	25563	Tff3	trefoil factor 3	4.097175	0.003419	0.023387
1371590_s_at	500954	LOC50095	Ubiquitin-Like 5 Protein	-0.993173	0.003436	0.023497
1388001_at	246214	Lrrc21	leucine rich repeat containing 21	2.000393	0.003447	0.023557
1370190_at	81504	Grb2	growth factor receptor bound protein 2	-1.136435	0.003451	0.023567
1374770_at	84431	Asah1	N-acylsphingosine amidohydrolase 1	-0.972755	0.003452	0.023567
1369714_at	114481	Dnajc14	DnaJ (Hsp40) homolog, subfamily C, r	1.472314	0.003456	0.023578
1388790_at	304542	RGD13108	similar to hypothetical protein D5Ertd3	1.451705	0.003457	0.023578
1374840_at	362580	RGD13056	similar to CG2919-PA (predicted)	1.128778	0.00346	0.023588
1394699_at	307376	RGD15646	similar to transcription factor ONECUT	1.639234	0.003467	0.023618
1376067_at	299805	Cnot2	CCR4-NOT transcription complex, sub	-1.090099	0.003474	0.023656
1371690_at	64187	Arl1	ADP-ribosylation factor-like 1	0.935568	0.003476	0.023656
1373639_at	290925	RGD15656	similar to RIKEN cDNA 2410022L05 (p	-1.096506	0.003479	0.023656
1370265_at	25388	Arrb2	arrestin, beta 2	1.307087	0.003479	0.023656
1389307_at	29572	Aplp1	amyloid beta (A4) precursor-like protei	-1.067091	0.003482	0.023666
1368151_at	29150	Matr3	matrin 3	-1.045708	0.003489	0.023696
1370376_a_at	83807	Csda	cold shock domain protein A	0.952086	0.003492	0.023708
1383091_at	303396	Appbp2	amyloid beta precursor protein (cytopla	1.545317	0.003501	0.023758
1373936_at	361212	Rnf44	ring finger protein 44	-0.936176	0.003508	0.023782
1372223_at	303010	Cpeb4_pre	cytoplasmic polyadenylation element b	-1.584486	0.003509	0.023782
1383752_at	314969	Nol1_predi	nucleolar protein 1 (predicted)	0.921236	0.00351	0.023782

1369989_at	64533	Pnpo	pyridoxine 5'-phosphate oxidase	1.102664	0.003516	0.023803
1368164_at	116599	Blvra	biliverdin reductase A	0.941893	0.003517	0.023803
1371066_at	170837	Snrk	SNF related kinase	1.965123	0.00352	0.023803
1373611_at	312679	Il17r_predi	interleukin 17 receptor (predicted)	1.407431	0.003521	0.023803
1372961_at	362301	MGC7299€	Unknown (protein for MGC:72996)	1.07644	0.003533	0.023875
1388309_at	117062	Hmga1	high mobility group AT-hook 1	1.759743	0.003548	0.02396
1379258_at	305351	Klhl5	kelch-like 5 (Drosophila)	-2.044682	0.00355	0.02396
1370941_at	25267	Pdgfra	platelet derived growth factor receptor,	-2.46275	0.003551	0.02396
1368710_at	60328	Mark2	MAP/microtubule affinity-regulating kin	1.473513	0.00356	0.024008
1369358_a_at	29430	Hap1	huntingtin-associated protein 1	-0.99233	0.003563	0.024015
1368643_at	171413	Spata6	spermatogenesis associated 6	-1.568974	0.003566	0.024023
1370262_at	170910	Mtdh	metadherin	1.378935	0.003573	0.024057
1368361_a_at	117063	Ptpn2	protein tyrosine phosphatase, non-rece	-1.022705	0.003576	0.024065
1369335_at	64555	Kpl2	KPL2 protein	2.640311	0.003583	0.024084
1382420_at	296145	RGD13101	similar to hypothetical protein FLJ3744	-1.353732	0.003584	0.024084
1389652_at	290706	RGD13117	similar to 2700029M09Rik protein (pre	-1.087516	0.003584	0.024084
1383395_at	298607	Agmat	agmatine ureohydrolase (agmatinase)	3.928642	0.003588	0.024099
1370814_at	266686	Dhrs4	dehydrogenase/reductase (SDR family	1.823446	0.003594	0.024125
1393752_at	315606	Mll_mappe	myeloid/lymphoid or mixed-lineage leu	2.832079	0.003607	0.024201
1372009_at	313047	Yars	tyrosyl-tRNA synthetase	1.313457	0.003616	0.024244
1385869_at	305083	Zfp281	zinc finger protein 281	-2.026827	0.003618	0.024244
1380824_at	306548	Hook3	hook homolog 3 (Drosophila)	1.384501	0.00362	0.024244
1373532_at	308543	Plekhf1	pleckstrin homology domain containing	2.365017	0.003621	0.024244
1388960_at	294504	Pyp_mapp€	pyrophosphatase (mapped)	1.069299	0.003622	0.024244
1368100_at	89841	Pcyt2	phosphate cytidylyltransferase 2, ethar	1.231703	0.003637	0.024329
1382410_at	288378	Cd209b	CD209b antigen	1.965184	0.003643	0.024355
1375299_at	287447	Dullard	Dullard homolog (Xenopus laevis)	1.847947	0.003649	0.024386
1372913_at	307652	RGD13074	similar to RIKEN cDNA 2310065K24	1.228594	0.00367	0.024514
1368500_a_at	29481	Rgs9	regulator of G-protein signaling 9	1.111492	0.003678	0.024547
1367982_at	65155	Alas1	aminolevulinic acid synthase 1	1.040277	0.003679	0.024547
1372287_at	305935	Mtmr6_pre	myotubularin related protein 6 (predict	-0.991051	0.003685	0.024577
1369158_at	24247	Casr	calcium-sensing receptor	-1.840366	0.003689	0.024592
1378326_at	366012	LOC36601	similar to RIKEN cDNA G430055L02	1.275758	0.003694	0.024599
1371567_at	291450	Aldh7a1	aldehyde dehydrogenase family 7, me	1.618599	0.003694	0.024599
1387059_at	54348	Stk39	serine/threonine kinase 39, STE20/SP	-1.303465	0.003697	0.024606
1367710_at	29614	Psme2	proteasome (prosome, macropain) 28	0.93665	0.003702	0.024627
1367569_at	29236	Rpsa	ribosomal protein SA	1.011589	0.003709	0.02465
1367516_at	641528	Dtnbp1	NA	0.968852	0.003711	0.02465
1379430_at	311437	Rassf2	Ras association (RalGDS/AF-6) doma	5.402047	0.003712	0.02465
1379949_at	308140	Tfb1m	transcription factor B1, mitochondrial	1.011045	0.003713	0.02465
1389644_at	689865	LOC68986	NA	-2.00411	0.003715	0.02465
1379188_at	366980	Arid2_pred	AT rich interactive domain 2 (Arid-rfx li	0.974647	0.003722	0.024679
1398812_at	94198	Psmb1	proteasome (prosome, macropain) sub	1.214513	0.003723	0.024679
1396013_at	305457	Letm1	leucine zipper-EF-hand containing trar	1.592985	0.003732	0.024728
1398417_at	316131	Fem1a	feminization 1 homolog a (C. elegans)	1.117613	0.00374	0.02477
1371869_at	29674	Psma7	proteasome (prosome, macropain) sub	1.244283	0.003746	0.024798
1381554_at	313653	RGD13085	similar to hypothetical protein FLJ3278	-2.837353	0.003752	0.024812
1375485_at	266813	Prrx1	paired related homeobox 1	-3.562085	0.003752	0.024812
1398860_at	25490	Nedd8	neural precursor cell expressed, devel	0.943899	0.003754	0.024812
1373897_at	116685	Lmnb1	lamin B1	-1.219367	0.003759	0.02483
1377146_at	117064	Vip	vasoactive intestinal polypeptide	2.878379	0.003761	0.02483

1377621_at	287398	Map2k4	mitogen activated protein kinase kinas	-1.159196	0.003765	0.024844
1398941_at	363005	Pcbp2	poly(rC) binding protein 2	-0.967379	0.003769	0.024848
1377035_at	317161	Mospd4_pı	motile sperm domain containing 4 (pre	2.313398	0.003769	0.024848
1386990_at	117278	Ebp	phenylalkylamine Ca2+ antagonist (em	0.997742	0.003774	0.024852
1373107_at	360475	Alg1_predi	asparagine-linked glycosylation 1 hom	2.005494	0.003776	0.024852
1374812_at	498331	LOC49833	NA	-2.55431	0.003777	0.024852
1392511_at	170844	Taf2	TAF2 RNA polymerase II, TATA box b	-1.075865	0.003778	0.024852
1383627_a_at	316123	Gtf2f1	general transcription factor IIF, polype	-1.064551	0.00378	0.024852
1377691_at	310710	Sec22l1	SEC22 vesicle trafficking protein-like 1	1.337817	0.003781	0.024852
1368331_at	81652	Ctbs	chitobiase, di-N-acetyl-	-1.241227	0.003783	0.024855
1380693_at	501560	RGD15638	similar to ribosomal protein S6 kinase	1.955604	0.003786	0.02486
1385845_at	502617	RGD15648	similar to divalent cation tolerant protei	1.078769	0.003794	0.02487
1388770_at	365797	RGD13048	similar to RIKEN cDNA 1810045K17	0.936687	0.003794	0.02487
1369986_at	24439	Hagh	hydroxyacyl glutathione hydrolase	1.213608	0.003794	0.02487
1395311_at	170818	Icmt	isoprenylcysteine carboxyl methyltrans	1.692484	0.003795	0.02487
1371846_at	361094	Phgdhl1	phosphoglycerate dehydrogenase like	1.138259	0.003801	0.024894
1382109_at	298342	2610020o0	nuclear NF-kappaB activating protein	2.307918	0.003802	0.024894
1383370_at	286895	Brinp2	BMP/retinoic acid-inducible neural-spe	2.562592	0.003805	0.024901
1380525_at	310326	Arse	arylsulfatase E (chondrodysplasia punc	-1.256509	0.003809	0.024901
1374017_at	365377	RGD15627	similar to tripartite motif protein 50 (pre	-1.748381	0.003809	0.024901
1377187_at	313069	RGD13076	similar to 3000004N20Rik protein (pre	-1.578776	0.003813	0.024914
1390579_at	290686	RGD13052	similar to RIKEN cDNA 1810029B16 (p	1.403123	0.003819	0.024938
1383779_at	309673	RGD13056	similar to RIKEN cDNA 2600005C20 (p	1.186113	0.003821	0.024938
1377920_at	294708	Sgtb	small glutamine-rich tetratricopeptide r	1.164671	0.003822	0.024938
1388633_at	309454	RGD13093	similar to RIKEN cDNA 4930538D17	1.395071	0.003833	0.024993
1372947_at	81748	Pls3	plastin 3 (T-isoform)	1.139096	0.003841	0.025036
1394590_at	305751	Nkiras1_pr	NFKB inhibitor interacting Ras-like pro	1.69438	0.003845	0.025044
1367894_at	64194	Insig1	insulin induced gene 1	-1.01291	0.003847	0.025044
1374548_at	497901	LOC49790	similar to Liver-expressed antimicrobia	-1.930233	0.003848	0.025044
1370001_at	117103	Rab8a	RAB8A, member RAS oncogene family	0.946478	0.003852	0.02506
1378535_at	364510	Phf7	PHD finger protein 7	1.276431	0.003859	0.02509
1387206_at	65196	B4galt6	UDP-Gal:betaGlcNAc beta 1,4-galacto	-1.319319	0.003861	0.025095
1369024_at	80754	Rabep2	rabaptin, RAB GTPase binding effecto	1.448163	0.003864	0.025097
1377254_a_at	315036	Cohh1_pre	Cohen syndrome homolog 1 (predicted	-1.755102	0.003867	0.025097
1374778_at	25423	Ctsc	cathepsin C	-2.148683	0.003867	0.025097
1376793_at	306687	RGD13083	similar to RIKEN cDNA 5730414C17 (p	-0.976053	0.003871	0.025105
1373640_at	364064	Pycr2	pyrroline-5-carboxylate reductase fami	0.934854	0.003877	0.025135
1373479_at	24674	Ppp3ca	protein phosphatase 3, catalytic subun	-0.991332	0.003885	0.025173
1388882_at	299104	Fkbp3_pre	FK506 binding protein 3 (predicted)	-0.997041	0.003889	0.025173
1388371_at	300445	Prkcsh_pre	protein kinase C substrate 80K-H (pred	0.983524	0.00389	0.025173
1392257_at	501706	LOC50170	hypothetical protein LOC501706	2.419393	0.003891	0.025173
1369909_s_at	245966	Tm6p1	fasting-inducible integral membrane pr	1.40614	0.003899	0.025216
1379972_at	309790	RGD13086	similar to 1700060H10Rik protein	-2.006495	0.003904	0.025232
1389034_at	312688	Usp18	ubiquitin specific peptidase 18	1.24621	0.003913	0.02526
1386927_at	25413	Cpt2	carnitine palmitoyltransferase 2	1.628678	0.003914	0.02526
1388821_at	313974	RGD15644	similar to Tribbles homolog 2 (predicte	-1.725695	0.003914	0.02526
1398869_at	117262	Psmc4	proteasome (prosome, macropain) 26S	1.039818	0.003917	0.02526
1388816_at	25252	Dlgh1	discs, large homolog 1 (Drosophila)	-1.456147	0.003917	0.02526
1388394_at	292023	Aars	alanyl-tRNA synthetase	1.413217	0.003922	0.025261
1370823_at	83837	Bambi	BMP and activin membrane-bound inh	-1.095076	0.003923	0.025261
1380449_at	500367	RGD15609	similar to C230080I20Rik protein (prec	-0.991369	0.003923	0.025261

1384858_a_at	299900	RGD13071	similar to RIKEN cDNA D530033C11 (	1.850481	0.003926	0.025266
1374133_at	362586	Trit1_predi	tRNA isopentenyltransferase 1 (predic	0.930821	0.003929	0.025273
1398361_at	498938	NA	NA	1.367372	0.003931	0.025275
1374785_at	296985	RGD15653	similar to CD69 antigen (p60, early T-c	1.017216	0.003942	0.025331
1373681_at	300741	Mpi_mappe	mannose phosphate isomerase (mapp	1.196578	0.003946	0.025344
1371868_at	295334	Bcas2_pre	breast carcinoma amplified sequence :	-1.224404	0.003957	0.025401
1379472_at	353255	Nadsyn1	NAD synthetase 1	1.075156	0.003967	0.025459
1369962_at	81643	Atic	5-aminoimidazole-4-carboxamide ribor	1.408287	0.003973	0.025482
1373861_at	361089	Ndfip2_pre	Nedd4 family interacting protein 2 (pre	-1.380677	0.003975	0.025482
1372611_at	298377	RGD13052	similar to RIKEN cDNA 2010305A19 (p	2.063779	0.003983	0.025518
1372073_at	290669	Gatad2a	GATA zinc finger domain containing 2A	1.070751	0.003984	0.025518
1383624_at	498014	RGD1565C	similar to hypothetical protein LOC284	-1.452584	0.003995	0.025565
1368528_at	171485	Mic2l1	MIC2 like 1	-1.432621	0.003996	0.025565
1370170_at	117280	Hnrpu	heterogeneous nuclear ribonucleoprote	-0.956109	0.004004	0.025605
1374691_at	292077	Sult5a1_pr	sulfotransferase family 5A, member 1 (	1.185937	0.004006	0.025605
1382063_at	363494	Gla_mappe	galactosidase, alpha (mapped)	1.483177	0.004008	0.025607
1375846_at	289424	Xpr1_predi	xenotropic and polytropic retrovirus rec	-1.035817	0.004011	0.025616
1389355_at	498256	Ier5	immediate early response 5	-1.14807	0.004015	0.025629
1376190_at	29741	Pik3r2	phosphatidylinositol 3-kinase, regulato	1.286191	0.004025	0.025671
1386902_at	83532	Vdac3	voltage-dependent anion channel 3	0.953905	0.004026	0.025671
1371656_at	29374	Cct4	chaperonin subunit 4 (delta)	0.90816	0.004028	0.025672
1372030_at	362789	Zfyve21_pr	zinc finger, FYVE domain containing 2	1.918501	0.004031	0.025681
1368614_at	171078	Wbscr14	Williams-Beuren syndrome chromosor	2.15494	0.004038	0.02571
1373204_at	297077	RGD13107	hypothetical LOC297077	-2.025597	0.00404	0.02571
1378298_at	298591	RGD13101	hypothetical LOC298591 (predicted)	-2.337872	0.004043	0.02572
1374808_at	313525	RGD13098	similar to hypothetical protein FLJ2115	0.884147	0.004049	0.025746
1372158_at	246233	Lrp16	LRP16 protein	-2.09166	0.004056	0.025774
1399102_at	303740	RGD13063	similar to hypothetical protein MGC155	1.172827	0.004061	0.025788
1389325_at	494345	MGC72992	similar to programmed cell death 10	-1.206673	0.004062	0.025788
1374062_x_at	59075	Gprk5	G protein-coupled receptor kinase 5	-1.285109	0.004066	0.025792
1372657_at	315286	Tmem106c	transmembrane protein 106C	-1.640773	0.004067	0.025792
1372982_at	29748	Ppp3r1	protein phosphatase 3, regulatory subu	0.911858	0.004068	0.025792
1374693_at	315760	Parp16	poly (ADP-ribose) polymerase family, r	0.889698	0.004081	0.025859
1383793_at	312654	RGD13119	hypothetical LOC312654 (predicted)	-1.109459	0.004085	0.025872
1374365_at	292041	Wwox_pred	WW domain-containing oxidoreductase	2.062481	0.004089	0.025887
1393954_at	308854	Chrdl2_pre	chordin-like 2 (predicted)	2.230408	0.004092	0.025887
1375864_at	313478	Cc2d1b	coiled-coil and C2 domain containing 1	1.064255	0.004094	0.025887
1390217_at	56785	Garnl1	GTPase activating RANGAP domain-li	-1.299228	0.004096	0.025887
1391684_at	363206	Tmem14a_	transmembrane protein 14A (predicted	-1.329338	0.004097	0.025887
1388842_at	501099	RGD15597	similar to serum response factor (predi	-1.206794	0.004104	0.025922
1389131_at	360629	Nt5c3l	5'-nucleotidase, cytosolic III-like	1.182225	0.004109	0.025935
1373763_at	311872	Zfp297b	zinc finger protein 297B	-1.236322	0.004113	0.025935
1382113_at	494340	MGC72612	similar to expressed sequence AI4491	-1.8658	0.004114	0.025935
1398986_at	296350	Serinc3	serine incorporator 3	-1.009839	0.004116	0.025935
1374292_at	303749	RGD1305C	similar to RIKEN cDNA 1110031I02	-1.491348	0.004116	0.025935
1368234_at	83471	Prep	prolyl endopeptidase	1.16757	0.004124	0.025974
1371920_at	287544	Poldip2_pr	polymerase (DNA-directed), delta inter	1.357247	0.00413	0.02599
1388729_at	361568	Rras_predi	Harvey rat sarcoma oncogene, subgro	1.225315	0.004131	0.02599
1387917_at	246314	Tor1aip1	torsin A interacting protein 1	1.240592	0.00414	0.026036
1371782_at	313211	Nipsnap3a	nipsnap homolog 3A (C. elegans)	1.265172	0.004143	0.026043
1372906_at	295930	RGD13095	similar to hypothetical protein MGC408	-1.503061	0.004155	0.026071

1377121_at	305645	Dlg5_predi	discs, large homolog 5 (Drosophila) (p	-1.331868	0.004156	0.026071
1372706_at	294673	Hexb	hexosaminidase B	-0.940529	0.004156	0.026071
1370005_at	80773	Cyb5b	cytochrome b5 type B	1.064217	0.004157	0.026071
1371352_at	114637	Hmgn2	high mobility group nucleosomal bindir	-1.009655	0.004157	0.026071
1372894_at	363136	Tmem115_	transmembrane protein 115 (predicted	1.854184	0.004163	0.026094
1392891_at	304218	Aprin_pred	androgen-induced proliferation inhibito	-0.91529	0.004171	0.026122
1383311_at	310677	RGD13081	similar to zinc finger protein Cezanne;	-1.099626	0.004172	0.026122
1375637_at	364154	RGD13111	similar to RIKEN cDNA 1110003E01	2.382138	0.004189	0.026217
1376804_at	315840	RGD15606	similar to Myosin VI (predicted)	-1.32737	0.004191	0.026217
1373013_at	361309	LOC36130	similar to polyadenylate-binding protei	-0.947886	0.004193	0.026218
1367973_at	24770	Ccl2	chemokine (C-C motif) ligand 2	-4.380292	0.004198	0.026239
1375163_at	79434	Rab11b	RAB11B, member RAS oncogene fam	-1.077171	0.004201	0.026246
1368402_at	81655	Dncli2	dynein, cytoplasmic, light intermediate	-0.989498	0.004205	0.026255
1392956_at	296550	RGD13048	similar to RIKEN cDNA 2810443J12 (p	1.437934	0.00421	0.026278
1388655_at	311802	Ssna1_pre	Sjogren's syndrome nuclear autoantige	0.935039	0.004212	0.026278
1370343_at	245976	Xab2	XPA binding protein 2	1.041387	0.004223	0.026329
1382326_at	83631	Dedd	death effector domain-containing	0.963193	0.004233	0.026372
1393619_at	360917	Cnot6l_pre	CCR4-NOT transcription complex, sub	-1.462606	0.004233	0.026372
1383245_at	362740	Mbip_predi	MAP3K12 binding inhibitory protein 1 (	-1.742457	0.004238	0.026384
1376963_at	314862	Dyrk2_pred	dual-specificity tyrosine-(Y)-phosphory	-1.987751	0.004239	0.026384
1368044_at	24765	Scg2	secretogranin 2	-1.19628	0.004242	0.026388
1388382_at	361985	LOC36198	similar to NICE-3	-0.936969	0.004251	0.026435
1372883_at	362217	Cenpb_pre	centromere autoantigen B (predicted)	0.966682	0.004254	0.026438
1372228_at	288527	Asmtl_prec	acetylserotonin O-methyltransferase-lil	1.494146	0.004269	0.026503
1369518_at	60664	Pik3r3	phosphatidylinositol 3 kinase, regulato	1.453033	0.004271	0.026503
1375974_at	305240	RGD13095	similar to RNA-binding protein isoform	-1.039108	0.004272	0.026503
1388429_at	499391	LOC49939	NA	1.06143	0.004272	0.026503
1368071_at	171451	Mosc2	MOCO sulphurase C-terminal domain	1.370965	0.004276	0.026503
1375451_at	309167	Pcnxl3	pecanex-like 3 (Drosophila)	1.227539	0.004276	0.026503
1383903_at	364901	St8sia5	ST8 alpha-N-acetyl-neuraminide alpha	4.967831	0.00428	0.026512
1379461_at	301076	RGD15591	similar to ZFP (predicted)	1.498746	0.004288	0.026538
1391902_at	24959	Pgam2	phosphoglycerate mutase 2	1.47588	0.004288	0.026538
1399009_at	308837	RGD13050	similar to RIKEN cDNA 2610034N24 (p	1.109414	0.004293	0.026556
1387843_at	24373	Fst	follistatin	1.081197	0.004298	0.026574
1374361_at	315173	RGD13066	similar to bK1191B2.3.1 (PUTATIVE n	0.87223	0.004306	0.026614
1396797_at	292681	Nova2_pre	neuro-oncological ventral antigen 2 (pr	-1.590294	0.004312	0.026637
1373111_at	306260	Capn7	calpain 7	-0.932253	0.004323	0.026691
1389996_at	290705	Nek1_pred	NIMA (never in mitosis gene a)-relatec	-1.712465	0.004335	0.026756
1388555_at	287474	Txnl5_prec	thioredoxin-like 5 (predicted)	1.512375	0.004344	0.026785
1389481_at	294311	RGD73506	similar to GI:13385412-like protein spli	1.151795	0.004347	0.026785
1398936_at	299027	Eif2s3x	eukaryotic translation initiation factor 2	0.921768	0.004347	0.026785
1387412_at	116723	Pip5k2a	phosphatidylinositol-4-phosphate 5-kin	1.043129	0.004348	0.026785
1388304_at	294964	Ndufb5_pre	NADH dehydrogenase (ubiquinone) 1	0.998787	0.00435	0.026785
1372746_at	306348	RGD15662	similar to RIKEN cDNA 2810428I15 (p	0.973299	0.004359	0.026824
1371796_at	295701	Tmx2	thioredoxin-related transmembrane prc	0.906021	0.004365	0.026844
1389406_at	369018	Ldhal6b	lactate dehydrogenase A-like 6B	0.893541	0.004366	0.026844
1398252_at	29470	Mecr	mitochondrial trans-2-enoyl-CoA reduc	1.687569	0.00437	0.026857
1379402_at	170924	Abcc4	ATP-binding cassette, sub-family C (C	1.470837	0.004373	0.026863
1372028_at	306768	RGD13057	similar to Protein CGI-117 (Protein HS	0.909349	0.004377	0.026876
1387671_at	81779	Sctr	secretin receptor	-3.037309	0.00438	0.02688
1390434_at	246756	Tradd	TNFRSF1A-associated via death doma	1.292375	0.004388	0.026916

1370317_at	245960	LOC24596	potassium channel regulator 1	-0.870964	0.004394	0.026941
1373667_at	311844	Ccbl1	cysteine conjugate-beta lyase 1	0.916696	0.004397	0.026947
1385518_at	313776	RGD13049	similar to RIKEN cDNA 2310042D19	2.145283	0.0044	0.026955
1382919_at	306009	Slc39a14_p	solute carrier family 39 (zinc transporte	1.730276	0.004405	0.026967
1383271_at	314799	Ccdc59_pr	coiled-coil domain containing 59 (predi	-1.7821	0.004407	0.026967
1387307_at	29301	Hal	histidine ammonia lyase	1.639835	0.004408	0.026967
1375728_at	361063	Entpd4_pre	ectonucleoside triphosphate diphospho	1.493177	0.00442	0.027023
1379369_at	315259	Prickle1	prickle-like 1 (Drosophila)	-1.001454	0.004423	0.02703
1368330_at	114512	Aatf	apoptosis antagonizing transcription fa	1.122178	0.004429	0.027044
1373248_at	304529	RGD15658	similar to hypothetical protein FLJ2067	-0.951032	0.00443	0.027044
1381171_at	364858	Stk32a_pre	serine/threonine kinase 32A (predicted	1.922971	0.004431	0.027044
1378763_at	500947	LOC50094	hypothetical gene supported by BC088	-2.078803	0.004436	0.027052
1381201_at	365657	Marveld2_p	MARVEL (membrane-associating) dom	1.486415	0.004436	0.027052
1373244_at	303567	RGD13047	similar to RIKEN cDNA 2010008E23 g	0.973142	0.00444	0.027061
1376932_at	499747	RGD15608	similar to RIKEN cDNA 2310002J15 (p	1.721324	0.004444	0.027061
1372928_at	500289	NA	NA	-1.450453	0.004444	0.027061
1367879_at	80278	Cdk5rap3	CDK5 regulatory subunit associated pr	1.612779	0.004449	0.027076
1378637_at	313166	Nfx1	nuclear transcription factor, X-box binc	-1.046564	0.004455	0.027102
1375495_at	503009	LOC50300	NA	1.263611	0.00447	0.027183
1398854_at	64307	Rpl24	ribosomal protein L24	-1.122443	0.004473	0.027186
1376489_at	85384	Sos2	son of sevenless homolog 2 (Drosophi	-1.249301	0.004483	0.027234
1371383_at	293674	Drap1_pre	Dr1 associated protein 1 (negative cofa	1.071142	0.00449	0.027258
1379257_at	307514	Epb4.1l4a_	erythrocyte protein band 4.1-like 4a (pr	-1.705454	0.004492	0.027258
1392476_at	500351	RGD15623	histone H4 variant H4-v.1 (predicted)	0.991111	0.004493	0.027258
1386952_a_at	116659	Dncic2	dynein, cytoplasmic, intermediate chai	-0.90013	0.004496	0.027258
1367955_at	50866	Rab4b	RAB4B, member RAS oncogene family	1.710467	0.004497	0.027258
1370902_at	286921	Akr1b8	aldo-keto reductase family 1, member	7.85216	0.004499	0.027258
1371778_at	308051	Rnf146	ring finger protein 146	-1.302478	0.004508	0.027299
1372142_at	288919	Asna1	arsA arsenite transporter, ATP-binding	1.073947	0.00451	0.027299
1368327_at	171443	Slc12a9	solute carrier family 12 (potassium/chlo	1.333218	0.00452	0.027345
1370964_at	25698	Ass	argininosuccinate synthetase	2.012105	0.004527	0.027379
1372563_at	501194	LOC50119	NA	-1.191647	0.00453	0.027385
1395223_at	306589	MGC94736	similar to hypothetical protein MGC350	-1.30616	0.004538	0.027421
1371527_at	25314	Emp1	epithelial membrane protein 1	-2.739961	0.004544	0.027439
1367744_at	113947	Maged2	melanoma antigen, family D, 2	-1.252481	0.004547	0.027439
1398963_at	293345	Taf10_pred	TAF10 RNA polymerase II, TATA box	0.853873	0.00455	0.027439
1367848_at	29167	Dctn1	dynactin 1	1.280085	0.00455	0.027439
1370745_at	25548	Slc34a1	solute carrier family 34 (sodium phosp	1.050481	0.004555	0.027461
1390602_a_at	499749	LOC49974	similar to RIKEN cDNA C430004E15	1.003192	0.004566	0.027509
1390281_a_at	362810	Coq10a_pr	coenzyme Q10 homolog A (yeast) (pre	-0.952589	0.004569	0.027509
1370275_at	171374	Atp5b	ATP synthase, H+ transporting, mitoch	0.874633	0.00457	0.027509
1393026_at	364303	Rnase1_pr	ribonuclease, RNase A family, 1 (panc	-4.647238	0.004573	0.027517
1380478_at	501017	NA	NA	2.437168	0.004578	0.027519
1368097_a_at	116644	Rtn1	reticulon 1	-1.283285	0.004579	0.027519
1388391_at	363441	Ndufa1_pre	NADH dehydrogenase (ubiquinone) 1	-0.856055	0.00458	0.027519
1392579_at	363227	RGD13066	similar to 5830411E10Rik protein	1.871948	0.004588	0.027556
1367462_at	29156	Capns1	calpain, small subunit 1	1.026404	0.004593	0.027575
1371742_at	361574	Snrp70_pre	U1 small nuclear ribonucleoprotein pol	1.046865	0.004601	0.027605
1377703_at	309419	Pip5k1a	phosphatidylinositol-4-phosphate 5-kin	-0.851041	0.004602	0.027605
1388748_at	298875	Laptm4a	lysosomal-associated protein transmer	1.171856	0.00462	0.027695
1381537_at	171549	Klc3	kinesin light chain 3	-1.448994	0.004622	0.027695

1369131_at	25549	Slc18a2	solute carrier family 18 (vesicular mon	1.865324	0.004627	0.027695
1381966_at	361475	NA	NA	2.383137	0.004627	0.027695
1371834_at	299850	Dctn2	dynactin 2	1.018047	0.004628	0.027695
1398663_at	499094	LOC49909	similar to zinc finger protein 61	-1.021171	0.004632	0.027701
1382093_at	289382	RGD1308C	similar to DKFZP434B168 protein (pre	1.372017	0.004633	0.027701
1373672_at	361241	MGC9401(	similar to SPI6	-1.837642	0.004641	0.027734
1370207_at	171082	Atp5g2	ATP synthase, H+ transporting, mitoch	1.207192	0.004647	0.02775
1388562_at	296128	Stard7_pre	START domain containing 7 (predicted	0.93172	0.004648	0.02775
1372554_at	316335	RGD13092	similar to RW1 protein (predicted)	-1.227276	0.00465	0.02775
1379829_at	298513	Mycbp_pre	c-myc binding protein (predicted)	-0.905631	0.004654	0.027762
1380009_at	362779	Vrk1	vaccinia related kinase 1	-1.070407	0.004658	0.027773
1375552_at	498351	LOC49835	similar to signal recognition particle,72	1.283513	0.004665	0.027807
1379075_at	313997	Oact2	O-acyltransferase (membrane bound)	-1.529451	0.00467	0.027815
1370709_at	246296	Lrrc15	leucine rich repeat containing 15	-1.371425	0.004671	0.027815
1382945_at	290500	RGD13047	similar to cDNA sequence BC006662	1.161686	0.00468	0.027859
1378433_at	310200	RGD15642	similar to RIKEN cDNA 5730557B15 (p	1.608363	0.004685	0.027873
1389755_at	619566	LOC61956	similar to transcription factor RAM2	-1.224993	0.004687	0.027873
1367560_at	64205	Arbp	acidic ribosomal phosphoprotein P0	0.949822	0.004691	0.027884
1377576_at	310856	Ppa2_pred	pyrophosphatase (inorganic) 2 (predict	0.946048	0.0047	0.027926
1372919_at	290843	Agpat6	1-acylglycerol-3-phosphate O-acyltran	0.942417	0.004702	0.027926
1377987_at	363858	RGD15639	similar to trafficking protein particle cor	1.445278	0.004706	0.027935
1379693_at	84409	Robo2	roundabout homolog 2 (Drosophila)	-1.61894	0.004708	0.027936
1368646_at	50658	Mapk9	mitogen-activated protein kinase 9	2.965094	0.004713	0.027952
1398820_at	117558	Mylk2	myosin light chain kinase 2, skeletal m	-1.549314	0.004715	0.027954
1367547_at	305913	LOC30591	NA	-0.999695	0.004727	0.028009
1399091_at	298584	Capzb	capping protein (actin filament) muscle	1.156788	0.004729	0.02801
1382085_at	363289	Mterfd2	MTERF domain containing 2	0.964686	0.004743	0.028083
1390803_at	317612	Htatsf1_pre	HIV TAT specific factor 1 (predicted)	-1.346977	0.004747	0.028092
1376502_at	298851	RGD13092	similar to putative protein, with at least	0.872051	0.004749	0.028092
1371623_at	367234	NA	NA	-1.284534	0.004754	0.028106
1389130_at	361944	Elf2	E74-like factor 2	-1.277034	0.004756	0.028109
1370884_at	29270	Spr	sepiapterin reductase	1.328697	0.004763	0.028134
1398958_at	360496	Narfl	nuclear prelamin A recognition factor-li	1.254886	0.00477	0.028157
1392590_at	305156	Arhgap24	Rho GTPase activating protein 24	-1.401346	0.004771	0.028157
1388857_at	362226	Sec23b_pr	SEC23B (S. cerevisiae) (predicted)	1.06403	0.004777	0.028184
1388803_at	288923	Dhps	deoxyhypusine synthase	1.024035	0.004781	0.028193
1369777_a_at	171093	Shank2	SH3/ankyrin domain gene 2	-2.316437	0.004787	0.028208
1398953_at	300036	Tsta3_prec	tissue specific transplantation antigen	1.073867	0.004788	0.028208
1370341_at	24334	Eno2	enolase 2, gamma	1.757866	0.00479	0.028208
1373150_at	305685	Comtd1_pr	catechol-O-methyltransferase domain	1.18402	0.004793	0.028213
1372781_at	310785	Wdr47	WD repeat domain 47	-1.050226	0.004809	0.028294
1388504_at	314949	MGC11637	NA	-0.943779	0.004815	0.028316
1370976_at	171092	G3bp	Ras-GTPase-activating protein SH3-do	-0.959004	0.004818	0.028316
1382265_at	306306	RGD13046	similar to KIAA1128 protein (predicted)	1.04215	0.004819	0.028316
1388528_at	292747	Fbl	fibrillarin	0.990415	0.004832	0.028376
1386897_at	60421	Hrmt1l2	heterogeneous nuclear ribonucleoprote	0.857524	0.004834	0.028376
1374448_at	313488	Reck_pred	reversion-inducing-cysteine-rich protei	-2.087263	0.004841	0.028407
1371724_at	300689	Rexo2	REX2, RNA exonuclease 2 homolog (S	1.190799	0.004847	0.02843
1391597_at	498434	RGD15663	similar to G protein-coupled receptor G	-2.420673	0.004851	0.028432
1373018_at	315327	RGD15615	similar to hypothetical protein FLJ1480	0.940953	0.004853	0.028432
1377457_a_at	300652	Sorl1_pred	sortilin-related receptor, L(DLR class) /	1.424577	0.004857	0.028432

1391134_at	306607	Tmco3_pre	transmembrane and coiled-coil domai	1.168709	0.004858	0.028432
1373719_at	170920	Map4k3	mitogen-activated protein kinase kinas	-0.982249	0.004858	0.028432
1387492_at	24546	Slco2a1	solute carrier organic anion transporte	2.789983	0.004865	0.028461
1367487_at	494342	B4galt3	UDP-Gal:betaGlcNAc beta 1,4-galacto	1.018188	0.004876	0.028505
1384020_at	316275	RGD13117	similar to putative membrane steroid re	-3.38416	0.004877	0.028505
1378925_at	25620	Crem	cAMP responsive element modulator	-1.242525	0.004883	0.028524
1367644_at	25289	Adcy6	adenylate cyclase 6	-0.88381	0.004891	0.028543
1372844_at	94268	Efna1	ephrin A1	-1.760254	0.004892	0.028543
1377613_at	300463	RGD13073	similar to RIKEN cDNA 5031400M07	-0.911338	0.004893	0.028543
1379544_at	301447	RGD13113	similar to A530083I02Rik protein (pred	1.008722	0.004895	0.028543
1399063_at	313658	Rbaf600	ZUBR1	2.335636	0.004909	0.028614
1383135_at	25054	Ntrk2	neurotrophic tyrosine kinase, receptor,	2.053194	0.004915	0.028639
1389876_at	287005	Camk2n1	calcium/calmodulin-dependent protein	0.954466	0.004919	0.028651
1367613_at	117254	Prdx1	peroxiredoxin 1	1.18221	0.004925	0.028657
1385994_at	366261	Spo11_pre	sporulation protein, meiosis-specific, S	-1.579929	0.004926	0.028657
1370985_at	114509	Mapk7	mitogen-activated protein kinase 7	-2.277139	0.004927	0.028657
1370180_at	94267	Nudt4	nudix (nucleoside diphosphate linked r	-0.945686	0.004935	0.028693
1383496_at	500409	RGD15604	similar to hypothetical protein FLJ2017	0.999826	0.00494	0.028705
1388602_at	54249	Cfd	complement factor D (adipsin)	3.17669	0.004942	0.028705
1371802_at	290659	Cope_pred	coatomer protein complex, subunit eps	0.923521	0.004946	0.028718
1372840_at	310330	NA	NA	-1.179344	0.004948	0.028718
1396831_at	310405	Glrp1_prec	glutamine repeat protein 1 (predicted)	1.545519	0.004953	0.028731
1386895_at	84469	Maged1	melanoma antigen, family D, 1	0.867981	0.004955	0.028733
1371020_at	266780	Rimbp2	RIM binding protein 2	1.116296	0.004957	0.028733
1369120_a_at	25329	Lhb	luteinizing hormone beta	1.111891	0.004963	0.028752
1384192_at	295934	Chst1	carbohydrate (keratan sulfate Gal-6) su	-1.213022	0.004977	0.028791
1390730_at	303321	RGD13056	similar to 1810009O10Rik protein	0.977318	0.004979	0.028791
1389983_at	313777	RGD13093	similar to cDNA sequence AF155546	0.887147	0.004981	0.028791
1369691_at	497770	Scn3a	sodium channel, voltage-gated, type II	1.178977	0.004981	0.028791
1374441_at	288772	RGD13066	similar to RIKEN cDNA 4633402N23 g	1.357292	0.004981	0.028791
1377933_at	314637	Slc39a3	solute carrier family 39 (zinc transporte	1.383939	0.004983	0.028791
1386396_at	361679	Dusp8_pre	dual specificity phosphatase 8 (predict	-3.098806	0.004987	0.0288
1367819_at	25721	Got2	glutamate oxaloacetate transaminase	1.096608	0.004989	0.0288
1392107_at	314619	Stno_predi	strawberry notch homolog (Drosophila	1.168366	0.004991	0.0288
1388323_at	362440	Ndufa9	NADH dehydrogenase (ubiquinone) 1	0.852352	0.004996	0.028819
1368055_a_at	60374	Lmna	lamin A	-1.312918	0.005001	0.028825
1398765_at	116563	Ap2m1	adaptor-related protein complex 2, mu	0.855037	0.005003	0.028825
1371365_at	292588	Ube2s_pre	ubiquitin-conjugating enzyme E2S (pre	1.068538	0.005007	0.028825
1385644_at	365319	LOC36531	NA	1.520188	0.005007	0.028825
1370192_at	65033	Stx12	syntaxin 12	1.401661	0.00501	0.028825
1368263_a_at	25037	Mobp	myelin-associated oligodendrocytic ba	2.012682	0.00501	0.028825
1384079_at	361503	Eps8l1_pre	EPS8-like 1 (predicted)	2.323344	0.005023	0.028862
1368907_at	65171	Scamp5	secretory carrier membrane protein 5	2.726939	0.005024	0.028862
1385497_x_at	360926	Lnx1_predi	ligand of numb-protein X 1 (predicted)	-2.456891	0.005027	0.028862
1398766_at	25596	Rpn1	ribophorin I	0.86728	0.005028	0.028862
1390343_at	114839	Ccnc	cyclin C	-0.889988	0.005028	0.028862
1368668_at	116645	Plaa	phospholipase A2, activating protein	1.001296	0.00503	0.028862
1373044_at	295975	RGD15658	similar to Dendritic cell protein GA17 (p	-0.937685	0.005044	0.028924
1399039_at	501165	NA	NA	1.277385	0.005045	0.028924
1376305_at	308857	Dnajb13	DnaJ (Hsp40) related, subfamily B, me	2.747579	0.00505	0.02894
1374199_at	687681	LOC68768	NA	-1.148651	0.005052	0.02894

1369906_s_at	246117	Mcfd2	multiple coagulation factor deficiency 2	2.114704	0.005056	0.028949
1396118_at	298409	RGD15649	RGD1564942 (predicted)	1.845539	0.005068	0.028997
1368849_at	64823	Csnk1g3	casein kinase 1, gamma 3	1.352817	0.005069	0.028997
1388156_at	29322	Plcb3	phospholipase C, beta 3	1.967325	0.005077	0.029016
1368912_at	25569	Trh	thyrotropin releasing hormone	7.029772	0.005077	0.029016
1398514_at	360719	Hgd	homogentisate 1, 2-dioxygenase	-2.623965	0.00508	0.029016
1390454_at	360971	Nipsnap1	4-nitrophenylphosphatase domain and	1.375258	0.005081	0.029016
1370252_at	171386	Avpi1	arginine vasopressin-induced 1	1.746289	0.005087	0.02904
1385297_at	300057	Recql4_pre	RecQ protein-like 4 (predicted)	4.443157	0.005093	0.029047
1388565_at	300791	Spg21	spastic paraplegia 21 homolog (human	1.217483	0.005093	0.029047
1388364_at	295923	Ndufs3_pre	NADH dehydrogenase (ubiquinone) Fe	0.897697	0.005097	0.029049
1376592_at	293829	Mcee_pred	methylmalonyl CoA epimerase (predict	-1.026273	0.005101	0.029049
1382307_at	116670	Ppp1r12a	protein phosphatase 1, regulatory (inhi	1.112447	0.005104	0.029049
1387028_a_at	25261	Id1	inhibitor of DNA binding 1	-1.597466	0.005104	0.029049
1376676_a_at	290270	RGD13051	similar to Ab2-008	-0.939676	0.005104	0.029049
1370069_at	171373	Slc12a5	solute carrier family 12, (potassium-ch	-1.219994	0.005107	0.029051
1368387_at	117099	Bdh1	3-hydroxybutyrate dehydrogenase, typ	4.088572	0.005113	0.029074
1376565_at	25638	Pde4a	phosphodiesterase 4A, cAMP specific	-1.665501	0.005116	0.029074
1368083_at	84389	Ccnh	cyclin H	-1.033367	0.005117	0.029074
1394218_s_at	314930	Zfpm2_pre	zinc finger protein, multitype 2 (predict	-2.074069	0.005122	0.029086
1394535_at	311478	Kif16b_pre	kinesin family member 16B (predicted)	1.38108	0.005129	0.029115
1375450_at	299114	Sdccag1	serologically defined colon cancer anti	-1.11347	0.005132	0.029123
1368371_at	84020	Kcnq1	potassium voltage-gated channel, subf	3.358792	0.00514	0.029152
1399088_at	171311	Tlk2	tousled-like kinase 2 (Arabidopsis)	-0.901685	0.005147	0.029161
1389372_at	313340	RGD13084	similar to hypothetical protein (predicte	-0.879585	0.005147	0.029161
1375887_at	501506	RGD15663	similar to kaiso protein (predicted)	-1.185248	0.005148	0.029161
1382892_a_at	500898	NA	NA	2.279239	0.005156	0.029197
1386971_at	65045	Ppp1r10	protein phosphatase 1, regulatory subu	1.153179	0.00516	0.029205
1395376_at	316767	Ddx11_pre	DEAD/H (Asp-Glu-Ala-Asp/His) box po	-2.834983	0.005164	0.029212
1398840_at	89818	Vamp5	vesicle-associated membrane protein 5	0.935747	0.005167	0.02922
1388301_at	301011	Uqcrc1	ubiquinol-cytochrome c reductase core	1.053145	0.005171	0.029227
1382699_s_at	114638	Hps1	Hermansky-Pudlak syndrome 1 homol	1.458109	0.005175	0.029227
1389447_at	301008	RGD13118	similar to prolyl-4-hydroxylase-alpha N	1.291541	0.005175	0.029227
1373238_at	360874	Tada1l	transcriptional adaptor 1 (HFI1 homolo	-1.295561	0.005185	0.02925
1367808_at	64372	Timm8b	translocase of inner mitochondrial mer	0.892016	0.005185	0.02925
1389187_at	315126	RGD13596	similar to RIKEN cDNA 1700088E04	-0.917203	0.005186	0.02925
1370904_at	294274	Hla-dma	major histocompatibility complex, class	1.154885	0.005188	0.029251
1370213_at	500538	Ybx1	Y box protein 1	0.940567	0.005201	0.029308
1398784_at	29681	C1qbp	complement component 1, q subcomp	1.033075	0.005203	0.029308
1376262_at	246232	Uxs1	UDP-glucuronate decarboxylase 1	1.624158	0.005218	0.029369
1380798_at	304005	Nfkbiz_pre	nuclear factor of kappa light polypeptic	1.89553	0.005218	0.029369
1389587_at	288051	Umps	uridine monophosphate synthetase	1.364389	0.005225	0.029396
1374221_at	353307	Slc29a3	solute carrier family 29 (nucleoside tra	1.490241	0.00523	0.029406
1371524_at	307642	Gtl3	gene trap locus 3	-0.938832	0.005231	0.029406
1382084_at	500464	RGD15629	similar to EHM2 (predicted)	1.106014	0.005234	0.029407
1393450_at	360829	RGD13101	similar to Ab2-034	-0.852402	0.005238	0.029422
1376238_at	498411	LOC49841	NA	1.717849	0.005245	0.029437
1380833_at	291132	Gpld1	glycosylphosphatidylinositol specific ph	1.431524	0.005246	0.029437
1385078_at	361095	Zic5_predi	zinc finger protein of the cerebellum 5	3.406483	0.005251	0.029457
1398372_at	497900	MGC11268	similar to RIKEN cDNA 0610039K22	1.247268	0.005258	0.029484
1368179_at	171499	Tslpr	thymic stromal-derived lymphopoietin,	0.973277	0.005264	0.029499

1372713_at	299295	RGD13095	similar to hypothetical protein D12Ertd	-0.868587	0.005266	0.029499
1371411_at	315217	Plxnb2	plexin B2	0.931104	0.005269	0.029505
1377123_at	311839	Slc27a4	solute carrier family 27 (fatty acid trans	1.859512	0.005278	0.029541
1379521_at	287983	Alg3	asparagine-linked glycosylation 3 hom	1.382474	0.005283	0.029559
1389170_at	64026	Casp7	caspase 7	2.25891	0.005294	0.029591
1393047_at	292188	Ibrdc1_pre	IBR domain containing 1 (predicted)	-0.872554	0.005296	0.029591
1398778_at	29668	Psma1	proteasome (prosome, macropain) sub	0.826933	0.005298	0.029591
1390926_at	311630	Zswim3_pr	zinc finger, SWIM domain containing 3	1.402465	0.0053	0.029591
1388766_at	288150	Mtx2	metaxin 2	-1.087395	0.0053	0.029591
1369323_at	56766	Leprot	leptin receptor overlapping transcript	2.035904	0.005309	0.029616
1390234_at	84486	Sf3b1	splicing factor 3b, subunit 1	-1.073347	0.005309	0.029616
1388380_at	300111	Samm50	sorting and assembly machinery comp	0.96226	0.005314	0.029624
1369304_at	29498	Pts	6-pyruvoyl-tetrahydropterin synthase	0.960085	0.005315	0.029624
1389585_at	192183	Pvrl1	poliovirus receptor-related 1	1.077927	0.005333	0.029702
1396104_at	292884	RGD13050	similar to Zinc finger protein 184 (predi	1.199562	0.005333	0.029702
1367550_a_at	362545	Tm2d1_pre	TM2 domain containing 1 (predicted)	-0.913649	0.005337	0.029708
1392944_at	315776	RGD15654	similar to talin 2 (predicted)	1.21869	0.005341	0.029722
1372561_at	298500	Smap1l	stromal membrane-associated protein	1.163805	0.005367	0.029844
1368060_at	65151	Hrsp12	heat-responsive protein 12	-0.818747	0.005368	0.029844
1387392_at	26955	Af6	afadin	0.84781	0.005378	0.029856
1368132_at	170842	Tob1	transducer of ErbB-2.1	-1.273463	0.005379	0.029856
1388878_at	291996	Nob1p	nin one binding protein	0.957358	0.005384	0.029856
1389326_at	288414	Rfc3	replication factor C (activator 1) 3	0.965068	0.005385	0.029856
1374050_at	360521	Rufy1	RUN and FYVE domain containing 1	-1.09415	0.005385	0.029856
1371497_at	296627	Asb6	ankyrin repeat and SOCS box-containi	1.152669	0.005388	0.029856
1374836_at	363134	Rnu3ip2_p	RNA, U3 small nucleolar interacting pr	1.131402	0.00539	0.029856
1375170_at	445415	S100a11	S100 calcium binding protein A11 (cali	-2.048186	0.005391	0.029856
1387806_at	171337	Rap1b	RAS related protein 1b	-0.931195	0.005392	0.029856
1371977_at	288669	Arpc3_pre	actin related protein 2/3 complex, subu	0.94829	0.005393	0.029856
1374047_at	291784	RGD13118	similar to RIKEN cDNA 2400010D15	-1.327387	0.0054	0.029881
1373061_at	298836	Snx17	sorting nexin 17	0.989949	0.005403	0.029881
1382869_at	309732	RGD13082	similar to hypothetical protein FLJ1454	1.361762	0.005407	0.029881
1398805_at	171415	Apg3l	APG3 autophagy 3-like (S. cerevisiae)	-0.84489	0.005407	0.029881
1371248_at	499660	LOC49966	NA	2.364072	0.005408	0.029881
1371600_at	266709	Pkig	protein kinase inhibitor, gamma	-1.170913	0.005418	0.029896
1379805_at	362861	Slc41a2_pr	solute carrier family 41, member 2 (pre	1.813804	0.005418	0.029896
1377061_at	315530	RICS_pred	RhoGAP involved in beta-catenin-N-ca	1.389723	0.005419	0.029896
1388493_at	362374	RGD13064	similar to Expressed sequence AW146	-1.606136	0.00542	0.029896
1370256_at	58868	Fzd1	frizzled homolog 1 (Drosophila)	-2.747424	0.005437	0.029966
1386394_at	314140	LOC31414	ribose-phosphate pyrophosphokinase	1.001732	0.005438	0.029966
1371419_at	305614	Spnb2	spectrin beta 2	-1.017593	0.00544	0.029966
1377685_at	498820	NA	NA	-1.021457	0.005445	0.029982
1386965_at	24539	Lpl	lipoprotein lipase	-6.557077	0.005449	0.029996
1386893_at	29143	Grn	granulin	0.938211	0.005456	0.030001
1369650_at	29432	Pak2	p21 (CDKN1A)-activated kinase 2	1.008601	0.005456	0.030001
1379282_at	301035	Lrrfip2	leucine rich repeat (in FLII) interacting	1.144998	0.005457	0.030001
1372006_at	290350	Loxl2_pred	lysyl oxidase-like 2 (predicted)	1.03888	0.005461	0.03001
1398493_at	316457	LOC31645	NA	-1.306796	0.005468	0.030026
1387322_at	84609	Sema6b	sema domain, transmembrane domain	1.13257	0.005469	0.030026
1370160_at	170751	Xpnpep1	X-prolyl aminopeptidase (aminopeptida	0.951554	0.00548	0.030068
1370905_at	259237	Dock9	dedicator of cytokinesis 9	-1.146822	0.005484	0.030068

1372739_at	362890	Tspan31	tetraspanin 31	1.153744	0.005484	0.030068
1372622_at	316001	RGD15607	similar to RIKEN cDNA 6530418L21 (p	-2.180511	0.005485	0.030068
1377742_at	366300	LOC36630	NA	1.078006	0.005492	0.030091
1374362_at	307834	RGD13066	similar to 4930566A11Rik protein (pred	-1.099057	0.005494	0.030091
1371695_at	304862	Tpr	translocated promoter region	-1.107608	0.005497	0.030097
1389288_at	291660	Ndufa2_pre	NADH dehydrogenase (ubiquinone) 1	0.858113	0.005514	0.030171
1387080_at	29486	Cspg6	chondroitin sulfate proteoglycan 6	-0.981822	0.005516	0.030171
1371471_at	292624	Gltscr2	glioma tumor suppressor candidate reg	-1.140351	0.005524	0.030201
1391944_at	361853	RGD13112	similar to Hypothetical protein C6orf60	-0.979798	0.005525	0.030201
1374624_at	311952	Galnt11	UDP-N-acetyl-alpha-D-galactosamine:	-1.217707	0.005534	0.030236
1382643_at	64088	Snx16	sorting nexin 16	-1.115467	0.005547	0.030283
1394492_at	498179	RGD15634	similar to hypothetical protein FLJ3866	2.978417	0.005547	0.030283
1373289_at	315287	Asb8_pred	ankyrin repeat and SOCS box-containi	0.924888	0.005554	0.030307
1371607_at	367171	LOC36717	microtubule-associated protein 4	-1.415281	0.005557	0.030311
1388897_at	362093	Wdr5	WD repeat domain 5	0.922533	0.005565	0.030344
1377125_at	313409	Dnajc6_pre	DnaJ (Hsp40) homolog, subfamily C, r	-1.116234	0.005571	0.030361
1372808_at	313410	RGD15640	similar to methylenetetrahydrofolate de	1.257966	0.005578	0.030389
1377860_at	298185	RGD13069	similar to bM410K19.2.2 (novel protein	1.860444	0.005589	0.030434
1367976_at	81815	Tpp2	tripeptidyl peptidase II	-1.330634	0.005603	0.030498
1371670_at	300045	Exosc4_pre	exosome component 4 (predicted)	0.983018	0.005609	0.03051
1385383_at	291847	RGD15657	similar to hypothetical protein 4933409	-2.688103	0.005611	0.03051
1393796_at	311391	Cep152_pr	centrosomal protein 152 (predicted)	-1.305757	0.005613	0.03051
1383347_at	316344	Rev1l_pred	REV1-like (S. cerevisiae) (predicted)	-0.868404	0.005617	0.03051
1375219_a_at	83789	Rps2	ribosomal protein S2	0.959041	0.005618	0.03051
1394097_at	65023	Kcnip1	Kv channel-interacting protein 1	-1.092815	0.005619	0.03051
1385765_at	360888	LOC36088	NA	-2.199053	0.005622	0.030511
1374991_at	287938	LOC28793	hypothetical LOC287938	1.285316	0.005624	0.030511
1381323_at	304794	Rbbp5_pre	retinoblastoma binding protein 5 (predi	2.179223	0.005626	0.030511
1373794_at	308457	Egln2	EGL nine homolog 2 (C. elegans)	1.050818	0.005638	0.030563
1377889_at	498058	NA	NA	1.452784	0.005642	0.030572
1398976_at	54299	Ncor1	nuclear receptor co-repressor 1	-0.85865	0.005649	0.030593
1375644_at	498824	RGD15620	similar to WAC (predicted)	1.002844	0.005653	0.030593
1372571_at	362849	2-Mar	membrane-associated ring finger (C3H	-0.846469	0.005657	0.030593
1392488_at	307526	RGD13079	similar to hypothetical protein MGC321	-0.951877	0.005659	0.030593
1386996_at	50685	Mrlcb	myosin light chain, regulatory B	-0.867092	0.005666	0.030593
1372033_at	361501	RGD13090	similar to RIKEN cDNA 0610042E07	3.025977	0.005667	0.030593
1375151_at	366733	RGD15651	similar to RAP2A, member of RAS onc	3.646411	0.005669	0.030593
1381190_at	361084	LMO7	LIM domain only protein 7	-1.597389	0.005669	0.030593
1383155_at	363236	Als2cr13_p	amyotrophic lateral sclerosis 2 (juvenile	-1.088919	0.00567	0.030593
1382263_at	308017	NA	NA	-1.862736	0.005671	0.030593
1396096_at	367252	Mgat4a	mannoside acetylglucosaminyltransfer	2.165274	0.005674	0.030593
1372861_at	314641	Pip5k1c	phosphatidylinositol-4-phosphate 5-kin	-0.976972	0.005675	0.030593
1398643_at	299284	Dicer1	Dicer1, Dcr-1 homolog (Drosophila)	0.959152	0.005676	0.030593
1372810_at	305178	Hnrpdl	heterogeneous nuclear ribonucleoprote	-0.820476	0.005687	0.03064
1370309_a_at	83498	Hnrpab	heterogeneous nuclear ribonucleoprote	0.888473	0.005693	0.030662
1368023_at	84491	Qscn6	quiescin Q6	-2.373819	0.005699	0.030664
1383454_a_at	289342	Rps6kc1	ribosomal protein S6 kinase, polypepti	2.72724	0.0057	0.030664
1371914_at	361825	Smarcb1	SWI/SNF related, matrix associated, a	1.023587	0.005702	0.030664
1390687_at	364206	Plek	pleckstrin	-2.170579	0.005703	0.030664
1387680_at	29691	Pde1b	phosphodiesterase 1B, Ca2+calmodul	1.904448	0.00571	0.030688
1398876_at	85493	Abcf1	ATP-binding cassette, sub-family F (G	0.955334	0.005712	0.030688

1376034_at	362353	RGD15654	similar to mKIAA0738 protein (predicte	-1.016487	0.005719	0.030688
1391032_at	192247	Sez6	seizure related 6 homolog (mouse)	-2.364852	0.005719	0.030688
1382601_at	498638	RGD15643	similar to scavenger receptor type A S	3.37544	0.005721	0.030688
1388366_at	363023	Mrpl4_prec	mitochondrial ribosomal protein L4 (pre	1.265656	0.005721	0.030688
1375413_at	361532	Sirt2	sirtuin (silent mating type information r	0.896861	0.00573	0.030719
1391443_at	498489	RGD15599	similar to chromosome 14 open readin	-1.132813	0.005733	0.030719
1375440_at	360746	Ppil2	peptidylprolyl isomerase (cyclophilin)-li	1.11087	0.005735	0.030719
1392524_at	81002	Trim23	tripartite motif protein 23	-1.621943	0.005736	0.030719
1397628_at	293500	RGD13055	similar to RIKEN cDNA 2900092E17	1.119061	0.005742	0.030735
1389971_at	432360	Sgce	sarcoglycan, epsilon	-3.295755	0.005745	0.030743
1375660_at	303135	Sept8_prec	septin 8 (predicted)	1.591647	0.005751	0.030758
1390104_at	292708	Irgq_predic	immunity-related GTPase family, Q (pr	0.903567	0.005759	0.030791
1369784_at	54314	Tpo	thyroid peroxidase	-2.638813	0.005775	0.030856
1388875_at	291440	Cxxc1	CXXC finger 1 (PHD domain)	1.175597	0.005777	0.030856
1371663_at	315329	RGD13051	similar to expressed sequence AW556	0.823911	0.005778	0.030856
1387331_at	24217	Atp4b	ATPase, H+/K+ exchanging, beta poly	3.677437	0.005786	0.030884
1397749_at	25744	Nf2	neurofibromatosis 2	1.459744	0.005795	0.030922
1388534_at	366381	Cdc26	cell division cycle 26	1.405298	0.005798	0.030922
1383243_at	24252	Cebpa	CCAAT/enhancer binding protein (C/E	-1.143417	0.005803	0.03094
1371704_at	362993	Rnd1	Rho family GTPase 1	0.815571	0.005808	0.030949
1372776_at	305424	Fbxl5_prec	F-box and leucine-rich repeat protein 5	-0.836105	0.005811	0.030949
1368233_at	81674	Gtf2f2	general transcription factor IIF, polype	1.141305	0.005812	0.030949
1370128_at	59112	Hand1	heart and neural crest derivatives expr	-2.784766	0.005815	0.030952
1388646_at	287871	RGD13117	similar to p150 target of rapamycin (TC	0.944116	0.005818	0.030952
1373397_at	114764	Mapre1	microtubule-associated protein, RP/EE	0.988021	0.00582	0.030952
1374431_at	360716	Atp6v1a1_	ATPase, H transporting, lysosomal V1	1.004064	0.005847	0.031084
1373840_at	305149	Nudt9	nudix (nucleoside diphosphate linked r	1.512451	0.005854	0.031109
1394654_at	316312	Zfp451	zinc finger protein 451	0.913024	0.005869	0.031167
1370906_at	29711	Bckdhb	branched chain keto acid dehydrogena	1.086482	0.005869	0.031167
1388113_at	171335	Cox8a	cytochrome c oxidase, subunit VIIIa	0.833563	0.005893	0.03128
1385062_at	304654	RGD13060	hypothetical LOC304654 (predicted)	1.116744	0.005906	0.031338
1372592_at	84581	Hdac6	histone deacetylase 6	-1.094698	0.005915	0.03137
1370276_at	192241	Atp5o	ATP synthase, H+ transporting, mitoch	0.918649	0.005917	0.03137
1367814_at	25650	Atp1b1	ATPase, Na+/K+ transporting, beta 1 p	1.392895	0.005925	0.0314
1387129_at	84495	Xrcc1	X-ray repair complementing defective r	-0.868577	0.005928	0.031403
1381063_at	302950	Adcy9_pre	adenylate cyclase 9 (predicted)	1.192176	0.00594	0.031453
1383445_at	360204	Zfp191	zinc finger protein 191	-1.169599	0.005957	0.031503
1373706_at	316477	Smarcal1_	Swi/SNF related matrix associated, ac	1.334584	0.005961	0.031503
1372562_at	362946	MGC94207	similar to RIKEN cDNA C030006K11	1.458699	0.005963	0.031503
1371952_at	311902	Rbm18_pre	RNA binding motif protein 18 (predicte	-1.061316	0.00597	0.031503
1373607_at	64445	St3gal3	ST3 beta-galactoside alpha-2,3-sialyltr	0.875891	0.00597	0.031503
1380142_at	24457	Hoxb8_ma	homeo box B8 (mapped)	5.297786	0.005971	0.031503
1396561_x_at	363464	Piga_mapp	phosphatidylinositol glycan, class A (m	4.722835	0.005971	0.031503
1370771_at	140728	Cacng7	calcium channel, voltage-dependent, g	-2.275753	0.005972	0.031503
1383961_a_at	362666	Dnajc11_p	DnaJ (Hsp40) homolog, subfamily C, r	1.143131	0.005973	0.031503
1372432_at	361995	Prpf3_pred	PRP3 pre-mRNA processing factor 3 h	0.952984	0.005973	0.031503
1387636_a_at	171432	Cdtw1	P11 protein	1.263416	0.005981	0.031531
1387583_at	154985	Cyp26a1	cytochrome P450, family 26, subfamily	-2.619193	0.005986	0.03154
1399150_at	306229	RGD13060	similar to RIKEN cDNA A630054L15; h	-1.053925	0.005988	0.03154
1389733_at	299851	Mars_pred	methionine-tRNA synthetase (predicte	1.137211	0.005991	0.03154
1397202_at	499334	RGD15602	similar to RIKEN cDNA 1700028P14 (p	2.820181	0.005992	0.03154

1384500_at	316638	Sned1	insulin responsive sequence DNA binc	-2.342593	0.006003	0.031576
1370111_at	54262	Kcnn2	potassium intermediate/small conducta	-1.398276	0.006004	0.031576
1391347_at	266688	Rab8b	RAB8B, member RAS oncogene famil	1.642974	0.006019	0.031642
1377571_at	361500	Zfp628_pre	zinc finger protein 628 (predicted)	1.044106	0.006027	0.031672
1397824_at	307029	RGD15624	similar to WAC (predicted)	1.3239	0.006052	0.031784
1373939_at	297393	Nagk	N-acetylglucosamine kinase	1.57889	0.006053	0.031784
1388258_at	112400	Nrg1	neuregulin 1	0.912052	0.006068	0.031854
1372413_at	300222	Mcrs1	microspherule protein 1	1.134241	0.006079	0.031892
1383568_at	361856	Tube1_pre	tubulin, epsilon 1 (predicted)	-0.991282	0.006081	0.031892
1398829_at	25639	Fkbp1a	FK506 binding protein 1a	0.835583	0.006084	0.031892
1376950_at	308775	Det1	de-etiolated homolog 1 (Arabidopsis)	1.40651	0.006085	0.031892
1370260_at	25230	Add3	adducin 3 (gamma)	0.947398	0.006099	0.03195
1372122_at	292925	Tsg101	tumor susceptibility gene 101	-0.872515	0.006103	0.03195
1371509_at	25346	Ros1	v-ros UR2 sarcoma virus oncogene ho	0.814463	0.006104	0.03195
1369652_at	24832	Thy1	thymus cell antigen 1, theta	-2.334853	0.006112	0.031972
1371611_at	311215	Ext2_predi	exostoses (multiple) 2 (predicted)	0.818802	0.006113	0.031972
1372610_at	360526	P4ha2_pre	procollagen-proline, 2-oxoglutarate 4-c	1.31016	0.006117	0.031978
1389478_at	303792	Klhl22_pre	kelch-like 22 (Drosophila) (predicted)	1.126838	0.006123	0.031978
1384602_at	311857	Gpr107_pr	G protein-coupled receptor 107 (predic	1.58055	0.006124	0.031978
1389963_at	652956	LOC65295	NA	0.904765	0.006125	0.031978
1391495_at	294012	RGD13117	similar to RIKEN cDNA 2010012O05 (	0.841539	0.006128	0.031978
1374393_at	288058	Ptplb_pred	protein tyrosine phosphatase-like (prol	1.284898	0.006129	0.031978
1373004_at	116456	Dnajc2	DnaJ (Hsp40) homolog, subfamily C, r	0.820391	0.00614	0.032018
1371768_at	298681	Ssu72	Ssu72 RNA polymerase II CTD phospl	1.201523	0.006142	0.032018
1380810_at	499061	NA	NA	-1.286769	0.00615	0.032018
1390178_at	300965	Srprb	signal recognition particle receptor, B s	1.153785	0.006152	0.032018
1373704_at	366163	Aqr_predic	aquarius (predicted)	1.056888	0.006152	0.032018
1390237_at	84383	Timm8a	translocase of inner mitochondrial mer	1.106564	0.006153	0.032018
1387790_at	140946	Paics	phosphoribosylaminoimidazole carbox	0.972836	0.006156	0.032018
1382688_at	306000	RGD13593	mitochondrial carrier domain containin	1.523538	0.006157	0.032018
1371369_at	361821	Col6a2	procollagen, type VI, alpha 2	-3.471123	0.006162	0.032018
1372853_at	309165	Rela	v-rel reticuloendotheliosis viral oncoge	-0.794442	0.006163	0.032018
1388568_at	362952	Eif3s7	eukaryotic translation initiation factor 3	0.928414	0.006164	0.032018
1398551_at	303534	RGD13118	similar to hypothetical protein DKFZp4	-3.167964	0.006166	0.032018
1371409_at	498886	RGD15629	similar to cDNA sequence BC031181 (	0.938563	0.006168	0.032018
1370890_at	81732	Actr3	ARP3 actin-related protein 3 homolog	0.804759	0.006173	0.032032
1368109_at	83505	St3gal5	ST3 beta-galactoside alpha-2,3-sialyltr	1.241577	0.006189	0.032104
1373344_at	363471	RGD15622	similar to GS2 gene (predicted)	1.068563	0.006193	0.032112
1370220_at	114861	Scpep1	serine carboxypeptidase 1	1.811145	0.006202	0.032137
1383115_at	309338	Cstf2t_pre	cleavage stimulation factor, 3' pre-RNA	-0.956831	0.006203	0.032137
1388756_at	298490	Ppcs	phosphopantothenoylcysteine syntheta	1.762205	0.006207	0.032148
1389095_at	360715	Boc_predic	biregional cell adhesion molecule-relat	-2.842058	0.006217	0.032188
1371455_at	300089	Pmm1	phosphomannomutase 1	1.246915	0.006229	0.032234
1387284_at	65135	Dpys	dihydropyrimidinase	1.739522	0.006231	0.032234
1370418_s_at	192189	Bk	brain and kidney protein	-1.391532	0.006236	0.032246
1372547_at	294709	RGD13065	similar to RIKEN cDNA 2410002O22 g	0.973461	0.006258	0.032328
1370232_at	24513	Ivd	isovaleryl coenzyme A dehydrogenase	1.650279	0.006259	0.032328
1375261_at	295319	Man1a2_pr	mannosidase, alpha, class 1A, membe	2.257722	0.006259	0.032328
1374995_at	291962	Elmo3	engulfment and cell motility 3, ced-12 l	0.869983	0.006269	0.032367
1386403_at	313270	Egfl5_pred	EGF-like-domain, multiple 5 (predicted	-0.918485	0.006275	0.032383
1392755_at	289443	Lrrc8c	leucine rich repeat containing 8 family,	-1.152088	0.00628	0.032396

1373046_at	362836	Dazap1	DAZ associated protein 1	1.13224	0.006295	0.032462
1367886_at	29606	Pcsk7	proprotein convertase subtilisin/kexin t	0.835503	0.006297	0.032462
1394597_at	305816	Ddhd1	DDHD domain containing 1	0.964268	0.006315	0.032534
1375663_at	363284	Ube2f	ubiquitin-conjugating enzyme E2F (put	0.984273	0.006316	0.032534
1381057_at	364835	RGD15617	similar to hypothetical protein MGC539	1.843139	0.00632	0.032541
1390063_at	287299	Mfap3	microfibrillar-associated protein 3	2.181566	0.006322	0.032541
1395072_at	289399	RGD13118	similar to hypothetical protein FLJ1090	3.123859	0.006325	0.032543
1368275_at	140910	Sc4mol	sterol-C4-methyl oxidase-like	-1.067552	0.006345	0.032633
1390148_a_at	305972	Zfp395_pre	zinc finger protein 395 (predicted)	-1.476873	0.006357	0.03267
1372744_at	295625	Pkp4_pred	plakophilin 4 (predicted)	-0.908395	0.006361	0.03267
1372305_at	360611	Copz2_pre	coatomer protein complex, subunit zeta	1.415639	0.006362	0.03267
1398327_at	289992	Plekhc1	pleckstrin homology domain containing	-1.407097	0.006362	0.03267
1391075_at	308118	Rgs17_pre	regulator of G-protein signaling 17 (pre	1.262495	0.006373	0.032689
1371777_at	288398	RGD15624	similar to Pabpc4_predicted protein (pr	1.135054	0.006373	0.032689
1375600_at	313341	Pigo_predi	phosphatidylinositol glycan, class O (p	1.453972	0.006373	0.032689
1370164_at	170670	Hadha	hydroxyacyl-Coenzyme A dehydrogena	0.814033	0.006382	0.032722
1388358_at	292845	Etfb	electron-transfer-flavoprotein, beta poly	0.948159	0.006387	0.032732
1369301_at	83518	Agtrl1	angiotensin receptor-like 1	1.429886	0.00639	0.032732
1398782_at	140673	Napa	N-ethylmaleimide sensitive fusion prote	1.017459	0.006392	0.032732
1387888_at	81772	Rps9	ribosomal protein S9	0.912352	0.006397	0.032748
1375991_at	308751	Nmb_predi	neuromedin B (predicted)	1.074742	0.006427	0.032886
1385357_at	502311	NA	NA	1.216089	0.006436	0.032921
1367638_at	85239	Mlycd	malonyl-CoA decarboxylase	1.230519	0.006444	0.032947
1374902_at	310621	Iqgap3_pre	IQ motif containing GTPase activating	-1.953682	0.006457	0.033004
1373213_at	116500	Snap29	synaptosomal-associated protein 29	0.817723	0.006463	0.033013
1388929_at	502635	RGD15597	similar to HMG-1 (predicted)	-0.795507	0.006464	0.033013
1393129_at	361612	P4ha3	procollagen-proline, 2-oxoglutarate 4-d	0.967199	0.006475	0.03305
1396803_at	313308	Thoc2_pre	THO complex 2 (predicted)	1.105273	0.006476	0.03305
1371722_at	362967	RGD13115	similar to CGI-96 protein; gastric cance	1.085429	0.006482	0.033064
1372818_at	361289	Colec12	collectin sub-family member 12	-2.808109	0.006484	0.033064
1378257_at	301014	Trex1	three prime repair exonuclease 1	2.119425	0.006487	0.033065
1369190_at	497761	Cd2	CD2 antigen	3.576026	0.006515	0.033199
1393610_at	362618	RGD13051	similar to hypothetical protein BC0081(	1.78037	0.006521	0.03321
1398573_at	306977	Zfp307	zinc finger protein 307	-1.30201	0.006529	0.03321
1372966_at	298504	RGD13101	hypothetical LOC298504 (predicted)	2.356976	0.006531	0.03321
1371721_at	500532	LOC50053	NA	1.444804	0.006531	0.03321
1370325_at	113961	Gorasp2	golgi reassembly stacking protein 2	0.967332	0.006533	0.03321
1376155_at	499507	RGD15611	similar to hypothetical protein 2BE212	-1.984078	0.006535	0.03321
1373382_at	298675	RGD13063	similar to RER1 homolog	0.807923	0.006537	0.03321
1387079_at	497757	Gucy1a3	guanylate cyclase 1, soluble, alpha 3	1.161496	0.00654	0.03321
1373682_at	304570	Ddx51_pre	DEAD (Asp-Glu-Ala-Asp) box polypept	1.01701	0.00654	0.03321
1388344_at	362819	RGD13056	similar to RIKEN cDNA 1110005A23	-0.870079	0.006548	0.033236
1393186_at	499690	RGD15644	similar to RIKEN cDNA 2010200O16 (	0.912509	0.006552	0.033247
1389317_at	311405	RGD13097	similar to hypothetical protein FLJ2050	0.86566	0.006557	0.03326
1370304_at	54311	Timm17a	translocator of inner mitochondrial mer	1.046517	0.006574	0.033332
1371417_at	497902	Qpc	low molecular mass ubiquinone-bindin	0.816739	0.006579	0.033343
1374993_at	287148	Rpusd1_pr	RNA pseudouridylate synthase domair	1.719938	0.006584	0.033358
1372726_at	64627	Hist1h4b	germinal histone H4 gene	2.553354	0.006589	0.033359
1375136_at	291665	NA	NA	-2.17507	0.006593	0.033359
1367503_at	293852	Bcap31	B-cell receptor-associated protein 31	0.804237	0.006594	0.033359
1371515_at	315466	NA	NA	0.957079	0.006596	0.033359

1376687_at	313387	Usp1	ubiquitin specific peptdiase 1	0.940732	0.006597	0.033359
1368692_a_at	29194	Chka	choline kinase alpha	-1.557395	0.006602	0.033371
1380979_a_at	361004	RGD13094	similar to KIAA0913 protein (predicted)	0.92427	0.006612	0.03341
1393922_at	362549	RGD15618	similar to CDNA sequence BC020077	-1.8789	0.006624	0.033458
1384411_at	296884	LOC29688	hypothetical LOC296884	-1.340686	0.006632	0.033476
1376537_at	500465	NA	NA	1.14642	0.006635	0.033476
1381177_at	140935	Afap	actin filament associated protein	1.403854	0.006635	0.033476
1371376_at	500252	RGD15655	similar to Gene model 461 (predicted)	-1.341379	0.006642	0.033495
1368407_at	64537	Hpse	heparanase	-1.977412	0.006648	0.033515
1373789_at	316087	RGD15624	similar to zinc finger protein 651 (predi	1.474541	0.006665	0.033585
1373960_at	288591	RGD13114	similar to transmembrane protein induc	3.95685	0.006677	0.033634
1378803_at	309095	Nkx6-2_pre	NK6 transcription factor related, locus	2.871783	0.00668	0.033635
1373512_at	362843	Ilvbl_predic	ilvB (bacterial acetolactate synthase)-li	1.663526	0.006692	0.033677
1373404_at	287653	Lrrc46	leucine rich repeat containing 46	0.911784	0.006695	0.033677
1369720_at	117057	Myo1b	myosin Ib	1.379189	0.006697	0.033677
1389315_at	304546	Git2	G protein-coupled receptor kinase-inte	-0.853778	0.006698	0.033677
1375870_a_at	362138	Rbms1	RNA binding motif, single stranded inte	-1.565874	0.006716	0.033743
1367477_at	362388	Mrpl53_pre	mitochondrial ribosomal protein L53 (p	0.791133	0.006717	0.033743
1376597_at	360524	Zcchc10	zinc finger, CCHC domain containing 1	-0.908437	0.00672	0.033745
1375093_at	362706	Rbks_pred	ribokinase (predicted)	1.510668	0.006725	0.033745
1398374_at	311346	RGD13071	similar to RIKEN cDNA 2810002D13 g	-0.779888	0.006726	0.033745
1368552_at	79563	Grpel1	GrpE-like 1, mitochondrial	1.217419	0.006727	0.033745
1367652_at	24484	Igfbp3	insulin-like growth factor binding protei	-1.480482	0.00673	0.033747
1368206_at	170588	Acot8	acyl-CoA thioesterase 8	1.352672	0.006741	0.033791
1388243_at	117257	Gpr176	G protein-coupled receptor 176	-3.02173	0.006753	0.033837
1388936_at	84407	Cdh11	cadherin 11	-1.328494	0.006756	0.033838
1392514_at	294436	Bxdc1_pre	brix domain containing 1 (predicted)	0.876008	0.006764	0.033864
1369732_a_at	64442	St3gal2	ST3 beta-galactoside alpha-2,3-sialyltr	-1.483726	0.006767	0.033866
1387023_at	81869	Gstm3	glutathione S-transferase, mu type 3	-1.222395	0.00677	0.033872
1377656_at	306526	Rbm13	RNA binding motif protein 13	0.952271	0.006777	0.033891
1371789_at	301117	LOC30111	NA	0.897309	0.006779	0.033891
1381511_at	140447	Slc8a2	solute carrier family 8 (sodium/calcium	-1.838054	0.006785	0.033907
1387172_a_at	81809	Tgfb2	transforming growth factor, beta 2	1.175049	0.006794	0.033936
1375368_at	287115	RGD13077	similar to RIKEN cDNA 1700012G19 g	0.93841	0.006796	0.033936
1390989_at	363463	RGD15639	similar to Mospd2 protein (predicted)	-1.367236	0.00681	0.033992
1371610_at	290794	Tnks_predi	tankyrase, TRF1-interacting ankyrin-re	-1.05447	0.006818	0.03402
1368527_at	29527	Ptgs2	prostaglandin-endoperoxide synthase	-4.460436	0.006835	0.034076
1375916_at	311726	Pcmtd2_pr	protein-L-isoaspartate (D-aspartate) O	0.781757	0.006836	0.034076
1373099_at	303277	Pigs	phosphatidylinositol glycan, class S	0.953357	0.006837	0.034076
1375346_at	500993	RGD15639	similar to hypothetical protein FLJ2001	0.865836	0.00684	0.03408
1387516_at	117554	Pnliprp2	pancreatic lipase-related protein 2	-3.090606	0.006846	0.034097
1390114_at	360871	Mpzl1	myelin protein zero-like 1	-1.054868	0.006864	0.034175
1370469_at	246781	Ptpn7	protein tyrosine phosphatase, non-rece	2.826482	0.00687	0.034189
1373028_at	140585	Ryk	receptor-like tyrosine kinase	1.365007	0.006879	0.034223
1372317_at	293692	Ehd1	EH-domain containing 1	1.300648	0.006889	0.034253
1378586_at	83681	Cish	cytokine inducible SH2-containing prot	-1.649184	0.00689	0.034253
1376159_at	311508	RGD15623	similar to zinc finger protein 336 (predi	-0.927878	0.006901	0.034292
1372042_at	291813	Cmtm3_pre	CKLF-like MARVEL transmembrane do	0.997826	0.006904	0.034292
1387266_at	140941	Siah1a	seven in absentia 1A	-1.845267	0.006911	0.034312
1367704_at	140670	Ap2b1	adaptor-related protein complex 2, bet	0.959762	0.006913	0.034312
1398582_at	314384	Rps6ka5_p	ribosomal protein S6 kinase, polypepti	1.083395	0.006927	0.034367

1369215_a_at	25306	Cpd	carboxypeptidase D	0.955265	0.006929	0.034367
1373026_at	363028	Spbc24_pr	spindle pole body component 24 homo	-2.469224	0.006935	0.03438
1387610_at	29333	Mcp	membrane cofactor protein	-2.42608	0.006937	0.03438
1370903_a_at	296318	LOC29631	similar to Ndr3 protein	1.011179	0.006941	0.03439
1384627_at	501765	RGD15595	similar to RIKEN cDNA 2310057N15 (	1.012438	0.006951	0.034423
1388477_at	501281	LOC50128	NA	0.975818	0.006953	0.034423
1374562_at	360642	RGD13114	similar to KIAA1267 protein (predicted)	-1.645013	0.006962	0.034448
1379704_at	361627	Zfp143	zinc finger protein 143	-1.118506	0.006963	0.034448
1382114_at	311118	Tlk1_predi	tousled-like kinase 1 (predicted)	-1.255377	0.006969	0.03445
1368591_at	81817	Usf2	upstream transcription factor 2	1.127158	0.006969	0.03445
1373509_at	298426	Nsun4_pre	NOL1/NOP2/Sun domain family, mem	0.759842	0.006978	0.034477
1370218_at	24534	Ldhb	lactate dehydrogenase B	-1.627298	0.00698	0.034477
1389907_at	297885	Zbtb8os_pi	zinc finger and BTB domain containing	1.196616	0.006987	0.034485
1399021_at	171552	Pprf18	PRP18 pre-mRNA processing factor 1	-0.976854	0.006989	0.034485
1369633_at	24772	Cxcl12	chemokine (C-X-C motif) ligand 12	-2.83214	0.006989	0.034485
1373001_at	312559	Chchd4	coiled-coil-helix-coiled-coil-helix domai	1.40716	0.006994	0.034495
1395404_at	303755	RGD13045	similar to Hypothetical protein 903001	-1.471851	0.007	0.034495
1398908_at	298203	Stoml2	stomatin (Epb7.2)-like 2	0.833618	0.007001	0.034495
1390828_at	29358	Npy1r	neuropeptide Y receptor Y1	-1.31385	0.007002	0.034495
1391170_at	297514	RGD13104	similar to mKIAA1757 protein (predicte	1.288105	0.007007	0.034507
1383427_at	499751	RGD15635	similar to Endoplasmic reticulum manr	1.576023	0.007018	0.034524
1389233_at	292944	Reps1_pre	RalBP1 associated Eps domain contai	-1.263949	0.007018	0.034524
1393196_at	311114	Klhl23_pre	kelch-like 23 (Drosophila) (predicted)	1.244494	0.007019	0.034524
1372210_at	288557	Mospd3	motile sperm domain containing 3	1.092282	0.007027	0.034524
1374321_at	499309	RGD15601	similar to RIKEN cDNA 2700081O15 (	-0.880548	0.007028	0.034524
1393397_at	296959	Cpa2_pred	carboxypeptidase A2 (pancreatic) (pre	-2.349561	0.007028	0.034524
1367983_at	84490	Fen1	flap structure-specific endonuclease 1	1.740257	0.007035	0.034524
1368554_at	25702	Pnlip	pancreatic lipase	-3.052527	0.007037	0.034524
1398923_at	287453	RGD13056	similar to D11Bwg0434e protein (predi	0.889833	0.007038	0.034524
1373508_at	365802	LOC36580	similar to Selenoprotein T	1.188876	0.007039	0.034524
1382526_at	313115	RGD13093	similar to RIKEN cDNA 1810074P20 (	1.002618	0.007039	0.034524
1398814_at	81830	Rab11a	RAB11a, member RAS oncogene fam	0.918809	0.00705	0.034563
1368398_at	29717	Cacna1g	calcium channel, voltage-dependent, T	1.062445	0.007052	0.034563
1374534_at	287156	Rhot2	ras homolog gene family, member T2	1.253241	0.007055	0.034563
1369743_a_at	29659	P2rx4	purinergic receptor P2X, ligand-gated i	1.047601	0.007062	0.034588
1372071_at	362851	Cd320	CD320 antigen	1.458271	0.007076	0.034641
1388769_at	360491	Traf7_pred	Tnf receptor-associated factor 7 (predi	0.835378	0.007086	0.034677
1379894_at	361032	RGD13101	similar to 3632451O06Rik protein (pre	-3.400358	0.007091	0.03469
1389061_at	288595	Nsun5_pre	NOL1/NOP2/Sun domain family, mem	1.114535	0.007117	0.034803
1388596_at	361422	Cotl1_pred	coactosin-like 1 (Dictyostelium) (predic	0.85532	0.007136	0.034883
1371658_at	361425	Cox4nb	COX4 neighbor	1.26315	0.007146	0.034917
1369407_at	25341	Tnfrsf11b	tumor necrosis factor receptor superfa	2.155546	0.007148	0.034917
1371654_at	313699	RGD13053	similar to RIKEN cDNA 2510039O18 (	1.224298	0.007157	0.034936
1372799_at	297389	Dguok_pre	deoxyguanosine kinase (predicted)	-1.306155	0.007157	0.034936
1371388_at	289950	Pdhb	pyruvate dehydrogenase (lipoamide) b	-0.806088	0.007161	0.03494
1371012_at	85255	Phyh2	phytanoyl-CoA 2-hydroxylase 2	1.038777	0.007172	0.034976
1386867_at	171087	Brp44l	brain protein 44-like	-0.913158	0.007173	0.034976
1389012_at	362344	Ndufb2_pre	NADH dehydrogenase (ubiquinone) 1	-0.801351	0.007179	0.034991
1368287_at	84030	Chn1	chimerin (chimaerin) 1	-1.743549	0.007182	0.034992
1375580_at	301130	LOC30113	NA	0.988781	0.007186	0.034998
1388617_at	361239	Bphl	biphenyl hydrolase-like (serine hydrola	-1.128683	0.007196	0.035037

1367698_a_at	83788	9-Sep	septin 9	1.776562	0.007201	0.035048
1384775_s_at	287106	Tmprss8	transmembrane protease, serine 8 (int	-3.054731	0.007204	0.035048
1373791_at	299612	RGD13591	similar to RIKEN cDNA 2310011J03	0.837646	0.007208	0.035051
1383250_at	317579	Utp14a	UTP14, U3 small nucleolar ribonucleo|	1.008309	0.00721	0.035051
1374215_at	314634	Plekhj1	pleckstrin homology domain containing	1.243817	0.007216	0.035067
1369460_at	64554	Slc7a2	solute carrier family 7 (cationic amino a	4.406367	0.007221	0.035077
1370534_at	245921	Acvr1c	activin A receptor, type IC	4.332784	0.007233	0.035124
1374660_at	293528	RGD1309C	similar to hypothetical protein FLJ2000	-0.938194	0.007237	0.03513
1398420_at	303614	Smurf2_pre	SMAD specific E3 ubiquitin protein liga	0.955175	0.007239	0.035131
1373708_at	499314	MGC12503	similar to RNA binding motif protein 21	1.175991	0.007245	0.035146
1384890_at	303547	Ezh1_pred	enhancer of zeste homolog 1 (Drosoph	2.101575	0.007248	0.035148
1368909_a_at	116493	Gripap1	GRIP1 associated protein 1	1.047539	0.007254	0.035152
1376025_at	499420	Hrmt1l1	HMT1 hnRNP methyltransferase-like 1	0.908235	0.007254	0.035152
1371609_at	294326	RGD1303C	homolog of zebrafish ES1	0.870579	0.007258	0.035154
1367662_at	63864	Hadh2	hydroxyacyl-Coenzyme A dehydrogena	1.075176	0.007267	0.035184
1398853_at	29676	Psmb3	proteasome (prosome, macropain) sub	0.810845	0.007289	0.035266
1389305_at	79124	Anxa4	annexin A4	-0.827596	0.007291	0.035266
1373492_at	361726	RGD13092	similar to hypothetical protein FLJ2048	0.935879	0.007291	0.035266
1378821_at	500709	LOC50070	NA	0.898986	0.007294	0.035268
1367701_at	58966	Ramp2	receptor (calcitonin) activity modifying	0.930802	0.0073	0.035284
1383013_at	499171	RGD1565C	similar to BTEB3 protein (predicted)	1.108911	0.007306	0.035294
1390973_at	295323	Trim45_pre	tripartite motif protein 45 (predicted)	1.195609	0.007309	0.035294
1387905_at	619393	LOC61939	NA	-0.998237	0.007311	0.035294
1367905_at	54410	Enpp3	ectonucleotide pyrophosphatase/phos|	1.043672	0.007313	0.035294
1375550_at	297994	Bag1_pred	Bcl2-associated athanogene 1 (predict	1.326764	0.007336	0.035389
1379450_at	310760	Cttnbp2nl_	CTTNBP2 N-terminal like (predicted)	-1.697214	0.007356	0.035475
1372132_at	291394	Cndp2	CNDP dipeptidase 2 (metallopeptidase	0.927786	0.00736	0.035476
1392453_at	84360	Clcn3	chloride channel 3	0.971762	0.007362	0.035476
1371673_at	310661	Vps72_pre	vacuolar protein sorting 72 (yeast) (pre	0.829306	0.007382	0.035556
1389647_at	362731	Snx13_pre	sorting nexin 13 (predicted)	-0.978771	0.007384	0.035556
1379784_at	308718	Pex7	peroxisome biogenesis factor 7	-1.079971	0.007387	0.035558
1383267_at	291000	Ubxd8	UBX domain containing 8	0.786301	0.007412	0.035656
1389845_at	360481	Dnaja3	DnaJ (Hsp40) homolog, subfamily A, n	0.89445	0.007412	0.035656
1388686_at	266766	Dscr1	Down syndrome critical region homolo	0.99634	0.007426	0.035709
1387484_at	29610	Tgfbr3	transforming growth factor, beta recep	1.520835	0.00743	0.035713
1377777_at	308067	Brd9_predi	bromodomain containing 9 (predicted)	-0.967324	0.007435	0.035719
1378745_at	78962	Per3	period homolog 3 (Drosophila)	-3.957878	0.007436	0.035719
1372773_at	296562	Npdc1	neural proliferation, differentiation and	0.977797	0.007442	0.035733
1379366_a_at	64027	Bspry	B-box and SPRY domain containing	1.549752	0.007448	0.035751
1373144_at	499654	NA	NA	1.279275	0.007465	0.035812
1380261_at	499806	LOC49980	similar to RIKEN cDNA 4933404M02	-1.346429	0.007466	0.035812
1398471_at	310411	Cog6	component of oligomeric golgi complex	0.813719	0.007472	0.035824
1373065_at	301333	Ptpn18	protein tyrosine phosphatase, non-rece	-1.059868	0.007483	0.035868
1383708_at	498564	LOC49856	similar to integrin, beta-like 1	-4.108238	0.007502	0.035939
1368896_at	81516	Madh7	MAD homolog 7 (Drosophila)	-4.262622	0.007504	0.035939
1377785_at	287595	Dhx40	DEAH (Asp-Glu-Ala-His) box polypepti	-1.041992	0.007512	0.035959
1371897_at	296635	RGD13058	similar to RIKEN cDNA D830019K17	0.806827	0.007516	0.035959
1368515_at	116724	Epb4.1l3	erythrocyte protein band 4.1-like 3	-0.869996	0.007518	0.035959
1370873_at	24244	Calm3	calmodulin 3	1.00364	0.007519	0.035959
1377690_at	292771	Sipa1l3	signal-induced proliferation-associated	0.867423	0.007524	0.035968
1373954_at	360819	RGD13059	similar to FLJ00052 protein (predicted)	-1.018682	0.007528	0.035977

1389509_at	307527	Sap130_pr	Sin3A associated protein 130 (predicte	0.791761	0.007535	0.035984
1387306_a_at	114090	Egr2	early growth response 2	-2.587513	0.007535	0.035984
1383213_at	312812	Eps8_pred	epidermal growth factor receptor pathv	1.611958	0.00754	0.035991
1377102_at	289318	Tmem63a_	transmembrane protein 63a (predicted	0.919926	0.007549	0.035991
1384368_at	309762	Dna2l_prec	DNA2 DNA replication helicase 2-like (	-0.790362	0.00755	0.035991
1388305_at	64363	Araf	v-raf murine sarcoma 3611 viral oncog	-1.15949	0.00755	0.035991
1372929_at	83731	Kcnma1	potassium large conductance calcium-	0.808225	0.00755	0.035991
1371299_at	140654	Rps3	ribosomal protein S3	1.141096	0.007553	0.035991
1376015_at	297748	Jph1_predi	junctophilin 1 (predicted)	-1.164795	0.007556	0.035996
1387406_at	246332	Uhmk1	U2AF homology motif (UHM) kinase 1	1.307096	0.007561	0.036006
1388306_at	288513	RGD13055	similar to RIKEN cDNA 1810042K04 (p	1.411135	0.007566	0.036013
1397556_at	288108	Mak3_prec	Mak3 homolog (S. cerevisiae) (predicte	1.056348	0.007574	0.036041
1393521_at	295324	Ttf2_predic	transcription termination factor, RNA p	0.890513	0.00758	0.036055
1367996_a_at	65096	Lphn1	latrophilin 1	1.005883	0.00759	0.03609
1391427_at	294718	RGD13117	similar to RIKEN cDNA 1500031M22 (	-0.93379	0.007596	0.036105
1379858_at	363687	Mettl2_pre	methyltransferase like 2 (predicted)	1.283446	0.007617	0.036194
1387665_at	81508	Bhmt	betaine-homocysteine methyltransfera	1.281792	0.007627	0.036229
1379348_at	366017	Exosc2_pr	exosome component 2 (predicted)	1.352613	0.007642	0.036287
1371452_at	300744	Bmsc-UbP	bone marrow stromal cell-derived ubiq	-1.320736	0.007646	0.036291
1388958_a_at	25139	Slc2a4	solute carrier family 2 (facilitated gluco	2.081302	0.007655	0.036322
1370869_at	29592	Bcat1	branched chain aminotransferase 1, cy	4.582652	0.00766	0.03633
1383902_at	304007	Lrriq2_prec	leucine-rich repeats and IQ motif conta	1.278487	0.007663	0.036333
1392449_at	362412	Rad18_pre	RAD18 homolog (S. cerevisiae) (predic	-1.235789	0.007668	0.036339
1382400_at	313566	Rlf_predict	rearranged L-myc fusion sequence (pr	-1.463079	0.00767	0.036339
1371578_at	25636	Prkaca	protein kinase, cAMP-dependent, cata	0.999375	0.007679	0.036372
1375073_at	500601	NA	NA	-2.112376	0.007705	0.036479
1368834_at	24246	Camk2d	calcium/calmodulin-dependent protein	-1.911258	0.007714	0.036512
1383273_a_at	294336	Pcbp3	poly(rC) binding protein 3	0.886721	0.007727	0.036557
1382182_at	300264	Hoxc9_pre	homeo box C9 (predicted)	2.651292	0.007736	0.036586
1367938_at	83472	Ugdh	UDP-glucose dehydrogenase	1.516151	0.007786	0.036811
1370339_at	117557	Tpm3	tropomyosin 3, gamma	0.912468	0.007791	0.03682
1372247_at	313648	Ddost_prec	dolichyl-di-phosphooligosaccharide-pro	0.947725	0.007793	0.03682
1388563_at	294734	RGD13054	similar to RIKEN cDNA 1700034P14 (p	-0.855796	0.007797	0.036825
1374011_at	291703	Ercc3	excision repair cross-complementing ro	0.863469	0.007811	0.036876
1370497_at	353498	Cyp11b3	cytochrome P450, subfamily 11B, poly	1.269442	0.00782	0.036906
1390865_at	681395	LOC68139	NA	1.448521	0.007831	0.036945
1388765_at	25233	Akt2	thymoma viral proto-oncogene 2	0.73629	0.007836	0.036955
1374922_at	362614	Atpbd1b_p	ATP binding domain 1 family, member	0.771371	0.007851	0.037012
1372399_at	311796	RGD13078	similar to cofactor of BRCA1; negative	-0.934797	0.007878	0.037109
1368082_at	24780	Slc4a2	solute carrier family 4, member 2	1.041422	0.007879	0.037109
1398342_at	501052	LOC50105	similar to Fus1 protein	1.975811	0.007881	0.037109
1372061_at	296649	Rabepk	Rab9 effector protein with kelch motifs	0.971323	0.007883	0.037109
1370398_at	192225	Spata7	spermatogenesis associated 7	-1.44778	0.007892	0.037138
1392669_at	306137	RGD13082	similar to CG10084-PA	-0.971571	0.007901	0.037171
1380460_at	311429	RGD13112	similar to RIKEN cDNA 4931426K16 g	1.112932	0.007906	0.037179
1369842_at	63866	Accn5	amiloride-sensitive cation channel 5, in	-2.057588	0.007918	0.0372
1370258_at	171439	Bzw2	basic leucine zipper and W2 domains 2	1.3709	0.007919	0.0372
1398826_s_at	245980	Nr2f6	nuclear receptor subfamily 2, group F,	1.240629	0.007919	0.0372
1386942_at	64528	Golga2	cis-Golgi matrix protein GM130	1.257074	0.007928	0.037228
1379645_at	306254	RGD15655	similar to polybromo-1 (predicted)	0.980232	0.007935	0.037228
1383126_at	24185	Akt1	thymoma viral proto-oncogene 1	1.015694	0.007936	0.037228

1372769_at	498225	LOC49822	similar to ligatin	1.100116	0.007936	0.037228
1388685_at	360742	Dgcr2	DiGeorge syndrome critical region gen	1.061744	0.00794	0.03723
1373174_at	497867	LOC49786	similar to 1110001K21Rik protein	0.817195	0.007942	0.03723
1395348_at	304315	Ttyh3_prec	tweety homolog 3 (Drosophila) (predic	-2.143597	0.007966	0.037315
1397676_at	362447	RGD15631	similar to osteoclast inhibitory lectin (pı	1.315341	0.007968	0.037315
1367580_at	81729	Rpl10a	ribosomal protein L10A	0.82757	0.007969	0.037315
1388412_at	307833	RGD13077	similar to RIKEN cDNA 2400003C14	-0.859618	0.007974	0.037316
1371827_at	361712	Syvn1	synovial apoptosis inhibitor 1, synoviol	0.982259	0.007975	0.037316
1369768_at	201097	H1f4	NA	1.737654	0.007998	0.037401
1390226_at	498840	RGD15625	similar to hypothetical protein LOC340	1.590507	0.008	0.037401
1373471_at	365022	Rnf166	ring finger protein 166	-0.92732	0.008001	0.037401
1378349_at	304872	NA	NA	2.970104	0.008015	0.037443
1371499_at	24936	Cd9	CD9 antigen	-2.844364	0.008016	0.037443
1383689_at	303923	B4galt4	UDP-Gal:betaGlcNAc beta 1,4-galacto	1.852243	0.008029	0.03749
1373294_at	289603	Commd8_p	COMM domain containing 8 (predicted	1.124305	0.008038	0.037514
1375941_at	304282	Baiap2l1	BAI1-associated protein 2-like 1	-1.978166	0.008041	0.037514
1398401_at	306066	Tdrd3	tudor domain containing 3	1.210591	0.008045	0.037514
1379317_a_at	309458	Pdcd11_pr	programmed cell death protein 11 (pre	0.920514	0.008048	0.037514
1378902_at	304423	Rsafd1_pre	radical S-adenosyl methionine and flav	0.90665	0.00805	0.037514
1390278_at	500116	RGD15625	similar to RIKEN cDNA G430041M01 (	-1.630576	0.008051	0.037514
1376281_at	500909	LOC50090	similar to hypothetical protein FLJ2234	1.454415	0.008056	0.037523
1388745_at	310630	Sema4a	sema domain, immunoglobulin domain	1.619778	0.008066	0.037559
1398399_at	303180	RGD13045	similar to RIKEN cDNA 2310033P09	-1.094399	0.008072	0.037566
1398618_s_at	364899	RGD13067	similar to hypothetical protein FLJ3274	0.900883	0.008073	0.037566
1389765_at	303372	Nle1_predi	notchless homolog 1 (Drosophila) (pre	0.89357	0.008079	0.037577
1388810_at	361390	Abce1	ATP-binding cassette, sub-family E (O	1.082936	0.008085	0.037586
1387893_at	192262	C1s	complement component 1, s subcomp	-3.181081	0.008086	0.037586
1381286_at	502544	RGD15637	similar to late envelope protein 7 (LEP	3.793359	0.00809	0.037588
1388310_at	287703	Sui1-rs1_p	suppressor of initiator codon mutations	0.867056	0.008093	0.037588
1380201_at	313981	Nol10	nucleolar protein 10	0.763668	0.008096	0.037588
1371886_at	311849	Crat	carnitine acetyltransferase	1.260056	0.0081	0.037588
1378970_at	310782	RGD13105	similar to Myosin-binding protein H (My	1.004406	0.008103	0.037588
1375988_at	58856	Ddx25	DEAD (Asp-Glu-Ala-Asp) box polypept	1.235129	0.008109	0.037588
1389738_at	304577	Ung	uracil-DNA glycosylase	0.951301	0.008109	0.037588
1368162_at	171096	Cst6	cystatin E/M	-1.276078	0.008111	0.037588
1372950_at	54400	Bet1l	blocked early in transport 1 homolog (S	0.924796	0.008114	0.037588
1373573_at	246185	Pp3111	PP3111 protein	0.799133	0.008115	0.037588
1387383_at	83633	Gabbr2	gamma-aminobutyric acid (GABA) B re	-0.969521	0.008121	0.037602
1367890_at	64314	Casp2	caspase 2	-1.38111	0.008126	0.037614
1373411_at	301068	Eif1b_pred	eukaryotic translation initiation factor 1	-0.769935	0.008138	0.037656
1383241_at	312705	C1r	complement component 1, r subcompo	-3.489998	0.008148	0.037684
1386885_at	64526	Ech1	enoyl coenzyme A hydratase 1, peroxi	1.016452	0.008152	0.037684
1373146_at	308023	Ssx2ip	synovial sarcoma, X breakpoint 2 inter	0.797036	0.008156	0.037684
1370072_at	24590	Mme	membrane metallo endopeptidase	-2.557521	0.008156	0.037684
1370311_at	64514	Eif2b1	eukaryotic translation initiation factor 2	0.780104	0.008161	0.037684
1370886_a_at	171041	Kns2	kinesin 2	1.124657	0.008161	0.037684
1370330_at	246212	Sipa1l1	signal-induced proliferation-associated	0.944815	0.008171	0.037713
1382579_at	291908	Tnrc9_prec	trinucleotide repeat containing 9 (predi	-0.832378	0.00818	0.037743
1375211_at	292306	Rnaset2_p	ribonuclease T2 (predicted)	-0.951999	0.00819	0.037772
1389489_at	501633	NA	NA	-0.990257	0.008192	0.037772
1384466_at	493574	LOC49357	notch1-induced protein	-1.853177	0.008201	0.037801

1393346_at	499518	RGD1561€	similar to RIKEN cDNA 5830436D01 (|	-1.029325	0.008211	0.037834
1371528_at	290652	Fkbp8	FK506 binding protein 8	1.172057	0.008226	0.037885
1373305_at	360725	Snx4_pred	sorting nexin 4 (predicted)	-0.841802	0.008228	0.037885
1370118_at	117518	Ccl17	chemokine (C-C motif) ligand 17	-2.462612	0.008239	0.037911
1374320_at	304929	F5_mappe	coagulation factor 5 (mapped)	-2.612333	0.008242	0.037911
1389760_at	309457	Pcgf6	polycomb group ring finger 6	-1.036782	0.008242	0.037911
1373754_at	683591	LOC68359	NA	1.008644	0.008246	0.037914
1373955_at	306182	Ranbp5_pr	RAN binding protein 5 (predicted)	0.95348	0.008248	0.037914
1371541_at	288057	Mylk_predi	myosin, light polypeptide kinase (predi	1.21141	0.008254	0.037926
1398843_at	58857	Vapa	vesicle-associated membrane protein,	-0.738917	0.008258	0.037926
1388780_at	307861	Terf2ip	telomeric repeat binding factor 2, intera	-0.935588	0.00826	0.037926
1381461_at	502348	RGD15617	similar to StAR-related protein 1-4E (p	0.988832	0.008263	0.037926
1382081_at	364405	Scara3_pre	scavenger receptor class A, member 3	-4.772515	0.008282	0.037995
1372333_at	360844	Snrpe_pre	small nuclear ribonucleoprotein E (pre	-0.816497	0.008283	0.037995
1372968_at	300147	Sbf1_predi	SET binding factor 1 (predicted)	1.691315	0.008291	0.038008
1369565_at	64546	Il12b	interleukin 12b	4.279498	0.008292	0.038008
1379967_at	306695	Zfp367	zinc finger protein 367	-2.080451	0.008303	0.038047
1378792_at	500633	RGD1564C	similar to Kiaa0575 (predicted)	3.127763	0.008315	0.038075
1396581_at	314717	Scyl2_prec	SCY1-like 2 (S. cerevisiae) (predicted)	1.857508	0.008315	0.038075
1387211_at	117232	Barhl1	BarH-like 1 (Drosophila)	2.644592	0.00832	0.038087
1371343_at	315548	Srpr	signal recognition particle receptor ('dc	1.088433	0.008325	0.038093
1383448_at	305896	Isgf3g	interferon dependent positive acting tra	-1.093955	0.008337	0.038138
1371883_at	303439	Mmd	NA	-0.985614	0.008347	0.038169
1377603_at	361328	Snx24	sorting nexing 24	-0.870076	0.008354	0.038186
1377011_at	304244	RGD1307C	similar to hypothetical protein CG003 (	-1.131171	0.00836	0.0382
1387116_at	24908	Dnajb9	DnaJ (Hsp40) homolog, subfamily B, n	-0.803543	0.008364	0.038206
1391273_at	337920	Naglt1	Na+ dependent glucose transporter 1	-2.997257	0.008376	0.038226
1389810_at	365668	RGD13059	similar to RIKEN cDNA 2610528A15	0.885796	0.008378	0.038226
1380650_at	362956	Triobp	TRIO and F-actin binding protein	1.285146	0.00838	0.038226
1377341_at	288545	RGD13054	similar to hypothetical protein FLJ1092	1.028925	0.008382	0.038226
1382758_at	291942	P7	P7 protein	2.43645	0.008383	0.038226
1370287_a_at	24851	Tpm1	tropomyosin 1, alpha	-1.049769	0.008392	0.038257
1370452_at	24239	Cacna1c	calcium channel, voltage-dependent, L	-0.998377	0.008426	0.038394
1371355_at	296658	Ndufa8	NADH dehydrogenase (ubiquinone) 1	0.726392	0.008428	0.038394
1383449_at	361786	NA	NA	1.224312	0.008432	0.038398
1374258_at	293509	RGD13069	similar to hypothetical protein MGC131	1.872977	0.008438	0.038413
1367749_at	81682	Lum	lumican	-7.37577	0.008452	0.038463
1378871_at	313058	Bai2_predi	brain-specific angiogenesis inhibitor 2	-3.096422	0.008457	0.038474
1373048_at	299121	Actr10	ARP10 actin-related protein 10 homolo	0.79044	0.008466	0.038501
1371565_at	298370	Txndc12	thioredoxin domain containing 12 (end	-0.72207	0.008472	0.038514
1383853_at	304775	Dyrk3	dual-specificity tyrosine-(Y)-phosphory	-2.190141	0.008478	0.038529
1368273_at	58840	Mapk6	mitogen-activated protein kinase 6	-0.763908	0.008494	0.038576
1373151_at	499615	RGD15601	similar to RIKEN cDNA 2810489O06 (	-1.656874	0.008494	0.038576
1389782_at	294499	RGD13055	similar to RIKEN cDNA 2010107G23 (	-0.9629	0.008517	0.038664
1385075_at	296081	Ccdc32	coiled-coil domain containing 32	1.325917	0.008529	0.038708
1389257_at	362375	Lancl2_pre	LanC (bacterial lantibiotic synthetase c	-0.999205	0.008536	0.038727
1371305_at	26962	Rpl8	ribosomal protein L8	0.860263	0.008542	0.038741
1379971_at	311415	Zc3h6_pre	zinc finger CCCH type containing 6 (pr	-1.524736	0.008567	0.03884
1397032_at	304074	Morc3_pre	microrchidia 3 (predicted)	1.626033	0.008582	0.038885
1387044_at	171158	Gpha2	glycoprotein hormone alpha 2	4.04556	0.008584	0.038885
1371962_at	293481	Tufm_pred	Tu translation elongation factor, mitoch	0.731667	0.008586	0.038885

1383582_at	362605	Tmem54	transmembrane protein 54	2.606231	0.008615	0.039004
1388648_at	293454	RGD13066	similar to D7Wsu128e protein	-0.938887	0.008634	0.039076
1374777_at	502894	LOC50289	hypothetical protein LOC502894	-1.714481	0.008641	0.039077
1372507_at	306587	Tcta	T-cell leukemia translocation altered g	0.964278	0.008643	0.039077
1393165_at	305070	RGD13593	similar to RIKEN cDNA 2310028N02	1.02147	0.008644	0.039077
1392485_at	313551	RGD15648	similar to chromosome 1 open reading	2.648234	0.008646	0.039077
1378642_at	311502	RGD1307C	similar to uncharacterized hypothalamu	-1.20088	0.008656	0.039097
1382061_at	307858	Ldhd	lactate dehydrogenase D	1.159827	0.008659	0.039097
1374925_at	314910	Nab2	Ngfi-A binding protein 2	-1.425436	0.008659	0.039097
1370054_at	54238	Cdkn2c	cyclin-dependent kinase inhibitor 2C (p	1.125666	0.008668	0.039123
1373976_at	300146	RGD15639	similar to 8430411H09Rik protein (pre	1.274406	0.008674	0.039136
1370370_at	64468	Hyal2	hyaluronoglucosaminidase 2	0.809139	0.008682	0.039161
1372218_at	363237	Wdr12	WD repeat domain 12	0.815145	0.008687	0.039168
1386547_at	303599	RGD13092	similar to KIAA1636 protein (predicted)	1.470072	0.008699	0.0392
1393043_at	303456	Wdr50_pre	WD repeat domain 50 (predicted)	-2.431431	0.0087	0.0392
1375664_at	308971	Tnrc6_prec	trinucleotide repeat containing 6 (predi	-1.052992	0.008704	0.0392
1388212_a_at	294228	RT1-S3	RT1 class Ib, locus S3	-1.631488	0.008706	0.0392
1369318_at	60398	Fhit	fragile histidine triad gene	-0.755512	0.008717	0.039237
1386829_at	308463	Map3k10	mitogen activated protein kinase kinas	3.426662	0.008724	0.039255
1367696_at	114709	Ifitm2	interferon induced transmembrane pro	-1.202852	0.008734	0.039279
1396939_at	116548	Pcsk5	proprotein convertase subtilisin/kexin t	2.591615	0.008735	0.039279
1393723_at	314627	RGD1305C	similar to Serine/threonine-protein kina	-2.374498	0.008755	0.039354
1372902_at	315212	Alg12_prec	asparagine-linked glycosylation 12 hor	1.094962	0.008775	0.039421
1391330_at	252959	Bm259	BM259 protein	-2.062694	0.008776	0.039421
1373623_at	308451	Itpkc	inositol 1,4,5-trisphosphate 3-kinase C	1.469445	0.00878	0.039425
1398366_at	497968	NA	NA	-0.941031	0.008802	0.03951
1388812_at	287550	Trp53i13_p	tumor protein p53 inducible protein 13	1.84808	0.008805	0.039511
1387098_at	83581	Rpo1-4	RNA polymerase 1-4	1.344	0.008811	0.039525
1371563_at	298594	Rcc2_pred	regulator of chromosome condensation	0.745274	0.008819	0.039545
1375648_at	287736	RGD13079	similar to hypothetical protein (predicte	-2.225862	0.008824	0.039545
1383073_at	291796	Usp14	ubiquitin specific protease 14	0.763599	0.008826	0.039545
1381162_at	309891	LOC30989	similar to septin 10 isoform 1	-1.375505	0.008827	0.039545
1387024_at	116663	Dusp6	dual specificity phosphatase 6	0.96076	0.008841	0.03958
1390527_at	500795	RGD15621	RGD1562114 (predicted)	1.028763	0.008847	0.03958
1385101_a_at	308060	RGD7351C	similar to RIKEN cDNA 0610011N22 g	1.411183	0.008847	0.03958
1392128_at	303348	RGD15614	similar to novel protein (predicted)	1.225012	0.008847	0.03958
1384323_at	289990	Psmc6	proteasome (prosome, macropain) 26S	-0.775814	0.008859	0.039622
1368658_at	25707	Cntf	ciliary neurotrophic factor	0.845325	0.008867	0.03963
1373258_at	361704	Ctsf	cathepsin F	-0.996612	0.008872	0.03963
1376656_at	311856	Usp20_pre	ubiquitin specific protease 20 (predicte	1.03488	0.008873	0.03963
1395649_at	353230	Lhfpl4	lipoma HMGIC fusion partner-like prote	1.918062	0.008873	0.03963
1398988_at	307347	LOC30734	hypothetical protein LOC307347	-0.783701	0.008919	0.039823
1393637_at	301525	Glb1l_pred	galactosidase, beta 1-like (predicted)	3.30432	0.008925	0.039832
1367764_at	25405	Ccng1	cyclin G1	1.208715	0.00895	0.039934
1390020_at	360975	LOC36097	similar to oxoglutarate dehydrogenase	1.244894	0.008961	0.039969
1372202_at	301374	RGD13105	similar to expressed sequence AI5974	-0.956006	0.00897	0.039994
1398901_at	81738	Nup98	nucleoporin 98	1.370395	0.008973	0.039994
1390695_at	498265	LOC49826	similar to hypothetical protein FLJ107C	-1.348077	0.008989	0.040054
1389525_at	363222	Rnf149	ring finger protein 149	0.881455	0.00902	0.040172
1381925_x_at	282546	Arid1b	AT rich interactive domain 1B (Swi1 lik	-1.202283	0.009024	0.040172
1382175_at	499020	RGD15638	similar to Wilms tumor 1-associating p	-0.795315	0.009026	0.040172

1387309_a_at	29559	Grik1	glutamate receptor, ionotropic, kainate	-2.459306	0.009031	0.040172
1398616_at	302671	Ap1s2_pre	adaptor-related protein complex 1, sigi	-1.005931	0.009034	0.040172
1367709_at	29186	Cd63	CD63 antigen	0.987993	0.009035	0.040172
1373337_at	298085	Grhpr_prec	glyoxylate reductase/hydroxypyruvate	1.079199	0.009037	0.040172
1386926_at	94340	Acsl5	acyl-CoA synthetase long-chain family	0.941882	0.00905	0.040218
1367745_at	81821	Dgkz	diacylglycerol kinase zeta	-0.908812	0.00907	0.040282
1371431_at	312703	Pex5_pred	peroxisome biogenesis factor 5 (predic	-0.721503	0.009071	0.040282
1386998_at	24191	Aldoc	aldolase C	-1.043063	0.009078	0.040299
1377096_at	315763	Mtfmt	mitochondrial methionyl-tRNA formyltra	-0.907876	0.009084	0.040314
1378825_a_at	361987	RGD15603	similar to brain specific protein 4 (pred	-0.996578	0.009088	0.040317
1395590_at	501485	LOC50148	NA	1.218637	0.009091	0.040317
1388085_at	259233	Gpx6	glutathione peroxidase 6	3.702659	0.009094	0.040317
1388286_a_at	299602	Cdc34_pre	cell division cycle 34 homolog (S. cere	1.434985	0.009101	0.040336
1373101_at	295543	Pigk	phosphatidylinositol glycan, class K	-0.800216	0.009104	0.040336
1373435_at	287433	Fxr2h_prec	fragile X mental retardation gene 2, au	1.54607	0.009111	0.040338
1388867_at	361178	MGC11283	similar to transcription factor	-0.8343	0.009116	0.040338
1387748_at	25608	Lep	leptin	3.56385	0.009117	0.040338
1391378_at	316737	Lpin2_prec	lipin 2 (predicted)	-1.630209	0.009119	0.040338
1376715_at	365567	Cbara1	calcium binding atopy-related autoanti	1.608255	0.00912	0.040338
1374198_at	315716	Cd276	CD276 antigen	-1.131996	0.009126	0.040352
1373605_at	316085	RGD13078	similar to 106 kDa O-GlcNAc transfera	-0.789423	0.009147	0.040405
1393020_at	500548	NA	NA	-1.673057	0.00915	0.040405
1399001_at	294030	RGD13054	hypothetical LOC294030	-0.751069	0.009153	0.040405
1374478_at	362576	RGD13053	similar to RIKEN cDNA 2610528J11 (p	1.271095	0.009154	0.040405
1372245_at	29231	Wdr39	WD repeat domain 39	0.909425	0.009155	0.040405
1372246_at	64040	Aldh9a1	aldehyde dehydrogenase family 9, sub	0.7123	0.009159	0.040405
1387254_at	59301	Ghrl	ghrelin precursor	-4.416896	0.009161	0.040405
1374010_at	303369	Lig3	ligase III, DNA, ATP-dependent	0.754457	0.009163	0.040405
1370158_at	79433	Myh10	myosin, heavy polypeptide 10, non-mu	1.326953	0.009169	0.040419
1372803_at	290529	RGD13056	similar to DNA segment, Chr 14, ERAT	1.13159	0.009172	0.040421
1398894_at	291339	Commd3	COMM domain containing 3	-1.049146	0.009176	0.040424
1368245_at	116593	Upb1	ureidopropionase, beta	-1.34754	0.009194	0.040488
1370378_at	65262	Atp5a1	ATP synthase, H+ transporting, mitoch	1.132674	0.009212	0.040532
1372306_at	292710	Ethe1_prec	ethylmalonic encephalopathy 1 (predic	0.855676	0.009213	0.040532
1389611_at	25696	Vldlr	very low density lipoprotein receptor	2.048627	0.009216	0.040532
1398767_at	50522	Ubc	ubiquitin C	-1.067312	0.009216	0.040532
1368194_at	170919	Agpat4	1-acylglycerol-3-phosphate O-acyltrans	2.260174	0.009225	0.040556
1388355_at	291295	Rbm17	RNA binding motif protein 17	-1.055284	0.009228	0.040558
1387367_at	29476	Glg1	golgi apparatus protein 1	1.280417	0.009233	0.040568
1377846_a_at	366547	Msh6_pred	mutS homolog 6 (E. coli) (predicted)	-1.105677	0.009247	0.040614
1370404_at	266776	LOC26677	cystatin TE-1	4.866489	0.009252	0.040625
1383361_at	362293	Dnajb6	DnaJ (Hsp40) homolog, subfamily B, n	-0.788684	0.009265	0.040657
1372491_at	313757	RGD15655	similar to Ski protein (predicted)	-1.054964	0.009266	0.040657
1371035_at	246299	Gtf3a	general transcription factor III A	1.553633	0.0093	0.040791
1393561_at	363087	RGD15603	similar to vacuolar protein sorting 13C	-1.267362	0.009323	0.040873
1376483_at	306222	Lsm5_prec	LSM5 homolog, U6 small nuclear RNA	-0.910511	0.009324	0.040873
1388553_at	290577	LOC29057	NA	-0.980067	0.009337	0.040909
1380305_at	501101	nod3l	NOD3-like protein	1.119037	0.009342	0.040909
1371615_at	252900	Dgat2	diacylglycerol O-acyltransferase homo	-0.927973	0.009342	0.040909
1392279_at	500442	LOC50044	similar to dynein, axonemal, intermedia	-1.49464	0.00938	0.041063
1381779_s_at	246136	Man2c1	mannosidase, alpha, class 2C, membe	-1.050367	0.009391	0.041098

1393622_at	291209	RGD1561C	similar to HECT type E3 ubiquitin ligas	1.600118	0.009401	0.041125
1382955_at	308376	Gpr126_pr	G protein-coupled receptor 126 (predic	-1.047054	0.009405	0.041128
1373032_at	290553	Mustn1	musculoskeletal, embryonic nuclear pr	-1.350596	0.009407	0.041128
1372361_at	317381	Ccdc22_pr	coiled-coil domain containing 22 (predi	1.244263	0.009431	0.041208
1368477_at	25391	Atp2a3	ATPase, Ca++ transporting, ubiquitous	0.946502	0.009432	0.041208
1371377_at	29287	Rps19	ribosomal protein S19	0.841899	0.009435	0.041208
1367751_at	29640	Dpm2	dolichol-phosphate (beta-D) mannosyl	0.919899	0.009438	0.041208
1379164_at	316524	Znf142_pre	zinc finger protein 142 (clone pHZ-49)	0.775797	0.009452	0.041249
1372718_at	300963	LOC30096	similar to centrosome protein Cep63	-1.033178	0.009454	0.041249
1372364_a_at	360462	RGD13051	similar to N-terminal asparagine amido	-0.811908	0.009474	0.041319
1369866_at	56825	LOC56825	prochymosin	2.751182	0.009478	0.041319
1388636_at	360554	Rnf167	ring finger protein 167	0.789311	0.009479	0.041319
1385168_at	310775	RGD13065	similar to receptor-interacting factor 1	-1.54494	0.009493	0.041366
1398459_at	25398	Cacna1a	calcium channel, voltage-dependent, F	0.991948	0.009501	0.041388
1378948_at	361104	RGD13061	similar to hypothetical protein FLJ9079	2.685264	0.009509	0.041407
1390653_at	314243	RGD13073	similar to hypothetical protein MGC99C	1.178543	0.009525	0.041464
1378605_at	309106	Sigirr	single immunoglobulin and toll-interleu	1.086371	0.009534	0.041483
1391158_a_at	288663	Atxn2_prec	ataxin 2 (predicted)	-0.760346	0.009537	0.041483
1367588_a_at	25060	Hk3	hexokinase 3	-0.814591	0.009539	0.041483
1373437_at	313030	Ppp1r8_pre	protein phosphatase 1, regulatory (inhi	-0.79025	0.009566	0.041588
1388980_at	308869	MGC7256C	Unknown (protein for MGC:72560)	0.822747	0.009579	0.041628
1380629_at	362000	NA	NA	2.732917	0.009588	0.041654
1381228_at	303287	Fbxo39	F-box protein 39	-2.109058	0.009597	0.041678
1374018_at	498402	RGD15614	similar to ASC-1 complex subunit P10C	1.768351	0.0096	0.041678
1389360_at	116831	Fxyd3	FXYD domain-containing ion transport	-1.303135	0.009604	0.041678
1378352_at	310669	Golph3l	golgi phosphoprotein 3-like	0.792831	0.009606	0.041678
1373938_at	360497	Wdr24	WD repeat domain 24	1.346155	0.009617	0.041701
1389289_at	289752	Ewsr1	Ewing sarcoma breakpoint region 1	-0.844511	0.00962	0.041701
1390801_at	361744	RGD13593	similar to hypothetical protein MGC347	1.312687	0.009623	0.041701
1371284_at	362797	RGD15642	similar to immunoglobulin 4G6 heavy c	1.31547	0.009623	0.041701
1381451_at	94339	Mmp23	matrix metallopeptidase 23	0.825477	0.009628	0.041705
1374254_a_at	300441	RGD15632	RGD1563250 (predicted)	0.874553	0.009636	0.041708
1383290_at	311331	Spint1	serine peptidase inhibitor, Kunitz type	1.122651	0.009637	0.041708
1398928_at	294288	Cuta	divalent cation tolerant protein CUTA	0.709056	0.009641	0.041708
1390580_at	500934	NA	NA	2.619693	0.009641	0.041708
1378728_at	60589	Epha8	Eph receptor A8	1.640014	0.009656	0.041761
1386891_at	29542	Pebp1	phosphatidylethanolamine binding prot	0.720146	0.009661	0.041765
1388321_at	315697	Imp3_pred	IMP3, U3 small nucleolar ribonucleopr	1.16478	0.009669	0.041765
1388395_at	289388	G0s2	G0/G1 switch gene 2	0.917288	0.009671	0.041765
1371633_at	296320	Ctnnbl1	beta catenin-like 1	1.135441	0.009673	0.041765
1374213_at	296380	Arfgef2	ADP-ribosylation factor guanine nucleo	0.945927	0.009675	0.041765
1372447_at	79114	Fgfr1	Fibroblast growth factor receptor 1	-1.94319	0.009677	0.041765
1367871_at	25086	Cyp2e1	cytochrome P450, family 2, subfamily e	1.099149	0.00968	0.041765
1377653_at	366669	LOC36666	similar to mKIAA1011 protein	1.017215	0.009683	0.041765
1372365_at	311494	Rin2_predi	Ras and Rab interactor 2 (predicted)	-1.091751	0.009686	0.041768
1368488_at	114519	Nfil3	nuclear factor, interleukin 3 regulated	-1.356911	0.009699	0.041799
1399113_at	299488	RGD15657	similar to chr2 synaptotagmin (predicte	-0.750686	0.0097	0.041799
1368072_at	54230	Btg3	B-cell translocation gene 3	-0.992641	0.009709	0.041824
1370750_a_at	25663	Il1r1	interleukin 1 receptor, type I	2.027623	0.009715	0.041824
1374210_at	361645	RGD13077	similar to RIKEN cDNA 2510027N19	0.982448	0.009715	0.041824
1367756_at	114017	Gfm	G elongation factor	0.995737	0.00973	0.041871

1388129_at	81785	Ssrp1	structure specific recognition protein 1	0.860966	0.009732	0.041871
1388923_at	290626	RGD13115	similar to 2410004L22Rik protein	1.094852	0.009739	0.041884
1373407_at	315203	RGD13099	similar to hypothetical protein 9930016	-1.127209	0.009744	0.041884
1373077_at	287278	RGD13086	similar to hypothetical protein D11Ertd	0.950299	0.009745	0.041884
1373472_at	314718	Actr6_pred	ARP6 actin-related protein 6 homolog	-0.847034	0.009753	0.041906
1371443_at	362671	RGD13045	similar to RIKEN cDNA A430005L14	0.874746	0.009759	0.041918
1373477_at	297372	Mrpl19	ribosomal protein, mitochondrial, L15	0.89641	0.009765	0.041929
1398943_at	293863	RGD15624	similar to Eso3 protein (predicted)	0.941993	0.009776	0.041965
1376573_at	360571	Rab34	RAB34, member of RAS oncogene far	1.610605	0.009782	0.041976
1369241_at	29462	Avpr1b	arginine vasopressin receptor 1B	-2.175204	0.009793	0.042001
1388467_at	64667	Sgta	small glutamine-rich tetratricopeptide r	1.082126	0.009799	0.042001
1380553_at	313085	RGD13063	similar to Protein C8orf1 (hT41) (predic	-0.763971	0.009799	0.042001
1377593_at	296394	Dpm1_pred	dolichol-phosphate (beta-D) mannosyl	-0.765792	0.009804	0.042001
1369649_at	25399	Cacna2d1	calcium channel, voltage-dependent, a	1.411894	0.009805	0.042001
1390738_at	378947	Bst2	bone marrow stromal cell antigen 2	1.057032	0.009807	0.042001
1373193_at	690089	LOC69008	NA	-0.763002	0.009814	0.042017
1387970_at	192208	Slc38a5	solute carrier family 38, member 5	0.828508	0.009824	0.04204
1390662_at	500084	LOC50008	Ab2-427	-1.168168	0.009826	0.04204
1367753_at	93646	Sec31l1	SEC31-like 1 (S. cerevisiae)	-1.452527	0.009833	0.042044
1368651_at	24651	Pklr	pyruvate kinase, liver and red blood ce	-1.103272	0.009833	0.042044
1367621_at	64391	Dapk3	death-associated protein kinase 3	1.092724	0.009836	0.042046
1372689_at	363150	Tmem103_	transmembrane protein 103 (predicted	1.233461	0.009847	0.042074
1370172_at	24787	Sod2	superoxide dismutase 2, mitochondrial	1.840512	0.009852	0.042074
1390638_at	316539	RGD15605	similar to Eph receptor A4 (predicted)	-3.015273	0.009853	0.042074
1387247_at	25204	Pcsk1	proprotein convertase subtilisin/kexin t	1.281142	0.009866	0.042118
1372555_at	360488	RGD13093	similar to RIKEN cDNA E030034P13 (	0.964579	0.00988	0.042163
1374852_at	362592	RGD15599	RGD1559909 (predicted)	0.883892	0.009891	0.042186
1387141_at	65208	Dpysl5	dihydropyrimidinase-like 5	1.197833	0.009892	0.042186
1372169_at	303514	RGD15647	similar to RIKEN cDNA 4121402D02 (p	-0.702009	0.009895	0.042187
1371596_at	287113	Rnps1	ribonucleic acid binding protein S1	0.718409	0.009904	0.042197
1381220_at	29263	Acvr2a	activin receptor IIA	-1.166049	0.009904	0.042197
1371679_at	499702	RGD15647	similar to Synaptopodin-2 (Myopodin) (	-0.735611	0.009909	0.042205
1368079_at	116551	Pdk1	pyruvate dehydrogenase kinase, isoer	1.42789	0.00994	0.042313
1374154_at	312030	LOC31203	NA	-0.925561	0.00994	0.042313
1389627_at	312303	RGD13062	similar to hypothetical protein FLJ3141	0.75202	0.009953	0.042351
1369042_at	79112	Pigm	phosphatidylinositol glycan, class M	1.054958	0.00996	0.042368
1373913_at	360992	Pnpt1	polyribonucleotide nucleotidyltransfera	-1.028744	0.009969	0.042375
1393218_at	314415	RGD13048	similar to 2410024A21Rik protein	-1.003069	0.009971	0.042375
1387368_at	25482	Mras	muscle and microspikes RAS	0.933606	0.009971	0.042375
1372468_at	361383	Cd97	CD97 antigen	-0.815925	0.009982	0.042407
1376791_at	497903	RGD15646	similar to hypothetical protein A830006	1.384136	0.009993	0.042415
1371664_at	360820	Pxn	paxillin	-1.161378	0.009993	0.042415
1379977_at	309000	RGD13065	similar to hypothetical protein MGC130	1.628289	0.009993	0.042415
1369661_at	25751	Dnm2	dynamin 2	2.687214	0.010004	0.042437
1393247_at	367033	RGD15658	similar to zinc finger protein 560 (predi	-1.692114	0.010005	0.042437
1369657_at	24269	Cpa1	carboxypeptidase A1	-2.974729	0.010011	0.042448
1371587_at	362045	Map2k1ip1	mitogen-activated protein kinase kinas	0.699892	0.010024	0.04249
1387103_s_at	65162	Dio2	deiodinase, iodothyronine, type II	-0.849166	0.010031	0.042506
1398954_at	301529	Dnpep	NA	0.858499	0.01006	0.042618
1376001_at	313245	Praf1_pred	polymerase (RNA) I associated factor	1.21116	0.010074	0.042658
1367614_at	25380	Anxa1	annexin A1	-4.354524	0.010078	0.042658

1371644_at	315265	Ptk9	protein tyrosine kinase 9	0.814993	0.01008	0.042658
1379255_at	302526	Atp6ap2	ATPase, H+ transporting, lysosomal ac	0.7782	0.010088	0.042674
1375003_at	291085	Serpinb6a	serine (or cysteine) peptidase inhibitor	0.947839	0.01009	0.042674
1375980_at	360577	Nxn_predic	nucleoredoxin (predicted)	-1.247359	0.0101	0.042703
1378714_at	363112	RGD13098	similar to hypothetical protein BC0100	-1.993945	0.010109	0.042729
1377729_at	315851	Elovl4_pre	elongation of very long chain fatty acid	-1.338369	0.010117	0.04275
1371671_at	293546	Gprc5b_pr	G protein-coupled receptor, family C, g	1.06748	0.010122	0.04275
1369703_at	29452	Epas1	endothelial PAS domain protein 1	3.552347	0.010124	0.04275
1371274_at	296234	CysS	cystatin S	3.893133	0.010142	0.042811
1367623_at	81766	Rpl18	ribosomal protein L18	0.753089	0.010157	0.042861
1377359_at	501665	LOC50166	similar to protein P3	1.402984	0.010172	0.042906
1396896_at	303130	Fstl4_predi	follistatin-like 4 (predicted)	-1.582281	0.010174	0.042906
1374169_at	360480	RGD13106	similar to chromosome 16 open readin	0.780821	0.010195	0.042979
1382318_at	289019	Pctk3	PCTAIRE-motif protein kinase 3	1.497933	0.010198	0.042979
1367485_at	362479	Tcea1	transcription elongation factor A (SII) 1	-0.73586	0.010205	0.042996
1381355_at	287612	Mks1	Meckel syndrome, type 1	-1.017871	0.010209	0.042999
1381791_at	363391	RGD15620	similar to GTPase activating protein te	1.147787	0.010219	0.043016
1375207_at	287949	Scarf2_pre	scavenger receptor class F, member 2	1.277776	0.01022	0.043016
1376249_at	292485	Fuca2	fucosidase, alpha-L- 2, plasma	0.771794	0.010236	0.043071
1377390_at	288227	Bace2	beta-site APP-cleaving enzyme 2	1.910651	0.010254	0.043126
1367887_at	24530	Lcat	lecithin cholesterol acyltransferase	2.176423	0.010255	0.043126
1388873_at	316575	Trip12	thyroid hormone receptor interactor 12	0.88192	0.01026	0.043133
1383230_at	360664	RGD13110	LOC360664 (predicted)	2.775688	0.010264	0.043135
1367863_at	140932	Bnip1	BCL2/adenovirus E1B 19kDa-interacti	0.741447	0.010279	0.043183
1387057_at	84551	Slc7a8	solute carrier family 7 (cationic amino a	0.992175	0.010287	0.043206
1374529_at	445442	Thbs1	NA	-1.867757	0.010298	0.04323
1383143_at	313633	Ephb2_pre	Eph receptor B2 (predicted)	-0.873101	0.0103	0.04323
1382348_at	65156	Dhodh	dihydroorotate dehydrogenase	1.962503	0.010308	0.04325
1370813_at	64352	Gstm5	glutathione S-transferase, mu 5	-1.863406	0.010311	0.043252
1399035_at	300088	Polr3h_pre	polymerase (RNA) III (DNA directed) p	0.785002	0.010316	0.043254
1374718_at	498089	RGD15651	similar to deltex 3-like (predicted)	-1.78997	0.010319	0.043254
1383117_at	282634	Pxmp4	peroxisomal membrane protein 4	0.743278	0.010324	0.043264
1382934_at	315435	Panx1	Pannexin 1	0.886971	0.010331	0.043281
1387078_at	80849	Inpp4a	inositol polyphosphate-4-phosphatase,	-0.996366	0.01035	0.043346
1387140_at	64666	Taok2	TAO kinase 2	1.028345	0.010358	0.043364
1370334_at	64471	Plekhb1	pleckstrin homology domain containing	-1.954725	0.010374	0.043415
1374107_at	282826	Elac2	elaC homolog 2 (E. coli)	1.446624	0.010377	0.043415
1390188_at	297601	Mrpl51_pre	mitochondrial ribosomal protein L51 (p	1.651998	0.010381	0.043418
1370554_at	114094	Uchl3	ubiquitin carboxyl-terminal esterase L3	0.692748	0.010398	0.043459
1374555_at	289125	Acbd6	acyl-Coenzyme A binding domain cont	-0.870508	0.010398	0.043459
1374176_at	362535	RGD13080	similar to DNA segment, Chr 4, Brigha	-1.311147	0.010406	0.043459
1380008_at	306647	RGD13112	similar to Neurofilament triplet H protei	1.021154	0.010407	0.043459
1373440_at	362498	Mdn1	midasin homolog (yeast)	0.731758	0.010412	0.043459
1398834_at	58960	Map2k2	mitogen activated protein kinase kinas	0.84183	0.010412	0.043459
1377025_at	84392	Kif3a	kinesin family member 3a	-1.039096	0.010413	0.043459
1389354_at	365960	LOC36596	similar to semaF cytoplasmic domain a	-1.703314	0.010422	0.043481
1382387_at	309135	Tmem16a_	transmembrane protein 16A (predicted	1.528428	0.010429	0.043499
1369347_s_at	192211	Prom2	prominin 2	-2.489649	0.010437	0.043507
1387147_at	171058	Rab3c	RAB3C, member RAS oncogene famil	0.68603	0.010438	0.043507
1375910_at	313838	Cdc42ep3_	CDC42 effector protein (Rho GTPase	-0.768001	0.010446	0.043512
1373200_at	291057	Eef1e1_pre	eukaryotic translation elongation factor	0.804217	0.010446	0.043512

1377622_at	308839	Hbxap_pre	hepatitis B virus x associated protein (|	-0.931022	0.01045	0.043512
1391252_at	362814	Rnf41	ring finger protein 41	1.134862	0.010455	0.043512
1388103_at	245953	Tmem37	transmembrane protein 37	-2.847656	0.010457	0.043512
1370516_at	246239	Slc15a3	solute carrier family 15, member 3	3.30164	0.010459	0.043512
1391685_at	361478	Pnldc1	poly(A)-specific ribonuclease (PARN)-l	1.249822	0.010464	0.04352
1394775_at	365652	Col4a3bp_	procollagen, type IV, alpha 3 (Goodpas	3.013709	0.010471	0.043534
1372016_at	299626	Gadd45b	growth arrest and DNA-damage-induci	-2.689739	0.010475	0.043538
1371498_at	288480	MGC12527	JTV1	0.821563	0.010495	0.04358
1393121_at	298961	Agr2_predi	anterior gradient 2 (Xenopus laevis) (p	1.989602	0.010495	0.04358
1371462_at	360622	Igfbp4	insulin-like growth factor binding protei	-1.268909	0.010498	0.04358
1377916_at	303380	Slfn2_pred	schlafen 2 (predicted)	-0.882116	0.0105	0.04358
1383478_at	84598	Jak1	Janus kinase 1	-1.417928	0.010502	0.04358
1384762_at	363174	LOC36317	NA	-1.571215	0.010527	0.043658
1394411_at	500724	RGD15606	similar to novel protein (predicted)	1.269264	0.010527	0.043658
1371043_a_at	192109	Pou3f3	POU domain, class 3, transcription fac	2.49531	0.010535	0.043666
1398000_at	316313	Dst_predic	dystonin (predicted)	-1.645842	0.010535	0.043666
1372408_at	293455	Gga2	golgi associated, gamma adaptin ear c	0.818867	0.010567	0.043782
1394913_at	361943	RGD15658	similar to OL-protocadherin isoform (pr	0.824176	0.010577	0.04381
1395571_at	305624	Rnf3_predi	ring finger protein 3 (predicted)	0.728558	0.010581	0.043813
1371473_at	292696	Clptm1_pre	cleft lip and palate associated transme	0.90204	0.01059	0.043829
1389476_at	294978	RGD13071	similar to RIKEN cDNA D630029K19	-0.860127	0.010591	0.043829
1368276_at	24804	Syp	synaptophysin	0.739168	0.010599	0.043845
1386992_at	29355	Pkn1	protein kinase N1	1.32256	0.010607	0.043866
1382145_at	303787	Slc7a4_pre	solute carrier family 7 (cationic amino a	2.24978	0.01061	0.043866
1389041_at	307842	Vac14	Vac14 homolog (S. cerevisiae)	0.850213	0.010618	0.043884
1378096_at	117550	Kif5b	kinesin family member 5B	1.249427	0.010623	0.043892
1387349_at	25546	Shox2	short stature homeobox 2	1.002057	0.010647	0.043972
1374621_at	361420	LOC36142	similar to TAFI95	1.137714	0.010652	0.043972
1399050_at	289276	Adss_pred	adenylosuccinate synthetase, non mus	-0.883402	0.010652	0.043972
1367586_at	24533	Ldha	lactate dehydrogenase A	5.018579	0.010658	0.04398
1383785_at	161452	Lef1	lymphoid enhancer binding factor 1	1.532914	0.010666	0.044
1393701_at	315951	RGD13102	similar to Alpha-1,4-N-acetylglucosami	-1.10464	0.01067	0.044003
1370320_at	171564	Mawbp	MAWD binding protein	0.776533	0.010682	0.044037
1387938_at	140720	Baalc	brain and acute leukemia, cytoplasmic	1.113908	0.010692	0.044056
1369912_at	54245	Crk	v-crk sarcoma virus CT10 oncogene h	1.19891	0.010696	0.044056
1384391_at	353252	Rdh10	retinol dehydrogenase 10 (all-trans)	1.02726	0.010696	0.044056
1369365_at	50678	Pde3a	phosphodiesterase 3A	2.337757	0.010702	0.044066
1372348_at	500244	RGD15635	similar to AP2 associated kinase 1 (pre	1.329871	0.010706	0.044066
1367940_at	84348	Cmkor1	chemokine orphan receptor 1	-1.86454	0.010712	0.044066
1377263_at	303074	Crsp9_pre	cofactor required for Sp1 transcriptiona	-0.692092	0.010712	0.044066
1387441_at	29553	Kcnk3	potassium channel, subfamily K, meml	-1.431873	0.010729	0.044115
1385660_at	314388	RGD13090	similar to KIAA2010 protein (predicted)	-0.877925	0.010731	0.044115
1384240_at	24180	Agtr1a	angiotensin II receptor, type 1 (AT1A)	-3.659053	0.010737	0.04413
1374479_at	291737	RGD13101	similar to RIKEN cDNA D030070L09	-0.978079	0.010747	0.044153
1374437_at	291556	Nars	asparaginyl-tRNA synthetase	1.156155	0.010759	0.044192
1391714_at	297804	Plag1	pleiomorphic adenoma gene 1	1.128611	0.01077	0.044223
1381246_at	300117	Phf21b_pre	PHD finger protein 21B (predicted)	-1.041759	0.010788	0.044284
1391595_at	315731	Larp6_pred	La ribonucleoprotein domain family, m	-3.211352	0.010794	0.044294
1372634_at	362600	Adprhl2_pr	ADP-ribosylhydrolase like 2 (predicted	0.97029	0.010802	0.04431
1379529_at	501603	NA	NA	-1.506582	0.010806	0.04431
1375378_at	266718	Qki	quaking homolog, KH domain RNA bir	-1.035842	0.010809	0.04431

1373665_at	303238	RGD15631	similar to novel protein (predicted)	-0.682599	0.010815	0.04431
1370883_at	294269	RT1-Da	RT1 class II, locus Da	-2.154534	0.010817	0.04431
1368805_at	29180	Uts2	urotensin 2	1.419848	0.010818	0.04431
1367631_at	64032	Ctgf	connective tissue growth factor	-1.255962	0.010829	0.044342
1389682_at	360994	RGD13091	similar to RIKEN cDNA 2510006C20	-1.263946	0.010838	0.044353
1370956_at	29139	Dcn	decorin	-5.673249	0.010839	0.044353
1368630_at	64822	Fabp9	fatty acid binding protein 9, testis	2.993465	0.010844	0.044362
1375279_at	498423	Sertad2	SERTA domain containing 2	0.790537	0.010866	0.044439
1369946_at	79227	Pdrp	PDRP protein	1.493278	0.01088	0.04448
1393065_at	499935	RGD15625	similar to KIAA0406-like protein (predic	1.051716	0.010888	0.044492
1370243_a_at	29222	Ptma	prothymosin alpha	1.264119	0.010889	0.044492
1374797_at	306261	Eaf1_predi	ELL associated factor 1 (predicted)	1.173563	0.010898	0.044513
1377208_at	290494	Mbnl2_pre	muscleblind-like 2 (predicted)	-0.947242	0.010913	0.044549
1390584_at	307251	RGD15607	similar to ring finger protein 111 (predi	-1.425978	0.010914	0.044549
1368532_at	84028	Pnliprp1	pancreatic lipase related protein 1	-3.333572	0.010926	0.044586
1370359_at	24203	Amy1	amylase 1, salivary	-3.401553	0.010951	0.044674
1382729_at	499783	RGD1564C	similar to GTPase activating RANGAP	-1.151443	0.010982	0.044786
1372400_at	361181	RGD15638	similar to cullin 4A (predicted)	-1.615216	0.010985	0.044787
1368199_at	113929	Nup88	nucleoporin 88	-0.733335	0.010989	0.044787
1382725_at	363273	Cops7b_pr	COP9 (constitutive photomorphogenic	0.770471	0.010992	0.044787
1376209_at	315166	RGD13113	similar to hypothetical protein supporte	0.783063	0.011004	0.044822
1367672_at	79244	Hsd17b4	hydroxysteroid (17-beta) dehydrogena	-1.15135	0.011016	0.044858
1372113_at	287375	Fliih	flightless I homolog (Drosophila)	0.790418	0.011043	0.044953
1373003_at	304582	Sart3_pred	squamous cell carcinoma antigen reco	-0.766257	0.011048	0.04496
1372504_at	502017	RGD15618	similar to dolichyl-phosphate mannosy	0.713624	0.011053	0.044967
1375307_at	315136	Cbx6	chromobox homolog 6	1.148927	0.011079	0.045057
1370159_at	83833	Smarcd2	SWI/SNF related, matrix associated, a	1.337508	0.011084	0.045066
1394392_at	311617	RGD13076	similar to dJ881L22.2 (novel protein) (|	0.918488	0.011091	0.045078
1375439_at	314617	Wdr18	WD repeat domain 18	1.065515	0.011116	0.045168
1370407_at	246302	Pcyox1	prenylcysteine oxidase 1	0.823596	0.011137	0.045228
1374618_at	305152	Aff1_predic	AF4/FMR2 family, member 1 (predicte	-0.746961	0.011138	0.045228
1387505_at	25686	Gnai1	guanine nucleotide binding protein, alp	-2.155105	0.011144	0.045237
1368596_at	59329	Snf1lk	SNF1-like kinase	-1.342457	0.011151	0.045253
1367499_at	311204	Slc35c1_pr	solute carrier family 35, member C1 (p	0.88002	0.011155	0.045257
1395850_at	315554	Pus3_pred	pseudouridylate synthase 3 (predicted	1.666698	0.011163	0.045263
1372899_at	311651	Slc9a8	solute carrier family 9 (sodium/hydroge	1.714071	0.011164	0.045263
1389132_at	192154	Hip1	huntingtin interacting protein 1	1.022811	0.011173	0.045285
1397882_at	313049	Zbtb8_prec	zinc finger and BTB domain containing	-2.486402	0.011176	0.045285
1373094_at	361580	Gtf2h1_pre	general transcription factor II H, polype	-1.019365	0.011179	0.045285
1370199_at	84595	Nucb1	nucleobindin 1	0.777196	0.0112	0.04535
1376765_at	361348	RGD15598	similar to maestro (predicted)	2.300215	0.011202	0.04535
1375061_at	361449	Ppil4_pred	peptidylprolyl isomerase (cyclophilin)-li	-1.043102	0.011214	0.04538
1373009_at	298384	RGD15597	similar to RIKEN cDNA 0610037L13	1.072775	0.011217	0.04538
1371091_at	207125	LOC20712	unknown protein	-1.153686	0.011223	0.045392
1374855_at	287422	Per1	period homolog 1 (Drosophila)	-2.487786	0.011236	0.045423
1369815_at	25542	Ccl3	chemokine (C-C motif) ligand 3	-1.389252	0.011237	0.045423
1377232_at	305332	RGD13052	similar to hypothetical protein (predicte	2.533744	0.01127	0.04554
1386935_at	79240	Nr4a1	nuclear receptor subfamily 4, group A,	-2.327596	0.011298	0.045642
1389268_at	361767	LOC36176	NA	1.174143	0.01131	0.045674
1373821_at	294698	Slc30a5_pr	solute carrier family 30 (zinc transporte	0.778901	0.011313	0.045674
1375901_at	606294	LOC60629	NA	-0.880672	0.011347	0.045784

1383424_at	314004	Tyki_predic	thymidylate kinase family LPS-inducibl	-2.110413	0.011347	0.045784
1373597_at	288564	Pop7_pred	processing of precursor 7, ribonucleas	0.773754	0.011356	0.045806
1381645_at	310652	RGD13111	similar to hypothetical protein	0.709344	0.011365	0.045826
1395671_at	288604	RGD15619	similar to Williams-Beuren syndrome c	2.387463	0.011378	0.045865
1392902_at	366203	RGD13060	similar to chromosome 20 open readin	1.059976	0.011393	0.04591
1376010_at	291078	Prpf4b	PRP4 pre-mRNA processing factor 4 h	-0.954335	0.011407	0.045951
1376693_at	500011	RGD15630	similar to OEF2 (predicted)	-2.509337	0.011413	0.045951
1375191_at	499178	NA	NA	-0.724043	0.011413	0.045951
1376492_at	302987	RGD15652	similar to chromosome 16 open readin	0.937307	0.011432	0.046013
1398652_at	312952	Impad1	inositol monophosphatase domain con	0.87762	0.011441	0.046013
1398913_at	308870	Numa1	nuclear mitotic apparatus protein 1	0.797821	0.011443	0.046013
1392040_at	310807	Sass6_pre	spindle assembly 6 homolog (C. elega	1.583505	0.011444	0.046013
1374745_at	311629	NA	NA	-2.816048	0.011448	0.046013
1367466_at	287530	Prpf8	pre-mRNA processing factor 8	0.739311	0.011449	0.046013
1375654_at	362859	Ckap4_pre	cytoskeleton-associated protein 4 (pre	4.065797	0.011455	0.046022
1371523_at	498425	LOC49842	similar to U2 small nuclear ribonucleop	-0.836915	0.01146	0.046027
1371379_at	289182	RGD15634	similar to Brain protein 44 (predicted)	-1.125302	0.011476	0.04607
1369220_at	114114	Dnm1l	dynamin 1-like	1.409939	0.011478	0.04607
1388819_at	29521	Scamp1	secretory carrier membrane protein 1	-0.813672	0.011499	0.046143
1367646_at	64529	Ctsb	cathepsin B	0.669623	0.011506	0.046157
1381878_at	302935	Ubn1_pred	ubinuclein 1 (predicted)	1.069581	0.011527	0.046228
1389013_at	317275	RGD13084	similar to RIKEN cDNA 1500016H10 (p	1.008041	0.011537	0.046252
1373054_at	85254	Cdw92	CDW92 antigen	0.833898	0.011575	0.046377
1384763_at	362319	RGD13066	similar to RIKEN cDNA 4930500J03	-2.217262	0.011575	0.046377
1392920_at	296102	Ell3	elongation factor RNA polymerase II-lil	0.946396	0.011592	0.046431
1376894_at	317630	Pldn	pallidin	-1.036041	0.011596	0.046432
1387534_at	25310	Cyct	cytochrome c, testis	-3.422058	0.0116	0.046432
1390222_at	305443	RGD13083	similar to RIKEN cDNA 2310079F23 (p	1.12756	0.011611	0.046432
1367571_a_at	24483	Igf2	insulin-like growth factor 2	5.112044	0.011611	0.046432
1368466_a_at	29479	Odf2	outer dense fiber of sperm tails 2	1.406557	0.011612	0.046432
1371836_at	287709	Rab5c_pre	RAB5C, member RAS oncogene famil	0.874341	0.011613	0.046432
1373647_at	294846	Zfp622	zinc finger protein 622	-0.669633	0.011624	0.046461
1367642_at	114597	Suclg1	succinate-CoA ligase, GDP-forming, a	0.852922	0.011629	0.046467
1398574_a_at	306998	Cdc2l5	cell division cycle 2-like 5 (cholinestera	0.820079	0.011649	0.046531
1372550_at	24917	Kpnb1	karyopherin (importin) beta 1	1.00248	0.011656	0.046542
1389531_at	361387	Zfp330_pre	zinc finger protein 330 (predicted)	-0.713106	0.011658	0.046542
1383475_at	24666	Ppm1a	protein phosphatase 1A, magnesium c	-1.677928	0.011673	0.046585
1383358_at	114124	Akap1	A kinase (PRKA) anchor protein 1	0.857756	0.011688	0.046632
1388865_at	297486	Ppp4r2_pre	protein phosphatase 4, regulatory subu	-0.867314	0.011711	0.046711
1389609_at	297725	Tm7sf3	transmembrane 7 superfamily member	0.963108	0.011727	0.046747
1389668_at	295661	Spbc25	spindle pole body component 25 homo	-2.292695	0.011727	0.046747
1379420_at	299135	RGD15650	similar to Dehydrogenase/reductase S	0.794387	0.011734	0.04676
1370136_at	89789	Lbr	lamin B receptor	1.026387	0.011739	0.046766
1368562_at	58953	Sult4a1	sulfotransferase family 4A, member 1	-0.874957	0.011749	0.046792
1377166_at	363235	Als2	amyotrophic lateral sclerosis 2 (juvenile	-1.071551	0.011761	0.046827
1391279_at	298975	Scin	scinderin	-1.627492	0.011768	0.046836
1389472_at	289159	RGD13115	similar to RIKEN cDNA 5630401D24 (p	1.159122	0.011772	0.046836
1390529_at	361226	Cd83_pred	CD83 antigen (predicted)	-2.233682	0.011775	0.046836
1374177_at	310784	Taf13_pred	TAF13 RNA polymerase II, TATA box	0.849414	0.011778	0.046836
1388594_at	312638	Creld1	cysteine-rich with EGF-like domains 1	1.284748	0.011787	0.046848
1379385_at	362721	Tssc1	tumor suppressing subtransferable car	1.266265	0.011788	0.046848

1374665_at	497958	RGD15637	similar to Gem (nuclear organelle) ass	1.973574	0.011793	0.046854
1368139_s_at	25586	Alpl	alkaline phosphatase, tissue-nonspeci	-2.462898	0.011798	0.046858
1367458_at	83510	Lypla2	lysophospholipase 2	1.973554	0.011804	0.046869
1367733_at	54231	Ca2	carbonic anhydrase 2	-1.634076	0.011828	0.046952
1368171_at	24914	Lox	lysyl oxidase	-3.22624	0.011851	0.047015
1389040_at	288165	LOC28816	similar to PEST-containing nuclear pro	-0.833535	0.011851	0.047015
1372065_at	305235	Art3	ADP-ribosyltransferase 3	-1.174058	0.011855	0.047016
1375894_at	316102	Lztfl1	leucine zipper transcription factor-like 1	-1.105559	0.011863	0.047023
1387083_at	29201	Ctf1	cardiotrophin 1	0.808004	0.011864	0.047023
1389504_at	500847	RGD15661	similar to Deltex3 (predicted)	-0.858754	0.011873	0.047044
1376900_at	259271	Aptx	aprataxin	1.156507	0.011877	0.047045
1387145_at	29584	Gjb1	gap junction membrane channel protei	1.291793	0.01188	0.047045
1369298_at	29170	Aqp6	aquaporin 6	1.292673	0.011891	0.047059
1392927_at	302967	Paqr4	progestin and adipoQ receptor family r	1.136235	0.011891	0.047059
1370974_at	286932	Vps54	vacuolar protein sorting 54 (yeast)	-0.670275	0.011912	0.047129
1372645_at	287979	Psarl	presenilin associated, rhomboid-like	0.799394	0.011917	0.047129
1383543_at	362460	Golt1b_pre	golgi transport 1 homolog B (S. cerevis	0.950802	0.011919	0.047129
1391602_at	305470	Drg1	developmentally regulated GTP bindin	1.258302	0.011923	0.04713
1373496_at	296988	MGC94941	similar to Mkrn1 protein	-0.9406	0.011933	0.047155
1370536_at	89820	Hrmt1l3	heterogeneous nuclear ribonucleoprot	1.318929	0.011939	0.047163
1370337_at	83726	Ctcf	CCCTC-binding factor	-0.748858	0.011948	0.047166
1373589_at	305482	Mtmr3	myotubularin related protein 3	-0.658455	0.01195	0.047166
1389312_at	298787	RGD13099	similar to RIKEN cDNA 0610016J10 ge	-0.80876	0.011953	0.047166
1367773_at	29743	Slc25a1	solute carrier family 25, member 1	1.006952	0.011957	0.047166
1392925_at	296911	ST7	suppression of tumorigenicity 7	2.799251	0.011957	0.047166
1381590_at	81918	Pard3	par-3 (partitioning defective 3) homolog	3.826116	0.011961	0.047166
1373806_at	289859	RGD13117	similar to hypothetical protein MGC154	-0.94851	0.012036	0.047448
1370306_at	64460	Pex14	peroxisomal biogenesis factor 14	0.7294	0.012045	0.047464
1370919_at	116655	Hnrpm	heterogeneous nuclear ribonucleoprot	-0.943606	0.012047	0.047464
1382461_at	361402	RGD13048	similar to arginine N-methyltransferase	1.281236	0.012056	0.047484
1393716_at	502421	RGD15595	similar to solute carrier family 35, mem	1.396599	0.012078	0.047556
1387874_at	24309	Dbp	D site albumin promoter binding protei	-1.004115	0.012083	0.047564
1377654_at	501664	RGD15620	similar to RIKEN cDNA 1810037C20 (p	0.68183	0.012095	0.047597
1373038_at	361285	Mllt10	myeloid/lymphoid or mixed-lineage leu	-0.744672	0.012101	0.047606
1374070_at	29326	Gpx2	glutathione peroxidase 2	2.673834	0.012116	0.047632
1377713_at	314374	Ches1_pre	checkpoint suppressor 1 (predicted)	-0.808001	0.012118	0.047632
1379411_at	306366	RGD15638	similar to ENSANGP00000020885 (pre	0.857962	0.012119	0.047632
1379182_at	316219	Trerf1_pre	transcriptional regulating factor 1 (prec	-1.593447	0.012124	0.047635
1367947_at	25520	Prkag1	protein kinase, AMP-activated, gamma	0.668721	0.012132	0.047635
1392770_at	81735	Neo1	neogenin	-0.930789	0.012133	0.047635
1372722_at	295549	Dnajb4	DnaJ (Hsp40) homolog, subfamily B, m	-1.604746	0.012134	0.047635
1372205_at	305471	Zfp278	zinc finger protein 278	-1.56527	0.012152	0.047692
1373304_at	287711	Coasy	Coenzyme A synthase	1.784736	0.012158	0.047702
1375705_at	24400	Gnb1	guanine nucleotide binding protein, bet	0.914976	0.012165	0.047715
1383528_at	299305	RGD15639	similar to mKIAA0215 protein (predicte	-0.852977	0.012172	0.04773
1377615_at	304280	LOC30428	similar to hypothetical protein	0.766262	0.012188	0.047779
1384149_at	362685	RGD13114	similar to hypothetical protein FLJ3834	1.722772	0.012205	0.047817
1368494_at	116547	S100a8	S100 calcium binding protein A8 (calgr	1.289578	0.012206	0.047817
1391703_at	295596	Orc4l	origin recognition complex, subunit 4-li	-1.190401	0.012209	0.047817
1375214_at	292090	Galnt2_pre	UDP-N-acetyl-alpha-D-galactosamine:	2.058278	0.012213	0.04782
1370044_at	140930	Faim	Fas apoptotic inhibitory molecule	-0.889804	0.012218	0.047823

1373863_at	301363	Map4k4_pr	mitogen-activated protein kinase kinas	0.789197	0.012222	0.047827
1389518_at	362511	Fbxo10_pr	F-box only protein 10 (predicted)	0.831589	0.012229	0.047831
1393096_at	292474	RGD15606	similar to ribosomal protein L27a (pred	-0.926917	0.012231	0.047831
1392332_at	362291	Myt1_predi	myelin transcription factor 1 (predicted	-0.788017	0.012253	0.047897
1389524_at	361920	Ythdf3_pre	YTH domain family 3 (predicted)	-1.176813	0.012255	0.047897
1388722_at	361384	Dnajb1_pre	DnaJ (Hsp40) homolog, subfamily B, n	-1.659943	0.012306	0.048083
1373112_at	304285	RGD13068	similar to RIKEN cDNA 2210010N04 g	1.205928	0.012311	0.048089
1373709_at	289900	RGD13595	similar to KIAA0974 protein	-1.01595	0.012316	0.048093
1384778_at	362653	Ctrc	chymotrypsin C (caldecrin)	-2.359785	0.012337	0.048161
1373782_a_at	362100	Endog	endonuclease G	1.116427	0.012352	0.048166
1393955_at	246152	Wdr44	WD repeat domain 44	-1.190148	0.012353	0.048166
1375927_at	499120	LOC49912	hypothetical protein LOC499120	0.975781	0.012357	0.048166
1370393_at	192228	Ccdc5	coiled-coil domain containing 5	1.681119	0.012358	0.048166
1388403_at	361596	LOC36159	NA	0.962448	0.012366	0.048166
1368837_at	84481	Arid4b	AT rich interactive domain 4B (Rbp1 lil	-1.326243	0.012366	0.048166
1383474_at	362418	Irak2	interleukin-1 receptor-associated kinas	-1.504109	0.012367	0.048166
1383623_at	300470	Thyn1	thymocyte nuclear protein 1	0.953071	0.012367	0.048166
1372933_at	303745	Sirt7_predi	sirtuin 7 (silent mating type information	0.898202	0.012374	0.048179
1372325_at	298845	Emilin1_pre	elastin microfibril interfacer 1 (predicte	5.147112	0.012379	0.048183
1383430_at	312649	Tsen2	tRNA splicing endonuclease 2 homolo	1.040728	0.012406	0.048272
1376082_at	294924	Evi1_predi	ecotropic viral integration site 1 (predic	-1.534793	0.012409	0.048272
1390483_at	314441	Slc25a29	solute carrier family 25 (mitochondrial	1.952198	0.012418	0.048294
1384364_at	310363	Fbxo4_pre	F-box only protein 4 (predicted)	0.691423	0.012436	0.04835
1369444_at	25147	Cyp19a1	cytochrome P450, family 19, subfamily	1.469313	0.012459	0.0484
1389767_at	302982	RGD13049	similar to hypothetical protein FLJ3136	2.774873	0.012459	0.0484
1390505_a_at	25104	Pc	Pyruvate carboxylase	-0.767139	0.01246	0.0484
1368423_at	81712	Retnla	resistin like alpha	0.671021	0.012466	0.048408
1380548_at	303968	Ift57_predi	intraflagellar transport 57 homolog (Ch	-0.712464	0.012484	0.048463
1384808_at	306459	Stox2	storkhead box 2	1.945953	0.012493	0.048463
1373466_at	25403	Cast	calpastatin	0.812678	0.012495	0.048463
1368874_a_at	64188	Mafg	v-maf musculoaponeurotic fibrosarcom	1.016988	0.012504	0.048463
1376038_at	303611	Tex2	testis expressed gene 2	0.810361	0.012504	0.048463
1372679_at	308759	Unc45a	unc-45 homolog A (C. elegans)	1.020387	0.012506	0.048463
1386960_at	29573	Slc37a4	solute carrier family 37 (glycerol-6-pho	0.842143	0.012507	0.048463
1370420_at	24950	Srd5a1	steroid 5 alpha-reductase 1	2.067324	0.012509	0.048463
1375026_at	315746	Cln6_predi	ceroid-lipofuscinosis, neuronal 6 (pred	-1.641733	0.012515	0.048469
1374287_at	294508	Pald	paladin	0.921155	0.012522	0.048469
1393572_at	293038	Zfp592_pre	zinc finger protein 592 (predicted)	-1.25602	0.012525	0.048469
1392502_at	360886	Ahctf1_pre	AT hook containing transcription factor	-0.909118	0.012526	0.048469
1388567_at	309041	Thumpd1	THUMP domain containing 1	-0.757049	0.012536	0.048495
1375457_at	311748	Metapl1_pr	methionine aminopeptidase-like 1 (pre	-2.042264	0.012552	0.048544
1371570_at	366951	Scrt1_pred	scratch homolog 1, zinc finger protein	0.959421	0.012587	0.048664
1367484_at	361013	Ube2e2	ubiquitin-conjugating enzyme E2E 2 (U	-0.70588	0.012591	0.048666
1384283_at	367785	Asb9_pred	ankyrin repeat and SOCS box-containi	3.925616	0.012596	0.04867
1392082_a_at	303747	Cd7_predic	CD7 antigen (predicted)	1.002189	0.01261	0.048707
1378288_at	303879	RGD13111	similar to RIKEN cDNA D630035O19 (	2.368373	0.012613	0.048707
1369458_at	84477	Gab2	growth factor receptor bound protein 2	2.465218	0.012625	0.048732
1369933_at	83531	Vdac2	voltage-dependent anion channel 2	0.652824	0.012627	0.048732
1383887_at	362220	RGD13069	similar to Protein C20orf103 precursor	3.520999	0.01263	0.048732
1375157_at	303504	Mllt6_predi	myeloid/lymphoid or mixed lineage-leu	1.135988	0.012641	0.048758
1368160_at	25685	Igfbp1	insulin-like growth factor binding protei	-3.021553	0.012649	0.048773

1396070_at	361119	NA	NA	1.683139	0.012652	0.048773
1388837_at	363024	Slc44a2_pr	solute carrier family 44, member 2 (pre	-1.009878	0.012687	0.048845
1388042_at	246325	Kcnh8	NA	-2.125966	0.012691	0.048845
1371814_at	294331	Ube2g2_pr	ubiquitin-conjugating enzyme E2G 2 (p	0.761072	0.012692	0.048845
1398839_at	116484	Txn1	thioredoxin 1	0.783685	0.012693	0.048845
1389016_at	290864	LOC29086	NA	1.259462	0.012695	0.048845
1388885_at	365872	Aph1a	anterior pharynx defective 1a homolog	1.109786	0.012696	0.048845
1389309_at	304470	Sbno1	sno, strawberry notch homolog 1 (Dros	-0.778429	0.012696	0.048845
1377433_at	308336	Zfp580_pre	zinc finger protein 580 (predicted)	-0.862663	0.012708	0.048874
1395875_at	294595	RGD1306C	similar to KIAA0372 gene product (pre	0.876096	0.012724	0.04892
1372112_at	308906	RGD13045	similar to 9230105E10Rik protein	-0.810575	0.012727	0.04892
1372068_at	291259	Zmynd11	zinc finger, MYND domain containing 1	-0.697901	0.012759	0.04903
1384843_at	287557	Spaca3_pr	sperm acrosome associated 3 (predict	2.721095	0.012765	0.049034
1382034_at	296972	Akr1b10	aldo-keto reductase family 1, member	1.254725	0.012772	0.049034
1371495_at	312689	M6pr	mannose-6-phosphate receptor, cation	0.768895	0.012775	0.049034
1368352_at	24923	Stx1b2	syntaxin 1B2	-2.259236	0.012775	0.049034
1369381_a_at	117261	Slc15a1	solute carrier family 15 (oligopeptide tr	-1.312682	0.012804	0.049122
1395064_at	500551	RGD15649	similar to 4930429A08Rik protein (pred	1.09861	0.012805	0.049122
1383380_at	291317	Rpp38	ribonuclease P/MRP 38 subunit (huma	-0.760262	0.012819	0.049159
1369351_at	54279	Cntn3	contactin 3	1.730288	0.012824	0.049163
1388615_at	295347	Rap1a	RAS-related protein 1a	-0.693909	0.012854	0.049253
1371608_at	287126	Mrps34_pr	mitochondrial ribosomal protein S34 (p	1.1996	0.012858	0.049253
1397183_at	499669	RGD15631	similar to trinucleotide repeat containin	-1.06604	0.012858	0.049253
1396381_at	688736	MGC1162C	NA	3.77964	0.012868	0.049273
1367627_at	81660	Gatm	glycine amidinotransferase (L-arginine	-2.085796	0.012871	0.049273
1368782_at	54305	Sstr2	somatostatin receptor 2	-3.211236	0.012878	0.049283
1379271_at	500616	RGD15649	similar to Suppressor of cytokine signa	-1.152761	0.012881	0.049283
1368135_at	59115	Ninj2	ninjurin 2	1.990367	0.012892	0.049312
1368574_at	24173	Adra1b	adrenergic receptor, alpha 1b	0.97868	0.012908	0.049351
1398375_at	298763	Mta3_pred	metastasis associated 3 (predicted)	0.819681	0.012911	0.049351
1397555_at	499162	NA	NA	-2.11035	0.012914	0.049351
1388499_at	303922	RGD13593	similar to hypothetical protein MGC753	0.833712	0.012933	0.049405
1379668_at	361174	RGD15647	similar to hypothetical protein B230397	1.448775	0.012935	0.049405
1369017_at	116745	Kcnh6	potassium voltage-gated channel, subf	-0.767932	0.012945	0.049426
1370148_at	24464	Hp	haptoglobin	1.859695	0.012966	0.049494
1376016_at	288518	RGD13116	similar to RIKEN cDNA 1110007L15 (p	-0.815186	0.012997	0.049596
1387823_at	60376	Plrg1	pleiotropic regulator 1, PRL1 homolog	-0.752641	0.013003	0.049607
1367840_at	56084	Hgs	HGF-regulated tyrosine kinase substra	0.999992	0.013016	0.049641
1389162_at	297416	Hirip5_pred	histone cell cycle regulation defective i	0.933118	0.013021	0.049647
1367502_at	309140	Mrpl21_pre	mitochondrial ribosomal protein L21 (p	0.966613	0.013027	0.049656
1371109_at	313421	C8b_mapp	complement component 8, beta polype	1.002124	0.013034	0.049667
1375877_at	64440	Syt4	synaptotagmin IV	0.735782	0.013039	0.049672
1397839_at	299156	Rab15	RAB15, member RAS onocogene fam	1.124077	0.01306	0.049726
1369968_at	24924	Ptn	pleiotrophin	-2.092027	0.013061	0.049726
1391935_at	297481	LOC29748	NA	-0.974247	0.013069	0.049743
1377721_at	499021	RGD1561C	similar to PACRG (predicted)	-1.221397	0.013075	0.049753
1386904_a_at	64001	Cyb5	cytochrome b-5	0.824586	0.013109	0.049867
1387860_at	29154	Capn2	calpain 2	-1.111714	0.013115	0.049871
1369134_x_at	117101	Kcnc3	potassium voltage gated channel, Sha	1.624457	0.013118	0.049871
1390848_at	498036	RGD15621	similar to CKT2 (predicted)	1.039563	0.013125	0.049872
1371907_at	288656	Arl6ip4	ADP-ribosylation factor-like 6 interactir	1.245832	0.013125	0.049872

1379304_at	498171	LOC49817	NA	0.72093	0.013138	0.049899
1378914_a_at	308990	LOC30899	hypothetical protein LOC308990	-0.982313	0.013142	0.049899
1388460_at	297339	Capg	capping protein (actin filament), gelsoli	0.944129	0.013144	0.049899
1374847_at	303786	RGD1306C	similar to hypothetical protein FLJ3047	0.742643	0.013148	0.049902
1376268_at	79121	Arf6	ADP-ribosylation factor 6	-1.084434	0.013161	0.049935
1370327_at	245974	Commd5	COMM domain containing 5	1.086571	0.013167	0.049946
1372846_at	361729	Cybasc3	cytochrome b, ascorbate dependent 3	0.864004	0.013179	0.049963
1369196_at	24677	Ppy	pancreatic polypeptide	-1.249246	0.01318	0.049963
1396825_at	304474	Pitpnm2_p	phosphatidylinositol transfer protein, m	2.007779	0.013192	0.049991
1399110_at	309630	Zfp297	zinc finger protein 297	0.937145	0.013194	0.049991
1391865_at	302977	Rnf151_pre	ring finger protein 151 (predicted)	1.05265	0.013236	0.050133
1375636_at	116549	Csnk2a1	casein kinase II, alpha 1 polypeptide	0.670631	0.013261	0.050213
1367899_at	25439	F2r	coagulation factor II (thrombin) recepto	-0.897073	0.01328	0.050271
1369966_a_at	81776	Rps24	ribosomal protein S24	-0.755262	0.013292	0.050294
1394548_at	314855	Cpm_predi	carboxypeptidase M (predicted)	0.871758	0.013293	0.050294
1369393_at	116596	Map3k8	mitogen-activated protein kinase kinas	-4.219318	0.013299	0.050302
1378614_at	29424	Hdh	Huntington disease gene homolog	-1.060714	0.013311	0.05033
1390205_at	310062	Zswim6	zinc finger, SWIM domain containing 6	-1.42827	0.01332	0.050343
1383721_at	266707	Cx39	connexin 39	1.116248	0.013325	0.050343
1380523_at	361354	Fbxo15_pr	F-box protein 15 (predicted)	4.6539	0.013325	0.050343
1376576_at	297412	Dusp11	dual specificity phosphatase 11 (RNA/	1.394841	0.013336	0.050357
1370677_at	207119	Rin1	Ras and Rab interactor 1	3.010725	0.013337	0.050357
1376369_at	300219	Troap_pred	trophinin associated protein (tastin) (pr	1.118679	0.01335	0.050393
1369340_at	56029	Snip	SNAP25-interacting protein	-0.906348	0.013355	0.050398
1398482_at	292700	Bcl3_predi	B-cell leukemia/lymphoma 3 (predicted	-2.905664	0.013392	0.050523
1388832_at	288525	Dhrsx_pred	dehydrogenase/reductase (SDR family	0.839744	0.013398	0.050532
1398757_at	25498	Npm1	nucleophosmin 1	0.716401	0.013421	0.050592
1374248_at	362867	Mybpc1	myosin binding protein C, slow type	1.858118	0.013422	0.050592
1372366_at	192218	Htatip	HIV-1 tat interactive protein, homolog (	0.974653	0.013436	0.05062
1398078_at	313040	RGD15657	similar to CUB and Sushi multiple dom	-1.50083	0.013437	0.05062
1368073_at	24508	Irf1	interferon regulatory factor 1	-1.93909	0.013441	0.05062
1372914_at	297604	Ltbr	lymphotoxin B receptor	0.933499	0.013449	0.050635
1388903_at	363448	Tcte1l	T-complex associated-testis-expresse	-0.672813	0.013486	0.050753
1386861_at	58940	H2afz	H2A histone family, member Z	-0.727432	0.013489	0.050753
1381968_at	289185	Creg_predi	cellular repressor of E1A-stimulated ge	0.956354	0.013497	0.050753
1389232_at	303907	Wdr5b	WD repeat domain 5B	1.017482	0.013497	0.050753
1383341_at	290291	Cab39l	calcium binding protein 39-like	1.361388	0.013499	0.050753
1391848_at	361317	Rbm27_pre	RNA binding motif protein 27 (predicte	0.944951	0.013514	0.050786
1384550_at	317344	RGD15656	similar to RIKEN cDNA 1810030O07 (	-1.480377	0.013516	0.050786
1369865_at	25660	Cd28	CD28 antigen	1.127311	0.013524	0.050805
1375456_at	309399	RGD13052	similar to Cgi67 serine protease precu	0.63868	0.013532	0.050819
1369947_at	29175	Ctsk	cathepsin K	-3.025512	0.013551	0.050867
1396578_at	117253	Dnah11	dynein, axonemal, heavy polypeptide 1	3.095315	0.013553	0.050867
1376415_at	311903	Mrrf	mitochondrial ribosome recycling facto	0.744467	0.013556	0.050867
1373980_at	500619	NA	NA	1.128913	0.013561	0.05087
1386846_at	291736	RGD13073	similar to hypothetical protein MGC363	1.264577	0.013568	0.050872
1393162_at	291733	Slc39a6	solute carrier family 39 (metal ion trans	0.854692	0.013569	0.050872
1382390_at	362106	Fubp3	far upstream element (FUSE) binding	-1.038316	0.013614	0.051013
1390118_at	311166	RGD13076	similar to ATP/GTP-binding protein	0.68984	0.013615	0.051013
1380565_at	304928	RGD13067	similar to 2810422O20Rik protein	1.261264	0.013619	0.051016
1374135_at	290228	Ipo4_predi	importin 4 (predicted)	1.058088	0.013627	0.051023

1376294_at	497916	RGD15607	similar to Smith-Magenis syndrome ch	0.939422	0.013629	0.051023
1367604_at	338401	Crip2	cysteine-rich protein 2	0.984136	0.013636	0.051037
1377618_at	363243	Klf7_predic	Kruppel-like factor 7 (ubiquitous) (pred	-1.154542	0.013649	0.05107
1372789_at	362425	Zfp637	zinc finger protein 637	-0.75427	0.013659	0.051078
1386974_at	171434	Phldb1	pleckstrin homology-like domain, famil	-2.222	0.013659	0.051078
1377156_at	365486	NA	NA	-1.872426	0.013667	0.051095
1372735_at	292762	Eif3s12_pr	eukaryotic translation initiation factor 3	0.738544	0.013684	0.051141
1370084_at	24271	Cpb1	carboxypeptidase B1 (tissue)	-3.292043	0.013691	0.051143
1386947_at	83502	Cdh1	cadherin 1	-0.887187	0.013692	0.051143
1369370_s_at	66026	Trpv4	transient receptor potential cation char	3.274844	0.013704	0.051176
1372941_at	296278	Pdrg1	p53 and DNA damage regulated 1	1.192455	0.013721	0.051224
1384253_at	364558	LOC36455	similar to palladin; CGI-151 protein	1.261001	0.013725	0.051224
1370910_at	116468	Rfc2	replication factor C (activator 1) 2	0.661952	0.013749	0.051301
1384625_at	366244	RGD13062	similar to RIKEN cDNA 2410116G06 (	3.242045	0.01377	0.051362
1373292_at	309001	RGD1311€	similar to KIAA0339 protein	0.894619	0.01378	0.051381
1369092_at	117513	Sec22l2	sec22 homolog	1.037983	0.013783	0.051381
1370613_s_at	154516	Ugt1a7	UDP glycosyltransferase 1 family, poly	0.718787	0.013787	0.051381
1389463_at	25521	Prkar1b	protein kinase, cAMP dependent regul	0.685903	0.013822	0.051498
1392922_at	170923	Rap2b	RAP2B, member of RAS oncogene far	-0.887615	0.013839	0.051547
1389993_at	307524	Wdr33_pre	WD repeat domain 33 (predicted)	0.746355	0.01385	0.051573
1392165_at	290744	Ing1l_predi	inhibitor of growth family, member 1-lik	-0.78445	0.013856	0.051576
1389238_at	289740	LOC28974	similar to PES1 protein	0.717524	0.013858	0.051576
1398996_at	308765	RGD1311C	hypothetical LOC308765 (predicted)	1.328251	0.013869	0.051589
1388235_at	498341	VCS-beta1	PR-Vbeta1	-2.69825	0.013872	0.051589
1371242_at	60590	Grm8	glutamate receptor, metabotropic 8	1.154374	0.013879	0.051589
1374457_at	366697	Pomt2	protein-O-mannosyltransferase 2	1.276613	0.013879	0.051589
1367876_at	116458	Ipo13	importin 13	1.64236	0.013881	0.051589
1368057_at	25270	Abcd3	ATP-binding cassette, sub-family D (A	0.798915	0.013906	0.051644
1392855_x_at	361706	MGC10583	similar to Ras-related protein Rab-1B	1.088003	0.013906	0.051644
1370161_at	64387	Ssg1	steroid sensitive gene 1	-4.613773	0.013909	0.051644
1399083_at	360831	Wbscr21	Williams Beuren syndrome chromosom	1.079916	0.013912	0.051644
1390884_a_at	316583	B3gnt7	UDP-GlcNAc:betaGal beta-1,3-N-acet	0.844968	0.013927	0.051685
1389156_at	498606	LOC49860	hypothetical protein LOC498606	1.027977	0.013934	0.051696
1396106_at	307548	RGD15619	similar to dystrobrevin alpha isoform 1	-1.393996	0.013948	0.051734
1378220_at	497860	LOC49786	similar to RIKEN cDNA 4930517K11	-0.748283	0.013958	0.051752
1367507_at	293703	Nudt22	nudix (nucleoside diphosphate linked r	1.226899	0.013961	0.051752
1388919_at	308108	Znf541_pre	zinc finger protein 541 (predicted)	-1.565499	0.013964	0.051752
1386888_at	116636	Eif4ebp1	eukaryotic translation initiation factor 4	2.613704	0.01397	0.051759
1370208_at	58851	Nr1h2	nuclear receptor subfamily 1, group H,	1.372788	0.013985	0.051793
1373263_at	499312	NA	NA	0.632172	0.013987	0.051793
1390340_a_at	54242	Cpa3	carboxypeptidase A3	3.138184	0.014009	0.051859
1398845_at	56783	Eif5	eukaryotic translation initiation factor 5	-0.66429	0.014029	0.051915
1374806_at	313017	Sfn_predic	stratifin (predicted)	1.444096	0.014032	0.051915
1369995_at	140657	Faf1	Fas-associated factor 1	0.825135	0.014036	0.051915
1369888_at	24952	Gcg	glucagon	-1.89471	0.014057	0.051965
1377446_at	500132	RGD15639	similar to phosphoinositol 4-phosphate	2.108383	0.014059	0.051965
1379550_a_at	246770	Gtf2ird1	general transcription factor II I repeat c	-0.748391	0.014061	0.051965
1388941_at	360650	RGD13048	similar to X83328 protein	0.890182	0.014066	0.051968
1388507_at	305506	Itgb4bp	integrin beta 4 binding protein	1.166442	0.014073	0.05198
1368037_at	29224	Cbr1	carbonyl reductase 1	0.696775	0.014096	0.052052
1386096_at	360905	Mtf2	metal response element binding transc	0.829285	0.0141	0.052053

1387060_at	58954	Klf6	Kruppel-like factor 6	-0.829901	0.014112	0.05208
1387872_at	29578	Hnrpa1	heterogeneous nuclear ribonucleoprot	-1.016458	0.01413	0.052132
1368550_at	64826	Foxq1	forkhead box Q1	1.591042	0.014161	0.052232
1376801_at	294291	RGD15644	RGD1564450 (predicted)	1.225984	0.014168	0.052245
1371463_at	192246	Phf5a	PHD finger protein 5A	0.834835	0.01418	0.052273
1372609_at	363193	Ppp2r5d	protein phosphatase 2, regulatory subu	0.856739	0.014195	0.052314
1387805_at	84480	Bnip3	BCL2/adenovirus E1B 19 kDa-interact	1.822109	0.0142	0.052316
1367742_at	25756	Cpt1b	carnitine palmitoyltransferase 1b, musc	-0.826447	0.014203	0.052316
1371932_at	361137	RGD13093	similar to hypothetical protein FLJ3848	1.044994	0.014238	0.052422
1379664_at	305955	RGD13077	similar to KIAA0853 protein (predicted)	0.760758	0.01424	0.052422
1368370_at	54223	Adcy4	adenylate cyclase 4	2.514056	0.014253	0.052457
1393843_at	315745	Fem1b_pre	feminization 1 homolog b (C. elegans)	-0.984351	0.01427	0.052502
1392109_at	311372	RGD15625	similar to hypothetical protein FLJ2143	-1.263009	0.014274	0.052502
1393792_at	362694	NA	NA	-0.871602	0.014278	0.052502
1372692_at	303882	Tnk2	tyrosine kinase, non-receptor, 2	-0.782593	0.014298	0.052561
1389885_at	360646	Limd2	LIM domain containing 2	0.772364	0.014306	0.052576
1384130_at	367949	RGD15601	similar to PRO0149 protein (predicted)	1.486053	0.014314	0.052592
1371121_at	246151	Sert1	Sertoli cell protein 1	1.00799	0.01432	0.0526
1382430_at	315959	Msl2l1_pre	male-specific lethal 2-like 1 (Drosophila	-0.885078	0.014327	0.052601
1387919_at	64476	Mfn2	mitofusin 2	1.090151	0.014328	0.052601
1368173_at	60373	Nol5	nucleolar protein 5	-0.688201	0.014358	0.052689
1387114_at	170538	Prkcd	protein kinase C, delta	-0.808233	0.01436	0.052689
1379665_at	294711	Ppwd1_pre	peptidylprolyl isomerase domain and W	-0.750707	0.01438	0.052748
1382812_at	303764	RGD13104	similar to Protein Njmu-R1 (predicted)	1.394887	0.014386	0.052754
1372080_at	312444	Immt	inner membrane protein, mitochondria	0.89283	0.014404	0.052804
1376867_at	360983	RGD13074	similar to RIKEN cDNA 1110067D22 (p	-1.376383	0.014424	0.052849
1392397_at	498333	Bmp2k	BMP-2 inducible kinase	-2.531787	0.014424	0.052849
1382238_at	361849	RGD15603	similar to cell division cycle and apopto	0.86806	0.014429	0.052854
1376871_at	288549	RGD15602	similar to cell surface receptor FDFAC	1.011312	0.014447	0.052903
1379393_at	316521	Vil1_predic	villin 1 (predicted)	2.110698	0.014456	0.052923
1397645_at	287702	Ka17	type I keratin KA17	0.752843	0.014514	0.053121
1368653_a_at	117287	Park7	Parkinson disease (autosomal recessiv	0.645563	0.014531	0.053153
1376753_at	310935	Fpgt	fucose-1-phosphate guanylyltransferas	-0.814806	0.014536	0.053153
1367565_a_at	25319	Fth1	ferritin, heavy polypeptide 1	0.623922	0.014539	0.053153
1371634_at	293113	RGD13056	similar to RIKEN cDNA 1810020E01	0.644881	0.014539	0.053153
1377235_a_at	309179	Ppp2r5b	protein phosphatase 2, regulatory subu	0.682744	0.01455	0.053164
1387152_at	58839	Nrbf2	nuclear receptor binding factor 2	-0.642397	0.01455	0.053164
1374650_at	291044	Nedd9	neural precursor cell expressed, develo	-1.056586	0.014555	0.053168
1394128_at	25642	Cyp3a3	cytochrome P450, subfamily 3A, polyp	-2.998758	0.014565	0.053168
1388428_at	362227	Hars2_pred	histidyl tRNA synthetase 2 (predicted)	-0.787889	0.014569	0.053168
1371875_at	310864	Manba	mannosidase, beta A, lysosomal	-1.081312	0.01457	0.053168
1392221_at	498819	RGD15623	similar to GREB1 protein isoform a (pr	-1.529088	0.014571	0.053168
1390629_at	312398	Smarcad1_	SWI/SNF-related, matrix-associated ac	1.505664	0.014585	0.053205
1370538_at	286966	Lama3	laminin, alpha 3	2.894553	0.014591	0.053207
1390455_at	293050	Abhd2_pre	abhydrolase domain containing 2 (pred	2.362938	0.014596	0.053207
1367780_at	64193	Pttg1	pituitary tumor-transforming 1	-1.450328	0.0146	0.053207
1372709_at	298943	Bcap29	B-cell receptor-associated protein 29	-1.112321	0.014602	0.053207
1371185_at	114517	Itga6	integrin, alpha 6	1.426975	0.014614	0.053228
1371421_at	361902	RGD13054	similar to FLJ40243 protein	-1.203235	0.014616	0.053228
1386890_at	81778	S100a10	S100 calcium binding protein A10 (calp	0.972023	0.014638	0.053295
1388362_at	290671	Ndufa13_p	NADH dehydrogenase (ubiquinone) 1	0.700375	0.014651	0.053309

1384957_at	304962	Atf6_predic	activating transcription factor 6 (predic	1.610811	0.014658	0.053309
1368343_at	117018	Kcnh2	potassium voltage-gated channel, subf	1.618696	0.014661	0.053309
1372791_at	116477	Prkwnk1	protein kinase, lysine deficient 1	-0.813086	0.014661	0.053309
1388152_at	25595	Mtap2	microtubule-associated protein 2	-1.250708	0.014662	0.053309
1383601_at	308720	Dufd1_pred	DUF729 domain containing 1 (predicte	-1.12854	0.01467	0.05331
1374841_at	499724	RGD1560C	similar to RIKEN cDNA 2410004B18 (p	-0.900232	0.014671	0.05331
1393242_at	287170	RGD13109	similar to chromosome 16 open readin	0.785897	0.014681	0.053334
1397380_at	306203	Pxk	PX domain containing serine/threonine	1.174291	0.014712	0.053432
1387400_at	24409	Grin2a	glutamate receptor, ionotropic, N-meth	-2.224304	0.014718	0.053437
1388776_at	301013	MGC94600	scotin	0.644454	0.014737	0.053494
1369980_s_at	116504	Mrip	myosin phosphatase-Rho interacting p	0.743479	0.014766	0.053571
1369061_at	116686	Gsr	glutathione reductase	1.962855	0.01477	0.053571
1392294_at	501285	LOC50128	NA	1.2852	0.014771	0.053571
1378856_at	501628	RGD15625	similar to transcription elongation facto	-1.338845	0.014782	0.053596
1375278_at	361970	Trim2	tripartite motif protein 2	-0.864514	0.014786	0.053597
1374683_at	305941	Sgcg	sarcoglycan, gamma (dystrophin-asso	1.561013	0.014792	0.053606
1388069_at	25192	Klrb1b	killer cell lectin-like receptor subfamily	-2.942996	0.014799	0.053614
1378776_at	116545	Pou6f1	POU domain, class 6, transcription fac	-1.563252	0.014807	0.05363
1369700_at	29233	Clcn7	chloride channel 7	2.093306	0.014816	0.053647
1371347_at	360698	Tmem50b	transmembrane protein 50B	-0.80589	0.014827	0.053672
1373034_at	288233	Wrb	tryptophan rich basic protein	0.672671	0.014834	0.053682
1379656_a_at	313235	Coro2a	coronin, actin binding protein 2A	0.718554	0.014846	0.053713
1374255_at	288917	Farsla	phenylalanine-tRNA synthetase-like, a	1.410495	0.014856	0.05373
1389746_at	360630	RGD15642	similar to Naglu (predicted)	1.335019	0.014859	0.05373
1394028_at	63995	Dusp10_pr	dual specificity phosphatase 10 (predic	-0.949641	0.01487	0.053742
1389824_at	25400	Camk2a	calcium/calmodulin-dependent protein	-0.745901	0.014871	0.053742
1372924_at	497914	RGD15636	similar to Mediator of RNA polymerase	0.668971	0.014879	0.053759
1372008_at	363521	Taz_mappe	tafazzin (cardiomyopathy, dilated 3A (X	0.94193	0.01491	0.053854
1389167_at	296648	Mapkap1	mitogen-activated protein kinase asso	0.964941	0.014931	0.053917
1368817_at	498433	Psme4	NA	0.7693	0.014949	0.053967
1380066_at	288562	Trfr2_predi	transferrin receptor 2 (predicted)	-1.867631	0.014954	0.053968
1373553_at	287930	Top3b_pre	topoisomerase (DNA) III beta (predicte	0.955386	0.014958	0.053968
1384367_at	690148	LOC69014	NA	1.058906	0.014982	0.05404
1398296_at	60418	Mir16	membrane interacting protein of RGS1	0.720985	0.014986	0.054041
1387866_at	25486	Myo9b	myosin IXb	1.049633	0.015019	0.054142
1388813_at	79119	Arf2	ADP-ribosylation factor 2	-1.169727	0.015022	0.054142
1390765_at	286888	Wfdc2	WAP four-disulfide core domain 2	-3.319745	0.015036	0.054155
1379778_at	298032	RGD13079	similar to HSPC043 protein (predicted)	-0.734453	0.01504	0.054155
1398755_at	170667	Atp6v0c	ATPase, H transporting, lysosomal V0	0.887492	0.01504	0.054155
1373068_at	300994	Ifrd2_predi	interferon-related developmental regul	1.313867	0.015042	0.054155
1372094_at	308472	Supt5h	suppressor of Ty 5 homolog (S. cerevi	0.672425	0.015046	0.054155
1371701_at	299954	Ndufb9_pre	NADH dehydrogenase (ubiquinone) 1	0.704364	0.015059	0.054176
1382478_at	311462	Btbd3_prec	BTB (POZ) domain containing 3 (predi	-1.604314	0.015061	0.054176
1374582_at	364410	Kctd9_prec	potassium channel tetramerisation dor	0.618604	0.015064	0.054176
1368084_at	25633	Dnase1	deoxyribonuclease I	3.56083	0.015074	0.054196
1373819_at	500855	LOC50085	hypothetical protein LOC500855	-0.674281	0.015078	0.054196
1391121_at	306506	RGD13117	similar to DNA segment, Chr 8, ERATC	-1.079172	0.015087	0.054215
1389067_at	171144	Slco4a1	solute carrier organic anion transporte	1.487894	0.015122	0.054326
1393018_at	502072	RGD15655	similar to RING finger protein 33 (pred	1.462165	0.015135	0.054345
1393082_at	171010	Ppp1r14c	protein phosphatase 1, regulatory (inhi	1.161224	0.015136	0.054345
1380671_at	690093	LOC69009	NA	1.544311	0.015141	0.05435

1388717_at	309686	Pofut2_pre	protein O-fucosyltransferase 2 (predict	0.77748	0.015151	0.05437
1390032_at	288771	Rbms2	RNA binding motif, single stranded inte	1.497579	0.015162	0.054395
1367620_at	114630	Atp5g3	ATP synthase, H+ transporting, mitoch	0.66261	0.015169	0.054404
1383197_at	619374	Jam2	junction adhesion molecule 2	-2.864953	0.015186	0.05445
1391538_at	364388	Cideb_prec	cell death-inducing DNA fragmentation	1.436199	0.015231	0.054598
1388699_at	361378	Man2b1	mannosidase 2, alpha B1	0.754655	0.015253	0.054663
1387898_at	192245	Hspb6	heat shock protein, alpha-crystallin-rela	-0.746214	0.015262	0.054678
1387766_a_at	24710	Rbp2	retinol binding protein 2, cellular	3.411328	0.015269	0.054688
1398965_at	316052	LOC31605	eomesodermin	0.653795	0.015276	0.054699
1373424_at	303794	Dgcr6_prec	DiGeorge syndrome critical region gen	-0.851694	0.015298	0.054743
1382340_at	498863	NA	NA	1.408387	0.015299	0.054743
1398807_at	24667	Ppm1b	protein phosphatase 1B, magnesium c	-0.757508	0.015301	0.054743
1378406_at	310315	Ccdc39_pr	coiled-coil domain containing 39 (predi	0.751068	0.015313	0.054769
1379676_a_at	363522	Dnase1l1	deoxyribonuclease 1-like 1	1.033651	0.015316	0.054769
1367683_at	85245	Kpna2	karyopherin (importin) alpha 2	1.141382	0.01532	0.054769
1374719_at	309637	RGD13072	similar to hypothetical protein FLJ2030	0.658883	0.015341	0.054807
1378387_at	290232	Tinf2	TERF1 (TRF1)-interacting nuclear fact	0.920428	0.015342	0.054807
1371094_at	117555	Lhx2	LIM homeobox protein 2	0.970462	0.015345	0.054807
1372485_at	29700	Pcbd1	pterin 4 alpha carbinolamine dehydrata	-0.698566	0.015347	0.054807
1372751_at	500936	NA	NA	0.82282	0.015356	0.054814
1376313_at	309139	Tpcn2_pre	two pore segment channel 2 (predicted	2.744474	0.015358	0.054814
1373074_at	307210	RGD13072	similar to RIKEN cDNA 2700002I20	-0.77447	0.01537	0.054826
1387491_at	79223	Gyk	glycerol kinase	-1.849128	0.015371	0.054826
1374489_at	363195	Gtpbp2	GTP binding protein 2	1.359509	0.015374	0.054826
1379307_at	266809	Sap1	sodium channel associated protein 1	-1.671587	0.015384	0.054837
1373377_at	310662	Scnm1_pre	sodium channel modifier 1 (predicted)	0.782387	0.015386	0.054837
1387217_a_at	29446	Ghrh	growth hormone releasing hormone	-1.833131	0.015389	0.054837
1368325_at	25313	Egf	epidermal growth factor	-0.935871	0.015394	0.05484
1374273_at	89843	Cxadr	coxsackie virus and adenovirus recept	-1.582503	0.015398	0.05484
1388628_at	300888	Tmed3	transmembrane emp24 domain contai	0.831379	0.015416	0.05488
1389445_at	293511	Zfp688_pre	zinc finger protein 688 (predicted)	1.177166	0.015418	0.05488
1384878_at	291318	Nmt2	N-myristoyltransferase 2	0.690428	0.015449	0.054976
1388217_a_at	64366	Calu	calumenin	0.947539	0.015475	0.055045
1389322_at	363082	Pdcd7_pre	programmed cell death protein 7 (pred	-0.773267	0.015477	0.055045
1380727_at	304054	Prdm15_pr	PR domain containing 15 (predicted)	3.111231	0.015492	0.055086
1373121_at	361919	Mrps28	mitochondrial ribosomal protein S28	0.748314	0.015502	0.055106
1373977_at	311024	Kif5c_pred	kinesin family member 5C (predicted)	0.71947	0.015518	0.055147
1371382_at	293860	Flna_predi	filamin, alpha (predicted)	1.375276	0.015539	0.055187
1392444_at	307859	RGD15611	similar to N-acetylglucosamine 6-O-su	1.958541	0.015544	0.055187
1383281_at	287918	RGD13062	similar to RIKEN cDNA 1110005A03	-0.755034	0.015545	0.055187
1369481_at	89814	Tnfsf4	tumor necrosis factor (ligand) superfan	0.804904	0.015546	0.055187
1378127_at	361258	Cul2_predi	cullin 2 (predicted)	-0.95555	0.01555	0.055187
1388431_at	361295	Ss18	synovial sarcoma translocation, Chrom	0.751002	0.015566	0.055226
1390161_at	304138	LOC30413	similar to cysteine and tyrosine-rich pro	-1.68681	0.015572	0.055226
1371930_at	290868	Qars	glutaminyl-tRNA synthetase	0.702789	0.015573	0.055226
1372907_at	436582	Atp6v0e2	ATPase, H+ transporting, V0 subunit E	0.908466	0.015596	0.055287
1373443_a_at	294679	Ankra2	ankyrin repeat, family A (RFXANK-like	-1.114526	0.015599	0.055287
1370433_at	246211	Hsd3b7	hydroxy-delta-5-steroid dehydrogenase	1.936094	0.015606	0.055299
1389188_at	24232	C3	complement component 3	1.396563	0.015614	0.055302
1370743_a_at	171105	Lnpep	leucyl/cystinyl aminopeptidase	1.882372	0.015616	0.055302
1374191_at	303008	Rhbdf1	rhomboid family 1 (Drosophila)	-3.148228	0.01565	0.055411

1368112_at	25539	Sag	retinal S-antigen	2.370406	0.015698	0.055564
1368756_at	64669	Thedc1	thioesterase domain containing 1	1.176084	0.015733	0.055641
1380389_at	24929	Ptger3	prostaglandin E receptor 3 (subtype EI	0.954115	0.015738	0.055641
1369433_at	60347	Ip63	IP63 protein	-1.131399	0.01574	0.055641
1398599_at	500972	RGD15657	similar to integral membrane protein 1	0.674453	0.015741	0.055641
1389623_at	315305	Atf1	activating transcription factor 1	-0.748414	0.015744	0.055641
1377696_at	308795	Mesdc1	mesoderm development candidate 1	-0.849392	0.015745	0.055641
1373155_at	293054	Mrpl46	mitochondrial ribosomal protein L46	0.649707	0.015757	0.055669
1398169_at	81811	Thpo	thrombopoietin	2.975646	0.015767	0.055691
1379027_at	303039	RGD13083	similar to KIAA0869 protein (predicted)	-0.676542	0.015776	0.055699
1387824_at	56763	Sfrs12	splicing factor, arginine/serine-rich 12	-1.113697	0.015779	0.055699
1399120_at	362754	Trmt5_pred	TRM5 tRNA methyltransferase 5 homo	1.17443	0.015788	0.055699
1390634_at	288106	RGD15663	similar to KIAA1407 (predicted)	3.14823	0.015788	0.055699
1377792_at	291445	Megf10	Megf10 protein	1.860308	0.015791	0.055699
1386983_at	25709	Hmbs	hydroxymethylbilane synthase	0.783871	0.015797	0.055706
1374914_at	25682	Ppard	peroxisome proliferator activated recep	-0.893179	0.015803	0.055706
1388352_at	362228	Nat5_predi	N-acetyltransferase 5 (ARD1 homolog	0.751581	0.015806	0.055706
1389654_at	315926	Pls1_predi	plastin 1 (I isoform) (predicted)	-0.885107	0.015811	0.055706
1369641_at	64189	Pafah1b2	platelet-activating factor acetylhydrolas	0.880571	0.015814	0.055706
1369440_at	155192	Abcg8	ATP-binding cassette, sub-family G (W	4.272925	0.015837	0.055773
1374792_at	498727	RGD15659	similar to nucleolar protein 7, 27kDa (p	-0.94136	0.015855	0.055822
1391083_at	306279	Arhgap22_	Rho GTPase activating protein 22 (pre	3.144203	0.015865	0.05584
1392834_at	303395	RGD13101	similar to Chromodomain-helicase-DN/	-1.00555	0.015869	0.05584
1376072_at	301634	RGD13099	hypothetical LOC301634	-1.301314	0.015884	0.055878
1384185_at	314300	RGD13077	similar to RIKEN cDNA 2410016O06 (	0.704403	0.015906	0.055935
1399010_at	312710	Cops7a_pr	COP9 (constitutive photomorphogenic	0.834263	0.015911	0.055935
1376117_at	294255	Slc44a4	solute carrier family 44, member 4	1.359538	0.015913	0.055935
1374427_at	80977	Syt13	synaptotagmin XIII	0.960297	0.015923	0.055946
1373408_at	366995	Tbca	tubulin cofactor a	-0.617133	0.015928	0.055946
1398918_at	361816	Zfand3	zinc finger, AN1-type domain 3	0.859053	0.015931	0.055946
1373413_at	360952	RGD13104	similar to RIKEN cDNA 2810021O14 (	0.725834	0.015933	0.055946
1372522_at	290912	RGD13100	similar to hypothetical protein FLJ1015	-0.812581	0.015949	0.055987
1368069_at	81859	Sharpin	SHANK-associated RH domain interac	0.6662	0.015958	0.056004
1384729_at	304799	Ppp1r15b_	protein phosphatase 1, regulatory (inhi	4.102908	0.015963	0.056007
1387479_at	156726	Runx3	runt-related transcription factor 3	1.082228	0.015996	0.05611
1378815_at	362203	RGD13069	similar to Gamma-tubulin complex com	2.440344	0.016025	0.056194
1371320_at	290364	Itm2b	integral membrane protein 2B	-0.849833	0.016031	0.056202
1384394_at	79435	Ube2d2	ubiquitin-conjugating enzyme E2D 2	0.678911	0.016046	0.05624
1383685_at	361262	Heatr1_pre	HEAT repeat containing 1 (predicted)	1.182474	0.016069	0.056304
1367678_at	157074	Sdha	succinate dehydrogenase complex, su	0.736125	0.016115	0.05645
1378041_at	362302	Abcb8	ATP-binding cassette, sub-family B (M	1.665959	0.016139	0.056519
1368474_at	25361	Vcam1	vascular cell adhesion molecule 1	-2.088672	0.016198	0.056711
1382219_at	310348	RGD13075	similar to RIKEN cDNA 1700108L22	0.668604	0.016212	0.056745
1374766_at	314997	RGD15647	similar to mKIAA0493 protein (predicte	0.652055	0.016219	0.056757
1374375_at	501196	RGD15609	similar to 2610034M16Rik protein (pre	-1.762631	0.016252	0.056857
1372151_at	307474	Tcerg1_pre	transcription elongation regulator 1 (C/	-0.684077	0.016265	0.056887
1382913_at	282587	Cttnbp2	cortactin binding protein 2	-1.364717	0.016275	0.056906
1393604_at	360810	Atpbd1c	ATP binding domain 1 family, member	0.975374	0.016289	0.056929
1384832_at	293645	Ppfia1_pre	protein tyrosine phosphatase, receptor	1.001357	0.01629	0.056929
1367992_at	25719	Sgne1	secretory granule neuroendocrine prot	-0.679174	0.016297	0.056939
1369990_at	360243	Top2a	topoisomerase (DNA) 2 alpha	0.910374	0.016311	0.056972

1373326_at	295645	Slc4a10	solute carrier family 4, sodium bicarbo	1.282859	0.01632	0.056987
1398849_at	117056	H3f3b	H3 histone, family 3B	-0.843436	0.01633	0.056987
1368291_at	60371	Birc2	baculoviral IAP repeat-containing 2	-1.215288	0.016331	0.056987
1373891_at	313436	Zcchc12	zinc finger, CCHC domain containing 1	-1.153681	0.016332	0.056987
1367825_at	29622	Ralgds	ral guanine nucleotide dissociation stin	-0.634231	0.016338	0.056993
1369236_at	170820	Prdm4	PR domain containing 4	1.200294	0.016346	0.057003
1374078_at	362166	RGD15606	similar to PHF21A protein (predicted)	0.76466	0.01636	0.057038
1385756_at	306251	Itih1_predi	inter-alpha trypsin inhibitor, heavy chai	-1.605891	0.016372	0.057052
1368601_at	24898	Slc6a3	solute carrier family 6 (neurotransmitte	1.626528	0.016373	0.057052
1379595_at	296761	Tmem60_p	transmembrane protein 60 (predicted)	0.733474	0.016384	0.057076
1379793_at	361888	RGD13053	similar to RIKEN cDNA 3110031B13	0.822688	0.016393	0.057089
1375857_at	309499	RGD15642	similar to Myoferlin (Fer-1 like protein 3	-1.256162	0.016396	0.057089
1372814_at	360868	RGD13106	similar to RIKEN cDNA 2010005O13	0.769676	0.016415	0.057139
1369969_at	25591	Parp1	poly (ADP-ribose) polymerase family, r	0.77616	0.016429	0.057172
1387065_at	140693	Plcd4	phospholipase C, delta 4	4.166822	0.016448	0.057212
1368965_at	80878	Slc16a3	solute carrier family 16 (monocarboxyl	2.61682	0.016449	0.057212
1399072_at	282837	Fibp	fibroblast growth factor (acidic) intrace	0.951695	0.016497	0.057363
1375546_at	314642	Fzr1_predi	fizzy/cell division cycle 20 related 1 (Dr	0.890657	0.016514	0.057407
1372488_at	305177	RGD1309C	similar to RIKEN cDNA 2310057D15	0.911777	0.016519	0.057411
1374557_at	362065	LOC36206	CG6210-like	0.845761	0.016526	0.057415
1367593_at	25545	Sepw1	selenoprotein W, muscle 1	-2.754501	0.016529	0.057415
1374313_at	290640	Bpy2ip1_pr	BPY2 interacting protein 1 (predicted)	0.995508	0.016542	0.057441
1391706_at	501168	NA	NA	0.867705	0.016545	0.057441
1369015_at	24598	Nos1	nitric oxide synthase 1, neuronal	3.053075	0.016558	0.05747
1384925_at	290279	RGD13067	similar to RIKEN cDNA 2510005D08 (p	-0.841189	0.016564	0.057477
1388219_at	79247	Htr5b	5-hydroxytryptamine (serotonin) recep	1.424068	0.016598	0.05757
1393408_at	500137	RGD15627	similar to NEX-1 (predicted)	2.977368	0.0166	0.05757
1369770_at	25033	Sstr1	somatostatin receptor 1	-1.284739	0.016617	0.057615
1383128_at	360906	RGD13085	similar to RIKEN cDNA 2900024C23 (p	-1.119308	0.016641	0.057676
1367471_at	299610	Polr2e_pre	polymerase (RNA) II (DNA directed) po	0.636031	0.016643	0.057676
1378726_at	288587	Alkbh4_pre	alkB, alkylation repair homolog 4 (E. co	1.39601	0.016661	0.057724
1370314_at	81826	Slc20a1	solute carrier family 20 (phosphate tran	-0.979931	0.016669	0.057736
1370366_at	64464	Timm10	translocase of inner mitochondrial mer	0.798122	0.016688	0.057785
1370006_at	29478	Ndufs6	NADH dehydrogenase (ubiquinone) Fe	-0.753119	0.016735	0.057935
1372989_at	499014	LOC49901	similar to zinc finger, DHHC domain co	-0.900741	0.016747	0.05796
1388752_at	293017	Bclaf1	BCL2-associated transcription factor 1	-0.652287	0.016754	0.05797
1376848_at	310178	Myo10_pre	myosin X (predicted)	0.96257	0.016764	0.057989
1382863_at	311287	Tmem16c_	transmembrane protein 16C (predicted	1.285124	0.016775	0.058011
1390524_at	317241	Rnf12	ring finger protein 12	0.705614	0.016808	0.058109
1370374_at	170824	Steap3	STEAP family member 3	1.031632	0.016812	0.058109
1389345_at	311264	RGD13107	similar to G2 (predicted)	-0.782461	0.016826	0.058142
1371304_a_at	304447	RGD15598	similar to myosin, light polypeptide 6, a	0.642852	0.016834	0.058156
1398691_at	499624	LOC49962	NA	-1.399549	0.016853	0.058204
1373169_at	306582	Agpat5_pre	1-acylglycerol-3-phosphate O-acyltran	0.686274	0.016857	0.058204
1398899_at	361365	Polr2c	polymerase (RNA) II (DNA directed) po	0.744995	0.016863	0.058208
1372697_at	298517	Mrps15	mitochondrial ribosomal protein S15	0.806466	0.016874	0.058224
1374189_at	305848	Zfp219	zinc finger protein 219	0.752312	0.016876	0.058224
1374886_at	301514	Bcs1l	BCS1-like (yeast)	0.952586	0.016899	0.058277
1387444_at	171125	Ptprh	protein tyrosine phosphatase, receptor	1.610223	0.0169	0.058277
1373429_at	406169	Crebl1	cAMP responsive element binding prot	1.966591	0.016928	0.058322
1369581_at	25511	Pemt	phosphatidylethanolamine N-methyltra	4.105388	0.016928	0.058322

1395268_at	297930	Wwp1	WW domain containing E3 ubiquitin pr	1.962299	0.01693	0.058322
1376682_at	362261	LOC36226	NA	1.450712	0.016931	0.058322
1385083_at	500434	RGD1563C	similar to hypothetical protein (predicte	-3.289642	0.016972	0.058447
1383570_at	363210	Phf3_predi	PHD finger protein 3 (predicted)	-0.779467	0.016983	0.058472
1376431_at	291673	Sil1	endoplasmic reticulum chaperone SIL1	0.719042	0.016995	0.058497
1367679_at	25599	Cd74	CD74 antigen (invariant polypeptide of	-1.493113	0.017008	0.058526
1392599_at	302678	Syap1	synapse associated protein 1	-0.774322	0.017019	0.058549
1370295_at	191575	Nme1	expressed in non-metastatic cells 1	0.765982	0.01703	0.058568
1376648_at	298894	Mycn_map	v-myc myelocytomatosis viral related c	-3.369204	0.017033	0.058568
1392446_at	290692	Klhl2_pred	kelch-like 2, Mayven (Drosophila) (pre	-1.523198	0.017058	0.058638
1393232_at	498911	RGD15655	RGD1565500 (predicted)	1.690447	0.017133	0.058882
1398529_at	406160	Btnl8	butyrophilin-like 8	1.083235	0.017144	0.058903
1373240_at	313689	Dhrs3	dehydrogenase/reductase (SDR family	3.812834	0.017157	0.058932
1397410_at	312192	RGD15648	similar to RIKEN cDNA 4631427C17 (p	1.133279	0.017173	0.05897
1371849_at	290558	Nt5dc2	5'-nucleotidase domain containing 2	-0.798612	0.017209	0.05908
1372262_at	500344	NA	NA	-0.692734	0.017217	0.059088
1373685_at	361149	RGD15657	similar to low density lipoprotein recep	-0.63612	0.017222	0.059088
1398022_at	313928	RGD13108	similar to KIAA0953 protein (predicted)	-1.78197	0.017225	0.059088
1390109_at	363113	Syncrip	synaptotagmin binding, cytoplasmic RI	-0.683967	0.017236	0.059111
1369965_at	81685	Mlh1	mutL homolog 1 (E. coli)	-1.092369	0.017254	0.059157
1391727_at	501538	NA	NA	-2.490173	0.017279	0.059228
1374790_at	287134	Gnptg	N-acetylglucosamine-1-phosphotransfe	0.721079	0.017299	0.059278
1373820_at	360860	RGD13047	similar to constitutive photomorphogen	0.794709	0.017302	0.059278
1388328_at	499619	RGD15604	similar to Eukaryotic translation initiatio	0.763987	0.017307	0.059278
1390736_at	309002	Zfp668_pre	zinc finger protein 668 (predicted)	1.379675	0.017319	0.059306
1388963_at	304900	Astn1	astrotactin 1	0.844962	0.017351	0.059399
1374364_at	498703	RGD15623	similar to mKIAA1931 protein (predicte	0.734786	0.017358	0.059406
1382865_at	500069	Tsga14	testis specific, 14	0.952093	0.017376	0.059427
1398501_at	500111	RGD15618	similar to CDNA sequence BC027309	1.627221	0.017378	0.059427
1390995_at	406168	Fkbpl	FK506 binding protein-like	1.166545	0.01738	0.059427
1371985_a_at	361796	Bat5	HLA-B associated transcript 5	0.953999	0.017382	0.059427
1388393_at	302562	Plp2_mapp	proteolipid protein 2 (mapped)	0.86507	0.0174	0.059472
1388450_at	84479	Ap1gbp1	AP1 gamma subunit binding protein 1	1.030841	0.017422	0.059532
1391457_a_at	306657	Irx2	Iroquois related homeobox 2 (Drosoph	-5.871733	0.017429	0.059543
1376874_a_at	310746	Ap4b1_pre	adaptor-related protein complex AP-4,	0.729188	0.017439	0.05956
1377756_at	362394	RGD13076	similar to RIKEN cDNA 5830446M03	0.747927	0.017449	0.059565
1399123_at	361057	Ddx26	DEAD/H (Asp-Glu-Ala-Asp/His) box po	-0.87439	0.017451	0.059565
1388438_at	361684	Nap1l4	nucleosome assembly protein 1-like 4	0.86818	0.017454	0.059565
1368248_at	81925	Cds1	CDP-diacylglycerol synthase 1	1.44936	0.017467	0.059583
1383078_at	360546	Efnb3_prec	ephrin B3 (predicted)	-3.241917	0.017471	0.059583
1371688_at	312903	Tram1	translocation associated membrane pr	0.787766	0.017474	0.059583
1368040_at	64531	Synj2bp	synaptojanin 2 binding protein	0.718099	0.017477	0.059583
1381148_at	498235	NA	NA	3.296316	0.0175	0.059645
1377903_at	313934	LOC31393	NA	-0.916671	0.01751	0.059664
1370216_at	25678	Ddr1	discoidin domain receptor family, mem	1.865391	0.017527	0.059693
1372473_at	292994	Tjp1_predi	tight junction protein 1 (predicted)	-0.915363	0.017527	0.059693
1370162_at	140943	Ppp4r1	protein phosphatase 4, regulatory subu	0.71083	0.017533	0.059697
1379406_at	290235	RGD13083	similar to chromosome 14 open readin	0.880935	0.017545	0.059698
1370632_at	286933	Obp2b	odorant binding protein 2B	1.286354	0.017546	0.059698
1374764_at	289724	RGD13056	similar to 2610033H07Rik protein (pre	0.956993	0.017547	0.059698
1398800_at	56011	Ywhab	tyrosine 3-monooxygenase/tryptophan	-0.698292	0.017597	0.059855

1387720_at	171394	Clstn2	calsyntenin 2	2.299473	0.017603	0.059858
1368120_at	81733	Nell1	NEL-like 1 (chicken)	-1.41701	0.017608	0.059859
1376670_at	363026	Prmt4	protein arginine N-methyltransferase 4	1.351224	0.017704	0.060172
1374152_at	309628	Wdr46	WD repeat domain 46	0.816812	0.017711	0.06018
1392017_at	311030	Stam2	signal transducing adaptor molecule (S	1.095802	0.017724	0.06021
1370113_at	78971	Birc3	baculoviral IAP repeat-containing 3	-1.183935	0.017752	0.060288
1371960_at	313053	Ythdf2_pre	YTH domain family 2 (predicted)	1.02878	0.017779	0.060363
1390383_at	298199	Adfp	adipose differentiation related protein	1.271557	0.017788	0.060378
1378022_at	313216	Rnf20_pre	ring finger protein 20 (predicted)	-0.830803	0.017802	0.060411
1371424_at	308976	RGD13048	similar to RIKEN cDNA 3110005O21	1.141095	0.017817	0.060439
1387134_at	114247	Slfn3	schlafen 3	-2.989302	0.017819	0.060439
1388681_at	287276	Sar1b	SAR1 gene homolog B (S. cerevisiae)	-0.621377	0.01783	0.060459
1397037_at	361651	RGD15641	similar to hypothetical protein (predicte	1.06165	0.017846	0.060489
1380894_at	308350	RGD15663	similar to PIRB1 (predicted)	-2.848029	0.01785	0.060489
1389744_at	499898	NA	NA	-1.051166	0.017852	0.060489
1372956_at	415064	Bat4	Bat4 gene	1.01132	0.017866	0.060517
1393899_at	192357	Gmpr2	guanosine monophosphate reductase	1.495684	0.01787	0.060517
1368369_at	25516	Pnoc	prepronociceptin	0.879046	0.017888	0.060537
1372194_at	360540	Akap10_pr	A kinase (PRKA) anchor protein 10 (pr	1.152279	0.017892	0.060537
1372448_at	362156	LOC36215	NA	0.853536	0.017897	0.060537
1369195_at	25598	Fabp2	fatty acid binding protein 2, intestinal	-2.382996	0.017897	0.060537
1370916_at	84492	Tec	tec protein tyrosine kinase	1.34259	0.017899	0.060537
1379491_at	288271	Mrap_pred	melanocortin 2 receptor accessory pro	2.003977	0.017905	0.060543
1389688_at	288016	Leprel1	leprecan-like 1	0.889563	0.017916	0.060564
1395523_at	302855	LOC30285	similar to heterogeneous nuclear ribon	-1.085249	0.017951	0.060666
1384540_at	308495	Lrfn3_pred	leucine rich repeat and fibronectin type	1.155439	0.017961	0.060685
1382232_at	307759	Gpt2_predi	glutamic pyruvate transaminase (alanin	1.156902	0.017988	0.060761
1373357_at	313504	Foxd2_pre	forkhead box D2 (predicted)	0.653694	0.018001	0.060789
1395652_at	311202	Gyltl1b	glycosyltransferase-like 1B	0.919722	0.01801	0.060805
1373029_at	261736	Cops2	COP9 (constitutive photomorphogenic	0.625499	0.018016	0.060809
1372321_at	304663	Lyl1	lymphoblastic leukemia derived seque	-1.356049	0.018021	0.06081
1371025_at	64459	Masp2	mannan-binding lectin serine peptidase	2.518943	0.01806	0.060927
1372897_at	300901	Plod2	procollagen lysine, 2-oxoglutarate 5-di	-1.225051	0.018085	0.060995
1384084_at	361719	RGD13081	LOC361719 (predicted)	1.012848	0.018113	0.061074
1393335_at	317470	Egfl6	EGF-like-domain, multiple 6	-1.902853	0.018121	0.061088
1383122_at	373545	Prkag2	protein kinase, AMP-activated, gamma	0.667899	0.018148	0.061161
1389260_at	363332	LOC36333	NA	0.80198	0.018153	0.061162
1376889_at	619550	Gpr153	G protein-coupled receptor 153	-1.880788	0.018168	0.061197
1381445_at	360896	Esrrg	estrogen-related receptor gamma	1.30226	0.018215	0.061342
1398300_at	25390	Atp1b3	ATPase, Na+/K+ transporting, beta 3 p	0.724535	0.01822	0.061342
1369841_at	60460	Hspa2	heat shock 70kDa protein 2	-1.799224	0.018233	0.061369
1370313_at	26759	Acot7	acyl-CoA thioesterase 7	-0.937216	0.018244	0.061382
1387819_at	24331	Ela1	elastase 1, pancreatic	-2.627304	0.018246	0.061382
1376770_at	501181	RGD15595	similar to EF hand domain containing 1	-2.055411	0.018253	0.06139
1369496_at	117255	Ptpn12	protein tyrosine phosphatase, non-rece	1.463873	0.018263	0.061406
1369040_at	114116	Cdc42bpa	CDC42 binding protein kinase alpha	0.928376	0.018267	0.061406
1381847_at	500585	RGD15614	similar to BC021442 protein (predicted	-0.925583	0.018284	0.061438
1372294_at	554172	Perc64	PE responsive protein c64	-0.714179	0.018286	0.061438
1383829_at	303970	Bbx_predic	bobby sox homolog (Drosophila) (pred	0.593988	0.018299	0.061468
1385710_at	287034	Tnfrsf17_p	tumor necrosis factor receptor superfa	1.390732	0.018312	0.061495
1385767_at	304000	LOC30400	cell adhesion molecule JCAM	0.839878	0.01832	0.061505

1392939_at	641603	Slc41a3	NA	-1.252667	0.018326	0.061505
1393359_at	308777	Ap3b2_pre	adaptor-related protein complex 3, bet	-0.690741	0.01833	0.061505
1370965_at	25705	Tcf8	transcription factor 8	-0.865178	0.018333	0.061505
1376519_at	315094	Brp16	brain protein 16	0.698659	0.018352	0.06154
1368569_at	116463	Akr1b7	aldo-keto reductase family 1, member	1.488082	0.018353	0.06154
1370390_at	245982	Coro6	coronin, actin binding protein 6	-0.787847	0.018363	0.061549
1372492_at	290485	Cldn10_pre	claudin 10 (predicted)	-2.289322	0.018365	0.061549
1372604_at	503164	LOC50316	hypothetical protein LOC503164	3.362397	0.01841	0.061683
1369971_a_at	79256	Hnrpd	heterogeneous nuclear ribonucleoprot	-0.8164	0.018449	0.061792
1376104_at	316764	RGD13083	similar to KIAA0802 protein (predicted)	1.115601	0.018453	0.061792
1369588_a_at	25392	Atpif1	ATPase inhibitory factor 1	-0.670615	0.018458	0.061792
1393947_at	306574	Slc25a15	solute carrier family 25 (mitochondrial	1.857161	0.018465	0.061792
1369608_at	60464	Fgf16	fibroblast growth factor 16	1.133799	0.018466	0.061792
1388587_at	294235	Ier3	immediate early response 3	1.271055	0.018505	0.061909
1369248_a_at	63879	Birc4	baculoviral IAP repeat-containing 4	1.232713	0.018524	0.061956
1391474_at	317343	RGD15601	similar to cofactor required for Sp1 trar	-0.642331	0.018533	0.061971
1376661_at	313672	LOC31367	similar to CG11206-PA	-1.927466	0.018546	0.061999
1390107_at	361604	Sytl2_pred	synaptotagmin-like 2 (predicted)	-2.414532	0.018579	0.062082
1389605_at	305434	MGC12521	multiple coiled-coil GABABR1-binding	1.159483	0.01858	0.062082
1383491_at	361594	Isg20l1_pre	interferon stimulated exonuclease gen	1.342163	0.018596	0.062104
1382322_a_at	308435	Cic_predict	capicua homolog (Drosophila) (predict	1.268568	0.018601	0.062104
1383160_at	315447	Chordc1_p	cysteine and histidine-rich domain (CH	-1.179299	0.018604	0.062104
1377145_at	294614	RGD15621	similar to very large G-protein coupled	-0.702152	0.018606	0.062104
1368362_a_at	29403	Asgr2	asialoglycoprotein receptor 2	-2.262214	0.018629	0.062164
1380499_at	304061	Brwd1_pre	bromodomain and WD repeat domain	-0.819548	0.018661	0.062256
1385132_at	297783	Mybl1_pre	myeloblastosis oncogene-like 1 (predic	-2.353044	0.018669	0.062267
1372538_at	300083	Slc25a17_	solute carrier family 25 (mitochondrial	-0.587951	0.018678	0.062281
1370002_at	60323	Arhgef1	Rho guanine nucleotide exchange fact	0.626086	0.018684	0.062282
1372401_at	298071	Nans_pred	N-acetylneuraminic acid synthase (sial	0.687635	0.018688	0.062282
1384551_at	309326	Ranbp6_pr	RAN binding protein 6 (predicted)	-1.428998	0.018692	0.062283
1374775_at	291234	Mki67_pre	antigen identified by monoclonal antibo	-1.98072	0.018709	0.062305
1387798_a_at	54243	Crry	complement receptor related protein	-0.627847	0.018713	0.062305
1367661_at	85247	S100a6	S100 calcium binding protein A6 (calcy	1.165824	0.018721	0.062305
1398364_at	362626	RGD13595	similar to chromosome 1 open reading	-0.953519	0.018722	0.062305
1392930_at	294948	Armc1_pre	armadillo repeat containing 1 (predicte	-0.850257	0.018723	0.062305
1388532_at	361301	RGD13105	similar to hypothetical protein	0.794415	0.018734	0.062328
1376388_at	286937	Pex2	peroxin 2	1.995474	0.018764	0.0624
1388190_at	54225	Apob	apolipoprotein B	-3.598151	0.018769	0.0624
1370228_at	24825	Tf	transferrin	-2.812589	0.018774	0.0624
1375452_at	299078	Mgea6_pre	meningioma expressed antigen 6 (coil	-0.799109	0.018781	0.0624
1370026_at	25420	Cryab	crystallin, alpha B	-1.802628	0.018783	0.0624
1377832_at	310344	Plk4_predi	polo-like kinase 4 (Drosophila) (predict	-0.867356	0.018784	0.0624
1393635_x_at	29213	Tubb4	tubulin, beta 4	-1.033184	0.018796	0.062424
1389622_at	362322	RGD15658	similar to citrin (predicted)	0.9942	0.018829	0.062503
1390700_s_at	362981	Hdac10	histone deacetylase 10	0.93343	0.018829	0.062503
1377727_at	314126	Baz1a_pre	bromodomain adjacent to zinc finger d	1.085115	0.018842	0.06251
1398352_at	362827	Pias4	protein inhibitor of activated STAT, 4	1.704218	0.018844	0.06251
1392958_at	498035	NA	NA	-0.613009	0.018846	0.06251
1388631_at	316051	Azi2	5-azacytidine induced gene 2	-1.065539	0.018852	0.062511
1372007_at	500569	RGD15629	similar to AOF2 protein (predicted)	-1.144701	0.018855	0.062511
1388199_at	171577	Tacstd1	tumor-associated calcium signal transc	-0.792929	0.018869	0.062528

1378013_at	361199	RGD15643	RGD1564391 (predicted)	3.122125	0.01887	0.062528
1384423_at	294016	Nt5c2_pred	5'-nucleotidase, cytosolic II (predicted)	1.323786	0.018885	0.062554
1379463_at	246776	Filip1	filamin A interacting protein 1	1.102999	0.018887	0.062554
1381970_at	361554	Lsm14a_pr	LSM14 homolog A (SCD6, S. cerevisia	-0.597923	0.018905	0.062597
1378266_at	292686	Fbxo46	F-box protein 46	1.250344	0.018957	0.062754
1390418_at	316333	Actr1b	ARP1 actin-related protein 1 homolog	1.086335	0.01901	0.062902
1367719_at	117060	Dd5	progestin induced protein	-0.720002	0.019011	0.062902
1395046_at	306570	Ank1	ankyrin 1, erythroid	0.969009	0.019059	0.063043
1384386_at	25597	Rasa2	RAS p21 protein activator 2	0.837185	0.019123	0.063239
1387099_at	116564	Npr2	natriuretic peptide receptor 2	-1.454021	0.019129	0.063244
1371645_at	287470	Sdf2_predi	stromal cell derived factor 2 (predicted	0.909659	0.019134	0.063244
1375438_at	64154	Gosr2	golgi SNAP receptor complex member	0.716506	0.019169	0.063346
1390131_at	303306	Srr	serine racemase	0.610375	0.019176	0.063352
1398924_at	499934	MGC10914	similar to mannosidase, beta A, lysoso	0.804698	0.019187	0.063373
1390200_at	362528	RGD13048	similar to mKIAA0674 protein (predicte	0.985772	0.019196	0.063373
1377367_at	293997	Kazald1	Kazal-type serine peptidase inhibitor d	-2.065855	0.019197	0.063373
1398708_at	499614	NA	NA	0.810369	0.019215	0.063408
1374028_at	500974	LOC50097	similar to CDNA sequence BC024479	-1.290645	0.019217	0.063408
1388313_at	122799	Rps25	ribosomal protein s25	-0.83441	0.019236	0.063454
1388570_at	498909	LOC49890	NA	0.657025	0.019254	0.063491
1387035_a_at	63994	Arhgap17	Rho GTPase activating protein 17	-0.86002	0.019256	0.063491
1398896_at	300674	Arcn1	archain 1	0.628376	0.019274	0.063533
1381139_at	498963	RGD15608	similar to RIKEN cDNA 2610039E05 (p	-1.919922	0.019288	0.063564
1376156_at	316091	RGD13048	hypothetical LOC316091	1.28507	0.019295	0.063572
1382993_at	317673	Bbc3	Bcl-2 binding component 3	-0.818458	0.019324	0.06365
1387375_at	25659	Khk	ketohexokinase	1.018187	0.01935	0.063721
1376937_at	304020	RGD15659	similar to 4631422O05Rik protein (pre	-4.390256	0.019404	0.063872
1370060_at	64201	Slc25a11	solute carrier family 25 (mitochondrial	0.889154	0.019407	0.063872
1373835_at	306436	Fbxo8	F-box only protein 8	-0.68343	0.019415	0.063872
1382320_at	362793	RGD13073	LOC362793	-1.244306	0.019419	0.063872
1389965_at	192152	Tgoln2	trans-golgi network protein 2	-0.661289	0.01942	0.063872
1380797_at	361268	Asb13_pre	ankyrin repeat and SOCS box-containi	1.123124	0.019429	0.063885
1397588_at	360483	RGD15617	RGD1561796 (predicted)	0.756583	0.019434	0.063886
1373845_at	246145	Pbp2	phosphatidylethanlomine binding prote	0.689619	0.019456	0.063943
1368359_a_at	29461	Vgf	VGF nerve growth factor inducible	1.244073	0.019464	0.063954
1376974_at	362696	Ttc7	tetratricopeptide repeat domain 7	1.41106	0.019502	0.064061
1369578_at	78983	Tas2r7	taste receptor, type 2, member 7	-1.353132	0.019509	0.06407
1395042_at	305556	Ehbp1_pre	EH domain binding protein 1 (predicte	1.001745	0.019516	0.064075
1376091_at	315150	Adsl_predi	adenylosuccinate lyase (predicted)	0.686841	0.019526	0.064094
1376641_at	291797	Thoc1	THO complex 1	-0.65817	0.019532	0.064096
1374344_at	314614	Prg-2	plasticity-related protein PRG-2	-1.218447	0.019537	0.064096
1387800_at	140926	Daxx	Fas death domain-associated protein	0.976033	0.019563	0.064166
1380969_at	309510	RGD15646	similar to mKIAA0940 protein (predicte	0.930583	0.019568	0.064167
1391528_at	307236	RGD13082	similar to mKIAA0863 protein (predicte	-0.790678	0.019577	0.06418
1389708_at	362191	RGD13100	similar to RIKEN cDNA 5730421E18	-1.155674	0.019585	0.064189
1388503_at	499882	RGD15627	similar to CREBBP/EP300 inhibitory pr	1.033227	0.019609	0.064239
1397797_at	309174	Tigd3_pred	tigger transposable element derived 3	-1.329958	0.019609	0.064239
1369543_s_at	192210	Gs3	putative regulation protein GS3	-0.719637	0.019625	0.064267
1373600_at	300173	LOC30017	similar to CG9996-PA	-0.725396	0.019628	0.064267
1376327_at	366518	Tnfrsf14	tumor necrosis factor receptor superfa	3.328745	0.019645	0.064306
1393452_at	313495	Car9_predi	carbonic anhydrase 9 (predicted)	2.250498	0.019649	0.064306

1398892_at	286898	Npc2	Niemann Pick type C2	-0.647443	0.019733	0.064563
1389276_at	309550	L3mbtl3_pr	l(3)mbt-like 3 (Drosophila) (predicted)	-1.272029	0.019739	0.064568
1398337_at	290341	LOC29034	NA	-0.80381	0.019747	0.064578
1385398_at	295248	Zbtb7b_pre	zinc finger and BTB domain containing	1.804005	0.019783	0.06468
1384771_at	287564	RGD15655	similar to hypothetical protein (predicte	1.764467	0.019794	0.064683
1385519_at	362489	Cbfa2t1_pr	CBFA2T1 identified gene homolog (hu	3.058226	0.019794	0.064683
1369447_at	140944	Slc28a3	solute carrier family 28 (sodium-couple	-1.798825	0.019832	0.064791
1367818_at	29309	Coq3	coenzyme Q3 homolog, methyltransfe	1.392239	0.019894	0.064977
1394991_at	363520	Irak1_pred	interleukin-1 receptor-associated kinas	1.461405	0.0199	0.064981
1368062_at	171126	Ap3m1	adaptor-related protein complex 3, mu	0.600649	0.019917	0.065021
1373618_at	362453	Crebl2	cAMP responsive element binding prot	0.997776	0.019923	0.065021
1388539_at	287925	Pkp2	plakophilin 2	1.496257	0.019927	0.065021
1381933_at	304528	Rfc5_predi	replication factor C (activator 1) 5 (pre	1.058554	0.019937	0.065038
1390688_at	361848	Ddx50	DEAD (Asp-Glu-Ala-Asp) box polypept	-0.642472	0.019992	0.065201
1374145_at	292898	RGD13098	similar to RIKEN cDNA 1110061L23	1.603207	0.020011	0.065247
1379885_at	246247	Fmo4	flavin containing monooxygenase 4	0.766425	0.020029	0.06529
1375775_at	500917	RGD15643	similar to shippo 1 (predicted)	-2.645732	0.020035	0.065294
1387009_at	29153	Capn1	calpain 1	1.80828	0.02005	0.065323
1385080_s_at	360619	LOC36061	NA	1.268854	0.020054	0.065323
1381901_at	499586	RGD15631	RGD1563109 (predicted)	1.374736	0.020071	0.06536
1398759_at	25564	Tgfb1i4	transforming growth factor beta 1 indu	-0.599482	0.020079	0.06536
1374103_at	65153	Freq	frequenin homolog (Drosophila)	-2.87499	0.020081	0.06536
1388976_at	297388	RGD13059	similar to BolA domain-containing prot	0.74123	0.020085	0.06536
1392719_at	300886	Mthfs	5,10-methenyltetrahydrofolate syntheta	-0.721486	0.020129	0.065487
1372091_at	404280	Mig12	MID1 interacting G12-like protein	-0.804333	0.020145	0.065522
1380621_at	361597	RGD15643	similar to tyrosine kinase Fps/Fes (pre	2.049009	0.020159	0.065552
1369162_at	25711	Gucy2c	guanylate cyclase 2C	1.24028	0.020175	0.065589
1391201_at	305827	Wdhd1_pre	WD repeat and HMG-box DNA binding	-1.46764	0.020182	0.065594
1387011_at	170496	Lcn2	lipocalin 2	3.475759	0.020199	0.065636
1398122_at	25681	Col10a1	procollagen, type X, alpha 1	2.032067	0.020208	0.065649
1374042_at	299521	RGD13104	similar to hypothetical protein FLJ2345	-0.737224	0.020239	0.065726
1375851_at	289457	Klhl8_pred	kelch-like 8 (Drosophila) (predicted)	-1.210923	0.020242	0.065726
1386581_at	296456	Itgav_predi	integrin alpha V (predicted)	1.723651	0.020296	0.065885
1388964_at	361946	RGD13078	similar to transcription factor (p38 inter	-0.884736	0.020306	0.065901
1369862_at	24649	Pim1	proviral integration site 1	1.054112	0.02032	0.06592
1374302_at	192223	Opn4	opsin 4 (melanopsin)	1.846499	0.020322	0.06592
1388480_at	288707	Gltp_predi	glycolipid transfer protein (predicted)	0.876994	0.020353	0.066005
1372056_at	316035	Cmtm6	CKLF-like MARVEL transmembrane d	-0.611848	0.020367	0.066033
1370303_at	257647	Slc35a4	solute carrier family 35, member A4	1.160253	0.02038	0.06606
1399024_at	293684	Scyl1	SCY1-like 1 (S. cerevisiae)	0.839532	0.020397	0.0661
1368187_at	113955	Gpnmb	glycoprotein (transmembrane) nmb	1.082073	0.020453	0.066264
1398893_at	291609	Ndfip1	Nedd4 family interacting protein 1	-0.573376	0.020514	0.066445
1387115_at	140934	Ikbkap	inhibitor of kappa light polypeptide enh	0.575464	0.020527	0.066473
1378252_at	288289	Chodl_pred	chondrolectin (predicted)	-1.033285	0.020545	0.066514
1393336_at	293410	Swap70_pr	SWAP complex protein (predicted)	-0.761465	0.020554	0.066526
1391494_at	364033	Vamp4_pre	vesicle-associated membrane protein 4	1.052513	0.020566	0.06655
1376113_at	311631	Zswim1_pr	zinc finger, SWIM domain containing 1	0.699993	0.0206	0.066644
1392785_at	364458	Kctd12_pre	potassium channel tetramerisation dor	-1.394279	0.02061	0.066651
1396949_at	298139	LOC29813	similar to RIKEN cDNA 2310003M01	2.016614	0.020615	0.066651
1382235_at	365699	RGD13068	similar to hypothetical protein FLJ3059	-0.689497	0.020618	0.066651
1373119_at	305055	Kctd3	potassium channel tetramerisation dor	-0.801558	0.020668	0.066796

1384392_at	312495	Cyp26b1	cytochrome P450, family 26, subfamily	-2.460119	0.020673	0.066798
1389614_at	499288	RGD15629	similar to ovary-specific MOB-like prote	0.749756	0.020695	0.066838
1393496_at	299599	Theg	testicular haploid expressed gene	2.420644	0.020697	0.066838
1380063_at	309527	Ch25h	cholesterol 25-hydroxylase	-1.162959	0.020703	0.066838
1391186_at	498250	NA	NA	-4.455441	0.020706	0.066838
1387951_at	64036	Daf1	decay accelarating factor 1	-0.719994	0.020716	0.066854
1395235_at	306342	Txnl6_prec	thioredoxin-like 6 (predicted)	3.405659	0.020731	0.066872
1398803_at	29489	Dync1h1	dynein cytoplasmic 1 heavy chain 1	0.619354	0.020731	0.066872
1391693_at	291555	Atp8b1_pre	ATPase, Class I, type 8B, member 1 (p	1.690631	0.020751	0.066919
1386970_at	117019	Eif2b4	eukaryotic translation initiation factor 2	0.579778	0.020772	0.06697
1368938_at	84010	Dll1	delta-like 1 (Drosophila)	-2.954348	0.020793	0.067022
1370086_at	24367	Fgg	fibrinogen, gamma polypeptide	1.781568	0.020802	0.067035
1389535_at	295238	Dap3	death associated protein 3	-0.567799	0.020821	0.067079
1387490_at	64679	Tgm4	transglutaminase 4 (prostate)	3.305848	0.020842	0.06713
1371712_at	292030	NA	NA	0.79814	0.020848	0.06713
1371891_at	361038	RGD13084	similar to 1700123O20Rik protein (pre	-0.687756	0.020856	0.06713
1370078_at	60377	Lin7b	lin-7 homolog b (C. elegans)	-2.195566	0.020857	0.06713
1393383_at	360500	Arhgdig_pr	Rho GDP dissociation inhibitor (GDI) g	1.59496	0.020887	0.067202
1392811_at	316599	Usp40	ubiquitin specific protease 40	2.577843	0.02089	0.067202
1389180_at	361377	LOC36137	NA	-1.173668	0.020898	0.067212
1389002_at	313494	Tln1	talin 1	0.79557	0.020914	0.067239
1389204_at	361269	RGD15644	similar to chromosome 10 open readin	-0.649547	0.020916	0.067239
1368564_at	84487	Slc17a6	solute carrier family 17 (sodium-depen	1.279021	0.020926	0.067254
1390628_at	360949	Cpeb2_pre	cytoplasmic polyadenylation element b	-0.768159	0.020932	0.067256
1384995_at	315073	Jrk_predict	jerky homolog (mouse) (predicted)	1.16697	0.020955	0.067314
1393745_at	313138	Orc3l	origin recognition complex, subunit 3-li	0.850386	0.020967	0.067319
1392739_a_at	315500	RGD13074	similar to RIKEN cDNA 2310005P05	-2.567698	0.02097	0.067319
1389175_at	305455	Whsc2	Wolf-Hirschhorn syndrome candidate 2	0.914192	0.020972	0.067319
1387775_at	83828	Gtf2a2	general transcription factor IIa 2	-0.607056	0.021007	0.067415
1380104_at	24946	F9	coagulation factor IX	1.757084	0.021015	0.067424
1388194_at	81654	Dlat	dihydrolipoamide S-acetyltransferase (	0.831051	0.021031	0.067447
1374906_at	313450	LOC31345	similar to RIKEN cDNA 2810428C21	-0.795922	0.021032	0.067447
1388925_at	306840	Sirt5	sirtuin 5 (silent mating type information	-0.627171	0.021124	0.067715
1369942_at	63836	Actn4	actinin alpha 4	0.881344	0.021129	0.067715
1387703_a_at	115771	Usp2	ubiquitin specific peptidase 2	-1.624068	0.021131	0.067715
1387015_at	81531	Pfn2	profilin 2	0.705791	0.021136	0.067715
1396467_at	361843	Amid_pred	apoptosis-inducing factor (AIF)-like mit	1.207693	0.021152	0.06775
1379827_at	497941	LOC49794	similar to G protein pathway suppresso	0.73136	0.02116	0.06775
1371547_at	297591	NA	NA	-0.678461	0.021163	0.06775
1398166_at	501183	RGD1566C	RGD1566074 (predicted)	-1.945741	0.021167	0.06775
1370202_at	24913	Hrasls3	HRAS like suppressor 3	-2.048961	0.021181	0.067757
1370178_at	116600	Cacnb2	calcium channel, voltage-dependent, b	-0.600662	0.021182	0.067757
1395998_at	362214	Nol5a	nucleolar protein 5A	1.785704	0.021185	0.067757
1379349_a_at	288585	Rabl5	RAB, member of RAS oncogene family	0.63276	0.021218	0.067846
1367945_at	84355	Atox1	ATX1 (antioxidant protein 1) homolog	-0.842826	0.021225	0.067851
1369979_at	155183	Scap2	src family associated phosphoprotein 2	0.844564	0.021232	0.067857
1374331_at	301513	Rqcd1	rcd1 (required for cell differentiation) h	-0.677605	0.021256	0.067904
1384940_at	309478	RGD13053	similar to KIAA0335	-0.88954	0.021256	0.067904
1382950_at	685067	LOC68506	NA	-2.238429	0.021286	0.067983
1373745_at	294236	Gtf2h4	general transcription factor II H, polype	1.254173	0.021303	0.068005
1367541_at	502632	RGD1566C	similar to 2810410A08Rik protein (pre	-0.843532	0.021304	0.068005

1377089_a_at	362048	Tspan5	tetraspanin 5	-1.32962	0.02132	0.068042
1373231_at	363452	Otud5	OTU domain containing 5	0.58733	0.021335	0.068072
1388365_at	291969	Atp6v0d1	ATPase, H+ transporting, V0 subunit D	0.65712	0.021343	0.06808
1376657_at	363058	Igsf4a	immunoglobulin superfamily, member 4	-1.056651	0.021356	0.068108
1369124_at	29595	Htr2a	5-hydroxytryptamine (serotonin) recep	1.10499	0.021386	0.068186
1375522_at	259274	Nmt1	N-myristoyltransferase 1	0.684958	0.021407	0.068222
1389286_at	362776	Glrx5_pred	glutaredoxin 5 homolog (S. cerevisiae)	0.996229	0.021408	0.068222
1396354_at	502145	LOC50214	NA	-1.792178	0.02142	0.068239
1370490_at	25133	Pcdh3	protocadherin 3	1.584732	0.021423	0.068239
1374505_at	502922	NA	NA	0.777218	0.021433	0.068255
1372527_at	308410	Rtn2	reticulon 2 (Z-band associated protein)	1.402973	0.021447	0.068281
1385055_at	306883	RGD15634	similar to D0H6S2654E protein (predic	2.350689	0.021471	0.068335
1368196_at	25680	Clps	colipase, pancreatic	-2.502303	0.021474	0.068335
1376192_at	303669	Nat9_predi	N-acetyltransferase 9 (predicted)	0.938381	0.021505	0.068411
1381305_at	308509	RGD13084	similar to RIKEN cDNA 4931406P16 (p	-0.887514	0.021511	0.068411
1371961_at	361527	Pld3	phospholipase D family, member 3	0.565273	0.021513	0.068411
1373687_at	362963	Rutbc3	RUN and TBC1 domain containing 3	1.51587	0.021542	0.06847
1373395_at	303088	Trim41_pre	tripartite motif protein 41 (predicted)	0.597018	0.021542	0.06847
1394790_at	295586	Clcn6_prec	chloride channel 6 (predicted)	0.980368	0.021566	0.06853
1399124_at	362643	RGD13104	similar to KIAA0090 protein (predicted)	-0.71627	0.021593	0.068598
1398877_at	192277	Stip1	stress-induced phosphoprotein 1	0.620152	0.021616	0.068654
1368058_at	64196	Safb	scaffold attachment factor B	0.794516	0.021631	0.068684
1381979_at	360800	Sumf2	sulfatase modifying factor 2	0.947354	0.021656	0.068749
1367457_at	114558	Becn1	beclin 1 (coiled-coil, myosin-like BCL2-	0.596022	0.021667	0.068755
1375865_at	316233	Tjap1_prec	tight junction associated protein 1 (pre	0.837775	0.021668	0.068755
1389243_at	500483	NA	NA	-1.253541	0.021689	0.068804
1374220_at	361261	RGD15659	similar to 5730405I09Rik protein (pred	1.205535	0.021707	0.068846
1373125_at	309159	Klc2_predi	kinesin light chain 2 (predicted)	0.860198	0.021726	0.068887
1382275_at	361232	MGC12501	similar to PAK/PLC-interacting protein	0.722052	0.021737	0.068906
1387754_at	25007	Tacr2	tachykinin receptor 2	3.557613	0.021744	0.068912
1371464_at	293067	Za20d3	zinc finger, A20 domain containing 3	-0.781418	0.021751	0.068913
1370326_at	81741	Pctk1	PCTAIRE-motif protein kinase 1	0.689892	0.021766	0.068913
1377820_a_at	293526	Ate1_predi	arginine-tRNA-protein transferase 1 (p	-0.797381	0.021767	0.068913
1368427_at	25228	Akap11	A kinase (PRKA) anchor protein 11	-0.641477	0.021769	0.068913
1372815_at	298385	Magoh_pre	mago-nashi homolog, proliferation-ass	-0.607465	0.021773	0.068913
1387414_at	79107	Duox2	dual oxidase 2	1.853682	0.021775	0.068913
1390424_at	301044	NA	NA	1.15123	0.021788	0.068936
1399018_at	360602	RGD13079	similar to cisplatin resistance-associate	-0.919484	0.021805	0.068974
1375486_at	317402	NA	NA	1.021107	0.021812	0.068979
1381562_at	308587	RGD15651	similar to hypothetical protein BC0186	-2.437132	0.021825	0.06898
1372496_at	313786	RGD15612	similar to hypothetical protein MGC196	1.025387	0.021825	0.06898
1387105_at	171110	Zfp422	zinc finger protein 422	-0.945332	0.021828	0.06898
1371548_at	297459	Mrps25	mitochondrial ribosomal protein S25	0.588642	0.021839	0.069
1385411_at	303233	Usp43_pre	ubiquitin specific protease 43 (predicte	0.656402	0.021854	0.069031
1389026_at	293117	Ankrd42_p	ankyrin repeat domain 42 (predicted)	-2.96578	0.021861	0.069035
1369479_at	81820	Doc2b	double C2, beta	3.547317	0.021869	0.069044
1393668_at	288369	Pex11c_pr	peroxisomal biogenesis factor 11c (pre	0.886793	0.021914	0.069163
1373547_at	307008	RGD13081	similar to expressed sequence AW209	0.710864	0.021919	0.069163
1387977_at	85482	Nbn	nibrin	-0.773453	0.021927	0.069163
1369338_at	58946	Robo1	roundabout homolog 1 (Drosophila)	1.743749	0.021931	0.069163
1382277_at	448830	Ly96	lymphocyte antigen 96	0.64664	0.021933	0.069163

1370020_at	170943	Slc25a10	solute carrier family 25 (mitochondrial	0.871939	0.021947	0.06919
1392888_at	317322	Gpc4	glypican 4	-0.697549	0.021971	0.069251
1389473_at	306307	Rgr_predic	retinal G protein coupled receptor (pre	1.038078	0.021977	0.069254
1392186_at	368088	RGD15633	similar to diacylglycerol kinase, delta 1	0.829891	0.021996	0.069271
1374000_at	287773	Helz_predi	helicase with zinc finger domain (predi	0.750784	0.021997	0.069271
1380118_at	315646	RGD15622	similar to hypothetical gene supported	-0.846672	0.022002	0.069271
1387644_at	64022	Btc	betacellulin	2.064183	0.022004	0.069271
1387715_at	171059	Expi	extracellular peptidase inhibitor	-3.240226	0.022031	0.069327
1393872_at	360945	RGD15628	similar to RIKEN cDNA 2310045A20 (p	0.69886	0.022032	0.069327
1391509_at	494343	Tacstd2	tumor-associated calcium signal transc	2.431049	0.022079	0.069459
1373369_at	361103	Rai17_prec	retinoic acid induced 17 (predicted)	-0.959819	0.022116	0.069558
1375627_at	297968	RGD13077	similar to hypothetical protein FLJ1034	0.9363	0.022151	0.069641
1372357_at	362237	Tbc1d20	TBC1 domain family, member 20	1.022446	0.022153	0.069641
1398373_at	310508	B3galt3	UDP-Gal:betaGlcNAc beta 1,3-galacto	-0.818367	0.022158	0.069641
1370457_at	286916	Testin	testin gene	2.97252	0.022171	0.069664
1372190_at	25293	Aqp4	aquaporin 4	-2.237188	0.022178	0.069671
1367723_a_at	58838	Lnk	linker of T-cell receptor pathways	-0.71479	0.022197	0.069714
1373017_at	362404	Suclg2	succinate-Coenzyme A ligase, GDP-fo	-0.593718	0.022223	0.069763
1368840_at	171411	Lr8	LR8 protein	-0.613384	0.022223	0.069763
1375339_at	360515	Enth	enthoprotin	0.882347	0.022235	0.069783
1399034_at	314288	Pcnx	pecanex homolog (Drosophila)	-0.914958	0.02225	0.069802
1378567_at	314640	Tjp3_predi	tight junction protein 3 (predicted)	1.585136	0.022256	0.069802
1371641_at	499323	RGD15628	similar to CCTeta, eta subunit of the ch	0.566931	0.022257	0.069802
1386229_at	362149	RGD13091	similar to CBF1 interacting corepresso	-0.953081	0.022279	0.069837
1378523_at	300696	Ttc12	tetratricopeptide repeat domain 12	0.934142	0.022284	0.069837
1390953_at	304010	Zbtb11_pre	zinc finger and BTB domain containing	2.732063	0.022292	0.069837
1386976_at	83628	Cd82	CD82 antigen	1.218784	0.022292	0.069837
1372898_at	497972	RGD15662	similar to 2700008B19Rik protein (prec	0.673151	0.022294	0.069837
1388470_at	300849	Fbxo9	F-box only protein 9	-0.764515	0.022309	0.069856
1389227_at	308875	Rhog	Ras homolog gene family, member G	0.688164	0.022312	0.069856
1395603_at	292625	RGD15654	RGD1565487 (predicted)	-1.664259	0.022316	0.069856
1373374_at	362051	Lmo4	LIM domain only 4	-1.138252	0.02235	0.069946
1385813_at	291675	RGD13102	similar to RIKEN cDNA 2010001M09	2.946888	0.02238	0.070021
1367794_at	24153	A2m	alpha-2-macroglobulin	-1.372852	0.022392	0.070044
1374899_at	290877	RGD15660	similar to hypothetical protein MGC205	4.020698	0.022398	0.070047
1375749_at	305287	Sec3l1	SEC3-like 1 (S. cerevisiae)	2.236566	0.022406	0.070055
1371549_at	305895	Wdr23	WD repeat domain 23	0.589874	0.022423	0.070083
1395503_at	361641	RGD13079	similar to RIKEN cDNA 3230401I01 (p	3.389513	0.022426	0.070083
1383555_at	360799	RGD15602	similar to RIKEN cDNA 4930579G22 (	0.91769	0.022436	0.0701
1371770_at	309629	Ke2	MHC class II region expressed gene K	0.635932	0.022457	0.070125
1392865_at	25444	Fgf9	fibroblast growth factor 9	1.023023	0.022459	0.070125
1397427_at	113902	Ces3	carboxylesterase 3	-1.820261	0.02246	0.070125
1398811_at	29439	Jtb	jumping translocation breakpoint	0.728959	0.022473	0.070137
1367755_at	81718	Cdo1	cysteine dioxygenase 1, cytosolic	0.892952	0.022475	0.070137
1391472_at	364475	Dzip1	DAZ interacting protein 1	-2.686239	0.022493	0.070177
1374550_at	500526	MGC11277	similar to map kinase interacting kinas	1.620433	0.02255	0.070337
1387288_at	29458	Neurod1	neurogenic differentiation 1	0.795845	0.022565	0.070354
1393932_at	305684	Samd8	sterile alpha motif domain containing 8	1.08792	0.022565	0.070354
1368065_at	83823	Gipc1	GIPC PDZ domain containing family, m	1.722961	0.022597	0.070437
1397734_at	367846	Sp3	Sp3 transcription factor	-1.756615	0.022606	0.070447
1375383_at	291105	RGD15616	similar to E2f3 protein (predicted)	2.662684	0.02262	0.070463

1381833_at	305984	Cdca2	cell division cycle associated 2	-1.639256	0.022622	0.070463
1377938_at	302941	RGD13594	hypothetical LOC302941	-0.797677	0.022637	0.070494
1394494_at	362648	Arhgef19_p	Rho guanine nucleotide exchange fact	1.259533	0.022647	0.070509
1371820_at	308796	Mesdc2	mesoderm development candiate 2	-0.591571	0.022663	0.07054
1383350_at	311637	Slc35c2	solute carrier family 35, member C2	0.995358	0.02267	0.070548
1388136_at	171139	Timm9	translocase of inner mitochondrial mem	0.591772	0.022692	0.070576
1390661_at	314453	Wdr20	WD repeat domain 20	-0.65782	0.022698	0.070576
1375045_at	307855	Wdr59	WD repeat domain 59	0.788143	0.022699	0.070576
1376919_at	301388	RGD15623	similar to expressed sequence AW212	-0.682602	0.022701	0.070576
1373844_at	361613	Ppme1	protein phosphatase methylesterase 1	0.657433	0.022713	0.070597
1389341_at	312652	Plxnd1_pre	plexin D1 (predicted)	2.893613	0.022735	0.070632
1367971_at	85237	Ptp4a2	protein tyrosine phosphatase 4a2	0.765377	0.022735	0.070632
1380185_at	313433	RGD15645	similar to hypothetical protein FLJ2296	-0.854908	0.022757	0.070685
1387485_a_at	117104	Ppp2r2a	protein phosphatase 2 (formerly 2A), re	0.590776	0.022779	0.070736
1367632_at	24957	Glul	glutamate-ammonia ligase (glutamine	-0.675763	0.022787	0.070745
1373658_at	315298	Racgap1_p	Rac GTPase-activating protein 1 (pred	-1.797533	0.022799	0.07075
1371761_at	362041	Rpl34_pred	ribosomal protein L34 (predicted)	-0.629886	0.0228	0.07075
1389811_at	360519	Rasgef1c_	RasGEF domain family, member 1C (p	0.927841	0.022838	0.070853
1387443_at	171454	Btbd14b	BTB (POZ) domain containing 14B	3.617815	0.022872	0.07094
1387884_at	29672	Psma5	proteasome (prosome, macropain) sub	1.059243	0.022893	0.070976
1388642_at	300514	Ei24	etoposide induced 2.4 mRNA	0.884886	0.022894	0.070976
1374397_at	296302	Eif2s2	eukaryotic translation initiation factor 2	0.719688	0.02291	0.071
1392334_at	309621	RT1-Ba	RT1 class II, locus Ba	2.219305	0.02292	0.071
1384972_at	688336	LOC68833	NA	2.816207	0.022921	0.071
1373716_at	361653	Armc5	armadillo repeat containing 5	0.902019	0.022923	0.071
1378034_at	366856	Mterfd3	MTERF domain containing 3	-0.82762	0.022948	0.071061
1390409_at	361774	RGD13061	LOC361774 (predicted)	0.631169	0.022955	0.071064
1387554_at	83627	Galnt5	UDP-N-acetyl-alpha-D-galactosamine:	1.265745	0.022964	0.071066
1393144_at	311021	Nmi	N-myc (and STAT) interactor	0.664916	0.022966	0.071066
1382428_at	311468	Tasp1_pre	taspase, threonine aspartase 1 (predic	-1.628897	0.022973	0.071068
1374987_at	360511	Pank3_pre	pantothenate kinase 3 (predicted)	-0.680309	0.022977	0.071068
1380372_at	311551	Pofut1	protein O-fucosyltransferase 1	0.648875	0.022984	0.071073
1376239_at	362802	Atp6v1c2	ATPase, H+ transporting, V1 subunit C	-1.308596	0.022992	0.07108
1367637_a_at	24669	Ppp1cc	protein phosphatase 1, catalytic subun	-0.678208	0.023024	0.071149
1387846_at	114493	Cacna1f	calcium channel, voltage-dependent, a	3.013954	0.023025	0.071149
1388485_at	306748	Cxcl14	chemokine (C-X-C motif) ligand 14	-2.244274	0.02306	0.071241
1372211_at	246760	Mafk	v-maf musculoaponeurotic fibrosarcom	-0.562997	0.023088	0.071303
1369615_at	114560	Rap2a	RAS related protein 2a?	0.911759	0.023094	0.071303
1381672_at	500564	RGD15634	similar to LDL receptor adaptor protein	2.900227	0.023099	0.071303
1389421_at	304573	Pole_mapp	polymerase (DNA directed), epsilon (m	0.730749	0.023107	0.071303
1375530_at	498486	RGD15631	similar to EMeg32 protein (predicted)	0.942435	0.02311	0.071303
1372109_at	316516	Tmbim1	transmembrane BAX inhibitor motif con	0.615225	0.023113	0.071303
1379715_at	500042	RGD15662	similar to RIKEN cDNA 2610101N10 (p	0.813814	0.023124	0.071323
1374516_at	364996	RGD13068	similar to RIKEN cDNA 5830457O10 (	1.29715	0.023142	0.071361
1376134_at	288912	RGD13077	similar to hypothetical protein MGC320	0.87334	0.02316	0.0714
1384413_at	498081	RGD15643	similar to solute carrier family 35, mem	-0.679974	0.023206	0.071514
1396776_at	501909	NA	NA	2.123226	0.023208	0.071514
1391530_a_at	289934	RGD13110	similar to hypothetical protein FLJ2060	0.721345	0.023227	0.071555
1371542_at	316531	Tuba4	tubulin, alpha 4	-0.798653	0.023236	0.071567
1372323_at	114123	Sardh	sarcosine dehydrogenase	-1.887055	0.023261	0.071621
1376353_at	362258	Actr5_pred	ARP5 actin-related protein 5 homolog	0.680748	0.023264	0.071621

1389350_at	287774	Apoh	apolipoprotein H	-3.091086	0.023279	0.071651
1391775_at	363676	Zpbp2	zona pellucida binding protein 2	-1.615439	0.023314	0.07174
1384856_at	306340	RGD15639	similar to mKIAA1623 protein (predicte	2.325689	0.023335	0.071789
1369685_at	59327	Twist2	twist homolog 2 (Drosophila)	3.073232	0.023347	0.0718
1380281_at	362559	RGD13078	similar to KIAA1730 protein (predicted)	3.192468	0.023349	0.0718
1375121_at	367100	Smad6_pre	MAD homolog 6 (Drosophila) (predicte	1.464966	0.023419	0.071997
1372603_at	311187	Pacsin3	protein kinase C and casein kinase sul	2.341024	0.023433	0.072024
1383581_at	499752	RGD15655	similar to 60S ribosomal protein L7a (p	-0.826854	0.023447	0.072049
1371965_at	362134	RGD13032	similar to RIKEN cDNA 2010311D03	0.607139	0.023459	0.072068
1396056_at	307429	Dmxl1_pre	Dmx-like 1 (predicted)	0.657907	0.023464	0.072068
1398878_at	252943	Cpsf4	cleavage and polyadenylation specific	0.670907	0.023473	0.072079
1384186_at	297504	RGD15636	similar to mKIAA0212 protein (predicte	1.339384	0.023484	0.072098
1382465_at	25330	Lipe	lipase, hormone sensitive	1.035402	0.023492	0.072106
1368504_at	25328	Lamp1	lysosomal membrane glycoprotein 1	0.650214	0.023508	0.072131
1398458_at	306811	RGD13072	similar to protein kinase, lysine deficier	-0.570696	0.023511	0.072131
1372234_at	499677	LOC49967	NA	-0.890367	0.023526	0.072152
1368590_at	65205	Mmp16	matrix metalloproteinase 16	-1.371277	0.023529	0.072152
1369659_at	116700	Cga	glycoprotein hormones, alpha subunit	2.589374	0.023599	0.072351
1373542_at	308589	Sphk2	sphingosine kinase 2	0.833905	0.023663	0.072521
1379395_a_at	313491	Hint2_pred	histidine triad nucleotide binding protei	0.586241	0.023673	0.072521
1369837_at	60671	Gulo	L-gulonolactone oxidase	-1.217471	0.023674	0.072521
1368031_at	64896	Nolc1	nucleolar and coiled-body phosphopro	0.708833	0.023677	0.072521
1373241_at	309176	Mrpl49	mitochondrial ribosomal protein L49	0.568669	0.023687	0.072534
1390825_at	306866	Slc35b3_pr	solute carrier family 35, member B3 (p	-0.590087	0.023697	0.07254
1384023_at	317395	RGD15608	similar to Spindlin-like (predicted)	-0.681491	0.023699	0.07254
1372154_at	300782	RGD13596	similar to 2010321M09Rik protein	0.648788	0.023743	0.072656
1371044_at	81744	Pde7a	phosphodiesterase 7A	-0.946453	0.023769	0.072708
1370346_at	25203	Ccnb1	cyclin B1	1.158934	0.023773	0.072708
1368339_at	24249	S100g	S100 calcium binding protein G	-1.525864	0.023776	0.072708
1367714_at	84005	Eif2b2	eukaryotic translation initiation factor 2	0.584026	0.023787	0.072724
1375922_at	309391	Cox15	COX15 homolog, cytochrome c oxidas	-0.761028	0.023808	0.072772
1367937_at	252899	Miox	myo-inositol oxygenase	1.35483	0.023818	0.072783
1395981_at	309887	Ascc3_pre	activating signal cointegrator 1 comple	1.268322	0.023838	0.07283
1376554_at	362917	Tatdn1_pre	TatD DNase domain containing 1 (pre	-1.163061	0.023849	0.072845
1385421_at	499836	RGD15632	similar to RIKEN cDNA 1700029I15 (p	1.741406	0.023863	0.072872
1384993_at	308894	RGD13098	similar to peptidylglycine alpha-amidat	-3.153969	0.023878	0.072889
1371892_at	303190	Atpaf2_pre	ATP synthase mitochondrial F1 compl	0.561248	0.02388	0.072889
1370415_at	54355	Rassf5	Ras association (RalGDS/AF-6) doma	-2.466744	0.023887	0.072894
1368533_at	117240	Heph	hephaestin	2.624376	0.023902	0.072923
1393145_at	360833	Fvt1_predi	follicular lymphoma variant translocatic	1.078153	0.023909	0.072929
1374726_at	308099	Fndc1	fibronectin type III domain containing 1	-1.093702	0.023931	0.072962
1387220_at	54272	Mcpt9	mast cell protease 9	2.266758	0.023933	0.072962
1372002_at	24392	Gja1	gap junction membrane channel protei	-1.012622	0.023937	0.072962
1370045_at	85472	Polg	polymerase (DNA directed), gamma	0.596184	0.023957	0.072972
1387062_a_at	140583	Chek1	checkpoint kinase 1 homolog (S. pomb	1.042859	0.023962	0.072972
1393983_at	314879	Xpot_predi	exportin, tRNA (nuclear export recepto	1.1812	0.023965	0.072972
1375181_at	24949	Syn1	synapsin I	0.587741	0.023966	0.072972
1393689_at	296086	Ndufaf1_pr	NADH dehydrogenase (ubiquinone) 1	1.644261	0.023968	0.072972
1398242_at	65179	Ppp5c	protein phosphatase 5, catalytic subun	0.866364	0.02398	0.072992
1375206_at	287466	RGD13082	hypothetical LOC287466	-0.854096	0.023987	0.072997
1373431_at	305131	Lrrc8d	leucine rich repeat containing 8 family,	0.661897	0.023994	0.073

1367537_at	305468	RGD15609	similar to Clast4 protein (predicted)	-1.107093	0.023999	0.073
1388979_at	287768	Smndc1	survival motor neuron domain containi	-0.628547	0.024052	0.073146
1376447_at	308341	RGD15657	similar to hypothetical protein A430110	0.738866	0.024068	0.073176
1375498_at	304488	RGD13103	similar to T-cell activation protein phos	3.203589	0.024086	0.073216
1382565_at	361028	RGD13114	similar to MAPK-interacting and spindle	-1.112348	0.024127	0.073323
1392292_at	287109	Kctd5_pred	potassium channel tetramerisation dor	0.767281	0.024137	0.073337
1398780_at	83583	Rabac1	Rab acceptor 1 (prenylated)	0.629193	0.024145	0.073342
1376583_at	307907	RGD13048	similar to RIKEN cDNA 6430548M08 (	0.97914	0.024174	0.073415
1371865_at	312640	Tmem111	transmembrane protein 111	1.037696	0.024194	0.073443
1374211_at	501795	RGD15660	similar to Hypothetical protein LOC730	-0.955694	0.024194	0.073443
1369466_at	65024	Chrna9	cholinergic receptor, nicotinic, alpha po	-2.003591	0.024224	0.073517
1382603_at	309304	Pdcd1lg2_p	programmed cell death 1 ligand 2 (pre	-2.039405	0.02425	0.073549
1372953_at	553106	Ncald	neurocalcin delta	-0.746866	0.02425	0.073549
1388372_at	296709	Rpl35	ribosomal protein L35	-0.64048	0.024251	0.073549
1367594_at	25181	Bgn	biglycan	-1.797145	0.024272	0.073595
1386914_at	117533	Gmpr	guanosine monophosphate reductase	-3.725351	0.024284	0.073614
1374979_at	301122	Dus3l	dihydrouridine synthase 3-like (S. cere	0.678401	0.024324	0.07371
1398937_at	289693	Dhx15_pre	DEAH (Asp-Glu-Ala-His) box polypepti	-0.664997	0.024331	0.07371
1368307_at	156275	Ggtl3	gamma-glutamyltransferase-like 3	2.490357	0.024332	0.07371
1383220_at	259243	Kcnip4	Kv channel interacting protein 4	-1.538255	0.024342	0.073723
1368223_at	79252	Adamts1	a disintegrin-like and metallopeptidse (	-3.553633	0.024348	0.073723
1374949_at	499871	LOC49987	similar to Zinc finger, FYVE domain co	0.804945	0.024356	0.073729
1398429_at	311254	LOC31125	hypothetical protein LOC311254	0.728435	0.024383	0.073778
1367734_at	24192	Akr1b4	aldo-keto reductase family 1, member	0.765353	0.024384	0.073778
1385614_at	316620	Mlph	melanophilin	1.417877	0.024388	0.073778
1391664_at	366478	Pigv	phosphatidylinositol glycan, class V	1.241052	0.024395	0.073781
1382967_at	266735	Gpr64	G protein-coupled receptor 64	-2.711189	0.024408	0.073805
1390913_at	295671	NA	NA	-0.68141	0.024415	0.073809
1375173_at	246120	LOC24612	RDCR-0918-3 protein	0.769769	0.024436	0.073854
1368588_at	85432	Ddx52	DEAD (Asp-Glu-Ala-Asp) box polypept	-0.825113	0.024449	0.073877
1379311_at	316327	Arid5a	AT rich interactive domain 5A (Mrf1 like	0.887948	0.024455	0.07388
1389453_at	287726	Rdm1_pred	RAD52 motif 1 (predicted)	-1.947143	0.024481	0.073939
1390565_at	291618	RGD15598	similar to potassium channel tetrameris	-2.051231	0.024502	0.073986
1388376_at	293700	RGD13097	similar to RIKEN cDNA 0610038D11 (p	0.622573	0.024518	0.073992
1368854_at	24877	Vsnl1	visinin-like 1	-1.661598	0.024524	0.073992
1387313_at	81523	Myoc	myocilin	-1.544927	0.024525	0.073992
1367956_at	89791	Ncdn	neurochondrin	1.106742	0.024526	0.073992
1391421_at	367046	RGD15642	similar to Acyl-CoA dehydrogenase far	1.023889	0.024533	0.073995
1369430_at	114106	Bcmo1	beta-carotene 15,15'-monooxygenase	-0.775965	0.024544	0.073995
1371887_at	305373	RGD15644	similar to high mobility group protein h	0.725305	0.024544	0.073995
1378895_at	308581	RGD15619	similar to hypothetical protein FLJ3265	1.362766	0.024559	0.074023
1374266_at	307481	Pcdh1_pre	protocadherin 1 (cadherin-like 1) (pred	1.404657	0.024571	0.074043
1375667_at	311692	RGD13119	similar to Protein C20orf177 (predicted	3.314799	0.024578	0.074048
1382358_at	25560	Abcc9	ATP-binding cassette, sub-family C (C	-3.2492	0.024699	0.074394
1368092_at	29383	Fah	fumarylacetoacetate hydrolase	-1.129008	0.024715	0.074427
1391986_at	307070	Larp5_pred	La ribonucleoprotein domain family, m	1.327543	0.024738	0.07446
1368346_at	171079	B3galt4	UDP-Gal:betaGlcNAc beta 1,3-galacto	3.6191	0.024738	0.07446
1392005_at	362773	Prima1_pre	proline rich membrane anchor 1 (predi	0.771476	0.024743	0.07446
1371911_at	287543	Tnfaip1	tumor necrosis factor, alpha-induced p	1.096173	0.024763	0.074492
1371067_at	192271	Adam6	a disintegrin and metallopeptidase dom	3.262498	0.024765	0.074492
1391527_at	362896	Stat6_pred	signal transducer and activator of trans	-1.030627	0.024772	0.074497

1376035_at	362768	Ttc7b_pred	tetratricopeptide repeat domain 7B (pr	-0.863238	0.024779	0.0745
1387092_at	64190	Fxyd4	FXYD domain-containing ion transport	2.63374	0.024789	0.074515
1370788_at	116499	Fgf4	fibroblast growth factor 4	2.580796	0.024868	0.074733
1398400_at	303605	Lyk5	protein kinase LYK5	1.128212	0.024905	0.074829
1388571_at	89815	Syngr2	synaptogyrin 2	0.882209	0.024914	0.074839
1379422_at	300284	LOC30028	similar to RIKEN cDNA 4833435D08	-0.60824	0.024926	0.074841
1373603_at	360494	RGD15657	similar to RIKEN cDNA 0610007P22 (p	0.658232	0.02493	0.074841
1373774_at	291713	Rit2	Ras-like without CAAX 2	-2.092026	0.024932	0.074841
1399133_at	366854	Fbxo7	F-box only protein 7	0.898636	0.024938	0.074843
1373195_at	317385	Fus	fusion, derived from t(12;16) malignant	-0.718025	0.024957	0.074861
1381449_s_at	24827	Tgfa	transforming growth factor alpha	1.078294	0.024958	0.074861
1367768_at	59073	Lxn	latexin	1.602984	0.024961	0.074861
1373732_at	295305	Acp6	acid phosphatase 6, lysophosphatidic	0.733244	0.024971	0.074874
1376690_at	312849	Surb7_pred	SRB7 (suppressor of RNA polymerase	-0.755727	0.025038	0.075057
1372174_at	297900	Peflin	PEF protein with a long N-terminal hyd	0.940947	0.025056	0.075095
1371605_at	299739	Ndufa12_p	NADH dehydrogenase (ubiquinone) 1	-0.677091	0.02515	0.07536
1370511_at	24366	Fgb	fibrinogen, B beta polypeptide	3.495396	0.025246	0.07563
1368717_at	29347	Faah	fatty acid amide hydrolase	0.789864	0.02526	0.075641
1398286_at	60356	Csad	cysteine sulfinic acid decarboxylase	-1.083101	0.025261	0.075641
1367648_at	25662	Igfbp2	insulin-like growth factor binding protei	-3.201836	0.025282	0.075687
1375631_at	301337	Plekhb2_pr	pleckstrin homology domain containing	0.659292	0.025303	0.07573
1387168_at	84398	C1qr1	complement component 1, q subcomp	1.162067	0.02532	0.075765
1369081_at	24591	Neu1	neuraminidase 1	1.281083	0.025396	0.07597
1368404_at	81653	Dbn1	drebrin 1	-0.917568	0.0254	0.07597
1372574_at	308582	Tead2_ma	TEA domain family member 2 (mappe	2.072724	0.025415	0.075985
1389137_at	83620	Cit	citron	0.788644	0.025416	0.075985
1389334_at	314071	Ndufa10	NADH dehydrogenase (ubiquinone) 1	0.561144	0.025461	0.076102
1388458_at	288003	Rfc4_predi	replication factor C (activator 1) 4 (pre	-0.964256	0.025479	0.076137
1385204_at	499807	RGD15655	similar to RIKEN cDNA 4833418A01 (p	1.285243	0.025501	0.076186
1375175_at	360537	Tom1l2_pr	target of myb1-like 2 (chicken) (predict	1.252083	0.025539	0.076284
1367590_at	84509	Ran	RAN, member RAS oncogene family	0.559468	0.025567	0.076349
1387582_a_at	140929	Pde7b	phosphodiesterase 7B	0.937248	0.025604	0.076441
1398357_at	64832	Cplx1	complexin 1	1.25507	0.025615	0.076458
1376455_at	500899	MGC11447	similar to 2310010G13Rik protein	2.195442	0.025683	0.076628
1390268_at	288700	Rab35	RAB35, member RAS oncogene family	-0.9868	0.025683	0.076628
1374731_at	50998	Coil	coilin	-0.801705	0.025698	0.076653
1381985_at	312510	Tia1	cytotoxic granule-associated RNA binc	-1.030784	0.025721	0.076706
1396924_at	362971	Mpped1_pr	metallophosphoesterase domain conta	2.815167	0.025742	0.076739
1368253_at	25257	Gamt	guanidinoacetate methyltransferase	-1.03332	0.025744	0.076739
1393041_at	362519	Smc2l1_pr	SMC2 structural maintenance of chron	-0.92446	0.025761	0.076765
1372312_at	361452	Ltv1	LTV1 homolog (S. cerevisiae)	0.969381	0.025764	0.076765
1377454_at	294289	Zbtb9	zinc finger and BTB domain containing	0.716726	0.025806	0.076856
1380558_at	287638	Dlx3_predi	distal-less homeobox 3 (predicted)	0.724364	0.025807	0.076856
1374409_at	309194	Taf6l_pred	TAF6-like RNA polymerase II, p300/CI	2.306538	0.025812	0.076856
1370013_at	85259	Cnga1	cyclic nucleotide gated channel alpha	1.564273	0.025836	0.07691
1371905_at	288616	MGC94190	similar to 0610007L01Rik protein	0.586026	0.025886	0.077038
1385797_at	29275	Actc1	actin alpha cardiac 1	3.169644	0.025891	0.077038
1387814_at	29161	Cav3	caveolin 3	1.547651	0.025919	0.077104
1375161_at	287356	Mrpl55_pre	mitochondrial ribosomal protein L55 (p	0.598508	0.025935	0.07713
1374165_at	288589	RGD15654	similar to Rasa4 protein (predicted)	-0.754246	0.025948	0.07713
1369670_at	24560	Cd200	Cd200 antigen	-0.995916	0.025949	0.07713

1393103_at	287054	RGD13114	similar to cDNA sequence BC024814 (	-0.729093	0.025951	0.07713
1371597_at	360533	Rnf187_pre	ring finger protein 187 (predicted)	0.670178	0.025962	0.077145
1398309_at	192263	Pigl	phosphatidylinositol glycan, class L	0.948672	0.02604	0.07736
1383120_at	365619	Arsk	arylsulfatase K	-0.928808	0.026091	0.077493
1370852_at	171573	LOC17157	spleen protein 1 precursor	1.665543	0.0261	0.077503
1390124_at	499866	RGD15646	similar to hypothetical protein FLJ3842	-0.806791	0.026106	0.077506
1372551_at	296741	Fastk	Fas-activated serine/threonine kinase	1.116577	0.026118	0.077524
1387726_at	66019	Cdx2	caudal type homeo box 2	1.240442	0.026171	0.077662
1390788_a_at	361305	Wdr36_pre	WD repeat domain 36 (predicted)	0.739333	0.026218	0.077785
1393191_at	500557	RGD15612	similar to RIKEN cDNA 2610200G18 (	0.729461	0.026239	0.07783
1374878_at	360972	Fmip	Fms interacting protein	1.076854	0.026254	0.077856
1382498_x_at	311616	Gdap1l1_p	ganglioside-induced differentiation-ass	0.647793	0.026263	0.077857
1379254_at	289034	RGD13098	similar to RIKEN cDNA 1300007B12; c	-0.575842	0.026266	0.077857
1388912_at	311826	Rexo4	REX4, RNA exonuclease 4 homolog (S	0.562942	0.02629	0.077911
1371250_at	360918	Cxcl4	chemokine (C-X-C motif) ligand 4	-3.449774	0.026345	0.078056
1380285_at	117275	Chrd	chordin	0.839335	0.026357	0.078073
1374524_at	363285	Scly	selenocysteine lyase	0.668995	0.026428	0.078248
1372148_at	295064	Commd2_p	COMM domain containing 2 (predicted	0.707211	0.026429	0.078248
1376471_at	307916	Jph3_predi	junctophilin 3 (predicted)	3.228761	0.026434	0.078248
1385764_at	301114	LOC30111	NA	2.500761	0.026452	0.078286
1371863_at	303698	RGD13050	similar to phosphatidylglycerophospha	0.961774	0.026483	0.07836
1377577_at	295088	Gmps	guanine monphosphate synthetase	0.587506	0.02649	0.078362
1370322_at	286927	Stk16	serine/threonine kinase 16	0.544374	0.026507	0.078396
1373296_a_at	293155	RGD13116	similar to RIKEN cDNA 3200002M19 (	-1.829955	0.026528	0.078442
1387093_at	170698	Slco1a4	solute carrier organic anion transporter	0.609232	0.026543	0.078465
1375276_at	317374	Timm17b_p	translocase of inner mitochondrial mem	0.972067	0.026549	0.078465
1373324_at	360580	Dusp14_pr	dual specificity phosphatase 14 (predic	-1.441405	0.026554	0.078465
1389133_at	298786	Slc30a6_pr	solute carrier family 30 (zinc transporte	0.688873	0.026569	0.07849
1374424_at	364382	Prmt5_pred	protein arginine N-methyltransferase 5	0.956748	0.026587	0.078518
1373866_at	301416	RGD13595	similar to hypothetical protein FLJ1344	-0.904504	0.02659	0.078518
1383376_at	368190	LOC36819	NA	0.739502	0.026626	0.078601
1375909_at	24423	Gstm1	glutathione S-transferase, mu 1	0.569893	0.02663	0.078601
1392227_at	298018	LOC29801	NA	0.758647	0.026662	0.07868
1372012_at	298298	Dhcr24	24-dehydrocholesterol reductase	-0.730499	0.026697	0.078765
1383931_at	360302	Ptprk	protein tyrosine phosphatase, receptor	-1.186529	0.026713	0.078783
1386898_at	25462	Hspe1	heat shock 10 kDa protein 1 (chaperon	-0.739854	0.026715	0.078783
1380091_at	286998	Anubl1	testis-specific gene including a ubiquiti	-1.176933	0.026748	0.078863
1389106_at	288921	Fbxw9	F-box and WD-40 domain protein 9	-0.808809	0.026787	0.078958
1376652_at	298566	C1qa	complement component 1, q subcomp	1.984998	0.02681	0.079007
1372241_at	25502	Oaz1	ornithine decarboxylase antizyme 1	0.551458	0.026815	0.079007
1371697_at	361676	Pnpla2_pre	patatin-like phospholipase domain con	0.890678	0.026823	0.079012
1385718_at	300442	RGD13080	similar to RIKEN cDNA 2510048L02	-1.358291	0.026829	0.079012
1392693_at	295714	RGD13112	similar to fatty acid desaturase 2; linole	2.93169	0.026838	0.079022
1377699_at	304127	Bach1_pre	BTB and CNC homology 1 (predicted)	1.255845	0.026846	0.079027
1384048_at	361607	RGD13107	similar to EMSY protein (predicted)	0.704111	0.026866	0.079053
1394257_at	303214	Trim16_pre	tripartite motif protein 16 (predicted)	2.030368	0.026873	0.079053
1393860_at	365352	Mical2_pre	microtubule associated monoxygenase	1.781433	0.026876	0.079053
1376914_at	689490	LOC68949	NA	-1.703899	0.026878	0.079053
1390435_at	361111	RGD13075	LOC361111 (predicted)	1.181467	0.026894	0.079081
1398478_at	314320	Mlh3_predi	mutL homolog 3 (E. coli) (predicted)	1.099835	0.026922	0.079147
1393246_at	363139	Zmynd10	zinc finger, MYND domain-containing 1	-2.545708	0.02693	0.07915

1393893_at	362122	RGD13077	similar to CG3306-PA	-1.180268	0.026961	0.079216
1386514_at	361129	Hapln4_pre	hyaluronan and proteoglycan link prote	-1.9177	0.026964	0.079216
1373128_at	494125	Rcn3_pred	reticulocalbin 3, EF-hand calcium bind	-1.682714	0.026991	0.079261
1370236_at	29411	Ppt1	palmitoyl-protein thioesterase 1	-0.815338	0.026991	0.079261
1381836_at	502099	NA	NA	1.227243	0.02704	0.079388
1373823_at	498709	RGD15620	similar to Cyclin-dependent kinases re	-2.213169	0.02712	0.079604
1373917_at	307503	Etf1	eukaryotic translation termination facto	-0.695643	0.027143	0.079653
1398887_at	295234	RGD15625	similar to Late endosomal/lysosomal M	0.534242	0.027199	0.079799
1389923_at	294318	Btbd9	BTB (POZ) domain containing 9	1.131843	0.02721	0.079815
1395974_at	303312	RGD13113	similar to hypothetical protein (predicte	0.71969	0.02725	0.079915
1377338_at	294800	Rad1_pred	RAD1 homolog (S. pombe) (predicted)	-0.617723	0.027277	0.079966
1384210_at	293180	RSB-11-77	RSB-11-77 protein	-1.03047	0.027279	0.079966
1370808_at	25035	Cyb5r3	cytochrome b5 reductase 3	-0.659618	0.027291	0.079979
1398770_at	81769	Rpl36a	large subunit ribosomal protein L36a	0.625674	0.027296	0.079979
1376639_at	314613	Rnf126	ring finger protein 126	1.798107	0.027325	0.080037
1367924_at	171444	Pacs1	phosphofurin acidic cluster sorting prot	-0.787649	0.027328	0.080037
1368152_at	170906	Zdhhc7	zinc finger, DHHC domain containing 7	0.847472	0.027393	0.080201
1369997_at	83721	Dvl1	dishevelled, dsh homolog 1 (Drosophil	1.516682	0.027396	0.080201
1373410_at	499497	RGD15631	similar to MADS box transcription enha	-3.300632	0.027413	0.080234
1374379_at	360881	Dusp23_pr	dual specificity phosphatase 23 (predic	1.064287	0.027445	0.080308
1368586_at	171449	Zg16	zymogen granule protein 16	-1.692105	0.027451	0.080308
1373666_at	362799	Rapgef5	Rap guanine nucleotide exchange fact	-3.239704	0.027459	0.080313
1382038_at	362607	Kpna6	karyopherin (importin) alpha 6	0.864952	0.027471	0.080332
1393060_at	311827	RGD13054	similar to KIAA0605 gene product (pre	-1.77438	0.027507	0.080419
1370042_at	84510	Stmn2	stathmin-like 2	0.710123	0.027538	0.080487
1372440_at	29366	Serpine2	serine (or cysteine) proteinase inhibito	0.916061	0.027542	0.080487
1387478_at	25646	Otx1	orthodenticle homolog 1 (Drosophila)	1.070524	0.027555	0.080506
1369679_a_at	25492	Nfia	nuclear factor I/A	-1.224768	0.027564	0.080515
1388381_at	298700	Eif3s4	eukaryotic translation initiation factor 3	0.672234	0.027586	0.08056
1391702_at	308365	Zfp446_pre	zinc finger protein 446 (predicted)	0.575862	0.027606	0.080591
1384425_at	304131	LOC30413	similar to C21ORF7	2.311745	0.027608	0.080591
1388062_at	286997	Mapk15	mitogen-activated protein kinase 15	1.891783	0.027636	0.08065
1367980_at	54190	Rabep1	rabaptin, RAB GTPase binding effecto	-0.700611	0.027645	0.08065
1393564_at	303963	RGD15632	similar to Ubiquitin ligase protein DZIP	1.245148	0.027647	0.08065
1380035_at	500501	MGC12500	similar to RIKEN cDNA 5830433M19	-0.998284	0.027675	0.080714
1394761_at	300350	NA	NA	-2.819974	0.027695	0.080755
1391022_at	305078	Lamb3	laminin, beta 3	0.641089	0.027709	0.080764
1382958_at	25708	Ucp3	uncoupling protein 3 (mitochondrial, pr	2.637386	0.02771	0.080764
1385592_at	317346	Bcor_predi	Bcl6 interacting corepressor (predicted	-0.705076	0.027725	0.080785
1371027_at	171136	Cblb	Casitas B-lineage lymphoma b	0.832338	0.02773	0.080785
1368779_a_at	25206	Gucy1b2	guanylate cyclase 1, soluble, beta 2	3.258552	0.02774	0.080797
1395054_at	361495	Usp29_pre	ubiquitin specific protease 29 (predicte	1.961039	0.027755	0.080822
1382264_at	54244	Crebbp	CREB binding protein	-0.935086	0.027764	0.080827
1378723_at	502499	NA	NA	1.796635	0.027769	0.080827
1381282_at	308996	NA	NA	0.953154	0.027813	0.080938
1394530_at	301004	RGD13110	similar to hypothetical protein FLJ2025	-0.769287	0.027825	0.080955
1372787_at	305258	Crlz1	charged amino acid rich leucine zipper	0.625105	0.027848	0.081004
1368272_at	24401	Got1	glutamate oxaloacetate transaminase	0.668681	0.027886	0.081096
1385175_at	303480	Hoxb13_pr	homeo box B13 (predicted)	2.302238	0.027897	0.081111
1389141_at	301430	Orc2l	origin recognition complex, subunit 2-li	-0.609132	0.027903	0.081112
1393554_at	361709	RGD15626	similar to hypothetical protein DKFZp7	0.73708	0.027924	0.081155

1388696_at	84478	Ufd1l	ubiquitin fusion degradation 1-like	0.543495	0.027947	0.081204
1367591_at	64371	Prdx3	peroxiredoxin 3	0.724763	0.027983	0.081273
1372300_at	361364	Dok4_pred	docking protein 4 (predicted)	-2.281807	0.027993	0.081273
1388747_at	361643	Lcmt1	leucine carboxyl methyltransferase 1	-0.585834	0.027994	0.081273
1373273_at	298374	Prpf38a_pr	PRP38 pre-mRNA processing factor 3	-0.792701	0.027996	0.081273
1377664_at	171457	Kab	KARP-1 binding protein 1	-0.816139	0.028021	0.081329
1396584_at	498281	RGD1559	similar to nectin 4 (predicted)	5.179444	0.028033	0.081345
1368089_at	81743	Pde2a	phosphodiesterase 2A, cGMP-stimulat	1.562097	0.02804	0.081349
1398921_at	56281	Mrpl37	mitochondrial ribosomal protein L37	0.772507	0.02805	0.081359
1376761_at	84579	Hdac4	histone deacetylase 4	1.376142	0.028056	0.081359
1391087_at	365389	Lrrc56	leucine rich repeat containing 56	2.023334	0.028085	0.081424
1388913_at	246115	Ppap2c	phosphatidic acid phosphatase type 2	1.189558	0.028118	0.081502
1387964_a_at	171562	Ero1l	ERO1-like (S. cerevisiae)	1.607866	0.028163	0.0816
1383743_at	498751	NA	NA	-0.799648	0.028164	0.0816
1387792_at	65278	Csnk1g2	casein kinase 1, gamma 2	0.696708	0.028248	0.081825
1376279_at	315045	Pop1_pred	processing of precursor 1, ribonucleas	1.168898	0.028254	0.081826
1370594_at	302822	Igsf1	immunoglobulin superfamily, member	0.683847	0.0283	0.08194
1385673_at	287463	RGD13084	similar to hypothetical protein MGC41	1.138032	0.028373	0.082135
1380331_at	499205	RGD15646	similar to A830059I20Rik protein (pred	1.341172	0.028402	0.082201
1392965_a_at	292401	Smoc2_pre	SPARC related modular calcium bindi	-1.495543	0.028438	0.082287
1372800_at	361514	Meis3_pre	Meis1, myeloid ecotropic viral integrati	0.575714	0.028484	0.082403
1388993_at	282845	Rnf34	ring finger protein 34	0.903883	0.028505	0.08243
1387146_a_at	50672	Ednrb	endothelin receptor type B	0.938714	0.028508	0.08243
1367909_at	171408	Dcxr	dicarbonyl L-xylulose reductase	2.221169	0.028512	0.08243
1367715_at	25625	Tnfrsf1a	tumor necrosis factor receptor superfa	1.42768	0.028524	0.082445
1382137_at	291793	Abhd3_pre	abhydrolase domain containing 3 (pre	-0.707427	0.028552	0.082508
1376086_at	361151	RGD13111	similar to RIKEN cDNA 1810047C23	0.677951	0.028561	0.082514
1368977_a_at	84384	Fxc1	fractured callus expressed transcript 1	0.596868	0.028571	0.082514
1389577_at	291987	Cirh1a	cirrhosis, autosomal recessive 1A (hur	0.537192	0.028574	0.082514
1393731_at	266712	Rnf40	ring finger protein 40	1.294925	0.028583	0.082514
1367854_at	24159	Acly	ATP citrate lyase	0.699479	0.028591	0.082514
1382592_at	303037	RGD13069	similar to RIKEN cDNA 2600001J17	1.13646	0.028593	0.082514
1388926_at	316249	Enpp5	ectonucleotide pyrophosphatase/phos	-0.57471	0.028598	0.082514
1381685_a_at	287044	RGD13057	similar to CG12753-PA (predicted)	0.599336	0.028617	0.082548
1396035_at	297757	RGD15597	similar to RPE-spondin (predicted)	-2.977278	0.028622	0.082548
1390789_at	315973	Acad11_pr	acyl-Coenzyme A dehydrogenase fam	1.051343	0.028655	0.082625
1369122_at	24887	Bax	Bcl2-associated X protein	0.717654	0.028671	0.082652
1376144_at	303905	Parp9_pre	poly (ADP-ribose) polymerase family,	-1.002115	0.02868	0.082662
1384465_at	305910	Pspc1	paraspeckle protein 1	-0.587504	0.02875	0.082847
1392206_at	305865	RGD15611	similar to solute carrier family 35, mem	-4.351927	0.028791	0.082947
1388468_at	499672	Cdc42se1	CDC42 small effector 1	0.672629	0.028844	0.083072
1367702_at	24158	Acadm	acetyl-Coenzyme A dehydrogenase, m	-0.999309	0.028847	0.083072
1383943_at	252893	Dnah7	dynein, axonemal, heavy polypeptide 7	-2.756518	0.02886	0.08309
1373286_at	362650	Fblim1	filamin binding LIM protein 1	0.758299	0.028873	0.083109
1397205_at	299131	Dhrs7	dehydrogenase/reductase (SDR family	3.79559	0.028883	0.08312
1376193_at	363455	Chrdl1	kohjirin	-2.131343	0.028895	0.083137
1384943_at	360692	RGD15638	similar to DNA segment, Chr 16, ERA	-0.817518	0.028915	0.083177
1393741_at	308766	Mesp1_pre	mesoderm posterior 1 (predicted)	1.305631	0.028951	0.083261
1388975_at	361071	Sucla2_pre	succinate-Coenzyme A ligase, ADP-fo	1.016707	0.028976	0.083317
1380717_at	361529	Znf626_pre	zinc finger protein 626 (predicted)	0.699025	0.029034	0.083459
1383883_at	316624	RGD13096	similar to Hypothetical protein KIAA04	0.653637	0.029039	0.083459

1380946_at	302405	RGD15663	RGD1566367 (predicted)	-2.216252	0.029045	0.083459
1388892_at	305853	Rab2b	RAB2B, member RAS oncogene famil	0.600205	0.029115	0.083644
1397581_at	171581	Rhov	ras homolog gene family, member V	-1.875559	0.029138	0.08369
1384198_at	302817	Cova1_pre	cytosolic ovarian carcinoma antigen 1	-1.143711	0.029152	0.083713
1393199_at	317432	RGD15606	similar to Jumonji/ARID domain-contai	-1.163112	0.029185	0.083774
1371982_at	286897	Aip1	AIP1	-0.535509	0.029186	0.083774
1369191_at	24498	Il6	interleukin 6	-3.68755	0.029215	0.083839
1377079_a_at	289219	Ppox_pred	protoporphyrinogen oxidase (predicted	1.2199	0.029232	0.083869
1372828_at	361286	Msrb2	methionine sulfoxide reductase B2	0.785477	0.029248	0.083898
1368917_at	117260	Nudt1	nudix (nucleoside diphosphate linked r	0.774914	0.029278	0.083966
1369941_at	64322	Dap	death-associated protein	-0.770059	0.029294	0.083993
1398301_at	293291	RGD15638	similar to ribosomal protein L36 (predic	1.106668	0.029305	0.084007
1374043_at	307288	Gramd3	GRAM domain containing 3	-1.127891	0.029316	0.084018
1372115_at	363190	NA	NA	0.782484	0.029348	0.084094
1398914_at	288588	Polr2j_pred	polymerase (RNA) II (DNA directed) po	0.748435	0.029358	0.084103
1367634_at	64298	Rpl31	ribosomal protein L31	-0.586817	0.029393	0.084186
1378800_at	300115	Arhgap8	Rho GTPase activating protein 8	0.607756	0.0294	0.084186
1393794_at	295654	RGD15651	similar to tetratricopeptide repeat doma	-0.759742	0.029425	0.08424
1374146_at	313702	Mad2l2	MAD2 mitotic arrest deficient-like 2 (ye	0.678512	0.029451	0.084296
1399089_at	299139	Slc38a6	solute carrier family 38, member 6	-2.537907	0.029488	0.084385
1391963_at	29737	Kcnab1	potassium voltage-gated channel, shal	1.997926	0.029536	0.084502
1384908_at	311349	Ttbk1_pred	tau tubulin kinase 1 (predicted)	1.179583	0.029579	0.084602
1379365_at	305236	Cxcl11	chemokine (C-X-C motif) ligand 11	2.39524	0.029583	0.084602
1389470_at	294257	Cfb	complement factor B	3.792855	0.029635	0.084731
1386887_at	94194	Cox5b	cytochrome c oxidase subunit Vb	0.546526	0.029641	0.084732
1370812_at	24888	Bcl2l1	Bcl2-like 1	1.049563	0.029688	0.084846
1368201_at	24603	Npr1	natriuretic peptide receptor 1	2.57423	0.029714	0.084902
1372644_at	362949	NA	NA	0.755327	0.029722	0.084908
1387551_at	170739	Kcnh7	potassium voltage-gated channel, subf	2.521072	0.029744	0.084952
1379906_at	24214	Atp1b2	ATPase, Na+/K+ transporting, beta 2 p	-1.256239	0.029753	0.08496
1392466_at	293098	RGD13099	similar to CG11737-PA	-0.708206	0.029763	0.084968
1398184_at	314212	Daam1_pre	dishevelled associated activator of mo	0.770177	0.029785	0.085013
1389171_at	306327	Tmem38a_	transmembrane protein 38a (predicted	0.809326	0.029829	0.085121
1383337_at	307913	RGD15626	similar to genetic suppressor element	-0.554588	0.029837	0.085126
1369156_at	79209	Frk	fyn-related kinase	-0.64971	0.029849	0.085141
1368475_at	29755	Colq	collagen-like tail subunit (single strand	0.970771	0.029858	0.085143
1383643_at	499317	RGD15603	similar to UPF0197 protein C11orf10 h	0.708825	0.029862	0.085143
1379062_at	500994	NA	NA	1.03423	0.029915	0.085274
1390214_a_at	296639	Ciz1_predi	CDKN1A interacting zinc finger protein	0.836237	0.029924	0.08528
1388368_at	305391	Med28_pre	mediator of RNA polymerase II transcr	-0.644006	0.029956	0.085354
1368328_at	25623	Gys2	glycogen synthase 2	-3.701393	0.029963	0.085356
1384125_at	311968	Mll5	myeloid/lymphoid or mixed-lineage leu	-0.648291	0.030082	0.085658
1371133_a_at	24679	Prkar2b	protein kinase, cAMP dependent regul	0.684807	0.030084	0.085658
1383618_at	303685	Mrpl38	mitochondrial ribosomal protein L38	-0.820663	0.030089	0.085658
1388621_at	287456	RGD15632	similar to RIKEN cDNA 1110030J09 (p	0.605209	0.030106	0.08569
1390155_at	305795	Abhd6	abhydrolase domain containing 6	-0.766289	0.030134	0.085731
1368510_at	25172	Gata1	GATA binding protein 1	1.498212	0.030137	0.085731
1382105_at	83579	Gnb5	guanine nucleotide binding protein, be	-0.785494	0.030143	0.085731
1372663_at	293620	Ptdss2_pre	phosphatidylserine synthase 2 (predict	1.084838	0.030146	0.085731
1391503_at	361314	Centd3_pre	centaurin, delta 3 (predicted)	1.751764	0.030156	0.085739
1386956_at	25073	Scarb1	scavenger receptor class B, member 1	0.885731	0.030176	0.08578

1384264_at	308572	Myh14	myosin, heavy polypeptide 14	0.966099	0.030184	0.085782
1390129_at	308265	RGD1564C	similar to LATS homolog 1 (predicted)	-0.650346	0.030206	0.085826
1389023_at	363160	RGD13115	similar to Oligosaccharyl transferase 3	0.732655	0.030221	0.085851
1373250_at	353229	Tmem23	transmembrane protein 23	-0.811894	0.030259	0.08594
1388519_at	298068	Sec61b_pr	Sec61 beta subunit (predicted)	0.595039	0.030314	0.086078
1387676_at	117016	Mark1	MAP/microtubule affinity-regulating kin	-0.535601	0.030352	0.086165
1368424_at	84351	Ikbkb	inhibitor of kappaB kinase beta	0.869963	0.030358	0.086165
1372085_at	298757	Arl6ip2	ADP-ribosylation factor-like 6 interactir	-0.774843	0.030406	0.086284
1381391_at	287798	MGC9521(	hypothetical LOC287798	2.282602	0.030424	0.086289
1379343_at	301418	MGC94335	similar to hypothetical protein FLJ2255	-0.941964	0.030425	0.086289
1380197_at	304813	Ppp1r12b_	protein phosphatase 1, regulatory (inhi	1.2832	0.030427	0.086289
1395007_at	307839	Zfp612_pre	zinc finger protein 612 (predicted)	-1.286095	0.030441	0.08631
1371599_at	294154	LOC29415	similar to chromosome 6 open reading	0.539388	0.030453	0.086325
1369953_a_at	25145	Cd24	CD24 antigen	3.551003	0.030463	0.086336
1393953_at	295361	Eps8l3_pre	EPS8-like 3 (predicted)	-2.044705	0.030514	0.08646
1386968_at	58977	Ppp1r1a	protein phosphatase 1, regulatory (inhi	1.062492	0.030521	0.086462
1394433_at	288449	Katnal1	katanin p60 subunit A-like 1	1.592864	0.030536	0.086487
1369328_at	116719	Acacb	acetyl-Coenzyme A carboxylase beta	2.47717	0.030545	0.086493
1389966_at	367313	Col6a3_pre	procollagen, type VI, alpha 3 (predicte	-2.421886	0.030552	0.086493
1384443_at	296083	Vps18_pre	vacuolar protein sorting 18 (yeast) (pre	1.117265	0.030578	0.086544
1397409_s_at	308937	Wee1	wee 1 homolog (S. pombe)	-0.907712	0.030582	0.086544
1378233_at	362606	Sync_pred	syncoilin (predicted)	2.624111	0.030615	0.086616
1372741_at	305021	Sccpdh	saccharopine dehydrogenase (putative	0.600486	0.030621	0.086616
1374982_at	362944	Ppp1r16a_	protein phosphatase 1, regulatory (inhi	0.989134	0.030636	0.08664
1394802_at	59267	Syt7	synaptotagmin VII	1.417188	0.030647	0.086653
1387943_at	286995	Defa	defensin, alpha 5, Paneth cell-specific	-0.655643	0.030673	0.086688
1376039_at	261730	Stk6	serine/threonine kinase 6	2.31049	0.030673	0.086688
1388967_at	317376	Tcfe3_prec	transcription factor E3 (predicted)	-1.106787	0.030683	0.086695
1372118_at	497878	MGC1252(	HMP19 protein	-0.752775	0.030688	0.086695
1389970_at	297728	RGD13106	similar to RIKEN cDNA 1200009B18; E	0.736181	0.030709	0.086728
1371036_at	497815	Nrcam	neuron-glia-CAM-related cell adhesion	0.542189	0.030713	0.086728
1373452_at	309301	Rcl1	RNA terminal phosphate cyclase-like 1	0.674978	0.030784	0.08691
1371483_at	310378	Nnt_mappe	nicotinamide nucleotide transhydroger	-0.655043	0.030794	0.08692
1379700_at	313950	RGD13113	similar to HS1 binding protein 3 (predic	3.074046	0.030828	0.086997
1384855_at	498278	RGD15626	similar to RIKEN cDNA 1700009P17 (p	-1.406453	0.030867	0.087087
1389559_at	362919	NA	NA	0.54228	0.030937	0.087268
1368215_at	83534	Tpp1	tripeptidyl peptidase I	0.800877	0.030955	0.087285
1399051_at	361736	RGD13055	similar to expressed sequence AV312(	-0.899197	0.030956	0.087285
1377660_at	293613	RGD13093	similar to transthyretin (4L369) (predict	-2.313285	0.030966	0.087292
1375226_at	365372	Tbc1d10b_	TBC1 domain family, member 10b (pre	0.921257	0.030981	0.087296
1398336_at	301515	Rnf25	ring finger protein 25	0.832975	0.030984	0.087296
1370448_at	171517	Gpc2	glypican 2 (cerebroglycan)	1.35641	0.030987	0.087296
1387771_a_at	50689	Mapk3	mitogen activated protein kinase 3	0.84687	0.031018	0.087363
1376523_at	314205	Arid4a_pre	AT rich interactive domain 4A (Rbp1 lil	1.135438	0.031024	0.087363
1379852_at	311721	Urkl1_pred	uridine kinase-like 1 (predicted)	-3.216765	0.031098	0.087534
1378160_at	310810	Sdfr2_pred	stromal cell derived factor receptor 2 (p	4.010409	0.031098	0.087534
1382035_at	117526	Tbp	TATA box binding protein	-0.615584	0.031105	0.087536
1379323_at	311800	RGD13063	similar to hypothetical protein MGC143	0.55472	0.031158	0.087666
1369459_at	89812	Pip5k2b	phosphatidylinositol-4-phosphate 5-kin	2.420065	0.031176	0.087691
1370455_a_at	252920	Olfm3	olfactomedin 3	-3.029226	0.03118	0.087691
1385229_at	306081	Pcdh20_pr	protocadherin 20 (predicted)	-1.566687	0.031193	0.087708

1391310_at	308053	RGD13075	similar to intracellular protein transport	1.16289	0.031212	0.087745
1389770_at	311548	LOC31154	similar to RIKEN cDNA 4930509O20	1.681937	0.031221	0.087749
1368877_at	24522	Zfp354a	zinc finger protein 354A	-0.538142	0.031232	0.087762
1386967_at	85428	Rhoq	ras homolog gene family, member Q	-0.882978	0.031245	0.08778
1383258_at	360894	RGD13105	similar to hypothetical protein FLJ1414	-1.061447	0.031253	0.08778
1388586_at	85238	Synj1	synaptojanin 1	-0.775088	0.031259	0.08778
1391101_at	307016	Arhgap12_	Rho GTPase activating protein 12 (pre	-0.545202	0.031265	0.08778
1369991_at	65166	Sec11l1	Sec11-like 1 (S. cerevisiae)	0.822046	0.031277	0.087795
1377797_at	312706	Emg1_pre	EMG1 nucleolar protein homolog (S. c	1.066562	0.031302	0.087848
1383098_at	360726	Fyttd1	forty-two-three domain containing 1	-0.767275	0.031327	0.087891
1368769_at	83569	Abcb11	ATP-binding cassette, sub-family B (M	-1.58908	0.031331	0.087891
1374440_at	289456	Dhrs8	dehydrogenase/reductase (SDR family	-0.910646	0.031341	0.087901
1391825_at	497910	NA	NA	1.218187	0.03137	0.087964
1383224_at	362279	Pard6b_pre	par-6 (partitioning defective 6) homolo	-0.639408	0.031383	0.08798
1371490_at	286899	Hsbp1	heat shock factor binding protein 1	0.524332	0.031395	0.087996
1388940_at	500246	RGD15621	similar to Rho-GTPase-activating prote	2.054503	0.031402	0.087997
1370030_at	29739	Gclm	glutamate cysteine ligase, modifier sub	0.72434	0.031416	0.088017
1398771_at	50567	Slc3a2	solute carrier family 3 (activators of dib	0.55445	0.031458	0.088118
1373674_at	362429	Mfap5_pre	microfibrillar associated protein 5 (pred	-1.962449	0.031485	0.088159
1388449_at	363241	Eef1b2_pre	eukaryotic translation elongation factor	-0.557792	0.031495	0.088159
1371168_at	85275	Mpp2	membrane protein, palmitoylated 2 (MA	-0.843504	0.031499	0.088159
1367765_at	64365	Tcn2	transcobalamin 2	0.878713	0.031501	0.088159
1390374_at	360903	Fgfrl1	fibroblast growth factor receptor-like 1	2.584736	0.031509	0.088159
1379026_at	287556	Rhbdl4_pre	rhomboid, veinlet-like 4 (Drosophila) (p	-1.453073	0.031513	0.088159
1392548_at	362153	RGD15653	similar to mKIAA1604 protein (predicte	-0.776404	0.031523	0.088168
1398813_at	117553	Ube1c	ubiquitin-activating enzyme E1C	-0.744806	0.031529	0.088168
1399135_at	300189	MGC9495	similar to RIKEN cDNA 2310042P20	-0.617968	0.031573	0.088247
1386981_at	25027	Slc16a1	solute carrier family 16 (monocarboxyl	-1.988143	0.031578	0.088247
1374771_at	303019	RGD15649	similar to neuralized homolog (predicte	-0.682882	0.031584	0.088247
1398880_at	192269	Sub1	SUB1 homolog (S. cerevisiae)	-0.648886	0.031585	0.088247
1384220_at	316221	Tbcc_predi	tubulin-specific chaperone c (predicted	-1.016645	0.031591	0.088247
1399029_at	362636	Usp48	ubiquitin specific protease 48	-0.598407	0.0316	0.088253
1392267_at	312257	RGD13061	similar to RIKEN cDNA B930096L08 (p	-2.757597	0.031607	0.088254
1383101_at	361389	Anapc10_p	anaphase promoting complex subunit	-0.841104	0.031636	0.088316
1377323_at	311341	Pla2g4b_p	phospholipase A2, group IVB (cytosoli	3.288491	0.031758	0.088638
1390697_at	363462	Fam51a1	family with sequence similarity 51, mer	-0.61843	0.031821	0.088795
1376766_at	287746	Fmnl1_pre	formin-like 1 (predicted)	-1.385221	0.031836	0.088796
1377480_at	300687	RGD13080	similar to RIKEN cDNA D030060M11	0.688266	0.031845	0.088796
1367650_at	94174	Lcn7	lipocalin 7	3.72732	0.031845	0.088796
1396208_at	29566	Ggtla1	gamma-glutamyltransferase-like activit	-4.626914	0.031852	0.088796
1370551_a_at	29744	Sema6c	sema domain, transmembrane domain	1.15217	0.031855	0.088796
1396628_at	502898	NA	NA	2.143888	0.031865	0.088805
1373179_at	305122	RGD13069	similar to mKIAA1107 protein (predicte	1.398512	0.031925	0.088955
1373481_at	304763	R3hdm1	R3H domain containing 1	0.587564	0.031948	0.089001
1389510_at	289707	Lyar	Ly1 antibody reactive clone	0.85196	0.031993	0.089106
1388107_at	246255	Ppp2r2d	protein phosphatase 2, regulatory subu	-0.556514	0.032005	0.089118
1385483_at	289236	RGD15624	similar to hypothetical protein A030011	-2.249373	0.032011	0.089118
1389485_at	314859	LOC31485	similar to transformed mouse 3T3 cell	-1.0079	0.032031	0.089156
1373232_at	311772	RGD15660	similar to nidogen 2 (predicted)	0.919797	0.032049	0.089188
1373274_at	297437	Txnrd3_pre	thioredoxin reductase 3 (predicted)	-0.597454	0.032066	0.089216
1368220_at	81673	Gtf2b	general transcription factor IIB	-0.512401	0.032085	0.089249

1393799_at	60630	Unc5b	unc-5 homolog B (C. elegans)	1.122935	0.032121	0.08933
1371310_s_at	29345	Serpinh1	serine (or cysteine) proteinase inhibito	-1.022932	0.032151	0.089396
1383371_at	367991	NA	NA	2.043386	0.032195	0.089494
1399154_at	361700	Fbxl11_pre	F-box and leucine-rich repeat protein 1	-0.653096	0.0322	0.089494
1388406_at	363545	RGD13089	similar to RIKEN cDNA 1200013P24	1.346555	0.032232	0.089564
1373465_at	361352	Pqlc1	PQ loop repeat containing 1	-0.738886	0.032246	0.089586
1379168_at	311786	Traf2_pred	Tnf receptor-associated factor 2 (predi	1.232577	0.032264	0.089617
1397568_at	362246	Trp53inp2	tumor protein p53 inducible nuclear pro	-1.61165	0.03229	0.089657
1374910_at	83465	Celsr2	cadherin EGF LAG seven-pass G-type	-0.83622	0.032292	0.089657
1393804_at	362472	RGD13070	similar to hypothetical protein FLJ2249	-1.066858	0.032322	0.089718
1373295_at	294025	Obfc1	oligonucleotide/oligosaccharide-bindin	-2.082982	0.032328	0.089718
1386984_at	50554	Smad4	MAD homolog 4 (Drosophila)	-0.512012	0.032335	0.089719
1387264_at	116491	Kcnk6	potassium channel, subfamily K, meml	1.403933	0.032349	0.089741
1383558_at	500255	RGD15605	similar to MJ0495-like protein SelB (pr	0.928418	0.032361	0.089742
1386205_at	314323	LOC31432	transporter	-1.443809	0.032366	0.089742
1372114_at	498839	NA	NA	-0.548988	0.03237	0.089742
1376439_at	296968	RGD13082	similar to hypothetical protein FLJ3278	2.228316	0.032405	0.089778
1374147_at	296478	Zgpat	zinc finger, CCCH-type with G patch d	0.924746	0.032407	0.089778
1388025_at	81644	Opn1sw	opsin 1 (cone pigments), short-wave-s	3.224249	0.032417	0.089778
1367900_at	81675	Gyg1	glycogenin 1	-0.537558	0.032418	0.089778
1383674_at	308986	RGD15605	RGD1560566 (predicted)	0.747366	0.032419	0.089778
1392482_at	291565	RGD13115	similar to actin filament associated pro	-0.592198	0.032425	0.089778
1398795_at	116483	Dars	aspartyl-tRNA synthetase	-0.600669	0.032431	0.089778
1387150_at	54282	Padi1	peptidyl arginine deiminase, type I	1.0436	0.032437	0.089778
1388404_at	360762	LOC36076	RNA polymerase 1-3	-0.537325	0.032448	0.089788
1371908_at	296219	Nxt1_predi	NTF2-related export protein 1 (predicte	0.757332	0.032458	0.089793
1372082_at	298598	Necap2	NECAP endocytosis associated 2	0.833954	0.032465	0.089793
1372670_at	300675	RGD13076	similar to hypothetical protein FLJ2182	-0.898068	0.03247	0.089793
1387225_at	171116	Opa1	optic atrophy 1 homolog (human)	1.177969	0.032493	0.089838
1382135_at	315903	RGD13078	similar to CG9346-PA (predicted)	-0.612962	0.032506	0.089854
1385332_at	366593	Dus4l_pred	dihydrouridine synthase 4-like (S. cere	0.860896	0.032518	0.089855
1380317_at	29181	Cdh10	cadherin 10	2.693274	0.03252	0.089855
1387169_at	84424	Tle3	transducin-like enhancer of split 3, E(s	-0.801794	0.032633	0.09015
1378787_a_at	360741	Dgcr14	DiGeorge syndrome critical region gen	1.512548	0.032649	0.090175
1381381_at	311909	Mnab_pred	membrane associated DNA binding pr	1.103835	0.032691	0.090273
1398315_at	245981	Rpl15	ribosomal protein L15	0.670895	0.032705	0.090287
1374593_at	29340	Prkce	protein kinase C, epsilon	0.752069	0.03271	0.090287
1373503_at	360779	Lrch4_pred	leucine-rich repeats and calponin hom	-1.439634	0.032727	0.090315
1372795_at	292008	Txnl4b	thioredoxin-like 4B	-0.606925	0.032742	0.090319
1386959_a_at	29568	Map2k5	mitogen activated protein kinase kinas	0.528639	0.032743	0.090319
1372584_at	364208	RGD13083	similar to DKFZP566K1924 protein	-0.846642	0.032756	0.090319
1398774_at	64640	Rpl30	ribosomal protein L30	-0.552817	0.032756	0.090319
1372556_at	502374	LOC50237	hypothetical protein LOC502374	0.501316	0.032777	0.09036
1373040_at	293427	Eif3s5_pre	eukaryotic translation initiation factor 3	-0.72538	0.032795	0.09039
1372346_at	313913	Zfp513	zinc finger protein 513	0.673666	0.03283	0.090468
1379419_at	291946	Tmem34	transmembrane protein 34	-0.819824	0.032845	0.090488
1367858_at	25481	Mmp11	matrix metallopeptidase 11	-3.076425	0.032857	0.090503
1389146_at	498796	LOC49879	hypothetical protein LOC498796	-1.756663	0.032902	0.090609
1379367_at	362676	RGD13082	similar to RIKEN cDNA 2810405K02 (p	0.907468	0.032949	0.090719
1371881_at	309177	Znhit2_pre	zinc finger, HIT domain containing 2 (p	0.857213	0.03296	0.09073
1372813_at	360617	RGD13066	similar to RIKEN cDNA 1810046J19 (p	0.555875	0.032988	0.090774

1371397_at	292894	Nosip_prec	nitric oxide synthase interacting protei	0.516977	0.032989	0.090774
1369508_at	170847	Kcnj15	potassium inwardly-rectifying channel,	0.724779	0.032998	0.090778
1398341_at	287661	RGD15597	RGD1559720 (predicted)	1.157536	0.033008	0.090778
1390798_at	24699	Ptprc	protein tyrosine phosphatase, receptor	-3.32776	0.033012	0.090778
1388880_at	303377	Slfn5_pred	schlafen 5 (predicted)	-1.926418	0.0331	0.091002
1396158_at	366232	Rem1	rad and gem related GTP binding prote	1.048607	0.033119	0.091032
1374883_at	306490	Mtmr7_pre	myotubularin related protein 7 (predict	0.814126	0.033131	0.091032
1373362_at	293448	Uqcrc2	ubiquinol cytochrome c reductase core	-0.52368	0.033131	0.091032
1370664_a_at	286986	LOC28698	putative pheromone receptor Go-VN13	0.574685	0.033159	0.091089
1383591_at	299620	Sf3a2	splicing factor 3a, subunit 2, 66kDa	0.549637	0.033227	0.091252
1392835_at	304337	Zfp68_prec	zinc finger protein 68 (predicted)	-0.84646	0.033232	0.091252
1368224_at	24795	Serpina3n	serine (or cysteine) peptidase inhibitor	1.763466	0.033247	0.091256
1373464_at	366968	Tbc1d22a_	TBC1 domain family, member 22a (pre	0.843124	0.033247	0.091256
1367494_at	299198	RGD13108	similar to CGI-35 protein (predicted)	-0.491535	0.033265	0.091286
1377643_at	303991	Hoxd10_pr	homeo box D10 (predicted)	1.561607	0.033275	0.091294
1380448_at	362766	Alkbh_prec	alkB, alkylation repair homolog (E. coli	0.768351	0.03329	0.091317
1399047_at	287635	Mrpl27_pre	mitochondrial ribosomal protein L27 (p	0.527345	0.033299	0.091322
1374428_at	296284	Kif3b_pred	kinesin family member 3B (predicted)	0.539923	0.033327	0.09138
1371657_at	308508	Uble1b	ubiquitin-like 1 (sentrin) activating enz	-0.603197	0.033407	0.09158
1385061_at	500053	NA	NA	-0.502431	0.033436	0.091633
1398646_at	500853	RGD15654	similar to DKFZP434I092 protein (prec	-1.091062	0.03344	0.091633
1390386_at	25402	Casp3	caspase 3, apoptosis related cysteine	-0.896182	0.033488	0.091745
1383151_at	246150	Akap9	A kinase (PRKA) anchor protein (yotia	-0.702678	0.033517	0.091801
1376166_at	361192	RGD13085	LOC361192 (predicted)	1.130707	0.033523	0.091801
1375634_at	299707	Ccdc53_pr	coiled-coil domain containing 53 (predi	-1.044677	0.033529	0.091801
1388661_at	289745	MGC94604	mitochondrial protein, 18 kDa	1.753589	0.033538	0.091806
1385128_at	498356	RGD15657	similar to MGC68837 protein (predicte	1.309539	0.033586	0.091919
1369950_at	94201	Cdk4	cyclin-dependent kinase 4	0.525378	0.0336	0.091937
1373530_at	25729	Ccne1	cyclin E	1.905811	0.033618	0.091968
1375341_at	362278	Kua_predic	Kua homolog (predicted)	1.072655	0.033641	0.092012
1383336_at	368070	LOC36807	NA	-1.136062	0.033654	0.09203
1371553_at	364656	Mrpl36_pre	mitochondrial ribosomal protein L36 (p	-0.543645	0.033687	0.092101
1392646_at	299714	Tmem16d_	transmembrane protein 16D (predicted	-1.689588	0.033764	0.092288
1369121_at	59304	Gdf9	growth differentiation factor 9	0.997237	0.03377	0.092288
1377988_at	362974	RGD13046	similar to CG9646-PA	3.021082	0.033781	0.092301
1375502_at	498858	RGD15619	similar to mutated in bladder cancer 1	2.736736	0.033806	0.092342
1374119_at	304815	Elf3	E74-like factor 3	0.98391	0.03381	0.092342
1371516_at	361070	Xpo7	exportin 7	-0.55314	0.033822	0.092355
1378578_at	501559	RGD15621	similar to chromosome X open reading	-0.570899	0.033846	0.092402
1395579_at	361667	Dhx32_pre	DEAH (Asp-Glu-Ala-His) box polypepti	1.386312	0.033862	0.092408
1398279_at	116469	Trpv5	transient receptor potential cation char	1.097446	0.033862	0.092408
1377353_a_at	287437	Tnfsf13	tumor necrosis factor (ligand) superfan	-0.985885	0.03388	0.092436
1373024_at	302290	Ap3s1_pre	adaptor-related protein complex 3, sigi	-0.543224	0.033887	0.092437
1384769_a_at	314119	RGD15633	similar to Zinc finger X-linked protein Z	-1.119891	0.0339	0.092452
1385208_at	309029	RGD13050	similar to RIKEN cDNA 2310057M21	0.64934	0.033912	0.092467
1377459_at	406866	Ly6g6e	lymphocyte antigen 6 complex, locus (	2.050819	0.033958	0.092562
1397462_at	305135	RGD15634	similar to T-cell activation leucine repe	1.705598	0.033961	0.092562
1367607_at	29445	Cox4i1	cytochrome c oxidase subunit IV isofor	0.5762	0.034016	0.092677
1393447_at	293047	Kif7_predic	kinesin family member 7 (predicted)	-1.285913	0.034029	0.092677
1386963_at	116717	Trip10	thyroid hormone receptor interactor 10	1.171493	0.034031	0.092677
1397825_at	368062	LOC36806	NA	1.274217	0.034031	0.092677

1393138_at	24518	Jund	Jun D proto-oncogene	-0.721902	0.034042	0.092689
1388906_at	498410	RGD1564!	similar to novel protein similar to Tensi	-0.483508	0.034179	0.093042
1392559_at	363009	LOC36300	NA	0.869761	0.034192	0.093059
1367889_at	171503	Camk1	calcium/calmodulin-dependent protein	0.808559	0.034223	0.093124
1379203_at	300947	Tcfdp2_pre	transcription factor Dp 2 (predicted)	-1.896847	0.034241	0.093153
1373885_at	300266	Cbx5_pred	chromobox homolog 5 (Drosophila HP	-0.621649	0.034275	0.093183
1375964_at	304429	Psph	phosphoserine phosphatase	-0.649934	0.034276	0.093183
1388629_at	301005	Impdh2	inosine 5-monophosphate dehydrogen	0.711342	0.034279	0.093183
1387717_at	85240	Plcb2	phospholipase C, beta 2	1.305467	0.034284	0.093183
1373989_at	363140	Rassf1	Ras association (RalGDS/AF-6) doma	-0.623451	0.034293	0.093183
1390003_at	361783	Zfp57	zinc finger protein 57	0.838092	0.034294	0.093183
1391297_at	314458	Rcor1_pre	REST corepressor 1 (predicted)	1.006028	0.034309	0.093193
1387323_at	25048	Klkb1	kallikrein B, plasma 1	-3.511256	0.034312	0.093193
1387852_at	25357	Thrsp	thyroid hormone responsive protein	2.154457	0.034346	0.093267
1389348_at	311670	Cstf1	cleavage stimulation factor, 3' pre-RN/	0.931263	0.034372	0.093317
1388625_at	290679	RGD13047	similar to RIKEN cDNA 4921521J11 (p	0.514105	0.034379	0.093317
1391536_at	362324	RGD13084	similar to cDNA sequence BC020002 (	-0.777006	0.034416	0.093378
1390569_at	307212	Cndp1	carnosine dipeptidase 1 (metallopeptic	-2.164013	0.034421	0.093378
1389055_at	298508	Ppie	peptidylprolyl isomerase E (cyclophilin	0.622154	0.034423	0.093378
1367757_at	54322	Cox6c	cytochrome c oxidase, subunit VIc	-0.51138	0.034505	0.093578
1367513_at	296279	Tm9sf4	transmembrane 9 superfamily protein r	0.526957	0.03451	0.093578
1372546_at	315994	Mapkapk3	mitogen-activated protein kinase-activa	3.380269	0.034534	0.093622
1372330_at	652955	LOC65295	goliath	-0.504356	0.034581	0.09373
1372710_at	29631	Bet1	blocked early in transport 1 homolog (S	-0.63199	0.034588	0.09373
1377644_at	291925	RGD13087	similar to RIKEN cDNA 4921524J17 (p	-0.865653	0.034598	0.093738
1372457_at	306487	Mtus1	mitochondrial tumor suppressor 1	-1.457081	0.034605	0.093738
1398891_at	297799	Mrpl15_pre	mitochondrial ribosomal protein L15 (p	0.641063	0.034629	0.093784
1368202_a_at	79128	Dab2	disabled homolog 2 (Drosophila)	-1.111474	0.03469	0.093931
1388863_at	298317	Ube2a	ubiquitin-conjugating enzyme E2A, RA	-0.58223	0.034703	0.093947
1368720_at	64206	Tdo2	tryptophan 2,3-dioxygenase	-5.04066	0.034711	0.093949
1368095_at	26956	Ak3	adenylate kinase 3	0.845691	0.034746	0.094012
1367779_at	117028	Bin1	bridging integrator 1	0.587221	0.034749	0.094012
1374508_at	296136	Nphp1_pre	nephronophthisis 1 (juvenile) homolog	0.552501	0.034758	0.094019
1388953_at	290556	Gnl3	guanine nucleotide binding protein-like	-0.65091	0.03477	0.094032
1374525_at	363239	Raph1_pre	Ras association (RalGDS/AF-6) and p	-1.119469	0.034801	0.094097
1388822_at	364981	Scoc	short coiled-coil protein	-0.526906	0.034838	0.094177
1398929_at	300069	Eif3s6ip	eukaryotic translation initiation factor 3	0.601297	0.034865	0.09423
1387746_at	56821	Olr414_pre	olfactory receptor 414 (predicted)	1.170484	0.034894	0.094291
1377252_at	287596	RGD13068	similar to RIKEN cDNA 1200011M11 (	0.593914	0.034918	0.094324
1370510_a_at	29657	Arntl	aryl hydrocarbon receptor nuclear tran	-0.787164	0.034921	0.094324
1376917_at	50552	Znf292	zinc finger protein 292	0.986375	0.034937	0.094348
1389249_at	293184	Sh2bp1	SH2 domain binding protein 1 (tetratric	-0.571014	0.034999	0.09449
1392364_at	361284	NA	NA	-1.189849	0.035008	0.09449
1383817_at	360987	NA	NA	-0.756767	0.035011	0.09449
1379228_at	499148	NA	NA	-1.109107	0.035045	0.094563
1390967_a_at	298688	RGD13068	similar to RIKEN cDNA 2410006F12	0.723471	0.035056	0.094575
1387314_at	64305	Sult1b1	sulfotransferase family 1B, member 1	3.917022	0.03509	0.094635
1368200_at	89808	Cx3cl1	chemokine (C-X3-C motif) ligand 1	-1.565625	0.035093	0.094635
1369102_at	25272	Mapk10	mitogen activated protein kinase 10	2.704694	0.035115	0.094674
1396173_at	361625	Nrip3_pred	nuclear receptor interacting protein 3 (	3.418387	0.035124	0.094681
1392210_at	361244	RGD13074	similar to mKIAA0319 protein (predicte	1.611286	0.035139	0.094701

1367824_at	25318	Fnta	farnesyltransferase, CAAX box, alpha	0.85585	0.035147	0.094702
1367486_at	362162	Zfp289	zinc finger protein 289	1.313247	0.035212	0.094859
1373426_at	116590	Mapk1	mitogen activated protein kinase 1	-0.682362	0.035287	0.095043
1388744_at	288532	Mcm7	minichromosome maintenance deficier	0.538741	0.035316	0.095101
1370535_at	116668	Myt1l	myelin transcription factor 1-like	-0.975308	0.035383	0.09526
1387415_a_at	81022	Stxbp5	syntaxin binding protein 5 (tomosyn)	0.907932	0.035393	0.09527
1387308_at	24335	Epo	erythropoietin	1.10042	0.035431	0.095351
1370221_at	65154	Wisp1	WNT1 inducible signaling pathway pro	2.064369	0.03553	0.095565
1398325_at	300733	Tspan3	tetraspanin 3	-0.601456	0.03553	0.095565
1369286_at	25268	Proc	protein C	-1.007371	0.035532	0.095565
1370238_at	171069	Usmg5	upregulated during skeletal muscle grc	-0.537827	0.035625	0.09576
1378181_at	291071	Rpp40	ribonuclease P 40 subunit (human)	0.698905	0.035626	0.09576
1394489_at	311936	Rnf32	ring finger protein 32	1.44714	0.035626	0.09576
1388716_at	361798	Ehmt2	euchromatic histone lysine N-methyltra	0.909797	0.035657	0.095824
1398308_at	296883	Rpa3_pred	replication protein A3 (predicted)	-0.765642	0.035702	0.095921
1386685_at	305940	Sacs_pred	sacsin (predicted)	1.002666	0.035708	0.095921
1389338_at	293114	RGD13083	hypothetical LOC293114 (predicted)	-0.544083	0.035722	0.09594
1382471_at	304735	RGD13103	similar to hypothetical protein MGC109	1.08748	0.035761	0.096022
1382269_at	294014	Cnnm2	cyclin M2	1.195358	0.035771	0.096022
1373877_at	89816	Picalm	phosphatidylinositol binding clathrin as	-0.479112	0.035774	0.096022
1394095_at	316687	RGD13074	similar to RIKEN cDNA 4930429M06R	-0.786868	0.035789	0.096043
1389112_at	364952	Nkd1_pred	naked cuticle 1 homolog (Drosophila) (	-1.559399	0.035825	0.096113
1370197_a_at	25522	Prkcz	protein kinase C, zeta	0.916374	0.03583	0.096113
1390997_at	64076	Slc26a1	solute carrier family 26 (sulfate transpo	-0.538291	0.035862	0.096181
1381986_at	685707	LOC68570	NA	-0.51962	0.03587	0.096181
1377463_at	308757	Man2a2_pı	mannosidase 2, alpha 2 (predicted)	-1.221005	0.03589	0.096215
1374500_at	499691	LOC49969	similar to sarcoma antigen NY-SAR-27	-0.735298	0.035929	0.096281
1372568_at	316021	Epm2aip1_	EPM2A (laforin) interacting protein 1 (p	-0.623504	0.035932	0.096281
1389432_at	406164	Pbx2	pre-B-cell leukemia transcription factor	-0.784307	0.035937	0.096281
1394639_at	502327	RGD15639	similar to CDC42 small effector 2 (prec	0.590895	0.035944	0.096281
1370581_at	63851	Fgf14	fibroblast growth factor 14	-1.080645	0.035958	0.096299
1373657_at	298091	Slc31a2	solute carrier family 31, member 2	-0.651717	0.036055	0.096541
1390189_at	298977	LOC29897	NA	-0.52572	0.036078	0.096581
1372076_at	295357	Hbxip_prec	hepatitis B virus x interacting protein (p	0.617473	0.036091	0.096581
1368335_at	25081	Apoa1	apolipoprotein A-I	-2.697676	0.036096	0.096581
1399057_at	300891	Morf4l1	mortality factor 4 like 1	0.631464	0.036099	0.096581
1367949_at	29237	Penk-rs	preproenkephalin, related sequence	-2.320898	0.036133	0.096652
1397739_at	361938	Pdzk6_pre	PDZ domain containing 6 (predicted)	1.254947	0.036159	0.096692
1387912_at	245957	Ddx46	DEAD (Asp-Glu-Ala-Asp) box polypept	0.545898	0.036164	0.096692
1383346_at	304719	RGD15603	similar to ERCC4_MOUSE (predicted)	1.045969	0.03617	0.096692
1385426_at	295462	RGD13053	similar to hypothetical protein FLJ2064	-1.847207	0.036186	0.096715
1373228_at	361165	RGD15607	similar to D8Ertd354e protein (predicte	-0.714267	0.036227	0.096794
1374768_at	362994	LOC36299	NA	-1.846261	0.03623	0.096794
1380031_at	171151	Slc25a21	solute carrier family 25 (mitochondrial (	1.370657	0.036246	0.096817
1393750_at	299002	RGD13102	similar to KIAA1333 protein (predicted)	0.871535	0.036256	0.096824
1377636_at	498208	NA	NA	0.642754	0.036269	0.096824
1371642_at	303831	Eif4a2	eukaryotic translation initiation factor 4	-0.683927	0.036271	0.096824
1388179_at	25366	Acvr2b	activin receptor IIB	0.891811	0.036312	0.096901
1376738_at	308708	Ttc23	tetratricopeptide repeat domain 23	-1.867203	0.036314	0.096901
1385035_at	360763	Usp12_pre	ubiquitin specific protease 12 (predicte	1.783791	0.036344	0.096963
1368409_at	29487	Gstt2	glutathione S-transferase, theta 2	1.157869	0.036372	0.097017

1397121_at	415057	RT1-T24-2	RT1 class I, T24, gene 2	1.814936	0.036382	0.097018
1372409_at	316237	Mad2l1bp	MAD2L1 binding protein	0.545718	0.036388	0.097018
1371912_at	362837	Ndufs7	NADH dehydrogenase (ubiquinone) Fe	0.638709	0.036395	0.097018
1394125_at	499291	RGD15658	RGD1565859 (predicted)	3.340402	0.036464	0.097177
1396198_at	309060	RGD13107	similar to RIKEN cDNA A930008G19 (	1.000735	0.036469	0.097177
1387038_at	84485	Ccs	copper chaperone for superoxide dism	0.599101	0.036515	0.097279
1378977_at	362575	Med8_prec	mediator of RNA polymerase II transcr	1.227622	0.036528	0.097295
1376970_at	361822	RGD13087	similar to RIKEN cDNA A130042E20; (	2.066344	0.036543	0.0973
1369839_at	60668	Amph1	amphiphysin 1	0.743447	0.036545	0.0973
1390016_at	298867	Kbtbd9_pre	kelch repeat and BTB (POZ) domain c	1.300272	0.036552	0.0973
1371322_at	117036	Lamc1	laminin, gamma 1	-0.909475	0.036589	0.09738
1392552_at	310614	Gatad2b	GATA zinc finger domain containing 2I	0.814835	0.036612	0.09742
1368441_at	60333	Msln	mesothelin	1.767321	0.036648	0.097497
1373813_at	295690	Dnajc10	DnaJ (Hsp40) homolog, subfamily C, r	0.766942	0.036687	0.097566
1391448_at	298744	Crim1_prec	cysteine-rich motor neuron 1 (predicte	-0.930334	0.036702	0.097566
1375415_at	366468	LOC36646	NA	0.567849	0.036702	0.097566
1370081_a_at	83785	Vegfa	vascular endothelial growth factor A	1.341066	0.036707	0.097566
1370066_at	117519	Keap1	Kelch-like ECH-associated protein 1	0.761677	0.036711	0.097566
1372797_at	302557	Pqbp1	polyglutamine binding protein 1	0.49258	0.036722	0.097574
1398872_at	161477	Rps13	ribosomal protein S13	-0.525697	0.036728	0.097574
1390010_at	313929	Ncoa1_pre	nuclear receptor coactivator 1 (predicte	-0.630195	0.036791	0.097722
1370107_at	117184	Ctrl	chymotrypsin-like	-1.485656	0.036804	0.097735
1389975_at	298359	RGD15619	similar to ELAV (embryonic lethal, abn	0.78942	0.036819	0.097756
1370566_at	299511	Rdh2	retinol dehydrogenase 2	2.873545	0.036872	0.097877
1369604_at	25443	Fgf10	fibroblast growth factor 10	2.035541	0.036898	0.097925
1383131_at	309684	Itgb2	integrin beta 2	-1.209584	0.036978	0.098119
1388651_at	289508	Rchy1	ring finger and CHY zinc finger domair	-0.588266	0.036998	0.098152
1377347_at	313554	Foxj3_prec	forkhead box J3 (predicted)	-0.569125	0.037012	0.09817
1395401_at	314622	Dos_predic	downstream of Stk11 (predicted)	0.857891	0.037044	0.098235
1382312_at	309728	Arid5b_pre	AT rich interactive domain 5B (Mrf1 like	3.056183	0.03706	0.098257
1371010_at	25628	Titf1	thyroid transcription factor 1	-1.967154	0.037077	0.098284
1377647_at	362581	Scmh1_pre	sex comb on midleg homolog 1 (predic	0.751096	0.03709	0.098298
1387559_at	170796	Grin3b	glutamate receptor, ionotropic, N-meth	1.988814	0.037144	0.098422
1389165_at	498301	RGD15655	similar to myocardial ischemic precond	-0.742193	0.037158	0.09844
1395127_at	361931	RGD13099	similar to RIKEN cDNA 4932438A13 (p	-0.990136	0.03718	0.098477
1372878_at	365703	Zfr	zinc finger RNA binding protein	-0.629422	0.03719	0.098485
1390739_at	315766	LOC31576	NA	1.264744	0.03723	0.098571
1378275_at	361176	Upf3a	UPF3 regulator of nonsense transcript	-0.505532	0.037242	0.098583
1389374_at	307644	Kifc3	kinesin family member C3	-1.335617	0.037257	0.098603
1379377_at	362004	Gdap2	ganglioside-induced differentiation-ass	0.807186	0.037265	0.098604
1388160_a_at	94173	Idh3B	isocitrate dehydrogenase 3 (NAD+) be	0.515454	0.037318	0.098725
1391498_at	363067	Sin3a_prec	transcriptional regulator, SIN3A (yeast	-0.754316	0.037357	0.098808
1371661_at	360594	Mrps23_pr	mitochondrial ribosomal protein S23 (p	0.659875	0.037441	0.099002
1367850_at	116591	Fcgr3	Fc receptor, IgG, low affinity III	-1.719002	0.037445	0.099002
1397718_at	308742	RGD15629	similar to multiple C2-domains with two	2.641975	0.037464	0.099032
1397176_at	290564	Mettl6	methyltransferase like 6	0.478275	0.037498	0.099086
1398710_at	310848	Cyp2u1	cytochrome P450, family 2, subfamily u	-0.984735	0.0375	0.099086
1387675_at	25619	Plau	plasminogen activator, urokinase	-3.610148	0.03754	0.099172
1382068_at	499016	RGD15644	similar to tubby super-family protein (p	0.545644	0.037587	0.099272
1370267_at	84027	Gsk3b	glycogen synthase kinase 3 beta	0.791719	0.037592	0.099272
1371921_at	307505	Catna1	catenin (cadherin-associated protein),	0.533557	0.037649	0.099402

1367763_at	25014	Acat1	acetyl-coenzyme A acetyltransferase 1	0.56941	0.037701	0.099494
1374895_at	316740	Myom1	myomesin 1 (skelemin) 185kDa	3.34641	0.037708	0.099494
1370895_at	85250	Col5a2	procollagen, type V, alpha 2	-1.780481	0.037708	0.099494
1396733_at	309142	Mtl5	metallothionein-like 5, testis-specific (t	2.689508	0.037714	0.099494
1387046_at	116475	Slb	selective LIM binding factor, rat homol	-0.92875	0.037743	0.099547
1369204_at	25734	Hck	hemopoietic cell kinase	0.849258	0.03775	0.099547
1368737_at	64675	Smr2	SMR2	1.077316	0.037757	0.099547
1373548_at	303602	Wdr68_pre	WD repeat domain 68 (predicted)	-0.482173	0.037772	0.099568
1388102_at	192227	Ltb4dh	leukotriene B4 12-hydroxydehydrogen	-1.284418	0.03782	0.099674
1371828_at	288667	RGD13108	similar to RIKEN cDNA 1500011H22	-0.540963	0.037903	0.099873
1374288_at	303608	Ftsj3	FtsJ homolog 3 (E. coli)	1.00281	0.037917	0.099874
1393601_at	300034	Tigd5_prec	tigger transposable element derived 5	0.694749	0.037919	0.099874

Differential Expression Analysis: Beta cells vs INS cells

probes	entrez_ger	symbols	gene_names	logFC	P.Value	adj.P.Val
1388185_at	24708	Rb1	retinoblastoma 1	6.407055	1.01E-06	0.005579
1383075_at	58919	Ccnd1	cyclin D1	6.44988	1.73E-06	0.005579
1370080_at	24451	Hmox1	heme oxygenase (decycling) 1	6.161488	2.15E-06	0.005579
1377029_at	300807	Rora_predi	RAR-related orphan receptor alpha (pr	8.600494	2.62E-06	0.005579
1387970_at	192208	Slc38a5	solute carrier family 38, member 5	5.813295	2.65E-06	0.005579
1390434_at	246756	Tradd	TNFRSF1A-associated via death doma	7.278305	2.75E-06	0.005579
1372389_at	494344	Ier2	immediate early response 2	5.826848	3.27E-06	0.005579
1369196_at	24677	Ppy	pancreatic polypeptide	8.995082	3.37E-06	0.005579
1372914_at	297604	Ltbr	lymphotoxin B receptor	6.557371	3.85E-06	0.005667
1367612_at	171341	Mgst1	microsomal glutathione S-transferase	-5.092842	5.07E-06	0.005789
1376843_at	140590	Bmpr2	bone morphogenic protein receptor, ty	8.268643	5.50E-06	0.005789
1388236_x_at	309600	RT1-CE12	RT1 class I, CE12	5.337681	6.04E-06	0.005789
1384308_at	305535	Meis1_pre	Meis1, myeloid ecotropic viral integrati	4.106035	6.70E-06	0.005789
1388122_at	29438	Gstp2	glutathione S-transferase, pi 2	7.187116	7.32E-06	0.005789
1387843_at	24373	Fst	follistatin	4.792818	7.70E-06	0.005789
1371447_at	360914	Plac8_prec	placenta-specific 8 (predicted)	-8.698625	8.53E-06	0.005789
1375961_at	295691	Frzb	frizzled-related protein	5.40334	8.80E-06	0.005789
1369502_a_at	497039	Amy2	NA	11.60598	9.24E-06	0.005789
1370907_at	25197	St6gal1	beta galactoside alpha 2,6 sialyltransfe	4.982231	9.42E-06	0.005789
1396556_at	498438	LOC49843	NA	8.902662	9.67E-06	0.005789
1367776_at	54237	Cdc2a	cell division cycle 2 homolog A (S. pon	-9.032922	9.73E-06	0.005789
1376184_at	300018	Lynx1_pre	Ly6/neurotoxin 1 (predicted)	-4.248362	1.02E-05	0.005789
1368536_at	84050	Enpp2	ectonucleotide pyrophosphatase/phos	-7.454566	1.29E-05	0.005789
1388223_at	286924	Gnat3	guanine nucleotide binding protein, alp	-5.844666	1.31E-05	0.005789
1376845_at	299269	isg12(b)	putative ISG12(b) protein	7.734309	1.31E-05	0.005789
1392512_at	64646	H2a	histone 2a	-3.668773	1.34E-05	0.005789
1368505_at	29480	Rgs4	regulator of G-protein signaling 4	10.17237	1.37E-05	0.005789
1397856_at	361974	Pet112l_pr	PET112-like (yeast) (predicted)	-4.556404	1.41E-05	0.005789
1372507_at	306587	Tcta	T-cell leukemia translocation altered g	4.441309	1.45E-05	0.005789
1370235_at	25045	Dbi	diazepam binding inhibitor	-3.321602	1.45E-05	0.005789
1369999_a_at	94270	Nnat	neuronatin	-4.036755	1.49E-05	0.005789
1374784_at	291355	Prtfdc1_pre	phosphoribosyl transferase domain co	-4.257536	1.53E-05	0.005789
1367762_at	24797	Sst	somatostatin	7.765948	1.55E-05	0.005789
1388484_at	296368	Ube2c_pre	ubiquitin-conjugating enzyme E2C (pre	-4.567421	1.60E-05	0.005789
1369018_at	58921	Foxm1	forkhead box M1	-4.866929	1.63E-05	0.005789

1368650_at	81813	Klf10	Kruppel-like factor 10	-3.431255	1.74E-05	0.005789
1378665_at	306091	Pcdh9_pre	protocadherin 9 (predicted)	4.680907	1.79E-05	0.005789
1368247_at	24472	Hspa1a	heat shock 70kD protein 1A	8.389674	1.81E-05	0.005789
1368979_at	84009	Kalrn	kalirin, RhoGEF kinase	-3.440137	1.91E-05	0.005789
1375992_at	499765	RGD15641	similar to FLJ46082 protein (predicted)	-3.393747	1.98E-05	0.005789
1395299_at	287675	Ka24	type I keratin KA24	4.303254	2.00E-05	0.005789
1383940_at	304951	Cdca1	cell division cycle associated 1	-4.336643	2.01E-05	0.005789
1393386_at	287346	RGD15619	similar to novel protein (predicted)	-5.836708	2.07E-05	0.005789
1390789_at	315973	Acad11_pr	acyl-Coenzyme A dehydrogenase fam	6.2649	2.08E-05	0.005789
1387599_a_at	24314	Nqo1	NAD(P)H dehydrogenase, quinone 1	4.777898	2.19E-05	0.005789
1376369_at	300219	Troap_pred	trophinin associated protein (tastin) (pr	-5.26993	2.20E-05	0.005789
1398317_at	64473	Bpnt1	bisphosphate 3'-nucleotidase 1	5.001846	2.25E-05	0.005789
1377011_at	304244	RGD13070	similar to hypothetical protein CG003 (	4.656085	2.27E-05	0.005789
1379656_a_at	313235	Coro2a	coronin, actin binding protein 2A	-3.446887	2.31E-05	0.005789
1370088_at	85244	Spa17	sperm autoantigenic protein 17	-3.391718	2.34E-05	0.005789
1393907_at	287730	Pyy_mappe	peptide YY (mapped)	8.362854	2.35E-05	0.005789
1387818_at	114555	Casp4	caspase 4, apoptosis-related cysteine	4.872218	2.37E-05	0.005789
1368260_at	114592	Aurkb	aurora kinase B	-3.465304	2.39E-05	0.005789
1390003_at	361783	Zfp57	zinc finger protein 57	5.110578	2.39E-05	0.005789
1393346_at	499518	RGD15616	similar to RIKEN cDNA 5830436D01 (p	4.136866	2.47E-05	0.005789
1387116_at	24908	Dnajb9	DnaJ (Hsp40) homolog, subfamily B, m	3.241868	2.48E-05	0.005789
1370042_at	84510	Stmn2	stathmin-like 2	-4.009463	2.49E-05	0.005789
1374771_at	303019	RGD15649	similar to neuralized homolog (predicte	-3.997138	2.56E-05	0.00584
1383581_at	499752	RGD15655	similar to 60S ribosomal protein L7a (p	-4.360118	2.72E-05	0.006072
1373329_at	156435	Tmprss2	transmembrane protease, serine 2	5.525012	2.76E-05	0.006072
1367646_at	64529	Ctsb	cathepsin B	2.865604	2.80E-05	0.006072
1368187_at	113955	Gpnmb	glycoprotein (transmembrane) nmb	5.411627	2.88E-05	0.00613
1371442_at	192235	Hyou1	hypoxia up-regulated 1	4.544492	2.93E-05	0.00613
1394935_at	313024	Wasf2	WAS protein family, member 2	4.814552	3.00E-05	0.00613
1372654_at	361674	Eps8l2_pre	EPS8-like 2 (predicted)	3.989357	3.01E-05	0.00613
1379868_at	310644	Pbxip1	pre-B-cell leukemia transcription factor	6.037595	3.14E-05	0.006189
1379544_at	301447	RGD13113	similar to A530083I02Rik protein (pred	3.349913	3.15E-05	0.006189
1368399_a_at	58952	Pgcp	plasma glutamate carboxypeptidase	6.473558	3.18E-05	0.006189
1396238_at	313878	Galnt14	UDP-N-acetyl-alpha-D-galactosamine:	-3.523091	3.31E-05	0.006356
1368512_a_at	64017	Enpep	glutamyl aminopeptidase	5.376179	3.47E-05	0.006365
1391812_at	315979	RGD13091	similar to RIKEN cDNA E330026B02 (	3.69729	3.51E-05	0.006365
1392957_at	361877	RGD15629	similar to Single-stranded DNA-binding	-2.872597	3.56E-05	0.006365
1369156_at	79209	Frk	fyn-related kinase	3.46273	3.57E-05	0.006365
1367780_at	64193	Pttg1	pituitary tumor-transforming 1	-6.256848	3.60E-05	0.006365
1369787_at	24889	Cckar	cholecystokinin A receptor	3.972483	3.69E-05	0.006365
1377952_at	315722	Adpgk	ADP-dependent glucokinase	2.767474	3.76E-05	0.006365
1372478_at	501065	LOC50106	NA	3.394364	3.76E-05	0.006365
1369944_at	81520	Marcksl1	MARCKS-like 1	-3.680174	3.79E-05	0.006365
1389408_at	362720	Rrm2_map	ribonucleotide reductase M2 (mapped)	-7.322147	3.84E-05	0.006365
1370530_a_at	25096	Pld1	phospholipase D1	6.294391	3.96E-05	0.006365
1384762_at	363174	LOC36317	NA	-6.059282	3.96E-05	0.006365
1387122_at	25157	Plagl1	pleiomorphic adenoma gene-like 1	9.108931	3.96E-05	0.006365
1367784_a_at	24854	Clu	clusterin	3.589402	3.99E-05	0.006365
1371862_at	365320	Rrm1_map	ribonucleotide reductase M1 (mapped)	-2.765841	4.33E-05	0.006638
1395416_at	296372	Ncoa5_pre	nuclear receptor coactivator 5 (predicte	-5.179002	4.40E-05	0.006638
1367568_a_at	25333	Mgp	matrix Gla protein	6.464485	4.42E-05	0.006638

1372702_at	287750	PRP-2	proline-rich protein	2.953603	4.52E-05	0.006638
1370073_at	63880	Dnajc3	DnaJ (Hsp40) homolog, subfamily C, r	3.46806	4.67E-05	0.006638
1381100_at	367072	Arhgef12	Rho guanine nucleotide exchange fact	2.668653	4.67E-05	0.006638
1378264_at	298441	Nasp	nuclear autoantigenic sperm protein (h	-2.760174	4.68E-05	0.006638
1372406_at	316273	Mcm3_pred	minichromosome maintenance deficier	-3.543062	4.79E-05	0.006638
1377208_at	290494	Mbnl2_pred	muscleblind-like 2 (predicted)	3.515139	4.89E-05	0.006638
1372059_at	288176	RGD13094	similar to RIKEN cDNA 2610528E23	-3.173318	4.91E-05	0.006638
1391714_at	297804	Plag1	pleiomorphic adenoma gene 1	4.164507	4.93E-05	0.006638
1399027_at	117273	Rhoa	ras homolog gene family, member A	5.88251	4.94E-05	0.006638
1374838_at	316580	Sp140	SP140 nuclear body protein	2.73404	4.95E-05	0.006638
1374344_at	314614	Prg-2	plasticity-related protein PRG-2	-5.296426	5.00E-05	0.006638
1372383_at	246254	Gpsm1	G-protein signalling modulator 1 (AGS	3.06799	5.11E-05	0.006638
1370594_at	302822	Igsf1	immunoglobulin superfamily, member	-3.284636	5.23E-05	0.006638
1392899_at	308761	Prc1_predi	protein regulator of cytokinesis 1 (pred	-4.790561	5.35E-05	0.006638
1370942_at	29372	Rasa3	RAS p21 protein activator 3	-4.505775	5.41E-05	0.006638
1371131_a_at	117514	Txnip	upregulated by 1,25-dihydroxyvitamin	-2.861551	5.44E-05	0.006638
1369577_at	84607	Socs2	suppressor of cytokine signaling 2	-3.323094	5.50E-05	0.006638
1373915_at	308405	Dmpk_pred	dystrophia myotonica-protein kinase (p	2.664789	5.52E-05	0.006638
1384834_at	305497	Cobl_predi	cordon-bleu (predicted)	-5.611808	5.54E-05	0.006638
1382351_at	297902	Gem_predi	GTP binding protein (gene overexpres	2.708465	5.56E-05	0.006638
1382056_at	502603	LOC50260	similar to splicing factor p54	2.503421	5.57E-05	0.006638
1373897_at	116685	Lmnb1	lamin B1	-3.317964	5.60E-05	0.006638
1381388_at	304984	Ccdc19	coiled-coil domain containing 19	3.737517	5.62E-05	0.006638
1394022_at	291023	Id4	inhibitor of DNA binding 4	-4.846062	5.62E-05	0.006638
1378240_at	312641	Fancd2	Fanconi anemia D2 protein	-4.991832	5.68E-05	0.006638
1370898_at	29140	Snn	stannin	-2.659624	5.70E-05	0.006638
1372715_at	364678	Sfxn1	sideroflexin 1	7.217156	5.72E-05	0.006638
1370894_at	65132	Cldn7	claudin 7	6.053275	5.73E-05	0.006638
1373556_at	298861	RGD15617	similar to CG14903-PA (predicted)	-2.758528	5.84E-05	0.006638
1387891_at	85274	Prdx4	peroxiredoxin 4	-2.931655	5.88E-05	0.006638
1380009_at	362779	Vrk1	vaccinia related kinase 1	-3.040021	5.89E-05	0.006638
1388596_at	361422	Cotl1_pred	coactosin-like 1 (Dictyostelium) (predic	2.710935	5.92E-05	0.006638
1392865_at	25444	Fgf9	fibroblast growth factor 9	4.444131	5.99E-05	0.00666
1394419_at	296060	RGD13091	similar to RIKEN cDNA 6530401L14 g	-5.22547	6.12E-05	0.006748
1388155_at	294853	Krt1-18	keratin complex 1, acidic, gene 18	4.987927	6.18E-05	0.006758
1373570_at	311671	Npepl1_pre	aminopeptidase-like 1 (predicted)	6.275931	6.33E-05	0.006778
1372042_at	291813	Cmtm3_pre	CKLF-like MARVEL transmembrane d	3.086306	6.33E-05	0.006778
1382521_at	24398	Gls	glutaminase	-2.933247	6.36E-05	0.006778
1372324_at	497975	LOC49797	NA	-2.731403	6.44E-05	0.006778
1373250_at	353229	Tmem23	transmembrane protein 23	-3.786562	6.49E-05	0.006778
1388827_at	289784	H2afv_pred	H2A histone family, member V (predict	-2.624978	6.55E-05	0.006778
1386912_at	29569	Pcolce	procollagen C-endopeptidase enhance	4.800547	6.57E-05	0.006778
1390137_at	311325	Traf4af1	TRAF4 associated factor 1	-7.042743	6.62E-05	0.006778
1368404_at	81653	Dbn1	drebrin 1	-4.03486	6.66E-05	0.006778
1375043_at	314322	Fos	FBJ murine osteosarcoma viral oncoge	5.254735	6.71E-05	0.006779
1374389_at	25202	Gucy1b3	guanylate cyclase 1, soluble, beta 3	-3.537931	6.78E-05	0.006797
1387042_at	25297	Cacnb3	calcium channel, voltage-dependent, b	-2.407696	6.93E-05	0.006842
1367755_at	81718	Cdo1	cysteine dioxygenase 1, cytosolic	3.749432	6.94E-05	0.006842
1388340_at	300795	Ns5atp9	NS5A (hepatitis C virus) transactivated	-4.832383	6.98E-05	0.006842
1388071_x_at	24737	RT1-Aw2	RT1 class Ib, locus Aw2	3.422113	7.16E-05	0.006897
1367799_at	24799	Eef1a2	eukaryotic translation elongation factor	-3.69844	7.22E-05	0.006897

1376163_at	309082	Tmem12	transmembrane protein 12	4.027655	7.23E-05	0.006897
1375877_at	64440	Syt4	synaptotagmin IV	2.619187	7.24E-05	0.006897
1368839_at	83725	Wfs1	Wolfram syndrome 1 homolog (human	2.41825	7.35E-05	0.00691
1398965_at	316052	LOC31605	eomesodermin	2.41608	7.46E-05	0.00691
1389566_at	363088	Ccnb2	cyclin B2	-4.409234	7.47E-05	0.00691
1389562_at	291423	Setbp1_pre	SET binding protein 1 (predicted)	-2.59957	7.49E-05	0.00691
1381184_at	171563	Nav2	neuron navigator 2	4.691339	7.52E-05	0.00691
1369943_at	56083	Tgm2	transglutaminase 2, C polypeptide	-2.737004	7.73E-05	0.007058
1398255_at	60577	Slc15a2	solute carrier family 15 (H+/peptide tra	-4.981979	7.86E-05	0.00713
1367845_at	24588	Nef3	neurofilament 3, medium	3.315192	7.96E-05	0.007165
1398892_at	286898	Npc2	Niemann Pick type C2	2.52329	8.13E-05	0.007249
1388324_at	289222	Nit1	nitrilase 1	2.332577	8.16E-05	0.007249
1376567_at	304323	Ftsj2_predi	FtsJ homolog 2 (E. coli) (predicted)	-2.868003	8.29E-05	0.00725
1388135_at	59102	Rpa2	replication protein A2	-3.044352	8.33E-05	0.00725
1378856_at	501628	RGD15625	similar to transcription elongation facto	-4.777386	8.34E-05	0.00725
1370089_at	83516	Ppargc1a	peroxisome proliferative activated rece	2.272471	8.38E-05	0.00725
1367986_at	29602	Ptgfrn	prostaglandin F2 receptor negative reg	-3.05593	8.62E-05	0.007395
1398431_at	297814	Car8	carbonic anhydrase 8	-3.999765	8.66E-05	0.007395
1379663_at	298267	Cachd1_pr	cache domain containing 1 (predicted)	-2.2449	8.93E-05	0.007551
1393759_at	501191	NA	NA	3.394057	8.99E-05	0.007551
1368585_at	29131	Cart	cocaine and amphetamine regulated tr	5.787794	9.02E-05	0.007551
1387914_at	301517	Cyp27a1	cytochrome P450, family 27, subfamily	2.949238	9.19E-05	0.007551
1376762_at	361659	RGD15641	similar to Pleckstrin homology domain-	-2.879678	9.21E-05	0.007551
1368826_at	24267	Comt	catechol-O-methyltransferase	3.452089	9.43E-05	0.007551
1383455_at	289352	Eprs	glutamyl-prolyl-tRNA synthetase	2.78681	9.49E-05	0.007551
1387022_at	24188	Aldh1a1	aldehyde dehydrogenase family 1, me	-3.28828	9.56E-05	0.007551
1398302_at	64361	Prlpf	prolactin-like protein F	2.735039	9.56E-05	0.007551
1370462_at	25460	Hmmr	hyaluronan mediated motility receptor	-3.985415	9.57E-05	0.007551
1389755_at	619566	LOC61956	similar to transcription factor RAM2	-3.113331	9.60E-05	0.007551
1367559_at	29292	Ftl1	ferritin light chain 1	2.609601	9.60E-05	0.007551
1389573_at	362196	Chac1_pre	ChaC, cation transport regulator-like 1	3.246609	9.61E-05	0.007551
1373561_at	316533	D1bwg136	chondroitin polymerizing factor	2.657261	9.64E-05	0.007551
1377761_at	360518	Gfpt2	glutamine-fructose-6-phosphate transa	3.665317	9.74E-05	0.007588
1388458_at	288003	Rfc4_predi	replication factor C (activator 1) 4 (pre	-3.863031	0.0001	0.007688
1389858_at	24834	Tk1	thymidine kinase 1	-3.972902	0.0001	0.007688
1370346_at	25203	Ccnb1	cyclin B1	-4.537783	0.000101	0.007688
1379594_at	308923	Ahi1	Abelson helper integration site 1	3.019	0.000101	0.007688
1390219_at	305349	Wdr19_pre	WD repeat domain 19 (predicted)	-2.398673	0.000102	0.007721
1386762_at	310097	Snag1_pre	sorting nexin associated golgi protein 1	-2.672521	0.000103	0.007723
1391856_at	290562	Sema3g	sema domain, immunoglobulin domair	-3.219756	0.000104	0.007758
1368028_at	24688	Prph1	peripherin 1	-8.581115	0.000104	0.007758
1386857_at	29332	Stmn1	stathmin 1	-4.390145	0.000106	0.007793
1377867_at	313837	RGD15622	similar to Glutaminyl-peptide cyclotran	-2.706022	0.000106	0.007793
1394097_at	65023	Kcnip1	Kv channel-interacting protein 1	-2.840366	0.000107	0.0078
1370845_at	64467	Entpd2	ectonucleoside triphosphate diphosph	2.611399	0.000109	0.007904
1387683_at	58959	Crhr1	corticotropin releasing hormone recept	-6.181389	0.00011	0.007924
1379319_at	502320	RGD15647	similar to RIKEN cDNA C230052I12 (p	-2.80455	0.000111	0.007924
1372601_at	282840	Atf5	activating transcription factor 5	3.296848	0.000111	0.007924
1389668_at	295661	Spbc25	spindle pole body component 25 homo	-7.162011	0.000112	0.007941
1387342_at	79218	Gng5	guanine nucleotide binding protein (G	2.324286	0.000113	0.007941
1372006_at	290350	Loxl2_pred	lysyl oxidase-like 2 (predicted)	2.644741	0.000113	0.007941

1388006_at	207126	Muc13	mucin 13, epithelial transmembrane	-2.728861	0.000114	0.007941
1368441_at	60333	Msln	mesothelin	7.681738	0.000114	0.007941
1390891_at	499350	NA	NA	-4.36199	0.000115	0.007941
1391071_at	499513	NA	NA	-2.121739	0.000116	0.007941
1390502_at	289084	RGD13091	similar to RIKEN cDNA 1700025G04 g	-3.180857	0.000116	0.007941
1389607_at	305811	Rcor2	REST corepressor 2	-3.432906	0.000118	0.007999
1370885_at	252929	Ctsz	cathepsin Z	2.662156	0.000119	0.007999
1377572_at	361091	RGD15635	similar to UDP-glucose ceramide gluco	3.151371	0.000119	0.007999
1385308_at	363261	Mogat1_pr	monoacylglycerol O-acyltransferase 1	2.914648	0.000119	0.007999
1387145_at	29584	Gjb1	gap junction membrane channel protei	3.968881	0.000123	0.008088
1392905_at	80850	Gng2	guanine nucleotide binding protein, ga	4.095204	0.000123	0.008088
1383976_at	362366	Fkbp14	FK506 binding protein 14	2.83989	0.000123	0.008088
1377417_at	363328	LOC36332	NA	2.156865	0.000123	0.008088
1383615_a_at	362736	RGD15616	similar to HECT domain containing 1 (p	2.37054	0.000124	0.008088
1370497_at	353498	Cyp11b3	cytochrome P450, subfamily 11B, poly	3.470508	0.000125	0.008088
1374806_at	313017	Sfn_predic	stratifin (predicted)	4.624243	0.000125	0.008088
1382291_at	363698	NA	NA	-2.808008	0.000125	0.008088
1368124_at	171109	Dusp5	dual specificity phosphatase 5	3.494575	0.000127	0.008185
1367849_at	25216	Sdc1	syndecan 1	-2.79898	0.000131	0.008323
1389326_at	288414	Rfc3	replication factor C (activator 1) 3	-2.366354	0.000131	0.008323
1370282_at	29317	Csrp2	cysteine and glycine-rich protein 2	-3.176201	0.000135	0.00851
1367939_at	25056	Rbp1	retinol binding protein 1, cellular	2.056973	0.000136	0.00851
1370008_at	140938	Psmc3ip	proteasome (prosome, macropain) 26S	-2.039776	0.000136	0.00851
1387817_at	25247	Nsg1	neuron specific gene family member 1	-2.128457	0.000136	0.00851
1370294_a_at	64515	Cdc20	cell division cycle 20 homolog (S. cere	-4.194568	0.000139	0.008583
1371988_at	294410	Man1a_pre	mannosidase 1, alpha (predicted)	4.568719	0.000141	0.008583
1375052_at	501015	LOC50101	NA	4.82464	0.000141	0.008583
1370912_at	294254	Hspa1b_m	heat shock 70kD protein 1B (mapped)	5.659719	0.000141	0.008583
1372886_at	360962	Tacc3	transforming, acidic coiled-coil contain	-4.333223	0.000141	0.008583
1372929_at	83731	Kcnma1	potassium large conductance calcium-	2.125837	0.000141	0.008583
1367960_at	29308	Arl4a	ADP-ribosylation factor-like 4A	2.009511	0.000143	0.008583
1385001_at	315084	Gsdmdc1_	gasdermin domain containing 1 (predic	3.708303	0.000143	0.008583
1393987_s_at	59076	Gprk6	G protein-coupled receptor kinase 6	-2.230453	0.000143	0.008583
1398260_a_at	79224	Serpind1	serine (or cysteine) peptidase inhibitor	4.330031	0.000144	0.008606
1398616_at	302671	Ap1s2_pre	adaptor-related protein complex 1, sigr	-2.756911	0.000146	0.00864
1371887_at	305373	RGD15644	similar to high mobility group protein ho	-2.627174	0.000147	0.008681
1372343_at	295050	Exosc8_pr	exosome component 8 (predicted)	-2.477561	0.000148	0.008681
1367465_at	192275	Dad1	defender against cell death 1	2.015562	0.000149	0.008681
1382882_x_at	500720	NA	NA	11.6046	0.000149	0.008681
1381833_at	305984	Cdca2	cell division cycle associated 2	-5.775335	0.00015	0.008681
1370333_a_at	24482	Igf1	insulin-like growth factor 1	-5.722317	0.000151	0.008714
1377314_at	297417	Gfpt1	glutamine fructose-6-phosphate transa	2.897457	0.000154	0.00882
1367652_at	24484	Igfbp3	insulin-like growth factor binding protei	-3.70294	0.000154	0.00882
1396947_at	286994	Lgr4	leucine-rich repeat-containing G protei	3.547336	0.000156	0.00882
1367905_at	54410	Enpp3	ectonucleotide pyrophosphatase/phosp	-2.657908	0.000157	0.00882
1378220_at	497860	LOC49786	similar to RIKEN cDNA 4930517K11	2.266763	0.000158	0.00882
1396820_at	297893	Hdac1_pre	histone deacetylase 1 (predicted)	-2.632529	0.00016	0.00882
1369964_at	155151	Coro1a	coronin, actin binding protein 1A	-5.959406	0.000161	0.00882
1370910_at	116468	Rfc2	replication factor C (activator 1) 2	-1.987104	0.000161	0.00882
1382855_at	311715	RGD13058	similar to Protein C20orf158 (predicted	4.702255	0.000161	0.00882
1391840_at	316238	RGD13106	similar to RIKEN cDNA 1700027N10 (p	-2.504022	0.000162	0.00882

1398959_at	25695	Cebpd	CCAAT/enhancer binding protein (C/E	2.035346	0.000162	0.00882
1371646_at	362660	Pgd_mapp	phosphogluconate dehydrogenase (ma	1.918488	0.000163	0.00882
1367617_at	24189	Aldoa	aldolase A	2.34791	0.000164	0.00882
1369903_at	24922	Gabrb3	gamma-aminobutyric acid (GABA-A) re	-1.957026	0.000164	0.00882
1391229_at	171358	Camk1g	calcium/calmodulin-dependent protein	2.608357	0.000165	0.00882
1397227_at	308134	Tmem35	transmembrane protein 35	4.060709	0.000165	0.00882
1368656_at	116635	Scg3	secretogranin III	1.953119	0.000166	0.00882
1392477_at	362733	Etv1_predi	ets variant gene 1 (predicted)	-2.977991	0.000166	0.00882
1372999_at	315405	Dcun1d5	DCN1, defective in cullin neddylation 1	9.833377	0.000166	0.00882
1386943_at	64364	Pllp	plasma membrane proteolipid	-3.497924	0.000166	0.00882
1369489_at	29427	Aif1	allograft inflammatory factor 1	3.071063	0.000167	0.00882
1367551_a_at	360768	RGD13064	similar to CG14980-PB	-1.928655	0.000168	0.008865
1387472_at	25710	Cd3d	CD3 antigen delta polypeptide	4.56705	0.000169	0.008874
1368651_at	24651	Pklr	pyruvate kinase, liver and red blood ce	-2.984777	0.00017	0.008883
1397676_at	362447	RGD15631	similar to osteoclast inhibitory lectin (pr	3.3555	0.000172	0.008935
1367676_at	29395	Hmgb2	high mobility group box 2	-3.439889	0.000173	0.008935
1387180_at	117022	Il1r2	interleukin 1 receptor, type II	-2.280852	0.000174	0.008935
1371036_at	497815	Nrcam	neuron-glia-CAM-related cell adhesion	2.021462	0.000174	0.008935
1383697_at	114507	Slc5a3	solute carrier family 5 (inositol transpor	2.605125	0.000174	0.008935
1369208_at	25647	Il7	interleukin 7	3.741015	0.000176	0.008935
1373333_at	317409	MGC1093	similar to Microsomal signal peptidase	-3.836137	0.000176	0.008935
1375026_at	315746	Cln6_predi	ceroid-lipofuscinosis, neuronal 6 (pred	-4.703214	0.000176	0.008935
1391049_at	306720	Ctsm	cathepsin M	2.857742	0.000178	0.008991
1368407_at	64537	Hpse	heparanase	-4.762754	0.000179	0.008991
1388506_at	306871	Dsp	desmoplakin	4.921094	0.000179	0.008991
1379164_at	316524	Znf142_pre	zinc finger protein 142 (clone pHZ-49)	2.043507	0.000182	0.009019
1398859_at	64474	Hdlbp	high density lipoprotein binding protein	2.616514	0.000184	0.009019
1394530_at	301004	RGD1311C	similar to hypothetical protein FLJ2025	2.747003	0.000184	0.009019
1368209_at	81916	Pdzk1ip1	PDZK1 interacting protein 1	-4.917811	0.000185	0.009019
1383906_at	316326	LOC31632	similar to lung inducible neuralized-rela	3.410129	0.000185	0.009019
1367899_at	25439	F2r	coagulation factor II (thrombin) recepto	-2.582226	0.000185	0.009019
1372485_at	29700	Pcbd1	pterin 4 alpha carbinolamine dehydrata	-2.092937	0.000186	0.009019
1392219_at	50659	Nr2c2	nuclear receptor subfamily 2, group C,	1.924267	0.000186	0.009019
1396830_at	499979	RGD15621	RGD1562135 (predicted)	2.206311	0.000187	0.009019
1377379_at	364081	Irf6_predic	interferon regulatory factor 6 (predicted	2.194479	0.000187	0.009019
1376791_at	497903	RGD15646	similar to hypothetical protein A830006	3.675642	0.000187	0.009019
1395845_at	312777	LOC31277	similar to TEL protein	5.527537	0.000189	0.009059
1395318_at	680231	LOC68023	NA	2.203003	0.00019	0.009078
1393263_at	362256	RGD15639	RGD1563912 (predicted)	8.257365	0.000192	0.009125
1369203_at	114557	Wif1	Wnt inhibitory factor 1	-2.17595	0.000192	0.009125
1388744_at	288532	Mcm7	minichromosome maintenance deficien	-2.048792	0.000193	0.009125
1397380_at	306203	Pxk	PX domain containing serine/threonine	3.435582	0.000195	0.009199
1367661_at	85247	S100a6	S100 calcium binding protein A6 (calcy	3.645417	0.000197	0.009229
1374249_at	292781	RGD13045	similar to Hypothetical protein MGC385	1.840031	0.000197	0.009229
1380736_at	117269	Rps6ka2	ribosomal protein S6 kinase polypeptic	5.02524	0.0002	0.009308
1370828_at	246326	Zdhhc2	zinc finger, DHHC domain containing 2	-1.852862	0.000202	0.009369
1369788_s_at	24516	Jun	Jun oncogene	2.639334	0.000203	0.009369
1375412_at	25227	Arsb	arylsulfatase B	-1.906933	0.000203	0.009369
1391495_at	294012	RGD13117	similar to RIKEN cDNA 2010012O05 (	-1.923481	0.000204	0.009394
1387791_at	24310	Ace	angiotensin I converting enzyme (pept	-2.681338	0.000207	0.009405
1389782_at	294499	RGD13055	similar to RIKEN cDNA 2010107G23 (	-2.392282	0.000207	0.009405

1393041_at	362519	Smc2l1_pr	SMC2 structural maintenance of chron	-3.135935	0.000208	0.009405
1371947_at	308690	Ndn	necdin	4.924081	0.000208	0.009405
1388413_at	311483	Rrbp1_pre	ribosome binding protein 1 homolog 18	2.061226	0.000209	0.009405
1372342_at	309375	Mrvldc1	MARVEL (membrane-associating) dom	2.925225	0.000209	0.009405
1372726_at	64627	Hist1h4b	germinal histone H4 gene	5.903904	0.000211	0.009426
1397223_at	500824	RGD1565€	similar to FYVE, RhoGEF and PH dom	3.52237	0.000211	0.009426
1369989_at	64533	Pnpo	pyridoxine 5'-phosphate oxidase	2.168599	0.000211	0.009426
1377653_at	366669	LOC36666	similar to mKIAA1011 protein	2.591757	0.000216	0.009548
1373772_at	84350	Dnmt1	DNA (cytosine-5-)-methyltransferase 1	-2.197581	0.000216	0.009548
1368004_at	64360	Mrpl23	mitochondrial ribosomal protein L23	-2.021722	0.000216	0.009548
1389766_at	292874	RGD1309C	hypothetical LOC292874 (predicted)	-2.856794	0.000219	0.009568
1379346_at	304832	Cdc73	cell division cycle 73, Paf1/RNA polym	2.008444	0.000219	0.009568
1398866_at	245903	Magi3	membrane associated guanylate kinas	-2.463356	0.00022	0.009568
1369286_at	25268	Proc	protein C	3.718553	0.000221	0.009568
1388750_at	64678	Tfrc	transferrin receptor	-2.313609	0.000221	0.009568
1380334_at	305340	RGD13597	hypothetical RNA binding protein RGD	1.821347	0.000221	0.009568
1370239_at	25632	Hba-a1	hemoglobin alpha, adult chain 1	4.10658	0.000222	0.00957
1367807_at	116552	Plod1	procollagen-lysine, 2-oxoglutarate 5-di	2.733463	0.000223	0.00957
1384273_at	287479	Carkl	carbohydrate kinase-like	-3.101352	0.000223	0.009575
1391330_at	252959	Bm259	BM259 protein	5.059669	0.000227	0.009631
1382434_at	314312	Entpd5	ectonucleoside triphosphate diphosph	2.646427	0.000228	0.009631
1393364_at	266733	Slc12a8	solute carrier family 12 (potassium/chl	3.843363	0.000229	0.009631
1393306_at	64624	Cul5	cullin 5	2.050436	0.000229	0.009631
1372119_at	300968	Ube1dc1	ubiquitin-activating enzyme E1-domair	1.848204	0.00023	0.009631
1371982_at	286897	Aip1	AIP1	-1.842231	0.00023	0.009631
1387899_at	25415	Crmp1	collapsin response mediator protein 1	-1.835987	0.00023	0.009631
1368008_at	60357	Prom1	prominin 1	3.966411	0.000231	0.009631
1390784_at	307075	Wdr37_pre	WD repeat domain 37 (predicted)	2.33484	0.000232	0.009663
1372602_at	305234	RGD13118	similar to genethonin 1	1.750819	0.000233	0.009671
1384805_at	641632	Defb24	beta-defensin 24	2.63267	0.000235	0.009742
1392534_at	311676	Tmepai_pr	transmembrane, prostate androgen inc	2.779594	0.000238	0.009804
1387968_at	282712	Slc6a15	solute carrier family 6 (neurotransmitte	-4.772015	0.000238	0.009804
1369756_a_at	84484	Slc4a4	solute carrier family 4, member 4	-3.538191	0.000241	0.009896
1379420_at	299135	RGD1565C	similar to Dehydrogenase/reductase S	2.07385	0.000243	0.009935
1387071_a_at	29477	Mapt	microtubule-associated protein tau	-2.521016	0.000245	0.009989
1375619_at	84408	Cdh8	cadherin 8	4.212968	0.000247	0.010002
1382874_at	304860	LOC30486	similar to N-acetylneuraminate pyruvat	5.436676	0.000247	0.010002
1388686_at	266766	Dscr1	Down syndrome critical region homolo	-2.289643	0.000248	0.010002
1375934_at	315911	RGD15662	similar to RIKEN cDNA D330045A20 (	2.503913	0.00025	0.010008
1391032_at	192247	Sez6	seizure related 6 homolog (mouse)	-5.062594	0.000251	0.010008
1393221_at	498789	RGD15648	similar to 20-alpha-hydroxysteroid deh	-5.408488	0.000251	0.010008
1385113_at	315338	Hoxc10	homeo box C10	-3.18604	0.000252	0.010008
1369230_at	29695	Gabrr2	gamma-aminobutyric acid (GABA-C) re	3.304118	0.000252	0.010008
1371848_at	24177	Afp	alpha-fetoprotein	-1.974801	0.000253	0.010008
1387803_at	60660	Ppp2r2b	protein phosphatase 2 (formerly 2A), re	-2.537656	0.000253	0.010008
1374468_at	301059	Myd88	myeloid differentiation primary respons	1.968535	0.000254	0.010011
1377753_at	307237	Pard6g_pre	par-6 partitioning defective 6 homolog	-2.433084	0.000255	0.010012
1372903_at	303575	RGD13103	similar to 3000004C01Rik protein	-6.919269	0.000256	0.010028
1367967_at	114200	Lepre1	leprecan 1	2.0557	0.000259	0.010087
1393723_at	314627	RGD1305C	similar to Serine/threonine-protein kina	-5.641605	0.000259	0.010087
1379603_at	500985	RGD15613	similar to CBL E3 ubiquitin protein liga	1.77852	0.00026	0.010087

1387074_at	84583	Rgs2	regulator of G-protein signaling 2	3.36768	0.000261	0.010095
1379967_at	306695	Zfp367	zinc finger protein 367	-4.862356	0.000262	0.010095
1374693_at	315760	Parp16	poly (ADP-ribose) polymerase family, r	1.72779	0.000262	0.010095
1368087_a_at	171070	Ptpn21	protein tyrosine phosphatase, non-rece	2.955213	0.000263	0.010105
1383860_at	25446	Fosl2	fos-like antigen 2	4.086811	0.000265	0.010111
1373074_at	307210	RGD13072	similar to RIKEN cDNA 2700002I20	-2.135985	0.000265	0.010111
1376633_at	503166	NA	NA	-3.220728	0.000266	0.010127
1374976_a_at	81782	Soat1	sterol O-acyltransferase 1	2.57919	0.000268	0.010127
1372137_at	288785	Bloc1s1_pr	biogenesis of lysosome-related organe	-2.754433	0.000268	0.010127
1368123_at	25718	Igf1r	insulin-like growth factor 1 receptor	2.506007	0.000269	0.010127
1373270_at	303630	Wipi1_prec	WD repeat domain, phosphoinositide i	-2.973323	0.000269	0.010127
1387923_at	24916	Zfp179	zinc finger protein 179	-2.192457	0.000271	0.010154
1388511_at	361617	Centd2	centaurin, delta 2	1.879585	0.000274	0.010157
1373026_at	363028	Spbc24_pr	spindle pole body component 24 homo	-5.442173	0.000274	0.010157
1389467_at	497979	MGC10877	similar to RIKEN cDNA 1810057C19	-2.310415	0.000275	0.010157
1390733_at	502205	NA	NA	3.118049	0.000275	0.010157
1379664_at	305955	RGD13077	similar to KIAA0853 protein (predicted)	2.034922	0.000276	0.010157
1390973_at	295323	Trim45_pre	tripartite motif protein 45 (predicted)	2.668816	0.000276	0.010157
1370362_at	116660	Ptprn	protein tyrosine phosphatase, receptor	2.361061	0.000276	0.010157
1368736_at	29391	Tsx	testis specific X-linked gene	2.15403	0.000277	0.010158
1388532_at	361301	RGD13105	similar to hypothetical protein	-2.289958	0.000279	0.010201
1390802_at	293888	RGD13068	similar to RIKEN cDNA 5033414D02 (p	-2.556791	0.00028	0.010201
1394330_at	361915	LOC36191	NA	3.946087	0.000281	0.010201
1367969_at	94167	Prdx6	peroxiredoxin 6	1.785886	0.000281	0.010201
1383798_at	313430	MGC94145	similar to CDNA sequence BC026682	4.50538	0.000283	0.010201
1378038_at	310820	Ptbp2	polypyrimidine tract binding protein 2	2.845414	0.000283	0.010201
1374805_at	299933	RGD15617	similar to hypothetical protein MGC552	-3.636245	0.000284	0.010201
1387892_at	29214	Tubb5	tubulin, beta 5	-2.26177	0.000285	0.010201
1367888_at	93662	Pcdh21	MT-protocadherin	-2.120809	0.000285	0.010201
1369976_at	58945	Dynll1	dynein light chain LC8-type 1	-1.784444	0.000288	0.010283
1370930_at	113886	Kif1c	kinesin family member 1C	1.78501	0.00029	0.010303
1392005_at	362773	Prima1_pre	proline rich membrane anchor 1 (predi	2.389214	0.000291	0.010303
1372789_at	362425	Zfp637	zinc finger protein 637	-1.967871	0.000292	0.010303
1367624_at	79255	Atf4	activating transcription factor 4	1.672847	0.000293	0.010303
1380828_at	29705	Gabra1	gamma-aminobutyric acid A receptor, a	2.081599	0.000294	0.010303
1373153_at	24558	Mog	myelin oligodendrocyte glycoprotein	3.126823	0.000294	0.010303
1370030_at	29739	Gclm	glutamate cysteine ligase, modifier sub	2.405032	0.000295	0.010303
1377659_at	310483	Mlf1_predic	myeloid leukemia factor 1 (predicted)	-5.379753	0.000295	0.010303
1377375_at	296925	Aass_pred	aminoadipate-semialdehyde synthase	7.096016	0.000296	0.010303
1391534_at	498728	Elovl2_pre	elongation of very long chain fatty acid	-2.195536	0.000298	0.010303
1380969_at	309510	RGD15646	similar to mKIAA0940 protein (predicte	2.674312	0.000299	0.010303
1375628_at	293991	Ndufb8_pre	NADH dehydrogenase (ubiquinone) 1	-2.700706	0.000299	0.010303
1376893_at	291129	Vmp_predi	vesicular membrane protein p24 (pred	-1.761403	0.000301	0.010303
1368606_at	80900	Slco1a5	solute carrier organic anion transporter	3.993644	0.000302	0.010303
1367667_at	83791	Fdps	farensyl diphosphate synthase	-2.03056	0.000302	0.010303
1398350_at	64160	Basp1	brain abundant, membrane attached s	-2.671052	0.000303	0.010303
1367925_at	64681	Mvp	major vault protein	2.540988	0.000304	0.010303
1374524_at	363285	Scly	selenocysteine lyase	2.091611	0.000304	0.010303
1378433_at	310200	RGD15642	similar to RIKEN cDNA 5730557B15 (p	3.122626	0.000305	0.010303
1374692_at	315871	Snx14_pre	sorting nexin 14 (predicted)	1.718336	0.000306	0.010303
1375045_at	307855	Wdr59	WD repeat domain 59	2.349334	0.000307	0.010303

1393352_at	300836	RGD13105	similar to hypothetical protein MGC389	2.048049	0.000307	0.010303
1377051_at	360463	Mpv17l_pre	Mpv17 transgene, kidney disease muta	-1.947021	0.000308	0.010303
1370678_s_at	29253	Maoa	monoamine oxidase A	1.839508	0.000308	0.010303
1375548_at	298425	RGD13103	similar to RIKEN cDNA 4732418C07 (p	1.924621	0.00031	0.010303
1374980_at	304656	RGD13075	similar to RIKEN cDNA 4930527D15	1.725404	0.00031	0.010303
1372294_at	554172	Perc64	PE responsive protein c64	-1.995299	0.00031	0.010303
1371808_at	287552	Blmh	bleomycin hydrolase	-1.821899	0.000311	0.010306
1374870_at	298101	Col27a1	procollagen, type XXVII, alpha 1	2.431888	0.000312	0.010317
1374596_at	309681	RGD13095	similar to RIKEN cDNA 1810043G02; I	-1.871554	0.000314	0.010317
1377037_at	50559	Cte1	cytosolic acyl-CoA thioesterase 1	-2.726193	0.000315	0.010317
1373052_at	316348	Pdcl3	phosducin-like 3	-1.943323	0.000315	0.010317
1382074_at	25008	Lta	lymphotoxin A	1.938149	0.000315	0.010317
1397410_at	312192	RGD15648	similar to RIKEN cDNA 4631427C17 (p	3.090633	0.000318	0.010317
1368887_at	29182	Cdh22	cadherin 22	10.35823	0.000318	0.010317
1367811_at	58835	Phgdh	3-phosphoglycerate dehydrogenase	4.824873	0.00032	0.010317
1382902_at	362376	Herc6	potential ubiquitin ligase	2.212213	0.000321	0.010317
1388182_at	246327	Prim1	DNA primase, p49 subunit	-3.154412	0.000322	0.010317
1374070_at	29326	Gpx2	glutathione peroxidase 2	6.595505	0.000322	0.010317
1374452_at	191569	Pde9a	phosphodiesterase 9A	-2.607221	0.000323	0.010317
1371548_at	297459	Mrps25	mitochondrial ribosomal protein S25	-1.715202	0.000323	0.010317
1383568_at	361856	Tube1_pre	tubulin, epsilon 1 (predicted)	-2.031545	0.000323	0.010317
1374189_at	305848	Zfp219	zinc finger protein 219	2.032884	0.000324	0.010317
1369652_at	24832	Thy1	thymus cell antigen 1, theta	-4.787012	0.000324	0.010317
1373945_at	314647	RGD15650	similar to bruno-like 5, RNA binding pro	-2.665497	0.000325	0.010317
1383140_at	361840	Spock2_pre	sparc/osteonectin, cwcv and kazal-like	2.081204	0.000325	0.010317
1376726_at	360599	NA	NA	3.611653	0.000326	0.01033
1384437_at	317575	Smarca1_p	SWI/SNF related, matrix associated, a	-2.760736	0.000328	0.010372
1378589_at	502697	RGD15648	similar to prepro-endothelin-3 (predicte	-2.255845	0.00033	0.010403
1372138_at	116639	Cutl1	cut-like 1 (Drosophila)	-2.173958	0.000331	0.010403
1368683_at	140914	Oldlr1	oxidized low density lipoprotein (lectin-	3.942307	0.000332	0.010417
1389725_at	293688	Tm7sf2	transmembrane 7 superfamily member	-3.686116	0.000333	0.010417
1375452_at	299078	Mgea6_pre	meningioma expressed antigen 6 (coil	2.209135	0.000335	0.010417
1372593_at	308362	Zfp110	zinc finger protein 110	2.087339	0.000335	0.010417
1391730_at	362996	Mll2_predic	myeloid/lymphoid or mixed-lineage leu	-2.118388	0.000337	0.010417
1372734_at	300236	Smagp	small cell adhesion glycoprotein	2.404812	0.000338	0.010417
1369029_at	117540	Plscr1	phospholipid scramblase 1	4.87977	0.000339	0.010417
1384154_at	114765	Wbp4	WW domain binding protein 4	1.638116	0.000339	0.010417
1374124_at	360498	RGD13073	similar to RIKEN cDNA 2610003J06	1.656288	0.00034	0.010417
1381294_at	309387	Cnnm1_pre	cyclin M1 (predicted)	2.086109	0.00034	0.010417
1383476_at	50693	Itih3	inter-alpha trypsin inhibitor, heavy chai	-1.604486	0.00034	0.010417
1372070_at	290644	Ifi30	interferon gamma inducible protein 30	1.999397	0.000341	0.010417
1370973_at	64155	Scn7a	sodium channel, voltage-gated, type V	4.74749	0.000343	0.010417
1375009_at	299346	Nudt14_pre	nudix (nucleoside diphosphate linked r	-3.115452	0.000344	0.010417
1371840_at	29733	Edg1	endothelial differentiation sphingolipid	4.597513	0.000344	0.010417
1371787_at	25562	Tceb3	transcription elongation factor B (SIII),	2.312724	0.000344	0.010417
1398896_at	300674	Arcn1	archain 1	1.738287	0.000345	0.010417
1369590_a_at	29467	Ddit3	DNA-damage inducible transcript 3	2.422364	0.000347	0.010454
1392476_at	500351	RGD15623	histone H4 variant H4-v.1 (predicted)	1.844514	0.000349	0.010454
1387770_at	170512	Ifi27l	interferon, alpha-inducible protein 27-li	-2.267829	0.00035	0.010454
1378389_at	307231	RGD15602	similar to nuclear factor of activated T-	-3.179126	0.00035	0.010454
1380981_at	314349	Ston2_pred	stonin 2 (predicted)	3.281565	0.00035	0.010454

1393918_at	360851	RGD1566(	similar to novel protein (predicted)	-3.938724	0.000352	0.010454
1383339_at	308537	RGD1310(	similar to NNX3 (predicted)	1.747708	0.000352	0.010454
1369958_at	64373	Rhob	ras homolog gene family, member B	-2.718081	0.000353	0.010454
1395806_at	501290	NA	NA	2.925293	0.000353	0.010454
1367577_at	24471	Hspb1	heat shock 27kDa protein 1	7.69131	0.000354	0.010456
1388904_at	360863	Dd25	hypothetical protein Dd25	1.934242	0.000355	0.010456
1374775_at	291234	Mki67_pre	antigen identified by monoclonal antibo	-5.389665	0.000356	0.010456
1386321_s_at	246273	Trib3	tribbles homolog 3 (Drosophila)	4.072171	0.000357	0.010456
1370562_at	171519	Calcb	calcitonin-related polypeptide, beta	8.549151	0.000358	0.010456
1369953_a_at	25145	Cd24	CD24 antigen	11.16146	0.000358	0.010456
1398521_at	114246	Trpv6	transient receptor potential cation char	-1.627426	0.00036	0.010501
1386990_at	117278	Ebp	phenylalkylamine Ca2+ antagonist (em	-1.757265	0.000366	0.010638
1376599_at	314993	Atad2_pre	ATPase family, AAA domain containing	-2.476628	0.000368	0.010644
1377832_at	310344	Plk4_predi	polo-like kinase 4 (Drosophila) (predict	-2.344997	0.000368	0.010644
1372843_at	363020	RGD1309	LOC363020 (predicted)	1.703274	0.000369	0.010644
1393376_at	293165	Sox6	SRY-box containing gene 6	2.673792	0.000369	0.010644
1382489_at	315843	RGD1564	similar to WD repeat domain 11 protein	1.882407	0.000371	0.010679
1391347_at	266688	Rab8b	RAB8B, member RAS oncogene family	3.245522	0.000373	0.010695
1387195_at	114093	St14	suppression of tumorigenicity 14 (colo	-1.788506	0.000374	0.010695
1374794_at	353302	Kif15	kinesin family member 15	-2.461114	0.000375	0.010695
1379909_at	361202	Gkap1	G kinase anchoring protein 1	-2.286639	0.000375	0.010695
1369982_at	81637	Ap2a2	adaptor protein complex AP-2, alpha 2	1.619644	0.000376	0.010695
1380682_at	308790	Rkhd3_pre	ring finger and KH domain containing 3	-2.353742	0.000377	0.010721
1373790_at	361998	Car14_pre	carbonic anhydrase 14 (predicted)	2.189914	0.000379	0.010736
1373658_at	315298	Racgap1_p	Rac GTPase-activating protein 1 (pred	-5.100685	0.000381	0.010782
1376436_at	315923	Lysmd3	LysM, putative peptidoglycan-binding,	1.894725	0.000384	0.01082
1377916_at	303380	Slfn2_pred	schlafen 2 (predicted)	2.005114	0.000386	0.01082
1393843_at	315745	Fem1b_pre	feminization 1 homolog b (C. elegans)	2.434807	0.000386	0.01082
1372259_at	306817	Dek	DEK oncogene (DNA binding)	-1.763752	0.000387	0.01082
1367595_s_at	24223	B2m	beta-2 microglobulin	1.950931	0.000387	0.01082
1370308_at	192252	Rs21c6	RS21-C6 protein	-3.059775	0.000387	0.01082
1374984_at	304733	Epb4.1l5	erythrocyte protein band 4.1-like 5	2.288875	0.000391	0.010875
1371594_at	288584	Fis1	fission 1 (mitochondrial outer membran	-1.848762	0.000391	0.010875
1379849_at	290519	Thoc3_pre	THO complex 3 (predicted)	-1.683422	0.000393	0.010917
1377844_at	299194	Zadh1	zinc binding alcohol dehydrogenase, d	2.089404	0.000396	0.010925
1368773_at	25448	Fshprh1	FSH primary response 1	-2.385342	0.000396	0.010925
1370381_at	286988	Pnrc1	proline rich 2	1.667372	0.000397	0.010925
1373451_at	361056	MGC9478(	similar to hypothetical protein FLJ1171	-1.652901	0.000398	0.010925
1388588_at	309170	Mtvr2	mammary tumor virus receptor 2	-1.725897	0.000398	0.010925
1385441_at	365550	Vpreb3_pre	pre-B lymphocyte gene 3 (predicted)	3.57254	0.000399	0.010925
1371774_at	302642	Sat_mappe	spermidine/spermine N1-acetyl transfe	2.921871	0.000399	0.010925
1389034_at	312688	Usp18	ubiquitin specific peptidase 18	2.160561	0.000406	0.011094
1371395_at	297093	Cbx3	chromobox homolog 3 (HP1 gamma h	-1.855352	0.000408	0.011106
1398877_at	192277	Stip1	stress-induced phosphoprotein 1	-1.70329	0.00041	0.011106
1379578_at	288105	Zbtb20_pre	zinc finger and BTB domain containing	1.93061	0.00041	0.011106
1369048_at	29689	Gabrd	gamma-aminobutyric acid A receptor,	-3.426227	0.000411	0.011106
1367671_at	25737	Pcna	proliferating cell nuclear antigen	-1.555971	0.000411	0.011106
1382147_at	363915	LOC36391	NA	3.445895	0.000412	0.011106
1369342_at	24941	Atp7a	ATPase, Cu++ transporting, alpha poly	-2.313083	0.000413	0.011123
1370229_at	64457	Ndrg4	N-myc downstream regulated gene 4	2.270761	0.000415	0.011145
1383585_s_at	297096	Snx10	sorting nexin 10	-3.577934	0.000417	0.011164

1389009_at	361956	Rsrc1	arginine/serine-rich coiled-coil 1	-1.889075	0.00042	0.011222
1397217_at	29428	Cugbp2	CUG triplet repeat, RNA binding protei	2.513272	0.000423	0.011268
1369837_at	60671	Gulo	L-gulonolactone oxidase	3.407164	0.000424	0.011268
1388669_at	313583	Sf3a3	splicing factor 3a, subunit 3	-1.513705	0.000426	0.011268
1398401_at	306066	Tdrd3	tudor domain containing 3	2.501988	0.000426	0.011268
1373414_at	306349	RGD13055	similar to Brain specific membrane-and	-1.637193	0.000428	0.011268
1367869_at	117520	Oxr1	oxidation resistance 1	-1.980142	0.000428	0.011268
1372592_at	84581	Hdac6	histone deacetylase 6	-2.084018	0.000429	0.011268
1368002_at	81709	Msh2	mutS homolog 2 (E. coli)	-2.85538	0.000429	0.011268
1372906_at	295930	RGD13095	similar to hypothetical protein MGC408	-2.612389	0.000429	0.011268
1373499_at	81714	Gas5	growth arrest specific 5	1.675895	0.00043	0.011268
1383098_at	360726	Fyttd1	forty-two-three domain containing 1	-2.326835	0.000432	0.011271
1399150_at	306229	RGD13060	similar to RIKEN cDNA A630054L15; h	-2.008829	0.000432	0.011271
1396949_at	298139	LOC29813	similar to RIKEN cDNA 2310003M01	5.392679	0.000433	0.011271
1387349_at	25546	Shox2	short stature homeobox 2	-2.220261	0.000437	0.011351
1372808_at	313410	RGD15640	similar to methylenetetrahydrofolate de	2.347555	0.000437	0.011351
1382942_at	299907	LOC29990	NA	3.916262	0.000438	0.011354
1382153_at	474143	Clecsf6	C-type (calcium dependent, carbohydr	-2.374058	0.00044	0.011383
1373597_at	288564	Pop7_pred	processing of precursor 7, ribonucleas	-1.738101	0.000444	0.011415
1368676_at	65209	Dnch2	dynein, cytoplasmic, heavy polypeptide	3.292426	0.000444	0.011415
1396521_at	291699	Stard4_pre	StAR-related lipid transfer (START) do	-1.779137	0.000444	0.011415
1386992_at	29355	Pkn1	protein kinase N1	2.913485	0.000446	0.011415
1367473_at	300075	Tomm22	translocase of outer mitochondrial mer	-1.590759	0.000446	0.011415
1388112_at	85333	Slc25a4	solute carrier family 25 (mitochondrial	-1.993127	0.00045	0.011481
1373386_at	394266	Gjb2	gap junction membrane channel protei	3.90525	0.00045	0.011481
1374902_at	310621	Iqgap3_pre	IQ motif containing GTPase activating	-3.750888	0.000456	0.011599
1387077_at	60336	Arpp19	cAMP-regulated phosphoprotein 19	1.607203	0.00046	0.011681
1369182_at	25584	F3	coagulation factor III	3.121699	0.000461	0.011681
1397525_at	303193	Alkbh5_pre	alkB, alkylation repair homolog 5 (E. co	1.847082	0.000463	0.011697
1377669_at	50645	Rab27a	RAB27A, member RAS oncogene fam	1.756758	0.000463	0.011697
1387039_at	58920	Gpc1	glypican 1	3.595501	0.000464	0.011697
1387647_at	117270	Tff1	trefoil factor 1	3.209419	0.000467	0.011716
1370506_at	25249	Coq7	demethyl-Q 7	-2.227468	0.000467	0.011716
1374746_at	500877	LOC50087	Ab1-152	1.523472	0.000468	0.011716
1389385_at	287383	Eppb9_pre	endothelial precursor protein B9 (predi	-1.937611	0.000468	0.011716
1391643_at	78969	Trib1	tribbles homolog 1 (Drosophila)	1.742603	0.00047	0.011731
1396404_at	361288	RGD15609	similar to FUN14 domain containing 2	-1.937919	0.000472	0.011773
1376079_at	291369	Dnajc1_pre	DnaJ (Hsp40) homolog, subfamily C, r	1.877231	0.000473	0.011773
1388516_at	315970	LOC31597	similar to CDV-3B	1.713031	0.000474	0.011777
1372087_at	298917	Harpb64	hypertrophic agonist responsive protei	-2.197972	0.000477	0.011791
1375980_at	360577	Nxn_predic	nucleoredoxin (predicted)	-2.66738	0.000478	0.011791
1388428_at	362227	Hars2_pred	histidyl tRNA synthetase 2 (predicted)	-1.863347	0.000478	0.011791
1373722_at	361308	Kif20a_pre	kinesin family member 20A (predicted)	-4.785263	0.000479	0.011791
1370561_at	171553	A3galt2	alpha 1,3-galactosyltransferase 2 (isog	-2.216306	0.000479	0.011791
1369022_at	171442	Svop	SV2 related protein	-2.056692	0.000482	0.011845
1376914_at	689490	LOC68949	NA	-4.799613	0.000484	0.011875
1384367_at	690148	LOC69014	NA	2.514012	0.000486	0.011883
1367687_a_at	25508	Pam	peptidylglycine alpha-amidating monoo	1.961808	0.000487	0.011883
1370086_at	24367	Fgg	fibrinogen, gamma polypeptide	4.643095	0.000488	0.011883
1380693_at	501560	RGD15638	similar to ribosomal protein S6 kinase	3.21894	0.000489	0.011883
1380239_at	310506	Ppm1l_pre	protein phosphatase 1 (formerly 2C)-lik	3.388529	0.00049	0.011883

1395590_at	501485	LOC50148	NA	2.517731	0.00049	0.011883
1389496_at	361458	Akap7	A kinase (PRKA) anchor protein 7	-2.059087	0.000492	0.011889
1370020_at	170943	Slc25a10	solute carrier family 25 (mitochondrial	-2.303419	0.000492	0.011889
1371935_at	289046	Tmem9_pr	transmembrane protein 9 (predicted)	-2.430431	0.000495	0.011939
1376890_at	406868	G4	G4 protein	4.515643	0.000497	0.011961
1384967_at	365583	Sart2_pred	squamous cell carcinoma antigen reco	2.845869	0.000499	0.011978
1369735_at	58935	Gas6	growth arrest specific 6	-1.919842	0.0005	0.01199
1386879_at	83781	Lgals3	lectin, galactose binding, soluble 3	6.375946	0.000503	0.012033
1382511_at	399489	E2f1	E2F transcription factor 1	-2.488861	0.000504	0.012036
1387870_at	79426	Zfp36	zinc finger protein 36	2.470444	0.000505	0.012036
1379368_at	303836	Bcl6_predi	B-cell leukemia/lymphoma 6 (predicted	5.530525	0.000507	0.012036
1385994_at	366261	Spo11_pre	sporulation protein, meiosis-specific, S	2.757073	0.000507	0.012036
1377363_at	310632	Rab25_pre	RAB25, member RAS oncogene family	3.799659	0.000508	0.012036
1369789_at	114516	Glra3	glycine receptor, alpha 3 subunit	4.29201	0.000508	0.012036
1374742_at	365216	Igsf4c_pred	immunoglobulin superfamily, member	-1.999057	0.000512	0.012115
1373232_at	311772	RGD1566C	similar to nidogen 2 (predicted)	2.695416	0.000515	0.012143
1387781_at	64198	Pmpcb	peptidase (mitochondrial processing) b	-1.479003	0.000516	0.012143
1379832_at	364834	Polr2d_pre	polymerase (RNA) II (DNA directed) po	-1.679751	0.000516	0.012143
1389605_at	305434	MGC12521	multiple coiled-coil GABABR1-binding	-2.881105	0.000519	0.012193
1386891_at	29542	Pebp1	phosphatidylethanolamine binding prot	-1.490627	0.000521	0.012208
1387709_at	360457	Figf	c-fos induced growth factor	3.798049	0.000526	0.012284
1371890_at	116665	Rere	arginine-glutamic acid dipeptide (RE) r	1.472082	0.000527	0.012284
1373305_at	360725	Snx4_pred	sorting nexin 4 (predicted)	-1.663974	0.000528	0.012284
1370511_at	24366	Fgb	fibrinogen, B beta polypeptide	9.46331	0.000529	0.012284
1368910_at	54705	Ppm2c	protein phosphatase 2C, magnesium d	-1.714267	0.000529	0.012284
1395426_at	499945	RGD15622	similar to ubiquitin-conjugating enzyme	-1.540691	0.00053	0.012284
1372474_at	366595	Sypl	synaptophysin-like protein	1.577019	0.000531	0.012284
1377837_at	314313	Znf183	zinc finger protein 183 (RING finger, C	1.739833	0.000533	0.012284
1370341_at	24334	Eno2	enolase 2, gamma	3.00949	0.000533	0.012284
1376580_at	499335	RGD15657	similar to frataxin (predicted)	-1.494103	0.000534	0.012284
1383673_at	317247	Nap1l2	nucleosome assembly protein 1-like 2	-2.089812	0.000538	0.012369
1371355_at	296658	Ndufa8	NADH dehydrogenase (ubiquinone) 1	-1.437795	0.000539	0.012371
1391475_at	313842	Hnrpll_pred	heterogeneous nuclear ribonucleoprote	-1.933728	0.000541	0.012381
1369926_at	64317	Gpx3	glutathione peroxidase 3	5.287858	0.000542	0.012381
1390707_at	54290	Rgs10	regulator of G-protein signalling 10	-2.813898	0.000543	0.012381
1399097_at	361217	Spin	spindlin	-1.516468	0.000545	0.012381
1389012_at	362344	Ndufb2_pre	NADH dehydrogenase (ubiquinone) 1	-1.516274	0.000545	0.012381
1391684_at	363206	Tmem14a_	transmembrane protein 14A (predicted	-2.175308	0.000545	0.012381
1371262_at	314487	Igha_mapp	immunoglobulin heavy chain (alpha po	2.443685	0.000548	0.012431
1382847_at	310638	Ash1l_pred	ash1 (absent, small, or homeotic)-like	1.810637	0.000554	0.012527
1388391_at	363441	Ndufa1_pre	NADH dehydrogenase (ubiquinone) 1	-1.434778	0.000556	0.012553
1398551_at	303534	RGD13118	similar to hypothetical protein DKFZp4	-5.727932	0.000558	0.012579
1376592_at	293829	Mcee_pred	methylmalonyl CoA epimerase (predic	-1.765993	0.000559	0.012579
1396455_at	365227	Cox6b_pre	cytochrome c oxidase, subunit VIb (pre	4.200575	0.00056	0.012579
1375175_at	360537	Tom1l2_pr	target of myb1-like 2 (chicken) (predict	3.35283	0.000562	0.012612
1378970_at	310782	RGD13105	similar to Myosin-binding protein H (My	1.944445	0.000567	0.012635
1398933_at	290999	Higd2a_pre	HIG1 domain family, member 2A (pred	-1.59914	0.000568	0.012635
1393020_at	500548	NA	NA	-3.343589	0.000568	0.012635
1387883_a_at	81814	Tmsb4x	thymosin, beta 4	-2.041236	0.000569	0.012635
1372158_at	246233	Lrp16	LRP16 protein	-3.378526	0.00057	0.012635
1388981_at	503165	LOC50316	similar to ADP-ribosylation factor GTPa	1.519589	0.00057	0.012635

1374995_at	291962	Elmo3	engulfment and cell motility 3, ced-12 l	1.571627	0.00057	0.012635
1374620_at	81613	Ceacam1	CEA-related cell adhesion molecule 1	-2.387297	0.000571	0.012635
1373754_at	683591	LOC68359	NA	1.954523	0.000575	0.012674
1370176_at	171086	Trak2	trafficking protein, kinesin binding 2	2.63335	0.000576	0.012674
1396983_at	289262	Fmn2_prec	formin 2 (predicted)	3.93678	0.000576	0.012674
1380815_at	304923	LOC30492	similar to hypothetical protein FLJ1175	1.517983	0.000576	0.012674
1374660_at	293528	RGD1309C	similar to hypothetical protein FLJ2000	-1.75427	0.000578	0.012674
1387806_at	171337	Rap1b	RAS related protein 1b	-1.612337	0.000578	0.012674
1367864_at	65152	Pfkm	phosphofructokinase, muscle	-1.858876	0.00058	0.012693
1393696_at	499856	LOC49985	hypothetical protein LOC499856	4.107515	0.000581	0.012699
1378352_at	310669	Golph3l	golgi phosphoprotein 3-like	1.59551	0.000583	0.012714
1372002_at	24392	Gja1	gap junction membrane channel protei	2.633798	0.000586	0.012752
1383410_at	116650	Srp54	signal recognition particle 54	1.56335	0.000588	0.012765
1377302_a_at	291939	Mmaa_pre	methylmalonic aciduria (cobalamin def	2.133599	0.000588	0.012765
1374036_at	312538	Mcm2_pre	minichromosome maintenance deficier	-1.833686	0.00059	0.012779
1367656_at	85492	Psmb7	proteasome (prosome, macropain) sub	-1.434308	0.000592	0.012789
1377576_at	310856	Ppa2_pred	pyrophosphatase (inorganic) 2 (predict	1.571628	0.000593	0.012789
1371885_at	292777	Ckap1_pre	cytoskeleton-associated protein 1 (pre	-1.637532	0.000594	0.012789
1378458_at	304947	Pbx1_pred	pre-B-cell leukemia transcription factor	3.470627	0.000595	0.012789
1389659_at	498690	RGD1565	similar to ctla-2-beta protein (141 AA) (	2.62504	0.000595	0.012789
1376862_at	298652	Ube4b_pre	ubiquitination factor E4B, UFD2 homol	1.810086	0.000601	0.012868
1388323_at	362440	Ndufa9	NADH dehydrogenase (ubiquinone) 1	-1.433804	0.000601	0.012868
1374185_at	302899	Txndc11_p	thioredoxin domain containing 11 (prec	1.420248	0.000602	0.012868
1390289_at	309855	RGD1311	similar to RIKEN cDNA A530089I17 (p	-1.559123	0.000603	0.012871
1370667_at	24785	Slc9a4	solute carrier family 9, member 4	3.484805	0.000606	0.012927
1373433_at	365273	Nsbp1_pre	nucleosome binding protein 1 (predicte	-1.493706	0.000608	0.012945
1377410_at	308607	E2f8	E2F transcription factor 8	-2.476666	0.000609	0.012945
1368071_at	171451	Mosc2	MOCO sulphurase C-terminal domain	2.207611	0.000611	0.012961
1393172_at	64824	Nab1	Ngfi-A binding protein 1	2.136898	0.000612	0.012968
1387788_at	24517	Junb	Jun-B oncogene	4.032678	0.000615	0.012999
1376787_at	313156	RGD13107	similar to hypothetical protein FLJ3181	5.113238	0.000618	0.01303
1369085_s_at	113938	Snurf	SNRPN upstream reading frame	-1.555955	0.00062	0.01303
1375968_at	500214	RGD1559€	similar to Catna2 protein (predicted)	-1.738671	0.00062	0.01303
1367930_at	29423	Gap43	growth associated protein 43	-2.48198	0.00062	0.01303
1389507_at	291553	Nedd4l	neural precursor cell expressed, devel	1.412041	0.000621	0.01303
1368331_at	81652	Ctbs	chitobiase, di-N-acetyl-	-1.928319	0.000623	0.013032
1388037_at	29599	Atp2b3	ATPase, Ca++ transporting, plasma m	-1.710775	0.000624	0.013032
1368886_at	25579	Map3k12	mitogen activated protein kinase kinas	-1.425988	0.000624	0.013032
1367800_at	25692	Plat	plasminogen activator, tissue	6.549071	0.000627	0.013052
1383768_at	286973	Elavl2	ELAV (embryonic lethal, abnormal visi	1.795094	0.000627	0.013052
1375027_at	363098	Ccpg1_pre	cell cycle progression 1 (predicted)	1.653326	0.000629	0.013066
1391152_at	363306	LOC36330	hypothetical protein LOC363306	3.061184	0.000631	0.013087
1391528_at	307236	RGD13082	similar to mKIAA0863 protein (predicte	-1.903877	0.000632	0.013094
1372254_at	295703	Serping1	serine (or cysteine) peptidase inhibitor	2.975983	0.000636	0.013147
1371879_at	298309	Lrrc42	leucine rich repeat containing 42	-2.260937	0.000637	0.013147
1396619_at	311362	RGD1561	similar to MGC14161 protein (predicte	2.232899	0.000638	0.013147
1390763_at	170901	Efna3	ephrin A3	-2.181422	0.000642	0.013208
1378640_at	316129	Uhrf1_map	ubiquitin-like, containing PHD and RIN	-5.267726	0.000644	0.013242
1373210_at	298941	Lamb1_pre	laminin, beta 1 (predicted)	-4.29691	0.000648	0.013259
1393451_at	361416	RGD1310	similar to RIKEN cDNA 2610510J17	-2.068679	0.000648	0.013259
1371560_at	292892	Irf3	interferon regulatory factor 3	1.545254	0.000648	0.013259

1384979_at	117097	Gpr50	G protein-coupled receptor 50	5.168834	0.00065	0.01328
1375848_at	296851	Pon2	paraoxonase 2	1.820857	0.000655	0.013309
1388753_at	311642	Sulf2	sulfatase 2	-1.766641	0.000657	0.013309
1367579_a_at	300218	Tuba6	tubulin, alpha 6	-1.558186	0.000657	0.013309
1377771_at	500258	RGD15602	similar to chromosome 3 open reading	1.688132	0.000657	0.013309
1398308_at	296883	Rpa3_pred	replication protein A3 (predicted)	-2.189555	0.000658	0.013309
1398582_at	314384	Rps6ka5_p	ribosomal protein S6 kinase, polypepti	1.941257	0.000659	0.013309
1370813_at	64352	Gstm5	glutathione S-transferase, mu 5	-3.712442	0.000659	0.013309
1376465_at	310233	RGD15638	sim ilar to glucocorticoid induced gene	-2.035897	0.000662	0.013309
1368658_at	25707	Cntf	ciliary neurotrophic factor	-1.614286	0.000664	0.013309
1375889_at	363469	Sms	spermine synthase	-3.004525	0.000666	0.013309
1386863_at	24668	Ppp1ca	protein phosphatase 1, catalytic subun	-1.373869	0.000667	0.013309
1393447_at	293047	Kif7_predic	kinesin family member 7 (predicted)	-3.610451	0.000667	0.013309
1372118_at	497878	MGC12520	HMP19 protein	-2.046414	0.000667	0.013309
1384565_at	362557	LOC36255	hypothetical protein LOC362557	-2.604802	0.000668	0.013309
1369683_at	64625	Bid	BH3 interacting domain death agonist	-3.261023	0.000669	0.013309
1390566_a_at	29593	Ckmt1	creatine kinase, mitochondrial 1, ubiqu	-3.044826	0.000669	0.013309
1374358_at	294004	RGD13076	similar to CG13901-PA	-1.601459	0.00067	0.013309
1398340_at	362201	Ccndbp1	cyclin D-type binding-protein 1	-1.506038	0.00067	0.013309
1394790_at	295586	Clcn6_pred	chloride channel 6 (predicted)	-2.394239	0.000671	0.013309
1367747_at	64664	Arl3	ADP-ribosylation factor-like 3	-1.822266	0.000675	0.013346
1375728_at	361063	Entpd4_pre	ectonucleoside triphosphate diphosphо	2.366655	0.000677	0.013346
1387079_at	497757	Gucy1a3	guanylate cyclase 1, soluble, alpha 3	2.036939	0.000677	0.013346
1368686_at	25377	Ambp	alpha 1 microglobulin/bikunin	-2.154814	0.000678	0.013346
1389542_at	362044	Cenpe_pre	centromere protein E (predicted)	-5.126243	0.000678	0.013346
1372250_at	499941	LOC49994	SCF apoptosis response protein 1	-2.045854	0.00068	0.013346
1398932_at	312393	Hint1_pred	histidine triad nucleotide binding protei	-1.703002	0.000682	0.013346
1375958_at	304361	LOC30436	similar to Hypothetical protein MGC288	1.570689	0.000683	0.013346
1374671_at	361857	MGC11284	NA	1.872377	0.000684	0.013346
1386892_at	83801	Ptms	parathymosin	-2.01655	0.000686	0.013346
1396722_at	140928	Pde11a	phosphodiesterase 11A	1.52924	0.000686	0.013346
1389193_at	305438	Sorcs2_pre	sortilin-related VPS10 domain containi	-2.212796	0.000687	0.013346
1388929_at	502635	RGD15597	similar to HMG-1 (predicted)	1.385741	0.000687	0.013346
1398426_at	498309	RGD15641	similar to RIKEN cDNA B130052G07 (	1.643004	0.000688	0.013346
1377967_at	292071	Ris2_predi	retroviral integration site 2 (predicted)	-2.601249	0.000689	0.013346
1377457_a_at	300652	Sorl1_pred	sortilin-related receptor, L(DLR class) /	2.302794	0.000689	0.013346
1368148_at	24596	Ngfr	nerve growth factor receptor (TNFR su	-3.021361	0.00069	0.013346
1393101_at	304495	Fbxl10	F-box and leucine-rich repeat protein 1	-1.664857	0.000691	0.013346
1373206_at	294925	Fndc3b_pr	fibronectin type III domain containing 3	1.693398	0.000693	0.013361
1369862_at	24649	Pim1	proviral integration site 1	2.509443	0.000694	0.013361
1371412_a_at	338475	Nrep	neuronal regeneration related protein	-2.949842	0.000695	0.013361
1377623_at	299613	Rkhd1_pre	ring finger (C3HC4 type) and KH doma	-1.709671	0.000695	0.013361
1371257_at	309288	Rorb_predi	RAR-related orphan receptor beta (pre	1.861803	0.000699	0.013405
1374701_at	360967	MGC10945	similar to 1700022N24Rik protein	1.458841	0.000701	0.013423
1374529_at	445442	Thbs1	NA	3.664661	0.000702	0.013427
1370094_at	60353	Acrv1	acrosomal vesicle protein 1	2.639896	0.000703	0.013438
1376340_a_at	361682	Tssc4	tumor-suppressing subchromosomal tr	-1.746341	0.000705	0.013445
1370075_at	24312	Dhfr	dihydrofolate reductase	-2.174812	0.000709	0.0135
1388531_at	361940	Pgrmc2	progesterone receptor membrane com	1.522926	0.000712	0.01353
1374983_at	361426	Klhdc4_pre	kelch domain containing 4 (predicted)	1.392662	0.000714	0.01353
1373489_at	363333	LOC36333	NA	-6.162345	0.000714	0.01353

1392791_at	25148	Egr3	early growth response 3	4.236473	0.000715	0.01353
1368162_at	171096	Cst6	cystatin E/M	-2.337659	0.000715	0.01353
1381605_at	310306	Usp13_pre	ubiquitin specific protease 13 (isopepti	1.660171	0.000717	0.013532
1387907_at	25262	Itpr1	inositol 1,4,5-triphosphate receptor 1	-1.725635	0.000719	0.013547
1375981_a_at	302553	Suv39h1_p	suppressor of variegation 3-9 homolog	-1.661375	0.000719	0.013547
1389043_at	308453	Adck4	aarF domain containing kinase 4	2.139574	0.000721	0.013548
1398835_at	81822	Actb	actin, beta	1.734695	0.000722	0.013548
1382589_at	361898	RGD13077	similar to hypothetical protein FLJ1179	1.457252	0.000724	0.013552
1378710_at	315438	Ccdc67	coiled-coil domain containing 67	3.661116	0.000724	0.013552
1381993_at	294141	Clic2	chloride intracellular channel 2	-3.001448	0.000725	0.013552
1374321_at	499309	RGD15601	similar to RIKEN cDNA 2700081O15 (	-1.547751	0.000726	0.013552
1373431_at	305131	Lrrc8d	leucine rich repeat containing 8 family,	1.636484	0.000727	0.013553
1399011_at	304343	Cops6_pre	COP9 (constitutive photomorphogenic	-1.514258	0.000728	0.013562
1372349_at	293844	RGD15629	similar to UCH37-interacting protein 1	-1.730196	0.00073	0.013574
1381171_at	364858	Stk32a_pre	serine/threonine kinase 32A (predicted	2.991332	0.000734	0.013626
1368012_at	64523	Tep1	telomerase associated protein 1	1.507781	0.000739	0.013663
1380525_at	310326	Arse	arylsulfatase E (chondrodysplasia pun	-1.877045	0.00074	0.013663
1372859_at	501167	Gmppa	GDP-mannose pyrophosphorylase A	1.935809	0.00074	0.013663
1378780_at	315663	Exph5_pre	exophilin 5 (predicted)	3.260604	0.000741	0.013663
1377145_at	294614	RGD15621	similar to very large G-protein coupled	-1.603933	0.000741	0.013663
1392938_s_at	361624	RGD13069	similar to C11orf17 protein (predicted)	-1.640573	0.000742	0.013663
1367842_at	29722	Apbb1	amyloid beta (A4) precursor protein-bi	-2.058106	0.000745	0.013673
1388584_at	315160	RGD13057	similar to D15Wsu75e protein	-1.414633	0.000745	0.013673
1396142_at	301371	Mrps9	mitochondrial ribosomal protein S9	-2.171729	0.000746	0.013673
1372674_at	312603	Rybp_pred	RING1 and YY1 binding protein (predi	-1.363672	0.000747	0.013673
1375421_a_at	192256	Pja2	praja 2, RING-H2 motif containing	1.374471	0.000751	0.01372
1398262_at	24689	Prps2	phosphoribosyl pyrophosphate synthet	-1.861303	0.000751	0.01372
1390738_at	378947	Bst2	bone marrow stromal cell antigen 2	2.010062	0.000757	0.013771
1371541_at	288057	Mylk_predi	myosin, light polypeptide kinase (predi	-2.199246	0.000758	0.013771
1372462_at	308100	Acat2	acetyl-Coenzyme A acetyltransferase 2	-1.697708	0.000759	0.013771
1390001_at	306004	Rhobtb2	Rho-related BTB domain containing 2	1.454728	0.000759	0.013771
1379255_at	302526	Atp6ap2	ATPase, H+ transporting, lysosomal ac	1.488986	0.000762	0.013771
1376645_at	360757	RGD13073	similar to RIKEN cDNA 6330406I15 (p	6.869089	0.000763	0.013771
1395403_at	362895	Stac3_prec	SH3 and cysteine rich domain 3 (predi	1.691908	0.000764	0.013771
1378310_at	499194	RGD15627	similar to neuromedin B precursor - rat	1.482499	0.000764	0.013771
1371605_at	299739	Ndufa12_p	NADH dehydrogenase (ubiquinone) 1	-1.677491	0.000764	0.013771
1367857_at	84575	Fads1	fatty acid desaturase 1	-2.996466	0.000765	0.013771
1370808_at	25035	Cyb5r3	cytochrome b5 reductase 3	-1.671113	0.000772	0.013837
1398375_at	298763	Mta3_pred	metastasis associated 3 (predicted)	-1.672812	0.000772	0.013837
1386755_at	312863	LOC31286	similar to RIKEN cDNA 1810060J02	-1.542863	0.000772	0.013837
1382740_at	291961	RGD13111	similar to RIKEN cDNA E430013E20 g	4.050002	0.000773	0.013837
1390944_at	361916	MGC10877	Snf7 homologue associated with Alix 3	3.300738	0.000774	0.013837
1367565_a_at	25319	Fth1	ferritin, heavy polypeptide 1	1.314139	0.000777	0.013861
1389527_at	378467	LOC37846	promethin	-2.690507	0.000777	0.013861
1394129_at	113970	Magi2	membrane associated guanylate kinas	-3.017094	0.000779	0.013868
1384488_at	494444	Cdkn2d	NA	-2.5071	0.000779	0.013868
1389391_at	362883	RGD15648	similar to Solute carrier family 35, mem	-1.410465	0.000783	0.013918
1380824_at	306548	Hook3	hook homolog 3 (Drosophila)	2.013585	0.000784	0.013918
1388931_at	299938	Mrpl13	mitochondrial ribosomal protein L13	-1.498366	0.000786	0.01393
1370174_at	171071	Myd116	myeloid differentiation primary respons	2.828426	0.000789	0.013955
1398759_at	25564	Tgfb1i4	transforming growth factor beta 1 indu	1.377836	0.000792	0.014005

1370664_a_at	286986	LOC28698	putative pheromone receptor Go-VN13	1.535544	0.000793	0.014006
1367727_at	116692	Pscd2	pleckstrin homology, Sec7 and coiled-c	-1.589741	0.000796	0.014019
1367995_at	24248	Cat	catalase	1.567221	0.000796	0.014019
1372052_at	361662	Bub3	budding uninhibited by benzimidazoles	-1.530769	0.000798	0.014027
1391459_at	367747	RGD15647	similar to nudix (nucleoside diphospha	-1.406932	0.000799	0.014027
1398977_at	25700	Ide	insulin degrading enzyme	-1.335862	0.000801	0.014035
1389756_at	362510	Melk_predi	maternal embryonic leucine zipper kina	-5.373392	0.000802	0.014035
1386890_at	81778	S100a10	S100 calcium binding protein A10 (calp	-2.035027	0.000803	0.014039
1389811_at	360519	Rasgef1c_	RasGEF domain family, member 1C (p	-2.205694	0.000806	0.01407
1382332_at	313304	Stag2_prec	stromal antigen 2 (predicted)	-1.46902	0.000807	0.01407
1374840_at	362580	RGD13056	similar to CG2919-PA (predicted)	-1.611754	0.000808	0.01407
1390584_at	307251	RGD15607	similar to ring finger protein 111 (predi	-2.739199	0.000819	0.014255
1393954_at	308854	Chrdl2_pre	chordin-like 2 (predicted)	3.308995	0.000822	0.01428
1387147_at	171058	Rab3c	RAB3C, member RAS oncogene famil	-1.300338	0.000824	0.014291
1383115_at	309338	Cstf2t_prec	cleavage stimulation factor, 3' pre-RNA	-1.578496	0.000825	0.014291
1391604_at	315508	LOC31550	NA	-1.793244	0.000826	0.014291
1393581_at	289054	Aspm_prec	asp (abnormal spindle)-like, microcepł	-3.745875	0.000827	0.014291
1389253_at	29142	Vnn1	vanin 1	6.346224	0.00083	0.014313
1370903_a_at	296318	LOC29631	similar to Ndr3 protein	-1.715455	0.00083	0.014313
1371439_at	313052	RGD15647	similar to erythrocyte membrane protei	1.480096	0.000832	0.014313
1371094_at	117555	Lhx2	LIM homeobox protein 2	-2.040821	0.000833	0.014313
1382370_at	117524	Ccnf	cyclin F	-1.934761	0.000834	0.014313
1371828_at	288667	RGD13108	similar to RIKEN cDNA 1500011H22	-1.489242	0.000836	0.014329
1388705_at	498398	RGD15650	similar to selenoprotein SelM (predicte	1.558829	0.000838	0.014339
1383181_at	364240	Dnajc9_pre	DnaJ (Hsp40) homolog, subfamily C, r	-1.369084	0.000838	0.014339
1369074_at	170573	Slc38a4	solute carrier family 38, member 4	1.798184	0.00084	0.014342
1384253_at	364558	LOC36455	similar to palladin; CGI-151 protein	-2.562975	0.000843	0.014376
1389503_at	288748	NA	NA	3.515488	0.000844	0.014379
1367452_at	171127	Sumo2	SMT3 suppressor of mif two 3 homolog	-1.409056	0.000845	0.014384
1393048_at	25083	Adra2a	adrenergic receptor, alpha 2a	2.017809	0.000849	0.014426
1372377_at	314336	Ddx41_pre	DEAD (Asp-Glu-Ala-Asp) box polypept	-1.329822	0.00085	0.014433
1369462_at	24380	Gad2	glutamic acid decarboxylase 2	3.829661	0.000853	0.014454
1367712_at	116510	Timp1	tissue inhibitor of metalloproteinase 1	6.165501	0.000855	0.01447
1370177_at	25066	PVR	poliovirus receptor	5.661537	0.000856	0.01447
1397607_at	303016	RGD13108	similar to adult retina protein (predicted	2.793304	0.000858	0.014472
1389170_at	64026	Casp7	caspase 7	3.542909	0.000858	0.014472
1393196_at	311114	Klhl23_pre	kelch-like 23 (Drosophila) (predicted)	-2.100196	0.000859	0.014472
1383867_at	310261	Eif5a2_pre	eukaryotic translation initiation factor 5	-2.110347	0.000862	0.014491
1372266_at	309812	Rev3l	REV3-like, catalytic subunit of DNA po	-1.503311	0.000863	0.014491
1376026_at	288257	Donson	downstream neighbor of SON	-2.189803	0.000864	0.014497
1374219_at	289338	Disp1_prec	dispatched homolog 1 (Drosophila) (pr	3.299036	0.000866	0.014509
1368842_at	84382	Tcf4	transcription factor 4	8.539123	0.00087	0.014552
1393191_at	500557	RGD15612	similar to RIKEN cDNA 2610200G18 (	-1.773269	0.000873	0.014587
1384910_at	312668	RGD15620	similar to putative voltage-gated calciu	-2.038374	0.000881	0.014652
1373003_at	304582	Sart3_pred	squamous cell carcinoma antigen reco	-1.451019	0.000882	0.014652
1386994_at	29619	Btg2	B-cell translocation gene 2, anti-prolife	2.041798	0.000882	0.014652
1384290_at	308968	Rbbp6	retinoblastoma binding protein 6	-1.510018	0.000883	0.014652
1386882_at	83462	Tctex1	t-complex testis expressed 1	-1.827223	0.000883	0.014652
1368268_at	114521	Tdg	thymine-DNA glycosylase	-1.808165	0.000883	0.014652
1370915_s_at	171437	Dnttip1	deoxynucleotidyltransferase, terminal,	-1.858277	0.000886	0.014666
1398184_at	314212	Daam1_pre	dishevelled associated activator of mo	1.937783	0.000888	0.014666

1399039_at	501165	NA	NA	1.962248	0.000889	0.014666
1371446_at	289014	Mapkapk2	MAP kinase-activated protein kinase 2	2.22975	0.00089	0.014666
1372320_at	317464	Msl31	male-specific lethal-3 homolog 1 (Dros	-1.542012	0.000893	0.014666
1367729_at	64313	Oat	ornithine aminotransferase	-2.381378	0.000896	0.014666
1393082_at	171010	Ppp1r14c	protein phosphatase 1, regulatory (inhi	2.390299	0.000896	0.014666
1398771_at	50567	Slc3a2	solute carrier family 3 (activators of dib	1.414671	0.000899	0.014666
1392017_at	311030	Stam2	signal transducing adaptor molecule (S	2.357479	0.000899	0.014666
1383380_at	291317	Rpp38	ribonuclease P/MRP 38 subunit (huma	-1.491924	0.000901	0.014666
1372455_at	362326	Tm4sf12	transmembrane 4 superfamily member	1.536556	0.000902	0.014666
1368034_at	24259	Chgb	chromogranin B	-1.666809	0.000903	0.014666
1379257_at	307514	Epb4.1l4a_	erythrocyte protein band 4.1-like 4a (pr	-2.532605	0.000904	0.014666
1390937_at	171026	Akap5	A kinase (PRKA) anchor protein 5	-1.639373	0.000907	0.014666
1371074_a_at	29685	Mcm6	minichromosome maintenance deficier	-2.9749	0.000908	0.014666
1367635_at	25506	P4hb	prolyl 4-hydroxylase, beta polypeptide	1.372965	0.000909	0.014666
1388018_at	25544	Sele	selectin, endothelial cell	4.807286	0.000909	0.014666
1370939_at	25288	Acsl1	acyl-CoA synthetase long-chain family	-1.548093	0.00091	0.014666
1388749_at	312439	RGD13097	similar to hypothetical protein FLJ1391	-1.314153	0.000913	0.014666
1398879_at	290796	Tmem66	transmembrane protein 66	1.450237	0.000914	0.014666
1377690_at	292771	Sipa1l3	signal-induced proliferation-associated	1.468756	0.000915	0.014666
1391212_at	302593	Tceal1	transcription elongation factor A (SII)-li	-1.414178	0.000916	0.014666
1377194_a_at	308820	RGD13086	similar to RIKEN cDNA 2310015N07	-1.713048	0.000917	0.014666
1380019_at	293650	Tcirg1	T-cell, immune regulator 1	2.228234	0.000917	0.014666
1367737_at	24375	Fuca	fucosidase, alpha-L- 1, tissue	-1.872025	0.000918	0.014666
1391530_a_at	289934	RGD1311C	similar to hypothetical protein FLJ2060	-1.669738	0.00092	0.014666
1388325_at	299159	Atp6v1d	ATPase, H+ transporting, V1 subunit D	1.305612	0.00092	0.014666
1387949_at	171518	Cyp2c70	cytochrome P450, family 2, subfamily (	3.21524	0.000921	0.014666
1370869_at	29592	Bcat1	branched chain aminotransferase 1, cy	7.783031	0.000921	0.014666
1381164_at	311919	Golga1_pre	golgi autoantigen, golgin subfamily a, 1	1.593731	0.000921	0.014666
1397959_at	312248	RGD13107	similar to RIKEN cDNA D130059P03 g	1.394262	0.000924	0.014666
1384178_at	308571	Lrrc4b_pre	leucine rich repeat containing 4B (prec	-2.75941	0.000927	0.014666
1381791_at	363391	RGD1562C	similar to GTPase activating protein te	2.102581	0.000927	0.014666
1371769_at	65168	Scamp2	secretory carrier membrane protein 2	1.741813	0.000927	0.014666
1377025_at	84392	Kif3a	kinesin family member 3a	-1.912816	0.000928	0.014666
1392676_at	308326	Mip1	myocardial ischemic preconditioning u	1.495119	0.000928	0.014666
1368263_a_at	25037	Mobp	myelin-associated oligodendrocytic ba	3.053124	0.00093	0.014666
1393833_at	362765	Angel1_pre	angel homolog 1 (Drosophila) (predicte	2.834761	0.000931	0.014666
1378155_at	360865	RGD1566C	similar to KIAA1096 protein (predicted)	1.385743	0.000934	0.014666
1367769_at	117017	Polr2g	polymerase (RNA) II (DNA directed) po	-1.564078	0.000935	0.014666
1388813_at	79119	Arf2	ADP-ribosylation factor 2	-2.377248	0.000937	0.014666
1384295_at	307133	Hspa14	heat shock 70kDa protein 14	-1.680939	0.000937	0.014666
1388347_at	362934	LOC36293	similar to lymphocyte antigen 6 comple	-1.55463	0.000938	0.014666
1368391_at	25648	Slc7a1	solute carrier family 7 (cationic amino a	1.83712	0.000938	0.014666
1393433_at	365691	RGD13065	similar to Agrin (predicted)	4.726237	0.000941	0.014666
1391437_at	313771	MGC94339	similar to BC002216 protein	1.678789	0.000941	0.014666
1368275_at	140910	Sc4mol	sterol-C4-methyl oxidase-like	-1.716505	0.000942	0.014666
1367530_at	362283	Stx16_prec	syntaxin 16 (predicted)	2.137781	0.000942	0.014666
1390857_at	316067	Xylb	xylulokinase homolog (H. influenzae)	2.582447	0.000942	0.014666
1376733_at	303926	Igsf11	immunoglobulin superfamily, member	2.611945	0.000944	0.014666
1389728_at	311311	Meis2_prec	Meis1, myeloid ecotropic viral integrati	8.206248	0.000945	0.014666
1390032_at	288771	Rbms2	RNA binding motif, single stranded inte	3.044429	0.000946	0.014666
1394039_at	84410	Klf5	Kruppel-like factor 5	2.548522	0.000947	0.014666

1398943_at	293863	RGD15624	similar to Eso3 protein (predicted)	-1.695926	0.000948	0.014666
1399047_at	287635	Mrpl27_pre	mitochondrial ribosomal protein L27 (p	-1.351107	0.000951	0.014666
1371254_at	291103	Uqcrfs1	ubiquinol-cytochrome c reductase, Rie	-1.641357	0.000952	0.014666
1387017_at	29230	Sqle	squalene epoxidase	-1.432783	0.000952	0.014666
1377846_a_at	366547	Msh6_pred	mutS homolog 6 (E. coli) (predicted)	-1.958447	0.000953	0.014666
1392965_a_at	292401	Smoc2_pre	SPARC related modular calcium bindir	3.646905	0.000954	0.014666
1388455_at	114119	Gng10	guanine nucleotide binding protein (G	-1.362503	0.000955	0.014666
1387000_at	56082	Gorasp1	golgi reassembly stacking protein 1	1.436817	0.000955	0.014666
1368514_at	25750	Maob	monoamine oxidase B	-2.307087	0.000955	0.014666
1382084_at	500464	RGD15629	similar to EHM2 (predicted)	1.685692	0.000955	0.014666
1392816_at	297607	RGD13111	similar to DNA segment, Chr 6, Wayne	-1.870319	0.000957	0.014666
1377016_at	362978	Creld2	cysteine-rich with EGF-like domains 2	2.264667	0.000958	0.014666
1370865_at	25179	Idh3g	isocitrate dehydrogenase 3 (NAD), gar	-1.356738	0.000958	0.014666
1369107_at	24773	Sftpa1	surfactant, pulmonary-associated prote	4.955374	0.000958	0.014666
1377175_at	316602	RGD13058	similar to hypothetical protein A730008	-4.292305	0.00096	0.014676
1371557_at	363291	Thap4	THAP domain containing 4	1.543793	0.000962	0.014692
1384068_at	306575	Ckap2_pre	cytoskeleton associated protein 2 (pre	-4.751327	0.000967	0.014735
1370411_at	89821	Trpc1	transient receptor potential cation char	1.288689	0.000967	0.014735
1378698_at	29605	Myh13	myosin, heavy polypeptide 13, skeleta	4.484636	0.000972	0.014797
1370995_at	171068	Pou2f1	POU domain, class 2, transcription fac	2.582359	0.000974	0.014799
1368214_at	29357	Smad2	MAD homolog 2 (Drosophila)	-1.47634	0.000978	0.014836
1369160_a_at	117955	Slc4a7	solute carrier family 4, sodium bicarbo	1.649053	0.000978	0.014836
1373290_at	312299	LOC31229	NA	-2.669676	0.000989	0.014968
1368212_at	81650	Csnk2b	casein kinase 2, beta subunit	1.538862	0.000989	0.014968
1392552_at	310614	Gatad2b	GATA zinc finger domain containing 2I	2.127148	0.000996	0.015049
1387224_at	54248	Dgkb	diacylglycerol kinase, beta	2.517086	0.000997	0.015055
1391412_at	362229	Xrn2_predi	5'-3' exoribonuclease 2 (predicted)	-1.33682	0.000999	0.015065
1391346_at	292082	RGD1309C	similar to FKSG26 protein (predicted)	1.387755	0.001006	0.015144
1371568_at	113893	Sncb	synuclein, beta	-2.389348	0.001007	0.015144
1369301_at	83518	Agtrl1	angiotensin receptor-like 1	2.265229	0.001009	0.015166
1399005_at	312754	Ppp2r5a_p	protein phosphatase 2, regulatory subu	1.372378	0.001011	0.01518
1374933_at	78967	Mcam	melanoma cell adhesion molecule	3.01373	0.001016	0.015196
1391062_at	499851	RGD1566C	similar to elongation protein 4 homolog	-2.089965	0.001016	0.015196
1372506_at	25634	G6pc	glucose-6-phosphatase, catalytic	-1.315607	0.001017	0.015196
1373576_at	287249	RGD13107	similar to CCR4	-1.428655	0.001019	0.015196
1370965_at	25705	Tcf8	transcription factor 8	1.823056	0.00102	0.015196
1375441_at	266975	Sars1	seryl-aminoacyl-tRNA synthetase 1	1.67911	0.00102	0.015196
1372352_at	315989	Armet_pred	arginine-rich, mutated in early stage tu	1.693935	0.001021	0.015196
1367856_at	24377	G6pdx	glucose-6-phosphate dehydrogenase X	1.379049	0.001026	0.015218
1374455_at	293679	NA	NA	-2.103301	0.001026	0.015218
1388414_at	362588	Ndufs5b	NADH dehydrogenase (ubiquinone) Fe	-1.307526	0.001026	0.015218
1383454_a_at	289342	Rps6kc1	ribosomal protein S6 kinase, polypepti	4.17665	0.001027	0.015218
1368819_at	24511	Itgb1	integrin beta 1 (fibronectin receptor be	1.757772	0.00103	0.015229
1392128_at	303348	RGD15614	similar to novel protein (predicted)	-2.104455	0.001031	0.015229
1386097_at	316775	Ankrd12_p	ankyrin repeat domain 12 (predicted)	1.45925	0.001033	0.015229
1380775_at	309523	Mphosph1_	M-phase phosphoprotein 1 (predicted)	-5.261881	0.001034	0.015229
1372938_at	303518	RGD13047	similar to RIKEN cDNA 6330509G02	-1.563142	0.001034	0.015229
1368300_at	25369	Adora2a	adenosine A2a receptor	-2.617909	0.001035	0.015229
1372056_at	316035	Cmtm6	CKLF-like MARVEL transmembrane do	1.324344	0.001035	0.015229
1377141_at	498685	LOC49868	NA	-1.601912	0.001041	0.015288
1390178_at	300965	Srprb	signal recognition particle receptor, B s	1.796079	0.001042	0.015288

1378489_at	312203	Tsga13_pr	testis specific gene A13 (predicted)	3.569361	0.001043	0.015294
1381174_at	307480	RGD73502	SEL1 domain containing protein RGD7	1.425875	0.001045	0.015299
1371788_at	501926	RGD15597	similar to RIKEN cDNA 2610510L01 (p	-3.859793	0.001046	0.015306
1382467_at	363498	LOC36349	similar to brain expressed X-linked pro	-1.657882	0.001049	0.015325
1387061_at	81679	Jup	junction plakoglobin	-1.265885	0.001051	0.015335
1395781_at	298506	Mycl1_map	v-myc myelocytomatosis viral oncogen	-1.511357	0.001054	0.015366
1390415_at	292206	Trip13	thyroid hormone receptor interactor 13	-2.826108	0.001056	0.015366
1378127_at	361258	Cul2_predi	cullin 2 (predicted)	-1.904931	0.001057	0.015366
1389089_at	294281	RT1-Ke4	RT1 class I, locus Ke4	1.629048	0.001057	0.015366
1372188_at	300149	MGC11441	similar to hypothetical protein D15Ertd	-1.610971	0.001059	0.015368
1398837_at	81807	Tceb2	transcription elongation factor B (SIII),	-1.224591	0.00106	0.015368
1373623_at	308451	Itpkc	inositol 1,4,5-trisphosphate 3-kinase C	2.501302	0.001062	0.015374
1389968_at	292148	Eif3s10	eukaryotic translation initiation factor 3	1.305429	0.001066	0.015395
1374494_at	361237	LOC36123	similar to testis-specific chromodomain	-1.364596	0.001067	0.015395
1368372_at	24800	Sts	steroid sulfatase	1.706024	0.001068	0.015395
1388425_at	315594	RGD13058	similar to RIKEN cDNA D130038B21	1.776093	0.001069	0.015395
1371987_at	306672	Pols_predi	polymerase (DNA directed) sigma (pre	-1.408617	0.001069	0.015395
1385767_at	304000	LOC30400	cell adhesion molecule JCAM	1.748091	0.001073	0.015428
1373296_a_at	293155	RGD13116	similar to RIKEN cDNA 3200002M19 (	-4.245876	0.001074	0.015428
1388504_at	314949	MGC11637	NA	-1.368615	0.001075	0.015428
1387172_a_at	81809	Tgfb2	transforming growth factor, beta 2	1.862792	0.001076	0.015428
1370954_at	64475	P4ha1	procollagen-proline, 2-oxoglutarate 4-c	3.134416	0.001079	0.015464
1394597_at	305816	Ddhd1	DDHD domain containing 1	1.497546	0.001082	0.015477
1374523_at	290636	Pgls_predi	6-phosphogluconolactonase (predicted	-1.470511	0.001083	0.015477
1373885_at	300266	Cbx5_pred	chromobox homolog 5 (Drosophila HP	-1.556209	0.001087	0.015477
1387190_at	140866	Dgka	diacylglycerol kinase, alpha	2.677617	0.001087	0.015477
1371432_at	287721	Vat1	vesicle amine transport protein 1 homo	1.327122	0.001088	0.015477
1383792_at	297872	Sytl1	synaptotagmin-like 1	3.952917	0.001089	0.015477
1388792_at	291005	Gadd45g	growth arrest and DNA-damage-induci	-1.324535	0.001089	0.015477
1379529_at	501603	NA	NA	-2.694935	0.00109	0.015477
1378466_at	362204	Casc4_pre	cancer susceptibility candidate 4 (pred	2.708212	0.001091	0.015477
1388817_at	310665	LOC31066	hypothetical protein LOC310665	1.709659	0.001093	0.015485
1391378_at	316737	Lpin2_prec	lipin 2 (predicted)	2.781468	0.001097	0.015521
1381933_at	304528	Rfc5_predi	replication factor C (activator 1) 5 (pre	-2.24436	0.001099	0.015521
1374003_at	498007	NA	NA	-1.322056	0.0011	0.015521
1388204_at	171052	Mmp13	matrix metallopeptidase 13	6.428522	0.001102	0.015521
1377738_a_at	315097	Cyhr1	cysteine and histidine rich 1	1.57265	0.001104	0.015521
1373916_at	170915	Ep300	E1A binding protein p300	1.355831	0.001104	0.015521
1367691_at	85332	Prkcdbp	protein kinase C, delta binding protein	1.7547	0.001104	0.015521
1370580_a_at	29296	Cyp2c37	cytochrome P450, 2c37	4.20921	0.001104	0.015521
1368077_at	24362	Fbp1	fructose-1,6- biphosphatase 1	-4.575533	0.001107	0.015525
1382118_at	619476	Apeg3	antisense paternally expressed gene 3	1.704578	0.001108	0.015525
1387299_at	83422	Miz1	Msx-interacting-zinc finger	-1.571136	0.001108	0.015525
1368226_at	171047	RGD62038	Nucleoside 2-deoxyribosyltransferase	-2.632521	0.00111	0.015529
1383206_at	361073	Cog3	component of oligomeric golgi complex	1.508712	0.001111	0.015529
1393377_at	500826	Galnt4	UDP-N-acetyl-alpha-D-galactosamine:	-1.439426	0.001116	0.015577
1389189_at	81634	Actn1	actinin, alpha 1	1.327618	0.001119	0.01558
1377541_at	500726	LOC50072	NA	1.48426	0.001119	0.01558
1388837_at	363024	Slc44a2_pi	solute carrier family 44, member 2 (pre	-1.875548	0.00112	0.01558
1388930_at	363013	Tmem123	transmembrane protein 123	1.306625	0.00112	0.01558
1397211_at	498416	RGD15662	similar to Grb10 protein (predicted)	3.930359	0.001124	0.015601

1384965_at	54308	Slk	serine/threonine kinase 2	-2.423618	0.001126	0.015601
1380021_at	310772	Dennd2d_p	DENN/MADD domain containing 2D (p	2.229146	0.001127	0.015601
1376755_at	24706	Rarb	retinoic acid receptor, beta	2.309679	0.001127	0.015601
1368888_a_at	83765	Rtn4	reticulon 4	-1.241378	0.001129	0.015601
1392974_at	294568	Wasf1	WAS protein family, member 1	-1.524793	0.001131	0.015601
1374799_at	362438	RGD15625	similar to mKIAA0159 protein (predicte	-2.36552	0.001131	0.015601
1389400_at	690911	LOC69091	NA	1.499974	0.001131	0.015601
1371128_at	287287	Il4	interleukin 4	1.798526	0.001133	0.015608
1372270_at	300668	H2afx	dolichyl-phosphate (UDP-N-acetylgluc	1.217629	0.001135	0.015608
1376571_at	363171	Tmem42_p	transmembrane protein 42 (predicted)	1.593947	0.001136	0.015608
1371970_at	499322	RGD15609	similar to expressed sequence AW413	-5.492173	0.001138	0.015608
1390423_at	290447	Phr1_predi	pam, highwire, rpm 1 (predicted)	1.72537	0.001139	0.015608
1388463_at	300988	Tex264	testis expressed gene 264 homolog (m	1.816456	0.00114	0.015608
1382061_at	307858	Ldhd	lactate dehydrogenase D	1.933342	0.00114	0.015608
1373326_at	295645	Slc4a10	solute carrier family 4, sodium bicarbo	2.545016	0.001141	0.015608
1367816_at	171160	Hod	homeobox only domain	-3.019763	0.001145	0.015643
1374797_at	306261	Eaf1_predi	ELL associated factor 1 (predicted)	2.077798	0.001149	0.015663
1388127_at	266761	Loc266761	cytochrome P450-like protein	2.306794	0.001149	0.015663
1371875_at	310864	Manba	mannosidase, beta A, lysosomal	2.072805	0.001152	0.015687
1398473_at	293938	Bloc1s2	biogenesis of lysosome-related organe	-1.423822	0.001153	0.015687
1378243_at	360985	Tmem17	transmembrane protein 17	-1.552568	0.001155	0.015688
1368314_at	81716	Ggcx	gamma-glutamyl carboxylase	1.212275	0.001155	0.015688
1371500_at	292734	Ltbp4	latent transforming growth factor beta I	2.940511	0.001162	0.01575
1373683_at	25150	Fyn	fyn proto-oncogene	-1.536104	0.001162	0.01575
1380990_at	362680	NA	NA	1.367468	0.001165	0.01577
1373823_at	498709	RGD1562C	similar to Cyclin-dependent kinases re	-5.067006	0.001167	0.015771
1379972_at	309790	RGD13086	similar to 1700060H10Rik protein	2.703111	0.001168	0.015771
1368472_at	83466	Celsr3	cadherin EGF LAG seven-pass G-type	1.262824	0.001169	0.015771
1376440_at	315000	Rnf139_pre	ring finger protein 139 (predicted)	-1.293029	0.001171	0.015771
1376569_at	306330	Klf2_predic	Kruppel-like factor 2 (lung) (predicted)	-6.185505	0.001176	0.015771
1387901_at	25529	Ptprd	protein tyrosine phosphatase, receptor	-2.415522	0.001177	0.015771
1370393_at	192228	Ccdc5	coiled-coil domain containing 5	-3.062121	0.001178	0.015771
1381177_at	140935	Afap	actin filament associated protein	2.16384	0.001178	0.015771
1382661_at	311182	Nup160_pr	nucleoporin 160 (predicted)	-1.207803	0.001178	0.015771
1368401_at	29627	Gria2	glutamate receptor, ionotropic, AMPA2	3.01562	0.001179	0.015771
1376281_at	500909	LOC50090	similar to hypothetical protein FLJ2234	2.35891	0.001179	0.015771
1370197_a_at	25522	Prkcz	protein kinase C, zeta	2.280941	0.001179	0.015771
1388326_at	293652	Ndufs8_pre	NADH dehydrogenase (ubiquinone) Fe	-1.7248	0.001184	0.015816
1377034_at	291091	Serpinb1a	serine (or cysteine) proteinase inhibito	4.100714	0.001185	0.015816
1376549_at	59320	Adcy10	adenylate cyclase 10 (soluble)	1.995479	0.001187	0.015825
1381127_at	500758	RGD1564C	similar to ubiquitin-conjugating enzyme	-3.736708	0.00119	0.015839
1384364_at	310363	Fbxo4_pre	F-box only protein 4 (predicted)	1.258374	0.001191	0.015839
1376992_a_at	315601	Foxr1_prec	forkhead box R1 (predicted)	1.440192	0.001193	0.015854
1372440_at	29366	Serpine2	serine (or cysteine) proteinase inhibito	2.09429	0.001197	0.015893
1387946_at	245955	Lgals3bp	lectin, galactoside-binding, soluble, 3 b	-1.689891	0.001202	0.015944
1392939_at	641603	Slc41a3	NA	-2.536159	0.001203	0.015945
1383679_at	364386	Rnf31_prec	ring finger protein 31 (predicted)	1.527942	0.001206	0.015965
1396797_at	292681	Nova2_pre	neuro-oncological ventral antigen 2 (pr	-2.17941	0.001208	0.01598
1398987_at	287608	Supt4h2_p	suppressor of Ty 4 homolog 2 (S. cere	-1.232809	0.001214	0.016042
1398777_at	29666	Psmb6	proteasome (prosome, macropain) sub	-1.195906	0.001219	0.01608
1371470_at	29646	Adh4	alcohol dehydrogenase 4 (class II), pi	-2.110278	0.00122	0.01608

1377121_at	305645	Dlg5_predi	discs, large homolog 5 (Drosophila) (p	-1.803685	0.001221	0.01608
1391269_at	317366	NA	NA	-1.459492	0.001225	0.016112
1398602_at	297176	Mad2l1_pr	MAD2 (mitotic arrest deficient, homolo	-4.429916	0.001226	0.016112
1387554_at	83627	Galnt5	UDP-N-acetyl-alpha-D-galactosamine:	2.722925	0.001229	0.016112
1386991_a_at	64639	Bad	bcl2-associated death promoter	-1.895632	0.00123	0.016112
1368344_at	24379	Gad1	glutamic acid decarboxylase 1	4.070185	0.00123	0.016112
1374502_at	289350	Rab3gap2	RAB3 GTPase activating protein subu	1.611404	0.001232	0.016112
1371385_at	288236	Dscr2_pre	Down syndrome critical region homolo	-1.379784	0.001233	0.016112
1388105_at	116656	D123	D123 gene product	-1.334998	0.001235	0.016112
1375529_at	359725	Cbr4	carbonic reductase 4	-1.909258	0.001238	0.016112
1385086_at	296137	Bub1_pred	budding uninhibited by benzimidazoles	-6.034458	0.001239	0.016112
1389282_at	360606	Itga3_pred	integrin alpha 3 (predicted)	2.31826	0.001239	0.016112
1369111_at	24360	Fabp1	fatty acid binding protein 1, liver	4.443469	0.001239	0.016112
1395379_at	302670	RGD15597	similar to U2af1-rs2 (predicted)	-1.319712	0.001239	0.016112
1392332_at	362291	Myt1_predi	myelin transcription factor 1 (predicted	-1.414262	0.00124	0.016112
1382066_at	362894	RGD1310C	similar to mKIAA1002 protein	1.406268	0.001247	0.016172
1379258_at	305351	Klhl5	kelch-like 5 (Drosophila)	-2.646274	0.001248	0.016172
1388556_at	60562	Stx6	syntaxin 6	1.421572	0.001249	0.016172
1389868_at	500988	RGD15645	similar to RCK (predicted)	2.16248	0.001251	0.016183
1390409_at	361774	RGD13061	LOC361774 (predicted)	1.350772	0.001255	0.016221
1377812_a_at	300666	Tmem24	transmembrane protein 24	1.252379	0.001256	0.016221
1371353_at	113894	Sqstm1	sequestosome 1	1.529012	0.001258	0.016234
1379284_at	315579	RGD13103	similar to RIKEN cDNA 2810457I06 (p	2.427632	0.00126	0.016238
1391602_at	305470	Drg1	developmentally regulated GTP bindin	2.232304	0.001261	0.01624
1368379_at	117106	Scarb2	scavenger receptor class B, member 2	1.424698	0.001268	0.016303
1371827_at	361712	Syvn1	synovial apoptosis inhibitor 1, synoviol	1.560859	0.001269	0.016303
1387169_at	84424	Tle3	transducin-like enhancer of split 3, E(s	1.903785	0.00127	0.016303
1374004_at	298771	RGD13101	similar to RIKEN cDNA D030028O16	-1.307706	0.001274	0.016304
1378906_at	499204	RGD15596	similar to hypothetical protein FLJ2541	-3.031502	0.001274	0.016304
1383684_at	304648	Asf1b_pre	ASF1 anti-silencing function 1 homolo	-2.769673	0.001276	0.016304
1367919_at	58958	Pom210	nuclear pore membrane glycoprotein 2	-1.719257	0.001276	0.016304
1398300_at	25390	Atp1b3	ATPase, Na+/K+ transporting, beta 3 p	1.443659	0.001277	0.016304
1374573_at	298767	Dync2li1	dynein cytoplasmic 2 light intermediate	-1.421288	0.001277	0.016304
1398356_at	291877	Cpsf5	cleavage and polyadenylation specific	-1.715463	0.001279	0.016309
1372860_at	361663	MGC95092	similar to phospholysine phosphohistic	-2.589882	0.001283	0.016326
1372209_at	312916	Cops5	COP9 (constitutive photomorphogenic	-1.292864	0.001284	0.016326
1398364_at	362626	RGD13595	similar to chromosome 1 open reading	1.911275	0.001286	0.016326
1376990_at	361044	RGD13093	similar to HCDI protein (predicted)	1.432153	0.001287	0.016326
1376799_a_at	290655	Crlf1_predi	cytokine receptor-like factor 1 (predicte	-2.249261	0.001289	0.016326
1377049_at	246246	Ntel1	neuropathy target esterase like 1	3.122348	0.001291	0.016326
1372798_at	654495	LOC65449	NA	-1.557414	0.001292	0.016326
1391619_at	501623	NA	NA	-1.496772	0.001295	0.016326
1378094_at	303953	RGD1308C	similar to hypothetical protein FLJ1134	1.475803	0.001295	0.016326
1376098_a_at	289785	Myo1g	myosin IG	1.205029	0.001295	0.016326
1378047_at	314711	Spic_predi	Spi-C transcription factor (Spi-1/PU.1 r	2.362479	0.001295	0.016326
1386604_at	114202	Mpp3	membrane protein, palmitoylated 3 (M.	-2.618765	0.001296	0.016326
1371451_at	364974	Rnaseh2a	ribonuclease H2, large subunit	-1.249325	0.001297	0.016326
1371365_at	292588	Ube2s_pre	ubiquitin-conjugating enzyme E2S (pre	-1.495112	0.001299	0.016326
1383721_at	266707	Cx39	connexin 39	2.027436	0.001299	0.016326
1373362_at	293448	Uqcrc2	ubiquinol cytochrome c reductase core	-1.242228	0.0013	0.016326
1392482_at	291565	RGD13115	similar to actin filament associated pro	-1.393302	0.001308	0.016367

1376810_at	361046	Adprtl1	ADP-ribosyltransferase (NAD+; poly (A	1.881285	0.001308	0.016367
1367973_at	24770	Ccl2	chemokine (C-C motif) ligand 2	5.847834	0.001309	0.016367
1384778_at	362653	Ctrc	chymotrypsin C (caldecrin)	4.187738	0.001309	0.016367
1370325_at	113961	Gorasp2	golgi reassembly stacking protein 2	1.447475	0.001309	0.016367
1370207_at	171082	Atp5g2	ATP synthase, H+ transporting, mitoch	-1.652797	0.001313	0.016391
1376583_at	307907	RGD13048	similar to RIKEN cDNA 6430548M08 (	2.104187	0.001314	0.016391
1389760_at	309457	Pcgf6	polycomb group ring finger 6	-1.647182	0.001317	0.016391
1390534_at	293967	Smc5l1_pr	SMC5 structural maintenance of chron	-1.516098	0.001318	0.016391
1370330_at	246212	Sipa1l1	signal-induced proliferation-associated	1.496942	0.001319	0.016391
1376175_at	498174	LOC49817	similar to NipSnap2 protein (Glioblasto	2.343533	0.00132	0.016391
1388397_at	114021	Ebna1bp2	EBNA1 binding protein 2	-1.22155	0.00132	0.016391
1370962_at	286894	Cipar1	castration induced prostatic apoptosis-	-1.536169	0.001322	0.016394
1376291_at	499412	NA	NA	-2.509907	0.001323	0.016394
1368430_at	63865	Lgmn	legumain	1.838558	0.001327	0.016407
1388136_at	171139	Timm9	translocase of inner mitochondrial mer	1.245161	0.001327	0.016407
1367583_at	116646	Tpt1	tumor protein, translationally-controlled	1.456313	0.001328	0.016407
1387435_at	25547	St8sia3	ST8 alpha-N-acetyl-neuraminide alpha	-2.157065	0.001329	0.016407
1376082_at	294924	Evi1_predi	ecotropic viral integration site 1 (predic	-2.717398	0.001331	0.016416
1382729_at	499783	RGD15640	similar to GTPase activating RANGAP	-1.971022	0.001332	0.016416
1379299_at	310071	Ercc8_prec	excision repair cross-complementing ro	-1.560444	0.001336	0.016439
1395748_at	362189	Slc12a6	solute carrier family 12, member 6	2.080742	0.001336	0.016439
1374491_at	301045	Cmtm8	CKLF-like MARVEL transmembrane do	-2.203497	0.00134	0.016474
1389738_at	304577	Ung	uracil-DNA glycosylase	-1.498079	0.001342	0.016476
1371416_at	293655	Ndufv1	NADH dehydrogenase (ubiquinone) fla	-1.367884	0.001349	0.016545
1375213_at	361042	Pck2_pred	phosphoenolpyruvate carboxykinase 2	1.894508	0.00135	0.016548
1373230_at	361068	Nudt18	nudix (nucleoside diphosphate linked r	1.428758	0.001352	0.016553
1390383_at	298199	Adfp	adipose differentiation related protein	2.480168	0.001355	0.016573
1387189_at	29504	Slc22a3	solute carrier family 22, member 3	1.143913	0.001356	0.016573
1386940_at	29543	Timp2	tissue inhibitor of metalloproteinase 2	-1.580404	0.00136	0.016587
1370013_at	85259	Cnga1	cyclic nucleotide gated channel alpha	-3.400614	0.001361	0.016587
1369637_at	85248	Kif3c	kinesin family member 3C	-1.234249	0.001362	0.016587
1372101_at	192270	Ppap2b	phosphatidic acid phosphatase type 2E	2.475248	0.001362	0.016587
1374594_at	363060	LOC36306	similar to RIKEN cDNA 1600029D21	-1.798279	0.001366	0.016621
1373357_at	313504	Foxd2_pre	forkhead box D2 (predicted)	-1.27533	0.001373	0.01668
1382797_at	296750	RGD15604	similar to 1500019C06Rik protein (pre	2.073092	0.001373	0.01668
1390846_at	366474	Col16a1	procollagen, type XVI, alpha 1	1.240974	0.001376	0.016689
1392669_at	306137	RGD13082	similar to CG10084-PA	1.510033	0.001377	0.016689
1385781_at	317252	RGD15657	similar to SNF2/RAD54 family protein (	-3.960107	0.001382	0.016742
1370538_at	286966	Lama3	laminin, alpha 3	5.306033	0.001388	0.016797
1398921_at	56281	Mrpl37	mitochondrial ribosomal protein L37	-1.71292	0.001389	0.016797
1387819_at	24331	Ela1	elastase 1, pancreatic	5.127737	0.001393	0.01682
1370059_at	83613	Nefl	neurofilament, light polypeptide	-2.118754	0.001394	0.016822
1376124_at	296936	Wasl	Wiskott-Aldrich syndrome-like (human)	3.747583	0.001397	0.016829
1367627_at	81660	Gatm	glycine amidinotransferase (L-arginine	-3.686452	0.001397	0.016829
1371363_at	60666	Gpd1	glycerol-3-phosphate dehydrogenase 1	-2.298184	0.0014	0.016829
1368382_at	114216	S100a3	S100 calcium binding protein A3	1.618787	0.001401	0.016829
1390218_at	362594	RGD13104	similar to hypothetical protein (predicte	-1.36868	0.001402	0.016829
1398659_at	501231	LOC50123	NA	1.889603	0.001402	0.016829
1373408_at	366995	Tbca	tubulin cofactor a	-1.156402	0.001404	0.016836
1378056_at	291137	Gmnn_prec	geminin (predicted)	-2.522733	0.001408	0.016857
1371872_at	89825	Nap1l1	nucleosome assembly protein 1-like 1	-1.528778	0.001408	0.016857

1393229_at	362818	Silv_predic	silver homolog (mouse) (predicted)	-1.638361	0.001413	0.016889
1378753_at	83497	Tpmt	thiopurine methyltransferase	-1.520935	0.001415	0.016889
1368889_at	65277	Vti1a	vesicle transport through interaction wi	1.16446	0.001416	0.016889
1381557_at	309242	Gna14	guanine nucleotide binding protein, alp	-1.709198	0.001418	0.016889
1371510_at	287097	Hcfc1r1	host cell factor C1 regulator 1 (XPO1-c	-1.226442	0.001419	0.016889
1372519_at	291874	Nup93	nucleoporin 93	-1.577304	0.001419	0.016889
1370113_at	78971	Birc3	baculoviral IAP repeat-containing 3	2.280436	0.001423	0.016895
1387828_at	65218	Centg1	centaurin, gamma 1	-1.281368	0.001426	0.016895
1368643_at	171413	Spata6	spermatogenesis associated 6	-1.96831	0.001426	0.016895
1398796_at	84599	Tmed10	transmembrane emp24-like trafficking	1.2964	0.001427	0.016895
1398769_at	29474	Coro1b	coronin, actin-binding protein, 1B	-1.193111	0.001431	0.016895
1368489_at	25445	Fosl1	fos-like antigen 1	4.619746	0.001432	0.016895
1375025_at	83506	Camkk2	calcium/calmodulin-dependent protein	-2.006677	0.001432	0.016895
1372557_at	363760	Arl6_predic	ADP-ribosylation factor-like 6 (predicte	-1.549354	0.001433	0.016895
1373750_at	297595	Leprel2_pr	leprecan-like 2 (predicted)	1.617742	0.001433	0.016895
1392996_at	293056	Cpeb1_pre	cytoplasmic polyadenylation element b	1.412895	0.001433	0.016895
1389116_at	282584	Mtmr9	myotubularin related protein 9	1.363899	0.001433	0.016895
1372015_at	306562	Tacc1	transforming, acidic coiled-coil contain	-3.2695	0.001435	0.016906
1367586_at	24533	Ldha	lactate dehydrogenase A	8.361187	0.00144	0.016942
1389025_at	309638	Taf11	TAF11 RNA polymerase II, TATA box	-1.603365	0.001443	0.016968
1368417_at	54309	Syt5	synaptotagmin V	-2.260761	0.001446	0.016979
1388703_at	300519	Esam	endothelial cell adhesion molecule	-1.36513	0.001453	0.017049
1371611_at	311215	Ext2_predi	exostoses (multiple) 2 (predicted)	-1.173757	0.001454	0.01705
1371660_at	360784	Znhit1_pre	zinc finger, HIT domain containing 1 (p	-1.479969	0.001455	0.01705
1383601_at	308720	Dufd1_pre	DUF729 domain containing 1 (predicte	-2.047084	0.001459	0.017057
1392107_at	314619	Stno_predi	strawberry notch homolog (Drosophila	1.587886	0.001459	0.017057
1368565_at	29483	Slc1a3	solute carrier family 1 (glial high affinity	3.036404	0.001461	0.017057
1390307_at	498430	RGD15654	similar to echinoderm microtubule asso	2.253068	0.001461	0.017057
1389209_at	315218	RGD13062	similar to hypothetical protein BC0029	-1.240719	0.001463	0.01706
1385522_at	313479	Orc1l	origin recognition complex, subunit 1-li	-2.626746	0.001469	0.017115
1375131_at	295922	Mtch2_pre	mitochondrial carrier homolog 2 (C. ele	-1.173178	0.00147	0.017115
1390699_at	309307	RGD13115	similar to KIAA2026 protein	-1.328366	0.001472	0.01712
1387018_at	114901	Argbp2	Arg/Abl-interacting protein ArgBP2	-1.773423	0.001474	0.017135
1379715_at	500042	RGD15662	similar to RIKEN cDNA 2610101N10 (	1.678157	0.001476	0.017135
1387161_at	84012	Slc1a6	solute carrier family 1 (high affinity asp	3.292396	0.001478	0.017137
1388488_at	297455	Lsm3_pred	LSM3 homolog, U6 small nuclear RNA	-1.15273	0.001479	0.017137
1389103_at	290842	RGD13073	similar to RIKEN cDNA 2810037C03	-2.035669	0.00148	0.017137
1371346_at	297990	Ndufb6_pre	NADH dehydrogenase (ubiquinone) 1	-1.156754	0.001481	0.017141
1387807_at	83572	Pafah1b1	platelet-activating factor acetylhydrolas	1.152057	0.001483	0.017143
1372145_at	294810	Tars	threonyl-tRNA synthetase	1.227778	0.001485	0.017143
1373146_at	308023	Ssx2ip	synovial sarcoma, X breakpoint 2 inter	1.226369	0.001485	0.017143
1371913_at	116487	Tgfbi	transforming growth factor, beta induce	-2.112793	0.001487	0.017143
1367532_at	300235	Dazap2	DAZ associated protein 2	-1.325268	0.001492	0.017194
1367850_at	116591	Fcgr3	Fc receptor, IgG, low affinity III	4.096852	0.001495	0.017206
1388802_at	501625	Bex1	brain expressed X-linked 1	-1.693744	0.0015	0.017251
1374459_at	313231	Alg2	asparagine-linked glycosylation 2 hom	1.197516	0.001506	0.017299
1372065_at	305235	Art3	ADP-ribosyltransferase 3	1.991646	0.001507	0.017299
1370999_at	252918	Spag5	sperm associated antigen 5	-6.895054	0.001509	0.017315
1389478_at	303792	Klhl22_pre	kelch-like 22 (Drosophila) (predicted)	1.600253	0.001514	0.017356
1371639_at	288666	Vps29_pre	vacuolar protein sorting 29 (S. pombe)	-1.188994	0.001521	0.017416
1392099_at	310463	Plcl3_predi	phospholipase C-like 3 (predicted)	1.803322	0.001523	0.017417

1382660_at	315912	RGD1563(	similar to FLJ20298 protein isoform a (	1.912852	0.001524	0.017417
1391625_at	360206	Asb15	ankyrin repeat and SOCS box-containi	1.339013	0.001525	0.017418
1383156_at	84391	Kif2	kinesin heavy chain family, member 2	-1.315574	0.001532	0.017485
1391410_at	290964	NA	NA	-1.870308	0.001536	0.01751
1374969_at	252886	Foxd4	forkhead box D4	2.214555	0.001538	0.017521
1388833_at	298098	Pole3	polymerase (DNA directed), epsilon 3	-1.262902	0.001543	0.017568
1373747_at	361705	Cnih2	cornichon homolog 2 (Drosophila)	-1.266756	0.001545	0.017569
1370313_at	26759	Acot7	acyl-CoA thioesterase 7	-1.78314	0.001546	0.017569
1388963_at	304900	Astn1	astrotactin 1	-1.583203	0.001552	0.017621
1388896_at	310960	Usp33	ubiquitin specific peptidase 33	1.270865	0.001555	0.017644
1379264_at	362367	Znrf1_pred	zinc and ring finger 1 (predicted)	-1.506919	0.001557	0.017645
1394699_at	307376	RGD15646	similar to transcription factor ONECUT	-1.997784	0.00156	0.017663
1379463_at	246776	Filip1	filamin A interacting protein 1	2.114488	0.001562	0.017669
1370295_at	191575	Nme1	expressed in non-metastatic cells 1	-1.42501	0.001564	0.017676
1381472_at	315023	Slc25a32_	solute carrier family 25, member 32 (pr	1.563677	0.00157	0.017701
1373668_at	292778	Polr2i_prec	polymerase (RNA) II (DNA directed) po	-1.156886	0.00157	0.017701
1388860_at	287302	Mrpl22_pre	mitochondrial ribosomal protein L22 (p	-1.60931	0.001571	0.017701
1376722_at	362335	Nup205_pr	nucleoporin 205kDa (predicted)	-1.204944	0.001572	0.017701
1374137_at	85424	Elf1	E74-like factor 1	2.282882	0.001572	0.017701
1397855_at	367181	RGD15651	similar to cysteine sulfinic acid decarbo	5.039537	0.00158	0.017776
1371486_at	361808	Snrp1c_pre	U1 small nuclear ribonucleoprotein 1C	-1.224812	0.001584	0.01778
1397611_at	501124	NA	NA	1.527705	0.001586	0.01778
1385616_a_at	294408	Asf1a_prec	ASF1 anti-silencing function 1 homolog	-1.306459	0.001588	0.01778
1399002_at	288621	Mrps17_pr	mitochondrial ribosomal protein S17 (p	-1.323736	0.001588	0.01778
1370007_at	116598	Pdia4	protein disulfide isomerase associated	1.769451	0.001589	0.01778
1377713_at	314374	Ches1_pre	checkpoint suppressor 1 (predicted)	1.361084	0.00159	0.01778
1387124_at	24504	Inha	inhibin alpha	1.749306	0.00159	0.01778
1370861_at	25282	Cox6a1	cytochrome c oxidase, subunit VIa, po	-1.209188	0.001594	0.017803
1387865_at	497778	Dut	deoxyuridine triphosphatase	-1.232899	0.001595	0.017803
1387212_at	25334	Bhlhb8	basic helix-loop-helix domain containir	2.359691	0.001599	0.017835
1388306_at	288513	RGD13055	similar to RIKEN cDNA 1810042K04 (p	-2.089127	0.001603	0.017866
1381957_at	291577	RGD15608	similar to chondroitin sulfate synthase	2.731598	0.001607	0.017867
1371284_at	362797	RGD15642	similar to immunoglobulin 4G6 heavy c	2.075313	0.001608	0.017867
1380265_at	314228	Snapc1_pr	small nuclear RNA activating complex,	1.166965	0.001611	0.017867
1378987_at	294560	LOC29456	NA	1.960742	0.001611	0.017867
1388339_at	364052	Pea15	phosphoprotein enriched in astrocytes	2.133208	0.001613	0.017867
1372552_at	289312	Acbd3	acyl-Coenzyme A binding domain cont	1.279227	0.001613	0.017867
1376687_at	313387	Usp1	ubiquitin specific peptdiase 1	-1.341254	0.001614	0.017867
1367744_at	113947	Maged2	melanoma antigen, family D, 2	-1.621726	0.001614	0.017867
1373295_at	294025	Obfc1	oligonucleotide/oligosaccharide-bindin	-4.649974	0.001617	0.017878
1372744_at	295625	Pkp4_pred	plakophilin 4 (predicted)	1.282042	0.001618	0.017878
1373803_a_at	25235	Ghr	growth hormone receptor	1.556142	0.00162	0.017884
1387495_at	25565	Tle4	transducin-like enhancer of split 4, E(s	-2.405789	0.001623	0.017908
1397745_at	307594	Mib1_predi	mindbomb homolog 1 (Drosophila) (pre	2.07368	0.001626	0.01791
1370913_at	65190	Best5	Best5 protein	3.966375	0.001626	0.01791
1376042_at	363460	Fgd1	FYVE, RhoGEF and PH domain conta	-1.154948	0.001629	0.017916
1380448_at	362766	Alkbh_prec	alkB, alkylation repair homolog (E. coli	1.727355	0.001631	0.017916
1393740_at	298545	Cnksr1	connector enhancer of kinase suppres	3.004841	0.001631	0.017916
1382711_at	498160	MGC11636	zinc finger protein 36 (KOX 18)	-1.747821	0.001634	0.017916
1372805_at	363015	RGD13104	LOC363015 (predicted)	-1.304346	0.001635	0.017916
1383117_at	282634	Pxmp4	peroxisomal membrane protein 4	-1.19025	0.001635	0.017916

1389576_at	362223	Snrpb2_pre	U2 small nuclear ribonucleoprotein B (	-1.766234	0.001642	0.017979
1373685_at	361149	RGD15657	similar to low density lipoprotein recep	-1.172891	0.001643	0.017979
1367945_at	84355	Atox1	ATX1 (antioxidant protein 1) homolog	-1.649988	0.001647	0.017988
1370611_at	25243	Arnt2	aryl hydrocarbon receptor nuclear tran	1.236315	0.001647	0.017988
1376986_at	361035	Mettl3	methyltransferase-like 3	1.835303	0.001648	0.017989
1394511_at	298247	Mysm1_pre	myb-like, SWIRM and MPN domains 1	2.542372	0.001654	0.018035
1369950_at	94201	Cdk4	cyclin-dependent kinase 4	-1.179761	0.001658	0.018045
1371701_at	299954	Ndufb9_pre	NADH dehydrogenase (ubiquinone) 1	-1.247442	0.001659	0.018045
1367576_at	24404	Gpx1	glutathione peroxidase 1	5.881859	0.001659	0.018045
1393459_at	24948	Fmr1	fragile X mental retardation syndrome	-1.357484	0.001661	0.018057
1367751_at	29640	Dpm2	dolichol-phosphate (beta-D) mannosyl	-1.431384	0.001665	0.01807
1379235_x_at	287961	LOC28796	NA	-2.322055	0.001665	0.01807
1386887_at	94194	Cox5b	cytochrome c oxidase subunit Vb	-1.17876	0.001668	0.018085
1389257_at	362375	Lancl2_pre	LanC (bacterial lantibiotic synthetase c	-1.512437	0.00167	0.018089
1368760_at	114105	Cxcl2	chemokine (C-X-C motif) ligand 2	5.209148	0.001673	0.0181
1390618_at	500131	RGD15661	similar to cAMP responsive element bi	3.440929	0.001674	0.0181
1376739_at	373065	Ddx24	DEAD (Asp-Glu-Ala-Asp) box polypept	1.452481	0.001677	0.018124
1376804_at	315840	RGD1560€	similar to Myosin VI (predicted)	1.666904	0.00168	0.018128
1371533_at	290798	Dctn6_pred	dynactin 6 (predicted)	-1.16054	0.00168	0.018128
1368057_at	25270	Abcd3	ATP-binding cassette, sub-family D (A	1.378993	0.001682	0.018128
1388363_at	296301	Raly	hnRNP-associated with lethal yellow	-1.487823	0.001685	0.018149
1373853_at	299647	RGD13081	similar to hypothetical protein MGC157	-1.202814	0.001693	0.0182
1368183_at	25738	Plcg1	phospholipase C, gamma 1	-1.196723	0.001694	0.0182
1378586_at	83681	Cish	cytokine inducible SH2-containing prot	-2.349805	0.001696	0.0182
1373812_at	83571	Cdkn1b	cyclin-dependent kinase inhibitor 1B	-1.581924	0.001698	0.0182
1385101_a_at	308060	RGD73510	similar to RIKEN cDNA 0610011N22 g	2.147354	0.001699	0.0182
1375170_at	445415	S100a11	S100 calcium binding protein A11 (cali	2.735745	0.0017	0.0182
1370862_at	25728	Apoe	apolipoprotein E	2.084692	0.001702	0.0182
1377742_at	366300	LOC36630	NA	1.446391	0.001702	0.0182
1368463_at	114111	Vegfc	vascular endothelial growth factor C	1.899025	0.001704	0.0182
1368801_at	83824	Cxxc4	CXXC finger 4	1.841151	0.001705	0.0182
1387037_at	80848	Cubn	cubilin (intrinsic factor-cobalamin recep	3.189269	0.001706	0.0182
1398870_at	266601	Tomm20	translocase of outer mitochondrial mer	1.150701	0.001706	0.0182
1372931_at	367743	Praf2_pred	PRA1 domain family 2 (predicted)	-1.871138	0.001709	0.0182
1392701_at	363145	Gmppb_pre	GDP-mannose pyrophosphorylase B (	1.572822	0.001711	0.0182
1369644_at	171447	Lphn2	latrophilin 2	-1.374153	0.001713	0.0182
1396939_at	116548	Pcsk5	proprotein convertase subtilisin/kexin t	3.922314	0.001713	0.0182
1367730_at	56042	Vdp	vesicle docking protein	1.365936	0.001714	0.0182
1378792_at	500633	RGD15640	similar to Kiaa0575 (predicted)	4.670976	0.001714	0.0182
1377267_at	317232	Magee1_pr	melanoma antigen, family E, 1 (predict	-1.595625	0.001717	0.0182
1375161_at	287356	Mrpl55_pre	mitochondrial ribosomal protein L55 (p	-1.230781	0.001717	0.0182
1372755_at	362911	Mal2	mal, T-cell differentiation protein 2	-1.197795	0.001719	0.0182
1381193_at	317456	LOC31745	hypothetical LOC317456	1.65701	0.00172	0.018202
1370054_at	54238	Cdkn2c	cyclin-dependent kinase inhibitor 2C (p	-1.697483	0.001724	0.01823
1377860_at	298185	RGD13069	similar to bM410K19.2.2 (novel protein	2.498893	0.001726	0.018238
1367961_at	24594	Ngfg	nerve growth factor, gamma	2.877236	0.001728	0.018238
1383445_at	360204	Zfp191	zinc finger protein 191	-1.596527	0.001729	0.018241
1370837_at	245917	Sycn	syncollin	3.283735	0.001733	0.018265
1387086_at	81715	Camlg	calcium modulating ligand	2.095188	0.001742	0.018337
1373254_at	450225	Krt10	keratin 10	-1.55474	0.001743	0.018337
1371782_at	313211	Nipsnap3a	nipsnap homolog 3A (C. elegans)	-1.569501	0.001745	0.018341

1371960_at	313053	Ythdf2_pre	YTH domain family 2 (predicted)	-1.886145	0.001746	0.018341
1371762_at	25703	Rbp4	retinol binding protein 4, plasma	4.091938	0.001747	0.018344
1383587_at	300783	RGD15654	similar to Butyrate-induced transcript 1	1.630522	0.001749	0.018348
1371514_at	361577	Kdelr1	KDEL (Lys-Asp-Glu-Leu) endoplasmic	1.109112	0.001752	0.018358
1373286_at	362650	Fblim1	filamin binding LIM protein 1	-1.602236	0.001756	0.018387
1386888_at	116636	Eif4ebp1	eukaryotic translation initiation factor 4	4.467015	0.00176	0.018421
1376073_at	314352	Sel1h	Sel1 (suppressor of lin-12) 1 homolog	1.453289	0.001768	0.018486
1371768_at	298681	Ssu72	Ssu72 RNA polymerase II CTD phospl	1.643505	0.001771	0.018508
1375977_at	84593	Cetn2	centrin 2	-1.356999	0.001775	0.018526
1373660_at	361436	Afg3l1_pre	AFG3(ATPase family gene 3)-like 1 (y	1.288691	0.001779	0.018562
1376388_at	286937	Pex2	peroxin 2	3.695774	0.001784	0.018565
1371357_at	289560	Igfbp7	insulin-like growth factor binding protei	1.433871	0.001785	0.018565
1384742_at	246284	Atrx	alpha thalassemia/mental retardation s	-2.071452	0.001788	0.018565
1373899_at	296190	RGD13098	similar to dJ842G6.1.1 (novel protein)	-1.137266	0.001789	0.018565
1374570_at	311821	Agpat2_pre	1-acylglycerol-3-phosphate O-acyltran	4.752223	0.001791	0.018565
1383848_at	24925	Adrb1	adrenergic receptor, beta 1	2.087206	0.001791	0.018565
1381514_at	307974	LOC30797	NA	2.42712	0.001791	0.018565
1387951_at	64036	Daf1	decay accelarating factor 1	-1.370923	0.001792	0.018565
1373982_at	287554	RGD13065	similar to hypothetical protein	-1.134433	0.001794	0.018565
1393646_at	297576	Dppa3	developmental pluripotency-associated	2.752321	0.001795	0.018565
1388898_at	288444	Hsph1	heat shock 105kDa/110kDa protein 1	2.103181	0.001795	0.018565
1389263_at	294804	Rai14	retinoic acid induced 14	-2.012933	0.001797	0.018565
1372039_at	287707	Nkiras2_pr	NFKB inhibitor interacting Ras-like pro	-1.688165	0.001799	0.018565
1385601_at	361156	Frg1_predi	FSHD region gene 1 (predicted)	-2.086536	0.001799	0.018565
1369777_a_at	171093	Shank2	SH3/ankyrin domain gene 2	-2.958226	0.001802	0.018582
1375904_at	289883	Tmed5	transmembrane emp24 protein transpo	1.216795	0.001806	0.018601
1388996_at	361351	RGD13085	similar to RIKEN cDNA 3110052N05	-1.135202	0.001808	0.018601
1370985_at	114509	Mapk7	mitogen-activated protein kinase 7	-2.927214	0.001808	0.018601
1372412_at	362667	Thap3_pre	THAP domain containing, apoptosis as	1.864326	0.001818	0.01861
1368977_a_at	84384	Fxc1	fractured callus expressed transcript 1	-1.24641	0.001818	0.01861
1378492_at	306183	Farp1_pred	FERM, RhoGEF (Arhgef) and pleckstri	2.245236	0.001819	0.01861
1370854_at	246172	Nexn	nexilin	2.168266	0.00182	0.01861
1367686_at	80881	RAMP4	ribosome associated membrane protei	1.115848	0.00182	0.01861
1368806_at	29360	Sepp1	selenoprotein P, plasma, 1	-2.327764	0.001822	0.01861
1373767_at	360772	Zfand2a	zinc finger, AN1-type domain 2A	1.863478	0.001823	0.01861
1373049_at	501195	RGD15621	similar to D1Ertd622e protein (predicte	-1.106415	0.001824	0.01861
1388344_at	362819	RGD13056	similar to RIKEN cDNA 1110005A23	-1.201399	0.001825	0.01861
1369888_at	24952	Gcg	glucagon	3.215059	0.001825	0.01861
1385142_at	499365	NA	NA	3.338669	0.001826	0.01861
1392732_at	311336	Nusap1_pr	nucleolar and spindle associated prote	-6.159232	0.001826	0.01861
1367654_at	83720	Fath	fat tumor suppressor homolog (Drosop	-1.577841	0.001829	0.01863
1389423_at	83573	Ddr2	discoidin domain receptor family, mem	2.789904	0.001836	0.018679
1389781_at	315670	Elmod1_pr	ELMO domain containing 1 (predicted)	-2.902703	0.001838	0.018691
1375415_at	366468	LOC36646	NA	-1.277644	0.001843	0.018723
1376366_at	295217	Snapap	SNAP-associated protein	-1.148685	0.001846	0.018739
1389973_a_at	619346	Surf4	surfeit 4	1.514653	0.001851	0.018763
1373427_at	297960	Rragd_pred	Ras-related GTP binding D (predicted)	1.684606	0.001853	0.018763
1389885_at	360646	Limd2	LIM domain containing 2	-1.312235	0.001853	0.018763
1399054_at	498138	NA	NA	-1.221007	0.001854	0.018763
1368453_at	83512	Fads2	fatty acid desaturase 2	-3.417552	0.001856	0.018772
1387987_at	286961	Slc22a19	solute carrier family 22 (organic anion	1.622479	0.001859	0.018782

1370473_a_at	117552	Ptpn23	protein tyrosine phosphatase, non-rece	1.783865	0.00186	0.018782
1378740_at	304893	Rasal2_pre	RAS protein activator like 2 (predicted)	1.244016	0.001864	0.018807
1367484_at	361013	Ube2e2	ubiquitin-conjugating enzyme E2E 2 (U	-1.154872	0.00187	0.018852
1369410_at	94189	Gosr1	golgi SNAP receptor complex member	1.764176	0.001873	0.018872
1391698_at	353118	MAST1	microtubule associated serine/threonin	-3.142521	0.001876	0.018879
1382949_at	314264	Rdh12_pre	retinol dehydrogenase 12 (predicted)	4.684606	0.001878	0.018879
1374100_at	500243	RGD15608	similar to 2300003P22Rik protein (pred	-1.141956	0.001878	0.018879
1384115_at	192272	Mte1	mitochondrial acyl-CoA thioesterase 1	1.807045	0.001884	0.01892
1371168_at	85275	Mpp2	membrane protein, palmitoylated 2 (MA	-1.798637	0.001887	0.018923
1382553_at	361746	Rfx3	regulatory factor X, 3 (influences HLA c	1.352084	0.001887	0.018923
1383689_at	303923	B4galt4	UDP-Gal:betaGlcNAc beta 1,4-galacto	2.676177	0.001889	0.018923
1386962_at	25031	Plcb4	phospholipase C, beta 4	-1.343879	0.00189	0.018923
1369175_a_at	25376	Ambn	ameloblastin	2.833813	0.001892	0.018926
1375280_at	289277	PNAS-4	CGI-146 protein	1.125375	0.001893	0.018926
1369412_a_at	29723	Slc19a1	solute carrier family 19, member 1	-2.084186	0.001896	0.018927
1373758_at	113917	Lenep	lens epithelial protein	1.489851	0.001896	0.018927
1367861_at	79115	Evl	Ena-vasodilator stimulated phosphopro	-1.31534	0.001898	0.018932
1375163_at	79434	Rab11b	RAB11B, member RAS oncogene fam	-1.313176	0.001901	0.01895
1383920_at	306586	Amt	aminomethyltransferase (glycine cleav	3.77385	0.001905	0.01898
1374938_at	363059	Zw10	ZW10 homolog, centromere/kinetocho	1.121232	0.001912	0.019035
1388330_at	309004	Vkorc1	vitamin K epoxide reductase complex,	-1.078861	0.001914	0.019041
1377644_at	291925	RGD13087	similar to RIKEN cDNA 4921524J17 (p	1.893447	0.001916	0.019044
1371991_at	298201	RGD13112	similar to RIKEN cDNA B230312A22	2.033269	0.00192	0.019065
1377041_at	315119	Mfng	manic fringe homolog (Drosophila)	2.642355	0.001923	0.01908
1389409_at	500040	LOC50004	similar to Testis derived transcript	1.402942	0.001927	0.019107
1382419_at	294712	Solt_predic	SoxLZ/Sox6 leucine zipper binding pro	-3.500081	0.00193	0.01911
1398306_at	25028	Ampd1	adenosine monophosphate deaminase	2.409604	0.00193	0.01911
1369879_a_at	24822	Tegt	testis enhanced gene transcript	1.317096	0.001932	0.01911
1367647_at	24648	Serpina1	serine (or cysteine) proteinase inhibito	2.402556	0.001937	0.019153
1394966_at	310812	RGD13095	similar to hypothetical protein FLJ2030	-4.15505	0.00194	0.019162
1373144_at	499654	NA	NA	1.800943	0.001941	0.019162
1393216_at	64018	Slc33a1	solute carrier family 33 (acetyl-CoA tra	1.391512	0.001943	0.019171
1379744_at	365245	Saa4	serum amyloid A 4	1.454408	0.001947	0.01918
1374884_at	287585	Ppm1d_pre	protein phosphatase 1D magnesium-d	-1.435287	0.001948	0.01918
1368550_at	64826	Foxq1	forkhead box Q1	2.662307	0.001949	0.01918
1391205_at	304332	MGC12521	similar to hypothetical protein FLJ2039	-1.701003	0.001952	0.019196
1369948_at	117089	Ngfrap1	nerve growth factor receptor (TNFRSF	-1.33266	0.001953	0.019196
1393559_at	81801	Stc1	stanniocalcin 1	-4.596615	0.001958	0.019206
1379243_at	315167	Ndufa6_pre	NADH dehydrogenase (ubiquinone) 1	-1.112461	0.00196	0.019206
1391621_at	314818	E2f7_predi	E2F transcription factor 7 (predicted)	1.345883	0.00196	0.019206
1370248_at	63847	Fxyd6	FXYD domain-containing ion transport	-2.597597	0.00196	0.019206
1387916_at	266689	Cyp4f6	cytochrome P450 4F6	2.268913	0.001964	0.019227
1398691_at	499624	LOC49962	NA	2.454311	0.001966	0.019238
1390386_at	25402	Casp3	caspase 3, apoptosis related cysteine	-1.926039	0.001975	0.019305
1374292_at	303749	RGD1305C	similar to RIKEN cDNA 1110031I02	-1.79106	0.001978	0.019326
1372627_at	290408	Sugt1	SGT1, suppressor of G2 allele of SKP	1.279578	0.001981	0.01934
1398983_at	301352	Mrpl30_pre	mitochondrial ribosomal protein L30 (p	-1.082076	0.001989	0.019388
1383686_at	29205	Syngr1	synaptogyrin 1	-2.661265	0.00199	0.019388
1375206_at	287466	RGD13082	hypothetical LOC287466	-1.654118	0.001994	0.019388
1387904_at	50560	Ihpk1	inositol hexaphosphate kinase 1	1.187661	0.001994	0.019388
1390058_at	361990	LOC36199	similar to DKFZP547E1010 protein	-1.130987	0.001995	0.019388

1373890_at	296147	Chchd5_pr	coiled-coil-helix-coiled-coil-helix domai	-1.629605	0.001995	0.019388
1373383_at	299514	Mterfd1	MTERF domain containing 1	-1.158345	0.001999	0.019414
1385397_at	499991	LOC49999	Ab1-219	1.977021	0.002	0.019414
1385132_at	297783	Mybl1_pre	myeloblastosis oncogene-like 1 (predic	-4.229583	0.002003	0.01943
1394334_at	308511	Chst8_pre	carbohydrate (N-acetylgalactosamine	-2.422175	0.002009	0.019474
1377777_at	308067	Brd9_predi	bromodomain containing 9 (predicted)	-1.348524	0.002011	0.019475
1390464_at	252858	Frmd4b	FERM domain containing 4B	1.861579	0.002013	0.019478
1384606_at	287111	NA	NA	-4.404101	0.002015	0.019489
1387872_at	29578	Hnrpa1	heterogeneous nuclear ribonucleoprot	-1.685444	0.002017	0.01949
1379479_at	84393	Kif4	kinesin family member 4	-3.727695	0.002027	0.019553
1383396_at	306022	Fndc3a_pr	fibronectin type III domain containing 3	1.051547	0.002027	0.019553
1371745_at	300361	RGD1310€	hypothetical LOC300361	-1.083845	0.002029	0.019553
1384544_at	312086	Pon3	paraoxonase 3	5.210729	0.002029	0.019553
1368325_at	25313	Egf	epidermal growth factor	1.586828	0.002031	0.019557
1383279_at	290363	Rcbtb2	regulator of chromosome condensatio	3.736291	0.002033	0.019557
1386518_at	290749	Irf2_predic	interferon regulatory factor 2 (predicte	2.715558	0.002034	0.019559
1399035_at	300088	Polr3h_pre	polymerase (RNA) III (DNA directed) p	-1.190736	0.002038	0.019573
1369030_at	24691	Prss1	pancreatic trypsin 1	4.641436	0.00204	0.019573
1377673_at	500715	LOC50071	NA	-1.30911	0.00204	0.019573
1372815_at	298385	Magoh_pre	mago-nashi homolog, proliferation-ass	-1.136258	0.002044	0.019587
1373205_at	361542	U2af1l4	U2 small nuclear RNA auxiliary factor	-1.677245	0.002045	0.019587
1368554_at	25702	Pnlip	pancreatic lipase	4.175384	0.002048	0.019596
1398853_at	29676	Psmb3	proteasome (prosome, macropain) sul	-1.119369	0.002049	0.019596
1393635_x_at	29213	Tubb4	tubulin, beta 4	-1.848551	0.002058	0.019665
1383579_at	502282	NA	NA	-1.397113	0.002059	0.019665
1370107_at	117184	Ctrl	chymotrypsin-like	3.254423	0.002062	0.019682
1384971_at	295971	RGD13097	similar to Hypothetical protein MGC19	1.603748	0.002064	0.019686
1396763_at	301455	LOC30145	NA	1.455957	0.002066	0.019686
1371418_at	299809	Cct2	chaperonin containing TCP1, subunit 2	-1.155759	0.002068	0.019691
1386550_at	309031	Zfpn1a5_p	zinc finger protein, subfamily 1A, 5 (pre	-1.29088	0.002074	0.019725
1376676_a_at	290270	RGD13051	similar to Ab2-008	1.178394	0.002075	0.019725
1378349_at	304872	NA	NA	4.189872	0.002078	0.019725
1377687_at	367838	RGD15617	similar to cDNA sequence AK129302 (	-1.651123	0.002079	0.019725
1379565_at	362339	Creb3l2	cAMP responsive element binding prot	1.502036	0.002081	0.019725
1374467_at	287069	Trap1	TNF receptor-associated protein 1	-1.152878	0.002081	0.019725
1398366_at	497968	NA	NA	-1.360454	0.002082	0.019725
1371931_at	353256	Gtf2i	general transcription factor II I	-1.086883	0.002084	0.019738
1367741_at	85430	Herpud1	homocysteine-inducible, endoplasmic	1.684747	0.002091	0.019783
1384411_at	296884	LOC29688	hypothetical LOC296884	1.795666	0.002094	0.019799
1391064_at	308865	Atg16l2_pr	ATG16 autophagy related 16-like 2 (S.	1.120946	0.002096	0.0198
1372781_at	310785	Wdr47	WD repeat domain 47	-1.293552	0.002098	0.0198
1387146_a_at	50672	Ednrb	endothelin receptor type B	1.891041	0.002098	0.0198
1387462_at	24260	Chrm3	cholinergic receptor, muscarinic 3	1.418109	0.002101	0.019806
1371899_at	311130	Prkra	protein kinase, interferon inducible dou	-1.447749	0.002103	0.019814
1371712_at	292030	NA	NA	-1.462977	0.002108	0.019844
1367938_at	83472	Ugdh	UDP-glucose dehydrogenase	2.114432	0.00211	0.019849
1374333_at	361224	RGD1306C	similar to RIKEN cDNA 1110007C09 (	-1.203765	0.002111	0.019849
1376285_at	314543	Gulp1	GULP, engulfment adaptor PTB doma	-1.494286	0.002114	0.019862
1379582_a_at	114494	Ccna2	cyclin A2	-5.4939	0.002116	0.019869
1375776_at	295062	MGC11609	transcriptional co-activator with PDZ-b	3.373861	0.002119	0.019884
1373288_at	308944	St5_predic	suppression of tumorigenicity 5 (predic	1.612472	0.002122	0.019886

1392131_at	499933	LOC49993	similar to RIKEN cDNA 1700022L09	-1.521252	0.002123	0.019886
1388920_at	25644	Bmp6	bone morphogenetic protein 6	3.140958	0.002127	0.019898
1371332_at	364716	Hist1h4a_p	histone 1, H4a (predicted)	1.434306	0.002127	0.019898
1397551_at	362274	Ddx27	DEAD (Asp-Glu-Ala-Asp) box polypept	-1.074246	0.002128	0.019898
1375989_a_at	309452	Nfkb2	nuclear factor of kappa light polypeptic	3.119105	0.002132	0.019909
1398864_at	64631	Ube2g1	ubiquitin-conjugating enzyme E2G 1 (U	-1.079872	0.002133	0.019909
1370072_at	24590	Mme	membrane metallo endopeptidase	-3.599165	0.002138	0.019925
1388443_at	360804	Cdk2ap1_p	CDK2 (cyclin-dependent kinase 2)-ass	-1.159169	0.00214	0.019925
1368717_at	29347	Faah	fatty acid amide hydrolase	-1.526582	0.00214	0.019925
1399079_at	298617	RGD13078	similar to KIAA1096 protein (predicted)	1.879335	0.002141	0.019925
1368126_at	65984	Aacs	acetoacetyl-CoA synthetase	-1.235633	0.002143	0.019925
1396420_at	368002	Baz1b	NA	2.729776	0.002145	0.019925
1396272_at	361292	NA	NA	-2.513091	0.002145	0.019925
1374706_at	29454	Gdf11	growth differentiation factor 11	-1.410447	0.00215	0.019952
1374114_at	309453	Fbxl15_pre	F-box and leucine-rich repeat protein 1	-1.318173	0.002151	0.019952
1375490_at	498130	RGD15648	similar to Serologically defined colon c	4.156711	0.00216	0.020021
1373820_at	360860	RGD13047	similar to constitutive photomorphogen	1.371808	0.002161	0.020021
1373532_at	308543	Plekhf1	pleckstrin homology domain containing	2.689315	0.002164	0.020031
1376805_at	304850	Rnf2	ring finger protein 2	-1.232716	0.002166	0.020037
1380548_at	303968	Ift57_predi	intraflagellar transport 57 homolog (Ch	-1.121373	0.002168	0.020037
1368510_at	25172	Gata1	GATA binding protein 1	3.044213	0.002171	0.020059
1372786_at	287150	RGD13061	similar to hypothetical protein MGC249	1.059918	0.002176	0.020079
1398840_at	89818	Vamp5	vesicle-associated membrane protein 5	1.163452	0.002177	0.020079
1368199_at	113929	Nup88	nucleoporin 88	-1.112846	0.002181	0.020101
1389181_at	303141	Rapgef6_p	Rap guanine nucleotide exchange fact	1.043247	0.002182	0.020101
1398810_at	64527	Pdap1	PDGFA associated protein 1	-1.210192	0.00219	0.020158
1389918_at	290704	LOC29070	NA	1.151388	0.002192	0.020162
1367902_at	64199	Gng11	guanine nucleotide binding protein (G	3.329611	0.002203	0.020248
1368217_at	84014	Ralbp1	ralA binding protein 1	1.046054	0.002204	0.020248
1367683_at	85245	Kpna2	karyopherin (importin) alpha 2	-1.893744	0.002206	0.020248
1367949_at	29237	Penk-rs	preproenkephalin, related sequence	4.970494	0.002207	0.020248
1371682_at	362245	Map1lc3a	microtubule-associated protein 1 light	1.148198	0.002209	0.020255
1372611_at	298377	RGD13052	similar to RIKEN cDNA 2010305A19 (p	-2.391075	0.002212	0.020264
1392936_at	497865	RGD15654	similar to RNA binding motif protein 25	1.080011	0.002216	0.020283
1386895_at	84469	Maged1	melanoma antigen, family D, 1	1.062549	0.002219	0.020299
1372173_at	313056	RGD13091	similar to U5 snRNP-specific protein (F	-1.392474	0.002221	0.020299
1379409_at	361015	RGD15626	similar to spinocerebellar ataxia 7 hom	2.779063	0.002222	0.020299
1387499_a_at	64013	Pdcl	phosducin-like	-1.250384	0.002225	0.020299
1375624_at	300177	Irak4_pred	interleukin-1 receptor-associated kinas	3.846466	0.002226	0.020299
1382232_at	307759	Gpt2_predi	glutamic pyruvate transaminase (alanin	2.004511	0.002227	0.020299
1376418_a_at	306804	Iars_predic	isoleucine-tRNA synthetase (predicted	1.262445	0.00223	0.020299
1370499_at	362443	Klrb1a_ma	killer cell lectin-like receptor subfamily	5.341993	0.002232	0.020299
1367679_at	25599	Cd74	CD74 antigen (invariant polypeptide of	2.54438	0.002232	0.020299
1369906_s_at	246117	Mcfd2	multiple coagulation factor deficiency 2	2.598234	0.002233	0.020299
1370224_at	25125	Stat3	signal transducer and activator of trans	1.354775	0.002239	0.020343
1388731_at	291403	Zadh2_pre	zinc binding alcohol dehydrogenase, d	-1.560927	0.002244	0.020363
1373723_at	300027	RGD15642	similar to high density lipoprotein-bindi	2.155889	0.002247	0.020363
1388362_at	290671	Ndufa13_p	NADH dehydrogenase (ubiquinone) 1	-1.142355	0.002247	0.020363
1372861_at	314641	Pip5k1c	phosphatidylinositol-4-phosphate 5-kin	-1.234676	0.002247	0.020363
1385078_at	361095	Zic5_predi	zinc finger protein of the cerebellum 5	4.218912	0.002249	0.020363
1390531_at	296474	RGD1306C	similar to Peroxisomal proliferator-activ	2.161197	0.002252	0.020377

1373989_at	363140	Rassf1	Ras association (RalGDS/AF-6) doma	1.306264	0.002256	0.0204
1367696_at	114709	Ifitm2	interferon induced transmembrane pro	-1.700613	0.002259	0.020416
1374551_at	287719	Ifi35	interferon-induced protein 35	-1.517031	0.002274	0.020533
1393094_at	498883	NA	NA	1.523776	0.002275	0.020533
1398957_at	315362	RGD15663	similar to cell division cycle 2-like 1 (pr	1.435476	0.002279	0.020548
1374751_at	24884	Yes1	Yamaguchi sarcoma viral (v-yes) onco	5.616439	0.002284	0.02057
1390146_at	360916	RGD13061	similar to RIKEN cDNA 2610318G18 (	1.200099	0.002284	0.02057
1390047_at	361293	Riok3_prec	RIO kinase 3 (yeast) (predicted)	1.772525	0.002287	0.020571
1393655_at	298429	Rad54l_pre	RAD54 like (S. cerevisiae) (predicted)	-3.845074	0.002287	0.020571
1370386_at	65137	Ruvbl1	RuvB-like protein 1	-1.7192	0.00229	0.020583
1367526_at	362216	Mrps26_pr	mitochondrial ribosomal protein S26 (p	-1.158319	0.002294	0.020601
1376311_at	295382	RGD15634	similar to netrin G1 (predicted)	1.686075	0.002298	0.020628
1383785_at	161452	Lef1	lymphoid enhancer binding factor 1	2.276944	0.002301	0.02064
1370266_at	57341	Parva	parvin, alpha	-2.658242	0.002313	0.020728
1383806_at	361249	RGD15663	similar to BAF57 (predicted)	-1.651776	0.002314	0.020728
1383774_at	289864	RGD13081	similar to RIKEN cDNA 2010316F05 (p	1.118378	0.002321	0.020774
1391063_at	315740	Kif23_pred	kinesin family member 23 (predicted)	-3.306978	0.002323	0.020774
1387025_at	29564	Dync1i1	dynein cytoplasmic 1 intermediate cha	-2.881451	0.002324	0.020778
1368067_at	58820	Zfp148	zinc finger protein 148	-1.005904	0.002326	0.020779
1370084_at	24271	Cpb1	carboxypeptidase B1 (tissue)	5.220574	0.002332	0.020808
1399123_at	361057	Ddx26	DEAD/H (Asp-Glu-Ala-Asp/His) box pc	1.484765	0.002333	0.020808
1380810_at	499061	NA	NA	1.644354	0.002339	0.020847
1393641_at	499356	Blnk	B-cell linker	1.611895	0.002342	0.020866
1388160_a_at	94173	Idh3B	isocitrate dehydrogenase 3 (NAD+) be	-1.098721	0.002345	0.020875
1371783_at	290549	LOC29054	selenoprotein K	1.244239	0.00235	0.020904
1387179_at	29241	Adcy8	adenylate cyclase 8	-1.320989	0.002355	0.020941
1383752_at	314969	Nol1_predi	nucleolar protein 1 (predicted)	1.017537	0.002358	0.020951
1379262_at	302640	LOC30264	similar to acyl-CoA thioesterase	-1.471641	0.002365	0.020997
1377594_at	85385	Shc1	src homology 2 domain-containing trar	1.267555	0.002367	0.021005
1388417_at	288671	Anapc5_pr	anaphase-promoting complex subunit	-1.030062	0.002383	0.021104
1392449_at	362412	Rad18_pre	RAD18 homolog (S. cerevisiae) (predi	-1.665557	0.002383	0.021104
1368005_at	25679	Itpr3	inositol 1,4,5-triphosphate receptor 3	1.277171	0.002385	0.021104
1374412_at	501682	NA	NA	-1.080735	0.002385	0.021104
1372067_at	362751	Txndc1	thioredoxin domain containing 1	1.031084	0.002387	0.021104
1368911_at	25472	Kcnj8	potassium inwardly-rectifying channel,	3.796525	0.002391	0.021129
1371764_at	298689	MGC94113	similar to Ubc6p homolog	1.175501	0.002397	0.02117
1375220_at	367454	LOC36745	NA	-1.182406	0.002399	0.02117
1391255_at	287457	Zmynd15_p	zinc finger, MYND domain containing 1	1.840231	0.002409	0.02125
1399063_at	313658	Rbaf600	ZUBR1	2.792899	0.002416	0.021286
1369241_at	29462	Avpr1b	arginine vasopressin receptor 1B	3.118785	0.002417	0.021286
1373476_at	500251	LOC50025	hypothetical protein LOC500251	-1.322382	0.002418	0.021286
1372327_at	362207	Myef2	myelin basic protein expression factor	-1.807375	0.002427	0.021314
1384627_at	501765	RGD15595	similar to RIKEN cDNA 2310057N15 (p	1.323371	0.002429	0.021314
1392990_at	312936	Sox17_pre	SRY-box containing gene 17 (predicte	2.816435	0.002429	0.021314
1388867_at	361178	MGC11283	similar to transcription factor	-1.171739	0.002431	0.021314
1385660_at	314388	RGD1309C	similar to KIAA2010 protein (predicted)	1.288425	0.002432	0.021314
1372510_at	296271	Srxn1	sulfiredoxin 1 homolog (S. cerevisiae)	2.606472	0.002433	0.021314
1374506_at	311835	Ddx31_pre	DEAD/H (Asp-Glu-Ala-Asp/His) box pc	1.242922	0.002433	0.021314
1379150_at	303073	Cyfip2_pre	cytoplasmic FMR1 interacting protein 2	4.245265	0.002434	0.021314
1395376_at	316767	Ddx11_pre	DEAD/H (Asp-Glu-Ala-Asp/His) box pc	-3.426666	0.002438	0.021332
1391706_at	501168	NA	NA	1.434986	0.002441	0.021341

1371374_at	315093	Maf1	MAF1 homolog (S. cerevisiae)	-1.607915	0.002447	0.021377
1374580_at	300860	Senp6_pre	SUMO/sentrin specific peptidase 6 (pr	1.165601	0.00245	0.021377
1373819_at	500855	LOC50085	hypothetical protein LOC500855	-1.085115	0.002451	0.021377
1375859_a_at	308488	RGD1560€	similar to zinc finger protein 565 (predi	2.111171	0.002453	0.021377
1387874_at	24309	Dbp	D site albumin promoter binding protei	-1.51891	0.002454	0.021377
1383431_at	305579	RGD13051	similar to KIAA1841 protein (predicted)	-1.405743	0.002457	0.021377
1374289_at	301246	Rpo1-1	RNA polymerase 1-1	-1.05268	0.002458	0.021377
1370464_at	170913	Abcb1a	ATP-binding cassette, sub-family B (M	4.153048	0.002459	0.021377
1378477_a_at	291964	LOC29196	NA	1.970209	0.002461	0.021377
1375516_at	293130	Ndufc2	NADH dehydrogenase (ubiquinone) 1,	-1.30578	0.002461	0.021377
1373371_a_at	500087	MGC1128§	similar to RIKEN cDNA 1110001J03	-1.300055	0.002465	0.021379
1373160_at	498653	NA	NA	1.043459	0.002466	0.021379
1388186_at	286911	LOC28691	cationic trypsinogen	3.060232	0.002467	0.021379
1371963_at	54280	Pcca	propionyl-coenzyme A carboxylase, al	1.729154	0.00247	0.021379
1389567_at	301024	Scap_pred	SREBP cleavage activating protein (pr	1.280967	0.002472	0.021379
1368060_at	65151	Hrsp12	heat-responsive protein 12	-0.995733	0.002476	0.021379
1375119_at	25489	Nedd4a	neural precursor cell expressed, devel	1.074884	0.002478	0.021379
1371371_at	288088	Ndufb4	NADH dehydrogenase (ubiquinone) 1	-1.096094	0.002479	0.021379
1389010_at	299732	Lta4h	leukotriene A4 hydrolase	-1.109501	0.00248	0.021379
1390358_at	306243	Cacna2d3	calcium channel, voltage-dependent, a	-2.451838	0.002481	0.021379
1390105_at	313536	B4galt2_pr	UDP-Gal:betaGlcNAc beta 1,4- galact	-1.327555	0.002481	0.021379
1388873_at	316575	Trip12	thyroid hormone receptor interactor 12	1.272247	0.002482	0.021379
1373824_at	292027	Cfdp1	craniofacial development protein 1	1.004359	0.002484	0.021379
1369157_at	29516	Pde3b	phosphodiesterase 3B	-1.537395	0.002485	0.021379
1372331_at	302697	RGD15601	similar to eukaryotic translation initiatic	-1.468463	0.002485	0.021379
1372541_at	361728	MGC7300З	Unknown (protein for MGC:73003)	-1.811456	0.002494	0.021442
1387087_at	24253	Cebpb	CCAAT/enhancer binding protein (C/E	1.116899	0.002509	0.02155
1367752_at	25414	Bcar1	breast cancer anti-estrogen resistance	1.930944	0.002513	0.021574
1382085_at	363289	Mterfd2	MTERF domain containing 2	1.132132	0.002515	0.021574
1385229_at	306081	Pcdh20_pr	protocadherin 20 (predicted)	-3.099236	0.002525	0.021651
1398363_at	500226	RGD15614	similar to D3Mm3e (predicted)	-1.51041	0.002528	0.021657
1375436_at	315085	Naprt1	nicotinate phosphoribosyltransferase c	2.500105	0.002529	0.021657
1372305_at	360611	Copz2_pre	coatomer protein complex, subunit zet	1.789594	0.002531	0.021657
1371758_at	445268	Ufc1	Ufm1-conjugating enzyme 1	0.990635	0.002532	0.021657
1388394_at	292023	Aars	alanyl-tRNA synthetase	1.576939	0.002534	0.021657
1387932_at	25550	Slc1a1	solute carrier family 1 (neuronal/epithe	-1.767979	0.002539	0.021679
1382905_at	498011	Endo180	collagen-binding factor Endo180	-2.384976	0.00254	0.021679
1367795_at	29596	Ifrd1	interferon-related developmental regul	1.29239	0.00255	0.021738
1371684_at	294754	Pelo	pelota homolog	1.325976	0.00255	0.021738
1373420_at	300447	MGC9470	evolutionarily conserved signaling inte	1.124463	0.002555	0.021752
1384117_at	499363	RGD1564	similar to 9130011E15Rik protein (pre	-1.510005	0.002556	0.021752
1391581_at	299012	Arhgap5	Rho GTPase activating protein 5	2.09416	0.002557	0.021752
1371591_at	301249	Mrps18a	mitochondrial ribosomal protein S18A	-1.861126	0.002572	0.021865
1368700_at	84587	Plcl1	phospholipase C-like 1	-2.407546	0.002574	0.021873
1388331_at	362862	Tra1_predi	tumor rejection antigen gp96 (predicte	1.30551	0.002584	0.021931
1382653_at	362763	RGD13067	similar to CG15929-PA (predicted)	2.082975	0.002584	0.021931
1398084_at	501026	LOC50102	NA	3.276814	0.002588	0.021951
1393091_at	310086	RGD13112	similar to RIKEN cDNA D130064H19 (	-1.076949	0.002595	0.021976
1368799_at	64041	Birc5	baculoviral IAP repeat-containing 5	-3.940337	0.002598	0.021976
1373911_at	361945	Postn_prec	periostin, osteoblast specific factor (pr	-3.151246	0.002599	0.021976
1379422_at	300284	LOC30028	similar to RIKEN cDNA 4833435D08	-1.115847	0.002599	0.021976

1376670_at	363026	Prmt4	protein arginine N-methyltransferase 4	2.242213	0.002602	0.021976
1370004_at	29384	H2afy	H2A histone family, member Y	-1.027075	0.002605	0.021976
1398897_at	296390	Ube2v1_pr	ubiquitin-conjugating enzyme E2 varia	-1.046089	0.002606	0.021976
1372897_at	300901	Plod2	procollagen lysine, 2-oxoglutarate 5-di	2.044527	0.002606	0.021976
1382783_at	308755	Blm_predic	Bloom syndrome homolog (human) (pr	-2.505466	0.002606	0.021976
1398926_at	361310	Pfdn1_prec	prefoldin 1 (predicted)	-1.449349	0.002611	0.021997
1387247_at	25204	Pcsk1	proprotein convertase subtilisin/kexin t	1.805414	0.002613	0.021997
1393114_at	407764	siat7D	alpha-2,6-sialyltransferase ST6GalNAc	1.000062	0.002616	0.021997
1399073_at	293705	Otub1_pre	OTU domain, ubiquitin aldehyde bindir	-2.302027	0.002618	0.021997
1378871_at	313058	Bai2_predi	brain-specific angiogenesis inhibitor 2	-4.184157	0.002619	0.021997
1370823_at	83837	Bambi	BMP and activin membrane-bound inh	-1.211867	0.00262	0.021997
1371463_at	192246	Phf5a	PHD finger protein 5A	-1.298714	0.002621	0.021997
1372090_at	60661	Max	Max protein	1.212537	0.002623	0.021997
1372055_at	311647	RGD13065	similar to P-Rex1 (predicted)	-2.943906	0.002624	0.021997
1371944_at	363836	Ube2l3_pre	ubiquitin-conjugating enzyme E2L 3 (p	-1.04313	0.002625	0.021997
1371968_at	362884	Tmbim4	transmembrane BAX inhibitor motif cor	-1.617863	0.002628	0.022008
1393547_at	498726	RGD15603	similar to hypothetical protein C630023	2.709608	0.002636	0.022054
1373849_at	361925	Actl6a	actin-like 6A	-1.093453	0.002645	0.022116
1384448_at	317486	RGD15658	similar to RIKEN cDNA 1700045I19 (p	-1.860888	0.002648	0.022131
1375456_at	309399	RGD13052	similar to Cgi67 serine protease precur	-0.977965	0.00265	0.022136
1390632_at	498997	RGD15632	similar to thrombospondin, type I, dom	3.640925	0.002653	0.022144
1368196_at	25680	Clps	colipase, pancreatic	4.361155	0.002675	0.022314
1386878_at	305880	Lrp10	low-density lipoprotein receptor-relatec	1.389091	0.002681	0.022353
1385518_at	313776	RGD13049	similar to RIKEN cDNA 2310042D19	2.428949	0.002689	0.022399
1370444_at	257648	Cacna1b	calcium channel, voltage-dependent, N	-1.913459	0.002693	0.022422
1371959_at	365877	Hist2h2aa_	histone 2, H2aa (predicted)	2.640909	0.002695	0.022422
1379927_at	365894	Trim33_pre	tripartite motif protein 33 (predicted)	2.8106	0.002699	0.022431
1397341_at	290326	Pbk_predic	PDZ binding kinase (predicted)	-5.454948	0.002702	0.022431
1372682_at	362518	RGD13072	similar to RIKEN cDNA 2810432L12	-1.485603	0.002702	0.022431
1391545_at	288111	Ccdc52	coiled-coil domain containing 52	-1.683932	0.002703	0.022431
1387969_at	245920	Cxcl10	chemokine (C-X-C motif) ligand 10	6.517272	0.002707	0.022456
1388380_at	300111	Samm50	sorting and assembly machinery comp	-1.14137	0.00271	0.022461
1389964_at	293453	Ndufab1_p	NADH dehydrogenase (ubiquinone) 1,	-1.165251	0.002716	0.02249
1375621_at	261737	Sfxn5	sideroflexin 5	2.338651	0.002716	0.02249
1381094_at	362118	RGD13097	similar to FLJ00022 protein (predicted)	2.775847	0.002723	0.022522
1374777_at	502894	LOC50289	hypothetical protein LOC502894	-2.307177	0.002725	0.022522
1389093_at	29431	Pak1	p21 (CDKN1A)-activated kinase 1	-2.506558	0.002726	0.022522
1368566_a_at	64539	Ndufv3l	NADH dehydrogenase (ubiquinone) fla	-0.978782	0.002733	0.022568
1384797_at	309187	RGD13098	similar to RIKEN cDNA 5730596K20 (p	-1.682194	0.00274	0.02261
1397316_at	299204	Nek9_pred	NIMA (never in mitosis gene a)- relate	2.276502	0.002741	0.02261
1388720_at	361225	NA	NA	-1.671785	0.002745	0.022615
1391972_a_at	304655	Zswim4_pr	zinc finger, SWIM domain containing 4	1.214323	0.002747	0.022615
1371523_at	498425	LOC49842	similar to U2 small nuclear ribonucleop	-1.213031	0.002748	0.022615
1374156_at	314259	Mpp5_prec	membrane protein, palmitoylated 5 (M/	1.31697	0.002749	0.022615
1374449_at	297594	Cdca3	cell division cycle associated 3	-3.404478	0.002753	0.022633
1376368_at	294009	Cuedc2_pr	CUE domain containing 2 (predicted)	-2.296032	0.002756	0.022648
1389622_at	362322	RGD15658	similar to citrin (predicted)	-1.654749	0.00276	0.022669
1389307_at	29572	Aplp1	amyloid beta (A4) precursor-like protei	-1.130371	0.002768	0.022715
1369158_at	24247	Casr	calcium-sensing receptor	1.977796	0.00277	0.022715
1383409_at	500504	NA	NA	1.291102	0.002772	0.022725
1384609_a_at	363089	RGD13114	similar to RIKEN cDNA B230380D07 (	1.851127	0.002779	0.022755

1374828_at	360722	Pdia5	protein disulfide isomerase-associated	2.861831	0.002779	0.022755
1376199_at	364666	Ccrk	cell cycle related kinase	-1.276519	0.002787	0.022773
1391585_at	59319	Nyw1	ischemia related factor NYW-1	-1.703946	0.002788	0.022773
1389148_at	317191	RGD15497	similar to RIKEN cDNA 9430083G14	-1.378832	0.002789	0.022773
1377168_at	297516	Cpne9	copine family member IX	-4.394334	0.002789	0.022773
1388609_at	313667	Plekhm2_p	pleckstrin homology domain containing	1.046511	0.002792	0.022777
1398867_at	246216	Prpf19	PRP19/PSO4 pre-mRNA processing fa	-1.383652	0.002795	0.022777
1371624_at	315158	Zc3h7b_pr	zinc finger CCCH-type containing 7B (	-1.572236	0.002795	0.022777
1384128_at	246295	LOC24629	glycine-, glutamate-, thienylcyclohexyl	1.434226	0.002796	0.022777
1394940_at	300870	RGD13113	similar to hypothetical protein FLJ2003	1.174801	0.0028	0.022786
1386689_at	304669	Hook2	hook homolog 2 (Drosophila)	1.230998	0.002801	0.022786
1373535_at	360891	Enah	enabled homolog (Drosophila)	1.1854	0.002802	0.022786
1390029_at	361110	Tmem110	transmembrane protein 110	-1.476611	0.002804	0.022786
1390859_at	291921	RGD13051	similar to Nedd4 binding protein 1 (pre	1.419618	0.002813	0.022846
1399152_at	313474	Eps15	epidermal growth factor receptor pathw	1.476923	0.002815	0.022848
1395875_at	294595	RGD13060	similar to KIAA0372 gene product (pre	1.298458	0.002821	0.022882
1374515_at	290303	RGD13064	similar to RIKEN cDNA 6330409N04	1.017773	0.002827	0.022919
1374575_at	362165	Creb3l1	cAMP responsive element binding prot	1.149903	0.00283	0.022929
1367890_at	64314	Casp2	caspase 2	-1.810228	0.002836	0.022965
1379827_at	497941	LOC49794	similar to G protein pathway suppresso	-1.250245	0.002841	0.02299
1393982_at	299112	Pole2_pred	polymerase (DNA directed), epsilon 2	-1.591677	0.002854	0.023076
1399016_at	303470	Myst2	MYST histone acetyltransferase 2	-1.026307	0.002862	0.023117
1371620_at	290995	RGD13080	similar to px19-like protein	-1.040241	0.002863	0.023117
1377630_at	366602	Tm4sf13	transmembrane 4 superfamily member	1.090614	0.002864	0.023117
1393418_at	58814	Tmod2	tropomodulin 2	1.263801	0.002866	0.023117
1372814_at	360868	RGD13106	similar to RIKEN cDNA 2010005O13	1.219576	0.002872	0.023129
1398804_at	64472	Mak10	MAK10 homolog, amino-acid N-acetylt	-1.252489	0.002872	0.023129
1371595_at	85242	Pola2	polymerase (DNA directed), alpha 2	5.818068	0.002873	0.023129
1381093_at	114101	Cds2	CDP-diacylglycerol synthase (phospha	2.284613	0.002874	0.023129
1368871_at	116667	Map3k1	mitogen activated protein kinase kinas	1.621535	0.002876	0.02313
1374564_at	304591	Dtx2	deltex 2 homolog (Drosophila)	1.497299	0.002878	0.023131
1386795_at	296846	RGD13056	similar to hypothetical protein FLJ2553	-2.340044	0.00288	0.023131
1368867_at	59117	Eif2c2	eukaryotic translation initiation factor 2	1.831432	0.002884	0.023138
1383897_at	361844	RGD15613	similar to macroH2A2 (predicted)	-2.26685	0.002885	0.023138
1377760_at	360828	Noc4l	nucleolar complex associated 4 homol	-1.372707	0.002886	0.023138
1375411_at	299643	Ndufa7_pre	NADH dehydrogenase (ubiquinone) 1	-1.284021	0.002888	0.023138
1389723_at	363131	Pik3r4_pre	phosphoinositide-3-kinase, regulatory	1.010148	0.002889	0.023138
1374233_at	308009	RGD13083	similar to RIKEN cDNA 1810055E12 (	0.973648	0.002895	0.023171
1389965_at	192152	Tgoln2	trans-golgi network protein 2	1.097241	0.002897	0.023171
1388877_at	296134	Mrps5_pre	mitochondrial ribosomal protein S5 (pr	-1.248519	0.002914	0.023293
1388482_at	362115	RGD13070	similar to RIKEN cDNA 9130404D14	-1.32121	0.002924	0.023348
1390524_at	317241	Rnf12	ring finger protein 12	1.120377	0.002926	0.023348
1392988_at	309262	Nsdhl	NAD(P) dependent steroid dehydroger	-1.309633	0.002927	0.023348
1375627_at	297968	RGD13077	similar to hypothetical protein FLJ1034	-1.610008	0.002928	0.023348
1393654_at	367090	Neil1	nei endonuclease VIII-like 1 (E. coli)	1.758049	0.002929	0.023348
1389549_at	306544	Prosc_pred	proline synthetase co-transcribed (pre	-0.947715	0.002932	0.02335
1377390_at	288227	Bace2	beta-site APP-cleaving enzyme 2	-2.642893	0.002935	0.02335
1372590_at	303701	C1qtnf1	C1q and tumor necrosis factor related	-4.711432	0.002936	0.02335
1382163_at	85471	Gata3	GATA binding protein 3	-2.985207	0.002937	0.02335
1390573_a_at	305897	Nfatc4	nuclear factor of activated T-cells, cyto	2.149145	0.002941	0.023373
1381056_at	293619	Pkp3_pred	plakophilin 3 (predicted)	3.13632	0.002949	0.023421

1370544_at	192360	Eml2	echinoderm microtubule associated pr	2.27305	0.002962	0.023504
1368323_at	29436	Tfpi	tissue factor pathway inhibitor	-1.260273	0.002971	0.023567
1371225_at	66027	Drp2	dystrophin related protein 2	-1.581994	0.002975	0.023576
1371612_at	292534	TSEN34	tRNA splicing endonuclease 34 homol	-0.977402	0.002976	0.023576
1371853_at	299743	Mrpl42_pre	mitochondrial ribosomal protein L42 (p	-1.123874	0.002981	0.023591
1372303_at	299909	RGD13076	similar to 0910001A06Rik protein (pre	-1.239975	0.002982	0.023591
1369963_at	114113	Pafah1b3	platelet-activating factor acetylhydrola	-1.712859	0.002983	0.023591
1377792_at	291445	Megf10	Megf10 protein	2.887612	0.002985	0.023591
1370351_at	85425	Tdrd7	tudor domain containing 7	-1.007801	0.002989	0.023592
1378614_at	29424	Hdh	Huntington disease gene homolog	1.56881	0.00299	0.023592
1378527_at	303590	LOC30359	NA	4.720826	0.00299	0.023592
1388433_at	360626	Krt1-19	keratin complex 1, acidic, gene 19	5.897291	0.003	0.023648
[truncated: 329,348 more chars]
